# Carbon dots from natural sources as theranostic agents: integrating fluorescence and ROS generation for photodynamic therapy

**DOI:** 10.1039/d6ra01035k

**Published:** 2026-04-22

**Authors:** Martín Lemos Vilches, M. Natalia Calienni, María Belén Rivas Aiello, Aldo A. Rubert, Jorge Montanari, Cristian R. Lillo

**Affiliations:** a Universidad Nacional de Hurlingham (UNAHUR), Laboratorio de Nanosistemas de Aplicación Biotecnológica (LANSAB) Av. Vergara 2222, Villa Tesei Buenos Aires 1688 Argentina jorge.montanari@unahur.edu.ar; b Consejo Nacional de Investigaciones Científicas y Técnicas (CONICET) Buenos Aires Argentina; c Comisión de Investigaciones Científicas de la Provincia de Buenos Aires (CIC) La Plata 1900 Argentina; d Instituto de Investigaciones Fisicoquímicas Teóricas y Aplicadas (INIFTA), Universidad Nacional de La Plata – CONICET 1900 La Plata Buenos Aires Argentina

## Abstract

Carbon dots (CDs) have emerged as versatile nanostructures for biomedical applications due to their biocompatibility, tunable fluorescence and capacity to generate reactive oxygen species (ROS). In this work, CDs were synthesized from three fruit-derived natural sources (watermelon, strawberry and blueberry) using both undoped and urea-doped formulations and their physicochemical and photochemical properties were systematically compared. While zeta potential values differed depending on the carbon source, nitrogen doping caused a blue shift in excitation and emission peaks, indicating the formation of smaller optical bandgap states. The doped CDs displayed higher fluorescence quantum yields but reduced singlet oxygen production compared to their undoped counterparts, suggesting an inverse correlation between nitrogen incorporation and ROS generation. Among the undoped systems, blueberry-derived CDs exhibited the highest singlet oxygen yields. Cytotoxicity assays on SK-Mel-28 cells showed no significant effects in darkness. Upon irradiation, undoped CDs induced phototoxicity in a source-dependent manner, while doped CDs showed no intrinsic or photoinduced toxicity under the tested conditions. Overall, nitrogen doping enhances CD fluorescence while diminishing ROS generation efficiency. These results support the strategic use of different fruit-derived CDs to balance fluorescence and photodynamic activity and suggest that combining doped watermelon CDs with undoped blueberry CDs could maximize both optical and therapeutic performance.

## Abstract 2

Carbon dots (CDs) have emerged as versatile nanostructures for biomedical applications due to their biocompatibility, tunable fluorescence and capacity to generate reactive oxygen species (ROS).

## Graphical abstract

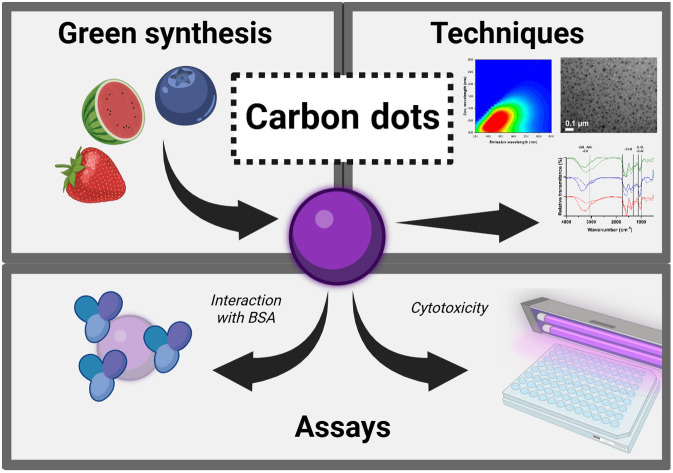

## 1 Introduction

Carbon-based nanomaterials include graphene, carbon nanotubes, fullerenes, and carbon dots (CDs), among others, and have exponentially grown as subjects of research over the last decades due to their remarkable properties that make them versatile for numerous applications.^1^ A growing number of studies focus on their unique photophysical and photochemical characteristics, particularly in biomedical applications, such as theragnostic approaches for cancer that combine photodynamic therapy (PDT) and bioimaging, owing to their intrinsic fluorescence.^2^ Recent advances have further expanded these platforms by integrating biomimetic coatings and synergistic modalities, such as chemodynamic and photothermal therapies, to overcome tumor microenvironment limitations.^3^ In this context, activatable fluorescent probes and near-infrared (NIR) sensors have gained prominence as non-invasive modalities for the early detection of malignancies and the sensitive monitoring of environmental and biological analytes.^4,5^

In particular, CDs have emerged as especially promising tools due to their biocompatibility, stability, and tunable optical properties. These nanostructures typically exhibit a complex architecture involving different degrees of carbonization. One of their main photochemical features is the strong fluorescence that they exhibit upon irradiation with light of a specific frequency,^6,7^ often attributed to the presence of surface states or to molecular fluorophores created during their synthesis. Beyond fluorescence, CDs can also generate reactive oxygen species (ROS), making them suitable photosensitizers for photodynamic therapy through light-induced pathways alternative to fluorescence.^8^ Furthermore, the development of multi-responsive nanohybrids has recently demonstrated that amplified ROS generation can be effectively triggered to tackle complex biological barriers, such as resistant fungal biofilms.^9^ Beyond therapy, carbon-based platforms are increasingly used in sensitive detection strips and combinatorial chemo-gene systems to enhance therapeutic efficacy.^10,11^

Although initially synthesized through chemical or physical routes, CDs can also be obtained from natural carbon sources *via* green synthesis, offering a sustainable alternative with lower costs and minimal toxic residues. Among these natural precursors, plant leaves, fruit peels, and juices have been extensively reported,^12^ highlighting the strong influence of the starting materials on their final composition and properties. Fruits particularly rich in organic compounds, sugars, and pigments such as anthocyanins and polyphenols are especially attractive as sources for these nanostructures. The use of such natural products in carrier-free nanoplatforms has shown significant promise in enhancing singlet oxygen yields and improving biocompatibility.^13^

Through modifications in their synthesis, such as nitrogen doping, CD properties can be tuned, for instance, to enhance fluorescence.^14^ Because nitrogen has more valence electrons than carbon but a similar atomic size, its incorporation modulates the electronic environment, increasing the fluorescence quantum yield without disrupting the stability of the carbonaceous matrix. This tunability supports the design of smart probes with precise spatiotemporal control for live-cell imaging.^15^ Beyond medical uses, nitrogen-doped carbon dots (NCDs) have also been integrated into living biocomposites, where their photoinduced catalytic activity is leveraged for environmental remediation and enhanced electron transfer.^16^

Furthermore, as previously mentioned, PDT is a key feature of CD applications in medicine, since these nanomaterials can act as photosensitizers. Upon irradiation, CDs generate ROS such as singlet oxygen, making them suitable for inducing cytotoxicity in target cells, for example, in cancer therapy.^17^ Despite the growing body of literature on carbon-based nanomaterials, a relevant challenge remains in achieving a balanced optimization of both fluorescence and ROS generation through sustainable routes. This requires understanding how the chemical diversity of different fruit precursors dictates the equilibrium between radiative (fluorescence) and non-radiative (ROS generation) pathways. Consequently, there is a need to explore specific natural sources and synthesis conditions to tailor these properties, and to systematically compare CDs obtained from different fruits to establish correlations between their optical and biological features. Furthermore, the correlation between the chemical fingerprint of natural precursors and the resulting biological performance of CDs is often overlooked. Furthermore, achieving a balance between stable delivery and on-demand drug release remains a pivotal challenge in the design of efficient antitumor nanoassemblies.^18^

In this work, we aim to address these gaps by synthesizing CDs from three distinct natural sources—in both doped and undoped forms—to systematically evaluate their structure–property relationships. Beyond a thorough physicochemical characterization, we quantified their fluorescence quantum yields and singlet oxygen generation efficiencies to assess their potential as dual-purpose agents. To evaluate the potential clinical applications of these materials, we investigated the formation of the protein corona^19^ using bovine serum albumin (BSA) as a model, determining the affinity constants and interaction mechanisms that govern their behavior in biological fluids. Finally, the therapeutic potential of these CDs was validated through *in vitro* assays on a melanoma cell line, evaluating both intrinsic biocompatibility and photo-induced cytotoxicity. This study aims to pave the way for the development of fruit-derived CDs as effective and sustainable theranostic platforms for skin cancer therapy.

## 2 Experimental

### 2.1 Materials

The natural precursors *i.e.* blueberries, strawberries, and watermelons, were all sourced from the Pereyra region in Buenos Aires, Argentina (34°54′ S, 58°09′ W, 11 m a.s.l.), either directly from organic farms or *via* local suppliers, to maintain a consistent geographical profile for the raw materials. 9,10-Anthracenediyl-bis(methylene)dimalonic acid (ABDA, ≥90%), furfuryl alcohol (FFA, >98%), quinine bisulfate (QBS) and bovine serum albumin (BSA, >98%) were purchased from Sigma-Aldrich (St. Louis, MI, USA) and employed without further purification. Urea was purchased from Ciccarelli (Milan, Italy). For purification purposes, 0.22 µm syringe filters with PES membranes from Membrane Solutions (Seattle, WA, USA) were utilized. 3.5 kD dialysis membrane was provided by Spectrum Laboratories, Inc. (Rancho Dominguez, CA, USA). Trypsin was purchased from Gibco (Waltham, MA, USA). MTT assay was purchased from Thermo Fisher Scientific (Buenos Aires, Argentina). Ethylenediaminetetraacetic acid (EDTA) was purchased from Santa Cruz Biotechnology (Dallas, TX, USA). Fetal bovine serum (FBS) was purchased from Internegocios (Buenos Aires, Argentina). Absolute ethanol (EtOH) was obtained from Biopack (Buenos Aires, Argentina). Dimethyl sulfoxide (DMSO) was purchased from Stanton (Buenos Aires, Argentina). Acetic acid was purchased from Sintorgan (Buenos Aires, Argentina). All other reagents used were from analytical grade.

### 2.2 Methods

#### 2.2.1 Synthesis and purification

The type of CDs depends on the synthesis conditions; therefore, different stocks were prepared from the following extracts: watermelon, blueberries and strawberries. Watermelon and strawberry juices were obtained by grinding the plant fruit with a mortar. Blueberry extract was prepared by resuspending 20 g of dehydrated blueberries in 200 mL of ultrapure water, followed by centrifugation and discarding of the pellet. CD stocks were prepared from plant extracts using a solvothermal method, which consists of subjecting 25–40 mL of the samples inside a steel and Teflon reactor to a high-pressure solvothermal oxidation process at 180 °C for 180 minutes. In addition, 30% m/v N-doped stocks were generated for each condition by adding 7.5 g urea per 25 mL of sample within the reactor prior to the solvothermal process (lower doping percentages were discarded during preliminary testing due to lower fluorescence levels; Fig. S1). The species that were generated were undoped CDs based on watermelon (W-CDs), blueberries (B-CDs) and strawberries (S-CDs), along with their 30% N-doped variants (dW-CDs, dB-CDs and dS-CDs).

Dialysis was carried out using 5 mL of sample in a 3.5 kD membrane, which was placed in 500 mL of water under stirring at 300 rpm for 3 hours. In all cases, to ensure reproducibility, each synthesis was performed in triplicate, verifying that all batches exhibited similar mass concentrations and consistent optical properties before conducting detailed fluorescence and ROS measurements. The concentration was measured using a calibration curve of absorbance at 400 nm. This curve was prepared using dilutions from a solution of known concentration obtained by dissolving lyophilized CD powder in ultrapure water. The lyophilized CD powder was obtained by BK-FD10PT series freeze dryer (Instrumentalia, Buenos Aires, Argentina).

#### 2.2.2 Characterization

The nanosystems were observed by Transmission Electron Microscopy (JEOL JEM 1200EX II) and High-Resolution Transmission Electron Microscopy (JEOL JEM-2100 plus). The size of dW-CDs and dB-CDs was determined using high-resolution TEM (HRTEM) images processed with the image analysis software ImageJ.^20^ To estimate the number of nanoparticles, their volume was first calculated from the measured diameter assuming a spherical geometry (*V* = 4/3 × π × *r*^3^). The mass of a single nanoparticle was then obtained using the density of graphitic carbon (2.2 g cm^−3^). Finally, this value, together with Avogadro's number, was used to convert concentration units into molarity and particle count.

The photoluminescent (PL) properties of CDs are shown in the UV-visible spectra and excitation–emission matrices (EEM) obtained using a Duetta spectrophotometer (HORIBA, Canada). The fluorescence decay lifetime was obtained using a Jobin Yvon Spex Fluorolog FL3-11 spectrofluorometer (Horiba Scientific, Edison, NJ, USA) with an Xe lamp as the excitation source, a monochromator for selecting the excitation and emission wavelengths (both with 1 nm bandpass gap) and a red sensitive R928 PM as detector. The fluorometer was equipped with a time-correlated single photon counting instrument with light-emitting diode excitation at 341 and 388 nm (full width at half-maximum, FWHM = ∼400 ps). Fourier transform infrared spectroscopy (FTIR) spectra were recorded by IRSpirit spectrophotometer (Shimadzu, Kyoto, Japan). Spectra were taken in the 4000–500 cm^−1^ range with 1.5 cm^−1^ resolution. Zeta potential was measured in triplicate using a Zetasizer Pro ZSV3200 series (Malvern Panalytical, Canada) by Electrophoretic Light Scattering (ELS). The results were analyzed by one-way ANOVA. The pH of the suspensions was determined using indicator strips. The PL decay time was obtained from biexponential decay fittings of the PL traces detected at 450 nm upon 341 nm excitation.^21^

The surface composition was evaluated by X-ray photoelectron spectroscopy (XPS) using a nonmonochromatic Mg Kα source (XR50, Specs GmbH) and a hemispherical electron energy analyzer (PHOIBOS 100, Specs GmbH) at a takeoff angle of 90° to the lenses axis. The elemental XPS composition of the surface was obtained by measuring on samples of droplets evaporated in a nitrogen stream on silicon wafers (Si 100) previously washed with piranha solution and rinsed with deionized water. The silicon substrate is suitable for accurately inferring carbon species. Spectral data were collected with a pass energy of 40 and 10 eV, for low- and high-resolution modes respectively, operating in fixed transmission analysis mode. Prior to measuring the samples, the energy scale calibration was performed using two points: gold (Au 4f_7/2_, binding energy BE = 84.00 eV) and copper (Cu 2p_3/2_ BE = 932.67 eV) samples cleaned with sputtering Ar^+^ gun. Ambient temperature and a typical pressure in the 10^−7^ Pa range were employed during measurement. The adventitious carbon C 1s component peak at 284.8 eV was used as a charge reference control. Spectra were analyzed using CasaXPS v2.2.99 software, with a Shirley-type background line. Quantification analysis based on peak areas and the relative sensitivity factor was employed and no geometry preference method by analytes was assumed.

#### 2.2.3 Quantum yields calculation

Fluorescence at 360 nm excitation was estimated by calculating the fluorescence quantum yield (*Φ*_PL_) from eqn (1), using quinine sulfate as reference (55%).^22,23^1
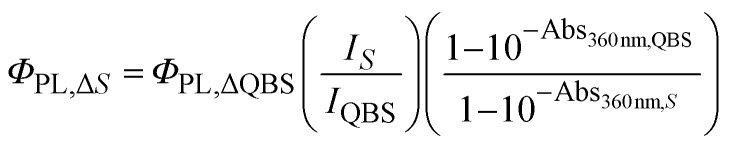
*I* is the area under the fluorescence spectrum between 370 and 700 nm of the suspension when excited at 360 nm, *A* is its absorbance at 360 nm, *S* is for the sample and *Φ*_PL_ is the fluorescence quantum yield of the photosensitizer. QBS = quinine bisulfate.

Reactive oxygen species (ROS) generation was determined through an indirect oxygen consumption assay using ABDA (an anthracene derivative that enables the detection of singlet oxygen generation) during irradiation at 405 nm.^24^ Singlet oxygen quantum yields (*Φ*_O_) were calculated from eqn (2), considering methylene blue as the standard (52%):^25^2
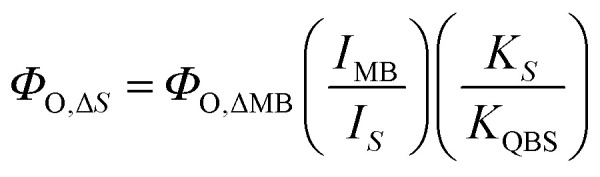
*I* is the area under the absorption spectrum of the suspension between 300 and 500 nm in the absence of ABDA, *K* is the slope of ABDA decay as a function of time in the presence of the photosensitizer, *S* is for the sample and *Φ*_O_ is the singlet oxygen quantum yield of the photosensitizer. MB = methylene blue.

Superoxide anion radical generation was evaluated indirectly using the commercial kit Colestat for cholesterol quantification (Wiener Lab, Argentina) in suspensions irradiated at 400 nm.^26^

#### 2.2.4 Interaction with BSA

8 mL of CD solutions ranging from 0.01 mg mL^−1^ to 0.05 mg mL^−1^ were prepared by adding 2 mL of 20 µM BSA.^27^ Aliquots were subsequently taken in triplicate for fluorescence spectral measurements in the 300–600 nm range using a Duetta spectrophotometer at an excitation wavelength of 290 nm. *K*_SV_ quenching constants were obtained by linear regression of plots of *I*_BSA_/*I*_Sample_ of BSA *versus* CD concentration (eqn (3)).^27^3
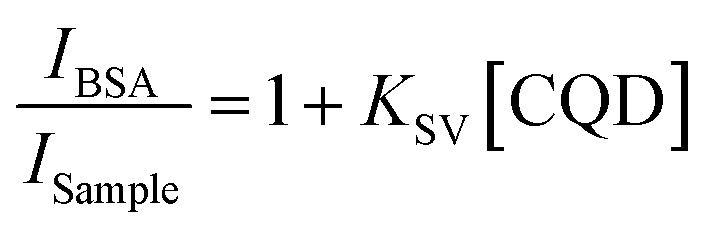
*I* is the area under the fluorescence spectrum between 300 and 400 nm of the suspension when excited at 290 nm and *K*_SV_ is the Stern–Volmer constant.

#### 2.2.5 Cytotoxicity assays

Cytotoxicity was evaluated using the MTT assay, both in darkness and after irradiation. Assays were carried out on SK-Mel-28 cells (SK-Mel-28 cells were kindly provided by IMBICE (Instituto de Biología Celular. CONICET-CIC La Plata, Argentina)), a human melanoma cell line.^28^ Subtoxic CDs concentrations were determined by darkness cytotoxicity assays with concentrations between 0.01 and 0.2 mg mL^−1^. Using the maximum subtoxic concentration found for each type of CD, combined cytotoxicity assays with nanoparticles and irradiation using a LED lamp at 450 nm and a laser at 405 ± 10 nm (50 mW) were performed. The cytotoxicity assays with CDs under irradiation were performed after demonstrating that irradiation with LED light and laser does not generate intrinsic cytotoxicity. Irradiation was applied 24 h after treatment incubation. Cell viability in MTT assay was assessed at 595 nm. Inferential statistics were analyzed by one-way ANOVA.

## 3 Results and discussion

### 3.1 Characterization

**Fig. 1:**
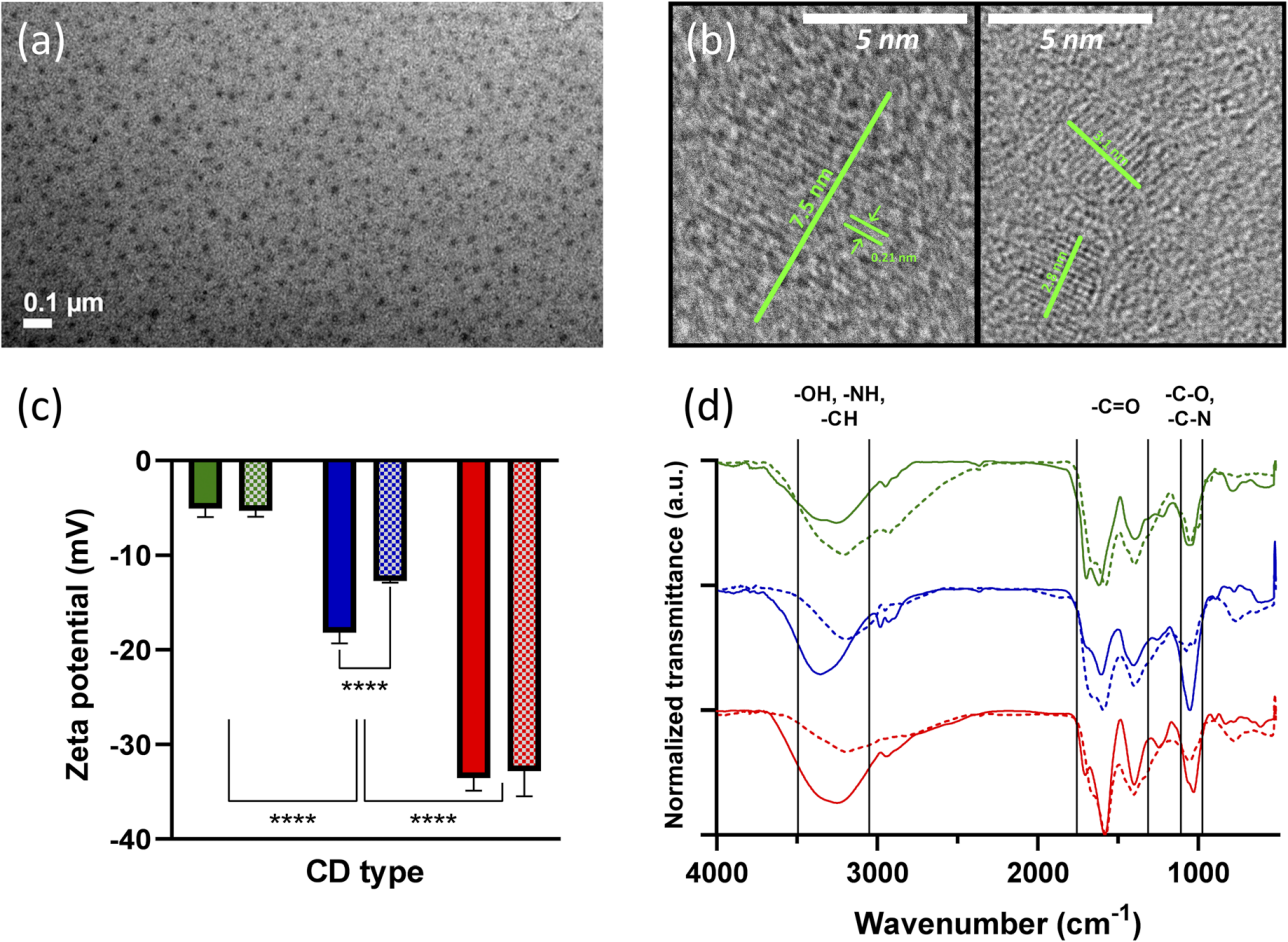
(a) TEM and (b) HRTEM image with lattice spacing of CDs. The interlayer spacing of 0.21 nm is observed in the HRTEM images. (c) Zeta potential of 0.1 mg mL^−1^ watermelon-derived (green), blueberry-derived (blue) and strawberry-derived (red) CDs in a 10 mM NaCl solution. CDs measured by ELS. Checkered columns indicate doped CDs. Zeta potentials are shown as mean ± SD (*n* = 3) (****p < 0.0001). (d) FTIR of watermelon-derived (green), blueberry-derived (blue) and strawberry-derived (red) CDs. Dotted line indicates doped CDs.

**Fig. 2:**
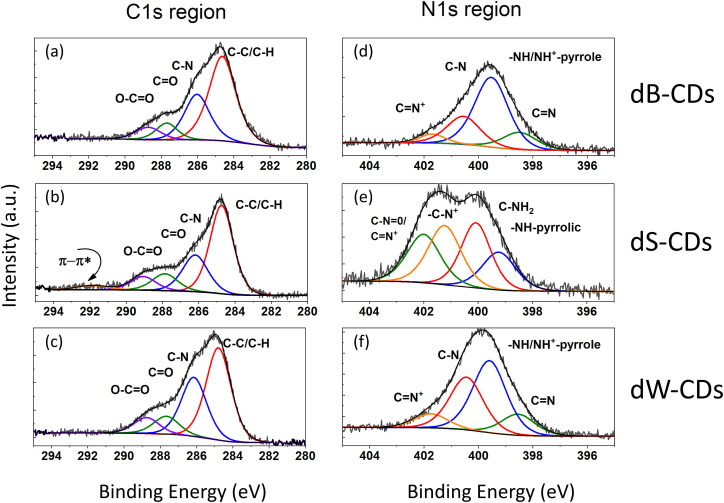
High resolution fitted XPS region for XPS (a–c) C 1s and (d–f) N 1s for N-doped CDs samples. Arbitrary intensity corresponds to kilo-count per second (kc.p.s.). BE = binding energy.

The CD suspension appeared homogeneous and non-aggregated (Fig. 1a). The nanoparticle size was in the expected range for this kind of structure, with diameters ranging approximately between 2 and 8 nm, as can be observed in Fig. 1b, which shows two representative HRTEM images of the CDs. The interlayer spacing (0.21 nm) was also observed in the HRTEM images.^29^ Zeta potential measurements showed significant differences according to the carbon source. Watermelon-derived CDs exhibited values close to zero, whereas strawberry-derived CDs showed the most negative potentials. Except for blueberry-derived CDs, nitrogen doping did not appear to alter the zeta potential of the formulations (Fig. 1c). The pH of all samples ranged between 4 and 5, as determined by indicator strips. In all the cases FTIR spectrum show C–O and C–N vibrations at 1060 cm^−1^ and two peaks for C=O vibrations at 1400 cm^−1^ and 1590–1600 cm^−1^. Additionally, they exhibit a region between 3000 and 3500 cm^−1^ corresponding to O–H, N–H and C–H stretching bands (Fig. 1d).^30^ All samples treated with urea exhibited an N/C ratio close to 0.2. Doped blueberry samples exhibit 30% less N than doped strawberry samples and 21% less than doped watermelon samples (Table 1). Doped strawberry and doped watermelon samples show a larger N/Si ratio than doped blueberry samples. To perform a more exhaustive comparison, fittings were made to the corresponding C 1s and N 1s spectral regions (Fig. 2). In all cases C 1s region was fitted and assigned to the same four species in different proportions, but doped strawberry sample exhibited a π → π* shake up typical for conjugated and extended structures near to 292 eV.^31^ The rest of assigned carbon species were 284.7(1) eV for C–C or C–H moieties; 286.1(1) eV for C–N; 287.7(1) eV to carbonyl group (C=O) and 288.8(1) eV to carboxylic group.^32^ The doped watermelon sample is distinguished by its large proportion of the components assigned to C–N. In the N 1s region the nitrogen species assignments are common to doped blueberry and doped watermelon samples. Doped strawberry sample standing out due to its broader N 1s region with shifted binding energy. The N 1s region of doped blueberry and doped watermelon samples was fitted with four peaks, assigned to the following species: 398.45(5) eV for C=N bonds; 399.5(1) eV for NH^+^ pyrrole species like general NH with formal positive charge on N bonded to twin carbons; 400.5(1) eV for C–N moieties; and 401.7(1) eV for C=N^+^ species.^33^ In Fig. 2d and f, the 399.5(1) eV peak is the most abundant, with dW-CDs sample standing out for also having a greater quantity of 400.5(1) eV relative to its main signal. Likewise, doped strawberry sample also presented four peaks for fit with the following values: 399.3(1) eV assigned to pyrrolic-type NH; 400.1(1) eV typical of C–NH_2_ groups; 401.2(1) eV for –C–N^+^ species; and 402.0(1) eV for C=N^+^ (or N-oxide) moieties.^34^ The doped strawberry sample fit shows a similar abundance for nitrogen species populations, except for NH-pyrrolic. This may be due to the extended structural character (already mentioned in the C 1s region), where NH-pyrrolic would be present in a lower proportion.

**Table 1:** Elemental XPS composition of each sample in atomic element/Si ratios, to simplify a comparison between samples. A final column showing the N/C ratio is also included

Sample	Na/Si	O/Si	N/Si	C/Si	S/Si	P/Si	Ca/Si	N/C
W-CDs	1.29	19.32	0.33	32.04	0.57	0.98	2.24	0.01
B-CDs	0.41	5.94	0.17	8.14	0.30	—	0.09	0.02
S-CDs	2.40	26.04	0.75	34.98	0.53	—	1.55	0.02
dW-CDs	0.38	14.84	5.80	24.94	0.90	0.05	—	0.23
dB-CDs	0.15	4.44	1.01	5.56	0.37	0.05	—	0.18
dS-CDs	—	19.04	4.22	16.49	2.94	—	—	0.26

### 3.2 Photophysical properties

**Fig. 3:**
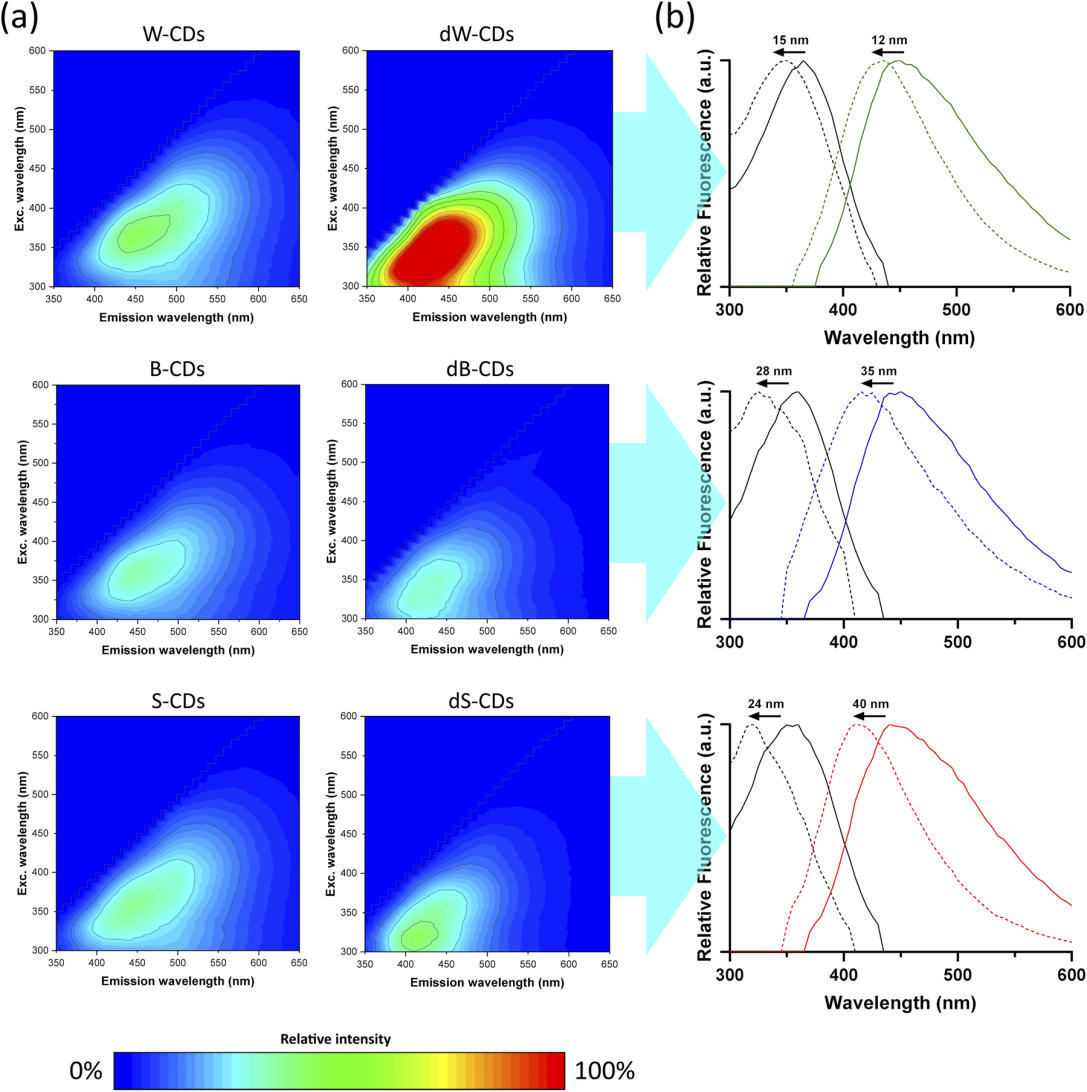
(a) Excitation–emission matrices of aqueous suspensions of the different CDs at 0.1 mg mL^−1^. (b) Normalized emission (colored line) and excitation (black line) spectra of watermelon-derived (up), blueberry-derived (middle) and strawberry-derived (bottom) CDs at 0.1 mg mL^−1^ in water. Dotted line indicates doped CDs and black arrows show the peak shifts. The fluorescence peaks are the values in Table 2.

The photophysical properties of the obtained CDs are shown in Fig. 3. Remarkably, their properties remained unchanged over a nearly 3 year period at 4 °C (Fig. S6), while the suspensions maintained their translucency and color and did not precipitate during the same period. The excitation wavelengths of the undoped species ranged from 360–365 nm, while their N-doped counterparts were consistently lower. The emission wavelengths of the undoped species ranged from 441–447 nm and again, their doped counterparts exhibited lower values. This indicates that in N-doped samples, the intensity peaks occur at shorter excitation and emission wavelengths (Fig. 3) (Table 2).^14^ This blue-shifting behavior can be attributed to pyrrolic and pyridinic nitrogen groups in N-doped CDs, which modify the electronic structure of the carbon core. The relatively high electron affinity of N also contributes to this effect by withdrawing electron density from the conjugated carbon plane.^35,36^ This could be explained by experimental and quantum-mechanical studies showing the strong electron-withdrawing nature of N, leading to an increased band gap and higher-energy (blue-shifted) emission.^36^

**Table 2:** Photophysical characterization of CDs. *Φ*_PL_ and *Φ*_O_ of the different types of CDs were calculated from eqn (1) and (2)

Sample	*λ*_ex_ (nm)	*λ*_em_ (nm)	*Φ*_PL_ (%)	*Φ*_O_ (%)	*τ* a (ns)
W-CDs	365	447	1.07	16.66	6.72 ± 0.04
B-CDs	360	443	0.94	29.37	6.73 ± 0.04
S-CDs	360	441	0.85	15.05	6.71 ± 0.04
dW-CDs	350	435	4.24	10.22	8.69 ± 0.04
dB-CDs	325	415	1.94	2.26	8.71 ± 0.04
dS-CDs	320	417	1.93	10.58	8.63 ± 0.04

a Average lifetimes.

**Fig. 4:**
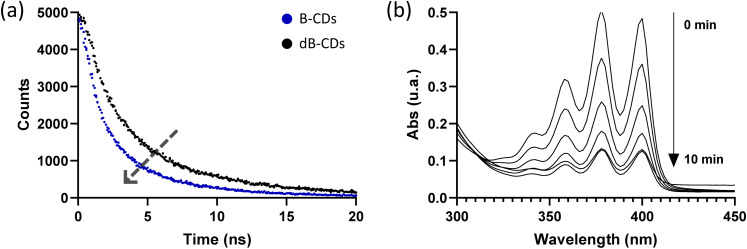
(a) Time decay of doped (black) and undoped (blue) blueberry CDs. (b) Absorbance decay of 0.5 mM ABDA in the presence of B-CDs at 0.01 mM under laser irradiation at 405 nm.

The fluorescence decay lifetime reflects the average excited-state lifetime of the nanoparticles. All undoped CDs exhibited a decay lifetime of 6.7 ns, whereas all doped CDs showed longer lifetimes in the range of 8.6–8.7 ns (Fig. 4a) (Table 2).

### 3.3 Quantum yields calculation

Table 2 shows that all doped CDs exhibited higher fluorescence yields compared to their undoped counterparts, particularly between W-CDs and dW-CDs. This effect can be attributed to an increased fluorescence quantum yield associated with a higher content of pyrrolic groups, as evidenced by the XPS results (Fig. 2), which enhance radiative recombination by introducing electron-donating lone pairs and stabilizing surface defect states. Furthermore, nitrogen-doping induces an upward shift of the Fermi level and increases the electron density in the conduction band, which further promotes these radiative transitions.^37^ However, an inverse correlation was observed for singlet oxygen generation, where doping with nitrogen resulted in lower yields. This trade-off suggests a competition between radiative decay and intersystem crossing pathways. Such a balance strongly depends on the nature and location of the nitrogen species; for instance, while graphitic and pyrrolic nitrogen can facilitate triplet formation and oxygen adsorption in certain configurations, in our system, the nitrogen-containing groups (primarily surface pyrrolic and amino-type moieties) appear to favor radiative decay pathways, thereby suppressing the population of the triplet excited states required for singlet oxygen production.^27,38^ Therefore, the surface chemistry of these CDs can be tuned to prioritize either diagnostic (imaging by fluorescence) or therapeutic (PDT) performance, depending on the nitrogen content. Regarding the specific nature of the reactive species, the Colestat kit quantification showed no difference in absorbance at 505 nm between the irradiated and non-irradiated samples. This confirms the absence of superoxide anion during ROS generation, further supporting that the photodynamic activity observed is primarily driven by the singlet oxygen pathway.

### 3.4 Interaction with BSA

**Fig. 5:**
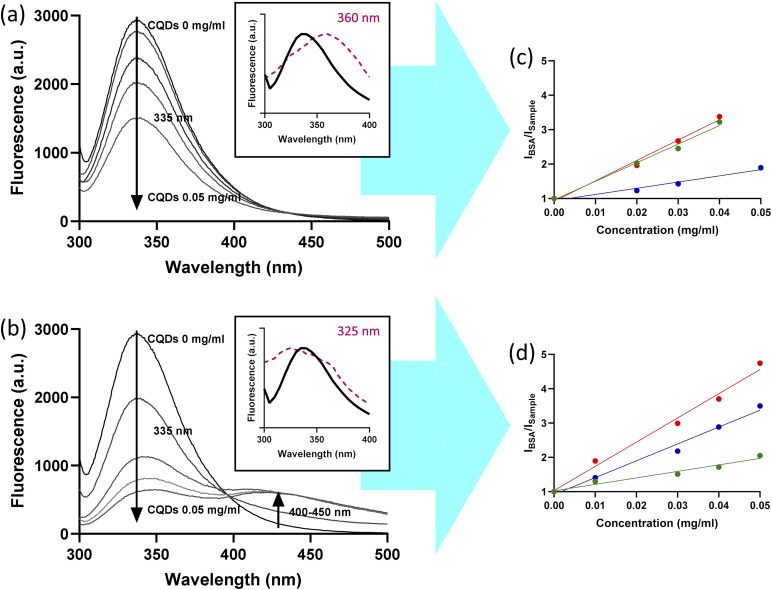
(a and b) Fluorescence spectrum (excited to 290 nm) of BSA at 5 µM with (a) B-CDs and (b) dB-CDs in different concentrations. 0.05 mg mL^−1^ is equivalent to 5.3 µM of B-CDs and 0.15 µM of dB-CDs. Inset: normalized fluorescence emission of BSA solution at 290 nm excitation (black curve, 340 nm emission) and excitation spectrum of CDs (violet curve, peak excitation in violet number). Fluorescence is shown as mean (*n* = 4). (c and d) Stern–Volmer plots of *I*_BSA_/*I*_Sample_ of BSA (5 µM) as a function of CD concentration: watermelon-derived (green), blueberry-derived (blue) and strawberry-derived (red), under (c) undoped and (d) doped conditions.

In all cases, fluorescence quenching of BSA at 335 nm was observed with increasing CD concentration from 0 to 0.15 mg mL^−1^ when the suspension is irradiated at 290 nm (Fig. S3). The Stern–Volmer plots exhibited a linear quenching behavior (Fig. 5c and d). *K*_SV_ quenching constants were positives in all cases. *K*_SV_ value for dW-CDs was 2.58 × 10^5^ M^−1^, while that for dB-CDs was 1.75 × 10^7^ M^−1^. These values indicate a high affinity toward BSA, with dB-CDs exhibiting affinity constants two orders of magnitude higher. Comparable high affinity values have been reported in previous studies on N-doped CDs.^39^

Furthermore, only suspensions containing N-doped CDs exhibited an increase in fluorescence between 400 and 600 nm, which was proportional to the concentration of CDs. This effect is associated with the overlap between the excitation peak of the CDs (320–350 nm) and the emission peak of BSA (340–350 nm) (Fig. 5a and b). This could be consistent with a FRET-type energy transfer between BSA and N-doped CDs.

### 3.5 Cytotoxicity assays

**Fig. 6:**
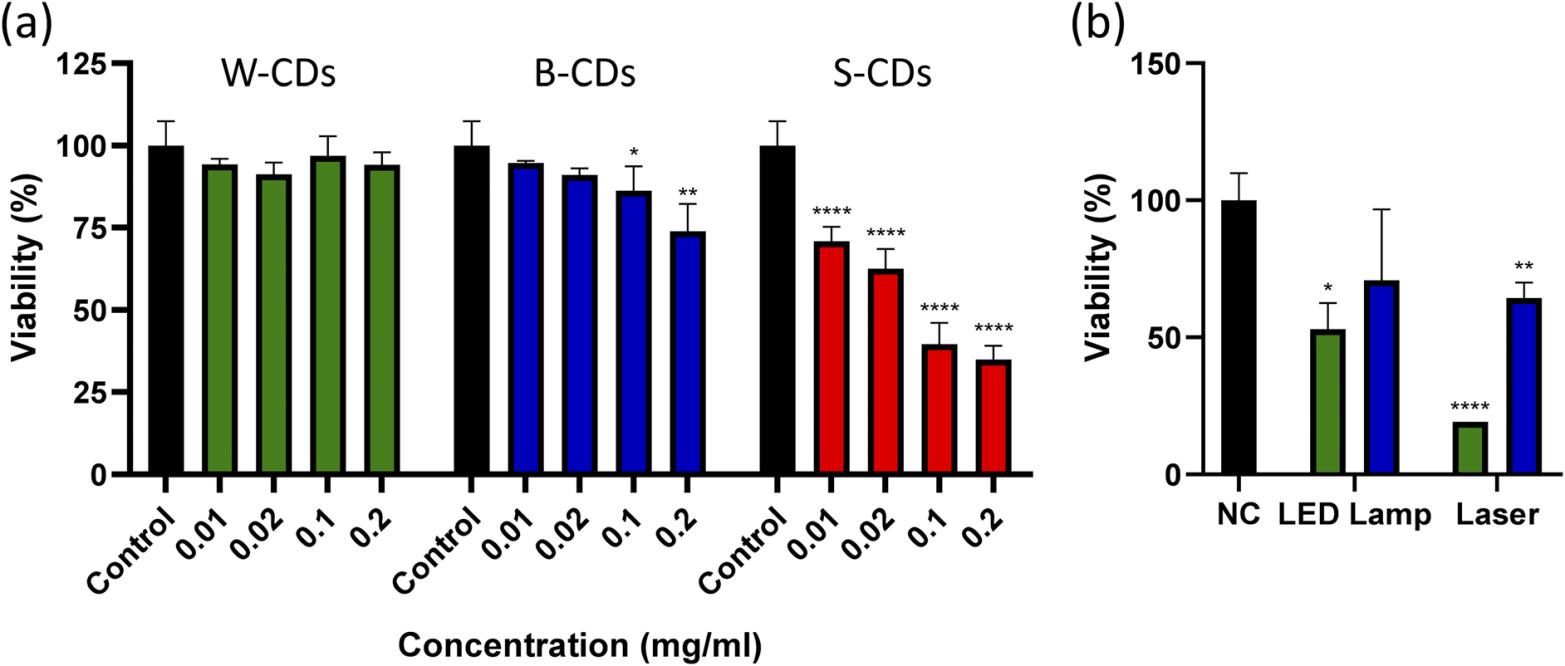
(a) Dark cytotoxicity assays of SK-Mel-28 cells treated with W-CDs (green), B-CDs (blue) and S-CDs (red). (b) Irradiation of SK-Mel-28 cells treated with W-CDs (green) and B-CDs (blue) at 0.1 mg mL^−1^. The LED lamp irradiation was 450 nm for 10 min. The laser irradiation was 405 ± 10 nm for 1 min with an intensity of 50 mW. All viability assays were measured by MTT assay and these are shown as mean ± SD (*n* = 4) (*p < 0.05) (**p < 0.01) (***p < 0.001) (****p < 0.0001). NC = negative control.

No significant differences were observed in SK-Mel-28 cells irradiated with a LED lamp at 450 nm for 10 or 30 minutes. A 10 minute irradiation was established for subsequent assays. No significant differences were observed in SK-Mel-28 cells irradiated with laser at 405 nm among the different times, although a drop was detected at 5 and 10 minutes with high dispersion (Fig. S1). A 1 minute irradiation was selected for subsequent assays. In CDs-treated cells, optimal concentrations for subsequent assays were determined to be 0.1 mg mL^−1^ for watermelon and blueberry (the same concentration used in ABDA assays). No non-cytotoxic concentration of S-CDs was found (Fig. 6a). A significant decrease in cell viability was observed in cells treated with W-CDs and B-CDs upon irradiation with a 405 nm laser, as well as in cells treated with B-CDs irradiated with an LED lamp (Fig. 6b). As no temperature variations attributable to photothermal activity were observed for the CDs (Fig. S2), this effect is attributed to ROS generation.

## 4 Conclusions

It is observed that urea doping in the synthesis of CDs enhances their fluorescent properties but decreases their ability to generate reactive oxygen species. Therefore, an increase in the nitrogen content of the nanosystem, mainly in the form of pyrrolic and pyridinic nitrogen group, appears to be associated with higher fluorescence yields and lower singlet oxygen yields. In addition, all undoped CDs showed cytotoxic effects when irradiated at their maximum concentration. Furthermore, a quenching interaction between BSA and N-doped CDs was successfully observed, potentially involving a FRET-type mechanism, arising from the spectral overlap between the emission band of the protein and the excitation band of the CDs. This overlap results from the shift in the fluorescence spectra of the N-doped CDs, which leads to the observed spectral coincidence. In cell-based assays, laser treatment induced cytotoxicity in W-CDs and B-CDs at concentrations that did not exhibit intrinsic toxicity. Cytotoxicity under LED lamp irradiation was only observed in B-CDs. These concentrations could not be applied to S-CDs, as intrinsic cytotoxicity was observed at all tested concentrations. These results can be attributed to ROS generation through photodynamic activity, as reflected in the reported singlet oxygen yields. Taking into account these findings, this study could provide pathways for the practical implementation of fruit-derived CDs in nanomedicine. The high fluorescence and low phototoxicity of N-doped CD make them suitable for long-term cell tracking and diagnostic bioimaging. Conversely, the high singlet oxygen yield of undoped CD allows for their implementation in targeted PDT or as light-activated antimicrobial agents. The strategic combination of these systems enables the development of versatile theranostic platforms where optical monitoring and therapeutic action can be independently tuned. Future work will investigate additional dopants in combination with nitrogen, including phosphorus. Moreover, formulations combining nitrogen-doped watermelon-derived CQDs and undoped blueberry-derived CQDs will be evaluated to maximize fluorescence and ROS generation.

## Author contributions

M. L. V., methodology, software, validation, formal analysis, investigation, data curation, writing—original draft, visualization; M. N. C., review & editing, supervision, project administration, funding acquisition; M. B. R. A., methodology; A. A. R., methodology, writing, visualization; J. M., conceptualization, writing—original draft preparation, review & editing, supervision, project administration, funding acquisition; C. R. L., conceptualization, review & editing, supervision, project administration, funding acquisition.

## Conflicts of interest

There are no conflicts to declare.

## Supplementary Material

RA-016-D6RA01035K-s001

RA-016-D6RA01035K-s002

RA-016-D6RA01035K-s003

RA-016-D6RA01035K-s004

RA-016-D6RA01035K-s005

RA-016-D6RA01035K-s006

RA-016-D6RA01035K-s007

## Data Availability

The authors confirm that the data supporting the findings of this study are available within the article and the accompanying supplementary information (SI). Supplementary information: figures of viability of cell culture after irradiation with different light sources, temperature rise of CDs upon irradiation, fluorescence spectra, excitation–emission matrices, and tables with raw zeta potential, FTIR, XPS, and EEM measurements. See DOI: https://doi.org/10.1039/d6ra01035k.
