# Supplementary figures and images for: Carbon dots from natural sources as theranostic agents: integrating fluorescence and ROS generation for photodynamic therapy
Source: RSC Adv. 2026 Apr 22;16(23):21142–51. doi: 10.1039/d6ra01035k (PMC13102146; doi:10.1039/d6ra01035k)

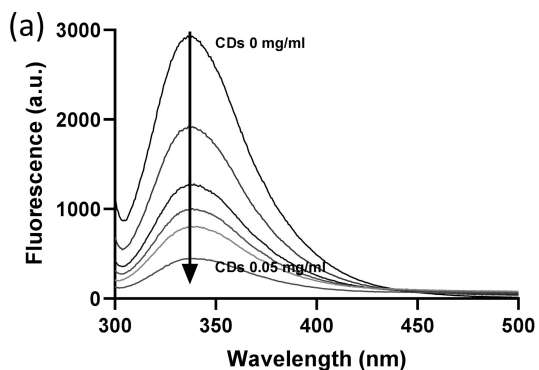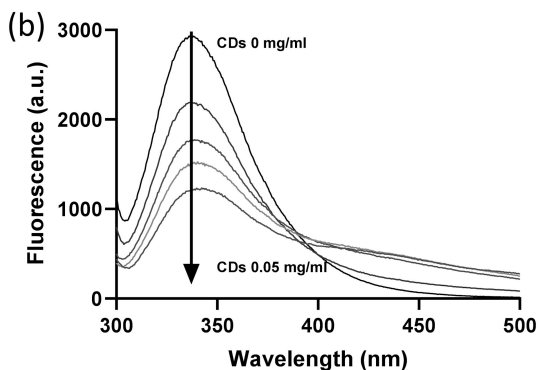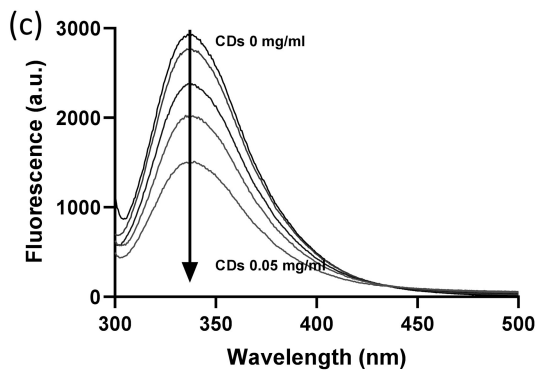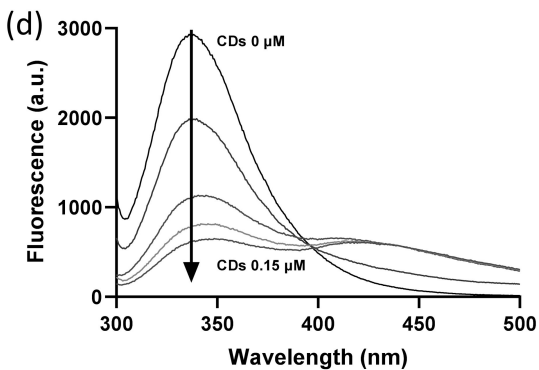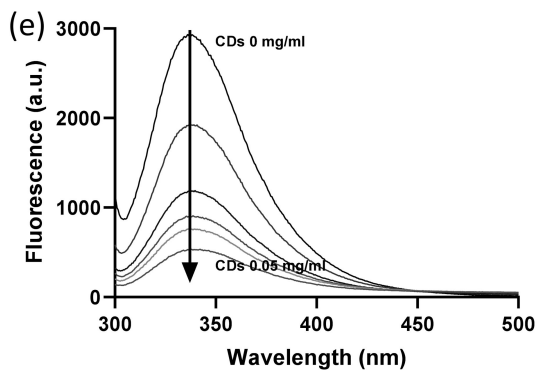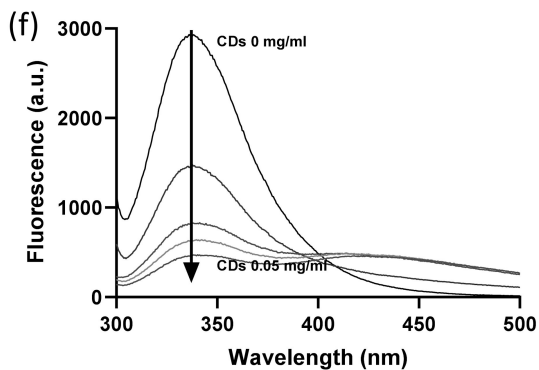

Supplement: RA-016-D6RA01035K-s001 [file RA-016-D6RA01035K-s001.pdf]

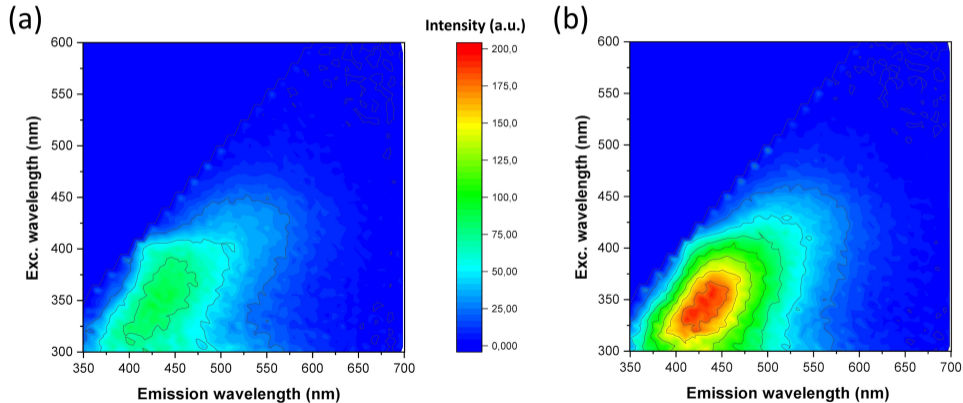

Supplement: RA-016-D6RA01035K-s002 [file RA-016-D6RA01035K-s002.pdf]

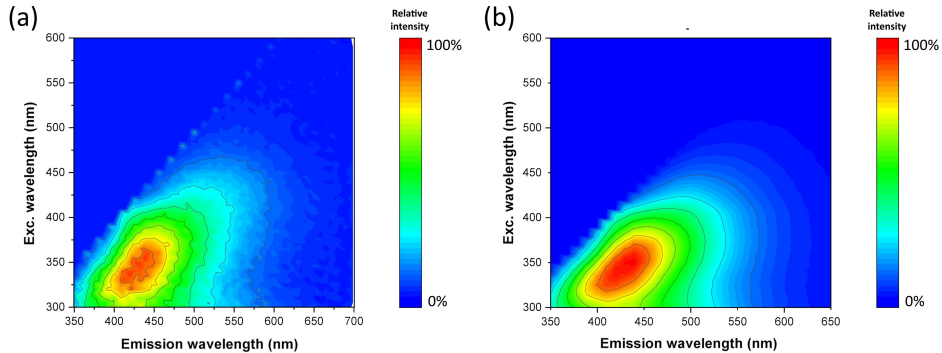

Supplement: RA-016-D6RA01035K-s003 [file RA-016-D6RA01035K-s003.pdf]

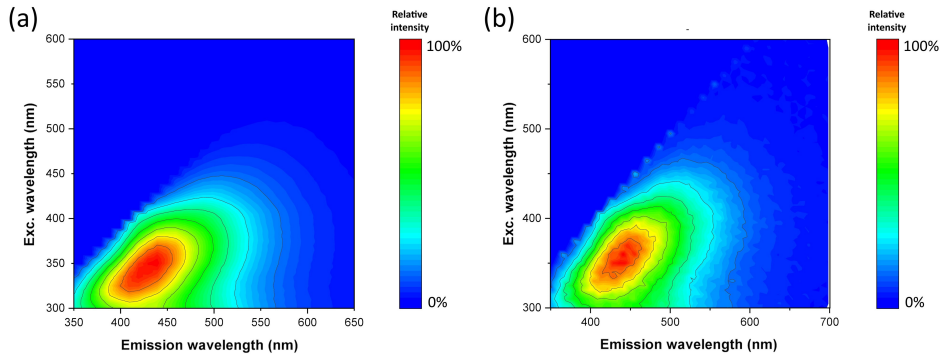

Supplement: RA-016-D6RA01035K-s004 [file RA-016-D6RA01035K-s004.pdf]

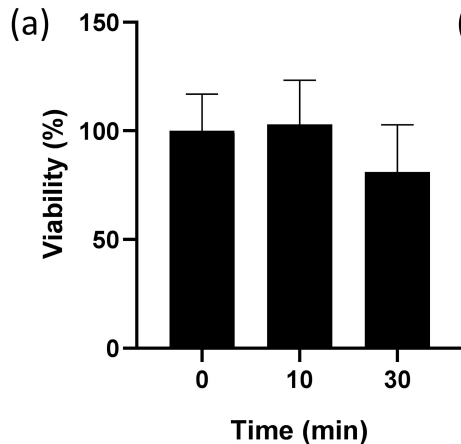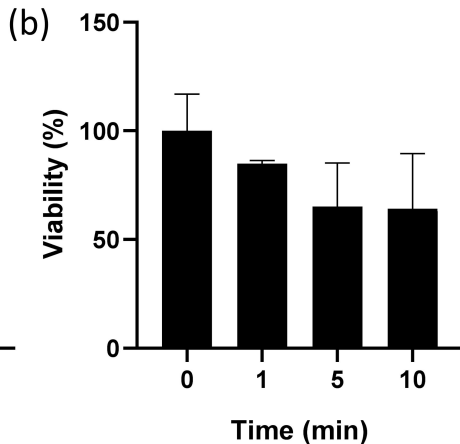

Supplement: RA-016-D6RA01035K-s005 [file RA-016-D6RA01035K-s005.pdf]

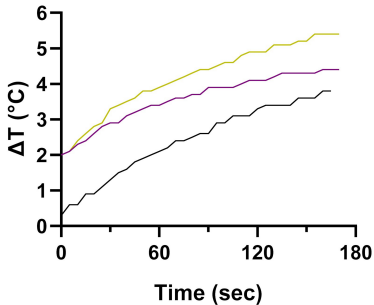

Supplement: RA-016-D6RA01035K-s006 [file RA-016-D6RA01035K-s006.pdf]
