# Supplementary material for: Carbon dots from natural sources as theranostic agents: integrating fluorescence and ROS generation for photodynamic therapy
Source: RSC Adv. 2026 Apr 22;16(23):21142–51. doi: 10.1039/d6ra01035k (PMC13102146; doi:10.1039/d6ra01035k)
Supplement: RA-016-D6RA01035K-s007 [file RA-016-D6RA01035K-s007.pdf]

Please do not adjust margins

PAPER

Supplementary Information for "*Carbon dots from natural sources as theranostic agents: integrating fluorescence and ROS generation for photodynamic therapy*"

Authors: Martín Lemos Vilches, M. Natalia Calienni, María Belén Rivas Aiello, Aldo A. Rubert, Jorge Montanari, Cristian R. Lillo.

Table of Contents

Figure S1. 2
Figure S2. 2
Figure S3. 3
Figure S4. 4
Figure S5. 4
Figure S6. 4
Figure S5. 4
Table S1. 5
Table S2. 5
Table S3. 57
Table S4. 76
Table S5. 106
Table S6. 125
Table S7. 138
Table S8. 154
Table S9. 168

Please do not adjust margins

Please do not adjust margins

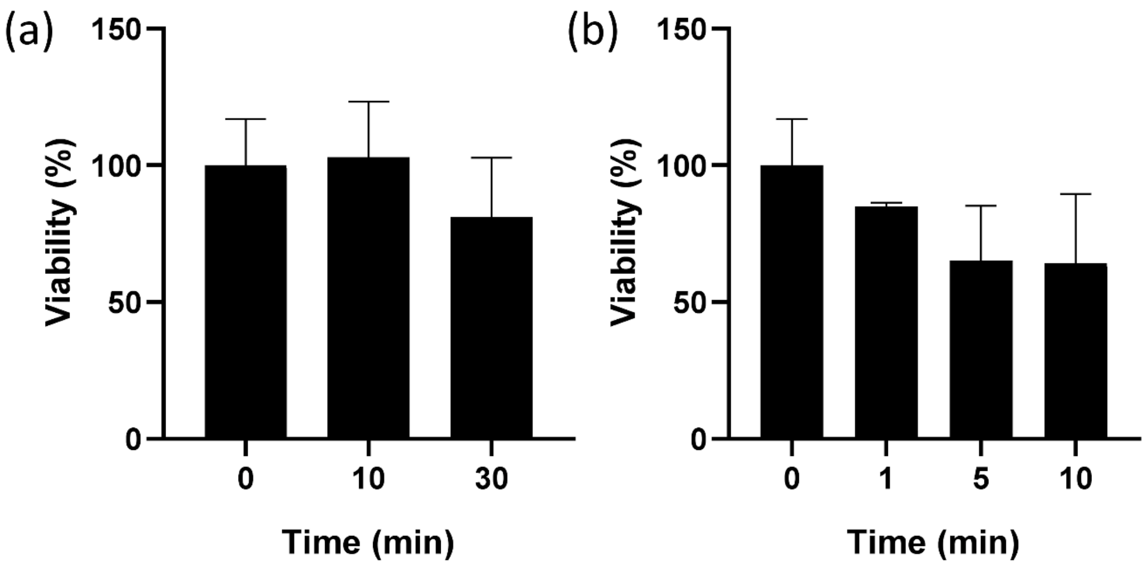

Fig. S1 (a) Irradiation of untreated SK-Mel-28 cells with a LED lamp at 450 nm to determine the minimum inhibition time. (b) Irradiation of untreated SK-Mel-28 cells with a laser at 405 nm ± 10 (50 mW) to determine the minimum inhibition time.

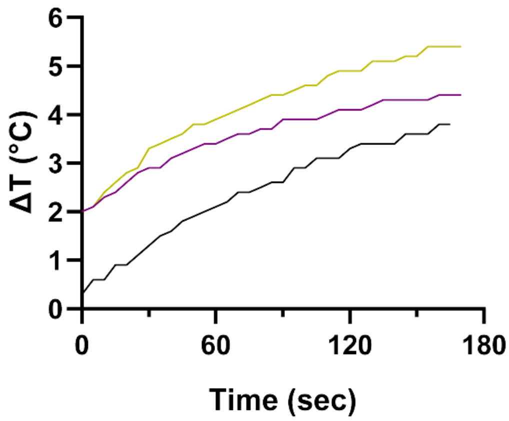

Fig. S2 Temperature rise in suspensions of doped blueberry-derived CDs at concentrations of 0.2 mg/ml (yellow) and 0.02 mg/ml (purple) under irradiation with a 806 nm laser at 505 W/cm².

 This journal is © The Royal Society of Chemistry 20xx

Please do not adjust margins

Please do not adjust margins

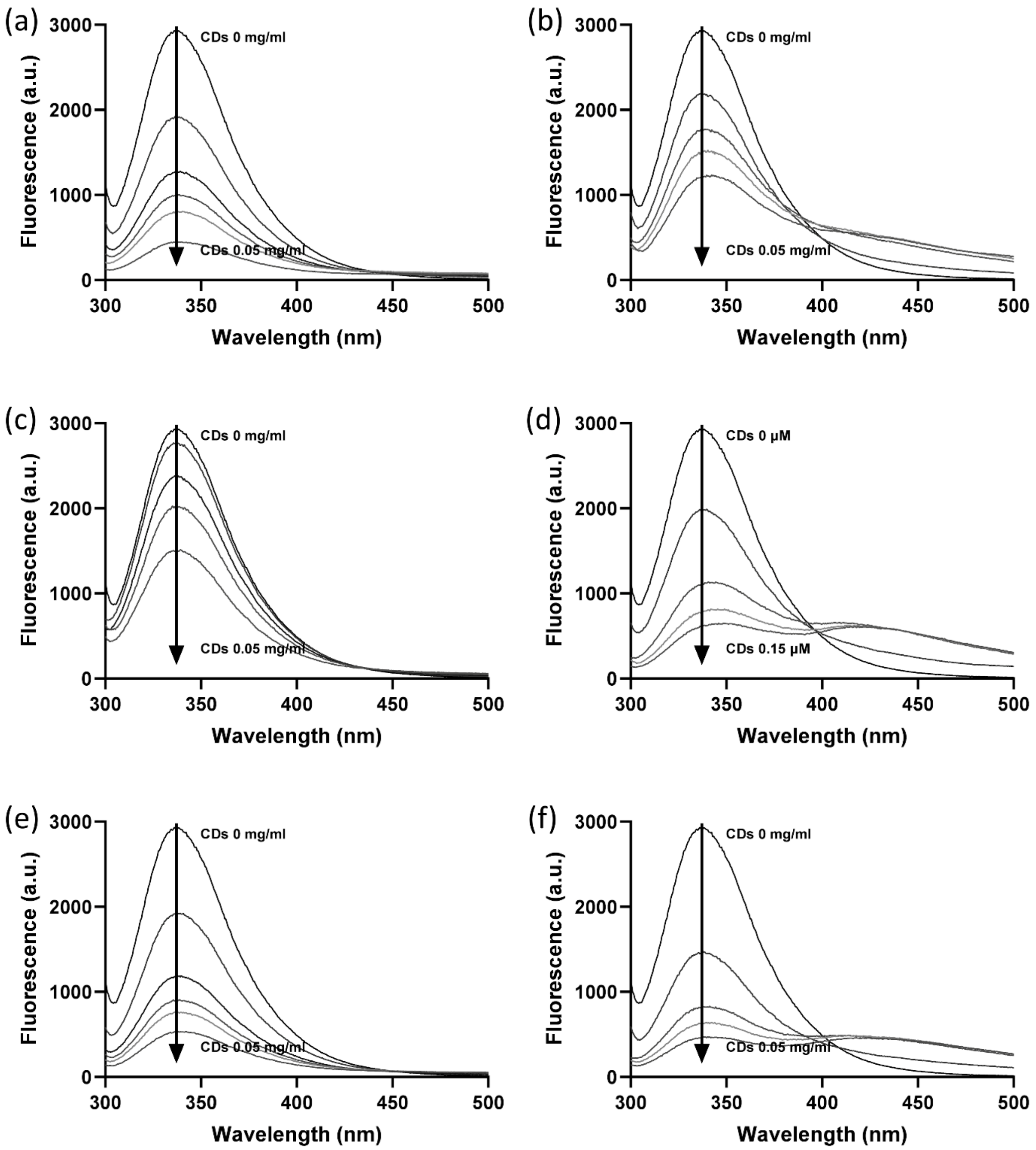

Fig. S3 Fluorescence spectrum of BSA at 5 µM with (a) non-doped watermelon, (b) doped watermelon, (c) non-doped blueberry, (d) doped blueberry, (e) non-doped strawberry and (f) doped strawberry CDs in different concentrations. Fluorescence is shown as mean (n = 4).

Please do not adjust margins

Please do not adjust margins

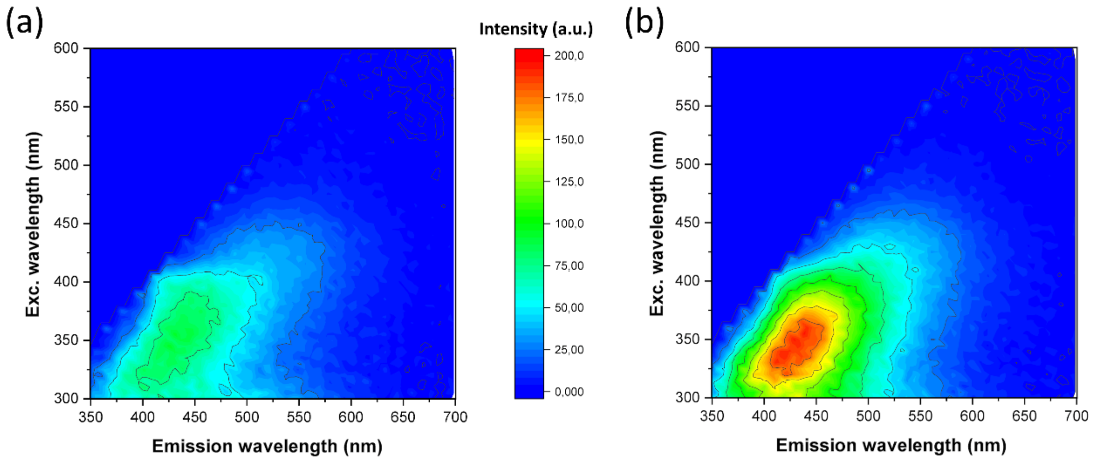

Fig. S4 Excitation–emission matrices of dW-CDs aqueous suspensions with 10% (a) and 30% (b) N-doping.

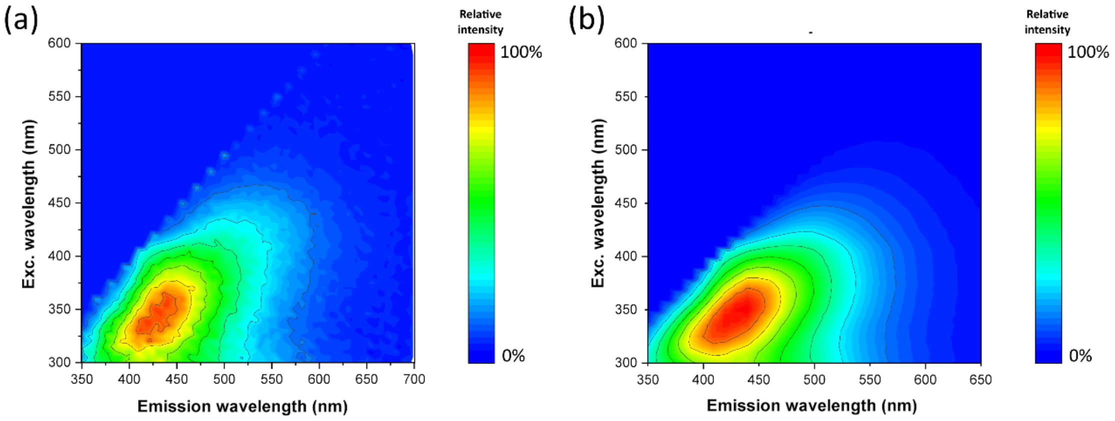

Fig. S5 Excitation–emission matrices of freshly prepared dW-CDs aqueous suspension batches produced several years apart. Subfigure (a) was prepared 3 years after subfigure (b). Fluorescence intensities were normalized to the maximum intensity of each EEM and expressed as relative percentage.

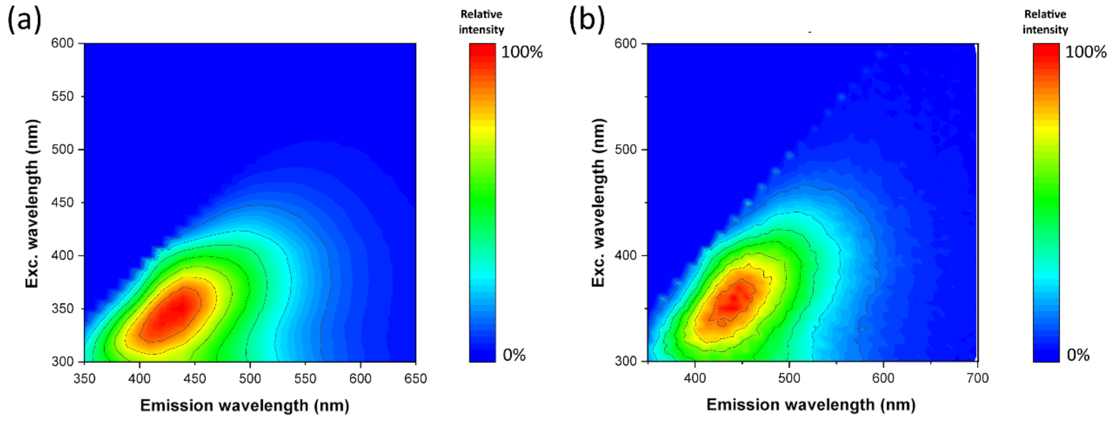

Fig. S6 Excitation–emission matrices of the same batch of dW-CDs aqueous suspension measured several years apart: (a) freshly prepared sample and (b) sample measured 3 years after preparation. Fluorescence intensities were normalized to the maximum intensity of each EEM and expressed as relative percentage.

This journal is © The Royal Society of Chemistry 20xx

Please do not adjust margins

Please do not adjust margins

Table S1 Raw zeta potential measurements corresponding to Fig. 1c. Zeta potential values (mV) were obtained in triplicate for each sample prior to calculation of mean ± SD.

CDs	Watermelon			Blueberry			Strawberry		
No Doped	-4,549	-6,133	-4,593	-18,47	-19,16	-16,93	-34,54	-32,05	-34,1
Doped	-4,644	-5,935	-5,343	-12,56	-12,84	-12,77	-35,73	-30,52	-32,16

Table S2 Raw FTIR measurements corresponding to Fig. 1.d.

Wavenumber (cm^{-1})	Watermelon	Doped watermelon	Blueberry	Doped blueberry	Strawberry	Doped strawberry
515,6196	291,93	293,85	203,09	196,63	96,74	92,05
517,0479	302,18	299,48	215,84	205,15	98,11	100,28
518,4763	310,01	303,79	225,6	211,6	99,12	106,56
519,9045	315,24	306,65	232,17	215,79	99,73	110,69
521,3329	317,68	307,98	235,29	217,59	99,91	112,54
522,7612	317,07	307,68	234,66	216,85	99,64	111,92
524,1895	313,2	305,61	230,01	213,34	98,88	108,67
525,6178	305,92	301,68	221,13	206,93	97,6	102,68
527,0461	295,06	295,78	207,84	197,48	95,79	93,76
528,4744	280,42	287,8	189,9	184,82	93,38	81,75
529,9027	280,34	287,82	189,87	184,68	93,23	81,48
531,331	280,33	287,78	189,68	184,55	93,07	81,12
532,7593	280,28	287,78	189,42	184,38	92,97	80,76
534,1876	280,21	287,86	189,23	184,14	92,95	80,46
535,616	280,07	287,88	189,02	183,91	92,92	80,24
537,0443	279,94	287,88	188,78	183,8	92,86	80,14
538,4726	279,83	287,89	188,63	183,73	92,81	80,07
539,9009	279,68	287,92	188,54	183,63	92,73	79,99
541,3292	279,54	288	188,45	183,54	92,65	79,88
542,7575	279,41	288,05	188,36	183,45	92,56	79,73
544,1858	279,33	288,09	188,28	183,37	92,47	79,65
545,6141	279,37	288,17	188,24	183,33	92,42	79,67
547,0424	279,36	288,22	188,23	183,31	92,36	79,72
548,4707	279,29	288,25	188,17	183,29	92,31	79,75
549,899	279,22	288,33	188,01	183,23	92,26	79,72
551,3273	279,11	288,43	187,85	183,15	92,18	79,64
552,7557	278,99	288,51	187,75	183,1	92,11	79,61
554,184	278,87	288,59	187,66	183,05	92,07	79,59
555,6123	278,69	288,68	187,59	182,98	92,04	79,58
557,0406	278,55	288,74	187,56	182,95	92,03	79,61
558,4689	278,48	288,8	187,47	182,9	92,01	79,64
559,8972	278,45	288,87	187,39	182,85	91,96	79,67
561,3255	278,5	288,91	187,4	182,81	91,9	79,75
562,7538	278,54	288,92	187,46	182,73	91,81	79,81
564,1821	278,54	288,96	187,5	182,66	91,75	79,83
565,6104	278,53	288,96	187,48	182,62	91,7	79,87
567,0388	278,51	288,9	187,38	182,56	91,63	79,92

Please do not adjust margins

Please do not adjust margins

568,467	278,5	288,87	187,3	182,49	91,56	79,99
569,8954	278,5	288,87	187,24	182,42	91,46	80,04
571,3237	278,48	288,87	187,2	182,33	91,34	80,09
572,752	278,48	288,86	187,27	182,24	91,23	80,18
574,1803	278,49	288,84	187,36	182,14	91,1	80,29
575,6086	278,53	288,82	187,39	182,05	90,96	80,37
577,0369	278,55	288,8	187,41	181,96	90,8	80,42
578,4652	278,51	288,78	187,36	181,87	90,62	80,47
579,8936	278,44	288,76	187,25	181,79	90,45	80,51
581,3218	278,39	288,71	187,15	181,69	90,27	80,55
582,7501	278,39	288,68	187,09	181,61	90,06	80,61
584,1785	278,42	288,67	187,06	181,56	89,85	80,67
585,6068	278,43	288,64	187,02	181,51	89,62	80,75
587,0351	278,43	288,64	186,93	181,45	89,38	80,83
588,4634	278,41	288,65	186,82	181,37	89,14	80,89
589,8917	278,4	288,67	186,71	181,28	88,89	80,96
591,32	278,42	288,72	186,62	181,2	88,65	81,05
592,7483	278,47	288,8	186,55	181,14	88,42	81,17
594,1766	278,52	288,9	186,5	181,08	88,2	81,33
595,6049	278,58	289,01	186,48	181,05	87,99	81,48
597,0333	278,63	289,1	186,46	181,02	87,8	81,6
598,4615	278,69	289,18	186,42	180,98	87,61	81,73
599,8899	278,74	289,26	186,4	180,92	87,45	81,86
601,3182	278,76	289,34	186,36	180,81	87,3	81,95
602,7465	278,77	289,44	186,32	180,71	87,18	82,04
604,1748	278,78	289,54	186,29	180,61	87,08	82,14
605,6031	278,77	289,61	186,23	180,49	86,98	82,2
607,0314	278,8	289,69	186,22	180,41	86,89	82,28
608,4597	278,86	289,79	186,27	180,33	86,82	82,37
609,888	278,88	289,88	186,3	180,21	86,78	82,44
611,3163	278,94	289,93	186,34	180,11	86,78	82,51
612,7446	279,03	289,98	186,41	180,04	86,77	82,6
614,173	279,08	290,05	186,47	179,96	86,77	82,68
615,6013	279,13	290,08	186,52	179,89	86,86	82,75
617,0296	279,23	290,08	186,58	179,82	86,97	82,83
618,4579	279,32	290	186,59	179,73	87,08	82,89
619,8862	279,37	289,91	186,51	179,58	87,2	82,89
621,3145	279,42	289,86	186,5	179,42	87,37	82,89
622,7428	279,49	289,8	186,54	179,27	87,57	82,89
624,1711	279,56	289,76	186,58	179,1	87,78	82,89
625,5994	279,66	289,75	186,65	178,95	88	82,88
627,0277	279,77	289,74	186,73	178,82	88,23	82,88
628,4561	279,89	289,65	186,79	178,68	88,47	82,88
629,8843	280,02	289,56	186,9	178,57	88,72	82,91
631,3127	280,15	289,51	187,04	178,48	88,97	82,94
632,741	280,28	289,46	187,18	178,41	89,2	82,97
634,1693	280,41	289,39	187,34	178,35	89,43	83,02

 This journal is © The Royal Society of Chemistry 20xx

Please do not adjust margins

Please do not adjust margins

635,5976	280,55	289,28	187,56	178,31	89,65	83,08
637,0259	280,73	289,14	187,83	178,3	89,87	83,15
638,4542	280,93	288,98	188,13	178,31	90,06	83,23
639,8825	281,08	289	188,45	178,32	90,19	83,31
641,3109	281,23	289,11	188,78	178,35	90,3	83,4
642,7391	281,39	289,19	189,14	178,39	90,41	83,48
644,1674	281,57	289,23	189,51	178,43	90,49	83,55
645,5958	281,75	289,29	189,89	178,49	90,54	83,62
647,024	281,93	289,36	190,28	178,56	90,56	83,7
648,4524	282,12	289,42	190,65	178,63	90,57	83,77
649,8807	282,31	289,51	191,02	178,72	90,57	83,83
651,309	282,51	289,59	191,4	178,81	90,55	83,88
652,7373	282,7	289,64	191,78	178,89	90,53	83,91
654,1656	282,87	289,7	192,13	178,95	90,5	83,93
655,5939	283,05	289,76	192,47	179	90,47	83,94
657,0222	283,23	289,82	192,82	179,06	90,45	83,96
658,4506	283,38	289,86	193,15	179,1	90,44	83,96
659,8788	283,52	289,9	193,48	179,13	90,44	83,95
661,3071	283,68	289,91	193,83	179,17	90,46	83,95
662,7355	283,82	289,9	194,16	179,23	90,48	83,95
664,1638	283,97	289,89	194,52	179,3	90,52	83,97
665,5921	284,11	289,89	194,88	179,35	90,6	83,99
667,0204	284,19	289,85	195,19	179,37	90,68	83,96
668,4487	284,26	289,8	195,46	179,38	90,8	83,92
669,877	284,32	289,74	195,71	179,36	90,93	83,88
671,3053	284,33	289,66	195,92	179,29	91,09	83,81
672,7336	284,35	289,52	196,1	179,21	91,3	83,72
674,1619	284,36	289,39	196,21	179,1	91,48	83,61
675,5903	284,3	289,26	196,25	178,98	91,65	83,49
677,0186	284,23	289,21	196,33	178,84	91,86	83,37
678,4469	284,18	289,15	196,48	178,73	92,07	83,27
679,8752	284,11	289,09	196,55	178,6	92,27	83,18
681,3035	284,03	289,04	196,58	178,47	92,46	83,09
682,7318	283,94	288,99	196,61	178,34	92,65	83,02
684,1601	283,83	288,87	196,62	178,21	92,84	82,94
685,5884	283,7	288,76	196,61	178,07	93,02	82,86
687,0167	283,56	288,69	196,61	177,92	93,18	82,8
688,445	283,42	288,65	196,62	177,77	93,34	82,73
689,8734	283,29	288,58	196,63	177,64	93,48	82,66
691,3016	283,18	288,51	196,67	177,52	93,6	82,62
692,73	283,08	288,46	196,72	177,41	93,7	82,59
694,1583	282,97	288,46	196,75	177,3	93,77	82,54
695,5866	282,86	288,55	196,8	177,19	93,82	82,49
697,0149	282,75	288,74	196,86	177,08	93,85	82,46
698,4432	282,67	288,81	196,91	176,98	93,9	82,43
699,8715	282,62	288,81	196,97	176,88	93,94	82,41
701,2998	282,58	288,82	197,05	176,79	93,94	82,38

This journal is © The Royal Society of Chemistry 20xx

Please do not adjust margins

Please do not adjust margins

702,7281	282,55	288,86	197,14	176,71	93,92	82,36
704,1564	282,53	288,91	197,23	176,64	93,91	82,35
705,5847	282,52	288,95	197,31	176,58	93,91	82,33
707,0131	282,52	289	197,4	176,52	93,91	82,31
708,4413	282,52	289,01	197,5	176,45	93,9	82,29
709,8697	282,51	289,02	197,6	176,39	93,91	82,26
711,298	282,5	289,05	197,73	176,32	93,92	82,23
712,7263	282,49	289,06	197,87	176,26	93,95	82,2
714,1546	282,47	289,04	198	176,17	93,98	82,15
715,5829	282,44	289,02	198,12	176,07	94,02	82,09
717,0112	282,45	288,98	198,29	175,99	94,06	82,04
718,4395	282,47	288,92	198,48	175,94	94,11	82,01
719,8679	282,49	288,86	198,67	175,86	94,17	81,97
721,2961	282,5	288,79	198,87	175,76	94,23	81,91
722,7244	282,52	288,74	199,08	175,65	94,27	81,84
724,1528	282,52	288,68	199,27	175,53	94,32	81,73
725,5811	282,5	288,61	199,38	175,38	94,38	81,58
727,0094	282,44	288,52	199,43	175,16	94,42	81,39
728,4377	282,35	288,41	199,48	174,93	94,47	81,2
729,866	282,26	288,29	199,59	174,71	94,52	81
731,2943	282,13	288,16	199,69	174,47	94,57	80,79
732,7226	281,97	288,04	199,74	174,22	94,6	80,56
734,1509	281,8	287,94	199,78	173,98	94,62	80,32
735,5792	281,64	287,83	199,84	173,76	94,63	80,1
737,0076	281,47	287,72	199,92	173,54	94,62	79,88
738,4359	281,29	287,64	199,98	173,31	94,61	79,67
739,8641	281,08	287,55	199,99	173,1	94,6	79,46
741,2925	280,85	287,44	200	172,9	94,57	79,25
742,7208	280,61	287,34	199,99	172,72	94,51	79,03
744,1491	280,34	287,24	199,93	172,52	94,41	78,78
745,5774	280,09	287,16	199,83	172,33	94,3	78,52
747,0057	279,85	287,08	199,72	172,16	94,16	78,26
748,434	279,57	287	199,57	171,99	93,99	78,01
749,8623	279,29	286,93	199,4	171,84	93,81	77,75
751,2906	279	286,87	199,19	171,69	93,64	77,49
752,7189	278,72	286,84	198,93	171,56	93,46	77,23
754,1473	278,45	286,82	198,65	171,44	93,26	76,99
755,5756	278,18	286,81	198,35	171,32	93,01	76,76
757,0038	277,92	286,81	198,04	171,24	92,75	76,53
758,4322	277,68	286,82	197,7	171,2	92,49	76,33
759,8605	277,44	286,84	197,34	171,16	92,24	76,14
761,2888	277,22	286,86	196,96	171,17	92,01	75,98
762,7171	277,03	286,91	196,61	171,2	91,78	75,84
764,1454	276,83	286,96	196,27	171,22	91,54	75,7
765,5737	276,62	287,03	195,95	171,26	91,34	75,57
767,002	276,42	287,11	195,66	171,33	91,17	75,47
768,4304	276,25	287,18	195,43	171,41	91,01	75,4

 This journal is © The Royal Society of Chemistry 20xx

Please do not adjust margins

Please do not adjust margins

769,8586	276,12	287,24	195,24	171,5	90,88	75,35
771,287	275,99	287,29	195,08	171,6	90,77	75,32
772,7153	275,89	287,33	194,98	171,71	90,68	75,32
774,1436	275,82	287,38	194,93	171,83	90,63	75,34
775,5719	275,75	287,43	194,94	171,94	90,59	75,36
777,0002	275,69	287,49	194,97	172,04	90,56	75,39
778,4285	275,65	287,56	195,03	172,15	90,55	75,43
779,8568	275,62	287,63	195,13	172,27	90,55	75,48
781,2852	275,59	287,69	195,24	172,38	90,6	75,53
782,7134	275,54	287,73	195,35	172,49	90,65	75,58
784,1417	275,52	287,78	195,55	172,62	90,73	75,66
785,5701	275,54	287,84	195,83	172,79	90,82	75,78
786,9984	275,56	287,91	196,11	172,99	90,93	75,9
788,4267	275,56	287,99	196,35	173,2	91,02	76,03
789,855	275,6	288,07	196,61	173,45	91,11	76,2
791,2833	275,67	288,15	196,92	173,75	91,19	76,4
792,7116	275,75	288,23	197,23	174,07	91,26	76,63
794,1399	275,83	288,33	197,5	174,41	91,31	76,85
795,5682	275,92	288,45	197,71	174,75	91,34	77,06
796,9965	276,03	288,54	197,88	175,11	91,35	77,25
798,4249	276,14	288,63	198	175,46	91,31	77,41
799,8531	276,27	288,72	198,07	175,82	91,23	77,56
801,2814	276,45	288,8	198,12	176,19	91,13	77,71
802,7098	276,67	288,87	198,17	176,57	91,01	77,86
804,1381	276,92	288,93	198,21	176,97	90,88	78,01
805,5664	277,19	288,96	198,26	177,38	90,76	78,17
806,9947	277,5	288,99	198,27	177,79	90,65	78,32
808,423	277,84	289,01	198,25	178,21	90,54	78,48
809,8513	278,21	289,01	198,23	178,65	90,41	78,66
811,2796	278,63	289	198,22	179,09	90,27	78,85
812,7079	279,05	288,98	198,2	179,55	90,15	79,04
814,1362	279,5	288,95	198,17	180,02	90,03	79,23
815,5646	279,98	288,93	198,15	180,48	89,91	79,43
816,9929	280,49	288,91	198,15	180,93	89,8	79,64
818,4211	281,02	288,9	198,19	181,38	89,68	79,87
819,8495	281,56	288,89	198,24	181,81	89,55	80,09
821,2778	282,08	288,88	198,29	182,21	89,43	80,3
822,7061	282,6	288,87	198,34	182,61	89,33	80,53
824,1344	283,15	288,86	198,41	183	89,23	80,77
825,5627	283,69	288,85	198,5	183,35	89,14	81,01
826,991	284,24	288,84	198,62	183,7	89,08	81,26
828,4193	284,76	288,83	198,72	184,02	89,05	81,52
829,8477	285,26	288,83	198,81	184,32	89,05	81,78
831,2759	285,74	288,85	198,92	184,6	89,09	82,04
832,7043	286,2	288,88	199,01	184,87	89,17	82,31
834,1326	286,64	288,91	199,08	185,12	89,29	82,59
835,5609	287,06	288,95	199,13	185,36	89,46	82,87

This journal is © The Royal Society of Chemistry 20xx

Please do not adjust margins

Please do not adjust margins

836,9892	287,44	288,99	199,16	185,58	89,66	83,15
838,4175	287,81	289,05	199,19	185,79	89,91	83,44
839,8458	288,13	289,12	199,17	185,98	90,19	83,72
841,2741	288,39	289,17	199,12	186,14	90,5	84
842,7025	288,63	289,24	199,04	186,27	90,84	84,27
844,1307	288,83	289,3	198,96	186,39	91,2	84,55
845,559	288,99	289,35	198,86	186,5	91,56	84,83
846,9874	289,13	289,42	198,74	186,58	91,93	85,1
848,4156	289,23	289,48	198,62	186,65	92,3	85,37
849,844	289,31	289,55	198,51	186,72	92,67	85,62
851,2723	289,35	289,61	198,38	186,76	93,07	85,85
852,7006	289,35	289,68	198,19	186,79	93,47	86,06
854,1289	289,32	289,73	197,91	186,85	93,89	86,25
855,5572	289,27	289,79	197,59	186,93	94,31	86,45
856,9855	289,23	289,85	197,24	187,04	94,73	86,66
858,4138	289,2	289,91	196,86	187,21	95,15	86,87
859,8422	289,17	289,97	196,42	187,4	95,57	87,08
861,2704	289,14	290,02	195,96	187,61	95,98	87,29
862,6987	289,12	290,08	195,48	187,83	96,35	87,5
864,1271	289,1	290,15	194,97	188,07	96,67	87,72
865,5554	289,1	290,25	194,45	188,32	96,93	87,93
866,9837	289,1	290,37	193,94	188,56	97,12	88,15
868,412	289,12	290,51	193,42	188,79	97,21	88,36
869,8403	289,14	290,68	192,91	189,02	97,23	88,55
871,2686	289,16	290,86	192,43	189,24	97,16	88,74
872,6969	289,19	291,05	191,96	189,44	97	88,93
874,1252	289,25	291,27	191,52	189,64	96,78	89,13
875,5535	289,31	291,49	191,11	189,84	96,49	89,32
876,9819	289,4	291,71	190,73	190,03	96,16	89,52
878,4102	289,49	291,93	190,39	190,21	95,78	89,71
879,8384	289,59	292,17	190,08	190,37	95,39	89,9
881,2668	289,71	292,39	189,81	190,52	95,01	90,08
882,6951	289,82	292,6	189,59	190,66	94,63	90,26
884,1234	289,94	292,78	189,41	190,78	94,27	90,45
885,5517	290,05	292,96	189,26	190,89	93,94	90,62
886,98	290,17	293,11	189,16	190,99	93,63	90,79
888,4083	290,29	293,23	189,1	191,07	93,36	90,96
889,8366	290,42	293,31	189,08	191,13	93,14	91,12
891,265	290,54	293,34	189,09	191,17	92,95	91,27
892,6932	290,66	293,31	189,13	191,18	92,8	91,41
894,1216	290,76	293,21	189,22	191,17	92,69	91,53
895,5499	290,84	293,04	189,34	191,13	92,61	91,64
896,9781	290,91	292,79	189,49	191,07	92,59	91,74
898,4065	290,94	292,45	189,68	190,97	92,6	91,8
899,8348	290,96	292,05	189,9	190,84	92,66	91,83
901,2631	290,96	291,58	190,14	190,7	92,76	91,84
902,6914	290,92	291,05	190,4	190,53	92,91	91,83

This journal is © The Royal Society of Chemistry 20xx

Please do not adjust margins

Please do not adjust margins

904,1198	290,86	290,47	190,68	190,36	93,12	91,81
905,548	290,79	289,86	190,97	190,21	93,38	91,77
906,9763	290,69	289,22	191,2	190,06	93,68	91,72
908,4047	290,55	288,57	191,34	189,91	94,02	91,64
909,8329	290,39	287,93	191,41	189,79	94,37	91,56
911,2613	290,25	287,31	191,44	189,7	94,74	91,49
912,6896	290,11	286,69	191,45	189,63	95,1	91,43
914,1179	289,96	286,09	191,44	189,57	95,44	91,37
915,5462	289,81	285,52	191,4	189,5	95,76	91,3
916,9745	289,66	284,97	191,35	189,42	96,03	91,23
918,4028	289,49	284,44	191,28	189,31	96,26	91,16
919,8311	289,32	283,97	191,22	189,19	96,45	91,08
921,2595	289,13	283,54	191,13	189,04	96,59	91
922,6877	288,93	283,17	191,03	188,88	96,68	90,91
924,116	288,73	282,84	190,94	188,71	96,73	90,83
925,5444	288,5	282,57	190,83	188,52	96,73	90,74
926,9727	288,28	282,36	190,73	188,32	96,7	90,66
928,401	288,05	282,21	190,63	188,11	96,64	90,59
929,8293	287,82	282,12	190,52	187,9	96,54	90,53
931,2576	287,59	282,08	190,41	187,67	96,42	90,48
932,6859	287,36	282,11	190,29	187,42	96,27	90,43
934,1142	287,11	282,19	190,14	187,16	96,1	90,39
935,5425	286,85	282,32	189,96	186,88	95,9	90,35
936,9708	286,59	282,5	189,76	186,6	95,66	90,32
938,3992	286,31	282,71	189,53	186,31	95,39	90,29
939,8275	286,02	282,94	189,27	186,01	95,08	90,27
941,2557	285,69	283,19	188,96	185,7	94,74	90,25
942,6841	285,35	283,45	188,62	185,39	94,36	90,24
944,1124	284,99	283,7	188,23	185,07	93,96	90,23
945,5407	284,61	283,92	187,82	184,74	93,53	90,22
946,969	284,21	284,1	187,38	184,4	93,07	90,21
948,3973	283,8	284,24	186,9	184,05	92,59	90,18
949,8256	283,35	284,31	186,37	183,69	92,09	90,14
951,2539	282,88	284,3	185,81	183,32	91,56	90,1
952,6823	282,39	284,2	185,2	182,94	91,01	90,03
954,1105	281,87	284	184,54	182,57	90,43	89,95
955,5389	281,35	283,69	183,84	182,18	89,82	89,84
956,9672	280,82	283,25	183,09	181,8	89,18	89,69
958,3954	280,28	282,68	182,29	181,42	88,49	89,52
959,8238	279,73	281,95	181,42	181,03	87,76	89,3
961,2521	279,17	281,06	180,5	180,65	86,97	89,04
962,6804	278,6	280,02	179,5	180,27	86,13	88,73
964,1087	278,02	278,81	178,45	179,87	85,22	88,37
965,537	277,45	277,47	177,37	179,49	84,27	87,96
966,9653	276,87	275,99	176,26	179,11	83,28	87,49
968,3936	276,28	274,38	175,13	178,7	82,25	86,96
969,822	275,68	272,65	173,98	178,3	81,21	86,36

This journal is © The Royal Society of Chemistry 20xx

Please do not adjust margins

Please do not adjust margins

971,2502	275,09	270,82	172,85	177,89	80,16	85,71
972,6786	274,5	268,92	171,72	177,49	79,12	85,01
974,1069	273,91	266,97	170,61	177,08	78,09	84,27
975,5352	273,33	264,99	169,52	176,66	77,06	83,5
976,9635	272,76	263,02	168,44	176,23	76,03	82,71
978,3918	272,19	261,1	167,36	175,81	74,99	81,9
979,8201	271,62	259,25	166,27	175,37	73,93	81,1
981,2484	271,04	257,49	165,17	174,91	72,85	80,29
982,6768	270,44	255,83	164,03	174,43	71,74	79,49
984,105	269,81	254,29	162,85	173,93	70,59	78,71
985,5333	269,15	252,88	161,62	173,4	69,4	77,95
986,9617	268,45	251,6	160,33	172,82	68,18	77,2
988,39	267,71	250,44	159	172,22	66,91	76,48
989,8183	266,93	249,43	157,62	171,6	65,59	75,8
991,2466	266,1	248,55	156,2	170,96	64,24	75,16
992,6749	265,22	247,82	154,74	170,29	62,84	74,56
994,1032	264,29	247,24	153,24	169,6	61,42	74,01
995,5315	263,29	246,8	151,7	168,87	59,97	73,5
996,9598	262,23	246,52	150,13	168,13	58,51	73,04
998,3881	261,12	246,37	148,53	167,36	57,04	72,64
999,8165	259,95	246,34	146,9	166,58	55,56	72,28
1001,245	258,76	246,41	145,24	165,78	54,09	71,97
1002,673	257,53	246,56	143,56	164,96	52,6	71,69
1004,101	256,25	246,77	141,84	164,12	51,11	71,44
1005,53	254,95	247,01	140,09	163,26	49,62	71,22
1006,958	253,61	247,26	138,32	162,38	48,14	71
1008,386	252,25	247,5	136,54	161,49	46,67	70,79
1009,815	250,89	247,69	134,75	160,59	45,22	70,59
1011,243	249,51	247,82	132,95	159,67	43,8	70,35
1012,671	248,13	247,86	131,19	158,73	42,42	70,1
1014,1	246,74	247,8	129,46	157,78	41,1	69,81
1015,528	245,34	247,65	127,77	156,82	39,84	69,48
1016,956	243,95	247,38	126,12	155,88	38,67	69,12
1018,384	242,57	247	124,51	154,97	37,6	68,72
1019,813	241,23	246,51	122,95	154,11	36,65	68,28
1021,241	239,93	245,92	121,43	153,32	35,85	67,81
1022,669	238,69	245,23	119,91	152,62	35,19	67,28
1024,098	237,51	244,47	118,38	152,03	34,69	66,73
1025,526	236,44	243,64	116,86	151,59	34,35	66,15
1026,954	235,49	242,75	115,33	151,3	34,16	65,57
1028,383	234,68	241,83	113,79	151,17	34,11	64,99
1029,811	234	240,89	112,25	151,15	34,19	64,42
1031,239	233,44	239,97	110,73	151,24	34,37	63,88
1032,668	233,01	239,1	109,27	151,41	34,63	63,35
1034,096	232,67	238,27	107,87	151,63	34,96	62,85
1035,524	232,43	237,52	106,57	151,88	35,35	62,37
1036,953	232,25	236,83	105,37	152,14	35,79	61,92

This journal is © The Royal Society of Chemistry 20xx

Please do not adjust margins

Please do not adjust margins

1038,381	232,11	236,23	104,27	152,38	36,26	61,49
1039,809	232,04	235,7	103,3	152,62	36,78	61,1
1041,237	232	235,23	102,46	152,83	37,32	60,75
1042,666	231,98	234,85	101,75	153,01	37,87	60,44
1044,094	231,99	234,55	101,18	153,14	38,43	60,16
1045,522	232,02	234,33	100,73	153,24	38,98	59,92
1046,951	232,05	234,22	100,41	153,28	39,51	59,73
1048,379	232,09	234,22	100,2	153,27	40	59,58
1049,807	232,11	234,3	100,06	153,2	40,44	59,46
1051,236	232,14	234,48	100	153,08	40,83	59,37
1052,664	232,15	234,74	100,01	152,9	41,14	59,31
1054,092	232,16	235,07	100,07	152,68	41,39	59,3
1055,521	232,17	235,48	100,19	152,39	41,59	59,31
1056,949	232,17	235,94	100,37	152,05	41,72	59,34
1058,377	232,18	236,48	100,63	151,67	41,83	59,4
1059,805	232,21	237,09	101	151,25	41,94	59,5
1061,234	232,24	237,77	101,45	150,8	42,06	59,62
1062,662	232,3	238,54	102,01	150,33	42,24	59,77
1064,09	232,36	239,39	102,66	149,85	42,5	59,96
1065,519	232,44	240,31	103,39	149,36	42,84	60,17
1066,947	232,56	241,28	104,23	148,89	43,29	60,41
1068,375	232,69	242,28	105,15	148,45	43,85	60,67
1069,804	232,87	243,32	106,16	148,06	44,53	60,97
1071,232	233,09	244,36	107,2	147,73	45,32	61,28
1072,66	233,36	245,39	108,24	147,48	46,2	61,61
1074,089	233,69	246,41	109,28	147,32	47,17	61,96
1075,517	234,1	247,39	110,29	147,27	48,21	62,32
1076,945	234,58	248,32	111,24	147,34	49,32	62,69
1078,373	235,15	249,22	112,13	147,52	50,5	63,07
1079,802	235,8	250,05	112,96	147,82	51,7	63,45
1081,23	236,56	250,84	113,74	148,23	52,95	63,84
1082,658	237,41	251,57	114,5	148,73	54,22	64,25
1084,087	238,32	252,25	115,26	149,27	55,51	64,66
1085,515	239,29	252,89	116,07	149,83	56,82	65,06
1086,943	240,3	253,5	116,96	150,38	58,13	65,47
1088,372	241,33	254,07	117,96	150,9	59,45	65,87
1089,8	242,38	254,61	119,08	151,38	60,78	66,25
1091,228	243,42	255,1	120,3	151,81	62,1	66,62
1092,656	244,46	255,56	121,6	152,2	63,41	66,99
1094,085	245,49	256	122,99	152,54	64,7	67,36
1095,513	246,5	256,38	124,43	152,84	65,96	67,7
1096,941	247,5	256,72	125,93	153,12	67,19	68,05
1098,37	248,46	257,01	127,44	153,35	68,37	68,37
1099,798	249,36	257,26	128,95	153,54	69,48	68,66
1101,226	250,24	257,48	130,46	153,71	70,54	68,95
1102,655	251,07	257,65	131,94	153,85	71,52	69,2
1104,083	251,85	257,78	133,38	153,96	72,43	69,43

This journal is © The Royal Society of Chemistry 20xx

Please do not adjust margins

Please do not adjust margins

1105,511	252,59	257,87	134,78	154,05	73,26	69,65
1106,94	253,27	257,92	136,14	154,12	74,02	69,85
1108,368	253,92	257,95	137,46	154,19	74,74	70,04
1109,796	254,53	257,97	138,74	154,25	75,42	70,22
1111,225	255,11	257,96	139,99	154,32	76,07	70,39
1112,653	255,67	257,97	141,21	154,4	76,72	70,57
1114,081	256,21	257,99	142,41	154,5	77,36	70,75
1115,51	256,74	258,04	143,6	154,64	78,03	70,95
1116,938	257,28	258,12	144,77	154,82	78,73	71,16
1118,366	257,79	258,23	145,87	155,02	79,44	71,38
1119,794	258,3	258,38	146,92	155,26	80,17	71,61
1121,223	258,82	258,57	147,92	155,54	80,9	71,85
1122,651	259,32	258,79	148,86	155,84	81,61	72,08
1124,079	259,83	259,05	149,73	156,18	82,3	72,33
1125,508	260,33	259,35	150,54	156,53	82,94	72,59
1126,936	260,83	259,68	151,29	156,88	83,52	72,85
1128,364	261,32	260,05	151,98	157,23	84,05	73,11
1129,793	261,79	260,45	152,62	157,57	84,5	73,36
1131,221	262,25	260,87	153,21	157,91	84,9	73,6
1132,649	262,68	261,31	153,77	158,23	85,25	73,84
1134,078	263,09	261,77	154,28	158,53	85,55	74,06
1135,506	263,47	262,26	154,76	158,81	85,81	74,26
1136,934	263,82	262,76	155,23	159,06	86,05	74,45
1138,362	264,13	263,28	155,68	159,27	86,28	74,61
1139,791	264,43	263,82	156,15	159,47	86,5	74,77
1141,219	264,68	264,38	156,62	159,62	86,72	74,91
1142,647	264,9	264,97	157,1	159,74	86,95	75,04
1144,076	265,11	265,57	157,61	159,84	87,18	75,15
1145,504	265,28	266,18	158,13	159,9	87,43	75,25
1146,932	265,41	266,81	158,66	159,96	87,69	75,33
1148,361	265,53	267,45	159,19	160,01	87,96	75,4
1149,789	265,62	268,09	159,73	160,05	88,23	75,46
1151,217	265,71	268,74	160,27	160,1	88,52	75,51
1152,646	265,77	269,37	160,8	160,15	88,81	75,55
1154,074	265,81	270	161,32	160,22	89,1	75,58
1155,502	265,84	270,61	161,83	160,31	89,38	75,61
1156,93	265,85	271,19	162,33	160,4	89,64	75,62
1158,359	265,85	271,75	162,8	160,49	89,89	75,62
1159,787	265,84	272,27	163,27	160,59	90,11	75,63
1161,215	265,82	272,74	163,71	160,68	90,3	75,63
1162,644	265,78	273,16	164,15	160,78	90,46	75,63
1164,072	265,73	273,53	164,57	160,89	90,58	75,62
1165,5	265,67	273,84	164,95	161	90,65	75,61
1166,929	265,6	274,09	165,31	161,11	90,67	75,6
1168,357	265,51	274,27	165,64	161,21	90,62	75,58
1169,785	265,41	274,41	165,93	161,3	90,51	75,57
1171,214	265,3	274,49	166,18	161,39	90,33	75,56

This journal is © The Royal Society of Chemistry 20xx

Please do not adjust margins

Please do not adjust margins

1172,642	265,18	274,52	166,39	161,46	90,07	75,54
1174,07	265,04	274,51	166,54	161,52	89,74	75,53
1175,498	264,88	274,46	166,63	161,55	89,34	75,51
1176,927	264,69	274,38	166,65	161,56	88,86	75,49
1178,355	264,49	274,26	166,62	161,55	88,33	75,47
1179,783	264,26	274,11	166,54	161,51	87,74	75,45
1181,212	263,99	273,93	166,41	161,46	87,1	75,43
1182,64	263,71	273,73	166,25	161,38	86,44	75,41
1184,068	263,4	273,48	166,05	161,27	85,75	75,38
1185,497	263,06	273,21	165,82	161,14	85,05	75,35
1186,925	262,7	272,9	165,57	161	84,34	75,3
1188,353	262,32	272,56	165,3	160,83	83,65	75,25
1189,782	261,92	272,2	165,01	160,64	82,96	75,2
1191,21	261,5	271,82	164,71	160,42	82,3	75,14
1192,638	261,07	271,42	164,41	160,16	81,66	75,08
1194,067	260,62	271,03	164,13	159,88	81,06	75,01
1195,495	260,17	270,64	163,85	159,57	80,47	74,94
1196,923	259,73	270,26	163,59	159,24	79,92	74,87
1198,351	259,28	269,89	163,34	158,9	79,41	74,8
1199,78	258,84	269,53	163,08	158,54	78,91	74,72
1201,208	258,39	269,18	162,85	158,17	78,45	74,64
1202,636	257,97	268,86	162,64	157,77	78	74,55
1204,065	257,56	268,57	162,45	157,35	77,56	74,47
1205,493	257,18	268,31	162,29	156,92	77,15	74,39
1206,921	256,82	268,06	162,16	156,47	76,74	74,31
1208,35	256,5	267,83	162,05	156,04	76,34	74,23
1209,778	256,21	267,6	161,95	155,64	75,96	74,15
1211,206	255,96	267,37	161,84	155,27	75,58	74,06
1212,635	255,74	267,15	161,73	154,94	75,21	73,97
1214,063	255,57	266,91	161,61	154,66	74,85	73,88
1215,491	255,43	266,66	161,45	154,43	74,49	73,78
1216,919	255,32	266,4	161,28	154,24	74,15	73,67
1218,348	255,24	266,12	161,09	154,08	73,8	73,55
1219,776	255,18	265,83	160,89	153,95	73,46	73,41
1221,204	255,15	265,53	160,67	153,83	73,11	73,26
1222,633	255,14	265,22	160,44	153,72	72,77	73,09
1224,061	255,14	264,9	160,2	153,61	72,43	72,91
1225,489	255,17	264,56	159,95	153,5	72,11	72,72
1226,918	255,22	264,2	159,7	153,4	71,79	72,52
1228,346	255,28	263,83	159,43	153,29	71,49	72,3
1229,774	255,35	263,43	159,18	153,18	71,2	72,07
1231,203	255,44	263,02	158,92	153,05	70,94	71,83
1232,631	255,52	262,58	158,67	152,92	70,71	71,58
1234,059	255,6	262,12	158,42	152,78	70,5	71,3
1235,487	255,68	261,63	158,18	152,63	70,33	71,01
1236,916	255,77	261,13	157,95	152,47	70,19	70,71
1238,344	255,85	260,61	157,73	152,31	70,1	70,4

This journal is © The Royal Society of Chemistry 20xx

Please do not adjust margins

Please do not adjust margins

1239,772	255,94	260,07	157,53	152,14	70,05	70,08
1241,201	256,02	259,53	157,33	151,95	70,03	69,74
1242,629	256,09	258,98	157,15	151,75	70,06	69,4
1244,057	256,16	258,42	156,98	151,53	70,13	69,06
1245,486	256,22	257,85	156,81	151,28	70,23	68,71
1246,914	256,27	257,29	156,66	151	70,36	68,35
1248,342	256,32	256,73	156,53	150,68	70,52	68
1249,771	256,36	256,19	156,44	150,34	70,71	67,64
1251,199	256,41	255,67	156,37	149,98	70,93	67,29
1252,627	256,47	255,16	156,33	149,61	71,16	66,93
1254,055	256,52	254,66	156,3	149,23	71,41	66,58
1255,484	256,57	254,18	156,29	148,84	71,67	66,23
1256,912	256,62	253,72	156,31	148,43	71,94	65,89
1258,34	256,67	253,28	156,37	148,01	72,22	65,57
1259,769	256,72	252,86	156,46	147,58	72,5	65,25
1261,197	256,76	252,45	156,57	147,15	72,79	64,94
1262,625	256,79	252,05	156,68	146,76	73,1	64,63
1264,054	256,81	251,64	156,8	146,4	73,41	64,33
1265,482	256,83	251,23	156,91	146,07	73,74	64,04
1266,91	256,84	250,83	157,03	145,78	74,08	63,76
1268,339	256,84	250,43	157,14	145,52	74,43	63,48
1269,767	256,83	250,03	157,23	145,28	74,77	63,19
1271,195	256,8	249,63	157,3	145,06	75,11	62,9
1272,624	256,76	249,23	157,38	144,84	75,43	62,61
1274,052	256,7	248,84	157,45	144,61	75,74	62,32
1275,48	256,63	248,45	157,52	144,37	76,03	62,01
1276,908	256,54	248,06	157,6	144,13	76,29	61,7
1278,337	256,43	247,68	157,66	143,87	76,53	61,39
1279,765	256,31	247,29	157,71	143,6	76,74	61,06
1281,193	256,19	246,91	157,76	143,32	76,93	60,73
1282,622	256,06	246,52	157,8	143,04	77,09	60,39
1284,05	255,93	246,13	157,84	142,74	77,22	60,05
1285,478	255,78	245,74	157,86	142,44	77,33	59,69
1286,907	255,63	245,33	157,86	142,13	77,42	59,32
1288,335	255,47	244,92	157,85	141,82	77,48	58,92
1289,763	255,3	244,51	157,81	141,51	77,52	58,51
1291,192	255,12	244,1	157,74	141,21	77,55	58,07
1292,62	254,94	243,69	157,65	140,91	77,55	57,62
1294,048	254,75	243,28	157,53	140,61	77,54	57,16
1295,476	254,56	242,87	157,39	140,3	77,51	56,67
1296,905	254,35	242,47	157,23	140	77,48	56,17
1298,333	254,16	242,09	157,07	139,7	77,43	55,67
1299,761	253,96	241,73	156,9	139,4	77,38	55,15
1301,19	253,76	241,39	156,69	139,09	77,31	54,62
1302,618	253,57	241,08	156,47	138,8	77,23	54,08
1304,046	253,4	240,79	156,26	138,52	77,12	53,53
1305,475	253,27	240,52	156,06	138,26	77,02	53

This journal is © The Royal Society of Chemistry 20xx

Please do not adjust margins

1306,903	253,18	240,28	155,87	138,04	76,9	52,48
1308,331	253,12	240,06	155,69	137,85	76,78	51,97
1309,76	253,1	239,85	155,52	137,69	76,64	51,48
1311,188	253,08	239,66	155,34	137,54	76,49	51
1312,616	253,04	239,48	155,12	137,41	76,32	50,53
1314,044	253,01	239,3	154,88	137,28	76,14	50,08
1315,473	252,99	239,12	154,64	137,17	75,94	49,65
1316,901	252,99	238,94	154,42	137,05	75,73	49,25
1318,329	252,98	238,77	154,19	136,92	75,5	48,87
1319,758	252,97	238,6	153,94	136,78	75,26	48,5
1321,186	252,95	238,43	153,71	136,62	74,99	48,16
1322,614	252,94	238,26	153,49	136,45	74,7	47,86
1324,043	252,92	238,07	153,26	136,26	74,38	47,57
1325,471	252,87	237,87	153,02	136,05	74,03	47,31
1326,899	252,81	237,66	152,77	135,81	73,64	47,07
1328,328	252,72	237,42	152,53	135,56	73,2	46,86
1329,756	252,61	237,16	152,28	135,3	72,73	46,68
1331,184	252,46	236,86	152,02	135,01	72,21	46,52
1332,612	252,29	236,55	151,76	134,71	71,66	46,38
1334,041	252,07	236,22	151,49	134,39	71,07	46,25
1335,469	251,77	235,87	151,18	134,04	70,41	46,13
1336,897	251,41	235,47	150,83	133,66	69,72	45,99
1338,326	251,02	235,04	150,46	133,27	69,02	45,84
1339,754	250,6	234,57	150,1	132,87	68,28	45,69
1341,182	250,15	234,09	149,75	132,46	67,51	45,55
1342,611	249,65	233,57	149,37	132,02	66,71	45,38
1344,039	249,11	233,03	148,98	131,55	65,91	45,19
1345,467	248,55	232,48	148,58	131,07	65,09	44,97
1346,896	247,97	231,93	148,2	130,58	64,23	44,75
1348,324	247,36	231,37	147,8	130,09	63,38	44,52
1349,752	246,72	230,79	147,39	129,58	62,52	44,27
1351,181	246,06	230,21	146,96	129,07	61,67	43,99
1352,609	245,4	229,64	146,53	128,57	60,83	43,7
1354,037	244,76	229,08	146,12	128,09	59,99	43,43
1355,465	244,12	228,52	145,69	127,64	59,15	43,14
1356,894	243,49	227,98	145,23	127,18	58,34	42,82
1358,322	242,87	227,47	144,77	126,75	57,51	42,49
1359,75	242,28	226,98	144,33	126,36	56,67	42,17
1361,179	241,72	226,48	143,89	125,99	55,84	41,85
1362,607	241,2	225,98	143,47	125,66	55,01	41,51
1364,035	240,74	225,45	143,06	125,37	54,19	41,19
1365,464	240,33	224,93	142,67	125,1	53,36	40,87
1366,892	239,94	224,44	142,28	124,81	52,49	40,52
1368,32	239,52	223,96	141,84	124,49	51,62	40,11
1369,749	239,11	223,47	141,37	124,16	50,77	39,66
1371,177	238,79	222,97	140,96	123,86	49,9	39,25
1372,605	238,54	222,46	140,61	123,59	49,05	38,86

This journal is © The Royal Society of Chemistry 20xx

Please do not adjust margins

1374,033	238,33	221,94	140,26	123,3	48,23	38,46
1375,462	238,13	221,44	139,9	122,98	47,44	38,04
1376,89	237,97	220,95	139,56	122,65	46,68	37,63
1378,318	237,83	220,48	139,25	122,33	45,94	37,22
1379,747	237,7	220,01	138,95	122,01	45,25	36,81
1381,175	237,56	219,56	138,62	121,67	44,61	36,38
1382,603	237,43	219,15	138,31	121,32	44	35,96
1384,032	237,33	218,74	138,02	121,01	43,44	35,56
1385,46	237,24	218,35	137,73	120,71	42,91	35,17
1386,888	237,16	218	137,46	120,42	42,43	34,8
1388,317	237,1	217,69	137,2	120,15	42,03	34,44
1389,745	237,04	217,42	136,97	119,92	41,65	34,1
1391,173	236,97	217,19	136,74	119,71	41,27	33,78
1392,601	236,87	216,99	136,48	119,53	40,94	33,45
1394,03	236,83	216,82	136,25	119,4	40,71	33,16
1395,458	236,86	216,71	136,13	119,34	40,54	32,94
1396,886	236,92	216,67	136,06	119,31	40,39	32,77
1398,315	236,96	216,69	135,97	119,32	40,33	32,61
1399,743	237	216,8	135,87	119,35	40,35	32,47
1401,171	237,05	216,99	135,79	119,42	40,43	32,38
1402,6	237,14	217,25	135,77	119,56	40,59	32,37
1404,028	237,27	217,54	135,78	119,77	40,82	32,42
1405,456	237,42	217,85	135,81	120,03	41,14	32,52
1406,885	237,59	218,2	135,85	120,35	41,57	32,67
1408,313	237,79	218,61	135,92	120,72	42,04	32,87
1409,741	238,02	219,09	136,03	121,13	42,56	33,13
1411,169	238,28	219,62	136,17	121,59	43,18	33,44
1412,598	238,57	220,17	136,33	122,07	43,85	33,78
1414,026	238,89	220,74	136,54	122,6	44,58	34,17
1415,454	239,25	221,32	136,8	123,19	45,35	34,6
1416,883	239,64	221,9	137,09	123,81	46,17	35,05
1418,311	240,04	222,48	137,38	124,43	47,08	35,48
1419,739	240,44	223,07	137,67	125,04	48	35,9
1421,168	240,84	223,66	137,98	125,66	48,97	36,34
1422,596	241,28	224,23	138,32	126,3	49,98	36,78
1424,024	241,73	224,76	138,72	126,94	51	37,22
1425,453	242,22	225,21	139,14	127,61	52,1	37,68
1426,881	242,74	225,59	139,57	128,28	53,25	38,14
1428,309	243,18	225,97	139,94	128,87	54,41	38,57
1429,738	243,55	226,36	140,27	129,42	55,62	38,96
1431,166	243,96	226,66	140,63	130,01	56,86	39,35
1432,594	244,4	226,88	141,03	130,61	58,11	39,74
1434,022	244,85	227,07	141,43	131,21	59,4	40,13
1435,451	245,28	227,23	141,76	131,76	60,7	40,47
1436,879	245,61	227,42	142	132,21	62,02	40,72
1438,307	245,91	227,64	142,19	132,59	63,32	40,94
1439,736	246,26	227,83	142,42	132,97	64,61	41,15

This journal is © The Royal Society of Chemistry 20xx

Please do not adjust margins

Please do not adjust margins

1441,164	246,67	227,99	142,68	133,39	65,92	41,39
1442,592	247,13	228,13	142,98	133,82	67,22	41,63
1444,021	247,65	228,29	143,31	134,23	68,5	41,88
1445,449	248,17	228,48	143,62	134,62	69,75	42,13
1446,877	248,7	228,68	143,92	135,01	70,94	42,37
1448,306	249,3	228,9	144,31	135,43	72,1	42,65
1449,734	250	229,15	144,8	135,88	73,29	42,99
1451,162	250,77	229,46	145,34	136,33	74,46	43,34
1452,59	251,56	229,85	145,88	136,74	75,6	43,69
1454,019	252,34	230,3	146,38	137,09	76,71	44,02
1455,447	253,16	230,79	146,89	137,45	77,83	44,37
1456,875	254,09	231,33	147,5	137,85	78,96	44,79
1458,304	255,11	231,89	148,19	138,27	80,06	45,27
1459,732	256,18	232,48	148,92	138,69	81,18	45,78
1461,16	257,27	233,09	149,67	139,13	82,29	46,32
1462,589	258,38	233,72	150,45	139,54	83,33	46,88
1464,017	259,54	234,36	151,3	139,98	84,34	47,5
1465,445	260,73	235	152,24	140,44	85,29	48,15
1466,874	261,96	235,6	153,23	140,92	86,22	48,83
1468,302	263,16	236,22	154,21	141,38	87,15	49,52
1469,73	264,19	236,89	155,07	141,7	88	50,14
1471,158	265,11	237,59	155,85	141,94	88,79	50,7
1472,587	266	238,29	156,67	142,18	89,53	51,26
1474,015	266,86	238,92	157,52	142,43	90,2	51,84
1475,443	267,69	239,48	158,38	142,69	90,82	52,42
1476,872	268,45	239,92	159,25	142,98	91,37	53,01
1478,3	269,1	240,36	160,04	143,2	91,82	53,55
1479,728	269,68	240,9	160,79	143,33	92,22	54,1
1481,157	270,19	241,33	161,58	143,51	92,5	54,63
1482,585	270,56	241,67	162,27	143,66	92,69	55,1
1484,013	270,74	241,95	162,82	143,74	92,79	55,49
1485,442	270,76	242,19	163,31	143,75	92,75	55,81
1486,87	270,72	242,35	163,78	143,79	92,65	56,13
1488,298	270,6	242,42	164,15	143,77	92,48	56,39
1489,726	270,23	242,46	164,33	143,59	92,18	56,49
1491,155	269,7	242,49	164,39	143,34	91,83	56,48
1492,583	269,12	242,49	164,44	143,09	91,36	56,46
1494,011	268,52	242,42	164,54	142,89	90,75	56,45
1495,44	267,86	242,26	164,61	142,66	90,1	56,39
1496,868	267,16	241,99	164,65	142,42	89,36	56,27
1498,296	266,48	241,68	164,74	142,2	88,55	56,17
1499,725	265,77	241,34	164,81	141,96	87,67	56,01
1501,153	264,93	240,98	164,78	141,64	86,68	55,76
1502,581	264	240,58	164,68	141,26	85,66	55,43
1504,01	263,07	240,16	164,56	140,87	84,62	55,04
1505,438	262,11	239,77	164,42	140,46	83,54	54,63
1506,866	261,16	239,36	164,27	140,02	82,45	54,2

This journal is © The Royal Society of Chemistry 20xx

Please do not adjust margins

1508,295	260,17	238,88	164,03	139,54	81,34	53,68
1509,723	259,19	238,34	163,76	139,04	80,23	53,11
1511,151	258,31	237,78	163,57	138,6	79,11	52,6
1512,579	257,51	237,21	163,49	138,21	77,93	52,12
1514,008	256,73	236,63	163,38	137,81	76,78	51,6
1515,436	255,91	236,01	163,21	137,34	75,58	51
1516,864	255,08	235,31	163,04	136,87	74,31	50,38
1518,293	254,3	234,56	162,9	136,44	73,02	49,76
1519,721	253,55	233,73	162,74	135,97	71,66	49,08
1521,149	252,77	232,91	162,53	135,43	70,3	48,3
1522,578	251,93	232,1	162,24	134,79	68,92	47,43
1524,006	251,05	231,29	161,86	134,08	67,49	46,47
1525,434	250,14	230,52	161,42	133,32	66,03	45,47
1526,863	249,25	229,71	160,98	132,54	64,5	44,45
1528,291	248,47	228,74	160,62	131,85	62,91	43,39
1529,719	247,88	227,67	160,41	131,39	61,33	42,43
1531,147	247,28	226,75	160,24	130,89	59,52	41,47
1532,576	246,53	225,77	159,87	130,12	57,69	40,27
1534,004	245,79	224,79	159,46	129,33	55,82	38,99
1535,432	245,06	223,86	159,03	128,5	53,85	37,66
1536,861	244,42	222,9	158,68	127,73	51,83	36,34
1538,289	243,77	221,79	158,31	126,99	49,73	34,91
1539,717	242,99	220,69	157,73	126,07	47,58	33,29
1541,146	242,14	219,68	157,06	125,04	45,36	31,55
1542,574	241,31	218,67	156,4	124,02	43,04	29,79
1544,002	240,52	217,61	155,74	123,05	40,7	28,02
1545,431	239,73	216,5	155,05	122,1	38,33	26,25
1546,859	238,99	215,33	154,39	121,19	35,92	24,45
1548,287	238,36	214,19	153,83	120,32	33,46	22,71
1549,715	237,77	213,09	153,29	119,47	30,96	21
1551,144	237,1	211,96	152,64	118,53	28,48	19,24
1552,572	236,35	210,84	151,89	117,51	26,08	17,43
1554	235,63	209,75	151,17	116,51	23,69	15,69
1555,429	234,94	208,71	150,46	115,53	21,36	14,01
1556,857	234,14	207,72	149,61	114,49	19,11	12,32
1558,285	233,25	206,78	148,66	113,38	16,95	10,64
1559,714	232,36	205,9	147,68	112,29	14,91	9,06
1561,142	231,53	205,07	146,77	111,28	12,98	7,62
1562,57	230,73	204,27	145,91	110,31	11,14	6,33
1563,999	229,96	203,49	145,1	109,39	9,45	5,16
1565,427	229,19	202,76	144,27	108,48	7,89	4,09
1566,855	228,39	202,13	143,45	107,6	6,45	3,16
1568,283	227,62	201,62	142,68	106,76	5,14	2,4
1569,712	226,76	201,18	141,87	105,91	3,95	1,71
1571,14	225,82	200,77	140,96	105,08	2,96	1,11
1572,568	224,88	200,43	140,06	104,31	2,09	0,64
1573,997	223,92	200,19	139,2	103,6	1,38	0,32

This journal is © The Royal Society of Chemistry 20xx

Please do not adjust margins

1575,425	222,95	200,04	138,32	102,93	0,82	0,12
1576,853	221,95	200	137,44	102,29	0,42	0
1578,282	220,87	200,06	136,53	101,7	0,16	0
1579,71	219,77	200,16	135,63	101,21	0,03	0,16
1581,138	218,71	200,29	134,82	100,88	0	0,46
1582,567	217,67	200,5	134,09	100,66	0,09	0,92
1583,995	216,62	200,76	133,41	100,49	0,27	1,46
1585,423	215,53	201,06	132,72	100,38	0,56	2,04
1586,852	214,42	201,48	132,04	100,33	0,95	2,71
1588,28	213,19	202,07	131,36	100,15	1,31	3,36
1589,708	211,95	202,52	130,67	100	1,86	3,97
1591,136	210,88	203,04	130,16	100,1	2,5	4,75
1592,565	209,86	203,62	129,71	100,23	3,19	5,57
1593,993	208,86	204,25	129,31	100,39	3,95	6,39
1595,421	207,87	204,91	128,96	100,57	4,74	7,2
1596,85	206,86	205,61	128,61	100,8	5,58	8,01
1598,278	205,89	206,33	128,31	101,11	6,47	8,88
1599,706	205,01	207	128,12	101,52	7,4	9,81
1601,135	204,23	207,65	128,03	101,95	8,4	10,77
1602,563	203,53	208,32	128,01	102,38	9,43	11,72
1603,991	202,91	209,02	128,05	102,79	10,46	12,66
1605,42	202,33	209,71	128,14	103,22	11,53	13,59
1606,848	201,86	210,36	128,3	103,72	12,68	14,56
1608,276	201,4	211,02	128,51	104,17	13,84	15,53
1609,704	200,95	211,68	128,72	104,56	15	16,45
1611,133	200,61	212,31	128,98	104,99	16,18	17,37
1612,561	200,41	212,91	129,35	105,46	17,38	18,33
1613,989	200,31	213,48	129,78	105,93	18,6	19,28
1615,418	200,26	214,01	130,23	106,37	19,83	20,19
1616,846	200,18	214,52	130,61	106,7	21,07	21
1618,274	200,03	215,05	130,87	106,93	22,31	21,69
1619,703	200	215,55	131,29	107,23	23,52	22,46
1621,131	200,16	215,93	131,88	107,6	24,73	23,29
1622,559	200,47	216,24	132,55	108,01	25,96	24,12
1623,988	200,84	216,42	133,25	108,49	27,19	24,94
1625,416	201,05	216,67	133,87	108,81	28,33	25,64
1626,844	201,33	216,98	134,51	109,04	29,47	26,33
1628,272	201,69	217,24	135,2	109,26	30,6	26,99
1629,701	202,11	217,45	135,91	109,47	31,72	27,61
1631,129	202,57	217,62	136,63	109,66	32,81	28,2
1632,557	203,02	217,75	137,3	109,81	33,89	28,71
1633,986	203,37	217,89	137,83	109,87	34,96	29,12
1635,414	203,63	218,01	138,26	109,85	35,98	29,46
1636,842	203,96	218,09	138,74	109,84	36,98	29,76
1638,271	204,4	218,12	139,3	109,85	37,98	30,07
1639,699	204,9	218,1	139,88	109,88	38,98	30,37
1641,127	205,41	218,07	140,43	109,89	39,97	30,63

This journal is © The Royal Society of Chemistry 20xx

Please do not adjust margins

1642,556	205,91	218,03	140,94	109,9	40,92	30,87
1643,984	206,41	217,98	141,46	109,89	41,82	31,07
1645,412	206,94	217,92	141,98	109,88	42,69	31,21
1646,84	207,48	217,85	142,48	109,85	43,54	31,34
1648,269	208,07	217,78	142,99	109,81	44,39	31,45
1649,697	208,67	217,71	143,47	109,79	45,22	31,56
1651,125	209,2	217,64	143,9	109,76	46	31,66
1652,554	209,67	217,58	144,26	109,7	46,75	31,71
1653,982	210,09	217,54	144,54	109,67	47,53	31,79
1655,41	210,59	217,48	144,88	109,75	48,3	31,95
1656,839	211,08	217,42	145,26	109,82	48,97	32,12
1658,267	211,59	217,4	145,64	109,94	49,64	32,35
1659,695	212,11	217,42	146,02	110,12	50,31	32,63
1661,124	212,59	217,5	146,37	110,35	50,94	32,97
1662,552	212,98	217,65	146,66	110,57	51,51	33,33
1663,98	213,28	217,89	146,9	110,8	52,02	33,71
1665,409	213,47	218,22	147,04	110,99	52,5	34,1
1666,837	213,62	218,6	147,12	111,18	52,99	34,51
1668,265	213,81	219,05	147,3	111,45	53,4	35,04
1669,693	213,92	219,59	147,47	111,79	53,77	35,65
1671,122	213,83	220,21	147,52	112,05	54,07	36,23
1672,55	213,67	220,85	147,64	112,35	54,26	36,89
1673,978	213,45	221,56	147,82	112,75	54,36	37,67
1675,407	213,25	222,31	148,15	113,29	54,38	38,59
1676,835	213,19	223,11	148,75	114,02	54,32	39,75
1678,263	212,9	223,99	149,18	114,62	54,18	40,86
1679,692	212,31	224,99	149,38	115,12	53,97	41,89
1681,12	211,55	226,16	149,48	115,55	53,67	42,93
1682,548	210,63	227,51	149,52	115,83	53,25	43,97
1683,977	209,9	228,83	149,66	116,28	52,87	45,11
1685,405	209,18	230,12	149,78	116,89	52,47	46,3
1686,833	208,42	231,46	149,85	117,49	52	47,5
1688,261	207,65	232,87	149,85	118,1	51,54	48,69
1689,69	206,87	234,31	149,79	118,79	51,03	49,91
1691,118	206,17	235,73	149,79	119,55	50,47	51,17
1692,546	205,74	237,17	149,96	120,46	49,96	52,5
1693,975	205,56	238,58	150,22	121,52	49,5	53,87
1695,403	205,47	240,01	150,44	122,6	49,09	55,23
1696,831	205,49	241,49	150,6	123,67	48,72	56,54
1698,26	205,6	242,99	150,72	124,73	48,39	57,79
1699,688	205,94	244,5	150,88	125,88	48,16	59,03
1701,116	206,53	246,01	151,1	127,13	48,03	60,25
1702,545	207,34	247,5	151,38	128,47	47,99	61,48
1703,973	208,4	248,99	151,75	129,9	48,06	62,72
1705,401	209,71	250,46	152,19	131,44	48,24	63,96
1706,829	211,22	251,79	152,66	133,06	48,5	65,21
1708,258	212,93	253,09	153,22	134,77	48,9	66,45

 This journal is © The Royal Society of Chemistry 20xx

Please do not adjust margins

1709,686	214,86	254,4	153,88	136,57	49,42	67,69
1711,114	216,99	255,7	154,61	138,48	50,05	68,94
1712,543	219,21	257,03	155,33	140,37	50,79	70,12
1713,971	221,47	258,43	156	142,16	51,62	71,16
1715,399	223,92	259,89	156,78	144,04	52,61	72,25
1716,828	226,49	261,3	157,61	145,99	53,7	73,31
1718,256	229,08	262,68	158,45	147,95	54,87	74,33
1719,684	231,72	264,09	159,31	149,93	56,17	75,31
1721,113	234,4	265,52	160,23	151,9	57,55	76,27
1722,541	237,07	266,97	161,19	153,85	59	77,22
1723,969	239,71	268,41	162,21	155,77	60,48	78,17
1725,397	242,33	269,79	163,33	157,69	61,95	79,12
1726,826	244,86	271,13	164,49	159,62	63,47	80,06
1728,254	247,32	272,45	165,71	161,49	65,01	81
1729,682	249,68	273,73	166,98	163,29	66,51	81,93
1731,111	251,78	275,05	168,1	164,96	68,05	82,75
1732,539	253,66	276,38	169,1	166,49	69,58	83,47
1733,967	255,33	277,71	170,01	167,9	71,07	84,14
1735,396	256,74	278,99	170,79	169,2	72,51	84,75
1736,824	258,08	280,19	171,57	170,47	73,92	85,32
1738,252	259,39	281,35	172,41	171,73	75,33	85,91
1739,681	260,62	282,42	173,26	172,99	76,7	86,53
1741,109	261,73	283,41	174,06	174,23	78,03	87,1
1742,537	262,72	284,38	174,81	175,4	79,32	87,63
1743,966	263,59	285,32	175,5	176,45	80,55	88,13
1745,394	264,37	286,18	176,15	177,48	81,71	88,6
1746,822	265,11	286,95	176,83	178,5	82,79	89,08
1748,25	265,84	287,68	177,53	179,42	83,83	89,54
1749,679	266,55	288,39	178,16	180,26	84,86	89,93
1751,107	267,14	289,06	178,69	181,01	85,83	90,27
1752,535	267,67	289,69	179,15	181,66	86,74	90,57
1753,964	268,21	290,32	179,58	182,24	87,62	90,85
1755,392	268,77	290,89	180,03	182,8	88,44	91,09
1756,82	269,41	291,41	180,53	183,4	89,22	91,37
1758,249	270,09	291,86	181,06	183,99	89,94	91,69
1759,677	270,76	292,24	181,57	184,52	90,59	91,98
1761,105	271,43	292,58	182,08	185,01	91,19	92,24
1762,534	272,12	292,9	182,57	185,45	91,73	92,49
1763,962	272,79	293,17	183,05	185,82	92,21	92,73
1765,39	273,5	293,51	183,55	186,18	92,7	92,94
1766,818	274,21	293,85	184,04	186,51	93,14	93,14
1768,247	274,9	294,16	184,49	186,8	93,52	93,32
1769,675	275,57	294,48	184,91	187,05	93,88	93,47
1771,103	276,27	294,77	185,36	187,31	94,21	93,62
1772,532	277,03	295,04	185,83	187,61	94,52	93,79
1773,96	277,74	295,27	186,25	187,91	94,8	93,92
1775,388	278,44	295,5	186,66	188,16	95,05	94,01

This journal is © The Royal Society of Chemistry 20xx

Please do not adjust margins

Please do not adjust margins

1776,817	279,18	295,76	187,1	188,4	95,28	94,14
1778,245	279,9	296,01	187,53	188,6	95,47	94,26
1779,673	280,6	296,21	187,93	188,76	95,64	94,36
1781,102	281,29	296,4	188,32	188,91	95,8	94,45
1782,53	281,98	296,59	188,73	189,04	95,94	94,54
1783,958	282,68	296,78	189,14	189,18	96,09	94,64
1785,386	283,37	296,95	189,53	189,3	96,22	94,75
1786,815	283,98	297,1	189,88	189,37	96,32	94,8
1788,243	284,62	297,26	190,25	189,46	96,43	94,86
1789,671	285,27	297,41	190,62	189,58	96,53	94,95
1791,1	285,86	297,55	190,95	189,67	96,61	95,01
1792,528	286,43	297,69	191,27	189,75	96,7	95,05
1793,956	286,96	297,84	191,58	189,81	96,79	95,07
1795,385	287,48	297,99	191,89	189,89	96,89	95,1
1796,813	288,06	298,13	192,23	190,01	96,99	95,2
1798,241	288,56	298,24	192,53	190,11	97,07	95,26
1799,67	288,98	298,35	192,8	190,16	97,13	95,27
1801,098	289,44	298,45	193,08	190,26	97,19	95,34
1802,526	289,91	298,5	193,41	190,38	97,22	95,45
1803,954	290,34	298,55	193,73	190,5	97,24	95,54
1805,383	290,7	298,6	194,01	190,58	97,25	95,6
1806,811	290,98	298,67	194,21	190,61	97,25	95,62
1808,24	291,23	298,76	194,38	190,63	97,27	95,62
1809,668	291,43	298,86	194,52	190,63	97,27	95,61
1811,096	291,57	298,95	194,61	190,6	97,27	95,57
1812,524	291,71	299	194,72	190,59	97,27	95,54
1813,953	291,85	299,02	194,85	190,61	97,26	95,55
1815,381	292,04	299,03	195,01	190,65	97,25	95,59
1816,809	292,25	299,06	195,17	190,7	97,26	95,66
1818,238	292,38	299,08	195,29	190,7	97,25	95,68
1819,666	292,5	299,1	195,4	190,7	97,25	95,72
1821,094	292,64	299,1	195,54	190,73	97,24	95,79
1822,523	292,75	299,07	195,69	190,76	97,21	95,87
1823,951	292,84	299,05	195,82	190,79	97,18	95,92
1825,379	292,89	299,02	195,9	190,78	97,16	95,94
1826,807	292,9	299	195,94	190,75	97,15	95,94
1828,236	292,91	299	195,97	190,73	97,15	95,93
1829,664	292,89	299	195,97	190,69	97,15	95,9
1831,092	292,86	298,99	195,96	190,63	97,15	95,87
1832,521	292,86	298,97	195,98	190,59	97,15	95,87
1833,949	292,89	298,94	196,02	190,58	97,15	95,9
1835,377	292,94	298,91	196,07	190,56	97,16	95,93
1836,806	292,97	298,88	196,1	190,53	97,18	95,95
1838,234	293,01	298,85	196,14	190,5	97,2	95,96
1839,662	293,08	298,84	196,19	190,48	97,23	96
1841,091	293,15	298,82	196,22	190,47	97,27	96,05
1842,519	293,19	298,79	196,24	190,45	97,3	96,06

This journal is © The Royal Society of Chemistry 20xx

Please do not adjust margins

1843,947	293,24	298,77	196,25	190,41	97,32	96,06
1845,375	293,32	298,74	196,29	190,39	97,34	96,08
1846,804	293,37	298,73	196,3	190,38	97,37	96,09
1848,232	293,4	298,73	196,29	190,33	97,38	96,07
1849,66	293,44	298,74	196,3	190,29	97,39	96,05
1851,089	293,48	298,76	196,3	190,25	97,42	96,03
1852,517	293,53	298,77	196,29	190,23	97,43	96,03
1853,945	293,62	298,75	196,32	190,25	97,44	96,06
1855,374	293,67	298,73	196,33	190,25	97,45	96,06
1856,802	293,71	298,7	196,32	190,25	97,45	96,05
1858,23	293,8	298,67	196,35	190,29	97,46	96,08
1859,659	293,85	298,64	196,38	190,3	97,46	96,1
1861,087	293,86	298,64	196,38	190,28	97,45	96,06
1862,515	293,89	298,66	196,37	190,25	97,47	96,04
1863,943	293,88	298,66	196,34	190,22	97,47	96
1865,372	293,91	298,66	196,33	190,23	97,47	95,98
1866,8	293,97	298,64	196,36	190,28	97,48	95,99
1868,228	294,01	298,59	196,37	190,32	97,48	95,99
1869,657	294,06	298,55	196,39	190,36	97,48	96
1871,085	294,13	298,53	196,42	190,42	97,48	96,02
1872,513	294,23	298,51	196,46	190,48	97,47	96,07
1873,942	294,31	298,49	196,49	190,52	97,46	96,1
1875,37	294,33	298,46	196,52	190,54	97,44	96,1
1876,798	294,39	298,42	196,56	190,59	97,43	96,13
1878,227	294,46	298,38	196,6	190,64	97,42	96,16
1879,655	294,47	298,35	196,61	190,68	97,41	96,16
1881,083	294,49	298,34	196,59	190,71	97,4	96,15
1882,511	294,52	298,33	196,58	190,74	97,41	96,14
1883,94	294,55	298,31	196,57	190,78	97,4	96,14
1885,368	294,6	298,29	196,58	190,86	97,4	96,16
1886,797	294,65	298,27	196,59	190,91	97,4	96,17
1888,225	294,68	298,26	196,58	190,96	97,4	96,17
1889,653	294,75	298,24	196,59	191,03	97,41	96,2
1891,081	294,82	298,21	196,61	191,1	97,42	96,24
1892,51	294,87	298,18	196,63	191,16	97,42	96,25
1893,938	294,93	298,16	196,65	191,24	97,44	96,28
1895,366	295	298,14	196,65	191,31	97,45	96,31
1896,795	295,05	298,13	196,64	191,39	97,45	96,33
1898,223	295,07	298,1	196,61	191,45	97,44	96,35
1899,651	295,09	298,07	196,58	191,5	97,42	96,34
1901,08	295,12	298,05	196,57	191,56	97,41	96,35
1902,508	295,16	298,05	196,57	191,64	97,41	96,37
1903,936	295,18	298,07	196,54	191,69	97,41	96,37
1905,365	295,17	298,12	196,46	191,71	97,42	96,35
1906,793	295,14	298,16	196,37	191,72	97,42	96,31
1908,221	295,14	298,18	196,3	191,76	97,43	96,3
1909,649	295,2	298,19	196,29	191,84	97,44	96,33

This journal is © The Royal Society of Chemistry 20xx

Please do not adjust margins

1911,078	295,27	298,18	196,32	191,92	97,44	96,37
1912,506	295,34	298,18	196,34	191,99	97,45	96,38
1913,934	295,43	298,19	196,36	192,06	97,46	96,41
1915,363	295,5	298,21	196,37	192,13	97,47	96,44
1916,791	295,56	298,24	196,37	192,2	97,49	96,46
1918,219	295,63	298,25	196,38	192,29	97,49	96,49
1919,648	295,68	298,25	196,39	192,37	97,49	96,52
1921,076	295,71	298,24	196,39	192,44	97,48	96,54
1922,504	295,76	298,25	196,4	192,52	97,48	96,55
1923,932	295,78	298,27	196,38	192,57	97,48	96,56
1925,361	295,77	298,29	196,34	192,59	97,48	96,54
1926,789	295,78	298,31	196,32	192,64	97,47	96,53
1928,217	295,82	298,34	196,31	192,69	97,48	96,53
1929,646	295,85	298,36	196,3	192,74	97,48	96,53
1931,074	295,89	298,37	196,31	192,78	97,48	96,54
1932,502	295,93	298,39	196,33	192,83	97,49	96,56
1933,931	295,98	298,4	196,35	192,88	97,49	96,58
1935,359	296,02	298,41	196,38	192,94	97,49	96,6
1936,787	296,05	298,42	196,4	192,98	97,5	96,61
1938,216	296,1	298,45	196,42	193,02	97,51	96,62
1939,644	296,14	298,47	196,44	193,07	97,53	96,64
1941,072	296,16	298,47	196,46	193,1	97,53	96,65
1942,5	296,19	298,48	196,48	193,14	97,54	96,65
1943,929	296,22	298,49	196,51	193,19	97,56	96,67
1945,357	296,27	298,5	196,54	193,24	97,58	96,69
1946,785	296,33	298,51	196,58	193,29	97,6	96,72
1948,214	296,36	298,52	196,6	193,33	97,62	96,73
1949,642	296,39	298,53	196,63	193,38	97,63	96,73
1951,07	296,44	298,54	196,66	193,44	97,65	96,75
1952,499	296,48	298,54	196,67	193,48	97,65	96,77
1953,927	296,48	298,54	196,67	193,5	97,66	96,76
1955,355	296,49	298,55	196,67	193,53	97,67	96,76
1956,784	296,51	298,56	196,67	193,54	97,69	96,76
1958,212	296,54	298,57	196,69	193,57	97,7	96,76
1959,64	296,58	298,59	196,71	193,61	97,73	96,77
1961,068	296,59	298,61	196,7	193,63	97,75	96,77
1962,497	296,62	298,63	196,71	193,66	97,77	96,77
1963,925	296,68	298,63	196,74	193,72	97,8	96,79
1965,354	296,75	298,63	196,79	193,79	97,82	96,83
1966,782	296,81	298,63	196,83	193,85	97,84	96,86
1968,21	296,84	298,63	196,85	193,9	97,86	96,86
1969,638	296,88	298,63	196,88	193,96	97,88	96,87
1971,067	296,94	298,63	196,9	194,02	97,9	96,9
1972,495	296,95	298,62	196,9	194,06	97,91	96,9
1973,923	296,95	298,62	196,9	194,1	97,92	96,88
1975,352	296,97	298,61	196,9	194,14	97,94	96,88
1976,78	297,01	298,61	196,92	194,19	97,96	96,89

This journal is © The Royal Society of Chemistry 20xx

Please do not adjust margins

Please do not adjust margins

1978,208	297,03	298,61	196,93	194,24	97,98	96,89
1979,637	297,05	298,61	196,92	194,26	98	96,88
1981,065	297,06	298,62	196,91	194,29	98,02	96,87
1982,493	297,09	298,63	196,92	194,34	98,04	96,87
1983,922	297,13	298,63	196,94	194,4	98,06	96,88
1985,35	297,17	298,63	196,95	194,44	98,08	96,88
1986,778	297,19	298,63	196,97	194,47	98,1	96,88
1988,206	297,23	298,63	197	194,52	98,12	96,89
1989,635	297,28	298,64	197,03	194,58	98,15	96,91
1991,063	297,3	298,63	197,04	194,62	98,18	96,91
1992,491	297,32	298,6	197,04	194,66	98,2	96,91
1993,92	297,34	298,59	197,05	194,72	98,23	96,91
1995,348	297,37	298,58	197,06	194,77	98,25	96,92
1996,776	297,39	298,56	197,07	194,82	98,28	96,92
1998,205	297,4	298,55	197,06	194,86	98,3	96,91
1999,633	297,41	298,54	197,06	194,9	98,32	96,89
2001,061	297,44	298,54	197,06	194,94	98,35	96,89
2002,49	297,46	298,54	197,06	194,99	98,37	96,89
2003,918	297,47	298,53	197,04	195,02	98,39	96,87
2005,346	297,48	298,53	197,03	195,06	98,41	96,86
2006,774	297,49	298,52	197,01	195,1	98,43	96,85
2008,203	297,49	298,5	197	195,12	98,45	96,84
2009,631	297,51	298,49	196,99	195,15	98,47	96,83
2011,059	297,52	298,48	196,98	195,17	98,49	96,82
2012,488	297,54	298,48	196,98	195,19	98,51	96,81
2013,916	297,58	298,47	196,98	195,23	98,54	96,81
2015,344	297,62	298,46	196,99	195,28	98,56	96,82
2016,773	297,65	298,45	196,99	195,32	98,58	96,82
2018,201	297,67	298,44	196,98	195,36	98,6	96,81
2019,629	297,7	298,43	196,98	195,41	98,62	96,81
2021,057	297,73	298,41	196,98	195,46	98,64	96,82
2022,486	297,75	298,4	196,98	195,49	98,65	96,81
2023,914	297,76	298,38	196,97	195,52	98,66	96,79
2025,342	297,79	298,38	196,97	195,56	98,68	96,78
2026,771	297,81	298,37	196,95	195,59	98,7	96,76
2028,199	297,83	298,37	196,94	195,62	98,72	96,75
2029,627	297,84	298,36	196,91	195,65	98,74	96,74
2031,056	297,86	298,35	196,9	195,68	98,75	96,73
2032,484	297,89	298,35	196,9	195,72	98,77	96,74
2033,912	297,92	298,34	196,91	195,77	98,78	96,75
2035,341	297,95	298,34	196,93	195,8	98,8	96,75
2036,769	297,99	298,35	196,94	195,84	98,82	96,75
2038,197	298,01	298,34	196,95	195,88	98,84	96,74
2039,625	298,04	298,33	196,95	195,93	98,86	96,74
2041,054	298,08	298,32	196,97	195,99	98,88	96,75
2042,482	298,11	298,29	196,99	196,04	98,89	96,75
2043,911	298,14	298,28	197	196,09	98,91	96,75

This journal is © The Royal Society of Chemistry 20xx

Please do not adjust margins

Please do not adjust margins

2045,339	298,17	298,26	197	196,15	98,93	96,75
2046,767	298,17	298,24	196,98	196,19	98,94	96,74
2048,195	298,17	298,21	196,95	196,23	98,96	96,72
2049,624	298,18	298,19	196,95	196,29	98,98	96,71
2051,052	298,2	298,18	196,95	196,34	99	96,7
2052,48	298,22	298,17	196,94	196,39	99,01	96,69
2053,909	298,22	298,16	196,91	196,42	99,02	96,67
2055,337	298,22	298,15	196,9	196,47	99,04	96,65
2056,765	298,25	298,14	196,89	196,51	99,05	96,65
2058,194	298,28	298,13	196,89	196,57	99,07	96,65
2059,622	298,31	298,11	196,9	196,62	99,09	96,65
2061,05	298,33	298,1	196,9	196,67	99,11	96,65
2062,479	298,36	298,09	196,9	196,72	99,13	96,64
2063,907	298,38	298,08	196,9	196,77	99,15	96,64
2065,335	298,39	298,07	196,89	196,81	99,16	96,63
2066,763	298,42	298,05	196,89	196,85	99,18	96,63
2068,192	298,44	298,04	196,9	196,89	99,19	96,63
2069,62	298,46	298,02	196,89	196,93	99,21	96,62
2071,048	298,48	298	196,88	196,96	99,22	96,61
2072,477	298,49	297,99	196,87	196,99	99,23	96,59
2073,905	298,5	297,98	196,86	197,02	99,24	96,58
2075,333	298,52	297,97	196,86	197,05	99,26	96,57
2076,761	298,52	297,95	196,85	197,07	99,27	96,56
2078,19	298,53	297,95	196,84	197,08	99,28	96,54
2079,618	298,53	297,94	196,83	197,09	99,3	96,52
2081,046	298,53	297,93	196,82	197,1	99,3	96,51
2082,475	298,53	297,92	196,82	197,11	99,32	96,5
2083,903	298,53	297,91	196,81	197,12	99,33	96,49
2085,332	298,53	297,9	196,81	197,12	99,33	96,48
2086,76	298,54	297,89	196,81	197,13	99,35	96,47
2088,188	298,54	297,88	196,81	197,14	99,35	96,47
2089,616	298,55	297,86	196,82	197,14	99,36	96,47
2091,045	298,56	297,85	196,83	197,15	99,37	96,47
2092,473	298,57	297,83	196,83	197,15	99,37	96,46
2093,901	298,57	297,82	196,83	197,16	99,38	96,46
2095,33	298,56	297,79	196,81	197,15	99,38	96,45
2096,758	298,54	297,76	196,81	197,15	99,38	96,44
2098,186	298,54	297,75	196,8	197,14	99,38	96,44
2099,615	298,53	297,73	196,79	197,13	99,39	96,43
2101,043	298,51	297,72	196,77	197,12	99,39	96,41
2102,471	298,49	297,71	196,75	197,11	99,39	96,4
2103,899	298,46	297,69	196,72	197,09	99,39	96,38
2105,328	298,45	297,68	196,7	197,07	99,4	96,36
2106,756	298,44	297,68	196,69	197,06	99,4	96,36
2108,184	298,44	297,67	196,69	197,05	99,4	96,36
2109,613	298,44	297,67	196,69	197,05	99,4	96,37
2111,041	298,43	297,66	196,68	197,03	99,4	96,37

This journal is © The Royal Society of Chemistry 20xx

Please do not adjust margins

Please do not adjust margins

2112,469	298,43	297,65	196,68	197,02	99,4	96,37
2113,898	298,43	297,65	196,68	197,03	99,41	96,37
2115,326	298,43	297,64	196,67	197,03	99,41	96,37
2116,754	298,43	297,64	196,66	197,03	99,41	96,36
2118,183	298,43	297,64	196,66	197,04	99,41	96,36
2119,611	298,42	297,62	196,64	197,03	99,41	96,36
2121,039	298,41	297,62	196,63	197,03	99,41	96,36
2122,468	298,4	297,62	196,62	197,03	99,4	96,36
2123,896	298,4	297,61	196,62	197,03	99,4	96,35
2125,324	298,4	297,61	196,6	197,02	99,4	96,34
2126,752	298,39	297,62	196,59	197,01	99,4	96,33
2128,181	298,38	297,62	196,58	197	99,41	96,33
2129,609	298,38	297,63	196,57	197	99,41	96,32
2131,037	298,38	297,63	196,57	196,99	99,42	96,32
2132,466	298,38	297,64	196,57	196,99	99,42	96,32
2133,894	298,38	297,64	196,57	196,99	99,43	96,31
2135,322	298,38	297,65	196,57	196,98	99,43	96,3
2136,75	298,38	297,64	196,58	196,99	99,44	96,3
2138,179	298,38	297,64	196,58	196,98	99,44	96,29
2139,607	298,38	297,64	196,58	196,99	99,45	96,29
2141,035	298,39	297,65	196,59	196,99	99,45	96,29
2142,464	298,4	297,64	196,6	197	99,45	96,3
2143,892	298,41	297,64	196,61	197,01	99,46	96,31
2145,32	298,42	297,64	196,62	197,02	99,46	96,31
2146,749	298,43	297,63	196,64	197,03	99,47	96,31
2148,177	298,43	297,63	196,65	197,04	99,48	96,32
2149,605	298,43	297,63	196,65	197,04	99,48	96,32
2151,034	298,43	297,63	196,67	197,04	99,49	96,32
2152,462	298,43	297,63	196,67	197,04	99,49	96,32
2153,89	298,42	297,62	196,66	197,03	99,5	96,32
2155,319	298,42	297,62	196,66	197,02	99,51	96,31
2156,747	298,41	297,62	196,66	197,01	99,51	96,3
2158,175	298,4	297,62	196,65	197	99,51	96,29
2159,604	298,4	297,63	196,65	196,99	99,52	96,29
2161,032	298,4	297,64	196,65	196,98	99,52	96,3
2162,46	298,4	297,65	196,64	196,97	99,53	96,3
2163,888	298,41	297,65	196,65	196,98	99,54	96,31
2165,317	298,42	297,65	196,66	196,99	99,54	96,32
2166,745	298,43	297,65	196,66	196,99	99,54	96,32
2168,173	298,44	297,65	196,66	196,99	99,54	96,32
2169,602	298,44	297,66	196,66	196,99	99,54	96,32
2171,03	298,45	297,67	196,66	197	99,55	96,33
2172,458	298,46	297,67	196,66	197,01	99,55	96,33
2173,887	298,47	297,67	196,66	197	99,55	96,34
2175,315	298,48	297,68	196,66	197,01	99,56	96,34
2176,743	298,47	297,69	196,65	197	99,56	96,34
2178,172	298,46	297,7	196,64	196,99	99,56	96,34

This journal is © The Royal Society of Chemistry 20xx

Please do not adjust margins

Please do not adjust margins

2179,6	298,45	297,72	196,63	196,99	99,57	96,34
2181,028	298,45	297,73	196,63	196,98	99,57	96,33
2182,457	298,45	297,74	196,63	196,98	99,57	96,33
2183,885	298,45	297,76	196,63	196,98	99,57	96,33
2185,313	298,47	297,77	196,64	196,98	99,57	96,34
2186,741	298,48	297,79	196,66	196,99	99,58	96,35
2188,17	298,49	297,8	196,67	197	99,57	96,35
2189,598	298,49	297,81	196,67	197	99,57	96,35
2191,026	298,5	297,83	196,68	197	99,57	96,35
2192,455	298,5	297,84	196,68	196,99	99,56	96,35
2193,883	298,5	297,86	196,68	196,99	99,56	96,36
2195,311	298,5	297,88	196,69	196,99	99,56	96,36
2196,74	298,51	297,9	196,69	196,99	99,55	96,37
2198,168	298,51	297,92	196,7	197,01	99,55	96,38
2199,596	298,52	297,93	196,7	197,02	99,55	96,38
2201,024	298,53	297,94	196,71	197,01	99,54	96,38
2202,453	298,53	297,96	196,71	197,01	99,54	96,39
2203,881	298,53	297,97	196,71	197	99,53	96,39
2205,309	298,53	297,99	196,72	197	99,52	96,4
2206,738	298,53	298	196,72	197	99,51	96,4
2208,166	298,52	298,01	196,72	196,99	99,5	96,4
2209,594	298,51	298,02	196,7	196,98	99,5	96,39
2211,023	298,5	298,04	196,69	196,97	99,49	96,39
2212,451	298,5	298,05	196,68	196,96	99,48	96,39
2213,879	298,5	298,06	196,68	196,96	99,47	96,39
2215,308	298,51	298,07	196,69	196,96	99,46	96,4
2216,736	298,53	298,07	196,69	196,97	99,45	96,42
2218,164	298,53	298,08	196,69	196,97	99,45	96,42
2219,593	298,52	298,07	196,68	196,97	99,43	96,43
2221,021	298,52	298,07	196,68	196,97	99,42	96,43
2222,449	298,52	298,07	196,68	196,97	99,41	96,43
2223,877	298,51	298,07	196,68	196,97	99,4	96,43
2225,306	298,5	298,08	196,68	196,97	99,4	96,44
2226,734	298,49	298,08	196,68	196,96	99,38	96,45
2228,162	298,48	298,08	196,68	196,96	99,37	96,45
2229,591	298,47	298,08	196,67	196,97	99,36	96,45
2231,019	298,46	298,08	196,67	196,97	99,35	96,45
2232,447	298,45	298,08	196,66	196,97	99,34	96,44
2233,875	298,44	298,08	196,66	196,98	99,34	96,44
2235,304	298,43	298,07	196,65	196,98	99,33	96,44
2236,732	298,44	298,06	196,66	196,98	99,32	96,44
2238,16	298,44	298,04	196,66	196,98	99,31	96,44
2239,589	298,44	298,03	196,66	196,98	99,31	96,44
2241,017	298,45	298,01	196,66	196,99	99,31	96,46
2242,446	298,45	297,99	196,66	196,99	99,29	96,47
2243,874	298,45	297,98	196,66	196,99	99,28	96,47
2245,302	298,45	297,96	196,66	196,99	99,28	96,47

This journal is © The Royal Society of Chemistry 20xx

Please do not adjust margins

Please do not adjust margins

2246,73	298,46	297,95	196,67	196,99	99,27	96,47
2248,159	298,48	297,94	196,68	197	99,27	96,48
2249,587	298,5	297,92	196,69	197	99,26	96,49
2251,015	298,52	297,9	196,7	197,01	99,25	96,51
2252,444	298,56	297,88	196,74	197,03	99,25	96,53
2253,872	298,6	297,85	196,77	197,06	99,24	96,56
2255,3	298,65	297,83	196,81	197,08	99,24	96,58
2256,729	298,7	297,82	196,86	197,1	99,23	96,61
2258,157	298,76	297,8	196,9	197,11	99,22	96,63
2259,585	298,82	297,78	196,96	197,14	99,22	96,67
2261,013	298,89	297,76	197,02	197,16	99,22	96,7
2262,442	298,96	297,75	197,08	197,18	99,21	96,73
2263,87	299,03	297,74	197,14	197,21	99,2	96,76
2265,298	299,1	297,72	197,19	197,23	99,19	96,79
2266,727	299,17	297,71	197,25	197,25	99,18	96,83
2268,155	299,26	297,69	197,33	197,28	99,18	96,88
2269,583	299,34	297,67	197,4	197,31	99,17	96,92
2271,012	299,41	297,66	197,48	197,34	99,16	96,97
2272,44	299,49	297,64	197,55	197,37	99,14	97,01
2273,868	299,56	297,61	197,62	197,39	99,12	97,03
2275,297	299,61	297,59	197,67	197,41	99,11	97,06
2276,725	299,66	297,57	197,72	197,42	99,09	97,08
2278,153	299,71	297,55	197,78	197,43	99,07	97,11
2279,582	299,77	297,53	197,83	197,46	99,06	97,14
2281,01	299,81	297,5	197,88	197,48	99,04	97,16
2282,438	299,86	297,47	197,92	197,49	99,02	97,19
2283,866	299,9	297,45	197,96	197,51	99,01	97,21
2285,295	299,93	297,42	197,98	197,52	98,99	97,22
2286,723	299,96	297,38	198	197,52	98,97	97,23
2288,151	299,98	297,33	198,01	197,52	98,96	97,24
2289,58	299,99	297,28	198,01	197,52	98,94	97,24
2291,008	300	297,24	198,01	197,52	98,92	97,25
2292,436	299,99	297,21	197,99	197,51	98,9	97,25
2293,865	299,98	297,17	197,98	197,5	98,87	97,25
2295,293	299,96	297,15	197,95	197,49	98,86	97,24
2296,721	299,93	297,11	197,92	197,48	98,83	97,23
2298,149	299,9	297,07	197,89	197,48	98,8	97,22
2299,578	299,87	297,03	197,86	197,48	98,77	97,22
2301,006	299,84	296,97	197,82	197,48	98,74	97,2
2302,434	299,79	296,93	197,78	197,48	98,71	97,18
2303,863	299,74	296,9	197,72	197,48	98,68	97,16
2305,291	299,68	296,88	197,67	197,48	98,66	97,13
2306,719	299,62	296,88	197,61	197,49	98,64	97,1
2308,148	299,54	296,88	197,55	197,49	98,61	97,06
2309,576	299,48	296,88	197,51	197,49	98,58	97,03
2311,004	299,45	296,85	197,49	197,52	98,55	97,02
2312,433	299,43	296,78	197,49	197,55	98,51	97,03

This journal is © The Royal Society of Chemistry 20xx

Please do not adjust margins

Please do not adjust margins

2313,861	299,43	296,68	197,5	197,6	98,49	97,06
2315,289	299,43	296,61	197,52	197,64	98,46	97,08
2316,718	299,42	296,56	197,54	197,68	98,43	97,1
2318,146	299,42	296,53	197,54	197,72	98,39	97,12
2319,574	299,39	296,48	197,54	197,73	98,35	97,12
2321,002	299,35	296,43	197,54	197,73	98,3	97,11
2322,431	299,32	296,37	197,55	197,73	98,25	97,1
2323,859	299,29	296,31	197,56	197,73	98,2	97,09
2325,287	299,26	296,24	197,54	197,72	98,16	97,07
2326,716	299,21	296,17	197,51	197,7	98,12	97,03
2328,144	299,16	296,08	197,47	197,69	98,07	96,99
2329,572	299,12	295,96	197,45	197,71	98,03	96,98
2331,001	299,1	295,8	197,45	197,74	98	97
2332,429	299,08	295,61	197,43	197,78	97,96	97,01
2333,857	299,01	295,43	197,37	197,8	97,93	97
2335,286	298,89	295,23	197,29	197,81	97,89	96,95
2336,714	298,75	295,04	197,18	197,83	97,86	96,88
2338,142	298,6	294,86	197,05	197,84	97,84	96,8
2339,571	298,41	294,65	196,88	197,83	97,8	96,69
2340,999	298,2	294,44	196,7	197,83	97,77	96,58
2342,427	297,98	294,22	196,51	197,83	97,73	96,46
2343,855	297,75	294,01	196,32	197,82	97,68	96,34
2345,284	297,53	293,83	196,14	197,81	97,63	96,23
2346,712	297,33	293,65	195,97	197,81	97,58	96,14
2348,14	297,13	293,49	195,81	197,81	97,53	96,05
2349,569	296,93	293,36	195,65	197,81	97,48	95,97
2350,997	296,73	293,21	195,5	197,8	97,42	95,88
2352,425	296,55	293,08	195,36	197,79	97,36	95,8
2353,854	296,37	292,95	195,22	197,77	97,3	95,72
2355,282	296,21	292,83	195,1	197,76	97,23	95,65
2356,71	296,06	292,74	195	197,76	97,18	95,59
2358,138	295,92	292,66	194,89	197,74	97,13	95,52
2359,567	295,81	292,63	194,81	197,73	97,08	95,47
2360,995	295,72	292,61	194,75	197,72	97,04	95,43
2362,423	295,66	292,6	194,73	197,72	97	95,41
2363,852	295,64	292,59	194,73	197,73	96,95	95,41
2365,28	295,64	292,58	194,76	197,74	96,91	95,42
2366,708	295,7	292,56	194,83	197,77	96,88	95,48
2368,137	295,78	292,54	194,93	197,8	96,85	95,55
2369,565	295,86	292,54	195,02	197,82	96,81	95,61
2370,993	295,94	292,59	195,11	197,82	96,77	95,67
2372,422	296,02	292,68	195,2	197,82	96,74	95,71
2373,85	296,1	292,77	195,28	197,8	96,7	95,75
2375,278	296,18	292,85	195,36	197,76	96,66	95,78
2376,707	296,27	292,93	195,45	197,72	96,63	95,81
2378,135	296,37	293,01	195,55	197,69	96,59	95,85
2379,563	296,5	293,09	195,66	197,66	96,58	95,92

This journal is © The Royal Society of Chemistry 20xx

Please do not adjust margins

Please do not adjust margins

2380,991	296,63	293,16	195,74	197,64	96,57	95,98
2382,42	296,75	293,22	195,81	197,61	96,56	96,03
2383,848	296,88	293,26	195,9	197,6	96,55	96,1
2385,276	297,01	293,26	196	197,6	96,53	96,19
2386,705	297,15	293,26	196,1	197,61	96,53	96,28
2388,133	297,26	293,25	196,18	197,6	96,52	96,35
2389,561	297,32	293,25	196,21	197,56	96,51	96,35
2390,99	297,34	293,26	196,21	197,51	96,5	96,33
2392,418	297,36	293,26	196,21	197,46	96,5	96,31
2393,846	297,37	293,26	196,21	197,41	96,48	96,3
2395,274	297,39	293,25	196,21	197,36	96,46	96,28
2396,703	297,4	293,22	196,21	197,32	96,43	96,26
2398,131	297,42	293,19	196,21	197,28	96,39	96,26
2399,56	297,46	293,17	196,23	197,24	96,35	96,28
2400,988	297,5	293,14	196,26	197,2	96,3	96,29
2402,416	297,55	293,12	196,3	197,17	96,25	96,32
2403,844	297,61	293,09	196,34	197,14	96,2	96,35
2405,273	297,66	293,05	196,38	197,12	96,15	96,37
2406,701	297,72	293,01	196,43	197,09	96,09	96,39
2408,129	297,77	292,95	196,48	197,06	96,02	96,4
2409,558	297,82	292,89	196,52	197,03	95,96	96,41
2410,986	297,88	292,83	196,56	197,01	95,9	96,43
2412,414	297,97	292,77	196,62	197	95,83	96,45
2413,843	298,06	292,72	196,68	197	95,77	96,47
2415,271	298,15	292,66	196,74	197	95,7	96,49
2416,699	298,24	292,61	196,8	197,01	95,64	96,51
2418,127	298,34	292,55	196,87	197,03	95,57	96,53
2419,556	298,43	292,49	196,93	197,05	95,5	96,54
2420,984	298,5	292,43	196,99	197,07	95,43	96,55
2422,412	298,59	292,37	197,04	197,09	95,36	96,55
2423,841	298,66	292,31	197,09	197,12	95,28	96,56
2425,269	298,72	292,24	197,14	197,15	95,2	96,55
2426,697	298,78	292,19	197,18	197,18	95,12	96,54
2428,126	298,83	292,12	197,22	197,21	95,04	96,52
2429,554	298,88	292,06	197,25	197,24	94,97	96,5
2430,982	298,92	292	197,27	197,26	94,9	96,47
2432,411	298,94	291,93	197,29	197,28	94,82	96,44
2433,839	298,97	291,87	197,3	197,3	94,74	96,41
2435,267	298,99	291,81	197,31	197,32	94,66	96,37
2436,696	299,01	291,74	197,32	197,33	94,59	96,33
2438,124	299,04	291,67	197,33	197,34	94,52	96,29
2439,552	299,06	291,6	197,34	197,34	94,44	96,25
2440,98	299,08	291,52	197,36	197,35	94,36	96,21
2442,409	299,1	291,44	197,38	197,36	94,28	96,16
2443,837	299,1	291,35	197,4	197,36	94,2	96,1
2445,265	299,11	291,27	197,4	197,35	94,13	96,04
2446,694	299,1	291,18	197,41	197,34	94,06	95,98

Please do not adjust margins

Please do not adjust margins

2448,122	299,1	291,09	197,41	197,34	93,98	95,92
2449,55	299,09	291,01	197,41	197,33	93,91	95,87
2450,979	299,08	290,93	197,4	197,33	93,83	95,81
2452,407	299,08	290,83	197,4	197,34	93,75	95,76
2453,835	299,07	290,74	197,39	197,34	93,67	95,71
2455,263	299,06	290,64	197,37	197,34	93,59	95,65
2456,692	299,05	290,54	197,35	197,36	93,51	95,59
2458,12	299,02	290,43	197,33	197,36	93,42	95,53
2459,548	299	290,33	197,3	197,36	93,33	95,47
2460,977	298,98	290,22	197,28	197,38	93,25	95,41
2462,405	298,97	290,11	197,26	197,39	93,16	95,35
2463,833	298,95	290	197,23	197,39	93,08	95,29
2465,262	298,93	289,9	197,2	197,4	93,01	95,23
2466,69	298,92	289,79	197,17	197,4	92,93	95,18
2468,118	298,92	289,69	197,15	197,41	92,85	95,13
2469,547	298,91	289,59	197,13	197,43	92,77	95,09
2470,975	298,9	289,49	197,11	197,44	92,68	95,03
2472,403	298,89	289,39	197,09	197,45	92,6	94,98
2473,832	298,88	289,28	197,07	197,46	92,52	94,93
2475,26	298,88	289,18	197,05	197,46	92,44	94,87
2476,688	298,88	289,08	197,04	197,47	92,37	94,82
2478,116	298,88	288,98	197,02	197,48	92,28	94,77
2479,545	298,88	288,88	197,01	197,48	92,2	94,73
2480,973	298,88	288,79	197	197,49	92,12	94,67
2482,401	298,88	288,68	196,99	197,49	92,04	94,62
2483,83	298,88	288,58	196,98	197,49	91,96	94,56
2485,258	298,87	288,48	196,96	197,5	91,88	94,51
2486,686	298,86	288,38	196,94	197,5	91,81	94,45
2488,115	298,85	288,28	196,92	197,5	91,73	94,39
2489,543	298,84	288,18	196,9	197,49	91,65	94,33
2490,971	298,84	288,08	196,88	197,49	91,58	94,27
2492,4	298,84	287,98	196,86	197,49	91,5	94,22
2493,828	298,84	287,88	196,85	197,48	91,42	94,17
2495,256	298,84	287,78	196,85	197,48	91,35	94,11
2496,685	298,84	287,68	196,84	197,48	91,27	94,06
2498,113	298,84	287,58	196,83	197,49	91,19	94
2499,541	298,84	287,48	196,82	197,49	91,11	93,95
2500,969	298,84	287,38	196,8	197,49	91,04	93,89
2502,398	298,85	287,28	196,8	197,49	90,97	93,84
2503,826	298,87	287,19	196,8	197,5	90,9	93,8
2505,254	298,89	287,09	196,81	197,51	90,83	93,75
2506,683	298,9	287	196,81	197,52	90,76	93,69
2508,111	298,92	286,9	196,8	197,53	90,68	93,64
2509,539	298,92	286,8	196,8	197,53	90,61	93,58
2510,968	298,93	286,7	196,79	197,54	90,54	93,53
2512,396	298,94	286,6	196,78	197,55	90,47	93,46
2513,824	298,94	286,5	196,76	197,55	90,39	93,4

This journal is © The Royal Society of Chemistry 20xx

Please do not adjust margins

Please do not adjust margins

2515,252	298,95	286,4	196,75	197,56	90,33	93,34
2516,681	298,96	286,29	196,74	197,56	90,26	93,28
2518,109	298,97	286,2	196,74	197,58	90,2	93,23
2519,537	298,98	286,11	196,73	197,59	90,15	93,18
2520,966	298,98	286,01	196,73	197,59	90,09	93,12
2522,394	298,99	285,92	196,74	197,6	90,03	93,08
2523,822	299	285,82	196,75	197,61	89,97	93,03
2525,251	299	285,73	196,75	197,63	89,91	92,97
2526,679	299,01	285,63	196,76	197,64	89,86	92,92
2528,107	299,01	285,53	196,76	197,65	89,8	92,86
2529,536	299,02	285,44	196,76	197,66	89,76	92,81
2530,964	299,04	285,35	196,78	197,67	89,71	92,76
2532,392	299,04	285,25	196,79	197,69	89,66	92,7
2533,821	299,05	285,15	196,8	197,71	89,61	92,65
2535,249	299,05	285,05	196,8	197,72	89,56	92,59
2536,677	299,06	284,96	196,81	197,73	89,52	92,53
2538,105	299,07	284,87	196,82	197,75	89,48	92,48
2539,534	299,06	284,76	196,82	197,76	89,44	92,42
2540,962	299,06	284,66	196,82	197,77	89,4	92,35
2542,39	299,05	284,57	196,82	197,77	89,37	92,29
2543,819	299,05	284,47	196,82	197,78	89,33	92,23
2545,247	299,04	284,38	196,82	197,79	89,3	92,17
2546,675	299,04	284,28	196,82	197,79	89,27	92,12
2548,104	299,03	284,18	196,83	197,79	89,24	92,06
2549,532	299,02	284,08	196,84	197,79	89,21	92
2550,96	299,02	283,98	196,85	197,79	89,18	91,94
2552,388	299,01	283,88	196,86	197,79	89,15	91,87
2553,817	299	283,78	196,87	197,78	89,12	91,81
2555,245	298,99	283,68	196,88	197,78	89,1	91,74
2556,674	298,99	283,58	196,89	197,77	89,07	91,68
2558,102	298,99	283,48	196,91	197,77	89,05	91,61
2559,53	298,98	283,38	196,92	197,75	89,02	91,54
2560,958	298,98	283,27	196,94	197,74	89	91,48
2562,387	298,96	283,16	196,94	197,73	88,96	91,4
2563,815	298,94	283,05	196,94	197,7	88,93	91,33
2565,243	298,93	282,94	196,95	197,69	88,9	91,26
2566,672	298,92	282,83	196,96	197,68	88,86	91,2
2568,1	298,92	282,71	196,96	197,68	88,83	91,15
2569,528	298,92	282,59	196,97	197,67	88,8	91,09
2570,957	298,9	282,48	196,97	197,66	88,76	91,02
2572,385	298,89	282,37	196,97	197,66	88,72	90,95
2573,813	298,88	282,26	196,98	197,65	88,68	90,89
2575,241	298,87	282,14	196,99	197,63	88,63	90,82
2576,67	298,87	282,03	197	197,62	88,59	90,76
2578,098	298,86	281,91	197,01	197,6	88,54	90,7
2579,526	298,85	281,79	197,01	197,6	88,48	90,64
2580,955	298,83	281,68	197,01	197,59	88,43	90,58

This journal is © The Royal Society of Chemistry 20xx

Please do not adjust margins

Please do not adjust margins

2582,383	298,81	281,55	197	197,57	88,38	90,52
2583,811	298,79	281,43	196,98	197,55	88,33	90,46
2585,24	298,77	281,31	196,97	197,53	88,27	90,39
2586,668	298,76	281,18	196,97	197,52	88,2	90,33
2588,096	298,75	281,05	196,97	197,51	88,14	90,27
2589,525	298,74	280,91	196,96	197,49	88,07	90,21
2590,953	298,74	280,79	196,95	197,46	88,01	90,14
2592,381	298,73	280,66	196,93	197,43	87,94	90,06
2593,81	298,72	280,53	196,91	197,39	87,87	89,99
2595,238	298,71	280,39	196,88	197,35	87,8	89,92
2596,666	298,7	280,26	196,85	197,31	87,74	89,85
2598,094	298,69	280,12	196,83	197,27	87,67	89,78
2599,523	298,69	279,99	196,8	197,23	87,6	89,71
2600,951	298,68	279,85	196,76	197,18	87,53	89,64
2602,379	298,68	279,71	196,73	197,12	87,46	89,57
2603,808	298,68	279,57	196,71	197,06	87,39	89,5
2605,236	298,67	279,43	196,68	197	87,32	89,42
2606,664	298,68	279,29	196,65	196,95	87,24	89,35
2608,093	298,68	279,15	196,62	196,89	87,17	89,28
2609,521	298,67	279,01	196,58	196,82	87,1	89,2
2610,949	298,67	278,87	196,54	196,75	87,04	89,12
2612,377	298,67	278,72	196,5	196,68	86,97	89,06
2613,806	298,67	278,57	196,45	196,61	86,9	88,98
2615,234	298,67	278,42	196,4	196,54	86,84	88,91
2616,662	298,67	278,26	196,37	196,47	86,77	88,84
2618,091	298,69	278,11	196,34	196,41	86,7	88,78
2619,519	298,7	277,95	196,31	196,34	86,64	88,71
2620,947	298,71	277,79	196,28	196,28	86,57	88,64
2622,376	298,75	277,63	196,26	196,23	86,51	88,59
2623,804	298,77	277,46	196,23	196,17	86,45	88,53
2625,232	298,78	277,3	196,19	196,11	86,39	88,46
2626,661	298,8	277,14	196,16	196,04	86,33	88,39
2628,089	298,81	276,98	196,13	195,97	86,27	88,32
2629,517	298,81	276,82	196,1	195,9	86,21	88,25
2630,946	298,82	276,67	196,07	195,83	86,16	88,19
2632,374	298,83	276,51	196,04	195,75	86,1	88,12
2633,802	298,84	276,34	196,01	195,68	86,04	88,06
2635,23	298,84	276,17	195,98	195,61	85,98	88
2636,659	298,83	276,01	195,94	195,53	85,92	87,92
2638,087	298,83	275,85	195,91	195,46	85,86	87,85
2639,515	298,82	275,68	195,86	195,37	85,8	87,77
2640,944	298,81	275,52	195,83	195,29	85,74	87,7
2642,372	298,81	275,36	195,8	195,22	85,68	87,63
2643,8	298,8	275,2	195,77	195,14	85,62	87,55
2645,229	298,8	275,04	195,73	195,05	85,56	87,47
2646,657	298,79	274,89	195,7	194,97	85,5	87,39
2648,085	298,79	274,73	195,66	194,88	85,44	87,3

This journal is © The Royal Society of Chemistry 20xx

Please do not adjust margins

2649,514	298,78	274,57	195,62	194,8	85,37	87,23
2650,942	298,77	274,41	195,58	194,73	85,31	87,15
2652,37	298,76	274,25	195,54	194,65	85,24	87,07
2653,799	298,76	274,1	195,51	194,56	85,18	87
2655,227	298,75	273,94	195,46	194,48	85,11	86,92
2656,655	298,73	273,79	195,41	194,39	85,05	86,84
2658,083	298,72	273,65	195,37	194,3	84,99	86,76
2659,512	298,72	273,49	195,34	194,22	84,92	86,68
2660,94	298,72	273,34	195,31	194,15	84,85	86,61
2662,368	298,72	273,18	195,26	194,07	84,79	86,54
2663,797	298,71	273,02	195,2	193,99	84,72	86,46
2665,225	298,71	272,86	195,14	193,91	84,65	86,38
2666,653	298,69	272,71	195,08	193,82	84,59	86,3
2668,082	298,67	272,55	195,02	193,74	84,51	86,22
2669,51	298,66	272,39	194,97	193,66	84,44	86,14
2670,938	298,65	272,23	194,92	193,59	84,37	86,06
2672,366	298,65	272,07	194,88	193,51	84,29	85,98
2673,795	298,66	271,9	194,84	193,44	84,22	85,91
2675,223	298,66	271,74	194,8	193,36	84,14	85,84
2676,651	298,67	271,58	194,75	193,3	84,06	85,76
2678,08	298,67	271,42	194,72	193,24	83,98	85,69
2679,508	298,68	271,25	194,67	193,17	83,91	85,61
2680,936	298,68	271,09	194,62	193,1	83,84	85,54
2682,365	298,69	270,93	194,57	193,04	83,77	85,46
2683,793	298,7	270,77	194,54	192,98	83,69	85,39
2685,221	298,71	270,62	194,5	192,92	83,61	85,32
2686,65	298,72	270,46	194,45	192,85	83,52	85,25
2688,078	298,74	270,3	194,41	192,78	83,44	85,18
2689,506	298,74	270,14	194,37	192,71	83,36	85,1
2690,935	298,74	269,97	194,32	192,64	83,27	85,02
2692,363	298,74	269,8	194,26	192,57	83,2	84,94
2693,791	298,73	269,63	194,19	192,49	83,12	84,86
2695,219	298,72	269,46	194,13	192,41	83,04	84,78
2696,648	298,72	269,3	194,07	192,34	82,96	84,7
2698,076	298,72	269,14	194,01	192,27	82,88	84,62
2699,504	298,73	268,98	193,97	192,21	82,81	84,55
2700,933	298,73	268,82	193,91	192,14	82,73	84,47
2702,361	298,73	268,65	193,86	192,07	82,66	84,38
2703,789	298,74	268,49	193,8	192	82,6	84,3
2705,218	298,74	268,33	193,74	191,93	82,52	84,22
2706,646	298,73	268,16	193,68	191,85	82,45	84,15
2708,074	298,73	268	193,61	191,78	82,39	84,07
2709,502	298,73	267,83	193,53	191,7	82,33	83,99
2710,931	298,73	267,66	193,47	191,62	82,28	83,91
2712,359	298,73	267,49	193,41	191,55	82,23	83,83
2713,788	298,73	267,32	193,35	191,48	82,18	83,75
2715,216	298,74	267,15	193,28	191,4	82,13	83,67

This journal is © The Royal Society of Chemistry 20xx

Please do not adjust margins

2716,644	298,75	266,97	193,22	191,33	82,08	83,59
2718,073	298,76	266,79	193,16	191,25	82,03	83,51
2719,501	298,77	266,61	193,11	191,17	81,98	83,43
2720,929	298,76	266,45	193,06	191,09	81,92	83,34
2722,357	298,75	266,28	193	191,01	81,87	83,26
2723,786	298,73	266,12	192,94	190,92	81,83	83,17
2725,214	298,72	265,95	192,9	190,84	81,79	83,08
2726,642	298,73	265,77	192,86	190,76	81,75	83
2728,071	298,73	265,6	192,82	190,69	81,71	82,92
2729,499	298,72	265,43	192,78	190,61	81,67	82,83
2730,927	298,73	265,27	192,75	190,54	81,63	82,75
2732,355	298,72	265,11	192,71	190,48	81,59	82,67
2733,784	298,73	264,95	192,68	190,42	81,55	82,6
2735,212	298,75	264,79	192,65	190,36	81,52	82,53
2736,64	298,77	264,62	192,63	190,3	81,49	82,46
2738,069	298,79	264,46	192,6	190,23	81,45	82,39
2739,497	298,79	264,31	192,57	190,17	81,41	82,33
2740,925	298,79	264,15	192,53	190,11	81,36	82,26
2742,354	298,8	264	192,5	190,04	81,31	82,2
2743,782	298,79	263,84	192,45	189,96	81,26	82,13
2745,21	298,77	263,67	192,41	189,88	81,2	82,06
2746,639	298,78	263,51	192,37	189,82	81,14	82
2748,067	298,78	263,34	192,32	189,75	81,08	81,95
2749,495	298,79	263,17	192,29	189,69	81,01	81,89
2750,924	298,78	263,02	192,25	189,62	80,95	81,84
2752,352	298,78	262,87	192,2	189,55	80,88	81,77
2753,78	298,79	262,72	192,17	189,49	80,8	81,72
2755,208	298,79	262,57	192,14	189,44	80,73	81,67
2756,637	298,78	262,42	192,12	189,38	80,65	81,62
2758,065	298,76	262,27	192,09	189,31	80,57	81,57
2759,493	298,73	262,11	192,03	189,24	80,48	81,51
2760,922	298,7	261,96	191,97	189,16	80,39	81,45
2762,35	298,67	261,81	191,92	189,09	80,29	81,4
2763,778	298,63	261,65	191,87	189,01	80,19	81,35
2765,207	298,59	261,49	191,81	188,93	80,09	81,29
2766,635	298,54	261,33	191,75	188,86	79,99	81,24
2768,063	298,48	261,17	191,7	188,77	79,87	81,19
2769,491	298,42	261,01	191,64	188,69	79,75	81,14
2770,92	298,35	260,85	191,59	188,61	79,63	81,09
2772,348	298,3	260,69	191,56	188,53	79,51	81,05
2773,776	298,23	260,53	191,53	188,45	79,39	81,01
2775,205	298,15	260,37	191,49	188,36	79,26	80,96
2776,633	298,06	260,21	191,45	188,28	79,13	80,91
2778,061	297,96	260,05	191,39	188,19	79,01	80,86
2779,49	297,85	259,88	191,31	188,08	78,88	80,8
2780,918	297,73	259,71	191,24	187,98	78,75	80,75
2782,346	297,6	259,54	191,17	187,88	78,62	80,7

This journal is © The Royal Society of Chemistry 20xx

Please do not adjust margins

Please do not adjust margins

2783,775	297,48	259,37	191,1	187,78	78,47	80,64
2785,203	297,35	259,2	191,03	187,68	78,33	80,59
2786,631	297,22	259,04	190,96	187,59	78,18	80,55
2788,06	297,09	258,87	190,88	187,49	78,05	80,49
2789,488	296,96	258,7	190,79	187,39	77,91	80,45
2790,916	296,83	258,53	190,7	187,28	77,77	80,4
2792,344	296,7	258,36	190,61	187,18	77,62	80,36
2793,773	296,55	258,19	190,51	187,07	77,47	80,32
2795,201	296,4	258,01	190,39	186,96	77,32	80,26
2796,629	296,25	257,84	190,29	186,86	77,17	80,22
2798,058	296,1	257,66	190,17	186,75	77,01	80,16
2799,486	295,94	257,47	190,05	186,64	76,85	80,11
2800,914	295,78	257,29	189,93	186,53	76,69	80,05
2802,343	295,62	257,1	189,81	186,42	76,52	80
2803,771	295,48	256,91	189,68	186,31	76,35	79,96
2805,199	295,34	256,73	189,57	186,2	76,19	79,91
2806,628	295,18	256,54	189,44	186,09	76,02	79,85
2808,056	295,02	256,36	189,31	185,98	75,85	79,8
2809,484	294,86	256,17	189,18	185,87	75,68	79,74
2810,913	294,7	255,97	189,05	185,76	75,5	79,69
2812,341	294,54	255,78	188,92	185,65	75,33	79,64
2813,769	294,37	255,59	188,77	185,53	75,16	79,59
2815,198	294,21	255,4	188,62	185,42	74,99	79,54
2816,626	294,05	255,22	188,46	185,31	74,81	79,49
2818,054	293,89	255,04	188,31	185,19	74,64	79,46
2819,482	293,73	254,86	188,18	185,09	74,46	79,44
2820,911	293,56	254,69	188,03	184,98	74,29	79,41
2822,339	293,4	254,5	187,88	184,89	74,12	79,39
2823,767	293,27	254,31	187,75	184,82	73,96	79,38
2825,196	293,17	254,1	187,61	184,77	73,8	79,39
2826,624	293,09	253,89	187,47	184,73	73,64	79,39
2828,052	293,02	253,66	187,31	184,71	73,5	79,4
2829,48	292,94	253,43	187,14	184,7	73,35	79,41
2830,909	292,87	253,18	186,93	184,71	73,2	79,43
2832,337	292,8	252,94	186,71	184,72	73,05	79,45
2833,765	292,73	252,69	186,48	184,73	72,89	79,47
2835,194	292,65	252,45	186,23	184,75	72,75	79,49
2836,622	292,56	252,21	185,96	184,76	72,6	79,51
2838,05	292,47	251,98	185,72	184,8	72,45	79,54
2839,479	292,39	251,75	185,49	184,85	72,3	79,58
2840,907	292,28	251,52	185,23	184,89	72,15	79,6
2842,335	292,15	251,29	184,94	184,92	72,01	79,62
2843,764	292	251,06	184,64	184,96	71,86	79,63
2845,192	291,84	250,83	184,35	184,99	71,7	79,63
2846,62	291,69	250,61	184,05	185,03	71,54	79,64
2848,049	291,53	250,4	183,74	185,07	71,39	79,65
2849,477	291,35	250,19	183,41	185,11	71,23	79,66

Please do not adjust margins

Please do not adjust margins

2850,905	291,18	249,98	183,06	185,16	71,07	79,66
2852,333	290,98	249,78	182,71	185,21	70,9	79,66
2853,762	290,77	249,59	182,33	185,25	70,74	79,64
2855,19	290,55	249,41	181,93	185,28	70,59	79,62
2856,618	290,31	249,22	181,51	185,31	70,42	79,59
2858,047	290,06	249,05	181,09	185,33	70,26	79,55
2859,475	289,81	248,88	180,66	185,34	70,09	79,5
2860,903	289,55	248,71	180,22	185,35	69,93	79,46
2862,332	289,3	248,55	179,79	185,36	69,78	79,41
2863,76	289,03	248,4	179,36	185,35	69,62	79,36
2865,188	288,75	248,25	178,93	185,34	69,47	79,31
2866,616	288,49	248,13	178,5	185,32	69,31	79,25
2868,045	288,22	248,01	178,08	185,3	69,16	79,19
2869,473	287,98	247,92	177,66	185,28	69,01	79,15
2870,902	287,75	247,83	177,27	185,25	68,88	79,1
2872,33	287,52	247,74	176,9	185,22	68,75	79,06
2873,758	287,31	247,65	176,53	185,21	68,63	79,03
2875,187	287,11	247,57	176,18	185,2	68,52	78,99
2876,615	286,93	247,49	175,83	185,2	68,42	78,95
2878,043	286,8	247,4	175,51	185,2	68,34	78,92
2879,471	286,69	247,29	175,19	185,21	68,26	78,89
2880,9	286,61	247,19	174,9	185,23	68,18	78,86
2882,328	286,53	247,09	174,61	185,23	68,1	78,83
2883,756	286,43	246,98	174,33	185,21	68,03	78,8
2885,185	286,34	246,88	174,09	185,2	67,95	78,77
2886,613	286,27	246,78	173,88	185,18	67,87	78,75
2888,041	286,2	246,69	173,71	185,14	67,79	78,72
2889,469	286,13	246,6	173,56	185,09	67,7	78,7
2890,898	286,06	246,49	173,44	185,03	67,62	78,68
2892,326	286,01	246,38	173,36	184,98	67,53	78,68
2893,754	285,98	246,26	173,31	184,92	67,43	78,7
2895,183	285,93	246,11	173,29	184,87	67,32	78,7
2896,611	285,89	245,94	173,28	184,82	67,21	78,71
2898,039	285,86	245,75	173,26	184,78	67,09	78,71
2899,468	285,81	245,53	173,23	184,74	66,96	78,7
2900,896	285,76	245,28	173,19	184,71	66,81	78,69
2902,324	285,7	245,02	173,14	184,68	66,66	78,68
2903,753	285,65	244,75	173,06	184,67	66,52	78,68
2905,181	285,59	244,47	172,95	184,66	66,36	78,67
2906,609	285,5	244,17	172,82	184,65	66,19	78,65
2908,038	285,39	243,86	172,67	184,64	66,02	78,62
2909,466	285,26	243,55	172,51	184,62	65,85	78,59
2910,894	285,09	243,24	172,33	184,59	65,67	78,55
2912,323	284,9	242,94	172,14	184,55	65,49	78,5
2913,751	284,68	242,64	171,94	184,51	65,3	78,45
2915,179	284,43	242,36	171,73	184,43	65,1	78,39
2916,607	284,17	242,11	171,53	184,34	64,91	78,33

This journal is © The Royal Society of Chemistry 20xx

Please do not adjust margins

Please do not adjust margins

2918,036	283,89	241,88	171,34	184,24	64,71	78,27
2919,464	283,6	241,68	171,17	184,13	64,51	78,21
2920,892	283,3	241,52	171	184,01	64,32	78,16
2922,321	283	241,39	170,85	183,88	64,14	78,1
2923,749	282,71	241,29	170,73	183,73	63,98	78,04
2925,177	282,42	241,22	170,65	183,58	63,82	77,99
2926,605	282,14	241,19	170,6	183,41	63,69	77,94
2928,034	281,85	241,19	170,59	183,24	63,57	77,89
2929,462	281,56	241,22	170,59	183,04	63,46	77,83
2930,89	281,26	241,27	170,63	182,83	63,36	77,77
2932,319	280,98	241,37	170,71	182,61	63,29	77,7
2933,747	280,7	241,49	170,85	182,35	63,22	77,63
2935,175	280,44	241,62	171,04	182,08	63,17	77,56
2936,604	280,16	241,79	171,26	181,79	63,11	77,5
2938,032	279,91	241,97	171,54	181,48	63,07	77,43
2939,46	279,68	242,18	171,88	181,18	63,03	77,38
2940,889	279,47	242,39	172,26	180,89	63,01	77,32
2942,317	279,29	242,61	172,64	180,6	63	77,26
2943,745	279,15	242,84	173,04	180,33	63	77,22
2945,174	279,04	243,05	173,41	180,1	63,02	77,18
2946,602	278,99	243,24	173,73	179,95	63,06	77,17
2948,03	279,01	243,4	173,98	179,87	63,13	77,18
2949,458	279,08	243,55	174,14	179,87	63,23	77,19
2950,887	279,19	243,68	174,19	179,96	63,35	77,21
2952,315	279,32	243,78	174,11	180,12	63,49	77,23
2953,743	279,51	243,86	173,91	180,35	63,66	77,25
2955,172	279,75	243,91	173,6	180,64	63,85	77,28
2956,6	280,01	243,95	173,19	180,98	64,05	77,3
2958,028	280,29	243,98	172,7	181,34	64,27	77,32
2959,457	280,58	244	172,16	181,74	64,49	77,34
2960,885	280,89	244	171,58	182,14	64,72	77,37
2962,313	281,18	243,98	170,97	182,53	64,93	77,39
2963,742	281,44	243,96	170,36	182,9	65,14	77,4
2965,17	281,68	243,94	169,75	183,25	65,33	77,39
2966,598	281,91	243,92	169,15	183,58	65,51	77,39
2968,027	282,12	243,9	168,59	183,84	65,66	77,38
2969,455	282,29	243,88	168,06	184,05	65,8	77,35
2970,883	282,44	243,87	167,58	184,21	65,92	77,31
2972,312	282,57	243,86	167,16	184,32	66,01	77,26
2973,74	282,7	243,84	166,82	184,38	66,08	77,21
2975,168	282,8	243,84	166,56	184,39	66,12	77,15
2976,596	282,88	243,86	166,38	184,34	66,13	77,1
2978,025	282,94	243,88	166,29	184,23	66,13	77,04
2979,453	282,96	243,9	166,3	184,05	66,11	76,97
2980,881	282,96	243,92	166,4	183,81	66,05	76,88
2982,31	282,94	243,94	166,6	183,5	65,96	76,79
2983,738	282,87	243,96	166,88	183,13	65,85	76,69

This journal is © The Royal Society of Chemistry 20xx

Please do not adjust margins

2985,166	282,77	243,97	167,25	182,68	65,71	76,58
2986,594	282,64	243,98	167,7	182,19	65,54	76,45
2988,023	282,49	243,98	168,23	181,65	65,34	76,32
2989,451	282,33	243,98	168,85	181,07	65,12	76,2
2990,879	282,15	243,96	169,55	180,44	64,87	76,06
2992,308	281,95	243,94	170,3	179,78	64,59	75,92
2993,736	281,74	243,92	171,09	179,11	64,31	75,77
2995,164	281,52	243,9	171,9	178,42	64	75,62
2996,593	281,28	243,86	172,71	177,74	63,7	75,46
2998,021	281,04	243,82	173,5	177,09	63,39	75,32
2999,449	280,81	243,76	174,25	176,47	63,08	75,18
3000,878	280,6	243,68	174,93	175,9	62,78	75,04
3002,306	280,41	243,58	175,53	175,4	62,48	74,92
3003,734	280,24	243,47	176,02	174,96	62,2	74,8
3005,163	280,08	243,37	176,43	174,56	61,93	74,7
3006,591	279,92	243,26	176,75	174,2	61,68	74,59
3008,019	279,78	243,14	176,99	173,89	61,43	74,49
3009,448	279,66	243,01	177,16	173,61	61,19	74,4
3010,876	279,55	242,87	177,29	173,35	60,95	74,31
3012,304	279,42	242,73	177,38	173,11	60,71	74,21
3013,732	279,27	242,59	177,43	172,88	60,47	74,11
3015,161	279,11	242,43	177,44	172,65	60,23	74
3016,589	278,96	242,26	177,44	172,43	59,99	73,91
3018,017	278,8	242,09	177,43	172,21	59,75	73,82
3019,446	278,63	241,94	177,4	171,99	59,49	73,72
3020,874	278,47	241,78	177,38	171,77	59,23	73,63
3022,302	278,3	241,61	177,35	171,56	58,97	73,56
3023,73	278,14	241,43	177,31	171,35	58,71	73,49
3025,159	277,95	241,25	177,25	171,11	58,45	73,41
3026,587	277,73	241,07	177,16	170,88	58,18	73,31
3028,016	277,54	240,89	177,08	170,68	57,91	73,23
3029,444	277,34	240,71	176,99	170,48	57,64	73,15
3030,872	277,14	240,54	176,91	170,28	57,36	73,07
3032,301	276,94	240,38	176,81	170,07	57,09	73
3033,729	276,72	240,21	176,7	169,86	56,81	72,92
3035,157	276,48	240,04	176,6	169,66	56,53	72,84
3036,585	276,25	239,87	176,48	169,46	56,24	72,75
3038,014	276	239,68	176,33	169,26	55,94	72,66
3039,442	275,76	239,5	176,18	169,06	55,65	72,58
3040,87	275,52	239,32	176,03	168,85	55,35	72,49
3042,299	275,27	239,14	175,87	168,66	55,05	72,4
3043,727	275,03	238,98	175,71	168,48	54,74	72,32
3045,155	274,8	238,81	175,56	168,3	54,44	72,25
3046,583	274,56	238,65	175,39	168,12	54,14	72,16
3048,012	274,32	238,48	175,23	167,92	53,84	72,06
3049,44	274,06	238,33	175,06	167,73	53,54	71,97
3050,868	273,81	238,19	174,88	167,55	53,24	71,89

This journal is © The Royal Society of Chemistry 20xx

Please do not adjust margins

3052,297	273,54	238,06	174,71	167,38	52,94	71,81
3053,725	273,29	237,92	174,55	167,23	52,63	71,73
3055,153	273,06	237,78	174,4	167,1	52,32	71,67
3056,582	272,82	237,63	174,23	166,96	52,02	71,59
3058,01	272,58	237,49	174,05	166,81	51,73	71,52
3059,438	272,35	237,36	173,87	166,68	51,43	71,46
3060,867	272,12	237,24	173,68	166,53	51,12	71,4
3062,295	271,89	237,12	173,5	166,39	50,82	71,35
3063,723	271,67	237	173,33	166,26	50,53	71,31
3065,152	271,44	236,89	173,14	166,14	50,23	71,27
3066,58	271,22	236,79	172,96	166,03	49,94	71,23
3068,008	270,99	236,69	172,78	165,91	49,64	71,2
3069,437	270,77	236,58	172,59	165,79	49,35	71,15
3070,865	270,56	236,47	172,39	165,67	49,06	71,1
3072,293	270,35	236,36	172,18	165,56	48,78	71,06
3073,721	270,15	236,26	171,97	165,46	48,5	71,03
3075,15	269,95	236,17	171,77	165,36	48,22	71
3076,578	269,75	236,07	171,57	165,24	47,94	70,98
3078,006	269,55	235,95	171,36	165,13	47,66	70,95
3079,435	269,34	235,84	171,16	165,03	47,39	70,92
3080,863	269,11	235,75	170,92	164,91	47,1	70,88
3082,291	268,87	235,67	170,68	164,79	46,82	70,83
3083,719	268,65	235,58	170,44	164,68	46,55	70,79
3085,148	268,43	235,48	170,19	164,57	46,28	70,74
3086,576	268,22	235,4	169,95	164,46	46	70,7
3088,004	267,99	235,31	169,71	164,33	45,72	70,65
3089,433	267,76	235,21	169,45	164,2	45,44	70,6
3090,861	267,51	235,1	169,17	164,07	45,17	70,53
3092,289	267,26	235	168,9	163,94	44,9	70,47
3093,718	267,01	234,9	168,62	163,82	44,63	70,41
3095,146	266,77	234,79	168,33	163,69	44,36	70,36
3096,574	266,54	234,66	168,06	163,55	44,09	70,3
3098,003	266,3	234,56	167,8	163,43	43,82	70,25
3099,431	266,06	234,45	167,52	163,3	43,55	70,19
3100,859	265,82	234,34	167,24	163,17	43,28	70,13
3102,288	265,59	234,22	166,95	163,03	43,01	70,06
3103,716	265,35	234,11	166,66	162,89	42,75	70
3105,144	265,14	234	166,39	162,77	42,48	69,95
3106,573	264,92	233,88	166,12	162,65	42,21	69,92
3108,001	264,7	233,76	165,84	162,54	41,95	69,87
3109,429	264,48	233,62	165,56	162,42	41,69	69,82
3110,857	264,27	233,48	165,28	162,3	41,42	69,78
3112,286	264,05	233,33	165	162,17	41,15	69,74
3113,714	263,84	233,2	164,72	162,04	40,9	69,7
3115,142	263,63	233,07	164,43	161,9	40,64	69,67
3116,571	263,43	232,95	164,15	161,76	40,38	69,64
3117,999	263,22	232,83	163,89	161,63	40,11	69,61

This journal is © The Royal Society of Chemistry 20xx

Please do not adjust margins

Please do not adjust margins

3119,427	263	232,69	163,62	161,49	39,85	69,58
3120,856	262,77	232,55	163,33	161,36	39,58	69,54
3122,284	262,54	232,4	163,05	161,23	39,32	69,48
3123,712	262,34	232,24	162,77	161,1	39,05	69,43
3125,141	262,14	232,07	162,47	160,97	38,79	69,38
3126,569	261,93	231,9	162,18	160,84	38,53	69,32
3127,997	261,72	231,74	161,89	160,71	38,28	69,26
3129,426	261,53	231,59	161,59	160,6	38,03	69,2
3130,854	261,35	231,43	161,32	160,49	37,77	69,15
3132,282	261,17	231,26	161,06	160,39	37,52	69,1
3133,71	260,97	231,09	160,78	160,27	37,27	69,03
3135,139	260,78	230,93	160,49	160,15	37,01	68,95
3136,567	260,6	230,77	160,19	160,05	36,75	68,9
3137,995	260,4	230,6	159,9	159,94	36,51	68,84
3139,424	260,21	230,42	159,61	159,83	36,27	68,78
3140,852	260,02	230,25	159,32	159,72	36,03	68,72
3142,28	259,83	230,07	159,01	159,6	35,79	68,65
3143,708	259,63	229,89	158,7	159,47	35,54	68,57
3145,137	259,43	229,71	158,4	159,34	35,31	68,51
3146,565	259,22	229,52	158,08	159,21	35,08	68,42
3147,993	259	229,34	157,75	159,08	34,86	68,33
3149,422	258,79	229,16	157,43	158,95	34,62	68,25
3150,85	258,59	228,97	157,13	158,83	34,39	68,17
3152,278	258,38	228,78	156,84	158,71	34,16	68,09
3153,707	258,16	228,61	156,52	158,58	33,92	67,99
3155,135	257,95	228,44	156,2	158,46	33,69	67,9
3156,563	257,76	228,26	155,88	158,34	33,47	67,82
3157,992	257,58	228,08	155,57	158,22	33,24	67,74
3159,42	257,42	227,91	155,26	158,12	33,01	67,67
3160,848	257,24	227,75	154,95	158,01	32,79	67,6
3162,277	257,05	227,57	154,64	157,92	32,57	67,53
3163,705	256,84	227,41	154,32	157,83	32,35	67,44
3165,133	256,64	227,24	154	157,74	32,14	67,36
3166,562	256,45	227,08	153,67	157,63	31,93	67,29
3167,99	256,25	226,93	153,34	157,54	31,72	67,22
3169,418	256,06	226,79	153,02	157,46	31,52	67,18
3170,846	255,88	226,65	152,69	157,39	31,32	67,13
3172,275	255,69	226,5	152,35	157,3	31,11	67,07
3173,703	255,5	226,35	152,01	157,23	30,91	67,01
3175,131	255,3	226,21	151,67	157,17	30,71	66,97
3176,56	255,1	226,07	151,32	157,09	30,52	66,91
3177,988	254,91	225,94	150,98	157,02	30,32	66,85
3179,416	254,74	225,81	150,65	156,97	30,14	66,81
3180,844	254,58	225,7	150,3	156,92	29,96	66,76
3182,273	254,42	225,61	149,95	156,9	29,78	66,72
3183,701	254,24	225,52	149,63	156,87	29,61	66,69
3185,13	254,07	225,42	149,3	156,84	29,45	66,66

This journal is © The Royal Society of Chemistry 20xx

Please do not adjust margins

Please do not adjust margins

3186,558	253,93	225,33	148,98	156,84	29,28	66,65
3187,986	253,78	225,23	148,65	156,83	29,12	66,65
3189,415	253,61	225,15	148,32	156,81	28,97	66,65
3190,843	253,46	225,07	147,99	156,81	28,81	66,66
3192,271	253,31	225	147,66	156,81	28,66	66,68
3193,699	253,16	224,94	147,33	156,8	28,52	66,71
3195,128	252,99	224,88	146,99	156,79	28,39	66,73
3196,556	252,82	224,83	146,66	156,79	28,26	66,74
3197,984	252,68	224,79	146,31	156,8	28,13	66,77
3199,413	252,53	224,75	145,96	156,8	27,99	66,8
3200,841	252,39	224,72	145,63	156,81	27,86	66,83
3202,269	252,27	224,69	145,32	156,84	27,75	66,87
3203,698	252,12	224,67	145,01	156,87	27,63	66,88
3205,126	251,97	224,66	144,69	156,89	27,51	66,9
3206,554	251,84	224,66	144,41	156,94	27,39	66,94
3207,982	251,72	224,66	144,15	157	27,27	67,01
3209,411	251,61	224,69	143,88	157,06	27,16	67,08
3210,839	251,5	224,72	143,59	157,13	27,05	67,15
3212,267	251,41	224,75	143,32	157,2	26,94	67,24
3213,696	251,35	224,79	143,06	157,28	26,84	67,33
3215,124	251,27	224,83	142,79	157,36	26,74	67,4
3216,552	251,18	224,89	142,51	157,45	26,65	67,47
3217,981	251,07	224,95	142,24	157,54	26,57	67,55
3219,409	250,97	225,01	141,96	157,65	26,48	67,63
3220,837	250,89	225,08	141,69	157,78	26,4	67,72
3222,266	250,82	225,15	141,42	157,88	26,33	67,83
3223,694	250,75	225,21	141,16	157,98	26,26	67,92
3225,122	250,68	225,29	140,91	158,1	26,2	68,03
3226,551	250,61	225,36	140,66	158,23	26,15	68,12
3227,979	250,54	225,44	140,42	158,34	26,09	68,21
3229,407	250,48	225,53	140,17	158,47	26,04	68,3
3230,835	250,42	225,63	139,92	158,59	25,99	68,39
3232,264	250,37	225,74	139,66	158,69	25,96	68,5
3233,692	250,31	225,85	139,41	158,79	25,93	68,59
3235,12	250,24	225,96	139,17	158,88	25,89	68,69
3236,549	250,2	226,05	138,93	159	25,86	68,8
3237,977	250,17	226,14	138,7	159,11	25,84	68,91
3239,405	250,14	226,24	138,5	159,22	25,81	69,02
3240,833	250,1	226,32	138,29	159,31	25,78	69,12
3242,262	250,06	226,41	138,06	159,4	25,76	69,22
3243,69	250,02	226,51	137,83	159,51	25,74	69,32
3245,118	249,98	226,61	137,59	159,63	25,72	69,43
3246,547	249,96	226,72	137,36	159,73	25,7	69,54
3247,975	249,96	226,82	137,16	159,85	25,7	69,66
3249,403	249,94	226,9	136,94	159,96	25,72	69,77
3250,832	249,92	227	136,7	160,07	25,72	69,88
3252,26	249,91	227,1	136,47	160,21	25,74	69,99

This journal is © The Royal Society of Chemistry 20xx

Please do not adjust margins

Please do not adjust margins

3253,688	249,92	227,2	136,26	160,36	25,75	70,1
3255,117	249,92	227,3	136,04	160,5	25,76	70,21
3256,545	249,94	227,41	135,85	160,64	25,77	70,31
3257,973	249,96	227,5	135,67	160,77	25,79	70,4
3259,402	249,97	227,6	135,5	160,92	25,81	70,48
3260,83	249,99	227,7	135,34	161,07	25,83	70,58
3262,258	250	227,81	135,17	161,23	25,85	70,69
3263,687	250,04	227,93	135,03	161,42	25,86	70,83
3265,115	250,09	228,04	134,9	161,61	25,89	70,96
3266,543	250,13	228,17	134,74	161,78	25,92	71,08
3267,971	250,18	228,29	134,58	161,97	25,95	71,21
3269,4	250,25	228,4	134,43	162,17	25,98	71,35
3270,828	250,32	228,53	134,28	162,38	26,02	71,47
3272,256	250,37	228,67	134,13	162,6	26,07	71,58
3273,685	250,41	228,8	133,97	162,81	26,11	71,68
3275,113	250,45	228,92	133,82	163,01	26,16	71,78
3276,541	250,5	229,04	133,68	163,21	26,21	71,88
3277,97	250,55	229,17	133,53	163,41	26,27	71,97
3279,398	250,6	229,31	133,41	163,6	26,34	72,07
3280,826	250,67	229,44	133,31	163,81	26,4	72,19
3282,255	250,74	229,59	133,19	164,05	26,46	72,3
3283,683	250,8	229,74	133,05	164,26	26,52	72,42
3285,111	250,88	229,9	132,91	164,47	26,58	72,55
3286,54	250,97	230,05	132,79	164,68	26,65	72,7
3287,968	251,05	230,2	132,68	164,87	26,73	72,84
3289,396	251,14	230,34	132,59	165,07	26,8	72,99
3290,824	251,22	230,5	132,5	165,28	26,86	73,12
3292,253	251,26	230,65	132,39	165,46	26,92	73,23
3293,681	251,33	230,78	132,26	165,64	26,98	73,34
3295,109	251,41	230,91	132,14	165,84	27,04	73,45
3296,538	251,45	231,05	132,03	166,02	27,09	73,54
3297,966	251,5	231,18	131,92	166,2	27,13	73,65
3299,394	251,56	231,34	131,79	166,39	27,18	73,77
3300,823	251,63	231,5	131,66	166,59	27,23	73,9
3302,251	251,72	231,66	131,55	166,8	27,29	74,03
3303,679	251,79	231,79	131,48	167	27,36	74,16
3305,107	251,84	231,91	131,4	167,2	27,42	74,27
3306,536	251,88	232,03	131,27	167,39	27,49	74,38
3307,964	251,93	232,17	131,13	167,56	27,54	74,48
3309,392	251,98	232,29	131,01	167,73	27,59	74,59
3310,821	252,02	232,41	130,9	167,92	27,64	74,68
3312,249	252,05	232,52	130,79	168,09	27,7	74,76
3313,677	252,09	232,63	130,69	168,27	27,76	74,85
3315,106	252,11	232,73	130,58	168,46	27,83	74,94
3316,534	252,14	232,84	130,48	168,66	27,89	75,04
3317,962	252,18	232,97	130,38	168,86	27,95	75,16
3319,391	252,18	233,1	130,27	169,03	28	75,25

 This journal is © The Royal Society of Chemistry 20xx

Please do not adjust margins

Please do not adjust margins

3320,819	252,18	233,23	130,16	169,2	28,05	75,34
3322,247	252,18	233,37	130,05	169,38	28,12	75,43
3323,676	252,17	233,5	129,94	169,55	28,2	75,52
3325,104	252,17	233,62	129,83	169,74	28,27	75,62
3326,532	252,15	233,74	129,72	169,92	28,36	75,69
3327,96	252,13	233,88	129,61	170,09	28,44	75,75
3329,389	252,12	234,01	129,51	170,27	28,52	75,83
3330,817	252,11	234,13	129,4	170,45	28,6	75,91
3332,245	252,12	234,25	129,33	170,63	28,68	76
3333,674	252,14	234,39	129,28	170,84	28,76	76,12
3335,102	252,14	234,53	129,22	171,04	28,85	76,22
3336,53	252,13	234,66	129,14	171,24	28,93	76,33
3337,958	252,12	234,79	129,06	171,45	29,01	76,43
3339,387	252,1	234,93	128,97	171,64	29,09	76,51
3340,815	252,09	235,09	128,91	171,83	29,19	76,6
3342,244	252,1	235,25	128,89	172,02	29,29	76,7
3343,672	252,1	235,41	128,87	172,23	29,4	76,8
3345,1	252,1	235,6	128,84	172,44	29,52	76,9
3346,529	252,08	235,79	128,78	172,64	29,62	76,98
3347,957	252,05	235,99	128,7	172,81	29,72	77,04
3349,385	252,07	236,17	128,66	173	29,84	77,14
3350,813	252,13	236,33	128,65	173,22	29,95	77,25
3352,242	252,2	236,52	128,65	173,44	30,06	77,38
3353,67	252,26	236,72	128,66	173,67	30,16	77,51
3355,098	252,31	236,93	128,68	173,89	30,28	77,65
3356,527	252,36	237,15	128,69	174,13	30,41	77,78
3357,955	252,4	237,34	128,71	174,37	30,54	77,9
3359,383	252,44	237,53	128,75	174,61	30,67	78,01
3360,812	252,49	237,71	128,8	174,82	30,8	78,13
3362,24	252,54	237,91	128,83	175	30,93	78,23
3363,668	252,61	238,13	128,87	175,23	31,07	78,35
3365,096	252,67	238,34	128,93	175,46	31,21	78,47
3366,525	252,71	238,55	128,97	175,69	31,36	78,58
3367,953	252,77	238,79	129,03	175,93	31,53	78,69
3369,381	252,83	239,03	129,11	176,17	31,69	78,82
3370,81	252,88	239,26	129,18	176,39	31,85	78,96
3372,238	252,94	239,5	129,26	176,6	32,02	79,08
3373,666	253,02	239,74	129,35	176,79	32,18	79,2
3375,095	253,09	239,98	129,43	176,99	32,33	79,32
3376,523	253,15	240,23	129,49	177,19	32,49	79,43
3377,951	253,2	240,48	129,57	177,41	32,66	79,55
3379,38	253,29	240,74	129,68	177,64	32,86	79,69
3380,808	253,35	241	129,79	177,85	33,07	79,83
3382,236	253,4	241,24	129,89	178,05	33,26	79,95
3383,665	253,47	241,49	130	178,26	33,45	80,08
3385,093	253,56	241,77	130,13	178,47	33,66	80,23
3386,521	253,67	242,04	130,27	178,68	33,86	80,38

This journal is © The Royal Society of Chemistry 20xx

Please do not adjust margins

Please do not adjust margins

3387,949	253,78	242,31	130,43	178,89	34,08	80,53
3389,378	253,87	242,56	130,59	179,11	34,29	80,66
3390,806	253,95	242,81	130,72	179,31	34,51	80,79
3392,234	254,03	243,06	130,85	179,49	34,72	80,89
3393,663	254,12	243,32	130,99	179,67	34,93	80,99
3395,091	254,22	243,6	131,14	179,85	35,16	81,09
3396,519	254,3	243,87	131,31	180,03	35,39	81,2
3397,948	254,41	244,13	131,49	180,22	35,63	81,33
3399,376	254,54	244,39	131,68	180,41	35,87	81,48
3400,804	254,67	244,65	131,87	180,59	36,1	81,61
3402,232	254,78	244,93	132,05	180,76	36,33	81,73
3403,661	254,89	245,19	132,22	180,92	36,56	81,85
3405,089	255,05	245,45	132,44	181,1	36,79	81,99
3406,517	255,24	245,71	132,67	181,3	37,03	82,15
3407,946	255,39	245,97	132,87	181,48	37,26	82,28
3409,374	255,52	246,25	133,06	181,64	37,49	82,4
3410,802	255,65	246,53	133,28	181,81	37,75	82,53
3412,231	255,79	246,82	133,52	181,97	38,01	82,65
3413,659	255,97	247,11	133,77	182,15	38,27	82,8
3415,087	256,17	247,38	134,04	182,34	38,53	82,96
3416,516	256,34	247,64	134,3	182,51	38,79	83,08
3417,944	256,53	247,93	134,6	182,7	39,06	83,2
3419,372	256,73	248,21	134,91	182,92	39,33	83,36
3420,801	256,91	248,49	135,17	183,11	39,6	83,51
3422,229	257,07	248,76	135,39	183,28	39,88	83,63
3423,657	257,22	249,03	135,62	183,43	40,15	83,72
3425,085	257,39	249,31	135,89	183,61	40,42	83,84
3426,514	257,58	249,58	136,18	183,79	40,68	83,97
3427,942	257,75	249,85	136,45	183,94	40,95	84,08
3429,37	257,93	250,18	136,71	184,09	41,23	84,18
3430,799	258,07	250,51	136,96	184,23	41,5	84,29
3432,227	258,19	250,82	137,21	184,38	41,78	84,38
3433,655	258,36	251,14	137,49	184,55	42,09	84,5
3435,084	258,54	251,45	137,79	184,72	42,4	84,64
3436,512	258,72	251,77	138,09	184,88	42,7	84,79
3437,94	258,9	252,08	138,39	185,05	43,01	84,92
3439,369	259,1	252,36	138,72	185,21	43,32	85,06
3440,797	259,33	252,67	139,06	185,39	43,64	85,22
3442,225	259,58	253,01	139,43	185,57	43,95	85,39
3443,654	259,79	253,35	139,78	185,73	44,26	85,53
3445,082	259,96	253,69	140,1	185,89	44,58	85,64
3446,51	260,12	254,02	140,4	186,02	44,88	85,74
3447,938	260,3	254,36	140,73	186,16	45,19	85,86
3449,367	260,48	254,71	141,09	186,3	45,51	85,98
3450,795	260,65	255,05	141,47	186,45	45,82	86,09
3452,223	260,85	255,38	141,88	186,65	46,15	86,23
3453,652	261,06	255,69	142,29	186,85	46,47	86,41

This journal is © The Royal Society of Chemistry 20xx

Please do not adjust margins

Please do not adjust margins

3455,08	261,28	256,01	142,69	187,03	46,78	86,59
3456,508	261,5	256,35	143,1	187,2	47,09	86,75
3457,937	261,7	256,68	143,5	187,36	47,4	86,89
3459,365	261,9	257,01	143,91	187,55	47,71	87,05
3460,793	262,12	257,35	144,34	187,74	48,04	87,22
3462,221	262,35	257,68	144,78	187,9	48,36	87,38
3463,65	262,57	258,03	145,21	188,06	48,7	87,53
3465,078	262,75	258,39	145,59	188,2	49,05	87,67
3466,506	262,89	258,75	145,95	188,33	49,41	87,79
3467,935	263,08	259,13	146,37	188,49	49,77	87,94
3469,363	263,28	259,49	146,81	188,66	50,11	88,1
3470,791	263,45	259,83	147,21	188,83	50,45	88,24
3472,22	263,61	260,17	147,6	188,99	50,81	88,35
3473,648	263,77	260,53	148,01	189,15	51,15	88,44
3475,076	263,96	260,91	148,45	189,34	51,49	88,62
3476,505	264,14	261,27	148,82	189,47	51,85	88,79
3477,933	264,23	261,58	149,13	189,53	52,18	88,87
3479,361	264,36	261,92	149,51	189,65	52,51	88,95
3480,79	264,55	262,27	149,96	189,82	52,84	89,11
3482,218	264,74	262,62	150,39	189,96	53,16	89,28
3483,646	264,92	262,99	150,79	190,08	53,51	89,41
3485,074	265,03	263,36	151,16	190,17	53,84	89,49
3486,503	265,12	263,72	151,52	190,26	54,19	89,58
3487,931	265,28	264,07	151,93	190,38	54,55	89,69
3489,359	265,51	264,42	152,39	190,52	54,91	89,81
3490,788	265,76	264,8	152,84	190,68	55,27	89,99
3492,216	265,93	265,19	153,25	190,78	55,63	90,11
3493,644	266,05	265,57	153,63	190,83	55,98	90,17
3495,073	266,28	265,97	154,09	190,95	56,35	90,31
3496,501	266,51	266,35	154,55	191,07	56,7	90,47
3497,929	266,67	266,74	154,97	191,15	57,05	90,58
3499,357	266,85	267,16	155,4	191,24	57,43	90,69
3500,786	267,02	267,55	155,83	191,32	57,8	90,8
3502,214	267,2	267,91	156,26	191,42	58,16	90,93
3503,643	267,43	268,23	156,74	191,56	58,53	91,09
3505,071	267,63	268,53	157,24	191,69	58,89	91,2
3506,499	267,82	268,87	157,72	191,82	59,25	91,28
3507,927	268,02	269,24	158,17	191,97	59,62	91,39
3509,356	268,2	269,62	158,57	192,07	60	91,49
3510,784	268,35	269,99	158,94	192,14	60,39	91,57
3512,212	268,46	270,34	159,33	192,2	60,75	91,59
3513,641	268,58	270,68	159,76	192,29	61,12	91,61
3515,069	268,76	271,01	160,23	192,38	61,5	91,66
3516,497	268,93	271,38	160,7	192,45	61,88	91,74
3517,926	269,11	271,8	161,14	192,5	62,27	91,82
3519,354	269,35	272,21	161,58	192,55	62,66	91,95
3520,782	269,59	272,54	162,02	192,64	63,06	92,11

This journal is © The Royal Society of Chemistry 20xx

Please do not adjust margins

Please do not adjust margins

3522,21	269,83	272,86	162,45	192,75	63,47	92,23
3523,639	270,13	273,17	162,96	192,88	63,85	92,4
3525,067	270,47	273,48	163,49	193,03	64,24	92,63
3526,495	270,76	273,8	163,95	193,15	64,64	92,81
3527,924	271,02	274,13	164,35	193,25	65,02	92,93
3529,352	271,25	274,5	164,74	193,35	65,41	93,03
3530,78	271,46	274,9	165,13	193,44	65,79	93,13
3532,209	271,67	275,28	165,51	193,51	66,15	93,25
3533,637	271,84	275,6	165,83	193,55	66,52	93,32
3535,065	271,92	275,86	166,11	193,56	66,86	93,31
3536,494	272,01	276,12	166,46	193,61	67,21	93,29
3537,922	272,22	276,44	166,88	193,72	67,6	93,38
3539,35	272,49	276,78	167,28	193,83	67,99	93,59
3540,779	272,78	277,1	167,64	193,92	68,39	93,78
3542,207	273,1	277,41	168,02	194,02	68,79	93,95
3543,635	273,43	277,67	168,43	194,12	69,18	94,13
3545,063	273,81	277,96	168,89	194,24	69,57	94,31
3546,492	274,15	278,28	169,33	194,32	69,95	94,48
3547,92	274,37	278,62	169,68	194,35	70,32	94,59
3549,348	274,5	279	169,94	194,33	70,71	94,6
3550,777	274,64	279,4	170,19	194,31	71,08	94,6
3552,205	274,85	279,79	170,49	194,33	71,46	94,67
3553,633	275,04	280,14	170,81	194,35	71,84	94,75
3555,062	275,21	280,44	171,18	194,37	72,2	94,81
3556,49	275,36	280,72	171,58	194,42	72,56	94,85
3557,918	275,46	281,01	171,98	194,43	72,9	94,83
3559,346	275,72	281,32	172,41	194,46	73,26	94,93
3560,775	276,06	281,64	172,79	194,51	73,64	95,12
3562,203	276,22	281,95	173,08	194,52	74,01	95,14
3563,631	276,32	282,26	173,36	194,55	74,36	95,12
3565,06	276,51	282,53	173,7	194,59	74,73	95,16
3566,488	276,75	282,77	174,1	194,61	75,08	95,25
3567,917	276,99	283,01	174,53	194,65	75,43	95,35
3569,345	277,13	283,25	174,88	194,7	75,78	95,39
3570,773	277,25	283,5	175,16	194,73	76,13	95,38
3572,201	277,53	283,79	175,51	194,82	76,52	95,48
3573,63	277,87	284,07	175,89	194,92	76,91	95,67
3575,058	278,14	284,31	176,21	195	77,27	95,84
3576,486	278,34	284,46	176,53	195,06	77,62	95,93
3577,915	278,45	284,6	176,87	195,08	77,95	95,9
3579,343	278,63	284,84	177,24	195,11	78,29	95,93
3580,771	278,88	285,15	177,57	195,12	78,66	96,09
3582,199	278,94	285,46	177,76	195,03	79,01	96,1
3583,628	278,92	285,78	177,91	194,95	79,36	95,99
3585,056	279,02	286,05	178,13	194,98	79,71	96,01
3586,484	279,16	286,23	178,4	195,04	80,05	96,14
3587,913	279,29	286,36	178,74	195,1	80,37	96,24

This journal is © The Royal Society of Chemistry 20xx

Please do not adjust margins

3589,341	279,42	286,42	179,09	195,18	80,69	96,28
3590,769	279,54	286,54	179,33	195,3	81,04	96,29
3592,198	279,67	286,76	179,47	195,41	81,42	96,32
3593,626	279,83	287,05	179,61	195,54	81,81	96,38
3595,054	280,06	287,37	179,78	195,7	82,19	96,51
3596,483	280,23	287,58	179,88	195,8	82,56	96,59
3597,911	280,17	287,66	179,92	195,78	82,88	96,42
3599,339	280,01	287,84	180,03	195,74	83,18	96,11
3600,768	280,16	288,21	180,21	195,83	83,56	96,09
3602,196	280,28	288,52	180,26	195,84	83,95	96,16
3603,624	280,27	288,81	180,27	195,81	84,31	96,09
3605,052	280,38	289,06	180,45	195,85	84,65	96,11
3606,481	280,67	289,22	180,75	195,96	85,01	96,3
3607,909	280,93	289,57	181,07	196,03	85,34	96,46
3609,337	281,21	289,86	181,44	196,1	85,65	96,61
3610,766	281,5	290,09	181,8	196,16	85,98	96,77
3612,194	281,58	290,29	182,02	196,14	86,28	96,75
3613,622	281,62	290,55	182,18	196,08	86,61	96,66
3615,051	281,79	290,78	182,38	196,06	86,96	96,7
3616,479	281,88	290,93	182,56	196,01	87,28	96,73
3617,907	281,74	290,99	182,7	195,94	87,55	96,54
3619,335	281,59	291,01	182,87	195,94	87,82	96,33
3620,764	281,5	291,12	183,02	195,91	88,09	96,19
3622,192	281,84	291,33	183,29	196,01	88,44	96,37
3623,62	282,38	291,4	183,6	196,15	88,81	96,7
3625,049	282,61	291,39	183,82	196,23	89,13	96,78
3626,477	282,75	291,41	184,08	196,29	89,44	96,71
3627,905	283,22	291,45	184,53	196,41	89,76	96,89
3629,334	283,75	291,54	184,96	196,52	90,1	97,2
3630,762	283,95	291,68	185,18	196,52	90,4	97,27
3632,19	283,95	291,86	185,29	196,43	90,68	97,13
3633,619	284,04	292,07	185,48	196,35	90,98	97,03
3635,047	284,26	292,3	185,76	196,33	91,28	97,07
3636,475	284,56	292,55	186,05	196,35	91,59	97,19
3637,904	284,73	292,77	186,25	196,26	91,88	97,23
3639,332	284,67	292,94	186,38	196,04	92,1	97,03
3640,76	284,68	293,17	186,59	195,91	92,34	96,86
3642,188	285,18	293,44	186,95	196,03	92,69	97,16
3643,617	285,54	293,54	187,25	196,05	92,99	97,33
3645,045	285,62	293,67	187,5	195,96	93,21	97,17
3646,473	285,89	293,85	187,81	196,13	93,49	97,09
3647,902	286,48	293,91	188,08	196,53	93,86	97,47
3649,33	286,69	294,1	188,1	196,69	94,19	97,56
3650,758	286,8	294,33	188,16	196,8	94,5	97,46
3652,187	286,95	294,54	188,25	196,92	94,8	97,36
3653,615	287,07	294,76	188,3	196,98	95,08	97,25
3655,043	287,23	295,04	188,39	197,04	95,35	97,16

Please do not adjust margins

3656,471	287,56	295,4	188,55	197,17	95,63	97,24
3657,9	287,9	295,69	188,64	197,29	95,92	97,44
3659,328	287,77	295,76	188,58	197,26	96,12	97,28
3660,757	287,83	295,86	188,68	197,39	96,33	97,21
3662,185	288,28	296,02	188,95	197,6	96,57	97,49
3663,613	288,71	296,25	189,15	197,73	96,81	97,7
3665,042	288,98	296,59	189,25	197,75	97,01	97,72
3666,47	289,28	296,7	189,42	197,75	97,2	97,75
3667,898	289,58	296,8	189,63	197,77	97,37	97,82
3669,326	289,77	296,89	189,81	197,8	97,54	97,82
3670,755	289,88	296,98	189,95	197,78	97,68	97,73
3672,183	289,95	297,05	190,07	197,74	97,81	97,59
3673,611	290,01	297,17	190,13	197,76	97,94	97,46
3675,04	290,21	297,33	190,18	197,83	98,1	97,48
3676,468	290,57	297,5	190,28	197,93	98,27	97,63
3677,896	291	297,63	190,47	198,01	98,4	97,78
3679,324	291,67	297,63	190,91	198,22	98,52	98,09
3680,753	292,11	297,46	191,4	198,34	98,56	98,25
3682,181	292,32	297,41	191,79	198,35	98,55	98,2
3683,609	292,69	297,49	192,1	198,37	98,62	98,32
3685,038	293,09	297,61	192,23	198,41	98,75	98,55
3686,466	293,14	297,72	192,13	198,37	98,81	98,52
3687,894	293,08	297,82	192,1	198,32	98,84	98,34
3689,323	293,2	297,9	192,29	198,28	98,86	98,3
3690,751	293,39	297,97	192,58	198,19	98,86	98,37
3692,179	293,51	298,04	192,84	198,06	98,84	98,39
3693,608	293,56	298,14	193,04	197,9	98,81	98,34
3695,036	293,55	298,24	193,15	197,72	98,8	98,29
3696,464	293,5	298,36	193,23	197,57	98,82	98,23
3697,893	293,78	298,48	193,5	197,65	98,89	98,42
3699,321	294,31	298,46	193,84	197,85	98,98	98,85
3700,749	294,32	298,28	194,01	197,81	98,93	98,87
3702,177	294,29	298,2	194,26	197,8	98,85	98,79
3703,606	294,72	298,16	194,66	198,04	98,89	99,11
3705,034	294,95	298,1	194,83	198,09	98,93	99,38
3706,462	294,64	298,31	194,75	197,87	98,89	99,05
3707,891	294,7	298,51	194,72	197,88	99,02	99,06
3709,319	294,62	298,54	194,58	197,83	99,11	99,08
3710,747	294,46	298,53	194,49	197,8	99,14	99,03
3712,176	294,46	298,45	194,53	197,9	99,16	99,1
3713,604	294,25	298,31	194,52	197,89	99,11	99,02
3715,032	293,85	298,26	194,46	197,81	99,03	98,73
3716,46	293,89	298,29	194,39	197,87	99,12	98,75
3717,889	294,11	298,25	194,32	197,9	99,25	99
3719,317	294,15	298,23	194,33	197,88	99,28	99,03
3720,745	294,08	298,12	194,33	197,95	99,27	98,91
3722,174	294,04	297,97	194,33	198,11	99,28	98,92

 This journal is © The Royal Society of Chemistry 20xx

Please do not adjust margins

3723,602	294,24	297,93	194,43	198,35	99,36	99,12
3725,031	294,45	297,87	194,42	198,58	99,49	99,31
3726,459	294,33	297,84	194,2	198,63	99,57	99,22
3727,887	294,09	297,87	193,96	198,61	99,61	98,98
3729,315	294,03	297,88	193,87	198,7	99,66	98,91
3730,744	294,04	297,93	193,79	198,84	99,73	98,93
3732,172	294,02	297,98	193,7	198,91	99,78	98,91
3733,6	293,92	297,93	193,63	198,95	99,78	98,8
3735,029	293,72	297,83	193,58	198,98	99,73	98,6
3736,457	293,68	297,73	193,63	199,11	99,69	98,53
3737,885	293,84	297,59	193,69	199,27	99,7	98,64
3739,313	293,86	297,46	193,63	199,35	99,7	98,67
3740,742	293,8	297,42	193,54	199,46	99,71	98,63
3742,17	293,82	297,41	193,51	199,65	99,73	98,63
3743,598	293,79	297,4	193,56	199,79	99,69	98,59
3745,027	293,81	297,43	193,73	199,91	99,65	98,55
3746,455	293,88	297,48	193,91	200	99,62	98,55
3747,883	293,83	297,51	193,98	200	99,57	98,48
3749,312	293,71	297,59	194,01	199,95	99,51	98,33
3750,74	293,69	297,71	194,08	199,96	99,48	98,26
3752,168	293,66	297,81	194,1	199,92	99,47	98,24
3753,597	293,6	297,91	194,17	199,8	99,43	98,17
3755,025	293,69	297,98	194,36	199,7	99,38	98,17
3756,453	293,82	297,98	194,58	199,61	99,32	98,27
3757,882	293,84	297,99	194,79	199,47	99,23	98,3
3759,31	293,97	298,04	195,06	199,34	99,17	98,36
3760,738	294,19	298,11	195,29	199,28	99,18	98,53
3762,167	294,2	298,2	195,35	199,18	99,19	98,55
3763,595	294,18	298,34	195,31	199,08	99,24	98,54
3765,023	294,19	298,42	195,25	198,94	99,31	98,59
3766,451	293,91	298,46	195,21	198,66	99,22	98,35
3767,88	293,77	298,57	195,28	198,46	99,14	98,14
3769,308	293,67	298,66	195,29	198,22	99,1	98,04
3770,736	293,26	298,75	195,16	197,78	99,01	97,7
3772,165	292,88	298,89	195,01	197,38	99	97,32
3773,593	293,09	298,94	194,89	197,33	99,16	97,51
3775,021	293,11	298,76	194,81	197,27	99,19	97,62
3776,449	293,14	298,63	194,88	197,31	99,15	97,61
3777,878	293,44	298,56	195,03	197,48	99,2	97,83
3779,306	293,97	298,57	195,16	197,59	99,33	98,27
3780,734	294,37	298,66	195,2	197,56	99,45	98,55
3782,163	294,42	298,73	195,09	197,41	99,48	98,51
3783,591	294,3	298,87	194,95	197,18	99,48	98,32
3785,019	294,12	299,03	194,84	196,91	99,46	98,08
3786,448	293,92	299,21	194,72	196,67	99,42	97,83
3787,876	294,03	299,4	194,6	196,63	99,49	97,84
3789,304	294,01	299,46	194,4	196,52	99,55	97,81

This journal is © The Royal Society of Chemistry 20xx

Please do not adjust margins

3790,733	293,63	299,39	194,17	196,29	99,45	97,47
3792,161	293,72	299,3	194,3	196,37	99,4	97,42
3793,589	294,74	299,17	194,75	196,81	99,55	98,17
3795,018	295,42	299,07	195,06	197,04	99,65	98,7
3796,446	295,72	299,11	195,27	197,17	99,66	98,82
3797,874	296,26	299,21	195,55	197,42	99,71	99,06
3799,302	296,82	299,23	195,76	197,67	99,79	99,41
3800,731	297,01	299,27	195,87	197,77	99,79	99,45
3802,159	297,09	299,38	195,96	197,81	99,76	99,33
3803,587	297,14	299,52	195,98	197,8	99,77	99,23
3805,016	297,02	299,64	195,88	197,72	99,77	99,06
3806,444	296,96	299,76	195,85	197,73	99,76	98,93
3807,872	297,16	299,85	195,96	197,86	99,79	98,99
3809,301	297,38	299,81	196,04	197,99	99,79	99,08
3810,729	297,67	299,66	196,15	198,2	99,82	99,23
3812,157	298,21	299,48	196,42	198,54	99,86	99,61
3813,585	298,63	299,37	196,66	198,82	99,87	99,91
3815,014	298,8	299,4	196,75	198,95	99,89	99,97
3816,442	298,92	299,52	196,77	199,02	99,94	100
3817,871	298,99	299,66	196,78	199,03	99,95	100
3819,299	298,89	299,81	196,74	198,97	99,94	99,86
3820,727	298,64	299,91	196,61	198,85	99,9	99,61
3822,156	298,22	299,95	196,47	198,63	99,81	99,24
3823,584	297,8	300	196,45	198,45	99,71	98,83
3825,012	297,78	299,96	196,41	198,75	99,76	98,86
3826,44	297,75	299,81	196,16	199	99,85	98,98
3827,869	297,74	299,7	196,08	199,11	99,89	98,98
3829,297	298,1	299,57	196,24	199,41	99,95	99,22
3830,725	298,62	299,39	196,45	199,76	100	99,64
3832,154	298,71	299,24	196,59	199,79	99,92	99,66
3833,582	298,66	299,24	196,68	199,67	99,85	99,52
3835,01	298,57	299,33	196,61	199,47	99,83	99,36
3836,438	298,34	299,47	196,28	199,27	99,86	99,18
3837,867	297,88	299,48	195,82	199,03	99,86	98,88
3839,295	297,28	299,43	195,45	198,74	99,78	98,42
3840,723	296,89	299,32	195,29	198,68	99,68	98,05
3842,152	296,93	299,11	195,23	198,91	99,7	98,13
3843,58	296,93	298,94	195,14	198,99	99,71	98,22
3845,008	297,1	298,82	195,14	199,12	99,76	98,38
3846,437	297,22	298,67	195,18	199,24	99,81	98,52
3847,865	297,3	298,51	195,22	199,33	99,8	98,61
3849,293	297,67	298,38	195,41	199,52	99,84	98,92
3850,722	297,91	298,33	195,54	199,54	99,87	99,12
3852,15	297,53	298,39	195,42	199,33	99,78	98,78
3853,578	297,24	298,4	195,2	199,2	99,75	98,53
3855,007	296,89	298,38	194,94	199	99,71	98,26
3856,435	296,41	298,45	194,73	198,74	99,63	97,87

 This journal is © The Royal Society of Chemistry 20xx

Please do not adjust margins

Please do not adjust margins

3857,863	296,23	298,52	194,65	198,7	99,63	97,72
3859,292	296,16	298,46	194,59	198,71	99,63	97,7
3860,72	296,06	298,33	194,59	198,68	99,58	97,67
3862,148	296,12	298,21	194,75	198,72	99,53	97,71
3863,576	296,39	298,11	194,92	198,86	99,55	97,9
3865,005	296,67	298,02	194,97	198,98	99,58	98,13
3866,433	296,76	297,93	194,91	199,04	99,62	98,24
3867,861	296,66	297,9	194,78	198,95	99,63	98,2
3869,29	296,36	298,01	194,65	198,72	99,59	97,98
3870,718	296,02	298,2	194,55	198,48	99,53	97,67
3872,146	295,85	298,36	194,5	198,32	99,52	97,5
3873,574	295,65	298,46	194,42	198,14	99,51	97,35
3875,003	295,33	298,49	194,31	198,04	99,44	97,09
3876,431	295,2	298,41	194,23	198,17	99,44	96,98
3877,859	295,22	298,3	194,2	198,36	99,5	97,06
3879,288	295,23	298,22	194,18	198,51	99,54	97,13
3880,716	295,36	298,18	194,15	198,73	99,65	97,26
3882,145	295,66	298,15	194,04	199,01	99,81	97,56
3883,573	295,8	298,18	193,98	198,87	99,86	97,65
3885,001	295,91	298,27	194,17	198,68	99,82	97,61
3886,429	295,84	298,35	194,25	198,52	99,76	97,46
3887,858	295,58	298,48	194,15	198,26	99,72	97,16
3889,286	295,34	298,62	193,96	198	99,71	96,93
3890,714	295,11	298,68	193,73	197,83	99,74	96,79
3892,143	294,94	298,68	193,47	197,78	99,78	96,68
3893,571	294,86	298,57	193,29	197,84	99,82	96,63
3894,999	294,83	298,38	193,35	197,91	99,8	96,59
3896,427	295,1	298,33	193,66	198,08	99,77	96,68
3897,856	295,59	298,3	193,96	198,35	99,82	97,01
3899,284	295,82	298,29	194	198,39	99,91	97,24
3900,712	295,6	298,38	193,96	198,1	99,88	97,02
3902,141	295,61	298,42	194,07	198,06	99,88	96,93
3903,569	295,66	298,38	194,13	198,12	99,91	97
3904,997	295,44	298,34	194	197,99	99,85	96,87
3906,426	295,25	298,33	193,91	197,85	99,79	96,67
3907,854	295,25	298,34	193,91	197,8	99,78	96,59
3909,282	295,39	298,32	193,94	197,85	99,8	96,68
3910,711	295,47	298,26	193,99	197,88	99,78	96,74
3912,139	295,49	298,24	194,17	197,84	99,7	96,68
3913,567	295,71	298,26	194,45	197,91	99,68	96,81
3914,996	296,07	298,2	194,62	198,04	99,71	97,11
3916,424	296,02	298,13	194,6	197,91	99,63	97,08
3917,852	295,89	298,15	194,61	197,74	99,54	96,9
3919,281	295,94	298,21	194,67	197,68	99,53	96,88
3920,709	295,99	298,22	194,63	197,65	99,54	96,92
3922,137	295,92	298,22	194,57	197,56	99,49	96,84
3923,565	295,85	298,21	194,57	197,43	99,42	96,69

This journal is © The Royal Society of Chemistry 20xx

Please do not adjust margins

Please do not adjust margins

3924,994	295,78	298,18	194,55	197,29	99,36	96,54
3926,422	295,86	298,12	194,63	197,29	99,32	96,52
3927,85	296,12	298,04	194,78	197,44	99,33	96,64
3929,279	296,25	297,95	194,82	197,56	99,34	96,74
3930,707	296,23	297,9	194,71	197,63	99,34	96,72
3932,135	296,28	297,85	194,63	197,72	99,36	96,72
3933,563	296,41	297,83	194,6	197,76	99,4	96,81
3934,992	296,4	297,88	194,55	197,65	99,4	96,78
3936,42	296,23	297,95	194,44	197,46	99,35	96,59
3937,848	296,09	298	194,34	197,27	99,31	96,39
3939,277	296,08	298,07	194,35	197,13	99,27	96,3
3940,705	296,1	298,14	194,37	197,05	99,24	96,24
3942,133	296,01	298,18	194,35	196,96	99,21	96,13
3943,562	295,94	298,2	194,35	196,9	99,19	96,03
3944,99	296,02	298,15	194,4	196,9	99,17	96,08
3946,418	296,17	298,08	194,5	196,93	99,17	96,2
3947,847	296,33	298,04	194,64	196,94	99,17	96,27
3949,275	296,51	298,01	194,78	196,92	99,17	96,31
3950,703	296,59	298,03	194,88	196,88	99,18	96,29
3952,132	296,66	298,09	195	196,86	99,19	96,3
3953,56	296,8	298,12	195,05	196,87	99,22	96,39
3954,988	296,77	298,11	194,98	196,84	99,24	96,34
3956,417	296,67	298,1	194,95	196,82	99,24	96,2
3957,845	296,72	298,08	195	196,86	99,26	96,19
3959,273	296,77	298,05	195,03	196,9	99,28	96,23
3960,701	296,81	298,06	195,02	196,93	99,28	96,25
3962,13	296,9	298,07	195,01	196,96	99,3	96,27
3963,558	296,92	298,06	195,01	196,91	99,29	96,22
3964,986	296,96	298,07	195,1	196,86	99,28	96,2
3966,415	297,05	298,09	195,23	196,87	99,31	96,26
3967,843	297,08	298,08	195,29	196,82	99,32	96,28
3969,271	297,07	298,09	195,31	196,73	99,34	96,26
3970,699	297,16	298,12	195,34	196,72	99,38	96,29
3972,128	297,28	298,13	195,34	196,75	99,4	96,36
3973,556	297,34	298,14	195,38	196,75	99,41	96,37
3974,985	297,4	298,15	195,42	196,75	99,41	96,37
3976,413	297,46	298,16	195,45	196,75	99,41	96,38
3977,841	297,52	298,17	195,49	196,76	99,41	96,38
3979,27	297,59	298,18	195,53	196,77	99,41	96,38
3980,698	297,65	298,19	195,57	196,78	99,41	96,39
3982,126	297,72	298,21	195,61	196,8	99,41	96,39
3983,554	297,79	298,22	195,66	196,82	99,4	96,39
3984,983	297,86	298,23	195,7	196,84	99,4	96,38
3986,411	297,93	298,24	195,74	196,87	99,39	96,38
3987,839	298	298,26	195,78	196,9	99,38	96,38
3989,268	298,07	298,27	195,83	196,93	99,37	96,37
3990,696	298,14	298,28	195,87	196,96	99,36	96,36

This journal is © The Royal Society of Chemistry 20xx

Please do not adjust margins

Please do not adjust margins

3992,124	298,21	298,3	195,92	197	99,35	96,35
3993,552	298,29	298,31	195,96	197,04	99,33	96,35
3994,981	298,36	298,32	196,01	197,09	99,32	96,33
3996,409	298,44	298,34	196,06	197,13	99,3	96,32
3997,837	298,52	298,35	196,1	197,18	99,28	96,31
3999,266	298,59	298,37	196,15	197,23	99,26	96,29
4000,694	298,67	298,38	196,2	197,29	99,24	96,28

Table S3 Raw XPS measurements corresponding to Fig. 2.a.

KE_MgKa	BE_MgKa	CPS_MgKa	C 1s_1_MgKa	C 1s_2_MgKa	C 1s_3_MgKa	C 1s_4_MgKa	Background_MgKa	Envelope_MgKa
955869	297731	2961,67	2961,67	2961,67	2961,67	2961,67	2961,67	2961,67
955899	297701	2660	2660	2660	2660	2660	2660	2660
955929	297671	2838,33	2838,33	2838,33	2838,33	2838,33	2838,33	2838,33
955959	297641	2813,33	2813,33	2813,33	2813,33	2813,33	2813,33	2813,33
955989	297611	2821,67	2821,67	2821,67	2821,67	2821,67	2821,67	2821,67
956019	297581	2875	2875	2875	2875	2875	2875	2875
956049	297551	2936,67	2936,67	2936,67	2936,67	2936,67	2936,67	2936,67
956079	297521	2858,33	2858,33	2858,33	2858,33	2858,33	2858,33	2858,33
956109	297491	2828,33	2828,33	2828,33	2828,33	2828,33	2828,33	2828,33
956139	297461	2830	2830	2830	2830	2830	2830	2830
956169	297431	2906,67	2906,67	2906,67	2906,67	2906,67	2906,67	2906,67
956199	297401	2871,67	2871,67	2871,67	2871,67	2871,67	2871,67	2871,67
956229	297371	2851,67	2851,67	2851,67	2851,67	2851,67	2851,67	2851,67
956259	297341	2820	2820	2820	2820	2820	2820	2820
956289	297311	2833,33	2833,33	2833,33	2833,33	2833,33	2833,33	2833,33
956319	297281	2826,67	2826,67	2826,67	2826,67	2826,67	2826,67	2826,67
956349	297251	2823,33	2823,33	2823,33	2823,33	2823,33	2823,33	2823,33
956379	297221	2853,33	2853,33	2853,33	2853,33	2853,33	2853,33	2853,33
956409	297191	2703,33	2703,33	2703,33	2703,33	2703,33	2703,33	2703,33
956439	297161	2875	2875	2875	2875	2875	2875	2875
956469	297131	2795	2795	2795	2795	2795	2795	2795
956499	297101	2801,67	2801,67	2801,67	2801,67	2801,67	2801,67	2801,67
956529	297071	2855	2855	2855	2855	2855	2855	2855
956559	297041	2868,33	2868,33	2868,33	2868,33	2868,33	2868,33	2868,33
956589	297011	2811,67	2811,67	2811,67	2811,67	2811,67	2811,67	2811,67
956619	296981	2876,67	2876,67	2876,67	2876,67	2876,67	2876,67	2876,67
956649	296951	2811,67	2811,67	2811,67	2811,67	2811,67	2811,67	2811,67
956679	296921	2835	2835	2835	2835	2835	2835	2835
956709	296891	2885	2885	2885	2885	2885	2885	2885
956739	296861	2785	2785	2785	2785	2785	2785	2785
956769	296831	2826,67	2826,67	2826,67	2826,67	2826,67	2826,67	2826,67
956799	296801	2758,33	2758,33	2758,33	2758,33	2758,33	2758,33	2758,33
956829	296771	2851,67	2851,67	2851,67	2851,67	2851,67	2851,67	2851,67
956859	296741	2763,33	2763,33	2763,33	2763,33	2763,33	2763,33	2763,33
956889	296711	2795	2795	2795	2795	2795	2795	2795
956919	296681	2806,67	2806,67	2806,67	2806,67	2806,67	2806,67	2806,67

Please do not adjust margins

956949	296651	2890	2890	2890	2890	2890	2890	2890
956979	296621	2590	2590	2590	2590	2590	2590	2590
957009	296591	2668,33	2668,33	2668,33	2668,33	2668,33	2668,33	2668,33
957039	296561	2736,67	2736,67	2736,67	2736,67	2736,67	2736,67	2736,67
957069	296531	2778,33	2778,33	2778,33	2778,33	2778,33	2778,33	2778,33
957099	296501	2710	2710	2710	2710	2710	2710	2710
957129	296471	2771,67	2771,67	2771,67	2771,67	2771,67	2771,67	2771,67
957159	296441	2840	2840	2840	2840	2840	2840	2840
957189	296411	2841,67	2841,67	2841,67	2841,67	2841,67	2841,67	2841,67
957219	296381	2686,67	2686,67	2686,67	2686,67	2686,67	2686,67	2686,67
957249	296351	2846,67	2846,67	2846,67	2846,67	2846,67	2846,67	2846,67
957279	296321	2745	2745	2745	2745	2745	2745	2745
957309	296291	2791,67	2791,67	2791,67	2791,67	2791,67	2791,67	2791,67
957339	296261	2820	2820	2820	2820	2820	2820	2820
957369	296231	2735	2735	2735	2735	2735	2735	2735
957399	296201	2716,67	2716,67	2716,67	2716,67	2716,67	2716,67	2716,67
957429	296171	2858,33	2858,33	2858,33	2858,33	2858,33	2858,33	2858,33
957459	296141	2690	2690	2690	2690	2690	2690	2690
957489	296111	2791,67	2791,67	2791,67	2791,67	2791,67	2791,67	2791,67
957519	296081	2703,33	2703,33	2703,33	2703,33	2703,33	2703,33	2703,33
957549	296051	2666,67	2666,67	2666,67	2666,67	2666,67	2666,67	2666,67
957579	296021	2693,33	2693,33	2693,33	2693,33	2693,33	2693,33	2693,33
957609	295991	2736,67	2736,67	2736,67	2736,67	2736,67	2736,67	2736,67
957639	295961	2690	2690	2690	2690	2690	2690	2690
957669	295931	2716,67	2716,67	2716,67	2716,67	2716,67	2716,67	2716,67
957699	295901	2875	2875	2875	2875	2875	2875	2875
957729	295871	2728,33	2728,33	2728,33	2728,33	2728,33	2728,33	2728,33
957759	295841	2765	2765	2765	2765	2765	2765	2765
957789	295811	2683,33	2683,33	2683,33	2683,33	2683,33	2683,33	2683,33
957819	295781	2760	2760	2760	2760	2760	2760	2760
957849	295751	2861,67	2861,67	2861,67	2861,67	2861,67	2861,67	2861,67
957879	295721	2846,67	2846,67	2846,67	2846,67	2846,67	2846,67	2846,67
957909	295691	2773,33	2773,33	2773,33	2773,33	2773,33	2773,33	2773,33
957939	295661	2800	2800	2800	2800	2800	2800	2800
957969	295631	2693,33	2693,33	2693,33	2693,33	2693,33	2693,33	2693,33
957999	295601	2790	2790	2790	2790	2790	2790	2790
958029	295571	2700	2700	2700	2700	2700	2700	2700
958059	295541	2690	2690	2690	2690	2690	2690	2690
958089	295511	2810	2810	2810	2810	2810	2810	2810
958119	295481	2716,67	2716,67	2716,67	2716,67	2716,67	2716,67	2716,67
958149	295451	2668,33	2668,33	2668,33	2668,33	2668,33	2668,33	2668,33
958179	295421	2663,33	2663,33	2663,33	2663,33	2663,33	2663,33	2663,33
958209	295391	2715	2715	2715	2715	2715	2715	2715
958239	295361	2710	2710	2710	2710	2710	2710	2710
958269	295331	2710	2710	2710	2710	2710	2710	2710
958299	295301	2630	2630	2630	2630	2630	2630	2630
958329	295271	2750	2750	2750	2750	2750	2750	2750

This journal is © The Royal Society of Chemistry 20xx

Please do not adjust margins

958359	295241	2643,33	2643,33	2643,33	2643,33	2643,33	2643,33	2643,33
958389	295211	2836,67	2836,67	2836,67	2836,67	2836,67	2836,67	2836,67
958419	295181	2816,67	2816,67	2816,67	2816,67	2816,67	2816,67	2816,67
958449	295151	2805	2805	2805	2805	2805	2805	2805
958479	295121	2641,67	2641,67	2641,67	2641,67	2641,67	2641,67	2641,67
958509	295091	2683,33	2683,33	2683,33	2683,33	2683,33	2683,33	2683,33
958539	295061	2731,67	2731,67	2731,67	2731,67	2731,67	2731,67	2731,67
958569	295031	2761,67	2761,67	2761,67	2761,67	2761,67	2761,67	2761,67
958599	295001	2781,67	2781,67	2781,67	2781,67	2781,67	2781,67	2781,67
958629	294971	2693,33	2693,33	2693,33	2693,33	2693,33	2693,33	2693,33
958659	294941	2718,33	2718,33	2718,33	2718,33	2718,33	2718,33	2718,33
958689	294911	2785	2785	2785	2785	2785	2785	2785
958719	294881	2753,33	2753,33	2753,33	2753,33	2753,33	2753,33	2753,33
958749	294851	2783,33	2783,33	2783,33	2783,33	2783,33	2783,33	2783,33
958779	294821	2725	2725	2725	2725	2725	2725	2725
958809	294791	2580	2580	2580	2580	2580	2580	2580
958839	294761	2706,67	2706,67	2706,67	2706,67	2706,67	2706,67	2706,67
958869	294731	2626,67	2626,67	2626,67	2626,67	2626,67	2626,67	2626,67
958899	294701	2716,67	2716,67	2716,67	2716,67	2716,67	2716,67	2716,67
958929	294671	2741,67	2741,67	2741,67	2741,67	2741,67	2741,67	2741,67
958959	294641	2800	2800	2800	2800	2800	2800	2800
958989	294611	2711,67	2711,67	2711,67	2711,67	2711,67	2711,67	2711,67
959019	294581	2720	2720	2720	2720	2720	2720	2720
959049	294551	2641,67	2641,67	2641,67	2641,67	2641,67	2641,67	2641,67
959079	294521	2680	2680	2680	2680	2680	2680	2680
959109	294491	2655	2655	2655	2655	2655	2655	2655
959139	294461	2736,67	2736,67	2736,67	2736,67	2736,67	2736,67	2736,67
959169	294431	2720	2720	2720	2720	2720	2720	2720
959199	294401	2698,33	2698,33	2698,33	2698,33	2698,33	2698,33	2698,33
959229	294371	2778,33	2778,33	2778,33	2778,33	2778,33	2778,33	2778,33
959259	294341	2676,67	2676,67	2676,67	2676,67	2676,67	2676,67	2676,67
959289	294311	2698,33	2698,33	2698,33	2698,33	2698,33	2698,33	2698,33
959319	294281	2698,33	2698,33	2698,33	2698,33	2698,33	2698,33	2698,33
959349	294251	2641,67	2641,67	2641,67	2641,67	2641,67	2641,67	2641,67
959379	294221	2646,67	2646,67	2646,67	2646,67	2646,67	2646,67	2646,67
959409	294191	2781,67	2781,67	2781,67	2781,67	2781,67	2781,67	2781,67
959439	294161	2741,67	2741,67	2741,67	2741,67	2741,67	2741,67	2741,67
959469	294131	2781,67	2781,67	2781,67	2781,67	2781,67	2781,67	2781,67
959499	294101	2765	2765	2765	2765	2765	2765	2765
959529	294071	2815	2815	2815	2815	2815	2815	2815
959559	294041	2691,67	2691,67	2691,67	2691,67	2691,67	2691,67	2691,67
959589	294011	2645	2645	2645	2645	2645	2645	2645
959619	293981	2701,67	2701,67	2701,67	2701,67	2701,67	2701,67	2701,67
959649	293951	2715	2715	2715	2715	2715	2715	2715
959679	293921	2623,33	2623,33	2623,33	2623,33	2623,33	2623,33	2623,33
959709	293891	2551,67	2551,67	2551,67	2551,67	2551,67	2551,67	2551,67
959739	293861	2741,67	2741,67	2741,67	2741,67	2741,67	2741,67	2741,67

This journal is © The Royal Society of Chemistry 20xx

Please do not adjust margins

Please do not adjust margins

959769	293831	2625	2625	2625	2625	2625	2625	2625
959799	293801	2570	2570	2570	2570	2570	2570	2570
959829	293771	2585	2585	2585	2585	2585	2585	2585
959859	293741	2716,67	2716,67	2716,67	2716,67	2716,67	2716,67	2716,67
959889	293711	2735	2735	2735	2735	2735	2735	2735
959919	293681	2766,67	2766,67	2766,67	2766,67	2766,67	2766,67	2766,67
959949	293651	2651,67	2651,67	2651,67	2651,67	2651,67	2651,67	2651,67
959979	293621	2671,67	2671,67	2671,67	2671,67	2671,67	2671,67	2671,67
960009	293591	2701,67	2701,67	2701,67	2701,67	2701,67	2701,67	2701,67
960039	293561	2723,33	2723,33	2723,33	2723,33	2723,33	2723,33	2723,33
960069	293531	2778,33	2778,33	2778,33	2778,33	2778,33	2778,33	2778,33
960099	293501	2673,33	2673,33	2673,33	2673,33	2673,33	2673,33	2673,33
960129	293471	2671,67	2671,67	2671,67	2671,67	2671,67	2671,67	2671,67
960159	293441	2673,33	2673,33	2673,33	2673,33	2673,33	2673,33	2673,33
960189	293411	2741,67	2741,67	2741,67	2741,67	2741,67	2741,67	2741,67
960219	293381	2746,67	2746,67	2746,67	2746,67	2746,67	2746,67	2746,67
960249	293351	2861,67	2861,67	2861,67	2861,67	2861,67	2861,67	2861,67
960279	293321	2713,33	2713,33	2713,33	2713,33	2713,34	2713,33	2713,34
960309	293291	2655	2655	2655	2655	2655	2655	2655
960339	293261	2668,33	2668,33	2668,33	2668,33	2668,34	2668,33	2668,34
960369	293231	2655	2655	2655	2655	2655	2655	2655
960399	293201	2665	2665	2665	2665	2665	2665	2665
960429	293171	2558,33	2558,33	2558,33	2558,33	2558,34	2558,33	2558,34
960459	293141	2655	2655	2655	2655	2655,01	2655	2655,01
960489	293111	2593,33	2593,33	2593,33	2593,33	2593,34	2593,33	2593,34
960519	293081	2591,67	2642,84	2642,84	2642,84	2642,84	2642,84	2642,84
960549	293051	2715	2642,95	2642,95	2642,95	2642,95	2642,95	2642,95
960579	293021	2625	2643,05	2643,05	2643,05	2643,06	2643,05	2643,06
960609	292991	2691,67	2643,16	2643,16	2643,16	2643,17	2643,16	2643,17
960639	292961	2741,67	2643,27	2643,27	2643,27	2643,28	2643,27	2643,28
960669	292931	2580	2643,38	2643,38	2643,38	2643,39	2643,38	2643,39
960699	292901	2653,33	2643,48	2643,48	2643,48	2643,5	2643,48	2643,5
960729	292871	2713,33	2643,59	2643,59	2643,59	2643,61	2643,59	2643,61
960759	292841	2721,67	2643,69	2643,69	2643,69	2643,71	2643,69	2643,71
960789	292811	2855	2643,8	2643,8	2643,8	2643,82	2643,8	2643,82
960819	292781	2611,67	2643,9	2643,9	2643,9	2643,92	2643,9	2643,92
960849	292751	2693,33	2644	2644	2644	2644,03	2644	2644,03
960879	292721	2740	2644,1	2644,1	2644,1	2644,13	2644,1	2644,13
960909	292691	2650	2644,2	2644,2	2644,2	2644,24	2644,2	2644,24
960939	292661	2623,33	2644,3	2644,3	2644,3	2644,34	2644,3	2644,34
960969	292631	2706,67	2644,39	2644,39	2644,39	2644,44	2644,39	2644,44
960999	292601	2801,67	2644,48	2644,49	2644,49	2644,54	2644,48	2644,54
961029	292571	2695	2644,58	2644,58	2644,58	2644,64	2644,58	2644,64
961059	292541	2691,67	2644,67	2644,67	2644,67	2644,74	2644,67	2644,74
961089	292511	2661,67	2644,75	2644,75	2644,75	2644,83	2644,75	2644,83
961119	292481	2585	2644,84	2644,84	2644,84	2644,93	2644,84	2644,93
961149	292451	2825	2644,92	2644,92	2644,92	2645,02	2644,92	2645,02

 This journal is © The Royal Society of Chemistry 20xx

Please do not adjust margins

Please do not adjust margins

961179	292421	2741,67	2645	2645	2645	2645,11	2645	2645,11
961209	292391	2816,67	2645,08	2645,08	2645,08	2645,2	2645,08	2645,2
961239	292361	2778,33	2645,16	2645,16	2645,16	2645,29	2645,16	2645,29
961269	292331	2788,33	2645,23	2645,23	2645,23	2645,38	2645,23	2645,38
961299	292301	2776,67	2645,3	2645,3	2645,3	2645,47	2645,3	2645,47
961329	292271	2651,67	2645,36	2645,36	2645,36	2645,55	2645,36	2645,55
961359	292241	2738,33	2645,43	2645,43	2645,43	2645,64	2645,43	2645,64
961389	292211	2648,33	2645,49	2645,49	2645,49	2645,72	2645,49	2645,72
961419	292181	2706,67	2645,54	2645,54	2645,54	2645,81	2645,54	2645,81
961449	292151	2710	2645,59	2645,59	2645,59	2645,89	2645,59	2645,89
961479	292121	2648,33	2645,64	2645,64	2645,64	2645,97	2645,64	2645,97
961509	292091	2571,67	2645,69	2645,69	2645,69	2646,05	2645,69	2646,05
961539	292061	2696,67	2645,73	2645,73	2645,73	2646,13	2645,73	2646,13
961569	292031	2740	2645,77	2645,77	2645,77	2646,21	2645,77	2646,21
961599	292001	2663,33	2645,8	2645,8	2645,8	2646,29	2645,8	2646,3
961629	291971	2930	2645,83	2645,83	2645,83	2646,38	2645,83	2646,38
961659	291941	2695	2645,85	2645,85	2645,85	2646,46	2645,85	2646,46
961689	291911	2543,33	2645,87	2645,87	2645,87	2646,54	2645,87	2646,55
961719	291881	2655	2645,89	2645,89	2645,89	2646,63	2645,89	2646,63
961749	291851	2718,33	2645,9	2645,9	2645,9	2646,72	2645,9	2646,72
961779	291821	2618,33	2645,9	2645,9	2645,9	2646,81	2645,9	2646,82
961809	291791	2545	2645,9	2645,9	2645,9	2646,91	2645,9	2646,91
961839	291761	2741,67	2645,9	2645,9	2645,9	2647	2645,9	2647,01
961869	291731	2656,67	2645,89	2645,89	2645,89	2647,11	2645,89	2647,12
961899	291701	2766,67	2645,87	2645,87	2645,88	2647,22	2645,87	2647,23
961929	291671	2623,33	2645,85	2645,85	2645,86	2647,33	2645,85	2647,34
961959	291641	2628,33	2645,82	2645,82	2645,83	2647,46	2645,82	2647,46
961989	291611	2711,67	2645,79	2645,79	2645,8	2647,59	2645,79	2647,6
962019	291581	2626,67	2645,75	2645,75	2645,76	2647,72	2645,75	2647,73
962049	291551	2705	2645,71	2645,71	2645,72	2647,87	2645,71	2647,89
962079	291521	2643,33	2645,66	2645,66	2645,67	2648,04	2645,66	2648,05
962109	291491	2735	2645,6	2645,6	2645,62	2648,21	2645,6	2648,22
962139	291461	2636,67	2645,54	2645,54	2645,56	2648,39	2645,54	2648,42
962169	291431	2708,33	2645,47	2645,47	2645,49	2648,6	2645,47	2648,62
962199	291401	2566,67	2645,39	2645,39	2645,42	2648,81	2645,39	2648,84
962229	291371	2736,67	2645,31	2645,31	2645,34	2649,05	2645,31	2649,09
962259	291341	2693,33	2645,22	2645,22	2645,25	2649,32	2645,22	2649,35
962289	291311	2630	2645,12	2645,12	2645,16	2649,59	2645,12	2649,64
962319	291281	2775	2645,02	2645,02	2645,06	2649,9	2645,02	2649,95
962349	291251	2658,33	2644,91	2644,91	2644,96	2650,24	2644,91	2650,29
962379	291221	2791,67	2644,79	2644,79	2644,85	2650,6	2644,79	2650,66
962409	291191	2781,67	2644,66	2644,67	2644,73	2650,99	2644,66	2651,06
962439	291161	2673,33	2644,53	2644,53	2644,61	2651,42	2644,53	2651,5
962469	291131	2601,67	2644,39	2644,39	2644,48	2651,88	2644,39	2651,97
962499	291101	2598,33	2644,24	2644,25	2644,34	2652,38	2644,24	2652,48
962529	291071	2711,67	2644,09	2644,09	2644,2	2652,94	2644,09	2653,06
962559	291041	2736,67	2643,92	2643,93	2644,05	2653,53	2643,92	2653,66

This journal is © The Royal Society of Chemistry 20xx

Please do not adjust margins

Please do not adjust margins

962589	291011	2750	2643,75	2643,76	2643,89	2654,17	2643,75	2654,32
962619	290981	2653,33	2643,57	2643,58	2643,73	2654,88	2643,57	2655,05
962649	290951	2586,67	2643,38	2643,39	2643,56	2655,64	2643,38	2655,83
962679	290921	2721,67	2643,18	2643,19	2643,38	2656,44	2643,18	2656,65
962709	290891	2725	2642,98	2642,99	2643,2	2657,34	2642,97	2657,58
962739	290861	2673,33	2642,76	2642,77	2643,02	2658,3	2642,76	2658,57
962769	290831	2685	2642,54	2642,55	2642,82	2659,31	2642,54	2659,62
962799	290801	2615	2642,3	2642,32	2642,63	2660,44	2642,3	2660,78
962829	290771	2690	2642,06	2642,08	2642,43	2661,64	2642,06	2662,03
962859	290741	2596,67	2641,81	2641,83	2642,22	2662,91	2641,81	2663,35
962889	290711	2745	2641,55	2641,58	2642,01	2664,31	2641,55	2664,8
962919	290681	2670	2641,28	2641,31	2641,79	2665,81	2641,28	2666,36
962949	290651	2655	2641	2641,03	2641,58	2667,39	2641	2668
962979	290621	2718,33	2640,71	2640,75	2641,35	2669,11	2640,71	2669,79
963009	290591	2836,67	2640,42	2640,45	2641,13	2670,96	2640,41	2671,72
963039	290561	2815	2640,11	2640,15	2640,91	2672,91	2640,1	2673,76
963069	290531	2720	2639,79	2639,84	2640,68	2675,01	2639,79	2675,96
963099	290501	2810	2639,46	2639,51	2640,46	2677,27	2639,46	2678,33
963129	290471	2685	2639,12	2639,18	2640,23	2679,65	2639,12	2680,83
963159	290441	2625	2638,77	2638,84	2640,01	2682,2	2638,77	2683,51
963189	290411	2813,33	2638,41	2638,49	2639,79	2684,94	2638,41	2686,41
963219	290381	2793,33	2638,04	2638,13	2639,57	2687,82	2638,04	2689,45
963249	290351	2791,67	2637,66	2637,75	2639,36	2690,87	2637,66	2692,68
963279	290321	2680	2637,27	2637,37	2639,15	2694,17	2637,26	2696,18
963309	290291	2736,67	2636,87	2636,98	2638,95	2697,62	2636,86	2699,85
963339	290261	2771,67	2636,46	2636,58	2638,76	2701,26	2636,45	2703,73
963369	290231	2811,67	2636,03	2636,17	2638,59	2705,19	2636,02	2707,92
963399	290201	2810	2635,6	2635,76	2638,43	2709,29	2635,59	2712,32
963429	290171	2736,67	2635,16	2635,33	2638,29	2713,58	2635,14	2716,93
963459	290141	2701,67	2634,71	2634,9	2638,16	2718,22	2634,69	2721,92
963489	290111	2820	2634,24	2634,46	2638,06	2723,05	2634,23	2727,14
963519	290081	2761,67	2633,78	2634,02	2637,98	2728,08	2633,76	2732,58
963549	290051	2720	2633,3	2633,57	2637,93	2733,49	2633,28	2738,46
963579	290021	2756,67	2632,81	2633,11	2637,92	2739,12	2632,79	2744,6
963609	289991	2696,67	2632,32	2632,65	2637,94	2744,96	2632,29	2751
963639	289961	2756,67	2631,82	2632,18	2638	2751,19	2631,79	2757,83
963669	289931	2735	2631,31	2631,72	2638,1	2757,68	2631,28	2764,98
963699	289901	2800	2630,8	2631,25	2638,25	2764,39	2630,76	2772,41
963729	289871	2895	2630,28	2630,78	2638,46	2771,49	2630,24	2780,3
963759	289841	2850	2629,76	2630,3	2638,73	2778,87	2629,71	2788,54
963789	289811	2693,33	2629,23	2629,83	2639,06	2786,49	2629,17	2797,08
963819	289781	2901,67	2628,69	2629,36	2639,46	2794,46	2628,63	2806,07
963849	289751	2728,33	2628,15	2628,89	2639,92	2802,75	2628,09	2815,44
963879	289721	2873,33	2627,61	2628,42	2640,47	2811,26	2627,54	2825,14
963909	289691	2726,67	2627,06	2627,95	2641,12	2820,09	2626,98	2835,28
963939	289661	2890	2626,51	2627,49	2641,87	2829,24	2626,42	2845,84
963969	289631	2841,67	2625,96	2627,04	2642,71	2838,59	2625,86	2856,72

This journal is © The Royal Society of Chemistry 20xx

Please do not adjust margins

963999	289601	2880	2625,4	2626,59	2643,66	2848,22	2625,29	2867,98
964029	289571	2876,67	2624,84	2626,15	2644,72	2858,13	2624,72	2879,67
964059	289541	2910	2624,28	2625,72	2645,92	2868,21	2624,15	2891,68
964089	289511	2966,67	2623,72	2625,3	2647,27	2878,5	2623,57	2904,06
964119	289481	2856,67	2623,15	2624,89	2648,76	2889,01	2623	2916,83
964149	289451	2816,67	2622,59	2624,49	2650,41	2899,64	2622,42	2929,88
964179	289421	2933,33	2622,03	2624,11	2652,22	2910,38	2621,84	2943,23
964209	289391	2998,33	2621,47	2623,75	2654,2	2921,24	2621,25	2956,89
964239	289361	2983,33	2620,9	2623,41	2656,39	2932,14	2620,67	2970,82
964269	289331	2835	2620,34	2623,08	2658,8	2943,05	2620,09	2985,01
964299	289301	3025	2619,78	2622,77	2661,42	2953,92	2619,5	2999,39
964329	289271	3076,67	2619,23	2622,5	2664,26	2964,72	2618,92	3013,96
964359	289241	2945	2618,68	2622,25	2667,34	2975,43	2618,34	3028,7
964389	289211	3050	2618,13	2622,03	2670,67	2985,89	2617,75	3043,46
964419	289181	3048,33	2617,58	2621,84	2674,32	2996,15	2617,17	3058,38
964449	289151	3035	2617,04	2621,69	2678,27	3006,19	2616,59	3073,42
964479	289121	3076,67	2616,5	2621,57	2682,51	3015,78	2616,01	3088,33
964509	289091	2968,33	2615,97	2621,49	2687,07	3024,99	2615,43	3103,22
964539	289061	3158,33	2615,45	2621,47	2691,96	3033,85	2614,86	3118,15
964569	289031	3176,67	2614,94	2621,48	2697,19	3042,06	2614,29	3132,81
964599	289001	3086,67	2614,43	2621,54	2702,87	3049,7	2613,72	3147,39
964629	288971	3251,67	2613,93	2621,67	2708,95	3056,85	2613,15	3161,95
964659	288941	3131,67	2613,44	2621,85	2715,43	3063,21	2612,59	3176,16
964689	288911	3250	2612,96	2622,08	2722,33	3068,78	2612,03	3190,06
964719	288881	3220	2612,49	2622,4	2729,67	3073,76	2611,48	3203,89
964749	288851	3183,33	2612,04	2622,79	2737,46	3077,84	2610,93	3217,35
964779	288821	3241,67	2611,6	2623,24	2745,85	3080,94	2610,38	3230,48
964809	288791	3166,67	2611,17	2623,79	2754,73	3083,37	2609,84	3243,53
964839	288761	3246,67	2610,75	2624,44	2764,12	3084,88	2609,31	3256,25
964869	288731	3375	2610,36	2625,16	2774,03	3085,27	2608,78	3268,46
964899	288701	3128,33	2609,98	2625,98	2784,47	3084,95	2608,26	3280,6
964929	288671	3295	2609,61	2626,94	2795,5	3083,75	2607,75	3292,56
964959	288641	3278,33	2609,27	2627,99	2807,2	3081,36	2607,24	3304,11
964989	288611	3398,33	2608,95	2629,15	2819,47	3078,28	2606,74	3315,63
965019	288581	3243,33	2608,65	2630,48	2832,31	3074,42	2606,24	3327,13
965049	288551	3328,33	2608,38	2631,93	2845,73	3069,39	2605,76	3338,16
965079	288521	3403,33	2608,13	2633,5	2859,73	3063,73	2605,28	3349,24
965109	288491	3285	2607,91	2635,27	2874,34	3057,44	2604,81	3360,52
965139	288461	3368,33	2607,71	2637,21	2889,59	3050,08	2604,35	3371,53
965169	288431	3518,33	2607,55	2639,28	2905,39	3042,19	2603,9	3382,71
965199	288401	3346,67	2607,42	2641,57	2921,69	3033,8	2603,46	3394,1
965229	288371	3445	2607,32	2644,08	2938,47	3024,59	2603,03	3405,37
965259	288341	3593,33	2607,26	2646,76	2955,7	3014,95	2602,61	3416,84
965289	288311	3433,33	2607,24	2649,65	2973,37	3004,93	2602,2	3428,6
965319	288281	3443,33	2607,26	2652,85	2991,41	2994,37	2601,8	3440,5
965349	288251	3303,33	2607,32	2656,25	3009,69	2983,5	2601,41	3452,55
965379	288221	3388,33	2607,43	2659,87	3028,16	2972,4	2601,03	3464,77

965409	288191	3535	2607,59	2663,87	3046,72	2960,98	2600,66	3477,18
965439	288161	3431,67	2607,79	2668,13	3065,29	2949,41	2600,31	3489,7
965469	288131	3413,33	2608,06	2672,64	3083,73	2937,73	2599,97	3502,27
965499	288101	3456,67	2608,39	2677,53	3101,89	2925,95	2599,64	3514,85
965529	288071	3580	2608,76	2682,8	3119,66	2914,14	2599,32	3527,4
965559	288041	3515	2609,22	2688,36	3136,9	2902,35	2599,02	3539,78
965589	288011	3646,67	2609,74	2694,3	3153,51	2890,61	2598,73	3551,97
965619	287981	3653,33	2610,32	2700,75	3169,33	2878,98	2598,46	3564,02
965649	287951	3686,67	2611	2707,54	3184,03	2867,46	2598,2	3575,44
965679	287921	3621,67	2611,75	2714,69	3197,52	2856,09	2597,95	3586,21
965709	287891	3595	2612,57	2722,5	3209,76	2844,95	2597,72	3596,63
965739	287861	3595	2613,51	2730,73	3220,6	2833,99	2597,5	3606,32
965769	287831	3670	2614,53	2739,36	3229,95	2823,24	2597,3	3615,19
965799	287801	3666,67	2615,64	2748,67	3237,71	2812,83	2597,12	3623,48
965829	287771	3665	2616,89	2758,54	3243,36	2802,63	2596,95	3630,56
965859	287741	3541,67	2618,24	2768,89	3247,15	2792,67	2596,8	3636,55
965889	287711	3650	2619,68	2779,89	3249,11	2783,13	2596,67	3641,81
965919	287681	3710	2621,29	2791,65	3249,21	2773,82	2596,55	3646,33
965949	287651	3523,33	2623,02	2803,97	3247,44	2764,77	2596,45	3649,85
965979	287621	3655	2624,86	2816,89	3243,83	2756,17	2596,37	3652,64
966009	287591	3558,33	2626,9	2830,81	3237,95	2747,83	2596,31	3654,57
966039	287561	3583,33	2629,08	2845,35	3230,36	2739,74	2596,26	3655,74
966069	287531	3656,67	2631,38	2860,53	3221,15	2732,09	2596,24	3656,44
966099	287501	3663,33	2633,93	2876,77	3210,42	2724,73	2596,23	3657,16
966129	287471	3741,67	2636,63	2893,81	3198,27	2717,61	2596,24	3657,6
966159	287441	3636,67	2639,47	2911,56	3184,77	2710,9	2596,26	3657,91
966189	287411	3690	2642,6	2930,33	3169,9	2704,48	2596,31	3658,38
966219	287381	3580	2645,9	2950,14	3154,07	2698,3	2596,36	3659,31
966249	287351	3630	2649,37	2970,73	3137,43	2692,48	2596,44	3660,7
966279	287321	3618,33	2653,17	2992,24	3120,1	2686,95	2596,52	3662,89
966309	287291	3718,33	2657,15	3015,08	3102,22	2681,64	2596,62	3666,23
966339	287261	3683,33	2661,35	3038,76	3083,89	2676,65	2596,73	3670,44
966369	287231	3630	2665,92	3063,29	3065,25	2671,94	2596,86	3675,83
966399	287201	3711,67	2670,71	3089,3	3046,48	2667,44	2596,99	3682,97
966429	287171	3763,33	2675,75	3116,27	3027,7	2663,2	2597,13	3691,52
966459	287141	3663,33	2681,22	3144,14	3008,98	2659,24	2597,29	3701,71
966489	287111	3748,33	2686,93	3173,34	2990,41	2655,45	2597,45	3713,78
966519	287081	3753,33	2692,95	3203,72	2972,1	2651,89	2597,62	3727,8
966549	287051	3795	2699,45	3235,01	2954,16	2648,58	2597,8	3743,82
966579	287021	3790	2706,23	3267,44	2936,61	2645,44	2597,98	3761,78
966609	286991	3696,67	2713,38	3301,23	2919,49	2642,47	2598,17	3782,06
966639	286961	3833,33	2721,07	3335,91	2902,83	2639,74	2598,36	3804,46
966669	286931	3888,33	2729,09	3371,47	2886,66	2637,15	2598,56	3828,69
966699	286901	3815	2737,53	3408,5	2871,1	2634,71	2598,76	3855,54
966729	286871	3893,33	2746,6	3446,31	2856,15	2632,48	2598,97	3884,63
966759	286841	3806,67	2756,04	3484,88	2841,76	2630,37	2599,18	3915,52
966789	286811	3896,67	2765,99	3524,56	2827,93	2628,37	2599,39	3948,7

This journal is © The Royal Society of Chemistry 20xx

Please do not adjust margins

966819	286781	4085	2776,64	3564,98	2814,67	2626,57	2599,59	3984,08
966849	286751	4018,33	2787,72	3605,97	2801,98	2624,87	2599,8	4021,13
966879	286721	4010	2799,4	3647,62	2789,95	2623,26	2600,01	4060,2
966909	286691	4135	2811,86	3689,8	2778,52	2621,81	2600,22	4101,33
966939	286661	4280	2824,81	3732,23	2767,64	2620,44	2600,42	4143,85
966969	286631	4281,67	2838,47	3774,87	2757,28	2619,16	2600,62	4187,91
966999	286601	4298,33	2853,01	3817,54	2747,44	2617,99	2600,82	4233,52
967029	286571	4251,67	2868,1	3860,04	2738,1	2616,91	2601,01	4280,11
967059	286541	4361,67	2884,03	3902,29	2729,35	2615,89	2601,2	4327,96
967089	286511	4396,67	2900,93	3943,84	2721,09	2614,96	2601,38	4376,67
967119	286481	4556,67	2918,46	3984,61	2713,27	2614,11	2601,56	4425,77
967149	286451	4355	2936,97	4024,58	2705,88	2613,3	2601,73	4475,55
967179	286421	4588,33	2956,55	4063,19	2698,91	2612,56	2601,89	4525,55
967209	286391	4578,33	2976,85	4100,11	2692,33	2611,88	2602,04	4575,05
967239	286361	4955	2998,28	4135,59	2686,21	2611,24	2602,18	4624,79
967269	286331	4648,33	3020,89	4169,27	2680,46	2610,65	2602,31	4674,34
967299	286301	4605	3044,3	4200,04	2675,04	2610,11	2602,43	4722,2
967329	286271	4570	3069,02	4228,75	2669,94	2609,6	2602,54	4769,69
967359	286241	4761,67	3095,03	4255,35	2665,14	2609,11	2602,63	4816,73
967389	286211	4803,33	3121,93	4277,9	2660,64	2608,67	2602,72	4861
967419	286181	4928,33	3150,34	4297,74	2656,47	2608,25	2602,78	4904,44
967449	286151	4991,67	3180,13	4314,93	2652,55	2607,84	2602,84	4946,95
967479	286121	4975	3210,92	4328,02	2648,87	2607,47	2602,88	4986,66
967509	286091	5018,33	3243,42	4337,38	2645,41	2607,1	2602,9	5024,62
967539	286061	4961,67	3277,4	4343,78	2642,16	2606,74	2602,9	5061,38
967569	286031	5111,67	3312,47	4346,43	2639,12	2606,41	2602,89	5095,76
967599	286001	5046,67	3349,45	4344,51	2636,31	2606,08	2602,86	5127,76
967629	285971	5140	3388,01	4339,58	2633,65	2605,74	2602,81	5158,55
967659	285941	5073,33	3427,74	4331,58	2631,16	2605,42	2602,74	5187,67
967689	285911	5153,33	3469,59	4318,51	2628,8	2605,09	2602,65	5214,05
967719	285881	5180	3513,06	4302,67	2626,59	2604,76	2602,54	5239,47
967749	285851	5210	3557,8	4284,08	2624,51	2604,43	2602,4	5263,61
967779	285821	5133,33	3604,83	4261,45	2622,57	2604,1	2602,25	5286,2
967809	285791	5183,33	3653,51	4235,97	2620,73	2603,75	2602,07	5307,75
967839	285761	5396,67	3703,5	4208,23	2618,98	2603,39	2601,86	5328,51
967869	285731	5370	3755,92	4177,67	2617,32	2603,02	2601,64	5349,03
967899	285701	5458,33	3809,96	4144,48	2615,74	2602,64	2601,38	5368,68
967929	285671	5495	3865,32	4109,63	2614,25	2602,24	2601,1	5388,15
967959	285641	5543,33	3923,17	4073,07	2612,83	2601,83	2600,79	5408,51
967989	285611	5533,33	3982,53	4034,41	2611,46	2601,39	2600,46	5428,42
968019	285581	5368,33	4043,18	3994,73	2610,14	2600,94	2600,09	5448,72
968049	285551	5410	4106,23	3954,1	2608,87	2600,47	2599,7	5470,57
968079	285521	5523,33	4170,61	3912,31	2607,63	2599,97	2599,27	5492,69
968109	285491	5478,33	4236,15	3870	2606,44	2599,45	2598,82	5515,58
968139	285461	5531,67	4303,82	3827,32	2605,28	2598,9	2598,33	5540,32
968169	285431	5510	4372,53	3784,33	2604,14	2598,33	2597,82	5565,87
968199	285401	5586,67	4442,18	3741,32	2603,01	2597,72	2597,26	5592,45

This journal is © The Royal Society of Chemistry 20xx

Please do not adjust margins

Please do not adjust margins

968229	285371	5748,33	4513,43	3698,41	2601,9	2597,09	2596,68	5620,79
968259	285341	5770	4585,3	3655,7	2600,8	2596,43	2596,06	5650,05
968289	285311	5653,33	4657,76	3613,53	2599,71	2595,74	2595,4	5680,52
968319	285281	5918,33	4730,97	3571,81	2598,62	2595,02	2594,71	5712,26
968349	285251	5846,67	4804,25	3530,55	2597,52	2594,26	2593,99	5744,62
968379	285221	5733,33	4877,62	3490,32	2596,42	2593,47	2593,22	5778,16
968409	285191	5860	4950,53	3450,79	2595,32	2592,64	2592,42	5812,01
968439	285161	5873,33	5022,86	3411,95	2594,19	2591,78	2591,58	5846,03
968469	285131	5883,33	5094,54	3374,23	2593,06	2590,87	2590,7	5880,61
968499	285101	5860	5164,31	3337,53	2591,91	2589,93	2589,78	5914,35
968529	285071	5903,33	5232,69	3301,64	2590,74	2588,96	2588,82	5947,58
968559	285041	5836,67	5299,5	3266,82	2589,55	2587,94	2587,81	5980,36
968589	285011	6046,67	5362,75	3233,29	2588,33	2586,88	2586,77	6010,94
968619	284981	6255	5423,73	3200,62	2587,08	2585,78	2585,68	6040,17
968649	284951	6140	5482,04	3168,85	2585,81	2584,63	2584,54	6067,7
968679	284921	5886,67	5535,14	3138,6	2584,5	2583,44	2583,36	6091,59
968709	284891	6140	5585,09	3109,2	2583,16	2582,21	2582,14	6113,24
968739	284861	5921,67	5631,2	3080,65	2581,79	2580,93	2580,87	6131,96
968769	284831	6155	5670,64	3053,46	2580,37	2579,61	2579,56	6145,41
968799	284801	6173,33	5706,14	3027,24	2578,92	2578,24	2578,19	6155,97
968829	284771	6238,33	5736,73	3001,83	2577,44	2576,82	2576,78	6162,49
968859	284741	6341,67	5759,58	2977,51	2575,91	2575,36	2575,32	6162,4
968889	284711	6301,67	5777,96	2954,29	2574,34	2573,85	2573,81	6159
968919	284681	6235	5790,59	2931,82	2572,73	2572,29	2572,26	6150,65
968949	284651	6125	5795,01	2910,18	2571,08	2570,7	2570,67	6134,97
968979	284621	6053,33	5794,74	2889,73	2569,4	2569,05	2569,03	6115,84
969009	284591	6118,33	5788,29	2869,96	2567,68	2567,37	2567,35	6091,26
969039	284561	6153,33	5773,82	2850,85	2565,92	2565,64	2565,62	6059,37
969069	284531	5905	5754,79	2832,84	2564,12	2563,87	2563,86	6024,05
969099	284501	5753,33	5729,66	2815,5	2562,29	2562,07	2562,05	5983,36
969129	284471	5855	5697,31	2798,76	2560,42	2560,22	2560,21	5936,08
969159	284441	5910	5660,87	2782,85	2558,51	2558,34	2558,33	5885,59
969189	284411	5768,33	5618,88	2767,68	2556,57	2556,42	2556,41	5830,33
969219	284381	5685	5570,95	2753,04	2554,6	2554,47	2554,46	5769,69
969249	284351	5455	5519,65	2739,01	2552,59	2552,47	2552,47	5706,33
969279	284321	5623,33	5463,76	2725,75	2550,56	2550,45	2550,44	5639,18
969309	284291	5588,33	5403,44	2712,94	2548,48	2548,39	2548,39	5568,1
969339	284261	5616,67	5340,61	2700,58	2546,38	2546,3	2546,3	5494,99
969369	284231	5280	5274,39	2688,97	2544,25	2544,18	2544,18	5419,27
969399	284201	5241,67	5205,29	2677,77	2542,09	2542,03	2542,02	5341,11
969429	284171	5186,67	5134,57	2666,95	2539,9	2539,85	2539,84	5261,74
969459	284141	5098,33	5061,74	2656,7	2537,69	2537,64	2537,63	5180,86
969489	284111	4990	4987,43	2646,88	2535,44	2535,4	2535,4	5098,96
969519	284081	4936,67	4912,32	2637,38	2533,17	2533,13	2533,13	5016,62
969549	284051	4993,33	4836,32	2628,3	2530,88	2530,84	2530,84	4933,82
969579	284021	4966,67	4759,99	2619,67	2528,56	2528,53	2528,53	4851,17
969609	283991	4751,67	4683,58	2611,31	2526,22	2526,19	2526,19	4768,73

 This journal is © The Royal Society of Chemistry 20xx

Please do not adjust margins

Please do not adjust margins

969639	283961	4713,33	4607,31	2603,25	2523,85	2523,83	2523,83	4686,76
969669	283931	4828,33	4531,59	2595,62	2521,47	2521,45	2521,44	4605,8
969699	283901	4590	4456,33	2588,24	2519,06	2519,04	2519,04	4525,55
969729	283871	4321,67	4382,08	2581,08	2516,63	2516,62	2516,62	4446,56
969759	283841	4263,33	4308,95	2574,27	2514,19	2514,17	2514,17	4369,07
969789	283811	4355	4236,69	2567,7	2511,72	2511,71	2511,71	4292,7
969819	283781	4311,67	4166,06	2561,33	2509,24	2509,23	2509,23	4218,16
969849	283751	4118,33	4096,91	2555,21	2506,74	2506,73	2506,73	4145,4
969879	283721	3928,33	4028,89	2549,33	2504,23	2504,22	2504,22	4074,01
969909	283691	4005	3962,93	2543,61	2501,7	2501,7	2501,7	4004,85
969939	283661	3851,67	3898,63	2538,07	2499,16	2499,16	2499,16	3937,55
969969	283631	3766,67	3835,61	2532,77	2496,61	2496,6	2496,6	3871,78
969999	283601	3765	3774,89	2527,61	2494,04	2494,04	2494,04	3808,47
970029	283571	3665	3715,89	2522,57	2491,47	2491,47	2491,47	3747
970059	283541	3755	3658,22	2517,75	2488,88	2488,88	2488,88	3687,09
970089	283511	3571,67	3602,98	2513,04	2486,29	2486,29	2486,29	3629,74
970119	283481	3570	3549,4	2508,45	2483,69	2483,69	2483,69	3574,17
970149	283451	3585	3497,15	2504,01	2481,08	2481,08	2481,08	3520,09
970179	283421	3491,67	3447,35	2499,69	2478,46	2478,46	2478,46	3468,58
970209	283391	3566,67	3399,1	2495,47	2475,84	2475,84	2475,84	3418,73
970239	283361	3416,67	3352,15	2491,35	2473,22	2473,22	2473,22	3370,28
970269	283331	3393,33	3307,58	2487,35	2470,59	2470,59	2470,59	3324,35
970299	283301	3306,67	3264,44	2483,44	2467,96	2467,96	2467,96	3279,91
970329	283271	3108,33	3222,51	2479,59	2465,33	2465,33	2465,33	3236,78
970359	283241	3133,33	3182,86	2475,87	2462,7	2462,7	2462,7	3196,04
970389	283211	3188,33	3144,49	2472,21	2460,07	2460,07	2460,07	3156,63
970419	283181	3271,67	3107,25	2468,62	2457,44	2457,44	2457,44	3118,43
970449	283151	3130	3072,14	2465,11	2454,81	2454,81	2454,81	3082,45
970479	283121	3215	3038,16	2461,67	2452,19	2452,19	2452,19	3047,65
970509	283091	3206,67	3005,23	2458,29	2449,57	2449,57	2449,57	3013,95
970539	283061	3201,67	2974,27	2454,97	2446,95	2446,95	2446,95	2982,29
970569	283031	2985	2944,3	2451,72	2444,34	2444,34	2444,34	2951,67
970599	283001	3096,67	2915,27	2448,51	2441,74	2441,74	2441,74	2922,04
970629	282971	2918,33	2888,06	2445,36	2439,15	2439,15	2439,15	2894,26
970659	282941	3010	2861,69	2442,27	2436,56	2436,56	2436,56	2867,39
970689	282911	3115	2836,21	2439,22	2433,99	2433,99	2433,99	2841,44
970719	282881	2933,33	2812,32	2436,21	2431,43	2431,43	2431,43	2817,11
970749	282851	2976,67	2789,19	2433,26	2428,87	2428,87	2428,87	2793,58
970779	282821	2926,67	2766,88	2430,35	2426,33	2426,33	2426,33	2770,9
970809	282791	2906,67	2745,96	2427,48	2423,81	2423,81	2423,81	2749,63
970839	282761	2900	2725,7	2424,66	2421,3	2421,3	2421,3	2729,06
970869	282731	2726,67	2706,21	2421,87	2418,8	2418,8	2418,8	2709,28
970899	282701	2686,67	2687,91	2419,13	2416,32	2416,32	2416,32	2690,72
970929	282671	2763,33	2670,21	2416,42	2413,86	2413,86	2413,86	2672,77
970959	282641	2751,67	2653,2	2413,75	2411,41	2411,41	2411,41	2655,54
970989	282611	2743,33	2637,23	2411,12	2408,99	2408,99	2408,99	2639,36
971019	282581	2751,67	2621,78	2408,52	2406,58	2406,58	2406,58	2623,72

This journal is © The Royal Society of Chemistry 20xx

Please do not adjust margins

971049	282551	2840	2606,97	2405,97	2404,2	2404,2	2404,2	2608,74
971079	282521	2641,67	2593,04	2403,45	2401,84	2401,84	2401,84	2594,65
971109	282491	2616,67	2579,57	2400,97	2399,5	2399,5	2399,5	2581,04
971139	282461	2671,67	2566,68	2398,52	2397,19	2397,19	2397,19	2568,01
971169	282431	2598,33	2554,55	2396,11	2394,9	2394,9	2394,9	2555,76
971199	282401	2708,33	2542,83	2393,73	2392,63	2392,63	2392,63	2543,93
971229	282371	2563,33	2531,63	2391,39	2390,4	2390,4	2390,4	2532,62
971259	282341	2640	2521,07	2389,09	2388,19	2388,19	2388,19	2521,98
971289	282311	2621,67	2510,88	2386,83	2386,01	2386,01	2386,01	2511,7
971319	282281	2548,33	2501,15	2384,6	2383,85	2383,85	2383,85	2501,89
971349	282251	2463,33	2491,97	2382,41	2381,73	2381,73	2381,73	2492,65
971379	282221	2518,33	2483,12	2380,25	2379,64	2379,64	2379,64	2483,73
971409	282191	2541,67	2474,68	2378,14	2377,59	2377,59	2377,59	2475,24
971439	282161	2633,33	2466,72	2376,06	2375,56	2375,56	2375,56	2467,22
971469	282131	2550	2459,04	2374,02	2373,57	2373,57	2373,57	2459,49
971499	282101	2601,67	2451,73	2372,03	2371,62	2371,62	2371,62	2452,14
971529	282071	2480	2444,82	2370,07	2369,7	2369,7	2369,7	2445,19
971559	282041	2423,33	2438,17	2368,15	2367,82	2367,82	2367,82	2438,5
971589	282011	2471,67	2431,85	2366,27	2365,98	2365,98	2365,98	2432,15
971619	281981	2575	2425,87	2364,44	2364,17	2364,17	2364,17	2426,14
971649	281951	2395	2420,11	2362,64	2362,4	2362,4	2362,4	2420,35
971679	281921	2451,67	2414,65	2360,89	2360,67	2360,67	2360,67	2414,87
971709	281891	2453,33	2409,48	2359,17	2358,97	2358,97	2358,97	2409,67
971739	281861	2351,67	2404,49	2357,49	2357,31	2357,31	2357,31	2404,67
971769	281831	2373,33	2399,77	2355,85	2355,69	2355,69	2355,69	2399,93
971799	281801	2386,67	2395,29	2354,24	2354,1	2354,1	2354,1	2395,44
971829	281771	2458,33	2390,98	2352,67	2352,55	2352,55	2352,55	2391,11
971859	281741	2390	2386,9	2351,14	2351,03	2351,03	2351,03	2387,02
971889	281711	2385	2383,02	2349,64	2349,54	2349,54	2349,54	2383,12
971919	281681	2373,33	2379,29	2348,18	2348,09	2348,09	2348,09	2379,38
971949	281651	2375	2375,76	2346,75	2346,67	2346,67	2346,67	2375,84
971979	281621	2308,33	2372,4	2345,35	2345,28	2345,28	2345,28	2372,47
972009	281591	2335	2369,16	2343,99	2343,92	2343,92	2343,92	2369,23
972039	281561	2426,67	2366,11	2342,66	2342,6	2342,6	2342,6	2366,17
972069	281531	2200	2363,19	2341,36	2341,31	2341,31	2341,31	2363,25
972099	281501	2376,67	2360,39	2340,09	2340,05	2340,05	2340,05	2360,44
972129	281471	2340	2357,74	2338,86	2338,82	2338,82	2338,82	2357,78
972159	281441	2383,33	2355,21	2337,65	2337,62	2337,62	2337,62	2355,25
972189	281411	2468,33	2352,78	2336,48	2336,45	2336,45	2336,45	2352,81
972219	281381	2268,33	2350,49	2335,33	2335,3	2335,3	2335,3	2350,51
972249	281351	2193,33	2348,29	2334,22	2334,19	2334,19	2334,19	2348,32
972279	281321	2263,33	2346,18	2333,13	2333,11	2333,11	2333,11	2346,2
972309	281291	2371,67	2344,18	2332,07	2332,05	2332,05	2332,05	2344,21
972339	281261	2425	2342,28	2331,04	2331,02	2331,02	2331,02	2342,29
972369	281231	2390	2340,44	2330,04	2330,02	2330,02	2330,02	2340,46
972399	281201	2295	2338,71	2329,06	2329,05	2329,05	2329,05	2338,72
972429	281171	2381,67	2337,05	2328,12	2328,1	2328,1	2328,1	2337,06

This journal is © The Royal Society of Chemistry 20xx

Please do not adjust margins

972459	281141	2315	2335,45	2327,19	2327,18	2327,18	2327,18	2335,46
972489	281111	2310	2333,95	2326,29	2326,28	2326,28	2326,28	2333,96
972519	281081	2353,33	2332,5	2325,42	2325,41	2325,41	2325,41	2332,51
972549	281051	2258,33	2331,11	2324,58	2324,57	2324,57	2324,57	2331,12
972579	281021	2348,33	2329,8	2323,75	2323,75	2323,75	2323,75	2329,81
972609	280991	2480	2328,54	2322,95	2322,95	2322,95	2322,95	2328,55
972639	280961	2298,33	2327,33	2322,18	2322,17	2322,17	2322,17	2327,34
972669	280931	2385	2326,19	2321,43	2321,42	2321,42	2321,42	2326,19
972699	280901	2318,33	2325,09	2320,7	2320,69	2320,69	2320,69	2325,09
972729	280871	2353,33	2324,04	2319,99	2319,99	2319,99	2319,99	2324,04
972759	280841	2281,67	2323,04	2319,31	2319,3	2319,3	2319,3	2323,04
972789	280811	2285	2322,08	2318,64	2318,64	2318,64	2318,64	2322,09
972819	280781	2338,33	2321,16	2318	2318	2318	2318	2321,17
972849	280751	2305	2320,3	2317,38	2317,38	2317,38	2317,38	2320,3
972879	280721	2331,67	2319,46	2316,78	2316,78	2316,78	2316,78	2319,46
972909	280691	2298,33	2318,66	2316,2	2316,2	2316,2	2316,2	2318,66
972939	280661	2328,33	2317,9	2315,64	2315,64	2315,64	2315,64	2317,91
972969	280631	2360	2317,18	2315,09	2315,09	2315,09	2315,09	2317,18
972999	280601	2306,67	2316,48	2314,57	2314,57	2314,57	2314,57	2316,48
973029	280571	2313,33	2315,82	2314,06	2314,06	2314,06	2314,06	2315,82
973059	280541	2303,33	2315,19	2313,58	2313,57	2313,57	2313,57	2315,19
973089	280511	2365	2314,58	2313,1	2313,1	2313,1	2313,1	2314,58
973119	280481	2395	2314,01	2312,65	2312,65	2312,65	2312,65	2314,01
973149	280451	2443,33	2313,46	2312,21	2312,21	2312,21	2312,21	2313,46
973179	280421	2425	2312,93	2311,79	2311,79	2311,79	2311,79	2312,93
973209	280391	2273,33	2312,43	2311,39	2311,39	2311,39	2311,39	2312,43
973239	280361	2258,33	2311,95	2311	2311	2311	2311	2311,95
973269	280331	2338,33	2311,49	2310,62	2310,62	2310,62	2310,62	2311,49
973299	280301	2316,67	2311,06	2310,26	2310,26	2310,26	2310,26	2311,06
973329	280271	2350	2310,64	2309,91	2309,91	2309,91	2309,91	2310,64
973359	280241	2303,33	2310,24	2309,58	2309,58	2309,58	2309,58	2310,24
973389	280211	2375	2309,87	2309,26	2309,26	2309,26	2309,26	2309,87
973419	280181	2285	2309,51	2308,95	2308,95	2308,95	2308,95	2309,51
973449	280151	2305	2309,16	2308,66	2308,66	2308,66	2308,66	2309,16
973479	280121	2378,33	2308,84	2308,37	2308,37	2308,37	2308,37	2308,84
973509	280091	2233,33	2308,52	2308,1	2308,1	2308,1	2308,1	2308,52
973539	280061	2371,67	2308,23	2307,84	2307,84	2307,84	2307,84	2308,23
973569	280031	2366,67	2307,94	2307,59	2307,59	2307,59	2307,59	2307,94
973599	280001	2291,67	2307,67	2307,35	2307,35	2307,35	2307,35	2307,67
973629	279971	2416,67	2307,41	2307,12	2307,12	2307,12	2307,12	2307,41
973659	279941	2310	2307,17	2306,9	2306,9	2306,9	2306,9	2307,17
973689	279911	2261,67	2306,93	2306,69	2306,69	2306,69	2306,69	2306,93
973719	279881	2291,67	2306,71	2306,49	2306,49	2306,49	2306,49	2306,71
973749	279851	2360	2306,5	2306,3	2306,3	2306,3	2306,3	2306,5
973779	279821	2383,33	2306,29	2306,11	2306,11	2306,11	2306,11	2306,29
973809	279791	2415	2306,1	2305,93	2305,93	2305,93	2305,93	2306,1
973839	279761	2375	2305,91	2305,76	2305,76	2305,76	2305,76	2305,91

This journal is © The Royal Society of Chemistry 20xx

Please do not adjust margins

Please do not adjust margins

973869	279731	2298,33	2305,73	2305,6	2305,6	2305,6	2305,6	2305,73
973899	279701	2398,33	2305,56	2305,44	2305,44	2305,44	2305,44	2305,56
973929	279671	2496,67	2305,4	2305,29	2305,29	2305,29	2305,29	2305,4
973959	279641	2381,67	2305,24	2305,14	2305,14	2305,14	2305,14	2305,24
973989	279611	2416,67	2305,09	2305	2305	2305	2305	2305,09
974019	279581	2291,67	2304,94	2304,86	2304,86	2304,86	2304,86	2304,94
974049	279551	2315	2304,8	2304,72	2304,72	2304,72	2304,72	2304,8
974079	279521	2393,33	2304,66	2304,59	2304,59	2304,59	2304,59	2304,66
974109	279491	2300	2304,53	2304,47	2304,47	2304,47	2304,47	2304,53
974139	279461	2463,33	2304,4	2304,34	2304,34	2304,34	2304,34	2304,4
974169	279431	2260	2304,27	2304,22	2304,22	2304,22	2304,22	2304,27
974199	279401	2293,33	2304,15	2304,1	2304,1	2304,1	2304,1	2304,15
974229	279371	2335	2304,02	2303,98	2303,98	2303,98	2303,98	2304,02
974259	279341	2328,33	2303,9	2303,87	2303,87	2303,87	2303,87	2303,9
974289	279311	2261,67	2303,78	2303,75	2303,75	2303,75	2303,75	2303,78
974319	279281	2395	2303,67	2303,64	2303,64	2303,64	2303,64	2303,67
974349	279251	2281,67	2281,69	2281,67	2281,67	2281,67	2281,67	2281,69
974379	279221	2271,67	2271,69	2271,67	2271,67	2271,67	2271,67	2271,69
974409	279191	2293,33	2293,35	2293,33	2293,33	2293,33	2293,33	2293,35
974439	279161	2250	2250,02	2250	2250	2250	2250	2250,02
974469	279131	2370	2370,02	2370	2370	2370	2370	2370,02
974499	279101	2296,67	2296,68	2296,67	2296,67	2296,67	2296,67	2296,68
974529	279071	2328,33	2328,35	2328,33	2328,33	2328,33	2328,33	2328,35
974559	279041	2280	2280,01	2280	2280	2280	2280	2280,01
974589	279011	2270	2270,01	2270	2270	2270	2270	2270,01
974619	278981	2308,33	2308,34	2308,33	2308,33	2308,33	2308,33	2308,34
974649	278951	2411,67	2411,68	2411,67	2411,67	2411,67	2411,67	2411,68
974679	278921	2231,67	2231,67	2231,67	2231,67	2231,67	2231,67	2231,67
974709	278891	2303,33	2303,34	2303,33	2303,33	2303,33	2303,33	2303,34
974739	278861	2351,67	2351,67	2351,67	2351,67	2351,67	2351,67	2351,67
974769	278831	2370	2370,01	2370	2370	2370	2370	2370,01
974799	278801	2351,67	2351,67	2351,67	2351,67	2351,67	2351,67	2351,67
974829	278771	2478,33	2478,34	2478,33	2478,33	2478,33	2478,33	2478,34
974859	278741	2406,67	2406,67	2406,67	2406,67	2406,67	2406,67	2406,67
974889	278711	2276,67	2276,67	2276,67	2276,67	2276,67	2276,67	2276,67
974919	278681	2271,67	2271,67	2271,67	2271,67	2271,67	2271,67	2271,67
974949	278651	2406,67	2406,67	2406,67	2406,67	2406,67	2406,67	2406,67
974979	278621	2521,67	2521,67	2521,67	2521,67	2521,67	2521,67	2521,67
975009	278591	2390	2390	2390	2390	2390	2390	2390
975039	278561	2356,67	2356,67	2356,67	2356,67	2356,67	2356,67	2356,67
975069	278531	2341,67	2341,67	2341,67	2341,67	2341,67	2341,67	2341,67
975099	278501	2278,33	2278,33	2278,33	2278,33	2278,33	2278,33	2278,33
975129	278471	2478,33	2478,33	2478,33	2478,33	2478,33	2478,33	2478,33
975159	278441	2391,67	2391,67	2391,67	2391,67	2391,67	2391,67	2391,67
975189	278411	2371,67	2371,67	2371,67	2371,67	2371,67	2371,67	2371,67
975219	278381	2293,33	2293,33	2293,33	2293,33	2293,33	2293,33	2293,33
975249	278351	2338,33	2338,33	2338,33	2338,33	2338,33	2338,33	2338,33

This journal is © The Royal Society of Chemistry 20xx

Please do not adjust margins

Please do not adjust margins

975279	278321	2368,33	2368,33	2368,33	2368,33	2368,33	2368,33	2368,33
975309	278291	2465	2465	2465	2465	2465	2465	2465
975339	278261	2286,67	2286,67	2286,67	2286,67	2286,67	2286,67	2286,67
975369	278231	2363,33	2363,33	2363,33	2363,33	2363,33	2363,33	2363,33
975399	278201	2198,33	2198,33	2198,33	2198,33	2198,33	2198,33	2198,33
975429	278171	2381,67	2381,67	2381,67	2381,67	2381,67	2381,67	2381,67
975459	278141	2310	2310	2310	2310	2310	2310	2310
975489	278111	2406,67	2406,67	2406,67	2406,67	2406,67	2406,67	2406,67
975519	278081	2383,33	2383,33	2383,33	2383,33	2383,33	2383,33	2383,33
975549	278051	2336,67	2336,67	2336,67	2336,67	2336,67	2336,67	2336,67
975579	278021	2350	2350	2350	2350	2350	2350	2350
975609	277991	2456,67	2456,67	2456,67	2456,67	2456,67	2456,67	2456,67
975639	277961	2406,67	2406,67	2406,67	2406,67	2406,67	2406,67	2406,67
975669	277931	2443,33	2443,33	2443,33	2443,33	2443,33	2443,33	2443,33
975699	277901	2341,67	2341,67	2341,67	2341,67	2341,67	2341,67	2341,67
975729	277871	2383,33	2383,33	2383,33	2383,33	2383,33	2383,33	2383,33
975759	277841	2373,33	2373,33	2373,33	2373,33	2373,33	2373,33	2373,33
975789	277811	2400	2400	2400	2400	2400	2400	2400
975819	277781	2311,67	2311,67	2311,67	2311,67	2311,67	2311,67	2311,67
975849	277751	2291,67	2291,67	2291,67	2291,67	2291,67	2291,67	2291,67
975879	277721	2436,67	2436,67	2436,67	2436,67	2436,67	2436,67	2436,67
975909	277691	2533,33	2533,33	2533,33	2533,33	2533,33	2533,33	2533,33
975939	277661	2460	2460	2460	2460	2460	2460	2460
975969	277631	2418,33	2418,33	2418,33	2418,33	2418,33	2418,33	2418,33
975999	277601	2558,33	2558,33	2558,33	2558,33	2558,33	2558,33	2558,33
976029	277571	2528,33	2528,33	2528,33	2528,33	2528,33	2528,33	2528,33
976059	277541	2396,67	2396,67	2396,67	2396,67	2396,67	2396,67	2396,67
976089	277511	2576,67	2576,67	2576,67	2576,67	2576,67	2576,67	2576,67
976119	277481	2431,67	2431,67	2431,67	2431,67	2431,67	2431,67	2431,67
976149	277451	2458,33	2458,33	2458,33	2458,33	2458,33	2458,33	2458,33
976179	277421	2430	2430	2430	2430	2430	2430	2430
976209	277391	2496,67	2496,67	2496,67	2496,67	2496,67	2496,67	2496,67
976239	277361	2451,67	2451,67	2451,67	2451,67	2451,67	2451,67	2451,67
976269	277331	2470	2470	2470	2470	2470	2470	2470
976299	277301	2473,33	2473,33	2473,33	2473,33	2473,33	2473,33	2473,33
976329	277271	2423,33	2423,33	2423,33	2423,33	2423,33	2423,33	2423,33
976359	277241	2421,67	2421,67	2421,67	2421,67	2421,67	2421,67	2421,67
976389	277211	2493,33	2493,33	2493,33	2493,33	2493,33	2493,33	2493,33
976419	277181	2456,67	2456,67	2456,67	2456,67	2456,67	2456,67	2456,67
976449	277151	2335	2335	2335	2335	2335	2335	2335
976479	277121	2543,33	2543,33	2543,33	2543,33	2543,33	2543,33	2543,33
976509	277091	2543,33	2543,33	2543,33	2543,33	2543,33	2543,33	2543,33
976539	277061	2496,67	2496,67	2496,67	2496,67	2496,67	2496,67	2496,67
976569	277031	2423,33	2423,33	2423,33	2423,33	2423,33	2423,33	2423,33
976599	277001	2333,33	2333,33	2333,33	2333,33	2333,33	2333,33	2333,33
976629	276971	2595	2595	2595	2595	2595	2595	2595
976659	276941	2631,67	2631,67	2631,67	2631,67	2631,67	2631,67	2631,67

This journal is © The Royal Society of Chemistry 20xx

Please do not adjust margins

976689	276911	2493,33	2493,33	2493,33	2493,33	2493,33	2493,33	2493,33
976719	276881	2480	2480	2480	2480	2480	2480	2480
976749	276851	2586,67	2586,67	2586,67	2586,67	2586,67	2586,67	2586,67
976779	276821	2545	2545	2545	2545	2545	2545	2545
976809	276791	2486,67	2486,67	2486,67	2486,67	2486,67	2486,67	2486,67
976839	276761	2565	2565	2565	2565	2565	2565	2565
976869	276731	2448,33	2448,33	2448,33	2448,33	2448,33	2448,33	2448,33
976899	276701	2598,33	2598,33	2598,33	2598,33	2598,33	2598,33	2598,33
976929	276671	2496,67	2496,67	2496,67	2496,67	2496,67	2496,67	2496,67
976959	276641	2425	2425	2425	2425	2425	2425	2425
976989	276611	2410	2410	2410	2410	2410	2410	2410
977019	276581	2546,67	2546,67	2546,67	2546,67	2546,67	2546,67	2546,67
977049	276551	2443,33	2443,33	2443,33	2443,33	2443,33	2443,33	2443,33
977079	276521	2405	2405	2405	2405	2405	2405	2405
977109	276491	2590	2590	2590	2590	2590	2590	2590
977139	276461	2528,33	2528,33	2528,33	2528,33	2528,33	2528,33	2528,33
977169	276431	2536,67	2536,67	2536,67	2536,67	2536,67	2536,67	2536,67
977199	276401	2561,67	2561,67	2561,67	2561,67	2561,67	2561,67	2561,67
977229	276371	2653,33	2653,33	2653,33	2653,33	2653,33	2653,33	2653,33
977259	276341	2618,33	2618,33	2618,33	2618,33	2618,33	2618,33	2618,33
977289	276311	2470	2470	2470	2470	2470	2470	2470
977319	276281	2501,67	2501,67	2501,67	2501,67	2501,67	2501,67	2501,67
977349	276251	2493,33	2493,33	2493,33	2493,33	2493,33	2493,33	2493,33
977379	276221	2546,67	2546,67	2546,67	2546,67	2546,67	2546,67	2546,67
977409	276191	2586,67	2586,67	2586,67	2586,67	2586,67	2586,67	2586,67
977439	276161	2601,67	2601,67	2601,67	2601,67	2601,67	2601,67	2601,67
977469	276131	2495	2495	2495	2495	2495	2495	2495
977499	276101	2556,67	2556,67	2556,67	2556,67	2556,67	2556,67	2556,67
977529	276071	2598,33	2598,33	2598,33	2598,33	2598,33	2598,33	2598,33
977559	276041	2506,67	2506,67	2506,67	2506,67	2506,67	2506,67	2506,67
977589	276011	2558,33	2558,33	2558,33	2558,33	2558,33	2558,33	2558,33
977619	275981	2503,33	2503,33	2503,33	2503,33	2503,33	2503,33	2503,33
977649	275951	2595	2595	2595	2595	2595	2595	2595
977679	275921	2628,33	2628,33	2628,33	2628,33	2628,33	2628,33	2628,33
977709	275891	2555	2555	2555	2555	2555	2555	2555
977739	275861	2460	2460	2460	2460	2460	2460	2460
977769	275831	2586,67	2586,67	2586,67	2586,67	2586,67	2586,67	2586,67
977799	275801	2633,33	2633,33	2633,33	2633,33	2633,33	2633,33	2633,33
977829	275771	2568,33	2568,33	2568,33	2568,33	2568,33	2568,33	2568,33
977859	275741	2530	2530	2530	2530	2530	2530	2530
977889	275711	2430	2430	2430	2430	2430	2430	2430
977919	275681	2443,33	2443,33	2443,33	2443,33	2443,33	2443,33	2443,33
977949	275651	2548,33	2548,33	2548,33	2548,33	2548,33	2548,33	2548,33
977979	275621	2541,67	2541,67	2541,67	2541,67	2541,67	2541,67	2541,67
978009	275591	2353,33	2353,33	2353,33	2353,33	2353,33	2353,33	2353,33
978039	275561	2418,33	2418,33	2418,33	2418,33	2418,33	2418,33	2418,33
978069	275531	2450	2450	2450	2450	2450	2450	2450

This journal is © The Royal Society of Chemistry 20xx

Please do not adjust margins

978099	275501	2525	2525	2525	2525	2525	2525	2525
978129	275471	2471,67	2471,67	2471,67	2471,67	2471,67	2471,67	2471,67
978159	275441	2453,33	2453,33	2453,33	2453,33	2453,33	2453,33	2453,33
978189	275411	2561,67	2561,67	2561,67	2561,67	2561,67	2561,67	2561,67
978219	275381	2420	2420	2420	2420	2420	2420	2420
978249	275351	2365	2365	2365	2365	2365	2365	2365
978279	275321	2363,33	2363,33	2363,33	2363,33	2363,33	2363,33	2363,33
978309	275291	2481,67	2481,67	2481,67	2481,67	2481,67	2481,67	2481,67
978339	275261	2278,33	2278,33	2278,33	2278,33	2278,33	2278,33	2278,33
978369	275231	2395	2395	2395	2395	2395	2395	2395
978399	275201	2373,33	2373,33	2373,33	2373,33	2373,33	2373,33	2373,33
978429	275171	2393,33	2393,33	2393,33	2393,33	2393,33	2393,33	2393,33
978459	275141	2475	2475	2475	2475	2475	2475	2475
978489	275111	2378,33	2378,33	2378,33	2378,33	2378,33	2378,33	2378,33
978519	275081	2465	2465	2465	2465	2465	2465	2465
978549	275051	2318,33	2318,33	2318,33	2318,33	2318,33	2318,33	2318,33
978579	275021	2425	2425	2425	2425	2425	2425	2425
978609	274991	2441,67	2441,67	2441,67	2441,67	2441,67	2441,67	2441,67
978639	274961	2435	2435	2435	2435	2435	2435	2435
978669	274931	2425	2425	2425	2425	2425	2425	2425
978699	274901	2410	2410	2410	2410	2410	2410	2410
978729	274871	2511,67	2511,67	2511,67	2511,67	2511,67	2511,67	2511,67
978759	274841	2315	2315	2315	2315	2315	2315	2315
978789	274811	2421,67	2421,67	2421,67	2421,67	2421,67	2421,67	2421,67
978819	274781	2336,67	2336,67	2336,67	2336,67	2336,67	2336,67	2336,67
978849	274751	2328,33	2328,33	2328,33	2328,33	2328,33	2328,33	2328,33
978879	274721	2465	2465	2465	2465	2465	2465	2465
978909	274691	2451,67	2451,67	2451,67	2451,67	2451,67	2451,67	2451,67
978939	274661	2275	2275	2275	2275	2275	2275	2275
978969	274631	2211,67	2211,67	2211,67	2211,67	2211,67	2211,67	2211,67
978999	274601	2340	2340	2340	2340	2340	2340	2340
979029	274571	2278,33	2278,33	2278,33	2278,33	2278,33	2278,33	2278,33
979059	274541	2346,67	2346,67	2346,67	2346,67	2346,67	2346,67	2346,67
979089	274511	2388,33	2388,33	2388,33	2388,33	2388,33	2388,33	2388,33
979119	274481	2363,33	2363,33	2363,33	2363,33	2363,33	2363,33	2363,33
979149	274451	2378,33	2378,33	2378,33	2378,33	2378,33	2378,33	2378,33
979179	274421	2298,33	2298,33	2298,33	2298,33	2298,33	2298,33	2298,33
979209	274391	2300	2300	2300	2300	2300	2300	2300
979239	274361	2346,67	2346,67	2346,67	2346,67	2346,67	2346,67	2346,67
979269	274331	2350	2350	2350	2350	2350	2350	2350
979299	274301	2225	2225	2225	2225	2225	2225	2225
979329	274271	2326,67	2326,67	2326,67	2326,67	2326,67	2326,67	2326,67
979359	274241	2270	2270	2270	2270	2270	2270	2270
979389	274211	2303,33	2303,33	2303,33	2303,33	2303,33	2303,33	2303,33
979419	274181	2338,33	2338,33	2338,33	2338,33	2338,33	2338,33	2338,33
979449	274151	2406,67	2406,67	2406,67	2406,67	2406,67	2406,67	2406,67
979479	274121	2246,67	2246,67	2246,67	2246,67	2246,67	2246,67	2246,67

This journal is © The Royal Society of Chemistry 20xx

Please do not adjust margins

979509	274091	2216,67	2216,67	2216,67	2216,67	2216,67	2216,67	2216,67
979539	274061	2330	2330	2330	2330	2330	2330	2330
979569	274031	2228,33	2228,33	2228,33	2228,33	2228,33	2228,33	2228,33
979599	274001	2338,33	2338,33	2338,33	2338,33	2338,33	2338,33	2338,33
979629	273971	2211,67	2211,67	2211,67	2211,67	2211,67	2211,67	2211,67
979659	273941	2181,67	2181,67	2181,67	2181,67	2181,67	2181,67	2181,67
979689	273911	2153,33	2153,33	2153,33	2153,33	2153,33	2153,33	2153,33
979719	273881	2100	2100	2100	2100	2100	2100	2100
979749	273851	2310	2310	2310	2310	2310	2310	2310
979779	273821	2145	2145	2145	2145	2145	2145	2145
979809	273791	2283,33	2283,33	2283,33	2283,33	2283,33	2283,33	2283,33
979839	273761	2191,67	2191,67	2191,67	2191,67	2191,67	2191,67	2191,67
979869	273731	2200	2200	2200	2200	2200	2200	2200
979899	273701	2281,67	2281,67	2281,67	2281,67	2281,67	2281,67	2281,67
979929	273671	2135	2135	2135	2135	2135	2135	2135
979959	273641	2231,67	2231,67	2231,67	2231,67	2231,67	2231,67	2231,67
979989	273611	2248,33	2248,33	2248,33	2248,33	2248,33	2248,33	2248,33
980019	273581	2188,33	2188,33	2188,33	2188,33	2188,33	2188,33	2188,33
980049	273551	2131,67	2131,67	2131,67	2131,67	2131,67	2131,67	2131,67
980079	273521	2181,67	2181,67	2181,67	2181,67	2181,67	2181,67	2181,67
980109	273491	2261,67	2261,67	2261,67	2261,67	2261,67	2261,67	2261,67
980139	273461	2268,33	2268,33	2268,33	2268,33	2268,33	2268,33	2268,33
980169	273431	2101,67	2101,67	2101,67	2101,67	2101,67	2101,67	2101,67
980199	273401	2201,67	2201,67	2201,67	2201,67	2201,67	2201,67	2201,67
980229	273371	2250	2250	2250	2250	2250	2250	2250
980259	273341	2218,33	2218,33	2218,33	2218,33	2218,33	2218,33	2218,33
980289	273311	2203,33	2203,33	2203,33	2203,33	2203,33	2203,33	2203,33
980319	273281	2233,33	2233,33	2233,33	2233,33	2233,33	2233,33	2233,33
980349	273251	2095	2095	2095	2095	2095	2095	2095
980379	273221	2211,67	2211,67	2211,67	2211,67	2211,67	2211,67	2211,67
980409	273191	2140	2140	2140	2140	2140	2140	2140
980439	273161	2161,67	2161,67	2161,67	2161,67	2161,67	2161,67	2161,67
980469	273131	2068,33	2068,33	2068,33	2068,33	2068,33	2068,33	2068,33
980499	273101	2118,33	2118,33	2118,33	2118,33	2118,33	2118,33	2118,33
980529	273071	2138,33	2138,33	2138,33	2138,33	2138,33	2138,33	2138,33
980559	273041	2191,67	2191,67	2191,67	2191,67	2191,67	2191,67	2191,67
980589	273011	2173,33	2173,33	2173,33	2173,33	2173,33	2173,33	2173,33
980619	272981	2141,67	2141,67	2141,67	2141,67	2141,67	2141,67	2141,67
980649	272951	2096,67	2096,67	2096,67	2096,67	2096,67	2096,67	2096,67
980679	272921	2128,33	2128,33	2128,33	2128,33	2128,33	2128,33	2128,33
980709	272891	2165	2165	2165	2165	2165	2165	2165
980739	272861	2173,33	2173,33	2173,33	2173,33	2173,33	2173,33	2173,33
980769	272831	2163,33	2163,33	2163,33	2163,33	2163,33	2163,33	2163,33
980799	272801	2180	2180	2180	2180	2180	2180	2180
980829	272771	2203,33	2203,33	2203,33	2203,33	2203,33	2203,33	2203,33
980859	272741	2141,67	2141,67	2141,67	2141,67	2141,67	2141,67	2141,67
980889	272711	2175	2175	2175	2175	2175	2175	2175

This journal is © The Royal Society of Chemistry 20xx

980919	272681	2046,67	2046,67	2046,67	2046,67	2046,67	2046,67	2046,67
980949	272651	2138,33	2138,33	2138,33	2138,33	2138,33	2138,33	2138,33
980979	272621	2208,33	2208,33	2208,33	2208,33	2208,33	2208,33	2208,33
981009	272591	2091,67	2091,67	2091,67	2091,67	2091,67	2091,67	2091,67
981039	272561	2228,33	2228,33	2228,33	2228,33	2228,33	2228,33	2228,33
981069	272531	2005	2005	2005	2005	2005	2005	2005
981099	272501	2015	2015	2015	2015	2015	2015	2015
981129	272471	2053,33	2053,33	2053,33	2053,33	2053,33	2053,33	2053,33
981159	272441	2018,33	2018,33	2018,33	2018,33	2018,33	2018,33	2018,33
981189	272411	2041,67	2041,67	2041,67	2041,67	2041,67	2041,67	2041,67
981219	272381	2200	2200	2200	2200	2200	2200	2200
981249	272351	2056,67	2056,67	2056,67	2056,67	2056,67	2056,67	2056,67
981279	272321	2013,33	2013,33	2013,33	2013,33	2013,33	2013,33	2013,33
981309	272291	2101,67	2101,67	2101,67	2101,67	2101,67	2101,67	2101,67
981339	272261	2113,33	2113,33	2113,33	2113,33	2113,33	2113,33	2113,33
981369	272231	2100	2100	2100	2100	2100	2100	2100
981399	272201	1990	1990	1990	1990	1990	1990	1990
981429	272171	2085	2085	2085	2085	2085	2085	2085
981459	272141	2083,33	2083,33	2083,33	2083,33	2083,33	2083,33	2083,33
981489	272111	2106,67	2106,67	2106,67	2106,67	2106,67	2106,67	2106,67
981519	272081	2128,33	2128,33	2128,33	2128,33	2128,33	2128,33	2128,33
981549	272051	2008,33	2008,33	2008,33	2008,33	2008,33	2008,33	2008,33
981579	272021	2070	2070	2070	2070	2070	2070	2070
981609	271991	2035	2035	2035	2035	2035	2035	2035
981639	271961	2091,67	2091,67	2091,67	2091,67	2091,67	2091,67	2091,67
981669	271931	2088,33	2088,33	2088,33	2088,33	2088,33	2088,33	2088,33
981699	271901	2081,67	2081,67	2081,67	2081,67	2081,67	2081,67	2081,67
981729	271871	2045	2045	2045	2045	2045	2045	2045
981759	271841	2108,33	2108,33	2108,33	2108,33	2108,33	2108,33	2108,33
981789	271811	2140	2140	2140	2140	2140	2140	2140
981819	271781	2045	2045	2045	2045	2045	2045	2045
981849	271751	1991,67	1991,67	1991,67	1991,67	1991,67	1991,67	1991,67
981879	271721	2210	2210	2210	2210	2210	2210	2210
981909	271691	1956,67	1956,67	1956,67	1956,67	1956,67	1956,67	1956,67
981939	271661	1973,33	1973,33	1973,33	1973,33	1973,33	1973,33	1973,33
981969	271631	2198,33	2198,33	2198,33	2198,33	2198,33	2198,33	2198,33
981999	271601	2148,33	2148,33	2148,33	2148,33	2148,33	2148,33	2148,33
982029	271571	2068,33	2068,33	2068,33	2068,33	2068,33	2068,33	2068,33
982059	271541	2116,67	2116,67	2116,67	2116,67	2116,67	2116,67	2116,67
982089	271511	2046,67	2046,67	2046,67	2046,67	2046,67	2046,67	2046,67
982119	271481	2033,33	2033,33	2033,33	2033,33	2033,33	2033,33	2033,33
982149	271451	1996,67	1996,67	1996,67	1996,67	1996,67	1996,67	1996,67
982179	271421	1971,67	1971,67	1971,67	1971,67	1971,67	1971,67	1971,67
982209	271391	2145	2145	2145	2145	2145	2145	2145
982239	271361	2000	2000	2000	2000	2000	2000	2000
982269	271331	2053,33	2053,33	2053,33	2053,33	2053,33	2053,33	2053,33
982299	271301	2080	2080	2080	2080	2080	2080	2080

Please do not adjust margins

982329	271271	2060	2060	2060	2060	2060	2060	2060
982359	271241	2153,33	2153,33	2153,33	2153,33	2153,33	2153,33	2153,33
982389	271211	2031,67	2031,67	2031,67	2031,67	2031,67	2031,67	2031,67
982419	271181	2003,33	2003,33	2003,33	2003,33	2003,33	2003,33	2003,33
982449	271151	2050	2050	2050	2050	2050	2050	2050
982479	271121	2106,67	2106,67	2106,67	2106,67	2106,67	2106,67	2106,67
982509	271091	2076,67	2076,67	2076,67	2076,67	2076,67	2076,67	2076,67
982539	271061	2070	2070	2070	2070	2070	2070	2070
982569	271031	2035	2035	2035	2035	2035	2035	2035
982599	271001	2143,33	2143,33	2143,33	2143,33	2143,33	2143,33	2143,33
982629	270971	2080	2080	2080	2080	2080	2080	2080
982659	270941	2090	2090	2090	2090	2090	2090	2090
982689	270911	2153,33	2153,33	2153,33	2153,33	2153,33	2153,33	2153,33
982719	270881	2095	2095	2095	2095	2095	2095	2095
982749	270851	2028,33	2028,33	2028,33	2028,33	2028,33	2028,33	2028,33
982779	270821	2168,33	2168,33	2168,33	2168,33	2168,33	2168,33	2168,33
982809	270791	2080	2080	2080	2080	2080	2080	2080
982839	270761	1983,33	1983,33	1983,33	1983,33	1983,33	1983,33	1983,33
982869	270731	2160	2160	2160	2160	2160	2160	2160

Table S4 Raw XPS measurements corresponding to Fig. 2.b.

KE_MgKa	BE_MgKa	CPS_MgKa	C 1s_1_MgKa	C 1s_2_MgKa	C 1s_3_MgKa	C 1s_4_MgKa	MgKa C1s-10_5_MgKa	Background_MgKa	Envelope_MgKa
952896	300704	2508,33	2508,33	2508,33	2508,33	2508,33	2508,33	2508,33	2508,33
952926	300674	2498,33	2498,33	2498,33	2498,33	2498,33	2498,33	2498,33	2498,33
952956	300644	2566,67	2566,67	2566,67	2566,67	2566,67	2566,67	2566,67	2566,67
952986	300614	2428,33	2428,33	2428,33	2428,33	2428,33	2428,33	2428,33	2428,33
953016	300584	2470	2470	2470	2470	2470	2470	2470	2470
953046	300554	2535	2535	2535	2535	2535	2535	2535	2535
953076	300524	2518,33	2518,33	2518,33	2518,33	2518,33	2518,33	2518,33	2518,33
953106	300494	2530	2530	2530	2530	2530	2530	2530	2530
953136	300464	2495	2495	2495	2495	2495	2495	2495	2495
953166	300434	2625	2625	2625	2625	2625	2625	2625	2625
953196	300404	2523,33	2523,33	2523,33	2523,33	2523,33	2523,33	2523,33	2523,33
953226	300374	2521,67	2521,67	2521,67	2521,67	2521,67	2521,67	2521,67	2521,67
953256	300344	2561,67	2561,67	2561,67	2561,67	2561,67	2561,67	2561,67	2561,67
953286	300314	2570	2570	2570	2570	2570	2570	2570	2570
953316	300284	2463,33	2463,33	2463,33	2463,33	2463,33	2463,33	2463,33	2463,33
953346	300254	2443,33	2443,33	2443,33	2443,33	2443,33	2443,33	2443,33	2443,33
953376	300224	2440	2440	2440	2440	2440	2440	2440	2440
953406	300194	2563,33	2563,33	2563,33	2563,33	2563,33	2563,33	2563,33	2563,33
953436	300164	2480	2480	2480	2480	2480	2480	2480	2480
953466	300134	2498,33	2498,33	2498,33	2498,33	2498,33	2498,33	2498,33	2498,33
953496	300104	2433,33	2433,33	2433,33	2433,33	2433,33	2433,33	2433,33	2433,33
953526	300074	2605	2605	2605	2605	2605	2605	2605	2605
953556	300044	2490	2490	2490	2490	2490	2490	2490	2490
953586	300014	2431,67	2431,67	2431,67	2431,67	2431,67	2431,67	2431,67	2431,67

This journal is © The Royal Society of Chemistry 20xx

Please do not adjust margins

953616	299984	2608,33	2608,33	2608,33	2608,33	2608,33	2608,33	2608,33	2608,33
953646	299954	2543,33	2543,33	2543,33	2543,33	2543,33	2543,33	2543,33	2543,33
953676	299924	2548,33	2548,33	2548,33	2548,33	2548,33	2548,33	2548,33	2548,33
953706	299894	2595	2595	2595	2595	2595	2595	2595	2595
953736	299864	2485	2485	2485	2485	2485	2485	2485	2485
953766	299834	2486,67	2486,67	2486,67	2486,67	2486,67	2486,67	2486,67	2486,67
953796	299804	2481,67	2481,67	2481,67	2481,67	2481,67	2481,67	2481,67	2481,67
953826	299774	2530	2530	2530	2530	2530	2530	2530	2530
953856	299744	2510	2510	2510	2510	2510	2510	2510	2510
953886	299714	2511,67	2511,67	2511,67	2511,67	2511,67	2511,67	2511,67	2511,67
953916	299684	2516,67	2516,67	2516,67	2516,67	2516,67	2516,67	2516,67	2516,67
953946	299654	2615	2615	2615	2615	2615	2615	2615	2615
953976	299624	2558,33	2558,33	2558,33	2558,33	2558,33	2558,33	2558,33	2558,33
954006	299594	2495	2495	2495	2495	2495	2495	2495	2495
954036	299564	2336,67	2336,67	2336,67	2336,67	2336,67	2336,67	2336,67	2336,67
954066	299534	2485	2485	2485	2485	2485	2485	2485	2485
954096	299504	2503,33	2503,33	2503,33	2503,33	2503,33	2503,33	2503,33	2503,33
954126	299474	2493,33	2493,33	2493,33	2493,33	2493,33	2493,33	2493,33	2493,33
954156	299444	2510	2510	2510	2510	2510	2510	2510	2510
954186	299414	2530	2530	2530	2530	2530	2530	2530	2530
954216	299384	2431,67	2431,67	2431,67	2431,67	2431,67	2431,67	2431,67	2431,67
954246	299354	2521,67	2521,67	2521,67	2521,67	2521,67	2521,67	2521,67	2521,67
954276	299324	2481,67	2481,67	2481,67	2481,67	2481,67	2481,67	2481,67	2481,67
954306	299294	2555	2555	2555	2555	2555	2555	2555	2555
954336	299264	2441,67	2441,67	2441,67	2441,67	2441,67	2441,67	2441,67	2441,67
954366	299234	2400	2400	2400	2400	2400	2400	2400	2400
954396	299204	2526,67	2526,67	2526,67	2526,67	2526,67	2526,67	2526,67	2526,67
954426	299174	2588,33	2588,33	2588,33	2588,33	2588,33	2588,33	2588,33	2588,33
954456	299144	2431,67	2431,67	2431,67	2431,67	2431,67	2431,67	2431,67	2431,67
954486	299114	2456,67	2456,67	2456,67	2456,67	2456,67	2456,67	2456,67	2456,67
954516	299084	2573,33	2573,33	2573,33	2573,33	2573,33	2573,33	2573,33	2573,33
954546	299054	2493,33	2493,33	2493,33	2493,33	2493,33	2493,33	2493,33	2493,33
954576	299024	2506,67	2506,67	2506,67	2506,67	2506,67	2506,67	2506,67	2506,67
954606	298994	2446,67	2446,67	2446,67	2446,67	2446,67	2446,67	2446,67	2446,67
954636	298964	2518,33	2518,33	2518,33	2518,33	2518,33	2518,33	2518,33	2518,33
954666	298934	2505	2505	2505	2505	2505	2505	2505	2505
954696	298904	2460	2460	2460	2460	2460	2460	2460	2460
954726	298874	2586,67	2586,67	2586,67	2586,67	2586,67	2586,67	2586,67	2586,67
954756	298844	2581,67	2581,67	2581,67	2581,67	2581,67	2581,67	2581,67	2581,67
954786	298814	2580	2580	2580	2580	2580	2580	2580	2580
954816	298784	2436,67	2436,67	2436,67	2436,67	2436,67	2436,67	2436,67	2436,67
954846	298754	2406,67	2406,67	2406,67	2406,67	2406,67	2406,67	2406,67	2406,67
954876	298724	2481,67	2481,67	2481,67	2481,67	2481,67	2481,67	2481,67	2481,67
954906	298694	2510	2510	2510	2510	2510	2510	2510	2510
954936	298664	2423,33	2423,33	2423,33	2423,33	2423,33	2423,33	2423,33	2423,33
954966	298634	2415	2415	2415	2415	2415	2415	2415	2415
954996	298604	2315	2315	2315	2315	2315	2315	2315	2315

This journal is © The Royal Society of Chemistry 20xx

Please do not adjust margins

Please do not adjust margins

955026	298574	2361,67	2361,67	2361,67	2361,67	2361,67	2361,67	2361,67	2361,67
955056	298544	2538,33	2538,33	2538,33	2538,33	2538,33	2538,33	2538,33	2538,33
955086	298514	2391,67	2391,67	2391,67	2391,67	2391,67	2391,67	2391,67	2391,67
955116	298484	2291,67	2291,67	2291,67	2291,67	2291,67	2291,67	2291,67	2291,67
955146	298454	2526,67	2526,67	2526,67	2526,67	2526,67	2526,67	2526,67	2526,67
955176	298424	2533,33	2533,33	2533,33	2533,33	2533,33	2533,33	2533,33	2533,33
955206	298394	2486,67	2486,67	2486,67	2486,67	2486,67	2486,67	2486,67	2486,67
955236	298364	2418,33	2418,33	2418,33	2418,33	2418,33	2418,33	2418,33	2418,33
955266	298334	2483,33	2483,33	2483,33	2483,33	2483,33	2483,33	2483,33	2483,33
955296	298304	2415	2415	2415	2415	2415	2415	2415	2415
955326	298274	2473,33	2473,33	2473,33	2473,33	2473,33	2473,33	2473,33	2473,33
955356	298244	2465	2465	2465	2465	2465	2465	2465	2465
955386	298214	2423,33	2423,33	2423,33	2423,33	2423,33	2423,33	2423,33	2423,33
955416	298184	2421,67	2421,67	2421,67	2421,67	2421,67	2421,67	2421,67	2421,67
955446	298154	2453,33	2453,33	2453,33	2453,33	2453,33	2453,33	2453,33	2453,33
955476	298124	2371,67	2371,67	2371,67	2371,67	2371,67	2371,67	2371,67	2371,67
955506	298094	2426,67	2426,67	2426,67	2426,67	2426,67	2426,67	2426,67	2426,67
955536	298064	2331,67	2331,67	2331,67	2331,67	2331,67	2331,67	2331,67	2331,67
955566	298034	2546,67	2546,67	2546,67	2546,67	2546,67	2546,67	2546,67	2546,67
955596	298004	2311,67	2311,67	2311,67	2311,67	2311,67	2311,67	2311,67	2311,67
955626	297974	2466,67	2466,67	2466,67	2466,67	2466,67	2466,67	2466,67	2466,67
955656	297944	2418,33	2418,33	2418,33	2418,33	2418,33	2418,33	2418,33	2418,33
955686	297914	2495	2495	2495	2495	2495	2495	2495	2495
955716	297884	2506,67	2506,67	2506,67	2506,67	2506,67	2506,67	2506,67	2506,67
955746	297854	2465	2465	2465	2465	2465	2465	2465	2465
955776	297824	2476,67	2476,67	2476,67	2476,67	2476,67	2476,67	2476,67	2476,67
955806	297794	2365	2365	2365	2365	2365	2365	2365	2365
955836	297764	2468,33	2468,33	2468,33	2468,33	2468,33	2468,33	2468,33	2468,33
955866	297734	2350	2350	2350	2350	2350	2350	2350	2350
955896	297704	2321,67	2321,67	2321,67	2321,67	2321,67	2321,67	2321,67	2321,67
955926	297674	2445	2445	2445	2445	2445	2445	2445	2445
955956	297644	2278,33	2278,33	2278,33	2278,33	2278,33	2278,33	2278,33	2278,33
955986	297614	2433,33	2433,33	2433,33	2433,33	2433,33	2433,33	2433,33	2433,33
956016	297584	2436,67	2436,67	2436,67	2436,67	2436,67	2436,67	2436,67	2436,67
956046	297554	2395	2395	2395	2395	2395	2395	2395	2395
956076	297524	2468,33	2468,33	2468,33	2468,33	2468,33	2468,33	2468,33	2468,33
956106	297494	2431,67	2431,67	2431,67	2431,67	2431,67	2431,67	2431,67	2431,67
956136	297464	2521,67	2521,67	2521,67	2521,67	2521,67	2521,67	2521,67	2521,67
956166	297434	2448,33	2448,33	2448,33	2448,33	2448,33	2448,33	2448,33	2448,33
956196	297404	2350	2350	2350	2350	2350	2350	2350	2350
956226	297374	2398,33	2398,33	2398,33	2398,33	2398,33	2398,33	2398,33	2398,33
956256	297344	2320	2320	2320	2320	2320	2320	2320	2320
956286	297314	2453,33	2453,33	2453,33	2453,33	2453,33	2453,33	2453,33	2453,33
956316	297284	2311,67	2311,67	2311,67	2311,67	2311,67	2311,67	2311,67	2311,67
956346	297254	2328,33	2328,33	2328,33	2328,33	2328,33	2328,33	2328,33	2328,33
956376	297224	2226,67	2226,67	2226,67	2226,67	2226,67	2226,67	2226,67	2226,67
956406	297194	2431,67	2431,67	2431,67	2431,67	2431,67	2431,67	2431,67	2431,67

This journal is © The Royal Society of Chemistry 20xx

Please do not adjust margins
Please do not adjust margins

956436	297164	2385	2385	2385	2385	2385	2385	2385	2385
956466	297134	2316,67	2316,67	2316,67	2316,67	2316,67	2316,67	2316,67	2316,67
956496	297104	2300	2300	2300	2300	2300	2300	2300	2300
956526	297074	2428,33	2428,33	2428,33	2428,33	2428,33	2428,33	2428,33	2428,33
956556	297044	2476,67	2476,67	2476,67	2476,67	2476,67	2476,67	2476,67	2476,67
956586	297014	2415	2415	2415	2415	2415	2415	2415	2415
956616	296984	2333,33	2333,33	2333,33	2333,33	2333,33	2333,33	2333,33	2333,33
956646	296954	2280	2280	2280	2280	2280	2280	2280	2280
956676	296924	2465	2465	2465	2465	2465	2465	2465	2465
956706	296894	2456,67	2456,67	2456,67	2456,67	2456,67	2456,67	2456,67	2456,67
956736	296864	2275	2275	2275	2275	2275	2275	2275	2275
956766	296834	2365	2365	2365	2365	2365	2365	2365	2365
956796	296804	2446,67	2446,67	2446,67	2446,67	2446,67	2446,67	2446,67	2446,67
956826	296774	2480	2480	2480	2480	2480	2480	2480	2480
956856	296744	2435	2435	2435	2435	2435	2435	2435	2435
956886	296714	2331,67	2331,67	2331,67	2331,67	2331,67	2331,67	2331,67	2331,67
956916	296684	2391,67	2391,67	2391,67	2391,67	2391,67	2391,67	2391,67	2391,67
956946	296654	2410	2410	2410	2410	2410	2410	2410	2410
956976	296624	2436,67	2436,67	2436,67	2436,67	2436,67	2436,67	2436,67	2436,67
957006	296594	2321,67	2321,67	2321,67	2321,67	2321,67	2321,67	2321,67	2321,67
957036	296564	2310	2310	2310	2310	2310	2310	2310	2310
957066	296534	2398,33	2398,33	2398,33	2398,33	2398,33	2398,33	2398,33	2398,33
957096	296504	2261,67	2261,67	2261,67	2261,67	2261,67	2261,67	2261,67	2261,67
957126	296474	2263,33	2263,33	2263,33	2263,33	2263,33	2263,33	2263,33	2263,33
957156	296444	2383,33	2383,33	2383,33	2383,33	2383,33	2383,34	2383,33	2383,34
957186	296414	2335	2335	2335	2335	2335	2335	2335	2335
957216	296384	2478,33	2478,33	2478,33	2478,33	2478,33	2478,34	2478,33	2478,34
957246	296354	2340	2340	2340	2340	2340	2340	2340	2340
957276	296324	2270	2270	2270	2270	2270	2270	2270	2270
957306	296294	2380	2380	2380	2380	2380	2380	2380	2380
957336	296264	2318,33	2318,33	2318,33	2318,33	2318,33	2318,34	2318,33	2318,34
957366	296234	2248,33	2248,33	2248,33	2248,33	2248,33	2248,34	2248,33	2248,34
957396	296204	2308,33	2308,33	2308,33	2308,33	2308,33	2308,34	2308,33	2308,34
957426	296174	2253,33	2253,33	2253,33	2253,33	2253,33	2253,34	2253,33	2253,34
957456	296144	2415	2415	2415	2415	2415	2415,01	2415	2415,01
957486	296114	2373,33	2373,33	2373,33	2373,33	2373,33	2373,34	2373,33	2373,34
957516	296084	2385	2385	2385	2385	2385	2385,01	2385	2385,01
957546	296054	2310	2310	2310	2310	2310	2310,01	2310	2310,01
957576	296024	2330	2330	2330	2330	2330	2330,01	2330	2330,01
957606	295994	2315	2315	2315	2315	2315	2315,01	2315	2315,01
957636	295964	2363,33	2363,33	2363,33	2363,33	2363,33	2363,35	2363,33	2363,35
957666	295934	2363,33	2363,33	2363,33	2363,33	2363,33	2363,35	2363,33	2363,35
957696	295904	2343,33	2343,33	2343,33	2343,33	2343,33	2343,35	2343,33	2343,35
957726	295874	2426,67	2426,67	2426,67	2426,67	2426,67	2426,69	2426,67	2426,69
957756	295844	2380	2380	2380	2380	2380	2380,02	2380	2380,02
957786	295814	2265	2265	2265	2265	2265	2265,03	2265	2265,03
957816	295784	2296,67	2296,67	2296,67	2296,67	2296,67	2296,7	2296,67	2296,7

957846	295754	2313,33	2313,33	2313,33	2313,33	2313,33	2313,37	2313,33	2313,37
957876	295724	2416,67	2416,67	2416,67	2416,67	2416,67	2416,7	2416,67	2416,7
957906	295694	2290	2290	2290	2290	2290	2290,04	2290	2290,04
957936	295664	2170	2170	2170	2170	2170	2170,05	2170	2170,05
957966	295634	2311,67	2311,67	2311,67	2311,67	2311,67	2311,72	2311,67	2311,72
957996	295604	2306,67	2306,67	2306,67	2306,67	2306,67	2306,73	2306,67	2306,73
958026	295574	2331,67	2331,67	2331,67	2331,67	2331,67	2331,73	2331,67	2331,73
958056	295544	2445	2445	2445	2445	2445	2445,08	2445	2445,08
958086	295514	2321,67	2321,67	2321,67	2321,67	2321,67	2321,75	2321,67	2321,75
958116	295484	2338,33	2338,33	2338,33	2338,33	2338,33	2338,43	2338,33	2338,43
958146	295454	2305	2305	2305	2305	2305	2305,1	2305	2305,1
958176	295424	2391,67	2391,67	2391,67	2391,67	2391,67	2391,78	2391,67	2391,78
958206	295394	2303,33	2303,33	2303,33	2303,33	2303,33	2303,46	2303,33	2303,46
958236	295364	2318,33	2318,33	2318,33	2318,33	2318,33	2318,48	2318,33	2318,48
958266	295334	2338,33	2338,33	2338,33	2338,33	2338,33	2338,49	2338,33	2338,49
958296	295304	2261,67	2261,67	2261,67	2261,67	2261,67	2261,85	2261,67	2261,85
958326	295274	2340	2340	2340	2340	2340	2340,2	2340	2340,2
958356	295244	2331,67	2331,67	2331,67	2331,67	2331,67	2331,89	2331,67	2331,89
958386	295214	2301,67	2301,67	2301,67	2301,67	2301,67	2301,91	2301,67	2301,91
958416	295184	2215	2215	2215	2215	2215	2215,27	2215	2215,27
958446	295154	2313,33	2313,33	2313,33	2313,33	2313,33	2313,64	2313,33	2313,64
958476	295124	2330	2330	2330	2330	2330	2330,33	2330	2330,33
958506	295094	2206,67	2206,67	2206,67	2206,67	2206,67	2207,04	2206,67	2207,04
958536	295064	2241,67	2241,67	2241,67	2241,67	2241,67	2242,08	2241,67	2242,08
958566	295034	2291,67	2291,67	2291,67	2291,67	2291,67	2292,12	2291,67	2292,12
958596	295004	2261,67	2261,67	2261,67	2261,67	2261,67	2262,17	2261,67	2262,17
958626	294974	2315	2315	2315	2315	2315	2315,55	2315	2315,55
958656	294944	2260	2260	2260	2260	2260	2260,61	2260	2260,61
958686	294914	2293,33	2293,33	2293,33	2293,33	2293,33	2294	2293,33	2294
958716	294884	2335	2335	2335	2335	2335	2335,73	2335	2335,73
958746	294854	2255	2255	2255	2255	2255	2255,81	2255	2255,81
958776	294824	2235	2235	2235	2235	2235	2235,89	2235	2235,89
958806	294794	2230	2230	2230	2230	2230	2230,98	2230	2230,98
958836	294764	2366,67	2366,67	2366,67	2366,67	2366,67	2367,74	2366,67	2367,74
958866	294734	2273,33	2273,33	2273,33	2273,33	2273,33	2274,51	2273,33	2274,51
958896	294704	2265	2265	2265	2265	2265	2266,29	2265	2266,29
958926	294674	2300	2300	2300	2300	2300	2301,41	2300	2301,41
958956	294644	2375	2375	2375	2375	2375	2376,54	2375	2376,54
958986	294614	2333,33	2333,33	2333,33	2333,33	2333,33	2335,02	2333,33	2335,02
959016	294584	2355	2355	2355	2355	2355	2356,84	2355	2356,84
959046	294554	2293,33	2293,33	2293,33	2293,33	2293,33	2295,34	2293,33	2295,34
959076	294524	2295	2295	2295	2295	2295	2297,19	2295	2297,19
959106	294494	2255	2255	2255	2255	2255	2257,39	2255	2257,39
959136	294464	2213,33	2213,33	2213,33	2213,33	2213,33	2215,93	2213,33	2215,93
959166	294434	2260	2260	2260	2260	2260	2262,83	2260	2262,83
959196	294404	2216,67	2216,67	2216,67	2216,67	2216,67	2219,74	2216,67	2219,74
959226	294374	2240	2240	2240	2240	2240	2243,35	2240	2243,35

This journal is © The Royal Society of Chemistry 20xx
Please do not adjust margins
Please do not adjust margins

959256	294344	2260	2260	2260	2260	2260	2263,63	2260	2263,63
959286	294314	2266,67	2266,67	2266,67	2266,67	2266,67	2270,6	2266,67	2270,6
959316	294284	2185	2185	2185	2185	2185	2189,27	2185	2189,27
959346	294254	2138,33	2138,33	2138,33	2138,33	2138,33	2142,96	2138,33	2142,96
959376	294224	2336,67	2336,67	2336,67	2336,67	2336,67	2341,68	2336,67	2341,68
959406	294194	2153,33	2215,44	2215,44	2215,44	2215,44	2220,86	2215,44	2220,86
959436	294164	2191,67	2215,43	2215,43	2215,43	2215,43	2221,28	2215,43	2221,28
959466	294134	2276,67	2215,39	2215,39	2215,39	2215,39	2221,72	2215,39	2221,72
959496	294104	2226,67	2215,38	2215,38	2215,38	2215,38	2222,21	2215,38	2222,21
959526	294074	2311,67	2215,33	2215,33	2215,33	2215,33	2222,69	2215,33	2222,69
959556	294044	2326,67	2215,26	2215,26	2215,26	2215,26	2223,19	2215,26	2223,19
959586	294014	2195	2215,25	2215,25	2215,25	2215,25	2223,79	2215,25	2223,79
959616	293984	2286,67	2215,2	2215,2	2215,2	2215,21	2224,4	2215,2	2224,4
959646	293954	2345	2215,13	2215,13	2215,13	2215,13	2225,01	2215,13	2225,01
959676	293924	2198,33	2215,12	2215,12	2215,12	2215,12	2225,74	2215,12	2225,74
959706	293894	2305	2215,06	2215,06	2215,06	2215,07	2226,45	2215,06	2226,45
959736	293864	2315	2215,01	2215,01	2215,01	2215,01	2227,23	2215,01	2227,23
959766	293834	2253,33	2214,98	2214,98	2214,98	2214,98	2228,07	2214,98	2228,07
959796	293804	2193,33	2214,97	2214,97	2214,97	2214,97	2228,99	2214,97	2229
959826	293774	2243,33	2214,95	2214,95	2214,95	2214,95	2229,96	2214,95	2229,96
959856	293744	2181,67	2214,93	2214,93	2214,93	2214,93	2230,97	2214,93	2230,97
959886	293714	2298,33	2214,88	2214,88	2214,88	2214,89	2232,02	2214,88	2232,02
959916	293684	2183,33	2214,87	2214,87	2214,87	2214,87	2233,14	2214,87	2233,14
959946	293654	2318,33	2214,8	2214,8	2214,8	2214,81	2234,31	2214,8	2234,31
959976	293624	2328,33	2214,74	2214,74	2214,74	2214,74	2235,51	2214,74	2235,51
960006	293594	2213,33	2214,74	2214,74	2214,74	2214,74	2236,86	2214,74	2236,87
960036	293564	2218,33	2214,73	2214,73	2214,73	2214,74	2238,27	2214,73	2238,28
960066	293534	2226,67	2214,73	2214,73	2214,73	2214,73	2239,74	2214,73	2239,74
960096	293504	2288,33	2214,68	2214,68	2214,68	2214,69	2241,26	2214,68	2241,26
960126	293474	2363,33	2214,59	2214,59	2214,59	2214,6	2242,77	2214,59	2242,78
960156	293444	2383,33	2214,49	2214,49	2214,49	2214,5	2244,39	2214,49	2244,4
960186	293414	2315	2214,44	2214,44	2214,44	2214,44	2246,1	2214,44	2246,1
960216	293384	2196,67	2214,42	2214,42	2214,42	2214,43	2247,94	2214,42	2247,95
960246	293354	2283,33	2214,38	2214,38	2214,38	2214,39	2249,83	2214,38	2249,84
960276	293324	2245	2214,37	2214,37	2214,37	2214,37	2251,8	2214,37	2251,81
960306	293294	2288,33	2214,32	2214,32	2214,32	2214,33	2253,86	2214,32	2253,86
960336	293264	2328,33	2214,25	2214,25	2214,25	2214,26	2255,93	2214,25	2255,94
960366	293234	2350	2214,17	2214,17	2214,17	2214,19	2258,1	2214,17	2258,12
960396	293204	2238,33	2214,16	2214,16	2214,16	2214,17	2260,4	2214,16	2260,41
960426	293174	2366,67	2214,07	2214,07	2214,07	2214,08	2262,7	2214,07	2262,72
960456	293144	2323,33	2214	2214	2214	2214,02	2265,12	2214	2265,13
960486	293114	2381,67	2213,91	2213,91	2213,91	2213,92	2267,54	2213,91	2267,56
960516	293084	2226,67	2213,9	2213,9	2213,9	2213,92	2270,18	2213,9	2270,2
960546	293054	2320	2213,83	2213,83	2213,83	2213,86	2272,79	2213,83	2272,82
960576	293024	2226,67	2213,83	2213,83	2213,83	2213,86	2275,56	2213,83	2275,59
960606	292994	2263,33	2213,8	2213,8	2213,8	2213,83	2278,35	2213,8	2278,38
960636	292964	2245	2213,78	2213,78	2213,78	2213,82	2281,22	2213,78	2281,26

This journal is © The Royal Society of Chemistry 20xx

960666	292934	2378,33	2213,68	2213,68	2213,68	2213,72	2284,08	2213,68	2284,12
960696	292904	2278,33	2213,64	2213,64	2213,64	2213,69	2287,03	2213,64	2287,08
960726	292874	2270	2213,61	2213,61	2213,61	2213,66	2290,07	2213,61	2290,12
960756	292844	2403,33	2213,5	2213,5	2213,5	2213,56	2293,05	2213,5	2293,11
960786	292814	2431,67	2213,37	2213,37	2213,37	2213,43	2296,07	2213,37	2296,14
960816	292784	2265	2213,34	2213,34	2213,34	2213,41	2299,21	2213,34	2299,29
960846	292754	2401,67	2213,23	2213,23	2213,23	2213,31	2302,3	2213,23	2302,39
960876	292724	2205	2213,22	2213,22	2213,22	2213,32	2305,53	2213,22	2305,62
960906	292694	2335	2213,15	2213,15	2213,15	2213,25	2308,69	2213,15	2308,8
960936	292664	2316,67	2213,09	2213,09	2213,09	2213,21	2311,87	2213,09	2311,99
960966	292634	2361,67	2213	2213	2213	2213,13	2315,03	2213	2315,16
960996	292604	2328,33	2212,93	2212,93	2212,93	2213,08	2318,19	2212,93	2318,34
961026	292574	2411,67	2212,81	2212,81	2212,81	2212,98	2321,28	2212,81	2321,45
961056	292544	2255	2212,79	2212,79	2212,79	2212,98	2324,45	2212,79	2324,64
961086	292514	2343,33	2212,71	2212,71	2212,71	2212,92	2327,5	2212,71	2327,71
961116	292484	2373,33	2212,62	2212,62	2212,62	2212,85	2330,52	2212,62	2330,75
961146	292454	2331,67	2212,55	2212,55	2212,55	2212,81	2333,47	2212,55	2333,73
961176	292424	2441,67	2212,41	2212,41	2212,41	2212,7	2336,31	2212,41	2336,6
961206	292394	2380	2212,31	2212,31	2212,31	2212,64	2339,1	2212,31	2339,43
961236	292364	2325	2212,24	2212,24	2212,25	2212,61	2341,82	2212,24	2342,19
961266	292334	2458,33	2212,1	2212,1	2212,1	2212,5	2344,41	2212,1	2344,82
961296	292304	2280	2212,06	2212,06	2212,06	2212,51	2346,93	2212,06	2347,39
961326	292274	2321,67	2211,99	2211,99	2212	2212,49	2349,37	2211,99	2349,88
961356	292244	2286,67	2211,95	2211,95	2211,95	2212,5	2351,65	2211,95	2352,21
961386	292214	2228,33	2211,94	2211,94	2211,94	2212,56	2353,86	2211,94	2354,48
961416	292184	2316,67	2211,88	2211,88	2211,88	2212,56	2355,87	2211,88	2356,57
961446	292154	2291,67	2211,83	2211,83	2211,84	2212,59	2357,71	2211,83	2358,48
961476	292124	2263,33	2211,8	2211,8	2211,81	2212,64	2359,48	2211,8	2360,34
961506	292094	2360	2211,71	2211,71	2211,72	2212,65	2360,91	2211,71	2361,86
961536	292064	2375	2211,61	2211,61	2211,63	2212,65	2362,25	2211,61	2363,3
961566	292034	2245	2211,59	2211,59	2211,61	2212,74	2363,43	2211,59	2364,59
961596	292004	2375	2211,5	2211,5	2211,51	2212,76	2364,36	2211,5	2365,64
961626	291974	2276,67	2211,46	2211,46	2211,48	2212,85	2365,19	2211,46	2366,6
961656	291944	2455	2211,31	2211,31	2211,34	2212,85	2365,65	2211,31	2367,21
961686	291914	2316,67	2211,25	2211,25	2211,28	2212,95	2366,09	2211,25	2367,81
961716	291884	2396,67	2211,14	2211,14	2211,17	2213,01	2366,16	2211,14	2368,06
961746	291854	2335	2211,07	2211,07	2211,1	2213,13	2366,15	2211,07	2368,25
961776	291824	2211,67	2211,07	2211,07	2211,1	2213,33	2365,98	2211,07	2368,28
961806	291794	2335	2210,99	2210,99	2211,03	2213,49	2365,53	2210,99	2368,06
961836	291764	2305	2210,94	2210,94	2210,98	2213,68	2364,95	2210,94	2367,74
961866	291734	2365	2210,85	2210,85	2210,9	2213,85	2364,05	2210,85	2367,1
961896	291704	2348,33	2210,77	2210,77	2210,82	2214,07	2363,06	2210,77	2366,42
961926	291674	2405	2210,65	2210,65	2210,72	2214,27	2361,75	2210,65	2365,44
961956	291644	2346,67	2210,57	2210,57	2210,64	2214,54	2360,36	2210,57	2364,4
961986	291614	2391,67	2210,46	2210,46	2210,54	2214,81	2358,74	2210,46	2363,17
962016	291584	2270	2210,43	2210,43	2210,52	2215,18	2357,01	2210,43	2361,86
962046	291554	2393,33	2210,32	2210,32	2210,42	2215,52	2355,1	2210,32	2360,4

This journal is © The Royal Society of Chemistry 20xx

Please do not adjust margins

962076	291524	2270	2210,28	2210,28	2210,4	2215,96	2353,01	2210,28	2358,8
962106	291494	2336,67	2210,21	2210,21	2210,34	2216,41	2350,81	2210,21	2357,15
962136	291464	2401,67	2210,1	2210,1	2210,24	2216,87	2348,38	2210,1	2355,29
962166	291434	2318,33	2210,03	2210,03	2210,2	2217,4	2345,89	2210,03	2353,43
962196	291404	2250	2210,01	2210,01	2210,19	2218,05	2343,33	2210,01	2351,56
962226	291374	2293,33	2209,96	2209,96	2210,17	2218,72	2340,6	2209,96	2349,57
962256	291344	2345	2209,88	2209,88	2210,11	2219,4	2337,79	2209,88	2347,54
962286	291314	2253,33	2209,85	2209,85	2210,11	2220,22	2334,87	2209,85	2345,5
962316	291284	2361,67	2209,76	2209,76	2210,05	2221,03	2331,86	2209,76	2343,42
962346	291254	2298,33	2209,71	2209,71	2210,04	2221,94	2328,78	2209,71	2341,34
962376	291224	2298,33	2209,66	2209,66	2210,02	2222,93	2325,66	2209,66	2339,29
962406	291194	2268,33	2209,62	2209,62	2210,03	2224,03	2322,5	2209,62	2337,31
962436	291164	2256,67	2209,59	2209,6	2210,05	2225,2	2319,3	2209,59	2335,36
962466	291134	2350	2209,51	2209,51	2210,01	2226,4	2316,03	2209,51	2333,41
962496	291104	2303,33	2209,46	2209,46	2210,02	2227,75	2312,74	2209,46	2331,59
962526	291074	2433,33	2209,32	2209,32	2209,95	2229,11	2309,37	2209,32	2329,78
962556	291044	2433,33	2209,19	2209,19	2209,88	2230,55	2305,99	2209,19	2328,04
962586	291014	2491,67	2209,02	2209,02	2209,79	2232,11	2302,58	2209,02	2326,44
962616	290984	2476,67	2208,86	2208,87	2209,72	2233,79	2299,19	2208,86	2324,97
962646	290954	2390	2208,76	2208,76	2209,7	2235,62	2295,88	2208,76	2323,69
962676	290924	2318,33	2208,69	2208,69	2209,74	2237,65	2292,61	2208,69	2322,62
962706	290894	2383,33	2208,59	2208,59	2209,75	2239,79	2289,36	2208,59	2321,73
962736	290864	2281,67	2208,54	2208,55	2209,83	2242,11	2286,2	2208,54	2321,06
962766	290834	2350	2208,46	2208,46	2209,88	2244,55	2283,04	2208,46	2320,56
962796	290804	2393,33	2208,35	2208,36	2209,92	2247,17	2279,91	2208,35	2320,3
962826	290774	2335	2208,28	2208,28	2210,01	2249,96	2276,85	2208,28	2320,27
962856	290744	2395	2208,17	2208,17	2210,08	2252,87	2273,84	2208,17	2320,46
962886	290714	2530	2207,98	2207,98	2210,09	2255,96	2270,78	2207,98	2320,89
962916	290684	2351,67	2207,89	2207,9	2210,22	2259,32	2267,92	2207,89	2321,69
962946	290654	2461,67	2207,74	2207,75	2210,3	2262,79	2265,05	2207,74	2322,67
962976	290624	2315	2207,68	2207,69	2210,49	2266,61	2262,33	2207,68	2324,1
963006	290594	2343,33	2207,6	2207,61	2210,7	2270,64	2259,69	2207,6	2325,85
963036	290564	2303,33	2207,54	2207,55	2210,95	2274,88	2257,11	2207,54	2327,87
963066	290534	2371,67	2207,44	2207,46	2211,18	2279,36	2254,61	2207,44	2330,28
963096	290504	2511,67	2207,26	2207,28	2211,37	2284,03	2252,07	2207,26	2332,96
963126	290474	2438,33	2207,12	2207,14	2211,63	2288,96	2249,67	2207,12	2336,04
963156	290444	2353,33	2207,04	2207,06	2211,97	2294,2	2247,4	2207,04	2339,51
963186	290414	2360	2206,95	2206,97	2212,35	2299,79	2245,18	2206,95	2343,45
963216	290384	2385	2206,84	2206,87	2212,76	2305,6	2243,06	2206,84	2347,76
963246	290354	2468,33	2206,69	2206,72	2213,15	2311,6	2240,92	2206,69	2352,33
963276	290324	2440	2206,55	2206,58	2213,6	2318,04	2238,93	2206,55	2357,51
963306	290294	2381,67	2206,44	2206,49	2214,16	2324,77	2237,01	2206,44	2363,1
963336	290264	2396,67	2206,33	2206,38	2214,75	2331,75	2235,18	2206,33	2369,06
963366	290234	2396,67	2206,22	2206,27	2215,39	2339,12	2233,41	2206,22	2375,53
963396	290204	2283,33	2206,17	2206,23	2216,17	2346,88	2231,77	2206,17	2382,54
963426	290174	2355	2206,08	2206,15	2216,98	2354,86	2230,2	2206,08	2389,94
963456	290144	2331,67	2206,01	2206,09	2217,85	2363,2	2228,66	2206,01	2397,77

This journal is © The Royal Society of Chemistry 20xx

963486	290114	2385	2205,9	2205,99	2218,79	2371,89	2227,2	2205,9	2406,17
963516	290084	2453,33	2205,76	2205,86	2219,77	2380,81	2225,75	2205,76	2414,91
963546	290054	2433,33	2205,62	2205,74	2220,83	2390,01	2224,37	2205,62	2424,08
963576	290024	2461,67	2205,47	2205,6	2221,96	2399,63	2223,05	2205,47	2433,83
963606	289994	2518,33	2205,29	2205,43	2223,19	2409,46	2221,73	2205,29	2443,95
963636	289964	2455	2205,14	2205,3	2224,53	2419,57	2220,54	2205,14	2454,53
963666	289934	2488,33	2204,97	2205,15	2225,95	2430,02	2219,36	2204,97	2465,58
963696	289904	2528,33	2204,78	2204,99	2227,51	2440,7	2218,23	2204,78	2477,09
963726	289874	2333,33	2204,7	2204,94	2229,29	2451,69	2217,25	2204,7	2489,06
963756	289844	2416,67	2204,58	2204,84	2231,12	2462,88	2216,28	2204,58	2501,39
963786	289814	2480	2204,41	2204,71	2233,1	2474,25	2215,33	2204,41	2514,15
963816	289784	2573,33	2204,19	2204,53	2235,16	2485,7	2214,34	2204,19	2527,15
963846	289754	2558,33	2203,98	2204,36	2237,36	2497,29	2213,44	2203,98	2540,5
963876	289724	2585	2203,76	2204,17	2239,73	2508,99	2212,54	2203,76	2554,16
963906	289694	2475	2203,6	2204,06	2242,36	2520,79	2211,77	2203,6	2568,19
963936	289664	2540	2203,4	2203,92	2245,11	2532,57	2210,98	2203,4	2582,39
963966	289634	2691,67	2203,11	2203,69	2247,94	2544,22	2210,14	2203,11	2596,66
963996	289604	2811,67	2202,75	2203,4	2250,97	2555,71	2209,27	2202,75	2611,1
964026	289574	2500	2202,57	2203,3	2254,36	2567,27	2208,6	2202,57	2625,82
964056	289544	2528,33	2202,38	2203,19	2257,91	2578,59	2207,97	2202,38	2640,53
964086	289514	2690	2202,09	2203	2261,69	2589,54	2207,25	2202,09	2655,21
964116	289484	2631,67	2201,84	2202,85	2265,72	2600,26	2206,61	2201,83	2669,93
964146	289454	2743,33	2201,51	2202,64	2269,89	2610,54	2205,92	2201,51	2684,45
964176	289424	2718,33	2201,21	2202,46	2274,4	2620,28	2205,27	2201,21	2698,79
964206	289394	2571,67	2200,99	2202,38	2279,29	2629,7	2204,74	2200,99	2713,14
964236	289364	2655	2200,72	2202,27	2284,36	2638,59	2204,17	2200,72	2727,23
964266	289334	2715	2200,42	2202,13	2289,71	2646,57	2203,59	2200,41	2740,76
964296	289304	2696,67	2200,12	2202,03	2295,45	2653,98	2203,04	2200,12	2754,15
964326	289274	2671,67	2199,84	2201,96	2301,47	2660,83	2202,53	2199,84	2767,26
964356	289244	2813,33	2199,48	2201,82	2307,69	2666,53	2201,95	2199,48	2779,55
964386	289214	2805	2199,12	2201,71	2314,39	2671,44	2201,38	2199,12	2791,57
964416	289184	2750	2198,79	2201,67	2321,41	2675,67	2200,87	2198,79	2803,25
964446	289154	2835	2198,42	2201,59	2328,68	2678,8	2200,32	2198,41	2814,15
964476	289124	2843,33	2198,04	2201,54	2336,43	2680,87	2199,78	2198,03	2824,53
964506	289094	2776,67	2197,69	2201,57	2344,58	2682,19	2199,28	2197,69	2834,57
964536	289064	2935	2197,26	2201,53	2352,95	2682,47	2198,71	2197,25	2843,91
964566	289034	2990	2196,79	2201,49	2361,73	2681,45	2198,11	2196,78	2852,45
964596	289004	2825	2196,42	2201,61	2371,06	2679,72	2197,62	2196,41	2860,79
964626	288974	2965	2195,96	2201,68	2380,62	2677,13	2197,06	2195,95	2868,63
964656	288944	2915	2195,54	2201,82	2390,6	2673,19	2196,54	2195,53	2875,58
964686	288914	2973,33	2195,08	2201,98	2401,06	2668,47	2195,99	2195,07	2882,31
964716	288884	2840	2194,7	2202,29	2411,92	2663,09	2195,52	2194,68	2888,79
964746	288854	2860	2194,31	2202,64	2423,09	2656,63	2195,05	2194,29	2894,56
964776	288824	3000	2193,83	2202,96	2434,73	2649,29	2194,51	2193,81	2900,07
964806	288794	2911,67	2193,41	2203,43	2446,72	2641,39	2194,01	2193,39	2905,42
964836	288764	2853,33	2193,02	2204	2459,05	2632,75	2193,57	2193	2910,4
964866	288734	2895	2192,61	2204,6	2471,78	2623,34	2193,1	2192,58	2915,11

This journal is © The Royal Society of Chemistry 20xx

Please do not adjust margins

964896	288704	2975	2192,15	2205,29	2484,78	2613,42	2192,59	2192,12	2919,76
964926	288674	2870	2191,75	2206,12	2498,09	2603,07	2192,14	2191,71	2924,32
964956	288644	2868,33	2191,36	2207,04	2511,7	2592,11	2191,7	2191,31	2928,65
964986	288614	2915	2190,94	2208,06	2525,56	2580,82	2191,23	2190,88	2933,07
965016	288584	2883,33	2190,54	2209,22	2539,6	2569,27	2190,79	2190,47	2937,51
965046	288554	2876,67	2190,14	2210,49	2553,79	2557,35	2190,35	2190,07	2941,86
965076	288524	2881,67	2189,74	2211,9	2568,15	2545,25	2189,91	2189,66	2946,33
965106	288494	3105	2189,21	2213,36	2582,41	2532,9	2189,34	2189,11	2950,76
965136	288464	2985	2188,75	2215,01	2596,76	2520,49	2188,85	2188,64	2955,3
965166	288434	2951,67	2188,32	2216,83	2611,08	2508,06	2188,38	2188,19	2959,91
965196	288404	3000	2187,86	2218,86	2625,24	2495,61	2187,88	2187,71	2964,62
965226	288374	2955	2187,42	2221,08	2639,3	2483,22	2187,4	2187,25	2969,42
965256	288344	2988,33	2186,97	2223,45	2653,07	2470,93	2186,91	2186,78	2974,21
965286	288314	3063,33	2186,48	2226,05	2666,42	2458,7	2186,38	2186,26	2978,99
965316	288284	2940	2186,06	2228,94	2679,52	2446,65	2185,92	2185,81	2983,85
965346	288254	3016,67	2185,61	2231,98	2692,14	2434,84	2185,42	2185,32	2988,71
965376	288224	3055	2185,13	2235,3	2704,02	2423,19	2184,89	2184,8	2993,32
965406	288194	3001,67	2184,7	2238,95	2715,41	2411,76	2184,4	2184,32	2997,93
965436	288164	3033,33	2184,25	2242,83	2726,22	2400,63	2183,89	2183,82	3002,54
965466	288134	2961,67	2183,84	2247,04	2735,96	2389,82	2183,42	2183,36	3006,66
965496	288104	2986,67	2183,44	2251,66	2744,96	2379,23	2182,94	2182,88	3010,7
965526	288074	2961,67	2183,05	2256,57	2753,23	2368,99	2182,47	2182,42	3014,63
965556	288044	3083,33	2182,6	2261,71	2760,2	2359,03	2181,93	2181,88	3017,93
965586	288014	3011,67	2182,21	2267,43	2766,15	2349,37	2181,43	2181,39	3021,02
965616	287984	2980	2181,84	2273,52	2771,22	2340,02	2180,95	2180,92	3023,88
965646	287954	3035	2181,46	2279,92	2775,05	2331,08	2180,44	2180,41	3026,3
965676	287924	3033,33	2181,09	2286,87	2777,49	2322,39	2179,94	2179,91	3028,15
965706	287894	2993,33	2180,77	2294,33	2778,97	2314	2179,45	2179,43	3029,81
965736	287864	3120	2180,38	2302,11	2779,29	2305,96	2178,89	2178,87	3031,17
965766	287834	2930	2180,14	2310,55	2778,07	2298,32	2178,44	2178,42	3031,83
965796	287804	3028,33	2179,85	2319,58	2775,8	2290,88	2177,94	2177,92	3032,37
965826	287774	3016,67	2179,6	2329,07	2772,55	2283,81	2177,44	2177,42	3032,78
965856	287744	3073,33	2179,34	2339,05	2767,68	2277,03	2176,9	2176,89	3032,45
965886	287714	3005	2179,16	2349,91	2761,83	2270,52	2176,41	2176,4	3032,24
965916	287684	2975	2179,03	2361,31	2755,09	2264,33	2175,94	2175,93	3031,99
965946	287654	3046,67	2178,89	2373,21	2747,07	2258,44	2175,42	2175,41	3031,38
965976	287624	3076,67	2178,78	2386	2737,96	2252,75	2174,88	2174,88	3030,88
966006	287594	3105	2178,71	2399,43	2728,09	2247,26	2174,33	2174,32	3030,52
966036	287564	2973,33	2178,76	2413,53	2717,41	2242,22	2173,86	2173,85	3030,38
966066	287534	3131,67	2178,77	2428,41	2705,65	2237,28	2173,29	2173,28	3030,26
966096	287504	2988,33	2178,94	2444,24	2693,41	2232,61	2172,8	2172,8	3030,8
966126	287474	3030	2179,16	2460,68	2680,6	2228,21	2172,3	2172,29	3031,78
966156	287444	2971,67	2179,48	2477,96	2667,02	2224,05	2171,82	2171,82	3033,06
966186	287414	3061,67	2179,83	2496,24	2653,01	2220,01	2171,29	2171,29	3035,23
966216	287384	2981,67	2180,33	2515,26	2638,72	2216,24	2170,81	2170,81	3038,13
966246	287354	3148,33	2180,84	2534,88	2623,89	2212,59	2170,23	2170,23	3041,52
966276	287324	2923,33	2181,59	2555,87	2608,97	2209,22	2169,79	2169,78	3046,3

This journal is © The Royal Society of Chemistry 20xx

966306	287294	3058,33	2182,36	2577,5	2593,82	2205,93	2169,26	2169,26	3051,85
966336	287264	3020	2183,31	2599,87	2578,54	2202,89	2168,75	2168,75	3058,35
966366	287234	3000	2184,43	2623,34	2563,23	2199,98	2168,26	2168,26	3066,21
966396	287204	3006,67	2185,7	2647,67	2547,93	2197,19	2167,76	2167,76	3075,2
966426	287174	3101,67	2187,06	2672,64	2532,6	2194,54	2167,21	2167,21	3085,21
966456	287144	3130	2188,6	2698,49	2517,42	2191,99	2166,64	2166,64	3096,58
966486	287114	3195	2190,35	2725,26	2502,33	2189,5	2166,03	2166,03	3109,35
966516	287084	3046,67	2192,39	2752,74	2487,48	2187,25	2165,51	2165,51	3123,34
966546	287054	3156,67	2194,58	2780,83	2472,89	2185,05	2164,92	2164,92	3138,59
966576	287024	3268,33	2196,96	2809,8	2458,47	2182,88	2164,27	2164,27	3155,31
966606	286994	3266,67	2199,69	2839,28	2444,3	2180,8	2163,61	2163,61	3173,24
966636	286964	3236,67	2202,73	2869,25	2430,52	2178,87	2162,98	2162,98	3192,44
966666	286934	3228,33	2206,07	2899,93	2417,11	2177,01	2162,35	2162,34	3213,09
966696	286904	3275	2209,71	2930,92	2403,97	2175,19	2161,69	2161,69	3234,74
966726	286874	3363,33	2213,79	2962,15	2391,15	2173,44	2160,97	2160,97	3257,61
966756	286844	3193,33	2218,37	2993,77	2378,91	2171,84	2160,36	2160,36	3281,81
966786	286814	3318,33	2223,29	3025,41	2366,91	2170,23	2159,67	2159,67	3306,81
966816	286784	3380	2228,6	3057	2355,21	2168,66	2158,95	2158,95	3332,62
966846	286754	3378,33	2234,54	3088,51	2344,07	2167,16	2158,23	2158,23	3359,6
966876	286724	3391,67	2241,04	3119,65	2333,26	2165,69	2157,5	2157,5	3387,15
966906	286694	3316,67	2248,13	3150,45	2322,81	2164,33	2156,81	2156,81	3415,3
966936	286664	3395	2255,75	3180,8	2312,82	2162,98	2156,08	2156,08	3444,13
966966	286634	3581,67	2264,03	3209,98	2303,11	2161,56	2155,23	2155,23	3472,98
966996	286604	3478,33	2273,18	3238,43	2293,77	2160,24	2154,45	2154,45	3502,27
967026	286574	3526,67	2283,01	3266,01	2284,82	2158,94	2153,63	2153,63	3531,88
967056	286544	3571,67	2293,56	3292,02	2276,28	2157,65	2152,79	2152,79	3561,12
967086	286514	3601,67	2305,03	3316,56	2268,01	2156,36	2151,93	2151,93	3590,15
967116	286484	3691,67	2317,54	3339,77	2260,01	2155,07	2151,02	2151,02	3619,33
967146	286454	3720	2330,96	3361,1	2252,46	2153,79	2150,09	2150,09	3648,04
967176	286424	3666,67	2345,38	3380,21	2245,21	2152,56	2149,19	2149,19	3675,79
967206	286394	3735	2360,83	3397,61	2238,19	2151,32	2148,25	2148,25	3703,19
967236	286364	3575	2377,88	3413,14	2231,69	2150,21	2147,41	2147,41	3730,69
967266	286334	3866,67	2395,91	3425,39	2225,28	2148,93	2146,39	2146,39	3756,34
967296	286304	3800	2415,18	3435,69	2219,15	2147,72	2145,41	2145,41	3781,51
967326	286274	3926,67	2435,64	3443,9	2213,27	2146,46	2144,35	2144,35	3806,21
967356	286244	3585	2458,22	3448,79	2207,89	2145,41	2143,5	2143,5	3829,81
967386	286214	3868,33	2482,08	3451,16	2202,54	2144,21	2142,48	2142,48	3852,56
967416	286184	3740	2507,49	3451,46	2197,52	2143,1	2141,53	2141,53	3874,99
967446	286154	3778,33	2534,41	3448,56	2192,77	2141,99	2140,56	2140,56	3896,06
967476	286124	3778,33	2563,5	3442,85	2188,2	2140,88	2139,59	2139,59	3916,66
967506	286094	3785	2594,48	3435,01	2183,8	2139,78	2138,61	2138,61	3937,23
967536	286064	3920	2627,13	3424,49	2179,62	2138,61	2137,56	2137,56	3957,18
967566	286034	4053,33	2661,51	3410,82	2175,51	2137,38	2136,42	2136,42	3975,96
967596	286004	4133,33	2698,22	3395,18	2171,5	2136,1	2135,24	2135,24	3995,3
967626	285974	4006,67	2737,36	3377,7	2167,8	2134,91	2134,13	2134,13	4015,38
967656	285944	3998,33	2778,53	3357,17	2164,26	2133,73	2133,02	2133,02	4034,61
967686	285914	3945	2821,8	3335,09	2160,88	2132,58	2131,95	2131,95	4054,5

This journal is © The Royal Society of Chemistry 20xx

Please do not adjust margins

967716	285884	4135	2867,43	3311,37	2157,55	2131,33	2130,76	2130,76	4075,4
967746	285854	4196,67	2915,84	3285,42	2154,34	2130,05	2129,54	2129,54	4097,05
967776	285824	4260	2966,45	3258,01	2151,2	2128,73	2128,27	2128,27	4119,57
967806	285794	4016,67	3019,44	3229,63	2148,33	2127,57	2127,15	2127,15	4143,51
967836	285764	4303,33	3074,71	3199,74	2145,44	2126,24	2125,86	2125,86	4168,53
967866	285734	4230	3133,18	3168,76	2142,68	2124,95	2124,62	2124,62	4195,72
967896	285704	4193,33	3193,96	3137,13	2140,03	2123,69	2123,39	2123,39	4224,64
967926	285674	4378,33	3256,91	3104,71	2137,4	2122,32	2122,05	2122,05	4255,19
967956	285644	4333,33	3322,14	3071,65	2134,88	2120,98	2120,74	2120,74	4287,43
967986	285614	4270	3390,58	3038,36	2132,47	2119,68	2119,47	2119,47	4322,68
968016	285584	4375	3461,09	3004,79	2130,09	2118,32	2118,13	2118,13	4359,91
968046	285554	4585	3533,53	2970,97	2127,67	2116,84	2116,67	2116,67	4399
968076	285524	4391,67	3608,04	2937,34	2125,42	2115,47	2115,32	2115,32	4440,3
968106	285494	4461,67	3684,95	2903,78	2123,19	2114,07	2113,93	2113,93	4484,2
968136	285464	4791,67	3763,44	2870,32	2120,85	2112,47	2112,34	2112,34	4530,04
968166	285434	4468,33	3843,55	2837,47	2118,74	2111,06	2110,95	2110,95	4577,97
968196	285404	4633,33	3924,83	2804,88	2116,58	2109,55	2109,45	2109,45	4627,49
968226	285374	4528,33	4007,46	2772,88	2114,55	2108,1	2108,02	2108,02	4678,94
968256	285344	4741,67	4090,75	2741,51	2112,43	2106,53	2106,46	2106,46	4731,85
968286	285314	4823,33	4174,33	2710,63	2110,3	2104,91	2104,85	2104,85	4785,63
968316	285284	4768,33	4258,05	2680,36	2108,25	2103,33	2103,27	2103,27	4840,19
968346	285254	4856,67	4341,43	2651,11	2106,19	2101,69	2101,64	2101,64	4895,52
968376	285224	5028,33	4423,73	2622,42	2104,05	2099,95	2099,9	2099,9	4950,44
968406	285194	4908,33	4504,87	2594,45	2102,02	2098,28	2098,24	2098,24	5004,9
968436	285164	5211,67	4584,27	2567,39	2099,84	2096,43	2096,39	2096,39	5058,76
968466	285134	5116,67	4661,67	2541,21	2097,74	2094,63	2094,6	2094,6	5111,45
968496	285104	5081,67	4735,54	2515,76	2095,68	2092,86	2092,83	2092,83	5161,35
968526	285074	5106,67	4806,25	2491,27	2093,64	2091,07	2091,04	2091,04	5209,1
968556	285044	5245	4873,37	2467,69	2091,53	2089,2	2089,17	2089,17	5254,26
968586	285014	5370	4936,56	2444,74	2089,36	2087,25	2087,23	2087,23	5296,22
968616	284984	5386,67	4993,32	2422,59	2087,21	2085,29	2085,27	2085,27	5332,6
968646	284954	5391,67	5044,92	2401,57	2085,07	2083,33	2083,31	2083,31	5364,94
968676	284924	5323,33	5091,18	2381,26	2082,98	2081,41	2081,39	2081,39	5392,65
968706	284894	5373,33	5131,76	2361,6	2080,88	2079,45	2079,44	2079,44	5415,37
968736	284864	5515	5164,08	2342,98	2078,71	2077,42	2077,41	2077,41	5430,98
968766	284834	5366,67	5189,32	2325,13	2076,63	2075,46	2075,46	2075,46	5440,18
968796	284804	5443,33	5207,85	2307,85	2074,52	2073,47	2073,46	2073,46	5443,32
968826	284774	5268,33	5219,71	2291,6	2072,52	2071,57	2071,57	2071,57	5440,7
968856	284744	5623,33	5222,68	2275,87	2070,32	2069,47	2069,46	2069,46	5429,97
968886	284714	5306,67	5217,44	2260,91	2068,32	2067,55	2067,54	2067,54	5411,59
968916	284684	5473,33	5205,16	2246,55	2066,22	2065,53	2065,52	2065,52	5386,9
968946	284654	5261,67	5186,13	2233,11	2064,26	2063,63	2063,63	2063,63	5356,25
968976	284624	5321,67	5159,23	2220,15	2062,26	2061,7	2061,7	2061,7	5318,25
969006	284594	5225	5124,03	2207,76	2060,33	2059,82	2059,82	2059,82	5272,48
969036	284564	5085	5082,76	2196,27	2058,49	2058,03	2058,03	2058,03	5221,47
969066	284534	4993,33	5035,66	2185,27	2056,7	2056,29	2056,29	2056,29	5165,06
969096	284504	5128,33	4982,48	2174,64	2054,84	2054,47	2054,47	2054,47	5103,02

Please do not adjust margins

Please do not adjust margins

969126	284474	5095	4922,31	2164,69	2053	2052,67	2052,67	2052,67	5034,67
969156	284444	5040	4857,66	2155,22	2051,19	2050,9	2050,89	2050,89	4962,28
969186	284414	4966,67	4788,88	2146,17	2049,43	2049,17	2049,17	2049,17	4886,15
969216	284384	5005	4716,24	2137,61	2047,65	2047,42	2047,41	2047,41	4806,67
969246	284354	4671,67	4639,22	2129,7	2046,07	2045,86	2045,86	2045,86	4723,27
969276	284324	4663,33	4559,59	2122,12	2044,5	2044,31	2044,31	2044,31	4637,59
969306	284294	4583,33	4477,8	2114,96	2042,97	2042,8	2042,8	2042,8	4550,13
969336	284264	4588,33	4394,16	2108,25	2041,44	2041,29	2041,29	2041,29	4461,27
969366	284234	4415	4308,84	2101,92	2040,02	2039,89	2039,89	2039,89	4371
969396	284204	4413,33	4222,67	2095,85	2038,6	2038,48	2038,48	2038,48	4280,16
969426	284174	4235	4136,2	2090,31	2037,28	2037,18	2037,18	2037,18	4189,44
969456	284144	4000	4049,72	2085,15	2036,11	2036,01	2036,01	2036,01	4098,96
969486	284114	4001,67	3963,54	2080,21	2034,93	2034,85	2034,85	2034,85	4008,99
969516	284084	3993,33	3878,15	2075,59	2033,76	2033,69	2033,69	2033,69	3920,13
969546	284054	3773,33	3793,83	2071,34	2032,72	2032,66	2032,66	2032,66	3832,58
969576	284024	3775	3710,62	2067,27	2031,68	2031,62	2031,62	2031,62	3746,32
969606	283994	3603,33	3629,06	2063,53	2030,74	2030,69	2030,69	2030,69	3661,95
969636	283964	3806,67	3549,45	2059,9	2029,69	2029,64	2029,64	2029,64	3579,76
969666	283934	3490	3471,81	2056,61	2028,81	2028,77	2028,77	2028,77	3499,69
969696	283904	3351,67	3396,13	2053,56	2028,03	2027,99	2027,99	2027,99	3421,74
969726	283874	3408,33	3322,54	2050,7	2027,2	2027,17	2027,17	2027,17	3346,09
969756	283844	3183,33	3252	2048,09	2026,51	2026,49	2026,49	2026,49	3273,64
969786	283814	3095	3183,72	2045,65	2025,88	2025,85	2025,85	2025,85	3203,55
969816	283784	3070	3117,68	2043,41	2025,26	2025,23	2025,23	2025,23	3135,88
969846	283754	3075	3053,9	2041,27	2024,63	2024,61	2024,61	2024,61	3070,58
969876	283724	2828,33	2993,53	2039,38	2024,15	2024,14	2024,14	2024,14	3008,79
969906	283694	3031,67	2935,4	2037,49	2023,55	2023,54	2023,54	2023,54	2949,36
969936	283664	2828,33	2879,66	2035,83	2023,07	2023,06	2023,06	2023,06	2892,44
969966	283634	2835	2826,19	2034,24	2022,59	2022,58	2022,58	2022,58	2837,86
969996	283604	2733,33	2775,77	2032,8	2022,17	2022,16	2022,16	2022,16	2786,42
970026	283574	2653,33	2727,84	2031,51	2021,79	2021,78	2021,78	2021,78	2737,57
970056	283544	2650	2682,05	2030,28	2021,42	2021,41	2021,41	2021,41	2690,92
970086	283514	2713,33	2638,33	2029,07	2021,01	2021	2021	2021	2646,4
970116	283484	2601,67	2597,24	2028,01	2020,66	2020,66	2020,66	2020,66	2604,6
970146	283454	2591,67	2558,52	2027,02	2020,32	2020,32	2020,32	2020,32	2565,22
970176	283424	2631,67	2521,69	2026,04	2019,96	2019,96	2019,96	2019,96	2527,78
970206	283394	2470	2486,81	2025,22	2019,69	2019,69	2019,69	2019,69	2492,35
970236	283364	2523,33	2453,97	2024,42	2019,4	2019,39	2019,39	2019,39	2459
970266	283334	2348,33	2423,45	2023,75	2019,2	2019,2	2019,2	2019,2	2428,01
970296	283304	2443,33	2394,5	2023,08	2018,95	2018,95	2018,95	2018,95	2398,63
970326	283274	2325	2367,19	2022,51	2018,77	2018,76	2018,76	2018,76	2370,94
970356	283244	2375	2341,47	2021,94	2018,55	2018,55	2018,55	2018,55	2344,86
970386	283214	2206,67	2317,83	2021,51	2018,44	2018,44	2018,44	2018,44	2320,9
970416	283184	2306,67	2295,48	2021,05	2018,27	2018,27	2018,27	2018,27	2298,26
970446	283154	2286,67	2274,44	2020,62	2018,11	2018,11	2018,11	2018,11	2276,95
970476	283124	2233,33	2254,67	2020,24	2017,98	2017,98	2017,98	2017,98	2256,93
970506	283094	2190	2236,6	2019,92	2017,88	2017,88	2017,88	2017,88	2238,64

 This journal is © The Royal Society of Chemistry 20xx

Please do not adjust margins

970536	283064	2116,67	2219,68	2019,67	2017,82	2017,82	2017,82	2017,82	2221,52
970566	283034	2146,67	2203,79	2019,4	2017,75	2017,75	2017,75	2017,75	2205,44
970596	283004	2278,33	2188,81	2019,09	2017,59	2017,59	2017,59	2017,59	2190,3
970626	282974	2241,67	2175,1	2018,8	2017,46	2017,46	2017,46	2017,46	2176,45
970656	282944	2185	2162,39	2018,57	2017,36	2017,36	2017,36	2017,36	2163,6
970686	282914	2161,67	2150,51	2018,36	2017,28	2017,27	2017,27	2017,27	2151,6
970716	282884	2221,67	2139,38	2018,13	2017,15	2017,15	2017,15	2017,15	2140,36
970746	282854	2196,67	2129,17	2017,92	2017,05	2017,05	2017,05	2017,05	2130,04
970776	282824	2206,67	2119,77	2017,72	2016,94	2016,93	2016,93	2016,93	2120,55
970806	282794	2178,33	2111,01	2017,54	2016,84	2016,84	2016,84	2016,84	2111,71
970836	282764	2108,33	2102,89	2017,41	2016,79	2016,78	2016,78	2016,78	2103,52
970866	282734	2133,33	2095,41	2017,28	2016,72	2016,72	2016,72	2016,72	2095,97
970896	282704	2163,33	2088,58	2017,13	2016,63	2016,63	2016,63	2016,63	2089,08
970926	282674	2200	2082,2	2016,97	2016,52	2016,52	2016,52	2016,52	2082,65
970956	282644	1930	2076,34	2016,87	2016,47	2016,47	2016,47	2016,47	2076,74
970986	282614	2228,33	2070,85	2016,7	2016,34	2016,34	2016,34	2016,34	2071,21
971016	282584	2110	2065,97	2016,61	2016,29	2016,29	2016,29	2016,29	2066,29
971046	282554	2045	2061,48	2016,55	2016,27	2016,27	2016,27	2016,27	2061,77
971076	282524	2170	2057,26	2016,43	2016,18	2016,18	2016,18	2016,18	2057,51
971106	282494	1931,67	2053,39	2016,35	2016,13	2016,13	2016,13	2016,13	2053,61
971136	282464	1991,67	2049,97	2016,31	2016,12	2016,12	2016,12	2016,12	2050,17
971166	282434	2180	2046,73	2016,19	2016,02	2016,02	2016,02	2016,02	2046,9
971196	282404	2123,33	2043,77	2016,11	2015,95	2015,95	2015,95	2015,95	2043,92
971226	282374	2038,33	2041,09	2016,08	2015,94	2015,94	2015,94	2015,94	2041,23
971256	282344	2055	2038,68	2016,04	2015,92	2015,92	2015,92	2015,92	2038,8
971286	282314	2105	2036,45	2015,97	2015,87	2015,87	2015,87	2015,87	2036,56
971316	282284	2063,33	2034,42	2015,93	2015,84	2015,84	2015,84	2015,84	2034,51
971346	282254	2071,67	2032,55	2015,89	2015,8	2015,8	2015,8	2015,8	2032,63
971376	282224	1950	2030,86	2015,84	2015,77	2015,77	2015,77	2015,77	2030,93
971406	282194	2025	2029,37	2015,83	2015,76	2015,76	2015,76	2015,76	2029,43
971436	282164	2048,33	2027,99	2015,8	2015,74	2015,74	2015,74	2015,74	2028,04
971466	282134	2086,67	2026,7	2015,75	2015,7	2015,7	2015,7	2015,7	2026,75
971496	282104	1963,33	2025,54	2015,71	2015,67	2015,67	2015,67	2015,67	2025,59
971526	282074	2118,33	2024,48	2015,65	2015,61	2015,61	2015,61	2015,61	2024,52
971556	282044	2000	2023,56	2015,63	2015,6	2015,6	2015,6	2015,6	2023,59
971586	282014	1988,33	2022,71	2015,61	2015,58	2015,58	2015,58	2015,58	2022,74
971616	281984	2013,33	2021,95	2015,61	2015,58	2015,58	2015,58	2015,58	2021,98
971646	281954	2035	2021,28	2015,59	2015,57	2015,57	2015,57	2015,57	2021,3
971676	281924	2023,33	2020,67	2015,58	2015,56	2015,56	2015,56	2015,56	2020,69
971706	281894	1903,33	2020,05	2015,52	2015,5	2015,5	2015,5	2015,5	2020,07
971736	281864	1916,67	2019,5	2015,45	2015,44	2015,44	2015,44	2015,44	2019,51
971766	281834	1976,67	2019,04	2015,43	2015,42	2015,42	2015,42	2015,42	2019,05
971796	281804	1965	2018,62	2015,4	2015,39	2015,39	2015,39	2015,39	2018,63
971826	281774	2008,33	2018,25	2015,39	2015,38	2015,38	2015,38	2015,38	2018,26
971856	281744	1950	2017,89	2015,35	2015,34	2015,34	2015,34	2015,34	2017,9
971886	281714	1910	2017,55	2015,29	2015,28	2015,28	2015,28	2015,28	2017,56
971916	281684	2036,67	2017,28	2015,27	2015,27	2015,27	2015,27	2015,27	2017,29

This journal is © The Royal Society of Chemistry 20xx

Please do not adjust margins

971946	281654	1983,33	2017,03	2015,26	2015,25	2015,25	2015,25	2015,25	2017,04
971976	281624	2096,67	2016,78	2015,21	2015,2	2015,2	2015,2	2015,2	2016,79
972006	281594	1946,67	2016,56	2015,16	2015,16	2015,16	2015,16	2015,16	2016,56
972036	281564	1983,33	2016,38	2015,15	2015,14	2015,14	2015,14	2015,14	2016,38
972066	281534	1953,33	2016,2	2015,11	2015,1	2015,1	2015,1	2015,1	2016,2
972096	281504	2041,67	2016,05	2015,09	2015,09	2015,09	2015,09	2015,09	2016,06
972126	281474	2061,67	2015,91	2015,06	2015,06	2015,06	2015,06	2015,06	2015,91
972156	281444	2048,33	2015,79	2015,04	2015,04	2015,04	2015,04	2015,04	2015,8
972186	281414	1935	2015,66	2015	2014,99	2014,99	2014,99	2014,99	2015,66
972216	281384	2098,33	2015,53	2014,95	2014,94	2014,94	2014,94	2014,94	2015,53
972246	281354	1966,67	2015,43	2014,92	2014,92	2014,92	2014,92	2014,92	2015,43
972276	281324	2060	2015,34	2014,89	2014,89	2014,89	2014,89	2014,89	2015,34
972306	281294	2021,67	2015,28	2014,89	2014,89	2014,89	2014,89	2014,89	2015,28
972336	281264	2010	2015,23	2014,88	2014,88	2014,88	2014,88	2014,88	2015,23
972366	281234	1988,33	2015,17	2014,87	2014,87	2014,87	2014,87	2014,87	2015,17
972396	281204	1971,67	2015,11	2014,84	2014,84	2014,84	2014,84	2014,84	2015,11
972426	281174	1975	2015,05	2014,82	2014,82	2014,82	2014,82	2014,82	2015,05
972456	281144	2036,67	2015,01	2014,81	2014,8	2014,8	2014,8	2014,8	2015,01
972486	281114	1865	2014,89	2014,72	2014,72	2014,72	2014,72	2014,72	2014,89
972516	281084	2005	2014,86	2014,71	2014,71	2014,71	2014,71	2014,71	2014,86
972546	281054	2078,33	2014,81	2014,67	2014,67	2014,67	2014,67	2014,67	2014,81
972576	281024	2078,33	2014,75	2014,64	2014,63	2014,63	2014,63	2014,63	2014,75
972606	280994	2045	2014,72	2014,62	2014,62	2014,62	2014,62	2014,62	2014,72
972636	280964	2081,67	2014,67	2014,58	2014,58	2014,58	2014,58	2014,58	2014,67
972666	280934	1938,33	2014,61	2014,53	2014,53	2014,53	2014,53	2014,53	2014,61
972696	280904	2016,67	2014,6	2014,53	2014,53	2014,53	2014,53	2014,53	2014,6
972726	280874	2000	2014,58	2014,52	2014,52	2014,52	2014,52	2014,52	2014,58
972756	280844	2018,33	2014,57	2014,52	2014,52	2014,52	2014,52	2014,52	2014,57
972786	280814	2073,33	2014,53	2014,49	2014,49	2014,49	2014,49	2014,49	2014,53
972816	280784	1968,33	2014,49	2014,46	2014,46	2014,46	2014,46	2014,46	2014,49
972846	280754	1925	2014,44	2014,4	2014,4	2014,4	2014,4	2014,4	2014,44
972876	280724	2160	2014,35	2014,32	2014,32	2014,32	2014,32	2014,32	2014,35
972906	280694	1981,67	2014,32	2014,3	2014,3	2014,3	2014,3	2014,3	2014,32
972936	280664	1996,67	2014,31	2014,29	2014,29	2014,29	2014,29	2014,29	2014,31
972966	280634	1990	2014,29	2014,27	2014,27	2014,27	2014,27	2014,27	2014,29
972996	280604	1960	2014,26	2014,24	2014,24	2014,24	2014,24	2014,24	2014,26
973026	280574	2026,67	2014,25	2014,23	2014,23	2014,23	2014,23	2014,23	2014,25
973056	280544	2163,33	2014,16	2014,15	2014,15	2014,15	2014,15	2014,15	2014,16
973086	280514	1940	2014,11	2014,1	2014,1	2014,1	2014,1	2014,1	2014,11
973116	280484	1980	2014,09	2014,08	2014,08	2014,08	2014,08	2014,08	2014,09
973146	280454	2023,33	2014,08	2014,08	2014,08	2014,08	2014,08	2014,08	2014,08
973176	280424	2036,67	2014,07	2014,06	2014,06	2014,06	2014,06	2014,06	2014,07
973206	280394	2003,33	2014,06	2014,06	2014,06	2014,06	2014,06	2014,06	2014,06
973236	280364	2090	2014,02	2014,01	2014,01	2014,01	2014,01	2014,01	2014,02
973266	280334	2006,67	2014,01	2014,01	2014,01	2014,01	2014,01	2014,01	2014,01
973296	280304	2001,67	2014	2014	2014	2014	2014	2014	2014
973326	280274	1988,33	2013,99	2013,99	2013,99	2013,99	2013,99	2013,99	2013,99

This journal is © The Royal Society of Chemistry 20xx

Please do not adjust margins

Please do not adjust margins

973356	280244	2045	2013,97	2013,97	2013,97	2013,97	2013,97	2013,97	2013,97
973386	280214	1998,33	2013,96	2013,96	2013,96	2013,96	2013,96	2013,96	2013,96
973416	280184	2008,33	2013,96	2013,95	2013,95	2013,95	2013,95	2013,95	2013,96
973446	280154	2070	2013,92	2013,92	2013,92	2013,92	2013,92	2013,92	2013,92
973476	280124	2036,67	2013,91	2013,91	2013,91	2013,91	2013,91	2013,91	2013,91
973506	280094	1988,33	2013,89	2013,89	2013,89	2013,89	2013,89	2013,89	2013,89
973536	280064	2056,67	2013,87	2013,87	2013,87	2013,87	2013,87	2013,87	2013,87
973566	280034	2103,33	2013,81	2013,81	2013,81	2013,81	2013,81	2013,81	2013,81
973596	280004	2041,67	2013,8	2013,8	2013,8	2013,8	2013,8	2013,8	2013,8
973626	279974	1933,33	2013,75	2013,75	2013,75	2013,75	2013,75	2013,75	2013,75
973656	279944	1996,67	2013,74	2013,74	2013,74	2013,74	2013,74	2013,74	2013,74
973686	279914	2006,67	2013,74	2013,74	2013,74	2013,74	2013,74	2013,74	2013,74
973716	279884	1988,33	2013,72	2013,72	2013,72	2013,72	2013,72	2013,72	2013,72
973746	279854	2013,33	2013,72	2013,72	2013,72	2013,72	2013,72	2013,72	2013,72
973776	279824	2020	2013,72	2013,72	2013,72	2013,72	2013,72	2013,72	2013,72
973806	279794	2051,67	2013,69	2013,69	2013,69	2013,69	2013,69	2013,69	2013,69
973836	279764	2026,67	2013,69	2013,69	2013,69	2013,69	2013,69	2013,69	2013,69
973866	279734	1958,33	2013,65	2013,65	2013,65	2013,65	2013,65	2013,65	2013,65
973896	279704	1976,67	2013,63	2013,63	2013,63	2013,63	2013,63	2013,63	2013,63
973926	279674	1911,67	2013,57	2013,57	2013,57	2013,57	2013,57	2013,57	2013,57
973956	279644	2061,67	2013,54	2013,54	2013,54	2013,54	2013,54	2013,54	2013,54
973986	279614	1976,67	2013,52	2013,52	2013,52	2013,52	2013,52	2013,52	2013,52
974016	279584	2100	2013,47	2013,47	2013,47	2013,47	2013,47	2013,47	2013,47
974046	279554	2011,67	2013,47	2013,47	2013,47	2013,47	2013,47	2013,47	2013,47
974076	279524	1958,33	2013,44	2013,44	2013,44	2013,44	2013,44	2013,44	2013,44
974106	279494	2023,33	2013,43	2013,43	2013,43	2013,43	2013,43	2013,43	2013,43
974136	279464	2008,33	2013,43	2013,43	2013,43	2013,43	2013,43	2013,43	2013,43
974166	279434	2045	2013,41	2013,41	2013,41	2013,41	2013,41	2013,41	2013,41
974196	279404	2148,33	2013,33	2013,33	2013,33	2013,33	2013,33	2013,33	2013,33
974226	279374	2035	2013,32	2013,32	2013,32	2013,32	2013,32	2013,32	2013,32
974256	279344	2118,33	2013,25	2013,25	2013,25	2013,25	2013,25	2013,25	2013,25
974286	279314	2025	2013,25	2013,25	2013,25	2013,25	2013,25	2013,25	2013,25
974316	279284	1921,67	2013,19	2013,19	2013,19	2013,19	2013,19	2013,19	2013,19
974346	279254	2021,67	2013,19	2013,19	2013,19	2013,19	2013,19	2013,19	2013,19
974376	279224	1958,33	2013,15	2013,15	2013,15	2013,15	2013,15	2013,15	2013,15
974406	279194	2040	2013,14	2013,14	2013,14	2013,14	2013,14	2013,14	2013,14
974436	279164	2086,67	2013,1	2013,1	2013,1	2013,1	2013,1	2013,1	2013,1
974466	279134	2025	2025	2025	2025	2025	2025	2025	2025
974496	279104	2066,67	2066,67	2066,67	2066,67	2066,67	2066,67	2066,67	2066,67
974526	279074	1893,33	1893,33	1893,33	1893,33	1893,33	1893,33	1893,33	1893,33
974556	279044	1938,33	1938,33	1938,33	1938,33	1938,33	1938,33	1938,33	1938,33
974586	279014	2003,33	2003,33	2003,33	2003,33	2003,33	2003,33	2003,33	2003,33
974616	278984	2008,33	2008,33	2008,33	2008,33	2008,33	2008,33	2008,33	2008,33
974646	278954	2025	2025	2025	2025	2025	2025	2025	2025
974676	278924	2043,33	2043,33	2043,33	2043,33	2043,33	2043,33	2043,33	2043,33
974706	278894	1963,33	1963,33	1963,33	1963,33	1963,33	1963,33	1963,33	1963,33
974736	278864	2040	2040	2040	2040	2040	2040	2040	2040

This journal is © The Royal Society of Chemistry 20xx

Please do not adjust margins

974766	278834	2026,67	2026,67	2026,67	2026,67	2026,67	2026,67	2026,67	2026,67
974796	278804	1905	1905	1905	1905	1905	1905	1905	1905
974826	278774	1936,67	1936,67	1936,67	1936,67	1936,67	1936,67	1936,67	1936,67
974856	278744	2006,67	2006,67	2006,67	2006,67	2006,67	2006,67	2006,67	2006,67
974886	278714	2113,33	2113,33	2113,33	2113,33	2113,33	2113,33	2113,33	2113,33
974916	278684	2015	2015	2015	2015	2015	2015	2015	2015
974946	278654	2060	2060	2060	2060	2060	2060	2060	2060
974976	278624	2033,33	2033,33	2033,33	2033,33	2033,33	2033,33	2033,33	2033,33
975006	278594	2060	2060	2060	2060	2060	2060	2060	2060
975036	278564	1938,33	1938,33	1938,33	1938,33	1938,33	1938,33	1938,33	1938,33
975066	278534	1985	1985	1985	1985	1985	1985	1985	1985
975096	278504	1951,67	1951,67	1951,67	1951,67	1951,67	1951,67	1951,67	1951,67
975126	278474	2036,67	2036,67	2036,67	2036,67	2036,67	2036,67	2036,67	2036,67
975156	278444	2041,67	2041,67	2041,67	2041,67	2041,67	2041,67	2041,67	2041,67
975186	278414	2035	2035	2035	2035	2035	2035	2035	2035
975216	278384	2020	2020	2020	2020	2020	2020	2020	2020
975246	278354	2065	2065	2065	2065	2065	2065	2065	2065
975276	278324	2051,67	2051,67	2051,67	2051,67	2051,67	2051,67	2051,67	2051,67
975306	278294	2033,33	2033,33	2033,33	2033,33	2033,33	2033,33	2033,33	2033,33
975336	278264	2090	2090	2090	2090	2090	2090	2090	2090
975366	278234	2098,33	2098,33	2098,33	2098,33	2098,33	2098,33	2098,33	2098,33
975396	278204	1848,33	1848,33	1848,33	1848,33	1848,33	1848,33	1848,33	1848,33
975426	278174	2166,67	2166,67	2166,67	2166,67	2166,67	2166,67	2166,67	2166,67
975456	278144	2098,33	2098,33	2098,33	2098,33	2098,33	2098,33	2098,33	2098,33
975486	278114	2078,33	2078,33	2078,33	2078,33	2078,33	2078,33	2078,33	2078,33
975516	278084	2091,67	2091,67	2091,67	2091,67	2091,67	2091,67	2091,67	2091,67
975546	278054	1943,33	1943,33	1943,33	1943,33	1943,33	1943,33	1943,33	1943,33
975576	278024	2028,33	2028,33	2028,33	2028,33	2028,33	2028,33	2028,33	2028,33
975606	277994	2061,67	2061,67	2061,67	2061,67	2061,67	2061,67	2061,67	2061,67
975636	277964	2068,33	2068,33	2068,33	2068,33	2068,33	2068,33	2068,33	2068,33
975666	277934	2055	2055	2055	2055	2055	2055	2055	2055
975696	277904	1978,33	1978,33	1978,33	1978,33	1978,33	1978,33	1978,33	1978,33
975726	277874	2163,33	2163,33	2163,33	2163,33	2163,33	2163,33	2163,33	2163,33
975756	277844	1976,67	1976,67	1976,67	1976,67	1976,67	1976,67	1976,67	1976,67
975786	277814	2093,33	2093,33	2093,33	2093,33	2093,33	2093,33	2093,33	2093,33
975816	277784	2033,33	2033,33	2033,33	2033,33	2033,33	2033,33	2033,33	2033,33
975846	277754	2178,33	2178,33	2178,33	2178,33	2178,33	2178,33	2178,33	2178,33
975876	277724	1976,67	1976,67	1976,67	1976,67	1976,67	1976,67	1976,67	1976,67
975906	277694	2071,67	2071,67	2071,67	2071,67	2071,67	2071,67	2071,67	2071,67
975936	277664	2223,33	2223,33	2223,33	2223,33	2223,33	2223,33	2223,33	2223,33
975966	277634	2025	2025	2025	2025	2025	2025	2025	2025
975996	277604	2136,67	2136,67	2136,67	2136,67	2136,67	2136,67	2136,67	2136,67
976026	277574	2091,67	2091,67	2091,67	2091,67	2091,67	2091,67	2091,67	2091,67
976056	277544	1991,67	1991,67	1991,67	1991,67	1991,67	1991,67	1991,67	1991,67
976086	277514	2175	2175	2175	2175	2175	2175	2175	2175
976116	277484	2128,33	2128,33	2128,33	2128,33	2128,33	2128,33	2128,33	2128,33
976146	277454	2058,33	2058,33	2058,33	2058,33	2058,33	2058,33	2058,33	2058,33

This journal is © The Royal Society of Chemistry 20xx

Please do not adjust margins
Please do not adjust margins

Please do not adjust margins

976176	277424	2135	2135	2135	2135	2135	2135	2135	2135
976206	277394	2106,67	2106,67	2106,67	2106,67	2106,67	2106,67	2106,67	2106,67
976236	277364	2068,33	2068,33	2068,33	2068,33	2068,33	2068,33	2068,33	2068,33
976266	277334	2136,67	2136,67	2136,67	2136,67	2136,67	2136,67	2136,67	2136,67
976296	277304	2138,33	2138,33	2138,33	2138,33	2138,33	2138,33	2138,33	2138,33
976326	277274	2133,33	2133,33	2133,33	2133,33	2133,33	2133,33	2133,33	2133,33
976356	277244	2085	2085	2085	2085	2085	2085	2085	2085
976386	277214	2140	2140	2140	2140	2140	2140	2140	2140
976416	277184	2210	2210	2210	2210	2210	2210	2210	2210
976446	277154	2211,67	2211,67	2211,67	2211,67	2211,67	2211,67	2211,67	2211,67
976476	277124	2086,67	2086,67	2086,67	2086,67	2086,67	2086,67	2086,67	2086,67
976506	277094	2123,33	2123,33	2123,33	2123,33	2123,33	2123,33	2123,33	2123,33
976536	277064	2120	2120	2120	2120	2120	2120	2120	2120
976566	277034	2035	2035	2035	2035	2035	2035	2035	2035
976596	277004	2143,33	2143,33	2143,33	2143,33	2143,33	2143,33	2143,33	2143,33
976626	276974	2138,33	2138,33	2138,33	2138,33	2138,33	2138,33	2138,33	2138,33
976656	276944	2068,33	2068,33	2068,33	2068,33	2068,33	2068,33	2068,33	2068,33
976686	276914	2145	2145	2145	2145	2145	2145	2145	2145
976716	276884	2183,33	2183,33	2183,33	2183,33	2183,33	2183,33	2183,33	2183,33
976746	276854	2096,67	2096,67	2096,67	2096,67	2096,67	2096,67	2096,67	2096,67
976776	276824	2123,33	2123,33	2123,33	2123,33	2123,33	2123,33	2123,33	2123,33
976806	276794	2183,33	2183,33	2183,33	2183,33	2183,33	2183,33	2183,33	2183,33
976836	276764	2176,67	2176,67	2176,67	2176,67	2176,67	2176,67	2176,67	2176,67
976866	276734	2208,33	2208,33	2208,33	2208,33	2208,33	2208,33	2208,33	2208,33
976896	276704	2220	2220	2220	2220	2220	2220	2220	2220
976926	276674	2196,67	2196,67	2196,67	2196,67	2196,67	2196,67	2196,67	2196,67
976956	276644	2213,33	2213,33	2213,33	2213,33	2213,33	2213,33	2213,33	2213,33
976986	276614	2115	2115	2115	2115	2115	2115	2115	2115
977016	276584	2191,67	2191,67	2191,67	2191,67	2191,67	2191,67	2191,67	2191,67
977046	276554	2148,33	2148,33	2148,33	2148,33	2148,33	2148,33	2148,33	2148,33
977076	276524	2305	2305	2305	2305	2305	2305	2305	2305
977106	276494	2246,67	2246,67	2246,67	2246,67	2246,67	2246,67	2246,67	2246,67
977136	276464	2168,33	2168,33	2168,33	2168,33	2168,33	2168,33	2168,33	2168,33
977166	276434	2120	2120	2120	2120	2120	2120	2120	2120
977196	276404	2181,67	2181,67	2181,67	2181,67	2181,67	2181,67	2181,67	2181,67
977226	276374	2131,67	2131,67	2131,67	2131,67	2131,67	2131,67	2131,67	2131,67
977256	276344	2221,67	2221,67	2221,67	2221,67	2221,67	2221,67	2221,67	2221,67
977286	276314	2200	2200	2200	2200	2200	2200	2200	2200
977316	276284	2276,67	2276,67	2276,67	2276,67	2276,67	2276,67	2276,67	2276,67
977346	276254	2236,67	2236,67	2236,67	2236,67	2236,67	2236,67	2236,67	2236,67
977376	276224	2168,33	2168,33	2168,33	2168,33	2168,33	2168,33	2168,33	2168,33
977406	276194	2011,67	2011,67	2011,67	2011,67	2011,67	2011,67	2011,67	2011,67
977436	276164	2243,33	2243,33	2243,33	2243,33	2243,33	2243,33	2243,33	2243,33
977466	276134	2268,33	2268,33	2268,33	2268,33	2268,33	2268,33	2268,33	2268,33
977496	276104	2135	2135	2135	2135	2135	2135	2135	2135
977526	276074	2091,67	2091,67	2091,67	2091,67	2091,67	2091,67	2091,67	2091,67
977556	276044	2148,33	2148,33	2148,33	2148,33	2148,33	2148,33	2148,33	2148,33

This journal is © The Royal Society of Chemistry 20xx

Please do not adjust margins

977586	276014	2191,67	2191,67	2191,67	2191,67	2191,67	2191,67	2191,67	2191,67
977616	275984	2296,67	2296,67	2296,67	2296,67	2296,67	2296,67	2296,67	2296,67
977646	275954	2205	2205	2205	2205	2205	2205	2205	2205
977676	275924	2150	2150	2150	2150	2150	2150	2150	2150
977706	275894	2195	2195	2195	2195	2195	2195	2195	2195
977736	275864	2066,67	2066,67	2066,67	2066,67	2066,67	2066,67	2066,67	2066,67
977766	275834	2140	2140	2140	2140	2140	2140	2140	2140
977796	275804	2095	2095	2095	2095	2095	2095	2095	2095
977826	275774	2113,33	2113,33	2113,33	2113,33	2113,33	2113,33	2113,33	2113,33
977856	275744	2133,33	2133,33	2133,33	2133,33	2133,33	2133,33	2133,33	2133,33
977886	275714	2168,33	2168,33	2168,33	2168,33	2168,33	2168,33	2168,33	2168,33
977916	275684	2115	2115	2115	2115	2115	2115	2115	2115
977946	275654	2166,67	2166,67	2166,67	2166,67	2166,67	2166,67	2166,67	2166,67
977976	275624	2085	2085	2085	2085	2085	2085	2085	2085
978006	275594	2188,33	2188,33	2188,33	2188,33	2188,33	2188,33	2188,33	2188,33
978036	275564	2130	2130	2130	2130	2130	2130	2130	2130
978066	275534	2141,67	2141,67	2141,67	2141,67	2141,67	2141,67	2141,67	2141,67
978096	275504	2081,67	2081,67	2081,67	2081,67	2081,67	2081,67	2081,67	2081,67
978126	275474	2025	2025	2025	2025	2025	2025	2025	2025
978156	275444	2101,67	2101,67	2101,67	2101,67	2101,67	2101,67	2101,67	2101,67
978186	275414	2090	2090	2090	2090	2090	2090	2090	2090
978216	275384	2023,33	2023,33	2023,33	2023,33	2023,33	2023,33	2023,33	2023,33
978246	275354	2085	2085	2085	2085	2085	2085	2085	2085
978276	275324	2070	2070	2070	2070	2070	2070	2070	2070
978306	275294	2060	2060	2060	2060	2060	2060	2060	2060
978336	275264	2058,33	2058,33	2058,33	2058,33	2058,33	2058,33	2058,33	2058,33
978366	275234	2193,33	2193,33	2193,33	2193,33	2193,33	2193,33	2193,33	2193,33
978396	275204	2105	2105	2105	2105	2105	2105	2105	2105
978426	275174	2140	2140	2140	2140	2140	2140	2140	2140
978456	275144	2145	2145	2145	2145	2145	2145	2145	2145
978486	275114	2058,33	2058,33	2058,33	2058,33	2058,33	2058,33	2058,33	2058,33
978516	275084	2070	2070	2070	2070	2070	2070	2070	2070
978546	275054	1988,33	1988,33	1988,33	1988,33	1988,33	1988,33	1988,33	1988,33
978576	275024	2075	2075	2075	2075	2075	2075	2075	2075
978606	274994	2081,67	2081,67	2081,67	2081,67	2081,67	2081,67	2081,67	2081,67
978636	274964	2056,67	2056,67	2056,67	2056,67	2056,67	2056,67	2056,67	2056,67
978666	274934	2110	2110	2110	2110	2110	2110	2110	2110
978696	274904	1930	1930	1930	1930	1930	1930	1930	1930
978726	274874	2040	2040	2040	2040	2040	2040	2040	2040
978756	274844	2041,67	2041,67	2041,67	2041,67	2041,67	2041,67	2041,67	2041,67
978786	274814	2090	2090	2090	2090	2090	2090	2090	2090
978816	274784	2088,33	2088,33	2088,33	2088,33	2088,33	2088,33	2088,33	2088,33
978846	274754	2078,33	2078,33	2078,33	2078,33	2078,33	2078,33	2078,33	2078,33
978876	274724	1913,33	1913,33	1913,33	1913,33	1913,33	1913,33	1913,33	1913,33
978906	274694	2040	2040	2040	2040	2040	2040	2040	2040
978936	274664	2008,33	2008,33	2008,33	2008,33	2008,33	2008,33	2008,33	2008,33
978966	274634	1983,33	1983,33	1983,33	1983,33	1983,33	1983,33	1983,33	1983,33

This journal is © The Royal Society of Chemistry 20xx

Please do not adjust margins

Please do not adjust margins

978996	274604	2041,67	2041,67	2041,67	2041,67	2041,67	2041,67	2041,67	2041,67
979026	274574	2135	2135	2135	2135	2135	2135	2135	2135
979056	274544	2073,33	2073,33	2073,33	2073,33	2073,33	2073,33	2073,33	2073,33
979086	274514	1966,67	1966,67	1966,67	1966,67	1966,67	1966,67	1966,67	1966,67
979116	274484	2036,67	2036,67	2036,67	2036,67	2036,67	2036,67	2036,67	2036,67
979146	274454	1906,67	1906,67	1906,67	1906,67	1906,67	1906,67	1906,67	1906,67
979176	274424	2035	2035	2035	2035	2035	2035	2035	2035
979206	274394	2005	2005	2005	2005	2005	2005	2005	2005
979236	274364	1986,67	1986,67	1986,67	1986,67	1986,67	1986,67	1986,67	1986,67
979266	274334	1966,67	1966,67	1966,67	1966,67	1966,67	1966,67	1966,67	1966,67
979296	274304	1943,33	1943,33	1943,33	1943,33	1943,33	1943,33	1943,33	1943,33
979326	274274	1953,33	1953,33	1953,33	1953,33	1953,33	1953,33	1953,33	1953,33
979356	274244	1996,67	1996,67	1996,67	1996,67	1996,67	1996,67	1996,67	1996,67
979386	274214	1913,33	1913,33	1913,33	1913,33	1913,33	1913,33	1913,33	1913,33
979416	274184	1910	1910	1910	1910	1910	1910	1910	1910
979446	274154	1976,67	1976,67	1976,67	1976,67	1976,67	1976,67	1976,67	1976,67
979476	274124	2015	2015	2015	2015	2015	2015	2015	2015
979506	274094	1863,33	1863,33	1863,33	1863,33	1863,33	1863,33	1863,33	1863,33
979536	274064	1913,33	1913,33	1913,33	1913,33	1913,33	1913,33	1913,33	1913,33
979566	274034	1985	1985	1985	1985	1985	1985	1985	1985
979596	274004	1885	1885	1885	1885	1885	1885	1885	1885
979626	273974	1965	1965	1965	1965	1965	1965	1965	1965
979656	273944	1890	1890	1890	1890	1890	1890	1890	1890
979686	273914	1990	1990	1990	1990	1990	1990	1990	1990
979716	273884	1848,33	1848,33	1848,33	1848,33	1848,33	1848,33	1848,33	1848,33
979746	273854	1896,67	1896,67	1896,67	1896,67	1896,67	1896,67	1896,67	1896,67
979776	273824	1870	1870	1870	1870	1870	1870	1870	1870
979806	273794	1941,67	1941,67	1941,67	1941,67	1941,67	1941,67	1941,67	1941,67
979836	273764	1870	1870	1870	1870	1870	1870	1870	1870
979866	273734	1866,67	1866,67	1866,67	1866,67	1866,67	1866,67	1866,67	1866,67
979896	273704	1931,67	1931,67	1931,67	1931,67	1931,67	1931,67	1931,67	1931,67
979926	273674	1811,67	1811,67	1811,67	1811,67	1811,67	1811,67	1811,67	1811,67
979956	273644	1788,33	1788,33	1788,33	1788,33	1788,33	1788,33	1788,33	1788,33
979986	273614	1805	1805	1805	1805	1805	1805	1805	1805
980016	273584	1845	1845	1845	1845	1845	1845	1845	1845
980046	273554	1816,67	1816,67	1816,67	1816,67	1816,67	1816,67	1816,67	1816,67
980076	273524	1845	1845	1845	1845	1845	1845	1845	1845
980106	273494	1843,33	1843,33	1843,33	1843,33	1843,33	1843,33	1843,33	1843,33
980136	273464	1926,67	1926,67	1926,67	1926,67	1926,67	1926,67	1926,67	1926,67
980166	273434	1923,33	1923,33	1923,33	1923,33	1923,33	1923,33	1923,33	1923,33
980196	273404	1950	1950	1950	1950	1950	1950	1950	1950
980226	273374	1925	1925	1925	1925	1925	1925	1925	1925
980256	273344	1855	1855	1855	1855	1855	1855	1855	1855
980286	273314	1898,33	1898,33	1898,33	1898,33	1898,33	1898,33	1898,33	1898,33
980316	273284	1825	1825	1825	1825	1825	1825	1825	1825
980346	273254	1863,33	1863,33	1863,33	1863,33	1863,33	1863,33	1863,33	1863,33
980376	273224	1855	1855	1855	1855	1855	1855	1855	1855

This journal is © The Royal Society of Chemistry 20xx

Please do not adjust margins

980406	273194	1786,67	1786,67	1786,67	1786,67	1786,67	1786,67	1786,67	1786,67
980436	273164	1828,33	1828,33	1828,33	1828,33	1828,33	1828,33	1828,33	1828,33
980466	273134	1803,33	1803,33	1803,33	1803,33	1803,33	1803,33	1803,33	1803,33
980496	273104	1841,67	1841,67	1841,67	1841,67	1841,67	1841,67	1841,67	1841,67
980526	273074	1956,67	1956,67	1956,67	1956,67	1956,67	1956,67	1956,67	1956,67
980556	273044	1826,67	1826,67	1826,67	1826,67	1826,67	1826,67	1826,67	1826,67
980586	273014	1765	1765	1765	1765	1765	1765	1765	1765
980616	272984	1800	1800	1800	1800	1800	1800	1800	1800
980646	272954	1885	1885	1885	1885	1885	1885	1885	1885
980676	272924	1795	1795	1795	1795	1795	1795	1795	1795
980706	272894	1838,33	1838,33	1838,33	1838,33	1838,33	1838,33	1838,33	1838,33
980736	272864	1756,67	1756,67	1756,67	1756,67	1756,67	1756,67	1756,67	1756,67
980766	272834	1796,67	1796,67	1796,67	1796,67	1796,67	1796,67	1796,67	1796,67
980796	272804	1816,67	1816,67	1816,67	1816,67	1816,67	1816,67	1816,67	1816,67
980826	272774	1913,33	1913,33	1913,33	1913,33	1913,33	1913,33	1913,33	1913,33
980856	272744	1760	1760	1760	1760	1760	1760	1760	1760
980886	272714	1758,33	1758,33	1758,33	1758,33	1758,33	1758,33	1758,33	1758,33
980916	272684	1760	1760	1760	1760	1760	1760	1760	1760
980946	272654	1776,67	1776,67	1776,67	1776,67	1776,67	1776,67	1776,67	1776,67
980976	272624	1853,33	1853,33	1853,33	1853,33	1853,33	1853,33	1853,33	1853,33
981006	272594	1806,67	1806,67	1806,67	1806,67	1806,67	1806,67	1806,67	1806,67
981036	272564	1821,67	1821,67	1821,67	1821,67	1821,67	1821,67	1821,67	1821,67
981066	272534	1901,67	1901,67	1901,67	1901,67	1901,67	1901,67	1901,67	1901,67
981096	272504	1798,33	1798,33	1798,33	1798,33	1798,33	1798,33	1798,33	1798,33
981126	272474	2000	2000	2000	2000	2000	2000	2000	2000
981156	272444	1791,67	1791,67	1791,67	1791,67	1791,67	1791,67	1791,67	1791,67
981186	272414	1786,67	1786,67	1786,67	1786,67	1786,67	1786,67	1786,67	1786,67
981216	272384	1818,33	1818,33	1818,33	1818,33	1818,33	1818,33	1818,33	1818,33
981246	272354	1798,33	1798,33	1798,33	1798,33	1798,33	1798,33	1798,33	1798,33
981276	272324	1818,33	1818,33	1818,33	1818,33	1818,33	1818,33	1818,33	1818,33
981306	272294	1903,33	1903,33	1903,33	1903,33	1903,33	1903,33	1903,33	1903,33
981336	272264	1778,33	1778,33	1778,33	1778,33	1778,33	1778,33	1778,33	1778,33
981366	272234	1775	1775	1775	1775	1775	1775	1775	1775
981396	272204	1780	1780	1780	1780	1780	1780	1780	1780
981426	272174	1755	1755	1755	1755	1755	1755	1755	1755
981456	272144	1723,33	1723,33	1723,33	1723,33	1723,33	1723,33	1723,33	1723,33
981486	272114	1768,33	1768,33	1768,33	1768,33	1768,33	1768,33	1768,33	1768,33
981516	272084	1775	1775	1775	1775	1775	1775	1775	1775
981546	272054	1791,67	1791,67	1791,67	1791,67	1791,67	1791,67	1791,67	1791,67
981576	272024	1821,67	1821,67	1821,67	1821,67	1821,67	1821,67	1821,67	1821,67
981606	271994	1815	1815	1815	1815	1815	1815	1815	1815
981636	271964	1761,67	1761,67	1761,67	1761,67	1761,67	1761,67	1761,67	1761,67
981666	271934	1826,67	1826,67	1826,67	1826,67	1826,67	1826,67	1826,67	1826,67
981696	271904	1848,33	1848,33	1848,33	1848,33	1848,33	1848,33	1848,33	1848,33
981726	271874	1795	1795	1795	1795	1795	1795	1795	1795
981756	271844	1846,67	1846,67	1846,67	1846,67	1846,67	1846,67	1846,67	1846,67
981786	271814	1826,67	1826,67	1826,67	1826,67	1826,67	1826,67	1826,67	1826,67

This journal is © The Royal Society of Chemistry 20xx

Please do not adjust margins

981816	271784	1766,67	1766,67	1766,67	1766,67	1766,67	1766,67	1766,67	1766,67
981846	271754	1791,67	1791,67	1791,67	1791,67	1791,67	1791,67	1791,67	1791,67
981876	271724	1788,33	1788,33	1788,33	1788,33	1788,33	1788,33	1788,33	1788,33
981906	271694	1846,67	1846,67	1846,67	1846,67	1846,67	1846,67	1846,67	1846,67
981936	271664	1876,67	1876,67	1876,67	1876,67	1876,67	1876,67	1876,67	1876,67
981966	271634	1868,33	1868,33	1868,33	1868,33	1868,33	1868,33	1868,33	1868,33
981996	271604	1836,67	1836,67	1836,67	1836,67	1836,67	1836,67	1836,67	1836,67
982026	271574	1828,33	1828,33	1828,33	1828,33	1828,33	1828,33	1828,33	1828,33
982056	271544	1776,67	1776,67	1776,67	1776,67	1776,67	1776,67	1776,67	1776,67
982086	271514	1758,33	1758,33	1758,33	1758,33	1758,33	1758,33	1758,33	1758,33
982116	271484	1733,33	1733,33	1733,33	1733,33	1733,33	1733,33	1733,33	1733,33
982146	271454	1771,67	1771,67	1771,67	1771,67	1771,67	1771,67	1771,67	1771,67
982176	271424	1933,33	1933,33	1933,33	1933,33	1933,33	1933,33	1933,33	1933,33
982206	271394	1670	1670	1670	1670	1670	1670	1670	1670
982236	271364	1725	1725	1725	1725	1725	1725	1725	1725
982266	271334	1883,33	1883,33	1883,33	1883,33	1883,33	1883,33	1883,33	1883,33
982296	271304	1758,33	1758,33	1758,33	1758,33	1758,33	1758,33	1758,33	1758,33
982326	271274	1745	1745	1745	1745	1745	1745	1745	1745
982356	271244	1865	1865	1865	1865	1865	1865	1865	1865
982386	271214	1848,33	1848,33	1848,33	1848,33	1848,33	1848,33	1848,33	1848,33
982416	271184	1721,67	1721,67	1721,67	1721,67	1721,67	1721,67	1721,67	1721,67
982446	271154	1780	1780	1780	1780	1780	1780	1780	1780
982476	271124	1746,67	1746,67	1746,67	1746,67	1746,67	1746,67	1746,67	1746,67
982506	271094	1770	1770	1770	1770	1770	1770	1770	1770
982536	271064	1761,67	1761,67	1761,67	1761,67	1761,67	1761,67	1761,67	1761,67
982566	271034	1846,67	1846,67	1846,67	1846,67	1846,67	1846,67	1846,67	1846,67
982596	271004	1915	1915	1915	1915	1915	1915	1915	1915
982626	270974	1763,33	1763,33	1763,33	1763,33	1763,33	1763,33	1763,33	1763,33
982656	270944	1820	1820	1820	1820	1820	1820	1820	1820
982686	270914	1895	1895	1895	1895	1895	1895	1895	1895
982716	270884	1811,67	1811,67	1811,67	1811,67	1811,67	1811,67	1811,67	1811,67
982746	270854	1691,67	1691,67	1691,67	1691,67	1691,67	1691,67	1691,67	1691,67
982776	270824	1831,67	1831,67	1831,67	1831,67	1831,67	1831,67	1831,67	1831,67
982806	270794	1755	1755	1755	1755	1755	1755	1755	1755
982836	270764	1853,33	1853,33	1853,33	1853,33	1853,33	1853,33	1853,33	1853,33
982866	270734	1726,67	1726,67	1726,67	1726,67	1726,67	1726,67	1726,67	1726,67
982896	270704	1778,33	1778,33	1778,33	1778,33	1778,33	1778,33	1778,33	1778,33
982926	270674	1853,33	1853,33	1853,33	1853,33	1853,33	1853,33	1853,33	1853,33
982956	270644	1798,33	1798,33	1798,33	1798,33	1798,33	1798,33	1798,33	1798,33
982986	270614	1805	1805	1805	1805	1805	1805	1805	1805
983016	270584	1880	1880	1880	1880	1880	1880	1880	1880
983046	270554	1803,33	1803,33	1803,33	1803,33	1803,33	1803,33	1803,33	1803,33
983076	270524	1716,67	1716,67	1716,67	1716,67	1716,67	1716,67	1716,67	1716,67
983106	270494	1856,67	1856,67	1856,67	1856,67	1856,67	1856,67	1856,67	1856,67
983136	270464	1801,67	1801,67	1801,67	1801,67	1801,67	1801,67	1801,67	1801,67
983166	270434	1810	1810	1810	1810	1810	1810	1810	1810
983196	270404	1815	1815	1815	1815	1815	1815	1815	1815

This journal is © The Royal Society of Chemistry 20xx

Please do not adjust margins

983226	270374	1868,33	1868,33	1868,33	1868,33	1868,33	1868,33	1868,33	1868,33
983256	270344	1736,67	1736,67	1736,67	1736,67	1736,67	1736,67	1736,67	1736,67
983286	270314	1750	1750	1750	1750	1750	1750	1750	1750
983316	270284	1808,33	1808,33	1808,33	1808,33	1808,33	1808,33	1808,33	1808,33
983346	270254	1816,67	1816,67	1816,67	1816,67	1816,67	1816,67	1816,67	1816,67
983376	270224	1790	1790	1790	1790	1790	1790	1790	1790
983406	270194	1848,33	1848,33	1848,33	1848,33	1848,33	1848,33	1848,33	1848,33
983436	270164	1846,67	1846,67	1846,67	1846,67	1846,67	1846,67	1846,67	1846,67
983466	270134	1788,33	1788,33	1788,33	1788,33	1788,33	1788,33	1788,33	1788,33
983496	270104	1791,67	1791,67	1791,67	1791,67	1791,67	1791,67	1791,67	1791,67
983526	270074	1711,67	1711,67	1711,67	1711,67	1711,67	1711,67	1711,67	1711,67
983556	270044	1843,33	1843,33	1843,33	1843,33	1843,33	1843,33	1843,33	1843,33
983586	270014	1795	1795	1795	1795	1795	1795	1795	1795
983616	269984	1885	1885	1885	1885	1885	1885	1885	1885
983646	269954	1876,67	1876,67	1876,67	1876,67	1876,67	1876,67	1876,67	1876,67
983676	269924	1721,67	1721,67	1721,67	1721,67	1721,67	1721,67	1721,67	1721,67
983706	269894	1780	1780	1780	1780	1780	1780	1780	1780
983736	269864	1933,33	1933,33	1933,33	1933,33	1933,33	1933,33	1933,33	1933,33
983766	269834	1768,33	1768,33	1768,33	1768,33	1768,33	1768,33	1768,33	1768,33
983796	269804	1830	1830	1830	1830	1830	1830	1830	1830
983826	269774	1788,33	1788,33	1788,33	1788,33	1788,33	1788,33	1788,33	1788,33
983856	269744	1833,33	1833,33	1833,33	1833,33	1833,33	1833,33	1833,33	1833,33
983886	269714	1896,67	1896,67	1896,67	1896,67	1896,67	1896,67	1896,67	1896,67
983916	269684	1760	1760	1760	1760	1760	1760	1760	1760
983946	269654	1830	1830	1830	1830	1830	1830	1830	1830
983976	269624	1823,33	1823,33	1823,33	1823,33	1823,33	1823,33	1823,33	1823,33
984006	269594	1823,33	1823,33	1823,33	1823,33	1823,33	1823,33	1823,33	1823,33
984036	269564	1936,67	1936,67	1936,67	1936,67	1936,67	1936,67	1936,67	1936,67
984066	269534	1836,67	1836,67	1836,67	1836,67	1836,67	1836,67	1836,67	1836,67
984096	269504	1898,33	1898,33	1898,33	1898,33	1898,33	1898,33	1898,33	1898,33
984126	269474	1856,67	1856,67	1856,67	1856,67	1856,67	1856,67	1856,67	1856,67
984156	269444	1830	1830	1830	1830	1830	1830	1830	1830
984186	269414	1853,33	1853,33	1853,33	1853,33	1853,33	1853,33	1853,33	1853,33
984216	269384	1868,33	1868,33	1868,33	1868,33	1868,33	1868,33	1868,33	1868,33
984246	269354	1890	1890	1890	1890	1890	1890	1890	1890
984276	269324	1836,67	1836,67	1836,67	1836,67	1836,67	1836,67	1836,67	1836,67
984306	269294	1771,67	1771,67	1771,67	1771,67	1771,67	1771,67	1771,67	1771,67
984336	269264	1876,67	1876,67	1876,67	1876,67	1876,67	1876,67	1876,67	1876,67
984366	269234	1941,67	1941,67	1941,67	1941,67	1941,67	1941,67	1941,67	1941,67
984396	269204	1805	1805	1805	1805	1805	1805	1805	1805
984426	269174	1815	1815	1815	1815	1815	1815	1815	1815
984456	269144	1810	1810	1810	1810	1810	1810	1810	1810
984486	269114	1901,67	1901,67	1901,67	1901,67	1901,67	1901,67	1901,67	1901,67
984516	269084	2011,67	2011,67	2011,67	2011,67	2011,67	2011,67	2011,67	2011,67
984546	269054	1841,67	1841,67	1841,67	1841,67	1841,67	1841,67	1841,67	1841,67
984576	269024	1793,33	1793,33	1793,33	1793,33	1793,33	1793,33	1793,33	1793,33
984606	268994	1893,33	1893,33	1893,33	1893,33	1893,33	1893,33	1893,33	1893,33

This journal is © The Royal Society of Chemistry 20xx

Please do not adjust margins

984636	268964	1771,67	1771,67	1771,67	1771,67	1771,67	1771,67	1771,67	1771,67
984666	268934	1840	1840	1840	1840	1840	1840	1840	1840
984696	268904	1841,67	1841,67	1841,67	1841,67	1841,67	1841,67	1841,67	1841,67
984726	268874	1851,67	1851,67	1851,67	1851,67	1851,67	1851,67	1851,67	1851,67
984756	268844	1860	1860	1860	1860	1860	1860	1860	1860
984786	268814	1816,67	1816,67	1816,67	1816,67	1816,67	1816,67	1816,67	1816,67
984816	268784	1911,67	1911,67	1911,67	1911,67	1911,67	1911,67	1911,67	1911,67
984846	268754	1880	1880	1880	1880	1880	1880	1880	1880
984876	268724	1880	1880	1880	1880	1880	1880	1880	1880
984906	268694	1858,33	1858,33	1858,33	1858,33	1858,33	1858,33	1858,33	1858,33
984936	268664	1865	1865	1865	1865	1865	1865	1865	1865
984966	268634	1855	1855	1855	1855	1855	1855	1855	1855
984996	268604	1918,33	1918,33	1918,33	1918,33	1918,33	1918,33	1918,33	1918,33
985026	268574	1855	1855	1855	1855	1855	1855	1855	1855
985056	268544	1910	1910	1910	1910	1910	1910	1910	1910
985086	268514	1871,67	1871,67	1871,67	1871,67	1871,67	1871,67	1871,67	1871,67
985116	268484	1831,67	1831,67	1831,67	1831,67	1831,67	1831,67	1831,67	1831,67
985146	268454	1990	1990	1990	1990	1990	1990	1990	1990
985176	268424	1885	1885	1885	1885	1885	1885	1885	1885
985206	268394	1823,33	1823,33	1823,33	1823,33	1823,33	1823,33	1823,33	1823,33
985236	268364	1863,33	1863,33	1863,33	1863,33	1863,33	1863,33	1863,33	1863,33
985266	268334	1853,33	1853,33	1853,33	1853,33	1853,33	1853,33	1853,33	1853,33
985296	268304	1890	1890	1890	1890	1890	1890	1890	1890
985326	268274	1840	1840	1840	1840	1840	1840	1840	1840
985356	268244	1875	1875	1875	1875	1875	1875	1875	1875
985386	268214	1908,33	1908,33	1908,33	1908,33	1908,33	1908,33	1908,33	1908,33
985416	268184	1835	1835	1835	1835	1835	1835	1835	1835
985446	268154	1918,33	1918,33	1918,33	1918,33	1918,33	1918,33	1918,33	1918,33
985476	268124	1843,33	1843,33	1843,33	1843,33	1843,33	1843,33	1843,33	1843,33
985506	268094	1871,67	1871,67	1871,67	1871,67	1871,67	1871,67	1871,67	1871,67
985536	268064	1896,67	1896,67	1896,67	1896,67	1896,67	1896,67	1896,67	1896,67
985566	268034	1896,67	1896,67	1896,67	1896,67	1896,67	1896,67	1896,67	1896,67
985596	268004	1888,33	1888,33	1888,33	1888,33	1888,33	1888,33	1888,33	1888,33
985626	267974	1756,67	1756,67	1756,67	1756,67	1756,67	1756,67	1756,67	1756,67
985656	267944	1906,67	1906,67	1906,67	1906,67	1906,67	1906,67	1906,67	1906,67
985686	267914	1906,67	1906,67	1906,67	1906,67	1906,67	1906,67	1906,67	1906,67
985716	267884	1868,33	1868,33	1868,33	1868,33	1868,33	1868,33	1868,33	1868,33
985746	267854	1838,33	1838,33	1838,33	1838,33	1838,33	1838,33	1838,33	1838,33
985776	267824	1871,67	1871,67	1871,67	1871,67	1871,67	1871,67	1871,67	1871,67
985806	267794	1798,33	1798,33	1798,33	1798,33	1798,33	1798,33	1798,33	1798,33
985836	267764	1910	1910	1910	1910	1910	1910	1910	1910
985866	267734	1825	1825	1825	1825	1825	1825	1825	1825
985896	267704	1816,67	1816,67	1816,67	1816,67	1816,67	1816,67	1816,67	1816,67
985926	267674	1825	1825	1825	1825	1825	1825	1825	1825
985956	267644	1725	1725	1725	1725	1725	1725	1725	1725
985986	267614	1811,67	1811,67	1811,67	1811,67	1811,67	1811,67	1811,67	1811,67
986016	267584	1898,33	1898,33	1898,33	1898,33	1898,33	1898,33	1898,33	1898,33

This journal is © The Royal Society of Chemistry 20xx

Please do not adjust margins

986046	267554	1783,33	1783,33	1783,33	1783,33	1783,33	1783,33	1783,33	1783,33
986076	267524	1810	1810	1810	1810	1810	1810	1810	1810
986106	267494	1896,67	1896,67	1896,67	1896,67	1896,67	1896,67	1896,67	1896,67
986136	267464	1961,67	1961,67	1961,67	1961,67	1961,67	1961,67	1961,67	1961,67
986166	267434	1801,67	1801,67	1801,67	1801,67	1801,67	1801,67	1801,67	1801,67
986196	267404	1713,33	1713,33	1713,33	1713,33	1713,33	1713,33	1713,33	1713,33
986226	267374	1921,67	1921,67	1921,67	1921,67	1921,67	1921,67	1921,67	1921,67
986256	267344	1865	1865	1865	1865	1865	1865	1865	1865
986286	267314	1845	1845	1845	1845	1845	1845	1845	1845
986316	267284	1795	1795	1795	1795	1795	1795	1795	1795
986346	267254	1898,33	1898,33	1898,33	1898,33	1898,33	1898,33	1898,33	1898,33
986376	267224	1881,67	1881,67	1881,67	1881,67	1881,67	1881,67	1881,67	1881,67
986406	267194	1811,67	1811,67	1811,67	1811,67	1811,67	1811,67	1811,67	1811,67
986436	267164	1830	1830	1830	1830	1830	1830	1830	1830
986466	267134	1895	1895	1895	1895	1895	1895	1895	1895
986496	267104	1883,33	1883,33	1883,33	1883,33	1883,33	1883,33	1883,33	1883,33
986526	267074	1680	1680	1680	1680	1680	1680	1680	1680
986556	267044	1876,67	1876,67	1876,67	1876,67	1876,67	1876,67	1876,67	1876,67
986586	267014	2005	2005	2005	2005	2005	2005	2005	2005
986616	266984	1733,33	1733,33	1733,33	1733,33	1733,33	1733,33	1733,33	1733,33
986646	266954	1783,33	1783,33	1783,33	1783,33	1783,33	1783,33	1783,33	1783,33
986676	266924	1820	1820	1820	1820	1820	1820	1820	1820
986706	266894	1785	1785	1785	1785	1785	1785	1785	1785
986736	266864	1920	1920	1920	1920	1920	1920	1920	1920
986766	266834	1798,33	1798,33	1798,33	1798,33	1798,33	1798,33	1798,33	1798,33
986796	266804	1803,33	1803,33	1803,33	1803,33	1803,33	1803,33	1803,33	1803,33
986826	266774	1726,67	1726,67	1726,67	1726,67	1726,67	1726,67	1726,67	1726,67
986856	266744	1853,33	1853,33	1853,33	1853,33	1853,33	1853,33	1853,33	1853,33
986886	266714	1928,33	1928,33	1928,33	1928,33	1928,33	1928,33	1928,33	1928,33
986916	266684	1823,33	1823,33	1823,33	1823,33	1823,33	1823,33	1823,33	1823,33
986946	266654	1806,67	1806,67	1806,67	1806,67	1806,67	1806,67	1806,67	1806,67
986976	266624	1785	1785	1785	1785	1785	1785	1785	1785
987006	266594	1801,67	1801,67	1801,67	1801,67	1801,67	1801,67	1801,67	1801,67
987036	266564	1801,67	1801,67	1801,67	1801,67	1801,67	1801,67	1801,67	1801,67
987066	266534	1751,67	1751,67	1751,67	1751,67	1751,67	1751,67	1751,67	1751,67
987096	266504	1825	1825	1825	1825	1825	1825	1825	1825
987126	266474	1828,33	1828,33	1828,33	1828,33	1828,33	1828,33	1828,33	1828,33
987156	266444	1781,67	1781,67	1781,67	1781,67	1781,67	1781,67	1781,67	1781,67
987186	266414	1841,67	1841,67	1841,67	1841,67	1841,67	1841,67	1841,67	1841,67
987216	266384	1826,67	1826,67	1826,67	1826,67	1826,67	1826,67	1826,67	1826,67
987246	266354	1858,33	1858,33	1858,33	1858,33	1858,33	1858,33	1858,33	1858,33
987276	266324	1856,67	1856,67	1856,67	1856,67	1856,67	1856,67	1856,67	1856,67
987306	266294	1851,67	1851,67	1851,67	1851,67	1851,67	1851,67	1851,67	1851,67
987336	266264	1888,33	1888,33	1888,33	1888,33	1888,33	1888,33	1888,33	1888,33
987366	266234	1900	1900	1900	1900	1900	1900	1900	1900
987396	266204	1835	1835	1835	1835	1835	1835	1835	1835
987426	266174	1861,67	1861,67	1861,67	1861,67	1861,67	1861,67	1861,67	1861,67

This journal is © The Royal Society of Chemistry 20xx

Please do not adjust margins

987456	266144	1841,67	1841,67	1841,67	1841,67	1841,67	1841,67	1841,67	1841,67
987486	266114	1883,33	1883,33	1883,33	1883,33	1883,33	1883,33	1883,33	1883,33
987516	266084	1860	1860	1860	1860	1860	1860	1860	1860
987546	266054	1923,33	1923,33	1923,33	1923,33	1923,33	1923,33	1923,33	1923,33
987576	266024	1891,67	1891,67	1891,67	1891,67	1891,67	1891,67	1891,67	1891,67
987606	265994	1891,67	1891,67	1891,67	1891,67	1891,67	1891,67	1891,67	1891,67
987636	265964	1898,33	1898,33	1898,33	1898,33	1898,33	1898,33	1898,33	1898,33
987666	265934	1915	1915	1915	1915	1915	1915	1915	1915
987696	265904	1903,33	1903,33	1903,33	1903,33	1903,33	1903,33	1903,33	1903,33
987726	265874	1868,33	1868,33	1868,33	1868,33	1868,33	1868,33	1868,33	1868,33
987756	265844	2051,67	2051,67	2051,67	2051,67	2051,67	2051,67	2051,67	2051,67
987786	265814	1898,33	1898,33	1898,33	1898,33	1898,33	1898,33	1898,33	1898,33
987816	265784	1868,33	1868,33	1868,33	1868,33	1868,33	1868,33	1868,33	1868,33
987846	265754	1876,67	1876,67	1876,67	1876,67	1876,67	1876,67	1876,67	1876,67
987876	265724	1945	1945	1945	1945	1945	1945	1945	1945
987906	265694	1885	1885	1885	1885	1885	1885	1885	1885
987936	265664	1895	1895	1895	1895	1895	1895	1895	1895
987966	265634	1900	1900	1900	1900	1900	1900	1900	1900
987996	265604	1900	1900	1900	1900	1900	1900	1900	1900
988026	265574	1948,33	1948,33	1948,33	1948,33	1948,33	1948,33	1948,33	1948,33
988056	265544	1976,67	1976,67	1976,67	1976,67	1976,67	1976,67	1976,67	1976,67
988086	265514	1946,67	1946,67	1946,67	1946,67	1946,67	1946,67	1946,67	1946,67
988116	265484	1923,33	1923,33	1923,33	1923,33	1923,33	1923,33	1923,33	1923,33
988146	265454	1945	1945	1945	1945	1945	1945	1945	1945
988176	265424	1845	1845	1845	1845	1845	1845	1845	1845
988206	265394	1933,33	1933,33	1933,33	1933,33	1933,33	1933,33	1933,33	1933,33
988236	265364	1978,33	1978,33	1978,33	1978,33	1978,33	1978,33	1978,33	1978,33
988266	265334	2020	2020	2020	2020	2020	2020	2020	2020
988296	265304	2108,33	2108,33	2108,33	2108,33	2108,33	2108,33	2108,33	2108,33
988326	265274	1975	1975	1975	1975	1975	1975	1975	1975
988356	265244	1886,67	1886,67	1886,67	1886,67	1886,67	1886,67	1886,67	1886,67
988386	265214	2008,33	2008,33	2008,33	2008,33	2008,33	2008,33	2008,33	2008,33
988416	265184	2058,33	2058,33	2058,33	2058,33	2058,33	2058,33	2058,33	2058,33
988446	265154	2055	2055	2055	2055	2055	2055	2055	2055
988476	265124	2030	2030	2030	2030	2030	2030	2030	2030
988506	265094	2056,67	2056,67	2056,67	2056,67	2056,67	2056,67	2056,67	2056,67
988536	265064	2036,67	2036,67	2036,67	2036,67	2036,67	2036,67	2036,67	2036,67
988566	265034	2033,33	2033,33	2033,33	2033,33	2033,33	2033,33	2033,33	2033,33
988596	265004	2111,67	2111,67	2111,67	2111,67	2111,67	2111,67	2111,67	2111,67
988626	264974	2096,67	2096,67	2096,67	2096,67	2096,67	2096,67	2096,67	2096,67
988656	264944	2145	2145	2145	2145	2145	2145	2145	2145
988686	264914	1975	1975	1975	1975	1975	1975	1975	1975
988716	264884	2098,33	2098,33	2098,33	2098,33	2098,33	2098,33	2098,33	2098,33
988746	264854	2060	2060	2060	2060	2060	2060	2060	2060
988776	264824	2230	2230	2230	2230	2230	2230	2230	2230
988806	264794	2093,33	2093,33	2093,33	2093,33	2093,33	2093,33	2093,33	2093,33
988836	264764	2111,67	2111,67	2111,67	2111,67	2111,67	2111,67	2111,67	2111,67

This journal is © The Royal Society of Chemistry 20xx

Please do not adjust margins

988866	264734	2148,33	2148,33	2148,33	2148,33	2148,33	2148,33	2148,33	2148,33
988896	264704	2283,33	2283,33	2283,33	2283,33	2283,33	2283,33	2283,33	2283,33
988926	264674	2206,67	2206,67	2206,67	2206,67	2206,67	2206,67	2206,67	2206,67
988956	264644	2218,33	2218,33	2218,33	2218,33	2218,33	2218,33	2218,33	2218,33
988986	264614	2253,33	2253,33	2253,33	2253,33	2253,33	2253,33	2253,33	2253,33
989016	264584	2486,67	2486,67	2486,67	2486,67	2486,67	2486,67	2486,67	2486,67
989046	264554	2240	2240	2240	2240	2240	2240	2240	2240
989076	264524	2370	2370	2370	2370	2370	2370	2370	2370
989106	264494	2240	2240	2240	2240	2240	2240	2240	2240
989136	264464	2216,67	2216,67	2216,67	2216,67	2216,67	2216,67	2216,67	2216,67
989166	264434	2230	2230	2230	2230	2230	2230	2230	2230
989196	264404	2191,67	2191,67	2191,67	2191,67	2191,67	2191,67	2191,67	2191,67
989226	264374	2303,33	2303,33	2303,33	2303,33	2303,33	2303,33	2303,33	2303,33
989256	264344	2376,67	2376,67	2376,67	2376,67	2376,67	2376,67	2376,67	2376,67
989286	264314	2378,33	2378,33	2378,33	2378,33	2378,33	2378,33	2378,33	2378,33
989316	264284	2370	2370	2370	2370	2370	2370	2370	2370
989346	264254	2405	2405	2405	2405	2405	2405	2405	2405
989376	264224	2300	2300	2300	2300	2300	2300	2300	2300
989406	264194	2421,67	2421,67	2421,67	2421,67	2421,67	2421,67	2421,67	2421,67
989436	264164	2465	2465	2465	2465	2465	2465	2465	2465
989466	264134	2425	2425	2425	2425	2425	2425	2425	2425
989496	264104	2356,67	2356,67	2356,67	2356,67	2356,67	2356,67	2356,67	2356,67
989526	264074	2446,67	2446,67	2446,67	2446,67	2446,67	2446,67	2446,67	2446,67
989556	264044	2425	2425	2425	2425	2425	2425	2425	2425
989586	264014	2416,67	2416,67	2416,67	2416,67	2416,67	2416,67	2416,67	2416,67
989616	263984	2415	2415	2415	2415	2415	2415	2415	2415
989646	263954	2495	2495	2495	2495	2495	2495	2495	2495
989676	263924	2276,67	2276,67	2276,67	2276,67	2276,67	2276,67	2276,67	2276,67
989706	263894	2405	2405	2405	2405	2405	2405	2405	2405
989736	263864	2360	2360	2360	2360	2360	2360	2360	2360
989766	263834	2415	2415	2415	2415	2415	2415	2415	2415
989796	263804	2355	2355	2355	2355	2355	2355	2355	2355
989826	263774	2443,33	2443,33	2443,33	2443,33	2443,33	2443,33	2443,33	2443,33
989856	263744	2415	2415	2415	2415	2415	2415	2415	2415
989886	263714	2438,33	2438,33	2438,33	2438,33	2438,33	2438,33	2438,33	2438,33
989916	263684	2356,67	2356,67	2356,67	2356,67	2356,67	2356,67	2356,67	2356,67
989946	263654	2233,33	2233,33	2233,33	2233,33	2233,33	2233,33	2233,33	2233,33
989976	263624	2286,67	2286,67	2286,67	2286,67	2286,67	2286,67	2286,67	2286,67
990006	263594	2310	2310	2310	2310	2310	2310	2310	2310
990036	263564	2285	2285	2285	2285	2285	2285	2285	2285
990066	263534	2333,33	2333,33	2333,33	2333,33	2333,33	2333,33	2333,33	2333,33
990096	263504	2296,67	2296,67	2296,67	2296,67	2296,67	2296,67	2296,67	2296,67
990126	263474	2373,33	2373,33	2373,33	2373,33	2373,33	2373,33	2373,33	2373,33
990156	263444	2363,33	2363,33	2363,33	2363,33	2363,33	2363,33	2363,33	2363,33
990186	263414	2393,33	2393,33	2393,33	2393,33	2393,33	2393,33	2393,33	2393,33
990216	263384	2356,67	2356,67	2356,67	2356,67	2356,67	2356,67	2356,67	2356,67
990246	263354	2230	2230	2230	2230	2230	2230	2230	2230

This journal is © The Royal Society of Chemistry 20xx

Please do not adjust margins

Please do not adjust margins

990276	263324	2268,33	2268,33	2268,33	2268,33	2268,33	2268,33	2268,33	2268,33
990306	263294	2315	2315	2315	2315	2315	2315	2315	2315
990336	263264	2230	2230	2230	2230	2230	2230	2230	2230
990366	263234	2246,67	2246,67	2246,67	2246,67	2246,67	2246,67	2246,67	2246,67
990396	263204	2170	2170	2170	2170	2170	2170	2170	2170
990426	263174	2281,67	2281,67	2281,67	2281,67	2281,67	2281,67	2281,67	2281,67
990456	263144	2263,33	2263,33	2263,33	2263,33	2263,33	2263,33	2263,33	2263,33
990486	263114	2096,67	2096,67	2096,67	2096,67	2096,67	2096,67	2096,67	2096,67
990516	263084	2163,33	2163,33	2163,33	2163,33	2163,33	2163,33	2163,33	2163,33
990546	263054	2135	2135	2135	2135	2135	2135	2135	2135
990576	263024	2078,33	2078,33	2078,33	2078,33	2078,33	2078,33	2078,33	2078,33
990606	262994	2168,33	2168,33	2168,33	2168,33	2168,33	2168,33	2168,33	2168,33
990636	262964	2051,67	2051,67	2051,67	2051,67	2051,67	2051,67	2051,67	2051,67
990666	262934	2048,33	2048,33	2048,33	2048,33	2048,33	2048,33	2048,33	2048,33
990696	262904	2055	2055	2055	2055	2055	2055	2055	2055
990726	262874	1960	1960	1960	1960	1960	1960	1960	1960
990756	262844	2058,33	2058,33	2058,33	2058,33	2058,33	2058,33	2058,33	2058,33
990786	262814	1993,33	1993,33	1993,33	1993,33	1993,33	1993,33	1993,33	1993,33
990816	262784	2018,33	2018,33	2018,33	2018,33	2018,33	2018,33	2018,33	2018,33
990846	262754	1988,33	1988,33	1988,33	1988,33	1988,33	1988,33	1988,33	1988,33
990876	262724	1906,67	1906,67	1906,67	1906,67	1906,67	1906,67	1906,67	1906,67
990906	262694	1978,33	1978,33	1978,33	1978,33	1978,33	1978,33	1978,33	1978,33
990936	262664	2010	2010	2010	2010	2010	2010	2010	2010
990966	262634	1925	1925	1925	1925	1925	1925	1925	1925
990996	262604	1901,67	1901,67	1901,67	1901,67	1901,67	1901,67	1901,67	1901,67
991026	262574	1888,33	1888,33	1888,33	1888,33	1888,33	1888,33	1888,33	1888,33
991056	262544	1835	1835	1835	1835	1835	1835	1835	1835
991086	262514	1853,33	1853,33	1853,33	1853,33	1853,33	1853,33	1853,33	1853,33
991116	262484	1891,67	1891,67	1891,67	1891,67	1891,67	1891,67	1891,67	1891,67
991146	262454	1863,33	1863,33	1863,33	1863,33	1863,33	1863,33	1863,33	1863,33
991176	262424	1860	1860	1860	1860	1860	1860	1860	1860
991206	262394	1868,33	1868,33	1868,33	1868,33	1868,33	1868,33	1868,33	1868,33
991236	262364	1800	1800	1800	1800	1800	1800	1800	1800
991266	262334	1923,33	1923,33	1923,33	1923,33	1923,33	1923,33	1923,33	1923,33
991296	262304	1871,67	1871,67	1871,67	1871,67	1871,67	1871,67	1871,67	1871,67
991326	262274	1898,33	1898,33	1898,33	1898,33	1898,33	1898,33	1898,33	1898,33
991356	262244	1846,67	1846,67	1846,67	1846,67	1846,67	1846,67	1846,67	1846,67
991386	262214	1788,33	1788,33	1788,33	1788,33	1788,33	1788,33	1788,33	1788,33
991416	262184	1753,33	1753,33	1753,33	1753,33	1753,33	1753,33	1753,33	1753,33
991446	262154	1838,33	1838,33	1838,33	1838,33	1838,33	1838,33	1838,33	1838,33
991476	262124	1831,67	1831,67	1831,67	1831,67	1831,67	1831,67	1831,67	1831,67
991506	262094	1830	1830	1830	1830	1830	1830	1830	1830
991536	262064	1813,33	1813,33	1813,33	1813,33	1813,33	1813,33	1813,33	1813,33
991566	262034	1876,67	1876,67	1876,67	1876,67	1876,67	1876,67	1876,67	1876,67
991596	262004	1810	1810	1810	1810	1810	1810	1810	1810
991626	261974	1835	1835	1835	1835	1835	1835	1835	1835
991656	261944	1775	1775	1775	1775	1775	1775	1775	1775

This journal is © The Royal Society of Chemistry 20xx

Please do not adjust margins

991686	261914	1641,67	1641,67	1641,67	1641,67	1641,67	1641,67	1641,67	1641,67
991716	261884	1716,67	1716,67	1716,67	1716,67	1716,67	1716,67	1716,67	1716,67
991746	261854	1793,33	1793,33	1793,33	1793,33	1793,33	1793,33	1793,33	1793,33
991776	261824	1841,67	1841,67	1841,67	1841,67	1841,67	1841,67	1841,67	1841,67
991806	261794	1775	1775	1775	1775	1775	1775	1775	1775
991836	261764	1701,67	1701,67	1701,67	1701,67	1701,67	1701,67	1701,67	1701,67
991866	261734	1831,67	1831,67	1831,67	1831,67	1831,67	1831,67	1831,67	1831,67
991896	261704	1641,67	1641,67	1641,67	1641,67	1641,67	1641,67	1641,67	1641,67
991926	261674	1753,33	1753,33	1753,33	1753,33	1753,33	1753,33	1753,33	1753,33
991956	261644	1765	1765	1765	1765	1765	1765	1765	1765
991986	261614	1775	1775	1775	1775	1775	1775	1775	1775
992016	261584	1641,67	1641,67	1641,67	1641,67	1641,67	1641,67	1641,67	1641,67
992046	261554	1736,67	1736,67	1736,67	1736,67	1736,67	1736,67	1736,67	1736,67
992076	261524	1743,33	1743,33	1743,33	1743,33	1743,33	1743,33	1743,33	1743,33
992106	261494	1710	1710	1710	1710	1710	1710	1710	1710
992136	261464	1801,67	1801,67	1801,67	1801,67	1801,67	1801,67	1801,67	1801,67
992166	261434	1773,33	1773,33	1773,33	1773,33	1773,33	1773,33	1773,33	1773,33
992196	261404	1676,67	1676,67	1676,67	1676,67	1676,67	1676,67	1676,67	1676,67
992226	261374	1781,67	1781,67	1781,67	1781,67	1781,67	1781,67	1781,67	1781,67
992256	261344	1715	1715	1715	1715	1715	1715	1715	1715
992286	261314	1745	1745	1745	1745	1745	1745	1745	1745
992316	261284	1740	1740	1740	1740	1740	1740	1740	1740
992346	261254	1738,33	1738,33	1738,33	1738,33	1738,33	1738,33	1738,33	1738,33
992376	261224	1655	1655	1655	1655	1655	1655	1655	1655
992406	261194	1718,33	1718,33	1718,33	1718,33	1718,33	1718,33	1718,33	1718,33
992436	261164	1660	1660	1660	1660	1660	1660	1660	1660
992466	261134	1696,67	1696,67	1696,67	1696,67	1696,67	1696,67	1696,67	1696,67
992496	261104	1716,67	1716,67	1716,67	1716,67	1716,67	1716,67	1716,67	1716,67
992526	261074	1715	1715	1715	1715	1715	1715	1715	1715
992556	261044	1713,33	1713,33	1713,33	1713,33	1713,33	1713,33	1713,33	1713,33
992586	261014	1705	1705	1705	1705	1705	1705	1705	1705
992616	260984	1803,33	1803,33	1803,33	1803,33	1803,33	1803,33	1803,33	1803,33
992646	260954	1706,67	1706,67	1706,67	1706,67	1706,67	1706,67	1706,67	1706,67
992676	260924	1733,33	1733,33	1733,33	1733,33	1733,33	1733,33	1733,33	1733,33
992706	260894	1628,33	1628,33	1628,33	1628,33	1628,33	1628,33	1628,33	1628,33
992736	260864	1663,33	1663,33	1663,33	1663,33	1663,33	1663,33	1663,33	1663,33
992766	260834	1731,67	1731,67	1731,67	1731,67	1731,67	1731,67	1731,67	1731,67
992796	260804	1828,33	1828,33	1828,33	1828,33	1828,33	1828,33	1828,33	1828,33
992826	260774	1641,67	1641,67	1641,67	1641,67	1641,67	1641,67	1641,67	1641,67
992856	260744	1660	1660	1660	1660	1660	1660	1660	1660
992886	260714	1675	1675	1675	1675	1675	1675	1675	1675
992916	260684	1708,33	1708,33	1708,33	1708,33	1708,33	1708,33	1708,33	1708,33
992946	260654	1705	1705	1705	1705	1705	1705	1705	1705
992976	260624	1685	1685	1685	1685	1685	1685	1685	1685
993006	260594	1743,33	1743,33	1743,33	1743,33	1743,33	1743,33	1743,33	1743,33
993036	260564	1743,33	1743,33	1743,33	1743,33	1743,33	1743,33	1743,33	1743,33
993066	260534	1628,33	1628,33	1628,33	1628,33	1628,33	1628,33	1628,33	1628,33

This journal is © The Royal Society of Chemistry 20xx

Please do not adjust margins

993096	260504	1550	1550	1550	1550	1550	1550	1550	1550
993126	260474	1706,67	1706,67	1706,67	1706,67	1706,67	1706,67	1706,67	1706,67
993156	260444	1696,67	1696,67	1696,67	1696,67	1696,67	1696,67	1696,67	1696,67
993186	260414	1773,33	1773,33	1773,33	1773,33	1773,33	1773,33	1773,33	1773,33
993216	260384	1711,67	1711,67	1711,67	1711,67	1711,67	1711,67	1711,67	1711,67
993246	260354	1676,67	1676,67	1676,67	1676,67	1676,67	1676,67	1676,67	1676,67
993276	260324	1638,33	1638,33	1638,33	1638,33	1638,33	1638,33	1638,33	1638,33
993306	260294	1653,33	1653,33	1653,33	1653,33	1653,33	1653,33	1653,33	1653,33
993336	260264	1596,67	1596,67	1596,67	1596,67	1596,67	1596,67	1596,67	1596,67
993366	260234	1593,33	1593,33	1593,33	1593,33	1593,33	1593,33	1593,33	1593,33
993396	260204	1705	1705	1705	1705	1705	1705	1705	1705
993426	260174	1665	1665	1665	1665	1665	1665	1665	1665
993456	260144	1695	1695	1695	1695	1695	1695	1695	1695
993486	260114	1778,33	1778,33	1778,33	1778,33	1778,33	1778,33	1778,33	1778,33
993516	260084	1690	1690	1690	1690	1690	1690	1690	1690
993546	260054	1611,67	1611,67	1611,67	1611,67	1611,67	1611,67	1611,67	1611,67
993576	260024	1710	1710	1710	1710	1710	1710	1710	1710
993606	259994	1703,33	1703,33	1703,33	1703,33	1703,33	1703,33	1703,33	1703,33
993636	259964	1693,33	1693,33	1693,33	1693,33	1693,33	1693,33	1693,33	1693,33
993666	259934	1713,33	1713,33	1713,33	1713,33	1713,33	1713,33	1713,33	1713,33
993696	259904	1731,67	1731,67	1731,67	1731,67	1731,67	1731,67	1731,67	1731,67
993726	259874	1663,33	1663,33	1663,33	1663,33	1663,33	1663,33	1663,33	1663,33
993756	259844	1691,67	1691,67	1691,67	1691,67	1691,67	1691,67	1691,67	1691,67
993786	259814	1708,33	1708,33	1708,33	1708,33	1708,33	1708,33	1708,33	1708,33
993816	259784	1695	1695	1695	1695	1695	1695	1695	1695
993846	259754	1638,33	1638,33	1638,33	1638,33	1638,33	1638,33	1638,33	1638,33
993876	259724	1776,67	1776,67	1776,67	1776,67	1776,67	1776,67	1776,67	1776,67
993906	259694	1643,33	1643,33	1643,33	1643,33	1643,33	1643,33	1643,33	1643,33
993936	259664	1650	1650	1650	1650	1650	1650	1650	1650
993966	259634	1726,67	1726,67	1726,67	1726,67	1726,67	1726,67	1726,67	1726,67
993996	259604	1753,33	1753,33	1753,33	1753,33	1753,33	1753,33	1753,33	1753,33
994026	259574	1601,67	1601,67	1601,67	1601,67	1601,67	1601,67	1601,67	1601,67
994056	259544	1671,67	1671,67	1671,67	1671,67	1671,67	1671,67	1671,67	1671,67
994086	259514	1788,33	1788,33	1788,33	1788,33	1788,33	1788,33	1788,33	1788,33
994116	259484	1740	1740	1740	1740	1740	1740	1740	1740
994146	259454	1610	1610	1610	1610	1610	1610	1610	1610
994176	259424	1700	1700	1700	1700	1700	1700	1700	1700
994206	259394	1670	1670	1670	1670	1670	1670	1670	1670
994236	259364	1598,33	1598,33	1598,33	1598,33	1598,33	1598,33	1598,33	1598,33
994266	259334	1738,33	1738,33	1738,33	1738,33	1738,33	1738,33	1738,33	1738,33
994296	259304	1766,67	1766,67	1766,67	1766,67	1766,67	1766,67	1766,67	1766,67
994326	259274	1691,67	1691,67	1691,67	1691,67	1691,67	1691,67	1691,67	1691,67
994356	259244	1573,33	1573,33	1573,33	1573,33	1573,33	1573,33	1573,33	1573,33
994386	259214	1778,33	1778,33	1778,33	1778,33	1778,33	1778,33	1778,33	1778,33
994416	259184	1670	1670	1670	1670	1670	1670	1670	1670
994446	259154	1680	1680	1680	1680	1680	1680	1680	1680
994476	259124	1800	1800	1800	1800	1800	1800	1800	1800

This journal is © The Royal Society of Chemistry 20xx

Please do not adjust margins

994506	259094	1721,67	1721,67	1721,67	1721,67	1721,67	1721,67	1721,67	1721,67
994536	259064	1676,67	1676,67	1676,67	1676,67	1676,67	1676,67	1676,67	1676,67
994566	259034	1583,33	1583,33	1583,33	1583,33	1583,33	1583,33	1583,33	1583,33
994596	259004	1740	1740	1740	1740	1740	1740	1740	1740
994626	258974	1671,67	1671,67	1671,67	1671,67	1671,67	1671,67	1671,67	1671,67
994656	258944	1640	1640	1640	1640	1640	1640	1640	1640
994686	258914	1661,67	1661,67	1661,67	1661,67	1661,67	1661,67	1661,67	1661,67
994716	258884	1665	1665	1665	1665	1665	1665	1665	1665
994746	258854	1663,33	1663,33	1663,33	1663,33	1663,33	1663,33	1663,33	1663,33
994776	258824	1790	1790	1790	1790	1790	1790	1790	1790
994806	258794	1701,67	1701,67	1701,67	1701,67	1701,67	1701,67	1701,67	1701,67
994836	258764	1595	1595	1595	1595	1595	1595	1595	1595
994866	258734	1648,33	1648,33	1648,33	1648,33	1648,33	1648,33	1648,33	1648,33
994896	258704	1700	1700	1700	1700	1700	1700	1700	1700

Table S5 Raw XPS measurements corresponding to Fig. 2.c.

KE_MgKa	BE_MgKa	CPS_MgKa	C 1s_1_MgKa	C 1s_2_MgKa	C 1s_3_MgKa	C 1s_4_MgKa	Background_MgKa	Envelope_MgKa
955416	298184	2260	2260	2260	2260	2260	2260	2260
955446	298154	2411,67	2411,67	2411,67	2411,67	2411,67	2411,67	2411,67
955476	298124	2408,33	2408,33	2408,33	2408,33	2408,33	2408,33	2408,33
955506	298094	2311,67	2311,67	2311,67	2311,67	2311,67	2311,67	2311,67
955536	298064	2330	2330	2330	2330	2330	2330	2330
955566	298034	2313,33	2313,33	2313,33	2313,33	2313,33	2313,33	2313,33
955596	298004	2233,33	2233,33	2233,33	2233,33	2233,33	2233,33	2233,33
955626	297974	2390	2390	2390	2390	2390	2390	2390
955656	297944	2373,33	2373,33	2373,33	2373,33	2373,33	2373,33	2373,33
955686	297914	2310	2310	2310	2310	2310	2310	2310
955716	297884	2335	2335	2335	2335	2335	2335	2335
955746	297854	2378,33	2378,33	2378,33	2378,33	2378,33	2378,33	2378,33
955776	297824	2401,67	2401,67	2401,67	2401,67	2401,67	2401,67	2401,67
955806	297794	2330	2330	2330	2330	2330	2330	2330
955836	297764	2346,67	2346,67	2346,67	2346,67	2346,67	2346,67	2346,67
955866	297734	2256,67	2256,67	2256,67	2256,67	2256,67	2256,67	2256,67
955896	297704	2231,67	2231,67	2231,67	2231,67	2231,67	2231,67	2231,67
955926	297674	2323,33	2323,33	2323,33	2323,33	2323,33	2323,33	2323,33
955956	297644	2268,33	2268,33	2268,33	2268,33	2268,33	2268,33	2268,33
955986	297614	2268,33	2268,33	2268,33	2268,33	2268,33	2268,33	2268,33
956016	297584	2353,33	2353,33	2353,33	2353,33	2353,33	2353,33	2353,33
956046	297554	2416,67	2416,67	2416,67	2416,67	2416,67	2416,67	2416,67
956076	297524	2210	2210	2210	2210	2210	2210	2210
956106	297494	2225	2225	2225	2225	2225	2225	2225
956136	297464	2323,33	2323,33	2323,33	2323,33	2323,33	2323,33	2323,33
956166	297434	2293,33	2293,33	2293,33	2293,33	2293,33	2293,33	2293,33
956196	297404	2160	2160	2160	2160	2160	2160	2160
956226	297374	2250	2250	2250	2250	2250	2250	2250
956256	297344	2353,33	2353,33	2353,33	2353,33	2353,33	2353,33	2353,33

 This journal is © The Royal Society of Chemistry 20xx

Please do not adjust margins

Please do not adjust margins

956286	297314	2213,33	2213,33	2213,33	2213,33	2213,33	2213,33	2213,33
956316	297284	2273,33	2273,33	2273,33	2273,33	2273,33	2273,33	2273,33
956346	297254	2295	2295	2295	2295	2295	2295	2295
956376	297224	2246,67	2246,67	2246,67	2246,67	2246,67	2246,67	2246,67
956406	297194	2293,33	2293,33	2293,33	2293,33	2293,33	2293,33	2293,33
956436	297164	2253,33	2253,33	2253,33	2253,33	2253,33	2253,33	2253,33
956466	297134	2325	2325	2325	2325	2325	2325	2325
956496	297104	2275	2275	2275	2275	2275	2275	2275
956526	297074	2263,33	2263,33	2263,33	2263,33	2263,33	2263,33	2263,33
956556	297044	2216,67	2216,67	2216,67	2216,67	2216,67	2216,67	2216,67
956586	297014	2340	2340	2340	2340	2340	2340	2340
956616	296984	2240	2240	2240	2240	2240	2240	2240
956646	296954	2286,67	2286,67	2286,67	2286,67	2286,67	2286,67	2286,67
956676	296924	2321,67	2321,67	2321,67	2321,67	2321,67	2321,67	2321,67
956706	296894	2325	2325	2325	2325	2325	2325	2325
956736	296864	2283,33	2283,33	2283,33	2283,33	2283,33	2283,33	2283,33
956766	296834	2195	2195	2195	2195	2195	2195	2195
956796	296804	2198,33	2198,33	2198,33	2198,33	2198,33	2198,33	2198,33
956826	296774	2273,33	2273,33	2273,33	2273,33	2273,33	2273,33	2273,33
956856	296744	2360	2360	2360	2360	2360	2360	2360
956886	296714	2313,33	2313,33	2313,33	2313,33	2313,33	2313,33	2313,33
956916	296684	2238,33	2238,33	2238,33	2238,33	2238,33	2238,33	2238,33
956946	296654	2268,33	2268,33	2268,33	2268,33	2268,33	2268,33	2268,33
956976	296624	2321,67	2321,67	2321,67	2321,67	2321,67	2321,67	2321,67
957006	296594	2225	2225	2225	2225	2225	2225	2225
957036	296564	2313,33	2313,33	2313,33	2313,33	2313,33	2313,33	2313,33
957066	296534	2146,67	2146,67	2146,67	2146,67	2146,67	2146,67	2146,67
957096	296504	2316,67	2316,67	2316,67	2316,67	2316,67	2316,67	2316,67
957126	296474	2206,67	2206,67	2206,67	2206,67	2206,67	2206,67	2206,67
957156	296444	2256,67	2256,67	2256,67	2256,67	2256,67	2256,67	2256,67
957186	296414	2300	2300	2300	2300	2300	2300	2300
957216	296384	2213,33	2213,33	2213,33	2213,33	2213,33	2213,33	2213,33
957246	296354	2236,67	2236,67	2236,67	2236,67	2236,67	2236,67	2236,67
957276	296324	2151,67	2151,67	2151,67	2151,67	2151,67	2151,67	2151,67
957306	296294	2210	2210	2210	2210	2210	2210	2210
957336	296264	2195	2195	2195	2195	2195	2195	2195
957366	296234	2218,33	2218,33	2218,33	2218,33	2218,33	2218,33	2218,33
957396	296204	2168,33	2168,33	2168,33	2168,33	2168,33	2168,33	2168,33
957426	296174	2135	2135	2135	2135	2135	2135	2135
957456	296144	2235	2235	2235	2235	2235	2235	2235
957486	296114	2353,33	2353,33	2353,33	2353,33	2353,33	2353,33	2353,33
957516	296084	2235	2235	2235	2235	2235	2235	2235
957546	296054	2258,33	2258,33	2258,33	2258,33	2258,33	2258,33	2258,33
957576	296024	2158,33	2158,33	2158,33	2158,33	2158,33	2158,33	2158,33
957606	295994	2278,33	2278,33	2278,33	2278,33	2278,33	2278,33	2278,33
957636	295964	2323,33	2323,33	2323,33	2323,33	2323,33	2323,33	2323,33
957666	295934	2230	2230	2230	2230	2230	2230	2230

Please do not adjust margins

957696	295904	2273,33	2273,33	2273,33	2273,33	2273,33	2273,33	2273,33
957726	295874	2336,67	2336,67	2336,67	2336,67	2336,67	2336,67	2336,67
957756	295844	2228,33	2228,33	2228,33	2228,33	2228,33	2228,33	2228,33
957786	295814	2196,67	2196,67	2196,67	2196,67	2196,67	2196,67	2196,67
957816	295784	2208,33	2208,33	2208,33	2208,33	2208,33	2208,33	2208,33
957846	295754	2085	2085	2085	2085	2085	2085	2085
957876	295724	2256,67	2256,67	2256,67	2256,67	2256,67	2256,67	2256,67
957906	295694	2258,33	2258,33	2258,33	2258,33	2258,33	2258,33	2258,33
957936	295664	2261,67	2261,67	2261,67	2261,67	2261,67	2261,67	2261,67
957966	295634	2250	2250	2250	2250	2250	2250	2250
957996	295604	2248,33	2248,33	2248,33	2248,33	2248,33	2248,33	2248,33
958026	295574	2293,33	2293,33	2293,33	2293,33	2293,33	2293,33	2293,33
958056	295544	2150	2150	2150	2150	2150	2150	2150
958086	295514	2321,67	2321,67	2321,67	2321,67	2321,67	2321,67	2321,67
958116	295484	2155	2155	2155	2155	2155	2155	2155
958146	295454	2253,33	2253,33	2253,33	2253,33	2253,33	2253,33	2253,33
958176	295424	2201,67	2201,67	2201,67	2201,67	2201,67	2201,67	2201,67
958206	295394	2170	2170	2170	2170	2170	2170	2170
958236	295364	2198,33	2198,33	2198,33	2198,33	2198,33	2198,33	2198,33
958266	295334	2210	2210	2210	2210	2210	2210	2210
958296	295304	2161,67	2161,67	2161,67	2161,67	2161,67	2161,67	2161,67
958326	295274	2305	2305	2305	2305	2305	2305	2305
958356	295244	2261,67	2261,67	2261,67	2261,67	2261,67	2261,67	2261,67
958386	295214	2101,67	2101,67	2101,67	2101,67	2101,67	2101,67	2101,67
958416	295184	2193,33	2193,33	2193,33	2193,33	2193,33	2193,33	2193,33
958446	295154	2200	2200	2200	2200	2200	2200	2200
958476	295124	2096,67	2096,67	2096,67	2096,67	2096,67	2096,67	2096,67
958506	295094	2210	2210	2210	2210	2210	2210	2210
958536	295064	2150	2150	2150	2150	2150	2150	2150
958566	295034	2145	2145	2145	2145	2145	2145	2145
958596	295004	2243,33	2243,33	2243,33	2243,33	2243,33	2243,33	2243,33
958626	294974	2238,33	2238,33	2238,33	2238,33	2238,33	2238,33	2238,33
958656	294944	2220	2220	2220	2220	2220	2220	2220
958686	294914	2196,67	2196,67	2196,67	2196,67	2196,67	2196,67	2196,67
958716	294884	2285	2285	2285	2285	2285	2285	2285
958746	294854	2063,33	2063,33	2063,33	2063,33	2063,33	2063,33	2063,33
958776	294824	2108,33	2108,33	2108,33	2108,33	2108,33	2108,33	2108,33
958806	294794	2160	2160	2160	2160	2160	2160	2160
958836	294764	2240	2240	2240	2240	2240	2240	2240
958866	294734	2220	2220	2220	2220	2220	2220	2220
958896	294704	2253,33	2253,33	2253,33	2253,33	2253,33	2253,33	2253,33
958926	294674	2146,67	2146,67	2146,67	2146,67	2146,67	2146,67	2146,67
958956	294644	2250	2250	2250	2250	2250	2250	2250
958986	294614	2158,33	2158,33	2158,33	2158,33	2158,33	2158,33	2158,33
959016	294584	2161,67	2161,67	2161,67	2161,67	2161,67	2161,67	2161,67
959046	294554	2221,67	2221,67	2221,67	2221,67	2221,67	2221,67	2221,67
959076	294524	2195	2195	2195	2195	2195	2195	2195

 This journal is © The Royal Society of Chemistry 20xx

959106	294494	2206,67	2206,67	2206,67	2206,67	2206,67	2206,67	2206,67
959136	294464	2126,67	2126,67	2126,67	2126,67	2126,67	2126,67	2126,67
959166	294434	2225	2225	2225	2225	2225	2225	2225
959196	294404	2103,33	2103,33	2103,33	2103,33	2103,33	2103,33	2103,33
959226	294374	2121,67	2121,67	2121,67	2121,67	2121,67	2121,67	2121,67
959256	294344	2233,33	2233,33	2233,33	2233,33	2233,33	2233,33	2233,33
959286	294314	2283,33	2283,33	2283,33	2283,33	2283,33	2283,33	2283,33
959316	294284	2161,67	2161,67	2161,67	2161,67	2161,67	2161,67	2161,67
959346	294254	2148,33	2148,33	2148,33	2148,33	2148,33	2148,33	2148,33
959376	294224	2156,67	2156,67	2156,67	2156,67	2156,67	2156,67	2156,67
959406	294194	2140	2140	2140	2140	2140	2140	2140
959436	294164	2185	2185	2185	2185	2185	2185	2185
959466	294134	2188,33	2188,33	2188,33	2188,33	2188,33	2188,33	2188,33
959496	294104	2138,33	2138,33	2138,33	2138,33	2138,33	2138,33	2138,33
959526	294074	2185	2185	2185	2185	2185	2185	2185
959556	294044	2166,67	2166,67	2166,67	2166,67	2166,67	2166,67	2166,67
959586	294014	2213,33	2170,72	2170,72	2170,72	2170,72	2170,72	2170,72
959616	293984	2148,33	2170,7	2170,7	2170,7	2170,7	2170,7	2170,7
959646	293954	2243,33	2170,64	2170,64	2170,64	2170,64	2170,64	2170,64
959676	293924	2221,67	2170,6	2170,6	2170,6	2170,6	2170,6	2170,6
959706	293894	2048,33	2170,49	2170,49	2170,49	2170,5	2170,49	2170,5
959736	293864	2140	2170,47	2170,47	2170,47	2170,47	2170,47	2170,47
959766	293834	2121,67	2170,43	2170,43	2170,43	2170,43	2170,43	2170,43
959796	293804	2170	2170,43	2170,43	2170,43	2170,43	2170,43	2170,43
959826	293774	2050	2170,33	2170,33	2170,33	2170,33	2170,33	2170,33
959856	293744	2138,33	2170,3	2170,3	2170,3	2170,3	2170,3	2170,3
959886	293714	2141,67	2170,27	2170,27	2170,27	2170,28	2170,27	2170,28
959916	293684	2226,67	2170,23	2170,23	2170,23	2170,23	2170,23	2170,23
959946	293654	2181,67	2170,22	2170,22	2170,22	2170,22	2170,22	2170,22
959976	293624	2166,67	2170,21	2170,21	2170,21	2170,21	2170,21	2170,21
960006	293594	2171,67	2170,21	2170,21	2170,21	2170,21	2170,21	2170,21
960036	293564	2080	2170,14	2170,14	2170,14	2170,14	2170,14	2170,14
960066	293534	2111,67	2170,09	2170,09	2170,09	2170,09	2170,09	2170,09
960096	293504	2135	2170,06	2170,06	2170,06	2170,06	2170,06	2170,06
960126	293474	2326,67	2169,93	2169,93	2169,93	2169,93	2169,93	2169,93
960156	293444	2166,67	2169,92	2169,92	2169,92	2169,92	2169,92	2169,92
960186	293414	2218,33	2169,88	2169,88	2169,88	2169,88	2169,88	2169,88
960216	293384	2193,33	2169,86	2169,86	2169,86	2169,86	2169,86	2169,86
960246	293354	2218,33	2169,82	2169,82	2169,82	2169,82	2169,82	2169,82
960276	293324	2163,33	2169,82	2169,82	2169,82	2169,82	2169,82	2169,82
960306	293294	2171,67	2169,81	2169,81	2169,81	2169,82	2169,81	2169,82
960336	293264	2323,33	2169,68	2169,68	2169,68	2169,69	2169,68	2169,69
960366	293234	2178,33	2169,68	2169,68	2169,68	2169,68	2169,68	2169,68
960396	293204	2210	2169,64	2169,64	2169,64	2169,65	2169,64	2169,65
960426	293174	2231,67	2169,59	2169,59	2169,59	2169,6	2169,59	2169,6
960456	293144	2210	2169,56	2169,56	2169,56	2169,56	2169,56	2169,56
960486	293114	2253,33	2169,49	2169,49	2169,49	2169,49	2169,49	2169,49

Please do not adjust margins

960516	293084	2118,33	2169,44	2169,44	2169,44	2169,45	2169,44	2169,45
960546	293054	2245	2169,38	2169,38	2169,38	2169,39	2169,38	2169,39
960576	293024	2053,33	2169,28	2169,28	2169,28	2169,29	2169,28	2169,29
960606	292994	2258,33	2169,21	2169,21	2169,21	2169,22	2169,21	2169,22
960636	292964	2140	2169,18	2169,18	2169,18	2169,19	2169,18	2169,19
960666	292934	2290	2169,08	2169,08	2169,08	2169,09	2169,08	2169,09
960696	292904	2246,67	2169,01	2169,01	2169,01	2169,03	2169,01	2169,03
960726	292874	2268,33	2168,93	2168,93	2168,93	2168,95	2168,93	2168,95
960756	292844	2156,67	2168,92	2168,92	2168,92	2168,94	2168,92	2168,94
960786	292814	2313,33	2168,8	2168,8	2168,8	2168,82	2168,8	2168,82
960816	292784	2175	2168,79	2168,79	2168,79	2168,82	2168,79	2168,82
960846	292754	2163,33	2168,79	2168,79	2168,79	2168,82	2168,79	2168,82
960876	292724	2183,33	2168,77	2168,77	2168,77	2168,81	2168,77	2168,81
960906	292694	2118,33	2168,73	2168,73	2168,73	2168,77	2168,73	2168,77
960936	292664	2228,33	2168,68	2168,68	2168,68	2168,72	2168,68	2168,72
960966	292634	2211,67	2168,64	2168,64	2168,65	2168,69	2168,64	2168,69
960996	292604	2163,33	2168,64	2168,64	2168,64	2168,69	2168,64	2168,69
961026	292574	2198,33	2168,62	2168,62	2168,62	2168,68	2168,62	2168,68
961056	292544	2316,67	2168,49	2168,49	2168,49	2168,56	2168,49	2168,56
961086	292514	2245	2168,43	2168,43	2168,43	2168,5	2168,43	2168,5
961116	292484	2176,67	2168,42	2168,42	2168,42	2168,5	2168,42	2168,51
961146	292454	2205	2168,39	2168,39	2168,39	2168,48	2168,39	2168,49
961176	292424	2188,33	2168,37	2168,37	2168,37	2168,48	2168,37	2168,48
961206	292394	2246,67	2168,3	2168,3	2168,31	2168,43	2168,3	2168,43
961236	292364	2211,67	2168,27	2168,27	2168,27	2168,41	2168,27	2168,41
961266	292334	2210	2168,23	2168,23	2168,23	2168,39	2168,23	2168,39
961296	292304	2195	2168,21	2168,21	2168,21	2168,38	2168,21	2168,39
961326	292274	2220	2168,17	2168,17	2168,17	2168,36	2168,17	2168,36
961356	292244	2166,67	2168,16	2168,16	2168,17	2168,38	2168,16	2168,38
961386	292214	2151,67	2168,15	2168,15	2168,15	2168,39	2168,15	2168,4
961416	292184	2218,33	2168,11	2168,11	2168,11	2168,38	2168,11	2168,38
961446	292154	2200	2168,08	2168,08	2168,09	2168,39	2168,08	2168,39
961476	292124	2253,33	2168,01	2168,01	2168,01	2168,35	2168,01	2168,35
961506	292094	2156,67	2168	2168	2168	2168,38	2168	2168,38
961536	292064	2043,33	2167,89	2167,89	2167,9	2168,32	2167,89	2168,32
961566	292034	2235	2167,84	2167,84	2167,84	2168,31	2167,84	2168,31
961596	292004	2301,67	2167,72	2167,72	2167,73	2168,25	2167,72	2168,26
961626	291974	2158,33	2167,72	2167,72	2167,73	2168,3	2167,72	2168,31
961656	291944	2145	2167,7	2167,7	2167,71	2168,34	2167,7	2168,35
961686	291914	2165	2167,7	2167,7	2167,71	2168,42	2167,7	2168,43
961716	291884	2165	2167,69	2167,69	2167,71	2168,49	2167,69	2168,51
961746	291854	2125	2167,66	2167,66	2167,67	2168,54	2167,66	2168,56
961776	291824	2226,67	2167,61	2167,61	2167,62	2168,59	2167,61	2168,61
961806	291794	2246,67	2167,54	2167,54	2167,56	2168,63	2167,54	2168,65
961836	291764	2091,67	2167,48	2167,48	2167,5	2168,68	2167,48	2168,7
961866	291734	2231,67	2167,42	2167,42	2167,45	2168,75	2167,42	2168,78
961896	291704	2240	2167,36	2167,36	2167,39	2168,83	2167,36	2168,86

 This journal is © The Royal Society of Chemistry 20xx

Please do not adjust margins

Please do not adjust margins

961926	291674	2266,67	2167,28	2167,28	2167,31	2168,9	2167,28	2168,93
961956	291644	2106,67	2167,23	2167,23	2167,26	2169,02	2167,23	2169,05
961986	291614	2176,67	2167,22	2167,22	2167,26	2169,2	2167,22	2169,24
962016	291584	2195	2167,19	2167,19	2167,24	2169,38	2167,19	2169,42
962046	291554	2168,33	2167,19	2167,19	2167,24	2169,59	2167,19	2169,65
962076	291524	2116,67	2167,15	2167,15	2167,21	2169,8	2167,15	2169,85
962106	291494	2195	2167,13	2167,13	2167,19	2170,04	2167,13	2170,1
962136	291464	2100	2167,07	2167,07	2167,14	2170,27	2167,07	2170,34
962166	291434	2271,67	2166,98	2166,98	2167,06	2170,49	2166,98	2170,58
962196	291404	2235	2166,92	2166,92	2167,02	2170,78	2166,92	2170,87
962226	291374	2241,67	2166,86	2166,86	2166,97	2171,09	2166,86	2171,19
962256	291344	2188,33	2166,84	2166,84	2166,96	2171,47	2166,84	2171,59
962286	291314	2268,33	2166,76	2166,76	2166,89	2171,83	2166,76	2171,97
962316	291284	2128,33	2166,72	2166,73	2166,87	2172,28	2166,72	2172,43
962346	291254	2171,67	2166,72	2166,72	2166,89	2172,79	2166,72	2172,96
962376	291224	2233,33	2166,66	2166,66	2166,85	2173,31	2166,66	2173,49
962406	291194	2220	2166,62	2166,62	2166,83	2173,87	2166,62	2174,08
962436	291164	2213,33	2166,58	2166,58	2166,81	2174,49	2166,58	2174,73
962466	291134	2245	2166,51	2166,51	2166,78	2175,14	2166,51	2175,41
962496	291104	2201,67	2166,48	2166,48	2166,78	2175,9	2166,48	2176,19
962526	291074	2243,33	2166,42	2166,42	2166,75	2176,67	2166,42	2177
962556	291044	2251,67	2166,35	2166,35	2166,71	2177,49	2166,35	2177,86
962586	291014	2263,33	2166,26	2166,27	2166,67	2178,4	2166,26	2178,81
962616	290984	2215	2166,22	2166,23	2166,68	2179,41	2166,22	2179,87
962646	290954	2225	2166,17	2166,18	2166,68	2180,48	2166,17	2180,99
962676	290924	2210	2166,14	2166,14	2166,7	2181,69	2166,14	2182,26
962706	290894	2155	2166,13	2166,13	2166,76	2183	2166,13	2183,63
962736	290864	2271,67	2166,04	2166,04	2166,74	2184,3	2166,04	2185,01
962766	290834	2208,33	2166	2166,01	2166,78	2185,8	2166	2186,59
962796	290804	2181,67	2165,99	2165,99	2166,85	2187,43	2165,99	2188,3
962826	290774	2096,67	2165,93	2165,94	2166,89	2189,11	2165,93	2190,07
962856	290744	2228,33	2165,88	2165,89	2166,94	2190,93	2165,88	2192
962886	290714	2200	2165,85	2165,86	2167,03	2192,93	2165,85	2194,12
962916	290684	2166,67	2165,85	2165,86	2167,15	2195,07	2165,85	2196,38
962946	290654	2223,33	2165,8	2165,81	2167,25	2197,3	2165,8	2198,76
962976	290624	2125	2165,76	2165,78	2167,36	2199,75	2165,76	2201,36
963006	290594	2283,33	2165,67	2165,68	2167,43	2202,28	2165,67	2204,05
963036	290564	2181,67	2165,65	2165,67	2167,6	2205,02	2165,65	2206,99
963066	290534	2198,33	2165,62	2165,64	2167,78	2208,02	2165,62	2210,19
963096	290504	2260	2165,54	2165,57	2167,91	2211,12	2165,54	2213,51
963126	290474	2281,67	2165,45	2165,47	2168,06	2214,37	2165,45	2217,01
963156	290444	2171,67	2165,44	2165,47	2168,32	2217,98	2165,44	2220,89
963186	290414	2241,67	2165,38	2165,41	2168,54	2221,76	2165,38	2224,96
963216	290384	2343,33	2165,23	2165,27	2168,7	2225,64	2165,23	2229,16
963246	290354	2268,33	2165,14	2165,18	2168,96	2229,84	2165,14	2233,71
963276	290324	2221,67	2165,09	2165,14	2169,29	2234,39	2165,09	2238,64
963306	290294	2276,67	2165	2165,05	2169,6	2239,11	2165	2243,76

Please do not adjust margins

Please do not adjust margins

963336	290264	2280	2164,9	2164,97	2169,95	2244,06	2164,9	2249,18
963366	290234	2281,67	2164,8	2164,88	2170,33	2249,41	2164,8	2255,02
963396	290204	2366,67	2164,63	2164,71	2170,68	2254,94	2164,63	2261,07
963426	290174	2510	2164,34	2164,43	2170,96	2260,59	2164,34	2267,31
963456	290144	2396,67	2164,14	2164,25	2171,39	2266,76	2164,14	2274,11
963486	290114	2293,33	2164,03	2164,16	2171,94	2273,33	2164,03	2281,35
963516	290084	2350	2163,88	2164,02	2172,51	2280,12	2163,88	2288,89
963546	290054	2361,67	2163,71	2163,87	2173,14	2287,3	2163,71	2296,89
963576	290024	2361,67	2163,54	2163,72	2173,81	2294,88	2163,54	2305,33
963606	289994	2351,67	2163,38	2163,58	2174,57	2302,76	2163,38	2314,15
963636	289964	2420	2163,17	2163,39	2175,36	2310,95	2163,16	2323,37
963666	289934	2288,33	2163,06	2163,32	2176,32	2319,73	2163,06	2333,25
963696	289904	2388,33	2162,87	2163,16	2177,27	2328,74	2162,87	2343,43
963726	289874	2426,67	2162,65	2162,97	2178,31	2338,03	2162,65	2354,02
963756	289844	2343,33	2162,49	2162,86	2179,5	2347,94	2162,49	2365,32
963786	289814	2393,33	2162,3	2162,71	2180,73	2358,13	2162,3	2376,98
963816	289784	2353,33	2162,14	2162,6	2182,14	2368,67	2162,14	2389,14
963846	289754	2330	2162	2162,52	2183,68	2379,7	2161,99	2401,91
963876	289724	2470	2161,74	2162,32	2185,2	2390,99	2161,73	2415,05
963906	289694	2468,33	2161,48	2162,13	2186,87	2402,58	2161,47	2428,63
963936	289664	2496,67	2161,19	2161,93	2188,67	2414,53	2161,19	2442,74
963966	289634	2448,33	2160,95	2161,77	2190,62	2426,92	2160,95	2457,42
963996	289604	2443,33	2160,71	2161,63	2192,74	2439,56	2160,71	2472,51
964026	289574	2508,33	2160,42	2161,45	2195	2452,43	2160,42	2488,05
964056	289544	2456,67	2160,17	2161,32	2197,46	2465,71	2160,16	2504,16
964086	289514	2510	2159,87	2161,15	2200,01	2479,13	2159,87	2520,56
964116	289484	2491,67	2159,59	2161,02	2202,86	2492,74	2159,59	2537,46
964146	289454	2610	2159,21	2160,81	2205,78	2506,49	2159,21	2554,67
964176	289424	2598,33	2158,84	2160,61	2208,88	2520,35	2158,84	2572,17
964206	289394	2641,67	2158,44	2160,41	2212,22	2534,24	2158,43	2590,02
964236	289364	2666,67	2158,01	2160,21	2215,79	2548,15	2158	2608,16
964266	289334	2710	2157,54	2159,98	2219,51	2561,99	2157,53	2626,43
964296	289304	2611,67	2157,16	2159,87	2223,6	2575,84	2157,15	2645,02
964326	289274	2620	2156,77	2159,78	2228	2589,55	2156,76	2663,83
964356	289244	2753,33	2156,27	2159,6	2232,53	2602,86	2156,25	2682,5
964386	289214	2711,67	2155,8	2159,49	2237,35	2615,96	2155,78	2701,26
964416	289184	2720	2155,33	2159,42	2242,58	2628,79	2155,31	2720,2
964446	289154	2728,33	2154,85	2159,38	2248,07	2640,99	2154,82	2738,82
964476	289124	2625	2154,45	2159,45	2253,91	2652,84	2154,42	2757,38
964506	289094	2716,67	2153,98	2159,51	2260,14	2664,14	2153,95	2775,94
964536	289064	2578,33	2153,63	2159,74	2266,82	2674,75	2153,59	2794,16
964566	289034	2861,67	2153,03	2159,77	2273,55	2684,36	2152,99	2811,74
964596	289004	2831,67	2152,47	2159,9	2280,75	2693,34	2152,42	2829,21
964626	288974	2815	2151,91	2160,11	2288,39	2701,41	2151,86	2846,26
964656	288944	2765	2151,4	2160,41	2296,4	2708,42	2151,34	2862,62
964686	288914	2868,33	2150,81	2160,72	2304,73	2714,52	2150,73	2878,58
964716	288884	2855	2150,22	2161,13	2313,6	2719,7	2150,14	2894,24

This journal is © The Royal Society of Chemistry 20xx

Please do not adjust margins

Please do not adjust margins

964746	288854	2765	2149,72	2161,69	2322,89	2723,48	2149,62	2908,92
964776	288824	2963,33	2149,04	2162,17	2332,36	2726,16	2148,93	2922,95
964806	288794	2861,67	2148,45	2162,88	2342,55	2728,01	2148,33	2936,92
964836	288764	2891,67	2147,84	2163,66	2353,07	2728,23	2147,7	2949,71
964866	288734	2978,33	2147,16	2164,46	2363,87	2727,33	2146,99	2961,83
964896	288704	2896,67	2146,55	2165,51	2375,3	2725,53	2146,36	2973,81
964926	288674	3005	2145,85	2166,6	2387,06	2722,26	2145,63	2984,87
964956	288644	3060	2145,1	2167,77	2399,13	2717,71	2144,86	2995,13
964986	288614	2981,67	2144,43	2169,19	2411,73	2712,32	2144,15	3005,2
965016	288584	3043,33	2143,7	2170,76	2424,74	2705,79	2143,39	3014,81
965046	288554	2980	2143,04	2172,53	2438,12	2698,02	2142,69	3023,65
965076	288524	3096,67	2142,28	2174,4	2451,76	2689,36	2141,88	3032,16
965106	288494	3095	2141,52	2176,56	2465,86	2679,92	2141,07	3040,64
965136	288464	2950	2140,9	2179,03	2480,33	2669,38	2140,39	3048,47
965166	288434	3108,33	2140,14	2181,56	2494,92	2658,09	2139,57	3056
965196	288404	3046,67	2139,44	2184,53	2509,91	2646,25	2138,8	3063,73
965226	288374	3135	2138,68	2187,67	2525,01	2633,51	2137,96	3070,98
965256	288344	3155	2137,92	2191,03	2540,22	2620,22	2137,1	3078,08
965286	288314	3071,67	2137,22	2194,87	2555,66	2606,6	2136,31	3085,41
965316	288284	3108,33	2136,52	2199,03	2571,11	2592,47	2135,49	3092,66
965346	288254	3093,33	2135,83	2203,51	2586,57	2577,97	2134,68	3099,84
965376	288224	3148,33	2135,12	2208,35	2601,94	2563,22	2133,82	3107,16
965406	288194	3190	2134,38	2213,66	2617,09	2548,22	2132,93	3114,56
965436	288164	3218,33	2133,64	2219,3	2632,04	2533,03	2132,01	3121,96
965466	288134	3036,67	2133,06	2225,51	2646,92	2517,95	2131,25	3129,7
965496	288104	3220	2132,35	2232,24	2661,12	2502,69	2130,33	3137,42
965526	288074	3190	2131,7	2239,43	2674,95	2487,5	2129,43	3145,29
965556	288044	3308,33	2130,97	2246,97	2688,26	2472,32	2128,43	3153,21
965586	288014	3211,67	2130,34	2255,38	2700,86	2457,32	2127,52	3161,34
965616	287984	3210	2129,75	2264,36	2712,75	2442,52	2126,6	3169,57
965646	287954	3140	2129,26	2273,93	2724,06	2428,03	2125,75	3178,03
965676	287924	3161,67	2128,78	2284,28	2734,39	2413,72	2124,87	3186,55
965706	287894	3270	2128,24	2295,33	2743,51	2399,59	2123,9	3194,97
965736	287864	3150	2127,86	2307,1	2751,9	2385,94	2123,03	3203,7
965766	287834	3150	2127,53	2319,66	2759,28	2372,56	2122,17	3212,53
965796	287804	3175	2127,22	2333,25	2765,09	2359,44	2121,28	3221,17
965826	287774	3108,33	2127,04	2347,62	2769,96	2346,88	2120,44	3230,17
965856	287744	3123,33	2126,91	2362,7	2773,8	2334,61	2119,59	3239,25
965886	287714	3088,33	2126,88	2379,19	2775,87	2322,67	2118,77	3248,29
965916	287684	3288,33	2126,74	2396,33	2776,62	2311,06	2117,79	3257,4
965946	287654	3331,67	2126,68	2414,27	2776,25	2299,78	2116,76	3266,7
965976	287624	3326,67	2126,7	2433,57	2774,26	2288,82	2115,74	3276,14
966006	287594	3303,33	2126,82	2454,01	2770,89	2278,32	2114,73	3285,83
966036	287564	3325	2127,06	2475,35	2766,44	2268,23	2113,71	3295,95
966066	287534	3253,33	2127,47	2498,06	2760,71	2258,51	2112,75	3306,51
966096	287504	3208,33	2128,03	2522,24	2753,5	2249,18	2111,82	3317,49
966126	287474	3420	2128,56	2547,27	2745,14	2240,16	2110,71	3328,99

This journal is © The Royal Society of Chemistry 20xx

Please do not adjust margins

Please do not adjust margins

966156	287444	3345	2129,32	2573,55	2735,85	2231,49	2109,67	3341,21
966186	287414	3506,67	2130,09	2601,47	2724,91	2222,98	2108,49	3353,99
966216	287384	3350	2131,13	2630,64	2713,31	2215,08	2107,44	3367,85
966246	287354	3336,67	2132,45	2660,94	2700,94	2207,48	2106,4	3382,62
966276	287324	3460	2133,84	2693,06	2687,35	2200,05	2105,25	3398,54
966306	287294	3440	2135,43	2726,47	2673,07	2193,01	2104,13	3415,6
966336	287264	3386,67	2137,34	2761,08	2658,28	2186,34	2103,04	3433,91
966366	287234	3350	2139,57	2797,44	2642,82	2179,93	2101,99	3453,79
966396	287204	3368,33	2142	2835,31	2626,79	2173,81	2100,92	3475,16
966426	287174	3376,67	2144,71	2874,34	2610,42	2168,01	2099,84	3497,96
966456	287144	3588,33	2147,65	2914,62	2593,51	2162,25	2098,58	3522,29
966486	287114	3573,33	2150,9	2956,64	2576,28	2156,71	2097,33	3548,54
966516	287084	3518,33	2154,49	2999,8	2558,97	2151,56	2096,13	3576,42
966546	287054	3523,33	2158,58	3044,05	2541,53	2146,59	2094,92	3605,99
966576	287024	3765	2162,88	3089,88	2523,83	2141,6	2093,51	3637,66
966606	286994	3683,33	2167,63	3136,7	2506,23	2136,95	2092,17	3671,01
966636	286964	3795	2172,78	3184,33	2488,59	2132,39	2090,73	3705,91
966666	286934	3733,33	2178,6	3233,31	2471,16	2128,04	2089,34	3743,1
966696	286904	3810	2184,81	3283,04	2453,87	2123,83	2087,88	3781,9
966726	286874	3848,33	2191,46	3333,35	2436,72	2119,78	2086,4	3822,13
966756	286844	3916,67	2198,94	3384,32	2419,78	2115,8	2084,85	3864,3
966786	286814	3925	2206,98	3435,75	2403,21	2111,96	2083,29	3908,02
966816	286784	4158,33	2215,39	3487,18	2386,71	2108,12	2081,54	3952,79
966846	286754	4115	2224,71	3538,79	2370,55	2104,42	2079,82	3999,02
966876	286724	3915	2235,06	3590,36	2355,09	2101	2078,27	4046,71
966906	286694	4118,33	2245,94	3641,41	2339,78	2097,57	2076,54	4095,09
966936	286664	4106,67	2257,65	3692,07	2324,82	2094,25	2074,83	4144,31
966966	286634	4215	2270,5	3741,47	2310,31	2090,93	2073,02	4194,17
966996	286604	4288,33	2284,12	3789,76	2296,15	2087,68	2071,14	4244,29
967026	286574	4180	2298,69	3837,06	2282,44	2084,61	2069,36	4294,72
967056	286544	4320	2314,56	3882,3	2269,11	2081,5	2067,46	4345,09
967086	286514	4425	2331,47	3925,33	2256,17	2078,38	2065,46	4394,97
967116	286484	4395	2349,41	3966,59	2243,62	2075,39	2063,49	4444,53
967146	286454	4581,67	2368,53	4005,2	2231,33	2072,3	2061,36	4493,26
967176	286424	4501,67	2389,33	4040,42	2219,66	2069,33	2059,3	4540,84
967206	286394	4628,33	2411,2	4073,03	2208,22	2066,35	2057,13	4587,42
967236	286364	4681,67	2434,23	4102,9	2197,08	2063,37	2054,91	4632,84
967266	286334	4588,33	2459,5	4127,87	2186,59	2060,51	2052,77	4676,17
967296	286304	4666,67	2486,07	4149,69	2176,36	2057,66	2050,56	4718,1
967326	286274	4796,67	2513,91	4168,25	2166,34	2054,74	2048,24	4758,53
967356	286244	4833,33	2543,73	4181,49	2156,75	2051,82	2045,88	4796,16
967386	286214	4636,67	2575,67	4190,85	2147,7	2049,12	2043,69	4832,28
967416	286184	5016,67	2608,85	4196,4	2138,61	2046,15	2041,18	4866,48
967446	286154	4806,67	2643,98	4197,23	2130,06	2043,37	2038,84	4898,13
967476	286124	4896,67	2681,65	4193,01	2121,83	2040,55	2036,42	4927,79
967506	286094	5138,33	2720,84	4185,04	2113,64	2037,57	2033,8	4955,69
967536	286064	4888,33	2761,96	4173,16	2105,94	2034,82	2031,39	4981,72

 This journal is © The Royal Society of Chemistry 20xx

Please do not adjust margins

Please do not adjust margins

967566	286034	5016,67	2805,73	4155,6	2098,53	2031,98	2028,86	5005,26
967596	286004	5093,33	2851,57	4134,66	2091,27	2029,12	2026,27	5027,81
967626	285974	5060	2899,29	4110,45	2084,26	2026,29	2023,7	5049,18
967656	285944	5023,33	2949,42	4080,92	2077,62	2023,52	2021,17	5067,97
967686	285914	4991,67	3002,33	4048,4	2071,23	2020,79	2018,66	5086,78
967716	285884	5051,67	3057,11	4013,04	2064,98	2018,03	2016,09	5104,88
967746	285854	4966,67	3113,88	3973,86	2059,05	2015,35	2013,6	5121,36
967776	285824	4998,33	3173,88	3931,74	2053,33	2012,66	2011,07	5138,38
967806	285794	5041,67	3235,77	3887,46	2047,73	2009,95	2008,51	5155,37
967836	285764	5210	3299,4	3840,52	2042,17	2007,11	2005,8	5171,78
967866	285734	5265	3365,58	3791,08	2036,79	2004,22	2003,05	5188,53
967896	285704	5288,33	3434,06	3740,22	2031,53	2001,33	2000,27	5206,33
967926	285674	5030	3504,46	3688,2	2026,62	1998,67	1997,71	5224,82
967956	285644	5228,33	3576,55	3634,31	2021,76	1995,84	1994,98	5243,53
967986	285614	5285	3651,03	3579,68	2016,96	1992,98	1992,2	5264,05
968016	285584	5398,33	3726,77	3524,32	2012,18	1990,02	1989,32	5285,33
968046	285554	5555	3803,68	3468,19	2007,42	1986,93	1986,3	5307,33
968076	285524	5478,33	3882,39	3411,99	2002,85	1983,92	1983,35	5331,09
968106	285494	5263,33	3962,28	3355,94	1998,54	1981,09	1980,58	5356,12
968136	285464	5376,67	4042,79	3299,96	1994,26	1978,16	1977,7	5382,05
968166	285434	5306,67	4124,04	3244,5	1990,14	1975,3	1974,89	5409,32
968196	285404	5556,67	4205,38	3189,27	1985,89	1972,23	1971,86	5437,19
968226	285374	5430	4286,72	3134,76	1981,83	1969,27	1968,94	5465,76
968256	285344	5590	4367,71	3081,21	1977,73	1966,17	1965,88	5495,19
968286	285314	5596,67	4447,62	3028,44	1973,69	1963,07	1962,81	5524,4
968316	285284	5721,67	4526,27	2976,38	1969,61	1959,86	1959,63	5553,24
968346	285254	5560	4603,78	2926,07	1965,74	1956,79	1956,58	5582,64
968376	285224	5671,67	4678,94	2876,72	1961,84	1953,63	1953,44	5610,8
968406	285194	5766,67	4751,19	2828,31	1957,9	1950,39	1950,22	5637,13
968436	285164	5686,67	4821,02	2781,6	1954,09	1947,21	1947,06	5662,75
968466	285134	5578,33	4887,97	2736,4	1950,42	1944,12	1943,99	5686,96
968496	285104	5715	4949,62	2692,23	1946,68	1940,92	1940,8	5707,04
968526	285074	5903,33	5007,4	2649,38	1942,81	1937,55	1937,45	5724,81
968556	285044	5570	5061,62	2608,55	1939,28	1934,47	1934,38	5740,79
968586	285014	5863,33	5109,09	2568,64	1935,52	1931,14	1931,06	5751,22
968616	284984	5735	5151,09	2530,09	1931,9	1927,91	1927,84	5757,48
968646	284954	5671,67	5188,25	2493,57	1928,38	1924,74	1924,67	5760,91
968676	284924	5695	5219,04	2458,16	1924,86	1921,54	1921,49	5759,16
968706	284894	5686,67	5241,87	2423,9	1921,37	1918,35	1918,3	5750,59
968736	284864	5586,67	5259,17	2391,55	1917,99	1915,25	1915,2	5738,35
968766	284834	5698,33	5270,69	2360,34	1914,53	1912,04	1912	5721,61
968796	284804	5623,33	5272,58	2330,26	1911,16	1908,9	1908,86	5696,31
968826	284774	5488,33	5268,45	2301,78	1907,92	1905,87	1905,84	5666,51
968856	284744	5586,67	5258,39	2274,58	1904,61	1902,75	1902,72	5632,16
968886	284714	5426,67	5240,42	2248,49	1901,45	1899,77	1899,75	5590,9
968916	284684	5468,33	5214,88	2223,51	1898,27	1896,75	1896,73	5543,23
968946	284654	5450	5183,83	2200	1895,12	1893,74	1893,72	5491,53

This journal is © The Royal Society of Chemistry 20xx

Please do not adjust margins

968976	284624	5463,33	5146,83	2177,35	1891,97	1890,72	1890,71	5434,76
969006	284594	5511,67	5101,52	2155,53	1888,78	1887,66	1887,64	5370,56
969036	284564	5335	5051,62	2135,3	1885,76	1884,74	1884,73	5303,23
969066	284534	5166,67	4997,24	2116	1882,88	1881,97	1881,95	5232,23
969096	284504	5231,67	4936,34	2097,41	1879,96	1879,13	1879,12	5155,48
969126	284474	5003,33	4871,15	2080,13	1877,23	1876,49	1876,48	5075,56
969156	284444	5001,67	4802,47	2063,7	1874,52	1873,85	1873,84	4993,01
969186	284414	4886,67	4729,89	2048,02	1871,9	1871,3	1871,29	4907,24
969216	284384	4940	4653,3	2033,14	1869,24	1868,7	1868,7	4818,29
969246	284354	4720	4574,61	2019,31	1866,78	1866,29	1866,28	4728,13
969276	284324	4725	4493,8	2006,04	1864,31	1863,87	1863,87	4636,42
969306	284294	4773,33	4410,27	1993,34	1861,8	1861,41	1861,41	4542,6
969336	284264	4446,67	4325,83	1981,78	1859,58	1859,23	1859,22	4448,75
969366	284234	4391,67	4240,49	1970,74	1857,4	1857,08	1857,08	4354,47
969396	284204	4346,67	4154,32	1960,21	1855,26	1854,98	1854,98	4259,84
969426	284174	4245	4067,96	1950,49	1853,21	1852,96	1852,95	4165,75
969456	284144	4083,33	3981,78	1941,36	1851,3	1851,07	1851,07	4072,31
969486	284114	4120	3895,79	1932,6	1849,35	1849,15	1849,15	3979,45
969516	284084	3901,67	3810,88	1924,55	1847,59	1847,42	1847,41	3888,2
969546	284054	3818,33	3726,88	1917,03	1845,91	1845,75	1845,75	3798,32
969576	284024	3770	3643,82	1909,88	1844,26	1844,12	1844,12	3709,73
969606	283994	3735	3562,49	1903,14	1842,65	1842,52	1842,52	3623,23
969636	283964	3498,33	3482,97	1897,05	1841,24	1841,12	1841,12	3539,01
969666	283934	3356,67	3405	1891,34	1839,94	1839,84	1839,84	3456,61
969696	283904	3328,33	3328,92	1885,92	1838,67	1838,58	1838,58	3376,35
969726	283874	3250	3255,39	1880,99	1837,47	1837,39	1837,39	3299,07
969756	283844	3225	3183,63	1876,3	1836,29	1836,22	1836,22	3223,78
969786	283814	3143,33	3113,71	1871,89	1835,17	1835,11	1835,11	3150,55
969816	283784	3008,33	3046,95	1867,9	1834,17	1834,12	1834,12	3080,79
969846	283754	3093,33	2982,14	1864,05	1833,1	1833,05	1833,05	3013,19
969876	283724	2923,33	2919,38	1860,53	1832,18	1832,13	1832,13	2947,82
969906	283694	2861,67	2859,29	1857,26	1831,3	1831,26	1831,26	2885,33
969936	283664	2828,33	2801,82	1854,24	1830,45	1830,42	1830,42	2825,68
969966	283634	2626,67	2746,44	1851,53	1829,78	1829,75	1829,75	2768,26
969996	283604	2588,33	2693,14	1848,99	1829,13	1829,1	1829,1	2713,06
970026	283574	2706,67	2642,82	1846,56	1828,39	1828,36	1828,36	2661,04
970056	283544	2535	2594,48	1844,37	1827,78	1827,76	1827,76	2611,11
970086	283514	2531,67	2547,97	1842,29	1827,19	1827,17	1827,17	2563,11
970116	283484	2503,33	2504,15	1840,4	1826,61	1826,6	1826,6	2517,97
970146	283454	2423,33	2462,49	1838,67	1826,11	1826,09	1826,09	2475,08
970176	283424	2426,67	2422,51	1837,02	1825,6	1825,59	1825,59	2433,96
970206	283394	2291,67	2384,65	1835,6	1825,2	1825,19	1825,19	2395,06
970236	283364	2443,33	2349,03	1834,14	1824,68	1824,67	1824,67	2358,51
970266	283334	2328,33	2315,03	1832,84	1824,25	1824,24	1824,24	2323,63
970296	283304	2236,67	2282,61	1831,69	1823,9	1823,89	1823,89	2290,42
970326	283274	2253,33	2252,52	1830,61	1823,54	1823,53	1823,53	2259,61
970356	283244	2181,67	2223,95	1829,64	1823,23	1823,23	1823,23	2230,37

This journal is © The Royal Society of Chemistry 20xx

Please do not adjust margins

970386	283214	2268,33	2196,62	1828,65	1822,86	1822,85	1822,85	2202,43
970416	283184	2168,33	2171,1	1827,82	1822,56	1822,56	1822,56	2176,37
970446	283154	2086,67	2147,28	1827,09	1822,34	1822,34	1822,34	2152,05
970476	283124	2151,67	2124,56	1826,35	1822,06	1822,06	1822,06	2128,86
970506	283094	1926,67	2103,22	1825,85	1821,97	1821,97	1821,97	2107,11
970536	283064	2003,33	2083,55	1825,32	1821,82	1821,82	1821,82	2087,06
970566	283034	2135	2064,76	1824,71	1821,55	1821,55	1821,55	2067,92
970596	283004	2001,67	2047,03	1824,25	1821,4	1821,4	1821,4	2049,88
970626	282974	1945	2030,72	1823,86	1821,3	1821,29	1821,29	2033,29
970656	282944	2075	2015,32	1823,39	1821,08	1821,08	1821,08	2017,63
970686	282914	1961,67	2000,84	1823,03	1820,96	1820,96	1820,96	2002,92
970716	282884	1951,67	1987,31	1822,72	1820,85	1820,85	1820,85	1989,17
970746	282854	2013,33	1974,81	1822,36	1820,69	1820,69	1820,69	1976,49
970776	282824	1923,33	1963,07	1822,1	1820,6	1820,6	1820,6	1964,58
970806	282794	2108,33	1951,84	1821,71	1820,36	1820,36	1820,36	1953,19
970836	282764	2023,33	1941,68	1821,39	1820,19	1820,19	1820,19	1942,89
970866	282734	1900	1932,23	1821,2	1820,12	1820,12	1820,12	1933,31
970896	282704	1893,33	1923,34	1821,02	1820,06	1820,06	1820,06	1924,31
970926	282674	1893,33	1915,14	1820,86	1820	1819,99	1819,99	1916
970956	282644	1931,67	1907,55	1820,67	1819,9	1819,9	1819,9	1908,32
970986	282614	1921,67	1900,42	1820,5	1819,81	1819,81	1819,81	1901,11
971016	282584	1886,67	1893,77	1820,37	1819,76	1819,76	1819,76	1894,39
971046	282554	1880	1887,78	1820,26	1819,71	1819,71	1819,71	1888,33
971076	282524	1973,33	1882,07	1820,06	1819,58	1819,58	1819,58	1882,56
971106	282494	1970	1876,72	1819,88	1819,45	1819,45	1819,45	1877,16
971136	282464	1780	1871,94	1819,8	1819,42	1819,42	1819,42	1872,33
971166	282434	1785	1867,53	1819,73	1819,39	1819,39	1819,39	1867,87
971196	282404	1830	1863,42	1819,68	1819,38	1819,38	1819,38	1863,72
971226	282374	1890	1859,58	1819,59	1819,32	1819,32	1819,32	1859,85
971256	282344	1758,33	1856,11	1819,51	1819,27	1819,27	1819,27	1856,35
971286	282314	1846,67	1852,89	1819,46	1819,24	1819,24	1819,24	1853,1
971316	282284	1878,33	1849,86	1819,38	1819,19	1819,19	1819,19	1850,05
971346	282254	1838,33	1847,18	1819,34	1819,18	1819,18	1819,18	1847,35
971376	282224	1890	1844,65	1819,27	1819,12	1819,12	1819,12	1844,8
971406	282194	1876,67	1842,3	1819,2	1819,07	1819,07	1819,07	1842,43
971436	282164	1776,67	1840,18	1819,15	1819,03	1819,03	1819,03	1840,29
971466	282134	1766,67	1838,24	1819,09	1818,99	1818,99	1818,99	1838,34
971496	282104	2026,67	1836,3	1818,9	1818,81	1818,81	1818,81	1836,39
971526	282074	1808,33	1834,66	1818,88	1818,81	1818,81	1818,81	1834,74
971556	282044	1870	1833,17	1818,83	1818,76	1818,76	1818,76	1833,24
971586	282014	1785	1831,8	1818,79	1818,73	1818,73	1818,73	1831,87
971616	281984	1756,67	1830,51	1818,73	1818,68	1818,68	1818,68	1830,56
971646	281954	1831,67	1829,39	1818,72	1818,67	1818,67	1818,67	1829,43
971676	281924	1833,33	1828,36	1818,7	1818,66	1818,66	1818,66	1828,4
971706	281894	1818,33	1827,43	1818,69	1818,66	1818,66	1818,66	1827,46
971736	281864	1843,33	1826,55	1818,67	1818,64	1818,64	1818,64	1826,58
971766	281834	1826,67	1833,82	1826,69	1826,67	1826,67	1826,67	1833,85

Please do not adjust margins

Please do not adjust margins

971796	281804	1920	1926,46	1920,02	1920	1920	1920	1926,48
971826	281774	1855	1860,81	1855,02	1855	1855	1855	1860,83
971856	281744	1746,67	1751,91	1746,69	1746,67	1746,67	1746,67	1751,93
971886	281714	1788,33	1793,05	1788,35	1788,33	1788,33	1788,33	1793,07
971916	281684	1750	1754,24	1750,01	1750	1750	1750	1754,26
971946	281654	1718,33	1722,15	1718,35	1718,33	1718,33	1718,33	1722,16
971976	281624	1846,67	1850,1	1846,68	1846,67	1846,67	1846,67	1850,11
972006	281594	1751,67	1754,74	1751,68	1751,67	1751,67	1751,67	1754,75
972036	281564	1720	1722,75	1720,01	1720	1720	1720	1722,76
972066	281534	1705	1707,47	1705,01	1705	1705	1705	1707,48
972096	281504	1840	1842,21	1840,01	1840	1840	1840	1842,22
972126	281474	1786,67	1788,64	1786,67	1786,67	1786,67	1786,67	1788,65
972156	281444	1903,33	1905,1	1903,34	1903,33	1903,33	1903,33	1905,11
972186	281414	1850	1851,58	1850	1850	1850	1850	1851,59
972216	281384	1863,33	1864,75	1863,34	1863,33	1863,33	1863,33	1864,75
972246	281354	1821,67	1822,92	1821,67	1821,67	1821,67	1821,67	1822,93
972276	281324	1798,33	1799,46	1798,34	1798,33	1798,33	1798,33	1799,46
972306	281294	1846,67	1847,67	1846,67	1846,67	1846,67	1846,67	1847,67
972336	281264	1720	1720,89	1720	1720	1720	1720	1720,89
972366	281234	1728,33	1729,13	1728,33	1728,33	1728,33	1728,33	1729,13
972396	281204	1793,33	1794,04	1793,33	1793,33	1793,33	1793,33	1794,04
972426	281174	1801,67	1802,29	1801,67	1801,67	1801,67	1801,67	1802,29
972456	281144	1803,33	1803,89	1803,33	1803,33	1803,33	1803,33	1803,89
972486	281114	1806,67	1807,16	1806,67	1806,67	1806,67	1806,67	1807,16
972516	281084	1756,67	1757,1	1756,67	1756,67	1756,67	1756,67	1757,1
972546	281054	1840	1840,38	1840	1840	1840	1840	1840,39
972576	281024	1875	1875,34	1875	1875	1875	1875	1875,34
972606	280994	1783,33	1783,64	1783,33	1783,33	1783,33	1783,33	1783,64
972636	280964	1748,33	1748,6	1748,33	1748,33	1748,33	1748,33	1748,6
972666	280934	1796,67	1796,9	1796,67	1796,67	1796,67	1796,67	1796,9
972696	280904	1783,33	1783,54	1783,33	1783,33	1783,33	1783,33	1783,54
972726	280874	1746,67	1746,85	1746,67	1746,67	1746,67	1746,67	1746,85
972756	280844	1856,67	1856,83	1856,67	1856,67	1856,67	1856,67	1856,83
972786	280814	1808,33	1808,48	1808,33	1808,33	1808,33	1808,33	1808,48
972816	280784	1761,67	1761,79	1761,67	1761,67	1761,67	1761,67	1761,79
972846	280754	1728,33	1728,44	1728,33	1728,33	1728,33	1728,33	1728,44
972876	280724	1946,67	1946,76	1946,67	1946,67	1946,67	1946,67	1946,76
972906	280694	1936,67	1936,75	1936,67	1936,67	1936,67	1936,67	1936,75
972936	280664	1800	1800,07	1800	1800	1800	1800	1800,07
972966	280634	1933,33	1933,4	1933,33	1933,33	1933,33	1933,33	1933,4
972996	280604	1903,33	1903,39	1903,33	1903,33	1903,33	1903,33	1903,39
973026	280574	1768,33	1768,38	1768,33	1768,33	1768,33	1768,33	1768,38
973056	280544	1825	1825,04	1825	1825	1825	1825	1825,04
973086	280514	1828,33	1828,37	1828,33	1828,33	1828,33	1828,33	1828,37
973116	280484	1741,67	1741,7	1741,67	1741,67	1741,67	1741,67	1741,7
973146	280454	1901,67	1901,7	1901,67	1901,67	1901,67	1901,67	1901,7
973176	280424	1760	1760,02	1760	1760	1760	1760	1760,02

This journal is © The Royal Society of Chemistry 20xx

Please do not adjust margins

973206	280394	1760	1760,02	1760	1760	1760	1760	1760,02
973236	280364	1751,67	1751,69	1751,67	1751,67	1751,67	1751,67	1751,69
973266	280334	1845	1845,02	1845	1845	1845	1845	1845,02
973296	280304	1893,33	1893,35	1893,33	1893,33	1893,33	1893,33	1893,35
973326	280274	1720	1720,01	1720	1720	1720	1720	1720,01
973356	280244	1841,67	1841,68	1841,67	1841,67	1841,67	1841,67	1841,68
973386	280214	1738,33	1738,34	1738,33	1738,33	1738,33	1738,33	1738,34
973416	280184	1811,67	1811,67	1811,67	1811,67	1811,67	1811,67	1811,67
973446	280154	1826,67	1826,67	1826,67	1826,67	1826,67	1826,67	1826,67
973476	280124	1916,67	1916,67	1916,67	1916,67	1916,67	1916,67	1916,67
973506	280094	1848,33	1848,34	1848,33	1848,33	1848,33	1848,33	1848,34
973536	280064	1890	1890	1890	1890	1890	1890	1890
973566	280034	1740	1740	1740	1740	1740	1740	1740
973596	280004	1680	1680	1680	1680	1680	1680	1680
973626	279974	1788,33	1788,34	1788,33	1788,33	1788,33	1788,33	1788,34
973656	279944	1900	1900	1900	1900	1900	1900	1900
973686	279914	1841,67	1841,67	1841,67	1841,67	1841,67	1841,67	1841,67
973716	279884	1890	1890	1890	1890	1890	1890	1890
973746	279854	1881,67	1881,67	1881,67	1881,67	1881,67	1881,67	1881,67
973776	279824	1920	1920	1920	1920	1920	1920	1920
973806	279794	1783,33	1783,33	1783,33	1783,33	1783,33	1783,33	1783,33
973836	279764	1783,33	1783,33	1783,33	1783,33	1783,33	1783,33	1783,33
973866	279734	1775	1775	1775	1775	1775	1775	1775
973896	279704	1691,67	1691,67	1691,67	1691,67	1691,67	1691,67	1691,67
973926	279674	1763,33	1763,33	1763,33	1763,33	1763,33	1763,33	1763,33
973956	279644	1821,67	1821,67	1821,67	1821,67	1821,67	1821,67	1821,67
973986	279614	1743,33	1743,33	1743,33	1743,33	1743,33	1743,33	1743,33
974016	279584	1870	1870	1870	1870	1870	1870	1870
974046	279554	1740	1740	1740	1740	1740	1740	1740
974076	279524	1765	1765	1765	1765	1765	1765	1765
974106	279494	1828,33	1828,33	1828,33	1828,33	1828,33	1828,33	1828,33
974136	279464	1861,67	1861,67	1861,67	1861,67	1861,67	1861,67	1861,67
974166	279434	1806,67	1806,67	1806,67	1806,67	1806,67	1806,67	1806,67
974196	279404	1836,67	1836,67	1836,67	1836,67	1836,67	1836,67	1836,67
974226	279374	1818,33	1818,33	1818,33	1818,33	1818,33	1818,33	1818,33
974256	279344	1800	1800	1800	1800	1800	1800	1800
974286	279314	1781,67	1781,67	1781,67	1781,67	1781,67	1781,67	1781,67
974316	279284	1865	1865	1865	1865	1865	1865	1865
974346	279254	1840	1840	1840	1840	1840	1840	1840
974376	279224	1838,33	1838,33	1838,33	1838,33	1838,33	1838,33	1838,33
974406	279194	1810	1810	1810	1810	1810	1810	1810
974436	279164	1863,33	1863,33	1863,33	1863,33	1863,33	1863,33	1863,33
974466	279134	1866,67	1866,67	1866,67	1866,67	1866,67	1866,67	1866,67
974496	279104	1780	1780	1780	1780	1780	1780	1780
974526	279074	1773,33	1773,33	1773,33	1773,33	1773,33	1773,33	1773,33
974556	279044	1868,33	1868,33	1868,33	1868,33	1868,33	1868,33	1868,33
974586	279014	1890	1890	1890	1890	1890	1890	1890

This journal is © The Royal Society of Chemistry 20xx

Please do not adjust margins

974616	278984	1803,33	1803,33	1803,33	1803,33	1803,33	1803,33	1803,33
974646	278954	1858,33	1858,33	1858,33	1858,33	1858,33	1858,33	1858,33
974676	278924	1841,67	1841,67	1841,67	1841,67	1841,67	1841,67	1841,67
974706	278894	1851,67	1851,67	1851,67	1851,67	1851,67	1851,67	1851,67
974736	278864	1801,67	1801,67	1801,67	1801,67	1801,67	1801,67	1801,67
974766	278834	1848,33	1848,33	1848,33	1848,33	1848,33	1848,33	1848,33
974796	278804	1866,67	1866,67	1866,67	1866,67	1866,67	1866,67	1866,67
974826	278774	1878,33	1878,33	1878,33	1878,33	1878,33	1878,33	1878,33
974856	278744	1943,33	1943,33	1943,33	1943,33	1943,33	1943,33	1943,33
974886	278714	1865	1865	1865	1865	1865	1865	1865
974916	278684	1821,67	1821,67	1821,67	1821,67	1821,67	1821,67	1821,67
974946	278654	1851,67	1851,67	1851,67	1851,67	1851,67	1851,67	1851,67
974976	278624	1738,33	1738,33	1738,33	1738,33	1738,33	1738,33	1738,33
975006	278594	1836,67	1836,67	1836,67	1836,67	1836,67	1836,67	1836,67
975036	278564	1850	1850	1850	1850	1850	1850	1850
975066	278534	1703,33	1703,33	1703,33	1703,33	1703,33	1703,33	1703,33
975096	278504	1915	1915	1915	1915	1915	1915	1915
975126	278474	1930	1930	1930	1930	1930	1930	1930
975156	278444	1821,67	1821,67	1821,67	1821,67	1821,67	1821,67	1821,67
975186	278414	1901,67	1901,67	1901,67	1901,67	1901,67	1901,67	1901,67
975216	278384	1895	1895	1895	1895	1895	1895	1895
975246	278354	1848,33	1848,33	1848,33	1848,33	1848,33	1848,33	1848,33
975276	278324	1891,67	1891,67	1891,67	1891,67	1891,67	1891,67	1891,67
975306	278294	1856,67	1856,67	1856,67	1856,67	1856,67	1856,67	1856,67
975336	278264	1943,33	1943,33	1943,33	1943,33	1943,33	1943,33	1943,33
975366	278234	1966,67	1966,67	1966,67	1966,67	1966,67	1966,67	1966,67
975396	278204	1761,67	1761,67	1761,67	1761,67	1761,67	1761,67	1761,67
975426	278174	1935	1935	1935	1935	1935	1935	1935
975456	278144	1945	1945	1945	1945	1945	1945	1945
975486	278114	1886,67	1886,67	1886,67	1886,67	1886,67	1886,67	1886,67
975516	278084	2005	2005	2005	2005	2005	2005	2005
975546	278054	1881,67	1881,67	1881,67	1881,67	1881,67	1881,67	1881,67
975576	278024	2023,33	2023,33	2023,33	2023,33	2023,33	2023,33	2023,33
975606	277994	1880	1880	1880	1880	1880	1880	1880
975636	277964	1900	1900	1900	1900	1900	1900	1900
975666	277934	1955	1955	1955	1955	1955	1955	1955
975696	277904	1893,33	1893,33	1893,33	1893,33	1893,33	1893,33	1893,33
975726	277874	1940	1940	1940	1940	1940	1940	1940
975756	277844	1906,67	1906,67	1906,67	1906,67	1906,67	1906,67	1906,67
975786	277814	1916,67	1916,67	1916,67	1916,67	1916,67	1916,67	1916,67
975816	277784	1965	1965	1965	1965	1965	1965	1965
975846	277754	1921,67	1921,67	1921,67	1921,67	1921,67	1921,67	1921,67
975876	277724	1915	1915	1915	1915	1915	1915	1915
975906	277694	1895	1895	1895	1895	1895	1895	1895
975936	277664	1946,67	1946,67	1946,67	1946,67	1946,67	1946,67	1946,67
975966	277634	1975	1975	1975	1975	1975	1975	1975
975996	277604	2008,33	2008,33	2008,33	2008,33	2008,33	2008,33	2008,33

This journal is © The Royal Society of Chemistry 20xx

Please do not adjust margins

Please do not adjust margins

976026	277574	1928,33	1928,33	1928,33	1928,33	1928,33	1928,33	1928,33
976056	277544	1968,33	1968,33	1968,33	1968,33	1968,33	1968,33	1968,33
976086	277514	2023,33	2023,33	2023,33	2023,33	2023,33	2023,33	2023,33
976116	277484	1996,67	1996,67	1996,67	1996,67	1996,67	1996,67	1996,67
976146	277454	1936,67	1936,67	1936,67	1936,67	1936,67	1936,67	1936,67
976176	277424	1986,67	1986,67	1986,67	1986,67	1986,67	1986,67	1986,67
976206	277394	2006,67	2006,67	2006,67	2006,67	2006,67	2006,67	2006,67
976236	277364	2061,67	2061,67	2061,67	2061,67	2061,67	2061,67	2061,67
976266	277334	2000	2000	2000	2000	2000	2000	2000
976296	277304	1901,67	1901,67	1901,67	1901,67	1901,67	1901,67	1901,67
976326	277274	2068,33	2068,33	2068,33	2068,33	2068,33	2068,33	2068,33
976356	277244	1986,67	1986,67	1986,67	1986,67	1986,67	1986,67	1986,67
976386	277214	1926,67	1926,67	1926,67	1926,67	1926,67	1926,67	1926,67
976416	277184	1956,67	1956,67	1956,67	1956,67	1956,67	1956,67	1956,67
976446	277154	2018,33	2018,33	2018,33	2018,33	2018,33	2018,33	2018,33
976476	277124	1853,33	1853,33	1853,33	1853,33	1853,33	1853,33	1853,33
976506	277094	1931,67	1931,67	1931,67	1931,67	1931,67	1931,67	1931,67
976536	277064	2040	2040	2040	2040	2040	2040	2040
976566	277034	2106,67	2106,67	2106,67	2106,67	2106,67	2106,67	2106,67
976596	277004	1988,33	1988,33	1988,33	1988,33	1988,33	1988,33	1988,33
976626	276974	2086,67	2086,67	2086,67	2086,67	2086,67	2086,67	2086,67
976656	276944	2091,67	2091,67	2091,67	2091,67	2091,67	2091,67	2091,67
976686	276914	1950	1950	1950	1950	1950	1950	1950
976716	276884	1911,67	1911,67	1911,67	1911,67	1911,67	1911,67	1911,67
976746	276854	2158,33	2158,33	2158,33	2158,33	2158,33	2158,33	2158,33
976776	276824	2066,67	2066,67	2066,67	2066,67	2066,67	2066,67	2066,67
976806	276794	2113,33	2113,33	2113,33	2113,33	2113,33	2113,33	2113,33
976836	276764	2076,67	2076,67	2076,67	2076,67	2076,67	2076,67	2076,67
976866	276734	2026,67	2026,67	2026,67	2026,67	2026,67	2026,67	2026,67
976896	276704	1990	1990	1990	1990	1990	1990	1990
976926	276674	2075	2075	2075	2075	2075	2075	2075
976956	276644	1933,33	1933,33	1933,33	1933,33	1933,33	1933,33	1933,33
976986	276614	2021,67	2021,67	2021,67	2021,67	2021,67	2021,67	2021,67
977016	276584	2028,33	2028,33	2028,33	2028,33	2028,33	2028,33	2028,33
977046	276554	2048,33	2048,33	2048,33	2048,33	2048,33	2048,33	2048,33
977076	276524	2161,67	2161,67	2161,67	2161,67	2161,67	2161,67	2161,67
977106	276494	1960	1960	1960	1960	1960	1960	1960
977136	276464	2055	2055	2055	2055	2055	2055	2055
977166	276434	2083,33	2083,33	2083,33	2083,33	2083,33	2083,33	2083,33
977196	276404	1911,67	1911,67	1911,67	1911,67	1911,67	1911,67	1911,67
977226	276374	2098,33	2098,33	2098,33	2098,33	2098,33	2098,33	2098,33
977256	276344	2135	2135	2135	2135	2135	2135	2135
977286	276314	1950	1950	1950	1950	1950	1950	1950
977316	276284	2030	2030	2030	2030	2030	2030	2030
977346	276254	1978,33	1978,33	1978,33	1978,33	1978,33	1978,33	1978,33
977376	276224	2080	2080	2080	2080	2080	2080	2080
977406	276194	2078,33	2078,33	2078,33	2078,33	2078,33	2078,33	2078,33

Please do not adjust margins

Please do not adjust margins

977436	276164	2101,67	2101,67	2101,67	2101,67	2101,67	2101,67	2101,67
977466	276134	2041,67	2041,67	2041,67	2041,67	2041,67	2041,67	2041,67
977496	276104	1931,67	1931,67	1931,67	1931,67	1931,67	1931,67	1931,67
977526	276074	1901,67	1901,67	1901,67	1901,67	1901,67	1901,67	1901,67
977556	276044	2028,33	2028,33	2028,33	2028,33	2028,33	2028,33	2028,33
977586	276014	2003,33	2003,33	2003,33	2003,33	2003,33	2003,33	2003,33
977616	275984	2043,33	2043,33	2043,33	2043,33	2043,33	2043,33	2043,33
977646	275954	1961,67	1961,67	1961,67	1961,67	1961,67	1961,67	1961,67
977676	275924	2050	2050	2050	2050	2050	2050	2050
977706	275894	1963,33	1963,33	1963,33	1963,33	1963,33	1963,33	1963,33
977736	275864	1831,67	1831,67	1831,67	1831,67	1831,67	1831,67	1831,67
977766	275834	1975	1975	1975	1975	1975	1975	1975
977796	275804	2055	2055	2055	2055	2055	2055	2055
977826	275774	2106,67	2106,67	2106,67	2106,67	2106,67	2106,67	2106,67
977856	275744	1985	1985	1985	1985	1985	1985	1985
977886	275714	1958,33	1958,33	1958,33	1958,33	1958,33	1958,33	1958,33
977916	275684	1830	1830	1830	1830	1830	1830	1830
977946	275654	1881,67	1881,67	1881,67	1881,67	1881,67	1881,67	1881,67
977976	275624	1900	1900	1900	1900	1900	1900	1900
978006	275594	1891,67	1891,67	1891,67	1891,67	1891,67	1891,67	1891,67
978036	275564	1863,33	1863,33	1863,33	1863,33	1863,33	1863,33	1863,33
978066	275534	1873,33	1873,33	1873,33	1873,33	1873,33	1873,33	1873,33
978096	275504	1881,67	1881,67	1881,67	1881,67	1881,67	1881,67	1881,67
978126	275474	1895	1895	1895	1895	1895	1895	1895
978156	275444	1933,33	1933,33	1933,33	1933,33	1933,33	1933,33	1933,33
978186	275414	1893,33	1893,33	1893,33	1893,33	1893,33	1893,33	1893,33
978216	275384	1915	1915	1915	1915	1915	1915	1915
978246	275354	1786,67	1786,67	1786,67	1786,67	1786,67	1786,67	1786,67
978276	275324	1850	1850	1850	1850	1850	1850	1850
978306	275294	1840	1840	1840	1840	1840	1840	1840
978336	275264	1880	1880	1880	1880	1880	1880	1880
978366	275234	1841,67	1841,67	1841,67	1841,67	1841,67	1841,67	1841,67
978396	275204	1851,67	1851,67	1851,67	1851,67	1851,67	1851,67	1851,67
978426	275174	1885	1885	1885	1885	1885	1885	1885
978456	275144	1813,33	1813,33	1813,33	1813,33	1813,33	1813,33	1813,33
978486	275114	1896,67	1896,67	1896,67	1896,67	1896,67	1896,67	1896,67
978516	275084	1805	1805	1805	1805	1805	1805	1805
978546	275054	1875	1875	1875	1875	1875	1875	1875
978576	275024	1881,67	1881,67	1881,67	1881,67	1881,67	1881,67	1881,67
978606	274994	1860	1860	1860	1860	1860	1860	1860
978636	274964	1823,33	1823,33	1823,33	1823,33	1823,33	1823,33	1823,33
978666	274934	1798,33	1798,33	1798,33	1798,33	1798,33	1798,33	1798,33
978696	274904	1705	1705	1705	1705	1705	1705	1705
978726	274874	1765	1765	1765	1765	1765	1765	1765
978756	274844	1898,33	1898,33	1898,33	1898,33	1898,33	1898,33	1898,33
978786	274814	1801,67	1801,67	1801,67	1801,67	1801,67	1801,67	1801,67
978816	274784	1816,67	1816,67	1816,67	1816,67	1816,67	1816,67	1816,67

This journal is © The Royal Society of Chemistry 20xx

Please do not adjust margins

Please do not adjust margins

978846	274754	1688,33	1688,33	1688,33	1688,33	1688,33	1688,33	1688,33
978876	274724	1828,33	1828,33	1828,33	1828,33	1828,33	1828,33	1828,33
978906	274694	1828,33	1828,33	1828,33	1828,33	1828,33	1828,33	1828,33
978936	274664	1846,67	1846,67	1846,67	1846,67	1846,67	1846,67	1846,67
978966	274634	1811,67	1811,67	1811,67	1811,67	1811,67	1811,67	1811,67
978996	274604	1723,33	1723,33	1723,33	1723,33	1723,33	1723,33	1723,33
979026	274574	1720	1720	1720	1720	1720	1720	1720
979056	274544	1898,33	1898,33	1898,33	1898,33	1898,33	1898,33	1898,33
979086	274514	1793,33	1793,33	1793,33	1793,33	1793,33	1793,33	1793,33
979116	274484	1800	1800	1800	1800	1800	1800	1800
979146	274454	1776,67	1776,67	1776,67	1776,67	1776,67	1776,67	1776,67
979176	274424	1811,67	1811,67	1811,67	1811,67	1811,67	1811,67	1811,67
979206	274394	1611,67	1611,67	1611,67	1611,67	1611,67	1611,67	1611,67
979236	274364	1768,33	1768,33	1768,33	1768,33	1768,33	1768,33	1768,33
979266	274334	1698,33	1698,33	1698,33	1698,33	1698,33	1698,33	1698,33
979296	274304	1745	1745	1745	1745	1745	1745	1745
979326	274274	1718,33	1718,33	1718,33	1718,33	1718,33	1718,33	1718,33
979356	274244	1668,33	1668,33	1668,33	1668,33	1668,33	1668,33	1668,33
979386	274214	1663,33	1663,33	1663,33	1663,33	1663,33	1663,33	1663,33
979416	274184	1620	1620	1620	1620	1620	1620	1620
979446	274154	1778,33	1778,33	1778,33	1778,33	1778,33	1778,33	1778,33
979476	274124	1750	1750	1750	1750	1750	1750	1750
979506	274094	1778,33	1778,33	1778,33	1778,33	1778,33	1778,33	1778,33
979536	274064	1688,33	1688,33	1688,33	1688,33	1688,33	1688,33	1688,33
979566	274034	1641,67	1641,67	1641,67	1641,67	1641,67	1641,67	1641,67
979596	274004	1723,33	1723,33	1723,33	1723,33	1723,33	1723,33	1723,33
979626	273974	1678,33	1678,33	1678,33	1678,33	1678,33	1678,33	1678,33
979656	273944	1693,33	1693,33	1693,33	1693,33	1693,33	1693,33	1693,33
979686	273914	1618,33	1618,33	1618,33	1618,33	1618,33	1618,33	1618,33
979716	273884	1640	1640	1640	1640	1640	1640	1640
979746	273854	1661,67	1661,67	1661,67	1661,67	1661,67	1661,67	1661,67
979776	273824	1658,33	1658,33	1658,33	1658,33	1658,33	1658,33	1658,33
979806	273794	1631,67	1631,67	1631,67	1631,67	1631,67	1631,67	1631,67
979836	273764	1640	1640	1640	1640	1640	1640	1640
979866	273734	1640	1640	1640	1640	1640	1640	1640
979896	273704	1615	1615	1615	1615	1615	1615	1615
979926	273674	1676,67	1676,67	1676,67	1676,67	1676,67	1676,67	1676,67
979956	273644	1640	1640	1640	1640	1640	1640	1640
979986	273614	1643,33	1643,33	1643,33	1643,33	1643,33	1643,33	1643,33
980016	273584	1641,67	1641,67	1641,67	1641,67	1641,67	1641,67	1641,67
980046	273554	1521,67	1521,67	1521,67	1521,67	1521,67	1521,67	1521,67
980076	273524	1588,33	1588,33	1588,33	1588,33	1588,33	1588,33	1588,33
980106	273494	1666,67	1666,67	1666,67	1666,67	1666,67	1666,67	1666,67
980136	273464	1568,33	1568,33	1568,33	1568,33	1568,33	1568,33	1568,33
980166	273434	1665	1665	1665	1665	1665	1665	1665
980196	273404	1596,67	1596,67	1596,67	1596,67	1596,67	1596,67	1596,67
980226	273374	1655	1655	1655	1655	1655	1655	1655

This journal is © The Royal Society of Chemistry 20xx

Please do not adjust margins

Please do not adjust margins

980256	273344	1608,33	1608,33	1608,33	1608,33	1608,33	1608,33	1608,33
980286	273314	1633,33	1633,33	1633,33	1633,33	1633,33	1633,33	1633,33
980316	273284	1625	1625	1625	1625	1625	1625	1625
980346	273254	1588,33	1588,33	1588,33	1588,33	1588,33	1588,33	1588,33
980376	273224	1605	1605	1605	1605	1605	1605	1605
980406	273194	1588,33	1588,33	1588,33	1588,33	1588,33	1588,33	1588,33
980436	273164	1588,33	1588,33	1588,33	1588,33	1588,33	1588,33	1588,33
980466	273134	1635	1635	1635	1635	1635	1635	1635
980496	273104	1670	1670	1670	1670	1670	1670	1670
980526	273074	1511,67	1511,67	1511,67	1511,67	1511,67	1511,67	1511,67
980556	273044	1626,67	1626,67	1626,67	1626,67	1626,67	1626,67	1626,67
980586	273014	1651,67	1651,67	1651,67	1651,67	1651,67	1651,67	1651,67
980616	272984	1635	1635	1635	1635	1635	1635	1635
980646	272954	1551,67	1551,67	1551,67	1551,67	1551,67	1551,67	1551,67
980676	272924	1635	1635	1635	1635	1635	1635	1635
980706	272894	1555	1555	1555	1555	1555	1555	1555
980736	272864	1520	1520	1520	1520	1520	1520	1520
980766	272834	1680	1680	1680	1680	1680	1680	1680
980796	272804	1583,33	1583,33	1583,33	1583,33	1583,33	1583,33	1583,33
980826	272774	1508,33	1508,33	1508,33	1508,33	1508,33	1508,33	1508,33
980856	272744	1581,67	1581,67	1581,67	1581,67	1581,67	1581,67	1581,67
980886	272714	1653,33	1653,33	1653,33	1653,33	1653,33	1653,33	1653,33
980916	272684	1588,33	1588,33	1588,33	1588,33	1588,33	1588,33	1588,33
980946	272654	1566,67	1566,67	1566,67	1566,67	1566,67	1566,67	1566,67
980976	272624	1560	1560	1560	1560	1560	1560	1560
981006	272594	1553,33	1553,33	1553,33	1553,33	1553,33	1553,33	1553,33
981036	272564	1573,33	1573,33	1573,33	1573,33	1573,33	1573,33	1573,33
981066	272534	1588,33	1588,33	1588,33	1588,33	1588,33	1588,33	1588,33
981096	272504	1576,67	1576,67	1576,67	1576,67	1576,67	1576,67	1576,67
981126	272474	1468,33	1468,33	1468,33	1468,33	1468,33	1468,33	1468,33
981156	272444	1535	1535	1535	1535	1535	1535	1535
981186	272414	1595	1595	1595	1595	1595	1595	1595
981216	272384	1581,67	1581,67	1581,67	1581,67	1581,67	1581,67	1581,67
981246	272354	1535	1535	1535	1535	1535	1535	1535
981276	272324	1621,67	1621,67	1621,67	1621,67	1621,67	1621,67	1621,67
981306	272294	1591,67	1591,67	1591,67	1591,67	1591,67	1591,67	1591,67
981336	272264	1600	1600	1600	1600	1600	1600	1600
981366	272234	1558,33	1558,33	1558,33	1558,33	1558,33	1558,33	1558,33
981396	272204	1533,33	1533,33	1533,33	1533,33	1533,33	1533,33	1533,33
981426	272174	1568,33	1568,33	1568,33	1568,33	1568,33	1568,33	1568,33
981456	272144	1581,67	1581,67	1581,67	1581,67	1581,67	1581,67	1581,67
981486	272114	1636,67	1636,67	1636,67	1636,67	1636,67	1636,67	1636,67
981516	272084	1583,33	1583,33	1583,33	1583,33	1583,33	1583,33	1583,33
981546	272054	1570	1570	1570	1570	1570	1570	1570
981576	272024	1641,67	1641,67	1641,67	1641,67	1641,67	1641,67	1641,67
981606	271994	1531,67	1531,67	1531,67	1531,67	1531,67	1531,67	1531,67
981636	271964	1648,33	1648,33	1648,33	1648,33	1648,33	1648,33	1648,33

This journal is © The Royal Society of Chemistry 20xx

Please do not adjust margins

Please do not adjust margins

981666	271934	1541,67	1541,67	1541,67	1541,67	1541,67	1541,67	1541,67
981696	271904	1596,67	1596,67	1596,67	1596,67	1596,67	1596,67	1596,67
981726	271874	1418,33	1418,33	1418,33	1418,33	1418,33	1418,33	1418,33
981756	271844	1543,33	1543,33	1543,33	1543,33	1543,33	1543,33	1543,33
981786	271814	1620	1620	1620	1620	1620	1620	1620
981816	271784	1506,67	1506,67	1506,67	1506,67	1506,67	1506,67	1506,67
981846	271754	1578,33	1578,33	1578,33	1578,33	1578,33	1578,33	1578,33
981876	271724	1550	1550	1550	1550	1550	1550	1550
981906	271694	1660	1660	1660	1660	1660	1660	1660
981936	271664	1646,67	1646,67	1646,67	1646,67	1646,67	1646,67	1646,67
981966	271634	1663,33	1663,33	1663,33	1663,33	1663,33	1663,33	1663,33
981996	271604	1510	1510	1510	1510	1510	1510	1510
982026	271574	1591,67	1591,67	1591,67	1591,67	1591,67	1591,67	1591,67
982056	271544	1525	1525	1525	1525	1525	1525	1525
982086	271514	1488,33	1488,33	1488,33	1488,33	1488,33	1488,33	1488,33
982116	271484	1600	1600	1600	1600	1600	1600	1600
982146	271454	1643,33	1643,33	1643,33	1643,33	1643,33	1643,33	1643,33
982176	271424	1575	1575	1575	1575	1575	1575	1575
982206	271394	1508,33	1508,33	1508,33	1508,33	1508,33	1508,33	1508,33
982236	271364	1541,67	1541,67	1541,67	1541,67	1541,67	1541,67	1541,67
982266	271334	1633,33	1633,33	1633,33	1633,33	1633,33	1633,33	1633,33
982296	271304	1451,67	1451,67	1451,67	1451,67	1451,67	1451,67	1451,67
982326	271274	1513,33	1513,33	1513,33	1513,33	1513,33	1513,33	1513,33
982356	271244	1518,33	1518,33	1518,33	1518,33	1518,33	1518,33	1518,33
982386	271214	1626,67	1626,67	1626,67	1626,67	1626,67	1626,67	1626,67
982416	271184	1523,33	1523,33	1523,33	1523,33	1523,33	1523,33	1523,33

Table S6 Raw XPS measurements corresponding to Fig. 2.d.

KE_MgKa	BE_MgKa	CPS_MgKa	C 1s_1_MgKa	C 1s_2_MgKa	N 1s_3_MgKa	N 1s_4_MgKa	N 1s_5_MgKa	N 1s_6_MgKa	Background_MgKa	Envelope_MgKa
845869	407731	4454,67	4454,67	4454,67	4454,67	4454,67	4454,67	4454,67	4454,67	4454,67
845899	407701	4310	4310	4310	4310	4310	4310	4310	4310	4310
845929	407671	4418	4418	4418	4418	4418	4418	4418	4418	4418
845959	407641	4249,33	4249,33	4249,33	4249,33	4249,33	4249,33	4249,33	4249,33	4249,33
845989	407611	4451,33	4451,33	4451,33	4451,33	4451,33	4451,33	4451,33	4451,33	4451,33
846019	407581	4448	4448	4448	4448	4448	4448	4448	4448	4448
846049	407551	4370	4370	4370	4370	4370	4370	4370	4370	4370
846079	407521	4408,67	4408,67	4408,67	4408,67	4408,67	4408,67	4408,67	4408,67	4408,67
846109	407491	4335,33	4335,33	4335,33	4335,33	4335,33	4335,33	4335,33	4335,33	4335,33
846139	407461	4294	4294	4294	4294	4294	4294	4294	4294	4294
846169	407431	4396	4396	4396	4396	4396	4396	4396	4396	4396
846199	407401	4404,67	4404,67	4404,67	4404,67	4404,67	4404,67	4404,67	4404,67	4404,67
846229	407371	4331,33	4331,33	4331,33	4331,33	4331,33	4331,33	4331,33	4331,33	4331,33
846259	407341	4443,33	4443,33	4443,33	4443,33	4443,33	4443,33	4443,33	4443,33	4443,33
846289	407311	4347,33	4347,33	4347,33	4347,33	4347,33	4347,33	4347,33	4347,33	4347,33
846319	407281	4338,67	4338,67	4338,67	4338,67	4338,67	4338,67	4338,67	4338,67	4338,67
846349	407251	4295,33	4295,33	4295,33	4295,33	4295,33	4295,33	4295,33	4295,33	4295,33

Please do not adjust margins

846379	407221	4440,67	4440,67	4440,67	4440,67	4440,67	4440,67	4440,67	4440,67	4440,67
846409	407191	4470,67	4470,67	4470,67	4470,67	4470,67	4470,67	4470,67	4470,67	4470,67
846439	407161	4352	4352	4352	4352	4352	4352	4352	4352	4352
846469	407131	4395,33	4395,33	4395,33	4395,33	4395,33	4395,33	4395,33	4395,33	4395,33
846499	407101	4440,67	4440,67	4440,67	4440,67	4440,67	4440,67	4440,67	4440,67	4440,67
846529	407071	4366,67	4366,67	4366,67	4366,67	4366,67	4366,67	4366,67	4366,67	4366,67
846559	407041	4440	4440	4440	4440	4440	4440	4440	4440	4440
846589	407011	4348	4348	4348	4348	4348	4348	4348	4348	4348
846619	406981	4457,33	4457,33	4457,33	4457,33	4457,33	4457,33	4457,33	4457,33	4457,33
846649	406951	4349,33	4349,33	4349,33	4349,33	4349,33	4349,33	4349,33	4349,33	4349,33
846679	406921	4400,67	4400,67	4400,67	4400,67	4400,67	4400,67	4400,67	4400,67	4400,67
846709	406891	4395,33	4395,33	4395,33	4395,33	4395,33	4395,33	4395,33	4395,33	4395,33
846739	406861	4446	4446	4446	4446	4446	4446	4446	4446	4446
846769	406831	4322,67	4322,67	4322,67	4322,67	4322,67	4322,67	4322,67	4322,67	4322,67
846799	406801	4430	4430	4430	4430	4430	4430	4430	4430	4430
846829	406771	4378	4378	4378	4378	4378	4378	4378	4378	4378
846859	406741	4356,67	4356,67	4356,67	4356,67	4356,67	4356,67	4356,67	4356,67	4356,67
846889	406711	4352	4352	4352	4352	4352	4352	4352	4352	4352
846919	406681	4291,33	4291,33	4291,33	4291,33	4291,33	4291,33	4291,33	4291,33	4291,33
846949	406651	4470	4470	4470	4470	4470	4470	4470	4470	4470
846979	406621	4380,67	4380,67	4380,67	4380,67	4380,67	4380,67	4380,67	4380,67	4380,67
847009	406591	4412	4412	4412	4412	4412	4412	4412	4412	4412
847039	406561	4383,33	4383,33	4383,33	4383,33	4383,33	4383,33	4383,33	4383,33	4383,33
847069	406531	4302	4302	4302	4302	4302	4302	4302	4302	4302
847099	406501	4411,33	4411,33	4411,33	4411,33	4411,33	4411,33	4411,33	4411,33	4411,33
847129	406471	4422,67	4422,67	4422,67	4422,67	4422,67	4422,67	4422,67	4422,67	4422,67
847159	406441	4322	4322	4322	4322	4322	4322	4322	4322	4322
847189	406411	4311,33	4311,33	4311,33	4311,33	4311,33	4311,33	4311,33	4311,33	4311,33
847219	406381	4384	4384	4384	4384	4384	4384	4384	4384	4384
847249	406351	4356,67	4356,67	4356,67	4356,67	4356,67	4356,67	4356,67	4356,67	4356,67
847279	406321	4363,33	4363,33	4363,33	4363,33	4363,33	4363,33	4363,33	4363,33	4363,33
847309	406291	4418,67	4418,67	4418,67	4418,67	4418,67	4418,67	4418,67	4418,67	4418,67
847339	406261	4310,67	4310,67	4310,67	4310,67	4310,67	4310,67	4310,67	4310,67	4310,67
847369	406231	4388	4388	4388	4388	4388	4388	4388	4388	4388
847399	406201	4461,33	4461,33	4461,33	4461,33	4461,33	4461,33	4461,33	4461,33	4461,33
847429	406171	4422	4422	4422	4422	4422	4422	4422	4422	4422
847459	406141	4386	4386	4386	4386	4386	4386	4386	4386	4386
847489	406111	4350,67	4350,67	4350,67	4350,67	4350,67	4350,67	4350,67	4350,67	4350,67
847519	406081	4438,67	4438,67	4438,67	4438,67	4438,67	4438,67	4438,67	4438,67	4438,67
847549	406051	4250,67	4250,67	4250,67	4250,67	4250,67	4250,67	4250,67	4250,67	4250,67
847579	406021	4332,67	4332,67	4332,67	4332,67	4332,67	4332,67	4332,67	4332,67	4332,67
847609	405991	4364,67	4364,67	4364,67	4364,67	4364,67	4364,67	4364,67	4364,67	4364,67
847639	405961	4261,33	4261,33	4261,33	4261,33	4261,33	4261,33	4261,33	4261,33	4261,33
847669	405931	4267,33	4267,33	4267,33	4267,33	4267,33	4267,33	4267,33	4267,33	4267,33
847699	405901	4410	4410	4410	4410	4410	4410	4410	4410	4410
847729	405871	4293,33	4293,33	4293,33	4293,33	4293,33	4293,33	4293,33	4293,33	4293,33
847759	405841	4368,67	4368,67	4368,67	4368,67	4368,67	4368,67	4368,67	4368,67	4368,67

This journal is © The Royal Society of Chemistry 20xx

Please do not adjust margins

847789	405811	4388,67	4388,67	4388,67	4388,67	4388,67	4388,67	4388,67	4388,67	4388,67
847819	405781	4370	4370	4370	4370	4370	4370	4370	4370	4370
847849	405751	4330,67	4330,67	4330,67	4330,67	4330,67	4330,67	4330,67	4330,67	4330,67
847879	405721	4462,67	4462,67	4462,67	4462,67	4462,67	4462,67	4462,67	4462,67	4462,67
847909	405691	4302,67	4302,67	4302,67	4302,67	4302,67	4302,67	4302,67	4302,67	4302,67
847939	405661	4372	4372	4372	4372	4372	4372	4372	4372	4372
847969	405631	4421,33	4421,33	4421,33	4421,33	4421,33	4421,33	4421,33	4421,33	4421,33
847999	405601	4328,67	4328,67	4328,67	4328,67	4328,67	4328,67	4328,67	4328,67	4328,67
848029	405571	4379,33	4379,33	4379,33	4379,33	4379,33	4379,33	4379,33	4379,33	4379,33
848059	405541	4357,33	4357,33	4357,33	4357,33	4357,33	4357,33	4357,33	4357,33	4357,33
848089	405511	4320	4320	4320	4320	4320	4320	4320	4320	4320
848119	405481	4336,67	4336,67	4336,67	4336,67	4336,67	4336,67	4336,67	4336,67	4336,67
848149	405451	4345,33	4336,66	4336,66	4336,66	4336,66	4336,66	4336,66	4336,66	4336,66
848179	405421	4300,67	4336,61	4336,61	4336,61	4336,61	4336,61	4336,61	4336,61	4336,61
848209	405391	4268,67	4336,54	4336,54	4336,54	4336,54	4336,54	4336,54	4336,54	4336,54
848239	405361	4274,67	4336,46	4336,46	4336,46	4336,46	4336,46	4336,46	4336,46	4336,46
848269	405331	4372,67	4336,42	4336,42	4336,42	4336,42	4336,42	4336,42	4336,42	4336,42
848299	405301	4416,67	4336,33	4336,33	4336,33	4336,33	4336,33	4336,33	4336,33	4336,33
848329	405271	4288	4336,27	4336,27	4336,27	4336,27	4336,27	4336,27	4336,27	4336,27
848359	405241	4398,67	4336,2	4336,2	4336,2	4336,2	4336,2	4336,2	4336,2	4336,2
848389	405211	4224,67	4336,07	4336,07	4336,07	4336,07	4336,07	4336,07	4336,07	4336,07
848419	405181	4358	4336,04	4336,04	4336,04	4336,04	4336,04	4336,04	4336,04	4336,04
848449	405151	4321,33	4336,03	4336,03	4336,03	4336,03	4336,03	4336,03	4336,03	4336,03
848479	405121	4272	4335,95	4335,95	4335,95	4335,95	4335,95	4335,95	4335,95	4335,95
848509	405091	4382	4335,9	4335,9	4335,9	4335,9	4335,9	4335,9	4335,9	4335,9
848539	405061	4450,67	4335,76	4335,76	4335,76	4335,76	4335,76	4335,76	4335,76	4335,76
848569	405031	4368	4335,73	4335,73	4335,73	4335,73	4335,73	4335,73	4335,73	4335,73
848599	405001	4364	4335,69	4335,69	4335,69	4335,69	4335,69	4335,69	4335,69	4335,69
848629	404971	4312	4335,67	4335,67	4335,67	4335,67	4335,67	4335,67	4335,67	4335,67
848659	404941	4302	4335,63	4335,63	4335,63	4335,63	4335,63	4335,63	4335,63	4335,63
848689	404911	4320	4335,61	4335,61	4335,61	4335,61	4335,61	4335,61	4335,61	4335,61
848719	404881	4373,33	4335,56	4335,56	4335,56	4335,56	4335,56	4335,56	4335,56	4335,56
848749	404851	4282	4335,5	4335,5	4335,5	4335,5	4335,5	4335,5	4335,5	4335,5
848779	404821	4239,33	4335,39	4335,39	4335,39	4335,39	4335,39	4335,39	4335,39	4335,39
848809	404791	4362,67	4335,36	4335,36	4335,36	4335,36	4335,36	4335,36	4335,36	4335,36
848839	404761	4280,67	4335,29	4335,29	4335,29	4335,29	4335,29	4335,3	4335,29	4335,3
848869	404731	4278,67	4335,23	4335,23	4335,23	4335,23	4335,23	4335,23	4335,23	4335,23
848899	404701	4456,67	4335,09	4335,09	4335,09	4335,09	4335,09	4335,09	4335,09	4335,09
848929	404671	4272,67	4335,01	4335,01	4335,01	4335,01	4335,02	4335,02	4335,01	4335,02
848959	404641	4280	4334,95	4334,95	4334,95	4334,95	4334,95	4334,95	4334,95	4334,95
848989	404611	4379,33	4334,9	4334,9	4334,9	4334,9	4334,9	4334,9	4334,9	4334,9
849019	404581	4266,67	4334,82	4334,82	4334,82	4334,82	4334,82	4334,82	4334,82	4334,82
849049	404551	4309,33	4334,79	4334,79	4334,79	4334,79	4334,79	4334,79	4334,79	4334,79
849079	404521	4453,33	4334,65	4334,65	4334,65	4334,65	4334,65	4334,65	4334,65	4334,66
849109	404491	4459,33	4334,5	4334,5	4334,5	4334,5	4334,51	4334,51	4334,5	4334,51
849139	404461	4340,67	4334,5	4334,5	4334,5	4334,5	4334,5	4334,5	4334,5	4334,51
849169	404431	4282,67	4334,44	4334,44	4334,44	4334,44	4334,44	4334,44	4334,44	4334,45

849199	404401	4358	4334,41	4334,41	4334,41	4334,41	4334,42	4334,42	4334,41	4334,42
849229	404371	4329,33	4334,4	4334,4	4334,4	4334,4	4334,41	4334,41	4334,4	4334,42
849259	404341	4314	4334,38	4334,38	4334,38	4334,38	4334,39	4334,39	4334,38	4334,4
849289	404311	4348,67	4334,36	4334,36	4334,36	4334,36	4334,38	4334,37	4334,36	4334,39
849319	404281	4377,33	4334,31	4334,31	4334,31	4334,31	4334,33	4334,33	4334,31	4334,34
849349	404251	4364,67	4334,28	4334,28	4334,28	4334,28	4334,3	4334,29	4334,28	4334,31
849379	404221	4433,33	4334,16	4334,16	4334,16	4334,16	4334,19	4334,18	4334,16	4334,2
849409	404191	4384,67	4334,1	4334,1	4334,1	4334,1	4334,13	4334,12	4334,1	4334,15
849439	404161	4320	4334,09	4334,09	4334,09	4334,09	4334,12	4334,11	4334,09	4334,14
849469	404131	4356	4334,06	4334,06	4334,06	4334,06	4334,1	4334,09	4334,06	4334,13
849499	404101	4264	4333,98	4333,98	4333,98	4333,98	4334,03	4334,01	4333,98	4334,06
849529	404071	4338	4333,98	4333,98	4333,98	4333,98	4334,03	4334,01	4333,98	4334,07
849559	404041	4446,67	4333,84	4333,84	4333,84	4333,84	4333,91	4333,88	4333,84	4333,95
849589	404011	4294,67	4333,8	4333,8	4333,8	4333,8	4333,88	4333,84	4333,8	4333,93
849619	403981	4386,67	4333,74	4333,74	4333,74	4333,74	4333,83	4333,79	4333,74	4333,89
849649	403951	4325,33	4333,73	4333,73	4333,73	4333,73	4333,84	4333,79	4333,73	4333,9
849679	403921	4240,67	4333,62	4333,62	4333,62	4333,62	4333,75	4333,69	4333,62	4333,82
849709	403891	4274	4333,55	4333,55	4333,55	4333,55	4333,71	4333,63	4333,55	4333,79
849739	403861	4270,67	4333,48	4333,48	4333,48	4333,48	4333,66	4333,57	4333,48	4333,76
849769	403831	4293,33	4333,43	4333,43	4333,43	4333,43	4333,65	4333,53	4333,43	4333,75
849799	403801	4430,67	4333,32	4333,32	4333,32	4333,32	4333,57	4333,43	4333,32	4333,69
849829	403771	4289,33	4333,26	4333,26	4333,26	4333,27	4333,57	4333,4	4333,26	4333,71
849859	403741	4308	4333,23	4333,23	4333,23	4333,24	4333,59	4333,39	4333,23	4333,75
849889	403711	4322,67	4333,22	4333,22	4333,22	4333,23	4333,64	4333,4	4333,22	4333,82
849919	403681	4318	4333,2	4333,2	4333,2	4333,21	4333,69	4333,41	4333,2	4333,89
849949	403651	4280	4333,14	4333,14	4333,14	4333,15	4333,7	4333,37	4333,14	4333,94
849979	403621	4371,33	4333,1	4333,1	4333,1	4333,1	4333,75	4333,36	4333,1	4334,02
850009	403591	4303,33	4333,06	4333,06	4333,06	4333,07	4333,82	4333,36	4333,06	4334,12
850039	403561	4349,33	4333,04	4333,04	4333,04	4333,05	4333,92	4333,38	4333,04	4334,27
850069	403531	4428,67	4332,93	4332,93	4332,93	4332,94	4333,95	4333,32	4332,93	4334,34
850099	403501	4358,67	4332,9	4332,9	4332,9	4332,91	4334,07	4333,34	4332,9	4334,52
850129	403471	4314	4332,88	4332,88	4332,88	4332,89	4334,23	4333,37	4332,88	4334,73
850159	403441	4233,33	4332,76	4332,76	4332,76	4332,78	4334,32	4333,32	4332,76	4334,88
850189	403411	4168,67	4332,57	4332,57	4332,57	4332,59	4334,36	4333,2	4332,57	4335
850219	403381	4350	4332,55	4332,55	4332,55	4332,57	4334,6	4333,26	4332,55	4335,32
850249	403351	4312,67	4332,53	4332,53	4332,53	4332,55	4334,87	4333,33	4332,53	4335,69
850279	403321	4232	4332,41	4332,41	4332,41	4332,44	4335,09	4333,31	4332,41	4336,01
850309	403291	4283,33	4332,36	4332,36	4332,36	4332,38	4335,41	4333,37	4332,36	4336,45
850339	403261	4284	4332,3	4332,3	4332,3	4332,33	4335,78	4333,44	4332,3	4336,95
850369	403231	4330	4332,3	4332,3	4332,3	4332,33	4336,26	4333,58	4332,3	4337,57
850399	403201	4344,67	4332,28	4332,28	4332,28	4332,32	4336,78	4333,72	4332,28	4338,26
850429	403171	4387,33	4332,22	4332,22	4332,22	4332,27	4337,32	4333,83	4332,22	4338,99
850459	403141	4200	4332,06	4332,06	4332,06	4332,12	4337,85	4333,88	4332,06	4339,72
850489	403111	4302	4332,03	4332,03	4332,03	4332,09	4338,57	4334,06	4332,03	4340,66
850519	403081	4330	4332,03	4332,03	4332,03	4332,1	4339,41	4334,3	4332,03	4341,76
850549	403051	4406	4331,94	4331,94	4331,94	4332,02	4340,27	4334,47	4331,94	4342,88
850579	403021	4368	4331,9	4331,9	4331,9	4332	4341,27	4334,73	4331,9	4344,2

This journal is © The Royal Society of Chemistry 20xx

850609	402991	4338	4331,89	4331,89	4331,89	4332	4342,43	4335,05	4331,89	4345,7
850639	402961	4388	4331,83	4331,83	4331,83	4331,96	4343,65	4335,35	4331,83	4347,3
850669	402931	4315,33	4331,81	4331,81	4331,81	4331,96	4345,05	4335,72	4331,81	4349,12
850699	402901	4263,33	4331,73	4331,73	4331,73	4331,9	4346,54	4336,08	4331,73	4351,06
850729	402871	4343,33	4331,71	4331,71	4331,71	4331,91	4348,25	4336,56	4331,71	4353,3
850759	402841	4358,67	4331,68	4331,68	4331,68	4331,91	4350,12	4337,07	4331,68	4355,73
850789	402811	4372,67	4331,63	4331,63	4331,63	4331,89	4352,16	4337,61	4331,63	4358,39
850819	402781	4417,33	4331,53	4331,53	4331,53	4331,83	4354,34	4338,15	4331,53	4361,25
850849	402751	4460,67	4331,38	4331,38	4331,38	4331,72	4356,69	4338,71	4331,38	4364,35
850879	402721	4442,67	4331,25	4331,25	4331,25	4331,64	4359,29	4339,37	4331,25	4367,78
850909	402691	4406	4331,17	4331,17	4331,17	4331,6	4362,16	4340,14	4331,17	4371,58
850939	402661	4398,67	4331,09	4331,09	4331,09	4331,59	4365,31	4341	4331,09	4375,72
850969	402631	4340	4331,08	4331,08	4331,08	4331,64	4368,8	4342	4331,08	4380,3
850999	402601	4406,67	4330,99	4330,99	4330,99	4331,63	4372,51	4343,04	4330,99	4385,2
851029	402571	4275,33	4330,92	4330,92	4330,93	4331,66	4376,54	4344,21	4330,92	4390,55
851059	402541	4373,33	4330,87	4330,87	4330,88	4331,71	4380,89	4345,5	4330,87	4396,35
851089	402511	4417,33	4330,77	4330,77	4330,78	4331,72	4385,52	4346,84	4330,77	4402,53
851119	402481	4358,67	4330,74	4330,74	4330,74	4331,81	4390,54	4348,38	4330,74	4409,25
851149	402451	4382	4330,68	4330,68	4330,68	4331,89	4395,88	4350,06	4330,68	4416,47
851179	402421	4313,33	4330,66	4330,66	4330,66	4332,04	4401,61	4351,92	4330,66	4424,24
851209	402391	4538,67	4330,42	4330,42	4330,42	4331,97	4407,45	4353,71	4330,42	4432,3
851239	402361	4476,67	4330,25	4330,25	4330,25	4332	4413,72	4355,74	4330,25	4440,96
851269	402331	4404	4330,16	4330,16	4330,17	4332,14	4420,4	4358,02	4330,16	4450,24
851299	402301	4483,33	4329,98	4329,98	4329,99	4332,21	4427,32	4360,45	4329,98	4460,03
851329	402271	4419,33	4329,88	4329,88	4329,88	4332,39	4434,62	4363,16	4329,88	4470,42
851359	402241	4544	4329,63	4329,63	4329,64	4332,45	4442,06	4365,93	4329,63	4481,2
851389	402211	4516,67	4329,41	4329,41	4329,42	4332,58	4449,79	4368,95	4329,41	4492,51
851419	402181	4542,67	4329,16	4329,16	4329,17	4332,72	4457,7	4372,2	4329,16	4504,31
851449	402151	4510	4328,95	4328,95	4328,96	4332,95	4465,81	4375,81	4328,95	4516,67
851479	402121	4561,33	4328,68	4328,68	4328,69	4333,16	4473,97	4379,62	4328,68	4529,41
851509	402091	4507,33	4328,47	4328,47	4328,49	4333,49	4482,24	4383,78	4328,47	4542,59
851539	402061	4666,67	4328,08	4328,08	4328,09	4333,68	4490,3	4388,05	4328,08	4555,9
851569	402031	4500	4327,88	4327,88	4327,9	4334,14	4498,44	4392,89	4327,88	4569,74
851599	402001	4590	4327,57	4327,57	4327,59	4334,57	4506,26	4398,02	4327,57	4583,74
851629	401971	4564	4327,29	4327,29	4327,32	4335,11	4513,81	4403,53	4327,29	4597,89
851659	401941	4624,67	4326,95	4326,95	4326,98	4335,66	4520,89	4409,34	4326,95	4612,02
851689	401911	4606,67	4326,62	4326,62	4326,65	4336,31	4527,49	4415,55	4326,62	4626,14
851719	401881	4552,67	4326,36	4326,36	4326,4	4337,14	4533,54	4422,34	4326,36	4640,35
851749	401851	4654,67	4325,97	4325,97	4326,02	4337,97	4538,77	4429,47	4325,97	4654,32
851779	401821	4694,67	4325,54	4325,54	4325,59	4338,87	4543,17	4437	4325,54	4668,01
851809	401791	4697,33	4325,11	4325,11	4325,17	4339,89	4546,68	4444,99	4325,11	4681,41
851839	401761	4815,33	4324,54	4324,54	4324,61	4340,91	4549,12	4453,31	4324,54	4694,33
851869	401731	4729,33	4324,06	4324,06	4324,14	4342,21	4550,65	4462,4	4324,06	4707,21
851899	401701	4731,33	4323,59	4323,59	4323,68	4343,68	4551,15	4472,01	4323,59	4719,75
851929	401671	4701,33	4323,15	4323,15	4323,25	4345,36	4550,63	4482,18	4323,15	4731,98
851959	401641	4668	4322,75	4322,75	4322,86	4347,27	4549,1	4492,92	4322,75	4743,92
851989	401611	4778,67	4322,21	4322,21	4322,35	4349,25	4546,41	4504,12	4322,21	4755,48

852019	401581	4695,33	4321,78	4321,78	4321,93	4351,62	4542,82	4516,15	4321,78	4767,18
852049	401551	4750	4321,28	4321,28	4321,45	4354,17	4538,23	4528,67	4321,28	4778,69
852079	401521	4790	4320,73	4320,73	4320,93	4356,93	4532,74	4541,73	4320,73	4790,13
852109	401491	4838,67	4320,13	4320,13	4320,35	4359,91	4526,4	4555,3	4320,13	4801,59
852139	401461	4796,67	4319,57	4319,57	4319,82	4363,26	4519,43	4569,58	4319,57	4813,37
852169	401431	4880	4318,92	4318,92	4319,2	4366,91	4511,76	4584,45	4318,92	4825,58
852199	401401	4852,67	4318,29	4318,29	4318,61	4370,96	4503,63	4599,91	4318,29	4838,23
852229	401371	4814,67	4317,71	4317,71	4318,08	4375,41	4495,15	4615,95	4317,71	4851,45
852259	401341	4806,67	4317,14	4317,14	4317,55	4380,27	4486,41	4632,53	4317,14	4865,33
852289	401311	4896,67	4316,47	4316,47	4316,93	4385,5	4477,37	4649,61	4316,47	4880,02
852319	401281	4944	4315,73	4315,73	4316,26	4391,23	4468,18	4667,16	4315,73	4895,63
852349	401251	4898	4315,06	4315,06	4315,64	4397,51	4459,03	4685,17	4315,06	4912,19
852379	401221	4912,67	4314,36	4314,36	4315,02	4404,29	4449,91	4703,53	4314,36	4929,68
852409	401191	4948	4313,62	4313,62	4314,36	4411,57	4440,87	4722,19	4313,62	4948,14
852439	401161	4962,67	4312,86	4312,86	4313,7	4419,54	4431,99	4741,16	4312,86	4967,81
852469	401131	5027,33	4312,03	4312,03	4312,97	4428,16	4423,25	4760,23	4312,03	4988,53
852499	401101	5027,33	4311,19	4311,19	4312,24	4437,45	4414,76	4779,39	4311,19	5010,27
852529	401071	4990,67	4310,4	4310,4	4311,58	4447,47	4406,61	4798,61	4310,4	5033,07
852559	401041	5126,67	4309,45	4309,45	4310,77	4458,08	4398,61	4817,61	4309,45	5056,72
852589	401011	5063,33	4308,57	4308,57	4310,04	4469,76	4391	4836,42	4308,57	5081,53
852619	400981	5105,33	4307,64	4307,64	4309,29	4482,29	4383,69	4854,86	4307,64	5107,22
852649	400951	5195,33	4306,6	4306,6	4308,44	4495,59	4376,62	4872,76	4306,6	5133,6
852679	400921	5180	4305,58	4305,58	4307,63	4509,82	4369,9	4890,14	4305,58	5160,76
852709	400891	5128,67	4304,62	4304,62	4306,91	4525,08	4363,6	4906,89	4304,62	5188,61
852739	400861	5228,67	4303,54	4303,54	4306,09	4541,57	4357,51	4922,5	4303,54	5217,04
852769	400831	5204,67	4302,49	4302,49	4305,32	4559,15	4351,78	4937,19	4302,49	5245,97
852799	400801	5310,67	4301,31	4301,31	4304,45	4577,72	4346,25	4950,69	4301,31	5275,18
852829	400771	5315,33	4300,13	4300,13	4303,61	4597,41	4341,04	4963,04	4300,13	5304,71
852859	400741	5470	4298,76	4298,76	4302,63	4618,21	4335,93	4973,73	4298,76	5334,21
852889	400711	5351,33	4297,54	4297,54	4301,82	4640,71	4331,25	4982,86	4297,54	5364,04
852919	400681	5320,67	4296,34	4296,34	4301,09	4664,52	4326,87	4990,53	4296,34	5393,98
852949	400651	5467,33	4294,98	4294,98	4300,22	4689,47	4322,58	4996,45	4294,98	5423,79
852979	400621	5411,33	4293,67	4293,67	4299,47	4715,82	4318,58	5000,8	4293,67	5453,65
853009	400591	5468,67	4292,3	4292,3	4298,7	4743,69	4314,75	5002,98	4292,3	5483,22
853039	400561	5428,67	4290,97	4290,97	4298,04	4773,28	4311,17	5003,26	4290,97	5512,82
853069	400531	5575,33	4289,48	4289,48	4297,26	4804,12	4307,61	5001,61	4289,48	5542,17
853099	400501	5570,67	4287,98	4287,98	4296,54	4836,4	4304,24	4998,22	4287,98	5571,45
853129	400471	5556	4286,5	4286,5	4295,92	4870,12	4301,06	4993,1	4286,5	5600,7
853159	400441	5597,33	4284,97	4284,97	4295,33	4905,56	4297,98	4985,71	4284,97	5629,68
853189	400411	5554	4283,49	4283,49	4294,87	4942,64	4295,1	4976,67	4283,49	5658,82
853219	400381	5599,33	4281,95	4281,95	4294,44	4981,06	4292,3	4966,02	4281,95	5687,95
853249	400351	5694	4280,31	4280,31	4293,97	5020,71	4289,51	4953,76	4280,31	5717,04
853279	400321	5804,67	4278,53	4278,53	4293,51	5061,56	4286,7	4939,93	4278,53	5746,12
853309	400291	5907,33	4276,63	4276,63	4293,03	5103,91	4283,87	4924,29	4276,63	5775,22
853339	400261	5847,33	4274,79	4274,79	4292,73	5147,52	4281,21	4907,58	4274,79	5804,66
853369	400231	5868	4272,93	4272,93	4292,51	5192,15	4278,6	4889,82	4272,93	5834,28
853399	400201	5785,33	4271,17	4271,17	4292,52	5237,81	4276,17	4871,23	4271,17	5864,24

This journal is © The Royal Society of Chemistry 20xx

853429	400171	5928	4269,23	4269,23	4292,54	5284,17	4273,64	4851,59	4269,23	5894,24
853459	400141	5863,33	4267,37	4267,37	4292,77	5331,46	4271,25	4831,21	4267,37	5924,57
853489	400111	5984,67	4265,37	4265,37	4293	5379,02	4268,78	4810,19	4265,37	5954,88
853519	400081	5958,67	4263,39	4263,39	4293,43	5426,86	4266,38	4788,79	4263,39	5985,28
853549	400051	6186,67	4261,15	4261,15	4293,77	5474,5	4263,77	4766,82	4261,15	6015,42
853579	400021	6091,33	4259,01	4259,01	4294,45	5522,08	4261,3	4744,73	4259,01	6045,53
853609	399991	6018	4256,96	4256,96	4295,39	5569,15	4258,96	4722,66	4256,96	6075,29
853639	399961	6162	4254,74	4254,74	4296,37	5615,26	4256,48	4700,45	4254,74	6104,35
853669	399931	6156,67	4252,52	4252,52	4297,56	5660,34	4254,03	4678,36	4252,52	6132,74
853699	399901	6231,33	4250,21	4250,21	4298,93	5704,05	4251,53	4656,35	4250,21	6160,23
853729	399871	6170,67	4247,96	4247,96	4300,63	5745,94	4249,11	4634,72	4247,96	6186,51
853759	399841	6221,33	4245,66	4245,66	4302,52	5785,4	4246,65	4613,41	4245,66	6211,01
853789	399811	6151,33	4243,44	4243,44	4304,74	5822,61	4244,29	4592,57	4243,44	6233,9
853819	399781	6359,33	4240,97	4240,97	4306,98	5856,98	4241,71	4571,9	4240,97	6254,67
853849	399751	6207,33	4238,67	4238,67	4309,76	5888,69	4239,31	4551,86	4238,67	6273,6
853879	399721	6276	4236,3	4236,3	4312,76	5916,35	4236,84	4532,41	4236,3	6289,47
853909	399691	6269,33	4233,92	4233,92	4316,06	5940,12	4234,39	4513,5	4233,92	6302,31
853939	399661	6406,67	4231,39	4231,39	4319,51	5960,01	4231,79	4494,98	4231,39	6312,12
853969	399631	6334	4228,93	4228,93	4323,37	5976,07	4229,28	4477,09	4228,93	6319,01
853999	399601	6242	4226,58	4226,58	4327,76	5988,19	4226,88	4459,91	4226,58	6322,99
854029	399571	6291,33	4224,17	4224,17	4332,44	5994,64	4224,43	4443,42	4224,17	6322,4
854059	399541	6205,33	4221,86	4221,86	4337,55	5996,71	4222,08	4427,6	4221,86	6318,36
854089	399511	6341,33	4219,39	4219,39	4342,85	5994,26	4219,57	4412,2	4219,39	6310,72
854119	399481	6199,33	4217,08	4217,08	4348,7	5987,61	4217,23	4397,52	4217,08	6299,84
854149	399451	6284	4214,66	4214,66	4354,89	5976,34	4214,8	4383,38	4214,66	6285,42
854179	399421	6268,67	4212,27	4212,27	4361,46	5959,52	4212,38	4369,93	4212,27	6266,48
854209	399391	6231,33	4209,91	4209,91	4368,41	5938,69	4210	4357,05	4209,91	6244,43
854239	399361	6230	4207,55	4207,55	4375,71	5913,96	4207,63	4344,7	4207,55	6219,34
854269	399331	6120	4205,32	4205,32	4383,54	5885,61	4205,39	4332,99	4205,32	6191,56
854299	399301	6081,33	4203,13	4203,13	4391,79	5853,39	4203,19	4321,9	4203,13	6160,88
854329	399271	6138	4200,87	4200,87	4400,29	5817,16	4200,92	4311,31	4200,87	6127,06
854359	399241	6140,67	4198,61	4198,61	4409,07	5778,09	4198,65	4301,16	4198,61	6091,14
854389	399211	6021,33	4196,48	4196,48	4418,27	5736,56	4196,51	4291,58	4196,48	6053,48
854419	399181	6010,67	4194,36	4194,36	4427,79	5692,69	4194,39	4282,45	4194,36	6014,23
854449	399151	6014	4192,24	4192,24	4437,53	5646,43	4192,26	4273,82	4192,24	5973,32
854479	399121	5922	4190,22	4190,22	4447,54	5598,41	4190,24	4265,71	4190,22	5931,24
854509	399091	6058	4188,04	4188,04	4457,53	5548,95	4188,06	4257,81	4188,04	5888,22
854539	399061	5918,67	4186,02	4186,02	4467,78	5498,61	4186,04	4250,41	4186,02	5844,77
854569	399031	5791,33	4184,15	4184,15	4478,22	5447,61	4184,16	4243,5	4184,15	5801,05
854599	399001	5743,33	4182,33	4182,33	4488,68	5396,07	4182,34	4237,06	4182,33	5757,17
854629	398971	5738	4180,51	4180,51	4499,06	5344,38	4180,52	4230,93	4180,51	5713,34
854659	398941	5676,67	4178,77	4178,77	4509,36	5292,81	4178,77	4225,14	4178,77	5669,79
854689	398911	5564	4177,15	4177,15	4519,57	5241,63	4177,16	4219,76	4177,15	5626,66
854719	398881	5638	4175,45	4175,45	4529,31	5190,77	4175,45	4214,55	4175,45	5583,74
854749	398851	5506	4173,89	4173,89	4538,8	5140,92	4173,9	4209,79	4173,89	5541,74
854779	398821	5569,33	4172,26	4172,26	4547,77	5091,85	4172,27	4205,18	4172,26	5500,28
854809	398791	5556	4170,65	4170,65	4556,23	5043,74	4170,65	4200,79	4170,65	5459,47

This journal is © The Royal Society of Chemistry 20xx

Please do not adjust margins

854839	398761	5454	4169,15	4169,15	4564,15	4996,79	4169,15	4196,71	4169,15	5419,35
854869	398731	5433,33	4167,68	4167,68	4571,23	4951,06	4167,68	4192,87	4167,68	5379,81
854899	398701	5467,33	4166,16	4166,16	4577,5	4906,91	4166,16	4189,19	4166,16	5341,27
854929	398671	5398,67	4164,72	4164,72	4583,02	4864,15	4164,72	4185,74	4164,72	5303,47
854959	398641	5212,67	4163,5	4163,5	4587,87	4822,96	4163,5	4182,66	4163,5	5266,5
854989	398611	5208	4162,28	4162,28	4591,61	4783,17	4162,28	4179,72	4162,28	5229,94
855019	398581	5177,33	4161,09	4161,09	4594,23	4745,01	4161,09	4176,97	4161,09	5194,02
855049	398551	5014,67	4160,1	4160,1	4596	4708,79	4160,1	4174,54	4160,1	5159,14
855079	398521	4995,33	4159,12	4159,12	4596,73	4674,04	4159,12	4172,25	4159,12	5124,77
855109	398491	5136,67	4157,98	4157,98	4596,23	4640,54	4157,98	4169,89	4157,98	5090,7
855139	398461	5043,33	4156,95	4156,95	4594,46	4608,57	4156,95	4167,74	4156,95	5056,87
855169	398431	4979,33	4155,99	4155,99	4591,6	4578,37	4155,99	4165,77	4155,99	5023,77
855199	398401	4896,67	4155,12	4155,12	4587,79	4549,84	4155,12	4163,99	4155,12	4991,37
855229	398371	4928,67	4154,22	4154,22	4582,92	4522,61	4154,22	4162,24	4154,22	4959,32
855259	398341	4950,67	4153,29	4153,29	4577,02	4496,65	4153,29	4160,53	4153,29	4927,62
855289	398311	4886,67	4152,44	4152,44	4569,93	4472,06	4152,44	4158,96	4152,44	4896,07
855319	398281	4786	4151,7	4151,7	4562,07	4449,16	4151,7	4157,59	4151,7	4865,43
855349	398251	4717,33	4151,04	4151,04	4553,49	4427,6	4151,04	4156,35	4151,04	4835,38
855379	398221	4864	4150,2	4150,2	4543,98	4407,02	4150,2	4154,99	4150,2	4805,58
855409	398191	4783,33	4149,46	4149,46	4533,83	4387,63	4149,46	4153,77	4149,46	4776,3
855439	398161	4686,67	4148,84	4148,84	4523,02	4369,42	4148,84	4152,7	4148,84	4747,47
855469	398131	4533,33	4148,39	4148,39	4511,89	4352,76	4148,39	4151,86	4148,39	4719,74
855499	398101	4660	4147,79	4147,79	4500,18	4336,9	4147,79	4150,91	4147,79	4692,41
855529	398071	4746,67	4147,09	4147,09	4487,98	4321,86	4147,09	4149,89	4147,09	4665,54
855559	398041	4584	4146,58	4146,58	4475,64	4307,88	4146,58	4149,08	4146,58	4639,44
855589	398011	4622	4146,03	4146,03	4463,01	4294,76	4146,03	4148,26	4146,03	4613,97
855619	397981	4633,33	4145,46	4145,46	4450,23	4282,63	4145,46	4147,46	4145,46	4589,4
855649	397951	4516,67	4145,03	4145,03	4437,51	4271,36	4145,03	4146,81	4145,03	4565,63
855679	397921	4502,67	4144,61	4144,61	4424,78	4260,82	4144,61	4146,2	4144,61	4542,58
855709	397891	4585,33	4144,09	4144,09	4412	4250,85	4144,09	4145,51	4144,09	4520,17
855739	397861	4615,33	4143,54	4143,54	4399,29	4241,57	4143,54	4144,81	4143,54	4498,58
855769	397831	4437,33	4143,2	4143,2	4386,95	4233,2	4143,2	4144,33	4143,2	4478,07
855799	397801	4484	4142,8	4142,8	4374,72	4225,32	4142,8	4143,8	4142,8	4458,23
855829	397771	4463,33	4142,43	4142,43	4362,73	4217,98	4142,43	4143,32	4142,43	4439,17
855859	397741	4414	4142,11	4142,11	4351,11	4211,19	4142,11	4142,9	4142,11	4420,98
855889	397711	4347,33	4141,87	4141,87	4339,87	4205,05	4141,87	4142,57	4141,87	4403,75
855919	397681	4457,33	4141,5	4141,5	4328,81	4199,25	4141,5	4142,12	4141,5	4387,17
855949	397651	4434,67	4141,16	4141,16	4318,08	4193,86	4141,16	4141,71	4141,16	4371,33
855979	397621	4414,67	4140,84	4140,84	4307,7	4188,88	4140,84	4141,33	4140,84	4356,22
856009	397591	4330	4140,62	4140,62	4297,88	4184,34	4140,62	4141,05	4140,62	4342,02
856039	397561	4353,33	4140,37	4140,37	4288,38	4180,19	4140,37	4140,75	4140,37	4328,58
856069	397531	4273,33	4140,22	4140,22	4279,33	4176,45	4140,22	4140,55	4140,22	4315,89
856099	397501	4232	4140,11	4140,11	4270,68	4173,03	4140,11	4140,4	4140,11	4303,89
856129	397471	4372	4139,84	4139,84	4262,25	4169,71	4139,84	4140,1	4139,84	4292,37
856159	397441	4280	4139,68	4139,68	4254,37	4166,74	4139,68	4139,9	4139,68	4281,66
856189	397411	4342	4139,44	4139,44	4246,77	4163,99	4139,44	4139,64	4139,44	4271,52
856219	397381	4276	4139,28	4139,28	4239,59	4161,52	4139,28	4139,46	4139,28	4262

 This journal is © The Royal Society of Chemistry 20xx

Please do not adjust margins

856249	397351	4192,67	4139,22	4139,22	4232,84	4159,33	4139,22	4139,37	4139,22	4253,1
856279	397321	4296,67	4139,04	4139,04	4226,34	4157,19	4139,04	4139,17	4139,04	4244,63
856309	397291	4368	4138,77	4138,77	4220,15	4155,15	4138,77	4138,89	4138,77	4236,65
856339	397261	4219,33	4138,68	4138,68	4214,43	4153,47	4138,68	4138,78	4138,68	4229,33
856369	397231	4290,67	4138,5	4138,5	4208,93	4151,84	4138,5	4138,59	4138,5	4222,36
856399	397201	4298	4138,31	4138,31	4203,7	4150,32	4138,31	4138,39	4138,31	4215,79
856429	397171	4241,33	4138,19	4138,19	4198,89	4148,98	4138,19	4138,26	4138,19	4209,75
856459	397141	4253,33	4138,06	4138,06	4194,36	4147,76	4138,06	4138,12	4138,06	4204,11
856489	397111	4280	4137,89	4137,89	4190,04	4146,61	4137,89	4137,94	4137,89	4198,81
856519	397081	4264	4137,74	4137,74	4185,99	4145,57	4137,74	4137,79	4137,74	4193,86
856549	397051	4177,33	4137,7	4137,7	4182,28	4144,71	4137,7	4137,74	4137,7	4189,33
856579	397021	4244,67	4137,57	4137,57	4178,77	4143,84	4137,57	4137,61	4137,57	4185,08
856609	396991	4211,33	4137,49	4137,49	4175,52	4143,1	4137,49	4137,52	4137,49	4181,16
856639	396961	4248	4137,36	4137,36	4172,43	4142,38	4137,36	4137,38	4137,36	4177,47
856669	396931	4316,67	4137,15	4137,15	4169,44	4141,64	4137,15	4137,17	4137,15	4173,95
856699	396901	4212,67	4137,06	4137,06	4166,76	4141,06	4137,06	4137,08	4137,06	4170,78
856729	396871	4136	4137,06	4137,06	4164,39	4140,62	4137,06	4137,08	4137,06	4167,96
856759	396841	4255,33	4136,92	4136,92	4162,04	4140,1	4136,92	4136,94	4136,92	4165,23
856789	396811	4299,33	4136,73	4136,73	4159,78	4139,56	4136,73	4136,74	4136,73	4162,62
856819	396781	4176	4136,69	4136,69	4157,81	4139,2	4136,69	4136,7	4136,69	4160,34
856849	396751	4229,33	4136,58	4136,58	4155,93	4138,81	4136,58	4136,59	4136,58	4158,16
856879	396721	4072	4136,5	4136,5	4154,23	4138,48	4136,5	4136,51	4136,5	4156,21
856909	396691	4184	4136,45	4136,45	4152,66	4138,2	4136,45	4136,45	4136,45	4154,42
856939	396661	4203,33	4136,37	4136,37	4151,18	4137,92	4136,37	4136,37	4136,37	4152,74
856969	396631	4234	4136,26	4136,26	4149,77	4137,63	4136,26	4136,26	4136,26	4151,15
856999	396601	4214,67	4136,16	4136,16	4148,49	4137,38	4136,16	4136,17	4136,16	4149,71
857029	396571	4212	4136,08	4136,08	4147,32	4137,15	4136,08	4136,08	4136,08	4148,39
857059	396541	4244	4135,95	4135,95	4146,19	4136,9	4135,95	4135,95	4135,95	4147,14
857089	396511	4254,67	4135,81	4135,81	4145,12	4136,65	4135,81	4135,81	4135,81	4145,96
857119	396481	4186	4135,75	4135,75	4144,21	4136,49	4135,75	4135,75	4135,75	4144,95
857149	396451	4202,67	4135,67	4135,67	4143,36	4136,32	4135,67	4135,68	4135,67	4144
857179	396421	4044,67	4135,57	4135,57	4142,54	4136,14	4135,57	4135,57	4135,57	4143,11
857209	396391	4208	4135,48	4135,48	4141,81	4135,98	4135,48	4135,48	4135,48	4142,31
857239	396361	4317,33	4135,27	4135,27	4141	4135,71	4135,27	4135,27	4135,27	4141,43
857269	396331	4204,67	4135,19	4135,19	4140,36	4135,57	4135,19	4135,19	4135,19	4140,75
857299	396301	4210,67	4135,1	4135,1	4139,78	4135,44	4135,1	4135,1	4135,1	4140,12
857329	396271	4207,33	4135,02	4135,02	4139,25	4135,31	4135,02	4135,02	4135,02	4139,54
857359	396241	4164	4134,98	4134,98	4138,8	4135,24	4134,98	4134,98	4134,98	4139,06
857389	396211	4092	4134,93	4134,93	4138,37	4135,16	4134,93	4134,93	4134,93	4138,6
857419	396181	4154,67	4134,91	4134,91	4138,01	4135,11	4134,91	4134,91	4134,91	4138,2
857449	396151	4213,33	4134,82	4134,82	4137,61	4134,99	4134,82	4134,82	4134,82	4137,78
857479	396121	4144	4134,81	4134,81	4137,32	4134,96	4134,81	4134,81	4134,81	4137,47
857509	396091	4157,33	4134,78	4134,78	4137,04	4134,91	4134,78	4134,78	4134,78	4137,17
857539	396061	4142,67	4134,77	4134,77	4136,79	4134,89	4134,77	4134,77	4134,77	4136,91
857569	396031	4132	4134,77	4134,77	4136,58	4134,87	4134,77	4134,77	4134,77	4136,68
857599	396001	4166	4134,73	4134,73	4136,36	4134,82	4134,73	4134,73	4134,73	4136,44
857629	395971	4118	4134,71	4134,71	4136,17	4134,79	4134,71	4134,71	4134,71	4136,24

857659	395941	4138	4134,71	4134,71	4136,01	4134,77	4134,71	4134,71	4134,71	4136,07
857689	395911	4193,33	4134,64	4134,64	4135,8	4134,7	4134,64	4134,64	4134,64	4135,86
857719	395881	4236	4134,52	4134,52	4135,56	4134,57	4134,52	4134,52	4134,52	4135,61
857749	395851	4222,67	4134,42	4134,42	4135,35	4134,46	4134,42	4134,42	4134,42	4135,39
857779	395821	4280,67	4134,25	4134,25	4135,07	4134,29	4134,25	4134,25	4134,25	4135,11
857809	395791	4090	4134,2	4134,2	4134,93	4134,23	4134,2	4134,2	4134,2	4134,96
857839	395761	4162	4134,17	4134,17	4134,82	4134,19	4134,17	4134,17	4134,17	4134,84
857869	395731	4208	4134,08	4134,08	4134,66	4134,1	4134,08	4134,08	4134,08	4134,68
857899	395701	4092	4134,03	4134,03	4134,55	4134,05	4134,03	4134,03	4134,03	4134,56
857929	395671	4260	4133,88	4133,88	4134,34	4133,9	4133,88	4133,88	4133,88	4134,36
857959	395641	4200,67	4133,81	4133,81	4134,21	4133,82	4133,81	4133,81	4133,81	4134,22
857989	395611	4012	4133,66	4133,66	4134,02	4133,68	4133,66	4133,66	4133,66	4134,03
858019	395581	4170	4133,62	4133,62	4133,94	4133,63	4133,62	4133,62	4133,62	4133,95
858049	395551	4006,67	4133,47	4133,47	4133,75	4133,48	4133,47	4133,47	4133,47	4133,76
858079	395521	4198	4133,4	4133,4	4133,65	4133,41	4133,4	4133,4	4133,4	4133,65
858109	395491	4167,33	4133,36	4133,36	4133,58	4133,37	4133,36	4133,36	4133,36	4133,58
858139	395461	4160,67	4133,33	4133,33	4133,52	4133,33	4133,33	4133,33	4133,33	4133,52
858169	395431	4197,33	4133,25	4133,25	4133,42	4133,26	4133,25	4133,25	4133,25	4133,43
858199	395401	4195,33	4133,18	4133,18	4133,33	4133,18	4133,18	4133,18	4133,18	4133,33
858229	395371	4228,67	4133,07	4133,07	4133,2	4133,07	4133,07	4133,07	4133,07	4133,2
858259	395341	4190	4133	4133	4133,12	4133	4133	4133	4133	4133,12
858289	395311	4188,67	4132,94	4132,94	4133,04	4132,94	4132,94	4132,94	4132,94	4133,04
858319	395281	4157,33	4132,91	4132,91	4133	4132,91	4132,91	4132,91	4132,91	4133
858349	395251	4067,33	4132,83	4132,83	4132,91	4132,83	4132,83	4132,83	4132,83	4132,91
858379	395221	4176,67	4132,78	4132,78	4132,85	4132,78	4132,78	4132,78	4132,78	4132,85
858409	395191	4101,33	4132,74	4132,74	4132,8	4132,75	4132,74	4132,74	4132,74	4132,8
858439	395161	4113,33	4132,72	4132,72	4132,77	4132,72	4132,72	4132,72	4132,72	4132,77
858469	395131	4189,33	4132,66	4132,66	4132,7	4132,66	4132,66	4132,66	4132,66	4132,7
858499	395101	4176,67	4132,6	4132,6	4132,64	4132,61	4132,6	4132,6	4132,6	4132,64
858529	395071	4208,67	4132,52	4132,52	4132,55	4132,52	4132,52	4132,52	4132,52	4132,55
858559	395041	4156	4132,49	4132,49	4132,52	4132,49	4132,49	4132,49	4132,49	4132,52
858589	395011	4186	4132,43	4132,43	4132,45	4132,43	4132,43	4132,43	4132,43	4132,45
858619	394981	4182,67	4132,37	4132,37	4132,39	4132,37	4132,37	4132,37	4132,37	4132,39
858649	394951	4278	4132,2	4132,2	4132,22	4132,2	4132,2	4132,2	4132,2	4132,22
858679	394921	4072	4132,13	4132,13	4132,14	4132,13	4132,13	4132,13	4132,13	4132,14
858709	394891	4075,33	4132,06	4132,06	4132,08	4132,06	4132,06	4132,06	4132,06	4132,08
858739	394861	4159,33	4132,03	4132,03	4132,04	4132,03	4132,03	4132,03	4132,03	4132,04
858769	394831	4120	4132,02	4132,02	4132,03	4132,02	4132,02	4132,02	4132,02	4132,03
858799	394801	4120	4132	4132	4132,01	4132	4132	4132	4132	4132,01
858829	394771	4162	4131,97	4131,97	4131,97	4131,97	4131,97	4131,97	4131,97	4131,97
858859	394741	4138	4131,96	4131,96	4131,97	4131,96	4131,96	4131,96	4131,96	4131,97
858889	394711	4151,33	4131,94	4131,94	4131,94	4131,94	4131,94	4131,94	4131,94	4131,94
858919	394681	4122,67	4131,93	4131,93	4131,93	4131,93	4131,93	4131,93	4131,93	4131,93
858949	394651	4178	4131,87	4131,87	4131,88	4131,87	4131,87	4131,87	4131,87	4131,88
858979	394621	4077,33	4131,81	4131,81	4131,81	4131,81	4131,81	4131,81	4131,81	4131,81
859009	394591	4074,67	4131,74	4131,74	4131,74	4131,74	4131,74	4131,74	4131,74	4131,75
859039	394561	4241,33	4131,61	4131,61	4131,62	4131,61	4131,61	4131,61	4131,61	4131,62

This journal is © The Royal Society of Chemistry 20xx

859069	394531	4144,67	4131,6	4131,6	4131,6	4131,6	4131,6	4131,6	4131,6	4131,6
859099	394501	4162	4131,56	4131,56	4131,57	4131,56	4131,56	4131,56	4131,56	4131,57
859129	394471	4060	4131,48	4131,48	4131,48	4131,48	4131,48	4131,48	4131,48	4131,48
859159	394441	4146	4131,46	4131,46	4131,46	4131,46	4131,46	4131,46	4131,46	4131,46
859189	394411	4168,67	4131,42	4131,42	4131,42	4131,42	4131,42	4131,42	4131,42	4131,42
859219	394381	4116,67	4131,4	4131,4	4131,4	4131,4	4131,4	4131,4	4131,4	4131,4
859249	394351	4107,33	4131,37	4131,37	4131,38	4131,37	4131,37	4131,37	4131,37	4131,38
859279	394321	4209,33	4131,28	4131,28	4131,28	4131,28	4131,28	4131,28	4131,28	4131,28
859309	394291	4058,67	4131,2	4131,2	4131,2	4131,2	4131,2	4131,2	4131,2	4131,2
859339	394261	4156,67	4131,17	4131,17	4131,17	4131,17	4131,17	4131,17	4131,17	4131,17
859369	394231	4186,67	4131,1	4131,1	4131,1	4131,1	4131,1	4131,1	4131,1	4131,1
859399	394201	4020	4130,97	4130,97	4130,98	4130,97	4130,97	4130,97	4130,97	4130,98
859429	394171	4118	4130,96	4130,96	4130,96	4130,96	4130,96	4130,96	4130,96	4130,96
859459	394141	4142	4130,95	4130,95	4130,95	4130,95	4130,95	4130,95	4130,95	4130,95
859489	394111	4110	4130,92	4130,92	4130,92	4130,92	4130,92	4130,92	4130,92	4130,92
859519	394081	4164	4130,88	4130,88	4130,88	4130,88	4130,88	4130,88	4130,88	4130,88
859549	394051	4149,33	4130,86	4130,86	4130,86	4130,86	4130,86	4130,86	4130,86	4130,86
859579	394021	4031,33	4130,75	4130,75	4130,75	4130,75	4130,75	4130,75	4130,75	4130,75
859609	393991	4156	4130,72	4130,72	4130,72	4130,72	4130,72	4130,72	4130,72	4130,72
859639	393961	4144	4130,7	4130,7	4130,7	4130,7	4130,7	4130,7	4130,7	4130,7
859669	393931	4112	4130,68	4130,68	4130,68	4130,68	4130,68	4130,68	4130,68	4130,68
859699	393901	4187,33	4130,61	4130,61	4130,61	4130,61	4130,61	4130,61	4130,61	4130,61
859729	393871	4203,33	4130,53	4130,53	4130,53	4130,53	4130,53	4130,53	4130,53	4130,53
859759	393841	4172,67	4130,48	4130,48	4130,48	4130,48	4130,48	4130,48	4130,48	4130,48
859789	393811	4088	4130,43	4130,43	4130,43	4130,43	4130,43	4130,43	4130,43	4130,43
859819	393781	4149,33	4130,41	4130,41	4130,41	4130,41	4130,41	4130,41	4130,41	4130,41
859849	393751	4069,33	4130,34	4130,34	4130,34	4130,34	4130,34	4130,34	4130,34	4130,34
859879	393721	4169,33	4130,29	4130,29	4130,29	4130,29	4130,29	4130,29	4130,29	4130,29
859909	393691	4130	4130,29	4130,29	4130,29	4130,29	4130,29	4130,29	4130,29	4130,29
859939	393661	4179,33	4130,23	4130,23	4130,23	4130,23	4130,23	4130,23	4130,23	4130,23
859969	393631	4239,33	4130,11	4130,11	4130,11	4130,11	4130,11	4130,11	4130,11	4130,11
859999	393601	4126,67	4130,1	4130,1	4130,1	4130,1	4130,1	4130,1	4130,1	4130,1
860029	393571	4142,67	4130,09	4130,09	4130,09	4130,09	4130,09	4130,09	4130,09	4130,09
860059	393541	4190	4130,02	4130,02	4130,02	4130,02	4130,02	4130,02	4130,02	4130,02
860089	393511	4114,67	4130	4130	4130	4130	4130	4130	4130	4130
860119	393481	4130	4130	4130	4130	4130	4130	4130	4130	4130
860149	393451	4124,67	4124,67	4124,67	4124,67	4124,67	4124,67	4124,67	4124,67	4124,67
860179	393421	4121,33	4121,33	4121,33	4121,33	4121,33	4121,33	4121,33	4121,33	4121,33
860209	393391	4200	4200	4200	4200	4200	4200	4200	4200	4200
860239	393361	4136	4136	4136	4136	4136	4136	4136	4136	4136
860269	393331	4161,33	4161,33	4161,33	4161,33	4161,33	4161,33	4161,33	4161,33	4161,33
860299	393301	4075,33	4075,33	4075,33	4075,33	4075,33	4075,33	4075,33	4075,33	4075,33
860329	393271	4116,67	4116,67	4116,67	4116,67	4116,67	4116,67	4116,67	4116,67	4116,67
860359	393241	4177,33	4177,33	4177,33	4177,33	4177,33	4177,33	4177,33	4177,33	4177,33
860389	393211	4156	4156	4156	4156	4156	4156	4156	4156	4156
860419	393181	4162,67	4162,67	4162,67	4162,67	4162,67	4162,67	4162,67	4162,67	4162,67
860449	393151	4136,67	4136,67	4136,67	4136,67	4136,67	4136,67	4136,67	4136,67	4136,67

This journal is © The Royal Society of Chemistry 20xx

Please do not adjust margins

Please do not adjust margins

860479	393121	4122,67	4122,67	4122,67	4122,67	4122,67	4122,67	4122,67	4122,67	4122,67
860509	393091	4065,33	4065,33	4065,33	4065,33	4065,33	4065,33	4065,33	4065,33	4065,33
860539	393061	4117,33	4117,33	4117,33	4117,33	4117,33	4117,33	4117,33	4117,33	4117,33
860569	393031	4144,67	4144,67	4144,67	4144,67	4144,67	4144,67	4144,67	4144,67	4144,67
860599	393001	4121,33	4121,33	4121,33	4121,33	4121,33	4121,33	4121,33	4121,33	4121,33
860629	392971	4166,67	4166,67	4166,67	4166,67	4166,67	4166,67	4166,67	4166,67	4166,67
860659	392941	4150	4150	4150	4150	4150	4150	4150	4150	4150
860689	392911	4225,33	4225,33	4225,33	4225,33	4225,33	4225,33	4225,33	4225,33	4225,33
860719	392881	4112,67	4112,67	4112,67	4112,67	4112,67	4112,67	4112,67	4112,67	4112,67
860749	392851	4268,67	4268,67	4268,67	4268,67	4268,67	4268,67	4268,67	4268,67	4268,67
860779	392821	4192	4192	4192	4192	4192	4192	4192	4192	4192
860809	392791	4162,67	4162,67	4162,67	4162,67	4162,67	4162,67	4162,67	4162,67	4162,67
860839	392761	4010	4010	4010	4010	4010	4010	4010	4010	4010
860869	392731	4129,33	4129,33	4129,33	4129,33	4129,33	4129,33	4129,33	4129,33	4129,33
860899	392701	4251,33	4251,33	4251,33	4251,33	4251,33	4251,33	4251,33	4251,33	4251,33
860929	392671	4148,67	4148,67	4148,67	4148,67	4148,67	4148,67	4148,67	4148,67	4148,67
860959	392641	4214	4214	4214	4214	4214	4214	4214	4214	4214
860989	392611	4120	4120	4120	4120	4120	4120	4120	4120	4120
861019	392581	4034,67	4034,67	4034,67	4034,67	4034,67	4034,67	4034,67	4034,67	4034,67
861049	392551	4145,33	4145,33	4145,33	4145,33	4145,33	4145,33	4145,33	4145,33	4145,33
861079	392521	4106,67	4106,67	4106,67	4106,67	4106,67	4106,67	4106,67	4106,67	4106,67
861109	392491	4124	4124	4124	4124	4124	4124	4124	4124	4124
861139	392461	4104	4104	4104	4104	4104	4104	4104	4104	4104
861169	392431	4200	4200	4200	4200	4200	4200	4200	4200	4200
861199	392401	4118	4118	4118	4118	4118	4118	4118	4118	4118
861229	392371	4115,33	4115,33	4115,33	4115,33	4115,33	4115,33	4115,33	4115,33	4115,33
861259	392341	4130,67	4130,67	4130,67	4130,67	4130,67	4130,67	4130,67	4130,67	4130,67
861289	392311	4084,67	4084,67	4084,67	4084,67	4084,67	4084,67	4084,67	4084,67	4084,67
861319	392281	4136,67	4136,67	4136,67	4136,67	4136,67	4136,67	4136,67	4136,67	4136,67
861349	392251	4160,67	4160,67	4160,67	4160,67	4160,67	4160,67	4160,67	4160,67	4160,67
861379	392221	4163,33	4163,33	4163,33	4163,33	4163,33	4163,33	4163,33	4163,33	4163,33
861409	392191	4160,67	4160,67	4160,67	4160,67	4160,67	4160,67	4160,67	4160,67	4160,67
861439	392161	4148	4148	4148	4148	4148	4148	4148	4148	4148
861469	392131	4222,67	4222,67	4222,67	4222,67	4222,67	4222,67	4222,67	4222,67	4222,67
861499	392101	4198	4198	4198	4198	4198	4198	4198	4198	4198
861529	392071	4174	4174	4174	4174	4174	4174	4174	4174	4174
861559	392041	4284	4284	4284	4284	4284	4284	4284	4284	4284
861589	392011	4250,67	4250,67	4250,67	4250,67	4250,67	4250,67	4250,67	4250,67	4250,67
861619	391981	4235,33	4235,33	4235,33	4235,33	4235,33	4235,33	4235,33	4235,33	4235,33
861649	391951	4182,67	4182,67	4182,67	4182,67	4182,67	4182,67	4182,67	4182,67	4182,67
861679	391921	4144,67	4144,67	4144,67	4144,67	4144,67	4144,67	4144,67	4144,67	4144,67
861709	391891	4204,67	4204,67	4204,67	4204,67	4204,67	4204,67	4204,67	4204,67	4204,67
861739	391861	4230	4230	4230	4230	4230	4230	4230	4230	4230
861769	391831	4208,67	4208,67	4208,67	4208,67	4208,67	4208,67	4208,67	4208,67	4208,67
861799	391801	4218	4218	4218	4218	4218	4218	4218	4218	4218
861829	391771	4261,33	4261,33	4261,33	4261,33	4261,33	4261,33	4261,33	4261,33	4261,33
861859	391741	4288	4288	4288	4288	4288	4288	4288	4288	4288

This journal is © The Royal Society of Chemistry 20xx

Please do not adjust margins

Please do not adjust margins

861889	391711	4254	4254	4254	4254	4254	4254	4254	4254	4254
861919	391681	4252	4252	4252	4252	4252	4252	4252	4252	4252
861949	391651	4278,67	4278,67	4278,67	4278,67	4278,67	4278,67	4278,67	4278,67	4278,67
861979	391621	4347,33	4347,33	4347,33	4347,33	4347,33	4347,33	4347,33	4347,33	4347,33
862009	391591	4300,67	4300,67	4300,67	4300,67	4300,67	4300,67	4300,67	4300,67	4300,67
862039	391561	4250	4250	4250	4250	4250	4250	4250	4250	4250
862069	391531	4160	4160	4160	4160	4160	4160	4160	4160	4160
862099	391501	4243,33	4243,33	4243,33	4243,33	4243,33	4243,33	4243,33	4243,33	4243,33
862129	391471	4284	4284	4284	4284	4284	4284	4284	4284	4284
862159	391441	4355,33	4355,33	4355,33	4355,33	4355,33	4355,33	4355,33	4355,33	4355,33
862189	391411	4224,67	4224,67	4224,67	4224,67	4224,67	4224,67	4224,67	4224,67	4224,67
862219	391381	4317,33	4317,33	4317,33	4317,33	4317,33	4317,33	4317,33	4317,33	4317,33
862249	391351	4226,67	4226,67	4226,67	4226,67	4226,67	4226,67	4226,67	4226,67	4226,67
862279	391321	4254,67	4254,67	4254,67	4254,67	4254,67	4254,67	4254,67	4254,67	4254,67
862309	391291	4251,33	4251,33	4251,33	4251,33	4251,33	4251,33	4251,33	4251,33	4251,33
862339	391261	4223,33	4223,33	4223,33	4223,33	4223,33	4223,33	4223,33	4223,33	4223,33
862369	391231	4292	4292	4292	4292	4292	4292	4292	4292	4292
862399	391201	4242	4242	4242	4242	4242	4242	4242	4242	4242
862429	391171	4264,67	4264,67	4264,67	4264,67	4264,67	4264,67	4264,67	4264,67	4264,67
862459	391141	4292	4292	4292	4292	4292	4292	4292	4292	4292
862489	391111	4342,67	4342,67	4342,67	4342,67	4342,67	4342,67	4342,67	4342,67	4342,67
862519	391081	4232,67	4232,67	4232,67	4232,67	4232,67	4232,67	4232,67	4232,67	4232,67
862549	391051	4179,33	4179,33	4179,33	4179,33	4179,33	4179,33	4179,33	4179,33	4179,33
862579	391021	4306,67	4306,67	4306,67	4306,67	4306,67	4306,67	4306,67	4306,67	4306,67
862609	390991	4444,67	4444,67	4444,67	4444,67	4444,67	4444,67	4444,67	4444,67	4444,67
862639	390961	4182	4182	4182	4182	4182	4182	4182	4182	4182
862669	390931	4255,33	4255,33	4255,33	4255,33	4255,33	4255,33	4255,33	4255,33	4255,33
862699	390901	4238,67	4238,67	4238,67	4238,67	4238,67	4238,67	4238,67	4238,67	4238,67
862729	390871	4308	4308	4308	4308	4308	4308	4308	4308	4308
862759	390841	4336,67	4336,67	4336,67	4336,67	4336,67	4336,67	4336,67	4336,67	4336,67
862789	390811	4210	4210	4210	4210	4210	4210	4210	4210	4210
862819	390781	4182,67	4182,67	4182,67	4182,67	4182,67	4182,67	4182,67	4182,67	4182,67
862849	390751	4208,67	4208,67	4208,67	4208,67	4208,67	4208,67	4208,67	4208,67	4208,67
862879	390721	4224	4224	4224	4224	4224	4224	4224	4224	4224
862909	390691	4270	4270	4270	4270	4270	4270	4270	4270	4270
862939	390661	4225,33	4225,33	4225,33	4225,33	4225,33	4225,33	4225,33	4225,33	4225,33
862969	390631	4336,67	4336,67	4336,67	4336,67	4336,67	4336,67	4336,67	4336,67	4336,67
862999	390601	4294,67	4294,67	4294,67	4294,67	4294,67	4294,67	4294,67	4294,67	4294,67
863029	390571	4232	4232	4232	4232	4232	4232	4232	4232	4232
863059	390541	4246,67	4246,67	4246,67	4246,67	4246,67	4246,67	4246,67	4246,67	4246,67
863089	390511	4209,33	4209,33	4209,33	4209,33	4209,33	4209,33	4209,33	4209,33	4209,33
863119	390481	4235,33	4235,33	4235,33	4235,33	4235,33	4235,33	4235,33	4235,33	4235,33
863149	390451	4310,67	4310,67	4310,67	4310,67	4310,67	4310,67	4310,67	4310,67	4310,67
863179	390421	4280,67	4280,67	4280,67	4280,67	4280,67	4280,67	4280,67	4280,67	4280,67
863209	390391	4158	4158	4158	4158	4158	4158	4158	4158	4158
863239	390361	4252,67	4252,67	4252,67	4252,67	4252,67	4252,67	4252,67	4252,67	4252,67
863269	390331	4196,67	4196,67	4196,67	4196,67	4196,67	4196,67	4196,67	4196,67	4196,67

This journal is © The Royal Society of Chemistry 20xx

Please do not adjust margins

Please do not adjust margins

863299	390301	4331,33	4331,33	4331,33	4331,33	4331,33	4331,33	4331,33	4331,33	4331,33
863329	390271	4200,67	4200,67	4200,67	4200,67	4200,67	4200,67	4200,67	4200,67	4200,67
863359	390241	4189,33	4189,33	4189,33	4189,33	4189,33	4189,33	4189,33	4189,33	4189,33
863389	390211	4270	4270	4270	4270	4270	4270	4270	4270	4270
863419	390181	4222,67	4222,67	4222,67	4222,67	4222,67	4222,67	4222,67	4222,67	4222,67
863449	390151	4169,33	4169,33	4169,33	4169,33	4169,33	4169,33	4169,33	4169,33	4169,33
863479	390121	4122,67	4122,67	4122,67	4122,67	4122,67	4122,67	4122,67	4122,67	4122,67
863509	390091	4271,33	4271,33	4271,33	4271,33	4271,33	4271,33	4271,33	4271,33	4271,33
863539	390061	4196	4196	4196	4196	4196	4196	4196	4196	4196
863569	390031	4270	4270	4270	4270	4270	4270	4270	4270	4270
863599	390001	4212	4212	4212	4212	4212	4212	4212	4212	4212
863629	389971	4181,33	4181,33	4181,33	4181,33	4181,33	4181,33	4181,33	4181,33	4181,33
863659	389941	4102,67	4102,67	4102,67	4102,67	4102,67	4102,67	4102,67	4102,67	4102,67
863689	389911	4115,33	4115,33	4115,33	4115,33	4115,33	4115,33	4115,33	4115,33	4115,33
863719	389881	4150,67	4150,67	4150,67	4150,67	4150,67	4150,67	4150,67	4150,67	4150,67
863749	389851	4223,33	4223,33	4223,33	4223,33	4223,33	4223,33	4223,33	4223,33	4223,33
863779	389821	4215,33	4215,33	4215,33	4215,33	4215,33	4215,33	4215,33	4215,33	4215,33
863809	389791	4120,67	4120,67	4120,67	4120,67	4120,67	4120,67	4120,67	4120,67	4120,67
863839	389761	4066	4066	4066	4066	4066	4066	4066	4066	4066
863869	389731	4154,67	4154,67	4154,67	4154,67	4154,67	4154,67	4154,67	4154,67	4154,67
863899	389701	4276	4276	4276	4276	4276	4276	4276	4276	4276
863929	389671	4186	4186	4186	4186	4186	4186	4186	4186	4186
863959	389641	4152	4152	4152	4152	4152	4152	4152	4152	4152
863989	389611	4141,33	4141,33	4141,33	4141,33	4141,33	4141,33	4141,33	4141,33	4141,33
864019	389581	4193,33	4193,33	4193,33	4193,33	4193,33	4193,33	4193,33	4193,33	4193,33
864049	389551	4150	4150	4150	4150	4150	4150	4150	4150	4150
864079	389521	4178	4178	4178	4178	4178	4178	4178	4178	4178
864109	389491	4210	4210	4210	4210	4210	4210	4210	4210	4210
864139	389461	4226,67	4226,67	4226,67	4226,67	4226,67	4226,67	4226,67	4226,67	4226,67
864169	389431	4200,67	4200,67	4200,67	4200,67	4200,67	4200,67	4200,67	4200,67	4200,67
864199	389401	4123,33	4123,33	4123,33	4123,33	4123,33	4123,33	4123,33	4123,33	4123,33
864229	389371	4168	4168	4168	4168	4168	4168	4168	4168	4168
864259	389341	4146,67	4146,67	4146,67	4146,67	4146,67	4146,67	4146,67	4146,67	4146,67
864289	389311	4174,67	4174,67	4174,67	4174,67	4174,67	4174,67	4174,67	4174,67	4174,67
864319	389281	4183,33	4183,33	4183,33	4183,33	4183,33	4183,33	4183,33	4183,33	4183,33
864349	389251	4224	4224	4224	4224	4224	4224	4224	4224	4224
864379	389221	4178,67	4178,67	4178,67	4178,67	4178,67	4178,67	4178,67	4178,67	4178,67
864409	389191	4066,67	4066,67	4066,67	4066,67	4066,67	4066,67	4066,67	4066,67	4066,67
864439	389161	4142,67	4142,67	4142,67	4142,67	4142,67	4142,67	4142,67	4142,67	4142,67
864469	389131	4092,67	4092,67	4092,67	4092,67	4092,67	4092,67	4092,67	4092,67	4092,67
864499	389101	4088	4088	4088	4088	4088	4088	4088	4088	4088
864529	389071	4174,67	4174,67	4174,67	4174,67	4174,67	4174,67	4174,67	4174,67	4174,67
864559	389041	4116,67	4116,67	4116,67	4116,67	4116,67	4116,67	4116,67	4116,67	4116,67
864589	389011	4222	4222	4222	4222	4222	4222	4222	4222	4222
864619	388981	4104	4104	4104	4104	4104	4104	4104	4104	4104
864649	388951	4066	4066	4066	4066	4066	4066	4066	4066	4066
864679	388921	4100,67	4100,67	4100,67	4100,67	4100,67	4100,67	4100,67	4100,67	4100,67

This journal is © The Royal Society of Chemistry 20xx

Please do not adjust margins

Please do not adjust margins

864709	388891	4021,33	4021,33	4021,33	4021,33	4021,33	4021,33	4021,33	4021,33	4021,33
864739	388861	4190	4190	4190	4190	4190	4190	4190	4190	4190
864769	388831	4199,33	4199,33	4199,33	4199,33	4199,33	4199,33	4199,33	4199,33	4199,33
864799	388801	4172	4172	4172	4172	4172	4172	4172	4172	4172
864829	388771	4051,33	4051,33	4051,33	4051,33	4051,33	4051,33	4051,33	4051,33	4051,33
864859	388741	4118	4118	4118	4118	4118	4118	4118	4118	4118
864889	388711	4136	4136	4136	4136	4136	4136	4136	4136	4136
864919	388681	4076	4076	4076	4076	4076	4076	4076	4076	4076
864949	388651	4175,33	4175,33	4175,33	4175,33	4175,33	4175,33	4175,33	4175,33	4175,33
864979	388621	4024,67	4024,67	4024,67	4024,67	4024,67	4024,67	4024,67	4024,67	4024,67
865009	388591	4111,33	4111,33	4111,33	4111,33	4111,33	4111,33	4111,33	4111,33	4111,33
865039	388561	4161,33	4161,33	4161,33	4161,33	4161,33	4161,33	4161,33	4161,33	4161,33
865069	388531	4084,67	4084,67	4084,67	4084,67	4084,67	4084,67	4084,67	4084,67	4084,67
865099	388501	4070,67	4070,67	4070,67	4070,67	4070,67	4070,67	4070,67	4070,67	4070,67
865129	388471	4179,33	4179,33	4179,33	4179,33	4179,33	4179,33	4179,33	4179,33	4179,33
865159	388441	4102	4102	4102	4102	4102	4102	4102	4102	4102
865189	388411	4176,67	4176,67	4176,67	4176,67	4176,67	4176,67	4176,67	4176,67	4176,67
865219	388381	4119,33	4119,33	4119,33	4119,33	4119,33	4119,33	4119,33	4119,33	4119,33
865249	388351	4140	4140	4140	4140	4140	4140	4140	4140	4140
865279	388321	4112,67	4112,67	4112,67	4112,67	4112,67	4112,67	4112,67	4112,67	4112,67
865309	388291	4006,67	4006,67	4006,67	4006,67	4006,67	4006,67	4006,67	4006,67	4006,67
865339	388261	4066,67	4066,67	4066,67	4066,67	4066,67	4066,67	4066,67	4066,67	4066,67
865369	388231	4112,67	4112,67	4112,67	4112,67	4112,67	4112,67	4112,67	4112,67	4112,67
865399	388201	4033,33	4033,33	4033,33	4033,33	4033,33	4033,33	4033,33	4033,33	4033,33
865429	388171	4108,67	4108,67	4108,67	4108,67	4108,67	4108,67	4108,67	4108,67	4108,67
865459	388141	4119,33	4119,33	4119,33	4119,33	4119,33	4119,33	4119,33	4119,33	4119,33
865489	388111	4112	4112	4112	4112	4112	4112	4112	4112	4112
865519	388081	4072	4072	4072	4072	4072	4072	4072	4072	4072
865549	388051	4030	4030	4030	4030	4030	4030	4030	4030	4030
865579	388021	4016	4016	4016	4016	4016	4016	4016	4016	4016
865609	387991	4072,67	4072,67	4072,67	4072,67	4072,67	4072,67	4072,67	4072,67	4072,67
865639	387961	4038,67	4038,67	4038,67	4038,67	4038,67	4038,67	4038,67	4038,67	4038,67
865669	387931	4118,67	4118,67	4118,67	4118,67	4118,67	4118,67	4118,67	4118,67	4118,67
865699	387901	4186	4186	4186	4186	4186	4186	4186	4186	4186
865729	387871	4145,33	4145,33	4145,33	4145,33	4145,33	4145,33	4145,33	4145,33	4145,33
865759	387841	3986	3986	3986	3986	3986	3986	3986	3986	3986
865789	387811	4029,33	4029,33	4029,33	4029,33	4029,33	4029,33	4029,33	4029,33	4029,33
865819	387781	4034,67	4034,67	4034,67	4034,67	4034,67	4034,67	4034,67	4034,67	4034,67
865849	387751	3998	3998	3998	3998	3998	3998	3998	3998	3998
865879	387721	4005,33	4005,33	4005,33	4005,33	4005,33	4005,33	4005,33	4005,33	4005,33

Table S7 Raw XPS measurements corresponding to Fig. 2.e.

KE_MgKa	BE_MgKa	CPS_MgKa	N 1s_1_MgKa	N 1s_2_MgKa	N 1s_3_MgKa	N 1s_4_MgKa	Background_MgKa	Envelope_MgKa
844896	408704	3743,75	3743,75	3743,75	3743,75	3743,75	3743,75	3743,75
844926	408674	3788,75	3788,75	3788,75	3788,75	3788,75	3788,75	3788,75
844956	408644	3693,75	3693,75	3693,75	3693,75	3693,75	3693,75	3693,75

Please do not adjust margins

Please do not adjust margins

844986	408614	3746,25	3746,25	3746,25	3746,25	3746,25	3746,25	3746,25
845016	408584	3847,5	3847,5	3847,5	3847,5	3847,5	3847,5	3847,5
845046	408554	3572,5	3572,5	3572,5	3572,5	3572,5	3572,5	3572,5
845076	408524	3655	3655	3655	3655	3655	3655	3655
845106	408494	3752,5	3752,5	3752,5	3752,5	3752,5	3752,5	3752,5
845136	408464	3808,75	3808,75	3808,75	3808,75	3808,75	3808,75	3808,75
845166	408434	3733,75	3733,75	3733,75	3733,75	3733,75	3733,75	3733,75
845196	408404	3800	3800	3800	3800	3800	3800	3800
845226	408374	3823,75	3823,75	3823,75	3823,75	3823,75	3823,75	3823,75
845256	408344	3737,5	3737,5	3737,5	3737,5	3737,5	3737,5	3737,5
845286	408314	3810	3810	3810	3810	3810	3810	3810
845316	408284	3627,5	3627,5	3627,5	3627,5	3627,5	3627,5	3627,5
845346	408254	3752,5	3752,5	3752,5	3752,5	3752,5	3752,5	3752,5
845376	408224	3671,25	3671,25	3671,25	3671,25	3671,25	3671,25	3671,25
845406	408194	3738,75	3738,75	3738,75	3738,75	3738,75	3738,75	3738,75
845436	408164	3728,75	3728,75	3728,75	3728,75	3728,75	3728,75	3728,75
845466	408134	3578,75	3578,75	3578,75	3578,75	3578,75	3578,75	3578,75
845496	408104	3758,75	3758,75	3758,75	3758,75	3758,75	3758,75	3758,75
845526	408074	3751,25	3751,25	3751,25	3751,25	3751,25	3751,25	3751,25
845556	408044	3892,5	3892,5	3892,5	3892,5	3892,5	3892,5	3892,5
845586	408014	3730	3730	3730	3730	3730	3730	3730
845616	407984	3698,75	3698,75	3698,75	3698,75	3698,75	3698,75	3698,75
845646	407954	3673,75	3673,75	3673,75	3673,75	3673,75	3673,75	3673,75
845676	407924	3755	3755	3755	3755	3755	3755	3755
845706	407894	3765	3765	3765	3765	3765	3765	3765
845736	407864	3626,25	3626,25	3626,25	3626,25	3626,25	3626,25	3626,25
845766	407834	3742,5	3742,5	3742,5	3742,5	3742,5	3742,5	3742,5
845796	407804	3656,25	3656,25	3656,25	3656,25	3656,25	3656,25	3656,25
845826	407774	3736,25	3736,25	3736,25	3736,25	3736,25	3736,25	3736,25
845856	407744	3670	3670	3670	3670	3670	3670	3670
845886	407714	3705	3705	3705	3705	3705	3705	3705
845916	407684	3726,25	3726,25	3726,25	3726,25	3726,25	3726,25	3726,25
845946	407654	3756,25	3756,25	3756,25	3756,25	3756,25	3756,25	3756,25
845976	407624	3725	3725	3725	3725	3725	3725	3725
846006	407594	3736,25	3736,25	3736,25	3736,25	3736,25	3736,25	3736,25
846036	407564	3656,25	3656,25	3656,25	3656,25	3656,25	3656,25	3656,25
846066	407534	3631,25	3631,25	3631,25	3631,25	3631,25	3631,25	3631,25
846096	407504	3890	3890	3890	3890	3890	3890	3890
846126	407474	3735	3735	3735	3735	3735	3735	3735
846156	407444	3625	3625	3625	3625	3625	3625	3625
846186	407414	3592,5	3592,5	3592,5	3592,5	3592,5	3592,5	3592,5
846216	407384	3723,75	3723,75	3723,75	3723,75	3723,75	3723,75	3723,75
846246	407354	3705	3705	3705	3705	3705	3705	3705
846276	407324	3621,25	3621,25	3621,25	3621,25	3621,25	3621,25	3621,25
846306	407294	3845	3845	3845	3845	3845	3845	3845
846336	407264	3790	3790	3790	3790	3790	3790	3790
846366	407234	3785	3785	3785	3785	3785	3785	3785

This journal is © The Royal Society of Chemistry 20xx

Please do not adjust margins

Please do not adjust margins

846396	407204	3715	3715	3715	3715	3715	3715	3715
846426	407174	3716,25	3716,25	3716,25	3716,25	3716,25	3716,25	3716,25
846456	407144	3728,75	3728,75	3728,75	3728,75	3728,75	3728,75	3728,75
846486	407114	3730	3730	3730	3730	3730	3730	3730
846516	407084	3763,75	3763,75	3763,75	3763,75	3763,75	3763,75	3763,75
846546	407054	3608,75	3608,75	3608,75	3608,75	3608,75	3608,75	3608,75
846576	407024	3556,25	3556,25	3556,25	3556,25	3556,25	3556,25	3556,25
846606	406994	3677,5	3677,5	3677,5	3677,5	3677,5	3677,5	3677,5
846636	406964	3686,25	3686,25	3686,25	3686,25	3686,25	3686,25	3686,25
846666	406934	3828,75	3828,75	3828,75	3828,75	3828,75	3828,75	3828,75
846696	406904	3676,25	3676,25	3676,25	3676,25	3676,25	3676,25	3676,25
846726	406874	3733,75	3733,75	3733,75	3733,75	3733,75	3733,75	3733,75
846756	406844	3758,75	3758,75	3758,75	3758,75	3758,75	3758,75	3758,75
846786	406814	3603,75	3603,75	3603,75	3603,75	3603,75	3603,75	3603,75
846816	406784	3702,5	3702,5	3702,5	3702,5	3702,5	3702,5	3702,5
846846	406754	3666,25	3666,25	3666,25	3666,25	3666,25	3666,25	3666,25
846876	406724	3631,25	3631,25	3631,25	3631,25	3631,25	3631,25	3631,25
846906	406694	3695	3695	3695	3695	3695	3695	3695
846936	406664	3675	3675	3675	3675	3675	3675	3675
846966	406634	3656,25	3656,25	3656,25	3656,25	3656,25	3656,25	3656,25
846996	406604	3766,25	3766,25	3766,25	3766,25	3766,25	3766,25	3766,25
847026	406574	3875	3875	3875	3875	3875	3875	3875
847056	406544	3773,75	3773,75	3773,75	3773,75	3773,75	3773,75	3773,75
847086	406514	3688,75	3688,75	3688,75	3688,75	3688,75	3688,75	3688,75
847116	406484	3875	3875	3875	3875	3875	3875	3875
847146	406454	3668,75	3668,75	3668,75	3668,75	3668,75	3668,75	3668,75
847176	406424	3537,5	3537,5	3537,5	3537,5	3537,5	3537,5	3537,5
847206	406394	3635	3635	3635	3635	3635	3635	3635
847236	406364	3660	3660	3660	3660	3660	3660	3660
847266	406334	3718,75	3718,75	3718,75	3718,75	3718,75	3718,75	3718,75
847296	406304	3610	3610	3610	3610	3610	3610	3610
847326	406274	3737,5	3737,5	3737,5	3737,5	3737,5	3737,5	3737,5
847356	406244	3651,25	3651,25	3651,25	3651,25	3651,25	3651,25	3651,25
847386	406214	3808,75	3808,75	3808,75	3808,75	3808,75	3808,75	3808,75
847416	406184	3742,5	3742,5	3742,5	3742,5	3742,5	3742,5	3742,5
847446	406154	3613,75	3681,08	3681,08	3681,08	3681,08	3681,08	3681,08
847476	406124	3636,25	3681,04	3681,04	3681,04	3681,04	3681,04	3681,04
847506	406094	3640	3681	3681	3681	3681	3681	3681
847536	406064	3631,25	3680,95	3680,95	3680,96	3680,95	3680,95	3680,96
847566	406034	3753,75	3680,89	3680,89	3680,89	3680,89	3680,89	3680,89
847596	406004	3667,5	3680,88	3680,88	3680,88	3680,88	3680,88	3680,88
847626	405974	3731,25	3680,83	3680,83	3680,83	3680,83	3680,83	3680,83
847656	405944	3738,75	3680,78	3680,78	3680,78	3680,78	3680,78	3680,78
847686	405914	3671,25	3680,77	3680,77	3680,77	3680,77	3680,77	3680,78
847716	405884	3745	3680,71	3680,71	3680,72	3680,71	3680,71	3680,72
847746	405854	3588,75	3680,63	3680,63	3680,64	3680,63	3680,63	3680,64
847776	405824	3622,5	3680,58	3680,58	3680,59	3680,58	3680,58	3680,59

Please do not adjust margins

Please do not adjust margins

847806	405794	3710	3680,55	3680,55	3680,56	3680,55	3680,55	3680,56
847836	405764	3748,75	3680,49	3680,49	3680,5	3680,49	3680,49	3680,5
847866	405734	3680	3680,49	3680,49	3680,5	3680,49	3680,49	3680,5
847896	405704	3661,25	3680,47	3680,47	3680,49	3680,47	3680,47	3680,49
847926	405674	3632,5	3680,43	3680,43	3680,45	3680,43	3680,43	3680,45
847956	405644	3732,5	3680,38	3680,38	3680,4	3680,38	3680,38	3680,4
847986	405614	3661,25	3680,36	3680,36	3680,39	3680,36	3680,36	3680,39
848016	405584	3597,5	3680,29	3680,29	3680,32	3680,29	3680,29	3680,32
848046	405554	3618,75	3680,23	3680,23	3680,27	3680,23	3680,23	3680,27
848076	405524	3632,5	3680,19	3680,19	3680,23	3680,19	3680,19	3680,23
848106	405494	3833,75	3680,05	3680,05	3680,1	3680,05	3680,05	3680,1
848136	405464	3787,5	3679,95	3679,95	3680,01	3679,95	3679,95	3680,01
848166	405434	3670	3679,94	3679,94	3680,01	3679,94	3679,94	3680,01
848196	405404	3660	3679,93	3679,93	3680	3679,93	3679,93	3680
848226	405374	3713,75	3679,89	3679,89	3679,98	3679,9	3679,89	3679,98
848256	405344	3697,5	3679,88	3679,88	3679,98	3679,88	3679,88	3679,98
848286	405314	3700	3679,86	3679,86	3679,97	3679,86	3679,86	3679,98
848316	405284	3665	3679,85	3679,85	3679,98	3679,85	3679,85	3679,98
848346	405254	3656,25	3679,83	3679,83	3679,97	3679,83	3679,83	3679,98
848376	405224	3708,75	3679,8	3679,8	3679,97	3679,8	3679,8	3679,97
848406	405194	3746,25	3679,74	3679,74	3679,93	3679,74	3679,74	3679,94
848436	405164	3801,25	3679,63	3679,63	3679,85	3679,63	3679,63	3679,86
848466	405134	3746,25	3679,57	3679,57	3679,82	3679,58	3679,57	3679,83
848496	405104	3635	3679,53	3679,53	3679,82	3679,54	3679,53	3679,82
848526	405074	3693,75	3679,52	3679,52	3679,84	3679,52	3679,52	3679,85
848556	405044	3692,5	3679,51	3679,51	3679,88	3679,51	3679,51	3679,89
848586	405014	3780	3679,41	3679,41	3679,84	3679,42	3679,41	3679,85
848616	404984	3610	3679,35	3679,35	3679,83	3679,36	3679,35	3679,84
848646	404954	3546,25	3679,23	3679,23	3679,77	3679,25	3679,23	3679,79
848676	404924	3643,75	3679,2	3679,2	3679,81	3679,22	3679,2	3679,83
848706	404894	3737,5	3679,15	3679,15	3679,84	3679,17	3679,15	3679,86
848736	404864	3713,75	3679,12	3679,12	3679,9	3679,14	3679,12	3679,92
848766	404834	3821,25	3678,99	3678,99	3679,87	3679,01	3678,99	3679,9
848796	404804	3607,5	3678,92	3678,92	3679,92	3678,95	3678,92	3679,95
848826	404774	3685	3678,92	3678,92	3680,04	3678,95	3678,92	3680,08
848856	404744	3792,5	3678,81	3678,81	3680,09	3678,86	3678,81	3680,13
848886	404714	3792,5	3678,71	3678,71	3680,14	3678,76	3678,71	3680,19
848916	404684	3787,5	3678,61	3678,61	3680,22	3678,67	3678,61	3680,27
848946	404654	3652,5	3678,59	3678,59	3680,39	3678,65	3678,59	3680,45
848976	404624	3841,25	3678,44	3678,44	3680,47	3678,52	3678,44	3680,54
849006	404594	3846,25	3678,29	3678,29	3680,56	3678,38	3678,29	3680,64
849036	404564	3655	3678,27	3678,27	3680,81	3678,37	3678,27	3680,91
849066	404534	3758,75	3678,2	3678,2	3681,04	3678,31	3678,2	3681,15
849096	404504	3802,5	3678,09	3678,09	3681,26	3678,21	3678,09	3681,39
849126	404474	3628,75	3678,04	3678,04	3681,59	3678,19	3678,04	3681,74
849156	404444	3673,75	3678,04	3678,04	3682	3678,21	3678,04	3682,17
849186	404414	3711,25	3678,01	3678,01	3682,42	3678,2	3678,01	3682,61

This journal is © The Royal Society of Chemistry 20xx

Please do not adjust margins

849216	404384	3745	3677,95	3677,95	3682,85	3678,17	3677,95	3683,08
849246	404354	3571,25	3677,85	3677,85	3683,31	3678,1	3677,85	3683,57
849276	404324	3761,25	3677,77	3677,77	3683,85	3678,07	3677,77	3684,15
849306	404294	3872,5	3677,6	3677,6	3684,35	3677,93	3677,6	3684,68
849336	404264	3656,25	3677,58	3677,58	3685,07	3677,96	3677,58	3685,45
849366	404234	3611,25	3677,52	3677,52	3685,81	3677,95	3677,52	3686,25
849396	404204	3690	3677,51	3677,51	3686,7	3678	3677,51	3687,2
849426	404174	3833,75	3677,37	3677,37	3687,55	3677,93	3677,37	3688,11
849456	404144	3577,5	3677,28	3677,28	3688,53	3677,92	3677,28	3689,17
849486	404114	3728,75	3677,23	3677,23	3689,66	3677,96	3677,23	3690,38
849516	404084	3733,75	3677,18	3677,18	3690,89	3678	3677,18	3691,71
849546	404054	3743,75	3677,12	3677,12	3692,25	3678,05	3677,12	3693,18
849576	404024	3687,5	3677,11	3677,11	3693,79	3678,16	3677,11	3694,84
849606	403994	3672,5	3677,11	3677,11	3695,46	3678,3	3677,11	3696,65
849636	403964	3615	3677,05	3677,05	3697,22	3678,4	3677,05	3698,56
849666	403934	3666,25	3677,04	3677,04	3699,2	3678,56	3677,04	3700,72
849696	403904	3627,5	3677	3677	3701,34	3678,71	3677	3703,05
849726	403874	3746,25	3676,93	3676,93	3703,64	3678,86	3676,93	3705,57
849756	403844	3697,5	3676,91	3676,92	3706,17	3679,09	3676,91	3708,35
849786	403814	3702,5	3676,89	3676,89	3708,9	3679,34	3676,89	3711,35
849816	403784	3892,5	3676,7	3676,7	3711,72	3679,44	3676,7	3714,47
849846	403754	3766,25	3676,62	3676,62	3714,92	3679,7	3676,61	3718
849876	403724	3865	3676,44	3676,45	3718,27	3679,9	3676,44	3721,73
849906	403694	3762,5	3676,37	3676,37	3721,99	3680,24	3676,37	3725,86
849936	403664	3750	3676,3	3676,3	3725,98	3680,64	3676,3	3730,32
849966	403634	3657,5	3676,28	3676,29	3730,43	3681,13	3676,28	3735,28
849996	403604	3787,5	3676,18	3676,19	3735,12	3681,59	3676,18	3740,54
850026	403574	4013,75	3675,88	3675,89	3739,95	3681,91	3675,88	3745,99
850056	403544	3756,25	3675,81	3675,81	3745,36	3682,53	3675,81	3752,1
850086	403514	3828,75	3675,67	3675,68	3751,1	3683,17	3675,67	3758,61
850116	403484	3865	3675,5	3675,51	3757,33	3683,84	3675,5	3765,7
850146	403454	3807,5	3675,38	3675,39	3764,05	3684,65	3675,38	3773,34
850176	403424	3777,5	3675,29	3675,3	3771,23	3685,58	3675,28	3781,54
850206	403394	3826,25	3675,15	3675,17	3778,83	3686,57	3675,15	3790,27
850236	403364	3848,75	3674,99	3675,02	3786,96	3687,66	3674,99	3799,66
850266	403334	3915	3674,78	3674,8	3795,66	3688,81	3674,77	3809,73
850296	403304	3975	3674,5	3674,54	3804,85	3690,03	3674,5	3820,42
850326	403274	3770	3674,42	3674,46	3814,79	3691,57	3674,42	3831,99
850356	403244	3912,5	3674,2	3674,25	3825,17	3693,14	3674,2	3844,17
850386	403214	3915	3673,99	3674,04	3836,28	3694,9	3673,98	3857,26
850416	403184	3973,75	3673,72	3673,78	3848,06	3696,78	3673,71	3871,21
850446	403154	3923,75	3673,49	3673,56	3860,54	3698,9	3673,49	3886,03
850476	403124	3961,25	3673,23	3673,32	3873,65	3701,17	3673,23	3901,69
850506	403094	3936,25	3673	3673,1	3887,47	3703,69	3672,99	3918,28
850536	403064	3902,5	3672,79	3672,91	3902,2	3706,53	3672,78	3936,08
850566	403034	4077,5	3672,42	3672,56	3917,55	3709,46	3672,42	3954,76
850596	403004	4012,5	3672,12	3672,28	3933,69	3712,72	3672,11	3974,49

This journal is © The Royal Society of Chemistry 20xx

Please do not adjust margins

850626	402974	4041,25	3671,79	3671,97	3950,53	3716,24	3671,77	3995,21
850656	402944	4017,5	3671,48	3671,69	3968,12	3720,08	3671,46	4016,99
850686	402914	4035	3671,15	3671,4	3986,69	3724,33	3671,13	4040,17
850716	402884	4143,75	3670,72	3671,02	4005,88	3728,84	3670,71	4064,35
850746	402854	4097,5	3670,34	3670,68	4025,83	3733,76	3670,32	4089,66
850776	402824	4217,5	3669,85	3670,24	4046,37	3738,97	3669,83	4115,95
850806	402794	4217,5	3669,36	3669,81	4067,62	3744,59	3669,33	4143,39
850836	402764	4085	3668,99	3669,51	4089,82	3750,9	3668,96	4172,35
850866	402734	4258,75	3668,46	3669,06	4112,44	3757,55	3668,42	4202,25
850896	402704	4173,75	3668,01	3668,7	4135,68	3764,78	3667,97	4233,28
850926	402674	4228,75	3667,51	3668,31	4159,36	3772,49	3667,46	4265,29
850956	402644	4352,5	3666,9	3667,81	4183,42	3780,64	3666,84	4298,25
850986	402614	4206,25	3666,42	3667,47	4208,01	3789,64	3666,35	4332,48
851016	402584	4476,25	3665,7	3666,9	4232,58	3799,08	3665,62	4367,4
851046	402554	4393,75	3665,05	3666,43	4257,36	3809,24	3664,96	4403,2
851076	402524	4443,75	3664,36	3665,94	4282,16	3820,04	3664,26	4439,72
851106	402494	4413,75	3663,7	3665,5	4306,89	3831,57	3663,58	4476,91
851136	402464	4593,75	3662,87	3664,92	4331,18	3843,8	3662,74	4514,55
851166	402434	4521,25	3662,11	3664,45	4355,14	3856,98	3661,96	4552,79
851196	402404	4498,75	3661,38	3664,03	4378,62	3870,98	3661,21	4591,39
851226	402374	4618,75	3660,54	3663,56	4401,36	3885,7	3660,34	4630,13
851256	402344	4648,75	3659,67	3663,1	4423,02	3901,24	3659,45	4668,68
851286	402314	4746,25	3658,72	3662,61	4443,5	3917,67	3658,47	4707,09
851316	402284	4667,5	3657,84	3662,25	4462,87	3935,24	3657,55	4745,53
851346	402254	4630	3657	3661,98	4480,98	3953,75	3656,68	4783,69
851376	402224	4885	3655,94	3661,56	4497,41	3972,97	3655,57	4821,18
851406	402194	4911,25	3654,85	3661,19	4511,63	3993,09	3654,43	4857,48
851436	402164	4912,5	3653,77	3660,91	4524,06	4014,23	3653,29	4893,09
851466	402134	4867,5	3652,74	3660,77	4534,64	4036,57	3652,2	4928,12
851496	402104	5000	3651,59	3660,63	4543,11	4059,72	3650,98	4962,11
851526	402074	4991,25	3650,46	3660,62	4549,36	4083,79	3649,77	4994,92
851556	402044	5063,75	3649,27	3660,67	4552,78	4108,68	3648,49	5025,94
851586	402014	5130	3648,03	3660,8	4553,93	4134,41	3647,15	5055,73
851616	401984	5138,75	3646,79	3661,09	4552,85	4161,18	3645,8	5084,5
851646	401954	5165	3645,54	3661,52	4549,5	4188,67	3644,43	5111,96
851676	401924	5108,75	3644,35	3662,2	4543,65	4216,9	3643,1	5137,79
851706	401894	5058,75	3643,23	3663,12	4535,25	4245,79	3641,83	5161,91
851736	401864	5207,5	3641,98	3664,13	4524,62	4275,1	3640,41	5184,6
851766	401834	5340	3640,64	3665,29	4511,87	4304,83	3638,87	5206
851796	401804	5212,5	3639,43	3666,84	4497,31	4334,94	3637,45	5226,16
851826	401774	5295	3638,16	3668,61	4480,46	4365,13	3635,95	5244,5
851856	401744	5336,25	3636,89	3670,66	4461,75	4395,3	3634,42	5261,35
851886	401714	5262,5	3635,7	3673,12	4441,63	4425,43	3632,95	5277,04
851916	401684	5252,5	3634,56	3675,95	4420,17	4455,17	3631,48	5291,4
851946	401654	5356,25	3633,35	3679,08	4397,39	4484,24	3629,92	5304,3
851976	401624	5261,25	3632,26	3682,73	4373,36	4512,66	3628,45	5315,67
852006	401594	5413,75	3631,08	3686,69	4348,34	4540,04	3626,83	5325,65

This journal is © The Royal Society of Chemistry 20xx

Please do not adjust margins

Please do not adjust margins

852036	401564	5343,75	3630,01	3691,27	4322,69	4566,4	3625,28	5334,52
852066	401534	5310	3629,01	3696,43	4296,53	4591,22	3623,76	5341,9
852096	401504	5283,75	3628,08	3702,19	4269,95	4614,29	3622,26	5347,72
852126	401474	5433,75	3627,07	3708,42	4242,91	4635,43	3620,62	5351,97
852156	401444	5511,25	3626,07	3715,24	4215,72	4654,56	3618,91	5354,84
852186	401414	5355	3625,26	3722,89	4188,69	4671,7	3617,34	5356,51
852216	401384	5303,75	3624,58	3731,32	4161,81	4686,25	3615,82	5356,49
852246	401354	5473,75	3623,81	3740,38	4135,01	4697,72	3614,14	5354,49
852276	401324	5321,25	3623,26	3750,4	4108,76	4706,7	3612,6	5351,33
852306	401294	5352,5	3622,8	3761,33	4082,9	4712,94	3611,03	5346,89
852336	401264	5233,75	3622,53	3773,36	4057,62	4716,49	3609,56	5341,31
852366	401234	5280	3622,32	3786,31	4032,81	4716,87	3608,05	5334,17
852396	401204	5290	3622,22	3800,28	4008,68	4713,75	3606,53	5325,34
852426	401174	5211,25	3622,32	3815,38	3985,38	4707,85	3605,08	5315,69
852456	401144	5473,75	3622,33	3831,35	3962,5	4698,91	3603,39	5304,92
852486	401114	5376,25	3622,56	3848,56	3940,4	4687,32	3601,79	5293,48
852516	401084	5236,25	3623,07	3867,09	3919,12	4673,06	3600,31	5281,41
852546	401054	5186,25	3623,78	3886,88	3898,8	4655,69	3598,88	5268,51
852576	401024	5136,25	3624,74	3908,06	3879,33	4636,05	3597,49	5255,71
852606	400994	5342,5	3625,71	3930,43	3860,41	4614,06	3595,91	5242,88
852636	400964	5263,75	3626,95	3954,18	3842,29	4590,13	3594,4	5230,34
852666	400934	5215	3628,43	3979,29	3824,94	4564,41	3592,94	5218,25
852696	400904	5261,25	3630,09	4005,67	3808,49	4536,57	3591,43	5206,53
852726	400874	5221,25	3632,09	4033,4	3792,8	4507,45	3589,96	5195,87
852756	400844	5108,75	3634,45	4062,52	3777,9	4477,3	3588,58	5186,4
852786	400814	5275	3636,91	4092,74	3763,51	4446,03	3587,06	5178,02
852816	400784	5222,5	3639,71	4124,22	3749,86	4414,04	3585,58	5171,07
852846	400754	5260	3642,78	4156,86	3737	4381,32	3584,07	5165,74
852876	400724	5097,5	3646,39	4190,78	3724,89	4348,42	3582,7	5162,37
852906	400694	5176,25	3650,26	4225,56	3713,29	4315,29	3581,26	5160,62
852936	400664	5112,5	3654,54	4261,2	3702,31	4282,19	3579,88	5160,62
852966	400634	5196,25	3659,1	4297,4	3691,87	4249,14	3578,42	5162,27
852996	400604	5176,25	3664,12	4334,1	3682,08	4216,43	3576,97	5165,82
853026	400574	5193,75	3669,6	4371,07	3672,77	4184,17	3575,51	5171,07
853056	400544	5177,5	3675,51	4408,11	3663,94	4152,44	3574,06	5177,82
853086	400514	5176,25	3681,86	4444,99	3655,56	4121,3	3572,61	5185,88
853116	400484	5123,75	3688,71	4481,46	3647,74	4090,89	3571,21	5195,16
853146	400454	5291,25	3695,99	4516,93	3640,23	4061,19	3569,66	5205,37
853176	400424	5220	3703,88	4551,37	3633,16	4032,49	3568,17	5216,41
853206	400394	5285	3712,22	4584,33	3626,4	4004,59	3566,61	5227,7
853236	400364	5301,25	3721,06	4615,55	3619,96	3977,56	3565,05	5238,99
853266	400334	5338,75	3730,39	4644,69	3613,9	3951,41	3563,44	5250,05
853296	400304	5285	3740,49	4671,51	3608,21	3926,34	3561,89	5260,88
853326	400274	5286,25	3751,16	4695,66	3602,79	3902,39	3560,33	5271,02
853356	400244	5308,75	3762,39	4716,83	3597,63	3879,36	3558,75	5279,96
853386	400214	5287,5	3774,19	4734,77	3592,73	3857,29	3557,19	5287,43
853416	400184	5151,25	3786,71	4748,87	3588,27	3836,27	3555,75	5292,88

This journal is © The Royal Society of Chemistry 20xx

Please do not adjust margins

853446	400154	5223,75	3799,92	4759,18	3583,97	3816,16	3554,24	5296,52
853476	400124	5212,5	3813,7	4765,64	3579,87	3797,19	3552,74	5298,18
853506	400094	5163,75	3828,08	4768,19	3576,01	3779,12	3551,28	5297,56
853536	400064	5247,5	3842,93	4766,65	3572,26	3761,84	3549,75	5294,43
853566	400034	5348,75	3858,26	4761,01	3568,63	3745,29	3548,12	5288,83
853596	400004	5352,5	3874,21	4751,41	3565,15	3729,57	3546,49	5280,87
853626	399974	5213,75	3890,75	4738,07	3561,94	3714,97	3544,99	5270,77
853656	399944	5058,75	3907,84	4721,15	3559	3701,25	3543,62	5258,38
853686	399914	5360	3925,05	4700,01	3555,92	3687,97	3541,98	5243,02
853716	399884	5360	3942,65	4675,6	3552,98	3675,37	3540,33	5225,6
853746	399854	5075	3960,8	4648,5	3550,4	3663,69	3538,95	5206,55
853776	399824	5133,75	3979,09	4618,67	3547,86	3652,76	3537,5	5185,86
853806	399794	5353,75	3997,28	4586,28	3545,22	3642,23	3535,86	5163,42
853836	399764	5150	4015,71	4552,03	3542,85	3632,44	3534,4	5139,82
853866	399734	5240	4033,97	4515,97	3540,49	3623,11	3532,86	5114,95
853896	399704	5055	4052,2	4478,66	3538,36	3614,45	3531,49	5089,22
853926	399674	5151,25	4070,04	4440,17	3536,21	3606,31	3530,02	5062,66
853956	399644	5061,25	4087,57	4400,91	3534,2	3598,71	3528,64	5035,48
853986	399614	5047,5	4104,62	4361,22	3532,27	3591,56	3527,27	5007,86
854016	399584	5127,5	4120,76	4321,27	3530,32	3584,73	3525,82	4979,62
854046	399554	4901,25	4136,27	4281,61	3528,61	3578,48	3524,58	4951,24
854076	399524	4863,75	4150,91	4242,25	3526,98	3572,68	3523,37	4922,73
854106	399494	4942,5	4164,47	4203,3	3525,32	3567,19	3522,08	4894,02
854136	399464	4826,25	4176,97	4165,11	3523,8	3562,1	3520,91	4865,26
854166	399434	4720	4187,9	4127,81	3522,41	3557,39	3519,82	4836,05
854196	399404	4720	4197,44	4091,43	3521,05	3552,95	3518,74	4806,66
854226	399374	4795	4205,44	4056,13	3519,65	3548,74	3517,58	4777,2
854256	399344	4847,5	4211,85	4021,97	3518,22	3544,75	3516,38	4747,64
854286	399314	4711,25	4216,58	3989,15	3516,94	3541,09	3515,3	4717,86
854316	399284	4795	4219,15	3957,51	3515,6	3537,56	3514,14	4687,38
854346	399254	4661,25	4220,11	3927,27	3514,4	3534,33	3513,11	4656,8
854376	399224	4580	4219,41	3898,42	3513,29	3531,37	3512,14	4626,06
854406	399194	4685	4216,87	3870,79	3512,1	3528,51	3511,08	4595,02
854436	399164	4590	4212,36	3844,56	3511,01	3525,88	3510,11	4563,5
854466	399134	4417,5	4206,04	3819,8	3510,09	3523,55	3509,29	4531,61
854496	399104	4317,5	4198,18	3796,5	3509,27	3521,42	3508,56	4499,7
854526	399074	4503,75	4188,59	3774,38	3508,28	3519,26	3507,66	4467,53
854556	399044	4456,25	4177,53	3753,52	3507,35	3517,27	3506,8	4435,27
854586	399014	4301,25	4164,85	3734	3506,57	3515,51	3506,08	4402,68
854616	398984	4267,5	4150,81	3715,65	3505,83	3513,88	3505,39	4369,98
854646	398954	4333,75	4135,55	3698,35	3505,03	3512,27	3504,65	4337,26
854676	398924	4206,25	4119,36	3682,24	3504,35	3510,84	3504,01	4304,75
854706	398894	4287,5	4102,13	3667,07	3503,6	3509,44	3503,3	4272,33
854736	398864	4213,75	4083,91	3652,94	3502,92	3508,17	3502,66	4239,95
854766	398834	4047,5	4065,18	3639,93	3502,4	3507,1	3502,17	4208,1
854796	398804	4140	4045,83	3627,8	3501,79	3506	3501,59	4176,64
854826	398774	4218,75	4025,98	3616,41	3501,12	3504,88	3500,94	4145,56

This journal is © The Royal Society of Chemistry 20xx

Please do not adjust margins

854856	398744	4098,75	4005,89	3605,92	3500,56	3503,92	3500,4	4115,07
854886	398714	4102,5	3985,55	3596,16	3499,99	3503	3499,86	4085,12
854916	398684	3940	3965,28	3587,24	3499,58	3502,26	3499,46	4055,97
854946	398654	3912,5	3945,05	3578,99	3499,19	3501,58	3499,09	4027,55
854976	398624	3870	3924,95	3571,4	3498,84	3500,97	3498,75	3999,9
855006	398594	4117,5	3904,8	3564,16	3498,27	3500,16	3498,19	3972,81
855036	398564	3982,5	3885,09	3557,6	3497,82	3499,5	3497,76	3946,74
855066	398534	3895	3865,78	3551,66	3497,46	3498,95	3497,4	3921,65
855096	398504	3972,5	3846,77	3546,11	3497,02	3498,34	3496,97	3897,34
855126	398474	3866,25	3828,26	3541,09	3496,68	3497,85	3496,63	3873,97
855156	398444	3887,5	3810,27	3536,44	3496,32	3497,35	3496,28	3851,54
855186	398414	3837,5	3792,91	3532,21	3496,01	3496,92	3495,97	3830,12
855216	398384	3810	3776,08	3528,34	3495,72	3496,53	3495,69	3809,6
855246	398354	3830	3759,78	3524,77	3495,41	3496,13	3495,39	3789,93
855276	398324	3678,75	3744,16	3521,63	3495,24	3495,87	3495,22	3771,24
855306	398294	3782,5	3729,17	3518,67	3494,98	3495,53	3494,96	3753,47
855336	398264	3747,5	3714,83	3516,02	3494,75	3495,24	3494,73	3736,64
855366	398234	3748,75	3701,06	3513,59	3494,52	3494,95	3494,5	3720,6
855396	398204	3737,5	3687,87	3511,37	3494,3	3494,67	3494,28	3705,36
855426	398174	3815	3675,17	3509,28	3494	3494,33	3493,99	3690,8
855456	398144	3781,25	3663,26	3507,39	3493,74	3494,03	3493,73	3677,22
855486	398114	3811,25	3651,87	3505,62	3493,45	3493,71	3493,45	3664,32
855516	398084	3557,5	3641,25	3504,24	3493,4	3493,62	3493,39	3652,33
855546	398054	3546,25	3631,16	3502,99	3493,35	3493,54	3493,34	3641,01
855576	398024	3700	3621,47	3501,73	3493,16	3493,33	3493,16	3630,22
855606	397994	3766,25	3612,36	3500,53	3492,91	3493,06	3492,91	3620,13
855636	397964	3656,25	3603,82	3499,52	3492,76	3492,89	3492,76	3610,72
855666	397934	3661,25	3595,74	3498,6	3492,61	3492,72	3492,61	3601,85
855696	397904	3605	3588,14	3497,81	3492,51	3492,61	3492,51	3593,55
855726	397874	3612,5	3581,03	3497,09	3492,4	3492,48	3492,4	3585,81
855756	397844	3561,25	3574,45	3496,48	3492,34	3492,41	3492,34	3578,67
855786	397814	3635	3568,19	3495,86	3492,21	3492,27	3492,21	3571,9
855816	397784	3578,75	3562,34	3495,34	3492,13	3492,18	3492,13	3565,61
855846	397754	3620	3556,81	3494,84	3492,01	3492,06	3492,01	3559,68
855876	397724	3596,25	3551,71	3494,4	3491,92	3491,96	3491,92	3554,24
855906	397694	3666,25	3546,9	3493,94	3491,76	3491,8	3491,76	3549,12
855936	397664	3597,5	3542,45	3493,58	3491,67	3491,7	3491,67	3544,39
855966	397634	3660	3538,22	3493,19	3491,51	3491,54	3491,51	3539,93
855996	397604	3613,75	3534,31	3492,87	3491,4	3491,43	3491,4	3535,8
856026	397574	3698,75	3530,64	3492,5	3491,22	3491,24	3491,22	3531,94
856056	397544	3581,25	3527,33	3492,25	3491,13	3491,15	3491,13	3528,46
856086	397514	3586,25	3524,23	3492,02	3491,05	3491,06	3491,05	3525,22
856116	397484	3607,5	3521,32	3491,79	3490,94	3490,96	3490,94	3522,18
856146	397454	3561,25	3518,66	3491,62	3490,88	3490,89	3490,88	3519,4
856176	397424	3438,75	3516,25	3491,47	3490,83	3490,84	3490,83	3516,9
856206	397394	3600	3513,97	3491,29	3490,73	3490,74	3490,73	3514,53
856236	397364	3586,25	3511,85	3491,13	3490,65	3490,65	3490,65	3512,34

This journal is © The Royal Society of Chemistry 20xx

Please do not adjust margins

Please do not adjust margins

856266	397334	3642,5	3509,83	3490,93	3490,51	3490,52	3490,51	3510,25
856296	397304	3515	3508,08	3490,85	3490,49	3490,49	3490,49	3508,45
856326	397274	3585	3506,43	3490,71	3490,4	3490,41	3490,4	3506,75
856356	397244	3611,25	3504,88	3490,56	3490,29	3490,3	3490,29	3505,15
856386	397214	3646,25	3503,4	3490,38	3490,15	3490,16	3490,15	3503,63
856416	397184	3537,5	3502,13	3490,31	3490,11	3490,11	3490,11	3502,33
856446	397154	3537,5	3500,97	3490,24	3490,07	3490,07	3490,07	3501,14
856476	397124	3517,5	3499,93	3490,19	3490,04	3490,04	3490,04	3500,08
856506	397094	3521,25	3498,97	3490,14	3490,01	3490,02	3490,01	3499,09
856536	397064	3528,75	3498,07	3490,09	3489,98	3489,98	3489,98	3498,18
856566	397034	3461,25	3497,26	3490,05	3489,95	3489,95	3489,95	3497,36
856596	397004	3402,5	3496,48	3489,95	3489,87	3489,88	3489,87	3496,56
856626	396974	3575	3495,76	3489,86	3489,8	3489,8	3489,8	3495,83
856656	396944	3578,75	3495,09	3489,77	3489,72	3489,72	3489,72	3495,15
856686	396914	3582,5	3494,47	3489,68	3489,63	3489,63	3489,63	3494,52
856716	396884	3533,75	3493,94	3489,63	3489,59	3489,59	3489,59	3493,98
856746	396854	3602,5	3493,4	3489,53	3489,49	3489,49	3489,49	3493,44
856776	396824	3645	3492,87	3489,38	3489,35	3489,35	3489,35	3492,9
856806	396794	3648,75	3492,36	3489,23	3489,21	3489,21	3489,21	3492,39
856836	396764	3470	3492,01	3489,21	3489,19	3489,19	3489,19	3492,04
856866	396734	3537,5	3491,67	3489,16	3489,15	3489,15	3489,15	3491,69
856896	396704	3607,5	3491,3	3489,05	3489,04	3489,04	3489,04	3491,32
856926	396674	3631,25	3490,94	3488,92	3488,91	3488,91	3488,91	3490,95
856956	396644	3391,25	3490,63	3488,83	3488,82	3488,82	3488,82	3490,64
856986	396614	3576,25	3490,36	3488,75	3488,74	3488,74	3488,74	3490,36
857016	396584	3520	3490,15	3488,72	3488,71	3488,71	3488,71	3490,16
857046	396554	3503,75	3489,98	3488,71	3488,7	3488,7	3488,7	3489,99
857076	396524	3543,75	3489,79	3488,66	3488,65	3488,65	3488,65	3489,8
857106	396494	3411,25	3489,6	3488,59	3488,58	3488,58	3488,58	3489,6
857136	396464	3542,5	3489,43	3488,54	3488,53	3488,53	3488,53	3489,44
857166	396434	3577,5	3489,25	3488,46	3488,45	3488,45	3488,45	3489,25
857196	396404	3530	3489,12	3488,42	3488,41	3488,41	3488,41	3489,13
857226	396374	3578,75	3488,96	3488,34	3488,33	3488,33	3488,33	3488,96
857256	396344	3468,75	3488,87	3488,32	3488,32	3488,32	3488,32	3488,87
857286	396314	3618,75	3488,69	3488,2	3488,2	3488,2	3488,2	3488,69
857316	396284	3528,75	3488,59	3488,16	3488,16	3488,16	3488,16	3488,6
857346	396254	3520	3488,51	3488,13	3488,13	3488,13	3488,13	3488,52
857376	396224	3441,25	3488,43	3488,09	3488,09	3488,09	3488,09	3488,43
857406	396194	3461,25	3488,36	3488,07	3488,07	3488,07	3488,07	3488,36
857436	396164	3408,75	3488,25	3487,99	3487,99	3487,99	3487,99	3488,26
857466	396134	3362,5	3488,11	3487,88	3487,88	3487,88	3487,88	3488,11
857496	396104	3592,5	3487,99	3487,79	3487,79	3487,79	3487,79	3487,99
857526	396074	3506,25	3487,95	3487,77	3487,77	3487,77	3487,77	3487,95
857556	396044	3502,5	3487,91	3487,76	3487,76	3487,76	3487,76	3487,91
857586	396014	3522,5	3487,86	3487,73	3487,72	3487,72	3487,72	3487,86
857616	395984	3476,25	3487,83	3487,71	3487,71	3487,71	3487,71	3487,83
857646	395954	3470	3487,8	3487,7	3487,7	3487,7	3487,7	3487,8

This journal is © The Royal Society of Chemistry 20xx

Please do not adjust margins

857676	395924	3451,25	3487,76	3487,67	3487,67	3487,67	3487,67	3487,76
857706	395894	3366,25	3487,64	3487,56	3487,56	3487,56	3487,56	3487,64
857736	395864	3538,75	3487,58	3487,51	3487,51	3487,51	3487,51	3487,58
857766	395834	3572,5	3487,49	3487,43	3487,43	3487,43	3487,43	3487,49
857796	395804	3505	3487,47	3487,42	3487,42	3487,42	3487,42	3487,47
857826	395774	3452,5	3487,43	3487,39	3487,39	3487,39	3487,39	3487,43
857856	395744	3596,25	3487,33	3487,29	3487,29	3487,29	3487,29	3487,33
857886	395714	3456,25	3487,29	3487,26	3487,26	3487,26	3487,26	3487,29
857916	395684	3481,25	3487,28	3487,25	3487,25	3487,25	3487,25	3487,28
857946	395654	3593,75	3487,18	3487,16	3487,16	3487,16	3487,16	3487,18
857976	395624	3396,25	3487,1	3487,08	3487,08	3487,08	3487,08	3487,1
858006	395594	3481,25	3487,09	3487,07	3487,07	3487,07	3487,07	3487,09
858036	395564	3337,5	3486,95	3486,94	3486,94	3486,94	3486,94	3486,95
858066	395534	3643,75	3486,81	3486,79	3486,79	3486,79	3486,79	3486,81
858096	395504	3563,75	3486,74	3486,72	3486,72	3486,72	3486,72	3486,74
858126	395474	3588,75	3486,64	3486,63	3486,63	3486,63	3486,63	3486,64
858156	395444	3475	3486,63	3486,62	3486,62	3486,62	3486,62	3486,63
858186	395414	3473,75	3486,62	3486,61	3486,61	3486,61	3486,61	3486,62
858216	395384	3556,25	3486,55	3486,55	3486,55	3486,55	3486,55	3486,55
858246	395354	3491,25	3486,55	3486,54	3486,54	3486,54	3486,54	3486,55
858276	395324	3482,5	3486,54	3486,54	3486,54	3486,54	3486,54	3486,54
858306	395294	3453,75	3486,51	3486,51	3486,51	3486,51	3486,51	3486,51
858336	395264	3522,5	3486,48	3486,48	3486,48	3486,48	3486,48	3486,48
858366	395234	3453,75	3486,45	3486,45	3486,45	3486,45	3486,45	3486,45
858396	395204	3538,75	3486,4	3486,4	3486,4	3486,4	3486,4	3486,4
858426	395174	3563,75	3486,33	3486,33	3486,33	3486,33	3486,33	3486,33
858456	395144	3441,25	3486,29	3486,29	3486,29	3486,29	3486,29	3486,29
858486	395114	3477,5	3486,28	3486,28	3486,28	3486,28	3486,28	3486,28
858516	395084	3466,25	3486,26	3486,26	3486,26	3486,26	3486,26	3486,26
858546	395054	3453,75	3486,24	3486,23	3486,23	3486,23	3486,23	3486,24
858576	395024	3433,75	3486,19	3486,19	3486,19	3486,19	3486,19	3486,19
858606	394994	3613,75	3486,07	3486,07	3486,07	3486,07	3486,07	3486,07
858636	394964	3556,25	3486,01	3486,01	3486,01	3486,01	3486,01	3486,01
858666	394934	3363,75	3485,9	3485,9	3485,9	3485,9	3485,9	3485,9
858696	394904	3563,75	3485,83	3485,83	3485,83	3485,83	3485,83	3485,83
858726	394874	3512,5	3485,8	3485,8	3485,8	3485,8	3485,8	3485,8
858756	394844	3456,25	3485,78	3485,78	3485,78	3485,78	3485,78	3485,78
858786	394814	3473,75	3485,77	3485,77	3485,77	3485,77	3485,77	3485,77
858816	394784	3518,75	3485,74	3485,74	3485,74	3485,74	3485,74	3485,74
858846	394754	3460	3485,71	3485,71	3485,71	3485,71	3485,71	3485,71
858876	394724	3342,5	3485,58	3485,58	3485,58	3485,58	3485,58	3485,58
858906	394694	3427,5	3485,53	3485,53	3485,53	3485,53	3485,53	3485,53
858936	394664	3496,25	3485,52	3485,52	3485,52	3485,52	3485,52	3485,52
858966	394634	3521,25	3485,49	3485,49	3485,49	3485,49	3485,49	3485,49
858996	394604	3466,25	3485,47	3485,47	3485,47	3485,47	3485,47	3485,47
859026	394574	3466,25	3485,45	3485,45	3485,45	3485,45	3485,45	3485,45
859056	394544	3467,5	3467,5	3467,5	3467,5	3467,5	3467,5	3467,5

859086	394514	3608,75	3608,75	3608,75	3608,75	3608,75	3608,75	3608,75
859116	394484	3611,25	3611,25	3611,25	3611,25	3611,25	3611,25	3611,25
859146	394454	3392,5	3392,5	3392,5	3392,5	3392,5	3392,5	3392,5
859176	394424	3540	3540	3540	3540	3540	3540	3540
859206	394394	3537,5	3537,5	3537,5	3537,5	3537,5	3537,5	3537,5
859236	394364	3463,75	3463,75	3463,75	3463,75	3463,75	3463,75	3463,75
859266	394334	3430	3430	3430	3430	3430	3430	3430
859296	394304	3498,75	3498,75	3498,75	3498,75	3498,75	3498,75	3498,75
859326	394274	3520	3520	3520	3520	3520	3520	3520
859356	394244	3507,5	3507,5	3507,5	3507,5	3507,5	3507,5	3507,5
859386	394214	3428,75	3428,75	3428,75	3428,75	3428,75	3428,75	3428,75
859416	394184	3498,75	3498,75	3498,75	3498,75	3498,75	3498,75	3498,75
859446	394154	3556,25	3556,25	3556,25	3556,25	3556,25	3556,25	3556,25
859476	394124	3688,75	3688,75	3688,75	3688,75	3688,75	3688,75	3688,75
859506	394094	3521,25	3521,25	3521,25	3521,25	3521,25	3521,25	3521,25
859536	394064	3536,25	3536,25	3536,25	3536,25	3536,25	3536,25	3536,25
859566	394034	3587,5	3587,5	3587,5	3587,5	3587,5	3587,5	3587,5
859596	394004	3521,25	3521,25	3521,25	3521,25	3521,25	3521,25	3521,25
859626	393974	3468,75	3468,75	3468,75	3468,75	3468,75	3468,75	3468,75
859656	393944	3686,25	3686,25	3686,25	3686,25	3686,25	3686,25	3686,25
859686	393914	3510	3510	3510	3510	3510	3510	3510
859716	393884	3577,5	3577,5	3577,5	3577,5	3577,5	3577,5	3577,5
859746	393854	3523,75	3523,75	3523,75	3523,75	3523,75	3523,75	3523,75
859776	393824	3407,5	3407,5	3407,5	3407,5	3407,5	3407,5	3407,5
859806	393794	3625	3625	3625	3625	3625	3625	3625
859836	393764	3723,75	3723,75	3723,75	3723,75	3723,75	3723,75	3723,75
859866	393734	3670	3670	3670	3670	3670	3670	3670
859896	393704	3401,25	3401,25	3401,25	3401,25	3401,25	3401,25	3401,25
859926	393674	3606,25	3606,25	3606,25	3606,25	3606,25	3606,25	3606,25
859956	393644	3498,75	3498,75	3498,75	3498,75	3498,75	3498,75	3498,75
859986	393614	3562,5	3562,5	3562,5	3562,5	3562,5	3562,5	3562,5
860016	393584	3500	3500	3500	3500	3500	3500	3500
860046	393554	3466,25	3466,25	3466,25	3466,25	3466,25	3466,25	3466,25
860076	393524	3621,25	3621,25	3621,25	3621,25	3621,25	3621,25	3621,25
860106	393494	3557,5	3557,5	3557,5	3557,5	3557,5	3557,5	3557,5
860136	393464	3531,25	3531,25	3531,25	3531,25	3531,25	3531,25	3531,25
860166	393434	3635	3635	3635	3635	3635	3635	3635
860196	393404	3628,75	3628,75	3628,75	3628,75	3628,75	3628,75	3628,75
860226	393374	3508,75	3508,75	3508,75	3508,75	3508,75	3508,75	3508,75
860256	393344	3602,5	3602,5	3602,5	3602,5	3602,5	3602,5	3602,5
860286	393314	3608,75	3608,75	3608,75	3608,75	3608,75	3608,75	3608,75
860316	393284	3610	3610	3610	3610	3610	3610	3610
860346	393254	3543,75	3543,75	3543,75	3543,75	3543,75	3543,75	3543,75
860376	393224	3561,25	3561,25	3561,25	3561,25	3561,25	3561,25	3561,25
860406	393194	3480	3480	3480	3480	3480	3480	3480
860436	393164	3605	3605	3605	3605	3605	3605	3605
860466	393134	3606,25	3606,25	3606,25	3606,25	3606,25	3606,25	3606,25

This journal is © The Royal Society of Chemistry 20xx

Please do not adjust margins

860496	393104	3555	3555	3555	3555	3555	3555	3555
860526	393074	3563,75	3563,75	3563,75	3563,75	3563,75	3563,75	3563,75
860556	393044	3652,5	3652,5	3652,5	3652,5	3652,5	3652,5	3652,5
860586	393014	3593,75	3593,75	3593,75	3593,75	3593,75	3593,75	3593,75
860616	392984	3480	3480	3480	3480	3480	3480	3480
860646	392954	3625	3625	3625	3625	3625	3625	3625
860676	392924	3551,25	3551,25	3551,25	3551,25	3551,25	3551,25	3551,25
860706	392894	3616,25	3616,25	3616,25	3616,25	3616,25	3616,25	3616,25
860736	392864	3631,25	3631,25	3631,25	3631,25	3631,25	3631,25	3631,25
860766	392834	3615	3615	3615	3615	3615	3615	3615
860796	392804	3537,5	3537,5	3537,5	3537,5	3537,5	3537,5	3537,5
860826	392774	3730	3730	3730	3730	3730	3730	3730
860856	392744	3593,75	3593,75	3593,75	3593,75	3593,75	3593,75	3593,75
860886	392714	3586,25	3586,25	3586,25	3586,25	3586,25	3586,25	3586,25
860916	392684	3707,5	3707,5	3707,5	3707,5	3707,5	3707,5	3707,5
860946	392654	3650	3650	3650	3650	3650	3650	3650
860976	392624	3683,75	3683,75	3683,75	3683,75	3683,75	3683,75	3683,75
861006	392594	3518,75	3518,75	3518,75	3518,75	3518,75	3518,75	3518,75
861036	392564	3543,75	3543,75	3543,75	3543,75	3543,75	3543,75	3543,75
861066	392534	3623,75	3623,75	3623,75	3623,75	3623,75	3623,75	3623,75
861096	392504	3586,25	3586,25	3586,25	3586,25	3586,25	3586,25	3586,25
861126	392474	3497,5	3497,5	3497,5	3497,5	3497,5	3497,5	3497,5
861156	392444	3633,75	3633,75	3633,75	3633,75	3633,75	3633,75	3633,75
861186	392414	3585	3585	3585	3585	3585	3585	3585
861216	392384	3701,25	3701,25	3701,25	3701,25	3701,25	3701,25	3701,25
861246	392354	3460	3460	3460	3460	3460	3460	3460
861276	392324	3482,5	3482,5	3482,5	3482,5	3482,5	3482,5	3482,5
861306	392294	3608,75	3608,75	3608,75	3608,75	3608,75	3608,75	3608,75
861336	392264	3593,75	3593,75	3593,75	3593,75	3593,75	3593,75	3593,75
861366	392234	3597,5	3597,5	3597,5	3597,5	3597,5	3597,5	3597,5
861396	392204	3635	3635	3635	3635	3635	3635	3635
861426	392174	3561,25	3561,25	3561,25	3561,25	3561,25	3561,25	3561,25
861456	392144	3608,75	3608,75	3608,75	3608,75	3608,75	3608,75	3608,75
861486	392114	3672,5	3672,5	3672,5	3672,5	3672,5	3672,5	3672,5
861516	392084	3583,75	3583,75	3583,75	3583,75	3583,75	3583,75	3583,75
861546	392054	3546,25	3546,25	3546,25	3546,25	3546,25	3546,25	3546,25
861576	392024	3603,75	3603,75	3603,75	3603,75	3603,75	3603,75	3603,75
861606	391994	3676,25	3676,25	3676,25	3676,25	3676,25	3676,25	3676,25
861636	391964	3537,5	3537,5	3537,5	3537,5	3537,5	3537,5	3537,5
861666	391934	3590	3590	3590	3590	3590	3590	3590
861696	391904	3592,5	3592,5	3592,5	3592,5	3592,5	3592,5	3592,5
861726	391874	3498,75	3498,75	3498,75	3498,75	3498,75	3498,75	3498,75
861756	391844	3611,25	3611,25	3611,25	3611,25	3611,25	3611,25	3611,25
861786	391814	3652,5	3652,5	3652,5	3652,5	3652,5	3652,5	3652,5
861816	391784	3716,25	3716,25	3716,25	3716,25	3716,25	3716,25	3716,25
861846	391754	3628,75	3628,75	3628,75	3628,75	3628,75	3628,75	3628,75
861876	391724	3723,75	3723,75	3723,75	3723,75	3723,75	3723,75	3723,75

This journal is © The Royal Society of Chemistry 20xx

Please do not adjust margins

Please do not adjust margins

861906	391694	3575	3575	3575	3575	3575	3575	3575
861936	391664	3588,75	3588,75	3588,75	3588,75	3588,75	3588,75	3588,75
861966	391634	3670	3670	3670	3670	3670	3670	3670
861996	391604	3522,5	3522,5	3522,5	3522,5	3522,5	3522,5	3522,5
862026	391574	3561,25	3561,25	3561,25	3561,25	3561,25	3561,25	3561,25
862056	391544	3732,5	3732,5	3732,5	3732,5	3732,5	3732,5	3732,5
862086	391514	3515	3515	3515	3515	3515	3515	3515
862116	391484	3652,5	3652,5	3652,5	3652,5	3652,5	3652,5	3652,5
862146	391454	3608,75	3608,75	3608,75	3608,75	3608,75	3608,75	3608,75
862176	391424	3643,75	3643,75	3643,75	3643,75	3643,75	3643,75	3643,75
862206	391394	3595	3595	3595	3595	3595	3595	3595
862236	391364	3552,5	3552,5	3552,5	3552,5	3552,5	3552,5	3552,5
862266	391334	3595	3595	3595	3595	3595	3595	3595
862296	391304	3545	3545	3545	3545	3545	3545	3545
862326	391274	3625	3625	3625	3625	3625	3625	3625
862356	391244	3552,5	3552,5	3552,5	3552,5	3552,5	3552,5	3552,5
862386	391214	3683,75	3683,75	3683,75	3683,75	3683,75	3683,75	3683,75
862416	391184	3552,5	3552,5	3552,5	3552,5	3552,5	3552,5	3552,5
862446	391154	3507,5	3507,5	3507,5	3507,5	3507,5	3507,5	3507,5
862476	391124	3493,75	3493,75	3493,75	3493,75	3493,75	3493,75	3493,75
862506	391094	3520	3520	3520	3520	3520	3520	3520
862536	391064	3588,75	3588,75	3588,75	3588,75	3588,75	3588,75	3588,75
862566	391034	3670	3670	3670	3670	3670	3670	3670
862596	391004	3627,5	3627,5	3627,5	3627,5	3627,5	3627,5	3627,5
862626	390974	3610	3610	3610	3610	3610	3610	3610
862656	390944	3636,25	3636,25	3636,25	3636,25	3636,25	3636,25	3636,25
862686	390914	3535	3535	3535	3535	3535	3535	3535
862716	390884	3522,5	3522,5	3522,5	3522,5	3522,5	3522,5	3522,5
862746	390854	3543,75	3543,75	3543,75	3543,75	3543,75	3543,75	3543,75
862776	390824	3453,75	3453,75	3453,75	3453,75	3453,75	3453,75	3453,75
862806	390794	3576,25	3576,25	3576,25	3576,25	3576,25	3576,25	3576,25
862836	390764	3745	3745	3745	3745	3745	3745	3745
862866	390734	3490	3490	3490	3490	3490	3490	3490
862896	390704	3448,75	3448,75	3448,75	3448,75	3448,75	3448,75	3448,75
862926	390674	3488,75	3488,75	3488,75	3488,75	3488,75	3488,75	3488,75
862956	390644	3452,5	3452,5	3452,5	3452,5	3452,5	3452,5	3452,5
862986	390614	3622,5	3622,5	3622,5	3622,5	3622,5	3622,5	3622,5
863016	390584	3526,25	3526,25	3526,25	3526,25	3526,25	3526,25	3526,25
863046	390554	3653,75	3653,75	3653,75	3653,75	3653,75	3653,75	3653,75
863076	390524	3535	3535	3535	3535	3535	3535	3535
863106	390494	3497,5	3497,5	3497,5	3497,5	3497,5	3497,5	3497,5
863136	390464	3610	3610	3610	3610	3610	3610	3610
863166	390434	3522,5	3522,5	3522,5	3522,5	3522,5	3522,5	3522,5
863196	390404	3555	3555	3555	3555	3555	3555	3555
863226	390374	3445	3445	3445	3445	3445	3445	3445
863256	390344	3488,75	3488,75	3488,75	3488,75	3488,75	3488,75	3488,75
863286	390314	3568,75	3568,75	3568,75	3568,75	3568,75	3568,75	3568,75

This journal is © The Royal Society of Chemistry 20xx

Please do not adjust margins

Please do not adjust margins

863316	390284	3522,5	3522,5	3522,5	3522,5	3522,5	3522,5	3522,5
863346	390254	3515	3515	3515	3515	3515	3515	3515
863376	390224	3397,5	3397,5	3397,5	3397,5	3397,5	3397,5	3397,5
863406	390194	3466,25	3466,25	3466,25	3466,25	3466,25	3466,25	3466,25
863436	390164	3568,75	3568,75	3568,75	3568,75	3568,75	3568,75	3568,75
863466	390134	3502,5	3502,5	3502,5	3502,5	3502,5	3502,5	3502,5
863496	390104	3633,75	3633,75	3633,75	3633,75	3633,75	3633,75	3633,75
863526	390074	3476,25	3476,25	3476,25	3476,25	3476,25	3476,25	3476,25
863556	390044	3395	3395	3395	3395	3395	3395	3395
863586	390014	3566,25	3566,25	3566,25	3566,25	3566,25	3566,25	3566,25
863616	389984	3531,25	3531,25	3531,25	3531,25	3531,25	3531,25	3531,25
863646	389954	3422,5	3422,5	3422,5	3422,5	3422,5	3422,5	3422,5
863676	389924	3473,75	3473,75	3473,75	3473,75	3473,75	3473,75	3473,75
863706	389894	3515	3515	3515	3515	3515	3515	3515
863736	389864	3455	3455	3455	3455	3455	3455	3455
863766	389834	3500	3500	3500	3500	3500	3500	3500
863796	389804	3406,25	3406,25	3406,25	3406,25	3406,25	3406,25	3406,25
863826	389774	3502,5	3502,5	3502,5	3502,5	3502,5	3502,5	3502,5
863856	389744	3455	3455	3455	3455	3455	3455	3455
863886	389714	3447,5	3447,5	3447,5	3447,5	3447,5	3447,5	3447,5
863916	389684	3513,75	3513,75	3513,75	3513,75	3513,75	3513,75	3513,75
863946	389654	3450	3450	3450	3450	3450	3450	3450
863976	389624	3595	3595	3595	3595	3595	3595	3595
864006	389594	3437,5	3437,5	3437,5	3437,5	3437,5	3437,5	3437,5
864036	389564	3608,75	3608,75	3608,75	3608,75	3608,75	3608,75	3608,75
864066	389534	3541,25	3541,25	3541,25	3541,25	3541,25	3541,25	3541,25
864096	389504	3461,25	3461,25	3461,25	3461,25	3461,25	3461,25	3461,25
864126	389474	3372,5	3372,5	3372,5	3372,5	3372,5	3372,5	3372,5
864156	389444	3525	3525	3525	3525	3525	3525	3525
864186	389414	3547,5	3547,5	3547,5	3547,5	3547,5	3547,5	3547,5
864216	389384	3496,25	3496,25	3496,25	3496,25	3496,25	3496,25	3496,25
864246	389354	3378,75	3378,75	3378,75	3378,75	3378,75	3378,75	3378,75
864276	389324	3401,25	3401,25	3401,25	3401,25	3401,25	3401,25	3401,25
864306	389294	3405	3405	3405	3405	3405	3405	3405
864336	389264	3470	3470	3470	3470	3470	3470	3470
864366	389234	3478,75	3478,75	3478,75	3478,75	3478,75	3478,75	3478,75
864396	389204	3417,5	3417,5	3417,5	3417,5	3417,5	3417,5	3417,5
864426	389174	3560	3560	3560	3560	3560	3560	3560
864456	389144	3463,75	3463,75	3463,75	3463,75	3463,75	3463,75	3463,75
864486	389114	3298,75	3298,75	3298,75	3298,75	3298,75	3298,75	3298,75
864516	389084	3527,5	3527,5	3527,5	3527,5	3527,5	3527,5	3527,5
864546	389054	3447,5	3447,5	3447,5	3447,5	3447,5	3447,5	3447,5
864576	389024	3407,5	3407,5	3407,5	3407,5	3407,5	3407,5	3407,5
864606	388994	3437,5	3437,5	3437,5	3437,5	3437,5	3437,5	3437,5
864636	388964	3452,5	3452,5	3452,5	3452,5	3452,5	3452,5	3452,5
864666	388934	3530	3530	3530	3530	3530	3530	3530
864696	388904	3462,5	3462,5	3462,5	3462,5	3462,5	3462,5	3462,5

Please do not adjust margins

864726	388874	3393,75	3393,75	3393,75	3393,75	3393,75	3393,75	3393,75
864756	388844	3318,75	3318,75	3318,75	3318,75	3318,75	3318,75	3318,75
864786	388814	3423,75	3423,75	3423,75	3423,75	3423,75	3423,75	3423,75
864816	388784	3211,25	3211,25	3211,25	3211,25	3211,25	3211,25	3211,25
864846	388754	3483,75	3483,75	3483,75	3483,75	3483,75	3483,75	3483,75
864876	388724	3408,75	3408,75	3408,75	3408,75	3408,75	3408,75	3408,75
864906	388694	3397,5	3397,5	3397,5	3397,5	3397,5	3397,5	3397,5

Table S8 Raw XPS measurements corresponding to Fig. 2.f.

KE_MgKa	BE_MgKa	CPS_MgKa	N 1s_1_MgKa	N 1s_2_MgKa	N 1s_3_MgKa	N 1s_4_MgKa	Background_MgKa	Envelope_MgKa
845416	408184	3851,33	3851,33	3851,33	3851,33	3851,33	3851,33	3851,33
845446	408154	3808,67	3808,67	3808,67	3808,67	3808,67	3808,67	3808,67
845476	408124	3707,33	3707,33	3707,33	3707,33	3707,33	3707,33	3707,33
845506	408094	3804	3804	3804	3804	3804	3804	3804
845536	408064	3859,33	3859,33	3859,33	3859,33	3859,33	3859,33	3859,33
845566	408034	3875,33	3875,33	3875,33	3875,33	3875,33	3875,33	3875,33
845596	408004	3867,33	3867,33	3867,33	3867,33	3867,33	3867,33	3867,33
845626	407974	3870,67	3870,67	3870,67	3870,67	3870,67	3870,67	3870,67
845656	407944	3910,67	3910,67	3910,67	3910,67	3910,67	3910,67	3910,67
845686	407914	3690	3690	3690	3690	3690	3690	3690
845716	407884	3813,33	3813,33	3813,33	3813,33	3813,33	3813,33	3813,33
845746	407854	3768,67	3768,67	3768,67	3768,67	3768,67	3768,67	3768,67
845776	407824	3846,67	3846,67	3846,67	3846,67	3846,67	3846,67	3846,67
845806	407794	3728	3728	3728	3728	3728	3728	3728
845836	407764	3740	3740	3740	3740	3740	3740	3740
845866	407734	3823,33	3823,33	3823,33	3823,33	3823,33	3823,33	3823,33
845896	407704	3853,33	3853,33	3853,33	3853,33	3853,33	3853,33	3853,33
845926	407674	3786,67	3786,67	3786,67	3786,67	3786,67	3786,67	3786,67
845956	407644	3724,67	3724,67	3724,67	3724,67	3724,67	3724,67	3724,67
845986	407614	3808,67	3808,67	3808,67	3808,67	3808,67	3808,67	3808,67
846016	407584	3782	3782	3782	3782	3782	3782	3782
846046	407554	3858	3858	3858	3858	3858	3858	3858
846076	407524	3764	3764	3764	3764	3764	3764	3764
846106	407494	3815,33	3815,33	3815,33	3815,33	3815,33	3815,33	3815,33
846136	407464	3749,33	3749,33	3749,33	3749,33	3749,33	3749,33	3749,33
846166	407434	3900,67	3900,67	3900,67	3900,67	3900,67	3900,67	3900,67
846196	407404	3824,67	3824,67	3824,67	3824,67	3824,67	3824,67	3824,67
846226	407374	3808	3808	3808	3808	3808	3808	3808
846256	407344	3755,33	3755,33	3755,33	3755,33	3755,33	3755,33	3755,33
846286	407314	3892	3892	3892	3892	3892	3892	3892
846316	407284	3800,67	3800,67	3800,67	3800,67	3800,67	3800,67	3800,67
846346	407254	3782	3782	3782	3782	3782	3782	3782
846376	407224	3754,67	3754,67	3754,67	3754,67	3754,67	3754,67	3754,67
846406	407194	3755,33	3755,33	3755,33	3755,33	3755,33	3755,33	3755,33
846436	407164	3906,67	3906,67	3906,67	3906,67	3906,67	3906,67	3906,67
846466	407134	3736	3736	3736	3736	3736	3736	3736

This journal is © The Royal Society of Chemistry 20xx

Please do not adjust margins

846496	407104	3844	3844	3844	3844	3844	3844	3844
846526	407074	3851,33	3851,33	3851,33	3851,33	3851,33	3851,33	3851,33
846556	407044	3830,67	3830,67	3830,67	3830,67	3830,67	3830,67	3830,67
846586	407014	3849,33	3849,33	3849,33	3849,33	3849,33	3849,33	3849,33
846616	406984	3726,67	3726,67	3726,67	3726,67	3726,67	3726,67	3726,67
846646	406954	3712,67	3712,67	3712,67	3712,67	3712,67	3712,67	3712,67
846676	406924	3759,33	3759,33	3759,33	3759,33	3759,33	3759,33	3759,33
846706	406894	3758	3758	3758	3758	3758	3758	3758
846736	406864	3832	3832	3832	3832	3832	3832	3832
846766	406834	3805,33	3805,33	3805,33	3805,33	3805,33	3805,33	3805,33
846796	406804	3783,33	3783,33	3783,33	3783,33	3783,33	3783,33	3783,33
846826	406774	3794	3794	3794	3794	3794	3794	3794
846856	406744	3733,33	3733,33	3733,33	3733,33	3733,33	3733,33	3733,33
846886	406714	3814	3814	3814	3814	3814	3814	3814
846916	406684	3738	3738	3738	3738	3738	3738	3738
846946	406654	3786,67	3786,67	3786,67	3786,67	3786,67	3786,67	3786,67
846976	406624	3774,67	3774,67	3774,67	3774,67	3774,67	3774,67	3774,67
847006	406594	3785,33	3785,33	3785,33	3785,33	3785,33	3785,33	3785,33
847036	406564	3742	3742	3742	3742	3742	3742	3742
847066	406534	3805,33	3805,33	3805,33	3805,33	3805,33	3805,33	3805,33
847096	406504	3692,67	3692,67	3692,67	3692,67	3692,67	3692,67	3692,67
847126	406474	3784,67	3784,67	3784,67	3784,67	3784,67	3784,67	3784,67
847156	406444	3803,33	3803,33	3803,33	3803,33	3803,33	3803,33	3803,33
847186	406414	3762	3762	3762	3762	3762	3762	3762
847216	406384	3732,67	3732,67	3732,67	3732,67	3732,67	3732,67	3732,67
847246	406354	3851,33	3851,33	3851,33	3851,33	3851,33	3851,33	3851,33
847276	406324	3741,33	3741,33	3741,33	3741,33	3741,33	3741,33	3741,33
847306	406294	3769,33	3769,33	3769,33	3769,33	3769,33	3769,33	3769,33
847336	406264	3787,33	3787,33	3787,33	3787,33	3787,33	3787,33	3787,33
847366	406234	3857,33	3857,33	3857,33	3857,33	3857,33	3857,33	3857,33
847396	406204	3746,67	3746,67	3746,67	3746,67	3746,67	3746,67	3746,67
847426	406174	3712,67	3712,67	3712,67	3712,67	3712,67	3712,67	3712,67
847456	406144	3736,67	3736,67	3736,67	3736,67	3736,67	3736,67	3736,67
847486	406114	3797,33	3797,33	3797,33	3797,33	3797,33	3797,33	3797,33
847516	406084	3795,33	3795,33	3795,33	3795,33	3795,33	3795,33	3795,33
847546	406054	3763,33	3763,33	3763,33	3763,33	3763,33	3763,33	3763,33
847576	406024	3764,67	3764,67	3764,67	3764,67	3764,67	3764,67	3764,67
847606	405994	3848,67	3848,67	3848,67	3848,67	3848,67	3848,67	3848,67
847636	405964	3720,67	3720,67	3720,67	3720,67	3720,67	3720,67	3720,67
847666	405934	3721,33	3721,33	3721,33	3721,33	3721,33	3721,33	3721,33
847696	405904	3791,33	3791,33	3791,33	3791,33	3791,33	3791,33	3791,33
847726	405874	3767,33	3767,33	3767,33	3767,33	3767,33	3767,33	3767,33
847756	405844	3738	3738	3738	3738	3738	3738	3738
847786	405814	3750,67	3750,67	3750,67	3750,67	3750,67	3750,67	3750,67
847816	405784	3745,33	3745,33	3745,33	3745,33	3745,33	3745,33	3745,33
847846	405754	3776,67	3776,67	3776,67	3776,67	3776,67	3776,67	3776,67
847876	405724	3739,33	3739,33	3739,33	3739,33	3739,33	3739,33	3739,33

Please do not adjust margins

847906	405694	3804	3739,27	3739,27	3739,27	3739,27	3739,27	3739,27
847936	405664	3656	3739,18	3739,18	3739,18	3739,18	3739,18	3739,18
847966	405634	3695,33	3739,14	3739,14	3739,14	3739,14	3739,14	3739,14
847996	405604	3856	3739,02	3739,02	3739,02	3739,02	3739,02	3739,02
848026	405574	3710	3738,99	3738,99	3738,99	3738,99	3738,99	3738,99
848056	405544	3782	3738,94	3738,94	3738,95	3738,94	3738,94	3738,95
848086	405514	3752,67	3738,93	3738,93	3738,94	3738,93	3738,93	3738,94
848116	405484	3730	3738,92	3738,92	3738,93	3738,92	3738,92	3738,93
848146	405454	3824	3738,83	3738,83	3738,84	3738,83	3738,83	3738,84
848176	405424	3767,33	3738,81	3738,81	3738,81	3738,81	3738,81	3738,81
848206	405394	3760,67	3738,78	3738,78	3738,79	3738,78	3738,78	3738,79
848236	405364	3703,33	3738,75	3738,75	3738,76	3738,75	3738,75	3738,76
848266	405334	3755,33	3738,73	3738,73	3738,74	3738,73	3738,73	3738,74
848296	405304	3688	3738,68	3738,68	3738,69	3738,68	3738,68	3738,69
848326	405274	3721,33	3738,66	3738,66	3738,68	3738,66	3738,66	3738,68
848356	405244	3814,67	3738,58	3738,58	3738,6	3738,58	3738,58	3738,6
848386	405214	3748	3738,57	3738,57	3738,59	3738,57	3738,57	3738,59
848416	405184	3794,67	3738,52	3738,52	3738,54	3738,52	3738,52	3738,54
848446	405154	3790,67	3738,46	3738,46	3738,49	3738,46	3738,46	3738,49
848476	405124	3693,33	3738,42	3738,42	3738,45	3738,42	3738,42	3738,45
848506	405094	3700	3738,38	3738,38	3738,41	3738,38	3738,38	3738,41
848536	405064	3790	3738,33	3738,33	3738,36	3738,33	3738,33	3738,36
848566	405034	3780,67	3738,28	3738,28	3738,33	3738,28	3738,28	3738,33
848596	405004	3728,67	3738,27	3738,27	3738,32	3738,27	3738,27	3738,32
848626	404974	3660	3738,19	3738,19	3738,25	3738,19	3738,19	3738,25
848656	404944	3635,33	3738,09	3738,09	3738,15	3738,09	3738,09	3738,15
848686	404914	3682,67	3738,03	3738,03	3738,11	3738,03	3738,03	3738,11
848716	404884	3743,33	3738,03	3738,03	3738,11	3738,03	3738,03	3738,11
848746	404854	3730	3738,02	3738,02	3738,12	3738,02	3738,02	3738,12
848776	404824	3764,67	3737,99	3737,99	3738,1	3737,99	3737,99	3738,1
848806	404794	3802,67	3737,92	3737,92	3738,05	3737,93	3737,92	3738,05
848836	404764	3778	3737,88	3737,88	3738,03	3737,88	3737,88	3738,03
848866	404734	3806,67	3737,81	3737,81	3737,98	3737,81	3737,81	3737,98
848896	404704	3688	3737,76	3737,76	3737,95	3737,76	3737,76	3737,95
848926	404674	3613,33	3737,64	3737,64	3737,85	3737,64	3737,64	3737,85
848956	404644	3648	3737,54	3737,54	3737,78	3737,55	3737,54	3737,78
848986	404614	3782,67	3737,5	3737,5	3737,77	3737,5	3737,5	3737,77
849016	404584	3752,67	3737,48	3737,48	3737,79	3737,49	3737,48	3737,79
849046	404554	3676,67	3737,42	3737,42	3737,77	3737,42	3737,42	3737,77
849076	404524	3736,67	3737,42	3737,42	3737,81	3737,42	3737,42	3737,81
849106	404494	3663,33	3737,34	3737,34	3737,78	3737,35	3737,34	3737,79
849136	404464	3722	3737,33	3737,33	3737,82	3737,33	3737,33	3737,83
849166	404434	3734,67	3737,33	3737,33	3737,88	3737,33	3737,33	3737,89
849196	404404	3689,33	3737,28	3737,28	3737,9	3737,28	3737,28	3737,91
849226	404374	3634,67	3737,17	3737,17	3737,87	3737,18	3737,17	3737,88
849256	404344	3780,67	3737,13	3737,13	3737,91	3737,14	3737,13	3737,92
849286	404314	3738,67	3737,13	3737,13	3738	3737,14	3737,13	3738,01

 This journal is © The Royal Society of Chemistry 20xx

Please do not adjust margins

849316	404284	3792,67	3737,07	3737,07	3738,05	3737,08	3737,07	3738,06
849346	404254	3784	3737,02	3737,02	3738,12	3737,04	3737,02	3738,13
849376	404224	3791,33	3736,97	3736,97	3738,19	3736,98	3736,97	3738,21
849406	404194	3772	3736,93	3736,93	3738,3	3736,95	3736,93	3738,32
849436	404164	3726	3736,92	3736,92	3738,45	3736,94	3736,92	3738,47
849466	404134	3680	3736,86	3736,86	3738,56	3736,89	3736,86	3738,59
849496	404104	3792	3736,81	3736,81	3738,7	3736,84	3736,81	3738,73
849526	404074	3784,67	3736,76	3736,76	3738,86	3736,79	3736,76	3738,9
849556	404044	3798	3736,69	3736,69	3739,03	3736,73	3736,69	3739,07
849586	404014	3817,33	3736,61	3736,61	3739,21	3736,66	3736,61	3739,26
849616	403984	3773,33	3736,57	3736,57	3739,46	3736,63	3736,57	3739,51
849646	403954	3762	3736,55	3736,55	3739,74	3736,61	3736,55	3739,81
849676	403924	3688	3736,5	3736,5	3740,03	3736,57	3736,5	3740,11
849706	403894	3736	3736,5	3736,5	3740,4	3736,58	3736,5	3740,49
849736	403864	3723,33	3736,48	3736,49	3740,81	3736,58	3736,48	3740,9
849766	403834	3799,33	3736,42	3736,42	3741,2	3736,53	3736,42	3741,31
849796	403804	3728,67	3736,41	3736,41	3741,68	3736,54	3736,41	3741,81
849826	403774	3774	3736,37	3736,38	3742,17	3736,52	3736,37	3742,32
849856	403744	3760	3736,35	3736,35	3742,74	3736,52	3736,35	3742,9
849886	403714	3708,67	3736,32	3736,33	3743,35	3736,51	3736,32	3743,55
849916	403684	3790,67	3736,27	3736,27	3744	3736,48	3736,27	3744,22
849946	403654	3708,67	3736,24	3736,24	3744,73	3736,49	3736,24	3744,98
849976	403624	3705,33	3736,21	3736,21	3745,52	3736,49	3736,21	3745,81
850006	403594	3686	3736,16	3736,16	3746,36	3736,48	3736,16	3746,69
850036	403564	3905,33	3735,98	3735,99	3747,17	3736,35	3735,98	3747,55
850066	403534	3680	3735,93	3735,94	3748,17	3736,35	3735,93	3748,6
850096	403504	3748	3735,91	3735,93	3749,3	3736,39	3735,91	3749,79
850126	403474	3782,67	3735,87	3735,88	3750,48	3736,41	3735,87	3751,03
850156	403444	3636	3735,76	3735,78	3751,72	3736,38	3735,76	3752,35
850186	403414	3707,33	3735,73	3735,75	3753,14	3736,43	3735,73	3753,86
850216	403384	3669,33	3735,67	3735,69	3754,63	3736,46	3735,67	3755,45
850246	403354	3832	3735,57	3735,59	3756,21	3736,46	3735,57	3757,13
850276	403324	3753,33	3735,55	3735,58	3757,98	3736,56	3735,55	3759,02
850306	403294	3764	3735,52	3735,56	3759,91	3736,67	3735,52	3761,09
850336	403264	3850	3735,4	3735,44	3761,89	3736,7	3735,4	3763,22
850366	403234	3793,33	3735,35	3735,39	3764,07	3736,8	3735,35	3765,58
850396	403204	3732,67	3735,34	3735,4	3766,46	3736,98	3735,34	3768,15
850426	403174	3818	3735,26	3735,32	3768,93	3737,1	3735,26	3770,84
850456	403144	3768,67	3735,22	3735,3	3771,67	3737,3	3735,22	3773,82
850486	403114	3788,67	3735,17	3735,25	3774,56	3737,51	3735,17	3776,98
850516	403084	3784,67	3735,12	3735,21	3777,64	3737,74	3735,12	3780,36
850546	403054	3774	3735,08	3735,19	3780,92	3738,01	3735,08	3783,97
850576	403024	3755,33	3735,06	3735,19	3784,45	3738,35	3735,06	3787,87
850606	402994	3862	3734,93	3735,08	3788,13	3738,62	3734,93	3791,96
850636	402964	3804	3734,86	3735,03	3792,08	3738,98	3734,86	3796,37
850666	402934	3866	3734,72	3734,92	3796,19	3739,33	3734,72	3800,99
850696	402904	3770,67	3734,69	3734,91	3800,63	3739,82	3734,69	3805,99

This journal is © The Royal Society of Chemistry 20xx

Please do not adjust margins

Please do not adjust margins

850726	402874	3771,33	3734,65	3734,9	3805,37	3740,38	3734,65	3811,35
850756	402844	3766	3734,62	3734,91	3810,39	3741,01	3734,62	3817,07
850786	402814	3790,67	3734,56	3734,89	3815,64	3741,68	3734,56	3823,08
850816	402784	3837,33	3734,46	3734,83	3821,1	3742,37	3734,46	3829,38
850846	402754	3850,67	3734,34	3734,77	3826,8	3743,12	3734,34	3836,02
850876	402724	3772,67	3734,3	3734,79	3832,93	3744,06	3734,3	3843,19
850906	402694	3914,67	3734,12	3734,67	3839,2	3744,95	3734,11	3850,6
850936	402664	3933,33	3733,91	3734,55	3845,72	3745,91	3733,91	3858,36
850966	402634	3884	3733,76	3734,48	3852,55	3747,04	3733,76	3866,56
850996	402604	3808,67	3733,68	3734,5	3859,73	3748,37	3733,68	3875,24
851026	402574	3879,33	3733,53	3734,46	3867,19	3749,78	3733,53	3884,37
851056	402544	3920	3733,34	3734,4	3874,84	3751,29	3733,34	3893,85
851086	402514	3829,33	3733,25	3734,44	3882,83	3753,04	3733,24	3903,82
851116	402484	3968,67	3733,01	3734,35	3890,9	3754,8	3733	3914,05
851146	402454	4004	3732,73	3734,25	3899,17	3756,74	3732,73	3924,71
851176	402424	3976	3732,48	3734,2	3907,7	3758,92	3732,48	3935,87
851206	402394	3880,67	3732,33	3734,27	3916,48	3761,4	3732,33	3947,5
851236	402364	4006	3732,05	3734,24	3925,28	3763,97	3732,05	3959,39
851266	402334	4011,33	3731,77	3734,23	3934,18	3766,76	3731,76	3971,65
851296	402304	4024	3731,47	3734,24	3943,15	3769,86	3731,46	3984,33
851326	402274	4004	3731,19	3734,31	3952,18	3773,27	3731,19	3997,39
851356	402244	3984,67	3730,94	3734,42	3961,21	3776,98	3730,93	4010,78
851386	402214	3994	3730,67	3734,58	3970,17	3781	3730,66	4024,45
851416	402184	3946	3730,45	3734,84	3979,09	3785,39	3730,44	4038,46
851446	402154	4041,33	3730,14	3735,05	3987,71	3790,15	3730,12	4052,7
851476	402124	4059,33	3729,8	3735,3	3996,07	3795,28	3729,78	4067,1
851506	402094	4028	3729,5	3735,63	4004,17	3800,84	3729,48	4081,71
851536	402064	4112,67	3729,11	3735,96	4011,86	3806,73	3729,09	4096,4
851566	402034	3990,67	3728,85	3736,5	4019,3	3813,24	3728,82	4111,42
851596	402004	4174	3728,4	3736,92	4025,96	3820,16	3728,37	4126,34
851626	401974	4036	3728,09	3737,57	4032,21	3827,73	3728,05	4141,45
851656	401944	4109,33	3727,71	3738,24	4037,82	3835,77	3727,66	4156,55
851686	401914	4102,67	3727,33	3739,05	4042,82	3844,37	3727,28	4171,74
851716	401884	4046	3727,01	3740,03	4047,21	3853,73	3726,95	4187,12
851746	401854	4055,33	3726,68	3741,12	4050,64	3863,82	3726,62	4202,42
851776	401824	4147,33	3726,26	3742,26	4053,23	3874,49	3726,19	4217,67
851806	401794	4232	3725,76	3743,45	4054,95	3885,76	3725,67	4232,9
851836	401764	4170	3725,32	3744,91	4055,95	3897,8	3725,22	4248,32
851866	401734	4270,67	3724,77	3746,44	4055,94	3910,69	3724,66	4263,86
851896	401704	4318	3724,18	3748,1	4054,87	3924,41	3724,06	4279,39
851926	401674	4297,33	3723,62	3749,99	4053	3938,97	3723,47	4295,16
851956	401644	4308	3723,04	3752,09	4050,33	3954,36	3722,87	4311,2
851986	401614	4286,67	3722,48	3754,51	4046,89	3970,65	3722,3	4327,65
852016	401584	4406	3721,81	3757,06	4042,44	3988,02	3721,6	4344,55
852046	401554	4346,67	3721,2	3759,95	4037,22	4006,42	3720,96	4361,91
852076	401524	4452,67	3720,49	3763	4031,22	4025,67	3720,21	4379,75
852106	401494	4290,67	3719,94	3766,6	4024,76	4046,06	3719,63	4398,47

This journal is © The Royal Society of Chemistry 20xx

Please do not adjust margins

Please do not adjust margins

852136	401464	4359,33	3719,33	3770,52	4017,65	4067,41	3718,98	4417,97
852166	401434	4394	3718,69	3774,77	4009,84	4090,04	3718,29	4438,48
852196	401404	4462,67	3717,98	3779,33	4001,47	4113,62	3717,53	4459,82
852226	401374	4502,67	3717,23	3784,27	3992,67	4138,16	3716,73	4482,15
852256	401344	4542	3716,46	3789,72	3983,48	4163,68	3715,88	4505,68
852286	401314	4613,33	3715,61	3795,62	3973,91	4190,24	3714,97	4530,48
852316	401284	4560,67	3714,83	3802,08	3964,11	4218,06	3714,1	4556,77
852346	401254	4634,67	3713,98	3809	3954,06	4246,74	3713,16	4584,3
852376	401224	4569,33	3713,21	3816,55	3943,98	4276,4	3712,29	4613,26
852406	401194	4586	3712,43	3824,89	3933,8	4306,9	3711,4	4643,83
852436	401164	4757,33	3711,48	3833,73	3923,42	4338,18	3710,33	4675,83
852466	401134	4748,67	3710,56	3843,26	3913,09	4370,29	3709,27	4709,41
852496	401104	4796,67	3709,61	3853,46	3902,77	4402,97	3708,16	4744,35
852526	401074	4869,33	3708,6	3864,39	3892,49	4436,11	3706,97	4780,67
852556	401044	4798,67	3707,67	3876,43	3882,4	4469,75	3705,86	4818,69
852586	401014	4817,33	3706,75	3889,31	3872,47	4503,74	3704,72	4858,11
852616	400984	4842,67	3705,82	3903,06	3862,75	4537,81	3703,56	4898,76
852646	400954	4900,67	3704,86	3917,68	3853,17	4571,78	3702,34	4940,47
852676	400924	4946	3703,87	3933,35	3843,77	4605,5	3701,07	4983,3
852706	400894	4977,33	3702,88	3950,25	3834,57	4638,86	3699,76	5027,26
852736	400864	5013,33	3701,89	3968,18	3825,62	4671,39	3698,42	5071,82
852766	400834	5126	3700,82	3987,11	3816,86	4702,94	3696,96	5116,83
852796	400804	5138	3699,77	4007,17	3808,34	4733,43	3695,49	5162,24
852826	400774	5206	3698,68	4028,59	3800,03	4762,64	3693,95	5208,1
852856	400744	5260	3697,59	4051,38	3791,92	4790,3	3692,35	5254,14
852886	400714	5248,67	3696,56	4075,45	3784,16	4815,75	3690,76	5299,64
852916	400684	5210,67	3695,62	4100,86	3776,73	4839,28	3689,21	5344,86
852946	400654	5263,33	3694,68	4127,52	3769,5	4860,63	3687,6	5389,54
852976	400624	5397,33	3693,67	4155,79	3762,39	4879,54	3685,85	5433,83
853006	400594	5485,33	3692,62	4185,44	3755,45	4895,55	3684,02	5477,02
853036	400564	5508	3691,64	4216,44	3748,82	4908,24	3682,15	5518,69
853066	400534	5530,67	3690,71	4248,8	3742,4	4918,07	3680,26	5559,19
853096	400504	5563,33	3689,82	4282,51	3736,19	4924,94	3678,34	5598,44
853126	400474	5628	3688,96	4318	3730,13	4928,75	3676,35	5636,78
853156	400444	5731,33	3688,08	4354,71	3724,19	4928,75	3674,25	5672,98
853186	400414	5756	3687,3	4392,69	3718,51	4925,17	3672,13	5707,29
853216	400384	5679,33	3686,71	4431,99	3713,09	4918,59	3670,08	5740,16
853246	400354	5746	3686,16	4472,52	3707,8	4908,92	3667,96	5771,53
853276	400324	5743,33	3685,73	4514,45	3702,7	4896,27	3665,84	5801,64
853306	400294	5710	3685,48	4557,39	3697,83	4879,98	3663,75	5829,44
853336	400264	5852	3685,26	4601,06	3693,02	4860,66	3661,51	5855,45
853366	400234	5994	3685,03	4645,34	3688,21	4838,65	3659,13	5879,84
853396	400204	5921,33	3685,04	4690,42	3683,63	4814,32	3656,82	5902,94
853426	400174	5963,33	3685,16	4735,92	3679,15	4787,7	3654,47	5924,54
853456	400144	5940,67	3685,54	4781,63	3674,85	4758,42	3652,13	5944,05
853486	400114	6012	3686,04	4827,26	3670,63	4727,37	3649,72	5962,14
853516	400084	5958,67	3686,8	4872,76	3666,58	4694,84	3647,36	5978,9

This journal is © The Royal Society of Chemistry 20xx

Please do not adjust margins

Please do not adjust margins

853546	400054	6007,33	3687,72	4917,65	3662,58	4660,93	3644,95	5994,04
853576	400024	6084	3688,78	4961,56	3658,62	4625,73	3642,46	6007,31
853606	399994	6055,33	3690,19	5004,41	3654,81	4589,46	3640	6018,88
853636	399964	5944	3691,96	5046,05	3651,21	4552,69	3637,64	6028,99
853666	399934	6152,67	3693,78	5085,91	3647,49	4515,26	3635,07	6037,22
853696	399904	6085,33	3695,94	5123,32	3643,91	4477,62	3632,57	6043,08
853726	399874	5896,67	3698,6	5158,48	3640,6	4440,04	3630,26	6046,94
853756	399844	5934	3701,6	5191,01	3637,35	4402,55	3627,91	6048,79
853786	399814	5966,67	3704,88	5220,69	3634,13	4365,28	3625,52	6048,43
853816	399784	6107,33	3708,35	5247,1	3630,83	4328,24	3622,98	6045,56
853846	399754	6006,67	3712,25	5269,08	3627,68	4291,77	3620,55	6039,13
853876	399724	6046	3716,5	5287,37	3624,55	4255,98	3618,07	6030,18
853906	399694	6025,33	3721,22	5301,86	3621,5	4221,02	3615,61	6018,75
853936	399664	5929,33	3726,4	5312,49	3618,6	4186,93	3613,25	6004,67
853966	399634	5904	3731,99	5318,69	3615,76	4153,69	3610,91	5987,4
853996	399604	5972,67	3737,89	5319,54	3612,88	4121,27	3608,5	5966,09
854026	399574	5881,33	3744,37	5316,27	3610,15	4090,15	3606,18	5942,4
854056	399544	5938,67	3751,24	5308,74	3607,39	4060	3603,8	5915,99
854086	399514	5913,33	3758,55	5297,08	3604,68	4030,87	3601,44	5886,86
854116	399484	5808,67	3766,36	5280,75	3602,11	4002,84	3599,18	5854,51
854146	399454	5831,33	3774,55	5259,77	3599,54	3975,82	3596,9	5818,97
854176	399424	5842	3783,24	5235,06	3596,99	3950,15	3594,61	5781,6
854206	399394	5662	3792,53	5207	3594,64	3925,66	3592,5	5742,34
854236	399364	5626,67	3802,23	5175,63	3592,35	3902,2	3590,42	5701,15
854266	399334	5640,67	3812,29	5140,44	3590,06	3879,71	3588,33	5657,51
854296	399304	5611,33	3822,72	5102,27	3587,81	3858,3	3586,26	5612,32
854326	399274	5532	3833,67	5061,77	3585,67	3838,16	3584,27	5566,44
854356	399244	5609,33	3844,82	5019,03	3583,45	3818,85	3582,21	5519,53
854386	399214	5428	3856,4	4974,55	3581,44	3800,61	3580,32	5472,03
854416	399184	5346	3868,29	4928,11	3579,52	3783,31	3578,52	5423,67
854446	399154	5353,33	3880,36	4880,46	3577,6	3766,98	3576,71	5375,28
854476	399124	5296,67	3892,64	4831,95	3575,75	3751,65	3574,95	5327,13
854506	399094	5418,67	3904,84	4782,62	3573,78	3736,95	3573,07	5278,99
854536	399064	5227,33	3917,23	4733,01	3572,01	3723,18	3571,38	5231,3
854566	399034	5144	3929,64	4683,29	3570,33	3710,2	3569,77	5184,16
854596	399004	5133,33	3941,91	4633,72	3568,67	3698,06	3568,17	5137,85
854626	398974	5156,67	3953,87	4584,42	3567	3686,61	3566,55	5092,24
854656	398944	5034	3965,63	4535,69	3565,45	3675,88	3565,05	5047,48
854686	398914	4940,67	3977,09	4487,74	3564	3665,82	3563,65	5003,7
854716	398884	4946,67	3988,07	4440,79	3562,55	3656,3	3562,24	4961
854746	398854	4948	3998,35	4394,78	3561,09	3647,46	3560,82	4919,23
854776	398824	4844	4007,98	4349,92	3559,75	3639,22	3559,51	4878,34
854806	398794	4858	4016,82	4306,15	3558,4	3631,43	3558,18	4838,24
854836	398764	4732	4024,96	4263,93	3557,17	3624,2	3556,98	4799,3
854866	398734	4784,67	4032,14	4223,14	3555,9	3617,33	3555,73	4761,32
854896	398704	4813,33	4037,99	4183,65	3554,59	3610,95	3554,45	4723,84
854926	398674	4740,67	4042,79	4145,57	3553,36	3604,99	3553,23	4687,01

This journal is © The Royal Society of Chemistry 20xx

Please do not adjust margins

Please do not adjust margins

854956	398644	4680,67	4046,52	4108,92	3552,2	3599,42	3552,08	4650,81
854986	398614	4624	4049,16	4074,07	3551,09	3594,22	3550,99	4615,57
855016	398584	4635,33	4050,55	4040,72	3549,97	3589,35	3549,88	4580,94
855046	398554	4484	4050,47	4008,9	3549	3584,96	3548,93	4546,55
855076	398524	4483,33	4049,15	3978,45	3548,04	3580,83	3547,97	4512,54
855106	398494	4378,67	4046,7	3949,44	3547,18	3577,04	3547,12	4479
855136	398464	4391,33	4043,03	3922,19	3546,31	3573,46	3546,26	4446,21
855166	398434	4448	4037,99	3896,19	3545,39	3570,07	3545,34	4413,61
855196	398404	4338,67	4031,61	3871,55	3544,57	3567,01	3544,53	4381,15
855226	398374	4318	4024,18	3848,15	3543,78	3564,14	3543,74	4349,02
855256	398344	4302	4015,73	3826,02	3543	3561,46	3542,97	4317,3
855286	398314	4304,67	4006,3	3805,37	3542,21	3558,92	3542,19	4286,24
855316	398284	4220	3995,91	3785,89	3541,52	3556,65	3541,5	4255,48
855346	398254	4294	3984,51	3767,39	3540,75	3554,44	3540,73	4224,89
855376	398224	4185,33	3972,51	3750,01	3540,09	3552,46	3540,07	4194,85
855406	398194	4122,67	3959,95	3733,79	3539,49	3550,65	3539,47	4165,46
855436	398164	4070	3946,9	3718,75	3538,95	3549	3538,93	4136,79
855466	398134	4126,67	3933,21	3704,52	3538,34	3547,41	3538,33	4108,49
855496	398104	4016	3919,27	3691,24	3537,85	3546,02	3537,84	4080,85
855526	398074	4067,33	3905,01	3678,7	3537,31	3544,66	3537,3	4053,77
855556	398044	3916,67	3890,7	3667,23	3536,92	3543,52	3536,92	4027,62
855586	398014	3933,33	3876,25	3656,55	3536,52	3542,43	3536,51	4002,22
855616	397984	3921,33	3861,76	3646,55	3536,12	3541,44	3536,12	3977,51
855646	397954	3972	3847,25	3637,13	3535,68	3540,44	3535,67	3953,48
855676	397924	3848	3832,97	3628,44	3535,36	3539,62	3535,35	3930,32
855706	397894	3912,67	3818,75	3620,4	3534,97	3538,78	3534,97	3908
855736	397864	3871,33	3804,77	3612,97	3534,63	3538,03	3534,63	3886,52
855766	397834	3759,33	3791,21	3606,14	3534,4	3537,44	3534,4	3866
855796	397804	3899,33	3777,78	3599,63	3534,03	3536,74	3534,02	3846,11
855826	397774	3786,67	3764,78	3593,68	3533,77	3536,19	3533,77	3827,12
855856	397744	3827,33	3752,07	3588,24	3533,47	3535,62	3533,47	3808,99
855886	397714	3776,67	3739,8	3583,22	3533,22	3535,13	3533,22	3791,71
855916	397684	3812,67	3727,95	3578,51	3532,93	3534,63	3532,93	3775,24
855946	397654	3834	3716,47	3574,12	3532,63	3534,14	3532,62	3759,48
855976	397624	3744,67	3705,48	3570,15	3532,41	3533,75	3532,41	3744,56
856006	397594	3704	3694,93	3566,58	3532,23	3533,42	3532,23	3730,46
856036	397564	3771,33	3684,79	3563,2	3531,99	3533,04	3531,99	3717,05
856066	397534	3818,67	3675,08	3560,02	3531,7	3532,63	3531,7	3704,33
856096	397504	3604	3665,98	3557,28	3531,62	3532,44	3531,62	3692,47
856126	397474	3750	3657,14	3554,64	3531,4	3532,12	3531,4	3681,11
856156	397444	3662	3648,78	3552,33	3531,27	3531,9	3531,27	3670,48
856186	397414	3593,33	3640,99	3550,25	3531,2	3531,77	3531,2	3660,6
856216	397384	3663,33	3633,53	3548,28	3531,07	3531,56	3531,07	3651,23
856246	397354	3663,33	3626,43	3546,45	3530,93	3531,37	3530,93	3642,39
856276	397324	3736	3619,62	3544,72	3530,72	3531,11	3530,72	3634,01
856306	397294	3596,67	3613,3	3543,28	3530,66	3530,99	3530,66	3626,26
856336	397264	3692,67	3607,33	3541,86	3530,49	3530,79	3530,49	3618,99

This journal is © The Royal Society of Chemistry 20xx

Please do not adjust margins

856366	397234	3610,67	3601,76	3540,63	3530,41	3530,67	3530,41	3612,24
856396	397204	3605,33	3596,51	3539,51	3530,33	3530,56	3530,33	3605,91
856426	397174	3618	3591,54	3538,49	3530,24	3530,44	3530,24	3599,98
856456	397144	3703,33	3586,78	3537,47	3530,07	3530,24	3530,07	3594,36
856486	397114	3670,67	3582,41	3536,56	3529,92	3530,07	3529,92	3589,19
856516	397084	3550	3578,41	3535,84	3529,9	3530,03	3529,9	3584,48
856546	397054	3669,33	3574,53	3535,06	3529,76	3529,87	3529,76	3579,95
856576	397024	3643,33	3570,92	3534,39	3529,64	3529,74	3529,64	3575,76
856606	396994	3510,67	3567,65	3533,86	3529,62	3529,71	3529,62	3571,98
856636	396964	3608,67	3564,57	3533,33	3529,54	3529,62	3529,54	3568,43
856666	396934	3503,33	3561,74	3532,88	3529,52	3529,58	3529,52	3565,18
856696	396904	3621,33	3559,03	3532,42	3529,42	3529,48	3529,42	3562,08
856726	396874	3537,33	3556,58	3532,08	3529,41	3529,46	3529,41	3559,3
856756	396844	3610,67	3554,26	3531,71	3529,33	3529,37	3529,33	3556,67
856786	396814	3592,67	3552,12	3531,37	3529,27	3529,3	3529,27	3554,27
856816	396784	3642,67	3550,08	3531,02	3529,15	3529,18	3529,15	3551,98
856846	396754	3556	3548,27	3530,78	3529,12	3529,15	3529,12	3549,95
856876	396724	3585,33	3546,55	3530,53	3529,07	3529,09	3529,07	3548,04
856906	396694	3668,67	3544,9	3530,22	3528,92	3528,94	3528,92	3546,22
856936	396664	3634,67	3543,4	3529,96	3528,82	3528,83	3528,82	3544,56
856966	396634	3502	3542,08	3529,8	3528,79	3528,8	3528,79	3543,11
856996	396604	3639,33	3540,78	3529,57	3528,67	3528,69	3528,67	3541,69
857026	396574	3484,67	3539,63	3529,42	3528,63	3528,64	3528,63	3540,43
857056	396544	3648,67	3538,52	3529,2	3528,51	3528,52	3528,51	3539,23
857086	396514	3582	3537,55	3529,06	3528,45	3528,46	3528,45	3538,17
857116	396484	3560	3536,68	3528,96	3528,42	3528,43	3528,42	3537,22
857146	396454	3551,33	3535,88	3528,87	3528,4	3528,4	3528,4	3536,36
857176	396424	3614,67	3535,09	3528,72	3528,31	3528,31	3528,31	3535,5
857206	396394	3594	3534,38	3528,6	3528,24	3528,25	3528,24	3534,75
857236	396364	3530,67	3533,79	3528,56	3528,24	3528,24	3528,24	3534,11
857266	396334	3644	3533,14	3528,4	3528,12	3528,12	3528,12	3533,42
857296	396304	3563,33	3532,61	3528,33	3528,09	3528,09	3528,09	3532,86
857326	396274	3610	3532,08	3528,21	3528	3528	3528	3532,3
857356	396244	3571,33	3531,64	3528,14	3527,96	3527,96	3527,96	3531,82
857386	396214	3570	3531,23	3528,07	3527,91	3527,92	3527,91	3531,39
857416	396184	3540,67	3530,88	3528,04	3527,9	3527,9	3527,9	3531,02
857446	396154	3617,33	3530,49	3527,93	3527,81	3527,81	3527,81	3530,61
857476	396124	3633,33	3530,1	3527,81	3527,7	3527,7	3527,7	3530,21
857506	396094	3604,67	3529,78	3527,72	3527,62	3527,62	3527,62	3529,87
857536	396064	3522,67	3529,55	3527,7	3527,62	3527,62	3527,62	3529,63
857566	396034	3510	3529,33	3527,67	3527,6	3527,6	3527,6	3529,4
857596	396004	3548,67	3529,12	3527,64	3527,58	3527,58	3527,58	3529,18
857626	395974	3564,67	3528,92	3527,59	3527,54	3527,54	3527,54	3528,98
857656	395944	3636	3528,67	3527,47	3527,43	3527,43	3527,43	3528,71
857686	395914	3563,33	3528,5	3527,43	3527,39	3527,39	3527,39	3528,53
857716	395884	3578	3528,32	3527,38	3527,34	3527,34	3527,34	3528,36
857746	395854	3593,33	3528,15	3527,3	3527,27	3527,27	3527,27	3528,18

This journal is © The Royal Society of Chemistry 20xx

Please do not adjust margins

857776	395824	3526,67	3528,05	3527,3	3527,27	3527,27	3527,27	3528,08
857806	395794	3575,33	3527,92	3527,25	3527,23	3527,23	3527,23	3527,94
857836	395764	3554	3527,81	3527,22	3527,2	3527,2	3527,2	3527,83
857866	395734	3538,67	3527,73	3527,2	3527,19	3527,19	3527,19	3527,75
857896	395704	3579,33	3527,61	3527,15	3527,13	3527,13	3527,13	3527,63
857926	395674	3577,33	3527,51	3527,09	3527,08	3527,08	3527,08	3527,52
857956	395644	3512	3527,44	3527,08	3527,07	3527,07	3527,07	3527,45
857986	395614	3677,33	3527,25	3526,92	3526,91	3526,91	3526,91	3527,26
858016	395584	3524,67	3527,2	3526,92	3526,91	3526,91	3526,91	3527,21
858046	395554	3500	3527,14	3526,89	3526,88	3526,88	3526,88	3527,15
858076	395524	3508,67	3527,09	3526,87	3526,86	3526,86	3526,86	3527,1
858106	395494	3478,67	3527,02	3526,82	3526,82	3526,82	3526,82	3527,02
858136	395464	3546,67	3526,97	3526,8	3526,79	3526,79	3526,79	3526,98
858166	395434	3520	3526,94	3526,79	3526,79	3526,79	3526,79	3526,95
858196	395404	3468,67	3526,87	3526,73	3526,73	3526,73	3526,73	3526,87
858226	395374	3492,67	3526,81	3526,7	3526,69	3526,69	3526,69	3526,82
858256	395344	3574,67	3526,75	3526,65	3526,65	3526,65	3526,65	3526,75
858286	395314	3552	3526,71	3526,62	3526,62	3526,62	3526,62	3526,71
858316	395284	3579,33	3526,65	3526,57	3526,57	3526,57	3526,57	3526,65
858346	395254	3586,67	3526,57	3526,51	3526,5	3526,5	3526,5	3526,58
858376	395224	3444,67	3526,48	3526,42	3526,42	3526,42	3526,42	3526,48
858406	395194	3492	3526,44	3526,39	3526,39	3526,39	3526,39	3526,44
858436	395164	3498	3526,4	3526,36	3526,36	3526,36	3526,36	3526,4
858466	395134	3590	3526,33	3526,29	3526,29	3526,29	3526,29	3526,33
858496	395104	3526,67	3526,33	3526,29	3526,29	3526,29	3526,29	3526,33
858526	395074	3552,67	3526,29	3526,26	3526,26	3526,26	3526,26	3526,3
858556	395044	3648	3526,17	3526,14	3526,14	3526,14	3526,14	3526,17
858586	395014	3538	3526,15	3526,13	3526,13	3526,13	3526,13	3526,15
858616	394984	3552	3526,12	3526,1	3526,1	3526,1	3526,1	3526,12
858646	394954	3548,67	3526,1	3526,08	3526,08	3526,08	3526,08	3526,1
858676	394924	3647,33	3525,97	3525,95	3525,95	3525,95	3525,95	3525,97
858706	394894	3538,67	3525,95	3525,94	3525,94	3525,94	3525,94	3525,95
858736	394864	3496,67	3525,92	3525,91	3525,91	3525,91	3525,91	3525,92
858766	394834	3514	3525,91	3525,9	3525,9	3525,9	3525,9	3525,91
858796	394804	3575,33	3525,86	3525,85	3525,85	3525,85	3525,85	3525,86
858826	394774	3514,67	3525,84	3525,84	3525,84	3525,84	3525,84	3525,84
858856	394744	3586	3525,78	3525,78	3525,78	3525,78	3525,78	3525,78
858886	394714	3510,67	3525,77	3525,76	3525,76	3525,76	3525,76	3525,77
858916	394684	3503,33	3525,74	3525,74	3525,74	3525,74	3525,74	3525,74
858946	394654	3590,67	3525,68	3525,67	3525,67	3525,67	3525,67	3525,68
858976	394624	3536	3525,66	3525,66	3525,66	3525,66	3525,66	3525,66
859006	394594	3610	3525,58	3525,57	3525,57	3525,57	3525,57	3525,58
859036	394564	3630	3525,47	3525,47	3525,47	3525,47	3525,47	3525,47
859066	394534	3600,67	3525,39	3525,39	3525,39	3525,39	3525,39	3525,39
859096	394504	3579,33	3525,34	3525,34	3525,34	3525,34	3525,34	3525,34
859126	394474	3548,67	3525,31	3525,31	3525,31	3525,31	3525,31	3525,31
859156	394444	3589,33	3525,25	3525,25	3525,25	3525,25	3525,25	3525,25

Please do not adjust margins

859186	394414	3489,33	3525,21	3525,21	3525,21	3525,21	3525,21	3525,21
859216	394384	3551,33	3525,18	3525,18	3525,18	3525,18	3525,18	3525,18
859246	394354	3537,33	3525,17	3525,17	3525,17	3525,17	3525,17	3525,17
859276	394324	3454	3525,1	3525,1	3525,1	3525,1	3525,1	3525,1
859306	394294	3584	3525,04	3525,04	3525,04	3525,04	3525,04	3525,04
859336	394264	3524	3525,04	3525,04	3525,04	3525,04	3525,04	3525,04
859366	394234	3430	3524,94	3524,94	3524,94	3524,94	3524,94	3524,94
859396	394204	3606,67	3524,86	3524,86	3524,86	3524,86	3524,86	3524,86
859426	394174	3628,67	3524,75	3524,75	3524,75	3524,75	3524,75	3524,75
859456	394144	3494,67	3524,72	3524,72	3524,72	3524,72	3524,72	3524,72
859486	394114	3505,33	3524,7	3524,7	3524,7	3524,7	3524,7	3524,7
859516	394084	3577,33	3524,65	3524,65	3524,65	3524,65	3524,65	3524,65
859546	394054	3460,67	3524,58	3524,58	3524,58	3524,58	3524,58	3524,58
859576	394024	3488	3524,54	3524,54	3524,54	3524,54	3524,54	3524,54
859606	393994	3536,67	3524,53	3524,53	3524,53	3524,53	3524,53	3524,53
859636	393964	3566	3524,49	3524,49	3524,49	3524,49	3524,49	3524,49
859666	393934	3545,33	3524,47	3524,47	3524,47	3524,47	3524,47	3524,47
859696	393904	3592,67	3524,4	3524,4	3524,4	3524,4	3524,4	3524,4
859726	393874	3508,67	3524,38	3524,38	3524,38	3524,38	3524,38	3524,38
859756	393844	3465,33	3524,32	3524,32	3524,32	3524,32	3524,32	3524,32
859786	393814	3413,33	3524,21	3524,21	3524,21	3524,21	3524,21	3524,21
859816	393784	3549,33	3524,18	3524,18	3524,18	3524,18	3524,18	3524,18
859846	393754	3560	3524,15	3524,15	3524,15	3524,15	3524,15	3524,15
859876	393724	3479,33	3524,1	3524,1	3524,1	3524,1	3524,1	3524,1
859906	393694	3602	3524,02	3524,02	3524,02	3524,02	3524,02	3524,02
859936	393664	3499,33	3524	3524	3524	3524	3524	3524
859966	393634	3548	3523,97	3523,97	3523,97	3523,97	3523,97	3523,97
859996	393604	3547,33	3523,95	3523,95	3523,95	3523,95	3523,95	3523,95
860026	393574	3577,33	3523,89	3523,89	3523,89	3523,89	3523,89	3523,89
860056	393544	3460	3523,83	3523,83	3523,83	3523,83	3523,83	3523,83
860086	393514	3514,67	3523,82	3523,82	3523,82	3523,82	3523,82	3523,82
860116	393484	3571,33	3523,77	3523,77	3523,77	3523,77	3523,77	3523,77
860146	393454	3563,33	3523,73	3523,73	3523,73	3523,73	3523,73	3523,73
860176	393424	3605,33	3523,65	3523,65	3523,65	3523,65	3523,65	3523,65
860206	393394	3551,33	3523,62	3523,62	3523,62	3523,62	3523,62	3523,62
860236	393364	3519,33	3523,61	3523,61	3523,61	3523,61	3523,61	3523,61
860266	393334	3536,67	3523,6	3523,6	3523,6	3523,6	3523,6	3523,6
860296	393304	3570	3523,55	3523,55	3523,55	3523,55	3523,55	3523,55
860326	393274	3533,33	3523,54	3523,54	3523,54	3523,54	3523,54	3523,54
860356	393244	3558,67	3523,51	3523,51	3523,51	3523,51	3523,51	3523,51
860386	393214	3536,67	3523,49	3523,49	3523,49	3523,49	3523,49	3523,49
860416	393184	3528	3523,49	3523,49	3523,49	3523,49	3523,49	3523,49
860446	393154	3558	3523,45	3523,45	3523,45	3523,45	3523,45	3523,45
860476	393124	3576	3523,4	3523,4	3523,4	3523,4	3523,4	3523,4
860506	393094	3560	3523,36	3523,36	3523,36	3523,36	3523,36	3523,36
860536	393064	3546,67	3523,34	3523,34	3523,34	3523,34	3523,34	3523,34
860566	393034	3516,67	3523,33	3523,33	3523,33	3523,33	3523,33	3523,33

 This journal is © The Royal Society of Chemistry 20xx

860596	393004	3523,33	3523,33	3523,33	3523,33	3523,33	3523,33	3523,33
860626	392974	3573,33	3573,33	3573,33	3573,33	3573,33	3573,33	3573,33
860656	392944	3532,67	3532,67	3532,67	3532,67	3532,67	3532,67	3532,67
860686	392914	3482	3482	3482	3482	3482	3482	3482
860716	392884	3571,33	3571,33	3571,33	3571,33	3571,33	3571,33	3571,33
860746	392854	3583,33	3583,33	3583,33	3583,33	3583,33	3583,33	3583,33
860776	392824	3549,33	3549,33	3549,33	3549,33	3549,33	3549,33	3549,33
860806	392794	3530,67	3530,67	3530,67	3530,67	3530,67	3530,67	3530,67
860836	392764	3596	3596	3596	3596	3596	3596	3596
860866	392734	3539,33	3539,33	3539,33	3539,33	3539,33	3539,33	3539,33
860896	392704	3603,33	3603,33	3603,33	3603,33	3603,33	3603,33	3603,33
860926	392674	3656	3656	3656	3656	3656	3656	3656
860956	392644	3553,33	3553,33	3553,33	3553,33	3553,33	3553,33	3553,33
860986	392614	3573,33	3573,33	3573,33	3573,33	3573,33	3573,33	3573,33
861016	392584	3658	3658	3658	3658	3658	3658	3658
861046	392554	3688,67	3688,67	3688,67	3688,67	3688,67	3688,67	3688,67
861076	392524	3675,33	3675,33	3675,33	3675,33	3675,33	3675,33	3675,33
861106	392494	3593,33	3593,33	3593,33	3593,33	3593,33	3593,33	3593,33
861136	392464	3604	3604	3604	3604	3604	3604	3604
861166	392434	3624	3624	3624	3624	3624	3624	3624
861196	392404	3572,67	3572,67	3572,67	3572,67	3572,67	3572,67	3572,67
861226	392374	3575,33	3575,33	3575,33	3575,33	3575,33	3575,33	3575,33
861256	392344	3590	3590	3590	3590	3590	3590	3590
861286	392314	3650,67	3650,67	3650,67	3650,67	3650,67	3650,67	3650,67
861316	392284	3571,33	3571,33	3571,33	3571,33	3571,33	3571,33	3571,33
861346	392254	3610	3610	3610	3610	3610	3610	3610
861376	392224	3638	3638	3638	3638	3638	3638	3638
861406	392194	3634	3634	3634	3634	3634	3634	3634
861436	392164	3630,67	3630,67	3630,67	3630,67	3630,67	3630,67	3630,67
861466	392134	3580	3580	3580	3580	3580	3580	3580
861496	392104	3665,33	3665,33	3665,33	3665,33	3665,33	3665,33	3665,33
861526	392074	3566,67	3566,67	3566,67	3566,67	3566,67	3566,67	3566,67
861556	392044	3639,33	3639,33	3639,33	3639,33	3639,33	3639,33	3639,33
861586	392014	3642	3642	3642	3642	3642	3642	3642
861616	391984	3662,67	3662,67	3662,67	3662,67	3662,67	3662,67	3662,67
861646	391954	3610,67	3610,67	3610,67	3610,67	3610,67	3610,67	3610,67
861676	391924	3590,67	3590,67	3590,67	3590,67	3590,67	3590,67	3590,67
861706	391894	3661,33	3661,33	3661,33	3661,33	3661,33	3661,33	3661,33
861736	391864	3666	3666	3666	3666	3666	3666	3666
861766	391834	3601,33	3601,33	3601,33	3601,33	3601,33	3601,33	3601,33
861796	391804	3672,67	3672,67	3672,67	3672,67	3672,67	3672,67	3672,67
861826	391774	3654	3654	3654	3654	3654	3654	3654
861856	391744	3653,33	3653,33	3653,33	3653,33	3653,33	3653,33	3653,33
861886	391714	3650,67	3650,67	3650,67	3650,67	3650,67	3650,67	3650,67
861916	391684	3688,67	3688,67	3688,67	3688,67	3688,67	3688,67	3688,67
861946	391654	3678	3678	3678	3678	3678	3678	3678
861976	391624	3617,33	3617,33	3617,33	3617,33	3617,33	3617,33	3617,33

This journal is © The Royal Society of Chemistry 20xx

Please do not adjust margins

862006	391594	3672	3672	3672	3672	3672	3672	3672
862036	391564	3694	3694	3694	3694	3694	3694	3694
862066	391534	3658	3658	3658	3658	3658	3658	3658
862096	391504	3718,67	3718,67	3718,67	3718,67	3718,67	3718,67	3718,67
862126	391474	3613,33	3613,33	3613,33	3613,33	3613,33	3613,33	3613,33
862156	391444	3706,67	3706,67	3706,67	3706,67	3706,67	3706,67	3706,67
862186	391414	3709,33	3709,33	3709,33	3709,33	3709,33	3709,33	3709,33
862216	391384	3694	3694	3694	3694	3694	3694	3694
862246	391354	3751,33	3751,33	3751,33	3751,33	3751,33	3751,33	3751,33
862276	391324	3644	3644	3644	3644	3644	3644	3644
862306	391294	3696,67	3696,67	3696,67	3696,67	3696,67	3696,67	3696,67
862336	391264	3626	3626	3626	3626	3626	3626	3626
862366	391234	3650	3650	3650	3650	3650	3650	3650
862396	391204	3714,67	3714,67	3714,67	3714,67	3714,67	3714,67	3714,67
862426	391174	3587,33	3587,33	3587,33	3587,33	3587,33	3587,33	3587,33
862456	391144	3627,33	3627,33	3627,33	3627,33	3627,33	3627,33	3627,33
862486	391114	3716	3716	3716	3716	3716	3716	3716
862516	391084	3720	3720	3720	3720	3720	3720	3720
862546	391054	3646,67	3646,67	3646,67	3646,67	3646,67	3646,67	3646,67
862576	391024	3702,67	3702,67	3702,67	3702,67	3702,67	3702,67	3702,67
862606	390994	3689,33	3689,33	3689,33	3689,33	3689,33	3689,33	3689,33
862636	390964	3703,33	3703,33	3703,33	3703,33	3703,33	3703,33	3703,33
862666	390934	3712,67	3712,67	3712,67	3712,67	3712,67	3712,67	3712,67
862696	390904	3714,67	3714,67	3714,67	3714,67	3714,67	3714,67	3714,67
862726	390874	3674	3674	3674	3674	3674	3674	3674
862756	390844	3678,67	3678,67	3678,67	3678,67	3678,67	3678,67	3678,67
862786	390814	3584,67	3584,67	3584,67	3584,67	3584,67	3584,67	3584,67
862816	390784	3708,67	3708,67	3708,67	3708,67	3708,67	3708,67	3708,67
862846	390754	3679,33	3679,33	3679,33	3679,33	3679,33	3679,33	3679,33
862876	390724	3584,67	3584,67	3584,67	3584,67	3584,67	3584,67	3584,67
862906	390694	3639,33	3639,33	3639,33	3639,33	3639,33	3639,33	3639,33
862936	390664	3578	3578	3578	3578	3578	3578	3578
862966	390634	3634	3634	3634	3634	3634	3634	3634
862996	390604	3646,67	3646,67	3646,67	3646,67	3646,67	3646,67	3646,67
863026	390574	3726,67	3726,67	3726,67	3726,67	3726,67	3726,67	3726,67
863056	390544	3650,67	3650,67	3650,67	3650,67	3650,67	3650,67	3650,67
863086	390514	3576	3576	3576	3576	3576	3576	3576
863116	390484	3587,33	3587,33	3587,33	3587,33	3587,33	3587,33	3587,33
863146	390454	3695,33	3695,33	3695,33	3695,33	3695,33	3695,33	3695,33
863176	390424	3604,67	3604,67	3604,67	3604,67	3604,67	3604,67	3604,67
863206	390394	3658,67	3658,67	3658,67	3658,67	3658,67	3658,67	3658,67
863236	390364	3683,33	3683,33	3683,33	3683,33	3683,33	3683,33	3683,33
863266	390334	3680,67	3680,67	3680,67	3680,67	3680,67	3680,67	3680,67
863296	390304	3555,33	3555,33	3555,33	3555,33	3555,33	3555,33	3555,33
863326	390274	3691,33	3691,33	3691,33	3691,33	3691,33	3691,33	3691,33
863356	390244	3596,67	3596,67	3596,67	3596,67	3596,67	3596,67	3596,67
863386	390214	3569,33	3569,33	3569,33	3569,33	3569,33	3569,33	3569,33

This journal is © The Royal Society of Chemistry 20xx

Please do not adjust margins

863416	390184	3640	3640	3640	3640	3640	3640	3640
863446	390154	3644	3644	3644	3644	3644	3644	3644
863476	390124	3620	3620	3620	3620	3620	3620	3620
863506	390094	3530	3530	3530	3530	3530	3530	3530
863536	390064	3596	3596	3596	3596	3596	3596	3596
863566	390034	3679,33	3679,33	3679,33	3679,33	3679,33	3679,33	3679,33
863596	390004	3717,33	3717,33	3717,33	3717,33	3717,33	3717,33	3717,33
863626	389974	3596,67	3596,67	3596,67	3596,67	3596,67	3596,67	3596,67
863656	389944	3631,33	3631,33	3631,33	3631,33	3631,33	3631,33	3631,33
863686	389914	3569,33	3569,33	3569,33	3569,33	3569,33	3569,33	3569,33
863716	389884	3664,67	3664,67	3664,67	3664,67	3664,67	3664,67	3664,67
863746	389854	3544	3544	3544	3544	3544	3544	3544
863776	389824	3630	3630	3630	3630	3630	3630	3630
863806	389794	3512,67	3512,67	3512,67	3512,67	3512,67	3512,67	3512,67
863836	389764	3574,67	3574,67	3574,67	3574,67	3574,67	3574,67	3574,67
863866	389734	3652,67	3652,67	3652,67	3652,67	3652,67	3652,67	3652,67
863896	389704	3656	3656	3656	3656	3656	3656	3656
863926	389674	3562,67	3562,67	3562,67	3562,67	3562,67	3562,67	3562,67
863956	389644	3594	3594	3594	3594	3594	3594	3594
863986	389614	3575,33	3575,33	3575,33	3575,33	3575,33	3575,33	3575,33
864016	389584	3489,33	3489,33	3489,33	3489,33	3489,33	3489,33	3489,33
864046	389554	3632,67	3632,67	3632,67	3632,67	3632,67	3632,67	3632,67
864076	389524	3625,33	3625,33	3625,33	3625,33	3625,33	3625,33	3625,33
864106	389494	3621,33	3621,33	3621,33	3621,33	3621,33	3621,33	3621,33
864136	389464	3546,67	3546,67	3546,67	3546,67	3546,67	3546,67	3546,67
864166	389434	3576,67	3576,67	3576,67	3576,67	3576,67	3576,67	3576,67
864196	389404	3555,33	3555,33	3555,33	3555,33	3555,33	3555,33	3555,33
864226	389374	3543,33	3543,33	3543,33	3543,33	3543,33	3543,33	3543,33
864256	389344	3558	3558	3558	3558	3558	3558	3558
864286	389314	3516	3516	3516	3516	3516	3516	3516
864316	389284	3587,33	3587,33	3587,33	3587,33	3587,33	3587,33	3587,33
864346	389254	3502,67	3502,67	3502,67	3502,67	3502,67	3502,67	3502,67
864376	389224	3654,67	3654,67	3654,67	3654,67	3654,67	3654,67	3654,67
864406	389194	3541,33	3541,33	3541,33	3541,33	3541,33	3541,33	3541,33
864436	389164	3532,67	3532,67	3532,67	3532,67	3532,67	3532,67	3532,67
864466	389134	3546,67	3546,67	3546,67	3546,67	3546,67	3546,67	3546,67
864496	389104	3426,67	3426,67	3426,67	3426,67	3426,67	3426,67	3426,67
864526	389074	3546	3546	3546	3546	3546	3546	3546
864556	389044	3605,33	3605,33	3605,33	3605,33	3605,33	3605,33	3605,33
864586	389014	3497,33	3497,33	3497,33	3497,33	3497,33	3497,33	3497,33
864616	388984	3482	3482	3482	3482	3482	3482	3482
864646	388954	3572	3572	3572	3572	3572	3572	3572
864676	388924	3540	3540	3540	3540	3540	3540	3540
864706	388894	3510	3510	3510	3510	3510	3510	3510
864736	388864	3588	3588	3588	3588	3588	3588	3588
864766	388834	3581,33	3581,33	3581,33	3581,33	3581,33	3581,33	3581,33
864796	388804	3447,33	3447,33	3447,33	3447,33	3447,33	3447,33	3447,33

This journal is © The Royal Society of Chemistry 20xx

864826	388774	3611,33	3611,33	3611,33	3611,33	3611,33	3611,33	3611,33
864856	388744	3554,67	3554,67	3554,67	3554,67	3554,67	3554,67	3554,67
864886	388714	3486	3486	3486	3486	3486	3486	3486
864916	388684	3499,33	3499,33	3499,33	3499,33	3499,33	3499,33	3499,33
864946	388654	3425,33	3425,33	3425,33	3425,33	3425,33	3425,33	3425,33
864976	388624	3480	3480	3480	3480	3480	3480	3480
865006	388594	3488	3488	3488	3488	3488	3488	3488
865036	388564	3480	3480	3480	3480	3480	3480	3480
865066	388534	3448,67	3448,67	3448,67	3448,67	3448,67	3448,67	3448,67
865096	388504	3559,33	3559,33	3559,33	3559,33	3559,33	3559,33	3559,33
865126	388474	3478	3478	3478	3478	3478	3478	3478
865156	388444	3460	3460	3460	3460	3460	3460	3460
865186	388414	3452	3452	3452	3452	3452	3452	3452
865216	388384	3460,67	3460,67	3460,67	3460,67	3460,67	3460,67	3460,67
865246	388354	3502	3502	3502	3502	3502	3502	3502
865276	388324	3467,33	3467,33	3467,33	3467,33	3467,33	3467,33	3467,33
865306	388294	3409,33	3409,33	3409,33	3409,33	3409,33	3409,33	3409,33
865336	388264	3490,67	3490,67	3490,67	3490,67	3490,67	3490,67	3490,67
865366	388234	3443,33	3443,33	3443,33	3443,33	3443,33	3443,33	3443,33
865396	388204	3373,33	3373,33	3373,33	3373,33	3373,33	3373,33	3373,33
865426	388174	3413,33	3413,33	3413,33	3413,33	3413,33	3413,33	3413,33

Table S9 Raw excitation–emission matrix (EEM) measurements for dW-CDs in Fig. 3.a.

This journal is © The Royal Society of Chemistry 20xx

Longitud em (nm)	Longitud exc (nm)	Watermelon (u.a.)	Doped watermelon (u.a.)	Blueberry (u.a.)	Doped blueberry (u.a.)	Strawberry (u.a.)	Doped strawberry (u.a.)
347,741211	300	52,5453463	794,544979	66,2395067	174,354652	87,9172515	122,885248
349,808533	300	57,6948902	857,825917	69,6121848	183,584217	99,9689711	120,451454
351,874329	300	65,4364067	896,237234	71,941473	195,228293	109,182186	133,296301
353,938568	300	66,4437778	918,107906	71,9546365	208,51703	124,13543	154,232886
356,001343	300	67,1407524	974,47636	79,3937967	221,135699	119,41901	166,212381
358,062561	300	74,0964142	1036,79911	81,1611222	214,382815	126,451541	192,680342
360,122253	300	76,1979846	1081,46798	81,4157575	231,443156	133,540333	206,254634
362,180481	300	88,3752036	1148,63438	100,84833	233,127834	152,968225	213,420878
364,237152	300	97,9586944	1149,19455	98,1723599	239,748353	156,865366	245,376219
366,292328	300	97,9426202	1224,64695	112,402818	256,495534	170,081489	270,429296
368,345978	300	108,157054	1271,40159	111,539886	262,205944	189,028315	308,235477
370,398132	300	109,364453	1312,19329	120,825932	274,436828	195,91868	322,085397
372,44873	300	126,090341	1377,2753	120,714943	290,886998	212,923376	354,36747
374,497803	300	128,695718	1428,76274	134,630239	316,894279	222,291196	382,574643
376,545349	300	141,093097	1463,32156	138,365727	327,669787	243,016321	409,888671
378,5914	300	144,262978	1488,05866	150,739308	325,657134	243,928331	451,780731
380,635925	300	156,564885	1541,65861	148,026155	349,70611	246,702034	480,397196
382,678955	300	166,011709	1573,81051	160,549293	365,549079	261,593153	508,610834
384,720398	300	170,854218	1612,66251	171,392063	387,682266	281,506534	548,828045
386,760376	300	176,568146	1655,52532	176,619413	396,269467	288,037745	575,894422
388,798767	300	172,763271	1688,42286	177,646028	406,05917	287,156553	594,021293
390,835663	300	182,490567	1696,20461	185,714042	414,18709	298,565742	606,466612
392,871033	300	189,92277	1758,13871	194,971337	429,130818	309,61666	637,216575
394,904877	300	200,167623	1752,5808	210,382481	436,531713	310,648323	659,197349
396,937195	300	214,216781	1806,02587	214,923459	455,914167	319,881023	685,431872
398,967957	300	219,635655	1816,85676	209,410784	453,97062	338,340643	692,506192
400,997253	300	225,635977	1865,07248	218,320205	474,145307	332,758085	704,585916
403,024963	300	229,543972	1857,96607	222,501108	488,846629	341,060166	717,203809
405,051147	300	243,406281	1915,04666	231,96205	479,498765	336,522722	729,200296
407,075806	300	249,161945	1921,30887	234,844362	495,171201	349,94478	739,80026
409,098938	300	251,986365	1927,15793	238,976308	503,351642	349,319847	742,587766
411,120544	300	263,553998	1935,97622	248,957599	516,751524	364,316584	730,164719
413,140564	300	273,147451	1970,25343	252,909491	509,986668	378,132555	735,822695
415,159119	300	278,869735	1962,05618	260,945541	518,652502	377,678674	741,922149
417,176086	300	290,245166	1952,30864	259,948667	526,753698	379,665474	732,430869
419,191528	300	304,578973	1929,78106	269,01098	522,196695	382,220698	723,115589
421,205444	300	298,057849	1935,79183	259,021013	504,916188	371,351815	718,28706
423,217834	300	310,53176	1929,24108	278,4427	505,121642	377,192879	697,268334
425,228668	300	321,901915	1920,85286	276,810238	502,292464	379,547466	705,456764
427,237976	300	325,213295	1880,23161	277,774981	505,499613	389,596478	697,73395
429,245728	300	322,088935	1866,92099	284,425687	491,050735	379,729733	674,950937
431,251953	300	337,198042	1871,29898	283,998988	494,26861	390,936804	648,172981
433,256592	300	332,325928	1862,44129	290,861483	510,033651	385,713001	662,493415
435,259766	300	352,596877	1872,60747	296,813361	486,733679	386,938503	640,853623
437,261353	300	347,671902	1868,16111	288,68028	483,144998	387,259984	637,429993
439,261383	300	353,194479	1849,85533	298,081498	488,885139	393,541891	625,838957

This journal is © The Royal Society of Chemistry 20xx

Please do not adjust margins

441,259888	300	359,082916	1812,88399	313,431333	479,474145	390,180863	616,185068
443,256836	300	367,153346	1772,32096	299,069636	463,180576	392,027654	587,156123
445,252258	300	364,807158	1753,12357	299,754989	466,949118	390,873839	575,829163
447,246094	300	379,916722	1725,27483	301,646543	463,881945	388,907067	574,025043
449,238434	300	369,781387	1669,04419	296,910155	443,456038	391,626865	544,4545
451,229187	300	373,950301	1651,84154	306,321299	456,67577	380,408457	545,323921
453,218445	300	380,666199	1630,22949	299,692589	438,296141	387,885319	523,859195
455,206116	300	376,343367	1595,29455	290,2451	428,870912	374,49032	502,46343
457,192261	300	368,252734	1589,4293	300,078064	432,845447	394,611729	497,142308
459,176819	300	375,825278	1557,10686	296,523233	421,655685	379,265732	472,911776
461,15979	300	379,700196	1522,24488	290,522708	420,188231	391,556148	460,862255
463,141296	300	393,154773	1500,79208	300,22732	420,357409	389,164905	459,241841
465,121216	300	383,349571	1471,14088	299,211958	403,547641	394,992245	444,292112
467,099579	300	386,137446	1431,23357	302,2944	400,072658	391,8934	440,048089
469,076385	300	374,023278	1397,53344	296,71253	391,421709	389,660532	433,510697
471,051636	300	393,160509	1391,08115	296,676813	382,854913	391,85397	403,526385
473,02536	300	388,573396	1357,97436	289,87058	384,132889	400,740551	398,46371
474,997498	300	382,793978	1336,41336	292,606112	377,191238	393,644754	382,787629
476,968079	300	394,79053	1324,63457	291,622307	385,997475	393,769396	377,706748
478,937134	300	397,941268	1301,30205	289,155482	356,360132	389,139906	370,255615
480,904572	300	393,259235	1259,46965	291,861821	362,250734	398,926119	346,267537
482,870453	300	393,167886	1236,98405	295,139034	356,000931	389,621083	346,139632
484,834808	300	390,169891	1222,10312	295,30367	346,567647	398,333973	336,162895
486,797607	300	399,356298	1185,8329	284,442174	356,4674	403,312525	326,21131
488,75885	300	396,361296	1164,99781	292,738174	340,767224	395,607079	315,387542
490,718506	300	399,312687	1158,86948	287,428272	332,033638	403,234556	315,333704
492,676605	300	394,760668	1111,92527	280,209517	330,295818	388,901983	297,381534
494,633118	300	393,531178	1102,27773	286,441572	321,779264	389,583631	290,475427
496,588104	300	393,210056	1081,253	283,887704	327,008285	392,599236	287,185238
498,541504	300	391,495749	1038,4961	282,113653	307,613085	388,15369	273,918142
500,493347	300	389,885664	1004,62443	279,657123	306,130363	386,418505	259,403428
502,443665	300	382,48554	984,858797	279,523004	302,447463	377,922852	255,963758
504,392365	300	386,806437	963,85471	265,297003	292,295705	379,392709	247,072785
506,339539	300	371,777134	931,657211	275,211106	274,10513	375,033479	238,270762
508,285095	300	382,554156	903,09772	268,990681	285,397918	367,118611	232,700226
510,229126	300	377,653901	897,651937	257,45779	268,588795	370,129958	226,777759
512,17157	300	366,267752	852,407087	255,237312	261,220249	363,929471	209,028551
514,112427	300	369,377209	832,241373	260,493442	259,966789	350,377145	208,6287
516,051697	300	359,249665	804,002278	254,007239	250,072343	353,949518	202,44305
517,989441	300	357,274309	777,854975	240,156568	240,323724	343,305781	193,218406
519,925598	300	344,760541	744,423888	244,601763	238,828016	335,524216	184,401328
521,860168	300	341,409088	729,495365	235,891261	220,489681	337,058035	179,655825
523,793152	300	343,577041	701,069612	230,829276	220,847899	319,479799	170,365762
525,724609	300	338,086305	681,661836	232,4587	215,047062	323,125446	161,667551
527,654419	300	332,244116	673,758573	228,015887	207,36663	313,679881	162,278981
529,582764	300	327,129446	645,492853	225,781389	206,186082	302,435972	161,867705
531,50946	300	321,161598	623,772719	216,792464	201,57326	307,009722	154,767769

This journal is © The Royal Society of Chemistry 20xx

Please do not adjust margins

Please do not adjust margins

533,43457	300	324,704842	614,408342	216,067305	202,961059	303,939261	145,752118
535,358093	300	320,876051	598,696743	210,84691	192,300551	292,302086	139,833657
537,280029	300	315,260287	574,317508	205,704781	184,973685	291,483053	129,693094
539,200439	300	309,133312	553,538671	207,768368	184,05377	278,93048	127,855208
541,119263	300	298,835796	536,698176	199,632314	175,88168	287,660448	129,072673
543,036438	300	287,048166	526,608155	196,635703	173,676769	271,669533	119,304177
544,952087	300	292,953805	507,559366	192,05569	164,963489	265,966746	120,88707
546,866211	300	283,391379	480,115106	188,791228	156,686385	261,834746	106,664529
548,778687	300	278,468653	477,162922	187,71796	152,454347	261,54546	105,603202
550,689514	300	274,852611	455,438946	178,993936	152,604737	253,368855	102,942394
552,598816	300	265,31655	429,676153	175,118621	146,962028	253,108024	97,052249
554,506531	300	266,651944	422,001854	164,940242	145,587787	237,269563	103,678746
556,41272	300	263,599327	412,445011	167,764353	140,136317	234,219145	96,9197398
558,317261	300	253,726931	392,472243	162,91223	132,39869	233,701614	90,1210678
560,220215	300	242,302965	380,171379	164,118914	128,219418	226,916894	90,3965527
562,121582	300	240,418518	360,650961	162,873428	128,742785	215,548768	83,6325753
564,021362	300	233,555234	347,738509	156,605328	121,91195	215,926211	82,9405585
565,919556	300	236,794418	342,770126	152,191214	114,424352	203,074345	79,5122881
567,816162	300	224,444618	337,939662	149,458048	117,020005	208,716963	80,0131721
569,711182	300	219,848643	325,063153	143,431722	114,349242	195,976665	71,6553249
571,604614	300	225,804646	311,250759	139,66224	105,637037	201,001106	71,1778657
573,49646	300	220,00556	300,877717	143,292837	104,606445	189,662632	70,451305
575,386719	300	203,496458	295,284266	136,712723	100,875826	189,676857	65,2934152
577,27533	300	200,125942	279,956496	130,828994	102,237433	176,78987	65,3799322
579,162354	300	195,953914	274,399803	128,50344	97,6580131	176,772404	62,0586977
581,047852	300	193,338963	267,336796	127,12274	91,9297305	162,524198	60,1409833
582,931641	300	187,45999	252,053282	120,99174	89,4785097	165,110869	59,3265911
584,813965	300	182,733456	239,430953	119,254228	84,5202562	162,943099	57,9821533
586,69458	300	174,480445	227,933993	118,925911	87,2382227	153,301671	55,8348392
588,57373	300	171,100232	227,184072	112,656144	79,7059523	154,233706	51,4586091
590,451111	300	167,901505	214,479144	106,862681	77,0909367	150,487737	49,430288
592,326965	300	163,681515	207,967838	104,101562	76,6729718	136,700499	45,0733783
594,201233	300	157,835703	197,975207	103,15381	73,7908023	140,989035	47,4371262
596,073914	300	148,241501	194,84068	100,621362	70,3329138	136,738808	41,2405334
597,944946	300	152,400483	184,524305	98,676663	72,1222872	133,481186	44,0448619
599,814453	300	144,472552	181,949161	98,442481	68,680619	131,885344	40,8219872
601,682251	300	136,156623	173,844783	94,5206567	61,0002439	117,254661	35,0694844
603,548523	300	135,01194	165,670714	86,3873261	62,7096731	120,540197	37,6752946
605,413147	300	134,924833	163,690522	90,0580272	60,2009313	119,629562	34,51233
607,276245	300	125,086689	157,786163	84,8391853	57,9928502	111,40924	37,4135444
609,137695	300	125,031363	152,173767	79,3499615	53,81611	115,176462	35,5290869
610,997437	300	125,420683	144,695775	78,9422174	51,3818233	111,172107	30,9560396
612,855713	300	118,377873	138,522741	76,9931671	52,8126426	101,897842	30,8265552
614,712341	300	121,96675	134,356435	76,209316	48,7073885	100,757617	30,0628805
616,567322	300	115,409298	128,961513	73,454994	52,1614914	101,99906	27,5518889
618,420715	300	110,5911	124,457432	73,4210555	48,2635888	93,2110832	29,573421
620,272522	300	112,25442	122,869049	70,1615938	49,6464922	88,8596604	29,2109006

This journal is © The Royal Society of Chemistry 20xx

Please do not adjust margins

Please do not adjust margins

622,122681	300	104,338068	111,991653	66,7471521	43,7659013	84,0641843	27,2843313
623,971313	300	97,5913354	113,444085	67,2158708	43,5997928	89,5805278	23,7966401
625,818237	300	93,2219165	107,708479	62,0253348	38,4619039	82,0452615	25,3211739
627,663574	300	97,5715437	100,118795	56,2373517	43,9926521	78,7409712	23,2160493
629,507324	300	86,4483205	98,8747348	58,9714881	43,1980086	79,1288014	22,6103235
631,349365	300	91,4472851	100,251456	60,7506853	34,6995423	78,4646108	22,4828668
633,18988	300	84,5316536	91,6277282	60,1676366	34,7805713	71,2610248	20,8018781
635,028809	300	80,7004823	90,266761	59,4071376	34,1804194	72,9553947	20,5006822
636,865967	300	83,1593977	85,3233023	52,100429	33,219489	66,2575168	17,4967513
638,70166	300	78,687857	84,7884782	50,0961876	30,4800371	66,0038218	15,7702028
640,535645	300	76,6337237	79,1019904	51,1032442	33,9572318	65,2665155	16,9245398
642,368042	300	73,3711466	79,1973314	46,8041989	31,5282084	66,2164497	16,2207961
644,198853	300	71,1905307	68,8635798	49,3815356	27,5192955	58,0643934	17,4169192
646,028015	300	66,7360961	72,1567945	47,8345203	30,4666343	58,1316406	14,9668361
647,855591	300	66,4141715	69,6430228	43,294525	28,4133052	60,581771	17,2841764
649,681519	300	67,2110861	69,7224518	39,8625651	29,2979307	57,3924162	16,3545862
651,505798	300	61,5801652	65,5872064	37,0831791	26,6235281	50,5716316	17,008249
653,328491	300	62,3625013	66,0850588	37,7879228	27,4433177	52,3496174	14,7074795
655,149536	300	63,0349671	58,4609978	36,8881304	26,5064119	51,0597281	14,022844
656,968994	300	57,324223	58,255648	38,2306515	25,1290399	46,3666026	10,5235465
658,786743	300	55,9492243	59,8824495	34,3783238	25,1001755	48,8288751	12,5101163
660,602905	300	49,0152635	51,6210117	35,8082639	22,7635278	45,3523816	11,3455135
662,41748	300	53,1622033	54,1139312	33,561147	21,6623236	43,1505101	13,9456923
664,230469	300	52,6617958	46,9711439	31,2146442	22,7601755	46,2485929	10,3672205
666,041748	300	48,0581981	50,9908733	34,7462293	19,026592	38,937822	11,4398054
667,85144	300	47,2781885	49,6138031	32,7209432	19,3817348	41,3149103	10,112815
669,659424	300	43,4138652	49,5627643	27,4747527	20,1798286	42,7638138	9,83091119
671,46582	300	44,9831054	41,6996403	29,4781871	21,1117608	36,4319476	9,08396581
673,27063	300	42,5569605	40,4879705	28,9835135	16,1626999	37,793857	10,1835085
675,073792	300	41,5652707	37,6574369	27,3581028	14,2077893	33,5913711	9,59397747
676,875305	300	39,8705594	37,1911478	24,1439149	15,4270395	33,2796847	9,13538919
678,675171	300	42,3308585	35,9724638	24,9119212	16,4988619	32,3688734	7,3094728
680,47345	300	37,8541349	40,3807729	27,9472504	13,738557	32,6493	9,28203134
682,27002	300	35,3709374	33,9207413	23,7531962	14,0925245	31,8438667	7,63458722
684,065063	300	37,0314824	32,0315178	24,139659	14,446743	33,4857536	6,28116981
685,858398	300	37,973908	30,8130373	21,0737769	13,530539	27,6609072	6,43718958
687,650024	300	30,4402082	31,9231741	21,7106152	14,0057066	25,6886684	6,13674683
689,440186	300	33,4532132	28,441745	22,3781259	12,3411694	28,0607454	5,53583849
691,228577	300	33,1597656	26,909229	22,5435423	10,9581758	24,8351783	3,38467662
693,015381	300	29,6706623	28,2175686	16,8729879	11,6047924	26,1351085	8,32883879
694,800476	300	31,5269111	29,1405376	17,3330516	9,59089886	25,3555456	3,70078153
696,583984	300	27,9117937	24,7449265	16,4707056	10,7168227	21,4218775	3,4060751
698,365845	300	24,8628237	26,5311806	18,6984753	12,4602154	21,0150691	7,14180488
700,146118	300	27,1600485	21,936473	16,8958411	8,44423743	25,7876704	3,42928786
701,924683	300	26,4764733	22,6499722	16,175574	9,57553232	23,8472426	6,72846708
347,741211	305	53,4507376	745,323466	63,3131998	166,020174	95,2898848	120,139721
349,808533	305	61,9718104	833,196602	71,6625418	201,047195	103,183935	124,732617

 This journal is © The Royal Society of Chemistry 20xx

Please do not adjust margins

Please do not adjust margins

351,874329	305	67,9261724	882,936998	66,5925113	199,765995	109,089493	134,56093
353,938568	305	70,7618484	933,078313	79,5955785	213,394614	114,237759	150,344866
356,001343	305	68,4837842	994,524475	80,2289597	227,674236	127,932129	171,131242
358,062561	305	86,682752	1062,49183	94,3724822	220,779117	143,323184	189,362188
360,122253	305	82,4397247	1130,41173	90,8337553	241,83661	144,193971	202,182107
362,180481	305	92,8203321	1161,47006	104,229563	243,802253	162,340939	235,183926
364,237152	305	99,2218905	1219,89062	117,951313	262,300564	177,900606	259,204554
366,292328	305	109,083637	1298,93342	122,087087	278,597724	199,232804	277,005633
368,345978	305	118,516916	1353,41414	124,708627	281,419897	197,583708	313,616345
370,398132	305	114,844637	1391,32239	131,725625	297,774508	211,488676	339,027687
372,44873	305	137,076978	1474,62771	142,994095	326,668922	223,379027	385,839324
374,497803	305	147,090442	1522,37161	145,247044	334,837964	253,987154	419,420673
376,545349	305	152,987898	1582,57508	161,355999	334,978438	253,722006	447,983046
378,5914	305	158,03291	1618,84075	167,93097	354,332991	267,331041	477,187795
380,635925	305	168,721569	1664,70304	182,9456	374,302024	270,741669	491,224221
382,678955	305	180,54513	1711,37775	180,689685	386,855653	289,237695	539,841689
384,720398	305	183,474712	1759,60007	191,035215	410,471147	308,681227	573,149524
386,760376	305	193,018261	1818,30519	202,71747	425,454861	318,44698	607,458342
388,798767	305	197,358369	1817,15604	213,205463	445,062429	329,220589	634,737578
390,835663	305	212,059839	1873,74145	210,262649	440,773673	332,131926	650,996343
392,871033	305	215,435871	1905,974	223,725545	463,305259	344,513998	688,810183
394,904877	305	219,373103	1917,09561	220,718253	468,542452	356,422379	705,657969
396,937195	305	232,832142	1965,81223	255,33891	472,402784	354,26363	736,719991
398,967957	305	236,073237	1925,6382	243,853177	488,76111	367,30939	747,1369
400,997253	305	251,343447	1964,8405	237,507349	497,011826	371,588943	758,569639
403,024963	305	256,149371	1995,01242	251,674874	511,940926	372,553568	766,803903
405,051147	305	265,052498	2006,92862	264,868963	512,348186	392,606292	771,957999
407,075806	305	275,01719	2026,38542	272,216049	524,667021	394,182539	773,176251
409,098938	305	287,99815	2021,50257	285,364949	529,464536	407,333702	785,46822
411,120544	305	286,616587	2034,07964	278,44299	541,091254	393,662009	781,949068
413,140564	305	313,441266	2033,43247	277,813518	538,724888	402,57081	783,353555
415,159119	305	307,228408	2035,65713	300,390865	531,379064	409,407781	785,646377
417,176086	305	313,90038	2014,49761	296,655511	531,889853	417,886287	760,961549
419,191528	305	321,430555	2001,40017	298,429806	529,57484	402,290826	766,244128
421,205444	305	333,203961	2005,82584	298,669456	527,468493	417,367007	763,417646
423,217834	305	334,079538	1972,05453	312,952414	530,391742	407,745997	745,674316
425,228668	305	351,005199	1962,45381	310,981568	523,581629	417,178384	743,881525
427,237976	305	352,037997	1942,13414	312,613556	529,11759	418,291668	731,274034
429,245728	305	352,114639	1912,98473	320,594609	516,844177	422,053509	716,852578
431,251953	305	366,363152	1931,11643	328,591966	513,340625	430,864719	705,262906
433,256592	305	366,626927	1897,43197	329,74653	501,015117	407,979405	695,202276
435,259766	305	367,12673	1884,73972	326,901392	503,975494	429,365018	682,605
437,261353	305	382,119433	1886,24189	331,422488	499,654757	418,188122	675,027179
439,261383	305	395,056748	1864,5192	337,466704	505,386664	414,692323	657,72824
441,259888	305	391,411738	1814,6729	332,169017	498,149575	431,909825	637,677409
443,256836	305	395,525594	1805,17593	335,332121	483,161217	418,872641	627,459637
445,252258	305	386,982605	1772,4342	335,664276	474,612671	421,080583	603,605631

This journal is © The Royal Society of Chemistry 20xx

Please do not adjust margins

Please do not adjust margins

447,246094	305	390,98969	1746,11235	335,139215	468,515426	421,572804	606,613668
449,238434	305	392,405266	1701,9661	320,214189	459,670876	413,259928	566,994039
451,229187	305	397,225993	1660,17197	321,575044	457,89194	418,471961	546,244862
453,218445	305	396,978056	1649,7204	325,613808	439,104216	416,041228	533,355165
455,206116	305	388,060033	1603,18272	325,044434	433,635498	420,057172	526,267513
457,192261	305	396,663731	1564,99159	333,262396	431,953446	406,067891	508,467844
459,176819	305	389,826587	1561,47112	331,060252	425,719778	415,102568	506,848448
461,15979	305	408,063973	1526,0148	324,76055	414,347811	405,717401	482,347299
463,141296	305	396,842888	1494,01106	320,798221	411,727101	419,93608	472,439992
465,121216	305	406,708496	1456,8762	323,719853	408,627307	414,254664	448,815176
467,099579	305	408,651284	1436,81184	322,474875	396,2357	412,921764	439,36109
469,076385	305	400,379647	1391,36788	310,966818	390,400527	405,194141	434,144456
471,051636	305	408,504651	1367,59905	313,265071	390,539725	406,806315	422,074258
473,02536	305	409,509257	1357,45827	321,345061	379,018842	411,393489	398,498647
474,997498	305	413,753783	1312,21948	321,865593	380,278204	419,440864	400,403467
476,968079	305	405,580654	1315,6857	313,081253	366,93836	409,550413	383,487745
478,937134	305	416,836161	1278,46951	305,789605	371,138263	414,746529	366,359424
480,904572	305	414,119645	1258,66337	317,916979	358,84402	422,061148	365,76707
482,870453	305	414,333054	1240,08876	317,120487	355,169148	402,813934	360,288335
484,834808	305	406,033252	1205,36019	319,129645	348,657842	416,855813	351,85785
486,797607	305	419,55739	1178,96637	315,4395	345,542344	404,369258	333,204155
488,75885	305	411,444237	1139,08208	318,414096	333,407542	409,112962	321,307303
490,718506	305	415,525576	1130,69187	318,612468	339,488391	408,114943	315,762471
492,676605	305	413,670132	1102,31466	307,815053	317,520884	402,60751	298,339013
494,633118	305	405,388897	1076,16042	306,515026	312,509677	406,618839	286,517402
496,588104	305	409,237704	1037,46952	310,018977	312,744792	404,636684	285,390199
498,541504	305	400,696739	1005,74696	293,629924	310,593198	398,009684	268,752742
500,493347	305	396,279333	1001,17103	301,017015	299,558619	397,893252	258,9649
502,443665	305	398,005092	970,533341	299,493908	288,99516	396,545531	259,642646
504,392365	305	399,555656	939,106234	293,423501	284,134469	387,603103	245,999336
506,339539	305	396,315424	913,734225	285,220673	274,678362	388,805334	237,39294
508,285095	305	389,687066	890,73223	283,696232	268,10723	381,569684	225,838818
510,229126	305	380,146512	855,99177	280,939773	259,77543	367,301074	221,781824
512,17157	305	376,209595	842,985918	274,897936	247,294889	370,263806	213,817163
514,112427	305	373,491873	803,890672	276,890021	254,862381	370,673562	202,431355
516,051697	305	366,87928	794,682145	266,096411	244,23577	354,374986	201,806619
517,989441	305	359,029564	748,666216	260,51884	241,934183	359,055127	191,732248
519,925598	305	351,589462	741,650149	249,531047	229,556062	346,695662	182,906821
521,860168	305	358,355968	712,626439	242,662939	225,550007	343,685629	174,413387
523,793152	305	345,045356	684,785882	254,285963	213,549195	333,084008	171,544555
525,724609	305	345,343004	667,94192	242,268714	213,08607	328,803361	168,839484
527,654419	305	340,706506	655,002138	240,792177	209,672368	322,638534	161,740975
529,582764	305	343,087248	643,864206	232,490575	201,585663	319,255821	154,14813
531,50946	305	327,201416	612,63667	234,024425	194,912918	307,089017	149,638759
533,43457	305	326,045248	605,711833	226,150938	191,457502	308,776877	144,972853
535,358093	305	325,407462	584,673656	218,703223	183,107773	308,567786	141,033724
537,280029	305	313,947941	553,935273	224,259487	182,593678	299,431403	134,862794

This journal is © The Royal Society of Chemistry 20xx

Please do not adjust margins

Please do not adjust margins

539,200439	305	310,151535	550,928898	217,158081	174,76423	293,358947	129,320893
541,119263	305	301,70388	532,322736	215,679056	171,213799	284,611637	130,172241
543,036438	305	296,817372	509,209932	209,661356	164,054222	276,240531	122,532064
544,952087	305	298,850202	495,42617	201,931174	159,983932	271,052227	113,601568
546,866211	305	294,624287	475,114111	198,778463	158,00888	270,410723	115,28423
548,778687	305	281,759834	458,413249	199,251085	156,514376	267,918096	109,012004
550,689514	305	281,924464	452,628995	187,258865	149,004104	256,902508	105,52809
552,598816	305	275,297236	442,860949	190,206881	144,32398	254,765775	103,299621
554,506531	305	257,612608	415,166343	177,875435	134,694036	241,770919	95,9238332
556,41272	305	259,930078	407,105486	178,928573	136,71265	239,291436	96,6965177
558,317261	305	257,848569	390,533284	176,727925	133,894068	242,982047	88,9977046
560,220215	305	253,83179	375,849275	174,180972	128,391119	228,283487	90,2340608
562,121582	305	243,877715	367,871212	170,972726	124,101044	217,057431	82,453705
564,021362	305	236,633914	346,351136	157,887486	114,022443	212,007895	79,8493132
565,919556	305	237,762878	336,238308	156,416821	114,071549	212,895593	80,2302687
567,816162	305	225,048799	329,039844	156,633985	112,052962	213,900932	77,6257978
569,711182	305	230,788021	321,913755	149,324313	105,08159	203,280701	67,4673929
571,604614	305	220,178399	303,581894	151,494657	105,304098	197,939044	72,3802628
573,49646	305	210,399824	300,330903	147,180103	96,9588373	199,273804	72,2181876
575,386719	305	206,350942	281,938858	139,442838	104,034027	188,77869	65,5363775
577,27533	305	210,009716	277,905839	143,866362	98,7832338	183,354849	63,4999792
579,162354	305	202,629557	271,619644	138,085883	93,8752636	180,59934	60,4029554
581,047852	305	195,806594	259,345171	133,563843	89,16498	174,840563	62,0463127
582,931641	305	193,511007	241,750126	126,949592	88,0188079	168,086966	58,4937146
584,813965	305	186,463744	239,688077	127,276461	84,0311122	161,620539	53,5558795
586,69458	305	181,505812	236,558605	123,563983	81,9768853	162,872888	54,3540463
588,57373	305	172,647179	221,41669	117,004072	83,431732	157,27646	49,7621139
590,451111	305	171,211506	216,130652	111,701984	75,8695048	147,517	48,399051
592,326965	305	162,830973	196,809681	108,992866	71,5797568	139,280844	44,0894274
594,201233	305	158,658158	197,174379	105,828317	74,4466033	139,814701	43,1486454
596,073914	305	157,273254	185,519233	102,467331	65,7813334	137,22735	43,1966389
597,944946	305	152,859058	185,484993	97,18276	68,7880088	134,702675	42,3608398
599,814453	305	151,563998	178,255382	102,264568	63,0390231	133,58468	38,5509392
601,682251	305	146,82233	171,327797	95,6638175	63,819332	127,031353	38,724484
603,548523	305	141,469217	164,466555	92,2855337	61,5641413	118,811677	36,7950941
605,413147	305	138,449699	162,467681	93,316925	58,1554405	118,213479	38,575906
607,276245	305	125,948283	150,331468	90,1438552	59,2547316	115,129068	38,2543234
609,137695	305	131,951903	149,567419	86,5809263	49,0157827	115,870715	32,681591
610,997437	305	127,71796	146,824047	80,9513123	53,9977294	110,948972	33,0093333
612,855713	305	120,754631	145,952109	85,069407	52,8287519	105,572048	31,9688839
614,712341	305	119,621614	130,173594	79,7445679	47,5417226	102,942164	30,3402491
616,567322	305	112,315389	126,597626	75,0641611	49,8464334	99,7369064	27,7316928
618,420715	305	110,88517	126,916787	70,136447	43,6798014	100,54777	26,449995
620,272522	305	106,457592	119,329042	72,2785507	44,7040099	92,1096446	30,48217
622,122681	305	106,820402	113,980452	70,5701053	44,400289	91,507668	23,6413574
623,971313	305	101,891032	109,340649	67,1583419	41,3130375	87,7453874	25,4374081
625,818237	305	98,1263317	105,875922	67,0213827	40,8050233	83,3918206	28,7467895

Please do not adjust margins

Please do not adjust margins

627,663574	305	93,7205173	101,88954	64,0507901	40,4733615	80,3098694	27,7257924
629,507324	305	90,6638298	97,5220466	63,5566269	38,6458295	83,9498664	22,3437226
631,349365	305	91,8140021	98,1353589	63,0633126	33,941442	78,8709243	23,4377899
633,18988	305	86,1917929	93,8872391	59,5560027	34,5628633	73,4954795	21,6023181
635,028809	305	83,9697293	90,3055865	57,0854239	35,7613063	73,7225172	18,2160436
636,865967	305	85,3264796	88,140836	55,2796166	32,8334783	71,9397965	20,407477
638,70166	305	78,0323719	77,0467384	52,4631733	30,8152798	66,2753285	18,2551274
640,535645	305	79,377239	79,2063464	52,9956928	30,4024444	66,5347261	15,9534029
642,368042	305	71,8522216	76,0372445	51,2954393	29,6237555	64,30379	15,295825
644,198853	305	71,052596	75,2470691	48,0062306	28,325847	55,8592673	17,7705843
646,028015	305	68,4041796	68,5104648	46,0195416	28,2223128	58,5155587	17,383734
647,855591	305	69,7388472	66,2976931	44,7744683	24,8029076	59,0351827	14,3895732
649,681519	305	66,4077908	66,7899189	45,1510949	28,2239786	56,8841036	16,1043746
651,505798	305	61,9963706	62,7952623	45,7486897	25,5884535	52,1629462	15,3482887
653,328491	305	64,398582	66,3040275	43,9604112	24,2873649	54,9468261	12,646385
655,149536	305	62,5519961	59,3357839	40,8006726	24,2370647	51,8209517	14,7617074
656,968994	305	56,2648642	57,214488	38,7472414	23,3637491	49,9481618	12,8832977
658,786743	305	58,913699	55,2509278	37,3981109	24,1745079	50,187758	10,5787894
660,602905	305	57,2298835	53,69944	34,5493855	22,1308008	47,3640194	11,0224459
662,41748	305	57,0642731	51,6872724	34,5640837	18,9941725	46,7322295	12,8939291
664,230469	305	51,2474196	47,2735775	35,476976	20,0184785	43,1574678	12,6385157
666,041748	305	49,9265876	45,522	34,6954849	19,7725443	42,9536341	11,1140528
667,85144	305	47,3691796	44,5130475	33,8008773	19,402036	38,1127975	11,4267467
669,659424	305	46,6129291	46,308257	30,0434005	18,731986	38,9002386	7,49197992
671,46582	305	44,0999472	44,3648741	27,5206559	19,6034633	36,3819097	9,76368255
673,27063	305	40,9879937	41,8264176	30,1919595	14,8315126	38,6645719	7,38696927
675,073792	305	43,6425501	38,4743103	30,8208567	13,1454498	34,5935233	6,68954103
676,875305	305	39,3423803	36,8940157	25,6791781	16,1429806	34,4440438	9,53434638
678,675171	305	37,9177733	36,3277878	28,2515849	14,7167367	33,1630211	7,5432935
680,47345	305	36,8879938	35,2996637	26,804262	13,5633284	31,4236886	6,11535918
682,27002	305	36,2984611	33,9878992	25,1803078	13,8968628	30,6726018	7,99178705
684,065063	305	35,267518	35,9713805	24,9769116	14,230539	31,9250024	6,44331719
685,858398	305	36,4350748	33,2521253	23,2645595	10,7854371	27,7667854	8,16511271
687,650024	305	32,0024572	29,8269592	22,1376617	12,8114327	25,3461598	6,16576532
689,440186	305	32,7988716	30,0403276	23,7737485	12,552483	28,1473042	6,89862387
691,228577	305	30,3574949	26,4427129	20,7582053	10,5377245	23,6842499	5,60788924
693,015381	305	27,9704942	26,9441234	20,5216299	13,3139831	28,521884	5,91037276
694,800476	305	30,5715034	26,7925687	18,782715	12,2978902	24,3171556	3,60298566
696,583984	305	28,6582654	26,3190696	17,9881307	10,1685507	21,5087187	3,90692993
698,365845	305	27,5717345	26,8304768	23,2757102	10,1971232	22,7350563	6,52984665
700,146118	305	26,8030666	23,5704327	16,7962338	11,7004004	21,6553029	6,11886499
701,924683	305	30,4347793	20,8687502	18,1803471	9,10267094	23,0604944	8,48234611
347,741211	310	56,0822218	707,983944	75,5186473	183,658426	100,332609	137,811134
349,808533	310	53,6047428	775,796107	74,6781012	191,92965	102,695322	143,167278
351,874329	310	59,8459956	832,127151	71,6711634	196,370269	110,360444	135,908567
353,938568	310	69,799722	896,593686	78,2594089	208,889317	121,544422	141,942964
356,001343	310	73,9116655	980,375515	90,1065883	226,732489	133,731865	169,990574

This journal is © The Royal Society of Chemistry 20xx

Please do not adjust margins

Please do not adjust margins

358,062561	310	82,4188885	1054,56798	93,9641125	236,614917	153,778822	193,31027
360,122253	310	97,5560218	1110,04438	93,6079083	254,065882	161,985036	202,431113
362,180481	310	103,27936	1169,31559	113,188946	263,546532	164,910207	235,469583
364,237152	310	98,9286993	1263,82366	122,212425	275,742878	191,227391	255,742044
366,292328	310	118,343532	1331,71404	132,000921	292,609167	202,992117	297,997992
368,345978	310	129,809388	1407,22115	149,82993	302,774946	213,255503	309,430074
370,398132	310	130,629628	1473,38901	142,791698	328,405041	234,44977	349,666193
372,44873	310	142,833417	1537,46205	160,084993	328,915912	258,737271	398,8039
374,497803	310	156,601745	1612,96942	175,138183	355,72621	264,094694	428,228557
376,545349	310	172,6237	1676,72249	178,103311	375,957942	279,786682	465,241843
378,5914	310	174,874587	1701,65369	185,717807	382,108033	292,973228	503,33225
380,635925	310	187,887418	1789,35411	193,464345	385,926809	303,935057	536,460488
382,678955	310	206,962271	1852,56394	210,397867	412,82888	323,129709	596,586944
384,720398	310	213,418938	1912,39148	228,233142	447,459152	336,057809	598,673959
386,760376	310	216,427756	1922,53792	230,989051	448,37419	343,362582	643,206206
388,798767	310	227,454867	1979,82482	234,541877	473,007831	366,699734	672,730924
390,835663	310	232,646264	1999,87267	238,614259	478,600926	365,744223	694,253141
392,871033	310	248,11806	2032,32329	248,742444	484,999517	375,966378	718,480496
394,904877	310	253,144832	2065,22765	268,780249	509,969543	384,020144	739,417073
396,937195	310	262,582939	2106,27582	269,989107	525,109679	405,218857	752,308475
398,967957	310	275,766685	2104,20362	277,781556	534,734011	406,193205	789,614935
400,997253	310	286,523149	2135,49183	284,003223	544,825577	427,589453	806,223836
403,024963	310	294,514315	2151,60222	295,922786	538,398666	415,232847	805,230486
405,051147	310	298,677689	2178,31851	307,658715	549,530104	421,38488	819,060405
407,075806	310	313,352426	2183,21303	302,656062	558,909286	435,788793	826,585463
409,098938	310	315,70239	2201,4604	320,662911	548,253438	448,742706	842,876008
411,120544	310	329,904198	2164,92283	319,96522	568,199671	443,686546	835,46085
413,140564	310	338,359462	2178,38962	323,602107	558,556651	451,143648	845,543041
415,159119	310	350,066636	2164,99041	343,307633	550,44137	448,984497	821,409672
417,176086	310	364,54274	2140,22286	343,987318	568,58766	454,753148	821,788687
419,191528	310	356,126277	2143,83936	341,414153	549,783215	462,896568	802,366235
421,205444	310	358,71626	2136,14642	352,325335	559,69389	463,348501	802,803575
423,217834	310	375,908818	2090,09233	346,300988	549,99322	460,837371	788,190943
425,228668	310	386,176247	2059,52385	355,696048	552,777672	454,899857	786,268828
427,237976	310	389,042314	2058,48039	360,299372	538,313772	466,356052	769,83833
429,245728	310	386,700654	2011,02329	352,251449	534,113453	457,431213	757,743446
431,251953	310	397,37603	1989,46703	364,558282	536,624729	457,44039	734,755906
433,256592	310	394,384624	1961,7112	373,199652	533,605111	461,499023	742,075081
435,259766	310	417,038615	1981,97093	366,960003	533,616333	464,45899	714,32627
437,261353	310	416,222007	1944,4325	374,477963	518,881604	461,964663	699,774999
439,261383	310	419,712791	1924,1909	363,936123	508,567119	459,332018	688,152623
441,259888	310	415,503868	1887,19124	374,871907	514,788626	459,029854	682,711501
443,256836	310	420,528625	1858,96083	361,287252	506,793607	460,334094	645,722755
445,252258	310	421,601812	1833,21367	373,83138	481,125394	453,555073	635,599912
447,246094	310	420,13224	1786,12181	361,533643	484,855461	450,372774	619,354438
449,238434	310	418,745332	1743,75364	378,520956	477,887606	455,668418	604,682834
451,229187	310	419,414744	1724,34051	360,446479	467,731595	447,15965	592,753643

This journal is © The Royal Society of Chemistry 20xx

Please do not adjust margins

Please do not adjust margins

453,218445	310	420,218591	1688,50948	364,125801	456,204907	442,721306	570,776597
455,206116	310	418,171113	1636,96669	354,614377	450,134985	434,854009	540,115328
457,192261	310	432,943002	1617,37266	363,917164	444,97155	446,410015	541,427233
459,176819	310	419,13272	1599,54424	356,660088	439,203531	446,306584	517,300239
461,15979	310	435,98813	1547,98491	357,553564	427,862507	446,927686	506,611845
463,141296	310	432,168864	1525,38673	352,856285	414,114726	435,473598	498,534319
465,121216	310	428,684595	1494,9146	351,946046	406,076007	424,724851	471,112945
467,099579	310	420,930495	1447,19354	343,389194	404,292936	426,529842	456,594155
469,076385	310	421,281733	1414,79717	340,076542	393,442513	427,645066	444,872624
471,051636	310	423,885016	1391,0655	343,135394	389,049774	424,764981	436,180726
473,02536	310	429,426364	1368,12218	350,601524	384,710541	431,917587	425,332299
474,997498	310	423,91808	1313,60738	347,442685	373,462073	429,87047	405,979872
476,968079	310	423,08615	1300,48691	343,959751	377,032126	428,071986	395,277444
478,937134	310	424,754917	1275,35167	342,413246	361,975799	430,346909	379,162073
480,904572	310	419,710048	1245,88836	341,211903	352,02679	436,858845	373,63658
482,870453	310	425,487338	1212,19961	345,73456	352,587722	428,549721	354,653941
484,834808	310	426,884773	1209,04736	349,117547	349,411962	430,331424	355,136182
486,797607	310	433,362059	1179,02919	337,88336	339,219083	426,222265	340,787871
488,75885	310	424,215572	1149,61019	332,199629	335,864408	419,584635	323,586644
490,718506	310	420,375459	1126,82557	328,983247	317,994967	433,902918	319,354196
492,676605	310	424,043245	1083,87473	326,502995	320,469829	423,565521	304,703752
494,633118	310	428,040142	1064,00705	324,481757	312,307513	416,509243	298,677978
496,588104	310	423,393747	1034,39878	326,479254	304,392975	415,629496	292,633673
498,541504	310	421,901779	1009,65677	320,228515	293,79287	411,374327	280,214252
500,493347	310	418,451066	981,929345	317,331331	297,138826	414,440655	262,718114
502,443665	310	409,283655	957,215433	309,313087	286,70718	409,828582	263,143923
504,392365	310	417,336587	932,849329	312,045824	279,449502	404,568734	249,826424
506,339539	310	395,890943	905,170267	297,915794	276,03049	397,686122	239,069862
508,285095	310	404,251204	877,756672	302,623038	265,144805	391,293211	232,252533
510,229126	310	387,180856	847,696782	292,720989	258,639328	377,787694	231,431395
512,17157	310	387,813006	810,595938	282,538384	244,244665	380,152588	216,039238
514,112427	310	396,875585	794,89606	293,541619	251,213288	367,946963	213,197379
516,051697	310	382,898122	777,820388	277,398332	241,327686	363,995347	195,219137
517,989441	310	375,670518	750,46097	271,278156	230,692061	359,867318	191,898295
519,925598	310	368,492515	720,016616	266,065202	226,969671	351,617661	178,241101
521,860168	310	358,537481	704,4857	259,403595	220,163699	345,559141	183,069452
523,793152	310	353,304932	672,489131	259,631241	213,979716	344,880925	172,381085
525,724609	310	351,154305	658,513577	253,267671	203,739432	336,634186	166,508958
527,654419	310	347,889518	641,399287	248,147608	203,349053	330,158096	160,749855
529,582764	310	345,549416	619,683462	249,749263	192,877309	323,676148	151,455419
531,50946	310	340,183408	598,623705	243,579535	188,705983	319,213382	151,095419
533,43457	310	338,803982	568,292449	236,178103	191,971906	318,14541	144,457209
535,358093	310	327,809968	567,589864	234,97114	183,455355	309,236378	138,822913
537,280029	310	325,500847	548,388668	232,343943	175,15707	310,890393	133,31538
539,200439	310	313,710653	535,576999	226,140874	172,83636	293,654421	126,910079
541,119263	310	309,294426	520,062238	221,487193	169,920623	292,376414	125,93993
543,036438	310	302,566872	502,710012	219,911958	165,625264	294,467576	122,932938

This journal is © The Royal Society of Chemistry 20xx

Please do not adjust margins

Please do not adjust margins

544,952087	310	306,849934	480,233655	213,320682	160,912405	286,374904	116,437951
546,866211	310	293,53059	462,976079	208,254034	159,000721	268,38799	115,665267
548,778687	310	282,074005	449,999927	209,630977	143,610622	270,690972	107,873901
550,689514	310	287,443081	437,997492	199,411707	144,775946	264,32647	102,020375
552,598816	310	275,553288	416,217975	198,896775	142,546327	254,016791	100,022439
554,506531	310	272,998353	403,285703	191,27685	137,964058	250,894082	96,165548
556,41272	310	265,330024	394,750492	182,773051	133,845043	245,777204	97,9393184
558,317261	310	261,157632	374,693646	188,229438	126,077939	234,765474	88,5758874
560,220215	310	254,577801	371,850658	173,411115	131,433533	235,033558	87,7799305
562,121582	310	246,482964	350,051744	175,739142	122,401183	223,653898	85,4086613
564,021362	310	244,433947	344,747901	171,750556	118,584114	217,275525	80,0531828
565,919556	310	245,951317	324,406909	166,957935	117,629803	220,066112	78,1036584
567,816162	310	224,374222	324,315681	165,929831	109,887465	207,93685	77,5174142
569,711182	310	226,184114	306,569634	159,071845	103,07688	205,219111	69,3300999
571,604614	310	224,953099	302,510664	157,896346	105,786242	199,404704	67,7565343
573,49646	310	219,798928	287,512591	155,515679	100,16998	202,243264	68,9011733
575,386719	310	211,987622	284,187534	147,409009	96,6570243	187,851265	64,0049878
577,27533	310	211,10935	267,326016	149,408277	91,3268623	187,680431	66,0225247
579,162354	310	203,573845	262,963398	140,980554	93,396345	182,722299	59,8835218
581,047852	310	200,792717	252,72368	142,641884	88,9638743	175,164625	59,6489527
582,931641	310	189,946078	244,175793	135,179125	83,6671957	167,460236	59,6491118
584,813965	310	186,085109	237,07891	131,906689	82,9170664	168,012111	54,6286199
586,69458	310	179,006424	225,43863	125,522805	81,7124412	164,035508	50,2059197
588,57373	310	174,086528	216,262342	122,158139	77,3902665	160,626545	51,0726634
590,451111	310	171,476769	210,230336	121,786322	71,7034781	154,216618	48,1057633
592,326965	310	167,268999	202,48129	112,696909	75,3451636	146,102388	42,5698692
594,201233	310	165,071094	191,699501	106,654849	69,2319678	142,474771	42,1866878
596,073914	310	161,221639	187,796577	110,708944	68,0961386	138,844988	43,2478332
597,944946	310	145,561167	177,798164	107,459424	66,9006798	137,585545	41,7014617
599,814453	310	154,779317	171,593568	107,078719	61,3371973	129,968709	41,6024947
601,682251	310	144,371577	165,420449	100,557389	63,7006499	125,900665	36,7540047
603,548523	310	139,552219	166,44231	96,8385491	55,9313587	124,254251	36,9246801
605,413147	310	137,439652	162,287117	98,797646	55,8364977	119,171713	33,4996378
607,276245	310	133,984049	148,443394	92,6612059	56,125753	119,863572	35,1895155
609,137695	310	131,404517	149,455351	87,2857726	51,6789965	113,6975	30,6647983
610,997437	310	123,919755	138,51997	87,5833883	48,5372051	109,929748	33,2134209
612,855713	310	125,839363	138,195913	86,6168268	55,7944313	106,849196	33,2255193
614,712341	310	118,786062	134,938677	81,7195725	49,1865077	104,509092	30,3205375
616,567322	310	115,025005	120,296973	79,5522394	50,7427781	96,6023645	28,6043369
618,420715	310	110,797516	125,870197	78,7910415	44,2927286	98,5012061	26,1445518
620,272522	310	106,627026	118,717958	73,0492525	44,8898747	91,2916294	27,579097
622,122681	310	107,235152	112,672078	71,064566	43,8524418	91,7402834	24,6233871
623,971313	310	102,468493	108,051795	72,0171522	39,9290249	90,6014061	24,4388371
625,818237	310	100,587013	107,844051	70,6525241	36,5346968	86,1384307	23,2706239
627,663574	310	96,9470133	101,424504	65,566941	37,6155542	84,8919241	24,4517669
629,507324	310	94,9952953	95,2823916	64,8683353	37,0345926	86,3317274	22,1531765
631,349365	310	94,809413	95,0969797	66,0662339	35,2726933	79,7495592	22,6791476

Please do not adjust margins

Please do not adjust margins

633,18988	310	89,5162	91,7317706	61,8655821	33,0492959	76,6051294	22,3489817
635,028809	310	84,3358106	86,6832802	59,7803933	34,5657659	74,2548072	21,6974415
636,865967	310	86,5243024	82,9741915	58,7218116	33,4668632	66,6794494	20,4536899
638,70166	310	78,2229578	78,5287893	56,5732708	30,2778641	67,8708151	18,4184427
640,535645	310	76,410692	78,3860745	59,2563505	28,8613058	67,0886894	19,0544716
642,368042	310	72,5169296	74,1133861	53,5490926	29,9235087	66,5273945	15,1083065
644,198853	310	71,5136978	73,500941	50,1899331	29,3445457	62,423547	16,4240288
646,028015	310	75,8536118	72,2973432	48,7019663	29,1208246	65,4595631	19,2691995
647,855591	310	68,8571663	64,6654263	48,6805467	25,3729174	58,3467329	14,5097459
649,681519	310	67,5406786	64,6232478	42,3380846	28,0985378	57,2348327	13,1674399
651,505798	310	66,7767291	64,7586309	44,3320601	26,0036351	55,9096385	13,8696349
653,328491	310	62,6737456	60,3929081	43,5558645	23,9970871	53,73436	13,6523429
655,149536	310	60,0183615	59,1721883	37,8244183	22,9061973	53,0132136	14,0934035
656,968994	310	55,1345658	56,5247864	42,8433373	23,6580383	48,2376843	13,3654499
658,786743	310	61,216601	56,79593	40,1437151	24,5639076	48,9973489	10,796315
660,602905	310	55,6629238	54,9446435	40,6359912	22,1069835	50,1725744	13,0654807
662,41748	310	54,3154591	53,9945423	38,159348	20,4776588	46,0097467	13,6544431
664,230469	310	52,421803	47,0386282	36,902427	18,9192553	46,6334879	12,8844123
666,041748	310	49,9784527	47,5249579	35,5016549	19,8881214	45,9176971	15,170138
667,85144	310	47,7038488	46,3169613	33,9932663	17,265908	39,8868865	11,6090358
669,659424	310	46,0554146	45,7405617	32,8555255	19,7151553	40,6386859	10,6967055
671,46582	310	46,2369143	46,8615195	29,1303627	16,6474752	39,4719137	10,3061059
673,27063	310	46,0132775	42,1923452	33,2875246	17,521385	37,2004611	7,92782904
675,073792	310	42,8571776	38,7620503	27,8155264	13,0988247	36,8669664	7,67383816
676,875305	310	40,8214951	36,8642285	26,4602408	16,3366733	34,2946308	9,82603483
678,675171	310	38,1241426	36,4649212	26,4644459	17,6896224	33,2197635	7,00047962
680,47345	310	39,3108418	39,2710834	29,8172363	14,5726791	33,3293436	7,26417989
682,27002	310	37,6848259	31,9590792	27,6189804	13,1337801	32,0907978	8,9092086
684,065063	310	36,9940383	32,744655	28,5258038	12,2264855	33,0125874	7,31358096
685,858398	310	39,5955298	31,6715727	25,5485805	12,6395732	29,9010405	5,01335396
687,650024	310	31,5989288	31,2948411	24,213643	14,4207063	27,0737182	6,51027132
689,440186	310	34,1292763	28,23046	26,1784577	14,4540391	27,2726612	6,38937537
691,228577	310	31,0046275	28,1079013	22,0989395	12,5511864	24,6939002	6,39251851
693,015381	310	28,2074662	25,9902823	21,6077577	10,5313458	28,3124631	6,95409322
694,800476	310	31,2121161	30,0864943	22,1492558	12,169554	26,254276	5,18018701
696,583984	310	28,8640318	24,3036501	20,591345	9,61019123	22,3787464	3,14803107
698,365845	310	27,8424648	26,0242758	21,3375113	8,94882379	19,4126969	7,54905299
700,146118	310	27,9536672	25,2987492	20,593466	10,0903579	21,0107454	3,44509264
701,924683	310	25,5606362	22,4817125	19,8401811	9,8516387	22,3403598	5,39500583
347,741211	315	57,5658778	621,414161	71,1917547	184,513078	94,2029936	144,106044
349,808533	315	79,1384205	689,393656	82,2701154	216,828992	113,905643	155,387246
351,874329	315	77,1785811	771,45895	85,1992111	206,587862	131,427494	163,947884
353,938568	315	72,6268385	829,761287	89,7842454	217,349151	130,564123	174,188541
356,001343	315	72,6936833	910,08812	97,1373621	218,30992	141,534533	168,806231
358,062561	315	84,8108724	981,669581	95,2176184	229,768388	151,145806	187,156911
360,122253	315	101,213464	1067,59	101,416815	231,542525	161,669294	205,665807
362,180481	315	101,037655	1134,00209	117,386887	265,641632	179,564663	228,085256

This journal is © The Royal Society of Chemistry 20xx

Please do not adjust margins

364,237152	315	112,689226	1228,4097	128,498087	285,859834	195,930096	259,083378
366,292328	315	121,476627	1317,85164	138,407803	297,03284	211,204632	287,843064
368,345978	315	136,430717	1407,67124	158,267534	321,961937	239,895491	329,716549
370,398132	315	143,006679	1459,65814	162,826566	333,632947	241,21009	352,103208
372,44873	315	164,169786	1553,88605	185,46862	344,65145	270,477221	390,728125
374,497803	315	174,998536	1662,90217	194,218358	369,913464	295,645055	431,835289
376,545349	315	187,543023	1716,63033	192,094696	384,072442	309,689432	471,398336
378,5914	315	195,202077	1776,97317	211,058464	398,89355	311,767589	499,124528
380,635925	315	216,000742	1841,62006	222,690325	414,723747	345,07955	530,165627
382,678955	315	226,807321	1932,72265	238,739764	431,374765	359,763804	585,390524
384,720398	315	237,838776	1989,99346	248,363039	446,132467	368,85302	612,947124
386,760376	315	242,080462	2055,56352	251,667758	461,72545	390,299062	648,463945
388,798767	315	268,782579	2113,15328	263,641447	499,654451	406,896412	685,526091
390,835663	315	281,934242	2118,68218	277,992727	497,266954	409,859871	708,771984
392,871033	315	276,014077	2167,79489	291,348144	512,745255	416,340651	743,633811
394,904877	315	291,577512	2184,74428	310,163642	530,442586	433,953684	776,094379
396,937195	315	312,496194	2282,17038	314,136488	548,917247	449,244862	786,417685
398,967957	315	308,764245	2254,34747	319,44831	548,279327	449,153681	800,654477
400,997253	315	334,822246	2316,63918	320,674521	564,590886	452,445679	823,509243
403,024963	315	321,214192	2329,75708	325,349553	563,163359	469,390172	840,122945
405,051147	315	338,003264	2327,47292	347,866287	579,960939	470,57454	854,469414
407,075806	315	358,25351	2349,74021	340,829884	583,710413	474,986419	852,28287
409,098938	315	360,223675	2351,06814	361,237092	581,433001	497,438639	859,693073
411,120544	315	371,9872	2318,1993	369,383669	589,922933	500,867968	872,32231
413,140564	315	384,319846	2327,07335	374,768319	592,904825	494,424861	861,860735
415,159119	315	390,266422	2316,56009	374,450594	592,002789	497,906388	874,798081
417,176086	315	398,099551	2309,95161	386,54366	596,000248	504,549275	874,185531
419,191528	315	409,189977	2283,8054	390,026426	588,508376	503,812335	830,88857
421,205444	315	407,490429	2270,27262	391,282899	579,413018	510,472527	844,273429
423,217834	315	412,282594	2256,44436	397,738811	583,800766	506,432918	829,767716
425,228668	315	432,925909	2235,30262	399,389615	570,546856	503,225025	806,141938
427,237976	315	441,277851	2221,20442	411,410108	576,891809	522,17768	802,348089
429,245728	315	424,391522	2169,35558	405,913989	564,846058	507,140693	774,448504
431,251953	315	443,141606	2135,54242	422,21636	559,58497	521,928187	772,310453
433,256592	315	441,168685	2118,18515	403,726927	553,027599	507,441047	766,360218
435,259766	315	450,294611	2085,36404	423,099971	557,088606	504,098229	748,834682
437,261353	315	452,658771	2096,79723	415,201662	536,419	520,022338	720,12428
439,261383	315	458,446015	2048,55973	414,710069	539,557239	524,700808	725,078525
441,259888	315	458,754894	2037,14933	419,755689	522,995746	497,060629	703,992558
443,256836	315	458,259333	1958,83891	408,622483	528,605064	495,729684	683,491698
445,252258	315	465,191778	1933,88727	412,582261	509,65707	492,870999	669,347443
447,246094	315	457,144824	1874,48399	408,591277	492,042845	492,432715	647,420385
449,238434	315	458,843414	1833,51346	409,017986	496,499019	483,028233	631,796138
451,229187	315	457,662143	1810,70639	395,813826	475,870564	479,427212	600,47905
453,218445	315	465,3292	1774,43422	406,806019	464,052845	484,276997	591,116627
455,206116	315	454,843155	1741,85381	389,999417	465,927425	472,336358	578,856329
457,192261	315	456,08842	1710,65348	392,8624	449,774334	475,426636	559,294917

This journal is © The Royal Society of Chemistry 20xx

Please do not adjust margins

459,176819	315	458,185798	1654,65605	394,825345	444,548426	470,766981	536,221839
461,15979	315	460,951443	1612,1018	384,871711	436,132428	470,355519	525,503933
463,141296	315	450,872321	1578,3607	377,460419	426,049821	466,571919	507,604953
465,121216	315	449,316713	1554,69077	381,955817	415,390174	456,182832	493,240183
467,099579	315	440,930011	1503,52755	377,652051	413,884749	458,748838	482,163804
469,076385	315	444,472247	1452,7682	377,573833	394,966752	458,576985	472,204519
471,051636	315	448,369519	1441,80146	371,792923	392,576997	450,243976	451,306606
473,02536	315	446,641337	1399,28444	378,670917	386,133394	451,884719	434,288038
474,997498	315	443,347422	1343,62056	364,823308	375,052135	445,968487	417,254271
476,968079	315	449,390594	1331,4782	363,996165	369,310863	456,343245	402,361671
478,937134	315	448,425205	1303,28489	367,007468	369,607217	445,045928	392,48058
480,904572	315	451,147362	1271,09361	357,006209	356,702187	440,453856	373,852712
482,870453	315	435,902852	1248,03313	358,757115	351,025814	447,789723	366,384712
484,834808	315	450,773549	1217,22189	355,416528	347,325725	435,601163	355,264682
486,797607	315	451,445588	1182,21477	352,441169	344,507559	438,208162	351,420434
488,75885	315	447,854834	1149,27068	354,821392	332,603043	441,91123	333,165113
490,718506	315	438,894183	1106,60911	342,356051	327,884376	440,679203	328,703003
492,676605	315	447,573405	1087,81735	340,962489	320,122867	426,177577	321,073772
494,633118	315	439,758783	1063,87979	341,496435	312,853999	438,194605	300,035239
496,588104	315	433,561952	1029,14588	336,148587	303,478232	429,797551	300,041643
498,541504	315	433,957201	1013,66459	339,569349	296,549676	420,068182	279,494796
500,493347	315	430,087241	965,197603	333,902739	291,537119	414,470933	274,723382
502,443665	315	424,76998	953,80159	323,035406	281,374598	410,924719	263,339192
504,392365	315	427,848831	923,862645	324,881983	281,019589	414,27201	257,494695
506,339539	315	412,917584	895,301977	316,734913	265,453244	405,908119	239,038983
508,285095	315	412,899731	861,971363	311,449447	261,180955	396,063887	236,110871
510,229126	315	397,949188	843,558649	303,523578	249,346073	385,154942	226,865901
512,17157	315	404,379644	810,61701	303,689103	245,281786	383,144982	220,161191
514,112427	315	394,880059	795,48413	294,781764	237,656638	384,642611	210,831003
516,051697	315	387,616296	751,979938	289,491298	235,61228	372,247043	201,374974
517,989441	315	382,662743	735,395493	287,196361	222,85167	366,906272	194,668092
519,925598	315	379,966046	713,864799	284,284665	220,871138	362,721835	184,900839
521,860168	315	369,928238	689,630431	276,135964	217,055629	347,199667	180,37703
523,793152	315	362,055332	658,878211	261,030561	205,656891	344,884241	172,009506
525,724609	315	355,970472	642,503739	258,955851	201,364073	341,900707	165,900932
527,654419	315	357,070953	624,646108	257,923277	194,664141	335,948445	157,558592
529,582764	315	359,832499	609,377212	250,067476	189,599487	336,42602	153,494157
531,50946	315	344,518182	588,537277	251,664136	184,358696	327,08697	154,41299
533,43457	315	342,551193	582,228599	245,847665	181,014609	326,448831	145,219309
535,358093	315	339,101498	564,661222	246,218593	174,894306	320,326068	138,882087
537,280029	315	327,62306	543,454976	232,644466	170,77691	307,687219	133,456409
539,200439	315	325,977478	525,891741	235,318786	176,933099	304,751858	129,46283
541,119263	315	317,238103	508,035955	233,373182	165,296778	297,679676	127,713455
543,036438	315	315,193065	484,410656	228,818933	160,612077	288,292324	121,113026
544,952087	315	313,796352	474,520031	217,690293	154,0613	289,374766	119,343681
546,866211	315	302,827605	467,067183	215,337562	151,781497	273,937712	110,647373
548,778687	315	300,000189	439,877698	210,059193	143,608199	273,798773	105,171526

This journal is © The Royal Society of Chemistry 20xx

Please do not adjust margins

Please do not adjust margins

550,689514	315	293,710095	439,284404	209,834288	141,704421	269,451731	104,557609
552,598816	315	288,94015	412,010109	205,616737	135,551416	261,404967	101,066805
554,506531	315	277,344511	403,717889	198,403494	135,159911	249,381145	97,5822591
556,41272	315	276,566445	394,761896	194,586185	131,320071	250,930973	93,4018485
558,317261	315	265,765846	375,530089	183,676176	121,085203	240,580213	87,1850875
560,220215	315	257,447975	362,793766	183,25878	124,77699	235,492918	85,1990259
562,121582	315	251,381877	350,04158	184,412308	119,788229	230,613197	81,1423841
564,021362	315	243,730595	343,074892	176,129117	110,656926	223,053715	79,8977367
565,919556	315	244,219398	332,040104	172,590097	112,643393	219,177414	77,1439025
567,816162	315	234,80509	318,405953	170,062331	109,602419	217,576649	75,2395796
569,711182	315	229,145924	306,266678	168,709518	106,468583	207,117226	69,6319614
571,604614	315	223,249706	295,288564	163,565894	100,59639	203,168223	71,8692104
573,49646	315	222,539273	284,674094	157,899209	94,6360977	200,236531	69,015629
575,386719	315	213,021255	277,293181	149,681519	95,3678018	199,106735	67,8503556
577,27533	315	213,49406	270,666128	150,727509	93,0204963	190,43561	61,9249175
579,162354	315	206,064885	257,211956	145,878623	86,739395	181,675355	60,1570739
581,047852	315	198,582408	245,104897	144,395715	83,1676377	175,484037	61,2106179
582,931641	315	193,494033	241,75743	140,927819	84,5539905	172,941252	56,4845251
584,813965	315	187,498777	232,54118	136,19077	78,6338583	173,966041	57,3233894
586,69458	315	189,10638	222,788173	128,809172	78,5206214	169,952822	49,6125072
588,57373	315	176,408576	215,657188	127,957564	75,5415909	157,746449	45,7914895
590,451111	315	172,101198	209,005867	125,927692	70,4500294	155,867449	46,3219241
592,326965	315	169,577221	198,204574	119,7996	69,5556088	152,69064	49,0884969
594,201233	315	171,154244	197,14576	113,735305	64,7474889	144,264471	43,5162199
596,073914	315	163,312657	181,52739	110,566133	66,5493833	143,618376	42,5044482
597,944946	315	155,539381	174,355273	108,651309	58,873193	142,278713	42,7073147
599,814453	315	149,615518	174,182571	110,445447	63,5575782	127,940065	40,8006232
601,682251	315	151,672592	161,866865	106,211454	61,9640791	127,157115	37,8228019
603,548523	315	141,970781	158,639761	102,739294	56,7886582	128,566618	38,3407689
605,413147	315	141,970012	154,537222	93,8080301	59,2093393	123,070681	36,1227315
607,276245	315	135,523154	147,364583	91,9884929	54,5716556	117,972965	33,4403406
609,137695	315	137,941997	146,177368	89,9303808	52,9324897	114,319539	36,1303024
610,997437	315	132,221446	134,021949	88,91022	53,8753739	112,509023	30,705861
612,855713	315	127,832642	134,669299	90,261163	53,775232	105,95762	30,2389597
614,712341	315	120,722544	129,151202	84,7354561	47,1952845	104,801264	30,9197616
616,567322	315	118,565046	124,243163	82,6606034	49,8907387	105,154351	24,5943909
618,420715	315	114,399775	118,890573	81,5772415	43,4627851	100,007465	28,8357974
620,272522	315	111,260507	121,434585	82,7167314	45,6062745	95,3902231	28,6563008
622,122681	315	111,6212	109,731958	77,5702486	40,1425762	93,9015702	25,339429
623,971313	315	106,221126	111,244115	76,083727	36,1302116	90,1544216	24,9248088
625,818237	315	99,084288	102,315578	76,7496115	38,0986842	82,8358874	23,9299747
627,663574	315	99,624726	101,520442	66,1450968	37,5559792	86,7644765	26,1623774
629,507324	315	94,2262747	95,161951	66,8388075	37,3733504	81,3054576	21,6444141
631,349365	315	92,69957	94,129013	66,2744171	36,4198883	75,8428443	20,6813669
633,18988	315	90,2085512	91,5232689	64,8299576	35,6445489	76,881017	21,3429648
635,028809	315	91,9994531	92,3279102	64,9140879	31,8152107	76,7120531	17,8977431
636,865967	315	86,2330542	89,8927408	61,4465725	33,9575386	73,7560957	19,1541151

Please do not adjust margins

638,70166	315	78,7617428	81,2063353	57,907352	28,9290195	70,7166567	19,9017488
640,535645	315	74,3108104	77,2548665	57,7735318	27,8159897	70,8268454	17,1958832
642,368042	315	81,9267829	78,0869746	56,1165041	30,6837589	62,1560958	15,9868515
644,198853	315	72,9534411	72,701033	53,1475947	25,5632171	64,1440676	13,9262433
646,028015	315	76,6279321	71,6730634	49,7443658	27,8182859	60,1351079	16,4100484
647,855591	315	68,6749561	64,4773868	51,9488678	23,2335005	61,840185	17,5127972
649,681519	315	69,2915713	69,9141572	47,7504895	25,4700893	64,160876	13,2669398
651,505798	315	63,7052862	62,2210532	44,9499471	28,9430752	59,8048186	14,3174245
653,328491	315	63,4004657	64,4140606	46,9671911	23,2758885	53,6020092	12,738077
655,149536	315	58,7263563	56,8651114	42,2354224	23,6116	51,0314111	14,1630368
656,968994	315	59,0953496	57,9824821	44,1770678	22,8263999	51,9996042	12,3337544
658,786743	315	59,8863353	51,8012887	42,7402471	26,1038372	52,122223	13,0252099
660,602905	315	57,86629	50,1467573	39,282047	20,9579876	51,8644497	10,7744456
662,41748	315	54,9324875	51,2646952	37,6707455	20,9245463	48,0113617	13,4967263
664,230469	315	53,5048565	46,8937002	39,7922686	17,3863021	42,6046031	11,4820966
666,041748	315	53,7307305	46,7232564	36,660311	18,8268408	44,3486281	11,3752381
667,85144	315	50,0207894	47,6218614	35,3472005	18,6217432	41,5144063	9,99671494
669,659424	315	48,5764133	43,4742713	32,972012	15,8285305	40,6973889	11,0444555
671,46582	315	46,0464481	38,8835921	32,1032244	18,2922722	40,3522379	9,64164081
673,27063	315	45,0614904	41,8796299	33,0056186	16,0361232	37,5335527	8,00165391
675,073792	315	42,0345917	35,3554189	30,7348826	13,2164065	38,5135579	7,49983282
676,875305	315	42,9346054	34,8307863	30,3400805	16,4526021	34,8760371	11,1183336
678,675171	315	44,8874148	35,8723461	30,4745812	13,3455695	31,6397971	8,01681664
680,47345	315	36,2973488	34,2921145	30,4513201	13,3353829	33,1705145	8,39831183
682,27002	315	36,5532128	33,3721261	27,8262317	15,0705332	32,5053325	6,48262657
684,065063	315	35,3695592	32,5710592	26,6141079	12,1225699	32,2220137	3,38217029
685,858398	315	38,7799404	34,4101156	26,7562927	15,127253	30,9310314	6,89744622
687,650024	315	33,5903878	30,6606441	25,6070385	13,1842266	30,0472755	6,25294915
689,440186	315	32,8820064	27,4622047	26,1900286	14,1305718	25,6646903	5,09226459
691,228577	315	33,5535244	29,9616122	22,7965338	11,5194633	22,9261747	4,70286429
693,015381	315	27,4625374	27,4139855	20,2272334	10,1050041	29,156992	6,28633293
694,800476	315	34,4181225	26,0957918	20,3534939	8,79046966	26,5236093	4,71357206
696,583984	315	29,9406396	23,4361752	20,9630143	11,7240218	28,2126811	4,99551859
698,365845	315	26,8503447	25,6145703	22,4762647	7,39764092	21,8138911	8,70080687
700,146118	315	30,6758722	21,4749159	20,973138	11,0081522	24,2902172	5,82370519
701,924683	315	28,7942059	23,9527497	19,3214959	9,98536652	23,7173808	5,97895981
347,741211	320	46,6266787	507,877834	56,212484	137,866262	75,9573866	87,2963636
349,808533	320	57,4227445	586,369187	62,7838005	160,341323	91,4376113	102,292774
351,874329	320	66,3711851	677,16254	75,4237231	176,561963	119,511493	135,108256
353,938568	320	76,4709921	752,156694	85,9079139	206,410328	135,420037	163,383209
356,001343	320	94,5040577	845,496222	102,320954	232,818123	156,897107	194,781163
358,062561	320	102,504293	932,712819	101,544399	235,185971	177,235489	209,202282
360,122253	320	104,796267	1002,25495	122,581562	256,883606	179,622129	214,411614
362,180481	320	117,813733	1066,19207	122,016143	255,565985	188,649786	231,443558
364,237152	320	118,41352	1169,48942	132,61736	264,653185	203,77595	244,349809
366,292328	320	133,734658	1253,58686	145,896072	290,55719	230,501405	269,598834
368,345978	320	146,338908	1343,72979	158,261947	308,480048	248,50734	304,798996

This journal is © The Royal Society of Chemistry 20xx

370,398132	320	155,648216	1422,83714	171,865951	327,86514	265,242149	337,32107
372,44873	320	174,604795	1534,49023	185,695545	358,315782	286,542268	390,480092
374,497803	320	200,144914	1640,92678	197,842205	369,816667	311,580667	413,333496
376,545349	320	199,157821	1723,67929	218,305342	383,205469	332,597579	457,849279
378,5914	320	215,942938	1808,68198	225,836189	394,08517	341,781949	491,536896
380,635925	320	238,495704	1864,01247	242,361034	416,193447	357,830971	520,411442
382,678955	320	247,47681	1970,29135	252,253415	436,370266	368,928387	566,738235
384,720398	320	261,129729	2008,85071	264,127208	463,201876	391,347001	596,758641
386,760376	320	272,074182	2103,95762	288,075072	477,251575	418,734064	632,356794
388,798767	320	286,005473	2159,06826	306,210384	494,498056	429,344616	678,203962
390,835663	320	302,030067	2179,92598	299,56927	505,53752	442,548391	700,021774
392,871033	320	304,237939	2241,7321	309,287477	514,641351	459,498545	739,978247
394,904877	320	326,060027	2305,96112	327,447789	535,959134	473,856652	763,663551
396,937195	320	336,910411	2344,07262	338,288586	563,982281	483,146528	788,926884
398,967957	320	351,036581	2344,59201	353,914648	565,442671	492,849983	801,974493
400,997253	320	360,925359	2396,79752	363,110878	579,802516	506,965005	816,697701
403,024963	320	377,509111	2424,69654	376,031787	586,255343	508,755535	827,247416
405,051147	320	386,402397	2449,87957	391,17061	587,482575	530,714153	856,576223
407,075806	320	401,468981	2461,76875	391,900855	584,675332	533,870416	864,991431
409,098938	320	409,853354	2493,50936	392,67907	604,436951	549,524857	875,25566
411,120544	320	426,770044	2480,31018	408,280227	608,23552	548,637264	868,207627
413,140564	320	434,046629	2489,8605	415,246955	607,20197	551,25889	882,881635
415,159119	320	437,714266	2487,90915	419,17118	618,412149	555,50209	869,455607
417,176086	320	434,76482	2472,60262	425,127825	618,489233	566,449741	882,997903
419,191528	320	448,017654	2451,6634	432,507041	612,837257	570,98046	861,539878
421,205444	320	460,659718	2420,92663	447,23645	609,147425	559,021506	858,920299
423,217834	320	466,950557	2425,17833	438,989111	607,367546	560,3357	843,640079
425,228668	320	491,044499	2390,20795	444,534276	598,430169	558,255744	838,576714
427,237976	320	469,438874	2372,87578	455,374567	593,036219	563,332702	820,795613
429,245728	320	473,157325	2329,06906	444,739217	585,980938	559,169498	814,758211
431,251953	320	482,545917	2317,61432	457,212872	585,169798	555,313075	794,868013
433,256592	320	491,445719	2267,30215	458,081509	578,383674	561,182806	783,712487
435,259766	320	499,231714	2259,19564	450,06893	571,398676	565,098854	773,760463
437,261353	320	498,568238	2258,15825	461,383884	566,182661	558,800578	750,965171
439,261383	320	509,9624	2210,82382	458,894116	567,563626	551,984607	753,36483
441,259888	320	502,045563	2153,04739	469,20694	546,355476	540,448173	721,134443
443,256836	320	504,099679	2103,36323	460,503226	540,25777	535,511252	695,947017
445,252258	320	494,380583	2066,60597	453,477884	539,478885	543,221417	687,372308
447,246094	320	498,959278	2017,11089	452,565432	515,277832	531,981631	674,063819
449,238434	320	507,523119	1965,98656	440,571353	495,656025	534,781012	647,317945
451,229187	320	492,816507	1912,17048	437,297145	498,514266	525,877811	628,37936
453,218445	320	490,363822	1874,75435	433,784167	481,625866	530,820332	598,837874
455,206116	320	490,267388	1835,57239	427,965175	474,525163	506,607987	593,872008
457,192261	320	486,777482	1790,87229	431,202134	470,036409	515,239053	580,615878
459,176819	320	493,483644	1753,94739	428,399522	460,868215	500,464341	560,610285
461,15979	320	498,94431	1707,68965	424,323337	451,130126	490,540739	540,759997
463,141296	320	491,283353	1680,28554	419,89873	440,044631	498,330414	529,266107

Please do not adjust margins

465,121216	320	478,000023	1616,3442	418,810043	430,098222	485,87775	512,03194
467,099579	320	475,465192	1564,79422	400,165603	418,294415	490,34685	485,31992
469,076385	320	475,389469	1526,93806	405,362473	412,772601	481,868284	476,649087
471,051636	320	470,245779	1491,84285	399,851582	399,253835	479,327266	464,833514
473,02536	320	483,515648	1464,85566	398,310406	387,4043	472,322578	453,643189
474,997498	320	474,49235	1414,37555	398,055812	385,883377	474,615215	431,25839
476,968079	320	457,430737	1376,86697	385,618737	376,109767	467,455802	415,091308
478,937134	320	461,924051	1336,83032	385,764249	368,739046	466,214351	406,629972
480,904572	320	472,159339	1299,26737	381,232851	359,008159	465,924198	387,167209
482,870453	320	468,659939	1269,19485	375,766582	347,741131	460,120309	379,025161
484,834808	320	462,390948	1240,07354	368,948705	353,392359	454,107347	368,094036
486,797607	320	464,396147	1215,49668	368,883708	344,533996	448,625319	357,875807
488,75885	320	457,650108	1178,5266	367,552824	325,221616	450,765816	336,031601
490,718506	320	451,725565	1136,514	364,739431	320,886667	450,029809	331,938473
492,676605	320	454,476361	1124,59103	359,033483	313,949795	441,39177	321,738671
494,633118	320	446,705925	1081,63789	352,62128	313,101694	446,817204	312,998364
496,588104	320	450,944965	1057,47535	356,368488	301,827533	430,368783	299,388063
498,541504	320	451,651554	1025,28057	357,285707	296,259692	432,121725	287,832021
500,493347	320	447,342588	982,844171	346,44089	286,641784	429,235424	274,196683
502,443665	320	432,222291	961,861505	346,90872	273,849238	418,05673	266,516374
504,392365	320	437,838475	929,389292	328,678813	276,769819	426,392683	260,951612
506,339539	320	426,215867	913,944234	335,495869	261,534217	419,716943	242,883198
508,285095	320	424,777868	878,156727	323,423893	262,894003	406,687946	239,638231
510,229126	320	413,854286	841,579956	320,919162	250,311904	400,939696	229,683256
512,17157	320	413,640574	818,026356	311,006464	247,151319	396,085289	222,26477
514,112427	320	408,617713	806,22605	308,507447	247,176098	388,445936	213,519497
516,051697	320	398,793719	759,856618	300,95442	231,699433	381,363377	205,74538
517,989441	320	385,959456	735,710828	295,954462	223,451019	369,832553	189,292911
519,925598	320	382,015377	711,131008	291,008682	221,698501	360,458856	189,449676
521,860168	320	377,297804	687,652984	284,358805	209,796953	356,628496	187,103239
523,793152	320	376,055675	665,214685	280,622792	203,25288	349,782053	173,144909
525,724609	320	364,956075	637,898762	268,035122	200,176297	350,790223	165,7966
527,654419	320	367,818821	636,740521	268,607772	194,132873	341,22609	162,106359
529,582764	320	366,557485	609,353583	261,128119	183,411456	340,382895	150,522536
531,50946	320	350,134233	582,166443	260,078066	179,652014	334,781262	153,609165
533,43457	320	353,338433	572,11289	263,412468	177,075017	321,73937	147,467851
535,358093	320	345,977511	558,031365	255,451956	176,854309	315,571318	136,49311
537,280029	320	344,426053	540,500144	248,91653	166,703826	320,059339	137,124174
539,200439	320	330,394759	514,851278	242,237179	166,309712	309,363813	132,293
541,119263	320	319,586666	497,528237	235,709364	154,39772	305,517829	129,809974
543,036438	320	325,478739	488,728641	234,582296	159,037488	290,525391	122,334614
544,952087	320	321,752202	473,838787	224,840686	152,347659	284,992492	119,298098
546,866211	320	307,05007	453,562025	217,625658	148,9327	282,261158	112,69923
548,778687	320	293,054419	440,853322	214,03153	141,878212	274,649239	109,126104
550,689514	320	294,746549	419,614678	212,823889	141,110228	267,502673	106,364872
552,598816	320	293,662022	405,704649	200,738847	131,039235	260,877963	101,038296
554,506531	320	289,765272	399,39057	197,635943	132,600461	263,247181	102,929181

This journal is © The Royal Society of Chemistry 20xx

Please do not adjust margins

556,41272	320	281,367234	382,416229	201,597753	125,320908	254,076409	92,9750881
558,317261	320	274,476071	369,708894	197,830489	123,059778	249,013167	89,2157764
560,220215	320	266,108018	354,888821	189,773924	123,725898	239,771343	89,3369878
562,121582	320	257,464773	350,640421	185,405291	113,924932	242,096374	83,3781418
564,021362	320	251,59301	330,817157	184,31875	108,103247	228,949474	82,3947794
565,919556	320	250,076303	329,787588	175,851269	109,162639	222,971934	79,9704907
567,816162	320	243,734438	316,273055	172,776801	111,45008	215,125763	75,3174847
569,711182	320	235,252422	304,207913	166,682393	100,662083	210,371786	70,771087
571,604614	320	238,72748	298,656233	166,003872	94,7402103	202,30235	71,9656966
573,49646	320	230,614515	281,357173	162,965867	94,0266217	202,345661	69,4307673
575,386719	320	223,439812	282,728365	159,793374	91,5364647	193,644858	65,5562118
577,27533	320	214,214439	264,941373	154,442768	89,9237943	189,919691	65,4706523
579,162354	320	212,175825	251,800787	149,663547	86,0658073	185,130281	59,6651474
581,047852	320	203,982284	245,001975	145,465566	85,0753541	182,081885	57,3437879
582,931641	320	203,75904	237,842734	143,004222	82,6128668	177,155172	56,4514282
584,813965	320	196,991839	232,622738	133,612334	80,9176719	171,660995	54,247901
586,69458	320	191,055098	222,631405	130,411367	75,4188904	165,643361	49,2496265
588,57373	320	182,884028	214,738746	130,295466	72,5284138	158,691692	48,8282469
590,451111	320	176,430391	202,868843	124,166337	69,5289372	155,554089	48,4720049
592,326965	320	171,328524	194,943884	120,336838	71,3306346	152,411441	46,4731449
594,201233	320	169,542127	189,405317	117,530482	69,2658924	147,19924	43,6237905
596,073914	320	161,04676	183,634359	115,572163	63,320365	146,744889	43,3567425
597,944946	320	156,217028	175,506766	112,540022	59,5142062	140,400863	40,1650638
599,814453	320	155,963116	174,272616	107,482939	59,2560896	128,84354	38,206034
601,682251	320	158,859261	166,404313	108,278807	55,9683253	127,490335	35,0946651
603,548523	320	147,469984	151,977625	102,580315	57,9426178	126,634981	35,7151577
605,413147	320	146,407143	158,452654	99,2714487	54,2435932	125,83923	35,5388222
607,276245	320	136,56251	147,664234	98,6567548	56,0167259	118,22268	38,0276575
609,137695	320	137,62089	146,506165	98,2700697	49,6757223	117,225863	34,3903225
610,997437	320	135,226014	140,882699	95,9131758	48,7298839	116,413419	34,3427937
612,855713	320	124,369718	135,946229	90,7272367	49,6761608	110,186019	32,0418151
614,712341	320	127,729823	131,195445	87,3657025	45,6205407	106,059302	29,4605712
616,567322	320	120,996079	122,3709	86,1784216	45,7859987	107,222022	27,2860763
618,420715	320	115,459443	118,469801	84,9056729	42,0030816	101,777356	25,6982774
620,272522	320	115,821814	119,189108	84,3849452	41,9754682	94,1570854	29,4818483
622,122681	320	113,494508	109,96041	80,818582	43,1204495	94,3598771	26,3922846
623,971313	320	104,996705	107,325776	74,9324441	38,6561362	93,6778451	26,5816226
625,818237	320	99,3978811	108,928422	76,8790865	39,8704047	88,0296138	24,6880236
627,663574	320	100,753773	97,9008595	67,6827466	35,8046395	85,9383703	25,9091069
629,507324	320	97,4516648	96,1282007	70,6275495	37,0497337	78,9965579	25,4449758
631,349365	320	99,8992133	90,3796006	68,6601863	33,0365935	81,4323675	21,9106508
633,18988	320	91,4408507	89,6383188	67,7489191	32,7563968	79,9543276	21,8431032
635,028809	320	87,4593593	86,8483359	66,9943864	30,117843	76,4529764	20,762253
636,865967	320	86,4070223	85,1994445	61,4995305	30,285109	72,1617114	21,1518575
638,70166	320	84,904314	79,2850122	56,5248179	30,659656	70,1725901	20,5690078
640,535645	320	82,3849793	79,27045	59,4951552	31,7275121	71,472213	16,6423826
642,368042	320	77,8288695	75,3081839	56,46318	28,0637291	65,4657432	17,6145811

Please do not adjust margins

644,198853	320	76,9201367	69,7296231	53,3549847	29,6800854	61,2970329	15,9827442
646,028015	320	74,3743805	76,9949821	53,4167777	26,5992338	62,6864776	16,3594917
647,855591	320	72,6656902	65,2314305	52,5552733	25,8689532	65,4144617	15,7554975
649,681519	320	68,8424864	60,2862033	48,5038969	27,0911492	58,5379091	17,3998698
651,505798	320	69,0043879	64,5210677	45,9235299	25,872522	56,9641897	15,4229712
653,328491	320	66,0653497	61,2051973	44,7316243	22,6779208	55,4413572	10,998567
655,149536	320	65,0886496	60,6976272	43,1907653	21,5431466	56,7384952	13,949762
656,968994	320	59,9824568	57,894964	44,5909463	21,9988336	56,9730005	12,1803631
658,786743	320	56,8331902	55,9635911	42,7180435	22,9639737	53,5609104	11,9940152
660,602905	320	55,3684111	50,5604648	41,3428004	18,9563474	48,7624087	9,93692571
662,41748	320	58,2126032	52,6411541	37,69702	19,041503	47,1261007	12,3229189
664,230469	320	55,9745564	50,3925487	40,2482177	19,9463067	47,4669599	8,89000897
666,041748	320	53,2232412	48,0036964	37,7162649	16,1351041	44,697882	11,7512782
667,85144	320	49,1397949	47,0162225	38,6907991	18,0463193	39,5916664	11,4114688
669,659424	320	48,9905087	44,9931386	34,5310858	18,0830546	43,1654144	9,81892555
671,46582	320	47,1688212	45,7877156	32,4449895	17,3525145	38,3391091	10,2022169
673,27063	320	49,1074835	38,7025678	33,0732296	16,0419062	41,3729006	9,61766873
675,073792	320	45,5621021	39,6555428	31,8817323	12,808011	34,2925825	5,38124865
676,875305	320	42,5416651	40,6517724	27,8598534	15,5675713	36,421112	11,5111865
678,675171	320	40,5402406	39,9053026	26,2325888	13,1843126	33,6634801	6,63249828
680,47345	320	39,7568017	36,7400283	32,6089333	12,4214348	35,0184403	7,12761955
682,27002	320	36,6224613	34,7282829	30,4565686	12,2126712	30,4796744	8,28163245
684,065063	320	36,2391129	33,5812599	29,9264411	13,2638424	30,5809832	5,28764668
685,858398	320	41,5606078	32,3329735	26,276069	10,4893476	31,9850424	9,32041368
687,650024	320	33,8884724	30,3422742	25,417002	15,0538835	28,9796475	5,92560275
689,440186	320	33,9668041	31,2994377	25,4757523	11,0312198	29,0466327	4,17014648
691,228577	320	33,8567094	26,2436066	24,0934034	10,0217824	24,1116817	5,18363982
693,015381	320	30,8912835	28,3435491	20,8489695	10,5558245	30,2793853	7,22473649
694,800476	320	31,391939	24,3973191	21,0979541	11,316069	26,8376239	4,43513364
696,583984	320	28,8389179	22,8372203	20,8002805	11,872382	26,5628258	3,94411612
698,365845	320	27,0004864	26,0999087	21,4985654	7,93716142	23,051652	7,27246206
700,146118	320	28,1361298	24,534279	21,5844304	9,38269276	23,5294487	6,14863847
701,924683	320	29,9208994	21,0181241	19,6038474	9,67681223	22,8454829	7,32957835
347,741211	325	41,7698244	393,333661	47,4188663	125,830488	72,1116128	68,622175
349,808533	325	46,0522304	472,717767	56,013503	137,202759	76,4365817	72,4904192
351,874329	325	51,1567443	534,637678	65,2981323	150,603	101,360833	89,4419623
353,938568	325	65,0579009	623,972606	62,5831732	160,411116	114,401701	110,650826
356,001343	325	71,2532152	711,61564	83,5532852	192,466894	124,239758	133,118091
358,062561	325	97,4622198	791,671116	98,0115392	206,973076	156,605635	161,314426
360,122253	325	106,047601	902,884655	111,573449	221,694995	173,42981	204,074521
362,180481	325	123,380785	990,006124	124,765514	255,766719	196,627515	230,700115
364,237152	325	137,398553	1072,35302	149,271015	259,237571	216,786832	242,920983
366,292328	325	149,539186	1159,71169	161,188462	285,734296	226,497829	273,023175
368,345978	325	158,255304	1248,52037	166,062573	302,373442	248,112367	295,738013
370,398132	325	159,146982	1342,46376	184,81292	314,618489	255,662831	304,534943
372,44873	325	198,488518	1455,82603	191,251098	338,722192	288,969626	356,602848
374,497803	325	187,673731	1562,42772	211,729016	361,118604	312,761203	374,796727

This journal is © The Royal Society of Chemistry 20xx

Please do not adjust margins

Please do not adjust margins

376,545349	325	206,988331	1652,13076	218,981382	376,715609	340,722751	409,17331
378,5914	325	232,700989	1720,63898	228,380615	400,374432	347,386304	452,37675
380,635925	325	250,768017	1800,17439	246,332741	416,049718	357,490606	479,059561
382,678955	325	273,61287	1911,70643	273,944238	442,918906	393,985445	526,092256
384,720398	325	284,547312	1998,82649	285,026943	450,767853	413,23689	571,511906
386,760376	325	301,549818	2096,64883	301,813598	475,793926	423,351497	587,38767
388,798767	325	322,286586	2144,91986	301,766638	483,521162	454,061508	627,934832
390,835663	325	321,711307	2218,76738	322,592724	505,931966	466,293814	652,906359
392,871033	325	344,243653	2239,76211	340,241185	520,885754	479,29731	694,650721
394,904877	325	359,134525	2323,31362	350,181188	533,340983	492,919455	718,64762
396,937195	325	362,810459	2393,24041	367,36625	548,427134	512,237506	745,271176
398,967957	325	384,071416	2435,5635	370,24624	568,682084	527,009372	762,952283
400,997253	325	404,269213	2476,43859	379,771808	573,41213	532,396647	793,455678
403,024963	325	407,060062	2500,25257	399,181038	588,262896	533,668461	799,808671
405,051147	325	436,234552	2538,79956	408,70599	598,399998	546,227076	832,447824
407,075806	325	437,850238	2568,30006	426,162994	606,404617	569,431721	838,386685
409,098938	325	451,03842	2584,06055	439,604217	613,472575	568,261704	842,148741
411,120544	325	452,119187	2557,06094	442,062113	614,779611	576,792859	845,328643
413,140564	325	489,27763	2607,99023	455,510562	618,183282	585,039141	872,937108
415,159119	325	488,838967	2586,66649	467,229898	630,979611	589,232337	846,690933
417,176086	325	500,34544	2601,51173	457,783569	615,84689	599,489332	865,710522
419,191528	325	499,445597	2576,60581	478,350652	615,708611	605,687226	838,05529
421,205444	325	508,345892	2565,90483	477,272247	621,078666	615,736508	841,713949
423,217834	325	514,814909	2540,79717	482,429648	619,343798	611,063315	844,032322
425,228668	325	535,688879	2533,31457	486,80198	627,788686	607,320822	830,601303
427,237976	325	532,755077	2513,30118	483,85476	616,022952	607,485739	804,231242
429,245728	325	520,925869	2456,08939	503,596599	607,153009	604,370872	801,183387
431,251953	325	544,474817	2458,8395	494,823936	601,851228	612,746797	781,630869
433,256592	325	541,743696	2422,6803	501,915124	592,758606	614,713003	775,805088
435,259766	325	550,107882	2402,22671	496,350349	584,803471	598,736995	773,536638
437,261353	325	553,309013	2388,49034	502,859629	580,632757	589,892185	749,448875
439,261383	325	561,809956	2330,56737	520,592933	578,931618	594,970758	753,853638
441,259888	325	550,790474	2288,06599	493,790888	572,983482	600,489276	735,053537
443,256836	325	566,111207	2233,68745	509,031008	548,445621	583,821087	719,481208
445,252258	325	555,284372	2190,04488	484,281	533,131758	584,024213	693,23327
447,246094	325	552,54181	2136,75215	489,882547	521,604445	568,324971	684,984684
449,238434	325	543,290702	2105,76199	487,963633	519,431423	565,257274	650,034623
451,229187	325	543,783184	2028,98426	482,712967	508,174888	560,420451	643,94221
453,218445	325	545,861162	1981,07121	468,055661	496,505845	559,125431	620,224478
455,206116	325	528,976987	1941,63379	458,803503	479,84262	550,214479	603,56352
457,192261	325	539,755253	1920,01069	469,074134	478,362498	536,900097	579,831467
459,176819	325	525,133444	1859,94213	464,502964	467,295094	541,642631	570,624078
461,15979	325	519,939736	1801,7857	448,782182	456,449743	538,135099	560,087349
463,141296	325	520,528908	1747,77723	446,08703	449,831922	534,751584	540,8712
465,121216	325	519,960153	1698,26199	437,43603	435,181241	519,776459	513,140719
467,099579	325	513,254086	1660,13531	431,840773	429,722083	506,913567	504,655205
469,076385	325	498,136915	1600,64399	425,532137	419,890469	505,397727	488,074635

Please do not adjust margins

471,051636	325	507,0606	1571,90399	422,033343	412,314354	492,831476	465,412331
473,02536	325	498,953725	1522,20224	418,117923	402,158313	496,119946	456,286148
474,997498	325	504,544977	1489,87229	424,453339	386,66774	497,794973	439,140925
476,968079	325	491,824694	1455,77893	405,535852	380,409909	491,766804	420,416294
478,937134	325	494,577475	1407,7359	404,393835	363,967541	480,745109	411,033379
480,904572	325	494,953948	1352,9692	394,540674	361,517199	466,856113	397,083789
482,870453	325	487,043689	1309,46639	405,821507	353,237724	481,369309	377,433015
484,834808	325	489,24622	1287,53438	402,813376	355,234827	470,851285	379,605432
486,797607	325	491,696753	1247,42213	392,8054	341,436507	464,528349	360,081361
488,75885	325	486,848274	1218,95874	377,463752	328,641212	460,971054	347,236582
490,718506	325	482,381686	1191,73101	379,35903	330,478232	465,554307	333,488214
492,676605	325	473,623584	1148,74957	376,046203	318,418032	455,494748	329,13701
494,633118	325	468,872623	1106,44379	372,054702	311,914767	456,32201	313,814832
496,588104	325	466,815103	1083,95313	370,468808	303,321705	443,489409	299,670937
498,541504	325	467,786686	1045,3637	366,450551	289,85317	441,165845	297,688782
500,493347	325	461,617789	1024,52122	352,293551	289,947102	430,212558	280,758101
502,443665	325	448,961117	973,370677	353,199474	285,450462	434,543227	272,742498
504,392365	325	449,577689	948,934303	351,33374	269,618399	426,905445	258,333628
506,339539	325	432,973306	915,687788	339,283856	267,632027	416,363559	257,20935
508,285095	325	438,632513	881,017513	331,139771	256,359088	405,111666	247,354166
510,229126	325	423,825643	864,18317	329,317981	245,782085	411,836197	232,198219
512,17157	325	426,402673	822,906863	324,460259	242,081646	393,466408	229,192192
514,112427	325	424,015641	805,828486	316,706391	235,234647	394,638639	213,67774
516,051697	325	403,450307	781,960343	309,296917	231,494507	383,034483	205,612316
517,989441	325	393,391894	747,295982	302,907628	218,38981	374,036647	196,627598
519,925598	325	393,37469	704,481639	298,982307	217,530347	368,202646	185,111136
521,860168	325	389,019317	691,98936	292,394538	204,072597	364,195168	188,219765
523,793152	325	389,902875	670,194418	282,09009	200,411903	359,201621	177,619656
525,724609	325	373,709325	656,387184	275,966435	195,134318	349,811774	167,609952
527,654419	325	368,825413	634,759016	272,037827	193,867245	347,401895	160,943165
529,582764	325	366,152243	605,134182	265,616339	187,376428	334,601641	158,02046
531,50946	325	363,087543	597,979362	268,017837	177,777581	341,492394	155,648867
533,43457	325	357,337337	575,630064	263,227477	177,326958	338,338215	148,722647
535,358093	325	355,17197	560,561702	255,038163	168,654162	324,208433	141,325401
537,280029	325	346,025482	535,556215	249,780685	169,736803	318,866044	139,376075
539,200439	325	334,381795	526,974946	247,187872	161,590486	306,844125	130,774541
541,119263	325	332,641012	509,281821	243,48929	157,281984	304,478181	126,392829
543,036438	325	319,742921	484,500511	235,922378	151,143506	296,289192	123,611706
544,952087	325	321,249342	473,067484	229,455898	146,087973	297,064574	117,550515
546,866211	325	309,678197	451,970495	228,598457	144,370549	282,174681	114,10569
548,778687	325	307,985032	442,372216	217,888916	142,901276	275,391878	106,950563
550,689514	325	298,258502	425,593491	220,336733	132,192376	267,065242	108,019613
552,598816	325	290,452076	411,671766	213,878362	129,056691	267,306994	98,9770825
554,506531	325	291,466008	399,609317	209,759326	129,415481	258,193525	100,58381
556,41272	325	283,617772	386,743337	208,29525	120,878551	251,572525	94,1296677
558,317261	325	274,623944	365,245613	198,485725	120,157726	250,027774	92,3797608
560,220215	325	271,664884	353,407699	194,692636	117,588412	243,648924	85,5881483

This journal is © The Royal Society of Chemistry 20xx

Please do not adjust margins

562,121582	325	262,572275	346,248957	192,292126	109,322052	237,713377	86,3819067
564,021362	325	254,981466	328,486854	185,980376	104,670134	229,470239	81,9288343
565,919556	325	252,964196	321,714079	177,701368	107,569209	229,939057	77,0188206
567,816162	325	242,312068	306,568329	178,960953	104,017131	217,542853	75,8925612
569,711182	325	235,668441	308,326961	172,955135	100,164876	211,13051	69,6611525
571,604614	325	235,46307	293,168994	168,889587	91,5452471	214,927557	66,1920711
573,49646	325	230,338576	281,876357	169,597238	95,4107559	202,126535	69,1264069
575,386719	325	222,62977	269,340893	162,888928	93,4606301	194,228513	64,5718681
577,27533	325	212,433222	264,628524	154,725946	87,4639229	192,834782	63,0114012
579,162354	325	209,855014	254,724795	154,75802	84,6211314	191,053784	59,574924
581,047852	325	205,775032	245,465335	150,352142	81,7454949	179,162479	58,4280185
582,931641	325	199,224271	231,942285	142,416674	79,2750804	177,336955	54,6827786
584,813965	325	198,660482	225,867833	137,407488	77,9714622	174,993664	55,3600098
586,69458	325	187,267076	223,305623	137,495015	76,5844449	168,604469	52,727813
588,57373	325	182,809701	209,599916	133,871626	69,3809123	164,158277	50,8377534
590,451111	325	183,472133	203,060778	125,891107	66,93889	156,87443	46,3847875
592,326965	325	176,183321	188,79934	121,080209	67,4518558	150,84314	45,9006976
594,201233	325	171,330557	187,519131	119,77831	61,9989664	145,883606	47,9642967
596,073914	325	172,914143	179,776096	116,155993	65,1504187	139,743652	41,8116035
597,944946	325	161,967513	177,479359	116,61218	57,8966133	141,528371	42,5577709
599,814453	325	159,789005	170,670619	112,080624	58,7685958	133,342234	38,9931929
601,682251	325	156,615994	162,554619	110,811333	55,8329555	129,00045	38,5669696
603,548523	325	149,470796	156,962786	105,111902	53,3685821	128,947105	37,4832305
605,413147	325	147,211381	149,012093	102,708102	52,0520033	125,161906	35,5320194
607,276245	325	143,093056	146,561477	102,774117	52,6405877	119,51866	35,3671135
609,137695	325	136,424467	141,556858	100,504736	50,0021191	120,775067	33,0975944
610,997437	325	128,457331	133,827556	96,0353804	49,0991161	111,773961	31,9430894
612,855713	325	126,833793	130,649028	91,0666979	45,6643321	104,798417	31,6218634
614,712341	325	122,709745	128,769794	92,0928346	46,7926221	108,654582	33,3759748
616,567322	325	120,494133	124,551465	85,271702	46,5074437	102,510965	28,5113938
618,420715	325	115,857994	118,358146	82,490977	41,8734991	102,213974	26,6572225
620,272522	325	117,899189	119,955098	78,2978267	42,7479284	97,158284	27,9434131
622,122681	325	113,768746	107,293315	81,3850995	41,9404697	95,4561675	24,6431649
623,971313	325	106,301952	103,869322	80,257108	39,4620449	91,6587507	26,2831095
625,818237	325	104,684571	104,731847	74,471618	38,259531	87,9459421	25,146477
627,663574	325	103,6215	100,610539	74,7157301	37,1928551	85,4926671	24,7388287
629,507324	325	100,372191	101,106085	69,6230992	37,83185	83,1790969	23,7254865
631,349365	325	96,7977259	92,8628279	71,1544249	31,5877892	84,6087371	20,5620959
633,18988	325	94,2810429	91,7091792	69,3806622	30,8614403	77,4914819	21,5210559
635,028809	325	91,8261535	86,4038423	64,4352063	31,1856635	75,6174759	19,2321745
636,865967	325	91,2862392	85,8557118	59,2835516	31,8074767	75,509258	20,2886789
638,70166	325	86,3003389	78,9254521	58,9732807	30,6801831	67,0799321	21,8964508
640,535645	325	78,9606059	77,1955003	56,334278	28,2709454	67,7543107	17,5595525
642,368042	325	79,9723652	76,4870327	56,5165833	28,4196154	65,315043	17,3523959
644,198853	325	76,5854556	72,6358475	56,5238607	27,6770314	66,2598003	16,9322052
646,028015	325	73,5704016	69,5032895	58,770251	27,1342082	64,2671149	15,9209616
647,855591	325	72,044303	64,9943144	48,1887683	24,1342271	67,3416204	16,1429372

Please do not adjust margins

Please do not adjust margins

649,681519	325	73,2387913	62,9180487	48,1008108	25,9965925	58,3441251	17,6106215
651,505798	325	67,8559379	64,5675071	46,7926658	23,5510366	54,059466	15,0247303
653,328491	325	68,1814516	57,4410551	47,0530828	21,1798077	55,6224824	14,3754568
655,149536	325	62,4736173	59,6335417	43,3760741	21,1399251	52,0872673	15,1257726
656,968994	325	58,6278751	58,1222821	46,0141212	22,1655214	53,5925217	12,6179629
658,786743	325	60,6763802	55,4574855	43,1760523	21,0934425	52,1999401	10,9175886
660,602905	325	59,1668794	54,4087434	40,2123773	19,6078973	50,6701365	11,8848486
662,41748	325	57,7718755	51,3336605	41,4591107	20,0519313	47,6944013	11,4654362
664,230469	325	59,6688042	49,771929	40,1166583	16,6438446	47,1941281	9,23782142
666,041748	325	52,8711052	50,9337931	38,2873865	17,1506233	47,6375319	10,0852272
667,85144	325	53,0164454	46,1812233	38,6311003	16,6006248	42,1556883	9,87499923
669,659424	325	48,6973356	46,3948044	35,603401	15,6770406	41,2841863	10,0143189
671,46582	325	49,2262142	48,3573855	34,683031	15,6930699	38,6910914	9,78587828
673,27063	325	46,5333125	40,9764391	31,0969073	15,6342969	38,2414162	8,74595435
675,073792	325	43,1292142	36,3598677	33,0815344	13,3749909	38,6765204	7,92252826
676,875305	325	43,1210378	35,7511042	29,3467786	13,1315101	33,6096682	11,0414306
678,675171	325	44,9349252	34,79364	33,3211048	14,8204811	33,6150095	8,64249287
680,47345	325	38,1595137	36,932506	30,4108474	14,3275697	33,7097432	8,8757821
682,27002	325	36,9630124	34,2839698	29,0688202	11,8400628	31,7728025	8,42510483
684,065063	325	36,601088	34,7612817	26,6240527	11,879417	33,8177803	4,34731286
685,858398	325	40,1333467	32,1107234	25,4250087	12,733526	30,5584813	7,00707425
687,650024	325	36,4107685	30,6871067	28,1164837	10,6825088	28,5253049	6,16751349
689,440186	325	35,1567829	29,5423059	27,2096988	13,2623285	27,1312617	6,78782526
691,228577	325	34,44382	29,6784718	25,1570465	10,1038052	26,6577097	6,66989362
693,015381	325	35,2629721	26,3391438	22,2964947	12,3260647	27,9454042	6,68675025
694,800476	325	34,034297	28,8926754	21,1946506	11,5909273	26,3524001	4,74032949
696,583984	325	31,3546443	26,0716399	21,4024945	9,5551721	25,6013014	5,12557445
698,365845	325	28,3721667	25,6539144	22,6890694	9,9505604	24,4448537	6,48616116
700,146118	325	28,2388572	21,8128159	20,1938867	8,38692087	22,9392082	5,28337495
701,924683	325	26,9857532	23,8760342	20,8895549	8,17154137	26,2462798	6,04373583
347,741211	330	32,5899658	302,532554	40,175103	132,086045	56,163492	53,6140373
349,808533	330	43,1241564	356,647436	51,7609239	141,859615	69,6366708	65,2099291
351,874329	330	48,0220109	420,11681	54,9111982	138,820017	86,8447588	68,84223
353,938568	330	55,4055976	493,773168	62,6595137	139,376384	95,5740862	87,3142904
356,001343	330	65,2580123	580,71398	72,1611891	166,024884	114,416798	103,544868
358,062561	330	78,8863812	668,314465	88,549303	167,793915	131,531431	117,453987
360,122253	330	92,5366628	720,785014	88,9928608	185,594504	154,872128	141,355473
362,180481	330	110,334957	828,112642	100,197921	211,126498	172,750655	157,249926
364,237152	330	122,204794	909,746284	129,058745	222,216674	183,698291	198,221453
366,292328	330	149,278095	1026,77364	153,929591	242,844123	221,32039	237,529799
368,345978	330	169,049423	1116,86252	168,732834	283,907126	243,945062	282,997379
370,398132	330	183,23765	1221,29785	180,336589	302,293131	272,685667	295,38509
372,44873	330	202,830556	1322,66301	201,519839	321,623218	300,845558	325,864235
374,497803	330	213,277562	1440,27888	208,89798	344,254491	321,493616	344,880575
376,545349	330	237,125973	1518,97789	226,68753	363,149978	332,07562	371,12455
378,5914	330	241,529048	1592,7148	236,829172	361,915911	334,464693	399,693234
380,635925	330	255,167844	1664,49272	252,693681	397,997562	360,041147	444,611635

 This journal is © The Royal Society of Chemistry 20xx

Please do not adjust margins

382,678955	330	273,526115	1805,54242	271,065226	414,423125	381,797018	479,49793
384,720398	330	291,473422	1895,2653	283,214382	436,381723	408,196444	502,088266
386,760376	330	311,812753	1975,35815	303,335767	445,218291	430,164617	533,694805
388,798767	330	333,858971	2061,73716	313,940113	457,114331	458,74806	572,906247
390,835663	330	338,24596	2117,33893	324,587703	467,348237	464,894958	600,035048
392,871033	330	368,319131	2205,68523	349,882541	494,673337	478,676589	631,445871
394,904877	330	380,505698	2268,37384	361,735037	509,95163	488,223623	655,017459
396,937195	330	402,781787	2363,28336	374,859132	537,762376	512,570752	695,643711
398,967957	330	419,390119	2384,0182	387,046919	530,914727	528,252179	704,639086
400,997253	330	437,834508	2449,15617	396,519877	562,00368	541,366562	744,024143
403,024963	330	451,518472	2494,28064	421,345246	566,632133	563,416193	762,267658
405,051147	330	465,746143	2572,06398	427,486703	589,423753	560,150501	770,256902
407,075806	330	479,004911	2598,20377	448,885243	593,327712	580,023975	787,230551
409,098938	330	491,126557	2607,07251	461,46547	597,444512	593,725797	795,26577
411,120544	330	515,635818	2660,90363	472,335246	609,576896	615,792766	790,084427
413,140564	330	525,421279	2662,03815	478,458192	613,514747	616,220073	814,249968
415,159119	330	533,73357	2677,98195	488,251766	617,507799	623,584735	824,811786
417,176086	330	539,331433	2667,20542	494,276189	615,839477	630,159066	812,457455
419,191528	330	547,466896	2638,18722	515,151317	618,746206	627,852508	821,431282
421,205444	330	571,638844	2647,8415	512,89501	624,978849	644,558436	805,754136
423,217834	330	569,626988	2653,56718	519,123316	612,587747	641,24716	807,976544
425,228668	330	580,593233	2620,41788	518,114108	607,153965	638,133495	806,541348
427,237976	330	591,683008	2604,66778	530,690618	618,62109	638,587484	793,656654
429,245728	330	594,574806	2559,14878	518,787626	608,368829	635,244224	784,616407
431,251953	330	603,70992	2556,25104	530,519593	607,259319	646,603141	771,963701
433,256592	330	596,900755	2534,16321	541,728283	599,25549	643,270739	762,211173
435,259766	330	606,265319	2524,39572	548,019914	592,783917	639,359154	756,011112
437,261353	330	610,341633	2504,84909	537,279361	586,637151	641,799559	739,527147
439,261383	330	611,477374	2437,39539	547,793887	589,320582	632,500469	739,530584
441,259888	330	615,449648	2420,8835	534,005143	580,840292	645,373928	736,259273
443,256836	330	611,912294	2358,44041	531,207978	566,9639	619,345399	697,014797
445,252258	330	609,309011	2304,06244	524,666079	554,332688	627,602334	699,313575
447,246094	330	597,738059	2261,45474	528,92258	536,857927	612,899569	683,44968
449,238434	330	593,625179	2216,23631	520,564888	533,362891	597,198301	650,618397
451,229187	330	594,498486	2143,39603	515,138265	524,083921	595,972557	640,781944
453,218445	330	575,584008	2108,5418	506,970879	517,725402	579,499559	620,94206
455,206116	330	581,516496	2063,73065	501,25929	491,209307	577,605738	604,621704
457,192261	330	575,614612	2026,92569	493,242014	498,05887	571,839113	590,276934
459,176819	330	572,862714	1969,93419	488,30995	477,606165	576,246124	572,980376
461,15979	330	560,823107	1910,8624	475,704019	467,930046	560,33099	555,680672
463,141296	330	558,080396	1840,99759	481,642526	460,005483	559,389365	552,126374
465,121216	330	553,987765	1798,97836	474,848334	444,748824	537,946809	522,754972
467,099579	330	549,389166	1745,52442	467,071999	442,260466	538,417952	500,061051
469,076385	330	544,658206	1698,10279	454,586652	427,542861	538,576472	500,441707
471,051636	330	530,679551	1640,06013	457,141844	417,78445	522,088369	468,790301
473,02536	330	540,638034	1605,23457	439,231628	408,437287	519,036819	472,9689
474,997498	330	534,645764	1554,26278	448,932052	399,339335	519,652626	440,248783

Please do not adjust margins

476,968079	330	526,287633	1506,72123	434,223985	392,508913	501,93107	428,788596
478,937134	330	515,664708	1469,90867	428,642785	375,489008	492,663668	420,116713
480,904572	330	518,345302	1410,99611	432,523013	369,342875	493,517865	402,411949
482,870453	330	514,359547	1386,19188	416,893978	359,120293	492,953489	396,937522
484,834808	330	509,891248	1352,20266	408,263197	350,817009	486,289741	381,118412
486,797607	330	498,27362	1312,24005	414,725441	343,139038	495,026381	368,128189
488,75885	330	496,710349	1279,42976	403,720056	332,807842	477,521423	360,242658
490,718506	330	505,017266	1233,21914	402,945134	325,578883	477,550708	343,405237
492,676605	330	498,741803	1193,06502	399,252538	322,365373	467,879991	332,534
494,633118	330	497,51267	1154,1484	393,07344	309,155494	468,756195	319,428497
496,588104	330	476,864025	1112,95482	382,760573	299,788018	457,737768	319,068422
498,541504	330	487,099473	1087,27923	373,896496	288,483084	456,769063	304,238161
500,493347	330	476,725814	1047,22701	368,703899	283,857929	441,846346	286,498643
502,443665	330	466,263514	1015,20656	361,904223	270,931487	444,227968	278,840645
504,392365	330	460,884775	986,713087	358,676068	264,785608	439,965601	261,587228
506,339539	330	452,198191	947,479206	348,132976	259,642349	427,505879	258,068476
508,285095	330	445,136297	907,014225	352,131069	257,184193	423,756495	244,453506
510,229126	330	438,39044	885,313583	338,775119	245,595312	406,615805	239,168549
512,17157	330	428,140235	853,096053	339,190013	238,685812	402,703236	224,789985
514,112427	330	428,543351	825,251599	325,888534	239,679409	401,073852	220,055413
516,051697	330	415,070348	787,13146	321,596407	222,270393	391,530834	211,179039
517,989441	330	407,87295	761,355406	314,538027	217,420697	384,955833	202,098453
519,925598	330	410,475704	720,849365	302,155386	213,37895	363,387966	188,02459
521,860168	330	403,187343	697,098771	298,189761	197,634845	355,23861	185,692387
523,793152	330	390,477904	677,471801	291,553467	199,864667	358,186226	176,016561
525,724609	330	376,744729	656,924304	289,445976	191,374238	356,19547	172,426305
527,654419	330	379,865271	634,37031	277,48787	185,638792	354,337716	162,051498
529,582764	330	387,806327	622,55413	280,917075	181,543751	334,745399	159,861504
531,50946	330	359,3096	607,525557	277,626184	179,939873	336,928289	155,193044
533,43457	330	363,651965	582,365601	271,265431	172,847402	327,553141	147,060151
535,358093	330	357,779376	564,515591	265,380747	172,612918	322,598321	139,559616
537,280029	330	352,771476	548,902221	260,157891	163,148005	317,434057	136,372717
539,200439	330	341,780451	533,318953	261,445688	157,267469	314,519112	133,766185
541,119263	330	338,824914	504,936576	247,381833	158,652958	301,134454	128,736285
543,036438	330	331,306955	490,049236	241,644816	149,468197	294,304031	119,471563
544,952087	330	326,242774	480,686856	235,483031	144,108831	290,901317	120,735247
546,866211	330	313,791612	460,164574	235,979842	142,047357	288,457008	113,333239
548,778687	330	311,971068	441,531405	230,094847	137,458005	279,079418	106,08449
550,689514	330	302,0417	427,128824	216,930538	131,974659	272,575027	109,181033
552,598816	330	293,341626	412,564084	221,218172	125,195792	267,513306	103,468954
554,506531	330	294,799944	403,780324	212,223546	124,548399	261,657277	95,9245771
556,41272	330	288,980455	389,197059	210,628432	121,528896	243,738846	94,0879512
558,317261	330	279,880899	371,787428	194,508203	115,0944	248,104101	91,8199676
560,220215	330	273,190672	364,221533	204,73006	115,904592	240,019398	86,4030263
562,121582	330	259,854735	352,164159	194,635651	110,680374	235,742297	82,4227067
564,021362	330	262,237109	336,675555	187,51203	103,353259	228,65758	82,8138993
565,919556	330	259,795397	329,999394	181,524658	100,950635	224,256891	79,6258361

This journal is © The Royal Society of Chemistry 20xx

Please do not adjust margins

Please do not adjust margins

567,816162	330	251,85878	310,863387	183,564833	102,051938	221,218241	75,3702828
569,711182	330	243,962216	301,458411	175,847465	97,2682327	211,306613	72,817573
571,604614	330	239,409724	294,946388	172,908017	94,4757854	206,302125	67,3095832
573,49646	330	232,929283	282,256947	170,180519	87,3635315	201,341044	69,8283212
575,386719	330	222,647279	274,352625	164,084003	85,9933351	193,399127	66,0846638
577,27533	330	228,555191	266,973687	159,574823	87,0748464	190,881325	60,6451489
579,162354	330	209,073352	249,246613	151,45804	83,4427367	184,486191	60,4169446
581,047852	330	208,491901	248,624644	153,413895	77,1025995	178,252241	60,2547382
582,931641	330	204,382207	236,70773	143,350983	77,2056089	173,009989	57,0781951
584,813965	330	196,563762	227,222983	142,999424	73,487797	169,126798	55,6840874
586,69458	330	191,158685	217,158424	142,394603	71,2265882	162,500091	53,7897522
588,57373	330	181,762869	218,595333	135,763123	69,309628	162,614858	47,2322569
590,451111	330	178,161635	203,696325	132,064871	66,8939069	156,532254	47,8671402
592,326965	330	181,705765	193,007693	122,078967	67,8398956	151,254156	45,6451948
594,201233	330	178,244232	184,836274	122,914104	60,9389417	143,145855	43,1599762
596,073914	330	168,992049	184,135826	118,75247	62,5667168	144,725364	39,3948471
597,944946	330	159,590783	175,797755	117,033108	58,6369024	139,215292	40,1099611
599,814453	330	157,924609	170,446293	114,503881	56,4583728	134,913696	38,5168919
601,682251	330	153,164345	164,694209	107,709615	54,3979296	129,563352	38,1059678
603,548523	330	152,195077	157,293274	107,905567	54,0675724	123,408901	38,6633161
605,413147	330	142,447555	154,894436	105,271895	51,7357671	123,09293	33,1749188
607,276245	330	144,147205	148,591732	105,324574	52,1896344	120,243128	36,5218475
609,137695	330	140,837967	147,401531	96,5549868	47,9602043	118,671607	35,0863828
610,997437	330	133,022619	136,086666	96,6397643	48,4813905	114,96437	34,8372316
612,855713	330	132,964532	130,916617	96,7814368	41,99698	111,382325	32,406177
614,712341	330	128,622758	131,803868	91,9181849	44,4616193	111,041582	31,8543872
616,567322	330	120,739967	126,492134	86,8869621	45,3652785	103,671564	27,8358414
618,420715	330	118,347177	120,895805	87,7640049	41,5745889	97,8239428	25,6338359
620,272522	330	114,412974	119,543213	83,1240566	41,8777453	97,0655819	29,2204872
622,122681	330	110,572097	111,842646	82,443183	39,7081278	95,8757769	26,0654627
623,971313	330	107,099826	109,668558	75,9863508	37,0112923	93,8723152	25,4914533
625,818237	330	107,197858	104,796779	76,8274146	34,3408302	91,10153	25,8080713
627,663574	330	100,118734	100,317579	77,1792577	35,0523213	86,0529169	23,3654491
629,507324	330	101,810916	97,4331292	72,0038774	36,9698578	86,0405608	22,0673529
631,349365	330	94,904474	91,9425772	70,2037414	34,2687052	82,3935371	21,6484719
633,18988	330	94,5793896	90,5174687	67,089681	31,8327385	78,3199106	21,3719036
635,028809	330	88,8404725	86,9671116	65,1930953	29,7364987	80,131459	20,378333
636,865967	330	89,8577523	85,1360812	60,8126799	31,8644185	74,1275804	19,4284937
638,70166	330	84,5412034	76,7521956	62,1593433	28,7047012	74,1683394	18,8931322
640,535645	330	82,3358915	77,3949154	59,8502039	27,1658718	69,2256738	16,0507534
642,368042	330	79,1464947	75,405772	60,5796243	27,5276301	66,896452	17,6005848
644,198853	330	77,4496667	73,1520211	59,9418352	25,8284838	61,3258246	14,7900942
646,028015	330	72,9199985	71,3673357	56,1643813	26,1885042	63,047302	18,2074005
647,855591	330	72,4481814	69,4673635	55,7002301	24,0872268	60,0500644	15,4585203
649,681519	330	68,5257141	63,430106	48,3774472	24,9902692	60,3716833	12,8959838
651,505798	330	70,360873	61,9152776	47,2452898	23,4274506	56,7337673	13,6129988
653,328491	330	65,5682952	64,2208538	50,5058177	23,1655463	56,463178	11,6566735

Please do not adjust margins

655,149536	330	63,6836133	58,3102641	47,6746675	19,5610203	51,8620385	15,2644142
656,968994	330	57,552141	57,541761	47,7595994	20,4275424	51,2907537	11,8592362
658,786743	330	62,2245096	54,2112156	43,4827163	22,7810842	54,1305456	12,4782051
660,602905	330	57,7393391	55,2435313	40,875816	20,6872428	46,6852536	13,8599848
662,41748	330	55,2747054	54,5838565	42,6473723	21,2343854	48,9388216	13,3563016
664,230469	330	55,2700499	46,5048834	42,95882	16,1563237	45,7360235	11,0009973
666,041748	330	52,9783394	46,8175707	40,9878224	17,7815903	46,7313155	11,0210725
667,85144	330	49,5823761	47,174719	38,0179588	16,8017901	41,2594628	11,5070954
669,659424	330	50,0267394	46,011721	35,92174	16,034278	40,3127277	10,6172109
671,46582	330	47,5568063	41,5903787	34,0116727	18,3436258	37,2586151	9,94238491
673,27063	330	46,3626253	38,7627463	32,1905177	15,7682739	38,4404269	9,75188642
675,073792	330	46,5671709	38,0306802	33,9803198	12,5697199	37,7072624	8,1613795
676,875305	330	45,7516388	38,138693	29,7127474	13,3744137	34,126097	9,99866771
678,675171	330	42,5315811	35,6094532	31,3300452	15,913469	33,5549744	6,43694685
680,47345	330	44,6874183	34,8913548	32,1118036	12,3292861	33,0684773	7,92518421
682,27002	330	38,1390474	32,2390153	26,4483352	12,4876371	32,489347	7,60688011
684,065063	330	36,8743304	30,7230665	28,1585477	11,8330802	32,0173119	7,16960859
685,858398	330	40,6027452	32,128377	27,2434543	9,86522269	32,2634578	6,13154554
687,650024	330	35,0691713	28,6772286	29,3593231	11,7338504	25,2090834	6,60085436
689,440186	330	36,7797131	30,8381838	26,7519871	11,4116369	28,0618991	5,45539056
691,228577	330	31,6740611	24,9155388	23,8558922	11,3007481	27,2602268	5,69033272
693,015381	330	32,4545683	29,413943	24,499504	11,0957142	28,613823	5,00617736
694,800476	330	33,380173	25,4390394	22,8604923	11,6767818	24,9870557	5,23771423
696,583984	330	33,866454	29,8767758	20,6106414	10,7859977	25,6740723	4,44081023
698,365845	330	32,7864728	25,3784976	19,4942054	9,87575696	22,2193351	6,91431626
700,146118	330	27,7261436	23,7104244	20,8690091	10,1512768	23,2523362	5,76533331
701,924683	330	28,0696092	23,5647459	19,6473085	9,0053838	22,7492986	5,55127336
347,741211	335	31,627261	219,17926	32,240815	174,855744	52,0159967	50,5741626
349,808533	335	35,419573	267,966514	39,5940917	157,936363	63,7801819	49,8273915
351,874329	335	39,5287865	319,193975	46,2754478	151,167282	69,2220982	51,72558
353,938568	335	46,6707293	375,874167	46,3533687	145,584137	83,8197195	71,2987168
356,001343	335	55,9841134	450,792557	61,6435539	147,475377	95,7164273	77,6082656
358,062561	335	70,8046971	507,405174	70,4424761	149,751189	116,047185	92,0610141
360,122253	335	86,8449167	593,50883	73,3590903	162,542211	126,613583	107,526414
362,180481	335	96,7233531	661,066959	94,0628343	169,970761	147,086528	132,763825
364,237152	335	109,846999	756,675701	113,828103	182,954766	161,676201	147,387262
366,292328	335	129,731719	851,018743	129,84915	205,73105	192,684513	174,05243
368,345978	335	148,705302	948,538882	146,839788	237,34165	211,159438	206,192357
370,398132	335	157,796542	1043,73571	165,228068	253,708282	236,762848	234,311798
372,44873	335	192,883152	1147,14585	193,592135	290,439425	269,199483	288,005016
374,497803	335	218,926045	1259,19115	213,309336	317,486331	296,919814	311,885893
376,545349	335	221,801768	1357,49985	221,684739	348,208245	316,954071	343,737887
378,5914	335	253,08169	1443,87163	236,074547	356,366526	330,332126	379,159851
380,635925	335	264,067133	1520,43859	243,038205	366,023057	349,398963	390,581602
382,678955	335	281,236248	1650,52249	264,914299	386,396972	370,601975	428,994194
384,720398	335	299,973759	1732,37228	285,570268	413,641729	386,986099	458,062429
386,760376	335	312,78077	1848,47959	291,326327	426,656783	406,385242	481,880414

 This journal is © The Royal Society of Chemistry 20xx

388,798767	335	331,848604	1937,48248	313,910683	453,352236	441,02087	519,274381
390,835663	335	345,678057	2012,5496	318,512513	453,628384	441,096949	542,909006
392,871033	335	369,551946	2081,24413	344,990737	475,362545	463,586956	568,192076
394,904877	335	383,793353	2162,98837	363,628023	492,81284	481,111428	598,262754
396,937195	335	413,6802	2230,70264	379,634392	509,876251	503,040824	623,211824
398,967957	335	437,168256	2317,71063	388,45387	526,055178	536,163	652,794691
400,997253	335	450,696231	2370,63144	401,311552	534,298681	541,816407	674,492487
403,024963	335	470,042349	2442,78323	422,264376	554,852805	555,869056	686,484648
405,051147	335	499,674916	2508,61665	430,870142	565,589565	578,813162	717,971183
407,075806	335	497,808759	2555,09704	454,768463	567,726015	586,495333	744,910164
409,098938	335	525,483286	2606,45016	473,824623	589,41282	597,81249	744,237552
411,120544	335	534,315824	2648,32147	489,6552	591,278693	613,679928	763,896642
413,140564	335	559,29142	2654,30859	501,764453	608,998787	625,821599	776,631474
415,159119	335	568,098317	2697,7241	508,610224	615,623889	639,794165	777,818583
417,176086	335	585,413449	2696,48441	521,880355	611,500091	646,717997	775,763414
419,191528	335	587,451914	2704,01866	524,512444	616,421421	648,286874	777,100834
421,205444	335	609,577135	2702,90857	532,648991	615,193161	649,894758	778,527176
423,217834	335	615,178398	2699,66625	553,151916	593,716777	654,485409	779,415482
425,228668	335	627,26611	2690,83417	549,517905	601,980303	653,343452	771,303174
427,237976	335	632,096929	2698,06337	560,408804	604,156535	663,96977	776,134078
429,245728	335	639,358193	2637,50824	548,585322	605,945762	671,709539	758,091079
431,251953	335	645,859596	2655,12544	570,721151	600,553988	664,229522	760,6785
433,256592	335	648,076674	2617,4162	572,688435	609,900652	666,063086	760,735732
435,259766	335	669,381578	2626,40818	580,261578	613,113285	672,198808	750,451789
437,261353	335	650,12105	2618,01375	574,545227	596,209455	665,622201	752,695994
439,261383	335	664,191664	2563,97667	575,430155	597,222031	680,797793	744,007732
441,259888	335	661,240273	2521,14141	585,716568	596,67348	667,277525	727,399985
443,256836	335	653,228808	2464,74972	572,382042	578,757942	658,241375	698,516223
445,252258	335	660,069704	2453,63359	575,818656	572,831542	647,457271	701,373251
447,246094	335	652,544961	2377,82845	568,733821	557,691294	650,951476	674,455833
449,238434	335	642,532788	2316,07872	552,589984	542,91595	640,960324	661,931441
451,229187	335	635,983528	2264,74154	550,766233	542,411693	630,551634	647,609817
453,218445	335	635,892579	2212,1993	542,34843	528,615565	620,991965	628,554117
455,206116	335	631,493638	2167,7358	538,842484	525,520199	611,536915	605,581591
457,192261	335	625,207759	2132,93017	531,068123	503,383563	593,961783	589,443733
459,176819	335	617,273071	2068,75032	528,405987	491,00987	592,874023	575,567558
461,15979	335	609,469595	2009,53015	524,357312	488,052531	586,686852	561,65689
463,141296	335	605,113199	1946,71964	502,916412	470,198645	586,564493	559,25292
465,121216	335	591,879832	1897,3379	498,731431	452,261784	582,024042	524,306416
467,099579	335	588,597138	1851,41357	502,774999	439,882961	551,925327	506,134991
469,076385	335	587,619408	1773,64621	493,334239	439,273452	557,409384	501,231318
471,051636	335	582,180588	1741,83967	483,505431	417,983363	551,531271	487,374585
473,02536	335	577,728628	1686,31451	476,023402	410,855974	548,964619	465,028641
474,997498	335	568,037514	1627,70315	470,570512	405,139361	537,476608	449,978555
476,968079	335	560,823454	1606,47177	460,312126	400,007246	534,778764	442,767819
478,937134	335	556,905617	1539,07602	457,451983	381,894176	520,242482	423,427342
480,904572	335	550,97936	1491,90045	452,097408	371,863901	514,558845	413,701969

Please do not adjust margins

482,870453	335	532,999621	1460,73905	450,002975	376,390287	517,439145	406,154225
484,834808	335	534,417336	1403,03999	444,431587	359,526929	511,947512	388,315854
486,797607	335	538,354475	1368,32535	434,778211	355,078396	505,542611	381,219094
488,75885	335	534,330565	1332,87667	425,557078	341,473927	500,307311	357,695356
490,718506	335	524,641879	1288,64225	415,901639	330,522354	488,875412	355,720306
492,676605	335	505,694932	1246,95719	412,015777	325,043112	488,424312	332,28265
494,633118	335	513,313606	1209,06165	414,148168	316,018791	474,320257	327,011187
496,588104	335	506,636343	1177,17087	398,485404	308,84584	472,320664	313,765335
498,541504	335	503,929673	1135,13296	394,010151	295,655133	464,384169	308,658107
500,493347	335	496,126667	1082,03195	397,453478	290,380563	457,058932	290,654683
502,443665	335	483,657765	1054,47748	390,934781	280,703568	451,364117	278,03006
504,392365	335	472,901031	1022,83341	373,852514	274,375946	434,658373	274,377935
506,339539	335	458,95537	996,370466	361,510804	261,98162	436,249363	256,640354
508,285095	335	462,355569	955,914051	359,715364	256,522516	431,195452	252,834172
510,229126	335	455,180468	899,704006	349,751932	255,7661	417,599957	243,419885
512,17157	335	447,297595	887,156814	348,994313	243,225592	409,211615	230,738005
514,112427	335	448,654479	855,027491	336,639378	233,063424	402,94784	226,614309
516,051697	335	428,750806	816,835478	329,088354	227,823283	394,561517	213,379439
517,989441	335	423,285662	801,778406	323,123963	220,175652	387,501571	202,884054
519,925598	335	417,838922	767,446069	311,810675	215,638804	372,600863	196,055397
521,860168	335	413,345762	730,056305	310,506495	203,242793	371,837356	189,972714
523,793152	335	400,27884	706,13777	307,884873	198,463622	363,134704	177,394476
525,724609	335	389,567848	692,948205	297,928122	189,082727	359,2575	177,002086
527,654419	335	389,437149	666,878857	292,991174	191,026457	348,969535	170,757282
529,582764	335	385,969461	649,157266	285,805191	182,597798	342,11084	160,510678
531,50946	335	376,906465	620,405127	284,437213	179,059355	344,921226	158,568521
533,43457	335	368,83926	599,707712	278,501599	176,072015	334,478668	146,763627
535,358093	335	366,727613	575,833399	274,93942	167,134212	328,621818	143,517841
537,280029	335	354,435317	557,667494	266,217391	164,324707	316,890566	141,578585
539,200439	335	358,216019	536,179716	260,566888	158,629083	312,408755	137,383797
541,119263	335	344,424141	515,63497	263,462282	155,221818	308,396661	126,322005
543,036438	335	331,577444	502,45484	251,766764	146,735481	298,147503	125,596839
544,952087	335	332,687844	487,039261	241,59373	145,225928	290,912689	122,373772
546,866211	335	325,532744	463,850324	241,611201	139,80002	282,442086	113,843396
548,778687	335	314,279671	454,768062	233,047298	140,451389	277,287979	112,315639
550,689514	335	306,465735	432,861645	227,084408	133,86967	270,967505	105,511627
552,598816	335	296,962453	421,892774	222,718326	126,048155	270,078733	106,560558
554,506531	335	296,646569	403,818081	220,451	125,332296	262,404258	100,948802
556,41272	335	294,211114	390,153942	210,808576	122,450025	253,625638	97,8754186
558,317261	335	284,276909	377,756924	207,129678	118,195275	245,786997	93,7253779
560,220215	335	274,754832	360,951602	197,049145	113,954342	251,30854	87,131736
562,121582	335	269,238968	357,792395	197,165167	108,11606	237,418363	88,8221392
564,021362	335	265,840642	340,143168	192,839279	103,681933	230,006479	79,9269367
565,919556	335	256,226025	328,588067	188,71051	97,3078059	224,813987	78,5955271
567,816162	335	249,040658	324,201458	187,265105	101,998524	220,034044	77,7793961
569,711182	335	246,030006	308,275667	181,755564	96,099108	213,960069	68,8469086
571,604614	335	243,952101	297,885801	175,062149	93,3489916	206,05964	70,2145972

 This journal is © The Royal Society of Chemistry 20xx

Please do not adjust margins

573,49646	335	230,538771	284,820518	170,265012	90,8185553	207,824759	67,3340433
575,386719	335	225,482802	278,173162	163,593011	87,4938508	202,906309	66,5625125
577,27533	335	222,808845	262,703949	163,219264	82,9970884	192,613998	66,3419063
579,162354	335	220,533694	262,700453	157,354869	84,515095	188,88622	58,6361286
581,047852	335	210,134949	255,715661	157,705746	81,8167774	180,339456	57,4070538
582,931641	335	209,840651	242,148925	147,702417	80,5401398	179,260644	58,4848167
584,813965	335	202,202204	222,763153	147,099436	74,6422145	176,672731	54,6085162
586,69458	335	200,929234	217,497775	142,904209	73,2852268	168,617433	51,6195356
588,57373	335	187,96271	216,339281	135,317542	68,4032233	158,284399	47,1340299
590,451111	335	183,24195	208,262749	133,836851	68,3187432	157,109709	47,7588285
592,326965	335	176,638519	201,829979	126,60861	63,8021564	154,28103	44,1414423
594,201233	335	174,578497	186,591622	121,474614	62,7159542	148,973972	43,7056145
596,073914	335	171,112243	185,683015	122,358096	60,9085908	141,218455	42,9972958
597,944946	335	162,214264	179,341634	119,16822	58,2837098	147,354672	41,0104368
599,814453	335	158,032411	173,7836	113,458252	55,3765613	139,526664	40,5039786
601,682251	335	154,384193	164,874599	111,25006	55,6553822	131,226387	34,4901629
603,548523	335	150,988412	158,594196	109,992105	50,8399726	128,701379	37,4708943
605,413147	335	145,225197	156,590983	108,482597	52,3843892	125,327015	34,2561807
607,276245	335	148,873836	148,306016	100,087103	49,251918	114,869335	34,102588
609,137695	335	140,811163	142,174226	103,683367	49,7840442	118,951407	31,6168765
610,997437	335	138,747002	143,067173	100,635818	45,4033754	114,203895	32,4917823
612,855713	335	133,498371	136,085647	94,9778711	46,1327828	108,650213	30,5737156
614,712341	335	129,464707	131,137778	91,4614545	44,5213214	109,555382	28,1100044
616,567322	335	123,159252	119,409203	87,9870396	46,3068819	104,260777	29,4656112
618,420715	335	119,550095	121,326916	88,6276125	41,2563588	102,471112	27,87525
620,272522	335	120,728231	116,142323	87,7916519	39,7026285	100,434345	27,1159263
622,122681	335	112,243351	115,054621	81,8210561	39,1712681	93,9748473	23,3801761
623,971313	335	109,249365	110,622669	77,4126054	37,113528	90,7305466	23,44417
625,818237	335	106,987904	105,879441	76,0646825	34,672563	89,1243311	24,0528938
627,663574	335	102,195865	103,263433	73,9207199	35,8740138	86,8987866	23,0610464
629,507324	335	102,07364	102,937698	74,4928892	34,9045867	82,643	21,4079552
631,349365	335	98,4969705	96,0207499	71,0078131	32,3235724	78,3291437	21,8397282
633,18988	335	94,020712	94,9896046	67,2079418	29,5772001	82,1662064	20,5355872
635,028809	335	92,9192123	85,9413088	67,2720028	28,201636	77,004265	21,356726
636,865967	335	91,9158388	83,6698529	65,3944748	30,6528062	72,8422373	19,9299524
638,70166	335	86,2053869	80,5725849	64,071653	28,2621446	72,045764	18,8968841
640,535645	335	83,2572237	78,3578987	59,3572833	27,4197107	67,9552184	16,7013599
642,368042	335	86,0837326	75,3058585	61,1028777	28,3957932	66,1530193	15,5718875
644,198853	335	80,8053695	69,266889	59,4521855	24,6767819	65,1418579	16,7575652
646,028015	335	76,1649451	69,557572	55,6369509	24,0745861	60,8064698	15,6235561
647,855591	335	73,9232144	63,4262766	51,2054544	23,2233342	62,8791989	17,0840242
649,681519	335	73,7905501	65,66944	52,11265	24,0872352	60,4720382	13,1714138
651,505798	335	69,3168131	64,4526064	52,1148381	21,7463337	56,5876062	13,860336
653,328491	335	69,8499149	62,414579	46,8566802	21,2813325	55,4864838	13,7969296
655,149536	335	67,7525341	56,8688316	46,1737414	23,387682	52,5942013	13,8470319
656,968994	335	60,0918523	57,6405661	46,257504	21,8758286	48,4195399	13,9042629
658,786743	335	63,0756473	55,9757079	41,8529751	19,0715459	50,8045382	12,1439885

This journal is © The Royal Society of Chemistry 20xx

Please do not adjust margins

660,602905	335	60,8475012	50,2705565	43,1385188	19,4407779	50,3855372	12,9268484
662,41748	335	60,9037146	50,9307592	43,2239747	20,0705572	46,6019442	12,768503
664,230469	335	56,3464802	49,2903182	41,0298299	15,5446107	46,1509475	10,7336958
666,041748	335	51,2184806	49,2712585	40,4522476	16,7708986	46,9966865	10,5360451
667,85144	335	53,3262289	47,6577931	39,0368676	16,4893972	41,6714942	12,0898548
669,659424	335	47,8504077	41,4021021	35,9476899	16,6323867	41,4283918	8,51286729
671,46582	335	49,1050371	43,1986961	38,8282008	17,5256766	38,2975272	11,580597
673,27063	335	47,4267854	39,0751954	33,7097614	17,0445248	38,6672905	7,78720885
675,073792	335	49,2821285	39,4812912	31,3543411	13,7720543	39,4021228	7,03297321
676,875305	335	47,9500263	38,0404	31,5683857	14,8709989	37,8540799	10,9869374
678,675171	335	47,2961618	38,0464454	31,7934263	15,0937083	35,4388106	7,36241788
680,47345	335	40,5400988	36,4746695	31,9890143	13,1005861	31,3427071	8,23522185
682,27002	335	38,1380462	34,6106751	30,3349249	12,8145723	32,9962962	8,37394287
684,065063	335	40,03941	33,2834325	27,5581905	14,4088925	32,3309137	6,85408068
685,858398	335	40,833689	32,5210685	28,4560625	13,639005	29,6865857	7,52067405
687,650024	335	35,7649709	31,997856	27,3759905	11,7656155	29,7160759	6,64248084
689,440186	335	34,4003751	29,3991662	28,6612607	11,1252936	29,1179402	5,32626772
691,228577	335	34,5286807	30,9734422	24,3407126	10,5742281	27,6867703	7,10518384
693,015381	335	30,9310209	25,3551819	26,6307422	9,15029548	29,9861994	5,78755171
694,800476	335	32,7094782	27,5822275	21,94538	8,25552117	25,5209089	5,56356357
696,583984	335	32,0568881	24,4420827	21,810259	10,6410801	24,5049659	5,02741686
698,365845	335	31,3601507	25,7475439	22,9931612	9,99702378	25,4714961	6,72205782
700,146118	335	31,5982537	25,2859605	21,621069	9,92417703	23,0947662	5,28664275
701,924683	335	27,5281952	22,3235921	21,1389765	9,5094967	24,87991	6,54902298
347,741211	340	0	0	0	0	0	0
349,808533	340	0	0	0	0	0	0
351,874329	340	35,8184623	238,984156	37,9290016	178,300077	57,3090098	41,8147506
353,938568	340	42,9534633	271,806665	43,9812996	167,038638	65,0128892	56,8071417
356,001343	340	45,9742313	329,249002	55,675937	157,875398	76,152171	61,2327239
358,062561	340	60,8094018	400,94321	63,8340732	156,378934	95,4274434	72,7282474
360,122253	340	68,3635284	460,412256	63,9037619	150,472033	103,070857	92,4811361
362,180481	340	79,731805	521,282338	79,6866095	160,710967	128,305187	104,392199
364,237152	340	104,305461	606,361334	94,3458458	166,12498	136,640168	120,085339
366,292328	340	111,69543	674,701884	112,013764	189,052145	154,157219	145,224043
368,345978	340	132,338957	755,788794	119,355579	193,569559	174,323996	165,853228
370,398132	340	145,380731	858,100478	134,121529	213,213677	197,114649	183,245367
372,44873	340	162,718445	955,262931	155,036986	234,656097	223,659777	219,593628
374,497803	340	193,929864	1066,35246	171,948512	265,225597	243,741017	240,006563
376,545349	340	214,314869	1151,61438	187,440788	285,048646	265,92314	287,807167
378,5914	340	228,577898	1249,44972	218,056696	304,11891	296,609197	315,882188
380,635925	340	253,96614	1347,45275	227,654946	340,239396	317,545743	347,162797
382,678955	340	281,718466	1468,02447	255,198017	360,860338	340,362428	389,205619
384,720398	340	295,162785	1555,58525	258,081432	383,130044	367,012559	419,313752
386,760376	340	318,972511	1670,49882	283,733964	403,437078	384,005566	440,634653
388,798767	340	334,611213	1750,91273	302,935783	422,031886	400,833888	459,339836
390,835663	340	359,757992	1814,64644	309,939788	441,469526	422,688803	474,270625
392,871033	340	372,418013	1903,95897	326,970361	460,778805	437,107086	509,321999

 This journal is © The Royal Society of Chemistry 20xx

Please do not adjust margins

Please do not adjust margins

394,904877	340	387,940653	2012,95097	340,662296	472,786398	459,684362	550,282207
396,937195	340	419,429421	2125,14516	364,199062	486,812497	489,183594	576,266286
398,967957	340	430,801838	2174,87728	369,90766	492,781894	506,132917	595,027681
400,997253	340	449,470882	2252,00419	399,271671	526,392901	529,425987	617,3606
403,024963	340	473,285163	2348,30727	410,144006	526,495903	532,990036	634,658746
405,051147	340	503,987027	2428,18603	439,591372	544,708008	552,689244	662,439333
407,075806	340	517,789481	2486,32286	456,403655	553,523775	568,30052	681,058652
409,098938	340	540,841143	2540,84099	466,076647	563,240245	593,647256	705,351852
411,120544	340	561,188182	2564,90428	480,190557	576,65191	607,39574	707,946289
413,140564	340	578,660862	2636,13591	498,219203	598,487449	619,935079	729,871805
415,159119	340	603,157419	2669,94166	518,683443	584,955358	632,445504	732,712352
417,176086	340	616,885587	2682,78674	529,83149	597,672626	649,124005	744,60118
419,191528	340	637,453742	2705,1317	539,127313	599,879001	652,29806	747,091688
421,205444	340	639,669472	2708,34981	550,616176	606,363113	663,348846	748,992872
423,217834	340	648,658406	2710,27023	567,003491	601,201413	673,755802	758,486517
425,228668	340	668,579303	2752,0104	579,637659	592,900189	669,195304	764,606699
427,237976	340	680,168072	2720,70441	572,901572	601,385368	673,876488	749,919738
429,245728	340	678,825071	2716,41931	585,204691	596,293501	683,156602	746,368297
431,251953	340	691,779853	2710,87793	591,462946	613,612023	686,191421	741,479384
433,256592	340	704,493029	2699,26489	591,898986	603,351852	688,862409	746,376376
435,259766	340	717,446958	2712,95123	604,819342	607,63811	687,33016	737,880227
437,261353	340	715,755782	2688,9019	609,707144	598,232584	694,758447	740,423698
439,261383	340	715,582592	2633,81377	607,533726	618,82917	691,98183	727,602553
441,259888	340	713,166104	2618,65468	612,549906	594,286182	689,607136	728,369766
443,256836	340	728,475044	2580,51058	599,860881	585,628068	676,795191	704,050094
445,252258	340	710,139675	2535,36932	610,443292	583,226338	681,752549	698,808488
447,246094	340	708,144879	2484,96625	595,940184	568,140719	667,370137	683,523443
449,238434	340	690,446944	2418,70703	592,353617	558,86673	650,35335	662,566248
451,229187	340	704,461474	2362,73317	586,794069	552,610943	658,574809	651,284104
453,218445	340	699,050527	2318,25223	583,380844	526,101335	655,658279	632,389384
455,206116	340	674,136207	2272,93234	576,570428	528,107702	636,248727	626,494779
457,192261	340	674,102742	2220,12182	570,632176	528,974894	626,266446	597,135435
459,176819	340	662,447015	2152,67173	566,692112	503,444505	624,652357	597,090519
461,15979	340	664,283364	2091,34925	551,73636	495,563678	621,324321	576,394647
463,141296	340	654,725407	2048,91352	542,511138	486,836241	606,119103	566,082382
465,121216	340	645,43249	1979,22178	530,470893	477,965094	600,475125	540,417353
467,099579	340	635,945415	1937,06032	525,866336	457,010786	592,957309	530,325319
469,076385	340	631,465072	1871,17983	519,817668	440,936674	573,470367	512,327902
471,051636	340	624,992392	1822,93834	516,093744	438,114771	564,753446	494,064781
473,02536	340	613,097063	1764,32728	510,908685	428,332317	569,286698	484,626321
474,997498	340	606,811041	1729,38631	503,366616	415,236751	557,187562	469,562988
476,968079	340	604,36011	1679,05441	495,178925	410,781284	560,671663	456,97969
478,937134	340	590,93139	1611,02208	481,230566	397,220242	545,376353	434,834279
480,904572	340	600,674682	1569,71571	478,609831	395,205219	537,351942	418,242459
482,870453	340	579,214691	1521,43038	476,89407	377,996076	523,194451	411,636139
484,834808	340	567,45504	1477,08311	464,102603	374,886698	528,875448	401,586437
486,797607	340	567,977	1425,12467	471,898574	361,550984	532,109723	386,964186

This journal is © The Royal Society of Chemistry 20xx

Please do not adjust margins

Please do not adjust margins

488,75885	340	560,888454	1385,59136	454,24308	356,094133	520,192005	375,69251
490,718506	340	558,126876	1368,43745	434,953303	349,066972	513,494557	362,462174
492,676605	340	548,434189	1321,30416	440,592352	333,270107	499,448359	354,042469
494,633118	340	532,345997	1260,74162	431,950697	328,119002	495,71998	338,93729
496,588104	340	533,277927	1217,01138	427,830787	316,149019	481,622893	318,424388
498,541504	340	523,636071	1181,8743	417,08765	301,627389	478,94524	316,22016
500,493347	340	517,349031	1129,94965	413,206521	289,161102	478,931765	299,726681
502,443665	340	513,343628	1097,76923	408,044764	290,173871	459,472587	294,657633
504,392365	340	496,925515	1074,87296	387,311016	275,624994	455,534571	282,020259
506,339539	340	494,041142	1029,80929	386,652015	272,990513	448,068107	274,061733
508,285095	340	478,587345	999,539596	378,167112	269,164031	438,447962	263,631725
510,229126	340	473,697706	954,090239	370,10216	257,035172	436,231501	242,439765
512,17157	340	477,303743	925,487294	358,691148	247,222029	423,329192	234,228673
514,112427	340	458,392606	889,852529	355,920644	240,119597	422,577152	236,009688
516,051697	340	452,38904	858,748416	348,18205	233,417176	404,979934	220,926123
517,989441	340	436,323068	825,795075	344,828288	224,740837	391,136492	212,053305
519,925598	340	437,066106	786,356298	336,751343	216,984135	388,544782	205,534886
521,860168	340	416,182561	767,78755	315,861531	204,59679	383,448112	193,156805
523,793152	340	403,294637	721,594106	320,314297	207,749166	373,986862	188,108706
525,724609	340	409,328581	699,384737	315,723883	193,829117	364,05788	179,647757
527,654419	340	407,599817	687,026856	306,269952	195,450861	355,916855	177,162172
529,582764	340	396,180673	668,932597	297,943481	181,280319	353,207478	170,161934
531,50946	340	383,940913	634,75352	292,755602	178,528456	345,911071	157,554567
533,43457	340	379,37475	608,690173	282,104534	173,416211	340,797345	158,091448
535,358093	340	372,977652	605,959493	283,276262	172,039275	330,893025	151,014013
537,280029	340	362,051664	577,548525	276,717008	164,925439	329,328418	144,842152
539,200439	340	362,119554	556,154778	269,716298	163,436964	314,827311	136,927233
541,119263	340	355,316143	537,748507	262,355359	157,145679	315,989627	131,683141
543,036438	340	340,047978	520,871764	258,364053	151,455779	304,886947	130,894728
544,952087	340	335,497023	498,824636	252,056344	146,821468	297,636633	123,518862
546,866211	340	328,063566	484,115065	244,64643	145,222637	285,61241	116,181282
548,778687	340	322,706237	462,402126	238,933345	136,127024	285,584163	116,352592
550,689514	340	321,180892	443,055457	232,595901	131,238184	277,61601	108,865852
552,598816	340	312,580436	437,980429	228,888606	127,062747	264,566114	106,673349
554,506531	340	304,039734	421,345642	224,224359	126,552052	265,001248	97,8938575
556,41272	340	299,135751	403,691803	216,64084	117,195274	258,623428	100,11827
558,317261	340	291,465002	390,813953	213,814774	115,645896	256,530007	93,5678916
560,220215	340	281,634291	373,867546	209,826556	114,019155	244,508461	89,7588662
562,121582	340	263,972718	358,04382	199,721677	109,490234	239,96935	88,0255459
564,021362	340	270,236255	345,794485	196,190691	105,032549	228,78546	81,642072
565,919556	340	263,230456	336,385013	192,152478	101,113395	228,127115	79,4623588
567,816162	340	253,567107	327,402134	191,245491	100,859814	225,110424	77,5818819
569,711182	340	253,765043	312,080486	185,024821	96,6559134	218,818814	71,1927402
571,604614	340	249,875382	307,33559	183,529159	93,8325778	212,91381	72,9970078
573,49646	340	238,369058	294,410751	181,733926	87,5730906	210,64516	72,7464495
575,386719	340	232,034231	283,741676	170,274279	85,5807388	202,08116	66,3340703
577,27533	340	229,719267	276,903948	166,588611	88,0245371	191,884931	62,4746136

 This journal is © The Royal Society of Chemistry 20xx

Please do not adjust margins

Please do not adjust margins

579,162354	340	220,383721	263,834886	164,249592	81,1096537	194,437613	62,9423149
581,047852	340	216,163076	252,202049	162,741693	78,4082425	185,088985	57,1542849
582,931641	340	212,46287	248,122693	154,491215	78,2188303	181,201841	55,7373852
584,813965	340	205,399024	236,394827	150,990626	73,1922127	177,200364	55,5020441
586,69458	340	199,715689	225,270739	144,575902	72,6484486	169,149041	52,9131697
588,57373	340	198,448701	215,8883	145,133409	67,8499364	165,425147	50,036242
590,451111	340	190,366392	214,931623	139,187574	66,8178324	156,278863	49,2348304
592,326965	340	180,029243	200,658118	130,05607	65,1070292	156,527098	50,2620921
594,201233	340	182,266554	191,231619	130,581552	59,750443	149,391432	45,8799141
596,073914	340	169,576745	185,764367	125,570637	61,6747343	143,6245	42,0573425
597,944946	340	162,571058	176,361326	123,472058	57,9476485	142,899271	41,8215908
599,814453	340	158,618552	176,636249	123,080012	52,2666889	134,838131	38,1715013
601,682251	340	160,419521	168,31359	115,578834	54,1187392	127,903206	38,1800109
603,548523	340	152,185164	157,893249	111,880913	51,5390603	132,753798	37,2144177
605,413147	340	150,35166	160,086818	111,002316	55,1900094	126,330199	33,1322949
607,276245	340	149,631442	149,226455	109,163141	52,6503688	120,636727	34,0606618
609,137695	340	139,972927	141,532318	103,007197	49,9195565	117,112112	34,0070837
610,997437	340	140,938582	140,435775	103,407776	45,9894247	115,869859	34,3681734
612,855713	340	129,183367	136,945243	96,5621919	44,7282276	109,91492	29,8749607
614,712341	340	127,854784	133,763465	95,8367326	46,6231422	107,155507	30,4466351
616,567322	340	127,300667	123,143092	92,6823646	42,2328069	104,112575	27,4313759
618,420715	340	118,982174	126,093384	88,3705473	37,9468661	103,771607	25,4039709
620,272522	340	117,847554	117,540964	83,3083376	40,8003937	97,2575166	25,6553353
622,122681	340	117,5054	110,04712	82,2964996	37,7124801	97,8640001	25,512817
623,971313	340	106,472602	104,671566	85,1098905	35,8254028	95,1461074	24,1944425
625,818237	340	106,493027	104,584925	81,0487217	36,4663961	90,1262018	23,6926778
627,663574	340	104,476498	100,161343	76,5860171	32,7273218	87,381905	22,7370843
629,507324	340	102,373858	98,4402738	74,448095	35,0006233	84,0905829	22,6486429
631,349365	340	98,9235222	94,382943	72,5900509	31,90625	84,7565252	22,8659136
633,18988	340	95,1156437	91,2715278	70,4385203	31,4763279	80,5150206	20,8083202
635,028809	340	92,939556	89,1988118	70,6989126	28,7352011	78,4501049	19,8916945
636,865967	340	92,3802726	87,4208434	68,791362	29,5889178	77,7672492	19,9234187
638,70166	340	84,3408212	81,8061845	64,8874273	29,2010209	71,0395281	19,6323376
640,535645	340	83,2131258	78,8592141	60,2271085	25,1547361	69,2078792	17,6155285
642,368042	340	83,7183474	75,5126664	61,7161357	26,2976477	67,4705017	14,8148787
644,198853	340	82,3772168	71,7193493	60,5311841	24,3271227	65,9940897	15,8568298
646,028015	340	77,3942584	71,7008516	54,9167452	22,1227282	65,3600849	16,1785421
647,855591	340	77,1571745	66,8981301	54,6896622	23,0259203	61,983615	13,9478554
649,681519	340	72,8753648	67,0523095	54,1665037	23,1492436	59,9698309	16,1564255
651,505798	340	70,2025976	69,4797338	53,0574194	24,0635053	58,0655626	13,7740612
653,328491	340	67,7461566	63,8346804	53,1182236	21,5698418	55,2655493	14,6352758
655,149536	340	68,4048695	58,5986576	49,9082802	22,2666898	55,7755323	13,3531114
656,968994	340	62,885926	58,633665	47,5259938	21,8411571	54,4532094	10,5203592
658,786743	340	62,3547981	53,2940662	50,4589891	19,3456986	50,969703	11,8144507
660,602905	340	59,9983632	51,4380289	45,0514794	17,5149643	50,6734876	11,6347005
662,41748	340	59,6390053	53,4507917	42,1112304	18,7456098	52,0532059	10,9565305
664,230469	340	58,4964394	50,7209301	40,3050268	17,9431532	47,2236682	12,0236882

Please do not adjust margins

Please do not adjust margins

666,041748	340	56,2885187	48,814614	41,640408	16,1888193	46,8893125	10,1602267
667,85144	340	52,3286983	46,7334478	37,0100034	15,3900322	42,8013293	9,66299402
669,659424	340	48,9465855	43,2345118	35,9231225	14,8932315	39,0855387	9,68266408
671,46582	340	52,5946565	45,7124841	36,9117312	16,0715306	40,0772038	9,69256428
673,27063	340	49,0823783	42,0673011	38,3305082	14,2238394	38,5356968	10,7882108
675,073792	340	45,9835912	40,5517831	30,5319673	12,6559432	38,078088	9,2194289
676,875305	340	43,5271373	38,3093814	31,6961719	14,9928545	37,43281	10,383135
678,675171	340	42,7889731	38,1026059	29,3608514	13,29365	34,7797561	8,5833428
680,47345	340	39,4591839	36,2655618	30,8266222	13,3897711	29,3329836	7,30615544
682,27002	340	35,8604577	35,6441989	31,1467799	11,7299903	31,8875173	7,75648646
684,065063	340	38,442544	33,7282701	30,8222197	11,1270343	32,3145106	8,20869322
685,858398	340	38,5665245	33,9572965	27,0883328	11,8811752	29,7595094	7,67902581
687,650024	340	34,208174	31,9537119	29,473089	12,5358414	30,6463115	5,76499052
689,440186	340	38,2641352	30,6303955	27,9298758	10,2022022	28,8913046	5,99232775
691,228577	340	32,4759929	27,9572651	28,2660418	10,6369987	26,8638635	4,06822271
693,015381	340	31,3721786	27,920121	24,8895717	10,3407334	29,0862962	6,33241443
694,800476	340	33,6277488	26,7276467	25,5296757	12,70731	25,7403276	4,72129899
696,583984	340	27,5951471	26,1862759	22,8206884	9,62301044	25,1954095	4,09394267
698,365845	340	75,3131155	24,9577149	23,3186474	8,9994849	23,0974329	8,53500662
700,146118	340	30,6160088	24,7305601	20,0361295	9,47087108	23,9517861	3,9049042
701,924683	340	29,7493564	24,4975431	21,2062635	7,64953661	24,2624477	6,42430735
347,741211	345	0	0	0	0	0	0
349,808533	345	0	0	0	0	0	0
351,874329	345	0	0	0	0	0	0
353,938568	345	0	0	0	0	0	0
356,001343	345	36,9944468	243,132266	48,0571262	215,601363	60,0237035	55,9907008
358,062561	345	52,1890876	299,414955	47,1543922	179,821512	71,6905322	60,7275135
360,122253	345	58,2942807	346,747124	50,5806722	171,307246	80,843965	66,2910436
362,180481	345	69,7200513	416,943414	61,3441295	159,882094	96,9248424	79,6593019
364,237152	345	82,8587053	452,48729	79,7322819	164,161233	105,203595	87,462796
366,292328	345	98,9687315	534,638647	90,1040342	168,626755	127,263856	106,957594
368,345978	345	113,65345	600,19203	96,3654942	177,541498	135,542982	127,795338
370,398132	345	122,767023	691,612858	112,614302	184,966364	164,061937	148,521138
372,44873	345	147,368511	774,365521	128,334964	203,384959	178,028806	172,141892
374,497803	345	165,687794	859,272995	144,678362	221,996901	207,487022	194,142518
376,545349	345	177,851253	960,750634	163,837269	244,268059	222,632302	220,019627
378,5914	345	195,955056	1034,43865	171,854311	260,746095	244,672076	247,419294
380,635925	345	231,30052	1119,53835	188,750532	278,992583	263,814694	276,912752
382,678955	345	245,624047	1246,58625	218,517214	305,2223	295,141773	316,946248
384,720398	345	273,737125	1350,16478	246,83468	347,219209	325,02204	358,900864
386,760376	345	297,217117	1463,65415	264,190221	375,221284	352,667871	391,635128
388,798767	345	330,368425	1564,05643	288,978999	401,763695	365,499506	421,356165
390,835663	345	341,842191	1644,32402	292,167485	420,950242	393,69655	433,788152
392,871033	345	349,581783	1724,31802	312,662598	443,662487	408,045315	468,012107
394,904877	345	385,260845	1823,6104	339,616778	460,129752	430,962838	493,19281
396,937195	345	402,77209	1932,78368	342,142746	460,094525	445,308071	517,481625
398,967957	345	423,747753	2001,04567	361,853607	480,220466	469,444657	542,578389

 This journal is © The Royal Society of Chemistry 20xx

Please do not adjust margins

Please do not adjust margins

400,997253	345	461,902073	2090,84377	377,896297	492,950503	480,894823	567,468918
403,024963	345	463,372859	2183,16837	398,491454	506,432674	500,573449	577,748341
405,051147	345	496,321856	2267,55094	433,879078	523,843945	532,849726	600,305129
407,075806	345	536,896558	2360,65179	445,395279	543,127561	561,605055	620,46248
409,098938	345	542,708697	2430,4938	461,337743	557,752164	562,497155	653,41719
411,120544	345	577,003684	2499,71315	486,248831	561,037015	599,353946	657,284205
413,140564	345	595,494077	2563,67552	508,349464	573,412908	606,098366	687,144883
415,159119	345	618,989213	2602,94211	520,978491	581,060056	630,080042	700,856607
417,176086	345	636,919566	2663,6539	523,607508	582,228112	638,012315	707,687039
419,191528	345	659,187532	2679,52952	558,362845	594,361122	638,054245	715,903225
421,205444	345	650,869006	2712,22431	564,698667	600,231302	653,037802	722,200842
423,217834	345	694,827724	2738,908	569,192571	605,399397	669,1594	725,086127
425,228668	345	721,529672	2751,34765	592,668598	607,332493	674,206582	732,287978
427,237976	345	703,239799	2750,7562	602,231282	589,61571	686,353493	736,296454
429,245728	345	721,424741	2718,00218	613,562113	595,82287	693,678407	731,002135
431,251953	345	736,114675	2741,54243	616,699959	603,373784	685,594999	745,009597
433,256592	345	747,645318	2737,72965	631,474555	617,415706	706,240958	741,488765
435,259766	345	768,429003	2766,27049	635,271265	613,046072	716,878989	735,626111
437,261353	345	764,232406	2742,7138	639,970213	600,369793	707,450064	734,191757
439,261383	345	772,008428	2717,16653	633,438116	602,024889	706,768059	722,640905
441,259888	345	764,099049	2689,11168	649,858936	597,915099	719,953537	722,39488
443,256836	345	766,512619	2640,50777	649,168978	594,149637	718,181537	696,372622
445,252258	345	762,987704	2609,57933	644,820587	596,529485	708,939274	690,642631
447,246094	345	762,051728	2553,2089	637,997403	572,420568	694,884796	686,694072
449,238434	345	752,199563	2483,47393	633,373107	573,764376	676,138705	675,032302
451,229187	345	739,512999	2457,75761	619,823418	563,928595	685,489843	659,406241
453,218445	345	742,828123	2412,47735	609,199721	556,979709	676,127338	650,686573
455,206116	345	729,012703	2349,50871	607,537542	554,785071	671,193917	632,067569
457,192261	345	730,676987	2291,51382	604,414879	545,521394	665,194858	625,140383
459,176819	345	712,219019	2256,91578	601,343587	528,981371	650,625436	590,788463
461,15979	345	721,635451	2203,90647	591,192747	519,925282	646,102373	588,494058
463,141296	345	698,549072	2136,07843	583,768644	511,232257	642,186454	570,652671
465,121216	345	691,042899	2085,40474	577,052979	490,685979	635,867545	552,019823
467,099579	345	668,5825	2015,48709	566,91913	475,045419	621,680236	544,097841
469,076385	345	682,812851	1975,73633	558,943148	467,499466	609,618648	532,985155
471,051636	345	667,022347	1910,09273	548,355519	458,943209	604,708295	514,072152
473,02536	345	661,121856	1856,20595	541,773079	446,368335	597,072525	491,461972
474,997498	345	636,359963	1795,66856	537,92027	443,10248	587,446276	478,939244
476,968079	345	637,214156	1745,4125	528,347313	417,052027	582,108092	468,988235
478,937134	345	640,71601	1693,79456	513,194656	416,848742	565,522991	455,031359
480,904572	345	631,587604	1642,00232	503,308851	410,536199	571,934807	441,035502
482,870453	345	611,535062	1616,33075	500,838134	385,620556	563,94326	420,523333
484,834808	345	612,758721	1554,3588	493,722744	389,696138	557,404153	412,677176
486,797607	345	609,73367	1522,02505	485,536562	376,99536	546,362571	402,814726
488,75885	345	590,858883	1454,4436	482,351778	370,654744	543,377217	394,46734
490,718506	345	592,404722	1414,3181	469,268589	357,697078	537,395791	379,425722
492,676605	345	580,928072	1372,36388	472,968064	345,077681	530,353435	366,529657

Please do not adjust margins

Please do not adjust margins

494,633118	345	573,927445	1316,54618	455,765191	338,388485	520,01	353,655996
496,588104	345	565,227574	1285,48493	451,676624	330,746789	517,081728	338,211474
498,541504	345	557,072955	1241,68931	436,633108	319,015425	505,143254	324,224988
500,493347	345	553,254808	1198,25834	431,093975	310,572161	494,185802	313,920685
502,443665	345	536,03849	1145,93678	425,530103	294,990355	487,912764	308,187136
504,392365	345	522,254983	1122,69461	416,335124	292,533757	477,999165	292,431973
506,339539	345	517,329938	1081,64152	407,06778	288,057647	467,863656	282,71221
508,285095	345	509,946379	1025,17056	403,563799	272,214074	461,911006	275,852444
510,229126	345	502,247841	1004,36833	386,809823	266,235485	443,215995	260,53848
512,17157	345	496,732351	955,745276	381,549532	257,380458	438,300478	248,993256
514,112427	345	474,06462	934,261183	380,62603	244,464493	435,828855	239,138593
516,051697	345	460,928543	893,488166	361,671319	234,317643	427,091712	230,257019
517,989441	345	461,332928	862,67889	353,123958	233,022609	420,261642	218,020748
519,925598	345	448,674684	824,556919	347,413052	228,378054	404,146044	211,664577
521,860168	345	443,686128	794,851079	338,691611	212,191512	387,902184	201,423259
523,793152	345	426,376134	766,736995	335,443661	208,130047	380,864195	191,360952
525,724609	345	422,666972	737,96134	326,831469	205,838956	378,457995	186,142439
527,654419	345	412,134416	720,333901	318,464423	193,68086	374,366945	183,465786
529,582764	345	411,715572	694,401174	309,176554	186,388109	362,842328	173,46799
531,50946	345	411,750428	677,550574	306,885692	183,179692	359,215848	168,061622
533,43457	345	394,419658	646,18523	300,916345	180,945323	350,438303	164,981636
535,358093	345	385,55344	623,387825	298,073103	176,02313	345,893631	155,49775
537,280029	345	382,406669	603,070759	288,68846	171,219271	329,239546	149,796262
539,200439	345	366,107139	584,852465	288,791764	167,433236	333,063415	150,132156
541,119263	345	366,73996	564,311392	270,490804	164,60452	326,775493	136,296553
543,036438	345	355,459912	542,28278	276,719948	154,721349	317,836887	135,168345
544,952087	345	345,420346	509,412104	264,340857	149,359809	303,465244	125,418657
546,866211	345	344,534177	508,479096	263,830171	146,070961	300,175076	126,533185
548,778687	345	331,774032	476,227122	248,944398	133,974634	292,529758	116,163298
550,689514	345	324,621161	469,569882	245,685587	138,584975	285,888845	115,709735
552,598816	345	322,89045	451,16582	238,365519	127,086952	278,994351	110,34282
554,506531	345	312,404751	433,433187	232,383061	127,4782	275,843211	107,007356
556,41272	345	308,33798	419,803943	224,187995	126,104392	264,625084	98,5154603
558,317261	345	303,170956	410,463572	225,710176	114,789645	262,491718	98,5184264
560,220215	345	285,008413	388,788675	212,103871	114,005055	252,180679	93,8203136
562,121582	345	281,204502	369,581459	208,693038	109,592061	243,751038	86,1691611
564,021362	345	275,078108	356,507734	203,922457	105,156698	239,374074	84,6616423
565,919556	345	273,60139	347,253118	203,457495	104,220864	231,431546	78,7909639
567,816162	345	259,976631	332,613953	200,276734	101,016302	232,751051	82,6213381
569,711182	345	262,565492	329,206896	193,168492	99,0934627	227,645217	74,6888281
571,604614	345	253,196603	313,531428	188,405158	92,6368155	219,031378	73,3937912
573,49646	345	244,910642	298,88516	181,852508	90,6319343	218,945699	70,6106233
575,386719	345	241,41528	287,61135	173,883969	85,4145341	207,785186	66,8059275
577,27533	345	234,361944	279,540712	175,842963	88,7277758	195,953041	63,7797138
579,162354	345	227,677475	276,135015	169,454081	83,09526	194,690175	64,4285844
581,047852	345	217,680537	261,087773	163,857261	81,6784041	188,728205	60,0784039
582,931641	345	218,42216	252,028034	159,971745	76,9610572	180,672085	59,0634118

 This journal is © The Royal Society of Chemistry 20xx

Please do not adjust margins

Please do not adjust margins

584,813965	345	210,997839	237,382862	155,931115	73,1714202	179,18757	56,9023831
586,69458	345	204,177297	230,655475	150,815267	72,9252229	173,018882	55,4949858
588,57373	345	197,276907	223,066441	146,54672	67,9737027	167,924879	50,372225
590,451111	345	194,993241	218,382846	142,300556	68,8695944	162,260913	48,4733185
592,326965	345	183,18832	204,954633	135,753011	64,1887685	155,638728	48,8148774
594,201233	345	176,284904	200,946489	126,142698	61,9504838	148,115756	45,8751598
596,073914	345	178,537331	190,592766	131,6153	60,7538154	151,036085	43,9853722
597,944946	345	173,528786	186,163129	123,072052	59,053159	146,732144	43,3661132
599,814453	345	169,538568	179,736539	126,837219	53,5046048	138,84234	39,5053679
601,682251	345	159,973176	171,994357	118,539514	54,5348186	134,849923	37,3936536
603,548523	345	159,822685	164,802791	115,207976	50,7643768	133,213217	36,7379373
605,413147	345	153,015772	158,191773	113,284511	50,9662239	129,335899	37,4416994
607,276245	345	149,136233	152,874534	112,537081	53,1887692	123,574514	36,3330128
609,137695	345	144,206542	148,139197	107,000464	48,7992831	120,697952	34,1756789
610,997437	345	137,291513	143,066372	100,020116	48,8000465	119,017337	34,434661
612,855713	345	136,669222	141,488871	101,111796	45,2691793	114,163839	31,0981706
614,712341	345	132,993252	132,162647	99,6531599	42,9871103	109,369229	28,3996702
616,567322	345	127,255229	125,376524	96,8712891	44,5735782	103,896605	29,4866708
618,420715	345	124,739136	120,935568	93,6385847	39,0472132	105,304241	26,1634362
620,272522	345	116,199878	117,329993	90,6485572	40,2896525	101,078746	29,3036628
622,122681	345	119,843779	112,937452	85,807676	38,5367335	100,714839	25,4061252
623,971313	345	113,754863	114,11994	85,362746	35,2440279	96,5193191	23,7320827
625,818237	345	106,883244	108,725098	82,4823952	34,0185452	93,2898279	25,2845085
627,663574	345	108,342288	100,52303	78,4276459	36,2265481	88,8593263	23,1857345
629,507324	345	101,899211	102,293132	76,4583484	34,1480657	83,5992755	21,3419558
631,349365	345	102,26932	101,48519	75,1395961	32,2092546	84,9389059	23,415077
633,18988	345	94,1300667	89,6217612	69,4079957	30,0206185	80,8015585	20,81836
635,028809	345	95,25579	92,6159775	72,5149896	29,2156908	79,4729501	20,0207681
636,865967	345	91,8558183	87,839923	68,2944821	28,671128	77,0351467	19,9544006
638,70166	345	88,2537645	81,8779286	65,2753339	28,2925713	71,748202	18,2933955
640,535645	345	84,1908662	82,2569354	63,3902277	27,8931257	70,4522795	16,8150327
642,368042	345	82,4147858	76,8327971	62,1834802	27,3346586	66,8819062	17,5244641
644,198853	345	83,2858126	74,4286446	61,5263013	24,8223877	68,5112698	17,6609362
646,028015	345	79,7138466	69,5669792	59,0227404	26,2372548	67,5003027	17,6813902
647,855591	345	77,2012134	69,536383	56,819048	23,4546964	62,7149773	13,1317912
649,681519	345	74,5260449	67,1243899	53,0431219	22,0878911	63,4492947	14,6910843
651,505798	345	66,8287009	64,5043759	51,3623715	25,0758163	59,6092568	13,8543775
653,328491	345	70,5469947	60,1960186	49,6162325	20,7438011	56,9839328	12,1974398
655,149536	345	67,3788438	62,4250846	48,7904211	21,5231589	56,3853595	12,9441282
656,968994	345	64,4211016	58,0399009	48,3859888	21,10716	53,8885893	13,1991411
658,786743	345	63,7139516	56,695542	45,9791136	20,5995551	53,3343731	11,1364134
660,602905	345	60,9111344	57,2366229	45,0707331	20,0380996	49,6931145	12,6850206
662,41748	345	62,9241252	51,1622811	43,4336566	20,332461	51,6552348	12,028452
664,230469	345	57,7173759	48,8075114	45,4649319	19,3488335	50,3223096	11,643638
666,041748	345	56,1794276	47,1539326	42,2651672	17,7431567	46,9231409	10,5393267
667,85144	345	53,0382798	49,4421537	40,2211377	16,7634965	44,7870388	9,43964557
669,659424	345	50,1535438	46,2468119	37,7260932	15,9731973	41,5768222	9,25323357

This journal is © The Royal Society of Chemistry 20xx

Please do not adjust margins

Please do not adjust margins

671,46582	345	51,5477515	47,015832	35,9073881	16,6084789	39,8636789	8,9539382
673,27063	345	51,7497332	43,2667181	35,4265668	13,9809325	41,1603412	8,98902278
675,073792	345	48,3165635	37,5423763	33,2114972	16,1868553	39,2659198	6,62534835
676,875305	345	50,6968021	37,2241933	34,8654611	13,6939733	38,012175	10,5571471
678,675171	345	47,6916603	40,8597854	33,9369572	12,2435276	35,8368429	8,38494918
680,47345	345	43,1908216	37,4089432	32,3555004	12,7525797	32,7994158	7,55103798
682,27002	345	40,4233426	32,6512827	32,7798108	13,5250429	32,6006099	7,16201534
684,065063	345	39,4078706	35,6810665	31,0094068	13,465613	35,2170042	7,08167823
685,858398	345	38,0656572	33,5048042	29,8739998	12,1139211	31,4687386	8,02247428
687,650024	345	34,5443556	31,4484868	28,3353015	11,4987503	30,2441852	8,44760957
689,440186	345	38,7161547	29,4267482	27,6671974	10,1632448	27,0624086	5,64475724
691,228577	345	36,2158378	31,4319119	27,4711047	9,11997179	26,3411089	5,54295011
693,015381	345	33,3606803	28,5702896	26,1740167	10,5092189	29,879606	4,71817244
694,800476	345	33,9836223	27,5130375	24,275854	10,7167258	25,7699823	6,8134357
696,583984	345	31,9024048	27,2023414	23,3186897	9,59958972	24,6066952	5,57799355
698,365845	345	31,6742454	25,0584115	25,712053	8,88605867	24,3581228	6,22691245
700,146118	345	30,9498957	24,4154166	22,3090403	9,24017624	24,4554088	5,40408332
701,924683	345	29,4673918	23,230535	21,7549543	8,52937884	24,2292161	6,16944745
347,741211	350	0	0	0	0	0	0
349,808533	350	0	0	0	0	0	0
351,874329	350	0	0	0	0	0	0
353,938568	350	0	0	0	0	0	0
356,001343	350	0	0	0	0	0	0
358,062561	350	0	0	0	0	0	0
360,122253	350	45,5374765	239,86808	48,5666428	225,761951	61,1194275	56,7341183
362,180481	350	55,0167081	287,233179	52,8879326	200,571635	69,9737064	56,7675422
364,237152	350	61,4378226	318,542602	64,3478802	170,906149	79,2806569	69,6189793
366,292328	350	73,3467123	390,62809	74,9192408	168,36335	96,182654	79,9007955
368,345978	350	90,3305663	441,064303	72,5548312	174,188	101,988159	94,5695252
370,398132	350	97,7558874	508,85464	86,9885436	171,653813	119,165127	112,195577
372,44873	350	119,729887	589,72488	105,810011	177,756091	136,875608	135,981029
374,497803	350	133,973676	669,033828	116,014136	194,151585	154,932731	151,113389
376,545349	350	149,679689	745,066575	130,415441	205,299907	175,642154	182,730292
378,5914	350	171,313325	821,7131	146,177714	222,460003	190,762034	204,14386
380,635925	350	188,584707	915,427159	158,882866	244,456861	212,26117	226,02659
382,678955	350	210,605017	1018,97631	175,186892	262,315134	235,416989	247,261849
384,720398	350	235,703796	1113,90242	197,347452	292,601355	252,443112	279,248556
386,760376	350	258,328179	1211,64784	219,241308	314,307927	275,097047	308,370465
388,798767	350	283,072614	1299,57465	238,278763	339,76566	313,857002	339,960739
390,835663	350	306,204846	1400,1759	254,81504	375,245729	339,314287	376,862847
392,871033	350	334,889535	1510,70812	286,721573	401,018146	358,667251	407,148533
394,904877	350	364,846325	1630,18836	308,107582	415,217205	386,762008	448,560605
396,937195	350	395,533824	1707,55604	330,160501	452,09827	406,200708	469,848856
398,967957	350	408,390769	1812,67124	348,026253	463,758149	430,951315	495,932435
400,997253	350	431,819601	1912,21108	360,125783	467,481872	453,527579	513,581626
403,024963	350	459,500074	2005,4574	381,491421	481,591062	466,871359	534,633147
405,051147	350	479,082421	2126,58381	403,713824	495,052151	500,641827	546,746416

This journal is © The Royal Society of Chemistry 20xx

Please do not adjust margins

Please do not adjust margins

407,075806	350	512,559043	2223,21216	420,339651	511,538132	516,26752	575,868808
409,098938	350	534,787242	2286,93159	445,350988	519,862706	541,45873	603,452374
411,120544	350	563,435986	2359,21297	469,585189	545,309935	560,402175	605,001714
413,140564	350	590,187096	2470,70235	488,108906	554,572339	580,073238	636,38392
415,159119	350	617,098001	2518,16735	501,232151	560,554013	604,500562	641,830479
417,176086	350	629,952334	2576,14412	532,65658	573,218	615,866875	666,163835
419,191528	350	654,823266	2618,99975	539,648754	571,523158	635,476043	671,244708
421,205444	350	689,171974	2650,88317	564,773248	586,835593	640,372638	687,841268
423,217834	350	708,612965	2676,98992	586,567844	587,602104	653,158025	686,891495
425,228668	350	726,782179	2725,50865	596,237917	589,671349	669,184782	703,286313
427,237976	350	740,635449	2718,61745	607,18536	591,307664	684,042524	718,169816
429,245728	350	743,850133	2739,86013	621,82549	602,760382	689,645047	702,987494
431,251953	350	766,413278	2758,18848	633,128153	606,990011	695,31349	713,884406
433,256592	350	790,112803	2771,72923	646,022805	600,591935	706,318484	711,229899
435,259766	350	795,866834	2780,29195	654,865596	610,062187	724,400942	730,85601
437,261353	350	800,795054	2772,31821	670,498302	606,903223	727,760904	713,839199
439,261383	350	815,911068	2772,68494	665,6603	605,70851	728,252088	712,591753
441,259888	350	818,316516	2721,53411	665,829536	606,407747	733,393262	718,820419
443,256836	350	817,707111	2708,74402	666,060687	595,606604	728,911358	702,622755
445,252258	350	819,192332	2674,67204	664,507059	586,146837	719,608496	696,896434
447,246094	350	807,283477	2624,28974	670,925508	594,527985	718,143902	685,757851
449,238434	350	803,340159	2579,61637	654,070885	583,715484	710,899999	669,993594
451,229187	350	790,077633	2525,15852	649,373089	580,083281	707,662359	665,254316
453,218445	350	786,557022	2482,74547	646,103127	577,09467	710,73409	648,998896
455,206116	350	785,145909	2419,72881	647,800697	559,931491	715,160196	634,565101
457,192261	350	776,63038	2364,46008	630,500506	554,002501	683,20404	615,840998
459,176819	350	766,196069	2332,58973	633,725827	535,341713	682,040962	617,097131
461,15979	350	762,978136	2283,25632	624,534977	534,57266	668,578975	606,402133
463,141296	350	759,246784	2216,69125	612,815249	524,336238	671,446041	580,223362
465,121216	350	745,617457	2159,12741	606,678119	503,957885	660,969335	572,834412
467,099579	350	727,482014	2106,56549	602,573738	498,850549	643,044719	554,150265
469,076385	350	725,28613	2055,16516	592,987078	488,669696	642,146346	534,022714
471,051636	350	714,010549	2003,73826	578,707949	470,843802	617,878331	525,70122
473,02536	350	710,749209	1936,15298	577,596768	465,868835	617,126984	507,011664
474,997498	350	692,369698	1878,23404	576,708343	456,343342	622,736105	493,090645
476,968079	350	686,005472	1828,02642	559,744843	444,72872	609,521222	482,007506
478,937134	350	689,837506	1757,93479	546,591288	430,264468	595,388809	467,223415
480,904572	350	667,904099	1723,84817	539,131858	408,03588	599,343882	449,676062
482,870453	350	667,515211	1675,87944	533,339121	412,74898	581,337166	436,722191
484,834808	350	654,280124	1622,53772	531,805259	397,731405	578,328024	436,164659
486,797607	350	646,790081	1573,67546	511,927027	394,749071	571,41915	421,380048
488,75885	350	623,277751	1529,39726	512,730989	381,16613	562,645327	402,926298
490,718506	350	634,799342	1493,16827	499,58343	369,215814	560,269509	391,96407
492,676605	350	613,29206	1441,32926	494,955256	361,707387	553,111861	371,084723
494,633118	350	607,798845	1394,61915	486,387429	355,240476	541,09613	370,675845
496,588104	350	596,45823	1345,75976	476,512294	340,883463	535,279701	355,996915
498,541504	350	597,376385	1307,01942	457,694578	330,92689	522,491186	335,957751

This journal is © The Royal Society of Chemistry 20xx

Please do not adjust margins

500,493347	350	586,531833	1255,66009	459,012639	317,187609	514,715943	331,244625
502,443665	350	573,42912	1221,18869	450,917592	307,524847	508,795376	319,364038
504,392365	350	562,00688	1175,05092	444,863505	299,11668	501,761526	304,827691
506,339539	350	548,537836	1132,63284	436,530533	290,031712	493,636602	298,33999
508,285095	350	532,394266	1095,11945	428,501198	280,88322	475,569305	279,959843
510,229126	350	526,321718	1045,48223	418,727806	271,843646	468,517386	272,609428
512,17157	350	517,527525	1002,2926	411,463633	267,101518	462,152237	251,412813
514,112427	350	503,8639	974,800877	395,774432	261,501039	448,167544	249,301192
516,051697	350	493,136971	942,532037	382,932728	247,911744	439,709803	237,29997
517,989441	350	482,202259	902,334026	383,107308	243,566674	428,844606	235,453199
519,925598	350	470,214874	866,341952	365,662662	236,382472	419,223709	216,158308
521,860168	350	460,338396	832,529918	354,574528	223,564783	407,297662	215,431884
523,793152	350	448,596265	790,571953	348,702973	214,519934	402,40052	207,60691
525,724609	350	437,520881	774,927097	343,934678	208,960682	389,94166	189,790002
527,654419	350	431,436789	746,656078	340,320063	201,226461	388,019696	182,839367
529,582764	350	425,712921	720,81945	333,290141	196,660438	378,526119	174,727354
531,50946	350	420,184749	702,254626	323,015092	193,365907	371,550492	176,284158
533,43457	350	417,369024	670,341247	321,592686	183,427239	367,731931	166,697929
535,358093	350	402,805024	651,793497	309,829456	180,629246	353,585406	164,492177
537,280029	350	396,249474	621,514963	305,526219	174,640705	345,637052	156,642242
539,200439	350	388,073764	606,298901	298,132094	168,53975	348,812173	149,015275
541,119263	350	388,044871	575,857569	287,933865	163,444312	332,204702	144,541337
543,036438	350	367,360646	561,260964	286,079118	157,115839	327,17313	135,843078
544,952087	350	360,091187	531,813146	273,626801	152,890687	317,258403	130,756529
546,866211	350	353,594168	517,242522	272,619793	147,614765	305,253862	126,298549
548,778687	350	346,803847	497,022832	260,409421	146,107481	303,468504	120,860046
550,689514	350	336,262763	478,979344	253,953978	138,86023	298,776604	117,083972
552,598816	350	330,502003	463,508896	251,988943	135,004141	288,217236	108,850938
554,506531	350	323,200251	445,941523	246,036002	135,121399	278,136001	106,713823
556,41272	350	317,479005	434,59741	239,066689	123,022974	274,317141	102,922889
558,317261	350	306,91726	417,058413	236,106194	119,703996	268,470095	99,5270774
560,220215	350	296,915761	403,061195	236,578959	120,159648	262,305511	95,445615
562,121582	350	293,40349	390,402398	223,588079	112,955089	254,342539	91,6216946
564,021362	350	285,235438	368,856494	213,158892	103,609092	239,638784	90,7643435
565,919556	350	275,498818	359,788301	210,216157	105,954523	243,689442	84,7836858
567,816162	350	273,402715	345,494726	206,604859	103,882424	236,991345	82,7446162
569,711182	350	268,732358	335,48683	195,606566	96,7782031	227,58738	79,219256
571,604614	350	264,866315	325,605382	198,087433	99,2629069	222,109816	75,8454644
573,49646	350	250,197297	314,156863	194,298436	89,7243321	216,42102	71,9710482
575,386719	350	254,238825	298,690969	178,938348	88,4617794	210,511529	69,9471826
577,27533	350	235,726509	289,250462	183,872433	91,28728	203,193318	68,1730216
579,162354	350	234,800694	277,652613	170,433488	83,0622283	200,544403	63,780992
581,047852	350	227,511585	268,277327	174,121815	80,4078805	192,386709	62,052453
582,931641	350	224,70116	249,969879	168,414837	76,3241625	184,132985	60,8766943
584,813965	350	216,643088	242,344528	161,491437	75,9747553	182,788301	60,2172419
586,69458	350	208,172774	238,147942	159,697045	73,1904511	177,679987	54,4849835
588,57373	350	202,560661	229,762829	150,81807	71,5516203	166,95688	51,7501064

This journal is © The Royal Society of Chemistry 20xx

Please do not adjust margins

590,451111	350	197,635748	219,355296	144,601324	67,5687491	164,669257	48,3419596
592,326965	350	189,052074	210,454437	139,836016	69,058747	161,799785	48,1262453
594,201233	350	183,792163	203,967894	135,478688	62,1960212	156,398009	44,40063
596,073914	350	178,66979	189,881097	135,799732	59,0358703	153,259795	46,5238672
597,944946	350	173,383658	186,294702	129,514163	60,217249	146,476206	44,6831311
599,814453	350	170,226589	183,108323	127,315042	56,8526875	142,908642	42,4993423
601,682251	350	164,064105	173,142025	124,750461	55,5844254	135,769453	37,499788
603,548523	350	164,749035	164,951779	122,788018	51,2204417	134,346965	37,7954472
605,413147	350	153,555157	167,775487	116,375576	51,9810883	132,989685	35,4787276
607,276245	350	154,824783	155,876543	115,541572	51,5259335	125,645293	36,6430836
609,137695	350	144,638515	151,037409	112,642167	49,2856992	121,976025	33,6848072
610,997437	350	144,079079	146,733475	106,949668	45,5266996	118,645062	35,0640724
612,855713	350	143,661465	145,471293	103,982799	45,0727949	114,45613	31,7004642
614,712341	350	135,847304	140,39858	101,249399	41,6090376	113,633304	32,2561959
616,567322	350	132,973107	131,965359	100,038035	44,2983656	109,035106	29,9372516
618,420715	350	128,443104	129,042105	96,7859123	42,9446657	107,021074	26,8700318
620,272522	350	125,211794	128,464472	92,5442807	40,2869835	100,71139	27,7774962
622,122681	350	118,172791	113,751395	89,8987031	39,1387901	98,1748448	24,4286705
623,971313	350	112,309691	113,682218	87,1019038	38,102832	95,2065371	23,451151
625,818237	350	110,364299	109,164709	84,2954777	37,0100463	98,3543432	22,2092826
627,663574	350	108,73959	108,782548	80,9052434	34,296865	89,5266867	25,4948584
629,507324	350	107,062737	101,626641	78,8050373	33,0216931	89,8714188	21,8781205
631,349365	350	104,638785	98,141507	78,9592928	32,8533426	83,1971464	20,6458891
633,18988	350	97,8394642	93,7414746	75,2877108	30,9021095	82,6192823	22,5228815
635,028809	350	96,237995	88,4806108	71,2926393	29,3394283	80,4476975	18,0844731
636,865967	350	98,5163419	86,3048157	69,862419	29,4828853	76,1317593	18,4987047
638,70166	350	90,3563018	82,8749978	67,1009376	29,0155047	73,5636105	19,2800793
640,535645	350	85,9918059	79,6728618	69,5018727	27,4629661	71,3271658	15,9995267
642,368042	350	88,9008085	78,2218578	65,1398934	26,8183471	71,8969647	17,6592914
644,198853	350	84,6331277	75,5805687	63,1496126	27,3613357	68,3670447	16,4401984
646,028015	350	82,6929392	73,5338737	60,1197922	25,7416716	69,028745	15,2005909
647,855591	350	78,9700094	72,5318556	59,5118024	23,49715	64,9225314	15,3874585
649,681519	350	75,2965178	65,511308	54,2762376	24,1110755	62,0506379	15,4698713
651,505798	350	73,9408816	65,2909386	55,2819136	27,0525864	59,3665539	15,6311035
653,328491	350	69,4534338	64,2157357	53,1949669	21,6301249	58,0755501	13,4253884
655,149536	350	70,0014282	62,7736512	49,8420638	20,5245633	57,3988353	13,1790873
656,968994	350	65,1426954	59,5697432	50,8393393	22,8883164	52,4864553	12,4434945
658,786743	350	67,9420772	56,6738058	48,7973858	20,2104597	55,3253858	13,3962693
660,602905	350	61,2230542	57,705152	47,7176537	19,1606345	50,8684306	11,6376279
662,41748	350	62,699997	53,3592604	43,128803	17,7430588	51,1971316	12,7889574
664,230469	350	58,407993	49,7531269	43,6149625	16,7736589	47,9853579	11,1123078
666,041748	350	56,7048497	49,6429355	44,2972368	16,4017709	48,475208	11,2328797
667,85144	350	54,0378557	48,2678813	43,7139443	16,2450567	46,288973	9,05123438
669,659424	350	48,9957275	45,5388878	39,7657924	14,2559979	42,7453012	10,379721
671,46582	350	51,9784608	46,0908317	37,5837563	15,4850911	42,2832266	10,491211
673,27063	350	50,3547404	43,9375652	36,9196025	14,3264912	40,4178586	9,21577922
675,073792	350	49,536254	41,9888188	36,2793389	13,8450697	38,7659757	8,01591819

This journal is © The Royal Society of Chemistry 20xx

Please do not adjust margins

Please do not adjust margins

676,875305	350	46,6793226	38,829753	35,4596328	13,4353143	37,1309676	9,33322361
678,675171	350	45,3644585	37,0059589	34,550691	14,9644323	35,7124407	6,49370912
680,47345	350	45,7363776	37,8919965	37,0628714	13,020308	35,5835143	7,60400684
682,27002	350	39,4695866	36,3922774	32,1985002	13,0654274	29,6876721	7,22340467
684,065063	350	41,9543107	32,7289534	33,8390121	13,3136803	32,9594646	6,83910893
685,858398	350	38,9015281	33,7665282	31,8082958	10,7580709	33,0763158	7,55695383
687,650024	350	37,8129567	31,4443088	28,8709003	11,7943538	31,0590697	7,05334857
689,440186	350	38,1057817	30,3877176	27,3983983	9,56008939	31,2336035	6,65981717
691,228577	350	33,7057815	29,478261	27,5145427	9,46194508	28,7819445	4,81792904
693,015381	350	36,4695172	28,2141337	24,908012	10,4138223	29,9882611	5,75501902
694,800476	350	34,9154754	28,8247599	26,34239	9,38040723	28,2293168	5,75358155
696,583984	350	33,6086413	25,8402677	25,0022969	10,7637344	26,2744028	5,05470319
698,365845	350	33,703078	26,0165276	25,2797619	8,40684929	26,1407647	5,79303585
700,146118	350	32,2759482	22,5822107	21,6360312	9,58665685	24,5788106	4,88143105
701,924683	350	29,4735766	23,2958199	24,4341107	9,41430534	24,2551106	5,21297594
347,741211	355	0	0	0	0	0	0
349,808533	355	0	0	0	0	0	0
351,874329	355	0	0	0	0	0	0
353,938568	355	0	0	0	0	0	0
356,001343	355	0	0	0	0	0	0
358,062561	355	0	0	0	0	0	0
360,122253	355	0	0	0	0	0	0
362,180481	355	0	0	0	0	0	0
364,237152	355	0	0	0	0	0	0
366,292328	355	59,7098128	255,434845	57,3939173	205,220851	64,4026642	64,5544485
368,345978	355	68,8471363	295,68076	57,1408248	177,943732	72,2329645	69,9121533
370,398132	355	69,9968241	356,514621	69,3137099	160,241918	86,1625893	82,6454667
372,44873	355	92,6590804	409,655194	80,1711926	167,652544	102,034798	98,343834
374,497803	355	103,805312	483,022111	91,5166793	173,786687	114,539297	110,862413
376,545349	355	122,038755	545,588605	99,1484885	174,975356	134,413416	136,561484
378,5914	355	135,887488	605,43422	120,39132	187,455917	139,458657	154,949459
380,635925	355	154,133356	703,812099	125,875663	198,958139	161,918755	179,848211
382,678955	355	162,838342	790,082873	144,563596	223,335418	183,397934	195,929536
384,720398	355	197,405441	853,318135	158,194029	243,360652	210,027058	227,227325
386,760376	355	217,284527	960,461963	169,697966	260,304554	221,852653	243,947809
388,798767	355	239,127404	1041,96096	195,588898	286,620447	243,63218	279,040744
390,835663	355	252,92653	1153,75102	209,520238	309,701275	262,936902	293,161504
392,871033	355	286,824611	1239,7437	234,116545	327,26016	282,540096	320,644822
394,904877	355	313,416093	1347,81906	262,505125	356,944135	320,396663	349,073324
396,937195	355	352,511319	1469,65441	292,071434	389,09878	354,189201	391,741748
398,967957	355	366,88753	1572,81929	310,778683	417,130018	370,966424	435,64538
400,997253	355	408,985527	1693,85379	331,72787	435,637076	409,623664	449,847151
403,024963	355	431,093837	1788,46923	350,663464	449,293525	428,214021	483,624922
405,051147	355	461,776558	1901,0274	372,416628	472,715922	452,316504	506,094902
407,075806	355	484,521001	2001,46822	399,063451	484,683086	467,853934	522,152874
409,098938	355	512,639096	2098,90316	420,342346	486,991998	505,72543	540,024543
411,120544	355	546,034497	2186,80799	437,637912	511,781186	520,972302	547,299991

This journal is © The Royal Society of Chemistry 20xx

Please do not adjust margins

413,140564	355	571,584893	2270,87631	462,886733	530,438404	544,407385	571,285296
415,159119	355	607,489091	2371,01584	489,869484	530,919578	566,142692	587,066066
417,176086	355	631,276987	2452,4316	512,476224	545,988039	591,444004	617,151063
419,191528	355	655,916161	2479,14134	542,353092	543,239208	602,063316	622,059101
421,205444	355	679,745858	2554,91381	545,27348	556,202041	618,739926	632,698796
423,217834	355	695,987367	2591,51986	573,791498	573,502593	641,619206	643,44582
425,228668	355	729,622023	2638,57443	597,768138	569,713952	651,792916	650,309051
427,237976	355	746,726389	2635,68519	616,349379	577,240214	663,352562	666,754344
429,245728	355	757,876141	2688,11447	614,479524	580,731174	685,438498	672,548369
431,251953	355	782,068419	2699,57014	629,680839	593,907801	696,018771	673,683519
433,256592	355	807,569775	2692,85056	651,958584	588,884284	699,605456	682,198392
435,259766	355	815,957738	2742,62766	657,008519	598,464733	713,466198	698,260208
437,261353	355	830,627345	2722,55357	670,627557	601,364172	717,604436	700,365188
439,261383	355	847,880769	2737,31414	674,319088	606,336974	725,347887	693,617995
441,259888	355	843,080215	2729,49198	680,140646	591,879374	729,073044	692,058014
443,256836	355	841,910832	2684,9825	684,399357	585,896633	729,234594	681,323844
445,252258	355	847,984021	2681,06004	684,750098	601,801322	733,233591	672,848671
447,246094	355	846,037632	2628,9254	679,286367	578,463756	721,593955	673,678084
449,238434	355	844,37534	2591,11637	679,721508	574,405565	720,202272	651,037706
451,229187	355	834,002123	2553,78955	678,931986	578,687817	716,328572	661,320076
453,218445	355	832,940871	2505,56437	667,27537	577,852476	712,500088	654,053212
455,206116	355	818,226965	2440,03561	664,251379	561,76567	709,797546	630,519595
457,192261	355	811,385937	2412,65275	664,503791	559,167362	693,410966	621,278764
459,176819	355	802,945012	2363,63604	655,972971	551,720202	709,182033	610,555326
461,15979	355	800,692289	2312,3294	642,353289	537,124224	700,088188	596,029787
463,141296	355	797,189815	2249,45541	644,171791	533,877226	691,700046	584,76407
465,121216	355	777,30121	2189,11473	627,502741	521,138001	673,947412	574,002622
467,099579	355	771,307019	2144,46576	616,530371	509,736872	665,266585	559,997668
469,076385	355	765,179701	2090,71778	623,708981	499,989346	656,293551	538,352568
471,051636	355	750,450894	2027,66025	599,634106	484,0144	651,756844	527,608124
473,02536	355	736,11565	1989,09644	601,402561	471,697627	650,838215	519,121497
474,997498	355	722,006774	1924,08501	592,797223	461,745912	642,555211	505,394962
476,968079	355	717,502762	1854,23725	582,817767	451,592502	625,823798	491,014361
478,937134	355	710,115856	1808,4909	574,37758	441,773185	622,027148	468,240613
480,904572	355	707,817969	1763,34116	574,200926	429,479007	613,646343	463,991118
482,870453	355	704,886399	1715,58255	558,766644	418,844325	607,37515	447,323996
484,834808	355	689,349454	1669,74762	544,092683	406,944744	601,464783	433,186895
486,797607	355	680,210829	1608,32965	539,631148	398,586363	594,958622	424,172204
488,75885	355	663,975186	1576,07024	527,524195	379,145562	589,835483	419,990873
490,718506	355	666,298778	1522,7302	525,228044	386,076834	570,379034	393,451972
492,676605	355	657,218527	1472,3271	518,627761	372,450287	566,409223	385,030083
494,633118	355	638,372443	1429,61687	509,441896	366,054551	556,44762	373,028932
496,588104	355	628,769664	1384,16997	506,394071	349,401967	551,946116	372,43068
498,541504	355	622,520361	1336,52156	489,862345	343,067363	548,680918	341,99173
500,493347	355	605,156204	1284,53179	477,104092	332,775145	528,819183	334,232778
502,443665	355	598,578043	1252,3994	474,227346	322,575717	532,255989	331,156783
504,392365	355	594,355848	1228,78595	461,219454	308,130247	517,728252	309,635104

Please do not adjust margins

506,339539	355	569,210137	1170,48332	451,401427	299,409561	509,66586	304,666871
508,285095	355	561,71693	1129,16701	441,585973	290,382799	488,565787	291,62982
510,229126	355	545,126099	1072,27728	431,017967	286,291456	488,33172	277,802969
512,17157	355	538,269788	1042,65612	425,129425	270,621742	473,027195	267,224117
514,112427	355	527,596428	1013,53514	416,282388	267,156485	457,888189	255,920152
516,051697	355	521,893796	951,917886	396,203416	247,038315	460,396526	245,246077
517,989441	355	496,43351	938,388207	391,761877	243,302839	442,640089	233,321979
519,925598	355	498,315432	893,75347	381,687072	234,843202	430,867453	225,828598
521,860168	355	480,512813	852,244068	376,475051	225,973327	421,192384	212,934072
523,793152	355	466,802507	823,545712	367,630955	214,844423	410,465275	210,215219
525,724609	355	465,604525	795,728104	360,556589	212,520456	404,741312	198,403104
527,654419	355	455,750149	775,728249	347,700119	206,896956	397,353177	195,536297
529,582764	355	448,186422	744,688876	340,924136	203,402209	385,826471	181,643106
531,50946	355	432,761537	723,84897	338,833871	197,773129	377,73717	177,022194
533,43457	355	427,268466	705,304028	335,749128	186,168779	378,141517	174,543533
535,358093	355	420,982408	672,231328	325,659326	185,503046	373,135424	162,458739
537,280029	355	413,337762	648,119966	313,21472	178,924562	356,841818	164,028572
539,200439	355	399,246213	619,273745	308,187374	177,154758	355,904166	154,00251
541,119263	355	394,036756	596,245378	298,495362	164,604519	343,419616	143,558752
543,036438	355	388,193579	576,366009	290,545796	155,83122	336,948913	140,065981
544,952087	355	375,795416	551,765457	291,325048	152,728726	323,302847	134,376131
546,866211	355	361,194295	533,689325	278,658576	152,79154	322,059673	130,58823
548,778687	355	356,87335	518,465699	272,545873	144,974714	314,478954	126,556297
550,689514	355	351,338638	498,808761	269,627674	136,696421	304,040229	119,779718
552,598816	355	339,421735	472,597729	260,652674	134,97744	291,637458	118,022806
554,506531	355	331,172632	455,588984	257,214424	133,042465	286,389899	112,269646
556,41272	355	325,077839	446,386579	250,321151	128,486692	284,285977	106,440046
558,317261	355	316,723597	429,841395	247,000076	124,904778	278,656484	100,482428
560,220215	355	309,772706	412,492594	237,989636	120,227577	270,326353	99,7313559
562,121582	355	301,762819	392,116221	228,892917	121,070643	258,632802	92,3385519
564,021362	355	292,173794	385,28427	225,81431	112,128792	249,071236	88,3428535
565,919556	355	288,405348	364,148065	218,029371	106,897156	245,953627	87,0474195
567,816162	355	274,247304	355,90999	210,565586	106,610674	238,220134	87,1339964
569,711182	355	269,795386	336,663763	204,08249	102,289775	237,022802	82,5009763
571,604614	355	265,094689	332,089418	201,260484	95,789662	224,421627	78,9670844
573,49646	355	258,110548	313,524806	199,40962	91,9700994	221,938201	73,7161838
575,386719	355	256,849565	302,713599	191,416422	90,9250586	215,141241	72,6214235
577,27533	355	248,486924	299,614467	190,349367	84,2058537	208,214975	70,9112425
579,162354	355	242,69617	278,976259	176,445839	85,5148163	203,623249	66,984703
581,047852	355	230,049402	280,031381	178,409394	81,0803306	197,098968	63,8123889
582,931641	355	231,290177	262,491362	169,905725	76,5613033	187,257819	63,7241761
584,813965	355	220,784319	252,775539	164,303919	74,9816175	189,340026	59,9893859
586,69458	355	216,278328	243,46526	161,944907	72,5327234	187,320843	56,1694339
588,57373	355	206,296381	233,354723	158,933917	71,565082	173,05619	52,5297228
590,451111	355	199,12183	221,273353	149,615542	68,8664211	167,92483	50,2089485
592,326965	355	196,739832	211,461175	148,791039	64,8709421	163,550798	47,5702845
594,201233	355	187,375326	204,709764	143,436328	62,2950312	157,210506	46,324489

This journal is © The Royal Society of Chemistry 20xx

Please do not adjust margins

Please do not adjust margins

596,073914	355	183,634565	196,361955	139,283099	60,3347128	155,010351	44,7171805
597,944946	355	180,780062	190,065297	135,356111	60,7438727	151,34414	42,6417963
599,814453	355	174,154353	185,54446	134,849901	57,5465984	145,430644	41,9869239
601,682251	355	168,300302	176,248925	129,457383	55,472762	139,916335	37,7452724
603,548523	355	161,678498	170,53039	123,102892	52,7749263	138,41827	39,9609367
605,413147	355	154,694428	162,795413	121,515339	52,7773653	134,595522	37,6004514
607,276245	355	160,241062	159,335426	116,007413	52,9725217	123,459517	35,8034585
609,137695	355	149,634614	154,044659	114,816961	47,7561014	123,081882	34,3757654
610,997437	355	144,513959	143,331295	112,289881	47,0240782	124,88787	32,8865388
612,855713	355	143,831828	141,179018	108,473145	47,2249621	120,520704	32,2570429
614,712341	355	142,93055	136,033819	109,963047	45,5098657	115,499511	33,8142542
616,567322	355	141,240059	135,25779	97,6346963	45,3742529	112,209191	31,0909845
618,420715	355	129,352782	128,623939	98,2400015	38,1445898	108,215698	26,0701015
620,272522	355	130,090447	123,067896	95,3029631	42,3907228	103,352267	29,9105048
622,122681	355	124,343539	116,192626	91,8918921	35,5253592	102,089919	24,3314977
623,971313	355	119,570074	116,04573	89,911884	34,5994925	98,1815126	25,4185481
625,818237	355	112,761245	108,942374	89,893504	36,8837681	94,4582322	26,1708541
627,663574	355	109,499606	105,580242	84,8148782	38,1646324	90,3189135	23,6045014
629,507324	355	109,381144	102,059246	80,8159398	36,4725784	90,6697139	22,9706992
631,349365	355	105,700188	99,5952114	79,5589249	31,2314963	86,2185375	21,7678773
633,18988	355	100,857034	96,9055337	76,7283595	29,0414562	85,2877336	20,2199241
635,028809	355	100,043032	93,8328087	76,1171964	31,1914512	83,8223474	18,1400419
636,865967	355	99,0703526	89,4531823	71,5208568	30,1085853	75,550984	21,1814445
638,70166	355	91,9500771	84,6873673	65,9901947	30,5028853	76,9153949	18,9324948
640,535645	355	89,9510563	82,5951352	70,4143683	27,2870425	75,673115	16,3863136
642,368042	355	86,6574368	78,2528004	65,2070702	27,6035293	67,6298266	15,5574744
644,198853	355	87,3179182	75,2014133	66,6736459	25,2946673	70,6194699	17,6698436
646,028015	355	84,1953494	70,8318831	58,9759053	22,4785732	67,7593262	16,4605004
647,855591	355	77,3346719	70,23205	62,8357366	21,994664	64,8837466	16,075025
649,681519	355	77,8441449	66,7966931	55,2638304	25,3530837	64,0868459	15,7808587
651,505798	355	74,2413222	66,7840031	55,8735052	24,1173605	59,4663532	13,9365565
653,328491	355	70,7274663	63,8227196	53,8384524	24,3836989	56,9601383	12,0645133
655,149536	355	69,3469481	60,8758423	52,0113001	20,6183491	54,4676995	15,5678457
656,968994	355	65,9584604	58,4192695	52,419698	19,8328558	53,6280034	11,0969243
658,786743	355	65,9833248	56,2698857	50,1444897	20,8028794	57,3868202	14,2528069
660,602905	355	62,243468	55,6239826	46,2695874	18,1238778	48,0657333	10,3990903
662,41748	355	63,7950654	51,3726351	45,1075781	18,8904393	49,8486042	12,5935407
664,230469	355	61,1758754	53,5329645	44,0201013	17,0643641	49,0638175	10,8576848
666,041748	355	58,3409973	48,5229729	44,5915276	17,6850042	46,5973774	11,9554487
667,85144	355	55,9407078	49,9367908	41,7594309	17,2410189	42,7823937	10,4160759
669,659424	355	53,2913271	45,6958558	40,3640911	15,0055398	41,5853729	9,05877036
671,46582	355	55,7167408	47,3233153	38,8247121	16,5032063	41,8256498	11,0393441
673,27063	355	53,6540185	43,3430506	38,5801298	16,865498	39,4086198	8,80670894
675,073792	355	51,0744412	41,14091	38,455686	13,7171804	42,2693105	9,02195266
676,875305	355	48,8791074	37,0595416	35,0705004	15,7022001	38,1843249	9,21848937
678,675171	355	45,3097869	39,7484517	36,4671929	15,1083147	36,5991268	6,84061132
680,47345	355	44,6794758	37,632911	35,8436188	11,9185126	34,2854914	9,11385321

Please do not adjust margins

Please do not adjust margins

682,27002	355	43,0409316	36,9662056	32,8791779	12,1591441	35,202082	8,54899405
684,065063	355	40,7848811	34,7897518	29,789581	13,799501	34,8188169	5,68502712
685,858398	355	41,6028133	32,9047288	32,0028341	11,7047054	31,630946	8,08226405
687,650024	355	37,5397904	34,0386667	31,3338678	12,1168911	31,3617755	6,4922103
689,440186	355	37,827237	30,9063014	27,6937295	11,8437856	29,8274034	5,10566238
691,228577	355	35,0350134	28,2108537	27,7073529	8,53573738	29,3396846	6,71073842
693,015381	355	31,5004961	29,2886324	24,6574541	9,76540026	29,3131017	7,33017872
694,800476	355	35,517245	29,583186	27,8710563	10,870101	29,1043656	5,01941631
696,583984	355	32,5289294	25,3583311	23,6395306	9,09497828	28,1095843	5,03967523
698,365845	355	30,5942247	25,8348377	24,2124927	7,70178444	26,2620124	7,17644738
700,146118	355	34,2762673	26,2431749	23,8006365	9,86916962	26,3669025	6,19030579
701,924683	355	31,5492119	22,9744754	22,4626924	7,35373187	24,2199984	4,78791621
347,741211	360	0	0	0	0	0	0
349,808533	360	0	0	0	0	0	0
351,874329	360	0	0	0	0	0	0
353,938568	360	0	0	0	0	0	0
356,001343	360	0	0	0	0	0	0
358,062561	360	0	0	0	0	0	0
360,122253	360	0	0	0	0	0	0
362,180481	360	0	0	0	0	0	0
364,237152	360	0	0	0	0	0	0
366,292328	360	0	0	0	0	0	0
368,345978	360	0	0	0	0	0	0
370,398132	360	45,7097427	231,495854	53,1246781	215,423049	64,0148547	58,4137905
372,44873	360	66,8746876	277,031307	60,46386	185,168743	72,2203271	76,0413702
374,497803	360	77,9959228	326,587566	67,1203273	174,734176	87,3325659	84,7097826
376,545349	360	85,6431871	387,376733	74,435	169,447679	97,3978625	94,4862641
378,5914	360	105,834898	437,008026	82,3687874	176,969966	109,757736	105,189232
380,635925	360	121,951383	512,239392	92,898281	175,674036	120,260745	128,411834
382,678955	360	133,006089	578,089146	113,538408	191,17647	137,409259	150,844744
384,720398	360	153,606564	654,379466	121,626874	203,373786	158,809404	179,232681
386,760376	360	175,131879	747,269794	149,308961	218,262034	173,001574	194,154476
388,798767	360	188,83493	838,808899	158,030807	241,373364	197,523637	216,628927
390,835663	360	213,581703	923,282339	178,220682	258,15004	212,26091	230,454646
392,871033	360	243,718432	1004,39547	193,999252	278,402268	240,018651	269,375683
394,904877	360	265,628882	1112,27916	216,529432	301,663328	263,984776	286,872554
396,937195	360	291,839357	1226,76304	237,074522	325,527134	287,842316	313,3694
398,967957	360	325,740349	1317,12891	261,623957	345,91038	313,067143	341,891706
400,997253	360	349,561028	1422,33141	280,469383	374,404696	350,2493	366,272388
403,024963	360	384,777033	1554,75613	315,990465	401,785888	377,16578	403,947562
405,051147	360	427,75736	1670,83624	337,278049	430,073805	395,816814	437,529084
407,075806	360	462,962849	1790,89403	365,071902	458,506199	429,632519	459,970138
409,098938	360	487,10769	1894,27517	393,179202	476,134348	473,954281	492,236738
411,120544	360	511,307311	1990,12709	417,780038	485,460541	490,784527	506,48918
413,140564	360	545,244089	2070,91054	448,927683	501,921391	508,649247	523,522848
415,159119	360	578,405537	2159,72181	456,188461	516,060341	534,673679	544,674438
417,176086	360	596,255458	2251,73459	484,50067	532,277668	558,540129	547,519357

 This journal is © The Royal Society of Chemistry 20xx

Please do not adjust margins

419,191528	360	639,4933	2309,90405	501,92061	536,168159	571,001391	571,936465
421,205444	360	665,255774	2385,96991	529,024648	541,876538	594,775656	579,333837
423,217834	360	695,357943	2440,77589	549,912427	541,510475	618,968765	606,967301
425,228668	360	717,912498	2502,87108	563,875953	555,268609	618,415137	612,727384
427,237976	360	747,076239	2524,99597	586,60671	566,606603	640,175577	621,775267
429,245728	360	764,588509	2568,74112	591,307448	558,371402	660,035432	638,380271
431,251953	360	781,342432	2581,48341	618,167761	565,235717	681,568936	636,255124
433,256592	360	807,05818	2622,54691	635,7368	569,9685	686,169561	648,876687
435,259766	360	817,001438	2651,45623	662,50638	582,31432	708,929075	655,09945
437,261353	360	847,970477	2665,09171	662,425605	579,518211	714,645305	652,679853
439,261383	360	852,866578	2679,85913	673,102706	586,822176	720,201332	653,593483
441,259888	360	859,927193	2653,72432	682,754053	592,490358	735,37767	656,836481
443,256836	360	868,180508	2655,40457	690,41923	580,539004	726,901835	647,082952
445,252258	360	866,484076	2618,86597	672,827624	575,135254	722,493792	660,803027
447,246094	360	856,531428	2585,07517	687,53298	572,539606	723,231267	653,023565
449,238434	360	850,617681	2555,7716	685,685067	564,249535	721,83335	640,57391
451,229187	360	857,395167	2528,72176	678,23266	571,135748	718,823907	638,215174
453,218445	360	852,840266	2477,82986	680,739177	552,089734	716,377734	623,302039
455,206116	360	851,342164	2470,20399	669,85088	553,297001	716,221042	629,093645
457,192261	360	847,834832	2399,09124	672,161836	548,687543	708,313123	607,64844
459,176819	360	842,913261	2358,81589	664,444939	543,638198	704,713898	600,726149
461,15979	360	838,819137	2305,64807	659,958766	542,552749	699,52905	597,24432
463,141296	360	828,04481	2294,77682	655,319123	528,765356	696,917424	587,975173
465,121216	360	805,418384	2230,41281	641,08891	516,032364	683,262289	568,815681
467,099579	360	801,504584	2163,99059	644,442555	514,376652	680,061455	552,196792
469,076385	360	791,532011	2110,48912	631,802778	497,297775	678,895496	529,818327
471,051636	360	773,971866	2049,57204	624,90403	489,62119	675,163781	524,939666
473,02536	360	769,236445	2003,10602	621,427007	482,470692	663,20814	509,428625
474,997498	360	757,961753	1939,04658	604,044756	468,573742	652,443205	504,740296
476,968079	360	756,4405	1900,22442	602,299818	459,022253	654,952153	484,985785
478,937134	360	748,316954	1829,83612	592,796563	445,442661	645,793527	477,829766
480,904572	360	731,396806	1796,80322	580,610158	440,562356	631,210558	457,846989
482,870453	360	730,98085	1744,78275	572,46608	423,57455	620,178104	453,217257
484,834808	360	716,642095	1702,24744	567,869178	419,53923	615,665886	446,320106
486,797607	360	708,386342	1649,48824	562,344355	415,128845	622,353249	434,427477
488,75885	360	707,146225	1612,31119	551,190422	399,617849	605,361348	420,464846
490,718506	360	691,998039	1570,58116	550,306004	387,685944	603,669363	402,235696
492,676605	360	685,336841	1522,16782	528,485409	380,805652	591,572545	390,123682
494,633118	360	670,974322	1459,66914	531,420891	370,863349	589,44435	377,690153
496,588104	360	658,500151	1420,69618	521,208739	356,523271	573,030467	363,005751
498,541504	360	645,128189	1369,81042	508,783025	350,343936	560,787186	355,994083
500,493347	360	641,092099	1329,62098	497,395838	337,345102	553,763159	332,84732
502,443665	360	621,734575	1292,55376	490,001644	329,565331	548,06187	325,027162
504,392365	360	616,533656	1234,68866	485,17853	321,903007	545,545164	325,959707
506,339539	360	599,009672	1193,51938	468,257857	302,347441	533,58622	300,551968
508,285095	360	585,731121	1152,89408	454,337547	295,060976	514,792559	292,234698
510,229126	360	572,890886	1115,08752	459,23936	289,633762	503,277394	283,499342

Please do not adjust margins

512,17157	360	559,751556	1069,20211	437,491222	276,740144	493,366079	268,56527
514,112427	360	550,955419	1032,43929	423,565685	264,241974	483,726026	267,517463
516,051697	360	536,924559	998,846697	424,724396	258,878061	471,01539	249,406774
517,989441	360	527,460389	960,278183	412,700565	248,817526	459,60678	237,36127
519,925598	360	514,99915	921,956066	398,959996	242,863033	449,87101	232,450793
521,860168	360	495,212799	877,0595	390,150759	232,482823	431,705311	218,42212
523,793152	360	486,091202	832,775551	387,931361	223,229998	424,938845	212,155726
525,724609	360	473,6536	814,462465	378,325082	218,628293	418,511564	201,943475
527,654419	360	475,854334	788,933493	366,170815	209,913275	412,266882	198,058267
529,582764	360	462,273498	760,873007	357,965677	203,773885	400,41268	193,293367
531,50946	360	450,558997	733,30793	354,878383	198,528052	397,58117	179,81386
533,43457	360	438,245741	714,530306	343,318889	192,730877	385,565746	172,216026
535,358093	360	437,053602	691,025828	338,346022	187,616018	371,421506	172,274432
537,280029	360	418,029438	658,727531	327,043316	181,82803	369,70378	164,376527
539,200439	360	420,410638	634,62913	325,827625	175,092586	365,772554	156,787233
541,119263	360	410,966403	620,018672	316,690255	173,130113	356,791592	151,108199
543,036438	360	399,334267	586,351626	307,418742	160,984148	349,95982	147,613428
544,952087	360	390,656391	566,306472	301,517328	159,511603	339,133877	137,88982
546,866211	360	377,690303	551,765244	292,336751	155,244876	325,138457	133,572004
548,778687	360	374,634409	531,746078	283,774524	147,505825	322,672619	125,284501
550,689514	360	361,225891	506,438933	276,470709	141,988898	311,263474	125,667871
552,598816	360	354,448265	488,758478	280,172231	135,435678	304,192019	119,68377
554,506531	360	346,702125	471,015454	265,64629	132,620252	292,618257	111,812535
556,41272	360	343,503986	454,919105	253,508489	129,664832	289,311587	113,189884
558,317261	360	328,067038	439,729392	249,517182	124,485331	280,294708	101,624272
560,220215	360	316,509313	416,570588	242,889591	120,323648	273,734703	101,223669
562,121582	360	311,126955	403,937913	242,046903	114,870529	261,727614	94,6728236
564,021362	360	306,203902	387,133137	229,116009	110,304188	255,23443	91,941561
565,919556	360	296,63767	372,474363	228,865309	112,431794	251,228735	90,2825646
567,816162	360	289,667849	360,489842	218,594887	103,467488	243,356715	86,3432022
569,711182	360	280,809441	349,406156	214,761238	100,420594	234,589517	83,0853559
571,604614	360	276,371456	339,655857	209,614498	96,8680467	233,089781	81,4234714
573,49646	360	264,638883	325,201026	201,460399	93,0513505	227,539804	75,6231676
575,386719	360	262,499005	310,049801	198,801622	92,3794896	224,004555	74,1369777
577,27533	360	259,714038	301,237599	193,961477	89,6953297	219,886353	72,654215
579,162354	360	247,020538	297,468995	189,111888	87,8011196	209,309104	64,4386549
581,047852	360	238,760161	283,602366	187,206629	84,0827581	200,4525	64,4436756
582,931641	360	234,080913	269,644058	176,052523	81,1327024	199,49891	63,7527607
584,813965	360	231,46878	251,823203	174,511897	78,2967298	189,14233	63,570478
586,69458	360	224,230771	245,950412	167,816938	75,3897108	181,945366	52,9890915
588,57373	360	215,669279	232,063728	162,481039	72,7105919	179,20594	53,6129647
590,451111	360	209,658788	226,374444	154,421438	70,0916816	172,59449	52,4223598
592,326965	360	200,968114	219,203362	149,963909	65,5819894	166,617965	50,2363432
594,201233	360	193,321365	208,823878	149,27384	64,6113016	162,817482	46,7150819
596,073914	360	187,19248	205,801572	139,943033	62,5361423	162,002737	45,9689585
597,944946	360	179,49519	190,82503	141,626468	58,0297622	149,41979	44,9329181
599,814453	360	177,530968	192,544737	139,484729	56,9860656	148,884059	41,579308

This journal is © The Royal Society of Chemistry 20xx

Please do not adjust margins

Please do not adjust margins

601,682251	360	176,119901	177,731867	132,851433	56,1287892	140,040654	38,7819505
603,548523	360	166,013801	172,123937	125,915749	50,5969841	135,773527	40,4945535
605,413147	360	164,023657	166,973932	120,587971	53,662491	135,993263	42,0393231
607,276245	360	161,170343	159,623618	120,762927	54,030385	130,753988	38,7533981
609,137695	360	156,344809	158,83441	118,96294	48,64959	131,355824	34,5819251
610,997437	360	153,330904	151,869976	114,448507	49,3754914	121,636872	36,4411269
612,855713	360	145,389439	145,003257	107,407405	45,3448314	120,062361	31,3489935
614,712341	360	146,232185	141,338199	111,922361	44,294403	116,147959	31,882621
616,567322	360	139,714553	131,202974	101,243188	46,4190512	111,758907	28,9499165
618,420715	360	133,235747	128,052375	103,194675	39,2519632	111,561713	30,4331325
620,272522	360	130,350794	123,615487	100,07233	39,7885965	103,192036	29,7759991
622,122681	360	128,907927	118,890154	98,5681236	40,6009301	101,144439	26,9465411
623,971313	360	121,801661	116,667449	89,3988484	34,7919609	97,7795839	25,1161817
625,818237	360	117,716394	115,483557	91,1186114	35,0207721	100,907716	24,6691699
627,663574	360	115,91066	107,723951	88,0840225	33,5251525	93,5769598	24,438963
629,507324	360	109,586445	103,655785	84,1149095	35,5312758	88,6090628	24,1011379
631,349365	360	107,919162	98,5777878	83,5343322	30,8594563	85,8198468	21,1842819
633,18988	360	104,390251	94,7486446	76,9206341	30,5621866	85,3741751	21,4175926
635,028809	360	103,498293	94,1387049	76,1389406	29,3442803	83,8472041	19,8430487
636,865967	360	97,9401795	91,0566088	71,8394793	30,1304781	82,3179387	20,79482
638,70166	360	92,9142282	83,3450528	72,8005056	27,1878262	79,5900294	20,5300648
640,535645	360	94,3746524	82,2211451	71,8657783	28,6621705	73,7528201	17,1205861
642,368042	360	87,6467755	81,1178151	67,3400643	27,3081659	73,1939795	18,3392638
644,198853	360	87,0946358	78,9754654	63,6026269	25,7899952	72,6100251	17,1782023
646,028015	360	83,6705797	74,3340511	62,7507608	24,7053373	68,2377739	16,550857
647,855591	360	83,1701786	70,5909283	62,6306503	23,5804354	68,6717606	16,3587333
649,681519	360	78,6751256	69,2903663	61,1989475	22,7733928	64,4680177	15,0525898
651,505798	360	75,067067	64,2374422	56,8742173	25,1161336	64,8264806	15,8608046
653,328491	360	73,4206911	62,7447254	57,7989489	20,6812749	61,1735909	14,4123118
655,149536	360	71,7084963	61,7470628	52,4618038	19,7185582	55,1712846	13,5253859
656,968994	360	66,8008423	57,931891	53,24507	20,6526908	56,4410089	12,4495136
658,786743	360	68,7375138	57,9244423	51,318724	20,6565681	53,4765165	10,4243605
660,602905	360	66,7086369	54,820238	48,873136	19,7513727	52,4587559	11,6839678
662,41748	360	59,4459393	55,5453404	47,8510399	17,5397983	53,077643	12,3089645
664,230469	360	61,8651998	48,5541401	47,2755636	19,1109683	50,6864528	11,1859734
666,041748	360	55,9244203	52,7764162	46,1154705	16,8367966	46,7397135	9,96123212
667,85144	360	56,9453419	47,7161031	42,3005936	16,593656	42,6249874	10,1807038
669,659424	360	54,6475013	47,6200495	41,2307	16,8207764	42,0353247	10,2976676
671,46582	360	51,3206589	45,541534	40,6942656	17,5153649	41,5946457	9,63382865
673,27063	360	52,4920286	43,0020206	39,7888122	15,4467148	42,7287353	10,5420192
675,073792	360	49,3850297	40,3616348	39,2834563	14,3084257	39,2110005	10,4654179
676,875305	360	48,3065091	39,770552	34,2331633	15,668162	38,5226117	9,68836474
678,675171	360	45,1062019	38,026301	32,3957327	12,6533215	36,193659	8,04262068
680,47345	360	42,9347488	38,0944554	36,1508531	11,8655917	33,9299606	7,45553129
682,27002	360	41,7188941	32,5705079	32,7749457	13,2730533	33,9499809	7,28704576
684,065063	360	42,7377424	34,4398974	34,3475579	12,7296483	35,5310495	6,82384879
685,858398	360	40,6039765	33,7698096	29,6768032	12,3433751	34,7632279	7,7045766

Please do not adjust margins

Please do not adjust margins

687,650024	360	38,0979212	30,8639337	31,1720542	11,5711519	28,8184275	5,75976685
689,440186	360	40,3456015	32,7030043	30,0687801	11,1064618	29,9657728	6,84941731
691,228577	360	35,2583847	30,4592147	29,103651	10,4235761	29,6856237	2,7411147
693,015381	360	34,0674994	30,2406819	29,2754436	9,85841441	28,8737406	5,88866195
694,800476	360	35,2402268	28,1650643	25,339523	9,855952	27,3887199	5,10223229
696,583984	360	33,2081191	27,5866046	23,173729	9,59885976	27,8949367	3,84211909
698,365845	360	32,9049848	24,2928652	26,4032832	9,72506692	24,8982374	5,63118352
700,146118	360	29,7526677	26,181801	23,0338094	9,76390871	28,1846359	3,76911627
701,924683	360	30,8659478	22,8847233	23,3216921	8,20120943	25,8935844	6,47195829
347,741211	365	0	0	0	0	0	0
349,808533	365	0	0	0	0	0	0
351,874329	365	0	0	0	0	0	0
353,938568	365	0	0	0	0	0	0
356,001343	365	0	0	0	0	0	0
358,062561	365	0	0	0	0	0	0
360,122253	365	0	0	0	0	0	0
362,180481	365	0	0	0	0	0	0
364,237152	365	0	0	0	0	0	0
366,292328	365	0	0	0	0	0	0
368,345978	365	0	0	0	0	0	0
370,398132	365	0	0	0	0	0	0
372,44873	365	0	0	0	0	0	0
374,497803	365	0	0	0	0	0	0
376,545349	365	64,9764343	266,203366	59,1649803	200,725472	68,0848588	72,9131607
378,5914	365	75,1163444	314,05082	64,5048339	173,897849	79,9405906	72,0025232
380,635925	365	86,4892588	365,911795	75,237204	169,390562	86,0675707	89,2461671
382,678955	365	105,384567	431,071534	83,2280685	185,759846	101,782004	105,728455
384,720398	365	119,711414	492,250421	96,5638255	177,718862	116,753816	125,932026
386,760376	365	136,448339	563,804927	110,448097	186,370069	131,029115	142,081532
388,798767	365	154,124527	646,426317	126,26422	200,484832	154,019896	161,898238
390,835663	365	174,937963	727,067048	138,567062	220,657812	173,233027	182,851975
392,871033	365	198,906221	790,540326	162,873233	236,186432	189,964986	208,457138
394,904877	365	224,132128	912,348357	179,329164	258,08451	213,566431	229,592736
396,937195	365	252,702892	1019,66973	194,02039	283,01995	240,918235	256,590634
398,967957	365	276,558658	1111,55375	209,321692	301,692686	258,791231	272,755296
400,997253	365	304,085905	1209,33037	240,649683	326,335103	302,368939	305,782224
403,024963	365	342,148908	1327,1832	269,706206	345,144619	310,082702	328,664553
405,051147	365	374,452644	1448,23306	285,030819	369,43912	341,990515	352,49062
407,075806	365	406,00911	1558,36289	316,496157	386,856681	374,391455	376,778664
409,098938	365	442,164719	1675,03865	349,65292	416,005264	416,264042	416,749329
411,120544	365	470,055312	1788,90233	369,204595	435,159546	441,557982	441,077402
413,140564	365	516,834188	1895,02419	406,19889	468,17332	465,010323	471,668461
415,159119	365	552,425711	1983,97643	433,618418	487,150945	494,620692	484,548714
417,176086	365	585,782713	2090,84255	465,84433	513,322871	526,442511	513,893361
419,191528	365	617,073729	2131,48156	476,469787	515,542291	536,474603	515,500194
421,205444	365	641,418955	2197,72623	494,056898	518,161117	556,595546	533,689347
423,217834	365	665,980236	2280,1377	515,561944	522,741714	583,797078	549,312777

 This journal is © The Royal Society of Chemistry 20xx

Please do not adjust margins

Please do not adjust margins

425,228668	365	704,182305	2343,24673	545,949928	533,603093	600,621142	558,901794
427,237976	365	714,928845	2382,8859	555,5786	530,824177	607,899956	573,514935
429,245728	365	745,081628	2436,26326	578,605556	534,957802	619,204509	582,678643
431,251953	365	777,7572	2469,11945	594,285455	540,637746	647,11316	592,379571
433,256592	365	785,713823	2508,14506	622,237497	546,970251	661,866354	604,325564
435,259766	365	826,388965	2550,24951	633,938734	567,439101	679,890212	613,876526
437,261353	365	835,591764	2554,44126	643,871285	568,876687	692,313201	616,687757
439,261383	365	847,688233	2570,50289	663,705355	564,574935	702,896639	622,470285
441,259888	365	862,014361	2596,2023	668,427543	567,983751	698,835515	635,323775
443,256836	365	862,827975	2569,25942	670,041428	561,721625	724,627744	630,005482
445,252258	365	865,76475	2565,73402	672,058146	559,26345	710,602773	624,69326
447,246094	365	882,90795	2534,37477	683,732552	555,520184	707,400075	626,980145
449,238434	365	863,608804	2496,38117	684,086496	561,812738	714,097949	622,804719
451,229187	365	876,983453	2478,7392	677,348371	551,356629	727,499198	610,378078
453,218445	365	869,889343	2437,99537	680,893199	545,192781	727,375893	616,506621
455,206116	365	855,449584	2434,0071	669,643568	546,253426	713,478818	597,821752
457,192261	365	865,805654	2359,86239	672,969548	534,586551	717,851566	600,612291
459,176819	365	849,016397	2340,26952	677,932048	540,260918	711,917871	590,412514
461,15979	365	858,649127	2298,50004	660,773298	531,989949	710,126612	577,857592
463,141296	365	861,093522	2254,95776	659,423016	531,568839	699,413629	575,229179
465,121216	365	840,489148	2180,84432	653,828791	510,525157	694,907195	567,805839
467,099579	365	830,621019	2152,42225	646,913823	505,552506	691,192317	545,997215
469,076385	365	810,095506	2087,35595	643,630571	488,424854	681,515724	541,524758
471,051636	365	807,576172	2046,11156	633,718725	486,166757	671,315961	519,577918
473,02536	365	811,147444	2031,35531	629,178558	471,503129	679,234049	514,720997
474,997498	365	794,63799	1942,99591	619,963722	467,263965	663,110156	506,965123
476,968079	365	782,614068	1904,91462	611,059066	458,429288	658,102339	485,561769
478,937134	365	775,751413	1859,19508	604,333757	445,046806	645,70997	466,751173
480,904572	365	766,80834	1811,10373	597,29934	434,389193	643,444523	458,822025
482,870453	365	754,420733	1752,23695	587,294071	424,50526	644,797056	460,07209
484,834808	365	746,107057	1721,56576	578,20909	416,746108	639,747226	439,830534
486,797607	365	737,200915	1684,02847	587,236185	414,245932	624,334557	425,316731
488,75885	365	730,552795	1611,10081	568,153049	404,331759	616,040822	414,551383
490,718506	365	712,541445	1580,42752	571,276929	395,286752	616,737933	403,971924
492,676605	365	713,589234	1529,51927	550,981607	382,828339	605,671476	396,134898
494,633118	365	701,776044	1489,51992	548,20168	373,503847	598,304543	374,576066
496,588104	365	692,487308	1445,54523	540,481489	357,516028	581,934408	372,210415
498,541504	365	675,46717	1409,53345	522,226479	357,622134	575,409074	353,799374
500,493347	365	661,227602	1355,70725	516,956035	339,354098	567,816561	346,01172
502,443665	365	660,012955	1312,91259	504,2326	333,199089	562,545515	337,802182
504,392365	365	634,780102	1268,42994	496,883061	319,650818	549,832994	323,490396
506,339539	365	626,676861	1220,87398	491,086274	311,539714	538,960416	312,388837
508,285095	365	619,893366	1174,74703	481,603154	303,178881	526,915207	298,382824
510,229126	365	597,455847	1124,40988	467,6748	295,227476	519,590817	283,84325
512,17157	365	588,726931	1097,18161	457,943126	284,995926	503,506244	272,891597
514,112427	365	576,343062	1058,46928	449,684763	276,283386	495,831429	263,672847
516,051697	365	554,957785	1005,24421	431,054339	267,224469	479,018156	253,803585

Please do not adjust margins

Please do not adjust margins

517,989441	365	547,661576	972,927269	426,300677	256,798868	478,19081	247,105857
519,925598	365	531,93157	937,587116	412,236381	247,335609	461,115497	235,472129
521,860168	365	513,04852	907,142061	400,298908	238,04026	448,310155	224,597151
523,793152	365	515,964963	863,265576	396,368701	228,510639	436,549265	220,642496
525,724609	365	495,512483	830,909133	379,426985	221,567906	434,10099	209,435191
527,654419	365	486,49312	813,791859	387,223614	215,547384	425,947986	202,828462
529,582764	365	475,353901	776,752953	376,360133	202,794174	417,262208	189,691552
531,50946	365	468,592171	761,005361	360,039699	208,437271	401,00669	183,467599
533,43457	365	465,95138	724,464292	355,741362	193,836339	395,953496	179,204087
535,358093	365	456,990746	711,369447	356,719143	188,394591	382,951083	170,745958
537,280029	365	442,784125	680,995187	338,715682	185,199847	379,94158	167,715257
539,200439	365	441,860935	653,220124	336,064642	176,621663	372,627726	160,122558
541,119263	365	419,619205	622,28153	326,119568	169,039342	366,092423	155,355768
543,036438	365	417,760261	607,715339	315,945702	169,756743	354,917648	143,660778
544,952087	365	399,712306	583,38789	304,478873	160,992069	338,797838	145,247348
546,866211	365	388,727052	558,817374	299,451903	157,202752	340,516181	134,328511
548,778687	365	383,753922	545,11973	296,794334	148,719484	329,294868	131,93869
550,689514	365	379,361838	522,584742	289,759428	144,459312	318,583093	127,88183
552,598816	365	366,097091	506,749716	286,148888	137,946241	309,978207	119,227701
554,506531	365	355,05287	476,020629	272,04847	134,695984	303,704844	116,485002
556,41272	365	345,69766	466,967512	265,372034	132,213202	301,781067	112,697158
558,317261	365	342,759599	450,040599	257,774372	127,796299	285,815918	106,171917
560,220215	365	333,801659	429,81019	257,405004	123,803773	274,471316	102,596927
562,121582	365	324,793925	410,014104	247,846619	114,783846	266,490446	99,5338093
564,021362	365	312,920397	396,604717	240,744519	112,368411	264,246468	92,441945
565,919556	365	305,908967	381,500957	234,060836	112,739448	258,07462	90,7439557
567,816162	365	299,530933	363,850894	226,653051	107,77682	257,765054	89,3634292
569,711182	365	291,059583	355,93366	221,519719	103,070589	244,082696	86,0192225
571,604614	365	285,608261	342,022235	218,849141	99,2793515	243,677895	81,4163543
573,49646	365	279,332643	330,648159	208,459311	97,4793486	232,42146	79,4036801
575,386719	365	270,239269	319,53046	204,036291	93,1475999	225,120774	74,9503541
577,27533	365	261,330883	300,524178	202,957746	89,436925	216,808389	72,5836458
579,162354	365	250,762206	295,748305	194,599497	86,4866617	213,641471	65,0383636
581,047852	365	239,464259	281,84092	190,219518	85,4816793	204,229342	65,7285082
582,931641	365	238,954855	274,791383	186,400346	84,7171587	203,719737	62,8488185
584,813965	365	228,746421	257,785229	175,476847	81,2720812	195,037938	61,5670015
586,69458	365	223,70348	249,416787	171,136545	78,4391554	187,071494	56,5555586
588,57373	365	220,480652	244,098158	173,910957	72,833452	185,204863	54,9651047
590,451111	365	211,11104	233,650513	164,311346	69,4169785	179,070199	51,4868639
592,326965	365	203,610657	220,011175	154,736554	67,1121688	167,797122	50,3050954
594,201233	365	198,464345	210,417433	149,533645	63,8005698	167,735412	48,8874441
596,073914	365	194,565024	208,299658	140,751688	62,9294528	158,534802	48,4444661
597,944946	365	190,207797	196,110088	143,289494	60,7382855	154,298246	43,0819233
599,814453	365	181,679763	192,008486	139,315878	57,8438933	155,529972	42,1837602
601,682251	365	176,293385	182,796925	134,832307	57,5306556	146,307328	41,9961916
603,548523	365	178,389287	176,577864	132,072871	54,139487	140,249286	40,409487
605,413147	365	167,766701	168,169578	126,828574	52,1079446	137,011805	38,8468421

 This journal is © The Royal Society of Chemistry 20xx

Please do not adjust margins

Please do not adjust margins

607,276245	365	165,045565	162,457505	123,557742	51,2363834	130,989511	38,6122535
609,137695	365	161,444501	165,716625	124,68043	49,1341671	130,695511	36,1858544
610,997437	365	152,072456	150,021385	117,273337	45,5325026	128,134316	37,3987914
612,855713	365	146,310679	149,283377	113,091621	45,9014992	121,304706	32,9334634
614,712341	365	146,705411	144,210884	108,984407	43,1101045	115,09072	32,1417195
616,567322	365	139,25428	131,98891	104,065908	44,1291516	116,364593	31,2946699
618,420715	365	140,965396	131,651895	103,776666	40,2086413	113,256848	30,5461608
620,272522	365	137,091989	126,253519	101,160226	40,3828893	107,281045	27,6915534
622,122681	365	126,709487	122,613198	101,378101	40,2030301	104,6641	26,6872508
623,971313	365	120,364434	117,153537	95,8645114	37,7266118	101,827047	26,8492012
625,818237	365	121,914609	115,020318	93,3807782	36,5324668	98,2868763	25,0808439
627,663574	365	115,757748	110,739135	87,6311636	34,8924263	96,30556	24,4990431
629,507324	365	111,368184	106,86923	87,7926503	34,7938666	91,9093391	24,0805927
631,349365	365	108,652727	102,529401	85,0639421	33,5566266	87,9152838	22,9293913
633,18988	365	101,036963	96,1706498	79,7530278	31,2841543	89,1157818	21,6309977
635,028809	365	99,8916769	95,4807506	81,600876	30,9039748	86,1690304	20,4470699
636,865967	365	103,5822	92,1044673	75,4926979	28,1475568	79,4236751	18,7655216
638,70166	365	95,5763182	86,3614694	75,805588	29,2497103	76,2959014	19,3174506
640,535645	365	97,2797273	82,2727015	69,2797318	27,6146226	76,8254646	17,5088649
642,368042	365	91,6837473	79,7439388	69,1821291	28,8263416	71,112925	16,6263616
644,198853	365	89,8821474	76,3742494	68,786897	26,3448879	70,7209401	15,213342
646,028015	365	85,7098504	76,8263773	65,9592669	22,8283628	69,9837893	17,225492
647,855591	365	84,5807893	73,9753454	61,480419	24,1819674	67,5878885	15,0430214
649,681519	365	80,096345	66,6054753	61,6270998	22,8491375	65,8464505	16,216861
651,505798	365	76,2020694	64,2185543	58,946634	25,1455098	61,89276	14,0879432
653,328491	365	73,5820848	64,508529	58,9444207	19,9758953	61,7150936	14,0396066
655,149536	365	72,7401655	60,9561178	56,8511992	19,4017122	58,8884692	13,0775412
656,968994	365	69,8852958	61,6717522	54,3059996	21,4300913	57,1824585	11,4670239
658,786743	365	70,5935458	58,6129491	51,5867594	19,6685698	53,8190726	10,4069948
660,602905	365	66,1806271	56,2299792	49,6556439	18,0333387	53,0117278	12,1081885
662,41748	365	63,0037947	52,6357554	49,8787068	17,5512076	53,4376971	12,7239307
664,230469	365	62,5636308	52,551597	45,6557975	15,5169176	51,284731	11,0616651
666,041748	365	58,059436	51,2347724	46,8684734	18,2774262	50,3413244	12,3966469
667,85144	365	55,4782073	49,8645669	47,1860569	16,7216371	43,1062696	11,5831753
669,659424	365	54,4546934	48,0734174	43,0267194	15,9036923	43,6676356	10,380024
671,46582	365	53,1831624	45,3754162	41,3668164	16,0147149	41,7210522	10,0127958
673,27063	365	51,7736264	42,5099917	40,7686511	14,4602061	41,6941453	9,10372474
675,073792	365	47,4869792	40,2097584	40,0854406	13,6302069	39,1992992	8,17112495
676,875305	365	50,2427584	36,3915065	35,2228059	15,9147764	40,5268706	11,114423
678,675171	365	47,8669317	41,6377373	37,1326415	13,820415	39,8657037	6,36568106
680,47345	365	44,2097676	39,4161683	35,0112354	11,1431332	37,8339807	7,02538567
682,27002	365	39,391256	36,1133872	34,5597465	13,0931574	36,6262678	7,24026404
684,065063	365	43,6470736	37,4795444	34,4830443	12,5613478	34,9249986	8,9848284
685,858398	365	44,050085	34,6190446	31,8149697	11,7034824	33,2124906	7,74563956
687,650024	365	39,7709178	32,7335767	31,4628805	11,9071594	30,843353	6,22181119
689,440186	365	38,6111146	29,345507	29,1319771	9,91348607	32,2629484	5,94836836
691,228577	365	35,6437412	30,226302	30,3006173	10,0146576	30,0626618	5,66332869

This journal is © The Royal Society of Chemistry 20xx

Please do not adjust margins

693,015381	365	35,7338226	30,2061866	26,3269025	10,1365055	30,0420401	4,90779177
694,800476	365	35,4352358	29,6197541	25,6454465	10,6165438	26,4612956	4,23311569
696,583984	365	35,2874258	27,3175354	27,1041624	9,39964689	24,9197108	4,25020102
698,365845	365	32,8589669	24,3828652	26,2095965	8,94017958	24,8924963	6,78068305
700,146118	365	31,4285387	25,4555588	25,534595	8,5856306	24,7966672	4,57092794
701,924683	365	31,3534049	23,6932103	24,3609065	8,03100312	24,7939964	6,541054
347,741211	370	0	0	0	0	0	0
349,808533	370	0	0	0	0	0	0
351,874329	370	0	0	0	0	0	0
353,938568	370	0	0	0	0	0	0
356,001343	370	0	0	0	0	0	0
358,062561	370	0	0	0	0	0	0
360,122253	370	0	0	0	0	0	0
362,180481	370	0	0	0	0	0	0
364,237152	370	0	0	0	0	0	0
366,292328	370	0	0	0	0	0	0
368,345978	370	0	0	0	0	0	0
370,398132	370	0	0	0	0	0	0
372,44873	370	0	0	0	0	0	0
374,497803	370	0	0	0	0	0	0
376,545349	370	0	0	0	0	0	0
378,5914	370	0	0	0	0	0	0
380,635925	370	62,3283161	262,578175	61,9135474	206,608534	68,5355481	66,6802536
382,678955	370	77,8231249	310,446877	65,1916864	185,037016	77,412854	75,9617709
384,720398	370	89,8723305	357,77822	71,3613211	181,269191	91,9483214	90,7365001
386,760376	370	100,154455	425,686296	88,3425128	178,820712	101,220791	98,8959028
388,798767	370	125,76052	491,596822	96,4369593	190,143031	113,897972	121,698233
390,835663	370	139,626553	560,760625	104,504636	190,884756	132,717492	135,427993
392,871033	370	160,058446	642,247398	128,609933	203,834812	159,668666	157,333082
394,904877	370	186,211723	722,384334	145,1028	225,212648	177,964829	174,801386
396,937195	370	212,784203	823,770399	158,306424	235,297148	194,883022	206,488188
398,967957	370	231,749595	923,032861	183,012919	255,02007	213,56476	225,665654
400,997253	370	269,749169	1022,38839	191,855572	274,341734	236,719117	240,814799
403,024963	370	291,914402	1115,63044	222,351098	296,509622	270,446668	264,55548
405,051147	370	320,928166	1243,52282	248,975789	313,845326	295,533978	288,721432
407,075806	370	357,525002	1355,66717	271,300284	331,980612	313,636884	303,925173
409,098938	370	381,902288	1466,53231	299,287333	352,034383	345,718184	343,569171
411,120544	370	416,843634	1574,40719	322,971044	384,17159	375,394417	362,134556
413,140564	370	456,750227	1673,4879	346,369986	400,864392	408,251094	391,128326
415,159119	370	502,076181	1767,59658	375,983994	416,178604	441,05713	414,741138
417,176086	370	540,941512	1873,72538	400,606697	444,73133	458,585113	446,708169
419,191528	370	568,982167	1956,54528	434,505823	472,320154	497,398735	453,739074
421,205444	370	616,166034	2020,89756	463,669143	485,821457	525,872154	482,707475
423,217834	370	639,01047	2110,1195	487,973829	497,621183	546,250186	500,28318
425,228668	370	657,107334	2166,42982	499,883514	501,817097	558,765956	506,816781
427,237976	370	694,679328	2212,09652	521,662932	504,603002	580,329119	517,249029
429,245728	370	711,548891	2234,29883	547,207728	503,776977	589,26734	522,016941

 This journal is © The Royal Society of Chemistry 20xx

Please do not adjust margins

431,251953	370	735,143877	2312,72932	562,307126	515,738768	615,660566	542,405253
433,256592	370	759,750857	2338,73732	582,349651	523,908153	624,423584	552,551728
435,259766	370	800,449292	2391,74235	589,105968	527,353606	644,167782	557,737458
437,261353	370	816,838948	2415,9715	608,007612	533,737027	659,270046	570,219765
439,261383	370	834,412793	2456,68803	635,122275	532,871621	662,461165	581,221906
441,259888	370	835,319078	2456,09946	653,333016	538,394127	678,538011	583,10052
443,256836	370	849,074145	2462,04273	644,637943	535,506807	685,673476	593,628928
445,252258	370	868,475846	2448,03777	646,87767	541,583843	691,44027	587,750535
447,246094	370	858,625984	2416,43188	660,004938	543,189625	691,854487	585,763843
449,238434	370	867,236764	2408,49111	656,142262	533,000738	702,092855	580,611551
451,229187	370	864,543515	2390,22143	656,541144	528,983932	708,922827	575,08019
453,218445	370	877,447364	2366,28689	663,597633	530,91112	709,800319	573,224765
455,206116	370	867,339897	2334,22268	670,557387	526,236003	701,351121	576,525967
457,192261	370	870,673773	2316,31092	663,139569	515,055253	705,416298	575,660736
459,176819	370	861,483258	2281,61639	658,779964	521,61555	695,192685	568,762808
461,15979	370	857,897077	2252,74786	662,655862	516,675491	704,263899	554,199266
463,141296	370	861,278755	2198,73442	644,776548	508,624372	700,043803	562,885743
465,121216	370	839,767529	2154,87803	641,803784	494,586274	699,803453	544,81526
467,099579	370	829,978046	2122,03294	642,83498	487,662487	680,766616	524,522193
469,076385	370	829,207935	2061,95231	632,546721	482,774697	685,615544	515,34436
471,051636	370	814,457512	2020,48595	630,040511	475,188218	680,56281	501,929874
473,02536	370	810,289293	1977,18279	626,398513	467,757022	678,686813	502,227373
474,997498	370	800,733217	1929,81988	616,169121	455,875418	668,889636	488,665922
476,968079	370	794,63931	1876,07501	616,690255	459,287739	666,182044	480,609352
478,937134	370	793,219954	1834,11338	604,191523	447,919212	659,591023	464,343176
480,904572	370	795,531286	1797,04901	596,897121	436,999635	650,919353	457,468266
482,870453	370	769,128624	1758,27698	601,921675	428,571821	648,74501	443,150336
484,834808	370	772,789205	1697,74887	586,557071	412,736359	637,622941	434,271433
486,797607	370	757,319247	1654,26584	584,603901	409,371702	637,457969	427,153647
488,75885	370	744,618544	1615,1879	576,354443	398,939797	638,536985	408,987091
490,718506	370	737,523571	1578,38148	574,558307	393,133623	632,822153	398,195383
492,676605	370	735,951158	1532,02542	562,808043	385,015914	609,294263	389,453797
494,633118	370	716,223776	1486,2197	560,963217	373,193299	611,600999	379,591561
496,588104	370	705,745677	1432,23218	548,873381	358,580251	605,970754	371,849943
498,541504	370	696,423958	1405,0589	544,697564	355,304441	597,569836	351,976922
500,493347	370	688,799553	1352,08375	532,159464	348,744207	582,492495	337,629822
502,443665	370	673,831517	1320,64595	515,837311	327,625816	566,517769	338,256649
504,392365	370	660,425345	1283,76491	516,737468	328,837364	560,811561	320,106697
506,339539	370	647,363546	1242,36811	508,076028	312,014696	557,652661	306,997845
508,285095	370	626,223463	1176,80967	495,043734	302,002482	536,292075	298,745814
510,229126	370	618,843967	1151,61073	477,048996	297,650574	530,130917	287,171086
512,17157	370	610,304467	1114,25343	471,451561	286,359234	517,990284	280,065256
514,112427	370	593,176611	1071,89561	456,760717	276,451045	512,436798	268,380673
516,051697	370	580,512731	1021,81103	444,969924	264,375901	503,10901	255,574649
517,989441	370	564,961424	988,318333	433,239283	259,557269	487,447444	242,794036
519,925598	370	553,055053	951,875574	430,385789	250,324235	467,473871	233,873525
521,860168	370	543,327184	914,492941	418,880132	236,277031	465,348707	231,544749

Please do not adjust margins

Please do not adjust margins

523,793152	370	519,525454	883,864507	406,096474	235,412805	451,755823	215,360899
525,724609	370	519,398548	839,862124	398,234708	224,123379	442,627449	204,082456
527,654419	370	509,247822	814,671936	388,90685	221,915778	436,404252	201,597939
529,582764	370	499,665822	789,907323	383,248485	208,313421	423,115445	191,031865
531,50946	370	498,930492	774,630329	379,717761	208,465041	411,584839	189,598854
533,43457	370	479,616403	739,639362	372,213538	201,318355	407,669439	181,539357
535,358093	370	464,71473	701,147136	353,021841	193,268545	401,013941	172,194564
537,280029	370	459,439073	681,302014	349,437555	185,557399	391,375162	165,728571
539,200439	370	451,246849	662,036608	346,800349	180,192346	376,852724	163,747038
541,119263	370	439,708524	637,166189	333,504468	169,585716	372,279372	155,179315
543,036438	370	430,659143	624,694134	325,657536	167,530191	361,599847	153,530746
544,952087	370	417,821507	592,888161	319,851735	161,537836	359,350887	142,869652
546,866211	370	402,586287	564,131741	311,12976	157,250752	341,271123	137,720034
548,778687	370	401,015569	545,319034	301,852056	151,688559	336,330374	130,897196
550,689514	370	391,524111	527,406238	291,413769	150,642936	327,358537	125,488754
552,598816	370	376,872818	510,812943	291,061623	136,485329	320,10471	123,808559
554,506531	370	367,087856	486,555896	285,49499	140,267919	310,599831	114,266781
556,41272	370	361,541184	463,58743	273,360891	133,52112	306,481231	113,356222
558,317261	370	345,664961	448,690466	263,520146	133,925395	293,352864	108,493388
560,220215	370	339,378295	428,875114	266,4437	126,179826	282,742026	108,350482
562,121582	370	333,473543	420,6674	254,706805	119,135484	275,287926	95,698735
564,021362	370	323,066252	402,084602	243,91049	112,364075	268,36828	97,9689718
565,919556	370	323,475133	390,991423	241,118756	113,707162	263,591848	93,1285312
567,816162	370	308,129749	377,887747	232,767743	108,945943	252,79284	88,4850695
569,711182	370	295,321146	356,866479	225,42212	104,843338	245,99825	83,5202262
571,604614	370	294,768811	346,217554	221,703309	98,2177724	242,206691	82,1887007
573,49646	370	290,55084	337,485134	210,570266	98,0155845	235,468163	79,1689549
575,386719	370	280,293203	322,361494	207,817903	95,7571406	228,763337	73,9207066
577,27533	370	272,477536	306,852068	202,184401	91,2468212	221,672543	74,9816203
579,162354	370	262,006554	301,630868	200,301486	86,635631	220,177632	70,2565503
581,047852	370	258,41703	285,727155	198,878727	85,3290934	216,341072	66,4874381
582,931641	370	247,424764	280,735583	190,65149	82,4213111	202,872261	64,2520418
584,813965	370	240,296855	262,855804	182,476814	81,3007304	202,811107	62,6742213
586,69458	370	233,394967	248,748974	178,091932	72,6041804	185,943769	56,2841241
588,57373	370	224,082212	240,870834	174,39457	73,1483981	187,663748	56,4084189
590,451111	370	221,176235	234,301872	163,08305	73,6384417	180,536688	53,2013593
592,326965	370	218,021587	225,290764	160,24507	67,1645625	175,487815	51,2239121
594,201233	370	207,942763	215,63118	149,348775	64,8037662	164,284875	47,4447265
596,073914	370	204,744348	202,5568	151,218501	60,9234101	165,828093	48,3101878
597,944946	370	194,971934	197,584323	147,42872	61,3900729	156,541542	44,5403815
599,814453	370	189,031313	191,555522	139,876748	58,7286763	152,871278	40,7173792
601,682251	370	181,733204	185,384283	139,201071	54,4362544	146,622137	38,8965871
603,548523	370	177,448288	172,187287	138,557039	53,8013696	147,25833	41,2228642
605,413147	370	169,283718	169,564177	132,802958	52,1583038	140,538129	37,0290628
607,276245	370	161,088334	159,651361	128,726287	51,2181716	134,122214	37,2300998
609,137695	370	167,295903	154,236072	126,546509	48,8170745	135,97538	37,4284822
610,997437	370	159,081839	152,730475	114,737602	48,741516	130,384668	32,5251402

This journal is © The Royal Society of Chemistry 20xx

Please do not adjust margins

Please do not adjust margins

612,855713	370	156,635689	145,280523	116,33047	45,4856801	121,03553	34,1681298
614,712341	370	151,257337	139,184688	115,521077	44,9173597	124,128159	33,313419
616,567322	370	143,185607	133,878453	112,012211	46,003226	112,579405	28,9543153
618,420715	370	141,486642	131,213535	106,459886	41,2213415	116,731418	29,0832164
620,272522	370	134,480641	125,148211	104,863293	40,1808025	105,950362	28,4531844
622,122681	370	131,719207	119,495245	101,174399	38,8758142	108,963564	27,907775
623,971313	370	127,272068	116,940292	97,874794	39,1566384	103,76381	27,2019954
625,818237	370	124,966359	112,930398	95,0209003	34,9336009	97,4272048	24,6046353
627,663574	370	120,438802	112,777576	91,5032342	33,065458	97,9495361	24,2108661
629,507324	370	115,731338	103,976894	91,6894348	35,1673714	92,1697267	23,6274394
631,349365	370	111,484562	102,198583	87,8749845	32,370771	89,5014211	23,293257
633,18988	370	108,909182	96,416722	82,06668	28,3795155	91,3913858	24,3167983
635,028809	370	103,707322	90,5190591	82,9897383	30,9248715	83,9094874	22,1890018
636,865967	370	103,695311	87,118421	76,659993	29,469731	82,8920318	19,7550131
638,70166	370	95,0545277	85,9248901	75,5507266	25,5013492	78,0641393	20,9130021
640,535645	370	98,2315924	84,3132736	73,3229918	28,4181583	75,9235865	15,8803755
642,368042	370	88,8437129	81,3081435	70,6628742	24,2864812	74,4185135	15,899516
644,198853	370	91,9735543	78,9927958	69,2159817	25,4936417	73,6012711	16,376424
646,028015	370	88,4253947	74,0969842	66,9833074	24,1002728	66,6560359	16,6612616
647,855591	370	84,4661351	69,8804694	64,3752797	22,0451622	68,7616215	15,0594082
649,681519	370	82,2312276	67,8383333	62,9322166	22,0738648	68,2355915	15,140064
651,505798	370	77,4421363	66,4994057	59,151514	24,8565743	63,7565564	15,9176472
653,328491	370	77,1395186	66,2536821	57,2630139	22,0528248	60,7103918	13,2756937
655,149536	370	75,970855	64,3230346	56,3866015	21,5005388	61,1116597	14,6742987
656,968994	370	69,2837368	61,5910909	55,258603	20,5008611	59,3671786	10,6670182
658,786743	370	66,601214	60,4260879	53,5178916	20,7814955	58,1925818	11,899004
660,602905	370	68,8653821	54,256239	52,7309033	19,3689605	54,0334791	10,6525524
662,41748	370	61,7237364	53,489008	47,2762689	19,0888759	55,5619389	12,8039044
664,230469	370	63,8732529	52,8578584	50,1289466	17,5633154	50,5185031	12,0960738
666,041748	370	60,3886539	49,2641199	46,3502267	15,6607968	48,5825768	10,2820644
667,85144	370	59,2583824	49,1220192	46,2004387	16,8123545	44,9260687	10,3103294
669,659424	370	56,4100587	46,6265013	42,5909259	16,2911962	45,5732924	9,68560983
671,46582	370	56,281987	44,6327601	40,3173827	16,1225368	41,8182321	9,41849838
673,27063	370	54,5445109	42,5719219	40,5684068	14,6043479	42,7267836	8,89920308
675,073792	370	49,4181375	42,560568	40,0871733	11,2772551	43,368658	5,38695557
676,875305	370	48,5681659	38,819824	38,7746573	14,816063	39,0710167	10,9575905
678,675171	370	48,295639	38,0793396	38,50115	14,72522	36,7456492	6,77992566
680,47345	370	44,9917401	36,2783248	35,7703552	13,5033364	34,3878044	6,49633616
682,27002	370	43,367971	35,0938691	35,5204114	11,587697	36,938354	7,72949114
684,065063	370	44,0759901	36,3372512	33,66898	12,0950113	35,3722686	5,88646386
685,858398	370	42,4029535	32,7836167	33,6837388	11,0685035	31,1637692	8,50639717
687,650024	370	37,7394133	30,7475415	32,0266437	14,3657907	31,4766068	5,52061527
689,440186	370	39,335939	30,0646409	31,3475769	11,3874418	31,9260724	6,47123626
691,228577	370	38,4115177	27,9106943	30,892082	10,7339419	30,4342014	6,75591618
693,015381	370	34,8185813	27,5085762	26,4379369	9,43953466	29,1886536	3,85684165
694,800476	370	34,4315161	29,5808719	27,5641047	9,15406158	29,9368675	5,54870291
696,583984	370	36,5649366	27,5178161	27,296242	9,6647717	29,7732383	4,81569496

This journal is © The Royal Society of Chemistry 20xx

Please do not adjust margins

698,365845	370	34,1914213	23,884068	23,9513239	9,02679633	24,2468439	7,00711304
700,146118	370	34,9017081	23,3107107	25,955476	8,87205241	25,2028743	3,61261859
701,924683	370	33,1161109	24,7429689	22,415187	8,4273346	25,1081002	5,15345741
347,741211	375	0	0	0	0	0	0
349,808533	375	0	0	0	0	0	0
351,874329	375	0	0	0	0	0	0
353,938568	375	0	0	0	0	0	0
356,001343	375	0	0	0	0	0	0
358,062561	375	0	0	0	0	0	0
360,122253	375	0	0	0	0	0	0
362,180481	375	0	0	0	0	0	0
364,237152	375	0	0	0	0	0	0
366,292328	375	0	0	0	0	0	0
368,345978	375	0	0	0	0	0	0
370,398132	375	0	0	0	0	0	0
372,44873	375	0	0	0	0	0	0
374,497803	375	0	0	0	0	0	0
376,545349	375	0	0	0	0	0	0
378,5914	375	0	0	0	0	0	0
380,635925	375	0	0	0	0	0	0
382,678955	375	0	0	0	0	0	0
384,720398	375	0	0	0	0	0	0
386,760376	375	80,9305797	308,844484	70,8423736	197,570091	74,2510921	80,6815087
388,798767	375	94,422333	366,526411	77,2432081	184,739619	86,6411165	91,5924745
390,835663	375	107,277016	420,774567	82,9730681	181,397407	102,708659	98,7776408
392,871033	375	120,269914	491,015497	97,7697348	180,506875	118,002842	120,134375
394,904877	375	143,1174	573,32207	107,394975	196,869569	134,88797	135,564116
396,937195	375	169,010486	663,301495	127,453583	206,31386	155,640729	153,129493
398,967957	375	188,459754	735,066733	141,014663	222,572333	169,430052	173,890848
400,997253	375	212,40203	839,332985	160,119853	237,852003	196,49974	194,990574
403,024963	375	236,440899	953,370887	176,796981	251,468294	215,124638	213,084101
405,051147	375	275,493481	1046,57232	202,35674	271,619402	234,222737	239,399955
407,075806	375	301,070131	1158,5939	225,522765	285,777722	263,581885	260,104537
409,098938	375	331,197824	1276,62801	246,738102	305,884147	293,775754	288,241316
411,120544	375	364,128144	1358,1243	266,196784	319,941432	319,674781	296,430034
413,140564	375	408,89056	1455,49887	286,957603	353,147779	347,076882	327,691177
415,159119	375	432,373689	1551,42525	313,230206	363,540968	378,528852	340,299695
417,176086	375	464,086882	1642,91559	351,630896	383,840686	396,802035	365,985881
419,191528	375	512,881626	1730,51704	376,096588	397,147917	418,593672	386,532824
421,205444	375	536,862888	1806,14062	404,410394	428,53283	451,756607	409,027307
423,217834	375	578,29209	1887,33035	431,37195	449,580009	484,490583	435,119822
425,228668	375	621,496978	1971,02645	457,134949	471,560115	506,406046	455,231728
427,237976	375	645,949566	2008,36954	480,241092	476,536187	524,645966	472,910186
429,245728	375	665,913535	2056,66322	501,619134	483,404861	549,108423	480,937892
431,251953	375	705,034604	2133,88346	514,470608	493,622646	566,485104	493,200699
433,256592	375	729,122344	2160,53414	532,317843	489,563848	584,204343	507,232828
435,259766	375	750,834926	2207,30519	559,358161	504,599474	600,200667	515,229406

 This journal is © The Royal Society of Chemistry 20xx

Please do not adjust margins

437,261353	375	779,391371	2238,77977	578,837449	507,267325	624,440867	524,847203
439,261383	375	784,747639	2277,66144	582,655482	506,206908	633,295878	528,752088
441,259888	375	814,259806	2283,86187	597,559924	507,800961	639,078214	532,595618
443,256836	375	821,125039	2285,13998	603,677971	501,289469	658,960973	540,271286
445,252258	375	821,211081	2293,21505	607,521527	503,907204	664,893645	547,219106
447,246094	375	833,654672	2291,30758	627,313182	500,083603	664,816135	547,964735
449,238434	375	824,532021	2248,81087	630,041335	497,588482	661,47389	543,418981
451,229187	375	844,177687	2269,68683	617,409634	500,739792	682,399517	541,661716
453,218445	375	852,087229	2242,39629	625,85625	512,013258	680,861234	533,206531
455,206116	375	852,500834	2234,94912	637,63485	494,881303	677,872541	537,065376
457,192261	375	854,130071	2192,4737	630,051663	497,686862	684,120264	534,789787
459,176819	375	852,140309	2175,12417	642,663061	484,571433	691,897119	527,478993
461,15979	375	842,163908	2143,45004	627,561837	494,28069	689,563914	520,564703
463,141296	375	845,203998	2104,77176	630,102525	476,742314	685,335163	529,304897
465,121216	375	833,058163	2065,63956	625,796548	473,543458	687,187316	513,195875
467,099579	375	832,574838	2033,3028	631,414818	473,567687	685,15433	503,219717
469,076385	375	824,037998	1989,63618	613,445031	466,872415	668,067654	496,34891
471,051636	375	828,391545	1954,99151	624,181739	457,088395	672,527057	498,741914
473,02536	375	824,830964	1924,61623	606,577149	454,68556	671,309396	472,61789
474,997498	375	812,531808	1891,00261	609,0432	448,963755	671,99413	476,140465
476,968079	375	799,974956	1823,90229	605,28901	444,182916	668,259279	459,25149
478,937134	375	795,07949	1790,22432	600,263692	432,642226	662,637381	449,370498
480,904572	375	796,044068	1756,55849	601,630175	422,396745	646,58945	434,91605
482,870453	375	785,044647	1713,27426	593,106499	416,363805	645,947285	437,458635
484,834808	375	786,715417	1675,38441	595,051001	412,625124	650,630019	425,375565
486,797607	375	765,560703	1637,53951	587,408886	402,935284	642,684447	412,576794
488,75885	375	753,152525	1603,51982	584,081885	399,811923	644,713741	402,572536
490,718506	375	759,551172	1554,186	570,362221	388,398827	628,544936	389,610538
492,676605	375	735,828074	1525,55623	556,359046	375,789891	627,979432	384,867238
494,633118	375	723,988158	1468,65618	556,792847	373,941768	619,668546	376,734401
496,588104	375	736,708562	1425,76813	552,087726	361,989098	610,063787	362,29196
498,541504	375	711,085572	1387,43851	541,121089	358,468289	597,178003	354,392043
500,493347	375	691,817508	1344,64937	532,543297	341,694038	587,879133	338,538037
502,443665	375	688,915314	1311,31731	532,828723	332,300566	578,087471	331,474604
504,392365	375	675,771471	1257,63917	516,661943	329,557364	565,809083	318,823188
506,339539	375	656,381359	1221,92713	497,552503	306,588126	563,8542	307,429499
508,285095	375	651,730768	1188,89002	500,280509	310,17406	551,43884	301,548926
510,229126	375	631,316169	1144,05194	482,667923	289,828125	541,762611	280,332818
512,17157	375	629,698987	1100,04989	478,140779	287,873704	524,328253	273,772205
514,112427	375	616,582382	1065,37552	467,595792	278,523087	517,712963	265,511554
516,051697	375	598,859976	1024,71064	455,463741	263,91737	507,62165	256,018878
517,989441	375	587,446817	982,607566	445,983507	256,482491	501,715028	241,821923
519,925598	375	573,240614	943,593109	435,681216	251,771134	487,198878	232,795655
521,860168	375	554,490156	916,348438	428,178415	238,998296	466,309864	226,886668
523,793152	375	539,824269	876,74647	415,632982	237,235016	459,482347	214,504539
525,724609	375	531,858506	845,671005	404,686826	224,067548	443,22313	207,236878
527,654419	375	519,44545	820,834055	404,593056	218,075879	446,328169	204,572804

This journal is © The Royal Society of Chemistry 20xx

Please do not adjust margins

529,582764	375	517,907746	798,386613	395,464154	211,683574	435,702906	194,600903
531,50946	375	506,149482	765,38899	385,237102	207,362084	422,833224	189,933754
533,43457	375	490,109259	749,478156	376,059065	200,114685	414,935944	183,469511
535,358093	375	483,764928	721,730209	370,648123	189,966276	409,560879	178,428487
537,280029	375	472,787303	688,539041	354,995869	185,826269	401,552142	170,529698
539,200439	375	460,054636	670,636135	360,824422	185,843464	390,700369	161,908324
541,119263	375	456,000077	636,977912	339,74574	179,697415	377,689506	157,062165
543,036438	375	440,484527	621,312339	336,510729	166,380326	371,27755	151,792902
544,952087	375	426,839468	591,568606	325,827908	163,661669	361,146865	142,030917
546,866211	375	416,544898	575,785411	320,450986	162,034307	348,656557	141,135696
548,778687	375	414,762001	541,044123	314,189136	152,947151	341,834323	131,839482
550,689514	375	396,866423	517,222833	300,143055	145,063042	334,509727	126,994545
552,598816	375	392,293937	511,4813	299,053877	144,531352	322,327912	124,943257
554,506531	375	383,537136	488,063372	293,038942	139,287681	314,726956	117,138209
556,41272	375	368,242088	480,552627	276,667071	133,453496	309,353881	116,011096
558,317261	375	362,419149	454,524576	277,61781	127,801869	301,503357	107,632711
560,220215	375	347,57212	437,83335	271,062587	124,52187	292,823917	106,160646
562,121582	375	334,372057	415,855848	263,1915	121,783275	282,233165	104,02102
564,021362	375	333,487842	394,700592	251,751193	111,834247	276,482874	94,6295559
565,919556	375	318,468005	390,952192	245,747693	111,813484	264,886462	94,2123465
567,816162	375	312,399912	372,624062	240,60217	108,25414	265,435513	87,997556
569,711182	375	305,765537	360,06046	232,960814	104,153254	253,325187	81,4571128
571,604614	375	297,3393	351,337068	228,886688	98,7782684	248,056925	80,3067634
573,49646	375	292,008936	332,26656	221,452673	96,9009962	241,475932	77,5004497
575,386719	375	279,686529	325,330075	215,174192	93,801308	234,444398	73,3097333
577,27533	375	276,418154	310,643861	207,687414	88,7270059	225,678552	72,9022472
579,162354	375	270,311778	298,309576	209,683944	87,8536435	224,967289	71,7421078
581,047852	375	261,518909	285,976822	200,436184	82,1807074	213,000252	66,4261823
582,931641	375	256,52815	272,91179	190,57383	82,7516276	203,374586	63,3367614
584,813965	375	244,14464	266,737279	186,978451	81,738617	200,953856	64,55953
586,69458	375	236,564371	257,84547	180,488724	76,7362116	198,437742	57,9667077
588,57373	375	228,526825	239,53671	172,773052	72,2718071	181,077946	54,733276
590,451111	375	225,913772	232,701521	168,844005	71,5098588	184,457817	52,9878405
592,326965	375	218,774289	218,667421	164,225123	68,0913276	173,864598	52,8147497
594,201233	375	209,761386	217,998175	160,210766	64,3522023	171,116323	48,1107136
596,073914	375	203,058354	207,094689	155,911391	61,7457567	165,69543	48,8012937
597,944946	375	199,02114	196,993721	149,798984	62,2018973	164,777274	47,3106362
599,814453	375	190,176277	196,270428	143,99679	59,0749918	155,442929	42,9539008
601,682251	375	185,125842	179,39163	144,931244	54,0953179	149,043739	39,5498662
603,548523	375	184,388656	180,959127	141,062128	51,3523786	148,554545	39,8875932
605,413147	375	174,821188	171,162043	133,97204	53,2109957	141,530785	39,9043071
607,276245	375	174,216213	164,556377	127,941267	49,6669038	138,019309	36,5584171
609,137695	375	171,148935	161,341016	126,728211	48,9140995	139,996235	35,3234613
610,997437	375	160,632054	154,974039	120,811854	46,0534065	129,958002	35,5619528
612,855713	375	153,563847	148,764922	117,221734	45,9011171	126,960324	31,4441711
614,712341	375	148,873899	138,55399	115,504164	43,9059412	123,655933	33,7267366
616,567322	375	150,651731	136,067998	111,386432	44,3773127	119,883085	30,6320297

This journal is © The Royal Society of Chemistry 20xx

Please do not adjust margins

618,420715	375	139,189015	132,438047	106,452815	40,9585772	113,682374	29,744922
620,272522	375	135,553406	125,298946	107,104857	41,3862941	108,896777	29,9780148
622,122681	375	137,901023	121,209375	101,362969	39,1712397	106,654939	26,1276831
623,971313	375	133,812335	114,598379	101,298187	34,9141825	102,151809	27,5646277
625,818237	375	118,992388	108,769101	97,1457873	34,3647434	103,156895	24,3320862
627,663574	375	124,125096	109,305107	91,0352876	34,3390869	99,8936171	27,207147
629,507324	375	123,781624	106,291756	91,7430777	34,1622733	92,7701706	23,8911651
631,349365	375	113,44877	101,60208	88,4359374	31,0716333	91,6261006	23,0456408
633,18988	375	105,964443	95,8436762	88,468981	30,7104275	90,7115927	22,4938524
635,028809	375	110,686997	94,136305	80,0945082	31,4723476	85,8858486	20,232347
636,865967	375	104,849703	91,4160941	77,8780899	30,8278294	84,2849883	20,2646144
638,70166	375	98,977429	88,3333536	75,4885973	27,7168587	81,8100864	19,2359745
640,535645	375	97,9129847	81,6172093	75,38788	25,0187749	79,0135068	17,1532586
642,368042	375	93,7535996	78,8407267	72,9569891	25,1359053	73,8000138	16,9137222
644,198853	375	90,3690963	75,780543	70,9357712	25,1901341	72,9126388	15,7332739
646,028015	375	85,8318315	74,9942492	68,7495396	24,0848755	72,0365945	16,6217438
647,855591	375	87,2823465	72,3402427	70,4634476	24,9465393	66,5936339	17,1363527
649,681519	375	80,9727501	64,2954904	62,6871284	21,6601288	67,7400142	15,391631
651,505798	375	78,3889348	65,7061144	59,2471521	23,5982042	63,8772293	15,6304707
653,328491	375	78,3646222	61,4852514	62,0789519	22,4344029	62,3065661	14,9739925
655,149536	375	76,5139238	62,4188435	58,4048226	21,7180649	58,2752725	15,7355866
656,968994	375	69,4151586	64,5491068	54,9085199	21,3627698	56,3785539	15,0017152
658,786743	375	70,0174338	56,8857754	52,0422235	19,2292819	56,4700103	12,9330364
660,602905	375	65,5045503	56,5716713	52,5241434	18,9162726	53,2848142	10,4603656
662,41748	375	63,99115	52,4060177	50,2391321	19,3614615	51,9022352	11,9442589
664,230469	375	65,2041542	47,5386542	47,7221183	15,7904256	53,1783655	12,3277622
666,041748	375	62,8735243	52,4241628	46,7246853	17,2655714	47,8483717	11,3586319
667,85144	375	59,5892731	49,6629466	45,8562542	16,7691687	45,0775286	10,8474822
669,659424	375	57,4315439	49,3091593	46,4944565	13,9876154	46,441663	8,78623041
671,46582	375	57,0339938	46,4453578	42,2695858	15,2748188	41,5764005	9,61126483
673,27063	375	53,6846062	42,8788736	42,6177295	16,7038378	43,2919769	9,83098023
675,073792	375	51,1261236	40,6713896	39,408165	13,4436756	41,8196883	8,11705688
676,875305	375	51,9423699	38,1908878	38,303691	13,2582545	39,1584975	9,11855961
678,675171	375	50,849198	39,0213519	36,7554436	12,9860092	40,1712905	6,65760638
680,47345	375	46,4997567	37,2525066	37,0928349	12,2450493	36,208858	7,10817704
682,27002	375	44,5441262	34,5343509	34,9294165	11,7372964	36,7013463	7,77293293
684,065063	375	47,2776809	34,4648328	33,8497367	11,9603138	33,325118	7,70701851
685,858398	375	43,3260781	32,3595163	31,9320854	10,769001	32,8792326	7,34323512
687,650024	375	40,6874449	31,1919705	32,0559209	11,977443	30,7915201	6,15606868
689,440186	375	40,5034366	31,7265556	30,3757917	12,2821698	29,9386606	4,97307613
691,228577	375	39,5960535	31,0943637	29,5593771	10,8099306	29,3986961	5,6204977
693,015381	375	35,5126912	30,1524047	27,0410995	9,72573742	28,5461708	5,72707438
694,800476	375	36,5261823	28,0115438	27,1269286	10,2789258	29,2803144	4,98685113
696,583984	375	34,9938989	25,9826616	26,2138883	9,29766905	28,2820945	4,91425679
698,365845	375	33,5013804	26,0556702	25,2621458	7,55227356	26,3090826	6,6017479
700,146118	375	32,131936	24,0031988	24,8950897	8,14409926	24,7281505	5,50787037
701,924683	375	31,6895797	23,1542479	23,393641	8,45731936	26,2336262	5,52904502

This journal is © The Royal Society of Chemistry 20xx

Please do not adjust margins

Please do not adjust margins

347,741211	380	0	0	0	0	0	0
349,808533	380	0	0	0	0	0	0
351,874329	380	0	0	0	0	0	0
353,938568	380	0	0	0	0	0	0
356,001343	380	0	0	0	0	0	0
358,062561	380	0	0	0	0	0	0
360,122253	380	0	0	0	0	0	0
362,180481	380	0	0	0	0	0	0
364,237152	380	0	0	0	0	0	0
366,292328	380	0	0	0	0	0	0
368,345978	380	0	0	0	0	0	0
370,398132	380	0	0	0	0	0	0
372,44873	380	0	0	0	0	0	0
374,497803	380	0	0	0	0	0	0
376,545349	380	0	0	0	0	0	0
378,5914	380	0	0	0	0	0	0
380,635925	380	0	0	0	0	0	0
382,678955	380	0	0	0	0	0	0
384,720398	380	0	0	0	0	0	0
386,760376	380	0	0	0	0	0	0
388,798767	380	0	0	0	0	0	0
390,835663	380	80,7566521	301,898859	69,3233796	206,65678	76,2845819	79,4868039
392,871033	380	94,2754626	369,283331	77,0850483	195,775376	88,439891	86,9589258
394,904877	380	109,646585	439,884773	85,113965	184,761527	98,7269147	106,359177
396,937195	380	126,492586	516,235898	100,472913	184,546884	119,148263	117,971552
398,967957	380	146,576627	582,920503	103,771051	194,293938	134,533283	135,259797
400,997253	380	170,95163	669,549454	125,033308	208,114527	158,551083	150,100238
403,024963	380	189,057324	778,824391	143,54621	222,355311	174,610607	169,059251
405,051147	380	217,299639	870,898387	159,276993	234,733417	200,371107	192,320007
407,075806	380	247,150354	987,245448	181,468682	248,921521	219,799359	208,871524
409,098938	380	275,497686	1075,25372	204,643115	267,476271	242,218426	234,214443
411,120544	380	309,050365	1180,38707	222,698707	280,494716	274,443882	250,286736
413,140564	380	340,691298	1259,06745	246,187243	296,865855	286,013435	274,366797
415,159119	380	372,901692	1363,82198	270,674292	309,807354	319,055588	288,991902
417,176086	380	396,585709	1446,03579	304,2414	331,928792	330,406873	308,810217
419,191528	380	435,943719	1514,16134	321,116795	349,249563	359,191862	327,065171
421,205444	380	471,607089	1591,40841	341,00734	364,716417	392,755131	342,566543
423,217834	380	509,907125	1666,69662	364,757834	375,004776	415,110623	360,726334
425,228668	380	540,802518	1755,29062	395,482116	401,354873	443,411341	379,318762
427,237976	380	575,81734	1792,17371	413,126241	419,893083	464,164019	406,535243
429,245728	380	603,614554	1833,69168	432,650236	435,644181	499,70823	419,243829
431,251953	380	647,133125	1904,24862	461,857431	450,862238	517,557854	436,39204
433,256592	380	676,469145	1952,77588	480,245831	462,642808	534,860351	455,098395
435,259766	380	701,040468	2033,11863	509,550657	473,826221	557,936385	470,088628
437,261353	380	726,891036	2045,48627	530,119626	472,983882	565,393356	482,018902
439,261383	380	744,458225	2091,314	537,512382	476,803519	587,606188	484,065691
441,259888	380	757,74321	2088,59533	549,357059	479,820073	601,397056	487,60509

This journal is © The Royal Society of Chemistry 20xx

Please do not adjust margins

Please do not adjust margins

443,256836	380	763,591593	2098,27243	556,784279	479,531764	602,563391	491,534063
445,252258	380	784,744984	2123,23782	568,017834	471,613658	618,047933	492,059822
447,246094	380	785,790794	2128,08074	571,05691	466,684594	619,814503	496,943625
449,238434	380	782,987663	2086,78619	575,0865	465,305573	627,107545	491,469144
451,229187	380	805,308539	2104,73508	585,241127	471,943652	649,690245	499,535128
453,218445	380	809,415117	2078,57099	582,988872	465,089729	649,189587	500,367781
455,206116	380	817,515986	2090,25818	591,595365	473,287466	647,868182	498,955869
457,192261	380	817,490171	2061,41048	606,012953	469,170388	650,577987	506,491105
459,176819	380	823,889745	2030,25723	595,712582	462,203557	651,806763	494,758968
461,15979	380	821,786861	2031,44524	609,148986	452,534039	660,938235	493,601427
463,141296	380	820,211808	1990,07973	613,193379	461,194611	664,167779	491,374263
465,121216	380	805,017027	1961,35212	601,233796	450,420855	655,981076	481,163891
467,099579	380	817,12862	1922,66938	609,457259	443,840212	656,340926	470,509994
469,076385	380	811,218928	1900,41001	599,62546	443,569362	660,85005	464,339224
471,051636	380	819,597123	1867,08625	601,090612	440,30042	658,786256	462,903538
473,02536	380	809,830556	1844,07903	592,822119	430,840448	663,912434	454,194002
474,997498	380	798,189904	1803,57962	598,688674	433,500462	663,573147	449,30464
476,968079	380	797,873911	1767,18287	591,710171	420,121618	659,530001	434,55083
478,937134	380	801,790617	1733,76018	586,714832	415,520853	642,626648	427,53341
480,904572	380	793,344146	1694,64202	578,2547	415,116387	642,476774	420,873095
482,870453	380	795,83443	1655,60336	578,730408	403,569046	650,839692	425,227798
484,834808	380	765,625584	1618,69559	577,687824	395,877316	645,814792	409,072617
486,797607	380	780,095891	1591,47292	578,368262	390,459137	643,279928	400,582575
488,75885	380	765,576136	1549,39942	574,832701	382,042361	638,473194	382,9036
490,718506	380	763,86798	1522,40738	565,03796	377,46925	639,933657	378,603248
492,676605	380	756,14697	1482,18075	564,221789	368,339273	626,62691	374,728695
494,633118	380	743,494243	1434,8921	559,268685	361,037894	614,384667	357,594752
496,588104	380	733,280741	1391,76648	547,164392	353,816227	610,281349	349,984489
498,541504	380	721,808401	1373,03044	540,250345	347,981778	598,825948	340,882717
500,493347	380	713,180164	1322,56621	532,259	333,424055	601,73785	331,124226
502,443665	380	688,276	1286,97235	519,083537	332,072739	591,061663	311,801232
504,392365	380	698,57772	1256,24492	508,94684	316,710906	572,236285	309,80562
506,339539	380	671,224646	1212,28228	514,785063	306,766992	573,513959	297,87222
508,285095	380	670,146425	1170,51125	501,148891	299,615775	568,039306	292,850333
510,229126	380	646,425941	1130,20484	483,564402	292,637645	554,447627	279,827018
512,17157	380	633,880526	1083,25822	488,15037	283,749511	542,224129	271,892236
514,112427	380	617,053929	1051,87172	475,228741	271,36423	533,936928	258,724697
516,051697	380	612,289128	1010,02351	456,236945	264,246346	515,192626	251,004512
517,989441	380	598,369242	978,103336	449,828235	249,118563	508,824857	244,540642
519,925598	380	580,161769	933,862689	439,206063	247,129148	492,678278	231,796809
521,860168	380	564,073373	911,758968	425,907881	240,721313	477,744773	220,440985
523,793152	380	555,939151	865,136125	423,447879	230,212146	475,732073	213,443145
525,724609	380	545,585359	844,58961	412,000251	222,193141	458,252969	205,418737
527,654419	380	542,566467	820,78157	401,946893	219,597112	448,927219	196,678744
529,582764	380	530,645736	787,087575	395,949642	212,984672	448,396527	190,544783
531,50946	380	519,03462	754,983149	392,755938	205,733934	433,961145	185,788647
533,43457	380	514,237465	742,24537	387,304456	197,16861	430,973438	177,605911

This journal is © The Royal Society of Chemistry 20xx

Please do not adjust margins

Please do not adjust margins

535,358093	380	488,427221	706,056777	381,528445	194,267408	417,572437	174,695184
537,280029	380	485,051952	691,002102	368,163276	187,564385	403,585465	169,739226
539,200439	380	472,398817	660,470017	362,852124	183,496011	392,609863	162,822264
541,119263	380	466,127098	628,574579	346,685598	173,043992	392,468939	153,503217
543,036438	380	460,295111	620,370494	340,446053	170,431015	382,01039	148,0791
544,952087	380	443,058562	583,921634	333,006227	166,78536	365,957147	141,040299
546,866211	380	433,439037	571,483111	325,090882	160,023967	354,548173	139,223555
548,778687	380	420,0645	548,781195	320,0877	151,222465	348,454382	129,758704
550,689514	380	412,308169	528,555944	311,66494	147,098923	332,695959	127,539854
552,598816	380	400,296658	514,327087	302,893335	140,060585	330,908638	119,49611
554,506531	380	386,778157	487,461322	291,089717	140,155669	321,798674	119,830733
556,41272	380	380,181949	469,139855	288,037504	131,309319	312,570874	113,091159
558,317261	380	372,351955	452,492581	282,090334	126,048354	308,171209	107,617269
560,220215	380	360,761666	433,727949	273,778739	124,596412	295,066656	101,615568
562,121582	380	349,200613	417,056562	264,95504	121,668042	288,378817	104,605192
564,021362	380	340,485045	397,938574	254,547629	115,126089	280,325912	97,6809521
565,919556	380	337,804735	386,634206	251,629317	113,724009	280,02575	95,8138517
567,816162	380	324,443349	378,067471	244,478359	109,521529	267,953292	89,2344991
569,711182	380	316,390838	362,191958	238,939165	102,975301	263,669328	82,4107246
571,604614	380	311,247174	346,684245	234,811597	101,702655	253,267845	82,2310888
573,49646	380	297,403015	334,713941	230,679526	97,3327981	244,76257	82,3939587
575,386719	380	288,559636	318,768548	220,647477	95,314528	240,87616	75,9123278
577,27533	380	281,731956	314,676876	214,198157	93,2011434	228,717239	75,4517978
579,162354	380	282,568245	297,02676	208,757544	89,8763441	226,117931	70,4127535
581,047852	380	270,591935	286,903462	199,899395	89,8430056	218,615054	65,8171796
582,931641	380	262,534507	282,158685	197,170372	80,2803238	212,835128	63,7355411
584,813965	380	250,930556	261,757502	188,663411	78,3117043	205,333979	60,9294374
586,69458	380	240,498967	254,533287	188,420498	76,0409012	194,403495	57,4884799
588,57373	380	239,220327	241,787375	176,49458	72,6172778	189,162338	55,4393333
590,451111	380	228,556339	230,103736	172,60003	70,4034203	188,001984	51,6110452
592,326965	380	224,998828	224,086382	169,030591	67,6958374	176,98545	48,8749805
594,201233	380	217,629819	214,384598	159,545704	63,9316921	171,90997	49,5446439
596,073914	380	205,707043	205,075005	160,2856	62,8814499	171,81468	47,0532676
597,944946	380	199,786426	198,997175	152,410952	59,1241015	166,617993	46,6755459
599,814453	380	199,985355	190,462985	152,971265	58,8998837	159,935786	41,2980224
601,682251	380	191,120859	187,333471	148,764389	56,1663097	151,447384	40,7003529
603,548523	380	189,03591	175,144016	138,682528	52,2036757	153,375689	40,0305101
605,413147	380	180,270353	171,174764	132,949411	54,1163582	140,786342	37,9686405
607,276245	380	177,658584	162,831212	131,915472	49,4549004	138,991836	37,1642936
609,137695	380	174,878478	156,002184	130,628392	49,9589919	136,447855	36,2775782
610,997437	380	167,2904	152,226186	127,475526	46,3931197	132,557441	36,1004505
612,855713	380	160,500209	146,360499	123,1103	47,5765186	129,775473	34,868304
614,712341	380	155,906678	140,065662	119,185804	44,6972125	123,03087	32,048844
616,567322	380	149,196994	137,286077	109,454444	44,8288116	120,48907	28,9165778
618,420715	380	143,537276	130,949001	112,962523	41,3768951	113,888279	29,9605913
620,272522	380	140,81244	123,162239	107,584503	41,0451886	108,428107	27,7662009
622,122681	380	135,548689	119,891379	104,202732	38,946528	107,821251	26,2335507

 This journal is © The Royal Society of Chemistry 20xx

Please do not adjust margins

Please do not adjust margins

623,971313	380	134,989322	112,874661	105,920442	35,3418162	105,166975	25,2968426
625,818237	380	126,266719	117,558196	94,072068	34,6345498	103,805594	26,1560955
627,663574	380	124,447159	105,793071	95,1696329	32,3201932	98,5640731	25,2723218
629,507324	380	118,905409	103,669089	94,1906047	35,5483009	93,2581653	24,3775284
631,349365	380	115,229066	101,44004	91,1066609	30,370557	93,4121286	23,2054711
633,18988	380	109,924738	93,0326689	86,364755	31,2110651	88,4190877	24,4524626
635,028809	380	108,316446	92,2071527	81,1052393	29,8250422	88,3661521	21,5427501
636,865967	380	106,085952	88,9273364	82,6882611	29,9582033	83,7182933	22,0888174
638,70166	380	103,653843	87,004698	80,252571	25,7782212	80,2563204	18,7730187
640,535645	380	97,2810555	85,2746546	78,2703789	26,2872438	77,6752179	18,8548869
642,368042	380	96,7105879	79,1993697	71,4275131	28,1192451	72,2016867	18,9630317
644,198853	380	93,5593669	78,5103205	72,7832223	24,3996784	73,3874557	17,035066
646,028015	380	90,6489653	74,9854175	70,8911566	24,4279368	68,8320841	17,0547951
647,855591	380	87,0743939	74,6082249	64,3322709	23,6434089	69,66076	15,5053279
649,681519	380	85,8939446	69,7304486	62,3453502	24,3750949	69,4293676	14,2103944
651,505798	380	79,2843283	64,8046733	60,1736593	22,7393779	65,2923377	14,441714
653,328491	380	80,4017017	67,3571383	59,2284954	21,0682901	67,3201134	15,6139006
655,149536	380	75,3491409	64,10359	53,1587946	20,1836705	59,4463554	14,016482
656,968994	380	74,0769724	60,5924826	55,7450393	19,6529371	59,0784778	12,3260391
658,786743	380	74,6091109	56,7360262	54,4271847	17,7195949	57,4242433	12,2094897
660,602905	380	65,393455	56,1629835	55,3507411	18,9987354	55,5298927	11,7101608
662,41748	380	66,7553	53,1280892	47,8846399	20,0604763	50,0151828	11,3205559
664,230469	380	65,9951955	53,143904	47,2683247	15,653144	51,7132524	11,7859756
666,041748	380	60,8442459	48,5143062	47,5326072	17,7310229	49,4041751	10,9197025
667,85144	380	62,982507	48,468818	48,0203004	17,511728	45,2554456	10,7716761
669,659424	380	58,3527234	46,327284	41,4101441	14,2348603	45,6158968	10,793603
671,46582	380	54,6380482	44,4909622	44,4965116	17,2068408	43,8718497	9,55451552
673,27063	380	53,3184441	41,9637631	44,041697	15,2949204	41,1674278	10,4883976
675,073792	380	52,5172086	42,2250214	43,0458588	12,259499	41,606937	8,622437
676,875305	380	49,0730431	39,691212	40,4266244	14,8708372	39,3480184	9,15960251
678,675171	380	50,4366663	40,2388496	38,9021764	14,061935	37,5531608	7,09532937
680,47345	380	44,4370945	37,4133996	36,1730046	11,529191	36,1746946	8,52584539
682,27002	380	45,67867	35,1909539	35,3954109	12,2018307	36,6612455	7,29459572
684,065063	380	44,011827	34,9448587	33,6105735	11,0634908	37,5984897	5,42136416
685,858398	380	46,3964276	33,5072863	33,3534039	11,4312369	35,5302858	7,32204983
687,650024	380	42,1625204	30,3592168	34,1123309	12,1691063	34,5675672	6,78641058
689,440186	380	41,073412	31,6137683	30,0082165	11,4690415	31,0099391	6,16723468
691,228577	380	37,1668939	28,6213444	31,1147232	9,74437411	30,752245	5,53509384
693,015381	380	37,9932242	28,3281538	25,9032568	9,67770171	32,1069451	4,91230256
694,800476	380	37,7091532	30,5136776	26,9910172	9,31017937	27,7217993	5,18391311
696,583984	380	35,5639394	25,5001195	27,4661716	10,814071	26,4603148	4,7482714
698,365845	380	33,4521574	26,8595592	25,523503	9,19021812	25,616511	6,95928133
700,146118	380	34,4184698	23,6421576	23,4131751	8,67330831	26,0875516	3,49353831
701,924683	380	31,9501923	21,508075	25,9090234	8,8919002	26,6505262	7,19851528
347,741211	385	0	0	0	0	0	0
349,808533	385	0	0	0	0	0	0
351,874329	385	0	0	0	0	0	0

This journal is © The Royal Society of Chemistry 20xx

Please do not adjust margins

Please do not adjust margins

353,938568	385	0	0	0	0	0	0
356,001343	385	0	0	0	0	0	0
358,062561	385	0	0	0	0	0	0
360,122253	385	0	0	0	0	0	0
362,180481	385	0	0	0	0	0	0
364,237152	385	0	0	0	0	0	0
366,292328	385	0	0	0	0	0	0
368,345978	385	0	0	0	0	0	0
370,398132	385	0	0	0	0	0	0
372,44873	385	0	0	0	0	0	0
374,497803	385	0	0	0	0	0	0
376,545349	385	0	0	0	0	0	0
378,5914	385	0	0	0	0	0	0
380,635925	385	0	0	0	0	0	0
382,678955	385	0	0	0	0	0	0
384,720398	385	0	0	0	0	0	0
386,760376	385	0	0	0	0	0	0
388,798767	385	0	0	0	0	0	0
390,835663	385	0	0	0	0	0	0
392,871033	385	0	0	0	0	0	0
394,904877	385	0	0	0	0	0	0
396,937195	385	96,9401724	386,685008	75,8733889	200,8236	89,338089	96,8087969
398,967957	385	113,832434	460,198309	85,2936912	192,427242	101,222128	104,199857
400,997253	385	133,672229	541,189962	96,2128645	193,506469	119,313136	117,766912
403,024963	385	147,670942	630,821751	114,556945	196,167788	134,638897	135,731789
405,051147	385	169,825715	725,953666	122,449736	213,35284	148,962544	149,85536
407,075806	385	196,304529	826,440686	138,212746	220,030578	167,927132	175,867722
409,098938	385	219,180608	922,984421	153,837098	231,219319	189,662874	199,114812
411,120544	385	245,831739	1020,88478	181,189498	244,71792	219,854374	206,500961
413,140564	385	285,948997	1080,81428	196,092027	256,605371	242,490734	222,106425
415,159119	385	311,307748	1181,71411	218,838877	277,311782	263,721618	244,59297
417,176086	385	345,220845	1241,0278	239,740592	291,963692	286,604566	265,680182
419,191528	385	376,435316	1344,38452	263,598323	302,492179	305,94476	269,585522
421,205444	385	406,695666	1408,83886	282,239924	324,075303	333,258136	290,189907
423,217834	385	444,925523	1461,24905	304,700017	338,138899	355,708059	310,985674
425,228668	385	470,733723	1514,9965	330,456214	336,862019	382,084408	323,396029
427,237976	385	497,707364	1594,20493	351,65186	357,052814	404,777619	340,831865
429,245728	385	528,899218	1620,70103	368,357772	369,079749	424,847465	350,990959
431,251953	385	558,06263	1672,06719	393,493336	395,536623	444,29946	365,622265
433,256592	385	598,588002	1740,21702	414,590142	405,701614	469,704167	389,305078
435,259766	385	636,374763	1800,35574	439,295134	424,353745	493,556019	406,771101
437,261353	385	654,263606	1846,91235	472,887185	438,981821	519,66327	418,135152
439,261383	385	672,342969	1878,83833	482,563151	443,663367	531,068497	428,359862
441,259888	385	696,466231	1896,17	500,274669	459,262784	551,521377	443,281235
443,256836	385	708,800104	1930,82322	503,568945	456,916509	566,131562	444,799323
445,252258	385	725,736084	1941,41857	516,016685	452,55916	575,604532	448,763349
447,246094	385	733,909776	1919,6338	520,410005	454,75098	575,46063	445,952473

 This journal is © The Royal Society of Chemistry 20xx

Please do not adjust margins

Please do not adjust margins

449,238434	385	738,244209	1910,77368	523,76371	437,341536	588,248614	449,968633
451,229187	385	753,67292	1913,32293	537,179069	441,918651	595,261493	447,913144
453,218445	385	754,5334	1911,52095	549,076748	426,961915	604,531449	450,383329
455,206116	385	753,018658	1908,56367	543,359222	435,094666	609,675125	451,082969
457,192261	385	769,639927	1893,86686	550,050087	437,56978	621,312701	449,427379
459,176819	385	774,879108	1920,50318	558,389948	431,988136	621,33533	452,341689
461,15979	385	784,464366	1872,1173	565,000912	425,493457	619,404842	449,693863
463,141296	385	789,422161	1867,91265	573,0608	435,542069	632,517475	452,78658
465,121216	385	781,137449	1824,86626	570,104711	422,088903	634,06851	445,924266
467,099579	385	791,686757	1800,61933	572,46051	422,704479	637,41544	440,959391
469,076385	385	788,191281	1779,23535	570,618771	420,248837	637,063001	437,333151
471,051636	385	783,286986	1755,55773	570,652604	416,790979	643,913241	437,071845
473,02536	385	787,655273	1752,92903	567,554641	408,625657	647,900483	424,927187
474,997498	385	789,896093	1711,15894	570,234443	403,09686	642,260557	420,582107
476,968079	385	783,836886	1691,15474	583,252241	406,930072	641,432917	409,140553
478,937134	385	782,733627	1642,19758	571,320478	400,440597	628,598443	408,924913
480,904572	385	778,235248	1613,51697	572,63616	392,395266	630,412291	398,274522
482,870453	385	772,527729	1585,30407	570,329977	387,315905	637,63568	395,975866
484,834808	385	779,130885	1557,04066	566,954934	386,472742	638,217498	394,770013
486,797607	385	771,051239	1527,9746	571,998176	377,815502	633,805309	383,18532
488,75885	385	757,922968	1499,31211	559,270153	369,663486	622,06654	370,06163
490,718506	385	756,486382	1475,58972	568,733725	367,96567	629,394288	361,084665
492,676605	385	752,000042	1426,8054	557,606523	362,515519	622,776431	353,809259
494,633118	385	739,837127	1396,08137	562,080138	353,046055	614,2563	344,330985
496,588104	385	731,631313	1360,70113	546,174211	344,444654	614,15009	340,708459
498,541504	385	727,32497	1317,4495	541,478778	340,956443	608,710521	330,769698
500,493347	385	713,036094	1277,28926	532,907496	328,853764	596,582788	321,374151
502,443665	385	705,323124	1251,83756	526,534834	321,758924	590,639783	309,43895
504,392365	385	695,803979	1211,707	515,164947	314,15746	580,050918	296,588329
506,339539	385	683,755061	1178,42719	511,570899	301,041784	566,438395	291,729243
508,285095	385	675,333694	1137,66269	499,637384	301,021907	565,783084	284,389367
510,229126	385	665,547331	1102,99973	487,494958	284,187323	557,786527	273,044201
512,17157	385	650,076397	1081,14576	488,147197	276,087907	542,017104	264,277543
514,112427	385	632,162364	1040,32678	474,29213	270,347683	538,197861	257,822201
516,051697	385	617,059211	981,61164	471,40592	267,030894	517,627108	243,119135
517,989441	385	606,85063	959,935296	453,524963	254,588803	513,337966	237,83628
519,925598	385	584,910946	925,66911	445,755339	244,635247	505,84199	227,257333
521,860168	385	584,110259	891,21617	436,954232	238,26445	479,768837	218,888258
523,793152	385	566,700003	862,388308	427,930994	232,202328	477,195541	214,742557
525,724609	385	556,201139	814,367912	413,53708	225,228773	466,966889	201,5453
527,654419	385	546,702155	804,495062	410,03187	217,716074	456,051871	198,076748
529,582764	385	536,651063	775,728244	405,161784	207,44746	452,000273	185,581989
531,50946	385	521,566884	762,16201	392,021006	205,250969	443,525973	183,035668
533,43457	385	523,597084	724,419675	392,499889	201,611116	431,434887	173,01671
535,358093	385	510,944124	706,047927	378,477957	190,53854	416,270409	169,978044
537,280029	385	505,20699	676,324613	371,269231	187,085132	420,870731	165,533965
539,200439	385	485,668093	660,339882	366,693061	180,214536	403,314521	158,105189

This journal is © The Royal Society of Chemistry 20xx

Please do not adjust margins

Please do not adjust margins

541,119263	385	473,962635	626,461909	356,187001	177,394228	394,146919	152,508126
543,036438	385	463,775587	599,135801	344,350467	174,873114	385,36807	152,40109
544,952087	385	458,523815	587,528192	331,161205	164,689588	375,495749	137,334634
546,866211	385	435,519892	565,351829	329,314606	157,191742	367,793345	134,617884
548,778687	385	430,898569	543,678924	319,221716	150,211223	360,714485	131,601379
550,689514	385	417,889917	527,181855	313,456337	147,575338	341,051828	126,393215
552,598816	385	410,938066	507,303011	300,470868	144,728179	333,851698	121,159124
554,506531	385	396,087291	487,188929	300,804982	141,431138	331,32136	116,201863
556,41272	385	389,372012	471,64845	294,608836	131,393604	322,594307	111,188372
558,317261	385	381,038142	449,749974	280,977284	128,854382	304,724389	106,897419
560,220215	385	361,466067	428,288871	277,629089	121,705937	300,608926	103,53923
562,121582	385	355,539966	414,157323	269,002121	118,014116	296,507953	101,597632
564,021362	385	349,200294	393,882753	262,659348	114,669215	284,474347	95,866144
565,919556	385	347,672362	388,528084	250,93827	109,622516	282,413074	92,9993484
567,816162	385	328,860249	369,321297	249,190804	108,234115	274,836885	90,3294526
569,711182	385	324,393047	358,579111	241,423443	108,517552	262,620218	82,0737938
571,604614	385	309,630372	352,643705	240,074803	98,7961808	252,806435	81,0363367
573,49646	385	311,093366	334,51768	231,880202	95,0695653	245,042764	76,4936852
575,386719	385	297,741308	325,051542	220,548077	94,4772873	245,202888	73,8865889
577,27533	385	287,180397	307,898846	217,064997	89,829947	238,518189	72,7879851
579,162354	385	279,64588	299,640941	213,32806	93,0440543	228,392116	66,0286341
581,047852	385	273,493821	282,530514	204,524323	88,9461997	224,689088	65,2646466
582,931641	385	264,030517	275,72425	202,285254	81,9874981	219,010454	66,2995069
584,813965	385	258,840398	264,952892	192,701879	80,5145304	210,833088	66,7096032
586,69458	385	256,146719	252,216619	191,830555	79,4684454	204,707235	57,0648698
588,57373	385	241,990019	245,914722	180,373937	75,3526446	196,38926	54,0621594
590,451111	385	231,411114	235,880532	170,905638	71,7899098	191,225575	52,439046
592,326965	385	221,462987	223,35799	170,88401	66,3920273	186,230847	53,260627
594,201233	385	214,524254	212,034963	162,952478	66,3800838	176,018453	50,1590217
596,073914	385	210,20349	200,826216	162,378112	62,7572499	179,142001	46,8554303
597,944946	385	204,305696	194,358837	155,283357	63,621362	169,43671	44,2308637
599,814453	385	200,220053	194,817318	150,989768	59,2946916	161,685268	45,1230891
601,682251	385	194,986657	183,93157	150,717508	57,3220812	154,222437	42,2809966
603,548523	385	187,232609	177,044675	142,496321	55,2038397	149,069615	41,2803414
605,413147	385	187,996935	167,354597	143,001605	54,6122287	150,227768	39,1456832
607,276245	385	184,304144	169,091346	135,243004	52,4573971	142,78939	34,6332977
609,137695	385	171,320732	157,890647	130,893307	49,1479922	138,593154	36,8017429
610,997437	385	169,914306	149,737035	123,525443	47,3385152	131,903505	36,0496862
612,855713	385	162,15929	147,383621	120,833644	44,7018459	127,307123	34,329822
614,712341	385	162,346631	141,037427	120,244915	42,4940083	126,403185	32,3537842
616,567322	385	147,746219	138,855072	114,296976	46,5355001	118,963146	31,8086568
618,420715	385	145,204956	129,794311	116,049147	38,4336841	119,304921	30,030405
620,272522	385	145,178609	127,728187	113,455338	39,3508796	112,874676	29,346058
622,122681	385	137,347806	121,464805	107,671433	37,9285377	111,945576	27,4097657
623,971313	385	136,292627	117,006069	107,462411	36,4371761	106,542832	27,0670871
625,818237	385	132,351439	114,936419	97,5194776	35,4010115	104,097661	26,5029972
627,663574	385	126,234274	107,530186	99,9706333	34,1122589	100,480944	26,6336019

This journal is © The Royal Society of Chemistry 20xx

Please do not adjust margins

629,507324	385	122,942294	103,302464	96,305352	33,2620492	100,042462	24,9917216
631,349365	385	120,478589	100,658111	88,734155	33,5413081	93,6625647	23,2357729
633,18988	385	115,639363	96,4620454	87,9200274	30,8109037	90,3064733	22,5278129
635,028809	385	112,755508	93,6790848	86,1661826	28,5808446	90,5926619	20,482958
636,865967	385	106,442835	93,1460993	82,5549778	28,3708335	83,5804106	19,922198
638,70166	385	102,825955	87,0524377	79,5316262	27,7044768	84,3935817	19,4242206
640,535645	385	99,9869246	82,6846265	77,3011104	26,0800395	77,8153416	17,7245261
642,368042	385	97,5405807	80,0504392	76,796971	27,4767794	76,0318033	17,0666209
644,198853	385	95,7788474	78,1683336	76,7916265	24,0299144	74,7420436	17,358716
646,028015	385	94,1728573	74,0587718	68,3192528	24,3145888	73,3731296	15,930585
647,855591	385	89,6694201	71,1131319	71,2843458	22,7635073	73,7687524	17,200871
649,681519	385	89,3732669	67,617822	67,2784113	22,7133542	68,3993711	15,7520361
651,505798	385	81,7711213	67,2036127	62,1123478	22,2883097	66,8661043	15,2150093
653,328491	385	80,806732	65,6756103	60,6573917	20,4506006	63,6926085	12,9338212
655,149536	385	77,0887677	61,3863199	60,529791	18,8724395	61,7496199	13,3269435
656,968994	385	77,5825123	59,6290835	59,470053	19,4744163	61,131529	11,6440996
658,786743	385	71,4437812	53,5019164	58,5268746	19,8310775	58,8800303	12,0492076
660,602905	385	71,4367889	54,1571329	55,0595092	17,8551052	55,5892847	13,3905783
662,41748	385	69,985902	51,3929146	56,0136441	17,2285034	52,9233586	11,6924659
664,230469	385	61,4250727	50,6966493	49,838972	17,5280759	53,8245324	11,0108436
666,041748	385	67,4952424	51,0555399	50,3733481	15,7863184	50,1091384	12,3546492
667,85144	385	61,3496432	48,969981	49,3557417	16,719024	48,4682353	10,7073
669,659424	385	58,3471892	45,2333091	45,0898064	15,3272653	46,4281718	9,13303202
671,46582	385	58,2279595	45,8154114	44,5114994	15,5213431	42,9966602	11,2726313
673,27063	385	54,4153711	42,7672173	44,5963598	13,9702134	43,0755815	9,44551918
675,073792	385	54,1602896	41,8614287	44,7719622	12,8306894	44,1455255	8,12450437
676,875305	385	53,9701215	38,5303612	40,9958072	14,3532946	39,920802	8,83707087
678,675171	385	48,8507222	37,3687123	39,9256749	13,8172415	36,3381893	8,3028101
680,47345	385	48,3639555	36,4425882	38,8193742	13,089464	37,5656287	7,22596001
682,27002	385	45,3742943	36,8390862	35,6252713	11,7853549	36,3463133	7,69859277
684,065063	385	44,5295312	32,8948603	36,4657778	12,7271629	35,5644702	6,19730839
685,858398	385	45,1828786	32,276409	34,3145291	10,6557697	35,0382315	6,82901302
687,650024	385	41,2397434	30,0459738	32,811968	12,0222476	30,7341054	6,92574154
689,440186	385	40,8808357	30,9318057	31,4382109	8,96957604	33,2514369	5,04854552
691,228577	385	35,9928328	29,0411937	32,2694778	10,3336837	29,4605809	5,41181686
693,015381	385	37,3594862	28,2047578	28,170842	11,3594295	30,8981913	5,51591889
694,800476	385	36,894665	30,471722	25,80168	8,35845184	29,4368043	5,78574808
696,583984	385	36,4033166	26,2109897	26,2706891	8,30096805	31,4712561	4,71989375
698,365845	385	35,6801024	24,4529576	27,9910386	7,31805969	26,1625232	7,09973422
700,146118	385	35,6384303	23,0794241	26,0823693	9,275951	25,5322742	4,84344609
701,924683	385	30,7835181	23,4450616	22,8637149	8,29747585	24,6162776	7,70591656
347,741211	390	0	0	0	0	0	0
349,808533	390	0	0	0	0	0	0
351,874329	390	0	0	0	0	0	0
353,938568	390	0	0	0	0	0	0
356,001343	390	0	0	0	0	0	0
358,062561	390	0	0	0	0	0	0

This journal is © The Royal Society of Chemistry 20xx

360,122253	390	0	0	0	0	0	0
362,180481	390	0	0	0	0	0	0
364,237152	390	0	0	0	0	0	0
366,292328	390	0	0	0	0	0	0
368,345978	390	0	0	0	0	0	0
370,398132	390	0	0	0	0	0	0
372,44873	390	0	0	0	0	0	0
374,497803	390	0	0	0	0	0	0
376,545349	390	0	0	0	0	0	0
378,5914	390	0	0	0	0	0	0
380,635925	390	0	0	0	0	0	0
382,678955	390	0	0	0	0	0	0
384,720398	390	0	0	0	0	0	0
386,760376	390	0	0	0	0	0	0
388,798767	390	0	0	0	0	0	0
390,835663	390	0	0	0	0	0	0
392,871033	390	0	0	0	0	0	0
394,904877	390	0	0	0	0	0	0
396,937195	390	0	0	0	0	0	0
398,967957	390	0	0	0	0	0	0
400,997253	390	99,7404083	439,018629	75,2149822	216,905045	87,9296335	88,4721063
403,024963	390	113,659037	507,63892	81,2878503	202,524149	104,557053	104,07242
405,051147	390	127,178907	594,636301	93,3473594	199,028647	108,633494	116,044754
407,075806	390	145,896533	683,311447	106,461815	198,541956	129,697805	132,666665
409,098938	390	167,62334	788,270126	122,702639	209,920228	150,169175	153,346666
411,120544	390	193,501732	865,894169	139,836001	211,863444	169,036868	163,990945
413,140564	390	221,277279	951,212647	150,567673	228,461949	182,654478	180,584682
415,159119	390	250,402582	1015,01361	176,588148	239,782154	204,185729	196,439071
417,176086	390	273,067513	1102,4579	188,861419	254,194193	226,795107	217,270344
419,191528	390	301,112744	1166,67209	209,560207	265,709479	246,8024	224,812754
421,205444	390	334,210245	1217,22076	229,054035	271,607817	265,43244	233,281482
423,217834	390	364,988157	1273,90683	251,237315	287,860586	294,078611	254,603712
425,228668	390	400,283131	1340,43409	272,631071	293,592164	311,053442	272,862207
427,237976	390	430,011395	1391,37066	293,136879	306,645502	335,90767	278,248137
429,245728	390	444,569395	1414,53019	311,579298	327,241404	353,401988	292,376532
431,251953	390	487,845947	1485,09164	334,468906	338,227974	378,812412	309,78637
433,256592	390	508,997162	1533,77015	353,152976	354,486948	397,820362	321,536466
435,259766	390	538,857026	1572,81488	377,911816	363,96013	428,909143	339,21132
437,261353	390	556,92149	1616,29598	395,37963	375,237167	440,352862	354,472952
439,261383	390	593,628707	1659,05817	406,840153	393,787793	469,870995	357,601069
441,259888	390	615,420942	1697,47193	429,921254	409,375237	484,247212	387,903009
443,256836	390	626,201546	1723,99071	448,383017	412,830677	504,398646	385,422304
445,252258	390	656,479021	1727,4794	457,675002	414,552525	524,085824	400,626874
447,246094	390	672,02104	1743,03735	470,110953	420,501366	532,342417	403,092701
449,238434	390	675,193799	1744,49269	477,76097	413,166582	548,487662	404,199479
451,229187	390	688,929054	1741,4804	490,488041	417,509555	546,338096	404,460873
453,218445	390	694,998185	1744,29461	496,370145	411,502918	562,738423	416,878414

 This journal is © The Royal Society of Chemistry 20xx

Please do not adjust margins

455,206116	390	709,503083	1735,82101	504,243381	401,210452	569,657214	412,444395
457,192261	390	714,598488	1742,87295	509,543754	395,786858	579,049276	411,689515
459,176819	390	724,002729	1728,0349	516,047868	391,90108	577,46354	408,49226
461,15979	390	729,281249	1713,32091	520,763682	396,448677	594,054453	411,258881
463,141296	390	738,768791	1696,74775	522,120237	403,34701	589,939949	409,247615
465,121216	390	743,349993	1694,25935	524,524798	391,246447	591,78735	401,621149
467,099579	390	737,628574	1675,779	541,902074	403,327195	601,642271	408,276174
469,076385	390	750,692961	1646,3153	543,135187	405,57594	601,241442	398,007941
471,051636	390	754,895401	1625,90138	547,397801	388,019407	616,053764	402,593184
473,02536	390	750,100179	1617,66191	546,361858	389,21114	612,962664	386,865009
474,997498	390	749,726343	1606,55505	554,085316	381,473914	610,932664	393,83036
476,968079	390	756,275829	1583,00041	533,269313	377,728154	616,621028	382,646528
478,937134	390	754,624206	1530,27404	539,915279	381,838771	612,609162	379,452833
480,904572	390	760,097147	1520,02209	545,244519	373,356036	619,083638	372,659109
482,870453	390	761,415487	1508,0379	549,174038	371,464834	620,052452	365,930772
484,834808	390	762,102146	1476,27311	545,242646	361,720441	626,473348	367,029047
486,797607	390	758,087697	1457,61899	552,843168	361,645291	621,679784	361,596243
488,75885	390	760,996358	1418,4411	545,916837	360,383295	621,804474	345,870885
490,718506	390	753,705063	1414,82094	554,497692	353,289436	615,540114	347,71356
492,676605	390	741,953313	1362,78288	541,336991	342,023784	615,836579	332,350972
494,633118	390	734,04271	1340,33067	533,524162	337,175189	616,129077	322,222577
496,588104	390	735,133315	1308,36544	527,708225	335,785616	603,048001	326,130897
498,541504	390	729,799399	1261,09177	527,739867	324,926676	601,714348	318,297063
500,493347	390	718,714514	1228,28053	530,321118	318,151545	603,555854	302,754187
502,443665	390	707,730077	1210,89223	517,318032	309,544501	594,004623	303,792587
504,392365	390	695,566321	1169,55381	513,565088	304,982205	581,691513	283,457835
506,339539	390	679,159708	1140,39345	501,082523	299,393008	570,733183	281,498985
508,285095	390	675,629271	1102,09234	501,533412	290,439633	568,650975	266,724979
510,229126	390	671,718949	1062,9399	490,460216	282,92521	559,744335	261,707536
512,17157	390	655,793677	1030,76258	474,164889	272,90464	548,881006	251,292364
514,112427	390	645,086006	1006,16733	467,603796	266,308578	544,378563	248,617854
516,051697	390	627,105583	960,458379	462,291249	253,271361	531,004376	237,207602
517,989441	390	619,027801	937,502665	459,623417	249,36499	512,888094	226,339132
519,925598	390	605,926888	894,867913	449,695093	240,185226	498,849205	216,812238
521,860168	390	597,600301	866,418226	430,927154	233,775168	492,686824	213,133753
523,793152	390	572,960349	834,975735	422,351519	228,453954	484,732521	205,601179
525,724609	390	564,896846	806,922632	420,955563	219,248689	473,028911	196,188288
527,654419	390	562,657611	786,981534	415,453719	213,778808	467,47626	189,452422
529,582764	390	550,693833	757,121555	402,483266	208,386684	457,11299	187,258997
531,50946	390	537,660296	739,952962	406,147814	208,159732	452,28452	177,781798
533,43457	390	529,487973	711,408022	388,061013	200,061463	436,718195	176,770331
535,358093	390	514,341809	690,071432	381,143788	190,982751	427,608688	168,037193
537,280029	390	503,990965	662,295682	383,91787	181,92113	420,835942	161,826181
539,200439	390	493,135427	644,849139	369,240806	178,968515	407,243561	154,254622
541,119263	390	482,291407	620,015424	357,360217	176,081636	404,933598	148,763714
543,036438	390	475,888015	598,075657	352,728048	171,387561	390,423173	145,400671
544,952087	390	464,998331	580,448593	344,581631	165,356529	381,638631	140,35811

This journal is © The Royal Society of Chemistry 20xx

Please do not adjust margins

Please do not adjust margins

546,866211	390	442,884793	561,843132	336,198227	158,030189	374,311684	133,178316
548,778687	390	433,483017	533,983946	332,002419	151,996427	365,324785	125,331901
550,689514	390	426,7657	520,019895	313,230727	146,437681	351,56721	125,284429
552,598816	390	416,96095	494,912062	308,276729	138,60674	343,898193	118,91935
554,506531	390	406,467574	481,785841	305,076156	135,398851	336,378924	111,835919
556,41272	390	394,927596	461,986678	297,6783	133,302909	323,60894	114,328804
558,317261	390	383,618093	443,388278	291,699741	127,698044	316,396424	107,831751
560,220215	390	379,748594	427,610424	285,525789	123,798527	308,99113	103,029562
562,121582	390	368,556098	413,06304	269,565451	118,96779	301,918518	96,5707875
564,021362	390	357,849234	400,261588	264,481627	114,632289	288,926633	94,6092043
565,919556	390	350,783049	380,539096	256,921449	110,919033	288,21377	91,3599232
567,816162	390	336,562953	370,783286	255,046291	108,762772	275,86392	87,2239298
569,711182	390	334,405885	358,05567	247,72151	102,656535	268,601294	83,4809566
571,604614	390	330,416125	342,972111	240,465573	100,064649	261,3534	78,5569607
573,49646	390	309,268643	331,403292	232,306935	96,2753297	251,363502	75,2705606
575,386719	390	301,958526	312,264817	230,150347	94,4290726	248,629767	77,3709649
577,27533	390	301,938622	298,432074	225,234822	90,2721313	235,178326	72,6698201
579,162354	390	283,88781	291,367924	213,921877	86,489616	230,634728	66,561408
581,047852	390	275,459342	280,189402	211,37294	87,561916	221,532352	67,3899176
582,931641	390	276,259438	276,11901	206,115061	83,4153082	217,545669	61,7544119
584,813965	390	261,74377	264,825192	199,057304	79,2327103	210,990183	61,2767752
586,69458	390	255,533547	251,248853	193,483724	78,5074318	206,890136	61,4600802
588,57373	390	246,030815	243,35255	185,064083	73,2717858	192,808452	56,8042029
590,451111	390	236,346172	228,205344	180,313219	69,9470645	193,896198	53,0257305
592,326965	390	233,08755	220,996969	169,804836	65,4520756	179,907668	52,4617416
594,201233	390	220,126591	210,609205	164,552194	66,014339	177,472466	47,3817855
596,073914	390	218,127005	203,444603	164,664015	64,0719227	175,338316	50,1376557
597,944946	390	206,050373	191,73175	158,011288	62,010965	170,422689	46,3541044
599,814453	390	204,559851	191,110038	156,22214	59,2826714	162,441519	42,8462319
601,682251	390	204,244646	182,178812	147,638284	54,7405595	159,400909	40,7610212
603,548523	390	194,418096	174,818633	143,0983	53,2140779	153,754355	40,5911107
605,413147	390	188,614071	168,475569	143,847654	53,2893202	152,16965	37,7219478
607,276245	390	181,039277	163,009017	138,015807	52,7951699	146,567928	38,7336621
609,137695	390	174,470262	157,880757	134,090008	47,6801354	141,47046	36,7842559
610,997437	390	170,176107	151,739914	131,795609	45,1295988	140,262854	36,7748298
612,855713	390	166,922846	147,077452	129,200136	47,6265894	131,71486	34,4838177
614,712341	390	162,303791	138,760242	120,437223	43,5267667	129,251389	33,9166731
616,567322	390	155,822432	133,758205	115,176626	44,8184305	123,414405	28,5007506
618,420715	390	150,302861	133,122986	112,771768	38,8166702	116,863454	29,5354731
620,272522	390	145,946804	120,065437	113,760523	39,7321643	113,446983	27,7742902
622,122681	390	139,065224	120,658263	110,085814	39,1499351	113,107004	27,9171963
623,971313	390	136,005859	117,218099	104,533267	34,4859746	110,319605	26,0773793
625,818237	390	131,657311	111,540319	100,748167	33,5296934	103,276276	26,8494898
627,663574	390	129,526048	103,562607	100,757054	32,8309936	99,1646105	24,969509
629,507324	390	123,889007	101,61695	97,365465	34,1770525	103,54441	23,8294487
631,349365	390	125,422934	101,776588	96,4222544	31,5778123	95,2396366	22,6673007
633,18988	390	119,081288	96,0683758	88,5086861	31,3969814	92,4921064	23,8182363

This journal is © The Royal Society of Chemistry 20xx

Please do not adjust margins

Please do not adjust margins

635,028809	390	114,759638	90,3232147	85,6596835	29,3428427	92,7759498	18,7393007
636,865967	390	109,834099	93,5281886	83,6747272	30,5788161	86,1287456	18,6846411
638,70166	390	105,464636	84,3917983	80,0696286	26,6861883	86,601035	21,6556479
640,535645	390	99,5682678	81,3142551	82,0923752	26,1714534	80,2972983	17,7611359
642,368042	390	101,905467	80,6458291	76,1565907	25,4373256	77,841887	16,6817192
644,198853	390	96,9976889	74,4190917	72,8285305	23,6165241	75,2816125	14,765895
646,028015	390	92,6609329	73,1382107	72,5717638	23,7292325	73,9177583	17,9265068
647,855591	390	89,2848269	71,3979587	71,0907539	22,9509214	71,2403955	14,6066496
649,681519	390	86,2378464	66,1993466	66,4153673	23,8458303	68,1909565	15,3678983
651,505798	390	83,4840529	66,6436691	65,9997547	23,3408692	66,1455991	16,4597518
653,328491	390	82,3558025	68,4922169	60,5064414	19,5250794	63,4985356	14,5326694
655,149536	390	76,1363471	56,1557932	60,2930254	19,595983	60,3475792	13,8950082
656,968994	390	75,4037511	60,0483095	57,6716855	19,5028424	62,5124236	11,4392656
658,786743	390	75,9408382	55,8702389	58,2105403	18,7215564	57,3883869	12,3649066
660,602905	390	69,0889422	54,949366	55,8954662	17,9767237	56,6475589	12,8306405
662,41748	390	66,9270635	53,520925	54,2984754	16,824285	57,5186163	13,0636289
664,230469	390	68,7400265	49,6509254	51,5839327	16,5927057	54,101815	12,1230743
666,041748	390	63,9908199	46,9977811	48,0561842	16,0924641	52,8742653	9,6809542
667,85144	390	61,1450259	46,3282135	46,0623346	15,7820488	48,0545667	10,0605691
669,659424	390	58,1525858	48,7347523	43,8482944	15,3699564	49,3961755	9,46203674
671,46582	390	58,390336	43,8883192	46,6475549	15,8303443	44,0217757	10,091356
673,27063	390	57,9031704	41,5578787	42,7271065	11,6960722	44,1049867	8,62014954
675,073792	390	54,607321	41,4588188	41,8262894	13,4182389	42,8481204	7,83540146
676,875305	390	53,879767	39,8394747	39,1376959	14,0417609	41,4984525	10,3265257
678,675171	390	51,5570542	39,0399358	39,7695034	13,7756359	39,4476852	7,3899814
680,47345	390	47,8442648	38,0259361	40,3642577	11,7092912	34,4121686	7,47330862
682,27002	390	45,222959	35,7335709	38,3534703	11,7498675	36,7737171	6,78499583
684,065063	390	43,8397815	34,591205	34,7946909	10,6190289	33,8362824	6,27010992
685,858398	390	41,783367	35,327334	32,111498	9,36324669	34,4813214	7,70665463
687,650024	390	39,5665114	32,4756988	33,4939599	10,1836339	31,6317668	6,54819362
689,440186	390	39,2957944	32,8220214	31,4054843	10,3878308	32,6984825	6,02387774
691,228577	390	40,0398281	26,0534222	31,0597735	10,5736877	29,3708138	6,65651105
693,015381	390	36,4175313	31,3793963	28,5132414	10,5098085	31,9818454	6,85369423
694,800476	390	37,8611407	27,8354579	29,9540493	9,05791672	27,4449821	5,77009039
696,583984	390	35,8260991	25,671925	24,2598516	9,82203351	26,7372654	4,97868517
698,365845	390	35,7439332	25,2876054	28,4282472	8,29922729	28,9099625	8,26060732
700,146118	390	35,7949122	22,1806575	24,9740656	8,42393885	27,2857341	3,91895388
701,924683	390	32,7999444	23,0019934	26,7230489	7,90482473	26,2876538	6,221241
347,741211	395	0	0	0	0	0	0
349,808533	395	0	0	0	0	0	0
351,874329	395	0	0	0	0	0	0
353,938568	395	0	0	0	0	0	0
356,001343	395	0	0	0	0	0	0
358,062561	395	0	0	0	0	0	0
360,122253	395	0	0	0	0	0	0
362,180481	395	0	0	0	0	0	0
364,237152	395	0	0	0	0	0	0

This journal is © The Royal Society of Chemistry 20xx

Please do not adjust margins

Please do not adjust margins

366,292328	395	0	0	0	0	0	0
368,345978	395	0	0	0	0	0	0
370,398132	395	0	0	0	0	0	0
372,44873	395	0	0	0	0	0	0
374,497803	395	0	0	0	0	0	0
376,545349	395	0	0	0	0	0	0
378,5914	395	0	0	0	0	0	0
380,635925	395	0	0	0	0	0	0
382,678955	395	0	0	0	0	0	0
384,720398	395	0	0	0	0	0	0
386,760376	395	0	0	0	0	0	0
388,798767	395	0	0	0	0	0	0
390,835663	395	0	0	0	0	0	0
392,871033	395	0	0	0	0	0	0
394,904877	395	0	0	0	0	0	0
396,937195	395	0	0	0	0	0	0
398,967957	395	0	0	0	0	0	0
400,997253	395	0	0	0	0	0	0
403,024963	395	0	0	0	0	0	0
405,051147	395	93,0023842	505,44947	75,9949733	229,945451	83,1572402	83,79511
407,075806	395	107,164594	580,66201	79,2795687	200,2376	88,641089	100,350674
409,098938	395	123,140754	676,425535	87,9807304	192,69502	108,489508	112,608865
411,120544	395	143,757286	753,539548	102,774349	198,464659	120,761471	116,937984
413,140564	395	166,044387	835,349829	119,102598	202,122614	139,905961	139,438374
415,159119	395	186,991521	893,209653	132,514956	212,354605	154,341586	153,394265
417,176086	395	210,346607	967,771597	147,215559	212,687771	179,777223	163,149871
419,191528	395	239,125913	1013,69121	160,153329	226,698415	194,946206	178,629134
421,205444	395	257,709098	1066,69808	181,410371	234,591628	210,767098	194,336622
423,217834	395	293,930887	1125,46297	197,469581	240,468682	231,455393	212,095116
425,228668	395	325,426736	1161,74995	222,090208	253,748635	258,680231	218,600669
427,237976	395	343,567729	1220,24599	239,09674	264,417858	268,684766	234,781681
429,245728	395	371,210318	1238,7681	252,379805	276,817266	296,619015	237,41912
431,251953	395	406,132993	1279,55495	275,547979	285,763409	321,546688	252,225198
433,256592	395	422,534533	1342,96873	291,891024	298,780212	339,964555	265,320156
435,259766	395	451,325634	1384,63615	309,047675	303,872633	364,509308	281,168552
437,261353	395	485,239527	1413,17847	330,260394	316,092768	379,174419	288,2044
439,261383	395	504,013272	1444,1563	337,166307	332,365373	398,409271	305,549267
441,259888	395	521,867323	1489,57874	364,636811	345,621261	419,655965	311,876822
443,256836	395	542,663785	1493,50266	376,101411	352,209024	439,182087	321,37317
445,252258	395	565,328924	1512,17616	392,359776	366,221504	451,476667	333,883884
447,246094	395	581,982811	1542,21463	404,847459	375,516678	469,278276	337,44961
449,238434	395	605,782438	1537,87851	422,028693	380,171327	481,344516	345,570805
451,229187	395	618,683681	1559,34162	426,151242	386,758849	489,139794	355,126213
453,218445	395	636,587537	1563,52075	448,134105	390,915413	509,293405	363,62941
455,206116	395	642,091093	1568,26227	452,145469	386,353615	515,635088	367,357919
457,192261	395	655,75526	1548,54245	460,947671	383,00203	524,289367	365,563604
459,176819	395	655,576669	1558,60006	463,639634	376,53538	532,980206	368,842163

 This journal is © The Royal Society of Chemistry 20xx

Please do not adjust margins

461,15979	395	669,112677	1559,12801	468,198478	378,378132	539,334385	367,780134
463,141296	395	683,695005	1546,65434	473,944807	370,314644	546,381793	361,155171
465,121216	395	686,693267	1539,39445	490,522202	367,004007	557,564746	365,868241
467,099579	395	679,867241	1527,56594	493,06127	368,131585	561,852937	359,472533
469,076385	395	696,49566	1501,58842	497,483172	357,430522	564,486722	361,380124
471,051636	395	703,046252	1490,25696	498,407265	372,991078	573,045301	363,646492
473,02536	395	709,85884	1483,98438	512,050877	362,051237	587,993344	357,948084
474,997498	395	721,18448	1468,12717	518,130554	359,783965	594,498711	353,978246
476,968079	395	716,40444	1447,66618	505,443778	357,572618	586,593479	351,712861
478,937134	395	715,891832	1430,32669	510,69098	355,212	583,263065	344,098696
480,904572	395	727,699204	1422,4813	515,569405	349,858827	605,516069	341,271212
482,870453	395	728,441905	1407,72271	524,599106	344,800325	594,610898	340,331148
484,834808	395	725,227147	1383,73725	519,999269	340,549593	604,864145	333,89381
486,797607	395	736,764549	1360,13964	522,862523	340,277064	605,032337	330,260161
488,75885	395	735,427413	1324,19953	532,107415	330,920259	604,929023	329,057425
490,718506	395	734,535563	1320,55002	531,186081	334,268267	606,879726	318,188008
492,676605	395	729,583925	1277,15251	523,102173	332,245974	606,572403	310,638823
494,633118	395	716,283146	1261,63138	529,664956	313,70715	603,174719	312,742546
496,588104	395	721,043873	1251,06702	518,519965	316,253778	587,674071	307,570278
498,541504	395	711,744861	1200,05843	526,659881	312,192332	588,455832	296,462497
500,493347	395	696,195213	1166,92895	512,539558	303,199368	586,745659	292,433314
502,443665	395	697,018157	1153,89707	509,128428	300,110665	577,476757	284,593106
504,392365	395	697,09677	1124,23081	503,725468	295,5562	573,39635	273,086544
506,339539	395	684,042635	1099,50544	496,082324	286,776134	565,695258	269,83174
508,285095	395	673,454571	1052,88344	493,525369	278,024343	562,170707	257,188408
510,229126	395	652,086492	1024,99111	478,489614	267,833479	553,949249	251,980682
512,17157	395	650,673968	988,792776	475,860822	264,146638	546,217322	242,78111
514,112427	395	644,246867	971,745926	467,21869	261,650358	531,933567	233,546362
516,051697	395	623,433147	929,254297	465,075984	245,07405	525,339132	229,45916
517,989441	395	616,953362	900,741206	451,776895	240,003832	514,95374	220,891858
519,925598	395	604,410757	870,579905	446,673953	235,757322	499,297314	212,230781
521,860168	395	590,53703	834,560412	434,223675	221,512387	491,264387	200,926
523,793152	395	577,866156	814,36344	427,431504	220,089523	484,873571	196,852526
525,724609	395	569,599142	789,731715	415,233396	216,037673	472,205104	189,136153
527,654419	395	565,586286	764,523467	405,612049	212,431716	465,078913	184,415461
529,582764	395	548,152511	742,946713	405,607898	201,146724	449,527994	176,97509
531,50946	395	547,405231	725,228645	398,999181	201,300594	447,788752	173,391837
533,43457	395	537,490689	702,58687	389,940452	190,330487	444,388125	167,165093
535,358093	395	525,260679	673,988497	385,346032	188,508253	433,854304	159,210021
537,280029	395	513,246996	649,016261	372,716847	183,663264	430,437587	157,477747
539,200439	395	494,089656	631,179559	370,25362	174,940431	416,664461	151,404404
541,119263	395	493,592863	613,986276	361,683595	171,114282	410,353863	147,072113
543,036438	395	483,938887	588,679577	358,358494	164,285127	399,011635	139,267367
544,952087	395	468,898742	567,315474	339,114624	157,770194	392,979431	134,779864
546,866211	395	453,962576	545,406846	336,177195	159,766393	369,40358	132,341414
548,778687	395	452,47474	520,16059	329,402889	146,109649	366,411503	123,71792
550,689514	395	435,599574	508,872411	318,817514	143,832675	360,074112	123,896731

This journal is © The Royal Society of Chemistry 20xx

Please do not adjust margins

552,598816	395	421,391344	484,738269	313,694987	139,687951	348,57902	116,205814
554,506531	395	412,367195	471,993505	299,613496	136,007578	339,680081	111,119086
556,41272	395	406,33448	450,500163	298,152054	130,403306	337,781371	110,18674
558,317261	395	388,649872	429,280188	288,883569	123,992524	326,546916	101,540326
560,220215	395	381,599499	417,480736	281,535015	123,709658	316,482914	99,5248066
562,121582	395	371,23283	409,846781	277,495622	117,532138	306,990847	95,6564128
564,021362	395	363,006041	391,815246	265,09832	113,728848	293,484541	91,3885312
565,919556	395	361,612028	377,230261	259,595653	111,15218	290,091717	88,1090957
567,816162	395	345,328841	363,070944	259,986068	110,109116	276,684925	84,1121855
569,711182	395	336,355133	347,725631	250,830659	101,289723	273,015706	80,425357
571,604614	395	327,694962	343,052321	241,812854	100,668635	263,476962	79,3307812
573,49646	395	320,310354	328,382974	234,814703	94,7940637	261,551853	78,5646174
575,386719	395	306,746262	314,931968	229,312923	93,9095895	249,70402	72,9338664
577,27533	395	302,885392	300,373293	224,655746	89,2884835	244,532647	71,3162062
579,162354	395	292,761928	289,158082	220,410734	88,1493769	236,287545	65,3858306
581,047852	395	284,856787	277,951781	213,407467	83,9677305	228,091843	66,12229
582,931641	395	274,653226	272,38406	205,970852	81,7747617	217,204796	61,2823331
584,813965	395	270,323801	257,123305	195,198954	75,7029155	218,313765	65,9796476
586,69458	395	263,318158	249,690386	198,931778	75,5355507	207,388373	57,4633207
588,57373	395	251,512076	239,330935	186,273122	72,4433793	202,366774	55,9462121
590,451111	395	240,410295	227,010667	178,690707	67,9837812	194,125587	53,743553
592,326965	395	237,868333	218,180098	172,095058	65,8222431	186,563982	49,1011598
594,201233	395	229,38141	205,617885	171,131031	63,0479378	175,948038	49,847702
596,073914	395	218,633857	202,512646	168,521401	64,1628815	176,407205	47,1845443
597,944946	395	212,192861	191,90532	159,520488	58,9404274	169,853654	43,7104239
599,814453	395	205,542669	183,916357	154,646376	57,4102712	169,450553	44,6662909
601,682251	395	201,379341	177,8464	147,677085	53,3588278	160,974235	40,4139934
603,548523	395	191,891403	172,003308	147,604514	52,7559239	155,092915	41,6255934
605,413147	395	190,507124	166,096373	142,642738	51,6098685	155,886456	37,9908242
607,276245	395	185,012649	161,049661	135,774943	52,0182907	145,261937	36,7198371
609,137695	395	179,768645	157,713451	137,423918	44,4012211	145,600607	35,6930344
610,997437	395	168,978719	148,271095	131,367072	43,7584882	135,659667	33,740883
612,855713	395	167,493923	148,894335	127,924596	45,7235674	132,541725	33,2675164
614,712341	395	166,150467	140,163784	125,014147	44,3811264	130,944908	31,4093992
616,567322	395	159,22139	132,151642	119,770831	45,1624364	125,70505	30,1431304
618,420715	395	154,383181	128,99451	114,806463	39,8406057	121,073198	28,6414908
620,272522	395	147,168541	122,387559	112,515698	39,679875	116,421773	30,2516671
622,122681	395	143,952427	117,314924	108,578772	38,1255283	114,048821	27,2933114
623,971313	395	139,238289	114,260886	105,34263	36,0100822	107,866004	26,4666741
625,818237	395	132,509155	109,670656	105,016111	38,2157504	104,412544	24,3500556
627,663574	395	133,322102	104,39924	98,8374296	34,8101769	102,363595	25,1345329
629,507324	395	125,235589	101,7519	96,832832	32,5719696	99,5441002	24,5958168
631,349365	395	126,911155	96,4982582	92,7487882	30,1013859	96,8734126	22,3763131
633,18988	395	120,776142	95,4717819	91,965744	30,3383718	95,5993759	22,5947029
635,028809	395	112,439879	92,2452571	88,0810732	28,8208576	91,9603524	19,9245668
636,865967	395	112,451988	89,4685645	84,3858292	27,8656609	86,850908	18,4596176
638,70166	395	107,076349	82,4967581	83,5144871	26,0445109	87,9838528	19,218542

 This journal is © The Royal Society of Chemistry 20xx

Please do not adjust margins

Please do not adjust margins

640,535645	395	102,205676	81,0609502	80,9132604	25,2885814	77,7020134	16,6363742
642,368042	395	106,850762	75,1345049	77,169036	25,5697453	76,2915617	17,4892472
644,198853	395	98,3536559	75,2127527	76,163772	24,2850455	74,2788713	17,6939025
646,028015	395	95,0228796	72,0259533	70,7216175	22,3848852	74,6164132	17,4637193
647,855591	395	96,7446621	66,8758014	68,5128436	21,1180153	70,8963173	16,5372997
649,681519	395	90,1707315	69,6803835	68,4587621	23,1688811	70,3492701	15,5342733
651,505798	395	83,8437535	67,3407105	63,7514288	22,1642857	65,4778125	14,5863086
653,328491	395	83,6770104	62,4503017	62,6757662	20,3657461	66,8676491	12,0940558
655,149536	395	79,3721692	62,2507095	62,2224108	19,4177173	63,1928563	14,0053549
656,968994	395	78,3290348	58,3978823	57,9495982	20,1821119	61,5726515	13,3813862
658,786743	395	75,9492431	54,978371	54,8782026	17,8729495	58,8605365	13,1030129
660,602905	395	74,4893465	52,2627355	56,6439353	17,5708011	56,4161504	11,7605818
662,41748	395	68,7548158	51,8911031	53,8718808	18,341879	54,3335847	12,503247
664,230469	395	70,5410755	50,2574855	50,8590571	16,5576021	52,9670807	12,2687203
666,041748	395	63,6202286	49,0448146	48,1742528	15,2851091	50,3714696	12,2911089
667,85144	395	60,4788188	48,3076571	49,4381894	15,8496429	46,1556334	9,63425012
669,659424	395	59,0278538	46,1342312	46,2246089	16,056433	44,5915841	9,56688995
671,46582	395	59,0882078	41,6332335	47,5814533	17,3831368	45,598056	9,05430785
673,27063	395	56,6833029	39,6011794	43,4731657	14,7327135	44,9874709	10,4882143
675,073792	395	53,1823945	40,9091114	41,3614745	12,3887671	44,1954656	7,00560761
676,875305	395	51,9398414	37,8227592	39,3342413	13,2648878	40,3218151	10,1562053
678,675171	395	50,3632386	36,7730835	40,2186284	13,7063004	38,3074189	6,39242077
680,47345	395	49,8848945	33,7561858	39,1349708	11,237611	38,190385	6,21254033
682,27002	395	47,8506286	33,6967386	37,9498258	11,1003567	36,5607619	6,93649895
684,065063	395	45,086577	34,1627586	36,1324114	10,6069069	35,7099817	7,22384148
685,858398	395	46,5242217	32,0526933	35,4411938	10,3462675	34,5760479	7,75581352
687,650024	395	42,7560974	29,8687512	33,176185	11,59579	30,6271016	6,35196928
689,440186	395	43,1216582	29,7601192	32,2774527	10,8240944	32,2061728	6,10137443
691,228577	395	40,2073768	28,1749214	31,3174337	10,1192933	31,3343573	4,68886844
693,015381	395	36,3851076	27,4441865	30,6850441	9,07698671	29,5447531	4,96679689
694,800476	395	38,1591577	26,3683446	26,8538222	9,25265516	28,5587255	6,38428667
696,583984	395	35,806703	24,2387254	27,051486	9,37932682	27,5127397	5,34171196
698,365845	395	38,7799014	24,4862199	27,8437354	7,88285708	26,0672203	7,14229548
700,146118	395	36,4112367	23,143196	27,325732	9,17344083	27,3404985	3,7646814
701,924683	395	32,8418116	22,0570004	26,6186884	8,3961745	23,9246247	5,75871157
347,741211	400	0	0	0	0	0	0
349,808533	400	0	0	0	0	0	0
351,874329	400	0	0	0	0	0	0
353,938568	400	0	0	0	0	0	0
356,001343	400	0	0	0	0	0	0
358,062561	400	0	0	0	0	0	0
360,122253	400	0	0	0	0	0	0
362,180481	400	0	0	0	0	0	0
364,237152	400	0	0	0	0	0	0
366,292328	400	0	0	0	0	0	0
368,345978	400	0	0	0	0	0	0
370,398132	400	0	0	0	0	0	0

This journal is © The Royal Society of Chemistry 20xx

Please do not adjust margins

Please do not adjust margins

372,44873	400	0	0	0	0	0	0
374,497803	400	0	0	0	0	0	0
376,545349	400	0	0	0	0	0	0
378,5914	400	0	0	0	0	0	0
380,635925	400	0	0	0	0	0	0
382,678955	400	0	0	0	0	0	0
384,720398	400	0	0	0	0	0	0
386,760376	400	0	0	0	0	0	0
388,798767	400	0	0	0	0	0	0
390,835663	400	0	0	0	0	0	0
392,871033	400	0	0	0	0	0	0
394,904877	400	0	0	0	0	0	0
396,937195	400	0	0	0	0	0	0
398,967957	400	0	0	0	0	0	0
400,997253	400	0	0	0	0	0	0
403,024963	400	0	0	0	0	0	0
405,051147	400	0	0	0	0	0	0
407,075806	400	0	0	0	0	0	0
409,098938	400	0	0	0	0	0	0
411,120544	400	100,782387	626,43877	75,3760388	209,651796	88,5556351	93,3179519
413,140564	400	122,873517	712,374203	85,9262884	191,187445	97,5914022	105,090228
415,159119	400	136,549105	766,997728	97,73604	185,253235	114,417	114,140659
417,176086	400	153,00327	813,619025	111,892996	200,708225	132,905073	120,680708
419,191528	400	176,613369	858,41695	122,340883	195,621238	151,411419	133,767928
421,205444	400	194,743069	900,405895	134,134228	194,708662	162,741148	146,861864
423,217834	400	224,735268	941,064109	151,785924	204,76372	184,186263	153,086838
425,228668	400	248,72166	991,547198	172,196393	209,863209	206,2913	164,589851
427,237976	400	275,658745	1030,74751	181,545723	220,30671	220,416976	176,200449
429,245728	400	296,676105	1045,62321	205,593567	235,037827	236,95058	185,03173
431,251953	400	320,798768	1097,43447	220,519397	236,924126	260,994878	193,38042
433,256592	400	336,893305	1126,95413	239,047145	252,109925	283,895433	210,957334
435,259766	400	367,418709	1176,76428	253,555234	260,43373	297,502949	223,881978
437,261353	400	394,691332	1215,81497	271,245399	265,029813	318,438048	232,293977
439,261383	400	416,569984	1236,12961	285,387718	276,114141	339,800355	242,653872
441,259888	400	447,986872	1273,71741	301,617316	289,047217	357,321143	251,738198
443,256836	400	461,207082	1278,90836	316,10115	305,576623	370,473622	263,09701
445,252258	400	479,889134	1318,41467	332,916071	312,895297	387,679638	272,600131
447,246094	400	491,277409	1334,11664	345,186141	309,835148	406,490118	280,253542
449,238434	400	510,768672	1338,48179	354,116498	325,392147	410,929645	287,541567
451,229187	400	533,454804	1350,71351	365,725328	335,045544	435,037495	294,120887
453,218445	400	549,985079	1366,29065	385,912015	342,440725	452,759072	302,33127
455,206116	400	564,74303	1375,88173	392,644763	346,791673	465,016869	314,198294
457,192261	400	574,590047	1380,56617	404,988671	349,672691	474,984238	313,071101
459,176819	400	599,531525	1388,46032	428,184191	354,917034	486,281066	320,093529
461,15979	400	608,056391	1394,89848	426,02564	350,000868	496,066635	327,509251
463,141296	400	625,744487	1383,08983	427,474254	345,208584	511,869858	324,101482
465,121216	400	625,704537	1385,15556	443,699001	339,267192	515,720406	318,820082

This journal is © The Royal Society of Chemistry 20xx

Please do not adjust margins

Please do not adjust margins

467,099579	400	629,683351	1383,92658	447,782203	338,075496	516,959522	323,279659
469,076385	400	643,628229	1355,85107	455,724048	332,0369	520,48056	325,478394
471,051636	400	647,207163	1357,42869	466,198268	332,743042	530,058463	320,024404
473,02536	400	658,325901	1363,72181	466,494444	333,467264	554,30266	316,182148
474,997498	400	663,726701	1350,04909	475,072089	326,705347	542,175736	317,002881
476,968079	400	666,919106	1324,74835	481,173842	333,185734	558,767135	317,768183
478,937134	400	676,860286	1322,15458	487,674909	325,853882	560,968216	317,215705
480,904572	400	685,432786	1313,44027	487,236079	327,42575	570,0408	306,462394
482,870453	400	694,381401	1294,65043	489,653097	328,552034	576,660655	309,827887
484,834808	400	699,1086	1287,38915	499,251222	314,817812	576,380313	311,748637
486,797607	400	696,206216	1277,20425	499,209348	321,19797	581,523615	309,896037
488,75885	400	706,267559	1253,83137	502,363137	310,910485	587,214124	301,362254
490,718506	400	699,301516	1228,93429	500,306729	312,074767	585,815988	300,8647
492,676605	400	702,942487	1218,79401	508,724968	308,247794	589,006636	286,64559
494,633118	400	701,880511	1188,42965	503,013646	300,397539	580,379201	286,922041
496,588104	400	705,884186	1181,89347	510,290842	296,871007	594,393501	280,508424
498,541504	400	703,450484	1145,58004	505,991127	298,997784	582,462107	276,318547
500,493347	400	686,925215	1114,58868	500,818131	289,171984	573,347504	268,946067
502,443665	400	682,637957	1097,01347	493,661303	281,461327	579,217541	265,240124
504,392365	400	683,945736	1070,38388	494,720128	277,060004	567,617673	255,802062
506,339539	400	670,108089	1043,72759	484,608134	271,128923	559,98549	245,645252
508,285095	400	655,195443	1024,16309	484,989125	266,360734	548,581325	238,488324
510,229126	400	650,695107	980,290545	461,412187	262,591182	544,70346	240,649771
512,17157	400	640,041692	955,953975	478,020657	256,255821	541,072392	238,790064
514,112427	400	647,449145	941,085717	463,134345	251,300644	535,075806	229,408967
516,051697	400	624,510162	897,046492	451,590097	245,600379	524,418334	217,377226
517,989441	400	611,238066	875,339956	445,653015	230,947163	512,38849	206,302427
519,925598	400	599,897274	839,645176	435,840588	230,020034	503,925376	200,721743
521,860168	400	593,27615	820,386499	432,253233	219,435123	490,620525	200,625044
523,793152	400	576,19725	785,619196	423,149213	211,704606	476,029529	192,859538
525,724609	400	572,788659	775,325535	414,317823	203,603644	474,567515	184,381338
527,654419	400	560,615485	748,193602	403,107495	203,810321	469,86804	182,954818
529,582764	400	554,178317	729,54573	399,75432	202,286949	457,563871	168,44109
531,50946	400	549,418887	707,997547	395,330787	193,285552	447,611256	173,012348
533,43457	400	538,322307	676,958559	394,146164	185,223602	448,760986	167,221115
535,358093	400	521,697467	660,963252	379,884546	182,054662	438,510258	155,983151
537,280029	400	511,166889	640,347022	371,316685	176,586869	430,752651	153,970438
539,200439	400	516,652802	616,416288	376,410265	171,197591	411,679369	145,059785
541,119263	400	500,58171	594,404529	359,76515	164,986115	412,329257	146,224594
543,036438	400	484,658602	577,89536	356,695212	160,487777	401,983478	137,426702
544,952087	400	472,919626	554,630351	341,52891	156,303204	393,248706	132,452802
546,866211	400	459,599263	534,230897	340,695028	157,08092	382,170186	130,414797
548,778687	400	454,367479	521,99492	332,698796	148,197578	370,397614	123,361245
550,689514	400	438,705741	499,534316	317,166133	141,957682	363,570629	120,621044
552,598816	400	426,270545	483,715695	320,61097	137,175743	349,972268	115,834322
554,506531	400	417,784051	460,602891	301,855999	133,152618	342,859583	109,820497
556,41272	400	405,679643	443,716503	293,975471	128,492413	332,437823	105,091873

Please do not adjust margins

Please do not adjust margins

558,317261	400	398,805575	433,394889	294,732374	125,951801	327,129266	102,63362
560,220215	400	384,568125	416,533478	283,480336	120,132916	322,459506	98,6209958
562,121582	400	378,522706	399,216036	277,608664	116,25179	305,847487	95,2331888
564,021362	400	366,037263	379,909487	270,269795	110,077107	298,455004	93,0967167
565,919556	400	356,08757	369,717697	265,827908	109,319713	286,889948	87,5253843
567,816162	400	345,491044	358,398195	255,04581	105,407227	286,07651	90,3497674
569,711182	400	338,140587	344,856239	249,29559	97,6357434	276,089066	77,2581952
571,604614	400	332,79655	334,272778	243,327447	98,5324717	273,132349	79,7339537
573,49646	400	326,354177	320,788864	236,779525	92,7795768	262,604187	78,5961367
575,386719	400	313,340156	311,742927	229,417491	91,2085262	255,382873	72,0203842
577,27533	400	310,189218	299,550167	224,115695	91,0889686	242,493311	69,7226879
579,162354	400	298,188974	285,266425	220,141516	84,938673	240,503014	66,4601543
581,047852	400	286,282096	272,289022	214,910383	81,946879	233,247584	63,7133941
582,931641	400	280,378519	268,170035	203,652976	78,9177736	225,395516	63,8683965
584,813965	400	269,962348	255,690249	200,75233	76,6289718	221,220939	57,7459836
586,69458	400	265,457643	243,63628	197,371092	74,9055036	211,797128	54,7269687
588,57373	400	257,567176	235,470613	189,211923	70,818459	204,407218	56,2625968
590,451111	400	251,252889	220,390523	181,177998	70,1894445	197,924066	55,0328253
592,326965	400	235,265412	215,274923	173,559356	65,6736645	190,796452	50,6078169
594,201233	400	231,388625	208,232325	171,739857	61,0680495	185,886207	45,8899999
596,073914	400	225,991473	199,406016	167,133156	59,7221583	178,116502	44,9262477
597,944946	400	221,315371	190,928097	160,887855	59,343011	175,270311	41,0776533
599,814453	400	211,624347	185,362104	157,101358	57,4180014	166,249637	43,2433712
601,682251	400	204,085749	182,091784	152,526007	55,0982711	161,808855	40,9642983
603,548523	400	200,337411	172,571804	148,728858	54,576446	156,855601	37,9796975
605,413147	400	192,339522	167,278507	144,287362	52,7107388	154,814727	37,754625
607,276245	400	189,068914	159,373535	139,563293	50,6187039	148,135124	36,2534848
609,137695	400	184,43193	150,599836	133,641505	46,6823614	139,940001	35,8761347
610,997437	400	175,456653	142,51932	128,031668	47,8214034	139,008357	32,095697
612,855713	400	171,654119	145,553222	127,756299	45,5032575	135,108912	31,7863144
614,712341	400	168,321115	136,248507	121,562738	42,4766141	128,683585	32,1135258
616,567322	400	159,60769	132,43069	117,976127	42,1988318	126,475046	29,2474157
618,420715	400	155,469931	128,331613	113,456088	39,1844541	123,338605	29,4518277
620,272522	400	151,986635	124,707375	113,048554	41,7828552	116,45442	29,7570347
622,122681	400	147,028122	115,456311	112,641864	38,7831986	116,05416	26,5796977
623,971313	400	145,147336	111,040265	109,934912	34,486784	108,863953	24,1334675
625,818237	400	136,305916	110,659605	104,887349	37,2421826	107,409417	26,1866358
627,663574	400	132,445049	104,453958	100,154167	32,8799262	102,591745	26,3143884
629,507324	400	127,905358	99,9385258	100,153288	34,1130557	102,02484	24,0623949
631,349365	400	127,092413	97,124042	94,1277562	29,8503082	97,4778826	24,0801294
633,18988	400	120,362535	93,8769805	90,7950684	30,0026654	96,8781975	22,6535806
635,028809	400	115,168216	89,9356879	88,2637119	28,7456658	91,2805278	20,7464893
636,865967	400	114,938147	85,6082825	86,9986077	26,4749524	85,1322799	21,1918699
638,70166	400	108,708976	82,755995	83,4884618	25,0822544	82,3618826	19,8834586
640,535645	400	104,79839	81,2499702	74,4557134	26,4846443	79,6957531	15,7553655
642,368042	400	101,858183	75,9578762	79,5979795	23,6991809	81,6995142	17,5912967
644,198853	400	95,0180282	74,7090096	75,8683782	24,9129127	77,3040309	16,5532851

This journal is © The Royal Society of Chemistry 20xx

Please do not adjust margins

646,028015	400	96,0427662	70,5524056	73,6293579	24,2766518	73,3158352	15,909558
647,855591	400	94,8368829	69,8560799	69,6097799	23,4349481	72,6181319	16,4823417
649,681519	400	90,4981894	65,5482037	70,0661105	20,6362913	71,1672519	15,9877325
651,505798	400	86,8424094	66,2371772	64,8194921	22,5666481	64,7418484	15,63534
653,328491	400	83,8276882	61,0519381	64,3463921	20,7007709	66,1206341	13,8384135
655,149536	400	80,0775416	58,9917276	60,105886	20,6070337	62,7256979	12,2893655
656,968994	400	79,8147755	58,7263576	60,94768	20,0137715	59,1651068	11,4949422
658,786743	400	76,3624701	55,7677349	56,0444314	19,0020337	61,4959373	13,1636967
660,602905	400	74,066114	55,1306959	53,5306624	17,0825901	57,1960164	11,236954
662,41748	400	70,3656788	51,1713161	54,3714484	18,0172948	55,6494962	12,9976496
664,230469	400	68,8708839	49,7237962	52,93202	15,7317665	54,8127732	13,1089764
666,041748	400	70,2886336	48,4355857	52,3399298	15,4159834	54,1369504	10,3003124
667,85144	400	63,3991185	46,408202	52,4838099	14,2493274	48,321251	9,29576474
669,659424	400	61,5375093	43,9047563	45,5843267	14,4514041	47,5534314	9,57342858
671,46582	400	59,2611725	41,6091337	46,1504524	16,1986569	44,3939002	10,2738994
673,27063	400	57,3189125	40,205716	42,6804151	14,4359004	45,6124097	9,10072593
675,073792	400	58,9979214	38,1033164	41,020747	12,8953466	41,1651065	7,81564386
676,875305	400	55,1530344	40,0139739	39,7068248	13,5898155	40,0237327	9,7242907
678,675171	400	51,937492	35,0504877	39,4518644	13,3305911	38,4602857	8,77062005
680,47345	400	47,9793866	33,1941358	38,8125848	10,7085697	40,2609734	6,76818007
682,27002	400	47,0097271	33,8337174	37,8118534	11,9687634	38,0394224	7,1399228
684,065063	400	49,796052	33,1567285	33,9072178	13,4110033	37,9026456	6,72684639
685,858398	400	46,6604088	30,6263775	35,7628141	10,2599686	32,9160245	6,38019535
687,650024	400	43,1071793	31,5352196	33,2538504	11,7624062	32,8608594	5,94909954
689,440186	400	41,4468677	29,7591694	32,8030522	10,3818817	32,3203514	6,22591756
691,228577	400	39,5303573	26,1621931	32,2912665	10,2109383	30,7502767	5,43939124
693,015381	400	37,3354132	26,5815553	30,4322669	10,0602485	32,6829704	4,92541502
694,800476	400	38,2967424	25,8686058	29,6309787	8,38144638	28,5242233	4,13279793
696,583984	400	35,173205	24,3774025	29,2193124	8,23811114	26,511349	5,65035351
698,365845	400	36,7824275	25,0681597	28,0583252	7,10645967	26,2301796	7,7910668
700,146118	400	35,9481218	23,5617956	25,5851325	9,007739	29,4594274	4,9777536
701,924683	400	33,668628	22,1293076	25,5045138	8,50519825	28,049241	4,63996951
347,741211	405	0	0	0	0	0	0
349,808533	405	0	0	0	0	0	0
351,874329	405	0	0	0	0	0	0
353,938568	405	0	0	0	0	0	0
356,001343	405	0	0	0	0	0	0
358,062561	405	0	0	0	0	0	0
360,122253	405	0	0	0	0	0	0
362,180481	405	0	0	0	0	0	0
364,237152	405	0	0	0	0	0	0
366,292328	405	0	0	0	0	0	0
368,345978	405	0	0	0	0	0	0
370,398132	405	0	0	0	0	0	0
372,44873	405	0	0	0	0	0	0
374,497803	405	0	0	0	0	0	0
376,545349	405	0	0	0	0	0	0

This journal is © The Royal Society of Chemistry 20xx

Please do not adjust margins

378,5914	405	0	0	0	0	0	0
380,635925	405	0	0	0	0	0	0
382,678955	405	0	0	0	0	0	0
384,720398	405	0	0	0	0	0	0
386,760376	405	0	0	0	0	0	0
388,798767	405	0	0	0	0	0	0
390,835663	405	0	0	0	0	0	0
392,871033	405	0	0	0	0	0	0
394,904877	405	0	0	0	0	0	0
396,937195	405	0	0	0	0	0	0
398,967957	405	0	0	0	0	0	0
400,997253	405	0	0	0	0	0	0
403,024963	405	0	0	0	0	0	0
405,051147	405	0	0	0	0	0	0
407,075806	405	0	0	0	0	0	0
409,098938	405	0	0	0	0	0	0
411,120544	405	0	0	0	0	0	0
413,140564	405	0	0	0	0	0	0
415,159119	405	96,5428408	594,242613	77,6507327	219,566118	86,7636818	82,616019
417,176086	405	109,906776	656,221406	78,6145429	193,604487	98,8372917	89,1607413
419,191528	405	132,384266	678,871751	90,8817843	182,020971	106,387419	91,5020826
421,205444	405	142,461847	720,234761	106,222735	174,799392	124,402917	109,388317
423,217834	405	165,563352	751,682711	110,157188	179,490063	130,350938	109,792316
425,228668	405	188,379062	793,02122	122,805571	179,406495	150,913297	125,161138
427,237976	405	211,119258	825,731301	144,242926	186,787574	171,955638	134,635281
429,245728	405	228,429945	849,891744	160,290261	191,643351	182,914894	143,35282
431,251953	405	248,880338	887,87045	170,750454	197,345804	205,503147	153,54681
433,256592	405	274,860898	920,726986	186,417848	207,032235	224,692454	162,813285
435,259766	405	293,124145	980,706784	200,21983	215,216606	240,246068	168,659063
437,261353	405	316,670407	998,6724	220,26641	223,840028	256,820159	188,280876
439,261383	405	343,913857	1022,10654	233,087671	238,586715	278,19099	198,539161
441,259888	405	359,126833	1057,16893	253,38306	242,744495	301,962039	205,648286
443,256836	405	380,977603	1072,13449	261,938285	257,331737	312,683037	215,351638
445,252258	405	399,490012	1102,4974	274,162951	251,271286	326,825821	219,186932
447,246094	405	418,434325	1127,11092	283,080237	271,576067	344,281488	228,704222
449,238434	405	433,25327	1127,38967	301,878268	274,786919	362,601877	234,856235
451,229187	405	457,156481	1149,83797	306,129322	284,423993	369,291467	243,002061
453,218445	405	462,313356	1164,93004	326,519533	295,216947	397,067427	254,981216
455,206116	405	481,952805	1181,09325	332,525785	296,559941	407,663463	261,478252
457,192261	405	500,535932	1178,5373	351,213197	312,261206	421,009613	258,627149
459,176819	405	507,250953	1197,04793	358,572778	310,257853	437,7977	268,28955
461,15979	405	539,604852	1209,4056	377,901999	318,225517	448,013612	280,15791
463,141296	405	559,680203	1233,72581	382,590775	313,672362	465,905515	284,403403
465,121216	405	563,444149	1220,4431	403,256138	321,700279	470,218168	294,030693
467,099579	405	574,60734	1209,38711	402,30437	316,893371	490,207008	288,189876
469,076385	405	577,256334	1219,24618	414,498618	322,452766	494,611448	298,490846
471,051636	405	601,8982	1214,56152	419,255032	312,96555	502,825435	288,586109

This journal is © The Royal Society of Chemistry 20xx

Please do not adjust margins

Please do not adjust margins

473,02536	405	607,802449	1220,19709	434,774771	306,162465	514,100845	290,090361
474,997498	405	606,514242	1211,94184	442,816289	299,562014	513,693432	290,649085
476,968079	405	629,257082	1196,75477	443,601709	297,765018	519,615077	282,198245
478,937134	405	630,120227	1194,53526	445,537903	299,720366	526,728387	285,565338
480,904572	405	639,906121	1183,62734	456,8978	303,771352	534,241089	283,715266
482,870453	405	645,092488	1173,70739	458,466939	297,666053	543,513148	282,412225
484,834808	405	657,385636	1172,34701	473,569089	303,350016	553,211256	285,491778
486,797607	405	670,167153	1162,42222	472,027041	296,211865	552,633523	279,614815
488,75885	405	667,544422	1151,33759	469,119261	288,125392	554,787812	269,534785
490,718506	405	679,728471	1127,94561	473,80075	291,917065	557,869957	272,499868
492,676605	405	675,301027	1114,69675	474,44864	286,095523	562,70929	273,356981
494,633118	405	681,426028	1102,8454	482,162863	283,724082	560,332742	266,899301
496,588104	405	674,547484	1093,83232	470,637744	278,537876	563,723306	261,475325
498,541504	405	677,368401	1072,86586	484,78349	276,645141	556,859121	257,202716
500,493347	405	676,278553	1049,27423	481,136867	267,359326	561,645719	252,060909
502,443665	405	667,81228	1025,83387	476,425939	267,958267	555,630229	249,325572
504,392365	405	662,176358	1002,32354	479,149044	262,684133	562,736375	244,831752
506,339539	405	658,313941	978,425595	475,341055	256,453611	553,202066	238,349639
508,285095	405	651,209832	959,141465	462,178465	251,122828	548,790084	233,453432
510,229126	405	642,381704	935,784681	458,824186	243,834154	539,671966	227,045942
512,17157	405	641,544692	907,872682	453,160177	244,572776	525,985153	221,342353
514,112427	405	627,946259	890,282655	447,517687	239,992881	531,176812	217,613272
516,051697	405	613,946081	853,786893	440,139666	230,394222	514,835399	210,630308
517,989441	405	611,814102	833,892793	439,529899	223,474678	505,475624	199,867417
519,925598	405	600,682803	810,646668	429,669185	216,125091	496,976778	192,783271
521,860168	405	582,305854	784,698885	421,43393	213,802475	487,75708	193,727235
523,793152	405	577,387492	749,153483	409,696512	205,24549	480,488634	184,073105
525,724609	405	564,299366	731,795421	409,938832	199,352433	478,557045	178,631971
527,654419	405	559,018077	715,589267	411,577259	197,947615	462,176299	168,590806
529,582764	405	558,137182	700,630399	399,826472	187,484542	463,132528	166,813311
531,50946	405	546,252145	678,371943	387,587666	184,395384	446,433376	165,746377
533,43457	405	544,936596	661,252218	387,300654	184,729242	440,905058	163,132662
535,358093	405	530,546804	634,586641	389,230097	175,287421	438,846014	151,72285
537,280029	405	515,464096	614,664682	368,8857	173,338801	427,114118	147,142371
539,200439	405	502,345723	592,777356	366,204143	167,013381	419,979182	142,297848
541,119263	405	497,699414	575,542826	354,880164	163,921516	408,129535	135,520361
543,036438	405	491,316602	554,216511	355,448774	159,792474	396,088104	133,819542
544,952087	405	475,661598	541,469962	335,941655	156,208456	381,155674	129,376942
546,866211	405	459,284959	522,387452	335,06025	144,711094	376,672941	123,141555
548,778687	405	456,375882	506,521122	330,282966	144,395487	366,988025	118,997518
550,689514	405	444,885519	480,221042	315,243386	137,771868	357,144055	116,706727
552,598816	405	433,755824	468,031994	309,255097	133,065596	353,970099	113,786988
554,506531	405	421,418955	451,490975	303,501497	128,735201	345,266344	109,625657
556,41272	405	416,638377	433,602502	302,125798	123,837329	331,447066	101,366632
558,317261	405	402,092154	421,391528	294,035969	121,890804	331,156812	101,519307
560,220215	405	393,972743	400,604719	284,93095	118,833659	318,74611	98,9909188
562,121582	405	380,708624	391,422177	274,086135	114,639717	307,583465	92,5191515

This journal is © The Royal Society of Chemistry 20xx

Please do not adjust margins

Please do not adjust margins

564,021362	405	368,342734	373,659694	271,765379	105,814618	295,508487	88,5714133
565,919556	405	360,718958	364,782435	262,704952	104,882703	292,98364	84,7705381
567,816162	405	352,458196	351,611338	254,159668	102,843364	281,104743	85,7789761
569,711182	405	349,143967	344,341146	252,346156	98,6972735	279,759565	78,4001188
571,604614	405	342,913901	335,114302	247,749751	92,5674537	271,150885	79,5913539
573,49646	405	327,12463	312,491925	234,298969	91,9195904	266,194442	74,0907334
575,386719	405	316,384294	303,869352	232,367338	85,0366262	256,582684	74,1529764
577,27533	405	308,210078	289,218568	227,761007	86,2734951	248,157937	68,8518916
579,162354	405	298,032149	281,549962	215,259518	84,7462723	244,23943	69,9842385
581,047852	405	293,957454	272,274058	218,554833	80,1110905	235,113256	63,5158029
582,931641	405	285,390298	258,41975	209,526773	75,5957982	220,54586	60,7982962
584,813965	405	275,739028	248,628078	201,950775	75,7123881	220,723198	60,9548395
586,69458	405	264,736879	237,709432	199,502485	74,5297012	209,789436	55,9838559
588,57373	405	257,842119	229,657446	185,273881	69,3287156	204,262117	58,1576264
590,451111	405	255,162144	220,137715	181,070092	64,3162054	203,481807	51,5546215
592,326965	405	239,40984	211,878957	179,044226	64,8613628	197,360609	47,0764011
594,201233	405	232,475276	205,037805	169,007548	58,2937589	190,54936	48,9343031
596,073914	405	228,373105	192,178818	167,138557	61,3274047	179,286428	43,6779504
597,944946	405	221,97138	186,547186	164,625226	57,9313474	174,803217	44,4511064
599,814453	405	212,173683	183,097539	157,814677	56,737154	175,201748	43,5349079
601,682251	405	208,185335	173,504656	154,037979	51,8190001	165,700768	42,7853474
603,548523	405	202,800447	172,808979	150,957101	49,927639	160,891961	41,1601796
605,413147	405	195,50589	163,227816	143,755579	51,2162673	157,62819	38,5927209
607,276245	405	198,113694	154,915275	140,311406	48,5572606	150,366522	35,7988369
609,137695	405	180,318127	151,611975	136,387146	46,7091526	144,650207	32,9313148
610,997437	405	181,886173	144,490056	132,542997	46,1561112	142,272086	34,2841985
612,855713	405	175,249342	135,423021	133,075482	48,2716935	138,123659	33,3305831
614,712341	405	165,76186	133,279774	127,122868	41,6029931	133,069839	33,0976023
616,567322	405	162,940335	128,724237	120,859864	43,6732993	132,888317	28,041932
618,420715	405	157,82192	124,28929	118,928451	38,1384999	121,066505	28,4878473
620,272522	405	152,299807	118,943774	112,271084	38,1382744	116,278933	28,5484201
622,122681	405	146,757523	114,881702	110,639032	35,9385992	118,078745	25,8464497
623,971313	405	139,883008	111,601371	111,758813	35,383235	109,080239	26,976702
625,818237	405	136,003116	105,316218	103,424199	34,7025514	108,772104	24,9558346
627,663574	405	137,228849	102,296863	102,209248	31,2296681	105,015954	25,7373981
629,507324	405	134,103905	100,323589	97,428416	32,0441915	101,153199	24,6286156
631,349365	405	126,016623	96,9269981	95,0214037	29,918935	96,6791187	21,596833
633,18988	405	122,664121	92,5151567	90,8538204	28,0834348	95,7443426	23,6302207
635,028809	405	122,839178	89,5626409	88,4818484	28,5633759	94,8585624	19,6525367
636,865967	405	111,586104	82,9900045	85,804272	29,4381743	89,0354286	19,0222364
638,70166	405	107,247969	79,8834345	82,3676245	25,8866496	88,5032192	17,3776788
640,535645	405	105,402403	82,7715731	79,7815723	25,881895	82,1783248	18,0363723
642,368042	405	106,443478	73,4881095	79,3795344	24,0858852	77,7064085	18,4722882
644,198853	405	101,177082	70,8172915	75,5566079	22,8893392	77,2077521	17,765014
646,028015	405	94,6246796	73,6486353	69,979136	21,7492235	74,8790278	18,0349192
647,855591	405	94,9997281	70,7848499	70,1149021	22,4893013	72,7624066	15,3686297
649,681519	405	90,3142083	63,3777879	68,9835054	21,939832	73,9062563	16,7037208

 This journal is © The Royal Society of Chemistry 20xx

Please do not adjust margins

Please do not adjust margins

651,505798	405	90,5168997	63,3858945	64,3071946	23,9638696	68,7408275	14,4242006
653,328491	405	87,0830703	58,7747367	63,7465316	19,0615039	68,6151285	12,1974191
655,149536	405	84,7739377	57,8033304	58,9850275	18,9614255	64,7985092	14,0990762
656,968994	405	80,2770771	56,5975544	60,2485393	19,2947921	61,5791122	12,0379853
658,786743	405	78,1841893	56,0151575	56,6098626	19,2165336	57,345014	11,3290995
660,602905	405	75,3718228	55,3776479	56,4873253	15,0672399	59,279938	12,1244096
662,41748	405	71,8388529	50,7257882	53,9899949	16,6828149	56,0182068	11,8358079
664,230469	405	69,8254984	46,8631852	51,6845816	13,9584993	55,0078922	11,6872906
666,041748	405	65,9802846	44,4459235	49,7940406	14,4155755	53,2943648	13,1721954
667,85144	405	64,5162798	45,2604233	48,8924974	15,2342262	49,7164644	11,050169
669,659424	405	61,7840242	43,5315162	45,9571192	13,790754	49,7308772	9,08304373
671,46582	405	64,1056613	41,9267339	41,9244991	16,1490751	42,3101226	10,3912352
673,27063	405	58,688563	39,3895233	41,3045108	15,0792313	46,4880062	8,69329304
675,073792	405	58,0152284	36,7586795	42,7809807	11,877021	42,1207665	7,75192998
676,875305	405	56,1697588	36,053341	41,8999546	12,2240273	40,4527338	8,70838244
678,675171	405	54,256553	36,2336908	42,7796679	13,5358953	39,7600842	8,79686405
680,47345	405	52,1198682	34,5484082	39,2574621	11,7803294	37,3721468	7,91983762
682,27002	405	48,3582322	32,7421218	36,9423501	11,033075	39,3416871	7,16128733
684,065063	405	45,3546857	30,3915211	36,0989867	10,6304713	37,4504775	6,74697488
685,858398	405	44,7590205	30,0533422	35,8511989	11,0745994	36,7632903	6,39928657
687,650024	405	41,3706035	31,3146725	32,8082873	12,1413722	32,4859569	4,91391831
689,440186	405	40,9368737	27,8604196	33,4130893	10,40575	34,1493905	6,33249851
691,228577	405	41,8397061	24,7868011	32,8120944	9,08745338	29,9285228	4,92769952
693,015381	405	37,8747912	24,8494441	31,3917525	10,702532	31,6858094	5,5576723
694,800476	405	40,077091	27,1418998	28,9085608	9,10814427	28,9348371	5,46808914
696,583984	405	37,9293322	24,6772065	29,2915255	8,4346218	30,1177358	4,69320039
698,365845	405	34,5619038	22,4321216	28,8397619	8,72542711	24,5895348	6,83758226
700,146118	405	34,0739137	21,5386245	26,0951997	7,5088083	28,4445759	5,43842052
701,924683	405	34,8333452	23,1465913	25,4778354	6,99927004	27,4764225	5,63832253
347,741211	410	0	0	0	0	0	0
349,808533	410	0	0	0	0	0	0
351,874329	410	0	0	0	0	0	0
353,938568	410	0	0	0	0	0	0
356,001343	410	0	0	0	0	0	0
358,062561	410	0	0	0	0	0	0
360,122253	410	0	0	0	0	0	0
362,180481	410	0	0	0	0	0	0
364,237152	410	0	0	0	0	0	0
366,292328	410	0	0	0	0	0	0
368,345978	410	0	0	0	0	0	0
370,398132	410	0	0	0	0	0	0
372,44873	410	0	0	0	0	0	0
374,497803	410	0	0	0	0	0	0
376,545349	410	0	0	0	0	0	0
378,5914	410	0	0	0	0	0	0
380,635925	410	0	0	0	0	0	0
382,678955	410	0	0	0	0	0	0

Please do not adjust margins

Please do not adjust margins

384,720398	410	0	0	0	0	0	0
386,760376	410	0	0	0	0	0	0
388,798767	410	0	0	0	0	0	0
390,835663	410	0	0	0	0	0	0
392,871033	410	0	0	0	0	0	0
394,904877	410	0	0	0	0	0	0
396,937195	410	0	0	0	0	0	0
398,967957	410	0	0	0	0	0	0
400,997253	410	0	0	0	0	0	0
403,024963	410	0	0	0	0	0	0
405,051147	410	0	0	0	0	0	0
407,075806	410	0	0	0	0	0	0
409,098938	410	0	0	0	0	0	0
411,120544	410	0	0	0	0	0	0
413,140564	410	0	0	0	0	0	0
415,159119	410	0	0	0	0	0	0
417,176086	410	0	0	0	0	0	0
419,191528	410	0	0	0	0	0	0
421,205444	410	105,764684	501,076587	73,7050134	188,466584	90,798032	72,9623609
423,217834	410	114,406611	541,484082	86,4918389	166,834143	101,140556	81,2463444
425,228668	410	132,863781	563,437587	94,4978038	160,760803	117,465975	87,4071756
427,237976	410	149,398491	597,000957	104,236336	156,506178	125,37995	91,7260395
429,245728	410	165,017749	618,58245	116,147418	157,848271	140,737244	101,313424
431,251953	410	192,395185	648,515348	127,895424	161,369068	157,705988	110,698359
433,256592	410	207,122868	696,675575	146,161824	175,109715	174,62887	122,474632
435,259766	410	229,102876	722,010643	155,59756	173,242773	191,11895	134,313639
437,261353	410	241,768174	751,961411	168,515503	181,584152	209,597651	144,80463
439,261383	410	267,668727	781,206328	184,641074	198,396529	221,434298	155,961977
441,259888	410	286,442758	816,679151	198,75607	199,292991	240,836405	163,799428
443,256836	410	309,448535	833,217989	210,643424	202,921519	262,857226	174,569153
445,252258	410	320,154654	874,337389	221,01923	208,729772	271,658707	182,002272
447,246094	410	334,770143	885,398502	235,635802	226,196218	284,595049	189,147668
449,238434	410	356,551227	903,367666	248,154794	230,988212	303,167857	196,593648
451,229187	410	374,142123	927,755652	255,224575	236,027694	319,950285	199,284853
453,218445	410	392,576265	931,541925	271,548216	246,535986	331,690189	206,538647
455,206116	410	405,585064	955,014942	290,717234	256,476049	341,064403	211,98001
457,192261	410	421,1675	972,269587	293,528331	258,525831	360,51634	217,420902
459,176819	410	442,761378	992,019113	318,095366	272,37708	376,329897	226,36114
461,15979	410	460,924162	998,180645	323,191992	272,450962	389,324405	235,690896
463,141296	410	478,069145	1022,0071	330,704467	282,334191	405,612479	237,450122
465,121216	410	486,866459	1008,97191	346,302564	287,299446	417,693539	249,571782
467,099579	410	516,325851	1030,80216	358,561355	286,808327	427,68922	249,880771
469,076385	410	527,595758	1037,35491	371,408258	291,552022	449,964363	255,991016
471,051636	410	538,863922	1052,33817	377,375851	289,232652	456,380307	257,686997
473,02536	410	557,770508	1042,83821	395,814019	290,3289	471,969309	263,40639
474,997498	410	568,333154	1053,35107	401,275851	283,474331	482,194575	263,710199
476,968079	410	576,848265	1045,20788	405,632529	277,663863	482,00912	259,684789

This journal is © The Royal Society of Chemistry 20xx

Please do not adjust margins

478,937134	410	583,198308	1040,7745	409,916092	282,748818	492,293193	262,031563
480,904572	410	598,737716	1040,92803	417,406397	274,975455	493,95663	263,343258
482,870453	410	604,684525	1045,00355	424,689512	271,316828	505,34023	258,31505
484,834808	410	616,634389	1039,42228	434,888967	274,821347	519,502348	258,633353
486,797607	410	622,030323	1029,90389	447,015062	273,61563	523,520544	252,490138
488,75885	410	616,025387	1025,90224	442,206766	265,251598	527,802609	252,47214
490,718506	410	635,792631	1010,34257	455,576056	267,813834	531,38099	250,728735
492,676605	410	629,570562	1013,77641	449,093186	259,308805	538,163841	250,580551
494,633118	410	643,038237	992,039586	454,949937	264,434682	537,426	245,808879
496,588104	410	640,226176	984,06739	456,429267	262,422045	545,257911	247,743573
498,541504	410	643,620587	969,971011	454,431569	255,340121	535,95882	236,818252
500,493347	410	641,314753	941,788001	456,640426	250,852657	532,29947	235,951218
502,443665	410	633,627726	939,791187	451,4839	248,238978	532,611185	236,811758
504,392365	410	631,969393	913,884514	451,79008	244,635654	534,561005	228,977354
506,339539	410	634,514229	904,895392	450,623647	240,540113	530,637342	224,066626
508,285095	410	625,834611	875,42817	447,619419	237,198013	531,917204	222,610725
510,229126	410	618,003981	864,635263	447,227555	232,178008	513,423466	217,258984
512,17157	410	619,13658	835,654198	441,640726	225,51555	509,693547	209,710818
514,112427	410	615,6665	817,824542	432,092858	220,674674	507,935132	199,382365
516,051697	410	606,634424	776,103962	429,952674	212,506485	501,15613	196,171391
517,989441	410	588,828654	765,170479	420,739223	209,912643	500,141349	189,730305
519,925598	410	583,148512	747,019945	418,922896	202,669932	483,41589	183,614901
521,860168	410	569,21135	721,330191	416,054753	204,480412	471,022094	176,432584
523,793152	410	568,205664	701,141108	403,868072	194,988865	465,754964	172,948239
525,724609	410	560,850871	680,554154	389,659495	189,788975	460,180118	168,626439
527,654419	410	548,656362	673,030443	393,161516	184,927759	453,819679	162,909112
529,582764	410	545,71539	645,540317	389,596255	179,498291	447,03219	157,319498
531,50946	410	540,69925	632,584519	380,747007	177,975858	443,222239	158,126723
533,43457	410	534,946966	614,674778	375,407492	171,884492	446,073267	148,196658
535,358093	410	524,634568	602,354223	368,295456	172,836941	427,440316	146,32472
537,280029	410	513,240434	565,264284	361,876169	166,268289	419,736521	145,049131
539,200439	410	503,74923	566,606012	358,608311	158,553906	413,480935	140,852281
541,119263	410	484,85775	546,552511	352,980187	157,927316	400,466239	129,662714
543,036438	410	482,268079	522,103316	351,244708	151,619893	388,839427	130,294765
544,952087	410	472,010711	501,827863	337,769558	144,913044	385,570844	126,923025
546,866211	410	461,884314	491,218934	324,192042	141,383487	382,828981	120,112393
548,778687	410	446,630138	472,9273	321,890053	139,318547	373,122457	115,416521
550,689514	410	439,09265	461,563087	315,891334	137,486433	359,214929	116,400711
552,598816	410	434,007952	444,174317	309,825576	128,451281	353,91087	109,759449
554,506531	410	415,3204	424,513249	302,040466	125,77173	336,188677	105,997297
556,41272	410	409,198183	414,326777	293,705669	121,22786	335,961395	104,43726
558,317261	410	398,967437	395,953357	283,724719	118,6785	329,333775	99,7759343
560,220215	410	390,686665	382,514532	281,361636	114,100608	320,629968	94,9073952
562,121582	410	380,627941	373,712629	278,132931	108,5572	304,394586	92,9088643
564,021362	410	372,62015	358,69313	269,23191	103,777569	294,203166	86,3604751
565,919556	410	363,407886	351,578199	261,6217	103,978545	294,650846	88,2419699
567,816162	410	347,399387	333,664346	254,731705	101,853787	284,463902	81,7246197

Please do not adjust margins
Please do not adjust margins

Please do not adjust margins

569,711182	410	347,669379	321,456077	249,44288	92,7960805	277,126427	78,1528725
571,604614	410	334,992411	314,872129	241,350699	92,6187965	271,014033	75,0136549
573,49646	410	325,882094	303,121037	233,042508	91,3254672	260,031712	74,3402841
575,386719	410	320,60581	291,364391	230,581483	88,3761926	254,657504	70,5972007
577,27533	410	313,682943	283,3288	222,29502	85,1745846	250,483505	68,0528274
579,162354	410	306,143696	268,483173	220,467551	81,2050545	246,175353	65,5427219
581,047852	410	296,99522	258,755076	211,154255	79,2667163	228,787699	64,2228741
582,931641	410	287,503683	251,788974	202,829718	74,4215759	225,532883	61,4998067
584,813965	410	278,727176	239,37887	199,406329	72,791511	220,952214	55,2117316
586,69458	410	269,023024	231,414071	193,990576	72,4002166	218,187346	57,2785725
588,57373	410	255,602946	226,107187	191,400434	68,6430553	208,528152	52,8348004
590,451111	410	256,281075	212,467553	184,017875	67,7176541	200,447675	50,2251406
592,326965	410	239,439923	201,056711	173,005045	65,1040005	190,313392	47,2663548
594,201233	410	235,652884	196,562549	172,051351	60,5456325	185,334796	49,4030755
596,073914	410	225,912084	189,187205	167,753185	59,955007	181,607244	47,4644383
597,944946	410	220,621517	178,317176	157,463092	55,8598883	172,569025	41,6422849
599,814453	410	212,882906	178,842757	155,50902	52,5654864	167,732973	42,4122379
601,682251	410	208,743007	166,353285	152,620155	52,9148546	159,373249	39,6333638
603,548523	410	203,322808	165,681721	146,078444	49,2603072	157,536387	39,3124987
605,413147	410	197,30467	155,028799	142,453891	50,1980031	155,226744	39,0888404
607,276245	410	188,192901	149,301134	138,708759	49,6510358	148,715748	37,8538098
609,137695	410	184,67623	148,282958	138,41228	44,7957632	145,66254	35,7597911
610,997437	410	181,185266	137,980808	132,097207	43,0796775	139,124257	33,9489117
612,855713	410	179,046645	141,250224	126,495406	41,8394028	135,642576	31,9220327
614,712341	410	169,976884	133,915979	120,227323	42,2671203	131,836969	32,0867994
616,567322	410	169,92218	126,41489	120,986474	41,4457399	129,929819	27,4762489
618,420715	410	161,703154	122,023048	118,640222	38,9026858	125,465354	28,3839628
620,272522	410	154,035846	116,666682	113,200822	37,3247675	114,186913	28,0492548
622,122681	410	146,259726	112,399769	110,275785	34,7107169	115,304562	28,030484
623,971313	410	146,102108	112,866385	108,035607	33,9316073	109,898752	26,3604307
625,818237	410	138,51681	101,901029	103,836583	34,3846989	110,332678	24,8702649
627,663574	410	135,714142	101,7604	98,5065742	32,7668389	105,971294	24,0356044
629,507324	410	135,09368	95,6744525	97,6269079	31,8841627	102,477662	24,3947266
631,349365	410	125,599927	91,0019331	93,4406048	30,0591073	98,69539	23,5293515
633,18988	410	120,47933	87,6689997	92,1286942	26,4235574	95,6229604	22,4574017
635,028809	410	117,419228	84,4758478	85,7957965	27,1323404	95,6462223	19,6284065
636,865967	410	117,20207	85,9011066	84,7234441	28,7077684	88,1380875	19,3374204
638,70166	410	109,416374	80,0570447	81,2211413	24,2052416	86,6535727	19,7511375
640,535645	410	107,292503	78,6706474	78,948255	25,0881584	80,9102143	17,3294474
642,368042	410	107,745869	74,3222882	77,0249375	24,4722646	82,1384508	15,6556507
644,198853	410	98,8854116	70,92042	77,5956786	25,9010552	75,9214233	15,6894265
646,028015	410	95,9112677	70,3541312	71,7720476	20,744842	74,1455828	15,1408282
647,855591	410	93,1880003	65,1380689	69,068432	23,0036873	73,8699795	17,400248
649,681519	410	90,0931754	62,4732359	67,8916593	21,9868335	72,2298258	15,2965903
651,505798	410	87,9207224	59,7037456	65,0644137	20,1147255	67,5387589	13,970533
653,328491	410	86,3818295	60,4620464	62,1493077	18,5363439	67,0907996	15,4067612
655,149536	410	83,9708008	57,6351779	59,7417519	17,8628034	65,7704309	12,9130073

 This journal is © The Royal Society of Chemistry 20xx

Please do not adjust margins

Please do not adjust margins

656,968994	410	78,3484537	55,8891587	60,649702	16,6967726	60,5869599	12,3882592
658,786743	410	75,6419568	53,0835575	57,0384394	16,6938115	59,6317996	13,1115609
660,602905	410	75,7378019	53,4592293	55,4260118	16,1506379	58,3579454	11,8117476
662,41748	410	70,9614269	46,1714284	53,8358823	17,8956836	56,8640064	12,5332482
664,230469	410	67,0673243	47,7710954	52,1831301	15,9200428	53,4570924	10,130103
666,041748	410	66,9371193	47,1865756	51,187476	15,9490945	51,6239904	11,2389332
667,85144	410	62,984101	44,9588941	50,4025977	13,8044306	48,5673588	9,92417736
669,659424	410	62,6047061	39,5667483	47,4698268	12,9047393	44,7020764	10,2814767
671,46582	410	60,6362184	40,6206079	44,3110846	13,7622433	45,2543615	10,5450708
673,27063	410	57,8981367	35,8624617	44,5694433	12,0361713	44,4993169	9,40071435
675,073792	410	60,4799383	36,3562445	42,3630799	12,8236743	44,0754661	8,57027518
676,875305	410	53,314456	35,3302114	41,3364904	12,0570631	41,6047862	8,22929794
678,675171	410	53,2377484	32,2779181	39,0509393	12,0589793	40,7621859	7,38208969
680,47345	410	46,9836896	34,9693462	37,2397324	10,5223385	37,9308111	7,29166814
682,27002	410	45,0966545	32,1947056	38,475372	9,70728533	37,0404475	8,42298446
684,065063	410	50,0454237	30,5071751	34,674607	11,106505	36,3092283	6,91443893
685,858398	410	46,552358	30,9480066	33,4081635	10,0002362	34,1883713	7,00287073
687,650024	410	41,5354025	27,4701069	33,8689894	11,3790824	30,8856559	6,66788447
689,440186	410	43,0076547	28,305573	32,0613163	9,60453448	32,7578722	6,85466359
691,228577	410	41,0494862	25,916631	30,9621093	8,66549273	31,4012544	6,08650661
693,015381	410	38,564976	26,4122971	29,5786246	9,80557204	33,6309291	5,32840999
694,800476	410	40,1941611	26,2336758	28,8835334	7,99728442	27,9471148	6,01444412
696,583984	410	36,9789231	24,0078592	27,7054627	7,7705442	27,8008978	4,83097527
698,365845	410	36,7354498	25,4609486	29,0815977	7,87896059	25,974238	7,35333451
700,146118	410	35,051141	21,3021994	25,8956229	9,04049035	26,7733915	5,73245223
701,924683	410	35,2734199	21,6459404	25,7334805	7,85357821	26,4400161	4,27227308
347,741211	415	0	0	0	0	0	0
349,808533	415	0	0	0	0	0	0
351,874329	415	0	0	0	0	0	0
353,938568	415	0	0	0	0	0	0
356,001343	415	0	0	0	0	0	0
358,062561	415	0	0	0	0	0	0
360,122253	415	0	0	0	0	0	0
362,180481	415	0	0	0	0	0	0
364,237152	415	0	0	0	0	0	0
366,292328	415	0	0	0	0	0	0
368,345978	415	0	0	0	0	0	0
370,398132	415	0	0	0	0	0	0
372,44873	415	0	0	0	0	0	0
374,497803	415	0	0	0	0	0	0
376,545349	415	0	0	0	0	0	0
378,5914	415	0	0	0	0	0	0
380,635925	415	0	0	0	0	0	0
382,678955	415	0	0	0	0	0	0
384,720398	415	0	0	0	0	0	0
386,760376	415	0	0	0	0	0	0
388,798767	415	0	0	0	0	0	0

Please do not adjust margins

390,835663	415	0	0	0	0	0	0
392,871033	415	0	0	0	0	0	0
394,904877	415	0	0	0	0	0	0
396,937195	415	0	0	0	0	0	0
398,967957	415	0	0	0	0	0	0
400,997253	415	0	0	0	0	0	0
403,024963	415	0	0	0	0	0	0
405,051147	415	0	0	0	0	0	0
407,075806	415	0	0	0	0	0	0
409,098938	415	0	0	0	0	0	0
411,120544	415	0	0	0	0	0	0
413,140564	415	0	0	0	0	0	0
415,159119	415	0	0	0	0	0	0
417,176086	415	0	0	0	0	0	0
419,191528	415	0	0	0	0	0	0
421,205444	415	0	0	0	0	0	0
423,217834	415	0	0	0	0	0	0
425,228668	415	98,1792225	356,661173	76,0821068	174,091867	85,9308083	61,2095118
427,237976	415	108,163856	381,094797	82,7627866	155,539037	94,229285	70,3414643
429,245728	415	121,066714	404,296392	85,139901	143,074481	104,422305	72,8160704
431,251953	415	130,414288	436,178709	97,2995943	144,189897	115,596907	85,1057191
433,256592	415	153,744216	464,280833	109,279496	144,144966	133,610062	92,1473173
435,259766	415	172,038801	499,935173	119,958674	150,52914	150,883249	98,474592
437,261353	415	188,8213	525,849878	130,963601	155,339822	162,805075	111,646746
439,261383	415	207,227376	553,220666	142,046862	162,438853	177,578368	124,21075
441,259888	415	224,522961	579,51312	152,18847	168,257108	192,32478	128,005988
443,256836	415	236,487061	613,001166	166,515053	171,603737	204,800377	139,431902
445,252258	415	255,755126	631,157175	181,732232	170,752619	220,007678	147,535751
447,246094	415	269,215225	651,646177	189,082917	182,823935	239,61457	152,578999
449,238434	415	287,176325	677,584622	202,096845	188,928732	250,912534	156,893368
451,229187	415	313,223328	702,464374	210,835297	193,840741	262,779717	170,064934
453,218445	415	325,522652	732,880591	224,594678	201,42297	277,654753	173,22736
455,206116	415	342,506938	744,669279	239,203658	213,61539	289,158603	181,867305
457,192261	415	358,781953	765,880349	254,972496	221,75081	307,105923	186,377642
459,176819	415	378,82239	793,378578	263,211669	226,589442	325,532879	198,287943
461,15979	415	399,097036	806,357323	276,475428	237,790975	338,52742	203,23092
463,141296	415	410,982163	812,967385	291,074986	244,953381	358,425464	207,723736
465,121216	415	426,582505	826,006422	295,648478	248,108785	365,24826	209,195333
467,099579	415	445,284212	846,166049	309,715599	257,90264	376,791829	215,052384
469,076385	415	457,068297	854,216165	323,304493	260,365263	394,882525	218,90764
471,051636	415	471,312832	870,978915	328,134517	260,769582	406,831145	223,509886
473,02536	415	496,558665	889,186386	345,53388	264,227502	409,97639	229,417832
474,997498	415	507,963659	872,241411	360,286667	260,190356	430,372301	231,924575
476,968079	415	526,408559	890,954834	368,747401	267,166327	443,761688	239,776275
478,937134	415	545,807394	888,391711	377,500699	261,567779	453,191062	234,118723
480,904572	415	554,194054	900,058866	386,456048	260,16784	463,582641	237,070546
482,870453	415	560,111053	894,918624	396,222541	259,355686	470,522601	234,79492

This journal is © The Royal Society of Chemistry 20xx

484,834808	415	569,673864	893,811348	399,526592	249,807609	475,287895	240,97019
486,797607	415	582,919688	895,762492	409,322565	249,099877	483,854103	231,935495
488,75885	415	583,866601	895,085895	416,108091	246,606206	495,442838	232,459141
490,718506	415	588,144712	879,212144	417,313908	248,237083	491,490238	233,865948
492,676605	415	602,222258	887,057716	419,25279	247,125816	499,54771	228,412761
494,633118	415	600,700966	877,659629	421,79738	243,41242	503,172753	229,800993
496,588104	415	607,474785	868,92884	423,09854	235,974862	511,430093	226,885672
498,541504	415	609,431168	852,071178	424,36215	239,136576	514,536693	225,114002
500,493347	415	605,902019	846,843225	425,232738	233,908612	505,148801	218,594967
502,443665	415	604,452416	829,880748	423,849097	231,111054	505,053488	218,410142
504,392365	415	608,317955	813,17262	429,082434	224,946248	510,276158	215,572831
506,339539	415	611,586679	803,195048	423,311171	225,555349	506,154244	203,938323
508,285095	415	600,234303	787,869562	425,768422	219,177374	499,356434	204,870762
510,229126	415	597,729272	759,943677	418,423929	214,848414	494,135656	202,168575
512,17157	415	584,296003	758,372322	414,825823	210,491947	498,680204	195,452256
514,112427	415	589,21921	727,012888	414,404948	208,888045	486,309277	190,66737
516,051697	415	571,803356	713,294426	407,355686	202,323054	483,027418	185,287634
517,989441	415	579,021019	700,223593	399,261369	196,00338	473,244121	181,76888
519,925598	415	570,168443	670,374769	396,765189	193,980235	461,671706	171,914234
521,860168	415	560,252419	662,463475	390,846597	188,940787	461,634673	173,154637
523,793152	415	548,329097	639,295347	389,454669	183,444515	454,690479	165,149105
525,724609	415	545,811661	623,152859	378,031805	182,004494	446,52967	154,706607
527,654419	415	536,582524	607,90817	376,534294	177,995716	436,880816	158,205565
529,582764	415	533,22658	605,693562	373,427236	173,439142	442,331992	152,774811
531,50946	415	517,217407	580,264829	372,983937	169,426305	431,988876	151,897653
533,43457	415	516,119271	563,806472	373,741938	163,643339	428,092566	146,082704
535,358093	415	516,468167	543,13091	357,203342	157,065856	416,425256	139,061593
537,280029	415	511,649915	528,280453	352,838884	157,922071	404,756034	136,613515
539,200439	415	498,284081	525,644529	349,379015	150,200135	404,292907	132,022138
541,119263	415	487,325003	500,878363	339,242727	151,904884	395,653046	129,850367
543,036438	415	473,738705	495,435112	330,161284	146,636526	384,996399	122,930532
544,952087	415	463,259503	472,145795	329,198544	141,122896	378,925036	120,671104
546,866211	415	457,544832	459,847876	318,49358	133,917954	369,473419	115,346415
548,778687	415	443,456666	446,707884	315,759029	131,199564	363,454094	115,192794
550,689514	415	437,194887	435,263304	306,44674	127,0092	348,578724	109,470787
552,598816	415	428,399468	418,406574	301,830296	121,222665	342,359164	104,443875
554,506531	415	421,830869	404,627318	292,496949	123,296803	338,95204	106,310397
556,41272	415	407,309487	382,54045	287,941444	119,519184	328,806519	95,3077794
558,317261	415	399,513212	370,575072	277,67883	113,213454	314,276434	96,9392856
560,220215	415	382,109174	363,685753	274,887596	108,622203	311,558147	90,2261672
562,121582	415	373,423216	349,01203	262,170424	107,208705	300,95988	87,7127085
564,021362	415	367,937649	342,086304	266,236286	103,205611	295,894793	84,8288274
565,919556	415	368,476488	328,715078	258,937133	99,6958695	291,085788	80,8790625
567,816162	415	361,03063	315,790904	249,249878	96,9100514	280,835339	78,2556198
569,711182	415	339,480035	302,394878	238,153413	93,206399	274,506234	75,4999537
571,604614	415	334,568763	291,04437	238,502722	93,1199298	263,887305	76,8497699
573,49646	415	321,332059	280,383241	232,905146	87,5973053	260,168579	71,2135745

Please do not adjust margins

575,386719	415	317,901384	272,84529	227,628987	86,0355908	255,036869	68,9530195
577,27533	415	311,734028	267,029577	220,036928	83,6554487	250,287663	65,9721166
579,162354	415	302,019711	252,878095	218,786319	77,9682199	237,833911	63,8989138
581,047852	415	290,880021	247,221231	213,286872	76,5955514	231,005701	61,3680797
582,931641	415	281,523689	239,424949	201,670958	76,1542816	225,264286	59,1633963
584,813965	415	276,634609	229,717057	196,588158	71,1865707	220,973145	57,4736267
586,69458	415	265,311596	224,339023	193,627508	66,6642152	210,678306	53,6547088
588,57373	415	258,345574	205,650327	180,362459	66,1915881	205,445268	52,7441605
590,451111	415	253,310754	206,754319	180,563932	66,6382999	194,984385	51,9454387
592,326965	415	236,186413	195,750417	173,751454	63,8037571	195,074419	48,1201778
594,201233	415	237,219287	187,105261	168,897961	58,7660273	183,276254	45,7820437
596,073914	415	228,216768	176,831413	160,730509	56,1875146	174,892943	46,3459753
597,944946	415	223,846622	176,363659	163,739997	55,1356628	173,425124	43,9196557
599,814453	415	215,238182	167,190092	157,747715	53,3147214	168,017598	42,7411408
601,682251	415	208,131271	165,034162	152,09206	48,7439629	162,56106	38,2003644
603,548523	415	202,094612	158,640456	143,818452	47,3393252	158,692758	39,7226304
605,413147	415	199,687702	149,746835	140,065272	47,7934683	154,375177	36,528595
607,276245	415	184,575249	143,041462	138,557332	47,0347022	148,877816	37,0060512
609,137695	415	184,361122	139,240337	131,798869	43,6879268	146,130698	35,3513283
610,997437	415	178,617038	135,551685	125,811222	41,4865796	141,994671	33,4407872
612,855713	415	177,383429	129,576043	128,372667	42,4171702	134,566251	32,0051179
614,712341	415	169,436144	127,172641	124,05638	39,3952839	129,342322	28,0418273
616,567322	415	164,940432	118,275748	116,843727	42,7304025	126,186625	28,4543694
618,420715	415	159,346794	117,069771	115,406388	35,967822	121,154729	30,7177154
620,272522	415	155,068193	111,556478	108,586724	37,4276626	117,721524	28,633591
622,122681	415	144,450754	103,866259	111,514173	34,8896254	113,505732	26,4642621
623,971313	415	144,304794	102,220408	108,192406	34,4445129	111,652892	25,6881402
625,818237	415	137,654133	102,313974	102,963939	31,7117271	106,896318	24,2406447
627,663574	415	133,929547	96,8256027	97,4017198	30,3693907	105,452313	23,740179
629,507324	415	128,414669	92,6207503	95,4636725	31,8458837	99,1776478	22,8435714
631,349365	415	124,989582	89,4093688	96,080386	27,9637966	95,7339853	21,7681014
633,18988	415	124,105469	82,7304706	89,7331822	27,4810416	94,3908858	20,7240766
635,028809	415	114,393876	84,0107194	86,0047131	28,7165361	91,5943724	19,8519396
636,865967	415	115,282609	80,9324754	88,0996471	25,7058466	86,4869381	18,709372
638,70166	415	109,614031	78,2333225	83,7602727	25,6415664	82,6902589	19,2679339
640,535645	415	108,808842	76,4163123	80,454631	23,3632518	80,0865728	15,6621097
642,368042	415	102,180364	73,0557287	76,2377385	22,998938	79,4762955	17,5627056
644,198853	415	101,061723	68,4949731	74,2799319	22,1045883	76,4209235	15,7148177
646,028015	415	96,4471026	67,4720729	72,4772998	22,0514336	73,5940349	15,8116829
647,855591	415	93,6457602	61,6975963	67,9618306	20,9396484	74,8218245	16,8269251
649,681519	415	92,4038437	60,4456911	69,1166317	21,0517979	72,8471094	14,8617053
651,505798	415	85,0646799	58,2312408	66,4624997	21,0587946	65,6726626	14,2877348
653,328491	415	85,7259851	55,3826217	61,9679953	18,413496	66,5928633	13,2170825
655,149536	415	86,5179463	56,7833876	62,1130867	18,080355	63,4729442	13,6646297
656,968994	415	73,361561	50,513199	58,2760346	19,2797339	61,4044851	11,7953374
658,786743	415	74,4409044	52,7728151	57,1844427	16,6278194	60,164636	10,078162
660,602905	415	74,4553699	50,8758481	54,4095614	14,8872474	57,1502619	13,0922665

This journal is © The Royal Society of Chemistry 20xx

Please do not adjust margins

Please do not adjust margins

662,41748	415	73,8132569	47,0420401	54,004304	16,0106652	54,3984104	11,44631
664,230469	415	67,1908917	42,413237	51,5070349	13,1921104	53,9354358	10,8181785
666,041748	415	67,803652	44,2848651	48,9109267	16,7241835	53,8706157	12,2232181
667,85144	415	65,2049069	44,3248221	48,3096998	13,9887655	46,4069772	10,9494252
669,659424	415	61,9722576	38,1871094	48,8175829	13,0335751	46,5014435	9,57978004
671,46582	415	61,2961247	39,2105189	43,7839656	14,1956853	44,2502742	9,01583978
673,27063	415	58,4017468	38,8700483	44,0378399	13,1804012	44,0939872	9,87400028
675,073792	415	55,2909705	34,57185	39,7516152	11,5550598	43,1879703	6,84266844
676,875305	415	55,0325938	33,4103696	39,3317961	11,1402668	40,0424371	8,65474671
678,675171	415	51,0743963	33,6632027	38,183471	11,4721716	36,7458068	6,59514067
680,47345	415	48,9708721	32,5657244	40,5441215	11,2984738	38,5330189	7,74337529
682,27002	415	47,2346131	33,6713414	37,2942343	9,02044729	36,1826152	6,94359053
684,065063	415	46,3938941	29,134948	34,7632441	10,1298386	35,8875148	6,30317739
685,858398	415	46,2478716	27,9020903	35,2765038	11,5464327	35,7370274	6,72080489
687,650024	415	44,9616174	28,0960566	33,649827	10,310647	32,7776186	6,06303863
689,440186	415	41,5604463	28,244316	33,8941632	9,75108159	31,0189298	6,7430315
691,228577	415	38,2410606	26,1742714	33,2442848	8,83865913	30,7839133	6,32990735
693,015381	415	36,3287434	25,3211705	27,5647611	8,94459112	32,1998898	5,26042103
694,800476	415	35,9012399	25,4819406	27,8084022	8,69163668	28,9308548	5,42606288
696,583984	415	34,6177394	22,9004828	26,2437247	8,64280634	27,1167115	4,27455564
698,365845	415	36,4002064	21,6189066	27,1582804	7,99391954	27,3612838	5,46327127
700,146118	415	34,7690667	19,7627588	24,9874863	8,61722537	26,7943659	4,89438934
701,924683	415	34,4781266	18,6518026	25,6767411	8,56554633	26,3882765	5,67559946
347,741211	420	0	0	0	0	0	0
349,808533	420	0	0	0	0	0	0
351,874329	420	0	0	0	0	0	0
353,938568	420	0	0	0	0	0	0
356,001343	420	0	0	0	0	0	0
358,062561	420	0	0	0	0	0	0
360,122253	420	0	0	0	0	0	0
362,180481	420	0	0	0	0	0	0
364,237152	420	0	0	0	0	0	0
366,292328	420	0	0	0	0	0	0
368,345978	420	0	0	0	0	0	0
370,398132	420	0	0	0	0	0	0
372,44873	420	0	0	0	0	0	0
374,497803	420	0	0	0	0	0	0
376,545349	420	0	0	0	0	0	0
378,5914	420	0	0	0	0	0	0
380,635925	420	0	0	0	0	0	0
382,678955	420	0	0	0	0	0	0
384,720398	420	0	0	0	0	0	0
386,760376	420	0	0	0	0	0	0
388,798767	420	0	0	0	0	0	0
390,835663	420	0	0	0	0	0	0
392,871033	420	0	0	0	0	0	0
394,904877	420	0	0	0	0	0	0

This journal is © The Royal Society of Chemistry 20xx

Please do not adjust margins

396,937195	420	0	0	0	0	0	0
398,967957	420	0	0	0	0	0	0
400,997253	420	0	0	0	0	0	0
403,024963	420	0	0	0	0	0	0
405,051147	420	0	0	0	0	0	0
407,075806	420	0	0	0	0	0	0
409,098938	420	0	0	0	0	0	0
411,120544	420	0	0	0	0	0	0
413,140564	420	0	0	0	0	0	0
415,159119	420	0	0	0	0	0	0
417,176086	420	0	0	0	0	0	0
419,191528	420	0	0	0	0	0	0
421,205444	420	0	0	0	0	0	0
423,217834	420	0	0	0	0	0	0
425,228668	420	0	0	0	0	0	0
427,237976	420	0	0	0	0	0	0
429,245728	420	0	0	0	0	0	0
431,251953	420	99,5457969	272,025414	77,4293236	153,426557	94,9941081	60,4964137
433,256592	420	106,718375	297,596385	82,9446006	134,074052	100,671625	68,2649576
435,259766	420	123,109304	317,977709	88,9979411	128,010371	109,926077	73,625612
437,261353	420	138,127089	350,657226	99,5098541	130,597845	128,098304	81,2507051
439,261383	420	152,296873	372,459042	109,196358	136,784968	139,578086	89,5594078
441,259888	420	168,121811	406,375984	122,464237	137,681129	148,745145	103,845289
443,256836	420	185,608246	424,30853	130,028443	141,817382	160,365283	110,064604
445,252258	420	202,215768	453,049539	147,984977	146,184196	179,142888	116,4043
447,246094	420	212,884027	475,656568	151,816593	154,88408	188,527859	125,509444
449,238434	420	230,105773	492,653266	162,128689	158,604242	204,818781	130,394614
451,229187	420	249,267305	526,180426	176,78677	163,868367	217,704699	136,158963
453,218445	420	264,368529	545,589397	187,568476	166,389006	232,608699	149,486794
455,206116	420	277,828258	574,720652	200,067394	179,33346	243,261897	149,894932
457,192261	420	297,859377	583,568053	207,103429	182,132379	260,39429	155,884954
459,176819	420	318,57421	611,538216	220,763496	189,539446	276,841377	160,919843
461,15979	420	327,108745	629,184655	230,204535	197,322637	282,745609	171,388593
463,141296	420	357,352289	645,080579	248,175441	207,435054	305,268412	180,230525
465,121216	420	369,133549	665,275998	258,761596	212,1732	316,471306	183,930305
467,099579	420	378,919725	678,968999	272,785175	216,319033	329,294338	190,22041
469,076385	420	391,76199	693,166038	283,432159	226,544573	337,929101	193,174639
471,051636	420	413,373408	708,145196	285,93867	232,807123	360,85071	195,636787
473,02536	420	432,85641	725,425854	302,357675	235,702531	374,490089	201,044405
474,997498	420	445,195528	727,231139	313,512698	238,18525	383,354391	209,016231
476,968079	420	460,033225	740,97565	325,808922	238,995362	385,172189	211,151468
478,937134	420	472,836535	746,915784	336,351949	243,668835	407,149694	215,624738
480,904572	420	486,819317	754,524801	344,535501	238,049862	412,49094	218,094707
482,870453	420	504,891454	762,454763	358,218487	235,920518	428,032446	219,080875
484,834808	420	528,529526	770,767315	369,209741	238,060839	430,719731	219,341461
486,797607	420	527,901735	768,990025	372,910312	233,881564	455,943231	217,367237
488,75885	420	543,633042	770,523177	373,652468	228,257672	448,691096	220,620107

This journal is © The Royal Society of Chemistry 20xx

Please do not adjust margins

490,718506	420	549,189002	775,667329	384,818624	228,461599	456,69232	225,073255
492,676605	420	555,703097	765,911812	382,597195	227,215514	465,974812	213,518014
494,633118	420	552,979757	757,86806	391,628715	224,379184	470,921071	215,242987
496,588104	420	564,292183	758,211475	402,105897	221,49254	467,354054	209,189908
498,541504	420	571,98778	744,654787	401,616023	218,735445	475,491203	209,390298
500,493347	420	565,204971	740,494123	396,130324	220,302388	473,0546	206,367345
502,443665	420	570,523868	734,835026	401,612147	212,795018	477,20818	205,690457
504,392365	420	577,697799	721,169001	403,032125	211,171248	475,610421	201,369723
506,339539	420	577,726838	709,077494	394,155937	202,835193	475,770816	195,584554
508,285095	420	571,980557	686,717571	390,279074	204,117313	474,313205	193,147875
510,229126	420	568,383686	676,151826	391,856518	199,739632	473,942347	190,730972
512,17157	420	566,219896	672,180906	393,437201	199,664658	467,615732	189,745649
514,112427	420	561,764896	657,995671	391,631789	191,668552	463,526068	183,302428
516,051697	420	551,617991	634,297386	386,058113	190,607013	456,727829	175,890828
517,989441	420	552,664938	618,431012	381,002788	186,965624	452,384498	168,529514
519,925598	420	542,454749	606,981391	375,697657	180,713557	446,130517	164,643713
521,860168	420	540,347459	593,213497	371,306513	175,505455	445,932085	164,305342
523,793152	420	528,289707	579,52566	366,749793	169,724912	435,055378	157,68608
525,724609	420	527,249711	559,825351	362,09962	164,5123	425,595499	149,894917
527,654419	420	513,447551	555,019329	361,634055	162,727164	420,777561	146,907674
529,582764	420	513,623478	536,378516	360,664004	163,676376	408,869209	146,810506
531,50946	420	510,9898	523,216449	355,760614	156,171	412,23032	139,406579
533,43457	420	495,452588	512,868676	350,185673	156,033694	413,07875	139,370608
535,358093	420	498,968602	501,111108	349,993975	152,993008	397,48038	131,47949
537,280029	420	486,680727	479,124903	336,443291	148,778589	394,790725	126,255355
539,200439	420	477,88663	476,473046	338,092867	148,216012	387,946048	130,05342
541,119263	420	471,729029	462,974471	331,407057	137,742482	376,651202	122,811922
543,036438	420	465,197619	444,901795	320,740819	139,640741	373,126109	121,13414
544,952087	420	452,438939	439,028739	314,811482	132,320451	363,668054	114,034238
546,866211	420	442,080314	425,681358	309,883029	128,303048	359,186262	112,484242
548,778687	420	433,983431	408,063244	303,80217	123,934612	350,224776	106,485437
550,689514	420	425,452217	392,990241	303,994098	121,357799	346,874466	104,480631
552,598816	420	420,411805	379,35733	297,251884	115,080307	340,754122	101,703812
554,506531	420	406,510176	370,99058	288,734495	116,047433	330,231908	100,509078
556,41272	420	398,792906	360,966509	275,042834	110,334177	325,617316	96,8913283
558,317261	420	392,620311	355,346646	279,992963	108,507577	314,702767	92,0156541
560,220215	420	382,416689	334,978218	265,411275	103,30478	300,228316	88,1669787
562,121582	420	368,549319	325,460163	258,962233	99,3033254	298,253899	82,3023531
564,021362	420	360,787038	308,066196	251,854133	97,8525841	289,607679	82,7361478
565,919556	420	358,808565	304,953787	246,504108	92,1018723	283,259758	81,9013316
567,816162	420	347,574756	296,257216	243,198449	90,5939501	279,848208	80,8431006
569,711182	420	331,637073	287,749446	235,357636	88,3526266	270,801943	73,1393638
571,604614	420	326,895537	276,945471	232,690967	86,3327043	260,403448	71,8801996
573,49646	420	326,953442	267,913849	222,858207	83,6079018	255,620964	72,43775
575,386719	420	303,244113	256,733023	218,996361	81,3502603	248,922889	66,744987
577,27533	420	309,871143	251,770863	215,586066	77,4632834	242,448376	63,9665668
579,162354	420	297,232494	242,490417	206,919193	77,6932244	238,74937	61,1447335

This journal is © The Royal Society of Chemistry 20xx

Please do not adjust margins

581,047852	420	291,326599	230,176689	210,086172	74,3538419	226,488184	60,1295224
582,931641	420	283,074276	222,361555	197,158318	70,4850978	218,037714	59,7780472
584,813965	420	272,346342	215,380592	196,397968	68,9739698	214,927555	56,4770621
586,69458	420	257,130302	204,766954	187,729704	63,7335897	208,84277	52,8978962
588,57373	420	255,325614	202,127188	180,819246	62,641812	200,31505	50,115346
590,451111	420	248,634818	195,762902	172,918606	60,4085823	192,927281	51,643095
592,326965	420	239,725774	183,172852	168,368112	60,781286	189,525415	45,6110137
594,201233	420	229,241058	178,961625	160,315383	58,0114751	181,412752	45,7834239
596,073914	420	226,622663	167,060625	161,113346	51,0647415	175,723863	45,821732
597,944946	420	212,981884	165,804264	160,01177	52,711844	169,30012	42,0266243
599,814453	420	212,550078	159,823565	150,461988	51,9991854	162,648285	41,4863229
601,682251	420	208,155343	153,873135	144,252651	49,3854738	163,438963	40,289811
603,548523	420	201,696923	147,029659	142,628764	43,6497092	160,453301	38,3028882
605,413147	420	191,839708	144,223145	138,595712	47,0062592	153,165934	34,6622417
607,276245	420	188,624677	138,24074	133,289224	46,3517312	145,928434	36,5039434
609,137695	420	181,760932	131,395253	132,788832	44,7507448	144,824693	33,4638725
610,997437	420	181,389759	127,86051	126,060568	41,9229837	137,009189	33,1656833
612,855713	420	171,582178	122,65063	124,21234	40,5524689	132,823919	32,5215009
614,712341	420	168,378315	118,662535	126,958049	39,4186167	130,400008	29,3921856
616,567322	420	162,098548	114,09983	117,880314	38,7288023	125,482522	26,1300249
618,420715	420	158,440368	108,836871	117,614347	34,826555	123,905457	26,9014807
620,272522	420	149,93583	104,140725	112,066612	36,076205	116,592255	27,252508
622,122681	420	147,969802	101,85908	106,056387	32,6195105	111,388219	25,9125164
623,971313	420	145,857473	98,3686803	103,249198	31,6012722	108,662381	24,8023761
625,818237	420	137,364916	93,934233	100,843325	32,7638151	108,868789	22,2324162
627,663574	420	129,862294	93,0587577	98,1279276	29,7026311	101,774613	25,6864592
629,507324	420	134,542687	85,7489143	96,4496996	28,9534604	99,5116801	21,6351525
631,349365	420	125,206435	86,8586023	92,940416	27,7799626	95,9216093	21,3524623
633,18988	420	121,445621	81,0675288	89,7930125	25,5992822	92,1738816	22,22549
635,028809	420	116,27195	78,3233533	83,6121779	25,6587631	91,5195521	19,8189519
636,865967	420	111,723956	74,9233447	80,9764606	23,2271204	85,7395087	19,1014823
638,70166	420	106,899006	76,1651746	78,8494434	22,5651853	84,2855738	18,8121894
640,535645	420	104,925178	68,9487088	75,9900148	23,6853451	83,219531	17,3849655
642,368042	420	100,988041	67,6046038	74,8073318	24,1641567	78,7368683	16,5806388
644,198853	420	97,7368598	67,0729511	72,8653921	22,1105248	76,1962003	14,962288
646,028015	420	98,303063	62,6287034	70,8440141	21,3832024	75,3045083	17,538948
647,855591	420	92,2223581	59,1358719	66,9038663	19,5675614	72,7849463	15,7254418
649,681519	420	91,8058159	56,9533135	64,8437771	19,9750139	69,6902583	15,5070875
651,505798	420	86,8499867	55,2100139	62,302325	19,6012131	67,0100954	14,2795429
653,328491	420	83,3731656	57,2872281	59,4495767	18,1374755	63,7833376	13,1045328
655,149536	420	79,5343143	49,1504099	58,9778962	18,8917016	61,4880546	12,4639283
656,968994	420	77,9376983	50,583548	57,5339933	17,6641701	61,3577432	11,8244061
658,786743	420	76,4543664	51,2896528	57,732898	18,9836097	58,329966	11,9582767
660,602905	420	74,4637811	47,9270648	54,1611731	15,3157187	57,4615293	12,682628
662,41748	420	70,9804578	47,5201673	51,1448637	14,4521248	52,1966177	11,03063
664,230469	420	67,3732235	42,7824062	50,545917	13,546407	53,6246403	11,0523958
666,041748	420	66,7922698	41,299076	49,0021538	15,2090218	52,785477	10,4487583

This journal is © The Royal Society of Chemistry 20xx

Please do not adjust margins

667,85144	420	62,1918874	40,7863277	48,1213113	13,7648523	47,2147054	11,0248126
669,659424	420	61,611214	39,6926918	45,3229803	14,1847151	47,232357	8,6184257
671,46582	420	57,7409053	38,476888	43,8805182	15,2975006	44,6103147	9,80367926
673,27063	420	59,704589	36,2626952	39,9618718	11,4983921	43,7601018	6,69262353
675,073792	420	55,9423681	34,0419974	41,4575248	12,8619666	43,9233693	8,2832789
676,875305	420	52,7673064	30,9557613	41,5284669	12,3072785	42,3353646	8,67607564
678,675171	420	51,4305847	33,646046	39,2494558	11,9147077	38,9452456	7,49416521
680,47345	420	49,8100563	31,2527329	38,9044808	9,22487913	35,2055201	7,40961921
682,27002	420	46,1744604	30,1731152	35,1669778	8,38654438	37,4661794	7,83079026
684,065063	420	44,3379042	30,0350319	35,1252872	12,6216296	35,2065213	7,46000936
685,858398	420	45,7907906	27,0274794	33,9508192	9,84759824	34,2678605	5,16077834
687,650024	420	42,6476677	26,0994694	32,2376762	10,0958657	31,3571908	6,67593873
689,440186	420	40,0296621	25,4419979	31,8346721	10,8363106	29,5949445	4,85920908
691,228577	420	40,4490481	24,8959506	31,7707068	7,33405152	28,6517835	5,26009124
693,015381	420	37,9867664	22,2389927	27,9394737	9,83008868	30,6444747	5,19348515
694,800476	420	37,576674	24,2328477	27,3738452	8,86883985	30,2368619	5,11230812
696,583984	420	35,0736784	22,0822769	27,9651044	8,5837477	25,8612862	5,13294196
698,365845	420	34,2041887	20,694879	26,3562412	7,80339356	26,4977353	5,95164076
700,146118	420	34,4217922	20,3733019	24,9286764	7,34994824	27,5739098	5,3294211
701,924683	420	34,9606437	17,5299649	25,9153533	7,7836005	25,488251	4,86355428
347,741211	425	0	0	0	0	0	0
349,808533	425	0	0	0	0	0	0
351,874329	425	0	0	0	0	0	0
353,938568	425	0	0	0	0	0	0
356,001343	425	0	0	0	0	0	0
358,062561	425	0	0	0	0	0	0
360,122253	425	0	0	0	0	0	0
362,180481	425	0	0	0	0	0	0
364,237152	425	0	0	0	0	0	0
366,292328	425	0	0	0	0	0	0
368,345978	425	0	0	0	0	0	0
370,398132	425	0	0	0	0	0	0
372,44873	425	0	0	0	0	0	0
374,497803	425	0	0	0	0	0	0
376,545349	425	0	0	0	0	0	0
378,5914	425	0	0	0	0	0	0
380,635925	425	0	0	0	0	0	0
382,678955	425	0	0	0	0	0	0
384,720398	425	0	0	0	0	0	0
386,760376	425	0	0	0	0	0	0
388,798767	425	0	0	0	0	0	0
390,835663	425	0	0	0	0	0	0
392,871033	425	0	0	0	0	0	0
394,904877	425	0	0	0	0	0	0
396,937195	425	0	0	0	0	0	0
398,967957	425	0	0	0	0	0	0
400,997253	425	0	0	0	0	0	0

This journal is © The Royal Society of Chemistry 20xx

Please do not adjust margins

Please do not adjust margins

403,024963	425	0	0	0	0	0	0
405,051147	425	0	0	0	0	0	0
407,075806	425	0	0	0	0	0	0
409,098938	425	0	0	0	0	0	0
411,120544	425	0	0	0	0	0	0
413,140564	425	0	0	0	0	0	0
415,159119	425	0	0	0	0	0	0
417,176086	425	0	0	0	0	0	0
419,191528	425	0	0	0	0	0	0
421,205444	425	0	0	0	0	0	0
423,217834	425	0	0	0	0	0	0
425,228668	425	0	0	0	0	0	0
427,237976	425	0	0	0	0	0	0
429,245728	425	0	0	0	0	0	0
431,251953	425	0	0	0	0	0	0
433,256592	425	0	0	0	0	0	0
435,259766	425	92,3692858	210,042002	72,4403201	162,345486	82,5723653	61,2761213
437,261353	425	104,173434	229,609741	77,633297	130,354567	94,9493067	66,368989
439,261383	425	117,320831	254,276153	80,3167202	122,840294	102,659157	71,7215423
441,259888	425	128,698808	280,236051	93,1350307	121,82927	116,277365	77,0098195
443,256836	425	140,914324	298,333068	98,2851753	123,260396	129,119088	83,9529529
445,252258	425	153,739523	325,815441	109,031288	123,941973	141,226866	90,9595158
447,246094	425	172,578067	341,406065	119,272043	132,378576	151,045306	99,5769671
449,238434	425	180,124825	362,440944	127,821561	138,399215	162,679887	106,199232
451,229187	425	197,867667	387,052161	140,274281	141,080836	176,617934	113,719302
453,218445	425	208,925992	411,574603	149,882557	144,956703	189,874262	123,976404
455,206116	425	223,213314	434,272126	160,85853	151,324685	199,59723	126,307737
457,192261	425	245,934786	447,745882	172,420957	157,076754	214,14242	134,38131
459,176819	425	258,329039	473,599263	188,229394	160,326232	229,937367	137,320606
461,15979	425	275,199887	486,435612	194,459976	167,663368	242,752177	147,948079
463,141296	425	299,555792	511,354956	208,85651	174,799931	260,653016	154,614186
465,121216	425	314,217616	523,304947	207,881661	178,817734	276,713315	161,24095
467,099579	425	329,225317	539,610671	232,353992	185,306359	285,510068	163,791224
469,076385	425	342,101302	561,52107	239,694744	193,47583	303,515139	165,123319
471,051636	425	361,909561	570,420144	253,627938	200,604724	309,758423	174,974814
473,02536	425	378,528611	592,70912	266,725591	205,460576	327,571134	182,041868
474,997498	425	391,964898	596,907557	278,395979	209,184569	339,603643	181,649026
476,968079	425	410,056378	619,569102	285,206544	217,52438	346,623903	188,059896
478,937134	425	427,951246	622,168292	290,743393	219,200065	362,801169	191,847212
480,904572	425	431,705206	635,429917	311,091378	221,472167	376,893058	194,063789
482,870453	425	447,793879	632,927259	315,849209	214,016006	383,76369	195,777337
484,834808	425	468,757572	641,538072	330,070595	221,545658	397,099202	197,060625
486,797607	425	488,433816	646,048282	335,682476	217,086549	399,969415	199,481255
488,75885	425	495,503823	660,817538	339,960974	215,194013	413,016529	194,523108
490,718506	425	495,935982	667,817318	352,031833	213,458773	419,836604	204,279928
492,676605	425	517,246133	662,152412	360,714157	205,722193	430,883112	202,629099
494,633118	425	521,38639	658,46395	359,785033	210,572601	425,050603	204,670649

 This journal is © The Royal Society of Chemistry 20xx

Please do not adjust margins

Please do not adjust margins

496,588104	425	525,360879	660,061831	367,641763	206,219989	435,179905	202,933652
498,541504	425	529,95221	656,426212	368,626197	206,495721	446,370269	199,619795
500,493347	425	534,61135	647,142427	368,795125	198,759092	444,192964	195,362959
502,443665	425	541,95737	638,484215	363,656813	200,661535	444,709382	191,611174
504,392365	425	532,887679	636,987018	367,530786	192,183636	442,31606	182,788042
506,339539	425	533,718021	620,290001	368,415532	193,064072	445,788794	183,38204
508,285095	425	542,450474	615,759223	375,797694	192,30149	444,132519	187,471669
510,229126	425	533,056475	607,14477	365,962383	187,274743	444,836158	179,293973
512,17157	425	529,419912	600,163004	372,603309	178,233459	440,969129	172,46301
514,112427	425	527,308307	588,693202	368,733389	179,019915	440,07789	171,594399
516,051697	425	531,979575	570,296061	363,646878	176,031651	431,129872	166,571726
517,989441	425	527,788404	567,663047	362,340504	169,570587	427,557848	161,752457
519,925598	425	518,729855	551,209793	356,574872	170,462491	417,931826	155,115425
521,860168	425	518,459267	537,377318	353,47527	162,199761	422,115406	152,285028
523,793152	425	503,20126	516,643497	348,691898	161,38281	408,451919	151,654516
525,724609	425	501,68016	514,404587	350,522906	156,379339	410,731947	144,566953
527,654419	425	497,901409	505,540198	342,609358	157,054417	401,989552	145,626115
529,582764	425	491,02611	496,083462	341,022211	150,415295	399,012433	138,431055
531,50946	425	489,754985	477,371794	333,800477	151,239437	400,612814	136,012197
533,43457	425	486,449958	473,701042	336,052153	142,389725	392,245582	134,669362
535,358093	425	472,408679	454,994377	330,085946	139,623451	390,069022	128,987335
537,280029	425	474,257724	446,832348	324,966285	136,45531	387,081436	125,863018
539,200439	425	470,67456	438,414831	318,190478	134,919617	375,939011	121,642495
541,119263	425	448,940251	417,305886	316,049632	135,81318	372,686086	118,307713
543,036438	425	449,218881	413,901849	308,196894	131,642626	362,945399	119,092986
544,952087	425	444,652529	398,797062	305,071674	126,661316	355,041774	112,406895
546,866211	425	435,504642	386,63496	297,121892	122,436709	348,138488	105,890128
548,778687	425	423,723334	377,701694	290,626652	119,259607	338,537427	105,340828
550,689514	425	411,863799	364,582685	285,117627	114,02609	333,891965	103,490902
552,598816	425	403,679275	357,870221	279,485191	109,574003	329,74241	98,9310597
554,506531	425	387,3911	343,095061	277,622961	108,119665	318,671018	97,7491558
556,41272	425	390,505276	336,99466	271,23066	104,475693	311,354528	97,5878988
558,317261	425	380,171321	326,938952	268,555949	99,8517271	307,539782	91,2859994
560,220215	425	371,763752	315,915038	262,02315	99,3305079	297,387808	87,7446221
562,121582	425	359,255283	306,065168	252,47244	92,8115972	284,678869	84,3601726
564,021362	425	361,013129	296,58424	246,300087	92,8501779	277,127951	81,6972628
565,919556	425	350,477259	284,130927	244,094359	91,12984	275,203666	77,962398
567,816162	425	335,556467	275,815674	234,715254	91,5363642	270,730651	74,8032674
569,711182	425	329,725947	268,295522	232,573576	86,1275695	261,766204	72,3994521
571,604614	425	324,431462	261,091004	224,665823	80,7746262	260,892537	68,8291041
573,49646	425	319,274278	253,148146	222,515642	79,5042221	249,796981	66,5635689
575,386719	425	302,850434	243,291964	214,981905	80,2854865	240,827542	66,6197722
577,27533	425	303,117518	233,553332	213,735083	75,1943828	234,535268	63,4577035
579,162354	425	289,226132	229,044212	200,087474	71,5358012	232,318805	62,6754333
581,047852	425	281,417115	213,824494	199,724319	70,3674366	222,756623	58,7554542
582,931641	425	275,21217	214,591213	194,851816	67,9315543	215,306444	55,7621378
584,813965	425	268,790327	201,45722	187,885558	65,3152157	204,970036	55,6963293

This journal is © The Royal Society of Chemistry 20xx

Please do not adjust margins

Please do not adjust margins

586,69458	425	259,006942	199,608532	180,117268	63,7608525	202,992393	52,5727677
588,57373	425	251,962189	189,167586	177,954595	60,2922337	199,117444	52,2119151
590,451111	425	238,944343	178,726043	170,96807	59,2741283	194,236946	47,6416053
592,326965	425	237,252463	174,726439	162,723157	57,4844699	183,372716	46,7287506
594,201233	425	231,096681	164,855572	158,763742	52,7667564	179,082355	45,1754644
596,073914	425	221,310212	164,303548	157,876605	54,0254334	171,485929	42,4412535
597,944946	425	211,769552	158,906745	154,601086	51,1357285	166,739758	41,8928924
599,814453	425	206,098141	153,802823	147,401943	48,6889304	159,873496	40,4851537
601,682251	425	203,94232	147,854368	145,024474	47,5592849	159,206873	35,6764592
603,548523	425	198,649274	141,528674	140,372078	43,9124697	154,450528	38,2771867
605,413147	425	192,958432	136,157439	139,267195	46,9434851	149,05859	34,5282871
607,276245	425	187,391436	130,177531	132,419973	42,6175494	142,122562	35,1970269
609,137695	425	178,513588	124,713329	129,907331	41,182559	142,6193	32,5492437
610,997437	425	177,638518	124,094024	126,268309	40,7591329	133,519294	32,6137013
612,855713	425	172,931699	120,239566	120,900833	38,8254889	130,752174	31,4706935
614,712341	425	166,87407	113,105649	120,430894	37,1935714	126,471736	29,8909681
616,567322	425	160,304249	108,87771	116,213005	37,6681149	120,447384	27,6770037
618,420715	425	156,106903	107,948976	108,910455	35,0393617	121,35038	27,4274872
620,272522	425	147,189419	101,5583	107,542345	34,8957663	114,407906	25,5952474
622,122681	425	142,908258	100,391815	104,421969	32,4209602	111,155643	25,2860215
623,971313	425	139,786454	93,3560241	100,615922	28,7838689	105,466635	26,0859303
625,818237	425	135,195097	89,0273917	96,9051792	32,5647175	102,874602	23,9148197
627,663574	425	129,765178	89,0368374	98,7657062	29,3156802	99,0126046	24,2508064
629,507324	425	128,037369	81,8651691	90,6119106	28,7955585	99,4264125	22,3763215
631,349365	425	125,170863	83,9958251	92,2306517	25,1223223	94,4729616	22,6145244
633,18988	425	120,417921	80,6345617	89,0385594	25,6253573	89,9514831	20,0746641
635,028809	425	114,710411	77,6976238	85,5798273	26,4242069	87,3058533	19,5444223
636,865967	425	110,585783	74,0380327	80,8966248	23,8716093	87,7417679	20,0204925
638,70166	425	106,856813	71,7776994	79,1605499	23,2913255	85,415304	19,4379748
640,535645	425	104,979795	68,3506922	79,5168916	23,6578605	79,8349146	17,134914
642,368042	425	101,907761	65,5352572	73,0030935	21,0133046	77,4054338	16,1901018
644,198853	425	97,0585116	59,1238728	73,0861663	22,0259972	71,9890019	16,5227377
646,028015	425	97,7679761	61,6524823	71,3825223	21,9770082	72,0723759	16,9144382
647,855591	425	92,1300353	57,8995285	66,5099758	18,5419264	73,8286573	15,7897965
649,681519	425	91,3484906	57,4604708	62,3735036	19,3150136	72,5014145	15,724606
651,505798	425	83,8164634	55,9663304	61,8778271	18,7929692	64,8273799	12,4809788
653,328491	425	82,1108122	56,7424878	59,1272823	18,247669	60,9535775	12,4440326
655,149536	425	80,6652856	49,6785162	59,1906149	17,9355467	60,8720786	12,8676832
656,968994	425	77,1162267	48,6671628	58,7512176	16,6418524	58,8429344	11,4007645
658,786743	425	75,2678846	47,0668412	55,2146038	15,576361	59,9654786	10,9972347
660,602905	425	74,8329615	46,4891424	54,9627698	14,6183282	55,743153	10,8702079
662,41748	425	70,8561432	45,6209268	49,1350714	14,607117	52,6093359	11,3804069
664,230469	425	69,2210587	40,9317246	48,1533895	16,0225027	51,4030234	10,4783064
666,041748	425	64,2448943	41,6242181	50,4024709	13,6594146	50,0299002	10,8061756
667,85144	425	62,5609518	40,1124604	48,5286717	13,9291156	46,2964864	9,52009572
669,659424	425	61,0570072	36,5471573	46,0681172	12,2515568	45,5373867	9,5394749
671,46582	425	60,1863148	35,264065	42,8545482	12,9626707	43,8755198	8,61759663

This journal is © The Royal Society of Chemistry 20xx

Please do not adjust margins

673,27063	425	58,70472	33,6867145	43,6459809	12,0783637	43,8135589	7,48226016
675,073792	425	52,2476977	33,9078422	38,4199548	10,9304982	41,5546246	8,58993292
676,875305	425	52,0813921	31,4017706	38,8030439	13,6605325	38,0324542	8,27600246
678,675171	425	50,68184	29,2192254	38,0283411	12,6477598	38,1166062	7,57452657
680,47345	425	47,9859522	29,1969225	37,8432595	10,8438068	36,2143556	6,86649028
682,27002	425	46,7406091	29,3764359	33,9029161	10,3334006	35,4000258	7,20347953
684,065063	425	47,3680733	27,6663343	34,0156032	12,2527918	34,8890573	5,73480269
685,858398	425	44,8698291	26,184454	31,3583719	10,0579797	33,3320828	6,75903044
687,650024	425	42,077703	23,7704519	30,9174999	10,6185633	31,4765984	6,5297304
689,440186	425	40,9112944	25,324345	32,9602725	9,69705364	30,9183681	5,91399722
691,228577	425	38,4028381	23,0478083	29,9786243	7,65103188	29,749895	5,60133816
693,015381	425	37,47006	23,739099	28,235307	8,85650751	28,1637371	5,06185398
694,800476	425	37,3815027	20,0940835	26,9631227	7,58939603	30,6085501	4,90244622
696,583984	425	37,5323786	20,7311945	27,1513387	7,85815353	27,7144684	4,92223303
698,365845	425	34,0494872	19,8336103	26,5907274	6,92505422	24,8458769	5,89143121
700,146118	425	32,5842869	17,7536034	24,1391407	7,11254527	25,1050156	4,87584665
701,924683	425	34,1561693	18,464085	25,9971828	5,77608997	24,8002319	4,5736347
347,741211	430	0	0	0	0	0	0
349,808533	430	0	0	0	0	0	0
351,874329	430	0	0	0	0	0	0
353,938568	430	0	0	0	0	0	0
356,001343	430	0	0	0	0	0	0
358,062561	430	0	0	0	0	0	0
360,122253	430	0	0	0	0	0	0
362,180481	430	0	0	0	0	0	0
364,237152	430	0	0	0	0	0	0
366,292328	430	0	0	0	0	0	0
368,345978	430	0	0	0	0	0	0
370,398132	430	0	0	0	0	0	0
372,44873	430	0	0	0	0	0	0
374,497803	430	0	0	0	0	0	0
376,545349	430	0	0	0	0	0	0
378,5914	430	0	0	0	0	0	0
380,635925	430	0	0	0	0	0	0
382,678955	430	0	0	0	0	0	0
384,720398	430	0	0	0	0	0	0
386,760376	430	0	0	0	0	0	0
388,798767	430	0	0	0	0	0	0
390,835663	430	0	0	0	0	0	0
392,871033	430	0	0	0	0	0	0
394,904877	430	0	0	0	0	0	0
396,937195	430	0	0	0	0	0	0
398,967957	430	0	0	0	0	0	0
400,997253	430	0	0	0	0	0	0
403,024963	430	0	0	0	0	0	0
405,051147	430	0	0	0	0	0	0
407,075806	430	0	0	0	0	0	0

This journal is © The Royal Society of Chemistry 20xx

Please do not adjust margins

409,098938	430	0	0	0	0	0	0
411,120544	430	0	0	0	0	0	0
413,140564	430	0	0	0	0	0	0
415,159119	430	0	0	0	0	0	0
417,176086	430	0	0	0	0	0	0
419,191528	430	0	0	0	0	0	0
421,205444	430	0	0	0	0	0	0
423,217834	430	0	0	0	0	0	0
425,228668	430	0	0	0	0	0	0
427,237976	430	0	0	0	0	0	0
429,245728	430	0	0	0	0	0	0
431,251953	430	0	0	0	0	0	0
433,256592	430	0	0	0	0	0	0
435,259766	430	0	0	0	0	0	0
437,261353	430	0	0	0	0	0	0
439,261383	430	0	0	0	0	0	0
441,259888	430	92,795082	187,717791	72,3927479	132,696523	88,7315041	64,5989896
443,256836	430	101,443123	202,531235	77,4488149	119,293709	99,2875554	70,9178177
445,252258	430	112,741191	227,554335	81,475991	110,999063	106,649408	72,2082807
447,246094	430	125,686285	241,410306	90,2416795	113,361812	115,810906	82,0437645
449,238434	430	138,067382	264,090195	98,3525875	114,837457	127,119251	86,0043056
451,229187	430	152,847871	282,210706	110,101823	120,438507	140,0397	99,5967087
453,218445	430	164,965867	302,793196	116,973825	123,994657	149,973126	101,644557
455,206116	430	180,926759	323,278507	130,191114	128,667785	158,716494	109,85449
457,192261	430	197,602912	342,818714	138,489439	137,050053	176,873785	117,576423
459,176819	430	212,906249	356,831249	151,519937	141,482226	191,543018	122,418017
461,15979	430	225,345548	385,247454	161,887683	144,947417	207,888513	127,586232
463,141296	430	248,944875	401,779638	174,842182	149,461673	214,76033	136,363907
465,121216	430	257,851683	417,08699	181,509926	158,042801	232,213411	140,774503
467,099579	430	276,414576	434,532345	191,773066	159,70883	245,674862	148,098188
469,076385	430	291,645158	456,123429	205,108552	162,61401	257,042641	151,110759
471,051636	430	311,507481	466,444335	220,53749	172,057539	270,244024	157,53194
473,02536	430	328,979461	496,707093	229,131385	173,870491	287,960533	163,579354
474,997498	430	345,691814	496,644946	242,239195	184,195281	301,534473	166,565538
476,968079	430	362,061232	519,587416	249,619771	187,00812	316,852903	167,796792
478,937134	430	375,186728	525,769419	259,188364	191,804344	325,119092	171,491482
480,904572	430	389,24113	529,196409	271,829062	198,204773	328,124263	173,297769
482,870453	430	405,101327	545,448747	283,612887	198,759042	345,272616	169,33577
484,834808	430	421,33797	549,781942	284,857802	202,11667	354,129012	178,369371
486,797607	430	436,554486	558,97617	306,17572	197,693721	366,168549	179,708284
488,75885	430	454,411144	557,417457	306,007843	195,088099	376,568672	183,132099
490,718506	430	454,357977	570,168878	313,167152	199,250628	384,403657	182,040888
492,676605	430	466,372639	570,981795	320,528271	196,3759	391,213257	183,973506
494,633118	430	482,216483	572,175374	327,852569	192,385435	405,614749	184,078678
496,588104	430	490,780176	574,218309	337,305166	194,227513	405,46954	186,692809
498,541504	430	491,959523	570,993727	341,901856	188,358291	407,710092	187,921735
500,493347	430	505,54683	578,760856	342,584581	186,521591	413,101072	182,254813

This journal is © The Royal Society of Chemistry 20xx

Please do not adjust margins

Please do not adjust margins

502,443665	430	491,100394	557,684607	347,905722	186,51326	417,78653	182,843732
504,392365	430	502,283235	563,689958	348,533854	180,373923	417,836941	182,169338
506,339539	430	511,716208	552,60226	349,956375	181,891821	422,865303	183,245795
508,285095	430	503,427147	550,813107	344,240704	176,090438	419,063112	173,399069
510,229126	430	502,29999	541,608229	344,933159	168,572649	417,343673	173,347163
512,17157	430	497,00301	519,400963	345,237249	169,118238	416,579997	163,864206
514,112427	430	509,715518	524,864842	342,101881	169,451734	416,025603	163,253392
516,051697	430	498,039547	518,877264	344,43989	156,954465	412,857271	158,267278
517,989441	430	499,259335	503,069276	341,43202	158,89212	408,903477	153,323235
519,925598	430	489,577665	495,138539	335,441552	153,288575	404,395322	151,24133
521,860168	430	485,15584	480,904946	331,992928	147,841585	397,986737	146,823628
523,793152	430	479,669098	472,399504	333,023342	150,879058	395,846888	143,450149
525,724609	430	477,944463	465,77445	324,291033	143,189864	391,249204	135,242572
527,654419	430	475,924313	451,976144	323,633429	142,213368	393,23451	137,151413
529,582764	430	477,564764	449,068848	322,320938	137,658889	386,829523	131,6475
531,50946	430	462,052734	437,09075	315,339961	142,984806	383,377219	129,377085
533,43457	430	466,342592	434,057421	315,97416	135,160925	385,046409	128,986612
535,358093	430	462,861044	422,892297	312,14989	131,62928	370,433356	123,309642
537,280029	430	450,949688	419,376926	305,525392	133,977083	371,251024	119,764522
539,200439	430	445,065866	407,277409	307,696288	124,758587	364,644531	116,590057
541,119263	430	439,536477	391,602762	294,439044	121,575823	352,695379	113,305083
543,036438	430	435,163349	378,263305	294,66706	120,807227	352,470395	115,157185
544,952087	430	425,054441	363,853251	287,398948	115,450304	340,175297	108,285059
546,866211	430	419,077075	357,49485	286,798542	113,847973	337,394559	109,382215
548,778687	430	413,162856	346,78707	283,903142	112,970274	333,422083	101,773384
550,689514	430	398,841877	342,635241	278,322478	108,796722	323,08918	100,013294
552,598816	430	391,033345	333,779613	269,901783	106,126443	314,609056	95,8391676
554,506531	430	385,751661	321,885343	264,211814	105,218759	310,686906	92,8792556
556,41272	430	383,051735	315,906033	259,472952	97,7620327	303,500631	89,0677925
558,317261	430	368,110243	303,294864	255,032143	97,1012628	300,11897	85,884645
560,220215	430	366,106284	292,446145	250,821241	94,3945374	286,067824	81,7821777
562,121582	430	356,526212	284,053063	248,469228	92,8567875	278,186504	81,4826269
564,021362	430	343,330352	277,512558	238,959752	85,570969	273,233919	80,0439453
565,919556	430	337,732665	267,318807	230,86263	88,722528	271,99052	75,2068488
567,816162	430	329,329205	258,5764	230,877598	86,1677161	262,648128	71,4038918
569,711182	430	320,181555	252,83639	224,306805	79,7896398	256,03875	67,1984711
571,604614	430	317,896846	245,512326	224,503746	79,336888	252,650914	68,515257
573,49646	430	305,654202	235,168069	218,920432	75,7035188	242,521229	65,7319242
575,386719	430	297,892337	230,814009	203,903997	73,6107309	239,546891	63,9502754
577,27533	430	295,476059	224,610989	202,415797	71,9601255	231,877104	64,2990746
579,162354	430	287,491404	213,399317	201,094919	70,8016069	224,455855	57,8351263
581,047852	430	271,563654	206,276821	194,958983	66,6830723	216,336166	57,658906
582,931641	430	267,908571	203,037052	185,931686	64,7543958	213,024411	54,972437
584,813965	430	262,236113	192,569674	181,021241	64,29954	212,465951	52,3513743
586,69458	430	257,679702	188,084842	175,048669	61,3053925	198,598689	50,4990992
588,57373	430	245,907438	181,951521	169,756265	54,7296621	193,280521	48,4629846
590,451111	430	239,471539	172,201033	163,836516	54,239499	184,53655	47,35425

This journal is © The Royal Society of Chemistry 20xx

Please do not adjust margins

Please do not adjust margins

592,326965	430	232,995515	164,778184	159,736458	52,2065533	180,228809	46,2401643
594,201233	430	222,945923	159,024002	157,81196	50,8640441	171,084289	43,4136059
596,073914	430	220,376634	149,925555	153,545563	49,1413216	172,111725	43,0799061
597,944946	430	212,525377	150,728036	151,243074	47,7270941	167,334071	41,9595368
599,814453	430	204,963241	142,16438	145,199902	46,3191912	155,120955	39,1268329
601,682251	430	201,247382	141,398714	139,115326	45,2772983	154,252497	38,8194258
603,548523	430	193,001143	134,861211	133,444831	44,6073976	151,124444	36,0869413
605,413147	430	189,453035	130,125657	130,858757	45,6754146	145,56949	35,3824202
607,276245	430	184,162442	129,284909	128,874777	42,1087813	139,70005	36,7560291
609,137695	430	177,593821	121,379438	126,248315	39,0496844	138,695098	33,776606
610,997437	430	172,154597	115,903247	119,141281	39,4126135	133,891603	31,344502
612,855713	430	167,926178	109,226062	120,828113	37,498477	127,429385	30,7130876
614,712341	430	161,853456	108,01555	114,849211	36,6686054	124,042573	29,2919603
616,567322	430	159,083799	103,395774	111,45801	36,9230391	119,866518	27,1008372
618,420715	430	152,84737	98,2303827	103,289856	30,8025036	119,970954	27,6433168
620,272522	430	144,454652	95,8468453	106,244777	34,5376201	116,189678	25,9032566
622,122681	430	143,321725	91,6511353	100,702676	30,8623719	113,767294	25,6706122
623,971313	430	137,415211	89,9481718	96,7106012	30,7299282	106,908027	23,716312
625,818237	430	131,125311	87,5426437	96,8019763	28,8187089	100,85834	23,3744059
627,663574	430	128,370444	85,367656	93,4802652	29,0875229	98,3403742	24,2154017
629,507324	430	127,758791	82,5701563	89,4812508	26,8923272	98,8979427	22,8000531
631,349365	430	123,386153	79,4838613	89,1814031	26,4733624	94,9416722	21,0617144
633,18988	430	115,453794	76,5149687	84,6965157	26,0953723	89,2156653	21,0375951
635,028809	430	111,203537	72,583383	80,9814881	26,0797493	88,5751762	19,266812
636,865967	430	112,189062	73,139743	76,7798465	24,5804767	85,9968803	18,4170434
638,70166	430	107,546068	64,6569918	75,0594162	23,6395986	81,6316444	17,8398499
640,535645	430	101,573047	61,119019	76,2205128	22,6810383	75,8814271	17,0291564
642,368042	430	102,136986	60,6042941	72,1218165	23,8842138	74,2796922	17,4906215
644,198853	430	97,563948	59,1871842	68,667227	21,0634531	74,2186171	15,8344394
646,028015	430	93,6162977	58,886767	70,6645817	20,5717116	70,3160574	16,2951812
647,855591	430	91,8039863	55,2466042	65,1774702	20,7100665	69,989816	14,5928817
649,681519	430	87,6215363	53,8369191	63,6734242	18,3758172	65,4658644	15,189712
651,505798	430	80,606174	51,0796337	60,4367569	18,4512903	65,2130414	14,5080943
653,328491	430	78,8370096	50,9383261	56,9647632	17,8364984	63,2460492	13,5079974
655,149536	430	79,7982844	49,6990477	53,9496335	17,6016671	57,8484411	13,8566538
656,968994	430	76,6639132	49,0012101	55,4516877	17,3735753	57,4848098	11,7328229
658,786743	430	74,4588954	49,0850005	53,5252647	16,4744908	58,2904719	11,335677
660,602905	430	71,9807233	45,4108288	50,2420785	14,0137943	53,344626	11,135303
662,41748	430	70,7802798	42,3403033	52,2185433	14,6854712	52,89826	12,250594
664,230469	430	65,6524782	37,0827176	48,5080553	12,7321914	51,5515587	10,5974697
666,041748	430	66,4605989	38,908534	47,2212006	13,5956035	50,2684158	10,6168085
667,85144	430	62,5020555	37,022559	43,9031204	13,6329772	45,8799812	8,80783648
669,659424	430	60,4007584	36,7906932	43,3015084	12,3560523	44,6664394	9,82346104
671,46582	430	57,6163163	34,3679641	41,4244271	13,2137514	43,8655806	8,60431705
673,27063	430	54,2124671	32,5729522	40,5837224	12,2629003	42,8785803	9,33215925
675,073792	430	52,2277537	30,2382702	37,8789324	9,50466252	42,7289291	6,95464227
676,875305	430	54,151847	31,3150326	37,0215692	11,8206315	40,7086156	8,42124772

This journal is © The Royal Society of Chemistry 20xx

Please do not adjust margins

Please do not adjust margins

678,675171	430	49,8276166	26,912008	36,0225323	9,58164215	37,696286	6,87715741
680,47345	430	48,2433231	30,6779082	36,2267641	9,41990387	34,9604018	8,03009486
682,27002	430	45,8503302	26,2867743	34,4920553	9,83994617	35,3143924	8,36784166
684,065063	430	44,9129851	27,8523438	34,7622362	10,0281272	33,4849704	6,84090393
685,858398	430	44,8547998	27,4753831	33,2214351	9,64366552	32,2531499	6,92167426
687,650024	430	39,9884905	24,386226	29,4383538	10,4317329	32,2072061	6,07221248
689,440186	430	41,4872703	25,8482381	29,3502808	9,28541495	30,7965394	5,6180752
691,228577	430	39,0844438	22,4232438	29,5209145	7,96284235	28,8566328	6,08924216
693,015381	430	36,6754946	21,5399868	27,0900808	9,23519667	30,9679586	5,16545727
694,800476	430	35,6478246	22,3959908	25,9091822	8,05921748	27,9817716	5,0859221
696,583984	430	34,6117569	20,127672	26,9568514	8,95591236	26,4420792	4,08515956
698,365845	430	34,7878964	20,4996072	26,1656627	5,82982225	27,147721	6,06617613
700,146118	430	36,1940257	18,5233341	23,8963576	7,43502716	24,7999259	4,27119267
701,924683	430	32,7555067	18,3561542	22,9556199	7,70181097	24,3385045	7,0666214
347,741211	435	0	0	0	0	0	0
349,808533	435	0	0	0	0	0	0
351,874329	435	0	0	0	0	0	0
353,938568	435	0	0	0	0	0	0
356,001343	435	0	0	0	0	0	0
358,062561	435	0	0	0	0	0	0
360,122253	435	0	0	0	0	0	0
362,180481	435	0	0	0	0	0	0
364,237152	435	0	0	0	0	0	0
366,292328	435	0	0	0	0	0	0
368,345978	435	0	0	0	0	0	0
370,398132	435	0	0	0	0	0	0
372,44873	435	0	0	0	0	0	0
374,497803	435	0	0	0	0	0	0
376,545349	435	0	0	0	0	0	0
378,5914	435	0	0	0	0	0	0
380,635925	435	0	0	0	0	0	0
382,678955	435	0	0	0	0	0	0
384,720398	435	0	0	0	0	0	0
386,760376	435	0	0	0	0	0	0
388,798767	435	0	0	0	0	0	0
390,835663	435	0	0	0	0	0	0
392,871033	435	0	0	0	0	0	0
394,904877	435	0	0	0	0	0	0
396,937195	435	0	0	0	0	0	0
398,967957	435	0	0	0	0	0	0
400,997253	435	0	0	0	0	0	0
403,024963	435	0	0	0	0	0	0
405,051147	435	0	0	0	0	0	0
407,075806	435	0	0	0	0	0	0
409,098938	435	0	0	0	0	0	0
411,120544	435	0	0	0	0	0	0
413,140564	435	0	0	0	0	0	0

This journal is © The Royal Society of Chemistry 20xx

Please do not adjust margins

Please do not adjust margins

415,159119	435	0	0	0	0	0	0
417,176086	435	0	0	0	0	0	0
419,191528	435	0	0	0	0	0	0
421,205444	435	0	0	0	0	0	0
423,217834	435	0	0	0	0	0	0
425,228668	435	0	0	0	0	0	0
427,237976	435	0	0	0	0	0	0
429,245728	435	0	0	0	0	0	0
431,251953	435	0	0	0	0	0	0
433,256592	435	0	0	0	0	0	0
435,259766	435	0	0	0	0	0	0
437,261353	435	0	0	0	0	0	0
439,261383	435	0	0	0	0	0	0
441,259888	435	0	0	0	0	0	0
443,256836	435	0	0	0	0	0	0
445,252258	435	84,9969722	163,412138	62,4272065	143,564102	84,4714855	63,8037151
447,246094	435	94,4937328	177,193898	70,3361302	121,918305	92,7936242	69,7073589
449,238434	435	102,909055	190,718671	77,1957672	107,290038	99,8893904	75,0065403
451,229187	435	117,625629	207,893027	86,2764727	109,618933	108,49958	78,6621621
453,218445	435	130,079855	229,894059	94,0226153	107,696345	118,578064	84,1314174
455,206116	435	140,793116	245,058487	99,318577	115,146605	131,129051	94,3007022
457,192261	435	162,764827	261,229714	111,228742	117,63471	145,124732	96,7048941
459,176819	435	169,040951	283,631952	124,688054	122,559869	153,659591	101,555687
461,15979	435	185,968037	306,240122	130,648969	128,376599	165,709604	110,291297
463,141296	435	205,897762	312,381114	143,741786	131,366578	179,846203	122,205465
465,121216	435	217,611386	333,088921	152,610198	132,086307	193,306867	120,307245
467,099579	435	234,180445	350,287289	167,023103	136,001778	206,874789	126,834627
469,076385	435	245,72702	359,498068	179,476758	139,598162	220,769868	132,625379
471,051636	435	264,060959	383,353907	189,099495	147,584545	236,00002	141,356532
473,02536	435	284,814933	398,213688	199,815157	157,2348	247,162179	140,966429
474,997498	435	302,325759	410,734377	208,969163	155,37463	262,011939	149,759301
476,968079	435	307,479963	418,347532	213,245032	164,600176	276,37275	149,251987
478,937134	435	336,472191	436,066422	228,805799	164,852451	283,04051	152,744339
480,904572	435	348,251281	442,969717	235,146938	168,715947	300,183397	154,992283
482,870453	435	364,665057	460,862229	252,635343	175,502605	316,921307	161,508618
484,834808	435	368,303736	470,906811	261,125795	184,828611	322,520734	169,807249
486,797607	435	386,336645	473,453167	269,682303	181,962706	332,941119	172,073796
488,75885	435	395,516985	477,887934	279,859689	179,879401	340,36497	167,515238
490,718506	435	410,383997	488,794512	281,035113	178,939756	351,935167	167,405518
492,676605	435	426,82296	492,656388	291,56581	179,706837	354,308519	173,984537
494,633118	435	424,799487	497,56396	295,109647	177,697507	362,663523	168,912974
496,588104	435	443,678353	490,599948	300,762454	180,344485	372,820631	171,673725
498,541504	435	445,590683	499,427696	310,254878	174,829813	379,577387	178,433396
500,493347	435	451,304492	499,7103	316,927902	172,649032	380,513758	170,516373
502,443665	435	465,664698	494,733723	320,50179	167,671845	392,908707	173,610727
504,392365	435	468,345459	502,380052	314,728341	164,658695	394,949074	169,222543
506,339539	435	471,479215	490,843622	322,746235	169,507979	395,439123	166,125163

This journal is © The Royal Society of Chemistry 20xx

Please do not adjust margins

Please do not adjust margins

508,285095	435	473,282251	491,326348	322,005801	165,406069	396,8986	170,118745
510,229126	435	477,411773	473,512773	322,118267	161,210595	393,475207	161,180075
512,17157	435	470,485159	480,688995	320,580375	157,707242	396,40618	165,664267
514,112427	435	478,471166	471,603409	323,228189	154,624088	389,001594	158,0094
516,051697	435	468,363028	461,485996	322,607336	150,051766	386,950324	151,477629
517,989441	435	466,074696	454,208005	317,780512	146,657931	394,536558	147,528745
519,925598	435	457,661918	444,451553	316,87819	142,166312	382,219073	141,463322
521,860168	435	466,462974	435,928535	313,328432	140,784219	380,418085	138,388652
523,793152	435	461,138019	429,204986	305,675918	136,628999	379,852861	137,016339
525,724609	435	457,643518	425,395467	308,267403	135,98347	377,056303	130,511057
527,654419	435	456,25584	416,916432	307,846791	133,032591	368,344453	131,2623
529,582764	435	444,895888	404,656189	308,076679	128,401359	371,118862	126,700238
531,50946	435	451,814926	401,926995	305,807069	128,899343	367,976276	129,292657
533,43457	435	445,958902	398,204982	301,605815	129,050556	358,617194	123,271099
535,358093	435	435,903293	386,137669	303,169305	125,604032	354,187804	119,506283
537,280029	435	439,077482	376,237791	290,731472	122,047847	353,99143	118,132621
539,200439	435	438,845283	369,110179	291,450857	117,931184	348,573927	111,444867
541,119263	435	426,001442	360,250016	287,073527	117,196025	341,95113	112,080203
543,036438	435	415,427644	350,133594	284,543036	112,791543	336,005488	107,206081
544,952087	435	411,70926	341,144024	280,806017	108,27174	332,909262	102,847456
546,866211	435	403,190894	336,359156	278,170477	107,969285	326,831287	101,237107
548,778687	435	399,146869	323,721172	268,884854	106,358232	320,067024	95,6959238
550,689514	435	386,847263	311,749893	261,937178	101,72726	311,768292	94,7441842
552,598816	435	387,111003	304,569243	259,735275	99,3396616	307,445392	89,4560161
554,506531	435	371,66745	296,805206	253,657711	96,3104353	305,323971	91,6732345
556,41272	435	371,752914	291,613593	245,669682	92,1000523	297,417634	89,1226326
558,317261	435	363,058154	282,85577	244,018891	91,1731644	288,665082	85,408996
560,220215	435	351,681458	276,256838	240,474143	87,3351275	282,27006	80,112273
562,121582	435	347,66677	269,030424	232,786337	87,9117638	272,665662	79,9424073
564,021362	435	335,527714	256,543433	226,796327	82,8818892	266,516868	75,7056521
565,919556	435	329,088863	247,791451	223,264622	81,1351312	264,844201	74,3274033
567,816162	435	326,774585	247,520823	224,666063	80,2349162	257,041133	75,0180688
569,711182	435	320,584941	236,422265	213,977635	78,8188248	246,52001	68,6709165
571,604614	435	307,737141	230,243993	210,614228	75,0454964	248,778685	65,3955416
573,49646	435	304,634273	224,879651	208,386147	71,5333266	239,605543	65,3842917
575,386719	435	294,757733	214,914294	198,658517	71,6053626	232,546722	62,9896758
577,27533	435	284,310794	211,911231	192,427741	70,9693566	229,163409	59,3663592
579,162354	435	278,903455	200,114624	194,781524	64,5905496	220,032418	59,0623994
581,047852	435	271,07985	196,178762	185,804504	64,3728977	215,423173	58,477992
582,931641	435	263,355122	190,389032	183,877419	63,1324749	207,363441	52,6923242
584,813965	435	262,788421	181,756849	179,657546	60,669252	201,819298	52,6208276
586,69458	435	252,129849	175,576928	173,064712	58,7022511	197,158087	50,4233044
588,57373	435	243,326882	169,031586	164,358899	56,0805211	189,336229	50,1276373
590,451111	435	234,335154	161,76771	163,874859	54,8991149	180,644499	48,7531136
592,326965	435	229,936455	158,780696	153,605954	51,80613	180,424576	45,6660212
594,201233	435	221,008349	148,890122	151,990219	51,5344171	167,359231	44,4108153
596,073914	435	209,826712	147,734593	147,038159	51,7648328	166,772316	40,5490378

This journal is © The Royal Society of Chemistry 20xx

Please do not adjust margins

Please do not adjust margins

597,944946	435	208,265467	135,120621	146,689114	46,5967322	164,098101	41,9541094
599,814453	435	198,408898	139,621944	143,106536	44,6871481	158,601755	37,8992166
601,682251	435	196,792082	130,318066	141,048546	45,7305597	149,904227	37,1642543
603,548523	435	193,143846	125,524874	134,091589	42,6237588	149,940796	38,8027624
605,413147	435	189,95337	124,239104	129,649659	40,9066211	144,539679	33,3052022
607,276245	435	178,516161	121,764838	124,673801	39,6305029	139,252251	34,324977
609,137695	435	179,733727	115,227244	119,208036	38,0709896	137,536691	32,9884931
610,997437	435	173,297866	109,739815	118,73058	38,8607337	130,668691	32,1257436
612,855713	435	167,210396	105,991815	114,705254	36,3022863	126,501939	30,9233645
614,712341	435	158,195594	103,841193	114,585291	34,6805306	125,410781	29,5029991
616,567322	435	154,775926	98,4285694	107,758406	36,7262099	121,028304	25,6636105
618,420715	435	151,337051	97,9986166	103,898767	32,901079	116,251848	26,9936173
620,272522	435	145,895078	93,5990355	102,250132	32,6110898	109,303291	26,7632363
622,122681	435	142,526993	91,2752239	101,1012	28,785539	110,908839	27,0371753
623,971313	435	135,38753	87,1115924	97,0424466	29,8047697	103,981432	24,7976915
625,818237	435	132,287816	81,571908	96,1198316	27,0899863	101,705969	22,6455632
627,663574	435	125,894334	79,168514	88,5130172	27,0648501	96,3578098	24,2122695
629,507324	435	127,172961	76,5603834	85,9548011	28,5141949	96,0285182	21,774157
631,349365	435	118,259899	72,8847592	88,0644629	25,6816895	89,444015	20,9127471
633,18988	435	115,893765	72,6148667	82,2633451	24,4949133	86,5740414	20,6684127
635,028809	435	108,195669	69,1260844	77,3822781	25,1398958	85,5698068	19,1910715
636,865967	435	111,453964	68,7958017	74,5726466	22,7574254	81,9639655	20,3955212
638,70166	435	102,988043	64,2055408	76,5945437	22,3292301	78,3380829	16,8832705
640,535645	435	101,71968	59,3461654	74,5999461	23,4266977	78,8375333	16,9535616
642,368042	435	102,430966	58,3146436	71,9725306	21,1756142	75,0373736	17,4148786
644,198853	435	95,8674845	57,4087175	71,6862071	21,9581496	72,8423792	15,9060303
646,028015	435	91,3272469	57,3276438	67,642868	18,7376826	69,9772272	15,8507275
647,855591	435	89,5159485	52,9512807	64,8143179	19,9829869	65,6715357	15,6963724
649,681519	435	86,5825316	51,7520138	59,6079204	18,1625576	68,0239672	14,4468815
651,505798	435	84,4797382	49,9544788	57,1760976	19,279278	64,4343198	14,1342634
653,328491	435	81,9752535	49,5110431	58,9931098	17,9947029	61,572947	13,5062502
655,149536	435	77,6230556	49,9906318	50,9737005	17,6853591	56,9277249	13,3306235
656,968994	435	72,9735443	43,2734893	54,566607	15,2000329	58,8929455	11,0544992
658,786743	435	70,448156	43,6323063	54,7528363	17,163162	57,4372174	12,6187546
660,602905	435	71,1461315	42,5987813	50,9462453	13,6418244	55,0735046	10,6036788
662,41748	435	66,8181755	42,028753	48,8938976	14,92116	51,4400718	10,9556358
664,230469	435	64,0491089	37,5343277	48,6094241	12,9673594	49,7879407	10,519868
666,041748	435	63,7071143	38,6728213	44,9579907	11,921174	48,6566512	10,4626952
667,85144	435	62,2717655	35,7902257	42,938481	13,1799904	46,4159907	10,1085568
669,659424	435	56,4814247	34,3271885	43,1792769	12,5157651	42,6729775	7,90379383
671,46582	435	58,3084282	33,6704287	43,3001996	12,3748374	43,0239229	9,21771867
673,27063	435	56,0656857	31,4871308	40,3098212	12,5004897	41,8813043	8,86826031
675,073792	435	53,2334326	29,7693375	36,7579812	10,8236955	41,575565	7,18553412
676,875305	435	52,372386	30,7687017	34,2031483	10,8216435	40,9495763	10,0423908
678,675171	435	49,6726961	27,2941653	36,2163112	9,81833672	36,8604756	6,72174499
680,47345	435	48,784341	28,2777321	35,3398928	9,88809315	35,9057404	7,25703155
682,27002	435	45,927748	26,7476078	32,9072083	9,84483999	34,3255118	8,13434931

This journal is © The Royal Society of Chemistry 20xx

Please do not adjust margins

Please do not adjust margins

684,065063	435	46,6252731	26,058642	31,7736085	10,1108907	35,6834718	6,60683661
685,858398	435	43,2971877	24,5914641	30,8548948	8,71473962	33,5991658	5,98764022
687,650024	435	41,7811985	24,3821977	31,1967349	9,58015887	29,6620341	5,83791062
689,440186	435	41,8777739	23,1112224	31,8926621	9,6803705	31,5253571	5,07121741
691,228577	435	39,3969794	19,7635664	31,440258	7,73248489	28,5741504	4,91759788
693,015381	435	37,459026	19,5785714	24,7152116	9,31809303	29,4290525	5,39955227
694,800476	435	37,1362833	18,0860814	24,9436178	6,65411827	26,9177272	5,24173392
696,583984	435	34,3756456	20,2029578	26,7714856	7,38837239	25,3765045	4,16318175
698,365845	435	33,6833992	18,6830936	24,9572061	6,85738899	24,5023803	6,85310466
700,146118	435	33,4219357	16,6999054	23,2388774	6,72650657	25,3121467	5,69418693
701,924683	435	33,3914174	16,3668534	24,2010422	8,10283942	24,9330302	5,319128
347,741211	440	0	0	0	0	0	0
349,808533	440	0	0	0	0	0	0
351,874329	440	0	0	0	0	0	0
353,938568	440	0	0	0	0	0	0
356,001343	440	0	0	0	0	0	0
358,062561	440	0	0	0	0	0	0
360,122253	440	0	0	0	0	0	0
362,180481	440	0	0	0	0	0	0
364,237152	440	0	0	0	0	0	0
366,292328	440	0	0	0	0	0	0
368,345978	440	0	0	0	0	0	0
370,398132	440	0	0	0	0	0	0
372,44873	440	0	0	0	0	0	0
374,497803	440	0	0	0	0	0	0
376,545349	440	0	0	0	0	0	0
378,5914	440	0	0	0	0	0	0
380,635925	440	0	0	0	0	0	0
382,678955	440	0	0	0	0	0	0
384,720398	440	0	0	0	0	0	0
386,760376	440	0	0	0	0	0	0
388,798767	440	0	0	0	0	0	0
390,835663	440	0	0	0	0	0	0
392,871033	440	0	0	0	0	0	0
394,904877	440	0	0	0	0	0	0
396,937195	440	0	0	0	0	0	0
398,967957	440	0	0	0	0	0	0
400,997253	440	0	0	0	0	0	0
403,024963	440	0	0	0	0	0	0
405,051147	440	0	0	0	0	0	0
407,075806	440	0	0	0	0	0	0
409,098938	440	0	0	0	0	0	0
411,120544	440	0	0	0	0	0	0
413,140564	440	0	0	0	0	0	0
415,159119	440	0	0	0	0	0	0
417,176086	440	0	0	0	0	0	0
419,191528	440	0	0	0	0	0	0

This journal is © The Royal Society of Chemistry 20xx

Please do not adjust margins

Please do not adjust margins

421,205444	440	0	0	0	0	0	0
423,217834	440	0	0	0	0	0	0
425,228668	440	0	0	0	0	0	0
427,237976	440	0	0	0	0	0	0
429,245728	440	0	0	0	0	0	0
431,251953	440	0	0	0	0	0	0
433,256592	440	0	0	0	0	0	0
435,259766	440	0	0	0	0	0	0
437,261353	440	0	0	0	0	0	0
439,261383	440	0	0	0	0	0	0
441,259888	440	0	0	0	0	0	0
443,256836	440	0	0	0	0	0	0
445,252258	440	0	0	0	0	0	0
447,246094	440	0	0	0	0	0	0
449,238434	440	0	0	0	0	0	0
451,229187	440	87,6380628	147,940449	66,8162968	124,762855	90,1573318	67,6427978
453,218445	440	95,8047178	164,249647	67,652609	110,170844	96,4777907	76,4615594
455,206116	440	108,398396	175,607522	76,8595815	105,408419	103,061811	80,882693
457,192261	440	123,118125	194,963064	85,2899503	101,086001	112,070644	79,4858127
459,176819	440	135,087892	212,323981	95,5948885	109,847554	124,597111	87,6006541
461,15979	440	156,146791	228,811677	106,93454	112,806378	137,224566	96,5269462
463,141296	440	163,505082	243,845158	116,249317	110,612346	145,011079	104,846901
465,121216	440	176,872764	257,076666	125,354536	119,057199	160,359973	105,622926
467,099579	440	191,360622	271,280242	136,266364	121,237457	174,904215	112,266609
469,076385	440	208,685681	288,73346	148,41886	125,39424	185,761289	119,630423
471,051636	440	223,008981	304,756232	159,212512	127,72477	198,997628	119,066513
473,02536	440	242,21555	322,60972	170,91414	134,349266	213,807574	127,390403
474,997498	440	253,479439	330,607919	183,30724	136,690795	229,971686	129,329232
476,968079	440	278,957458	351,76267	191,957619	144,986778	239,475103	132,213801
478,937134	440	287,953518	356,979518	202,155628	148,319146	252,945677	133,122054
480,904572	440	303,614378	366,783555	213,422606	147,183587	266,975645	140,041822
482,870453	440	317,337361	388,611505	221,31153	152,951303	281,340226	143,68214
484,834808	440	332,779323	392,974228	231,503066	152,958912	289,441271	151,827998
486,797607	440	348,813887	399,404255	242,883988	157,641481	299,630857	149,955189
488,75885	440	358,16872	415,323766	246,538947	161,344095	307,41605	151,723976
490,718506	440	371,938115	416,608756	251,414663	169,960661	317,994235	153,757202
492,676605	440	376,260119	421,391033	261,808416	173,521582	326,503163	155,435754
494,633118	440	388,105739	420,661482	274,202833	167,830445	338,64601	155,606179
496,588104	440	396,744898	428,016182	273,836427	165,442372	339,985402	157,765061
498,541504	440	413,081524	427,330083	279,301612	162,874534	348,050386	153,37111
500,493347	440	418,001638	434,585867	280,756431	162,895268	354,889797	157,449562
502,443665	440	415,592743	429,963966	287,778316	159,717921	353,388223	158,933883
504,392365	440	425,855787	431,104997	291,784156	155,563995	365,994156	153,604459
506,339539	440	439,127458	429,423299	297,146437	154,062568	360,890014	152,773777
508,285095	440	440,773444	427,14803	293,61535	145,929901	367,843343	153,842805
510,229126	440	438,112211	426,898485	295,471902	140,986204	370,05807	151,914295
512,17157	440	442,956746	424,186652	303,837636	141,754704	368,612163	148,719719

This journal is © The Royal Society of Chemistry 20xx

Please do not adjust margins

Please do not adjust margins

514,112427	440	441,715274	426,749673	301,490911	142,633237	373,500618	146,781029
516,051697	440	445,607027	413,982159	297,634803	141,828789	364,751763	145,872502
517,989441	440	435,422889	406,127352	299,942076	139,119086	365,763334	140,189535
519,925598	440	443,58181	395,413687	298,225923	132,935827	364,959236	135,181631
521,860168	440	436,810533	395,347481	291,830413	127,775465	366,587216	134,518041
523,793152	440	431,438614	379,435778	291,600818	127,615027	361,428137	131,837741
525,724609	440	433,070632	374,062031	289,630224	122,239366	359,607633	124,656698
527,654419	440	429,172168	375,549908	289,766984	121,672821	355,685865	127,614735
529,582764	440	432,044236	371,695049	285,021678	118,795519	356,365616	120,250496
531,50946	440	423,635805	363,982457	286,021376	117,269424	354,208948	118,449713
533,43457	440	426,795756	357,038649	286,519729	116,69844	350,074746	116,978869
535,358093	440	422,361388	353,375071	279,901291	112,188232	348,018825	111,566052
537,280029	440	417,643835	340,836517	274,886245	111,789463	340,855228	111,927583
539,200439	440	410,791833	338,983159	278,25766	109,625475	334,238021	107,622388
541,119263	440	406,971547	328,998319	271,73834	108,367635	328,725619	105,20303
543,036438	440	401,057361	325,121916	267,775681	103,727745	331,304406	102,072444
544,952087	440	396,818842	311,655051	263,719601	101,146618	318,613653	100,045156
546,866211	440	389,941195	306,671875	260,115456	101,153986	315,091476	95,3549358
548,778687	440	378,402884	299,22883	258,185806	98,1471229	311,178247	94,0506053
550,689514	440	374,467666	294,675521	246,570535	95,9498232	307,007792	88,1842616
552,598816	440	369,231727	283,62081	251,215445	91,3612115	300,517995	88,7030919
554,506531	440	363,994412	274,970554	239,128266	90,1697252	290,017606	86,6979593
556,41272	440	358,654621	269,39438	236,449824	89,8674597	285,456536	86,1904652
558,317261	440	353,989827	257,415502	236,863226	82,8162053	278,682875	80,2206277
560,220215	440	337,168825	259,148386	230,398082	84,5248521	277,029864	79,8935569
562,121582	440	339,619135	245,014459	228,422895	79,8702813	271,135175	75,4986598
564,021362	440	326,952874	238,023445	224,539148	76,9670849	255,861391	74,6314541
565,919556	440	325,711949	231,853866	217,487068	76,171882	254,278799	70,8823596
567,816162	440	317,156634	232,132278	210,876196	81,1989423	250,371552	72,8772712
569,711182	440	309,210808	222,052559	207,459048	72,8002605	249,281517	68,7142361
571,604614	440	303,420041	216,287996	205,14897	70,6394251	238,445886	66,2568348
573,49646	440	297,109285	203,911111	198,277659	71,4709654	233,878629	65,6885807
575,386719	440	285,610412	201,380713	196,286557	67,7160999	227,537753	63,0970686
577,27533	440	279,765513	196,476606	190,448252	65,5689338	222,6277	59,734065
579,162354	440	278,074384	189,079427	182,264329	64,8521324	212,562282	58,0172252
581,047852	440	263,67123	186,902391	178,935259	61,2461237	208,576552	54,803446
582,931641	440	261,140007	178,468009	173,805215	58,5007639	198,2996	52,5494599
584,813965	440	250,036826	170,89376	174,252728	61,2628253	200,71571	52,5435181
586,69458	440	243,500294	165,538331	167,07065	58,5436812	191,40606	49,9381616
588,57373	440	232,60582	162,772707	162,040207	53,6525882	182,151929	48,772927
590,451111	440	233,483623	154,705618	158,268586	49,2540102	183,507073	46,9855316
592,326965	440	226,038047	148,79314	148,77293	52,6253262	174,342904	44,3052861
594,201233	440	218,398375	145,027541	144,642399	48,6847893	170,203735	42,5614778
596,073914	440	210,26198	138,940193	143,485773	44,5405699	162,593022	41,5428361
597,944946	440	204,919504	139,40312	136,442039	44,7950158	162,151375	40,8417926
599,814453	440	192,757581	131,293044	137,145539	44,9827117	155,739812	38,1707317
601,682251	440	190,398922	124,980122	126,939716	42,1636404	148,847235	36,9802386

This journal is © The Royal Society of Chemistry 20xx

Please do not adjust margins

603,548523	440	191,525759	121,789744	131,050464	41,9268827	144,577054	38,1456124
605,413147	440	179,159647	120,999703	124,264911	40,1756989	144,843144	33,4761223
607,276245	440	180,271643	115,790802	122,692453	38,5100156	137,548526	36,8553098
609,137695	440	172,276376	111,900458	118,355557	37,5027668	135,657377	33,4454992
610,997437	440	164,817674	105,638065	114,219301	34,2936038	132,205018	30,518309
612,855713	440	164,002641	100,210517	113,605073	36,2768714	125,027423	31,9901638
614,712341	440	161,666574	98,8731806	109,965946	34,7645933	122,656915	28,7077084
616,567322	440	156,716217	96,7114608	104,524891	35,0216997	118,319501	29,3983267
618,420715	440	149,176426	89,7472375	103,067112	30,6707303	115,778872	26,3579239
620,272522	440	144,204406	87,5152429	98,8083198	31,7653781	112,201663	27,8854982
622,122681	440	139,876962	85,1285363	96,5934195	29,5494237	106,106408	25,1436277
623,971313	440	136,347309	83,5908616	95,0873658	27,0441174	106,692379	24,1033964
625,818237	440	127,028711	79,1063858	91,9079499	28,0410922	99,0281964	22,8624494
627,663574	440	124,505022	76,6484445	89,9921923	26,9756851	96,155607	24,0923845
629,507324	440	124,446783	76,504011	85,4402419	27,2579094	94,4636415	22,4923634
631,349365	440	118,04279	75,5157123	82,3695876	25,9327827	90,1242019	22,2098186
633,18988	440	114,352039	72,999451	80,9166779	22,2479019	91,5331262	19,863117
635,028809	440	109,646417	66,8262682	78,7755255	22,9832214	85,1152676	16,5551727
636,865967	440	108,023393	64,9113839	74,2599154	21,4452266	81,4270891	18,3822897
638,70166	440	101,936612	60,377558	72,0537488	20,4066747	78,9211773	18,3173262
640,535645	440	97,0468137	59,1681267	70,3930186	20,1778943	77,1065295	16,5876458
642,368042	440	99,339652	59,0894683	67,4788048	19,3761017	73,9703049	17,734854
644,198853	440	91,7340205	56,2110129	69,6531167	18,2136931	70,5927279	15,6644409
646,028015	440	90,5605207	50,9343969	61,30856	18,3101376	69,2429121	16,9643322
647,855591	440	89,6177418	50,6863884	62,1839015	18,6033535	68,5346432	14,7713188
649,681519	440	87,4500125	50,9578568	62,8949232	18,3243871	65,7216254	15,1535018
651,505798	440	82,7958598	46,6881273	55,4868055	18,399649	63,4066345	13,4659301
653,328491	440	78,8604241	48,4311844	55,2010054	16,320918	60,5516233	11,7522344
655,149536	440	77,23504	43,2572244	49,7466928	15,6912996	59,3172808	13,2501279
656,968994	440	74,4060171	44,9708517	54,1725407	14,5264046	58,1022038	10,9207783
658,786743	440	69,053485	41,7166136	50,1148167	15,8317338	55,7553175	12,4414045
660,602905	440	68,9078618	40,9653015	49,4608148	14,3984607	52,7912534	12,2385262
662,41748	440	68,5182437	40,0623623	46,5778148	12,8302061	50,4625117	10,9707853
664,230469	440	65,3842332	37,9631977	46,1252428	13,0892596	50,2504543	11,7720382
666,041748	440	63,4760589	34,2915757	42,0793994	12,9570367	48,3909077	10,9343898
667,85144	440	60,2099813	32,4321007	46,102025	11,9751579	45,0020337	9,71136817
669,659424	440	56,5715554	31,1668714	39,0707448	10,8231097	43,6035895	9,57418288
671,46582	440	56,1587934	30,258073	40,1296092	12,0118037	40,822032	8,71984352
673,27063	440	54,4890492	30,2979385	39,6573686	10,3249149	43,6615781	8,51741596
675,073792	440	52,1482945	30,6716731	36,9744494	10,897573	40,902979	8,05971693
676,875305	440	53,0060671	28,5373798	35,942756	11,6060836	38,1320338	9,48022232
678,675171	440	48,0443824	26,6496334	33,8199404	10,8972386	37,9012112	8,05946959
680,47345	440	42,9625651	24,0293905	34,739416	8,83738497	34,8740591	8,36913421
682,27002	440	42,0830971	24,5079492	35,492176	7,12607883	34,4406904	7,44740346
684,065063	440	44,5246019	25,9378606	31,4860306	9,77134496	34,5551649	6,83622899
685,858398	440	48,1171769	22,6956572	28,6434245	9,37824496	30,596713	6,91875152
687,650024	440	39,1837178	21,9238599	29,2278428	8,67156072	30,0702508	6,1295757

This journal is © The Royal Society of Chemistry 20xx

Please do not adjust margins

689,440186	440	40,7879029	20,621032	29,693437	7,97394923	29,262674	5,42564401
691,228577	440	38,0183606	19,9142627	26,2832563	7,89809316	29,1972952	5,82745372
693,015381	440	38,5938703	20,6034583	26,9086138	7,03827002	28,8712065	4,64173307
694,800476	440	35,8681562	19,0015437	27,3808581	7,03651203	27,1049705	4,48055389
696,583984	440	33,4463014	17,0742196	24,7662782	7,62689382	26,0904719	4,41830508
698,365845	440	33,540282	17,7652836	26,2826184	6,27967702	24,6342083	6,52524226
700,146118	440	34,9662987	17,674824	23,643927	7,27472074	24,6517716	5,01457836
701,924683	440	33,3173157	18,0668426	21,7096688	6,97812402	24,5031351	6,08934264
347,741211	445	0	0	0	0	0	0
349,808533	445	0	0	0	0	0	0
351,874329	445	0	0	0	0	0	0
353,938568	445	0	0	0	0	0	0
356,001343	445	0	0	0	0	0	0
358,062561	445	0	0	0	0	0	0
360,122253	445	0	0	0	0	0	0
362,180481	445	0	0	0	0	0	0
364,237152	445	0	0	0	0	0	0
366,292328	445	0	0	0	0	0	0
368,345978	445	0	0	0	0	0	0
370,398132	445	0	0	0	0	0	0
372,44873	445	0	0	0	0	0	0
374,497803	445	0	0	0	0	0	0
376,545349	445	0	0	0	0	0	0
378,5914	445	0	0	0	0	0	0
380,635925	445	0	0	0	0	0	0
382,678955	445	0	0	0	0	0	0
384,720398	445	0	0	0	0	0	0
386,760376	445	0	0	0	0	0	0
388,798767	445	0	0	0	0	0	0
390,835663	445	0	0	0	0	0	0
392,871033	445	0	0	0	0	0	0
394,904877	445	0	0	0	0	0	0
396,937195	445	0	0	0	0	0	0
398,967957	445	0	0	0	0	0	0
400,997253	445	0	0	0	0	0	0
403,024963	445	0	0	0	0	0	0
405,051147	445	0	0	0	0	0	0
407,075806	445	0	0	0	0	0	0
409,098938	445	0	0	0	0	0	0
411,120544	445	0	0	0	0	0	0
413,140564	445	0	0	0	0	0	0
415,159119	445	0	0	0	0	0	0
417,176086	445	0	0	0	0	0	0
419,191528	445	0	0	0	0	0	0
421,205444	445	0	0	0	0	0	0
423,217834	445	0	0	0	0	0	0
425,228668	445	0	0	0	0	0	0

Please do not adjust margins

Please do not adjust margins

427,237976	445	0	0	0	0	0	0
429,245728	445	0	0	0	0	0	0
431,251953	445	0	0	0	0	0	0
433,256592	445	0	0	0	0	0	0
435,259766	445	0	0	0	0	0	0
437,261353	445	0	0	0	0	0	0
439,261383	445	0	0	0	0	0	0
441,259888	445	0	0	0	0	0	0
443,256836	445	0	0	0	0	0	0
445,252258	445	0	0	0	0	0	0
447,246094	445	0	0	0	0	0	0
449,238434	445	0	0	0	0	0	0
451,229187	445	0	0	0	0	0	0
453,218445	445	0	0	0	0	0	0
455,206116	445	79,8217339	129,679895	63,6901976	140,928255	84,8281196	65,5736642
457,192261	445	92,2608859	144,590974	65,5355982	114,75107	89,6126105	64,0615539
459,176819	445	109,478746	150,716476	74,583151	99,8234358	100,072049	68,9582679
461,15979	445	117,119373	170,610214	82,1189735	96,1252644	108,748652	74,3737975
463,141296	445	132,179891	182,085625	92,0352195	98,3810872	117,889365	82,6928175
465,121216	445	136,881149	197,096743	102,016953	98,3292893	129,111633	85,0048528
467,099579	445	154,07377	211,138921	112,696988	104,254728	140,74529	89,5736484
469,076385	445	165,456783	219,967289	119,130485	108,117164	156,270197	99,0531799
471,051636	445	183,267859	238,791211	127,189301	112,750281	169,583487	101,280884
473,02536	445	198,810404	250,042806	138,939754	118,679195	180,234298	102,32384
474,997498	445	212,64502	259,830931	149,288155	118,273754	198,177992	109,250994
476,968079	445	226,009722	273,000576	159,363778	119,19968	206,75048	115,405493
478,937134	445	239,956804	290,985587	171,427024	122,261083	217,91935	113,844811
480,904572	445	257,859925	292,285631	177,93687	128,716618	229,696348	116,15569
482,870453	445	275,139287	305,444197	193,914013	126,07934	234,680941	121,889644
484,834808	445	281,61947	318,634564	205,00134	131,223839	254,723996	122,962241
486,797607	445	300,218321	319,958865	203,917662	134,332168	267,732487	132,24978
488,75885	445	308,221512	333,961696	217,561736	138,710262	271,076908	132,363879
490,718506	445	326,820416	345,721656	222,275916	145,344816	283,142986	135,195783
492,676605	445	332,32097	345,582209	231,523801	144,951555	296,900158	137,054714
494,633118	445	346,354709	357,349442	236,889207	147,588891	301,999333	132,768938
496,588104	445	355,208883	359,819395	243,775108	148,373475	309,483526	138,219652
498,541504	445	365,446434	366,727035	250,924534	152,836474	311,466258	139,81406
500,493347	445	371,34359	372,97736	253,111683	149,882613	321,216631	140,815283
502,443665	445	376,575426	366,281359	261,809597	149,198197	325,069602	136,844572
504,392365	445	385,386823	366,908421	259,706267	141,353268	330,512815	134,48579
506,339539	445	392,656184	375,041643	262,735704	140,473344	331,533296	139,818276
508,285095	445	401,022794	372,646667	265,867641	143,517293	337,844057	136,382715
510,229126	445	402,913242	367,77848	262,51772	132,366243	333,931241	134,893233
512,17157	445	405,213523	368,638453	274,039022	130,855224	339,838895	131,42886
514,112427	445	406,211484	363,401847	276,012291	126,939155	343,797219	132,30812
516,051697	445	400,070744	356,901677	270,437297	126,406181	338,661993	135,357768
517,989441	445	408,224799	358,603439	269,367621	123,545463	339,037744	128,985228

This journal is © The Royal Society of Chemistry 20xx

Please do not adjust margins

Please do not adjust margins

519,925598	445	404,944705	355,197049	272,997249	120,462893	339,77691	127,967386
521,860168	445	405,963123	349,362876	267,403072	118,274575	341,426305	123,780817
523,793152	445	396,410115	338,854137	269,594409	118,164851	338,872837	122,392849
525,724609	445	406,897335	341,106974	265,600824	114,931762	329,625176	115,055663
527,654419	445	403,806411	335,243293	266,376271	111,916266	329,912767	119,191164
529,582764	445	403,968898	328,271255	265,95073	107,555954	328,838775	115,5266
531,50946	445	400,924014	323,108429	268,438974	107,037331	329,315105	112,726335
533,43457	445	399,511762	320,618824	261,57888	104,904138	320,275294	110,533426
535,358093	445	386,850913	314,336838	259,51506	107,08123	323,107145	105,386854
537,280029	445	396,124474	311,295329	258,087431	101,857114	322,176223	101,481693
539,200439	445	388,117635	302,623148	257,764634	99,1707663	314,147259	100,041885
541,119263	445	390,667708	300,182787	251,703613	97,8802353	311,314617	100,407718
543,036438	445	387,191188	293,915634	243,284756	97,0123924	310,513767	95,7325567
544,952087	445	373,963557	283,180735	243,100949	95,0680223	301,145816	98,2716482
546,866211	445	364,749014	280,427264	242,246524	94,0100984	298,993765	91,8478314
548,778687	445	363,409413	277,932672	243,249512	91,0547114	290,598682	88,2036066
550,689514	445	354,265033	271,124835	236,913005	89,3355243	290,475356	87,7198897
552,598816	445	357,927659	261,895049	229,006303	83,5784268	282,965245	81,9610065
554,506531	445	348,146107	256,463174	231,839052	85,520551	277,862093	80,0827355
556,41272	445	343,027877	244,831383	223,480027	81,3254906	272,103425	80,1154695
558,317261	445	329,647349	237,400787	221,850489	76,6884258	265,829492	80,45939
560,220215	445	317,740034	236,072284	216,770452	77,1778776	265,802037	76,3730199
562,121582	445	320,781675	223,968383	215,229144	76,5140663	252,107673	71,8734396
564,021362	445	312,64761	224,360778	204,393088	73,3154919	243,488779	72,0239493
565,919556	445	312,692853	212,842263	201,656759	72,9086756	246,096046	68,2362155
567,816162	445	301,671629	210,715152	202,394124	69,9520544	242,408919	65,9528889
569,711182	445	294,916749	208,857771	194,00964	67,9016178	234,836749	63,4015889
571,604614	445	287,540431	194,637816	191,894758	65,7735443	233,040017	61,8770789
573,49646	445	279,587441	189,4213	186,954031	63,8749106	219,836921	60,8024278
575,386719	445	274,378463	188,083997	185,750989	60,9836359	219,216797	57,3388085
577,27533	445	272,706436	185,030872	186,131732	60,4021359	208,659939	57,158799
579,162354	445	264,264556	174,153289	172,800968	61,004586	208,502907	56,6930544
581,047852	445	257,12231	170,901053	173,014059	60,192465	201,065605	53,7353822
582,931641	445	249,180973	166,439514	163,815385	54,5274373	196,672028	50,6872042
584,813965	445	243,992897	161,116439	161,32844	55,2358119	186,946457	53,0920727
586,69458	445	238,020003	155,687139	158,608977	52,3390343	181,447928	48,6885682
588,57373	445	230,222346	152,198599	154,209549	52,6102687	173,505228	45,0714738
590,451111	445	221,522113	141,868843	145,640386	48,6067287	171,455437	45,4055464
592,326965	445	217,664817	131,449023	145,779164	47,6797341	168,075234	41,7373321
594,201233	445	211,201632	130,884175	140,555613	47,5738191	161,927571	42,0958749
596,073914	445	201,892828	129,907146	137,575146	42,3563754	161,323506	40,2485186
597,944946	445	197,257579	125,490583	134,192266	45,2084689	157,394876	38,728297
599,814453	445	190,644399	123,344083	132,913334	41,9958519	150,287628	36,341888
601,682251	445	184,655766	118,996089	129,062353	40,7167573	147,054624	35,7394118
603,548523	445	181,09052	110,127964	120,842219	39,4517789	139,520178	34,9996212
605,413147	445	174,014126	108,912322	119,021918	39,0909507	138,180458	33,9104427
607,276245	445	175,292292	106,692095	117,814522	37,4907839	133,050669	34,5198907

This journal is © The Royal Society of Chemistry 20xx

Please do not adjust margins

Please do not adjust margins

609,137695	445	167,828112	99,3445011	114,262089	33,9157106	130,642017	32,8523073
610,997437	445	162,354224	97,4124025	109,867757	31,8543573	124,803781	31,6682687
612,855713	445	156,090344	98,7697114	106,165925	35,096611	119,631882	30,7242649
614,712341	445	154,520971	92,4092259	105,937376	32,7617735	115,607386	28,0065054
616,567322	445	147,45429	88,011964	99,4228639	32,5758263	113,961171	26,3396088
618,420715	445	144,567041	86,3697695	97,9465376	29,0070948	110,361483	26,9694744
620,272522	445	141,393296	81,5969372	96,3809164	28,9948054	106,37661	27,9153717
622,122681	445	137,095798	78,5848033	89,6999009	27,5747403	103,935013	23,2263391
623,971313	445	127,571937	76,123675	88,9055872	27,352901	100,500025	22,7690513
625,818237	445	125,011991	76,648142	87,39245	26,5654375	94,9221947	23,1577628
627,663574	445	122,686681	75,3623537	80,4497576	26,9859002	91,3483312	23,9468073
629,507324	445	117,498769	69,7229443	81,146268	23,2125048	90,2194179	20,981458
631,349365	445	116,606744	68,6465138	78,3514669	23,9813804	84,7182803	22,1257886
633,18988	445	109,786316	64,6618256	74,3178165	22,0782212	87,1031158	18,7829473
635,028809	445	110,550091	61,9869723	71,714769	22,4415391	82,9056839	18,6209254
636,865967	445	106,345684	57,3332104	72,2060009	20,9687842	80,6222573	18,8016401
638,70166	445	97,486764	58,8743334	66,6651376	20,3010951	76,5879398	19,5674793
640,535645	445	94,0687822	56,0712761	68,5378733	19,5428175	71,1366749	16,9956533
642,368042	445	91,4588229	51,9832228	65,8273485	20,6995599	73,7930758	15,654847
644,198853	445	87,6381274	49,293732	62,9432483	18,851497	65,6174932	15,3096689
646,028015	445	89,5612092	50,6395526	61,1226319	18,2669578	67,8933854	15,8585473
647,855591	445	84,8939964	48,4955984	57,8403379	16,4406077	63,23819	14,4863654
649,681519	445	83,82323	47,1555489	57,9978963	17,3665195	64,2629654	14,6402032
651,505798	445	83,9377521	43,9835299	56,7840239	17,5908108	60,163877	13,934691
653,328491	445	79,1262604	44,1191074	54,6592309	15,3435076	60,2725493	13,0560412
655,149536	445	75,7127721	41,8920888	49,3180913	14,9372494	56,869644	12,7180574
656,968994	445	69,1353868	39,4361555	49,9082138	14,6124145	57,0273137	11,764452
658,786743	445	72,1119033	41,490048	49,2937116	14,9932497	54,8129062	11,43204
660,602905	445	66,5907027	38,8625889	47,3874774	12,7705857	51,6696767	10,7575652
662,41748	445	65,6366358	37,1596282	46,9750593	14,3141191	48,7704575	10,0228548
664,230469	445	65,1383118	36,4491005	43,3869121	13,4802546	49,1804429	9,96417399
666,041748	445	64,3923254	35,8873891	44,3291318	11,777489	48,9558665	10,925572
667,85144	445	58,1045193	32,2850704	41,854738	12,5184567	44,5990229	8,59108662
669,659424	445	58,5387962	31,1672246	39,8902416	9,94048023	42,6369203	8,7665302
671,46582	445	53,8538202	29,7773613	40,7990939	12,1618983	42,4434011	8,93361068
673,27063	445	50,8098329	30,5283954	36,9977441	11,1788762	39,3564536	7,69872314
675,073792	445	52,0212827	24,7080888	36,7514831	11,1209679	40,70425	7,63402743
676,875305	445	48,9893502	26,6892089	33,2525963	10,8805983	35,6094667	8,90467687
678,675171	445	47,1676972	26,6934503	36,9884785	9,21423344	34,9791414	6,99764373
680,47345	445	45,144692	25,3235448	34,2635751	8,81033818	32,0926967	7,2306764
682,27002	445	44,0250439	25,4112986	31,1987617	8,60192629	31,9647698	6,85706626
684,065063	445	42,6509835	23,4177366	31,78158	9,66937611	33,2706787	7,51984467
685,858398	445	43,8705148	23,2680837	27,4016593	9,51372021	29,6846936	6,40267319
687,650024	445	38,7854416	20,5698895	28,4684582	8,56285122	30,8354717	6,40903349
689,440186	445	38,8750925	21,8207882	26,1296892	9,14412525	28,2575969	6,10265531
691,228577	445	38,4924166	20,0657376	25,2604329	6,58058887	27,9502307	6,185995
693,015381	445	36,1728061	17,5415437	25,0830893	7,240851	27,5377501	5,15460047

 This journal is © The Royal Society of Chemistry 20xx

Please do not adjust margins

Please do not adjust margins

694,800476	445	33,9085692	18,4220649	26,1216918	7,2390424	26,8063751	4,83123091
696,583984	445	35,17758	16,1540772	25,66223	7,02598464	24,9747799	4,52734827
698,365845	445	30,8161876	16,4424605	24,0349007	7,20769765	21,6407876	5,5129417
700,146118	445	32,4862304	15,9388855	22,4246708	6,0981616	24,6567335	3,50004844
701,924683	445	29,7504969	15,0205598	20,6349743	6,12160558	21,8924377	4,65743573
347,741211	450	0	0	0	0	0	0
349,808533	450	0	0	0	0	0	0
351,874329	450	0	0	0	0	0	0
353,938568	450	0	0	0	0	0	0
356,001343	450	0	0	0	0	0	0
358,062561	450	0	0	0	0	0	0
360,122253	450	0	0	0	0	0	0
362,180481	450	0	0	0	0	0	0
364,237152	450	0	0	0	0	0	0
366,292328	450	0	0	0	0	0	0
368,345978	450	0	0	0	0	0	0
370,398132	450	0	0	0	0	0	0
372,44873	450	0	0	0	0	0	0
374,497803	450	0	0	0	0	0	0
376,545349	450	0	0	0	0	0	0
378,5914	450	0	0	0	0	0	0
380,635925	450	0	0	0	0	0	0
382,678955	450	0	0	0	0	0	0
384,720398	450	0	0	0	0	0	0
386,760376	450	0	0	0	0	0	0
388,798767	450	0	0	0	0	0	0
390,835663	450	0	0	0	0	0	0
392,871033	450	0	0	0	0	0	0
394,904877	450	0	0	0	0	0	0
396,937195	450	0	0	0	0	0	0
398,967957	450	0	0	0	0	0	0
400,997253	450	0	0	0	0	0	0
403,024963	450	0	0	0	0	0	0
405,051147	450	0	0	0	0	0	0
407,075806	450	0	0	0	0	0	0
409,098938	450	0	0	0	0	0	0
411,120544	450	0	0	0	0	0	0
413,140564	450	0	0	0	0	0	0
415,159119	450	0	0	0	0	0	0
417,176086	450	0	0	0	0	0	0
419,191528	450	0	0	0	0	0	0
421,205444	450	0	0	0	0	0	0
423,217834	450	0	0	0	0	0	0
425,228668	450	0	0	0	0	0	0
427,237976	450	0	0	0	0	0	0
429,245728	450	0	0	0	0	0	0
431,251953	450	0	0	0	0	0	0

This journal is © The Royal Society of Chemistry 20xx

Please do not adjust margins

Please do not adjust margins

433,256592	450	0	0	0	0	0	0
435,259766	450	0	0	0	0	0	0
437,261353	450	0	0	0	0	0	0
439,261383	450	0	0	0	0	0	0
441,259888	450	0	0	0	0	0	0
443,256836	450	0	0	0	0	0	0
445,252258	450	0	0	0	0	0	0
447,246094	450	0	0	0	0	0	0
449,238434	450	0	0	0	0	0	0
451,229187	450	0	0	0	0	0	0
453,218445	450	0	0	0	0	0	0
455,206116	450	0	0	0	0	0	0
457,192261	450	0	0	0	0	0	0
459,176819	450	0	0	0	0	0	0
461,15979	450	85,1897988	121,711268	66,3775893	113,203866	84,2281681	57,8639043
463,141296	450	96,7643697	127,387174	69,2198922	99,3795304	95,0369332	62,102982
465,121216	450	105,523934	143,14022	78,2578832	89,3828622	105,08062	63,1443043
467,099579	450	122,940047	154,476474	87,7547876	86,7158073	111,922069	69,7117116
469,076385	450	131,706894	165,924379	97,3704736	90,0259103	127,241289	71,122095
471,051636	450	149,004125	175,511259	107,513583	92,8611456	136,49521	79,4135883
473,02536	450	153,984049	186,621388	119,370212	98,6749466	150,037377	81,865375
474,997498	450	174,328985	203,319508	126,448866	100,553348	159,906475	87,9983426
476,968079	450	185,480033	213,18425	133,13144	101,009506	171,542303	90,0952317
478,937134	450	200,12474	219,540902	141,860059	104,303088	185,272314	94,1986567
480,904572	450	213,849834	233,558189	154,00164	106,196648	195,200347	95,1385607
482,870453	450	224,160544	242,006837	157,630531	105,719291	200,760859	99,7578572
484,834808	450	239,005682	255,833413	167,023649	110,714724	217,850282	104,973368
486,797607	450	255,298201	258,091605	180,116386	113,796503	230,942652	107,09537
488,75885	450	262,880686	272,324396	186,547923	116,790994	241,917138	108,867445
490,718506	450	275,063128	279,13996	193,652076	117,289256	249,437812	110,97438
492,676605	450	291,733712	285,784489	200,205376	118,805162	256,890274	116,397221
494,633118	450	298,625095	294,116417	208,672484	125,960354	263,108142	119,215248
496,588104	450	309,744322	297,016173	211,293521	122,669165	273,27486	119,869164
498,541504	450	316,463275	297,460518	214,393983	127,349972	279,625278	121,009754
500,493347	450	325,483565	307,306217	218,459073	135,321735	289,415513	122,032997
502,443665	450	329,273582	301,150471	226,536706	132,813955	294,08777	122,292903
504,392365	450	338,748983	307,26436	230,234518	135,497356	298,242843	123,582943
506,339539	450	350,311917	311,238131	232,98957	134,777538	298,927324	119,480826
508,285095	450	349,683823	312,362384	233,83086	125,155403	300,97632	121,378438
510,229126	450	351,512802	306,378126	236,516117	123,263182	303,130284	116,424816
512,17157	450	358,691412	311,840609	244,812841	119,577787	304,049381	115,186993
514,112427	450	357,907687	306,15176	244,164274	119,727527	305,598685	117,256441
516,051697	450	359,635826	308,214312	239,071387	115,903752	309,204516	114,361121
517,989441	450	357,022462	301,969804	240,519149	109,87065	302,777794	115,237894
519,925598	450	363,746209	302,831639	240,865561	104,324366	306,693667	109,846358
521,860168	450	370,685171	297,99834	240,069072	102,293705	308,656686	110,333055
523,793152	450	367,287246	294,274792	242,092034	102,412221	305,139963	109,397269

This journal is © The Royal Society of Chemistry 20xx

Please do not adjust margins

Please do not adjust margins

525,724609	450	368,630147	293,085008	244,333045	106,048763	304,237132	109,412074
527,654419	450	371,776378	293,838104	244,221701	103,42528	303,748543	108,696242
529,582764	450	368,481472	287,071283	248,523959	101,917968	311,444987	103,519098
531,50946	450	368,368295	287,857647	243,440493	99,8649647	312,540676	103,121768
533,43457	450	364,446213	284,224013	239,207261	96,8401984	307,449976	104,560519
535,358093	450	363,952144	274,627198	240,240872	91,3980221	303,007746	101,82468
537,280029	450	355,246276	269,682414	238,165016	88,931264	297,452195	94,5770754
539,200439	450	353,49241	269,017585	234,708076	88,0537619	293,310811	94,0926362
541,119263	450	350,952276	259,165952	229,426726	86,9486099	296,058794	91,4503013
543,036438	450	344,647171	255,137158	227,984681	86,0925534	283,545133	88,7033969
544,952087	450	348,81225	250,480451	229,927846	85,3340239	283,920388	87,3681818
546,866211	450	339,261057	246,309534	219,353487	81,1851809	274,04459	82,6489305
548,778687	450	334,128705	239,579699	220,83278	79,6761872	274,329793	84,39109
550,689514	450	328,06277	232,000551	218,48083	81,2968413	270,834537	81,8842236
552,598816	450	327,591738	230,839811	213,1834	75,6063834	265,345763	76,8172312
554,506531	450	323,128688	221,072612	210,104383	76,0721751	262,434851	75,8511284
556,41272	450	315,801385	219,478044	206,889498	73,5498867	253,150068	73,4544025
558,317261	450	311,42289	217,612448	200,044875	69,3170945	249,550037	71,3400675
560,220215	450	305,201193	203,914278	201,445988	69,9506342	243,367329	71,8491148
562,121582	450	298,659137	202,452047	197,361048	66,222909	238,625212	65,1324001
564,021362	450	290,383268	196,859825	192,21222	65,5486036	235,028106	65,8937355
565,919556	450	289,051306	194,024239	188,420313	66,5519651	230,066962	64,5764409
567,816162	450	282,368117	187,66002	185,453997	63,5382661	224,021467	63,5724983
569,711182	450	277,206503	179,613397	181,082166	61,4128219	221,614832	60,1851575
571,604614	450	276,856323	180,93003	180,881167	58,3888082	216,019471	61,4708577
573,49646	450	267,355208	173,170114	177,529989	59,0696615	211,441523	58,9157221
575,386719	450	252,58369	169,771984	168,534624	58,4116665	209,123999	57,0452824
577,27533	450	251,764777	163,816014	168,940353	57,061419	196,097389	54,8542945
579,162354	450	243,116614	158,174008	167,188618	54,6557353	201,82311	52,5075703
581,047852	450	239,298711	156,944064	160,892752	53,7809171	190,836233	50,2676042
582,931641	450	237,412541	153,317206	156,109696	51,3893548	183,799799	49,8800694
584,813965	450	233,13159	147,802466	155,993182	51,068022	184,467222	47,4173865
586,69458	450	225,389159	141,275286	145,474485	50,5057773	174,567089	46,5196861
588,57373	450	213,20708	137,097058	143,614817	45,4098073	168,489414	43,36473
590,451111	450	213,669548	131,198494	139,767299	45,8851592	163,81924	40,7244466
592,326965	450	203,267854	121,351136	134,435796	46,5885821	154,367046	40,3718503
594,201233	450	195,812069	121,585275	132,095017	41,0731852	155,290302	39,5763903
596,073914	450	192,601768	118,12715	127,571	38,0540312	149,995222	36,750893
597,944946	450	188,614538	114,766516	123,937809	40,2521056	146,481387	38,0024561
599,814453	450	180,55872	112,330054	121,551453	39,0767375	140,40434	35,753562
601,682251	450	175,186277	108,422211	119,851228	36,493462	135,030233	34,3233499
603,548523	450	175,486856	104,554212	114,487948	35,9448981	135,00908	34,4512157
605,413147	450	167,02048	103,435947	115,031586	38,4997728	129,421052	33,1189787
607,276245	450	162,182756	93,4138201	109,297985	33,8872098	128,756024	33,2024923
609,137695	450	155,466686	91,2568162	106,317666	30,6912297	123,211006	31,8803951
610,997437	450	152,537684	93,8543843	103,44626	32,4939516	117,566633	28,3942473
612,855713	450	152,188492	91,1350445	100,803011	29,6908538	117,073957	27,7346707

This journal is © The Royal Society of Chemistry 20xx

Please do not adjust margins

Please do not adjust margins

614,712341	450	145,902579	87,9465576	95,9002515	29,8381599	111,483406	25,7271425
616,567322	450	140,574307	82,1086168	93,1775584	28,0537486	106,698146	25,0150571
618,420715	450	139,085795	78,6512965	92,7082748	27,8929019	108,470071	25,1192057
620,272522	450	134,420606	78,2096318	90,5422515	29,0324884	102,092352	25,1726159
622,122681	450	128,444529	70,852154	88,1660305	25,4412245	99,2036723	22,927335
623,971313	450	124,971821	72,4738038	86,5754247	25,5787077	95,273934	21,3624725
625,818237	450	116,552428	68,8011634	79,6287441	24,862312	92,1125575	23,0127787
627,663574	450	118,886408	66,5289737	80,8605031	24,2918941	86,413613	23,1896402
629,507324	450	111,810856	64,2456038	78,9958703	22,2172454	88,4143936	20,4251464
631,349365	450	110,442795	61,0611158	75,6229248	20,792552	83,4764526	19,6171049
633,18988	450	109,454535	61,3415496	71,9476354	19,7405855	82,3661649	17,8066249
635,028809	450	102,124081	58,136261	68,2612134	20,4161806	79,4333604	16,9714408
636,865967	450	101,524652	57,0561207	66,5110742	20,7241451	76,1218401	17,4802467
638,70166	450	96,3801099	53,5669826	68,201003	19,4266523	71,0035537	18,247293
640,535645	450	91,5789502	50,8654834	64,8134913	20,1118461	68,3765208	15,8343644
642,368042	450	90,7870122	48,7846873	62,9607231	18,8258619	67,4258519	14,5438166
644,198853	450	84,2356771	48,7514382	60,1248672	17,6916417	65,1555917	15,9567288
646,028015	450	84,6814312	47,6986291	58,9490973	16,259183	62,0524198	15,8368955
647,855591	450	81,1637521	45,5986835	56,2259835	15,6296138	64,5807296	13,2031296
649,681519	450	77,9594135	43,4741207	54,4412495	17,868691	61,4546025	11,9534089
651,505798	450	77,7877257	41,2741663	52,6402264	16,0571155	57,6629337	13,6772719
653,328491	450	74,3622546	39,8576115	52,1722194	14,1458061	56,5817508	11,1295388
655,149536	450	73,8541377	41,0587508	46,7389474	14,6188736	52,6457561	12,0832215
656,968994	450	70,6787227	39,884813	47,5672953	15,0321623	52,5814078	11,9920792
658,786743	450	65,9501622	37,7312561	47,0863391	13,969677	54,0376191	10,2776034
660,602905	450	65,7488123	34,1882194	44,4258113	14,9268421	49,5513617	10,7993084
662,41748	450	61,7322536	35,2003446	44,1255765	12,1379161	47,8486026	10,562352
664,230469	450	60,4859304	32,0439059	39,3478239	11,5895437	48,1573684	10,72621
666,041748	450	58,2878775	30,5223983	43,6482788	9,74724834	43,4493559	9,59956678
667,85144	450	57,1460463	30,3902585	40,3904194	10,7801946	43,7841279	10,128804
669,659424	450	53,0583003	28,5759241	37,5920236	11,4502671	42,075171	10,1494222
671,46582	450	53,4027831	28,0994955	36,0445021	11,9665899	39,310312	9,29513587
673,27063	450	48,8044326	27,5569347	34,0145926	9,98710911	40,1871334	7,95714196
675,073792	450	49,4092942	28,33658	34,0800944	9,28124407	37,1505388	7,39264937
676,875305	450	48,5243007	24,3357812	31,6812294	8,91700467	34,7541482	9,0579018
678,675171	450	45,1019302	24,48496	31,1061953	8,19334683	32,08028	6,81262466
680,47345	450	45,0675041	23,9580693	32,0967979	8,98406653	34,588418	6,7350052
682,27002	450	42,2234004	22,3655001	28,9363277	7,99735401	31,5859847	5,66828856
684,065063	450	40,8953293	22,293651	28,7407228	8,46160497	32,8566156	7,07245518
685,858398	450	40,9867086	22,0109195	29,7020349	7,00577719	29,3725507	6,5649483
687,650024	450	36,9835024	19,5440317	27,6861273	8,61982208	28,0155432	5,76829016
689,440186	450	38,1744252	19,4424712	28,3358772	9,29871014	28,2265515	6,07436373
691,228577	450	38,1194724	17,6169379	27,3242417	7,03240401	25,1676434	4,68615087
693,015381	450	32,7458493	16,9991559	24,8963213	5,801708	27,1382618	4,91821252
694,800476	450	34,8807682	16,0384864	23,6419266	6,97499484	25,51826	5,79763792
696,583984	450	31,014848	16,1770875	22,336697	7,07686402	23,9278957	4,27367334
698,365845	450	31,1764031	15,2595614	24,0258243	5,02686406	20,8203902	5,24626579

This journal is © The Royal Society of Chemistry 20xx

Please do not adjust margins

Please do not adjust margins

700,146118	450	29,4333245	15,3948793	20,5591813	6,23445689	23,7203365	3,78349556
701,924683	450	29,3215058	12,9903725	19,9676638	6,48194002	20,6118538	6,9258394
347,741211	455	0	0	0	0	0	0
349,808533	455	0	0	0	0	0	0
351,874329	455	0	0	0	0	0	0
353,938568	455	0	0	0	0	0	0
356,001343	455	0	0	0	0	0	0
358,062561	455	0	0	0	0	0	0
360,122253	455	0	0	0	0	0	0
362,180481	455	0	0	0	0	0	0
364,237152	455	0	0	0	0	0	0
366,292328	455	0	0	0	0	0	0
368,345978	455	0	0	0	0	0	0
370,398132	455	0	0	0	0	0	0
372,44873	455	0	0	0	0	0	0
374,497803	455	0	0	0	0	0	0
376,545349	455	0	0	0	0	0	0
378,5914	455	0	0	0	0	0	0
380,635925	455	0	0	0	0	0	0
382,678955	455	0	0	0	0	0	0
384,720398	455	0	0	0	0	0	0
386,760376	455	0	0	0	0	0	0
388,798767	455	0	0	0	0	0	0
390,835663	455	0	0	0	0	0	0
392,871033	455	0	0	0	0	0	0
394,904877	455	0	0	0	0	0	0
396,937195	455	0	0	0	0	0	0
398,967957	455	0	0	0	0	0	0
400,997253	455	0	0	0	0	0	0
403,024963	455	0	0	0	0	0	0
405,051147	455	0	0	0	0	0	0
407,075806	455	0	0	0	0	0	0
409,098938	455	0	0	0	0	0	0
411,120544	455	0	0	0	0	0	0
413,140564	455	0	0	0	0	0	0
415,159119	455	0	0	0	0	0	0
417,176086	455	0	0	0	0	0	0
419,191528	455	0	0	0	0	0	0
421,205444	455	0	0	0	0	0	0
423,217834	455	0	0	0	0	0	0
425,228668	455	0	0	0	0	0	0
427,237976	455	0	0	0	0	0	0
429,245728	455	0	0	0	0	0	0
431,251953	455	0	0	0	0	0	0
433,256592	455	0	0	0	0	0	0
435,259766	455	0	0	0	0	0	0
437,261353	455	0	0	0	0	0	0

This journal is © The Royal Society of Chemistry 20xx

Please do not adjust margins

439,261383	455	0	0	0	0	0	0
441,259888	455	0	0	0	0	0	0
443,256836	455	0	0	0	0	0	0
445,252258	455	0	0	0	0	0	0
447,246094	455	0	0	0	0	0	0
449,238434	455	0	0	0	0	0	0
451,229187	455	0	0	0	0	0	0
453,218445	455	0	0	0	0	0	0
455,206116	455	0	0	0	0	0	0
457,192261	455	0	0	0	0	0	0
459,176819	455	0	0	0	0	0	0
461,15979	455	0	0	0	0	0	0
463,141296	455	0	0	0	0	0	0
465,121216	455	84,2200513	103,068096	65,5785814	129,816073	90,673622	55,3162515
467,099579	455	91,274076	113,072626	72,0881237	103,681935	96,7064847	54,3007918
469,076385	455	104,456989	122,475722	79,3985301	87,8669928	106,715084	58,1323533
471,051636	455	117,759011	135,723314	87,7677776	80,2537464	114,675714	58,7423135
473,02536	455	125,863234	141,989715	96,2019241	82,4542158	130,626655	63,5399913
474,997498	455	139,590269	152,353155	105,01082	84,1660479	138,9823	67,1882454
476,968079	455	154,533708	161,778923	112,648432	88,8732381	148,413787	73,8847567
478,937134	455	166,675884	171,767943	119,781403	90,0474913	160,265731	74,5540346
480,904572	455	177,426916	182,145718	132,121687	90,1622749	169,828529	77,669395
482,870453	455	191,581838	189,564018	141,51587	90,3660196	182,009788	83,710059
484,834808	455	197,279025	202,483223	145,878577	95,8353759	192,449333	85,4779277
486,797607	455	219,22984	213,459945	155,421649	94,6889808	203,043598	88,5030839
488,75885	455	226,861219	220,059929	156,850835	98,5450282	211,258332	92,3934132
490,718506	455	235,453167	223,426574	167,350042	100,418324	222,990012	96,6834709
492,676605	455	249,387541	232,776112	178,785599	103,387847	233,263559	101,718225
494,633118	455	261,161026	237,191693	182,862973	105,546017	238,311598	99,5338006
496,588104	455	269,304168	243,640493	186,919855	106,846007	245,108413	101,912488
498,541504	455	285,524955	249,502292	192,07096	108,563585	251,036828	103,794421
500,493347	455	288,444779	257,423632	199,469626	112,530076	259,643661	105,656225
502,443665	455	291,719205	258,591723	202,162186	116,491286	267,394105	106,907998
504,392365	455	303,493153	260,304092	205,701748	116,929615	269,979944	106,544496
506,339539	455	307,517547	268,063926	206,748655	123,224494	275,834465	109,253105
508,285095	455	314,174843	265,224693	214,726311	118,860451	280,114482	109,971736
510,229126	455	313,618444	261,149121	211,364707	118,751901	283,209857	108,132242
512,17157	455	319,351509	264,458773	215,71233	114,016863	282,663234	101,409413
514,112427	455	327,775214	270,749145	217,302964	111,22342	285,395495	103,808449
516,051697	455	328,540446	263,347079	220,014935	107,816575	287,4306	101,7128
517,989441	455	328,294634	262,361421	220,347861	106,649749	289,480345	101,099504
519,925598	455	331,674221	256,6586	222,305175	100,592454	282,012978	98,4017612
521,860168	455	332,852872	260,51079	219,612398	95,5141331	290,718552	96,4422993
523,793152	455	330,339275	254,303273	222,881549	89,6854341	286,418464	98,922947
525,724609	455	328,466971	256,044423	224,257324	89,2458738	281,773877	95,24232
527,654419	455	335,475385	253,565916	221,392725	90,7243284	286,626976	96,3480587
529,582764	455	336,949537	254,27064	223,488561	91,9519483	285,874138	93,8402082

 This journal is © The Royal Society of Chemistry 20xx

Please do not adjust margins

531,50946	455	339,91778	252,659036	221,486217	89,321301	282,072581	95,7363632
533,43457	455	335,046267	249,727064	224,629644	91,2031206	284,578356	93,487607
535,358093	455	336,455442	243,27373	218,840546	86,7898302	282,596379	92,48655
537,280029	455	336,337551	244,82947	225,949922	85,7212403	281,868469	89,3354582
539,200439	455	329,882593	237,973968	218,738661	82,3712977	279,536997	91,0828335
541,119263	455	331,784578	236,0074	217,93829	82,4306648	275,15964	86,4210904
543,036438	455	323,231867	234,286014	212,175973	79,9542419	271,202983	81,1819629
544,952087	455	322,62957	227,435788	206,387097	75,1919187	268,978713	85,5539025
546,866211	455	315,984374	225,680897	205,429192	77,3228573	260,224439	79,1742042
548,778687	455	309,916206	215,805878	205,78985	72,228683	257,781799	77,6390481
550,689514	455	308,594847	213,048463	201,910046	70,3989989	254,765222	75,2908663
552,598816	455	300,773803	210,915815	199,428522	69,774	247,456986	72,8784074
554,506531	455	298,241957	201,841138	195,677722	70,0607042	252,732848	74,4309823
556,41272	455	300,831773	198,695395	190,699367	67,4301254	241,435725	69,4550648
558,317261	455	293,310318	200,916907	189,69341	64,8361759	235,552596	68,3798095
560,220215	455	285,44396	190,311597	190,544683	63,8329156	228,430533	65,4746941
562,121582	455	281,520763	185,876663	184,404563	61,5512467	229,846517	61,7969395
564,021362	455	277,206943	176,180886	179,920225	61,0497083	222,546428	61,4859215
565,919556	455	272,915508	178,361537	180,013681	60,766499	219,968643	60,4376279
567,816162	455	262,465524	175,819576	169,871902	60,6270478	219,213665	59,5920598
569,711182	455	263,599237	169,664108	171,465816	58,9025875	211,914157	57,5409569
571,604614	455	256,272533	164,234563	168,821937	58,3287222	208,026479	55,1519548
573,49646	455	256,185039	160,964927	167,896428	53,1290331	201,517217	53,5652667
575,386719	455	248,238639	158,318879	158,598208	54,1922556	198,597835	51,9022922
577,27533	455	240,722823	151,745176	158,876737	52,8564601	189,302058	51,9241255
579,162354	455	233,784124	150,036347	155,099927	49,8718262	189,733039	49,8510103
581,047852	455	229,028348	143,490083	153,176285	48,6962294	182,227801	47,2927996
582,931641	455	223,774495	142,11075	145,563799	49,4192472	176,706218	45,9774063
584,813965	455	219,807025	137,536412	144,754243	45,3297691	176,304394	44,3812101
586,69458	455	208,179837	130,812609	140,831647	46,522704	167,643153	44,9769715
588,57373	455	204,478658	127,041087	136,387249	45,4352797	164,325704	42,5506388
590,451111	455	198,95363	119,006406	133,065413	41,0861765	156,755952	40,250892
592,326965	455	194,228913	115,163701	126,269878	40,5883493	153,460027	38,115348
594,201233	455	192,482202	116,289661	125,170592	37,246539	142,540987	38,3059693
596,073914	455	189,272431	107,913254	119,962522	38,963058	144,886066	34,9328139
597,944946	455	178,721747	104,240195	117,930933	35,4889858	140,971112	34,557046
599,814453	455	174,045888	108,451604	117,925142	38,1493177	135,557442	32,8268255
601,682251	455	173,704431	101,352675	115,459915	35,2529332	128,938505	32,6124922
603,548523	455	166,163819	96,2868465	111,665228	34,2893649	126,202193	32,2690908
605,413147	455	160,975056	93,7374961	109,364814	35,6631921	127,898095	30,4398417
607,276245	455	155,150278	91,008316	106,410734	32,4770014	126,000873	31,2794593
609,137695	455	150,557979	86,9233637	100,54083	30,5904853	116,146466	28,236506
610,997437	455	148,133095	85,8016644	97,1347892	31,4003122	117,194106	28,019725
612,855713	455	145,463028	84,4023701	98,0547743	29,0950484	112,192886	30,1441114
614,712341	455	142,304684	78,2777006	92,3833132	27,6704574	106,959642	27,5584869
616,567322	455	136,783701	80,0711593	87,9744402	27,4949635	105,569217	23,8229394
618,420715	455	129,65992	72,3823571	86,3937872	27,1929369	101,2023	24,7492371

This journal is © The Royal Society of Chemistry 20xx

Please do not adjust margins

Please do not adjust margins

620,272522	455	127,465875	72,9480094	85,9605419	26,4270559	99,2888838	22,5346545
622,122681	455	123,202802	71,2482655	82,9169347	25,796078	95,5804215	23,2028494
623,971313	455	117,404384	68,133166	83,2127715	23,3834828	93,2200065	20,780931
625,818237	455	115,168679	65,4251659	79,2536128	24,2375625	91,3020626	20,3084849
627,663574	455	114,053784	61,4482444	78,0073591	24,0720516	87,7846209	21,5245624
629,507324	455	109,738287	60,7751533	72,0562	23,7723344	81,9406867	19,5372975
631,349365	455	105,775032	60,4712077	71,5519403	21,7666709	78,0929423	20,1801443
633,18988	455	101,898566	58,2354601	69,3446859	20,4888698	81,0733466	19,5273627
635,028809	455	100,021527	55,8949293	69,1635866	21,2453496	78,3682577	17,9768017
636,865967	455	96,6131457	54,0946105	67,1768011	17,5693664	70,8609829	17,5150505
638,70166	455	92,1164441	49,7131846	62,8773681	18,8396742	69,4987611	16,9638913
640,535645	455	88,9502253	50,1940938	61,6770331	17,6458213	69,6961354	16,2599171
642,368042	455	86,6750973	47,8715293	59,1608949	17,5268746	66,6242539	15,1567899
644,198853	455	82,5786746	44,6734674	60,4111531	15,6677013	65,2217846	16,1036717
646,028015	455	80,5652744	43,3890755	53,7375789	15,5451666	62,6938718	15,3478875
647,855591	455	78,2811892	43,1591174	54,9075877	16,803895	58,5870108	14,7077876
649,681519	455	80,254679	38,9381748	50,4721099	16,4697795	60,8099431	11,6736002
651,505798	455	75,618719	39,8786444	50,0414863	14,4081421	55,0247311	12,5740221
653,328491	455	70,6574559	38,2221395	48,7737616	12,4590988	54,126065	11,187608
655,149536	455	69,3429353	37,3608407	47,3810257	13,2903301	54,6800045	11,0851999
656,968994	455	66,187499	35,5067914	46,3587736	13,9192499	52,1068723	11,274642
658,786743	455	61,9656496	33,9473144	45,7892003	13,6976476	48,9673323	11,5451978
660,602905	455	62,9070347	32,9441162	42,5059514	11,4899468	48,7219958	9,90901455
662,41748	455	62,3190451	34,6160863	40,3047104	10,8882659	46,7624878	11,2612906
664,230469	455	60,1158853	28,2128801	41,183209	11,2006775	43,799015	8,88121551
666,041748	455	56,2202586	30,8868313	40,0941052	11,0025239	43,6602759	9,9184381
667,85144	455	55,4254888	30,3143188	39,7665254	11,9826104	40,0527583	8,84875085
669,659424	455	52,099182	28,2533647	34,2907624	10,3963068	41,6722828	8,2072521
671,46582	455	51,3467255	26,6703109	35,4236644	10,1870718	37,4627583	9,68272298
673,27063	455	50,5919667	25,8921281	33,7990383	9,85911099	38,6399488	8,39511821
675,073792	455	49,2158627	21,8885528	32,3917717	9,2146424	36,7233335	7,96858547
676,875305	455	47,2176456	23,4317853	32,3120272	9,06548917	34,062176	8,92607453
678,675171	455	46,4884077	24,2461713	31,8018545	8,69835548	32,6665412	5,68113196
680,47345	455	43,6554413	22,533937	30,3799552	8,47074023	32,5679239	6,48776637
682,27002	455	40,4085603	20,0995769	29,7470898	7,90878305	31,9413975	7,17606933
684,065063	455	40,172278	20,0922431	28,5128934	8,30586808	32,0475648	6,08653138
685,858398	455	37,965355	20,6944395	27,0435534	5,78697945	30,4288455	6,53475058
687,650024	455	36,2959	18,6360725	27,4412457	7,27804304	26,4473902	5,64925454
689,440186	455	36,8261742	18,6047301	26,6869684	8,48586444	26,4340601	5,21528787
691,228577	455	34,2391384	16,7524941	26,1051083	6,77713632	24,8080909	4,77060886
693,015381	455	32,0124183	18,1384188	24,1579228	5,89831708	28,6051402	5,60468611
694,800476	455	32,3028319	15,5966364	20,8720026	7,83757723	25,6859279	5,45386522
696,583984	455	32,5830166	14,9852499	21,854369	5,17119551	23,8403852	4,0506492
698,365845	455	29,5948992	14,2008522	21,8407235	5,11057057	19,9979922	4,28769949
700,146118	455	28,9589613	12,4470851	19,4412797	5,88553832	22,266804	4,53139422
701,924683	455	28,5403649	14,3123825	19,5160204	6,135402	22,2766364	5,45857784
347,741211	460	0	0	0	0	0	0

This journal is © The Royal Society of Chemistry 20xx

Please do not adjust margins

Please do not adjust margins

349,808533	460	0	0	0	0	0	0
351,874329	460	0	0	0	0	0	0
353,938568	460	0	0	0	0	0	0
356,001343	460	0	0	0	0	0	0
358,062561	460	0	0	0	0	0	0
360,122253	460	0	0	0	0	0	0
362,180481	460	0	0	0	0	0	0
364,237152	460	0	0	0	0	0	0
366,292328	460	0	0	0	0	0	0
368,345978	460	0	0	0	0	0	0
370,398132	460	0	0	0	0	0	0
372,44873	460	0	0	0	0	0	0
374,497803	460	0	0	0	0	0	0
376,545349	460	0	0	0	0	0	0
378,5914	460	0	0	0	0	0	0
380,635925	460	0	0	0	0	0	0
382,678955	460	0	0	0	0	0	0
384,720398	460	0	0	0	0	0	0
386,760376	460	0	0	0	0	0	0
388,798767	460	0	0	0	0	0	0
390,835663	460	0	0	0	0	0	0
392,871033	460	0	0	0	0	0	0
394,904877	460	0	0	0	0	0	0
396,937195	460	0	0	0	0	0	0
398,967957	460	0	0	0	0	0	0
400,997253	460	0	0	0	0	0	0
403,024963	460	0	0	0	0	0	0
405,051147	460	0	0	0	0	0	0
407,075806	460	0	0	0	0	0	0
409,098938	460	0	0	0	0	0	0
411,120544	460	0	0	0	0	0	0
413,140564	460	0	0	0	0	0	0
415,159119	460	0	0	0	0	0	0
417,176086	460	0	0	0	0	0	0
419,191528	460	0	0	0	0	0	0
421,205444	460	0	0	0	0	0	0
423,217834	460	0	0	0	0	0	0
425,228668	460	0	0	0	0	0	0
427,237976	460	0	0	0	0	0	0
429,245728	460	0	0	0	0	0	0
431,251953	460	0	0	0	0	0	0
433,256592	460	0	0	0	0	0	0
435,259766	460	0	0	0	0	0	0
437,261353	460	0	0	0	0	0	0
439,261383	460	0	0	0	0	0	0
441,259888	460	0	0	0	0	0	0
443,256836	460	0	0	0	0	0	0

Please do not adjust margins

445,252258	460	0	0	0	0	0	0
447,246094	460	0	0	0	0	0	0
449,238434	460	0	0	0	0	0	0
451,229187	460	0	0	0	0	0	0
453,218445	460	0	0	0	0	0	0
455,206116	460	0	0	0	0	0	0
457,192261	460	0	0	0	0	0	0
459,176819	460	0	0	0	0	0	0
461,15979	460	0	0	0	0	0	0
463,141296	460	0	0	0	0	0	0
465,121216	460	0	0	0	0	0	0
467,099579	460	0	0	0	0	0	0
469,076385	460	0	0	0	0	0	0
471,051636	460	93,077131	98,7176284	76,3595213	107,706656	105,786361	55,7975202
473,02536	460	105,832916	104,320424	86,7508883	89,9955039	110,29957	51,2258032
474,997498	460	116,014326	113,242405	88,791394	78,2850933	124,44497	53,9466983
476,968079	460	126,360948	125,527772	95,2360939	72,9422776	132,566233	55,2464333
478,937134	460	133,832499	131,275518	104,006357	74,583421	140,296263	59,042714
480,904572	460	148,927678	137,044226	111,093248	75,2106007	151,562618	63,2089404
482,870453	460	158,403484	146,73836	120,362053	76,6590195	161,411785	61,82365
484,834808	460	173,589643	159,332007	126,184462	84,9498876	171,577803	68,9821274
486,797607	460	184,288175	160,968465	136,147787	83,5429217	176,758382	74,006863
488,75885	460	188,186712	172,810281	141,75315	87,2483583	193,633578	73,2384205
490,718506	460	203,729631	178,154153	144,832514	85,8719161	200,362605	76,5936199
492,676605	460	218,292348	186,433668	153,662396	86,1981039	203,023841	78,1706172
494,633118	460	226,145612	191,731951	164,567902	90,5766366	213,653875	81,7792257
496,588104	460	233,1757	198,517048	164,297385	93,5251375	222,781078	84,4364982
498,541504	460	242,887577	202,347508	166,166667	93,2153297	229,26879	88,0706045
500,493347	460	251,282262	207,180445	175,372827	97,2584889	231,61408	87,8107355
502,443665	460	261,157368	213,856366	178,015705	96,047164	236,064278	90,3069549
504,392365	460	266,165842	217,649383	184,749523	97,3444002	245,414928	87,8334924
506,339539	460	269,808207	224,0578	187,131289	100,600793	245,512307	90,1910764
508,285095	460	278,633903	219,857864	189,134257	103,749908	256,700524	92,5622459
510,229126	460	282,130995	220,796743	188,490036	108,131615	253,611764	92,8313713
512,17157	460	285,605403	226,819035	195,083087	110,634604	262,736818	92,3758048
514,112427	460	291,295553	223,020561	195,017015	109,480821	259,23757	90,2539715
516,051697	460	292,834549	227,620895	199,02038	101,311072	256,694595	92,7210245
517,989441	460	291,446859	228,981541	195,890975	100,081305	261,849504	88,9100475
519,925598	460	302,427698	222,953231	198,792279	97,2995903	264,266156	87,1412517
521,860168	460	303,014516	222,979304	196,912649	95,538449	263,018222	86,7920265
523,793152	460	298,288202	225,788319	201,688651	92,8480438	260,589027	85,0669176
525,724609	460	303,66586	221,490779	198,441238	86,4115406	263,849234	83,4872408
527,654419	460	304,929659	221,042976	198,497274	86,9028001	258,526196	86,7915768
529,582764	460	307,865502	217,336901	196,55902	79,7868768	261,787556	83,1760339
531,50946	460	306,783287	220,237779	202,053935	82,4306661	260,358831	82,4447858
533,43457	460	308,64103	214,617358	204,291495	78,3601767	261,554937	81,45762
535,358093	460	308,113032	219,980431	201,444114	79,6531044	262,708339	82,3100582

This journal is © The Royal Society of Chemistry 20xx

Please do not adjust margins

537,280029	460	307,810855	216,673761	200,788079	76,5056978	262,041734	80,9271073
539,200439	460	315,083249	212,123676	202,710342	76,309199	258,519988	80,2502819
541,119263	460	306,791726	208,226824	205,14713	71,913974	258,964526	78,4065337
543,036438	460	305,847116	208,537663	196,901072	74,6871391	259,474475	75,9880682
544,952087	460	300,34243	202,374496	198,036287	69,8534311	250,415528	75,2256519
546,866211	460	294,818321	201,556488	191,848572	69,286958	244,590372	73,726423
548,778687	460	295,943055	192,017529	190,134134	67,5844385	243,718465	70,2692212
550,689514	460	289,829916	187,714643	186,262623	66,8715231	234,380741	69,0689293
552,598816	460	287,811477	189,101611	186,686132	65,0102653	236,368325	67,8659563
554,506531	460	278,258365	183,287614	183,833982	62,450768	229,938982	66,451371
556,41272	460	280,791153	178,049427	181,436904	61,1495782	227,138715	65,0320374
558,317261	460	270,941684	175,518911	173,84458	58,8597487	221,477467	59,4889281
560,220215	460	269,894536	170,171406	177,429528	59,471955	217,289928	61,3065192
562,121582	460	263,169355	168,701653	172,290533	57,779551	212,111112	60,4041359
564,021362	460	256,916168	166,107447	167,586508	57,4219013	214,954528	59,9892502
565,919556	460	253,020518	161,726158	166,501587	55,7503699	206,132391	57,1766158
567,816162	460	245,517452	159,764989	160,71667	55,7331755	202,050594	56,005865
569,711182	460	241,552107	149,395765	159,296083	51,5143566	201,074004	52,0350713
571,604614	460	241,405956	150,559946	160,135464	51,7460584	197,308376	50,4879115
573,49646	460	238,963176	144,70817	154,135092	51,0352496	185,809218	49,7122988
575,386719	460	232,033642	142,839396	149,515321	51,060134	185,54644	51,2161394
577,27533	460	226,006657	138,20478	148,963603	48,5417289	178,976796	46,9608995
579,162354	460	221,363801	132,998027	146,53859	47,002699	178,261484	47,1002977
581,047852	460	220,074344	132,101936	143,571956	43,0642329	174,548783	45,7294586
582,931641	460	214,488317	128,091593	137,689253	42,7513802	170,293477	42,7268174
584,813965	460	204,153247	120,505015	134,467186	42,7987016	166,214234	42,021488
586,69458	460	199,335565	119,983267	132,47791	42,9267308	162,683693	42,2100985
588,57373	460	195,102395	119,591563	127,91378	41,6307417	154,860159	38,6981392
590,451111	460	187,002403	114,02276	122,68394	40,0909223	152,171278	38,5366312
592,326965	460	184,329535	107,132328	121,900532	39,3045664	146,355417	36,9598963
594,201233	460	181,865747	105,185143	115,853325	36,5827954	146,441148	35,4558156
596,073914	460	172,667938	105,293707	115,84365	36,5865917	137,279113	35,0583879
597,944946	460	173,825747	98,9023651	114,428939	34,4572633	133,88008	35,2885821
599,814453	460	165,941901	95,4616031	109,713926	35,2904254	129,053643	31,6125042
601,682251	460	162,543471	91,9077495	104,109898	32,4689708	129,401623	31,0167677
603,548523	460	155,931552	89,235516	101,609855	28,8861621	124,700737	30,0399535
605,413147	460	154,690098	85,7678982	100,853055	30,0711935	120,982502	27,7820219
607,276245	460	154,259744	83,7546066	96,8739972	31,7115562	117,102583	27,9990211
609,137695	460	146,485057	79,409304	98,1458932	28,4890311	111,674531	28,0843992
610,997437	460	140,919752	78,9819886	93,2805299	27,8318121	111,70108	27,2322727
612,855713	460	137,586736	78,4909512	92,600698	28,4260471	106,810076	25,4909086
614,712341	460	134,956009	73,0997044	88,6862413	27,3313923	106,903042	25,2344938
616,567322	460	131,899695	72,0693859	86,045664	28,0773112	98,3547246	23,0479293
618,420715	460	126,270905	70,41186	83,56296	24,7913602	99,5431377	21,9081871
620,272522	460	123,326733	67,8778783	80,2759583	26,1516561	95,1788852	23,3922845
622,122681	460	118,27019	65,4001917	79,5971962	23,8492622	89,9464726	22,7878428
623,971313	460	112,532442	64,5267742	75,1509718	23,3232465	86,2512523	20,4207385

This journal is © The Royal Society of Chemistry 20xx

Please do not adjust margins

Please do not adjust margins

625,818237	460	108,478862	58,9043272	71,8204059	21,9642451	85,9982618	19,2488729
627,663574	460	108,271975	58,8094124	73,0037194	23,7207944	80,3356646	19,2166719
629,507324	460	105,276078	56,9717712	68,1847061	20,3184244	79,0051856	19,4440455
631,349365	460	103,82101	54,818365	67,9027544	19,3366643	76,9373391	17,9309268
633,18988	460	98,118874	54,4795972	68,4608955	17,7167138	76,1180753	18,0393643
635,028809	460	95,5902709	49,448788	64,2934162	19,6364721	74,2089608	17,7624601
636,865967	460	96,4781763	48,9936602	59,7295806	18,8010731	70,8609266	15,6585591
638,70166	460	88,3698878	44,9466804	61,5632995	16,8101969	68,3653685	15,8671754
640,535645	460	86,3471394	43,9368257	60,4183153	16,0734657	67,6859552	14,8644127
642,368042	460	83,9735252	43,4549522	59,6899299	16,1596142	64,5627664	14,8155917
644,198853	460	83,0809476	42,2757409	55,5363829	15,1237681	60,3528999	12,9080098
646,028015	460	79,3475243	42,8613073	51,3134192	15,00729	60,7577354	14,3290842
647,855591	460	77,5665398	37,5458147	51,7595664	13,3265055	59,5927255	14,5235688
649,681519	460	74,20103	36,8706369	49,680654	13,5998583	54,9977732	12,3808723
651,505798	460	69,2856376	36,3452516	46,9781411	13,6557157	55,2912532	13,1073601
653,328491	460	70,587258	36,2536123	47,3263577	11,8669136	53,0208296	10,436884
655,149536	460	67,4993348	33,7279132	44,5040619	13,9517227	48,5861203	10,8828931
656,968994	460	62,9810637	33,1831256	42,5014323	13,4626787	49,2652343	11,8157628
658,786743	460	61,0796283	32,1736024	43,3232298	10,7806064	47,991105	10,2940247
660,602905	460	63,8473684	32,1120667	40,6042957	10,7373384	46,7973208	9,90569474
662,41748	460	60,3901337	30,387392	38,8510583	11,4078172	46,1088699	10,3647844
664,230469	460	57,1746854	29,2682982	39,0662526	11,6381514	44,8145497	9,20824293
666,041748	460	55,8870639	29,1132603	36,9863657	10,7571747	43,5778902	9,15568536
667,85144	460	51,0158754	27,8694905	38,131793	10,3691942	37,7136061	7,30295198
669,659424	460	49,0917183	25,6921241	34,6534287	9,34429828	39,254579	7,80567245
671,46582	460	48,6518938	24,8098632	33,6419131	9,00482817	36,7122919	8,37177159
673,27063	460	46,874772	23,0828965	32,021984	10,2314448	37,2765555	7,63415567
675,073792	460	47,5283374	22,6352759	30,1179108	8,49581397	34,6806003	6,31556979
676,875305	460	45,2665589	23,1229627	28,9891353	8,98560353	32,7086962	7,92804545
678,675171	460	41,9644738	20,0337133	31,0998253	9,40829863	32,9244988	6,24520513
680,47345	460	40,3142442	22,0553838	28,6910148	7,71733511	32,5486281	6,38067276
682,27002	460	38,688939	19,5944072	27,9457307	7,11047165	31,0424263	6,05098243
684,065063	460	39,1717085	19,2352289	25,7079513	8,3348956	28,7443571	5,43574766
685,858398	460	37,8424261	19,0314144	24,5887052	7,91455517	29,5341723	5,65000563
687,650024	460	33,2691545	17,9880344	23,9058573	8,55904017	27,1588235	5,58492302
689,440186	460	35,6923656	17,4617511	25,3080412	6,80633972	25,3784708	4,3932355
691,228577	460	34,0738117	17,3283058	24,5403078	7,02249073	25,1072575	6,16773404
693,015381	460	30,0236506	14,8801997	23,6779629	7,18246551	26,1661621	5,33044963
694,800476	460	32,5115559	16,2288905	21,8237806	6,39861816	23,5294466	4,76067893
696,583984	460	30,0657148	15,150926	18,8427754	6,42444369	22,196957	3,99513491
698,365845	460	30,3655549	13,0434751	21,6155603	5,4403297	21,0425803	6,58187849
700,146118	460	29,3336682	11,5845432	17,5339795	5,46205831	20,3361721	4,38150166
701,924683	460	27,5653402	12,5680732	20,5589979	4,97803844	19,692998	4,47045008
347,741211	465	0	0	0	0	0	0
349,808533	465	0	0	0	0	0	0
351,874329	465	0	0	0	0	0	0
353,938568	465	0	0	0	0	0	0

This journal is © The Royal Society of Chemistry 20xx

Please do not adjust margins

Please do not adjust margins

356,001343	465	0	0	0	0	0	0
358,062561	465	0	0	0	0	0	0
360,122253	465	0	0	0	0	0	0
362,180481	465	0	0	0	0	0	0
364,237152	465	0	0	0	0	0	0
366,292328	465	0	0	0	0	0	0
368,345978	465	0	0	0	0	0	0
370,398132	465	0	0	0	0	0	0
372,44873	465	0	0	0	0	0	0
374,497803	465	0	0	0	0	0	0
376,545349	465	0	0	0	0	0	0
378,5914	465	0	0	0	0	0	0
380,635925	465	0	0	0	0	0	0
382,678955	465	0	0	0	0	0	0
384,720398	465	0	0	0	0	0	0
386,760376	465	0	0	0	0	0	0
388,798767	465	0	0	0	0	0	0
390,835663	465	0	0	0	0	0	0
392,871033	465	0	0	0	0	0	0
394,904877	465	0	0	0	0	0	0
396,937195	465	0	0	0	0	0	0
398,967957	465	0	0	0	0	0	0
400,997253	465	0	0	0	0	0	0
403,024963	465	0	0	0	0	0	0
405,051147	465	0	0	0	0	0	0
407,075806	465	0	0	0	0	0	0
409,098938	465	0	0	0	0	0	0
411,120544	465	0	0	0	0	0	0
413,140564	465	0	0	0	0	0	0
415,159119	465	0	0	0	0	0	0
417,176086	465	0	0	0	0	0	0
419,191528	465	0	0	0	0	0	0
421,205444	465	0	0	0	0	0	0
423,217834	465	0	0	0	0	0	0
425,228668	465	0	0	0	0	0	0
427,237976	465	0	0	0	0	0	0
429,245728	465	0	0	0	0	0	0
431,251953	465	0	0	0	0	0	0
433,256592	465	0	0	0	0	0	0
435,259766	465	0	0	0	0	0	0
437,261353	465	0	0	0	0	0	0
439,261383	465	0	0	0	0	0	0
441,259888	465	0	0	0	0	0	0
443,256836	465	0	0	0	0	0	0
445,252258	465	0	0	0	0	0	0
447,246094	465	0	0	0	0	0	0
449,238434	465	0	0	0	0	0	0

This journal is © The Royal Society of Chemistry 20xx

Please do not adjust margins

Please do not adjust margins

451,229187	465	0	0	0	0	0	0
453,218445	465	0	0	0	0	0	0
455,206116	465	0	0	0	0	0	0
457,192261	465	0	0	0	0	0	0
459,176819	465	0	0	0	0	0	0
461,15979	465	0	0	0	0	0	0
463,141296	465	0	0	0	0	0	0
465,121216	465	0	0	0	0	0	0
467,099579	465	0	0	0	0	0	0
469,076385	465	0	0	0	0	0	0
471,051636	465	0	0	0	0	0	0
473,02536	465	0	0	0	0	0	0
474,997498	465	0	0	0	0	0	0
476,968079	465	105,222248	92,7733742	85,8671209	88,5841344	114,823725	48,0908646
478,937134	465	110,515989	99,176666	89,4128809	72,9096371	121,439231	48,7704693
480,904572	465	120,514121	103,561884	97,4109051	67,9989588	132,466311	48,8508445
482,870453	465	133,856876	112,367356	103,133794	64,4403053	135,775196	49,8573008
484,834808	465	146,898352	117,92815	110,705657	67,6961724	149,230278	51,8254695
486,797607	465	154,004069	124,303451	117,655096	71,8066179	155,966805	53,7490636
488,75885	465	163,871793	135,837411	123,850262	72,3848179	166,378966	57,0835865
490,718506	465	173,243716	137,865777	130,790787	74,6617919	174,447203	60,1198854
492,676605	465	182,229812	144,201263	129,422417	77,0809787	182,995223	62,9774413
494,633118	465	191,359135	149,20276	139,272001	76,5096206	192,612232	65,8439643
496,588104	465	204,956092	158,966884	144,775907	77,6655059	194,8546	68,5791029
498,541504	465	210,114783	165,787267	151,402704	78,5765191	200,570432	71,5399041
500,493347	465	216,563132	167,348758	155,414564	82,1628425	206,730631	71,3037703
502,443665	465	223,88499	171,510935	156,314163	84,9351862	212,966004	73,1073446
504,392365	465	233,563205	176,868743	162,712794	82,774509	225,185482	72,412594
506,339539	465	235,403797	181,169289	162,043172	86,8952184	223,493257	74,2303536
508,285095	465	242,621829	186,652519	167,622758	88,1673076	225,631965	79,1851024
510,229126	465	247,517622	186,42274	173,021143	89,8562671	229,106399	78,3707726
512,17157	465	249,280881	185,842653	173,818823	90,305829	233,94162	75,9767288
514,112427	465	262,82721	190,888156	176,065215	93,2705744	237,173783	76,1139695
516,051697	465	262,206046	194,205884	172,150769	93,6109373	238,195573	78,2091133
517,989441	465	268,196412	190,806079	178,584208	94,0294603	240,516725	77,2065953
519,925598	465	265,479375	189,919449	175,958665	92,7681307	242,630918	76,5253401
521,860168	465	269,581151	190,825219	176,02698	87,6688781	238,704022	77,5282477
523,793152	465	268,734903	190,373868	182,995987	86,6062803	240,495224	75,8399055
525,724609	465	271,963156	186,630269	180,688895	86,2553443	240,903589	75,2372898
527,654419	465	274,645935	187,538461	180,171252	81,4180032	236,902617	72,1272126
529,582764	465	275,078415	192,550331	180,13623	78,5990949	241,71153	72,8583011
531,50946	465	282,091315	185,327195	183,269075	76,232788	244,700999	72,4599159
533,43457	465	281,805712	190,63369	180,043311	71,1842279	238,833779	71,2039654
535,358093	465	284,060619	186,637365	180,219449	71,3587537	237,525668	68,6655128
537,280029	465	279,305255	183,034842	179,299191	70,2730089	235,916007	69,6737357
539,200439	465	277,374842	188,993922	181,226527	69,3018898	235,823977	69,8863736
541,119263	465	275,542997	183,566239	179,666305	68,52211	239,334559	66,8572443

 This journal is © The Royal Society of Chemistry 20xx

Please do not adjust margins

Please do not adjust margins

543,036438	465	274,49825	178,434489	180,680192	67,0818851	230,297607	70,5876855
544,952087	465	274,747186	177,531467	178,478614	65,2171741	228,269033	66,1904425
546,866211	465	275,60368	175,365832	177,706018	62,685658	227,655513	65,6047742
548,778687	465	276,305609	171,044429	174,007514	62,7908884	225,960865	67,9371041
550,689514	465	268,638704	170,212291	172,448009	60,2427434	227,546504	64,2144485
552,598816	465	262,135904	170,552044	170,058857	60,1426633	217,967151	62,8841097
554,506531	465	261,57751	164,08081	167,87681	59,1187754	218,484538	62,5247282
556,41272	465	256,266417	161,309105	163,557966	56,2579188	217,241754	59,8331186
558,317261	465	253,816404	158,085456	165,284605	56,5370269	207,368326	58,3376607
560,220215	465	248,492255	155,437465	163,421375	53,7156523	204,846541	55,7398822
562,121582	465	246,925593	150,286113	160,07962	52,267812	199,866707	56,6866937
564,021362	465	240,706214	145,542811	155,247052	50,3790683	196,697581	54,8040017
565,919556	465	240,277906	144,217025	150,388739	52,4092814	193,551236	50,9844738
567,816162	465	236,078617	140,368298	151,509697	50,9943044	189,636859	50,8556916
569,711182	465	228,027349	134,964354	148,691612	48,5754889	188,524851	47,5376361
571,604614	465	226,776104	135,117603	145,568377	47,5500935	183,490252	46,564729
573,49646	465	221,162912	129,086074	144,91776	44,8545585	181,590259	48,0618455
575,386719	465	215,508446	126,837115	139,24809	45,4400582	176,315482	43,540695
577,27533	465	213,92064	124,201574	138,691075	42,828831	168,868065	43,6067208
579,162354	465	211,380031	122,03857	135,658205	42,7265592	171,205313	43,4495559
581,047852	465	201,637188	118,494701	134,830887	42,556207	158,65698	40,692313
582,931641	465	201,811652	115,028561	127,971513	41,8076978	160,961795	40,749978
584,813965	465	194,19385	112,544431	127,491908	40,4719783	158,026267	41,4896522
586,69458	465	189,353021	109,395322	120,975321	39,3769931	151,605212	37,8517422
588,57373	465	184,415651	106,049613	120,145583	38,2422838	144,587898	34,90633
590,451111	465	182,412397	100,524153	113,93865	36,5314562	140,662236	35,0449479
592,326965	465	172,81705	99,1847599	114,951086	37,0510486	136,800166	34,717326
594,201233	465	169,0563	93,9084218	108,924705	33,0574879	133,541918	34,7761411
596,073914	465	163,969962	94,2283004	108,750775	32,8756579	130,469925	31,5321895
597,944946	465	159,089129	90,8175876	104,670768	31,5803196	129,025568	34,0938585
599,814453	465	155,86663	88,6717867	104,362174	31,4452476	127,636675	29,7463044
601,682251	465	155,076852	83,9495511	102,194995	31,0712793	118,214942	30,3357251
603,548523	465	149,501798	85,1506753	97,3024537	32,8601405	117,422531	29,0742037
605,413147	465	148,406049	79,8507299	95,1037609	29,3984866	116,326719	28,248507
607,276245	465	138,569952	75,8385989	92,331327	30,069723	109,345555	26,9532919
609,137695	465	136,19304	77,8707594	90,039452	27,9971006	110,305811	26,8513565
610,997437	465	135,501317	74,4321067	89,022821	25,4860343	100,851139	24,6376299
612,855713	465	130,561448	71,2244667	87,5012645	26,211814	103,038239	23,8708034
614,712341	465	128,683577	70,9361077	84,4411913	25,8634472	100,022086	24,4737466
616,567322	465	125,503073	63,9170174	81,8320646	26,0110672	98,5105576	22,163822
618,420715	465	117,771817	62,6862772	78,1672705	21,9078531	89,4684669	22,5563741
620,272522	465	112,451146	63,0609467	74,2993915	23,2175669	87,1314086	22,2436275
622,122681	465	111,843739	61,442979	75,6058723	23,267572	85,8717914	19,7008971
623,971313	465	110,506868	59,003596	73,4531168	21,9410532	84,2323145	20,479768
625,818237	465	104,28416	58,201601	72,4675175	20,8747214	83,290051	19,2263124
627,663574	465	99,9999106	53,5468945	68,3962019	19,574057	79,6660062	19,3816233
629,507324	465	99,8159397	51,7268509	67,4489211	20,4035279	77,7360881	17,6453846

This journal is © The Royal Society of Chemistry 20xx

Please do not adjust margins

Please do not adjust margins

631,349365	465	97,3045652	50,3543178	62,2358197	17,7286948	74,9800986	16,8640676
633,18988	465	91,9180029	48,1972864	63,1172111	17,1573323	75,032587	18,0058467
635,028809	465	90,8765246	48,7213852	62,7729152	16,7183838	72,4145596	15,7915892
636,865967	465	90,7744531	44,8607002	58,3294695	15,9476639	67,9892511	15,1424158
638,70166	465	84,0897696	44,2080842	56,0270803	15,6495782	65,0796328	14,9053857
640,535645	465	84,3831946	43,0918875	57,2452282	17,8556503	61,9370649	13,9214037
642,368042	465	82,7531461	41,7262429	55,2233631	16,3413321	60,3523352	13,2627645
644,198853	465	74,8740427	38,7279138	51,151757	14,7143021	59,8050378	12,4914339
646,028015	465	76,8223662	39,2674748	51,8279983	13,5602325	59,0726882	12,6907171
647,855591	465	73,0049737	35,6033351	50,5100737	14,6632541	53,3146158	11,1456774
649,681519	465	69,9038999	36,1668755	45,448325	13,9365645	54,2817812	12,3197183
651,505798	465	69,1891749	34,8802746	46,0707952	13,4962473	52,824787	12,0595058
653,328491	465	66,4504884	35,0503816	46,5250552	13,6626217	50,5535571	10,8495984
655,149536	465	65,6201899	33,2931461	42,870739	13,0861067	48,6074307	10,7012564
656,968994	465	60,8909997	31,4122893	41,7892162	11,9456298	47,6132802	10,2427729
658,786743	465	60,2926892	30,48552	39,270464	12,8873583	46,8304915	10,481328
660,602905	465	59,2881323	27,5083746	36,8914666	12,031391	44,1541765	10,1897283
662,41748	465	56,160502	29,4825862	39,2220433	10,813287	43,5889242	10,2355026
664,230469	465	55,7580114	27,047061	36,237143	9,49622917	40,6782601	8,5357995
666,041748	465	51,488687	26,7120715	37,389812	9,32201023	39,922112	9,2533547
667,85144	465	52,2103419	25,3723578	34,0958299	10,5000843	38,3665308	8,70286674
669,659424	465	49,9914385	24,973454	32,3670653	9,36666412	37,7386352	7,56638764
671,46582	465	48,7494638	23,6459504	31,6930132	10,3395537	37,7772216	7,76668649
673,27063	465	44,7214739	21,8628009	32,2689627	8,70378337	33,6038316	8,24819202
675,073792	465	44,8726267	21,1920206	30,1972502	8,3975611	35,0256238	7,81213391
676,875305	465	42,9785263	21,7711591	29,415726	9,04181287	29,656298	8,13340714
678,675171	465	42,1399562	18,6639591	27,6745748	8,00973555	31,0826717	6,77891642
680,47345	465	39,5735257	19,3620284	28,105727	6,38998833	30,7360883	7,03178942
682,27002	465	38,0805016	17,4797134	27,6196091	8,03135675	28,898737	6,34406751
684,065063	465	36,6369205	19,8196843	23,8734461	7,14827149	29,1898233	5,45584631
685,858398	465	34,6894277	16,3714521	24,5997776	6,50127721	28,5522263	5,97807006
687,650024	465	31,3825324	16,9100328	22,539625	6,96327695	26,5623703	6,24418285
689,440186	465	34,672814	15,6403073	23,7670164	8,08824449	24,9924097	4,23760459
691,228577	465	30,6821394	16,6300934	22,2108812	6,72176618	22,3932469	4,56581915
693,015381	465	29,5082169	15,6219097	23,3803649	5,82280677	25,9187665	4,83892153
694,800476	465	32,398251	16,4710922	20,3626814	6,14839464	23,1640192	4,70696389
696,583984	465	30,2810739	13,3091211	18,4069546	6,50157248	22,2063247	3,74138636
698,365845	465	27,1836808	14,601884	21,6230207	5,53198649	21,2804653	4,80506396
700,146118	465	27,4919515	12,0067732	18,5985871	5,48796115	21,0347245	4,16339843
701,924683	465	27,1967082	12,2527048	19,4673873	3,84970406	19,7211653	4,84280185
347,741211	470	0	0	0	0	0	0
349,808533	470	0	0	0	0	0	0
351,874329	470	0	0	0	0	0	0
353,938568	470	0	0	0	0	0	0
356,001343	470	0	0	0	0	0	0
358,062561	470	0	0	0	0	0	0
360,122253	470	0	0	0	0	0	0

This journal is © The Royal Society of Chemistry 20xx

Please do not adjust margins

Please do not adjust margins

362,180481	470	0	0	0	0	0	0
364,237152	470	0	0	0	0	0	0
366,292328	470	0	0	0	0	0	0
368,345978	470	0	0	0	0	0	0
370,398132	470	0	0	0	0	0	0
372,44873	470	0	0	0	0	0	0
374,497803	470	0	0	0	0	0	0
376,545349	470	0	0	0	0	0	0
378,5914	470	0	0	0	0	0	0
380,635925	470	0	0	0	0	0	0
382,678955	470	0	0	0	0	0	0
384,720398	470	0	0	0	0	0	0
386,760376	470	0	0	0	0	0	0
388,798767	470	0	0	0	0	0	0
390,835663	470	0	0	0	0	0	0
392,871033	470	0	0	0	0	0	0
394,904877	470	0	0	0	0	0	0
396,937195	470	0	0	0	0	0	0
398,967957	470	0	0	0	0	0	0
400,997253	470	0	0	0	0	0	0
403,024963	470	0	0	0	0	0	0
405,051147	470	0	0	0	0	0	0
407,075806	470	0	0	0	0	0	0
409,098938	470	0	0	0	0	0	0
411,120544	470	0	0	0	0	0	0
413,140564	470	0	0	0	0	0	0
415,159119	470	0	0	0	0	0	0
417,176086	470	0	0	0	0	0	0
419,191528	470	0	0	0	0	0	0
421,205444	470	0	0	0	0	0	0
423,217834	470	0	0	0	0	0	0
425,228668	470	0	0	0	0	0	0
427,237976	470	0	0	0	0	0	0
429,245728	470	0	0	0	0	0	0
431,251953	470	0	0	0	0	0	0
433,256592	470	0	0	0	0	0	0
435,259766	470	0	0	0	0	0	0
437,261353	470	0	0	0	0	0	0
439,261383	470	0	0	0	0	0	0
441,259888	470	0	0	0	0	0	0
443,256836	470	0	0	0	0	0	0
445,252258	470	0	0	0	0	0	0
447,246094	470	0	0	0	0	0	0
449,238434	470	0	0	0	0	0	0
451,229187	470	0	0	0	0	0	0
453,218445	470	0	0	0	0	0	0
455,206116	470	0	0	0	0	0	0

This journal is © The Royal Society of Chemistry 20xx

Please do not adjust margins

Please do not adjust margins

457,192261	470	0	0	0	0	0	0
459,176819	470	0	0	0	0	0	0
461,15979	470	0	0	0	0	0	0
463,141296	470	0	0	0	0	0	0
465,121216	470	0	0	0	0	0	0
467,099579	470	0	0	0	0	0	0
469,076385	470	0	0	0	0	0	0
471,051636	470	0	0	0	0	0	0
473,02536	470	0	0	0	0	0	0
474,997498	470	0	0	0	0	0	0
476,968079	470	0	0	0	0	0	0
478,937134	470	0	0	0	0	0	0
480,904572	470	101,934383	77,5300252	88,0432868	94,3737385	118,51371	47,6133797
482,870453	470	112,30908	86,1213159	92,040509	74,8359749	117,985335	45,0614675
484,834808	470	119,70085	89,7028118	96,5317021	64,4236163	130,40253	43,643941
486,797607	470	128,55255	95,2820117	100,991334	58,6061007	137,157341	46,5882351
488,75885	470	139,466604	102,648033	107,011603	59,2899656	147,131519	44,4108644
490,718506	470	147,135613	109,332958	109,520802	62,4802488	157,149249	48,6552195
492,676605	470	158,305994	110,969042	115,360442	65,4204628	158,387771	49,5043254
494,633118	470	163,125856	119,21813	123,870688	70,2945734	166,44636	52,8690124
496,588104	470	169,087402	125,002587	126,195074	71,5852443	176,914175	56,3934327
498,541504	470	179,103791	132,653443	128,90521	70,7423374	179,726257	57,6437854
500,493347	470	185,36669	135,164617	136,951627	70,4925484	181,982576	58,3191086
502,443665	470	195,948633	138,581061	139,360493	74,2908335	190,103761	64,4649655
504,392365	470	201,170916	150,286179	143,266099	74,5204975	195,900962	60,948584
506,339539	470	208,324978	146,977394	144,509799	77,1770531	201,4622	61,354069
508,285095	470	214,312444	150,924339	146,669986	77,6017093	201,057521	65,315969
510,229126	470	219,613152	150,514956	148,999921	77,513231	203,996623	64,5785108
512,17157	470	221,887437	153,156011	152,290283	78,9634279	213,492136	66,4928748
514,112427	470	227,932818	157,408249	155,473447	82,0938903	215,14197	64,1152251
516,051697	470	232,144701	159,109912	157,934176	80,151257	214,621034	67,1131923
517,989441	470	232,050071	160,632517	158,032546	81,835858	217,155217	65,0443686
519,925598	470	234,285056	160,240736	161,652286	83,9144374	217,717034	64,8298334
521,860168	470	237,560036	166,114713	165,321573	87,4311947	218,577321	67,0972442
523,793152	470	240,885978	161,221502	163,033244	86,2628731	222,370543	65,2479609
525,724609	470	244,78494	163,78594	161,651392	82,5512811	220,720164	64,1848889
527,654419	470	246,554716	161,95293	160,749828	80,1294195	220,411234	63,4886964
529,582764	470	249,026341	165,610784	165,65333	81,3853355	220,575829	62,2851914
531,50946	470	250,11913	164,166338	166,45054	79,9563709	223,326445	64,4655891
533,43457	470	248,69148	161,605901	162,956143	72,0373661	218,540608	62,7766049
535,358093	470	257,289526	162,509869	166,719086	69,782449	220,989944	60,9634214
537,280029	470	256,580048	161,054481	165,298292	65,550279	221,263183	62,8923645
539,200439	470	250,088681	160,219949	164,785519	61,9550159	216,242671	61,5747668
541,119263	470	251,84341	162,175789	162,892037	60,7265405	216,632221	60,9163267
543,036438	470	251,0432	158,549686	161,856401	59,2957829	213,745244	60,9356572
544,952087	470	251,064694	153,41233	163,510377	61,8686734	212,901326	59,3221015
546,866211	470	247,180341	153,932164	162,238388	61,3762504	208,984221	57,4008666

 This journal is © The Royal Society of Chemistry 20xx

Please do not adjust margins

Please do not adjust margins

548,778687	470	249,105235	155,251332	159,400638	58,4453471	204,801751	58,6294947
550,689514	470	245,274605	152,286825	157,557364	56,9684729	206,833302	54,6331826
552,598816	470	245,509671	151,740257	157,81467	54,4197432	204,150256	58,4868277
554,506531	470	240,656241	145,340864	158,155944	54,6008541	202,886936	54,9296124
556,41272	470	237,842873	143,202659	157,811884	53,0902488	200,616296	56,2836155
558,317261	470	233,763264	144,382474	154,342524	53,9605301	200,827001	55,4146547
560,220215	470	233,441529	135,9704	150,533906	50,5957686	195,945151	52,3497945
562,121582	470	226,28731	139,422168	145,617023	49,1621884	193,574025	51,7375176
564,021362	470	223,265124	132,607265	144,80918	47,1162341	186,094365	48,3023204
565,919556	470	222,218841	132,673165	145,551559	45,6925863	181,927241	47,3968941
567,816162	470	217,559281	127,589849	139,320436	48,6700955	179,467473	47,123525
569,711182	470	215,539449	125,164421	139,112126	44,5300369	175,08397	44,7870275
571,604614	470	209,585201	122,342018	136,614029	46,4487189	172,117315	45,5103791
573,49646	470	205,658918	123,850824	133,93484	44,182875	167,236943	41,4566182
575,386719	470	200,864025	116,143534	133,696182	41,4830603	166,307563	41,5247638
577,27533	470	199,958941	113,669274	126,15873	41,8673081	159,04111	40,642809
579,162354	470	195,324824	114,059518	126,850079	38,0829356	158,296759	39,069387
581,047852	470	191,671808	108,479838	123,817334	39,6180032	155,706269	37,6880179
582,931641	470	187,695715	107,737477	119,575518	36,3828952	146,253823	38,5386261
584,813965	470	183,44794	101,154259	118,92646	36,0511909	145,884999	36,6659922
586,69458	470	178,398434	102,096961	114,323236	36,6923546	139,699559	35,4928035
588,57373	470	173,159719	97,3714279	112,182153	34,148481	139,51098	33,7961855
590,451111	470	169,04294	93,7588789	110,651758	33,9695466	135,731711	32,7997522
592,326965	470	163,079973	89,3180724	106,617039	33,5766831	129,609797	32,611552
594,201233	470	160,014611	86,0031043	103,121321	30,2800523	127,685699	31,5649762
596,073914	470	156,735157	83,3501629	102,927035	31,111395	123,329594	29,2331653
597,944946	470	149,823681	81,6648465	100,170198	30,5837222	121,106129	29,596166
599,814453	470	146,644829	81,6371918	98,3269079	29,6251541	120,208801	28,4159303
601,682251	470	145,968116	79,9530711	93,7871096	27,4561267	112,789913	27,2969977
603,548523	470	138,160173	74,709118	92,4285679	25,9639931	114,369568	27,1832114
605,413147	470	137,265654	75,7933415	91,7766091	27,3507832	107,232484	25,5927197
607,276245	470	135,024393	71,4462579	85,4290103	26,8696035	104,813574	25,1132155
609,137695	470	131,905148	70,1815552	86,0625547	24,90718	103,824695	25,898505
610,997437	470	126,835743	67,6814546	83,2101592	25,561177	101,553388	25,0131754
612,855713	470	123,08868	65,8910788	80,7113669	25,9554201	99,6631914	23,7546379
614,712341	470	120,719726	63,6422778	80,8188606	24,0404536	94,2453306	23,1555592
616,567322	470	120,518173	61,7230657	75,3315986	24,2870421	88,9852401	23,5123458
618,420715	470	114,892825	58,6070772	75,4424348	21,2824289	87,7323737	20,721307
620,272522	470	110,817399	57,6789538	71,8365501	21,4384742	85,3673263	20,7098437
622,122681	470	106,893552	53,1389468	71,4362089	19,319528	84,661766	19,8085346
623,971313	470	103,680042	56,7921435	69,5167363	20,6527678	80,9358851	19,4721928
625,818237	470	103,083719	51,6501348	68,3942754	18,8341338	78,9610503	19,5490771
627,663574	470	97,8917858	49,6690486	65,77168	19,8749863	77,3620061	18,1806153
629,507324	470	94,3804881	48,5631487	62,2705225	18,6225993	74,5882542	17,1987636
631,349365	470	94,6751976	48,317371	59,9521087	17,9606874	69,625669	15,9699585
633,18988	470	90,8905507	45,1563635	60,5435026	17,3818482	69,1666603	15,7244373
635,028809	470	85,5359513	44,0575073	58,589971	17,3714419	67,0921554	14,2452729

Please do not adjust margins

Please do not adjust margins

636,865967	470	85,8982672	43,5062542	56,2547441	15,817406	63,6921302	13,3054686
638,70166	470	81,7640606	41,2688683	53,6296248	14,4130583	64,6326881	14,5024377
640,535645	470	77,9073465	39,9612753	50,7382062	13,8452969	60,8861639	13,8200972
642,368042	470	79,0193929	36,7271948	49,7811106	15,3330523	55,23751	12,9861343
644,198853	470	74,5983326	36,6362934	49,6050521	13,6650843	53,3695178	13,4119634
646,028015	470	69,8608669	34,2372606	47,9030306	13,1132379	55,4776338	12,972327
647,855591	470	70,4541313	33,9951898	47,144444	13,6748935	54,0896301	13,3647197
649,681519	470	65,8128176	33,9490339	43,0051679	12,2078867	53,4082664	11,7782026
651,505798	470	63,6780189	32,4843061	41,2346088	11,9716245	49,6700781	11,1376511
653,328491	470	66,1144997	33,9636731	40,2702232	13,7322384	50,340977	10,7688697
655,149536	470	61,0508333	30,280338	39,3210075	13,3207801	47,4057463	10,4033992
656,968994	470	58,4666605	27,393898	39,7150996	11,8703307	45,9782351	10,0981842
658,786743	470	58,2236299	28,3400062	39,4402374	11,5199988	44,8588483	9,62179874
660,602905	470	54,3970499	27,1822498	38,0177075	10,8474758	42,9797329	9,11854249
662,41748	470	55,9298484	25,8395318	36,0799236	10,7790413	42,644878	9,68793774
664,230469	470	52,0499123	24,8924715	34,3905107	9,15678513	37,9095205	9,4717316
666,041748	470	52,6151992	23,9380281	32,6894491	9,11469046	39,4507424	10,019458
667,85144	470	49,5765609	23,1781479	34,1942451	8,78594969	35,7804023	8,51040087
669,659424	470	46,0151681	23,3435246	31,900827	8,9220067	36,2673835	7,52097943
671,46582	470	45,8848273	22,0659175	30,8689963	10,1732067	34,8830836	7,88435454
673,27063	470	44,1650477	21,0833637	28,6152427	9,32239421	32,5226024	7,79622185
675,073792	470	42,9416257	20,5884268	27,8375983	7,31759616	35,4446298	6,02240256
676,875305	470	41,5657976	18,5022741	25,7508439	6,95932065	31,6856609	7,57128838
678,675171	470	40,1450247	19,1597398	27,6583033	8,20973399	28,2356957	6,14146958
680,47345	470	37,6183761	18,7289171	26,2107562	6,65788669	27,1427203	5,06433452
682,27002	470	35,4229838	15,631684	25,5262543	7,0985182	28,0132652	6,51676887
684,065063	470	34,8227289	17,8386449	24,7733526	6,94256332	28,2262328	6,6584006
685,858398	470	34,6584162	14,7323163	25,502595	6,82585475	26,5598776	5,34105782
687,650024	470	32,4758586	15,3464207	23,5504132	6,17334605	23,3454984	4,80572003
689,440186	470	32,3106969	14,3604394	22,3435212	7,74953783	23,7603777	4,33514542
691,228577	470	32,0862452	14,547849	22,8352549	6,25076285	22,5082348	4,33727802
693,015381	470	30,0589898	14,0422123	20,4229352	6,32681561	24,4354901	4,10667064
694,800476	470	29,4492477	14,2194608	19,6348493	5,24090926	22,1975661	4,04526775
696,583984	470	25,7587199	13,792891	20,1374065	5,56447952	21,0758994	3,27352425
698,365845	470	26,3161903	12,4970149	19,7694922	5,58011512	20,1633908	5,16724839
700,146118	470	26,1777825	11,3896869	18,6307549	5,60240202	21,2195338	3,84513927
701,924683	470	27,5006735	12,3505974	17,7244774	5,86845922	20,0156991	4,53387623
347,741211	475	0	0	0	0	0	0
349,808533	475	0	0	0	0	0	0
351,874329	475	0	0	0	0	0	0
353,938568	475	0	0	0	0	0	0
356,001343	475	0	0	0	0	0	0
358,062561	475	0	0	0	0	0	0
360,122253	475	0	0	0	0	0	0
362,180481	475	0	0	0	0	0	0
364,237152	475	0	0	0	0	0	0
366,292328	475	0	0	0	0	0	0

 This journal is © The Royal Society of Chemistry 20xx

Please do not adjust margins

Please do not adjust margins

368,345978	475	0	0	0	0	0	0
370,398132	475	0	0	0	0	0	0
372,44873	475	0	0	0	0	0	0
374,497803	475	0	0	0	0	0	0
376,545349	475	0	0	0	0	0	0
378,5914	475	0	0	0	0	0	0
380,635925	475	0	0	0	0	0	0
382,678955	475	0	0	0	0	0	0
384,720398	475	0	0	0	0	0	0
386,760376	475	0	0	0	0	0	0
388,798767	475	0	0	0	0	0	0
390,835663	475	0	0	0	0	0	0
392,871033	475	0	0	0	0	0	0
394,904877	475	0	0	0	0	0	0
396,937195	475	0	0	0	0	0	0
398,967957	475	0	0	0	0	0	0
400,997253	475	0	0	0	0	0	0
403,024963	475	0	0	0	0	0	0
405,051147	475	0	0	0	0	0	0
407,075806	475	0	0	0	0	0	0
409,098938	475	0	0	0	0	0	0
411,120544	475	0	0	0	0	0	0
413,140564	475	0	0	0	0	0	0
415,159119	475	0	0	0	0	0	0
417,176086	475	0	0	0	0	0	0
419,191528	475	0	0	0	0	0	0
421,205444	475	0	0	0	0	0	0
423,217834	475	0	0	0	0	0	0
425,228668	475	0	0	0	0	0	0
427,237976	475	0	0	0	0	0	0
429,245728	475	0	0	0	0	0	0
431,251953	475	0	0	0	0	0	0
433,256592	475	0	0	0	0	0	0
435,259766	475	0	0	0	0	0	0
437,261353	475	0	0	0	0	0	0
439,261383	475	0	0	0	0	0	0
441,259888	475	0	0	0	0	0	0
443,256836	475	0	0	0	0	0	0
445,252258	475	0	0	0	0	0	0
447,246094	475	0	0	0	0	0	0
449,238434	475	0	0	0	0	0	0
451,229187	475	0	0	0	0	0	0
453,218445	475	0	0	0	0	0	0
455,206116	475	0	0	0	0	0	0
457,192261	475	0	0	0	0	0	0
459,176819	475	0	0	0	0	0	0
461,15979	475	0	0	0	0	0	0

This journal is © The Royal Society of Chemistry 20xx

Please do not adjust margins

Please do not adjust margins

463,141296	475	0	0	0	0	0	0
465,121216	475	0	0	0	0	0	0
467,099579	475	0	0	0	0	0	0
469,076385	475	0	0	0	0	0	0
471,051636	475	0	0	0	0	0	0
473,02536	475	0	0	0	0	0	0
474,997498	475	0	0	0	0	0	0
476,968079	475	0	0	0	0	0	0
478,937134	475	0	0	0	0	0	0
480,904572	475	0	0	0	0	0	0
482,870453	475	0	0	0	0	0	0
484,834808	475	0	0	0	0	0	0
486,797607	475	104,790998	72,8782488	86,7848192	76,8790532	125,894403	47,1006386
488,75885	475	115,909436	78,5837356	91,2683967	64,1205069	125,58408	43,4064721
490,718506	475	122,900701	79,3540279	99,0772162	56,1130716	131,795585	42,8029398
492,676605	475	134,179326	88,4723318	98,9072926	52,0476337	138,955015	41,1224841
494,633118	475	136,687652	91,8578778	107,360312	56,2789103	146,615261	43,2135395
496,588104	475	148,561461	99,0125198	109,67869	60,5553164	149,183314	45,4393095
498,541504	475	151,621333	103,588781	113,974327	63,2885446	159,489901	47,2856744
500,493347	475	160,514717	109,279461	116,859299	69,3443553	161,970531	48,4752794
502,443665	475	167,906026	112,702873	122,51258	67,3820424	171,316349	51,5851312
504,392365	475	178,092324	117,320499	120,710156	68,4900478	174,951452	51,7748299
506,339539	475	177,817134	122,225269	129,428165	70,0845935	174,774544	54,2787857
508,285095	475	182,885246	122,45069	133,419013	70,0891199	180,529382	55,143323
510,229126	475	187,149043	125,60005	132,244623	69,0645447	182,791002	55,1255983
512,17157	475	197,890807	126,772516	139,253785	68,0433343	191,418742	55,6478291
514,112427	475	204,729869	132,693973	140,39591	72,0353839	188,967259	55,496107
516,051697	475	207,204702	131,446265	142,625287	70,0756795	197,93932	55,3333663
517,989441	475	206,673926	134,225793	143,142741	71,9106166	197,515536	58,2769566
519,925598	475	210,921196	138,193253	147,619083	73,8730548	197,255481	56,7122982
521,860168	475	213,785997	139,403408	144,96728	73,5850916	199,626763	55,6700533
523,793152	475	217,306843	140,149561	144,627859	77,1356687	204,570401	55,783292
525,724609	475	220,741401	142,874686	148,683116	78,7668799	201,600773	55,7419392
527,654419	475	225,032753	142,177897	145,765264	82,0141943	200,741448	56,1420215
529,582764	475	230,352994	142,22836	151,456746	76,8651897	203,585622	56,9150686
531,50946	475	229,715762	140,935576	151,580638	77,210113	206,303386	54,509114
533,43457	475	227,444667	143,208045	150,235748	72,3170102	204,845623	55,5908856
535,358093	475	234,449041	143,0976	152,172628	70,6335304	207,468986	55,7559908
537,280029	475	230,654276	143,39006	152,930159	70,3949964	203,805491	56,1966112
539,200439	475	231,946668	144,268396	149,59051	63,9455161	204,03809	54,5238968
541,119263	475	230,588825	142,200827	151,666993	59,677151	197,755615	54,9802805
543,036438	475	228,455234	138,69535	151,159184	53,8938517	197,538885	53,9336166
544,952087	475	229,749716	140,787133	148,718239	56,6543818	195,439254	52,5237101
546,866211	475	226,059232	135,625669	150,341791	54,180003	194,144426	52,8904192
548,778687	475	226,802174	138,305811	147,903183	52,6267975	192,90675	52,6632147
550,689514	475	224,965198	131,333664	147,336217	53,2599654	195,36248	51,0829121
552,598816	475	229,174629	133,293637	148,217415	50,8654191	191,659089	49,4036839

This journal is © The Royal Society of Chemistry 20xx

Please do not adjust margins

Please do not adjust margins

554,506531	475	223,091564	133,619844	145,960987	53,815126	191,797919	50,1559562
556,41272	475	220,025534	129,295282	139,931884	50,3140393	187,574405	51,6684888
558,317261	475	225,203622	130,120296	143,446842	47,3428628	184,987796	49,0174646
560,220215	475	219,684847	127,074297	143,798063	47,2400193	178,506998	49,9093241
562,121582	475	215,33006	125,467057	139,747012	47,8324743	177,487597	46,1338724
564,021362	475	214,92026	121,930289	136,21887	45,8434113	179,12883	47,7944271
565,919556	475	209,854151	121,51867	137,642968	44,3417344	174,590054	45,236926
567,816162	475	203,377685	118,558947	135,112519	44,5848785	175,220618	44,6450188
569,711182	475	207,260249	114,789969	130,16587	42,3882457	166,517756	42,3274181
571,604614	475	203,588157	113,409605	128,962514	40,6154461	164,793692	42,4051689
573,49646	475	195,062183	110,945552	125,92164	40,1159346	160,3175	41,1742856
575,386719	475	190,719555	107,003965	122,408351	38,8219924	157,78771	38,8583769
577,27533	475	191,759782	108,885184	123,358667	37,7855645	153,134566	38,7285964
579,162354	475	185,239048	104,172363	119,642263	38,4098581	156,325683	35,7017995
581,047852	475	184,642465	100,849537	120,018888	36,5675387	147,521096	35,2745946
582,931641	475	175,659411	96,247646	115,008918	36,6819102	141,396228	35,5614748
584,813965	475	173,67593	92,6219745	112,279713	34,7141329	145,490829	34,9054747
586,69458	475	168,173056	92,1563111	110,096481	35,277621	137,233812	32,3567534
588,57373	475	163,733374	90,1069879	107,616924	35,0561666	132,621612	32,2925312
590,451111	475	160,887788	88,6594654	103,593371	32,7499165	130,664593	31,1797228
592,326965	475	155,60404	83,481041	100,738091	32,0488238	125,487484	30,1616108
594,201233	475	150,007389	82,1892424	96,9851371	30,9574924	120,850387	32,0908696
596,073914	475	147,073833	75,883126	99,539351	29,6863572	120,530025	29,4403163
597,944946	475	144,39826	76,4091625	95,0400025	27,2676279	114,489896	28,8734548
599,814453	475	143,465662	78,9566045	92,3772144	26,2484274	112,604427	25,7507704
601,682251	475	141,185693	73,3851419	90,2026106	26,8412786	109,775242	26,7057845
603,548523	475	136,478507	72,8734463	89,1806926	26,2492333	107,695333	26,0523527
605,413147	475	129,739539	70,6274891	84,2441813	26,0268538	104,364518	25,2344689
607,276245	475	130,186137	67,6945662	84,1750498	25,5728965	100,82519	25,255517
609,137695	475	122,032852	63,6503409	82,6291696	24,1141881	101,673256	23,0798639
610,997437	475	123,914212	65,7197133	75,7114496	23,3328555	97,7212975	24,9684809
612,855713	475	118,394066	61,3843064	75,9161268	21,8010624	95,6201311	21,29041
614,712341	475	115,306564	59,2462236	76,5352852	22,7509072	93,4975187	23,1964062
616,567322	475	112,666555	57,535827	73,8916582	23,5483732	88,2357818	20,5350293
618,420715	475	110,417852	57,2651637	70,7958688	21,1413439	88,5446027	20,0271985
620,272522	475	105,164259	54,7757308	68,6271127	22,3202105	82,0360518	19,710323
622,122681	475	103,320794	52,4553059	68,3577401	19,6171037	80,774614	20,2330849
623,971313	475	97,4360066	51,1079835	67,8874811	19,2509941	78,3519257	17,2783069
625,818237	475	95,8559747	48,0168527	64,2557849	19,4474225	77,8770917	18,1322601
627,663574	475	93,2212717	48,2811867	64,0081247	18,6372032	72,514935	17,1953793
629,507324	475	91,9422706	45,5656607	60,9411271	18,9108337	71,293204	16,3673934
631,349365	475	89,6108155	45,7801929	57,7826126	18,3182082	68,6718024	15,7705548
633,18988	475	85,9122367	41,957372	58,0387108	15,442682	68,2230279	15,3191227
635,028809	475	82,0754762	41,8114104	57,5801351	16,5879063	65,199213	13,7850557
636,865967	475	84,071965	40,607226	53,366278	15,3363336	60,7301426	15,3343682
638,70166	475	78,9398351	39,4790897	51,4539625	15,3447663	58,811293	13,9368859
640,535645	475	78,7447291	37,1713326	49,6852068	14,5504983	60,5693857	13,5676333

This journal is © The Royal Society of Chemistry 20xx

Please do not adjust margins

642,368042	475	73,6559935	35,8220452	49,9880472	14,2632652	57,2835461	13,8899319
644,198853	475	70,4322212	34,1985143	49,0002352	14,6605509	54,4082511	12,0189431
646,028015	475	69,9695176	33,2651019	47,6553566	13,8213408	53,9198113	12,2784315
647,855591	475	66,4931881	32,0347322	44,5278062	12,531543	53,3448867	12,7639525
649,681519	475	66,1200501	30,3729292	44,9500096	12,8444875	51,7218125	12,0302947
651,505798	475	61,7539685	31,0499487	40,7687446	11,7247658	49,2757056	11,7699694
653,328491	475	61,7606932	29,2990783	40,70937	13,1845956	50,9780224	9,94210516
655,149536	475	59,4048239	27,9228462	38,876247	12,6733556	44,5654639	10,2276642
656,968994	475	57,7380838	26,4254339	39,8450153	12,9128789	42,7496723	9,39323421
658,786743	475	54,1101885	27,4248013	38,7245111	11,2197725	45,2783131	9,62705845
660,602905	475	52,4988772	26,8025805	37,7520091	10,6809146	42,7418437	8,32539048
662,41748	475	52,2978569	24,8519843	34,9029638	10,9182288	39,9619915	9,37646126
664,230469	475	52,0258653	23,3289883	34,9088226	8,97946672	38,963797	9,01408615
666,041748	475	50,8052556	22,4896146	33,0155796	8,55239538	35,6074455	8,9669402
667,85144	475	45,7310721	24,0043313	32,283427	9,40173345	37,6147046	7,33356624
669,659424	475	46,0759438	22,0909603	31,7148465	8,52970815	35,5229091	8,11529392
671,46582	475	44,8628621	21,9868224	30,604372	10,3225789	31,8564736	7,16411222
673,27063	475	43,0144411	19,7829827	28,3657867	7,80425439	33,5194768	7,57747921
675,073792	475	41,3226803	18,9925449	29,8893307	8,07565296	31,9786702	5,66188356
676,875305	475	40,681183	18,5428953	27,5849209	8,65084498	29,1477187	7,46197706
678,675171	475	39,4201202	19,0556935	25,3540602	8,7163103	30,2439588	5,98339787
680,47345	475	35,4003655	18,022224	26,2919383	6,78840927	28,4243037	5,78596526
682,27002	475	35,0146587	16,5509937	23,0531486	5,59092629	27,1707059	5,93503796
684,065063	475	33,7283497	17,118933	24,8002567	6,12532647	26,8088193	6,01949064
685,858398	475	34,6361476	16,0359898	22,4971366	6,32152766	25,3341297	5,24508034
687,650024	475	32,6912043	14,8319738	21,7473883	6,26323789	24,6476833	4,9910171
689,440186	475	29,3108984	13,9652719	20,1209136	6,79546479	25,2233882	5,13249008
691,228577	475	29,9656082	12,6843869	20,9050738	5,43904616	21,1487651	4,48501303
693,015381	475	28,0514216	13,0391962	19,9876331	6,10193402	22,4379257	4,62667679
694,800476	475	29,007038	12,8423362	18,8186033	5,19183821	21,2619392	4,10433568
696,583984	475	25,2580808	12,2462211	19,219206	6,12503183	22,3922932	4,77501253
698,365845	475	25,5875131	11,3059784	18,7523124	3,5285223	20,6221345	4,65723996
700,146118	475	26,5979313	10,3725882	16,8660413	5,51073471	20,3101277	3,29284571
701,924683	475	25,3326148	10,2160034	17,1277524	4,34650886	18,5407334	4,69381687
347,741211	480	0	0	0	0	0	0
349,808533	480	0	0	0	0	0	0
351,874329	480	0	0	0	0	0	0
353,938568	480	0	0	0	0	0	0
356,001343	480	0	0	0	0	0	0
358,062561	480	0	0	0	0	0	0
360,122253	480	0	0	0	0	0	0
362,180481	480	0	0	0	0	0	0
364,237152	480	0	0	0	0	0	0
366,292328	480	0	0	0	0	0	0
368,345978	480	0	0	0	0	0	0
370,398132	480	0	0	0	0	0	0
372,44873	480	0	0	0	0	0	0

 This journal is © The Royal Society of Chemistry 20xx

Please do not adjust margins

374,497803	480	0	0	0	0	0	0
376,545349	480	0	0	0	0	0	0
378,5914	480	0	0	0	0	0	0
380,635925	480	0	0	0	0	0	0
382,678955	480	0	0	0	0	0	0
384,720398	480	0	0	0	0	0	0
386,760376	480	0	0	0	0	0	0
388,798767	480	0	0	0	0	0	0
390,835663	480	0	0	0	0	0	0
392,871033	480	0	0	0	0	0	0
394,904877	480	0	0	0	0	0	0
396,937195	480	0	0	0	0	0	0
398,967957	480	0	0	0	0	0	0
400,997253	480	0	0	0	0	0	0
403,024963	480	0	0	0	0	0	0
405,051147	480	0	0	0	0	0	0
407,075806	480	0	0	0	0	0	0
409,098938	480	0	0	0	0	0	0
411,120544	480	0	0	0	0	0	0
413,140564	480	0	0	0	0	0	0
415,159119	480	0	0	0	0	0	0
417,176086	480	0	0	0	0	0	0
419,191528	480	0	0	0	0	0	0
421,205444	480	0	0	0	0	0	0
423,217834	480	0	0	0	0	0	0
425,228668	480	0	0	0	0	0	0
427,237976	480	0	0	0	0	0	0
429,245728	480	0	0	0	0	0	0
431,251953	480	0	0	0	0	0	0
433,256592	480	0	0	0	0	0	0
435,259766	480	0	0	0	0	0	0
437,261353	480	0	0	0	0	0	0
439,261383	480	0	0	0	0	0	0
441,259888	480	0	0	0	0	0	0
443,256836	480	0	0	0	0	0	0
445,252258	480	0	0	0	0	0	0
447,246094	480	0	0	0	0	0	0
449,238434	480	0	0	0	0	0	0
451,229187	480	0	0	0	0	0	0
453,218445	480	0	0	0	0	0	0
455,206116	480	0	0	0	0	0	0
457,192261	480	0	0	0	0	0	0
459,176819	480	0	0	0	0	0	0
461,15979	480	0	0	0	0	0	0
463,141296	480	0	0	0	0	0	0
465,121216	480	0	0	0	0	0	0
467,099579	480	0	0	0	0	0	0

Please do not adjust margins

Please do not adjust margins

469,076385	480	0	0	0	0	0	0
471,051636	480	0	0	0	0	0	0
473,02536	480	0	0	0	0	0	0
474,997498	480	0	0	0	0	0	0
476,968079	480	0	0	0	0	0	0
478,937134	480	0	0	0	0	0	0
480,904572	480	0	0	0	0	0	0
482,870453	480	0	0	0	0	0	0
484,834808	480	0	0	0	0	0	0
486,797607	480	0	0	0	0	0	0
488,75885	480	0	0	0	0	0	0
490,718506	480	91,9613289	61,0532616	80,85051	83,1976601	109,957571	46,140614
492,676605	480	101,297104	64,4183807	81,8174834	67,159768	115,389484	42,4652882
494,633118	480	107,156714	68,5306593	86,3278784	55,8199916	116,992504	39,1161807
496,588104	480	114,640341	75,110222	89,1956832	51,7910228	122,46139	38,4557096
498,541504	480	126,151049	75,6236222	94,8526108	50,1440276	125,422078	39,2758527
500,493347	480	133,023821	81,7544143	96,4750043	52,8599872	132,213304	37,6865245
502,443665	480	134,071297	89,2635476	101,994284	56,7497733	138,144639	41,9847736
504,392365	480	140,192818	89,8651331	104,874478	60,6463559	143,318779	40,9077601
506,339539	480	151,02712	92,9575542	105,43125	62,1999834	145,732801	43,400069
508,285095	480	154,710546	99,6971504	110,148094	61,7740579	151,644126	45,4225812
510,229126	480	158,255903	98,1753942	110,015898	63,4194024	150,824514	47,6635796
512,17157	480	164,806894	101,562411	112,862671	59,1703003	159,108694	45,1180453
514,112427	480	168,902351	105,44126	118,911242	62,3368267	163,224334	45,9401896
516,051697	480	175,091965	103,95178	120,671559	62,2744398	162,216641	46,1377819
517,989441	480	171,733031	105,982566	122,433589	64,9427391	168,511273	48,6944761
519,925598	480	180,262805	109,211721	124,351752	63,2138689	167,335885	45,9574663
521,860168	480	185,902437	112,73014	123,490651	61,875124	170,911456	47,514182
523,793152	480	189,872859	112,782033	125,532989	65,4653881	171,413509	48,6379696
525,724609	480	189,226469	114,758181	127,226622	66,5822921	176,25477	45,5382659
527,654419	480	192,423998	115,374937	127,126903	68,8909246	175,145476	47,4931782
529,582764	480	196,978114	118,126058	128,52318	71,2342544	179,73717	46,1653716
531,50946	480	196,807719	118,645721	130,433126	73,8811934	179,342515	48,6936157
533,43457	480	202,79948	122,458495	130,350029	71,8056462	181,920118	49,2377603
535,358093	480	204,719498	122,264009	135,653955	70,4038642	180,982337	50,0155095
537,280029	480	205,167098	120,950953	130,453759	69,1838489	182,081558	47,7060015
539,200439	480	201,653682	121,284109	133,22371	64,3861984	180,074814	47,3794374
541,119263	480	200,856766	120,558014	132,265386	62,9601128	182,508816	46,8220637
543,036438	480	199,807028	117,016648	132,5542	61,6847129	177,569129	46,9603391
544,952087	480	204,094819	119,140211	134,731481	54,7175305	178,747881	46,5679383
546,866211	480	201,921557	117,180589	129,152846	51,4072528	174,37212	45,8014511
548,778687	480	205,946973	114,111353	131,280178	51,4021685	175,883231	44,8186158
550,689514	480	198,824281	117,347589	129,726774	46,804152	175,174159	44,9909875
552,598816	480	200,264065	116,252903	132,862531	44,005241	165,163862	44,7530322
554,506531	480	197,433189	110,907547	130,310465	47,6368153	168,257806	43,2123939
556,41272	480	197,758036	112,41439	126,649489	45,5869207	164,126026	43,7171563
558,317261	480	189,584863	109,311975	123,669643	45,1013689	163,156897	42,6833555

This journal is © The Royal Society of Chemistry 20xx

Please do not adjust margins

Please do not adjust margins

560,220215	480	195,108276	109,206953	126,124657	43,76905	160,198412	40,0103748
562,121582	480	193,546965	107,048494	121,926007	43,491935	158,083208	40,2136631
564,021362	480	189,725799	104,78767	121,698518	39,1446411	154,781847	38,3656844
565,919556	480	185,154481	102,64774	118,841567	40,6199456	156,625022	39,2853242
567,816162	480	183,853422	104,492263	120,241513	40,063742	157,1522	42,1110087
569,711182	480	183,724619	100,39319	119,654878	38,3451745	154,095088	40,0897027
571,604614	480	181,751923	100,47239	121,992546	39,0758705	155,284493	39,2772292
573,49646	480	179,478896	100,145318	114,136312	36,1425648	147,652125	38,2725123
575,386719	480	177,123177	97,3156571	111,597384	38,2441759	142,839971	37,3228704
577,27533	480	167,709619	93,8296509	112,331572	35,3163271	145,187631	35,7065938
579,162354	480	175,904861	93,5416361	109,958537	35,4840747	139,8291	34,8055525
581,047852	480	166,743066	89,6902788	108,702948	34,9346453	132,567119	33,4315028
582,931641	480	165,729873	87,4782784	102,888879	34,176661	128,207532	33,5579874
584,813965	480	158,026607	82,5705802	102,330087	30,9078625	126,890534	31,553498
586,69458	480	156,460756	79,7667445	98,1240204	31,6990988	124,919147	28,4703892
588,57373	480	150,675903	79,8606736	98,888049	29,0537758	120,96794	29,3844818
590,451111	480	146,800776	79,0335654	96,4278746	28,2204181	119,545264	29,198232
592,326965	480	143,185779	73,3183936	90,5784981	29,5722589	112,973574	27,433408
594,201233	480	141,121655	71,516157	90,1078496	28,5123643	108,752845	27,9976849
596,073914	480	138,232654	72,3673413	86,5924637	24,4845659	106,697649	26,082903
597,944946	480	132,936131	70,6481822	87,3740473	28,2970874	108,122195	26,7207321
599,814453	480	128,982671	69,2266512	83,4715075	26,3171563	103,77528	25,0737147
601,682251	480	127,491116	68,1418986	83,8015749	25,2681434	101,556368	24,2247301
603,548523	480	128,600524	63,9225978	78,2353867	25,1482968	98,1861258	22,5306097
605,413147	480	122,334702	62,4368988	77,4469234	22,8769452	98,6752491	21,5743402
607,276245	480	121,098478	61,5561218	78,1803517	26,9000723	96,4302958	23,6367546
609,137695	480	117,696882	60,0208838	75,1155117	21,4339285	91,245715	21,965254
610,997437	480	112,34864	57,3882523	69,1723782	22,2620953	87,3659247	22,0742543
612,855713	480	113,440549	57,6057634	72,6050315	22,9907112	87,2012022	20,9028254
614,712341	480	108,609232	54,6069841	70,2384257	21,5421596	87,1736933	21,616248
616,567322	480	104,440043	52,2973973	68,1432117	21,3680325	80,8882452	18,2192277
618,420715	480	98,6508829	49,5415169	67,7823671	19,0290686	81,2048357	19,8269177
620,272522	480	98,0665798	48,5245197	62,4474831	19,9273284	77,9455201	18,6808912
622,122681	480	97,1504486	45,4532565	62,9789058	18,5815875	75,6661517	17,3319252
623,971313	480	92,0966946	46,1084109	62,3552609	18,1019861	73,0877009	17,0476972
625,818237	480	90,5910965	44,2257115	60,2039625	18,1068908	67,6500019	15,5833152
627,663574	480	89,0115693	43,5027868	56,3736414	17,3384815	66,7683918	16,144407
629,507324	480	85,888116	42,1078383	55,1763585	16,8877609	66,35992	15,7546375
631,349365	480	84,3373063	42,7427738	53,2042534	15,7601142	64,1281214	15,9004298
633,18988	480	78,8716663	38,2018655	52,4542563	15,0574865	61,2522389	16,2737953
635,028809	480	79,6329025	37,6412621	52,4900613	15,1156524	62,6938801	14,3150544
636,865967	480	78,1404141	37,701294	49,8138199	14,5341691	57,7461286	14,0013146
638,70166	480	71,8368877	34,8255115	49,3023861	14,7441352	58,047241	13,6722581
640,535645	480	68,9210442	33,6068442	47,9465185	14,0683026	53,9295826	11,110878
642,368042	480	69,9503019	32,5010478	47,1952467	14,0178654	53,3204938	10,9220104
644,198853	480	66,9856048	32,6387413	43,7835704	13,0350232	52,5573216	10,4726169
646,028015	480	65,4357661	30,7822496	42,684305	13,6586745	51,8742318	11,5670421

Please do not adjust margins

Please do not adjust margins

647,855591	480	65,8136623	29,8219816	42,0569903	12,5712679	50,0261389	11,4267273
649,681519	480	61,7369021	28,7579606	41,738418	11,8911537	48,1191924	10,196385
651,505798	480	59,1851597	27,3059813	39,0430487	12,2129072	44,3586693	10,7843043
653,328491	480	57,5831361	24,9250373	39,0949298	12,250553	44,4269486	9,17437484
655,149536	480	55,1747167	26,9398234	36,3508228	11,6768534	45,0692024	8,24569319
656,968994	480	52,0827433	27,4652165	34,1550599	10,5526034	40,2884128	8,07277904
658,786743	480	55,1125299	24,1993482	34,1109049	11,15764	40,3430363	8,73546513
660,602905	480	49,8791089	22,4496341	31,1339809	9,72530003	38,6321512	8,06124243
662,41748	480	49,8232696	23,2486396	32,8096098	9,2107601	37,4796005	10,7500697
664,230469	480	47,8882588	22,0353512	29,7967515	9,15901873	37,9032165	8,11343313
666,041748	480	44,1825512	20,8141018	30,762076	8,40525122	36,5711892	8,89902022
667,85144	480	42,2613739	21,152414	29,8629198	9,76284671	32,4565813	7,16689203
669,659424	480	41,6416114	20,6321372	26,7553163	8,23437585	31,677906	6,6182276
671,46582	480	41,8254909	19,5254112	26,2893077	7,18602661	30,7942204	6,97738784
673,27063	480	40,0033934	18,5404427	25,9677768	8,06291143	30,0659614	7,07548245
675,073792	480	39,4408465	18,576146	26,2304623	7,51153022	29,9112203	6,09663261
676,875305	480	38,0833981	18,2890728	24,1699782	7,29755601	27,9921615	7,37127429
678,675171	480	36,099703	17,2993913	24,882729	7,08613179	26,5081827	6,45089002
680,47345	480	32,7347644	15,5859061	25,8554521	6,6558796	25,4255982	6,3042965
682,27002	480	33,34698	15,2133729	23,3150031	6,96315465	25,371568	6,46830333
684,065063	480	34,8161495	14,5506711	22,8219491	6,70114384	24,8854582	5,91927067
685,858398	480	33,8300975	14,7711237	20,9055071	5,27768101	24,3970126	4,56625764
687,650024	480	29,4249096	13,2857887	21,2833779	7,42464962	22,4218478	4,21370043
689,440186	480	28,9906114	12,4573693	20,6883012	5,65291441	22,4736749	4,43819141
691,228577	480	27,7843708	11,8188338	20,0538891	5,79887742	21,6970488	4,01066102
693,015381	480	26,4870514	11,3460309	18,5249259	6,67479687	21,3211528	4,23619689
694,800476	480	26,8402241	12,7790447	18,8074352	6,88839191	20,7416635	3,8044467
696,583984	480	24,7811102	10,9564865	17,0814983	5,33123304	19,7444771	3,0270128
698,365845	480	24,3435845	10,9149883	17,3463245	4,40701362	18,860543	4,91464882
700,146118	480	26,9867304	11,394023	15,3112199	4,9323579	17,122091	3,26533094
701,924683	480	25,3379953	9,98077864	15,2243949	5,16976061	17,9890474	4,73472175
347,741211	485	0	0	0	0	0	0
349,808533	485	0	0	0	0	0	0
351,874329	485	0	0	0	0	0	0
353,938568	485	0	0	0	0	0	0
356,001343	485	0	0	0	0	0	0
358,062561	485	0	0	0	0	0	0
360,122253	485	0	0	0	0	0	0
362,180481	485	0	0	0	0	0	0
364,237152	485	0	0	0	0	0	0
366,292328	485	0	0	0	0	0	0
368,345978	485	0	0	0	0	0	0
370,398132	485	0	0	0	0	0	0
372,44873	485	0	0	0	0	0	0
374,497803	485	0	0	0	0	0	0
376,545349	485	0	0	0	0	0	0
378,5914	485	0	0	0	0	0	0

 This journal is © The Royal Society of Chemistry 20xx

Please do not adjust margins

Please do not adjust margins

380,635925	485	0	0	0	0	0	0
382,678955	485	0	0	0	0	0	0
384,720398	485	0	0	0	0	0	0
386,760376	485	0	0	0	0	0	0
388,798767	485	0	0	0	0	0	0
390,835663	485	0	0	0	0	0	0
392,871033	485	0	0	0	0	0	0
394,904877	485	0	0	0	0	0	0
396,937195	485	0	0	0	0	0	0
398,967957	485	0	0	0	0	0	0
400,997253	485	0	0	0	0	0	0
403,024963	485	0	0	0	0	0	0
405,051147	485	0	0	0	0	0	0
407,075806	485	0	0	0	0	0	0
409,098938	485	0	0	0	0	0	0
411,120544	485	0	0	0	0	0	0
413,140564	485	0	0	0	0	0	0
415,159119	485	0	0	0	0	0	0
417,176086	485	0	0	0	0	0	0
419,191528	485	0	0	0	0	0	0
421,205444	485	0	0	0	0	0	0
423,217834	485	0	0	0	0	0	0
425,228668	485	0	0	0	0	0	0
427,237976	485	0	0	0	0	0	0
429,245728	485	0	0	0	0	0	0
431,251953	485	0	0	0	0	0	0
433,256592	485	0	0	0	0	0	0
435,259766	485	0	0	0	0	0	0
437,261353	485	0	0	0	0	0	0
439,261383	485	0	0	0	0	0	0
441,259888	485	0	0	0	0	0	0
443,256836	485	0	0	0	0	0	0
445,252258	485	0	0	0	0	0	0
447,246094	485	0	0	0	0	0	0
449,238434	485	0	0	0	0	0	0
451,229187	485	0	0	0	0	0	0
453,218445	485	0	0	0	0	0	0
455,206116	485	0	0	0	0	0	0
457,192261	485	0	0	0	0	0	0
459,176819	485	0	0	0	0	0	0
461,15979	485	0	0	0	0	0	0
463,141296	485	0	0	0	0	0	0
465,121216	485	0	0	0	0	0	0
467,099579	485	0	0	0	0	0	0
469,076385	485	0	0	0	0	0	0
471,051636	485	0	0	0	0	0	0
473,02536	485	0	0	0	0	0	0

This journal is © The Royal Society of Chemistry 20xx

Please do not adjust margins

Please do not adjust margins

474,997498	485	0	0	0	0	0	0
476,968079	485	0	0	0	0	0	0
478,937134	485	0	0	0	0	0	0
480,904572	485	0	0	0	0	0	0
482,870453	485	0	0	0	0	0	0
484,834808	485	0	0	0	0	0	0
486,797607	485	0	0	0	0	0	0
488,75885	485	0	0	0	0	0	0
490,718506	485	0	0	0	0	0	0
492,676605	485	0	0	0	0	0	0
494,633118	485	0	0	0	0	0	0
496,588104	485	90,7156738	56,9190511	74,071913	72,058346	104,401321	46,1570102
498,541504	485	97,3694513	55,985851	76,779643	59,2653266	105,77519	39,804747
500,493347	485	103,359892	60,2957026	80,8230019	48,0877593	106,592373	34,8365979
502,443665	485	111,144975	64,7819871	80,7425367	46,0017394	110,271422	37,2302016
504,392365	485	116,458369	68,1816085	82,4727638	47,7431302	116,549429	35,7047643
506,339539	485	118,337568	71,0954939	85,8641903	52,7148822	121,385725	35,2312734
508,285095	485	127,310158	76,0454982	94,0318213	54,8059078	126,131403	38,5943597
510,229126	485	130,46607	79,0264563	95,1912864	54,837189	128,815528	39,4119252
512,17157	485	136,870966	82,2420871	96,9039775	57,8579833	128,729009	40,0322493
514,112427	485	142,591629	84,8100992	98,6896064	58,0289414	135,758756	37,6932525
516,051697	485	145,516794	88,6587986	101,231617	58,4425885	137,694488	37,6404267
517,989441	485	147,890028	89,7437977	101,002994	57,9141126	138,679429	40,0434745
519,925598	485	151,45707	88,0261768	106,305284	57,8631577	143,41209	38,9993032
521,860168	485	158,164524	92,006803	105,542937	55,1903131	149,598306	41,8958354
523,793152	485	161,481688	93,7611326	108,772765	60,2862091	149,649056	39,7057175
525,724609	485	164,833625	94,977537	111,732556	60,6130974	148,743688	39,6581283
527,654419	485	166,044852	97,950907	109,567924	62,2241224	154,99673	40,3609417
529,582764	485	170,713007	99,6875661	110,944821	59,9230866	155,359963	40,2673724
531,50946	485	172,458993	101,892523	115,588166	63,651912	155,034482	43,7696327
533,43457	485	175,988922	100,448334	114,792373	66,8964305	158,176487	41,381695
535,358093	485	182,513631	102,604006	119,99365	69,472318	161,34034	39,8284007
537,280029	485	180,834345	100,701765	118,940016	68,3818859	162,210378	43,1783455
539,200439	485	184,063079	102,774936	120,994205	65,5715363	156,637588	42,8424145
541,119263	485	181,379448	104,454183	117,630539	64,2669137	161,685255	40,5794756
543,036438	485	182,170825	103,812426	116,439601	62,2571819	156,869062	42,686647
544,952087	485	185,138175	101,336664	119,041821	62,0425484	165,191041	40,9030675
546,866211	485	178,400874	102,629515	115,142157	57,974941	159,11245	40,1638701
548,778687	485	180,07981	98,7285828	117,017161	52,4831623	156,288407	39,4274984
550,689514	485	180,819684	99,5415269	114,034987	48,0781233	151,649418	40,5830335
552,598816	485	179,327001	99,7082603	118,951859	46,3757412	150,406691	37,0746622
554,506531	485	178,104617	99,0001129	117,93202	44,1716615	153,198727	35,9045674
556,41272	485	180,711604	97,6481996	114,405066	41,5796098	150,464829	39,1404278
558,317261	485	177,519047	96,8035785	114,044924	40,8524379	147,855144	36,8927872
560,220215	485	175,675504	94,6992221	111,759377	41,8300949	148,425104	35,8042668
562,121582	485	176,032225	93,3747072	113,565295	39,7858231	146,35028	36,1206165
564,021362	485	173,690317	87,8322507	108,283686	38,1258924	144,260284	36,5277035

This journal is © The Royal Society of Chemistry 20xx

Please do not adjust margins

Please do not adjust margins

565,919556	485	171,112546	91,7300776	106,967617	37,7570374	142,926002	34,8638123
567,816162	485	170,462046	91,451862	111,819673	35,7211067	139,969982	35,4590908
569,711182	485	169,86851	92,2487486	108,197347	34,4856601	136,305301	33,6044569
571,604614	485	166,635749	89,8257642	109,733787	35,0119212	133,263089	34,3749722
573,49646	485	164,473761	86,3154375	107,393193	33,1915868	135,581032	34,4760164
575,386719	485	163,962803	85,4496338	106,463436	32,2926428	131,114862	36,0715377
577,27533	485	161,374634	87,0387763	103,172037	34,0953409	131,516708	31,1318397
579,162354	485	154,743323	80,9767029	97,966131	32,690646	129,142016	30,7844728
581,047852	485	152,434249	81,5600217	102,078565	32,7616638	124,371017	32,1092565
582,931641	485	150,367957	80,9947214	96,1037971	31,1236636	121,342518	31,036928
584,813965	485	146,171565	75,47053	97,5493298	30,0036546	118,048954	32,8244734
586,69458	485	142,074533	74,8116968	95,2480667	29,7132811	117,196494	27,9113156
588,57373	485	138,126403	73,7385913	94,34305	28,2115085	112,509998	27,1632696
590,451111	485	135,922618	72,0963516	87,04704	26,9237007	110,49967	26,4431029
592,326965	485	136,187109	67,5747407	86,640297	26,5045362	108,003564	25,1721825
594,201233	485	130,518476	64,4958971	82,2827828	25,74942	103,15669	26,0444547
596,073914	485	126,416328	64,7675121	82,6835733	25,4747181	102,717171	24,1193061
597,944946	485	124,008048	61,5524365	81,8189541	24,3967002	100,643165	22,4469916
599,814453	485	121,621339	61,0127282	78,3414011	22,8916294	97,1760936	24,3696363
601,682251	485	122,182965	59,8872865	76,1915223	22,903346	93,4637191	21,6181869
603,548523	485	116,648345	58,9751409	73,1517496	23,2471413	88,9346186	21,3095572
605,413147	485	114,241066	55,3543464	72,9624208	21,7694891	88,3909346	22,1243523
607,276245	485	111,991017	54,0332574	70,8965901	22,3238517	87,2758815	22,0907055
609,137695	485	108,057459	52,8432976	68,7031546	20,8549575	85,9636923	19,1195125
610,997437	485	105,285544	51,710538	66,3620397	18,8066312	82,9292481	20,6611963
612,855713	485	101,492755	51,5990736	65,3428148	20,1852981	82,632329	20,2762783
614,712341	485	99,8132792	49,1252382	65,5191032	19,7812502	81,6088258	19,4783922
616,567322	485	94,510476	48,1970099	62,9710702	20,0647614	77,5590402	17,3983599
618,420715	485	93,6703973	49,8397411	62,4737271	17,4675793	74,845088	16,897952
620,272522	485	91,3184068	46,335971	60,7014315	19,5447438	73,8178701	18,9764898
622,122681	485	90,4662012	43,7390728	58,4620833	17,4764721	72,4576885	16,9703531
623,971313	485	88,347369	42,3418705	58,3580365	15,9371999	67,7548777	15,3614717
625,818237	485	86,1224275	40,191582	52,2259108	18,390187	64,9637213	15,0232771
627,663574	485	83,2988546	37,8743556	52,7160718	16,7598316	64,4784252	15,8487833
629,507324	485	77,9956536	38,2608714	53,2425994	16,1071971	61,9846098	14,3207764
631,349365	485	79,5158033	35,754254	50,8781483	15,5839956	59,9536857	13,3267986
633,18988	485	74,7701227	37,3086982	48,5876519	14,6818227	57,8107985	14,4990476
635,028809	485	72,4609538	32,3415755	49,0267585	15,8790356	57,8432914	12,142391
636,865967	485	73,376994	35,0033475	48,0330806	12,884545	53,2308	13,5055967
638,70166	485	69,7464734	30,8707761	44,3729197	14,1673637	55,4119909	11,4961537
640,535645	485	65,1959702	32,1372037	43,8286396	13,2920512	51,6366417	12,0989918
642,368042	485	63,7378926	30,1649423	42,7408162	13,8457718	49,6793914	11,2387053
644,198853	485	62,2015186	29,1551757	41,6899157	14,414503	48,234957	10,9257378
646,028015	485	60,1955375	27,7093179	40,3244856	12,1383901	46,60233	11,2759961
647,855591	485	58,4269742	27,6971311	37,9515982	12,335269	46,6493437	9,65124711
649,681519	485	60,0871454	27,5075421	37,3430555	11,2492982	44,9309175	9,49937956
651,505798	485	55,8697652	28,0955669	35,594438	12,3842244	42,9346501	8,92521284

Please do not adjust margins

Please do not adjust margins

653,328491	485	54,888575	24,3020164	37,4757366	10,1017304	43,1353547	9,84116274
655,149536	485	53,9303029	24,6635473	34,3977248	11,0974531	40,6162632	9,87690002
656,968994	485	49,9823112	24,3538692	34,6085614	9,0797421	40,0263206	8,05814923
658,786743	485	49,3976323	22,8163356	32,4976685	10,7820779	39,3184291	7,75078611
660,602905	485	48,0685662	21,9031041	29,6008082	9,5606068	35,9423183	9,29524907
662,41748	485	46,4167611	21,0298222	29,0390663	10,0211008	34,8492068	8,77957201
664,230469	485	47,2014714	19,4023032	32,5071848	8,64630866	34,7782999	6,4231304
666,041748	485	45,6210835	19,2287019	29,2889343	7,68410934	34,4919482	8,67306094
667,85144	485	39,9672563	19,4911429	28,8100302	7,98542288	29,3250867	7,57472178
669,659424	485	38,6531919	18,2707664	26,6264488	8,42281902	29,8768707	6,74679195
671,46582	485	37,6450768	17,4485535	26,513391	8,29090721	29,6963076	7,03509408
673,27063	485	36,9511897	16,1099411	26,194783	7,61802146	27,9758684	6,42702058
675,073792	485	38,2166158	15,506603	23,9875724	5,86586474	27,2511376	5,23643278
676,875305	485	36,5236632	15,9264904	23,6303333	7,20728643	28,0951964	6,72117306
678,675171	485	33,7195085	14,8013031	23,9162867	5,44165932	25,4100212	5,02399079
680,47345	485	34,5361148	14,7900054	23,6160488	6,56737704	24,0468323	5,51510095
682,27002	485	31,1338628	14,4172213	20,7268075	7,15702835	23,6333644	6,45658121
684,065063	485	31,0253279	13,5433433	22,4991016	6,11435902	24,2103661	4,27123628
685,858398	485	30,756113	12,9817709	21,4438711	5,8325258	22,297548	4,98529334
687,650024	485	27,6726121	12,4265939	19,617178	6,55030994	20,7509014	4,70508875
689,440186	485	26,6725131	11,9571047	18,593907	7,42181383	19,7982335	4,07287831
691,228577	485	28,4599449	11,9629868	18,1753975	5,64049735	19,7364639	3,78892527
693,015381	485	25,7569508	11,7790559	18,2213316	5,65475241	22,2237914	4,15685005
694,800476	485	25,1103111	11,3478914	16,8594516	4,72304353	18,6346533	3,4392925
696,583984	485	23,2832886	10,8920837	15,4212378	5,10135673	18,7098648	3,66899723
698,365845	485	23,778443	9,91665209	16,6154216	3,89080724	15,1542764	5,05002883
700,146118	485	24,4486768	9,0183506	14,80418	4,99144349	17,3883456	3,04211915
701,924683	485	23,1711661	8,11150687	15,3685457	4,57492557	16,5824343	4,65343143
347,741211	490	0	0	0	0	0	0
349,808533	490	0	0	0	0	0	0
351,874329	490	0	0	0	0	0	0
353,938568	490	0	0	0	0	0	0
356,001343	490	0	0	0	0	0	0
358,062561	490	0	0	0	0	0	0
360,122253	490	0	0	0	0	0	0
362,180481	490	0	0	0	0	0	0
364,237152	490	0	0	0	0	0	0
366,292328	490	0	0	0	0	0	0
368,345978	490	0	0	0	0	0	0
370,398132	490	0	0	0	0	0	0
372,44873	490	0	0	0	0	0	0
374,497803	490	0	0	0	0	0	0
376,545349	490	0	0	0	0	0	0
378,5914	490	0	0	0	0	0	0
380,635925	490	0	0	0	0	0	0
382,678955	490	0	0	0	0	0	0
384,720398	490	0	0	0	0	0	0

This journal is © The Royal Society of Chemistry 20xx

Please do not adjust margins

Please do not adjust margins

386,760376	490	0	0	0	0	0	0
388,798767	490	0	0	0	0	0	0
390,835663	490	0	0	0	0	0	0
392,871033	490	0	0	0	0	0	0
394,904877	490	0	0	0	0	0	0
396,937195	490	0	0	0	0	0	0
398,967957	490	0	0	0	0	0	0
400,997253	490	0	0	0	0	0	0
403,024963	490	0	0	0	0	0	0
405,051147	490	0	0	0	0	0	0
407,075806	490	0	0	0	0	0	0
409,098938	490	0	0	0	0	0	0
411,120544	490	0	0	0	0	0	0
413,140564	490	0	0	0	0	0	0
415,159119	490	0	0	0	0	0	0
417,176086	490	0	0	0	0	0	0
419,191528	490	0	0	0	0	0	0
421,205444	490	0	0	0	0	0	0
423,217834	490	0	0	0	0	0	0
425,228668	490	0	0	0	0	0	0
427,237976	490	0	0	0	0	0	0
429,245728	490	0	0	0	0	0	0
431,251953	490	0	0	0	0	0	0
433,256592	490	0	0	0	0	0	0
435,259766	490	0	0	0	0	0	0
437,261353	490	0	0	0	0	0	0
439,261383	490	0	0	0	0	0	0
441,259888	490	0	0	0	0	0	0
443,256836	490	0	0	0	0	0	0
445,252258	490	0	0	0	0	0	0
447,246094	490	0	0	0	0	0	0
449,238434	490	0	0	0	0	0	0
451,229187	490	0	0	0	0	0	0
453,218445	490	0	0	0	0	0	0
455,206116	490	0	0	0	0	0	0
457,192261	490	0	0	0	0	0	0
459,176819	490	0	0	0	0	0	0
461,15979	490	0	0	0	0	0	0
463,141296	490	0	0	0	0	0	0
465,121216	490	0	0	0	0	0	0
467,099579	490	0	0	0	0	0	0
469,076385	490	0	0	0	0	0	0
471,051636	490	0	0	0	0	0	0
473,02536	490	0	0	0	0	0	0
474,997498	490	0	0	0	0	0	0
476,968079	490	0	0	0	0	0	0
478,937134	490	0	0	0	0	0	0

This journal is © The Royal Society of Chemistry 20xx

Please do not adjust margins

Please do not adjust margins

480,904572	490	0	0	0	0	0	0
482,870453	490	0	0	0	0	0	0
484,834808	490	0	0	0	0	0	0
486,797607	490	0	0	0	0	0	0
488,75885	490	0	0	0	0	0	0
490,718506	490	0	0	0	0	0	0
492,676605	490	0	0	0	0	0	0
494,633118	490	0	0	0	0	0	0
496,588104	490	0	0	0	0	0	0
498,541504	490	0	0	0	0	0	0
500,493347	490	82,2484541	47,8987326	65,6920455	83,1275616	91,21103	45,6360285
502,443665	490	85,4415148	47,7257401	65,0829976	63,0874448	89,7796718	40,8992649
504,392365	490	88,2388248	52,1241049	66,7396306	50,4322651	92,4929276	36,4546981
506,339539	490	97,9294673	54,8093644	69,0281282	45,029075	97,0548165	34,8670211
508,285095	490	100,845652	59,476078	73,4614173	43,5393718	98,5373307	33,3681619
510,229126	490	107,068814	60,3739128	75,2441996	46,0647685	101,72548	31,3835239
512,17157	490	109,539906	59,7703732	78,4480789	47,7198888	105,214616	32,9353116
514,112427	490	111,384461	64,053343	81,2691661	51,4666739	112,309635	33,6147906
516,051697	490	120,142661	66,3306425	84,0624923	52,313614	114,191542	31,4120385
517,989441	490	123,220877	71,8490803	80,2937792	50,6083993	117,290929	33,3164848
519,925598	490	127,450504	73,1634773	86,6574808	52,4065034	119,583075	32,6772272
521,860168	490	130,556969	72,8321979	87,8839439	51,075967	122,528835	32,5654326
523,793152	490	131,658311	74,2883919	93,4784486	52,1851569	123,797261	35,9148162
525,724609	490	137,467759	78,4341995	91,991204	52,8851153	124,494035	33,5450416
527,654419	490	143,620874	81,0743444	91,9282397	54,3636981	125,019929	35,3723212
529,582764	490	146,790328	80,9395387	96,2169289	53,623372	132,071253	35,2685935
531,50946	490	147,584445	82,7570017	95,3966569	58,8993574	131,396804	36,8496358
533,43457	490	150,334614	82,810431	98,4786253	54,977457	137,437692	34,5785095
535,358093	490	152,933789	85,3569395	101,943359	56,8267718	139,094262	34,3406935
537,280029	490	156,389645	83,3128338	102,036406	58,6666099	138,449665	35,8847264
539,200439	490	157,456628	90,2681327	103,368322	59,2534561	136,225388	35,9001558
541,119263	490	158,650489	84,660628	102,636657	60,6389217	138,082278	35,4343844
543,036438	490	155,197217	87,0219112	104,350209	62,4727246	143,500904	36,7416299
544,952087	490	166,394838	83,9894302	102,471736	60,4538886	138,029776	36,1272266
546,866211	490	159,514575	89,3185684	105,007326	56,191719	137,389138	35,0605604
548,778687	490	157,76302	84,4266124	104,292263	53,5534386	138,979655	35,1855071
550,689514	490	154,942832	87,8836606	102,718059	52,2495425	139,205398	34,8727124
552,598816	490	157,295562	85,097402	108,385636	48,7895161	137,568014	32,6087457
554,506531	490	156,588597	85,0611308	101,643946	46,2120379	136,040398	35,1513054
556,41272	490	156,332512	86,1196098	102,004457	40,7957559	135,201477	34,1426722
558,317261	490	156,209179	83,3708991	102,198912	40,9888195	132,841367	32,2894543
560,220215	490	157,001537	80,8239617	100,87472	36,7101355	128,324408	32,8060095
562,121582	490	153,919858	83,2965291	96,47631	37,5536956	130,249925	31,7191989
564,021362	490	155,380087	81,2730476	98,5705661	35,6076354	126,808366	33,8724039
565,919556	490	150,312827	77,7261127	98,5263416	34,1014531	121,873648	31,0044645
567,816162	490	151,083341	79,1863807	95,2070533	36,4657787	128,115173	29,7389866
569,711182	490	148,925264	78,5421964	98,5669381	34,4468493	125,172442	28,9302234

This journal is © The Royal Society of Chemistry 20xx

Please do not adjust margins

Please do not adjust margins

571,604614	490	147,768385	77,8522185	97,840093	30,6268151	121,958822	30,2331852
573,49646	490	146,566954	75,0729146	92,0639865	30,5207767	119,835705	28,6229741
575,386719	490	145,156057	75,0290436	94,4244079	29,5608719	115,206771	29,3616635
577,27533	490	147,729231	71,4817164	93,1642368	30,3811303	118,205837	30,0489838
579,162354	490	144,395724	71,9584549	89,945822	30,1408218	114,990109	27,9571283
581,047852	490	140,004163	72,2534612	92,5658136	29,3352195	112,410853	29,4653761
582,931641	490	141,798615	70,9254618	89,5864664	27,8088004	112,809068	27,4745456
584,813965	490	135,035546	68,8443401	86,4315651	27,614241	109,243107	28,2771666
586,69458	490	130,361349	69,1170627	84,1599925	27,1629319	107,10241	26,3597494
588,57373	490	127,611553	66,6559598	82,0528932	26,968271	99,8144032	24,8864474
590,451111	490	126,525732	65,4282061	79,4161627	25,993001	100,443796	25,5169342
592,326965	490	124,48837	61,2844419	77,1470519	27,3731328	101,477528	24,0862462
594,201233	490	117,611087	59,7390296	75,8817505	25,5376595	98,7629043	23,7410409
596,073914	490	119,301721	59,1300387	74,6996611	22,7339187	95,5908779	21,828211
597,944946	490	116,481777	56,2270169	74,9786251	24,1720274	90,9930016	22,0862965
599,814453	490	110,72461	56,1629144	68,302288	21,9665165	89,3229599	21,3409914
601,682251	490	108,941172	53,4712278	71,658861	21,223468	86,0040281	21,0142311
603,548523	490	108,914751	53,0804624	68,2706001	21,1779562	86,5778459	18,6929096
605,413147	490	105,489502	50,234107	70,9716938	21,0731712	86,3621931	19,0055087
607,276245	490	103,263797	48,5064281	66,0617625	19,7346481	81,8334232	19,1828725
609,137695	490	98,4274328	49,1170787	66,5057824	20,0468735	81,4988923	17,1619152
610,997437	490	97,313813	47,7688895	61,2158973	19,1235177	78,017192	18,6403569
612,855713	490	95,9713521	46,2025944	63,2291239	17,6165605	73,0259371	17,1333564
614,712341	490	95,6085415	45,0844868	59,8201388	18,3540917	75,3603471	18,0075121
616,567322	490	92,5991413	45,9622599	56,5765651	19,4099028	71,78304	17,3361521
618,420715	490	86,15595	41,6691588	55,2105304	16,3947161	69,2619544	16,0474298
620,272522	490	87,7272298	43,4225197	55,4662426	17,4694222	69,4092239	15,7349658
622,122681	490	84,5080304	38,3024582	55,3084682	16,117599	62,9638266	16,1856531
623,971313	490	81,4613548	37,6405441	51,0814436	14,6291381	62,7185057	14,3492305
625,818237	490	79,6824074	37,4392649	50,2365398	14,7568376	63,9445307	14,3359557
627,663574	490	78,2091896	36,1731198	48,3677684	14,8528378	57,3362051	15,2018085
629,507324	490	74,506082	35,7172525	50,5727153	16,3341065	56,5633226	11,263914
631,349365	490	71,5277659	34,6155745	48,8526973	13,3869291	56,0417771	12,8925967
633,18988	490	71,1998602	31,7991514	45,5848981	13,700678	57,4428793	13,3463982
635,028809	490	65,9988642	31,8507361	44,5014027	13,8336351	52,1190089	11,9269361
636,865967	490	69,0766843	31,5478575	43,4439609	13,0073895	50,0123254	12,7235053
638,70166	490	65,5739557	29,7250801	42,8327664	12,3779608	51,0999905	11,2452762
640,535645	490	63,7210806	29,8611425	41,1316579	12,2342509	48,7586686	10,8189386
642,368042	490	64,1523158	30,4639042	41,2518703	12,3906037	47,8976883	9,55762819
644,198853	490	58,3234042	28,2576555	40,5621873	12,3463793	44,384994	9,862048
646,028015	490	57,6797339	26,6555107	38,0578033	12,502755	47,3467696	11,1520485
647,855591	490	54,8818377	24,938585	37,6868652	10,367042	43,7782739	9,37212085
649,681519	490	54,6754778	24,9292909	34,7550575	10,6367286	41,444739	9,85060359
651,505798	490	52,17298	23,3820282	32,2518634	10,5370545	42,1163459	9,24599272
653,328491	490	50,6781238	24,5332788	32,7816724	9,99432177	40,9529178	8,62743545
655,149536	490	49,6339512	22,7450037	31,5328525	10,319266	38,2894174	7,93720142
656,968994	490	48,605811	21,5339245	32,0246325	9,41992406	35,6962369	6,52091448

Please do not adjust margins

Please do not adjust margins

658,786743	490	45,121375	20,9086946	31,1613315	10,1203874	36,9587704	6,55219464
660,602905	490	46,2512942	20,3010417	28,7596974	9,34159012	34,566917	8,10020038
662,41748	490	44,624188	20,3922379	28,450073	8,43053714	34,429184	8,28319327
664,230469	490	42,1383456	18,8155176	28,2863689	8,07990403	31,3410007	8,44643226
666,041748	490	41,9942196	17,7453696	26,8696978	8,16823637	32,8688348	6,40157026
667,85144	490	39,8933619	16,7604657	27,606108	8,04311047	28,3139347	6,49295142
669,659424	490	37,3836248	16,7205985	25,6706173	7,02432012	28,7409923	6,13649985
671,46582	490	37,273643	17,3301795	25,47534	7,17953388	27,808901	7,25291414
673,27063	490	35,7086542	16,5058755	23,8701503	7,72780657	27,1753694	5,94394574
675,073792	490	35,7028819	14,3028418	19,9796133	5,28587353	25,6654558	5,06208318
676,875305	490	34,2801924	16,0131667	21,6095362	5,8803499	25,363076	6,99625897
678,675171	490	31,6769503	14,8238447	21,538699	6,6257508	24,4744227	4,9130476
680,47345	490	30,3121834	13,6960073	23,0065523	5,28167675	23,5637387	5,13244738
682,27002	490	27,5025518	14,1916248	21,4478961	5,89716015	22,9740739	4,85166866
684,065063	490	28,193834	13,0397387	20,0247971	5,99165694	23,2749528	3,22023342
685,858398	490	29,0313739	12,9704808	19,51031	4,72049816	21,563102	5,61914828
687,650024	490	25,9063916	11,632495	19,6045176	6,6002618	19,3361347	3,74982016
689,440186	490	27,2457706	11,3584957	17,7748485	6,23963631	20,5827412	3,75848771
691,228577	490	24,0965072	11,2888245	17,5584838	5,11450595	16,9853571	3,76033663
693,015381	490	24,1574056	10,6383132	17,0762776	4,37339762	18,6105578	3,46825282
694,800476	490	24,2268446	11,2390974	16,6207697	4,59845896	18,0032888	3,91965434
696,583984	490	22,6575185	9,54256322	14,7245762	4,76839652	17,2440045	3,10297027
698,365845	490	21,7333062	9,26558708	16,8861897	5,00949974	16,6098613	4,78137623
700,146118	490	23,1171082	9,30259371	13,6085215	5,02950762	16,980789	2,3621375
701,924683	490	21,7508199	8,87909347	14,0424263	4,89584798	15,7465943	4,43647348
347,741211	495	0	0	0	0	0	0
349,808533	495	0	0	0	0	0	0
351,874329	495	0	0	0	0	0	0
353,938568	495	0	0	0	0	0	0
356,001343	495	0	0	0	0	0	0
358,062561	495	0	0	0	0	0	0
360,122253	495	0	0	0	0	0	0
362,180481	495	0	0	0	0	0	0
364,237152	495	0	0	0	0	0	0
366,292328	495	0	0	0	0	0	0
368,345978	495	0	0	0	0	0	0
370,398132	495	0	0	0	0	0	0
372,44873	495	0	0	0	0	0	0
374,497803	495	0	0	0	0	0	0
376,545349	495	0	0	0	0	0	0
378,5914	495	0	0	0	0	0	0
380,635925	495	0	0	0	0	0	0
382,678955	495	0	0	0	0	0	0
384,720398	495	0	0	0	0	0	0
386,760376	495	0	0	0	0	0	0
388,798767	495	0	0	0	0	0	0
390,835663	495	0	0	0	0	0	0

This journal is © The Royal Society of Chemistry 20xx

Please do not adjust margins

Please do not adjust margins

392,871033	495	0	0	0	0	0	0
394,904877	495	0	0	0	0	0	0
396,937195	495	0	0	0	0	0	0
398,967957	495	0	0	0	0	0	0
400,997253	495	0	0	0	0	0	0
403,024963	495	0	0	0	0	0	0
405,051147	495	0	0	0	0	0	0
407,075806	495	0	0	0	0	0	0
409,098938	495	0	0	0	0	0	0
411,120544	495	0	0	0	0	0	0
413,140564	495	0	0	0	0	0	0
415,159119	495	0	0	0	0	0	0
417,176086	495	0	0	0	0	0	0
419,191528	495	0	0	0	0	0	0
421,205444	495	0	0	0	0	0	0
423,217834	495	0	0	0	0	0	0
425,228668	495	0	0	0	0	0	0
427,237976	495	0	0	0	0	0	0
429,245728	495	0	0	0	0	0	0
431,251953	495	0	0	0	0	0	0
433,256592	495	0	0	0	0	0	0
435,259766	495	0	0	0	0	0	0
437,261353	495	0	0	0	0	0	0
439,261383	495	0	0	0	0	0	0
441,259888	495	0	0	0	0	0	0
443,256836	495	0	0	0	0	0	0
445,252258	495	0	0	0	0	0	0
447,246094	495	0	0	0	0	0	0
449,238434	495	0	0	0	0	0	0
451,229187	495	0	0	0	0	0	0
453,218445	495	0	0	0	0	0	0
455,206116	495	0	0	0	0	0	0
457,192261	495	0	0	0	0	0	0
459,176819	495	0	0	0	0	0	0
461,15979	495	0	0	0	0	0	0
463,141296	495	0	0	0	0	0	0
465,121216	495	0	0	0	0	0	0
467,099579	495	0	0	0	0	0	0
469,076385	495	0	0	0	0	0	0
471,051636	495	0	0	0	0	0	0
473,02536	495	0	0	0	0	0	0
474,997498	495	0	0	0	0	0	0
476,968079	495	0	0	0	0	0	0
478,937134	495	0	0	0	0	0	0
480,904572	495	0	0	0	0	0	0
482,870453	495	0	0	0	0	0	0
484,834808	495	0	0	0	0	0	0

This journal is © The Royal Society of Chemistry 20xx

Please do not adjust margins

Please do not adjust margins

486,797607	495	0	0	0	0	0	0
488,75885	495	0	0	0	0	0	0
490,718506	495	0	0	0	0	0	0
492,676605	495	0	0	0	0	0	0
494,633118	495	0	0	0	0	0	0
496,588104	495	0	0	0	0	0	0
498,541504	495	0	0	0	0	0	0
500,493347	495	0	0	0	0	0	0
502,443665	495	0	0	0	0	0	0
504,392365	495	0	0	0	0	0	0
506,339539	495	72,6244693	40,7570526	54,9152057	68,6374328	74,1744644	40,6467802
508,285095	495	75,9143732	43,5595644	58,3898194	54,1978581	76,4601733	35,4529175
510,229126	495	80,9983052	44,1802379	58,0772809	45,6525899	83,0562556	32,5153856
512,17157	495	87,5636491	49,4949113	64,6510688	40,5219944	82,8091817	29,5564781
514,112427	495	88,4445132	52,3262111	66,204195	40,2848306	89,4686624	28,648905
516,051697	495	94,5054002	52,8237075	67,9791827	43,6626387	91,7228115	26,4955028
517,989441	495	98,9045334	54,8021824	66,342036	43,9824655	94,9792271	26,9632692
519,925598	495	105,8501	58,7593451	69,7706799	46,7311824	95,8517167	28,5320745
521,860168	495	107,086791	59,5409452	70,8204531	45,6977433	98,6507004	28,7678781
523,793152	495	108,477634	59,4342326	71,0450292	48,7660788	105,016825	28,8820145
525,724609	495	111,130621	62,5456538	76,7107086	48,3738858	105,760903	27,1880425
527,654419	495	115,226064	67,183952	80,811921	50,1577794	107,022139	26,8578231
529,582764	495	120,331276	67,2341224	79,9033513	45,60263	110,922508	29,5980504
531,50946	495	127,70209	66,2911546	81,7701028	49,7918074	112,215593	29,1466835
533,43457	495	129,149921	68,4442001	85,7155973	47,7584096	113,376082	28,3658066
535,358093	495	129,97278	69,6502355	85,6431973	51,8203512	118,600252	28,9910551
537,280029	495	135,013845	67,6901547	84,8997399	51,3463404	117,137312	28,7410461
539,200439	495	134,995023	70,7384168	88,1244355	51,8493771	119,988727	29,1291506
541,119263	495	132,731921	73,4080083	89,9625428	52,6103926	119,707933	27,9179493
543,036438	495	139,117974	75,7400599	90,1840664	54,6239536	119,552469	30,5066522
544,952087	495	139,586037	76,8090884	91,4494809	57,1394715	121,205422	31,2704124
546,866211	495	138,620675	75,1180102	92,1724104	57,5764213	123,198864	28,4409614
548,778687	495	141,273705	73,3948569	92,1632944	56,1665628	124,592336	30,7387628
550,689514	495	140,413421	74,9267878	90,1065835	51,2973143	122,478968	28,9668142
552,598816	495	142,959343	74,7965872	92,6665971	50,5928221	122,544455	29,4011248
554,506531	495	138,727115	73,120136	89,835598	46,759354	118,421868	29,2613698
556,41272	495	143,020046	74,7319882	90,3800717	48,1767364	121,07891	30,6097216
558,317261	495	141,317165	72,1397389	88,7325453	44,3010505	116,119634	29,2742118
560,220215	495	135,963571	72,7204333	90,5871206	40,3029855	117,537486	28,0057217
562,121582	495	138,584244	70,8386983	91,5979902	37,136202	116,054467	26,9145742
564,021362	495	138,425119	72,1258287	82,9779983	32,7783536	115,484614	27,8391927
565,919556	495	139,21302	69,0888563	87,9168932	33,319189	116,16387	26,8172038
567,816162	495	136,749951	70,7658697	84,3918539	32,6470516	110,333125	28,9560221
569,711182	495	135,090049	68,3702117	86,7987084	31,9178807	113,025526	24,8562558
571,604614	495	134,666717	68,9356725	85,8955055	32,2557531	108,281897	27,8215465
573,49646	495	129,440344	67,262712	84,3964899	30,2068556	108,340069	25,8219601
575,386719	495	129,752215	64,8638961	82,7553722	29,5781097	106,698768	26,6798881

 This journal is © The Royal Society of Chemistry 20xx

Please do not adjust margins

Please do not adjust margins

577,27533	495	130,525077	64,8263724	82,2255271	26,791694	106,624977	26,1039405
579,162354	495	129,474645	61,9444725	81,4854801	27,4625204	100,364514	24,4053001
581,047852	495	128,561673	63,0522573	80,2384901	25,3981256	100,511819	24,3732496
582,931641	495	121,933755	61,4104353	77,3400975	24,0788067	99,9837714	25,2319951
584,813965	495	124,484751	57,3542365	79,9169262	24,7360575	100,572257	24,8334055
586,69458	495	120,831617	60,2997271	76,9100054	25,0754018	97,021472	25,1069543
588,57373	495	118,804432	59,4836203	76,5761058	25,5387531	95,7219658	22,833535
590,451111	495	115,725802	56,6036244	73,4368005	24,1542475	93,7561665	22,8373072
592,326965	495	112,191266	57,9019194	71,3217841	24,0111567	91,2440846	22,7473587
594,201233	495	110,902859	54,9841584	71,7289439	22,2964948	89,8302317	22,7366962
596,073914	495	108,409727	53,1136544	69,5593425	23,9098314	86,1254112	21,1722968
597,944946	495	105,770501	51,5534139	71,7716531	22,4383573	85,4946503	21,8444399
599,814453	495	102,025007	49,2366738	65,7549131	22,385106	82,6716337	20,6891754
601,682251	495	99,8023663	50,899468	65,6686819	21,4390942	80,0005729	18,9892168
603,548523	495	96,041367	50,3807194	63,0628635	20,2274693	79,9663002	18,2619565
605,413147	495	96,7637199	45,7291705	62,8191952	20,1247116	78,6657641	18,2995213
607,276245	495	94,5088109	45,1832955	62,0411387	18,8638545	75,6040701	19,7087906
609,137695	495	91,7805167	44,5687005	58,6726004	16,3775489	76,5781409	16,0565286
610,997437	495	90,8739598	42,8870199	56,5355615	16,888606	70,0219221	17,11524
612,855713	495	88,3894262	44,468929	56,4195463	18,1259549	71,211462	15,9572142
614,712341	495	88,4914366	41,8575755	55,8868725	17,0089227	67,284146	15,7942531
616,567322	495	81,2098865	37,7327494	55,5354693	18,6063076	69,0058899	14,7112918
618,420715	495	83,0394088	38,8835781	50,6047668	15,4020113	65,5969825	14,7329367
620,272522	495	76,9096554	38,0058192	51,8133131	17,1573896	61,5977525	14,5572873
622,122681	495	78,0370242	36,7814186	52,1317786	15,2608421	59,794863	15,003483
623,971313	495	78,1128543	36,8131546	49,7852082	15,0256422	61,7607493	13,3113061
625,818237	495	72,3356746	35,228241	50,1206192	15,2240017	57,8988019	13,9206924
627,663574	495	71,700789	32,3088743	45,4103809	15,7382297	55,5606485	12,550646
629,507324	495	71,4970049	31,0745315	45,4243938	14,0662172	55,8343065	12,8927398
631,349365	495	67,5785869	30,5396326	46,2271597	12,1856999	52,2298819	11,710187
633,18988	495	65,5017911	31,7990437	40,8674246	13,1267073	53,6869949	15,6734712
635,028809	495	64,4855225	28,9163131	43,7152755	12,3471515	48,3174593	11,1685647
636,865967	495	62,1418378	27,1435335	41,5396102	12,0857786	48,7462225	11,6788588
638,70166	495	58,609104	28,1384027	39,8774866	13,3579102	45,0429096	11,1221344
640,535645	495	57,4795166	27,1534702	39,7297744	12,7931218	46,6530689	11,5423736
642,368042	495	54,7483682	25,9249827	37,3881896	12,5270348	44,2434993	10,5697733
644,198853	495	55,1471306	23,6636972	34,9333656	11,1434923	42,5032623	8,89776453
646,028015	495	53,6656531	24,4644034	33,1405184	12,921651	39,4423388	9,54436013
647,855591	495	51,1846266	21,572611	31,4344053	10,2339637	40,1315327	8,26814077
649,681519	495	49,6234716	22,6065413	33,232502	12,7013156	39,4940807	8,02823822
651,505798	495	50,8195781	20,3585162	30,6653233	11,1147668	37,9445084	8,98860784
653,328491	495	47,8479791	21,9155098	29,9747938	10,791687	35,9866914	7,58515377
655,149536	495	47,2368325	21,6380307	28,9376009	8,89422928	37,1944326	8,68996722
656,968994	495	43,9928835	21,3689236	28,3379649	9,29111049	34,3199313	8,58165415
658,786743	495	43,2679994	20,4627034	28,1848241	8,90146137	33,3254156	7,97067346
660,602905	495	43,587081	19,8612174	28,1067707	8,55986096	32,6782477	7,40851232
662,41748	495	40,6658894	18,6445911	26,5594243	8,08824409	31,5849875	6,93108154

This journal is © The Royal Society of Chemistry 20xx

Please do not adjust margins

664,230469	495	41,1819194	16,8641259	24,7161944	7,52011716	29,893188	6,43303903
666,041748	495	38,7103376	17,9872431	28,1942801	6,87554354	29,9477386	5,78565332
667,85144	495	36,9196913	16,9413437	23,5109921	7,55455044	28,0476388	6,09530757
669,659424	495	35,2401491	15,5123961	25,3202606	6,90847847	26,8539986	6,10771523
671,46582	495	34,8370583	15,4550105	22,9217354	8,16622532	24,8929647	5,89297365
673,27063	495	35,0470357	15,589102	23,1590606	7,01649748	26,9867867	4,51099905
675,073792	495	32,3907574	14,2192949	22,0213059	5,99400779	25,7794158	4,96424513
676,875305	495	30,3973789	13,7009713	20,5394713	6,58475998	24,0710395	6,07449148
678,675171	495	31,7272455	14,1451857	20,1732618	7,32578469	23,6304059	4,37136529
680,47345	495	28,1722966	13,9135389	21,0439236	6,06319012	21,9839297	5,10837249
682,27002	495	25,9817562	12,7798062	19,2644922	5,86160821	23,5456401	5,64611111
684,065063	495	27,1049148	12,3775804	18,8081405	6,32775627	21,4627243	3,57781752
685,858398	495	28,8949468	11,7156585	17,1058046	4,31965579	19,9810122	4,32509124
687,650024	495	22,5456147	12,3953072	17,420585	6,5604711	18,3589993	3,88152002
689,440186	495	24,010086	11,6056138	17,5354709	5,75368093	18,7754482	4,41421206
691,228577	495	24,1706397	9,82496563	16,1256383	5,53223169	16,6143424	4,3415296
693,015381	495	23,4115747	10,1482747	14,5946596	4,12218544	19,7325007	3,90224301
694,800476	495	23,6293487	10,9663512	14,2168856	3,29692465	16,4271417	3,75121954
696,583984	495	20,9555881	8,76354877	14,8001605	3,76162659	17,0959437	3,38972389
698,365845	495	20,5641609	8,56283564	15,2184415	4,45119171	15,1048295	4,68340926
700,146118	495	20,8723483	8,29538513	12,8587527	4,84769596	15,3168095	0,60672448
701,924683	495	20,8013084	8,55438372	12,1488822	4,86633261	15,4518113	3,80660621
347,741211	500	0	0	0	0	0	0
349,808533	500	0	0	0	0	0	0
351,874329	500	0	0	0	0	0	0
353,938568	500	0	0	0	0	0	0
356,001343	500	0	0	0	0	0	0
358,062561	500	0	0	0	0	0	0
360,122253	500	0	0	0	0	0	0
362,180481	500	0	0	0	0	0	0
364,237152	500	0	0	0	0	0	0
366,292328	500	0	0	0	0	0	0
368,345978	500	0	0	0	0	0	0
370,398132	500	0	0	0	0	0	0
372,44873	500	0	0	0	0	0	0
374,497803	500	0	0	0	0	0	0
376,545349	500	0	0	0	0	0	0
378,5914	500	0	0	0	0	0	0
380,635925	500	0	0	0	0	0	0
382,678955	500	0	0	0	0	0	0
384,720398	500	0	0	0	0	0	0
386,760376	500	0	0	0	0	0	0
388,798767	500	0	0	0	0	0	0
390,835663	500	0	0	0	0	0	0
392,871033	500	0	0	0	0	0	0
394,904877	500	0	0	0	0	0	0
396,937195	500	0	0	0	0	0	0

 This journal is © The Royal Society of Chemistry 20xx

Please do not adjust margins

398,967957	500	0	0	0	0	0	0
400,997253	500	0	0	0	0	0	0
403,024963	500	0	0	0	0	0	0
405,051147	500	0	0	0	0	0	0
407,075806	500	0	0	0	0	0	0
409,098938	500	0	0	0	0	0	0
411,120544	500	0	0	0	0	0	0
413,140564	500	0	0	0	0	0	0
415,159119	500	0	0	0	0	0	0
417,176086	500	0	0	0	0	0	0
419,191528	500	0	0	0	0	0	0
421,205444	500	0	0	0	0	0	0
423,217834	500	0	0	0	0	0	0
425,228668	500	0	0	0	0	0	0
427,237976	500	0	0	0	0	0	0
429,245728	500	0	0	0	0	0	0
431,251953	500	0	0	0	0	0	0
433,256592	500	0	0	0	0	0	0
435,259766	500	0	0	0	0	0	0
437,261353	500	0	0	0	0	0	0
439,261383	500	0	0	0	0	0	0
441,259888	500	0	0	0	0	0	0
443,256836	500	0	0	0	0	0	0
445,252258	500	0	0	0	0	0	0
447,246094	500	0	0	0	0	0	0
449,238434	500	0	0	0	0	0	0
451,229187	500	0	0	0	0	0	0
453,218445	500	0	0	0	0	0	0
455,206116	500	0	0	0	0	0	0
457,192261	500	0	0	0	0	0	0
459,176819	500	0	0	0	0	0	0
461,15979	500	0	0	0	0	0	0
463,141296	500	0	0	0	0	0	0
465,121216	500	0	0	0	0	0	0
467,099579	500	0	0	0	0	0	0
469,076385	500	0	0	0	0	0	0
471,051636	500	0	0	0	0	0	0
473,02536	500	0	0	0	0	0	0
474,997498	500	0	0	0	0	0	0
476,968079	500	0	0	0	0	0	0
478,937134	500	0	0	0	0	0	0
480,904572	500	0	0	0	0	0	0
482,870453	500	0	0	0	0	0	0
484,834808	500	0	0	0	0	0	0
486,797607	500	0	0	0	0	0	0
488,75885	500	0	0	0	0	0	0
490,718506	500	0	0	0	0	0	0

This journal is © The Royal Society of Chemistry 20xx

Please do not adjust margins

492,676605	500	0	0	0	0	0	0
494,633118	500	0	0	0	0	0	0
496,588104	500	0	0	0	0	0	0
498,541504	500	0	0	0	0	0	0
500,493347	500	0	0	0	0	0	0
502,443665	500	0	0	0	0	0	0
504,392365	500	0	0	0	0	0	0
506,339539	500	0	0	0	0	0	0
508,285095	500	0	0	0	0	0	0
510,229126	500	66,9230015	40,3127543	50,5106242	78,5642523	67,1780864	38,8826468
512,17157	500	68,2924402	37,4089281	48,9570494	54,16712	69,6385856	34,455314
514,112427	500	72,4810602	39,2002536	53,3365701	44,1441367	70,8862219	30,2957762
516,051697	500	73,4573306	41,0185361	53,5445842	38,1522256	72,8093594	25,6959056
517,989441	500	78,1341621	45,0057978	52,0152674	34,2097736	73,529981	24,4375091
519,925598	500	84,6557679	45,0972842	55,431753	36,9174817	78,4904626	23,7643541
521,860168	500	86,2230471	48,6312984	56,9502325	39,5902214	82,0627916	23,1445507
523,793152	500	88,0829565	50,4053561	62,8587743	44,2710955	83,3941126	24,004923
525,724609	500	95,1894959	49,9889298	63,5188275	46,4436376	86,9226876	22,1295314
527,654419	500	97,8245847	51,2785456	65,6024321	46,2641634	88,6427307	23,4321016
529,582764	500	98,7319912	53,4057193	68,6450706	45,1830801	92,4778337	22,6819046
531,50946	500	106,136644	54,9939	70,4773738	46,6845511	93,2497802	23,4334841
533,43457	500	105,953883	56,3053974	71,1238303	44,6171682	93,7378352	23,5557632
535,358093	500	110,568048	59,1983615	71,460475	43,4310975	98,1292477	23,2728722
537,280029	500	113,782059	58,8821841	72,5511642	47,0049048	101,826719	23,5238561
539,200439	500	116,538927	61,7979077	75,0796582	49,0616203	103,031653	26,1574755
541,119263	500	113,932623	62,5241296	76,6542573	47,9653466	103,597226	23,4139546
543,036438	500	118,469366	64,1387722	77,7185266	48,1888766	104,18613	24,6028787
544,952087	500	117,668662	59,7126374	76,890976	46,8692779	105,592736	25,3241563
546,866211	500	121,170697	64,9324171	78,7325678	48,6471719	105,171523	24,2424316
548,778687	500	119,512162	64,5137667	78,0378282	50,4311886	104,26584	25,7550361
550,689514	500	122,813983	64,1360176	78,7358107	51,3807453	106,574043	26,263796
552,598816	500	124,478111	63,3546441	79,7638403	51,7961747	106,847481	25,9216727
554,506531	500	123,195828	63,6011938	82,533379	54,5134677	105,91078	25,6364376
556,41272	500	122,836274	63,0798603	77,436598	49,3973315	108,971534	26,6755362
558,317261	500	124,479661	62,8080859	80,4489232	44,7398456	107,67178	24,8933449
560,220215	500	126,61704	61,0385834	81,4191548	44,267045	106,659452	25,3772799
562,121582	500	122,629031	62,6024514	83,0927664	41,6790369	100,827742	25,0164511
564,021362	500	126,539027	60,9577058	81,3343564	38,495689	103,144635	22,7056188
565,919556	500	124,387747	58,2136936	77,1707698	34,0516414	103,014496	23,7126769
567,816162	500	121,234479	62,2607565	76,5009886	34,776352	99,5762321	23,4364775
569,711182	500	119,352295	62,8852334	78,2139883	27,941837	101,85701	20,1058333
571,604614	500	120,446095	61,9828063	81,1451358	26,6711204	101,266832	23,9000789
573,49646	500	123,034596	57,9693897	73,6228466	29,5505161	96,0972627	23,619889
575,386719	500	119,120815	58,1380853	74,3279399	28,5830801	97,1501784	22,7045276
577,27533	500	115,827464	55,2115907	72,9707624	26,7618499	95,4889706	23,1110674
579,162354	500	117,159976	58,4872567	72,9752306	26,0622546	94,4255085	22,9670125
581,047852	500	112,463838	54,9633923	75,7594869	23,6801923	90,3994222	23,417052

This journal is © The Royal Society of Chemistry 20xx

Please do not adjust margins

582,931641	500	115,655113	54,3244281	70,4345	23,1824723	91,3250143	21,9224051
584,813965	500	111,118568	51,9724716	69,6904452	22,6718027	86,7073979	22,0502107
586,69458	500	111,379665	53,3418819	69,613326	24,3128052	85,9646905	20,1121179
588,57373	500	106,581214	53,1876441	68,3951778	21,1323745	83,8919832	20,4321769
590,451111	500	106,385029	51,349584	68,8670132	21,9031239	85,3017471	20,1082473
592,326965	500	104,408381	50,3021729	65,3032656	21,5568729	85,2835955	20,3288837
594,201233	500	99,9220433	50,8819897	64,9043456	22,7034398	79,9462292	19,0231197
596,073914	500	99,4603011	47,9790628	63,5169964	21,080791	79,4092361	20,0561736
597,944946	500	99,0735539	46,6722752	65,2429938	22,6246222	79,1884712	20,9319362
599,814453	500	96,7654141	47,896077	63,1607177	21,0859993	77,2168956	18,1326386
601,682251	500	94,543263	46,3778927	61,2369912	20,9622709	73,3278796	17,2002678
603,548523	500	92,866453	45,3927278	58,8179012	18,8477611	75,1682152	19,6047268
605,413147	500	91,3845355	43,7683738	59,3075362	20,1439686	71,5693557	15,8787452
607,276245	500	89,2619585	41,4946485	56,2748645	20,9974361	67,6994332	17,6244122
609,137695	500	85,16059	41,5762893	53,5422798	15,9623729	67,0038954	16,4186677
610,997437	500	83,5653227	39,6713745	55,1201432	15,5517534	65,5872782	17,7053077
612,855713	500	82,4193929	38,6550247	52,1218648	16,0735412	64,0612991	16,1619783
614,712341	500	78,5870722	39,4975847	50,0479724	16,4865598	65,8621842	15,9095894
616,567322	500	77,785795	34,9577665	50,1016775	17,984453	61,2657016	13,7784142
618,420715	500	75,1656037	34,3430633	49,4353426	15,9355821	61,4300334	13,2051946
620,272522	500	75,9920226	34,4902585	47,6863965	13,6668913	56,5792932	14,42278
622,122681	500	71,7763678	32,1115747	47,9377457	14,5895652	55,8815553	13,9323128
623,971313	500	70,4098184	33,6156476	45,3860377	14,4802178	56,6322102	14,1198635
625,818237	500	70,1647656	33,0000751	45,4904209	12,6640165	52,5803802	12,8255561
627,663574	500	67,8927667	31,6338116	44,9707737	14,3377453	51,2451552	12,171885
629,507324	500	66,7227074	28,7763864	42,7820179	14,7855467	50,4404231	11,7788054
631,349365	500	62,7821317	29,4006862	43,190429	13,2111107	48,9662934	11,9386082
633,18988	500	61,4951098	28,8648422	40,0425504	10,1395585	48,6261872	10,3002671
635,028809	500	58,894389	25,0717024	39,4145234	12,2509333	46,3261839	10,0670247
636,865967	500	59,5925501	27,3051786	36,225415	11,7402661	45,0353591	10,3863306
638,70166	500	54,2597942	22,7794402	36,1696648	10,9888685	43,163652	10,3920415
640,535645	500	53,343146	24,7425542	35,8604021	11,5930212	43,5349485	8,3424424
642,368042	500	54,0126235	24,6208632	35,146164	12,2897586	41,0815238	9,03588364
644,198853	500	52,8403624	23,6870216	34,8424419	12,0881937	40,7135535	8,67489981
646,028015	500	52,2943678	21,814258	32,9828602	10,0471041	38,9321881	10,5895402
647,855591	500	47,3401071	20,5890728	31,525039	9,28187579	36,0973405	9,21417855
649,681519	500	46,9834783	21,2340141	29,2500166	9,63754178	36,52036	8,26827316
651,505798	500	46,1042568	19,0970697	30,6737882	10,4451509	34,74843	8,68659522
653,328491	500	45,2476955	19,7713874	28,6145552	10,8625442	35,3953388	7,86516702
655,149536	500	42,7906021	18,607812	27,0972626	8,81437535	34,8273272	6,96505468
656,968994	500	39,173936	19,3824894	27,7518928	9,54955379	34,8935577	7,61551671
658,786743	500	44,7293743	18,6185447	26,9503225	9,04928451	33,2649439	6,87122638
660,602905	500	38,7534053	17,4913912	27,1703739	7,7414079	33,0307283	6,88751822
662,41748	500	38,2225688	16,6287178	25,4886186	8,09037307	28,6975705	7,70464675
664,230469	500	39,4751824	15,2468716	23,181475	7,39801639	26,7059528	6,53824003
666,041748	500	36,0878199	15,7471081	24,4833725	7,56920852	27,3860662	7,65501952
667,85144	500	37,3696061	16,1062041	23,0507956	8,61783058	25,4828659	4,51067609

This journal is © The Royal Society of Chemistry 20xx

Please do not adjust margins

669,659424	500	34,1277047	13,6864965	21,1992754	6,41711212	27,0414472	5,47140712
671,46582	500	31,3947959	14,7300071	20,1123938	7,93046099	24,0525986	6,50890027
673,27063	500	33,1850005	13,5951659	19,1577931	4,6973058	25,1828482	4,86095935
675,073792	500	29,74902	13,382375	21,3451271	5,50403806	24,1933444	4,71063746
676,875305	500	32,5268517	11,7870002	19,3503081	7,25757259	22,1929858	7,26418206
678,675171	500	29,4299455	12,9836916	18,6365913	5,82293403	23,1546356	4,31129858
680,47345	500	25,9898555	12,2574375	19,0998626	4,78232006	21,1423961	4,22822986
682,27002	500	25,6811428	11,2616089	19,8847763	4,95885534	22,4966065	3,84261009
684,065063	500	25,4464214	12,1805258	18,4285142	4,17286384	21,9287795	4,17666407
685,858398	500	26,8985707	11,3039933	17,2342058	4,73667092	20,0097421	2,65173715
687,650024	500	23,3993603	10,9942233	17,4118035	6,26826013	19,3056573	3,61959729
689,440186	500	25,2204874	10,6978948	17,2912017	6,44384507	18,2215151	1,85429262
691,228577	500	21,455996	10,0593585	16,4950702	5,23819969	15,9718354	3,38776531
693,015381	500	21,751909	8,4712162	14,7620813	4,92827262	15,9313309	2,83027259
694,800476	500	22,9546134	10,1629203	15,2422758	4,60395696	16,4932981	3,31463405
696,583984	500	19,1656169	8,42229886	12,955594	3,7304701	14,5304714	2,02927578
698,365845	500	19,1383754	8,03990864	13,0731978	4,39155273	15,8737631	5,04674502
700,146118	500	20,0290009	7,33819994	13,5330335	4,73569198	14,8746852	3,02379615
701,924683	500	19,0434888	7,12086413	11,8664684	3,44247789	13,7012214	3,77376657
347,741211	505	0	0	0	0	0	0
349,808533	505	0	0	0	0	0	0
351,874329	505	0	0	0	0	0	0
353,938568	505	0	0	0	0	0	0
356,001343	505	0	0	0	0	0	0
358,062561	505	0	0	0	0	0	0
360,122253	505	0	0	0	0	0	0
362,180481	505	0	0	0	0	0	0
364,237152	505	0	0	0	0	0	0
366,292328	505	0	0	0	0	0	0
368,345978	505	0	0	0	0	0	0
370,398132	505	0	0	0	0	0	0
372,44873	505	0	0	0	0	0	0
374,497803	505	0	0	0	0	0	0
376,545349	505	0	0	0	0	0	0
378,5914	505	0	0	0	0	0	0
380,635925	505	0	0	0	0	0	0
382,678955	505	0	0	0	0	0	0
384,720398	505	0	0	0	0	0	0
386,760376	505	0	0	0	0	0	0
388,798767	505	0	0	0	0	0	0
390,835663	505	0	0	0	0	0	0
392,871033	505	0	0	0	0	0	0
394,904877	505	0	0	0	0	0	0
396,937195	505	0	0	0	0	0	0
398,967957	505	0	0	0	0	0	0
400,997253	505	0	0	0	0	0	0
403,024963	505	0	0	0	0	0	0

This journal is © The Royal Society of Chemistry 20xx

Please do not adjust margins

405,051147	505	0	0	0	0	0	0
407,075806	505	0	0	0	0	0	0
409,098938	505	0	0	0	0	0	0
411,120544	505	0	0	0	0	0	0
413,140564	505	0	0	0	0	0	0
415,159119	505	0	0	0	0	0	0
417,176086	505	0	0	0	0	0	0
419,191528	505	0	0	0	0	0	0
421,205444	505	0	0	0	0	0	0
423,217834	505	0	0	0	0	0	0
425,228668	505	0	0	0	0	0	0
427,237976	505	0	0	0	0	0	0
429,245728	505	0	0	0	0	0	0
431,251953	505	0	0	0	0	0	0
433,256592	505	0	0	0	0	0	0
435,259766	505	0	0	0	0	0	0
437,261353	505	0	0	0	0	0	0
439,261383	505	0	0	0	0	0	0
441,259888	505	0	0	0	0	0	0
443,256836	505	0	0	0	0	0	0
445,252258	505	0	0	0	0	0	0
447,246094	505	0	0	0	0	0	0
449,238434	505	0	0	0	0	0	0
451,229187	505	0	0	0	0	0	0
453,218445	505	0	0	0	0	0	0
455,206116	505	0	0	0	0	0	0
457,192261	505	0	0	0	0	0	0
459,176819	505	0	0	0	0	0	0
461,15979	505	0	0	0	0	0	0
463,141296	505	0	0	0	0	0	0
465,121216	505	0	0	0	0	0	0
467,099579	505	0	0	0	0	0	0
469,076385	505	0	0	0	0	0	0
471,051636	505	0	0	0	0	0	0
473,02536	505	0	0	0	0	0	0
474,997498	505	0	0	0	0	0	0
476,968079	505	0	0	0	0	0	0
478,937134	505	0	0	0	0	0	0
480,904572	505	0	0	0	0	0	0
482,870453	505	0	0	0	0	0	0
484,834808	505	0	0	0	0	0	0
486,797607	505	0	0	0	0	0	0
488,75885	505	0	0	0	0	0	0
490,718506	505	0	0	0	0	0	0
492,676605	505	0	0	0	0	0	0
494,633118	505	0	0	0	0	0	0
496,588104	505	0	0	0	0	0	0

This journal is © The Royal Society of Chemistry 20xx

Please do not adjust margins

Please do not adjust margins

498,541504	505	0	0	0	0	0	0
500,493347	505	0	0	0	0	0	0
502,443665	505	0	0	0	0	0	0
504,392365	505	0	0	0	0	0	0
506,339539	505	0	0	0	0	0	0
508,285095	505	0	0	0	0	0	0
510,229126	505	0	0	0	0	0	0
512,17157	505	0	0	0	0	0	0
514,112427	505	0	0	0	0	0	0
516,051697	505	57,8286887	32,6370469	44,0321598	64,2454803	58,0634804	28,654365
517,989441	505	60,4504804	34,8697327	42,8818374	45,3307887	60,6161133	25,833622
519,925598	505	63,3121133	33,2491227	43,9329646	38,7440778	60,170789	21,393871
521,860168	505	67,1857943	35,9971764	49,6303652	32,2427299	61,2762097	19,4151574
523,793152	505	70,6164551	35,8158694	52,0130641	32,0092014	67,4489463	19,1035236
525,724609	505	76,2374478	37,6307353	50,8765248	35,3260345	67,4764176	18,7666102
527,654419	505	77,1110278	39,8417047	52,7629485	38,1028275	72,6031481	18,0230919
529,582764	505	83,2852017	44,4911445	54,7237292	39,9097924	75,6491392	16,9671901
531,50946	505	87,1120132	44,4508154	55,2059901	41,5700992	73,8951137	19,4355919
533,43457	505	88,6634184	44,4069937	57,4734327	41,4482639	77,7089502	18,6911154
535,358093	505	89,8834832	49,606846	60,216519	40,9632159	82,1753467	18,9185756
537,280029	505	94,5446048	47,3446992	61,5267672	39,7283281	84,8163258	18,5553491
539,200439	505	92,1264529	49,4398206	64,3674531	41,0261556	84,1521763	20,6632851
541,119263	505	100,117286	50,9425234	61,633568	42,3512764	87,2234575	19,0912323
543,036438	505	98,378387	52,9404158	65,1964093	40,2393956	84,839316	20,588157
544,952087	505	100,198857	48,7599309	63,4259204	41,965395	87,4294729	21,9105059
546,866211	505	102,330672	52,8820093	67,4854409	43,8064999	87,3751551	20,5424944
548,778687	505	104,215377	49,5106181	66,0042044	42,0472728	92,0054434	18,5004852
550,689514	505	103,768128	55,3126303	70,034625	44,2926496	92,7702877	20,5777821
552,598816	505	107,41375	53,9826006	68,8049221	45,0870049	89,8709974	20,8048449
554,506531	505	106,878978	54,5180349	71,6412451	47,5133263	92,7711441	22,9666011
556,41272	505	105,80452	55,1688249	69,9224669	52,3559233	93,508982	21,9252576
558,317261	505	105,178729	56,0085186	68,7358544	49,7011779	94,0017544	22,6964771
560,220215	505	105,754063	52,3850901	72,7327271	48,5202763	94,0862003	22,6299023
562,121582	505	107,299542	54,7310973	68,1852352	45,2561165	94,5578357	20,780349
564,021362	505	105,24825	53,4622246	70,3677267	40,7489088	91,7863932	20,4526318
565,919556	505	111,457098	51,1827906	70,2804944	39,8862626	91,2364342	19,0672051
567,816162	505	103,803554	53,8769823	70,701532	35,8347226	89,2429751	20,7529609
569,711182	505	104,079471	52,0023991	65,6883581	34,607044	90,606264	19,0456517
571,604614	505	108,179704	52,9479622	72,6106959	29,3488368	88,0893695	19,8451705
573,49646	505	106,140603	52,8894737	67,9644262	27,4346627	87,7044717	22,051365
575,386719	505	104,873512	53,401304	67,230443	27,2995428	88,6054063	18,5971555
577,27533	505	103,212722	49,1582079	66,5882206	26,4398365	83,6784364	19,3483465
579,162354	505	105,68628	48,4378773	65,051182	26,7350912	84,5779828	20,5643339
581,047852	505	100,896997	47,7666544	67,0596806	26,119164	79,9847068	19,1945113
582,931641	505	99,8092297	49,2889403	65,4631962	25,321146	78,0960847	19,2672692
584,813965	505	97,5209292	46,0003421	61,2660023	22,9131748	81,1377146	18,0776826
586,69458	505	97,0103151	45,5548467	61,5952353	20,6470672	80,7229601	17,3275293

 This journal is © The Royal Society of Chemistry 20xx

Please do not adjust margins

Please do not adjust margins

588,57373	505	92,3807819	46,4503129	60,6673459	19,1406753	75,2668049	17,7847791
590,451111	505	94,027478	44,7252637	61,3623757	19,387249	75,8713341	18,8349719
592,326965	505	92,5910898	42,9738926	55,6001243	21,4702009	73,7824901	17,8421641
594,201233	505	90,218289	42,9661619	57,9646242	19,541992	72,4520271	16,4076039
596,073914	505	87,7591832	41,7601044	55,8774174	18,1641111	72,5223746	16,4507749
597,944946	505	87,6798616	40,7361945	58,4853367	18,4514205	68,9594336	16,3598137
599,814453	505	89,1379066	41,3963063	56,6037191	18,4076311	70,464473	16,7764576
601,682251	505	87,4168941	39,562306	57,453171	19,2711599	67,4986978	15,6766904
603,548523	505	84,5906896	38,2720494	53,6336937	18,0232568	66,6743728	16,0964955
605,413147	505	83,4687778	38,2823499	54,371098	19,1219371	65,8074828	17,3517134
607,276245	505	79,1334136	37,5482613	51,9574675	18,4915066	59,836638	17,1746699
609,137695	505	76,1577105	37,9390675	54,0528667	15,9857531	64,4768262	16,3955637
610,997437	505	78,5639509	37,788818	49,9474709	16,8446001	60,5384923	17,9351909
612,855713	505	74,6817425	35,6231101	48,6864235	17,9039069	59,8073037	15,2278498
614,712341	505	75,4111042	33,6845788	48,3650596	16,3681914	59,9686579	14,5886341
616,567322	505	73,1517729	31,910216	45,4711532	16,0837141	57,1598923	12,8638813
618,420715	505	68,647613	33,1659476	43,9495186	12,7800325	57,471151	12,0492146
620,272522	505	69,0966317	31,040892	42,2994417	14,3986343	54,634008	13,3658545
622,122681	505	66,9676384	30,5007662	44,5936374	13,5942449	48,7442009	13,9273827
623,971313	505	63,496463	30,1277686	42,4303951	14,0122206	50,2501572	10,6839917
625,818237	505	60,689755	28,3372101	41,4507665	13,9146386	48,6878419	12,4883341
627,663574	505	61,7918432	29,9173189	37,8462595	12,5605057	46,2427288	13,0475376
629,507324	505	61,4806167	27,450354	41,1892246	14,2386503	45,0833136	10,7979545
631,349365	505	56,7517019	24,8543393	38,9082035	12,4006069	44,1139512	11,3462085
633,18988	505	57,4245107	25,9150435	37,6089949	11,6575417	44,4488715	11,1406077
635,028809	505	55,8473858	24,4349718	35,9657066	11,4190943	45,3493337	9,43291964
636,865967	505	54,5457683	25,7092196	34,0131956	10,5099568	41,7848309	9,44796368
638,70166	505	50,2468048	21,9382374	33,8772073	10,8250221	41,1110095	9,68561336
640,535645	505	50,4694418	21,8569749	33,252337	11,2868781	39,0134523	8,13442091
642,368042	505	47,8992932	22,6565811	34,5312034	10,9134793	39,5254806	8,60960956
644,198853	505	45,676172	21,1556001	32,4331465	10,7043217	35,960351	7,77313887
646,028015	505	48,0582265	20,3266908	31,1501299	10,7167189	37,8681927	9,41639099
647,855591	505	45,3985422	18,8443262	30,9034888	9,00429452	38,9396725	7,31199553
649,681519	505	44,70545	18,6333975	27,2439249	9,28663703	35,6319195	7,89858406
651,505798	505	40,4236599	21,0584551	28,6099561	11,5188448	34,0521224	7,45987511
653,328491	505	39,6052813	18,6890756	28,0691447	9,58932543	33,44876	6,69519936
655,149536	505	40,6166442	17,4959723	24,2255465	10,0974669	32,3853946	7,82625557
656,968994	505	39,0422652	17,251739	25,8311705	9,18865061	30,4570502	7,1441838
658,786743	505	37,240219	18,5272342	25,95508	8,75517275	31,0016281	6,54036895
660,602905	505	37,8070872	17,0567761	24,9791475	7,65899473	26,2820539	7,27542371
662,41748	505	36,9353628	15,5321463	23,6484052	7,05228374	27,6037708	6,58532659
664,230469	505	33,074613	14,8408092	24,1770022	6,58441308	28,3818557	6,27645155
666,041748	505	33,456668	14,9482585	21,6461187	7,64220394	25,6144481	5,72360596
667,85144	505	35,9680119	13,9417078	22,4318333	8,14720434	24,069461	5,25432525
669,659424	505	28,4450952	14,2123435	19,9710123	6,14304903	24,2803258	5,50802196
671,46582	505	31,9525593	13,0951919	19,991432	7,28210143	22,8468425	5,2704043
673,27063	505	30,3723604	10,9554194	19,5822406	7,22940591	23,6683759	4,96545213

This journal is © The Royal Society of Chemistry 20xx

Please do not adjust margins

Please do not adjust margins

675,073792	505	31,4072392	11,7895915	19,6199501	6,91778474	22,8175503	3,83320757
676,875305	505	28,9607706	11,9499407	17,9069324	6,67236245	21,0207569	5,87103453
678,675171	505	26,9312879	12,1144498	19,3751237	6,26650681	19,9647438	3,50686952
680,47345	505	25,5286093	11,3740162	16,6759186	5,93643926	20,275211	2,93374276
682,27002	505	22,8432504	10,2720876	17,1418449	5,5489964	19,3649662	3,51633584
684,065063	505	24,3106987	9,65186627	15,0694434	5,32181788	18,4455681	3,85621172
685,858398	505	25,7134867	9,4924345	16,3870099	4,75078062	17,87954	4,02206814
687,650024	505	22,5421441	9,17421363	14,5169103	5,411431	17,1584219	2,87575971
689,440186	505	21,2796748	8,62070572	14,7970835	5,42393928	17,4449452	2,63534345
691,228577	505	19,3173112	8,78923122	15,4623344	5,59105015	14,3250646	2,47184987
693,015381	505	19,2013131	8,81144399	14,0996885	3,95659782	16,2595964	4,13016151
694,800476	505	22,9039989	8,56225497	13,6839981	5,19173754	15,4303811	2,22973014
696,583984	505	18,9431123	8,10084318	14,8151919	5,21269197	17,0667803	2,98497277
698,365845	505	20,4895026	6,63151485	12,864845	3,23597181	13,4591615	4,24059366
700,146118	505	19,5719202	6,15865096	12,0829221	2,83237108	17,3499184	2,25398675
701,924683	505	19,3963483	8,27095164	11,5438181	3,09413582	14,2347367	4,52530412
347,741211	510	0	0	0	0	0	0
349,808533	510	0	0	0	0	0	0
351,874329	510	0	0	0	0	0	0
353,938568	510	0	0	0	0	0	0
356,001343	510	0	0	0	0	0	0
358,062561	510	0	0	0	0	0	0
360,122253	510	0	0	0	0	0	0
362,180481	510	0	0	0	0	0	0
364,237152	510	0	0	0	0	0	0
366,292328	510	0	0	0	0	0	0
368,345978	510	0	0	0	0	0	0
370,398132	510	0	0	0	0	0	0
372,44873	510	0	0	0	0	0	0
374,497803	510	0	0	0	0	0	0
376,545349	510	0	0	0	0	0	0
378,5914	510	0	0	0	0	0	0
380,635925	510	0	0	0	0	0	0
382,678955	510	0	0	0	0	0	0
384,720398	510	0	0	0	0	0	0
386,760376	510	0	0	0	0	0	0
388,798767	510	0	0	0	0	0	0
390,835663	510	0	0	0	0	0	0
392,871033	510	0	0	0	0	0	0
394,904877	510	0	0	0	0	0	0
396,937195	510	0	0	0	0	0	0
398,967957	510	0	0	0	0	0	0
400,997253	510	0	0	0	0	0	0
403,024963	510	0	0	0	0	0	0
405,051147	510	0	0	0	0	0	0
407,075806	510	0	0	0	0	0	0
409,098938	510	0	0	0	0	0	0

 This journal is © The Royal Society of Chemistry 20xx

Please do not adjust margins

Please do not adjust margins

411,120544	510	0	0	0	0	0	0
413,140564	510	0	0	0	0	0	0
415,159119	510	0	0	0	0	0	0
417,176086	510	0	0	0	0	0	0
419,191528	510	0	0	0	0	0	0
421,205444	510	0	0	0	0	0	0
423,217834	510	0	0	0	0	0	0
425,228668	510	0	0	0	0	0	0
427,237976	510	0	0	0	0	0	0
429,245728	510	0	0	0	0	0	0
431,251953	510	0	0	0	0	0	0
433,256592	510	0	0	0	0	0	0
435,259766	510	0	0	0	0	0	0
437,261353	510	0	0	0	0	0	0
439,261383	510	0	0	0	0	0	0
441,259888	510	0	0	0	0	0	0
443,256836	510	0	0	0	0	0	0
445,252258	510	0	0	0	0	0	0
447,246094	510	0	0	0	0	0	0
449,238434	510	0	0	0	0	0	0
451,229187	510	0	0	0	0	0	0
453,218445	510	0	0	0	0	0	0
455,206116	510	0	0	0	0	0	0
457,192261	510	0	0	0	0	0	0
459,176819	510	0	0	0	0	0	0
461,15979	510	0	0	0	0	0	0
463,141296	510	0	0	0	0	0	0
465,121216	510	0	0	0	0	0	0
467,099579	510	0	0	0	0	0	0
469,076385	510	0	0	0	0	0	0
471,051636	510	0	0	0	0	0	0
473,02536	510	0	0	0	0	0	0
474,997498	510	0	0	0	0	0	0
476,968079	510	0	0	0	0	0	0
478,937134	510	0	0	0	0	0	0
480,904572	510	0	0	0	0	0	0
482,870453	510	0	0	0	0	0	0
484,834808	510	0	0	0	0	0	0
486,797607	510	0	0	0	0	0	0
488,75885	510	0	0	0	0	0	0
490,718506	510	0	0	0	0	0	0
492,676605	510	0	0	0	0	0	0
494,633118	510	0	0	0	0	0	0
496,588104	510	0	0	0	0	0	0
498,541504	510	0	0	0	0	0	0
500,493347	510	0	0	0	0	0	0
502,443665	510	0	0	0	0	0	0

This journal is © The Royal Society of Chemistry 20xx

Please do not adjust margins

Please do not adjust margins

504,392365	510	0	0	0	0	0	0
506,339539	510	0	0	0	0	0	0
508,285095	510	0	0	0	0	0	0
510,229126	510	0	0	0	0	0	0
512,17157	510	0	0	0	0	0	0
514,112427	510	0	0	0	0	0	0
516,051697	510	0	0	0	0	0	0
517,989441	510	0	0	0	0	0	0
519,925598	510	0	0	0	0	0	0
521,860168	510	51,4038372	30,685549	37,65673	51,1950214	51,4873452	21,9890924
523,793152	510	57,7372668	28,065309	40,8006909	42,8829999	54,2030171	18,8255483
525,724609	510	58,1563312	31,748748	38,7371026	33,3581398	56,5141389	17,0340906
527,654419	510	61,466103	31,6716281	41,2823174	30,3112642	55,4021104	16,8525624
529,582764	510	67,0183784	33,5810396	44,9371056	30,4790378	59,5799727	13,5938903
531,50946	510	67,1722373	34,3850915	46,7837652	34,1130619	61,6079065	15,5194975
533,43457	510	73,5808976	35,3359383	47,3247693	34,2619094	65,9731872	15,627074
535,358093	510	72,9168395	37,9134444	48,8470187	39,0243645	68,3116206	15,7968858
537,280029	510	76,8035159	38,1893901	55,365322	40,4538094	68,7505857	16,7459499
539,200439	510	77,4826315	41,3921186	52,8091693	41,2698252	68,6871542	17,8733377
541,119263	510	80,8549304	39,9288672	53,3220155	40,4565366	71,5222305	15,1250616
543,036438	510	82,1792951	44,9252017	57,7417441	38,5904394	73,0625463	17,5911784
544,952087	510	86,0531441	43,000358	57,8451602	40,721722	73,6741004	16,2880215
546,866211	510	88,4421394	43,8028053	55,7170637	38,9572433	72,759249	16,0689511
548,778687	510	87,5040195	44,3710021	58,5007066	39,2398124	78,1225294	17,573677
550,689514	510	88,2203199	42,0849112	57,4073407	40,1778092	74,6372477	18,3332767
552,598816	510	90,9113578	45,397051	59,7517227	40,8584275	74,9436642	16,6430585
554,506531	510	90,4456425	44,3091527	59,1567552	40,8410123	77,7627854	18,8493334
556,41272	510	91,8352242	47,2491578	59,3945853	43,1394629	78,2224056	18,0713281
558,317261	510	92,1939503	48,1770623	62,8148698	42,7129194	79,7935344	17,9290116
560,220215	510	93,8457869	45,9748211	61,5423868	46,4280173	82,1593149	18,646179
562,121582	510	91,4678278	47,282376	61,1677331	47,2369206	81,8258993	17,5386594
564,021362	510	93,5721744	44,4761077	61,6662366	46,8615344	79,6297863	19,7087051
565,919556	510	92,8702819	44,113531	63,8271528	43,2751675	81,8068575	16,4902766
567,816162	510	95,3058694	44,965271	63,2247909	41,457135	80,0312502	18,272676
569,711182	510	93,9631472	45,6216071	60,6905073	37,8339535	79,0688423	17,754004
571,604614	510	96,7798807	48,2960776	62,1246909	36,7688078	79,2064796	19,7264308
573,49646	510	91,243411	46,1800285	63,1079825	32,3583765	79,9494653	18,470322
575,386719	510	90,7062268	45,2917003	60,0795998	30,8445332	75,6504094	17,7186777
577,27533	510	93,1819934	43,8493914	60,2604897	27,044308	75,9160421	17,6680853
579,162354	510	89,705532	42,1378636	57,0529948	25,871548	72,9719036	17,7943666
581,047852	510	90,7383158	41,4229798	57,730405	23,461263	72,6789752	15,2724791
582,931641	510	91,6252539	41,4230903	57,434884	23,3873151	72,5311464	16,6811503
584,813965	510	90,4536201	41,3072656	57,2122217	23,1507285	74,2019382	18,0620289
586,69458	510	91,615844	41,8771259	58,6469913	24,2619045	69,9584734	15,2085443
588,57373	510	81,5595766	42,0193764	53,8702278	20,5723923	69,1988723	16,2615534
590,451111	510	87,193444	38,0980558	55,8637267	19,4746633	67,3318097	15,3495795
592,326965	510	81,7503289	37,5245811	50,3793395	19,5357249	66,1627952	14,2184646

This journal is © The Royal Society of Chemistry 20xx

Please do not adjust margins

Please do not adjust margins

594,201233	510	81,9147004	36,3084412	50,8121212	16,8557676	63,4215445	14,803208
596,073914	510	80,8076213	35,4102495	51,7236911	17,055886	63,8052801	14,8332106
597,944946	510	76,9453161	38,0877373	49,6655625	16,3433598	62,9730333	15,8284812
599,814453	510	78,8457156	36,3755085	51,4771223	15,6091176	60,1190017	14,7846328
601,682251	510	76,4280004	37,9849126	49,9699792	15,341086	59,9003318	12,5103147
603,548523	510	74,7280732	34,9818927	46,7896138	15,6928163	59,3872486	16,4134743
605,413147	510	73,9879607	34,1550337	50,0083543	16,6645372	59,5937103	13,2315939
607,276245	510	74,2582139	32,9535482	45,9499707	14,1854877	55,5924394	16,2839064
609,137695	510	71,4661989	33,4373209	46,9481699	14,5459621	56,6533706	14,5711103
610,997437	510	72,9096829	33,8102057	46,428228	13,9610932	56,9956925	14,5999656
612,855713	510	70,1015344	32,6720957	47,3648458	16,4217706	54,9445236	15,9889423
614,712341	510	67,678768	32,5295358	47,5694804	15,1580223	55,0927585	14,2593009
616,567322	510	68,6742261	31,1787214	43,3892999	15,4823953	51,7626555	13,4258421
618,420715	510	66,0797081	31,7642794	42,374515	13,4995302	53,6902008	11,7455778
620,272522	510	63,9819991	26,2689767	42,3874063	15,1516219	47,1561206	13,6290603
622,122681	510	63,8104171	26,6352661	42,0144505	13,402429	49,4268887	12,5719493
623,971313	510	58,7111515	28,02805	40,8104785	14,2936229	47,5424	11,4391044
625,818237	510	58,0864647	27,1254108	39,1800807	13,4141866	48,0420787	11,3280222
627,663574	510	57,5645631	25,221002	36,83003	15,3128032	45,1576829	12,1305508
629,507324	510	55,4965428	24,795948	34,6590109	14,2105545	43,7313442	10,7746509
631,349365	510	52,2428508	22,7725466	34,9199708	12,3353668	43,9207155	10,1529517
633,18988	510	53,3869829	22,5108866	34,6082392	9,76533322	39,8493352	10,8077712
635,028809	510	50,4476042	22,6547341	33,1725756	11,5697142	40,848662	10,1705678
636,865967	510	49,268794	20,3272331	31,6508133	11,0363487	39,7313292	9,002278
638,70166	510	48,1934205	20,5749032	29,9351301	9,54380327	37,3869151	8,29613096
640,535645	510	46,7673757	22,2262721	32,2137354	10,882669	37,3011874	8,05761844
642,368042	510	45,9566377	19,4911565	30,4388486	9,39564139	35,767028	7,2764155
644,198853	510	44,2388336	17,3189165	30,2674363	9,81153832	34,6572988	7,92621066
646,028015	510	44,0528005	19,9516965	28,4036399	8,71386426	34,2221298	8,57022162
647,855591	510	44,2705895	16,3816856	27,44214	9,34348868	35,7115565	7,61462431
649,681519	510	39,341624	17,3446101	26,5555179	9,71195573	34,4698935	9,25028353
651,505798	510	39,4234034	15,6582852	26,3452503	8,07324845	29,4956854	6,40570778
653,328491	510	37,1434108	16,7483123	25,0650954	8,09813391	29,9073266	5,46163515
655,149536	510	38,1620464	15,9244411	24,6738911	8,04707083	28,9698006	5,64268824
656,968994	510	35,012172	15,0211517	25,0986832	8,08033009	27,2310373	5,90883897
658,786743	510	35,5043626	15,498938	22,7863704	8,28147242	28,8231155	6,99448964
660,602905	510	33,3947299	14,1529289	21,3773114	7,08035681	24,9850054	5,86973615
662,41748	510	33,3815108	13,4811701	22,0448764	8,09315118	26,4869926	5,81421382
664,230469	510	33,2838228	12,8528491	21,2702876	7,2900176	25,9658781	5,33337498
666,041748	510	32,8529948	13,4504063	21,6369351	7,38538059	23,8796822	6,08292249
667,85144	510	31,0537899	13,8985815	22,5182479	7,9816802	23,8630399	4,61594694
669,659424	510	28,5650797	13,5148359	19,4347604	5,68924759	22,4274465	4,95572485
671,46582	510	28,9239036	12,7862282	21,0208945	6,43789917	22,6979922	6,28366975
673,27063	510	27,3826939	11,1800285	20,1101669	4,64019235	23,698408	5,56125682
675,073792	510	28,2642895	10,6207363	18,8222169	4,40006753	21,5854945	4,82349305
676,875305	510	25,9385497	10,2868877	15,6683902	5,89331262	21,4153915	5,48776188
678,675171	510	25,6111361	11,0352702	16,9146009	7,2225307	20,588609	3,40960597

Please do not adjust margins

680,47345	510	24,9290219	11,7730246	18,2273129	4,64543673	19,9922073	3,9055893
682,27002	510	21,1924558	9,06835611	16,1288743	4,16208445	17,564206	4,08589457
684,065063	510	22,3468864	9,09849766	15,9322392	5,17813889	18,040183	2,67720838
685,858398	510	25,6935003	8,68494075	15,1047088	4,26130395	19,5520985	4,0175729
687,650024	510	20,2078044	9,36230424	15,1197135	5,93829668	16,225962	3,68643356
689,440186	510	19,1664615	8,29473692	14,5685702	5,11370973	13,5808338	2,01542978
691,228577	510	20,5156903	7,87968518	15,413423	4,78073514	14,9294914	2,35249144
693,015381	510	16,957726	8,5719056	11,5053023	4,70873284	15,6399064	2,35843682
694,800476	510	19,1356228	7,72959155	12,5938999	3,69879456	15,7200644	1,85259465
696,583984	510	17,8645857	6,32673019	12,3075375	4,97976533	13,0825904	2,87465662
698,365845	510	17,2387537	7,69800259	13,2720061	3,72415847	12,1036594	3,47623818
700,146118	510	16,5440343	6,87943531	11,712433	3,56907668	13,0017916	2,80912277
701,924683	510	16,6076367	7,33217192	10,4794758	3,92401659	13,392999	3,16173102
347,741211	515	0	0	0	0	0	0
349,808533	515	0	0	0	0	0	0
351,874329	515	0	0	0	0	0	0
353,938568	515	0	0	0	0	0	0
356,001343	515	0	0	0	0	0	0
358,062561	515	0	0	0	0	0	0
360,122253	515	0	0	0	0	0	0
362,180481	515	0	0	0	0	0	0
364,237152	515	0	0	0	0	0	0
366,292328	515	0	0	0	0	0	0
368,345978	515	0	0	0	0	0	0
370,398132	515	0	0	0	0	0	0
372,44873	515	0	0	0	0	0	0
374,497803	515	0	0	0	0	0	0
376,545349	515	0	0	0	0	0	0
378,5914	515	0	0	0	0	0	0
380,635925	515	0	0	0	0	0	0
382,678955	515	0	0	0	0	0	0
384,720398	515	0	0	0	0	0	0
386,760376	515	0	0	0	0	0	0
388,798767	515	0	0	0	0	0	0
390,835663	515	0	0	0	0	0	0
392,871033	515	0	0	0	0	0	0
394,904877	515	0	0	0	0	0	0
396,937195	515	0	0	0	0	0	0
398,967957	515	0	0	0	0	0	0
400,997253	515	0	0	0	0	0	0
403,024963	515	0	0	0	0	0	0
405,051147	515	0	0	0	0	0	0
407,075806	515	0	0	0	0	0	0
409,098938	515	0	0	0	0	0	0
411,120544	515	0	0	0	0	0	0
413,140564	515	0	0	0	0	0	0
415,159119	515	0	0	0	0	0	0

 This journal is © The Royal Society of Chemistry 20xx

Please do not adjust margins

417,176086	515	0	0	0	0	0	0
419,191528	515	0	0	0	0	0	0
421,205444	515	0	0	0	0	0	0
423,217834	515	0	0	0	0	0	0
425,228668	515	0	0	0	0	0	0
427,237976	515	0	0	0	0	0	0
429,245728	515	0	0	0	0	0	0
431,251953	515	0	0	0	0	0	0
433,256592	515	0	0	0	0	0	0
435,259766	515	0	0	0	0	0	0
437,261353	515	0	0	0	0	0	0
439,261383	515	0	0	0	0	0	0
441,259888	515	0	0	0	0	0	0
443,256836	515	0	0	0	0	0	0
445,252258	515	0	0	0	0	0	0
447,246094	515	0	0	0	0	0	0
449,238434	515	0	0	0	0	0	0
451,229187	515	0	0	0	0	0	0
453,218445	515	0	0	0	0	0	0
455,206116	515	0	0	0	0	0	0
457,192261	515	0	0	0	0	0	0
459,176819	515	0	0	0	0	0	0
461,15979	515	0	0	0	0	0	0
463,141296	515	0	0	0	0	0	0
465,121216	515	0	0	0	0	0	0
467,099579	515	0	0	0	0	0	0
469,076385	515	0	0	0	0	0	0
471,051636	515	0	0	0	0	0	0
473,02536	515	0	0	0	0	0	0
474,997498	515	0	0	0	0	0	0
476,968079	515	0	0	0	0	0	0
478,937134	515	0	0	0	0	0	0
480,904572	515	0	0	0	0	0	0
482,870453	515	0	0	0	0	0	0
484,834808	515	0	0	0	0	0	0
486,797607	515	0	0	0	0	0	0
488,75885	515	0	0	0	0	0	0
490,718506	515	0	0	0	0	0	0
492,676605	515	0	0	0	0	0	0
494,633118	515	0	0	0	0	0	0
496,588104	515	0	0	0	0	0	0
498,541504	515	0	0	0	0	0	0
500,493347	515	0	0	0	0	0	0
502,443665	515	0	0	0	0	0	0
504,392365	515	0	0	0	0	0	0
506,339539	515	0	0	0	0	0	0
508,285095	515	0	0	0	0	0	0

This journal is © The Royal Society of Chemistry 20xx

Please do not adjust margins

Please do not adjust margins

510,229126	515	0	0	0	0	0	0
512,17157	515	0	0	0	0	0	0
514,112427	515	0	0	0	0	0	0
516,051697	515	0	0	0	0	0	0
517,989441	515	0	0	0	0	0	0
519,925598	515	0	0	0	0	0	0
521,860168	515	0	0	0	0	0	0
523,793152	515	0	0	0	0	0	0
525,724609	515	46,2235485	25,7223057	33,3104494	61,6680692	47,0985041	19,4660544
527,654419	515	50,5001065	25,7772607	34,1136692	44,0589474	48,2251968	15,9874669
529,582764	515	53,8385464	27,1156499	34,139144	35,0680596	48,3345548	13,7242608
531,50946	515	54,7708444	26,223002	36,6406487	28,2365063	50,062821	14,4178109
533,43457	515	59,0189563	25,8396814	40,4018078	26,8991432	54,0791817	13,7863947
535,358093	515	58,6885848	31,7772114	40,7897548	28,6522042	52,5155529	13,376064
537,280029	515	63,4179235	31,1331847	42,3000547	31,5323808	58,0017571	14,1253859
539,200439	515	66,1259927	32,301693	44,3718704	33,9494882	58,4250417	12,6166544
541,119263	515	70,6840354	33,4872661	46,0927374	34,9838872	60,6036963	13,0782339
543,036438	515	68,2960178	35,0304759	48,0041859	40,4013126	61,1342053	14,1055872
544,952087	515	70,5385847	32,8408562	45,7577859	36,367344	61,1541658	13,824795
546,866211	515	73,251266	38,1123973	49,0878309	37,4293029	62,173144	14,0503274
548,778687	515	74,9086582	35,505382	49,3721246	37,4256011	63,0354512	14,121355
550,689514	515	73,6324794	39,2749073	49,2208444	35,4865113	65,8969208	14,2433307
552,598816	515	75,3162382	37,3732371	50,101533	36,7645351	68,3933041	15,2161534
554,506531	515	81,695895	36,9254311	51,9509894	38,9105629	67,6437718	16,1467563
556,41272	515	80,9364999	37,8766391	50,5325507	36,4034559	69,1127721	14,7110924
558,317261	515	78,7046473	37,8057685	52,1897468	38,062581	69,1869227	14,9278814
560,220215	515	79,8700973	40,1162821	52,4137554	36,5543432	68,1163399	15,7956921
562,121582	515	78,6598842	41,2802654	54,6844784	40,1686085	72,2993751	14,8882185
564,021362	515	79,6896837	40,6770953	54,024599	38,91252	70,8633415	15,8458199
565,919556	515	81,3050754	39,3466612	53,1937968	42,3684619	72,7998249	16,7211252
567,816162	515	81,1824093	41,283911	56,1177911	46,2846563	69,6642042	16,5520845
569,711182	515	84,7439644	41,5679528	54,9177362	41,3931374	71,0667817	13,8272953
571,604614	515	83,1420565	39,5534281	55,018031	37,9062315	70,5051127	16,0206791
573,49646	515	80,4399324	40,1112863	51,9425426	34,8495177	69,8284559	17,0259412
575,386719	515	80,5262645	39,7113234	55,709268	36,5765574	68,5542012	17,2972477
577,27533	515	81,1883137	39,631056	54,4551381	33,0613013	70,7850108	16,2767452
579,162354	515	77,7042749	37,7389308	52,3100787	28,3159263	67,8662767	15,430537
581,047852	515	81,5387272	39,7621712	54,6165486	26,4118431	65,5264408	15,9428928
582,931641	515	81,6885575	35,5020333	51,7775228	22,6708493	63,3573555	15,4938385
584,813965	515	80,1739124	36,0430453	51,7610246	22,7292221	66,7452588	14,2580149
586,69458	515	77,32959	35,9253922	49,5867875	22,4210233	63,7105048	14,4512001
588,57373	515	77,7303318	35,8849252	49,0885506	21,7053042	61,1675339	13,8451794
590,451111	515	77,2867422	34,1503059	49,4549421	20,9852826	62,0241961	14,0463495
592,326965	515	77,3634454	35,835119	45,8375377	21,8327217	60,4680516	13,3515309
594,201233	515	72,8157914	34,9770573	46,7752009	17,8963364	60,2095982	13,699745
596,073914	515	75,4611108	32,8826815	48,5241617	16,929231	60,9609248	12,8585159
597,944946	515	73,8271126	32,540075	46,8382282	15,8176562	55,5041516	14,8835963

 This journal is © The Royal Society of Chemistry 20xx

Please do not adjust margins

Please do not adjust margins

599,814453	515	71,8555162	32,3067787	47,1171128	15,5457592	57,9497056	13,285298
601,682251	515	68,101984	30,517955	44,5157614	15,2753432	52,4370726	13,2540115
603,548523	515	67,80044	29,6756622	45,9734588	13,4539157	55,823301	12,5254113
605,413147	515	67,2197231	30,6335234	45,4303296	13,8192796	51,2268682	11,183852
607,276245	515	66,3424663	30,1997594	43,8130739	14,7284831	52,388937	13,3381742
609,137695	515	63,7619173	28,915239	44,51702	12,3060267	53,2350464	13,5494696
610,997437	515	62,0273648	29,3598331	38,7972117	13,8813897	52,0224626	12,5677765
612,855713	515	62,5153338	29,0605486	41,2903122	14,2743358	54,4457102	12,9603901
614,712341	515	63,9283413	31,2369556	38,9937301	14,857357	49,6932636	11,6724755
616,567322	515	59,4813418	27,8478361	41,9904572	16,7421963	48,1895743	11,7629009
618,420715	515	63,8571913	27,6551038	35,9001515	14,2713012	49,1960589	13,1064563
620,272522	515	58,5234844	27,7919733	37,1470863	14,0671927	48,2849599	12,4306997
622,122681	515	57,2400619	26,5218274	38,5566312	12,8443798	46,9012678	12,3791235
623,971313	515	57,2396975	25,7314746	36,3094974	12,3297999	43,8890977	12,0987531
625,818237	515	55,4950005	24,7304419	35,5080798	11,7477323	43,2741542	10,4113063
627,663574	515	53,0656157	25,0895044	31,9424631	14,2958204	42,8764486	12,4049117
629,507324	515	52,9302065	25,2923665	33,0508973	13,1756611	40,0722913	11,116188
631,349365	515	52,4133184	22,6132826	31,4095961	13,5825216	39,8635967	10,1708625
633,18988	515	47,600539	22,3479677	31,3239378	12,4996229	42,1057494	8,84064103
635,028809	515	48,5266639	21,6987281	32,0997323	12,6512751	39,694121	8,67615277
636,865967	515	47,5682322	21,414897	29,5247369	11,5557266	36,5704588	8,76971456
638,70166	515	45,4418776	18,7184681	30,3372236	10,2065262	35,633951	8,13638847
640,535645	515	42,0857975	17,9186458	27,6248901	10,1249277	35,4679994	7,41710929
642,368042	515	40,7000508	16,3455548	25,8249346	9,0196522	33,8349605	7,42604908
644,198853	515	39,8281438	17,3396963	30,6733629	10,3189856	31,0289794	8,32231508
646,028015	515	40,6749588	17,0397823	27,5900158	8,00847792	33,066521	7,53080418
647,855591	515	38,4961623	15,9927587	24,0607024	8,56530261	32,571804	7,52749205
649,681519	515	37,4149458	13,6666514	25,1539359	11,0255257	31,0566434	6,44068777
651,505798	515	37,407031	16,4673397	24,0468369	10,1819126	28,1142521	7,35637284
653,328491	515	37,6844202	16,4371285	25,0922741	9,56483461	28,6060987	5,59510275
655,149536	515	36,8452349	14,6277208	21,2840301	8,62334112	26,4327003	6,91754743
656,968994	515	33,4856137	14,9329814	25,4506246	7,67872003	27,603627	5,39347207
658,786743	515	31,6767048	12,2988635	21,966301	8,2901166	26,0127937	5,00878764
660,602905	515	31,0114838	12,9033322	19,3893229	6,66427312	23,5246013	4,11529802
662,41748	515	34,0427769	12,7136284	19,9715869	6,77685492	24,0433952	6,20067712
664,230469	515	29,3080644	11,4152122	19,5975448	5,2168818	25,0014882	4,97032991
666,041748	515	29,1956628	13,5906601	22,1185362	6,13894819	23,8030503	7,22013004
667,85144	515	27,6125158	12,2984573	19,022729	7,56999963	21,5398513	4,99308817
669,659424	515	27,0020078	13,1561605	17,3134584	5,33479327	21,0003547	2,91856375
671,46582	515	26,6124927	10,669053	17,4978066	6,50842715	21,3555067	4,25711262
673,27063	515	24,7067304	10,9618937	16,6462258	5,2774045	19,6805017	3,10059524
675,073792	515	25,3416985	9,97692636	16,5106605	4,53220045	20,305757	2,93864356
676,875305	515	26,8470402	9,80738729	14,3288716	5,62221968	19,8824466	4,95277431
678,675171	515	23,0753418	8,04836586	15,9234986	4,78384255	19,2143622	3,44230539
680,47345	515	22,638497	8,29354203	16,4975517	4,44473906	17,942074	3,52357247
682,27002	515	23,3059042	8,57447205	15,294209	5,30167756	17,2470607	4,12507985
684,065063	515	20,4287915	9,69942923	14,0803427	5,91033262	17,1355635	2,61841871

This journal is © The Royal Society of Chemistry 20xx

Please do not adjust margins

Please do not adjust margins

685,858398	515	22,3801769	8,10046412	13,7491133	4,56139806	15,8763452	2,70406863
687,650024	515	19,3590628	7,77065635	14,016074	6,59523117	15,8921165	3,04509916
689,440186	515	20,0816728	9,05850124	13,7099541	4,15273478	15,7593946	3,05213777
691,228577	515	18,6503855	8,72415527	12,5313048	3,56123798	14,5803726	2,12058279
693,015381	515	18,1875877	8,06688679	12,7327447	4,33528923	14,2772858	2,80624354
694,800476	515	17,2484024	7,1310446	11,9657905	4,92909745	12,9143178	2,63550972
696,583984	515	17,147398	6,47793806	12,7809423	4,5223546	12,5399137	1,87791073
698,365845	515	16,5111791	6,92351804	12,3896269	2,90928502	12,3185138	4,45117035
700,146118	515	16,0617736	5,32064901	10,2086496	4,8109018	11,8523924	1,71882626
701,924683	515	15,5199677	4,99651655	10,5062464	3,44956928	13,0187803	3,19205322
347,741211	520	0	0	0	0	0	0
349,808533	520	0	0	0	0	0	0
351,874329	520	0	0	0	0	0	0
353,938568	520	0	0	0	0	0	0
356,001343	520	0	0	0	0	0	0
358,062561	520	0	0	0	0	0	0
360,122253	520	0	0	0	0	0	0
362,180481	520	0	0	0	0	0	0
364,237152	520	0	0	0	0	0	0
366,292328	520	0	0	0	0	0	0
368,345978	520	0	0	0	0	0	0
370,398132	520	0	0	0	0	0	0
372,44873	520	0	0	0	0	0	0
374,497803	520	0	0	0	0	0	0
376,545349	520	0	0	0	0	0	0
378,5914	520	0	0	0	0	0	0
380,635925	520	0	0	0	0	0	0
382,678955	520	0	0	0	0	0	0
384,720398	520	0	0	0	0	0	0
386,760376	520	0	0	0	0	0	0
388,798767	520	0	0	0	0	0	0
390,835663	520	0	0	0	0	0	0
392,871033	520	0	0	0	0	0	0
394,904877	520	0	0	0	0	0	0
396,937195	520	0	0	0	0	0	0
398,967957	520	0	0	0	0	0	0
400,997253	520	0	0	0	0	0	0
403,024963	520	0	0	0	0	0	0
405,051147	520	0	0	0	0	0	0
407,075806	520	0	0	0	0	0	0
409,098938	520	0	0	0	0	0	0
411,120544	520	0	0	0	0	0	0
413,140564	520	0	0	0	0	0	0
415,159119	520	0	0	0	0	0	0
417,176086	520	0	0	0	0	0	0
419,191528	520	0	0	0	0	0	0
421,205444	520	0	0	0	0	0	0

This journal is © The Royal Society of Chemistry 20xx

Please do not adjust margins

Please do not adjust margins

423,217834	520	0	0	0	0	0	0
425,228668	520	0	0	0	0	0	0
427,237976	520	0	0	0	0	0	0
429,245728	520	0	0	0	0	0	0
431,251953	520	0	0	0	0	0	0
433,256592	520	0	0	0	0	0	0
435,259766	520	0	0	0	0	0	0
437,261353	520	0	0	0	0	0	0
439,261383	520	0	0	0	0	0	0
441,259888	520	0	0	0	0	0	0
443,256836	520	0	0	0	0	0	0
445,252258	520	0	0	0	0	0	0
447,246094	520	0	0	0	0	0	0
449,238434	520	0	0	0	0	0	0
451,229187	520	0	0	0	0	0	0
453,218445	520	0	0	0	0	0	0
455,206116	520	0	0	0	0	0	0
457,192261	520	0	0	0	0	0	0
459,176819	520	0	0	0	0	0	0
461,15979	520	0	0	0	0	0	0
463,141296	520	0	0	0	0	0	0
465,121216	520	0	0	0	0	0	0
467,099579	520	0	0	0	0	0	0
469,076385	520	0	0	0	0	0	0
471,051636	520	0	0	0	0	0	0
473,02536	520	0	0	0	0	0	0
474,997498	520	0	0	0	0	0	0
476,968079	520	0	0	0	0	0	0
478,937134	520	0	0	0	0	0	0
480,904572	520	0	0	0	0	0	0
482,870453	520	0	0	0	0	0	0
484,834808	520	0	0	0	0	0	0
486,797607	520	0	0	0	0	0	0
488,75885	520	0	0	0	0	0	0
490,718506	520	0	0	0	0	0	0
492,676605	520	0	0	0	0	0	0
494,633118	520	0	0	0	0	0	0
496,588104	520	0	0	0	0	0	0
498,541504	520	0	0	0	0	0	0
500,493347	520	0	0	0	0	0	0
502,443665	520	0	0	0	0	0	0
504,392365	520	0	0	0	0	0	0
506,339539	520	0	0	0	0	0	0
508,285095	520	0	0	0	0	0	0
510,229126	520	0	0	0	0	0	0
512,17157	520	0	0	0	0	0	0
514,112427	520	0	0	0	0	0	0

This journal is © The Royal Society of Chemistry 20xx

Please do not adjust margins

Please do not adjust margins

516,051697	520	0	0	0	0	0	0
517,989441	520	0	0	0	0	0	0
519,925598	520	0	0	0	0	0	0
521,860168	520	0	0	0	0	0	0
523,793152	520	0	0	0	0	0	0
525,724609	520	0	0	0	0	0	0
527,654419	520	0	0	0	0	0	0
529,582764	520	0	0	0	0	0	0
531,50946	520	46,1168987	21,6194311	31,4544907	53,0841124	43,0246822	17,002729
533,43457	520	44,2035345	22,0411437	30,5533869	39,7522801	43,2699374	12,1825229
535,358093	520	47,1924362	23,8347135	32,7669922	31,881741	44,5509261	12,5171876
537,280029	520	50,4796475	23,4386775	34,1877791	28,3365965	45,4780767	10,9810851
539,200439	520	54,5303153	25,2753795	36,1969245	25,054571	47,2902356	10,9313456
541,119263	520	53,4268362	29,6922735	38,7930841	28,065746	46,6589781	11,9251123
543,036438	520	57,5890148	28,6647287	39,4718992	30,6672981	50,3039675	12,7439142
544,952087	520	59,1024736	27,5790759	38,1173407	33,2600542	50,0810559	11,5809792
546,866211	520	62,128993	29,0658421	40,2153844	33,6929247	51,3169095	12,0411812
548,778687	520	62,5629157	29,2097501	41,4588412	34,3501726	52,7799839	11,8931612
550,689514	520	62,096536	27,9905675	41,8319772	30,9242442	56,0667485	13,1201528
552,598816	520	64,5655532	33,10729	44,2814667	34,1334835	55,5564666	12,3551965
554,506531	520	65,4871221	30,7554706	42,436578	34,4842339	55,4597173	14,6153022
556,41272	520	67,2667701	32,960539	43,0384875	35,3754055	56,1402805	13,6708932
558,317261	520	67,999184	34,0578129	45,8165436	33,7684492	57,5308997	12,6477847
560,220215	520	66,4025286	31,1391786	46,1159177	36,0398334	60,0986844	12,942207
562,121582	520	69,0993082	34,9379085	45,4040043	36,1862663	61,1731147	12,5288773
564,021362	520	68,5918694	32,484586	46,628846	34,3941637	57,8690305	13,7167978
565,919556	520	70,7599048	34,4078512	46,9986402	35,2214207	62,5668018	12,455607
567,816162	520	70,2317587	33,9189603	47,7294364	38,6518101	61,7180101	15,0059469
569,711182	520	74,0971702	34,9220194	46,3321444	39,6707605	61,8500545	12,3860388
571,604614	520	72,1231015	36,3452163	49,1468078	41,264397	64,1395904	14,3787876
573,49646	520	71,8872098	34,3605408	46,0798722	41,7592095	63,8798471	14,794656
575,386719	520	69,2514817	34,8351038	47,0378803	38,059771	62,6420595	15,1374614
577,27533	520	74,597332	34,8744567	46,993197	32,7683081	61,1002389	13,562398
579,162354	520	72,7048148	35,2046168	45,7730942	33,8477668	62,0372382	12,6202424
581,047852	520	70,4254168	33,3008295	47,8568195	32,6210014	58,9531674	14,0378236
582,931641	520	71,2594923	32,9216368	46,3400867	31,1038285	60,6225308	14,493143
584,813965	520	69,6188952	32,8543001	44,178254	25,7837732	59,3333226	14,3022328
586,69458	520	71,1230739	31,6558362	45,6831938	21,9703251	58,8241507	13,0478611
588,57373	520	68,4266294	30,9764663	45,2299869	20,3971949	56,9049449	11,2747096
590,451111	520	66,8787188	29,785808	44,6322121	19,5752573	55,7929745	12,4598282
592,326965	520	64,4859497	28,1572652	43,2451138	19,7900455	54,9187542	13,4571762
594,201233	520	64,0938785	31,7123831	39,3574828	18,6977554	52,7867031	13,2571335
596,073914	520	67,498188	31,227884	42,66269	18,1980055	53,5680082	12,0023
597,944946	520	63,5496244	25,3249137	44,4497436	17,4669981	51,058007	11,3561429
599,814453	520	62,053264	29,700014	41,0184353	15,8414045	49,7485727	11,5668676
601,682251	520	63,4074311	26,3115035	40,8428386	15,8831191	47,260489	10,9863582
603,548523	520	61,9319643	27,9108068	38,3903465	12,8527196	46,8436974	11,2782752

 This journal is © The Royal Society of Chemistry 20xx

Please do not adjust margins

Please do not adjust margins

605,413147	520	60,8830035	27,155076	39,5056842	12,9096384	49,6771739	11,0229411
607,276245	520	60,5547907	25,6205559	38,1883734	12,4184198	47,0861699	11,7137173
609,137695	520	56,3818508	26,8357269	39,3835551	11,4571979	46,3842279	11,6952914
610,997437	520	58,772102	24,9233093	34,6664186	10,7722225	43,9596116	10,5387418
612,855713	520	56,0424772	26,9773156	35,2295073	11,9560164	44,7623083	10,9359606
614,712341	520	55,9572353	27,3655508	37,9265849	13,0135854	45,3563563	9,70325294
616,567322	520	55,6227935	24,3159485	36,2307335	13,3433693	45,1681311	10,9772314
618,420715	520	53,0181675	26,0911338	33,4382331	12,2560073	44,4437731	9,9652243
620,272522	520	51,547265	24,1658067	31,9249662	13,7876106	41,6060374	10,7789855
622,122681	520	54,6736692	23,3444233	36,3601142	11,7526509	41,2191252	10,563917
623,971313	520	50,7652772	24,5230012	33,5142065	13,6162437	41,4115838	9,95566873
625,818237	520	49,3680482	23,4208106	32,9272499	15,1888958	40,2958603	12,3937725
627,663574	520	49,2947795	23,8623548	34,9065933	15,6160053	39,8060439	12,7359598
629,507324	520	47,6075402	22,2900285	33,1475942	16,739115	38,4728488	8,53245963
631,349365	520	46,4354668	21,4206408	32,6084224	14,8185922	37,5346467	10,230387
633,18988	520	45,4145864	20,2599099	29,8599759	14,0459571	39,0755179	10,1735343
635,028809	520	42,4045664	18,7957484	29,1162102	15,6510592	38,325948	8,3921756
636,865967	520	44,167853	20,6032609	28,3548165	14,9487821	37,4981081	8,56720519
638,70166	520	42,4147584	16,4916717	26,7538599	12,2081473	35,3355247	7,6015103
640,535645	520	40,064488	18,1051763	26,9105711	11,7209022	33,3887673	7,3575573
642,368042	520	39,7084147	16,265744	24,8393481	10,7638545	31,4864046	6,55692802
644,198853	520	40,0372236	17,111823	25,0551061	11,8414526	30,0131198	5,92207913
646,028015	520	36,1076898	15,5077888	22,4052366	9,50037338	29,3981956	7,30964931
647,855591	520	36,4973352	14,2024579	23,2068095	8,60339036	28,1676444	5,92633016
649,681519	520	36,3666509	14,0337493	23,167947	9,13906135	26,6863154	6,85586214
651,505798	520	31,3579241	13,6817553	20,5599954	9,2585312	25,9764743	5,4908259
653,328491	520	33,2613743	15,2853339	22,1845024	8,9583244	28,6868595	4,76790398
655,149536	520	32,8051843	13,2789001	20,2859466	8,33097641	24,9137307	6,27028589
656,968994	520	28,9680147	14,1619683	21,9430667	7,86845408	27,0047836	3,56232456
658,786743	520	32,4334823	11,3173488	21,9651241	7,57330569	23,3055533	5,41074007
660,602905	520	29,0092647	13,1792708	18,180873	7,25758032	25,2797351	4,58917382
662,41748	520	29,0558456	11,6465062	19,6866884	7,20638759	22,4600776	6,1184476
664,230469	520	27,183487	11,3336747	17,2073812	6,46496239	20,9929068	4,36694619
666,041748	520	27,6533567	12,1113141	19,1728893	5,21505349	19,9375925	4,45904821
667,85144	520	26,5494011	13,0723206	16,1899745	6,74759927	19,6549756	5,90549839
669,659424	520	26,5189899	10,4791446	16,814392	4,47938426	17,9199009	2,95875983
671,46582	520	24,0098526	11,0820286	16,0703567	8,37570686	19,6305086	3,63876451
673,27063	520	25,4618994	9,6816909	16,1333258	5,43581462	19,9622651	4,2477005
675,073792	520	23,8102025	9,7003349	14,3778028	4,34000625	18,9793937	3,74517465
676,875305	520	23,380587	8,59252708	13,3543616	5,70049594	16,8484642	4,59547933
678,675171	520	22,5339644	9,35968503	14,037069	5,53121077	18,8937045	3,48971474
680,47345	520	21,0722927	9,09747153	15,811527	4,33656046	14,4571086	3,14685095
682,27002	520	20,6337344	9,21431478	13,6484463	4,86353949	17,3233117	4,35258225
684,065063	520	20,4456768	9,24494147	11,4685669	5,90701132	16,69593	3,68202207
685,858398	520	21,3104818	9,07771631	12,1585848	4,3679657	16,617591	2,91264248
687,650024	520	18,0762186	7,71515148	12,5134985	4,88669356	13,6331014	3,00128691
689,440186	520	18,2897356	7,56114061	12,2847019	6,27286303	14,4380825	3,26607205

This journal is © The Royal Society of Chemistry 20xx

Please do not adjust margins

Please do not adjust margins

691,228577	520	18,7282806	6,10346674	13,4080856	4,72845461	14,5311683	2,49375482
693,015381	520	18,4311054	7,15307076	11,0293099	4,7404047	15,4298979	3,70698139
694,800476	520	17,9098709	7,23744413	10,5096853	3,36053828	13,7886426	3,01655678
696,583984	520	16,5989142	6,22856165	10,9845668	5,01789496	12,0272314	1,38456317
698,365845	520	15,3451214	5,55205622	11,8827891	3,73061677	12,4948765	3,55791247
700,146118	520	16,9731631	4,79035482	10,449853	3,57130673	12,3705478	1,74249889
701,924683	520	16,3394034	4,45904222	8,56685521	3,58503638	12,505557	3,14855606
347,741211	525	0	0	0	0	0	0
349,808533	525	0	0	0	0	0	0
351,874329	525	0	0	0	0	0	0
353,938568	525	0	0	0	0	0	0
356,001343	525	0	0	0	0	0	0
358,062561	525	0	0	0	0	0	0
360,122253	525	0	0	0	0	0	0
362,180481	525	0	0	0	0	0	0
364,237152	525	0	0	0	0	0	0
366,292328	525	0	0	0	0	0	0
368,345978	525	0	0	0	0	0	0
370,398132	525	0	0	0	0	0	0
372,44873	525	0	0	0	0	0	0
374,497803	525	0	0	0	0	0	0
376,545349	525	0	0	0	0	0	0
378,5914	525	0	0	0	0	0	0
380,635925	525	0	0	0	0	0	0
382,678955	525	0	0	0	0	0	0
384,720398	525	0	0	0	0	0	0
386,760376	525	0	0	0	0	0	0
388,798767	525	0	0	0	0	0	0
390,835663	525	0	0	0	0	0	0
392,871033	525	0	0	0	0	0	0
394,904877	525	0	0	0	0	0	0
396,937195	525	0	0	0	0	0	0
398,967957	525	0	0	0	0	0	0
400,997253	525	0	0	0	0	0	0
403,024963	525	0	0	0	0	0	0
405,051147	525	0	0	0	0	0	0
407,075806	525	0	0	0	0	0	0
409,098938	525	0	0	0	0	0	0
411,120544	525	0	0	0	0	0	0
413,140564	525	0	0	0	0	0	0
415,159119	525	0	0	0	0	0	0
417,176086	525	0	0	0	0	0	0
419,191528	525	0	0	0	0	0	0
421,205444	525	0	0	0	0	0	0
423,217834	525	0	0	0	0	0	0
425,228668	525	0	0	0	0	0	0
427,237976	525	0	0	0	0	0	0

This journal is © The Royal Society of Chemistry 20xx

Please do not adjust margins

Please do not adjust margins

429,245728	525	0	0	0	0	0	0
431,251953	525	0	0	0	0	0	0
433,256592	525	0	0	0	0	0	0
435,259766	525	0	0	0	0	0	0
437,261353	525	0	0	0	0	0	0
439,261383	525	0	0	0	0	0	0
441,259888	525	0	0	0	0	0	0
443,256836	525	0	0	0	0	0	0
445,252258	525	0	0	0	0	0	0
447,246094	525	0	0	0	0	0	0
449,238434	525	0	0	0	0	0	0
451,229187	525	0	0	0	0	0	0
453,218445	525	0	0	0	0	0	0
455,206116	525	0	0	0	0	0	0
457,192261	525	0	0	0	0	0	0
459,176819	525	0	0	0	0	0	0
461,15979	525	0	0	0	0	0	0
463,141296	525	0	0	0	0	0	0
465,121216	525	0	0	0	0	0	0
467,099579	525	0	0	0	0	0	0
469,076385	525	0	0	0	0	0	0
471,051636	525	0	0	0	0	0	0
473,02536	525	0	0	0	0	0	0
474,997498	525	0	0	0	0	0	0
476,968079	525	0	0	0	0	0	0
478,937134	525	0	0	0	0	0	0
480,904572	525	0	0	0	0	0	0
482,870453	525	0	0	0	0	0	0
484,834808	525	0	0	0	0	0	0
486,797607	525	0	0	0	0	0	0
488,75885	525	0	0	0	0	0	0
490,718506	525	0	0	0	0	0	0
492,676605	525	0	0	0	0	0	0
494,633118	525	0	0	0	0	0	0
496,588104	525	0	0	0	0	0	0
498,541504	525	0	0	0	0	0	0
500,493347	525	0	0	0	0	0	0
502,443665	525	0	0	0	0	0	0
504,392365	525	0	0	0	0	0	0
506,339539	525	0	0	0	0	0	0
508,285095	525	0	0	0	0	0	0
510,229126	525	0	0	0	0	0	0
512,17157	525	0	0	0	0	0	0
514,112427	525	0	0	0	0	0	0
516,051697	525	0	0	0	0	0	0
517,989441	525	0	0	0	0	0	0
519,925598	525	0	0	0	0	0	0

This journal is © The Royal Society of Chemistry 20xx

Please do not adjust margins

Please do not adjust margins

521,860168	525	0	0	0	0	0	0
523,793152	525	0	0	0	0	0	0
525,724609	525	0	0	0	0	0	0
527,654419	525	0	0	0	0	0	0
529,582764	525	0	0	0	0	0	0
531,50946	525	0	0	0	0	0	0
533,43457	525	0	0	0	0	0	0
535,358093	525	34,514407	22,1666645	28,005982	57,856843	41,7288905	12,8212332
537,280029	525	39,7737803	20,1221264	29,0138187	44,7704738	41,1040954	10,5146537
539,200439	525	41,6680822	21,3712619	30,6568012	31,4698345	38,0331147	7,60258107
541,119263	525	41,9529095	20,3124088	29,8620681	24,9713903	42,6087862	9,67185346
543,036438	525	45,4883875	20,9186731	30,9945346	25,2793662	39,8458541	9,82492142
544,952087	525	46,0328911	20,8259763	32,7950798	24,2711793	41,7610744	10,4533878
546,866211	525	48,3441236	22,2895878	34,7201413	27,1318869	43,203169	10,33055
548,778687	525	49,527406	24,3675391	34,0490784	28,467082	45,0577126	9,51208356
550,689514	525	51,0048834	23,7351171	33,4760866	31,3897689	45,5793849	11,6484707
552,598816	525	52,4259906	26,8549974	35,7535113	30,7165855	49,3118627	9,54632157
554,506531	525	54,8427427	25,3645674	37,2150601	32,1091952	48,6247519	11,3915264
556,41272	525	54,8651598	25,6708528	35,8267497	29,6798394	49,4590732	11,9881922
558,317261	525	55,9759791	30,1845052	37,4530082	33,4555893	47,6835361	9,62044387
560,220215	525	61,0875322	28,1913315	38,3453599	30,5736307	48,2832947	11,1762323
562,121582	525	55,5795168	27,5071434	39,2375878	28,4103581	51,5721309	9,71562487
564,021362	525	58,0912004	27,0189241	39,9494019	29,1853946	51,4823771	12,0284465
565,919556	525	60,7537738	30,2056895	40,5123539	33,652445	51,8772831	11,9366256
567,816162	525	59,7381274	29,3978422	41,3797172	33,0789355	52,7052955	11,9721113
569,711182	525	61,1379722	30,4717596	38,5952251	32,7389194	53,4823132	11,4865813
571,604614	525	63,221439	31,5889011	39,2204947	35,2244099	52,2137973	12,3677929
573,49646	525	62,9538276	30,1695001	41,6007984	35,1010049	57,4375204	11,9503373
575,386719	525	59,574675	29,0439706	39,9802942	35,5066271	58,363623	12,3643895
577,27533	525	60,1282332	30,269833	42,40622	37,2259416	58,4530231	14,1097431
579,162354	525	61,3767982	29,5184852	39,6304469	36,6478607	56,1008481	12,9277018
581,047852	525	58,4050187	28,3341348	43,4731888	35,4921179	55,4854964	11,6749502
582,931641	525	62,0123272	29,4092211	39,7163525	33,4179924	52,3347976	12,8271177
584,813965	525	61,0947588	28,9460545	42,5859308	30,1921437	52,0843101	13,2451792
586,69458	525	60,274235	28,8010011	41,4797258	29,6644519	52,0082817	10,9676847
588,57373	525	59,0350784	28,6397968	37,122219	26,2462111	52,7438746	11,2574345
590,451111	525	55,8884221	29,4462205	40,0374606	21,0765899	50,1626392	11,9856338
592,326965	525	59,5878789	27,4732795	37,3145215	19,6444132	52,9317412	10,2956138
594,201233	525	59,5410698	25,6330873	35,561952	18,1354398	49,8699765	11,8218504
596,073914	525	58,4466196	24,7738659	39,1135177	16,0140984	49,1861654	11,4250805
597,944946	525	54,21707	24,5907501	37,2325841	18,5724279	47,372285	12,0578387
599,814453	525	57,4539011	27,2582119	35,703283	16,5231185	46,6981977	10,5055211
601,682251	525	57,7982527	26,1542484	37,6157952	16,3241663	42,2656062	9,4407035
603,548523	525	55,2811848	23,6240252	33,7895876	15,8100676	43,0536168	11,7356563
605,413147	525	54,5503422	22,8668784	33,5731651	16,1818204	42,9805967	8,44809349
607,276245	525	53,5132087	24,3436353	35,7463225	13,2123006	44,1891532	10,185957
609,137695	525	51,761855	23,4316208	31,2486591	11,522711	40,9398487	9,69321857

This journal is © The Royal Society of Chemistry 20xx

Please do not adjust margins

610,997437	525	50,5916146	22,443398	32,1846933	11,6251539	39,0296154	10,6677336
612,855713	525	48,9389569	23,0884974	31,0039571	11,8683485	40,0796893	10,1937858
614,712341	525	50,666794	21,0752	34,5152102	11,181554	39,9481316	10,3809952
616,567322	525	50,0821563	20,2187396	30,4027859	12,9525394	37,5915388	8,2337683
618,420715	525	48,0760151	23,2097289	29,2487967	11,0498786	38,3677448	8,80628254
620,272522	525	45,773334	21,0133749	30,9927655	12,1967594	37,4860851	9,5470531
622,122681	525	45,3899021	19,8529857	30,1766878	11,579711	37,7277068	9,00481427
623,971313	525	45,8363628	19,5849395	30,017854	10,3212496	38,4762758	8,86822098
625,818237	525	42,0545146	21,5233068	28,8436638	10,9286742	36,6846391	9,30792896
627,663574	525	42,2904706	19,2915468	28,8943652	13,948416	33,5041677	9,72969439
629,507324	525	44,4527341	20,2011268	28,3859994	15,8914994	35,3811816	8,79985527
631,349365	525	45,2187741	20,2975317	30,6045906	18,7576344	32,9597519	8,1540194
633,18988	525	37,7294733	18,6290922	25,9441122	13,3244963	36,7983248	9,72594181
635,028809	525	40,6450839	18,209651	27,7223809	14,9504478	33,425565	7,10630548
636,865967	525	39,1567172	18,565844	26,7865963	13,5014103	33,2333073	8,26300611
638,70166	525	37,2152456	16,1210676	26,5561909	13,6725775	32,5963771	8,92240494
640,535645	525	40,7248095	16,4453778	25,3258681	13,9156363	31,1983383	7,20208645
642,368042	525	37,7445091	16,3832829	24,1294709	13,522632	29,5142753	7,86629134
644,198853	525	34,5438628	15,2693246	23,4437869	11,9912951	29,0849843	6,56938523
646,028015	525	33,7624001	15,1226323	23,8812693	10,1139553	28,1315955	6,65920597
647,855591	525	33,1727752	13,5550918	23,2145251	10,027316	29,0236424	8,21762615
649,681519	525	32,7725892	11,8106654	20,7823051	10,7420713	26,9472602	7,68332379
651,505798	525	29,8402756	13,269006	20,5364294	8,21409939	24,5679437	6,88532366
653,328491	525	29,6828215	13,9754025	19,1876537	7,57360738	24,6436734	4,0773593
655,149536	525	28,6223527	12,5233508	19,7575224	6,43170866	24,3989326	6,09649199
656,968994	525	26,4782678	12,3236087	18,7509568	7,38090455	21,8148687	5,11538424
658,786743	525	27,5314145	13,0566135	19,1773479	8,51190125	23,2684054	4,97140017
660,602905	525	28,946989	10,301185	17,3679845	7,09598996	22,3940126	5,06764828
662,41748	525	27,6358892	11,4500581	17,1072476	6,19445544	21,5608723	6,70237732
664,230469	525	28,2850004	10,8777733	16,8864345	6,20667843	21,3482581	4,16537373
666,041748	525	25,0179135	10,4719352	15,897114	5,62175768	21,4724235	6,0465555
667,85144	525	23,9774113	12,8057586	16,4522845	6,32051001	17,0884526	4,78222427
669,659424	525	22,829184	10,4365519	15,204497	5,22075596	18,7499455	4,27853482
671,46582	525	24,4787358	9,16272831	16,2461134	7,19658847	18,8548205	3,42632758
673,27063	525	22,684294	9,19863101	14,764635	6,27868408	17,3799883	3,61174076
675,073792	525	19,6287075	8,44113821	15,0510858	5,60137493	17,7582778	3,27405818
676,875305	525	20,4857313	9,98965716	13,3284345	4,91104373	16,8068334	4,1348684
678,675171	525	20,6611633	7,49343355	14,1905883	6,29058187	13,1027931	1,20619494
680,47345	525	20,1292579	9,55329007	13,2344396	3,96090265	14,1264333	2,75491259
682,27002	525	17,8683567	6,8227497	14,3151296	5,27070287	15,5583502	3,19640596
684,065063	525	17,8411404	6,49882335	13,3244422	4,6813766	15,263173	2,68697127
685,858398	525	19,4952244	6,93511691	10,3872335	4,4232382	16,22423	4,50915333
687,650024	525	16,3922561	7,28910645	11,3506609	4,94853007	13,8086372	1,99641444
689,440186	525	18,6903779	8,17571144	12,0716699	4,95996838	12,7959851	1,47902148
691,228577	525	15,3945316	6,7874383	11,990719	4,52710937	13,0635509	2,69836594
693,015381	525	14,9102704	7,58973723	9,32064814	4,18943133	13,4458077	3,05424162
694,800476	525	15,4295828	6,7156758	11,0600621	4,45015896	12,4822743	2,442783

This journal is © The Royal Society of Chemistry 20xx

696,583984	525	14,178989	5,16654648	11,4544561	3,76723868	10,0787079	0,78834932
698,365845	525	14,7454538	5,35669322	11,7496948	2,89926045	11,864772	4,56770601
700,146118	525	12,6013191	4,6727648	9,24362246	4,23394917	11,9121598	1,32287001
701,924683	525	14,5958816	4,86773757	9,54427779	3,45330888	9,74351911	3,54121519
347,741211	530	0	0	0	0	0	0
349,808533	530	0	0	0	0	0	0
351,874329	530	0	0	0	0	0	0
353,938568	530	0	0	0	0	0	0
356,001343	530	0	0	0	0	0	0
358,062561	530	0	0	0	0	0	0
360,122253	530	0	0	0	0	0	0
362,180481	530	0	0	0	0	0	0
364,237152	530	0	0	0	0	0	0
366,292328	530	0	0	0	0	0	0
368,345978	530	0	0	0	0	0	0
370,398132	530	0	0	0	0	0	0
372,44873	530	0	0	0	0	0	0
374,497803	530	0	0	0	0	0	0
376,545349	530	0	0	0	0	0	0
378,5914	530	0	0	0	0	0	0
380,635925	530	0	0	0	0	0	0
382,678955	530	0	0	0	0	0	0
384,720398	530	0	0	0	0	0	0
386,760376	530	0	0	0	0	0	0
388,798767	530	0	0	0	0	0	0
390,835663	530	0	0	0	0	0	0
392,871033	530	0	0	0	0	0	0
394,904877	530	0	0	0	0	0	0
396,937195	530	0	0	0	0	0	0
398,967957	530	0	0	0	0	0	0
400,997253	530	0	0	0	0	0	0
403,024963	530	0	0	0	0	0	0
405,051147	530	0	0	0	0	0	0
407,075806	530	0	0	0	0	0	0
409,098938	530	0	0	0	0	0	0
411,120544	530	0	0	0	0	0	0
413,140564	530	0	0	0	0	0	0
415,159119	530	0	0	0	0	0	0
417,176086	530	0	0	0	0	0	0
419,191528	530	0	0	0	0	0	0
421,205444	530	0	0	0	0	0	0
423,217834	530	0	0	0	0	0	0
425,228668	530	0	0	0	0	0	0
427,237976	530	0	0	0	0	0	0
429,245728	530	0	0	0	0	0	0
431,251953	530	0	0	0	0	0	0
433,256592	530	0	0	0	0	0	0

 This journal is © The Royal Society of Chemistry 20xx

Please do not adjust margins

435,259766	530	0	0	0	0	0	0
437,261353	530	0	0	0	0	0	0
439,261383	530	0	0	0	0	0	0
441,259888	530	0	0	0	0	0	0
443,256836	530	0	0	0	0	0	0
445,252258	530	0	0	0	0	0	0
447,246094	530	0	0	0	0	0	0
449,238434	530	0	0	0	0	0	0
451,229187	530	0	0	0	0	0	0
453,218445	530	0	0	0	0	0	0
455,206116	530	0	0	0	0	0	0
457,192261	530	0	0	0	0	0	0
459,176819	530	0	0	0	0	0	0
461,15979	530	0	0	0	0	0	0
463,141296	530	0	0	0	0	0	0
465,121216	530	0	0	0	0	0	0
467,099579	530	0	0	0	0	0	0
469,076385	530	0	0	0	0	0	0
471,051636	530	0	0	0	0	0	0
473,02536	530	0	0	0	0	0	0
474,997498	530	0	0	0	0	0	0
476,968079	530	0	0	0	0	0	0
478,937134	530	0	0	0	0	0	0
480,904572	530	0	0	0	0	0	0
482,870453	530	0	0	0	0	0	0
484,834808	530	0	0	0	0	0	0
486,797607	530	0	0	0	0	0	0
488,75885	530	0	0	0	0	0	0
490,718506	530	0	0	0	0	0	0
492,676605	530	0	0	0	0	0	0
494,633118	530	0	0	0	0	0	0
496,588104	530	0	0	0	0	0	0
498,541504	530	0	0	0	0	0	0
500,493347	530	0	0	0	0	0	0
502,443665	530	0	0	0	0	0	0
504,392365	530	0	0	0	0	0	0
506,339539	530	0	0	0	0	0	0
508,285095	530	0	0	0	0	0	0
510,229126	530	0	0	0	0	0	0
512,17157	530	0	0	0	0	0	0
514,112427	530	0	0	0	0	0	0
516,051697	530	0	0	0	0	0	0
517,989441	530	0	0	0	0	0	0
519,925598	530	0	0	0	0	0	0
521,860168	530	0	0	0	0	0	0
523,793152	530	0	0	0	0	0	0
525,724609	530	0	0	0	0	0	0

This journal is © The Royal Society of Chemistry 20xx

Please do not adjust margins

Please do not adjust margins

527,654419	530	0	0	0	0	0	0
529,582764	530	0	0	0	0	0	0
531,50946	530	0	0	0	0	0	0
533,43457	530	0	0	0	0	0	0
535,358093	530	0	0	0	0	0	0
537,280029	530	0	0	0	0	0	0
539,200439	530	0	0	0	0	0	0
541,119263	530	32,9805043	18,8638495	24,4810684	50,8884349	36,5494076	9,86504475
543,036438	530	34,5836375	18,7940531	23,4274013	38,007744	34,5888354	8,42590345
544,952087	530	36,6955443	16,6737114	23,1726259	27,1144033	34,9641782	8,31299314
546,866211	530	40,0575044	18,3235428	26,9718626	21,9516955	35,854224	6,92041564
548,778687	530	44,4120895	18,6971759	27,4199337	20,6716412	34,1220289	6,54365885
550,689514	530	40,9647152	19,4169049	28,2760316	21,9894039	36,1684256	8,63580466
552,598816	530	45,2740933	22,3577717	29,6991968	24,1782825	38,3203632	9,73700541
554,506531	530	45,1799948	20,0312003	31,3316359	27,6098561	39,8002811	9,05904214
556,41272	530	47,5182508	22,5069247	31,1948273	29,5673467	41,0140397	10,4858207
558,317261	530	48,4176912	22,1337219	31,0461465	31,2899318	43,8593994	10,4861364
560,220215	530	47,2275992	20,7161583	33,2207391	32,7920947	41,6952876	11,0870472
562,121582	530	48,0734743	24,2803773	33,1382492	31,8834236	43,7301839	9,30800992
564,021362	530	50,3057199	23,0326486	34,6021673	30,7181044	39,1993024	10,2955797
565,919556	530	50,4702836	23,2933015	32,730992	29,2044567	43,3853136	10,2691904
567,816162	530	47,518032	24,0430115	33,3577848	30,9564142	42,379136	9,84531978
569,711182	530	50,6376138	24,8819368	35,214797	29,6929054	43,9633958	9,04236496
571,604614	530	52,9243903	26,2892641	36,1355397	32,4200635	45,3052057	10,6859245
573,49646	530	53,8448008	23,7680989	34,4831926	31,5972805	45,5913702	10,2580211
575,386719	530	51,9304136	25,9067393	36,2705435	32,7634424	45,033581	11,8233761
577,27533	530	53,9875297	25,2773884	36,314171	33,7907682	48,5960796	12,1194452
579,162354	530	55,9269271	25,5843033	35,8033334	34,3553336	48,8950628	11,4586129
581,047852	530	52,7386807	26,2324666	37,192452	37,6816372	49,0178339	11,2723681
582,931641	530	54,4475903	25,2235929	35,1737489	36,4386289	51,1154053	10,8059541
584,813965	530	52,0959002	23,8101317	36,3541605	34,3514091	48,4432289	11,6911265
586,69458	530	51,5488031	24,8959254	34,5890144	33,0478763	47,0258236	10,7098377
588,57373	530	55,9769194	25,5740792	36,1024859	30,5490426	45,1494618	11,0796553
590,451111	530	52,2831263	24,854796	35,0817419	29,398154	45,2396244	11,5759899
592,326965	530	52,216111	24,8874418	32,0240849	26,1958936	47,4981713	11,7030067
594,201233	530	48,6259695	23,3255862	32,2991865	24,4009797	46,1289065	11,5610024
596,073914	530	50,8509395	23,0074423	32,3714272	18,9628419	41,9074719	9,60923121
597,944946	530	48,7727786	22,5805224	33,3173089	18,0512699	45,3655014	10,495512
599,814453	530	49,3868852	24,3906728	32,9950327	18,1706683	41,8513348	9,17192424
601,682251	530	51,778607	20,7762161	32,8364526	15,3763242	39,4062056	9,23130101
603,548523	530	50,6988056	21,5383429	32,2818107	15,4241056	41,3402705	10,5043482
605,413147	530	46,0189804	22,3876558	30,6157069	17,2521488	41,5119737	8,63122293
607,276245	530	47,3974743	20,102166	31,6953781	18,83581	39,4367624	9,98732683
609,137695	530	50,134975	21,1944954	31,4045649	16,1540272	39,9372237	9,00662141
610,997437	530	45,9675661	20,4320555	29,1329043	13,6091388	37,6008403	9,42733489
612,855713	530	46,3064422	19,8762049	30,5121345	12,4863012	39,1448928	9,51137378
614,712341	530	42,0708609	20,1718916	28,9799702	11,7122468	36,5853443	8,00141033

This journal is © The Royal Society of Chemistry 20xx

Please do not adjust margins

Please do not adjust margins

616,567322	530	45,026133	18,9819252	28,2029601	12,4525621	33,1480955	7,60545114
618,420715	530	45,6590711	20,0614625	28,5681741	10,28443	35,5449377	7,69766923
620,272522	530	44,2958518	19,5366636	26,276967	10,3874494	35,782796	8,60724077
622,122681	530	39,1065813	18,1976679	28,202919	9,92186112	34,3148808	7,73065072
623,971313	530	39,1320947	17,5143192	30,3175854	10,7645116	33,2672798	8,56779033
625,818237	530	39,69584	17,583473	26,9190027	10,3157864	33,3167735	7,70049749
627,663574	530	39,0276987	15,0746333	26,146676	10,9900414	31,735272	8,69880366
629,507324	530	38,3959775	15,5605362	25,4517765	10,7144949	30,7378801	8,08190162
631,349365	530	35,3734216	16,3135286	25,3881061	11,2997513	29,1936441	7,01497901
633,18988	530	36,1998569	18,4162223	23,6297206	10,9954478	31,2456679	7,36224765
635,028809	530	35,5173957	17,0846444	25,0192967	13,7154255	32,6313486	6,69646108
636,865967	530	35,4085797	16,6158949	24,0667551	14,1510735	30,6975642	7,70079139
638,70166	530	33,7725328	15,3843572	23,3352463	12,4200478	28,3963721	7,1250775
640,535645	530	33,1870056	15,6296208	22,5036213	13,9078986	29,2188871	6,79243076
642,368042	530	34,0556092	16,642012	20,0457361	13,3444675	28,0938849	6,80061762
644,198853	530	30,8905556	16,2630424	24,6546613	13,9547031	28,7358566	6,89840273
646,028015	530	33,5035258	14,0389657	20,777117	12,9729458	28,6028414	6,24071575
647,855591	530	32,8240007	13,7006541	20,9341229	13,1334868	29,1720402	6,98652755
649,681519	530	29,5745052	11,6035791	18,2066703	12,1174369	27,3229286	8,11098561
651,505798	530	27,8504611	13,6629617	20,8811043	10,7407056	25,5054599	7,22071874
653,328491	530	27,1789994	12,3597936	17,3283804	9,17457548	23,3959673	5,55856322
655,149536	530	27,1932463	11,5608214	15,7028304	9,46132033	22,7207537	5,74780161
656,968994	530	24,3376343	10,2528541	18,7347779	8,48252215	20,6095261	6,11106119
658,786743	530	26,2437243	10,5574583	18,8246467	7,50042651	24,6284943	4,94641346
660,602905	530	26,6475844	9,30235035	15,0271192	7,6036444	19,4761616	4,53071552
662,41748	530	24,8798534	10,2013987	14,4942696	6,35053155	18,7914029	5,92497583
664,230469	530	24,4131754	9,87794749	15,7259479	5,84713853	18,8284824	3,44154613
666,041748	530	23,3381821	10,9285966	16,615555	5,16865473	19,5518951	6,11989193
667,85144	530	22,0206498	10,6134848	15,9706094	7,42877047	16,2372721	3,71660218
669,659424	530	20,0752559	8,81934268	14,7055691	5,62619784	18,0012104	3,63755918
671,46582	530	21,2218389	9,17457036	15,586523	6,49840435	16,6334917	4,76834084
673,27063	530	21,5658708	8,16781915	14,6913543	5,04512406	16,8726119	4,61295119
675,073792	530	21,6074001	7,13883965	11,7582966	5,05483944	16,0337064	3,2265636
676,875305	530	18,3802302	8,00791143	11,2335756	4,7924735	14,2882029	3,74907924
678,675171	530	17,5119119	7,57390231	12,5417982	5,14183405	13,2448292	2,96485934
680,47345	530	17,1503153	8,09006058	12,010049	4,61541006	14,8890594	3,13686665
682,27002	530	18,2580559	6,80872493	11,0036964	4,80617382	14,5911656	4,546731
684,065063	530	16,0398557	7,53200777	12,7050734	5,96192925	14,9903146	1,57909967
685,858398	530	17,2745174	8,67436782	11,746387	5,8768288	13,7690704	4,03724589
687,650024	530	14,3951022	5,70094961	12,1967891	3,68764184	10,7979493	2,98701562
689,440186	530	15,0442212	5,62621746	10,3780418	4,48820116	13,2866598	2,28946822
691,228577	530	16,6360032	6,33260833	10,823111	3,25774774	10,2119916	3,08349257
693,015381	530	13,8542505	5,55503597	10,6740336	3,53079022	14,7389018	1,85477123
694,800476	530	15,6152219	6,17072051	9,61305004	3,4416606	12,9705233	2,82561212
696,583984	530	12,8437614	5,66457254	9,65184938	3,80996707	11,7826001	1,50716507
698,365845	530	12,9686776	4,1716094	8,52459748	2,75443844	10,4831093	3,37842363
700,146118	530	12,396205	4,09915861	8,38033951	3,38989374	11,3277313	1,33891461

This journal is © The Royal Society of Chemistry 20xx

Please do not adjust margins

Please do not adjust margins

701,924683	530	13,5181516	3,0414608	8,59154776	2,95517255	11,1026671	2,59851983
347,741211	535	0	0	0	0	0	0
349,808533	535	0	0	0	0	0	0
351,874329	535	0	0	0	0	0	0
353,938568	535	0	0	0	0	0	0
356,001343	535	0	0	0	0	0	0
358,062561	535	0	0	0	0	0	0
360,122253	535	0	0	0	0	0	0
362,180481	535	0	0	0	0	0	0
364,237152	535	0	0	0	0	0	0
366,292328	535	0	0	0	0	0	0
368,345978	535	0	0	0	0	0	0
370,398132	535	0	0	0	0	0	0
372,44873	535	0	0	0	0	0	0
374,497803	535	0	0	0	0	0	0
376,545349	535	0	0	0	0	0	0
378,5914	535	0	0	0	0	0	0
380,635925	535	0	0	0	0	0	0
382,678955	535	0	0	0	0	0	0
384,720398	535	0	0	0	0	0	0
386,760376	535	0	0	0	0	0	0
388,798767	535	0	0	0	0	0	0
390,835663	535	0	0	0	0	0	0
392,871033	535	0	0	0	0	0	0
394,904877	535	0	0	0	0	0	0
396,937195	535	0	0	0	0	0	0
398,967957	535	0	0	0	0	0	0
400,997253	535	0	0	0	0	0	0
403,024963	535	0	0	0	0	0	0
405,051147	535	0	0	0	0	0	0
407,075806	535	0	0	0	0	0	0
409,098938	535	0	0	0	0	0	0
411,120544	535	0	0	0	0	0	0
413,140564	535	0	0	0	0	0	0
415,159119	535	0	0	0	0	0	0
417,176086	535	0	0	0	0	0	0
419,191528	535	0	0	0	0	0	0
421,205444	535	0	0	0	0	0	0
423,217834	535	0	0	0	0	0	0
425,228668	535	0	0	0	0	0	0
427,237976	535	0	0	0	0	0	0
429,245728	535	0	0	0	0	0	0
431,251953	535	0	0	0	0	0	0
433,256592	535	0	0	0	0	0	0
435,259766	535	0	0	0	0	0	0
437,261353	535	0	0	0	0	0	0
439,261383	535	0	0	0	0	0	0

 This journal is © The Royal Society of Chemistry 20xx

Please do not adjust margins

441,259888	535	0	0	0	0	0	0
443,256836	535	0	0	0	0	0	0
445,252258	535	0	0	0	0	0	0
447,246094	535	0	0	0	0	0	0
449,238434	535	0	0	0	0	0	0
451,229187	535	0	0	0	0	0	0
453,218445	535	0	0	0	0	0	0
455,206116	535	0	0	0	0	0	0
457,192261	535	0	0	0	0	0	0
459,176819	535	0	0	0	0	0	0
461,15979	535	0	0	0	0	0	0
463,141296	535	0	0	0	0	0	0
465,121216	535	0	0	0	0	0	0
467,099579	535	0	0	0	0	0	0
469,076385	535	0	0	0	0	0	0
471,051636	535	0	0	0	0	0	0
473,02536	535	0	0	0	0	0	0
474,997498	535	0	0	0	0	0	0
476,968079	535	0	0	0	0	0	0
478,937134	535	0	0	0	0	0	0
480,904572	535	0	0	0	0	0	0
482,870453	535	0	0	0	0	0	0
484,834808	535	0	0	0	0	0	0
486,797607	535	0	0	0	0	0	0
488,75885	535	0	0	0	0	0	0
490,718506	535	0	0	0	0	0	0
492,676605	535	0	0	0	0	0	0
494,633118	535	0	0	0	0	0	0
496,588104	535	0	0	0	0	0	0
498,541504	535	0	0	0	0	0	0
500,493347	535	0	0	0	0	0	0
502,443665	535	0	0	0	0	0	0
504,392365	535	0	0	0	0	0	0
506,339539	535	0	0	0	0	0	0
508,285095	535	0	0	0	0	0	0
510,229126	535	0	0	0	0	0	0
512,17157	535	0	0	0	0	0	0
514,112427	535	0	0	0	0	0	0
516,051697	535	0	0	0	0	0	0
517,989441	535	0	0	0	0	0	0
519,925598	535	0	0	0	0	0	0
521,860168	535	0	0	0	0	0	0
523,793152	535	0	0	0	0	0	0
525,724609	535	0	0	0	0	0	0
527,654419	535	0	0	0	0	0	0
529,582764	535	0	0	0	0	0	0
531,50946	535	0	0	0	0	0	0

533,43457	535	0	0	0	0	0	0
535,358093	535	0	0	0	0	0	0
537,280029	535	0	0	0	0	0	0
539,200439	535	0	0	0	0	0	0
541,119263	535	0	0	0	0	0	0
543,036438	535	0	0	0	0	0	0
544,952087	535	0	0	0	0	0	0
546,866211	535	26,4256923	16,7004447	21,3136479	40,0393667	32,1017139	7,5525131
548,778687	535	32,5910021	13,4200346	21,7682158	32,0283254	34,0808716	6,02615682
550,689514	535	31,5504541	14,464324	23,1011689	23,7543476	30,1440727	7,46350771
552,598816	535	33,4405334	17,312368	25,1637607	18,8329036	31,5847295	6,98846552
554,506531	535	35,5485835	14,8003195	24,2438892	21,2538928	31,874837	7,44106205
556,41272	535	36,7763546	18,526944	24,3295921	21,4144559	32,4193304	8,05178552
558,317261	535	38,2182284	18,7552989	26,9073683	24,3008235	36,6721499	8,96357828
560,220215	535	40,5751853	18,2265719	27,1389232	24,6083637	35,9184093	8,20521335
562,121582	535	38,6816937	20,0899404	28,3334453	25,6477102	35,0021528	9,8967009
564,021362	535	39,5627188	18,2082291	28,8220489	27,5055912	33,9755792	9,98420464
565,919556	535	43,4208362	18,3716478	27,6605794	28,0961562	35,7450536	9,72552291
567,816162	535	42,6298509	22,0347405	29,1989244	30,0992785	34,9300851	10,368888
569,711182	535	43,079995	20,5677082	31,6032899	30,1228023	36,2021781	7,24394033
571,604614	535	45,2828996	22,3625579	31,668196	28,2462289	36,8292428	8,28284974
573,49646	535	43,7884712	21,1089806	31,373951	28,6397154	36,9375013	10,0928561
575,386719	535	44,6154445	21,481739	29,9114122	30,2020245	40,0801914	9,6550474
577,27533	535	46,734416	21,2076208	31,4025231	31,1494014	39,9857974	10,334012
579,162354	535	45,9325436	20,4856812	30,3205239	29,750419	38,7702904	8,24463763
581,047852	535	47,2227155	21,2789647	31,3888538	31,7640412	37,9835866	11,2744441
582,931641	535	46,7506131	22,3823781	32,3329658	31,9217642	39,4021659	10,7225768
584,813965	535	44,3459501	23,3881775	33,7570334	35,7970178	42,1898645	11,7777765
586,69458	535	43,6080301	20,4466234	31,8012198	35,745585	41,9198545	9,35526879
588,57373	535	45,1068753	23,6593689	31,7274695	36,2495048	42,455711	9,80221419
590,451111	535	48,8849443	23,3100311	30,2483743	34,7274245	42,1955217	9,81018181
592,326965	535	47,2302377	21,7780486	29,3635509	31,613876	38,9208114	8,94229977
594,201233	535	45,5078998	22,4568887	30,4339132	27,4591953	37,8619696	11,0689178
596,073914	535	46,0862097	22,1248839	32,0561706	26,0076274	38,4675321	9,08471404
597,944946	535	42,1731876	18,8056495	30,0512255	21,5296997	38,4327631	10,3142077
599,814453	535	41,1691868	21,6410004	28,172899	22,3838334	38,6706655	8,89032651
601,682251	535	40,3344102	19,5377518	30,4829086	17,8977361	40,1391982	9,19788603
603,548523	535	44,9107852	18,4309552	29,4363298	16,4666539	36,2853449	9,01426626
605,413147	535	42,7929662	19,8716715	27,8354559	19,9554684	38,9221912	9,65344894
607,276245	535	42,89097	17,0652806	26,5705522	20,1701011	36,8218743	9,06697663
609,137695	535	38,5899996	18,7504326	28,8886762	15,8987156	34,7260397	8,48182221
610,997437	535	42,0925583	17,5622883	25,847452	16,2569734	36,591723	8,49861886
612,855713	535	41,2102422	17,8955449	27,8960524	17,6521877	35,216021	9,31918715
614,712341	535	40,585002	18,5173722	27,7259508	15,5692787	33,6724675	7,37710216
616,567322	535	41,0381147	16,2405932	24,971945	12,7969146	30,4279581	7,6311856
618,420715	535	41,0164616	16,1002004	25,3366693	8,95458952	32,6082821	8,54635483
620,272522	535	36,9933063	15,3112483	24,5688415	11,4434356	29,3851611	7,90571693

 This journal is © The Royal Society of Chemistry 20xx

Please do not adjust margins

622,122681	535	38,3911314	15,6742084	24,2923688	10,3130234	30,8507568	9,16067269
623,971313	535	39,4875306	16,6270483	23,863425	8,76938104	30,6870551	7,11688458
625,818237	535	33,8379185	17,6062296	21,8022885	9,96679947	28,5661127	7,80963477
627,663574	535	35,0605449	15,0581566	23,7506281	9,73471115	27,7012693	8,48918065
629,507324	535	36,3186158	13,4602439	22,2819989	8,52847145	26,6673548	6,52370068
631,349365	535	32,5855162	13,1355019	22,6324799	8,86945348	26,9379842	5,77521932
633,18988	535	32,6616086	13,1661754	20,0903295	7,79986182	26,9170351	7,38269672
635,028809	535	31,7213811	14,4992655	21,2495359	9,72303533	28,4931864	5,36601948
636,865967	535	32,7779445	14,606334	21,4510116	8,98296552	24,5935829	6,80219957
638,70166	535	29,6925127	12,1786378	20,8759329	10,4158897	24,691089	6,13374818
640,535645	535	29,6870591	13,6879542	22,9676908	13,1854381	24,8544897	4,95650239
642,368042	535	31,1502087	13,3681466	19,8062342	13,1172455	27,5746571	6,81289136
644,198853	535	29,5345336	13,8182762	22,2039266	11,7130176	23,0003726	6,6590072
646,028015	535	29,9057046	14,0029906	20,9665942	10,9673079	22,9426625	6,66671931
647,855591	535	29,9767562	10,2868283	19,442382	9,52892871	25,7991435	5,31415943
649,681519	535	29,3754088	11,6982407	17,7695567	11,8689822	25,9373198	6,95388797
651,505798	535	26,0959373	12,1718778	16,8229661	12,3433637	24,1714159	5,02395719
653,328491	535	26,4321722	11,4409736	17,9827651	12,4668006	25,7826367	6,57690062
655,149536	535	25,9291353	11,7395917	16,8505657	11,2265857	23,4771402	6,25788615
656,968994	535	22,7710238	11,2718448	17,6073258	8,51927966	22,1115421	3,35707221
658,786743	535	24,7797469	10,201969	16,1383612	9,42480697	24,4653047	3,027209
660,602905	535	20,9439619	9,35953451	17,3877099	8,49377095	20,5371911	3,98805094
662,41748	535	22,6897937	9,92388934	14,685191	8,88016854	20,45536	4,00596605
664,230469	535	24,0411497	8,37344952	14,4529698	7,9381361	21,8039604	4,62467709
666,041748	535	21,9906716	7,95181682	13,1709698	6,20479301	17,9992495	5,33245477
667,85144	535	20,913565	10,5147376	16,355907	6,92290318	15,420273	2,80504311
669,659424	535	20,3423587	7,98990727	13,584632	5,53203438	16,5929786	3,24993325
671,46582	535	19,3098913	8,43753144	13,1598598	6,59248893	14,764395	3,69288541
673,27063	535	18,9449733	7,50000384	12,9471962	6,26534346	15,5280682	2,9129221
675,073792	535	19,2463131	6,01155726	10,1482638	4,5975387	15,0275852	3,2722929
676,875305	535	18,1834352	8,48529542	10,6757142	6,27621854	13,9641667	4,33275553
678,675171	535	16,5089454	6,98380072	12,0892998	4,59739761	15,5574931	1,85719033
680,47345	535	17,2902859	5,74177913	10,9337879	3,09204029	12,8074695	3,09295457
682,27002	535	14,8716017	6,91401138	10,5292705	4,96440825	12,6745844	3,01499624
684,065063	535	16,1644516	7,38167127	11,09692	4,3582954	12,9834966	2,58016201
685,858398	535	15,7272642	6,85105832	11,3682271	3,73731931	12,2774515	2,40327282
687,650024	535	13,6082586	5,78910601	9,06805511	4,63175378	12,4677586	2,31656166
689,440186	535	14,709495	5,17760404	10,2474195	5,2674064	11,4254421	3,12565655
691,228577	535	15,5194619	3,84045683	10,3416123	4,19813369	10,5380109	2,23371012
693,015381	535	12,1608103	5,37232005	8,93771414	4,20874351	12,6238534	2,41850373
694,800476	535	14,0350759	6,08710859	8,9354817	3,13338785	10,2934789	1,43282942
696,583984	535	11,3990371	5,66228883	7,2669525	5,12354199	9,88567565	1,43861248
698,365845	535	12,2411428	4,68676764	9,80646337	2,61403894	9,82333105	2,07381631
700,146118	535	11,5670906	5,15793716	8,0390926	4,70596302	9,68160081	1,26736467
701,924683	535	12,9722892	3,9968724	7,70730181	2,99795784	8,71968995	2,81709616
347,741211	540	0	0	0	0	0	0
349,808533	540	0	0	0	0	0	0

This journal is © The Royal Society of Chemistry 20xx

Please do not adjust margins

Please do not adjust margins

351,874329	540	0	0	0	0	0	0
353,938568	540	0	0	0	0	0	0
356,001343	540	0	0	0	0	0	0
358,062561	540	0	0	0	0	0	0
360,122253	540	0	0	0	0	0	0
362,180481	540	0	0	0	0	0	0
364,237152	540	0	0	0	0	0	0
366,292328	540	0	0	0	0	0	0
368,345978	540	0	0	0	0	0	0
370,398132	540	0	0	0	0	0	0
372,44873	540	0	0	0	0	0	0
374,497803	540	0	0	0	0	0	0
376,545349	540	0	0	0	0	0	0
378,5914	540	0	0	0	0	0	0
380,635925	540	0	0	0	0	0	0
382,678955	540	0	0	0	0	0	0
384,720398	540	0	0	0	0	0	0
386,760376	540	0	0	0	0	0	0
388,798767	540	0	0	0	0	0	0
390,835663	540	0	0	0	0	0	0
392,871033	540	0	0	0	0	0	0
394,904877	540	0	0	0	0	0	0
396,937195	540	0	0	0	0	0	0
398,967957	540	0	0	0	0	0	0
400,997253	540	0	0	0	0	0	0
403,024963	540	0	0	0	0	0	0
405,051147	540	0	0	0	0	0	0
407,075806	540	0	0	0	0	0	0
409,098938	540	0	0	0	0	0	0
411,120544	540	0	0	0	0	0	0
413,140564	540	0	0	0	0	0	0
415,159119	540	0	0	0	0	0	0
417,176086	540	0	0	0	0	0	0
419,191528	540	0	0	0	0	0	0
421,205444	540	0	0	0	0	0	0
423,217834	540	0	0	0	0	0	0
425,228668	540	0	0	0	0	0	0
427,237976	540	0	0	0	0	0	0
429,245728	540	0	0	0	0	0	0
431,251953	540	0	0	0	0	0	0
433,256592	540	0	0	0	0	0	0
435,259766	540	0	0	0	0	0	0
437,261353	540	0	0	0	0	0	0
439,261383	540	0	0	0	0	0	0
441,259888	540	0	0	0	0	0	0
443,256836	540	0	0	0	0	0	0
445,252258	540	0	0	0	0	0	0

This journal is © The Royal Society of Chemistry 20xx

Please do not adjust margins

Please do not adjust margins

447,246094	540	0	0	0	0	0	0
449,238434	540	0	0	0	0	0	0
451,229187	540	0	0	0	0	0	0
453,218445	540	0	0	0	0	0	0
455,206116	540	0	0	0	0	0	0
457,192261	540	0	0	0	0	0	0
459,176819	540	0	0	0	0	0	0
461,15979	540	0	0	0	0	0	0
463,141296	540	0	0	0	0	0	0
465,121216	540	0	0	0	0	0	0
467,099579	540	0	0	0	0	0	0
469,076385	540	0	0	0	0	0	0
471,051636	540	0	0	0	0	0	0
473,02536	540	0	0	0	0	0	0
474,997498	540	0	0	0	0	0	0
476,968079	540	0	0	0	0	0	0
478,937134	540	0	0	0	0	0	0
480,904572	540	0	0	0	0	0	0
482,870453	540	0	0	0	0	0	0
484,834808	540	0	0	0	0	0	0
486,797607	540	0	0	0	0	0	0
488,75885	540	0	0	0	0	0	0
490,718506	540	0	0	0	0	0	0
492,676605	540	0	0	0	0	0	0
494,633118	540	0	0	0	0	0	0
496,588104	540	0	0	0	0	0	0
498,541504	540	0	0	0	0	0	0
500,493347	540	0	0	0	0	0	0
502,443665	540	0	0	0	0	0	0
504,392365	540	0	0	0	0	0	0
506,339539	540	0	0	0	0	0	0
508,285095	540	0	0	0	0	0	0
510,229126	540	0	0	0	0	0	0
512,17157	540	0	0	0	0	0	0
514,112427	540	0	0	0	0	0	0
516,051697	540	0	0	0	0	0	0
517,989441	540	0	0	0	0	0	0
519,925598	540	0	0	0	0	0	0
521,860168	540	0	0	0	0	0	0
523,793152	540	0	0	0	0	0	0
525,724609	540	0	0	0	0	0	0
527,654419	540	0	0	0	0	0	0
529,582764	540	0	0	0	0	0	0
531,50946	540	0	0	0	0	0	0
533,43457	540	0	0	0	0	0	0
535,358093	540	0	0	0	0	0	0
537,280029	540	0	0	0	0	0	0

This journal is © The Royal Society of Chemistry 20xx

Please do not adjust margins

539,200439	540	0	0	0	0	0	0
541,119263	540	0	0	0	0	0	0
543,036438	540	0	0	0	0	0	0
544,952087	540	0	0	0	0	0	0
546,866211	540	0	0	0	0	0	0
548,778687	540	0	0	0	0	0	0
550,689514	540	25,949699	15,289526	19,3010872	48,8541214	36,5743911	7,50370138
552,598816	540	27,654422	14,3257994	21,0223615	34,0016355	30,6955999	7,09853495
554,506531	540	27,4886369	13,1648793	19,1660691	29,5171786	29,4490485	6,17000811
556,41272	540	30,889213	14,941699	19,0968997	21,2024596	27,6101276	7,71566264
558,317261	540	30,8131102	14,3259778	21,484659	19,3526458	28,3050891	6,63566965
560,220215	540	31,1260224	14,1741122	23,7984929	19,9725311	27,6152007	6,94537229
562,121582	540	32,6560459	16,3642037	23,3065982	21,9448518	29,6036268	8,96999043
564,021362	540	31,7658249	16,6919798	24,3375908	23,1302638	27,78022	8,74797473
565,919556	540	36,1885594	16,0727988	23,2892417	26,1942736	30,8673113	9,02306586
567,816162	540	33,4142598	14,6409139	26,4729406	28,3770351	32,7526738	8,73782486
569,711182	540	37,5114961	18,1278758	23,8271283	27,8460903	32,7838601	7,35803352
571,604614	540	38,072718	17,8169674	25,189868	27,8927656	34,7390013	7,94153176
573,49646	540	39,4443307	18,6565354	25,0278015	28,5263704	32,3186818	9,30549828
575,386719	540	37,0323869	20,5266461	27,1528876	28,3722901	34,1519516	8,5416816
577,27533	540	39,8542723	18,4953635	27,2216222	27,9708015	32,0331118	8,19066477
579,162354	540	39,0966821	19,0279541	27,2228276	31,0026697	33,4037597	9,49109089
581,047852	540	36,2987016	19,825434	28,21361	28,9690924	33,3806054	9,44991724
582,931641	540	42,5351558	19,185955	25,7359962	30,3296003	34,5013892	9,61011096
584,813965	540	38,7168762	18,5140292	28,366461	31,5309322	36,9176397	9,23340276
586,69458	540	38,9934928	18,3282513	26,3616725	30,9814836	36,4646055	9,01943189
588,57373	540	39,5188296	19,5544842	23,589937	30,4949303	35,6826188	8,09479724
590,451111	540	39,5489631	20,8722425	26,5741682	32,320616	35,9226987	9,4746268
592,326965	540	40,2302285	18,1338969	27,030572	35,4807028	38,380316	9,08314239
594,201233	540	41,5735908	20,2017778	25,6004126	34,2042786	35,8796105	10,8268382
596,073914	540	41,0384353	16,245468	26,0377719	30,6808142	36,559826	9,3116888
597,944946	540	38,0726752	19,1590188	28,4400025	27,8892806	37,1969974	8,38132648
599,814453	540	38,5666636	17,9450362	26,5366398	24,1467684	35,1864219	8,02697826
601,682251	540	39,808337	18,2358511	26,9736591	22,8395626	32,185211	9,17591928
603,548523	540	37,8947178	15,2885903	26,8327431	21,7092801	34,4458648	10,1551052
605,413147	540	38,0793319	16,2580513	25,2115144	20,6760257	36,4639801	7,89425226
607,276245	540	34,7080071	16,3161491	25,3387856	19,483842	30,9348065	8,46304377
609,137695	540	35,4804611	14,6366273	25,8776608	17,7976026	34,6951607	7,70416671
610,997437	540	38,2025257	15,4113215	22,1182686	16,6716387	31,7769962	8,71547799
612,855713	540	36,6413604	15,6655955	24,6124295	17,0096063	34,2785378	7,39009611
614,712341	540	35,6596206	15,8740805	24,4295503	18,3034597	32,4568934	6,99373983
616,567322	540	35,9475215	13,8111257	22,7921075	17,9899534	28,8259893	6,91789646
618,420715	540	34,0003886	15,6645294	24,491747	14,0961822	29,4524441	7,76278266
620,272522	540	38,2482565	14,4453494	20,7871154	13,6246342	28,5953349	7,6119919
622,122681	540	33,6439189	14,4764613	22,1704809	12,7325442	29,6624284	7,12541577
623,971313	540	32,56112	14,2643608	22,1472726	9,32355783	26,2987353	6,55651369
625,818237	540	32,3526778	15,2473148	20,8871605	9,44469878	25,9808073	6,83557082

 This journal is © The Royal Society of Chemistry 20xx

Please do not adjust margins

627,663574	540	32,8315467	13,0800446	21,0082465	9,63025249	25,0124578	7,43935312
629,507324	540	30,0247102	13,314075	19,3312138	9,76257724	26,5792557	7,30608589
631,349365	540	30,1317171	13,4936188	19,6000069	8,32554524	23,9644548	6,37628253
633,18988	540	26,1184181	12,249173	20,156056	7,23799869	26,0647064	6,56160343
635,028809	540	29,9289218	12,2418396	19,2940314	8,51019456	23,2398849	6,13185201
636,865967	540	32,5314822	12,3465118	17,0262576	6,13711224	23,618003	6,56813354
638,70166	540	27,6073188	11,5865439	17,3763319	7,24918572	21,5835391	6,40105035
640,535645	540	28,6245536	10,3067229	19,4170392	8,35634963	24,0531229	4,69324143
642,368042	540	27,3796325	9,29575919	17,6498754	10,8421993	20,7516088	5,0406362
644,198853	540	26,4129561	10,5978068	19,2260366	11,122258	23,0215058	5,99331813
646,028015	540	25,3310346	11,9791233	18,221727	10,7925195	23,0481681	6,51456722
647,855591	540	28,1427197	9,06591864	19,4963688	10,5309211	24,4939664	5,56921885
649,681519	540	25,3695048	11,0061576	16,1620535	10,1569441	21,8698905	6,28816017
651,505798	540	24,3511325	9,66994185	17,6958991	10,1986607	21,0087032	5,1030853
653,328491	540	24,5994293	9,26672455	17,1443332	10,0567062	19,8593528	4,59825786
655,149536	540	23,4717083	9,9088116	15,5554537	11,3983847	22,9777648	5,39862778
656,968994	540	22,8703866	9,86248695	17,8012745	10,4844232	21,3247995	5,68324438
658,786743	540	22,8923832	10,4359802	13,8533977	11,3248196	22,8321476	6,14977604
660,602905	540	20,8366237	9,31795594	13,8862444	9,85571432	22,5341865	4,31505011
662,41748	540	22,7848029	8,4768127	12,4478228	9,01606061	18,5681135	6,01513312
664,230469	540	25,0419485	8,84743674	13,7992345	7,35107503	19,4906929	4,69751647
666,041748	540	20,9211467	9,74994021	14,2675063	7,00957449	17,751146	4,61729462
667,85144	540	18,8452352	9,42122444	13,3292488	7,20678889	14,5959533	3,65056695
669,659424	540	17,9037874	7,30295276	10,3289354	6,95399763	15,339112	3,74721753
671,46582	540	17,7437641	7,22126832	12,7461033	6,60412797	14,1049868	4,19760238
673,27063	540	17,9028043	7,69706765	12,1696669	5,01730123	15,3253392	2,95880108
675,073792	540	19,372262	5,82875397	9,59310233	3,0520847	14,2773177	2,87466553
676,875305	540	16,5888382	7,17249103	9,14309284	6,10301217	13,9155013	4,31118084
678,675171	540	17,21926	7,98066437	10,3100272	5,02680876	12,9300042	2,69491623
680,47345	540	14,6072761	6,27213588	9,67506976	3,22905331	12,4715189	3,59047895
682,27002	540	14,6578948	6,11404587	10,4277522	5,22039145	11,6143958	2,79226382
684,065063	540	14,7968396	6,31479042	9,65067322	3,97346026	13,6403332	2,62079997
685,858398	540	14,8033257	4,33204012	8,6623434	4,51727503	13,9174313	1,53700447
687,650024	540	11,7460003	4,51702447	9,84513936	4,25045667	11,669689	3,52957181
689,440186	540	12,5882149	5,07076122	10,5016141	3,80705999	10,6086013	2,5399089
691,228577	540	11,8695495	4,07672332	10,3256288	2,90204404	9,88808872	1,81511312
693,015381	540	11,5362021	5,54019122	6,17471282	4,54590359	11,8229378	3,45743076
694,800476	540	13,077151	5,90200789	7,26255355	3,72670986	12,6382913	1,18250982
696,583984	540	11,4886903	3,73783063	7,38301434	4,28932463	10,3157626	1,00462371
698,365845	540	11,2466634	4,753984	8,50061314	1,83037327	7,87299464	4,02978638
700,146118	540	11,383384	2,84542523	8,25925595	4,31855682	9,74268097	1,10342217
701,924683	540	12,3486907	4,14633524	8,84374199	2,21369833	9,5956052	1,75380166
347,741211	545	0	0	0	0	0	0
349,808533	545	0	0	0	0	0	0
351,874329	545	0	0	0	0	0	0
353,938568	545	0	0	0	0	0	0
356,001343	545	0	0	0	0	0	0

Please do not adjust margins

Please do not adjust margins

358,062561	545	0	0	0	0	0	0
360,122253	545	0	0	0	0	0	0
362,180481	545	0	0	0	0	0	0
364,237152	545	0	0	0	0	0	0
366,292328	545	0	0	0	0	0	0
368,345978	545	0	0	0	0	0	0
370,398132	545	0	0	0	0	0	0
372,44873	545	0	0	0	0	0	0
374,497803	545	0	0	0	0	0	0
376,545349	545	0	0	0	0	0	0
378,5914	545	0	0	0	0	0	0
380,635925	545	0	0	0	0	0	0
382,678955	545	0	0	0	0	0	0
384,720398	545	0	0	0	0	0	0
386,760376	545	0	0	0	0	0	0
388,798767	545	0	0	0	0	0	0
390,835663	545	0	0	0	0	0	0
392,871033	545	0	0	0	0	0	0
394,904877	545	0	0	0	0	0	0
396,937195	545	0	0	0	0	0	0
398,967957	545	0	0	0	0	0	0
400,997253	545	0	0	0	0	0	0
403,024963	545	0	0	0	0	0	0
405,051147	545	0	0	0	0	0	0
407,075806	545	0	0	0	0	0	0
409,098938	545	0	0	0	0	0	0
411,120544	545	0	0	0	0	0	0
413,140564	545	0	0	0	0	0	0
415,159119	545	0	0	0	0	0	0
417,176086	545	0	0	0	0	0	0
419,191528	545	0	0	0	0	0	0
421,205444	545	0	0	0	0	0	0
423,217834	545	0	0	0	0	0	0
425,228668	545	0	0	0	0	0	0
427,237976	545	0	0	0	0	0	0
429,245728	545	0	0	0	0	0	0
431,251953	545	0	0	0	0	0	0
433,256592	545	0	0	0	0	0	0
435,259766	545	0	0	0	0	0	0
437,261353	545	0	0	0	0	0	0
439,261383	545	0	0	0	0	0	0
441,259888	545	0	0	0	0	0	0
443,256836	545	0	0	0	0	0	0
445,252258	545	0	0	0	0	0	0
447,246094	545	0	0	0	0	0	0
449,238434	545	0	0	0	0	0	0
451,229187	545	0	0	0	0	0	0

This journal is © The Royal Society of Chemistry 20xx

Please do not adjust margins

Please do not adjust margins

453,218445	545	0	0	0	0	0	0
455,206116	545	0	0	0	0	0	0
457,192261	545	0	0	0	0	0	0
459,176819	545	0	0	0	0	0	0
461,15979	545	0	0	0	0	0	0
463,141296	545	0	0	0	0	0	0
465,121216	545	0	0	0	0	0	0
467,099579	545	0	0	0	0	0	0
469,076385	545	0	0	0	0	0	0
471,051636	545	0	0	0	0	0	0
473,02536	545	0	0	0	0	0	0
474,997498	545	0	0	0	0	0	0
476,968079	545	0	0	0	0	0	0
478,937134	545	0	0	0	0	0	0
480,904572	545	0	0	0	0	0	0
482,870453	545	0	0	0	0	0	0
484,834808	545	0	0	0	0	0	0
486,797607	545	0	0	0	0	0	0
488,75885	545	0	0	0	0	0	0
490,718506	545	0	0	0	0	0	0
492,676605	545	0	0	0	0	0	0
494,633118	545	0	0	0	0	0	0
496,588104	545	0	0	0	0	0	0
498,541504	545	0	0	0	0	0	0
500,493347	545	0	0	0	0	0	0
502,443665	545	0	0	0	0	0	0
504,392365	545	0	0	0	0	0	0
506,339539	545	0	0	0	0	0	0
508,285095	545	0	0	0	0	0	0
510,229126	545	0	0	0	0	0	0
512,17157	545	0	0	0	0	0	0
514,112427	545	0	0	0	0	0	0
516,051697	545	0	0	0	0	0	0
517,989441	545	0	0	0	0	0	0
519,925598	545	0	0	0	0	0	0
521,860168	545	0	0	0	0	0	0
523,793152	545	0	0	0	0	0	0
525,724609	545	0	0	0	0	0	0
527,654419	545	0	0	0	0	0	0
529,582764	545	0	0	0	0	0	0
531,50946	545	0	0	0	0	0	0
533,43457	545	0	0	0	0	0	0
535,358093	545	0	0	0	0	0	0
537,280029	545	0	0	0	0	0	0
539,200439	545	0	0	0	0	0	0
541,119263	545	0	0	0	0	0	0
543,036438	545	0	0	0	0	0	0

This journal is © The Royal Society of Chemistry 20xx

Please do not adjust margins

Please do not adjust margins

544,952087	545	0	0	0	0	0	0
546,866211	545	0	0	0	0	0	0
548,778687	545	0	0	0	0	0	0
550,689514	545	0	0	0	0	0	0
552,598816	545	0	0	0	0	0	0
554,506531	545	0	0	0	0	0	0
556,41272	545	24,1758159	14,2912442	17,8766698	43,1979996	29,9717715	7,3216377
558,317261	545	25,6535673	15,3014124	17,0999381	30,8232845	29,6612695	7,71131868
560,220215	545	24,6467581	11,8079796	19,2792553	22,8096207	26,4734407	6,38813409
562,121582	545	25,0084366	12,4546911	18,6952288	19,1896264	24,6546888	6,08885657
564,021362	545	28,1568325	14,6509543	18,3266249	18,2743676	24,2141497	5,93949908
565,919556	545	30,0936511	10,8057325	20,1379294	19,6955123	24,0157424	5,96783601
567,816162	545	27,0390404	15,3143621	21,2194747	22,5873162	25,1104473	8,820851
569,711182	545	27,8391604	15,8383142	19,9498659	21,1624588	24,5625103	5,68949284
571,604614	545	31,3708011	15,1971839	23,445536	23,71655	27,529025	8,49324851
573,49646	545	30,7479461	15,0037016	22,8789277	24,5817914	27,928216	7,72211937
575,386719	545	31,0764749	16,1619831	23,0251791	27,6053428	28,0892885	8,22363826
577,27533	545	34,2904524	12,9680874	22,1894863	27,8361279	28,1622595	6,58268282
579,162354	545	31,9012131	13,8897446	23,7818937	27,3555538	29,0505812	7,56840171
581,047852	545	31,2251463	14,4303882	24,1214126	26,7406164	25,7759769	7,76111969
582,931641	545	34,8560703	17,5744862	23,7987816	28,598802	29,4121649	6,14423615
584,813965	545	32,5190247	16,5690187	23,3747694	28,5104469	30,7840665	8,267444
586,69458	545	34,563545	15,8079211	24,0936813	30,7891566	30,7949018	7,07273789
588,57373	545	32,5406625	17,1121373	24,3816288	28,9072421	31,6078818	6,0470679
590,451111	545	34,3152919	17,3486452	23,3672721	31,1453593	31,4808129	6,92612874
592,326965	545	35,9397212	14,297196	22,7086667	30,9873045	30,8264571	8,91937381
594,201233	545	36,2484599	17,6058329	21,4775041	33,857958	33,0239711	7,90302393
596,073914	545	32,7087509	15,1160212	23,9998964	32,8348187	35,2109107	9,06142296
597,944946	545	34,9591302	15,3554707	25,413519	34,1579301	35,7710409	10,1531582
599,814453	545	34,1179868	18,8541891	25,353207	32,7272868	33,6614021	8,69411197
601,682251	545	37,3993824	14,4432302	22,4364278	26,5072623	28,9530149	6,56838579
603,548523	545	35,8935062	14,7474268	22,8914232	23,0916364	30,6546182	7,05847744
605,413147	545	34,241929	14,7224148	22,9363079	23,2201268	27,5004431	7,63371492
607,276245	545	34,2734475	14,1988877	21,9820259	23,0174141	29,1329264	7,37082734
609,137695	545	32,8006759	15,5108163	21,9474478	19,05351	31,1766902	6,10481818
610,997437	545	33,3673969	15,7086458	20,9039074	16,2443023	31,6571755	8,37932552
612,855713	545	31,3717588	13,7082784	21,6643367	17,0040957	29,4066565	7,71178757
614,712341	545	33,0501875	14,6672003	21,5550414	16,462041	30,0740326	6,47184432
616,567322	545	33,7574426	13,1726941	19,0593188	18,4135491	26,2378964	6,64706931
618,420715	545	31,2000944	13,7802569	19,1714463	15,7461182	29,561063	6,74111314
620,272522	545	30,9293298	14,1463759	21,403076	17,5516392	25,741581	5,99545882
622,122681	545	30,2358256	12,3203523	19,1691429	15,3061964	28,3344342	7,10849597
623,971313	545	29,4716963	13,3694778	19,8990244	13,228189	27,5638694	5,85510396
625,818237	545	28,1426689	12,8276087	18,2773732	11,3224088	26,3103545	6,90052189
627,663574	545	30,491799	12,1693541	20,2681921	8,6133342	23,8830059	5,8885551
629,507324	545	26,704745	10,4333828	18,3141028	9,16996171	22,9720403	6,26060542
631,349365	545	25,8678749	11,8103934	19,7835316	7,97602781	21,1875965	4,72037095

 This journal is © The Royal Society of Chemistry 20xx

Please do not adjust margins

Please do not adjust margins

633,18988	545	25,7565701	11,4948429	18,0270266	7,65079708	21,7529533	5,76369787
635,028809	545	25,8269537	10,6306505	19,3888995	9,36446755	21,9117872	4,90050882
636,865967	545	27,7588385	11,4204148	16,9278977	7,22816333	22,0327987	5,51110104
638,70166	545	23,8186569	9,96613937	16,5073272	5,85458769	21,8726875	4,30791509
640,535645	545	23,4703936	11,2527971	17,2779342	7,23080941	21,2659903	4,13483891
642,368042	545	25,3062732	11,3523628	15,6635531	7,67044874	17,8435861	3,70859107
644,198853	545	25,8784384	9,22214732	19,4923088	6,13232357	18,5731772	4,92664531
646,028015	545	24,2675435	9,57798038	15,8018749	8,82002003	20,1514523	5,10541605
647,855591	545	20,8039351	9,57376789	14,413948	7,43321684	19,9696917	4,15173204
649,681519	545	22,0435721	9,88517982	15,5324269	11,3833333	20,337382	4,34788341
651,505798	545	22,2212516	10,186985	12,8952	12,3026127	18,6755346	4,88962996
653,328491	545	20,0170675	9,69436614	12,4105591	11,7278843	19,6960176	4,81711808
655,149536	545	22,2829626	7,62587945	12,1924799	10,1893647	16,8683024	4,92251302
656,968994	545	19,7323365	8,18549429	14,1805933	9,43765668	19,5845863	3,44234768
658,786743	545	21,4202308	7,95944446	15,3106248	10,3691831	19,4126054	3,81361517
660,602905	545	18,7205989	7,97831648	13,5727155	9,2389055	22,9238692	5,6895365
662,41748	545	20,230644	8,81557231	13,0990324	9,54811257	21,8665802	6,34018353
664,230469	545	20,0919681	8,83296733	12,76774	8,40461296	22,4462924	4,65267735
666,041748	545	18,6972635	8,22339322	12,5226958	8,24080226	21,3225751	5,91609759
667,85144	545	20,1839657	9,05267276	13,0055887	7,81435498	15,0027677	4,13467562
669,659424	545	18,1576025	7,99334594	10,605403	6,6602228	15,7534659	3,51262164
671,46582	545	17,2763577	7,64190004	13,7651333	8,28874329	13,6970008	3,42605387
673,27063	545	17,2537188	8,12312853	11,2900897	6,24091607	13,8411352	3,43947831
675,073792	545	14,8432401	5,15455511	10,2258952	4,9842229	12,5082019	3,17404105
676,875305	545	15,2023877	4,70150967	8,68583925	4,89267294	10,7840133	4,80549382
678,675171	545	14,4807654	6,87252923	10,4065652	5,61840611	10,6044544	1,81368208
680,47345	545	15,3740693	6,23477051	11,1220043	3,80311193	10,1435242	2,62783167
682,27002	545	12,160378	4,80562206	10,888337	3,81629087	10,9966038	2,8187957
684,065063	545	13,5665577	4,73062157	8,83065999	5,37879895	13,403915	2,18954699
685,858398	545	14,210139	4,27762839	8,65237563	4,28668418	12,0414446	2,00796454
687,650024	545	10,5770102	5,55733065	10,0284925	5,38650229	9,40530951	1,46178852
689,440186	545	13,343215	4,65703254	8,68099021	4,57538386	9,88467324	1,73988627
691,228577	545	12,2525369	4,20252707	9,05095586	5,49316157	8,05814031	1,0077981
693,015381	545	11,8251627	4,94586938	6,8741137	4,31385135	9,18011979	1,92884061
694,800476	545	12,5553693	5,21933591	6,8723967	4,49629614	9,45316161	1,46922577
696,583984	545	10,3056712	3,40166428	8,28016134	3,86952316	9,85991039	0,73757787
698,365845	545	8,9505269	3,96439382	9,04151018	2,58693074	8,03946323	3,14351412
700,146118	545	10,5611894	3,05459325	5,55772767	3,98865376	9,64877661	0,2784767
701,924683	545	11,4387747	4,27428714	5,85804869	4,00398787	9,0339371	2,79547286
347,741211	550	0	0	0	0	0	0
349,808533	550	0	0	0	0	0	0
351,874329	550	0	0	0	0	0	0
353,938568	550	0	0	0	0	0	0
356,001343	550	0	0	0	0	0	0
358,062561	550	0	0	0	0	0	0
360,122253	550	0	0	0	0	0	0
362,180481	550	0	0	0	0	0	0

Please do not adjust margins

Please do not adjust margins

364,237152	550	0	0	0	0	0	0
366,292328	550	0	0	0	0	0	0
368,345978	550	0	0	0	0	0	0
370,398132	550	0	0	0	0	0	0
372,44873	550	0	0	0	0	0	0
374,497803	550	0	0	0	0	0	0
376,545349	550	0	0	0	0	0	0
378,5914	550	0	0	0	0	0	0
380,635925	550	0	0	0	0	0	0
382,678955	550	0	0	0	0	0	0
384,720398	550	0	0	0	0	0	0
386,760376	550	0	0	0	0	0	0
388,798767	550	0	0	0	0	0	0
390,835663	550	0	0	0	0	0	0
392,871033	550	0	0	0	0	0	0
394,904877	550	0	0	0	0	0	0
396,937195	550	0	0	0	0	0	0
398,967957	550	0	0	0	0	0	0
400,997253	550	0	0	0	0	0	0
403,024963	550	0	0	0	0	0	0
405,051147	550	0	0	0	0	0	0
407,075806	550	0	0	0	0	0	0
409,098938	550	0	0	0	0	0	0
411,120544	550	0	0	0	0	0	0
413,140564	550	0	0	0	0	0	0
415,159119	550	0	0	0	0	0	0
417,176086	550	0	0	0	0	0	0
419,191528	550	0	0	0	0	0	0
421,205444	550	0	0	0	0	0	0
423,217834	550	0	0	0	0	0	0
425,228668	550	0	0	0	0	0	0
427,237976	550	0	0	0	0	0	0
429,245728	550	0	0	0	0	0	0
431,251953	550	0	0	0	0	0	0
433,256592	550	0	0	0	0	0	0
435,259766	550	0	0	0	0	0	0
437,261353	550	0	0	0	0	0	0
439,261383	550	0	0	0	0	0	0
441,259888	550	0	0	0	0	0	0
443,256836	550	0	0	0	0	0	0
445,252258	550	0	0	0	0	0	0
447,246094	550	0	0	0	0	0	0
449,238434	550	0	0	0	0	0	0
451,229187	550	0	0	0	0	0	0
453,218445	550	0	0	0	0	0	0
455,206116	550	0	0	0	0	0	0
457,192261	550	0	0	0	0	0	0

This journal is © The Royal Society of Chemistry 20xx

Please do not adjust margins

Please do not adjust margins

459,176819	550	0	0	0	0	0	0
461,15979	550	0	0	0	0	0	0
463,141296	550	0	0	0	0	0	0
465,121216	550	0	0	0	0	0	0
467,099579	550	0	0	0	0	0	0
469,076385	550	0	0	0	0	0	0
471,051636	550	0	0	0	0	0	0
473,02536	550	0	0	0	0	0	0
474,997498	550	0	0	0	0	0	0
476,968079	550	0	0	0	0	0	0
478,937134	550	0	0	0	0	0	0
480,904572	550	0	0	0	0	0	0
482,870453	550	0	0	0	0	0	0
484,834808	550	0	0	0	0	0	0
486,797607	550	0	0	0	0	0	0
488,75885	550	0	0	0	0	0	0
490,718506	550	0	0	0	0	0	0
492,676605	550	0	0	0	0	0	0
494,633118	550	0	0	0	0	0	0
496,588104	550	0	0	0	0	0	0
498,541504	550	0	0	0	0	0	0
500,493347	550	0	0	0	0	0	0
502,443665	550	0	0	0	0	0	0
504,392365	550	0	0	0	0	0	0
506,339539	550	0	0	0	0	0	0
508,285095	550	0	0	0	0	0	0
510,229126	550	0	0	0	0	0	0
512,17157	550	0	0	0	0	0	0
514,112427	550	0	0	0	0	0	0
516,051697	550	0	0	0	0	0	0
517,989441	550	0	0	0	0	0	0
519,925598	550	0	0	0	0	0	0
521,860168	550	0	0	0	0	0	0
523,793152	550	0	0	0	0	0	0
525,724609	550	0	0	0	0	0	0
527,654419	550	0	0	0	0	0	0
529,582764	550	0	0	0	0	0	0
531,50946	550	0	0	0	0	0	0
533,43457	550	0	0	0	0	0	0
535,358093	550	0	0	0	0	0	0
537,280029	550	0	0	0	0	0	0
539,200439	550	0	0	0	0	0	0
541,119263	550	0	0	0	0	0	0
543,036438	550	0	0	0	0	0	0
544,952087	550	0	0	0	0	0	0
546,866211	550	0	0	0	0	0	0
548,778687	550	0	0	0	0	0	0

This journal is © The Royal Society of Chemistry 20xx

Please do not adjust margins

Please do not adjust margins

550,689514	550	0	0	0	0	0	0
552,598816	550	0	0	0	0	0	0
554,506531	550	0	0	0	0	0	0
556,41272	550	0	0	0	0	0	0
558,317261	550	0	0	0	0	0	0
560,220215	550	20,8036876	11,5166584	17,8272086	51,992714	31,9763885	7,98046362
562,121582	550	20,214236	10,7496298	16,5987419	37,1565944	26,6615417	6,25481991
564,021362	550	21,0278122	9,4166547	15,114171	25,0895223	22,573313	6,02415859
565,919556	550	21,763567	10,3361808	15,9813728	20,1196635	20,2139517	5,65468237
567,816162	550	21,7486015	11,2441086	16,3478657	18,0259352	23,1475312	6,63005503
569,711182	550	24,8586577	12,4018426	17,4426429	17,1258342	21,3828967	2,9654415
571,604614	550	26,4959942	12,1361367	20,5752327	18,6498328	22,1226407	5,87705143
573,49646	550	25,5270286	13,2197147	17,7338158	18,5434064	23,397906	7,42846671
575,386719	550	26,2474043	13,8240833	19,563965	23,3679987	23,870582	7,93595921
577,27533	550	27,4461861	12,9240806	20,4835089	24,9588376	26,4415345	7,16503518
579,162354	550	26,9575576	12,1471962	20,2182117	25,6037084	26,1936229	7,18630377
581,047852	550	27,4769581	11,0509693	21,856426	28,2467929	25,1539238	7,46175728
582,931641	550	29,4396765	16,1262724	18,7458236	28,2468683	24,5804472	5,73982861
584,813965	550	27,5477792	12,8029234	20,6817064	26,5957966	26,9438189	6,905529
586,69458	550	26,5611613	14,816795	21,7313634	25,9343739	27,0244194	6,43145879
588,57373	550	30,0878209	13,9006742	22,092208	27,2273846	26,9160114	6,38190637
590,451111	550	30,0254829	14,3599583	20,6216287	27,4728728	27,7436486	5,18500932
592,326965	550	30,1019777	12,829438	18,3157648	29,2938845	28,048064	6,84829065
594,201233	550	31,551178	13,3652951	18,7284942	28,7792218	25,5623582	8,6125825
596,073914	550	29,81042	14,4914815	21,3514173	27,702059	27,1215137	8,5893283
597,944946	550	31,152831	13,5360033	21,8461457	30,5047036	27,2684998	6,52198925
599,814453	550	29,1151217	12,8201386	20,9396214	29,4919849	29,8557269	8,81803476
601,682251	550	30,750167	14,4100077	21,6577085	33,9425155	31,4069734	8,1139856
603,548523	550	29,5792539	14,5492737	20,5902608	31,1465346	30,0621937	9,03408532
605,413147	550	30,5473311	14,8643544	20,3854604	25,8264689	28,7359436	6,97677914
607,276245	550	28,1285844	11,364539	20,6088888	20,527962	25,8060401	7,64579506
609,137695	550	28,7610686	13,7176067	20,6611475	18,8865068	26,6129942	7,71858772
610,997437	550	29,5808545	11,0297552	18,8354835	18,7541868	27,76969	7,64888531
612,855713	550	29,5916297	13,6649033	20,4549777	21,4775458	27,1001772	6,20635579
614,712341	550	29,2463724	12,4252075	19,7442235	16,7687413	26,491829	7,67231497
616,567322	550	29,1926461	12,3533452	19,0838446	18,7466081	25,9233377	6,31511718
618,420715	550	27,616133	13,7366541	17,1495379	14,7633405	29,0358647	6,92266356
620,272522	550	27,3331875	11,371799	19,0670575	16,5051048	23,7915693	6,50914949
622,122681	550	27,3920566	13,4527647	19,9649898	16,6263557	27,1876653	5,5790258
623,971313	550	26,0936799	10,9978792	16,754631	14,6953026	27,5200815	6,54102321
625,818237	550	26,1105352	13,0252877	18,2872127	16,1337539	24,7795282	4,75232557
627,663574	550	25,4658335	12,1840654	15,2949357	14,4335455	22,8337792	6,75150871
629,507324	550	24,7237623	8,94425464	16,1518084	12,593846	23,5546677	4,08825436
631,349365	550	23,7002165	9,47225529	18,5969597	9,64791172	24,5288261	6,00632887
633,18988	550	23,3204768	7,57807876	15,853039	8,01514041	20,4884208	6,19485764
635,028809	550	21,7411521	8,35701169	16,1047054	8,35861756	21,7831431	4,88315965
636,865967	550	23,256582	9,41663229	17,6126419	6,1045456	18,9379232	4,80360918

This journal is © The Royal Society of Chemistry 20xx

Please do not adjust margins

638,70166	550	22,3978576	8,72389817	14,5689529	6,63143666	17,3765848	5,76750054
640,535645	550	21,6966623	8,63507291	14,6535002	6,8046981	18,3337316	4,63062704
642,368042	550	22,5952153	9,43143348	14,3218486	7,2496241	15,5587504	4,72368393
644,198853	550	21,5073932	8,83916564	15,3154311	6,38993153	17,344263	4,90920362
646,028015	550	20,3944161	7,53513498	11,477972	6,57260138	18,9429275	4,47605981
647,855591	550	21,1728671	7,79455889	14,1878906	6,65730683	16,3924513	4,73727303
649,681519	550	20,4066679	6,77972484	13,2072951	6,86909292	17,6259323	4,76264513
651,505798	550	17,9291719	7,24962056	12,2890271	8,31213758	17,344359	3,54237501
653,328491	550	17,8958445	7,98142743	12,3269076	8,60385811	16,6877071	3,99745602
655,149536	550	21,784177	8,81145237	10,9475943	8,90216724	14,8774855	3,92281747
656,968994	550	18,2136304	7,32853953	12,3334321	9,83285676	15,4757051	4,8342651
658,786743	550	18,9289748	8,80051208	13,5600126	11,1374816	16,538665	3,41820877
660,602905	550	17,4451565	7,47116733	11,2517912	10,0835125	14,9561296	4,23780871
662,41748	550	17,0718864	7,86640298	12,2065233	9,04357998	17,6478706	3,89456103
664,230469	550	16,8340558	5,97939144	11,868221	7,33975417	16,1411254	3,99299574
666,041748	550	17,2274588	7,53325977	11,526829	9,07796066	18,9868053	4,63669092
667,85144	550	18,1840172	8,37307335	13,5607783	10,4683529	17,7636595	3,92011353
669,659424	550	14,7590365	6,93096675	11,1260583	8,02687206	17,525976	3,92809334
671,46582	550	17,2364816	8,94643732	11,0461439	10,5916954	15,3509087	4,9379982
673,27063	550	14,0080162	7,33183062	10,7228339	7,88323246	12,7508165	4,49833336
675,073792	550	14,8605792	5,41763776	10,8353074	5,78604686	13,3268256	3,58720073
676,875305	550	13,9406161	5,1411898	8,26265081	7,34596818	11,5783565	4,69006549
678,675171	550	13,02554	6,06022236	11,4777277	5,60219091	12,0397281	1,83953366
680,47345	550	13,4738934	5,41333637	11,5607185	5,04730021	12,2142106	3,49244615
682,27002	550	12,5088401	5,155887	8,47041074	5,15687775	12,348691	3,32009862
684,065063	550	10,981615	5,81965225	9,14519476	5,17401826	12,3897357	3,05353952
685,858398	550	13,9407626	5,63737144	8,31745778	4,52925051	11,0076481	2,22172948
687,650024	550	10,5352693	3,97783238	7,21562417	5,55153035	8,98153397	2,96524868
689,440186	550	11,2080189	5,65601499	7,88135559	4,54422935	8,72387292	1,57892957
691,228577	550	11,491554	4,36005699	9,83334899	4,91760479	9,00672292	1,2080107
693,015381	550	11,2418722	4,09207118	6,23112675	2,97662364	10,4257975	1,86317488
694,800476	550	9,66002213	3,53317875	8,36807959	4,46382023	8,65497309	0,93135475
696,583984	550	9,88553049	2,80060978	5,97465179	4,2950935	9,53086411	1,12213656
698,365845	550	9,35217715	3,65102295	8,4254624	2,7153849	7,49619203	2,34435343
700,146118	550	10,6101685	2,9136861	5,54541891	3,7603174	9,59581794	1,60052739
701,924683	550	11,5935213	3,30229242	7,07636175	3,68040435	8,02725703	2,64629731
347,741211	555	0	0	0	0	0	0
349,808533	555	0	0	0	0	0	0
351,874329	555	0	0	0	0	0	0
353,938568	555	0	0	0	0	0	0
356,001343	555	0	0	0	0	0	0
358,062561	555	0	0	0	0	0	0
360,122253	555	0	0	0	0	0	0
362,180481	555	0	0	0	0	0	0
364,237152	555	0	0	0	0	0	0
366,292328	555	0	0	0	0	0	0
368,345978	555	0	0	0	0	0	0

Please do not adjust margins

370,398132	555	0	0	0	0	0	0
372,44873	555	0	0	0	0	0	0
374,497803	555	0	0	0	0	0	0
376,545349	555	0	0	0	0	0	0
378,5914	555	0	0	0	0	0	0
380,635925	555	0	0	0	0	0	0
382,678955	555	0	0	0	0	0	0
384,720398	555	0	0	0	0	0	0
386,760376	555	0	0	0	0	0	0
388,798767	555	0	0	0	0	0	0
390,835663	555	0	0	0	0	0	0
392,871033	555	0	0	0	0	0	0
394,904877	555	0	0	0	0	0	0
396,937195	555	0	0	0	0	0	0
398,967957	555	0	0	0	0	0	0
400,997253	555	0	0	0	0	0	0
403,024963	555	0	0	0	0	0	0
405,051147	555	0	0	0	0	0	0
407,075806	555	0	0	0	0	0	0
409,098938	555	0	0	0	0	0	0
411,120544	555	0	0	0	0	0	0
413,140564	555	0	0	0	0	0	0
415,159119	555	0	0	0	0	0	0
417,176086	555	0	0	0	0	0	0
419,191528	555	0	0	0	0	0	0
421,205444	555	0	0	0	0	0	0
423,217834	555	0	0	0	0	0	0
425,228668	555	0	0	0	0	0	0
427,237976	555	0	0	0	0	0	0
429,245728	555	0	0	0	0	0	0
431,251953	555	0	0	0	0	0	0
433,256592	555	0	0	0	0	0	0
435,259766	555	0	0	0	0	0	0
437,261353	555	0	0	0	0	0	0
439,261383	555	0	0	0	0	0	0
441,259888	555	0	0	0	0	0	0
443,256836	555	0	0	0	0	0	0
445,252258	555	0	0	0	0	0	0
447,246094	555	0	0	0	0	0	0
449,238434	555	0	0	0	0	0	0
451,229187	555	0	0	0	0	0	0
453,218445	555	0	0	0	0	0	0
455,206116	555	0	0	0	0	0	0
457,192261	555	0	0	0	0	0	0
459,176819	555	0	0	0	0	0	0
461,15979	555	0	0	0	0	0	0
463,141296	555	0	0	0	0	0	0

This journal is © The Royal Society of Chemistry 20xx

Please do not adjust margins

465,121216	555	0	0	0	0	0	0
467,099579	555	0	0	0	0	0	0
469,076385	555	0	0	0	0	0	0
471,051636	555	0	0	0	0	0	0
473,02536	555	0	0	0	0	0	0
474,997498	555	0	0	0	0	0	0
476,968079	555	0	0	0	0	0	0
478,937134	555	0	0	0	0	0	0
480,904572	555	0	0	0	0	0	0
482,870453	555	0	0	0	0	0	0
484,834808	555	0	0	0	0	0	0
486,797607	555	0	0	0	0	0	0
488,75885	555	0	0	0	0	0	0
490,718506	555	0	0	0	0	0	0
492,676605	555	0	0	0	0	0	0
494,633118	555	0	0	0	0	0	0
496,588104	555	0	0	0	0	0	0
498,541504	555	0	0	0	0	0	0
500,493347	555	0	0	0	0	0	0
502,443665	555	0	0	0	0	0	0
504,392365	555	0	0	0	0	0	0
506,339539	555	0	0	0	0	0	0
508,285095	555	0	0	0	0	0	0
510,229126	555	0	0	0	0	0	0
512,17157	555	0	0	0	0	0	0
514,112427	555	0	0	0	0	0	0
516,051697	555	0	0	0	0	0	0
517,989441	555	0	0	0	0	0	0
519,925598	555	0	0	0	0	0	0
521,860168	555	0	0	0	0	0	0
523,793152	555	0	0	0	0	0	0
525,724609	555	0	0	0	0	0	0
527,654419	555	0	0	0	0	0	0
529,582764	555	0	0	0	0	0	0
531,50946	555	0	0	0	0	0	0
533,43457	555	0	0	0	0	0	0
535,358093	555	0	0	0	0	0	0
537,280029	555	0	0	0	0	0	0
539,200439	555	0	0	0	0	0	0
541,119263	555	0	0	0	0	0	0
543,036438	555	0	0	0	0	0	0
544,952087	555	0	0	0	0	0	0
546,866211	555	0	0	0	0	0	0
548,778687	555	0	0	0	0	0	0
550,689514	555	0	0	0	0	0	0
552,598816	555	0	0	0	0	0	0
554,506531	555	0	0	0	0	0	0

Please do not adjust margins

556,41272	555	0	0	0	0	0	0
558,317261	555	0	0	0	0	0	0
560,220215	555	0	0	0	0	0	0
562,121582	555	0	0	0	0	0	0
564,021362	555	0	0	0	0	0	0
565,919556	555	17,4191257	11,0115287	14,9331866	45,6383565	27,3301663	7,15957309
567,816162	555	19,725221	11,5279547	15,9438762	31,7517055	23,7028115	6,77743843
569,711182	555	19,7103445	9,4634759	14,4620664	23,4486276	19,737446	5,82864208
571,604614	555	20,2858585	10,4810194	15,5821558	17,5437398	22,4263815	5,85485821
573,49646	555	21,9731272	9,12654835	14,9767941	15,0700564	21,4328831	5,38272936
575,386719	555	21,6289769	10,4606941	15,0203778	17,5648161	21,0048685	5,15301201
577,27533	555	23,7985449	8,79218089	16,0051574	18,153473	21,2016105	4,19423814
579,162354	555	21,2353468	9,4775901	15,4764173	22,4914441	22,3360144	4,20668826
581,047852	555	24,380269	11,3369825	18,0196313	23,9899597	22,9241365	6,46013484
582,931641	555	24,0497533	12,2472838	18,2676567	25,3136112	21,765574	5,71474991
584,813965	555	24,1945345	10,6195183	17,1544854	24,5494165	23,9788859	6,80892851
586,69458	555	23,8513814	11,4834176	18,7019585	25,0387809	23,9673969	6,24630816
588,57373	555	25,478643	12,9648824	19,2166297	29,6838242	23,004134	5,52523676
590,451111	555	25,1183361	13,9257623	18,5468524	27,3359805	24,393223	4,89928946
592,326965	555	26,5869495	11,5188233	16,3639475	28,4035873	23,3808998	5,1238413
594,201233	555	24,3228918	10,5850841	16,074716	26,1527523	24,2702707	6,71821469
596,073914	555	26,0580822	12,222372	17,3172482	26,3429605	24,1867156	6,42004654
597,944946	555	25,169595	12,384535	19,8968353	25,3668127	25,5506524	7,28103345
599,814453	555	24,3332682	12,5279429	19,5907075	25,0474994	23,5890646	5,10192239
601,682251	555	27,2889384	11,2916941	18,2530806	27,4873648	22,3266664	5,08990754
603,548523	555	24,3957073	12,5576496	17,8702793	28,2882457	24,5152696	6,6285159
605,413147	555	25,897916	12,9656786	19,1265072	30,2145118	27,0498691	5,75788756
607,276245	555	25,6872818	10,8025894	16,7851759	27,5973772	26,2370535	7,55109736
609,137695	555	26,9292189	11,1279966	17,7848198	20,1092609	26,0245679	7,62489228
610,997437	555	26,2116173	8,49117939	14,821475	18,4342679	25,5614447	5,83729721
612,855713	555	26,3068553	11,7547002	17,3123036	19,298703	24,2836372	7,12753166
614,712341	555	25,4326947	11,0981555	15,6402966	18,1467211	25,0374673	5,5107555
616,567322	555	26,06208	11,6268118	15,7431077	19,0271509	22,1358554	6,63724231
618,420715	555	23,3439449	10,9539085	16,2831976	18,1066996	23,8935933	6,30170866
620,272522	555	23,3935804	10,5450263	15,8861317	16,4170851	23,6850719	7,09368272
622,122681	555	24,1360373	11,2607041	16,7855828	14,0278729	24,6889923	5,54845721
623,971313	555	24,4623377	9,90179957	16,5712453	13,7189534	23,7141868	5,56364391
625,818237	555	22,4688039	10,6385027	14,2848941	12,5527183	24,5054853	5,67288666
627,663574	555	23,1189899	10,8319114	14,920797	15,1945301	24,2864768	6,29485862
629,507324	555	22,706833	9,21735961	17,0110634	14,7428391	22,2113043	5,62298958
631,349365	555	22,0216822	8,69705854	15,356341	12,0312951	22,8426695	5,62713384
633,18988	555	22,688691	9,95012663	15,480156	13,2917365	21,3109024	3,87768845
635,028809	555	23,0266595	9,41616059	15,6466937	12,1401423	19,8019932	4,49190256
636,865967	555	22,9753553	9,60746159	15,3194759	11,0140434	19,9217235	3,88154752
638,70166	555	20,4337674	7,05522514	14,3588942	7,22918528	18,9625029	4,41327476
640,535645	555	20,5180747	6,70123285	15,325084	5,37682082	17,4599347	2,55922922
642,368042	555	19,3084734	8,47491763	13,8444723	6,97181665	15,9800869	3,88764859

 This journal is © The Royal Society of Chemistry 20xx

Please do not adjust margins

644,198853	555	19,4384867	7,25460972	16,437117	6,45621039	16,5454322	2,56784184
646,028015	555	18,134113	6,02298526	11,7670345	6,64077497	16,1216907	3,01405988
647,855591	555	18,3029778	6,46300807	10,6122039	6,28383539	16,1146002	3,45578343
649,681519	555	19,2899432	6,23059734	13,1584841	6,40646938	16,2899235	3,83062971
651,505798	555	18,2980646	7,32867695	10,9806826	5,89671686	13,8541452	3,48856167
653,328491	555	20,1451474	7,70986571	10,656334	5,46679527	15,1520494	3,7684931
655,149536	555	16,7137969	7,19801244	11,2342768	5,75648255	13,5873844	4,41254104
656,968994	555	18,0461038	6,77602731	11,280709	6,41249214	14,6374427	2,80314556
658,786743	555	15,9567488	5,90072706	10,2466786	8,25825284	15,6155371	3,54345441
660,602905	555	15,7219477	8,46259627	10,634548	8,18686814	14,5605227	2,9143434
662,41748	555	17,0705855	5,75847904	9,22149032	8,68051434	15,1744038	5,21449396
664,230469	555	16,9213361	4,67082427	11,8926655	8,78919699	17,0361951	3,8498403
666,041748	555	15,1195431	6,23913045	9,62314329	7,88802389	15,4156111	4,31601637
667,85144	555	14,7935641	6,34828567	12,4985255	9,19733454	13,0656802	3,68330289
669,659424	555	14,4553878	5,80805974	10,6822076	8,29445103	15,7660517	3,69080065
671,46582	555	15,4840014	5,7217126	10,6931298	8,67195102	16,7051059	4,43348925
673,27063	555	13,6015889	7,13384163	10,2723124	8,42808184	17,3264925	3,6163247
675,073792	555	14,0913114	6,49779932	9,92120748	4,82532101	18,3810262	2,88004995
676,875305	555	13,7178863	5,38287019	8,43606283	8,16437977	15,7786973	4,92302288
678,675171	555	13,53466	5,66219421	8,53012222	6,40263331	14,2030844	2,78705958
680,47345	555	12,8759024	5,56512027	9,63538659	5,84142045	11,8732492	3,6204119
682,27002	555	10,8755467	5,4913317	9,94768315	5,30340916	9,58736364	2,32881906
684,065063	555	11,7510562	6,16326332	10,1673034	4,57422455	11,3002608	0,84116147
685,858398	555	14,088788	5,13830415	8,95861456	4,48283722	10,2774675	2,15057715
687,650024	555	9,33959179	3,74066069	8,22022107	6,07653907	7,76251991	3,65025332
689,440186	555	9,36117985	5,71769328	8,89461439	4,68506517	9,37405137	0,84431324
691,228577	555	9,36578492	3,5635939	10,3040936	5,0623595	7,97186335	0,84472859
693,015381	555	9,29556025	4,88882115	6,94936345	3,75937293	10,1545544	1,12915126
694,800476	555	10,5135627	4,98159234	5,72709847	5,0738858	8,46001503	2,16366601
696,583984	555	8,67099718	2,17054845	5,65594788	3,30190296	9,4379562	0,47226061
698,365845	555	8,69536177	3,78547384	6,14449387	2,55433959	7,00371214	3,50454834
700,146118	555	10,3432599	3,04047438	6,8333925	3,22942275	7,98191256	1,42643735
701,924683	555	10,3830239	3,719824	4,47783559	3,0511417	8,77570311	2,29107391
347,741211	560	0	0	0	0	0	0
349,808533	560	0	0	0	0	0	0
351,874329	560	0	0	0	0	0	0
353,938568	560	0	0	0	0	0	0
356,001343	560	0	0	0	0	0	0
358,062561	560	0	0	0	0	0	0
360,122253	560	0	0	0	0	0	0
362,180481	560	0	0	0	0	0	0
364,237152	560	0	0	0	0	0	0
366,292328	560	0	0	0	0	0	0
368,345978	560	0	0	0	0	0	0
370,398132	560	0	0	0	0	0	0
372,44873	560	0	0	0	0	0	0
374,497803	560	0	0	0	0	0	0

This journal is © The Royal Society of Chemistry 20xx

Please do not adjust margins

376,545349	560	0	0	0	0	0	0
378,5914	560	0	0	0	0	0	0
380,635925	560	0	0	0	0	0	0
382,678955	560	0	0	0	0	0	0
384,720398	560	0	0	0	0	0	0
386,760376	560	0	0	0	0	0	0
388,798767	560	0	0	0	0	0	0
390,835663	560	0	0	0	0	0	0
392,871033	560	0	0	0	0	0	0
394,904877	560	0	0	0	0	0	0
396,937195	560	0	0	0	0	0	0
398,967957	560	0	0	0	0	0	0
400,997253	560	0	0	0	0	0	0
403,024963	560	0	0	0	0	0	0
405,051147	560	0	0	0	0	0	0
407,075806	560	0	0	0	0	0	0
409,098938	560	0	0	0	0	0	0
411,120544	560	0	0	0	0	0	0
413,140564	560	0	0	0	0	0	0
415,159119	560	0	0	0	0	0	0
417,176086	560	0	0	0	0	0	0
419,191528	560	0	0	0	0	0	0
421,205444	560	0	0	0	0	0	0
423,217834	560	0	0	0	0	0	0
425,228668	560	0	0	0	0	0	0
427,237976	560	0	0	0	0	0	0
429,245728	560	0	0	0	0	0	0
431,251953	560	0	0	0	0	0	0
433,256592	560	0	0	0	0	0	0
435,259766	560	0	0	0	0	0	0
437,261353	560	0	0	0	0	0	0
439,261383	560	0	0	0	0	0	0
441,259888	560	0	0	0	0	0	0
443,256836	560	0	0	0	0	0	0
445,252258	560	0	0	0	0	0	0
447,246094	560	0	0	0	0	0	0
449,238434	560	0	0	0	0	0	0
451,229187	560	0	0	0	0	0	0
453,218445	560	0	0	0	0	0	0
455,206116	560	0	0	0	0	0	0
457,192261	560	0	0	0	0	0	0
459,176819	560	0	0	0	0	0	0
461,15979	560	0	0	0	0	0	0
463,141296	560	0	0	0	0	0	0
465,121216	560	0	0	0	0	0	0
467,099579	560	0	0	0	0	0	0
469,076385	560	0	0	0	0	0	0

 This journal is © The Royal Society of Chemistry 20xx

Please do not adjust margins

Please do not adjust margins

471,051636	560	0	0	0	0	0	0
473,02536	560	0	0	0	0	0	0
474,997498	560	0	0	0	0	0	0
476,968079	560	0	0	0	0	0	0
478,937134	560	0	0	0	0	0	0
480,904572	560	0	0	0	0	0	0
482,870453	560	0	0	0	0	0	0
484,834808	560	0	0	0	0	0	0
486,797607	560	0	0	0	0	0	0
488,75885	560	0	0	0	0	0	0
490,718506	560	0	0	0	0	0	0
492,676605	560	0	0	0	0	0	0
494,633118	560	0	0	0	0	0	0
496,588104	560	0	0	0	0	0	0
498,541504	560	0	0	0	0	0	0
500,493347	560	0	0	0	0	0	0
502,443665	560	0	0	0	0	0	0
504,392365	560	0	0	0	0	0	0
506,339539	560	0	0	0	0	0	0
508,285095	560	0	0	0	0	0	0
510,229126	560	0	0	0	0	0	0
512,17157	560	0	0	0	0	0	0
514,112427	560	0	0	0	0	0	0
516,051697	560	0	0	0	0	0	0
517,989441	560	0	0	0	0	0	0
519,925598	560	0	0	0	0	0	0
521,860168	560	0	0	0	0	0	0
523,793152	560	0	0	0	0	0	0
525,724609	560	0	0	0	0	0	0
527,654419	560	0	0	0	0	0	0
529,582764	560	0	0	0	0	0	0
531,50946	560	0	0	0	0	0	0
533,43457	560	0	0	0	0	0	0
535,358093	560	0	0	0	0	0	0
537,280029	560	0	0	0	0	0	0
539,200439	560	0	0	0	0	0	0
541,119263	560	0	0	0	0	0	0
543,036438	560	0	0	0	0	0	0
544,952087	560	0	0	0	0	0	0
546,866211	560	0	0	0	0	0	0
548,778687	560	0	0	0	0	0	0
550,689514	560	0	0	0	0	0	0
552,598816	560	0	0	0	0	0	0
554,506531	560	0	0	0	0	0	0
556,41272	560	0	0	0	0	0	0
558,317261	560	0	0	0	0	0	0
560,220215	560	0	0	0	0	0	0

This journal is © The Royal Society of Chemistry 20xx

Please do not adjust margins

562,121582	560	0	0	0	0	0	0
564,021362	560	0	0	0	0	0	0
565,919556	560	0	0	0	0	0	0
567,816162	560	0	0	0	0	0	0
569,711182	560	0	0	0	0	0	0
571,604614	560	18,5040831	10,6099943	14,7327291	38,8290111	22,8810969	7,33608214
573,49646	560	18,0635826	8,16648857	12,9599865	24,9995207	22,0403267	4,46418991
575,386719	560	17,2062055	8,10752389	12,169822	21,7624006	17,9650528	6,46703932
577,27533	560	17,9654387	7,48629564	13,4015513	16,8060141	16,1485227	4,75168954
579,162354	560	18,8529692	8,00908573	13,6917919	14,2691042	19,0350122	5,93634039
581,047852	560	18,9302603	8,29323039	14,5862118	17,3439404	16,5143454	4,11370698
582,931641	560	20,3542722	10,6388189	13,9156185	18,3494351	17,268854	5,03720565
584,813965	560	20,5746365	8,9864933	14,1195381	19,1527092	20,1708557	6,22854971
586,69458	560	20,1308081	9,0976129	15,2576274	20,6423279	20,3997886	5,23412543
588,57373	560	20,7431261	9,82214864	16,1840276	23,4594147	19,8276376	4,32780243
590,451111	560	20,3321464	9,39808452	14,7055015	25,6363863	22,5713321	4,70931877
592,326965	560	24,0099017	9,93307731	14,0889397	24,7945804	22,0410743	5,54006279
594,201233	560	21,1673609	10,018407	13,6869038	26,227459	20,0509646	5,76226867
596,073914	560	21,1458265	9,38926918	16,2451779	23,4340938	21,2505405	5,276525
597,944946	560	21,1267138	10,432833	17,1078187	24,9036226	22,9859888	6,32610695
599,814453	560	21,2514847	11,2827016	17,0672179	25,7193277	22,5813395	4,82097433
601,682251	560	23,9061484	8,46391263	17,5509337	24,0005404	20,8691106	4,98451641
603,548523	560	21,3300969	8,7069717	14,9865032	26,3875246	22,8283765	6,6317701
605,413147	560	23,2060276	10,3437233	16,0918654	26,3427707	20,3541229	4,96539184
607,276245	560	21,7822273	9,54688384	15,0251836	23,8720952	21,7996378	6,00159633
609,137695	560	22,6144161	11,264933	15,0015487	27,2147156	23,5863516	6,60005556
610,997437	560	22,485566	8,85614301	14,4230553	23,2738448	23,3724012	7,04819978
612,855713	560	23,6227869	11,2044961	15,905907	21,2842128	22,5117359	4,70051146
614,712341	560	24,2090156	10,3638158	16,9946438	20,7318745	21,613794	6,1096946
616,567322	560	22,4042927	8,63125005	15,0062329	20,0565038	21,1135117	4,45612554
618,420715	560	21,4769069	9,25513764	13,6303292	16,8547849	21,6688219	4,02516405
620,272522	560	22,0475133	10,8497854	15,6731837	18,2033655	20,2263746	6,2259632
622,122681	560	22,0949984	9,55785243	15,7946883	19,5581412	19,6556968	5,88785849
623,971313	560	19,4299996	8,52889252	14,8700471	15,5662164	21,7332394	5,110903
625,818237	560	20,5659056	8,91566373	13,8687299	14,8330506	21,9957237	6,10421939
627,663574	560	21,397844	9,01976477	14,5125463	13,7977582	19,2026593	7,62153114
629,507324	560	20,7035296	8,17554451	12,2719246	14,9323112	21,1647148	3,74047613
631,349365	560	18,6735862	9,24873138	15,751678	12,5417122	18,4215333	5,34747564
633,18988	560	18,3606741	7,75498647	14,4504555	12,8384672	19,2673571	5,18129742
635,028809	560	19,0622908	8,01759687	13,9069225	14,9692444	21,6627997	5,53531236
636,865967	560	19,8064377	8,74419557	13,4826563	11,1555937	18,7507949	5,36529709
638,70166	560	17,3178454	5,08870616	12,7753641	10,7152585	16,795656	3,75777304
640,535645	560	17,0469121	8,1225826	11,0759034	10,5347356	16,5246046	3,04144802
642,368042	560	18,6759102	8,04300595	12,2518361	9,2960427	13,2356172	3,58248689
644,198853	560	18,8953045	7,52300092	12,1886461	7,434963	12,7264354	2,51315108
646,028015	560	17,3033995	6,72474435	12,02331	6,18803122	14,3562529	4,04367056
647,855591	560	16,9373273	5,37742939	10,672721	5,46817231	13,9015033	3,77243264

 This journal is © The Royal Society of Chemistry 20xx

649,681519	560	16,2171819	6,66768375	10,0987129	4,68635848	14,6972973	3,160531
651,505798	560	15,5600652	5,60938235	10,50234	6,87742458	13,399595	2,99216839
653,328491	560	15,7895171	6,80645932	11,3520615	6,44476712	14,4398844	2,27378154
655,149536	560	15,8468553	7,92416457	6,74482498	5,19275676	11,7579212	3,10357245
656,968994	560	15,4551003	6,03628099	11,1658059	4,02501107	11,9895646	3,39137624
658,786743	560	15,6211258	7,62749427	8,73639244	5,42306362	13,6104385	2,11826539
660,602905	560	14,7370954	5,71115557	9,58672725	4,97525049	10,6929342	2,95413962
662,41748	560	15,1733797	5,64428199	8,88903943	5,73798552	12,8706438	3,61653117
664,230469	560	13,9981788	5,65541937	9,37046375	7,51119245	11,6899359	2,50869279
666,041748	560	13,2807447	6,03726359	10,5958637	9,38289908	11,339482	3,90953233
667,85144	560	13,0378699	6,79895018	8,38815098	9,31553686	10,8114412	3,64025949
669,659424	560	11,1980657	6,71946428	8,68540017	6,81418474	11,8607589	2,15118978
671,46582	560	14,3855446	5,5118576	7,7594118	7,56867552	9,81616568	2,52789179
673,27063	560	12,2850032	4,50179385	9,76073309	8,06736502	12,1071153	3,38372924
675,073792	560	13,2482527	4,41649497	7,71078272	6,39113049	12,6006017	1,78929612
676,875305	560	11,9305612	5,26120916	7,23924037	7,611521	13,538378	4,04869195
678,675171	560	12,2143263	5,44997547	9,12101183	7,5187463	14,2927812	1,22421767
680,47345	560	10,421195	5,44581553	10,0536426	6,85561914	14,9395893	3,10525743
682,27002	560	10,0804677	3,20343717	8,20278403	6,97361398	12,8227981	3,96584121
684,065063	560	9,83040396	5,01019103	8,4192451	6,80769048	10,9734454	1,1368637
685,858398	560	12,2933914	5,39068062	8,61221512	4,35126435	9,08545292	2,17994391
687,650024	560	9,56050729	3,50268979	7,57869921	6,15463355	8,24187092	2,75135537
689,440186	560	9,96211513	4,36476111	8,64069689	4,65037117	7,12148418	1,8067788
691,228577	560	10,1568637	2,65811808	7,59995387	3,51323218	8,54998496	1,90280801
693,015381	560	8,75507501	3,14069941	6,09532881	3,80768765	9,71447217	2,67066369
694,800476	560	9,60914899	4,18655325	4,95121765	5,61493645	9,90247799	2,38392556
696,583984	560	7,92849905	3,43918689	4,78000126	3,72654846	8,12603717	1,81909598
698,365845	560	8,71711728	4,59846752	6,80667423	3,3537356	6,23148919	3,07234939
700,146118	560	8,27105787	3,07788916	6,06384761	3,75194527	8,18141696	1,92788769
701,924683	560	10,1372072	2,22073762	4,54121436	3,86294296	6,37705191	2,6126541
347,741211	565	0	0	0	0	0	0
349,808533	565	0	0	0	0	0	0
351,874329	565	0	0	0	0	0	0
353,938568	565	0	0	0	0	0	0
356,001343	565	0	0	0	0	0	0
358,062561	565	0	0	0	0	0	0
360,122253	565	0	0	0	0	0	0
362,180481	565	0	0	0	0	0	0
364,237152	565	0	0	0	0	0	0
366,292328	565	0	0	0	0	0	0
368,345978	565	0	0	0	0	0	0
370,398132	565	0	0	0	0	0	0
372,44873	565	0	0	0	0	0	0
374,497803	565	0	0	0	0	0	0
376,545349	565	0	0	0	0	0	0
378,5914	565	0	0	0	0	0	0
380,635925	565	0	0	0	0	0	0

382,678955	565	0	0	0	0	0	0
384,720398	565	0	0	0	0	0	0
386,760376	565	0	0	0	0	0	0
388,798767	565	0	0	0	0	0	0
390,835663	565	0	0	0	0	0	0
392,871033	565	0	0	0	0	0	0
394,904877	565	0	0	0	0	0	0
396,937195	565	0	0	0	0	0	0
398,967957	565	0	0	0	0	0	0
400,997253	565	0	0	0	0	0	0
403,024963	565	0	0	0	0	0	0
405,051147	565	0	0	0	0	0	0
407,075806	565	0	0	0	0	0	0
409,098938	565	0	0	0	0	0	0
411,120544	565	0	0	0	0	0	0
413,140564	565	0	0	0	0	0	0
415,159119	565	0	0	0	0	0	0
417,176086	565	0	0	0	0	0	0
419,191528	565	0	0	0	0	0	0
421,205444	565	0	0	0	0	0	0
423,217834	565	0	0	0	0	0	0
425,228668	565	0	0	0	0	0	0
427,237976	565	0	0	0	0	0	0
429,245728	565	0	0	0	0	0	0
431,251953	565	0	0	0	0	0	0
433,256592	565	0	0	0	0	0	0
435,259766	565	0	0	0	0	0	0
437,261353	565	0	0	0	0	0	0
439,261383	565	0	0	0	0	0	0
441,259888	565	0	0	0	0	0	0
443,256836	565	0	0	0	0	0	0
445,252258	565	0	0	0	0	0	0
447,246094	565	0	0	0	0	0	0
449,238434	565	0	0	0	0	0	0
451,229187	565	0	0	0	0	0	0
453,218445	565	0	0	0	0	0	0
455,206116	565	0	0	0	0	0	0
457,192261	565	0	0	0	0	0	0
459,176819	565	0	0	0	0	0	0
461,15979	565	0	0	0	0	0	0
463,141296	565	0	0	0	0	0	0
465,121216	565	0	0	0	0	0	0
467,099579	565	0	0	0	0	0	0
469,076385	565	0	0	0	0	0	0
471,051636	565	0	0	0	0	0	0
473,02536	565	0	0	0	0	0	0
474,997498	565	0	0	0	0	0	0

This journal is © The Royal Society of Chemistry 20xx

476,968079	565	0	0	0	0	0	0
478,937134	565	0	0	0	0	0	0
480,904572	565	0	0	0	0	0	0
482,870453	565	0	0	0	0	0	0
484,834808	565	0	0	0	0	0	0
486,797607	565	0	0	0	0	0	0
488,75885	565	0	0	0	0	0	0
490,718506	565	0	0	0	0	0	0
492,676605	565	0	0	0	0	0	0
494,633118	565	0	0	0	0	0	0
496,588104	565	0	0	0	0	0	0
498,541504	565	0	0	0	0	0	0
500,493347	565	0	0	0	0	0	0
502,443665	565	0	0	0	0	0	0
504,392365	565	0	0	0	0	0	0
506,339539	565	0	0	0	0	0	0
508,285095	565	0	0	0	0	0	0
510,229126	565	0	0	0	0	0	0
512,17157	565	0	0	0	0	0	0
514,112427	565	0	0	0	0	0	0
516,051697	565	0	0	0	0	0	0
517,989441	565	0	0	0	0	0	0
519,925598	565	0	0	0	0	0	0
521,860168	565	0	0	0	0	0	0
523,793152	565	0	0	0	0	0	0
525,724609	565	0	0	0	0	0	0
527,654419	565	0	0	0	0	0	0
529,582764	565	0	0	0	0	0	0
531,50946	565	0	0	0	0	0	0
533,43457	565	0	0	0	0	0	0
535,358093	565	0	0	0	0	0	0
537,280029	565	0	0	0	0	0	0
539,200439	565	0	0	0	0	0	0
541,119263	565	0	0	0	0	0	0
543,036438	565	0	0	0	0	0	0
544,952087	565	0	0	0	0	0	0
546,866211	565	0	0	0	0	0	0
548,778687	565	0	0	0	0	0	0
550,689514	565	0	0	0	0	0	0
552,598816	565	0	0	0	0	0	0
554,506531	565	0	0	0	0	0	0
556,41272	565	0	0	0	0	0	0
558,317261	565	0	0	0	0	0	0
560,220215	565	0	0	0	0	0	0
562,121582	565	0	0	0	0	0	0
564,021362	565	0	0	0	0	0	0
565,919556	565	0	0	0	0	0	0

This journal is © The Royal Society of Chemistry 20xx

Please do not adjust margins

567,816162	565	0	0	0	0	0	0
569,711182	565	0	0	0	0	0	0
571,604614	565	0	0	0	0	0	0
573,49646	565	0	0	0	0	0	0
575,386719	565	13,3357598	10,8980694	12,4260427	48,0852766	23,2624641	8,81639633
577,27533	565	16,3600517	7,67025694	13,1691586	33,9192197	20,940639	5,23426612
579,162354	565	16,4086147	7,77756398	11,5148844	25,0494823	21,1721764	4,4877352
581,047852	565	14,2677764	6,96059626	13,5174454	19,7988421	15,56157	3,82596237
582,931641	565	16,3910012	7,21527147	9,69177916	16,399943	14,8813225	4,93125355
584,813965	565	16,6886431	7,31895344	11,7623616	14,48274	16,2837202	4,68822895
586,69458	565	17,6779753	6,91405409	12,3104848	15,295091	16,1614115	4,36027137
588,57373	565	18,4563344	10,1245231	11,3430908	18,9817444	17,8782758	4,89849888
590,451111	565	17,1502451	8,65746445	12,2257282	18,4595708	20,5545888	6,24336611
592,326965	565	17,9513996	7,08949091	12,0092395	20,2391976	21,2185219	4,90923303
594,201233	565	18,8121708	7,23866934	11,5819341	23,5059497	22,0198301	6,18925699
596,073914	565	19,7496528	8,80293029	15,8517414	24,6560281	22,4472084	4,00774583
597,944946	565	18,0430442	7,55124006	15,7484395	21,9658283	25,4527736	6,1396559
599,814453	565	17,9115529	8,59684669	14,5571449	24,1316841	21,6634192	5,05985292
601,682251	565	19,4616921	8,22292713	14,7885254	24,0748548	19,5751679	4,07377383
603,548523	565	18,2724153	7,32300416	15,9055467	24,6414733	19,3564198	5,21385308
605,413147	565	19,4751443	9,424488	15,1728563	25,8340733	22,5000715	3,88170256
607,276245	565	20,4134033	7,91508382	13,0358583	24,7383431	22,9063603	6,16604427
609,137695	565	20,2055918	10,9758795	16,0053661	21,8866828	22,2545885	5,54071046
610,997437	565	19,1012884	7,4783783	13,9222844	22,8988215	18,2443578	5,99229254
612,855713	565	19,2843592	9,94546555	13,1340254	24,8454611	20,1025546	6,25893746
614,712341	565	19,3363865	8,82504198	15,9978	20,3186989	18,4770569	5,2151209
616,567322	565	19,8874683	7,77437046	13,3605524	20,2520654	18,8508914	6,54801806
618,420715	565	18,146353	9,55531102	12,582714	18,776222	21,0057962	4,07637841
620,272522	565	19,0720072	7,53637403	12,2542719	18,1948575	18,4746655	5,8611528
622,122681	565	20,1798605	8,70770995	13,6155195	14,3203962	21,5408568	4,36083393
623,971313	565	19,0762568	7,92966736	12,6712137	17,5704332	21,2427946	4,818971
625,818237	565	18,7936043	7,51373109	13,3483486	16,4757603	18,2800028	4,39003549
627,663574	565	19,4541944	7,52693874	11,935913	17,1326184	17,7735433	6,37225334
629,507324	565	17,9283538	6,57348651	11,9045163	16,5860136	18,7631599	4,96056557
631,349365	565	18,2120433	7,83992909	14,981865	14,613666	17,783975	4,24215302
633,18988	565	15,3627581	7,49693836	11,5792579	12,4777481	18,1874426	3,9806511
635,028809	565	16,3470262	7,67299098	12,7476398	13,4642854	19,804305	3,88785274
636,865967	565	19,2677884	8,4085438	11,5907815	14,7528772	17,2997943	4,1657314
638,70166	565	15,0244674	5,69924232	12,9561963	11,9536844	16,2218109	5,34594116
640,535645	565	16,6505677	7,23580387	11,5044386	12,0420304	18,3028154	3,17073855
642,368042	565	15,7646237	7,87842108	12,153172	12,872401	15,6940766	2,17684129
644,198853	565	16,8881956	5,71737178	11,0886997	10,5381689	13,8187641	3,90858832
646,028015	565	14,9079127	7,26856298	11,5565233	10,2775191	13,1976408	3,36709899
647,855591	565	15,4465276	6,81128081	9,55040368	7,7274771	14,6474872	4,09331932
649,681519	565	15,072514	4,0173531	11,0646481	6,39788822	12,896628	3,5665435
651,505798	565	14,2171822	6,14245824	9,09007596	6,60771321	11,9393447	3,39754113
653,328491	565	13,8009737	5,51766458	9,48650366	5,70751438	13,3580104	2,48692907

 This journal is © The Royal Society of Chemistry 20xx

Please do not adjust margins

Please do not adjust margins

655,149536	565	15,0515185	4,24557113	7,57978784	4,98911286	11,83472	3,1430609
656,968994	565	11,3121153	5,00453035	10,3028518	4,73141479	10,9552251	2,13497598
658,786743	565	13,7887212	4,84229448	10,0724824	5,03376449	13,8996496	2,89137976
660,602905	565	13,3544749	4,57375015	9,53545535	3,83099419	11,8754427	4,3006072
662,41748	565	13,2268509	4,78181863	8,35752797	5,16222456	11,7409345	2,15996319
664,230469	565	13,2529504	3,38206178	8,75037958	4,51410392	10,728861	3,10519276
666,041748	565	12,4296583	4,61176232	7,63520609	3,9570488	13,0112689	2,16817465
667,85144	565	10,4809453	4,71881617	9,26305064	7,65242977	8,60348045	2,93035568
669,659424	565	8,9884382	5,01212713	8,52419991	6,4373393	11,652552	3,69408092
671,46582	565	10,228883	6,43722881	8,81734614	9,76064545	9,10397374	3,12895677
673,27063	565	10,7443782	3,99151451	9,2326222	9,13294694	9,04444157	2,9508403
675,073792	565	12,4798583	4,09442003	6,4848175	7,81608132	10,0157383	2,38429252
676,875305	565	12,6679885	4,47444789	7,7230976	7,33809958	9,72772978	4,48162014
678,675171	565	12,860528	4,47515897	7,81968701	6,38611436	11,2554366	2,1934818
680,47345	565	9,2334743	4,66203004	7,90900752	6,47648226	8,10154117	1,04825577
682,27002	565	8,11922727	3,43703417	10,1356621	6,40335282	12,337887	2,96441248
684,065063	565	10,0629703	4,02320128	6,90748381	6,90408686	14,2981046	-0,1918881
685,858398	565	12,0808577	4,69579233	6,23865639	4,89253856	11,7123047	1,63176387
687,650024	565	7,86995562	4,50860166	5,47625638	8,25835864	8,55270997	1,34514045
689,440186	565	9,33110035	5,09591966	7,70373966	6,15996091	8,28351921	1,15564259
691,228577	565	8,66198099	2,98209852	7,61118527	2,88890212	6,64934879	1,05986016
693,015381	565	8,00845988	3,56827415	5,21573067	4,05468443	9,56448111	1,352322
694,800476	565	9,26048334	3,95304589	5,31099138	4,7292836	7,34059597	2,70396845
696,583984	565	6,29542582	3,09775673	5,04156747	4,55456043	5,91557407	1,26048091
698,365845	565	6,99298926	2,91230728	7,68082626	2,52662372	7,0019365	4,08376574
700,146118	565	8,28858513	1,94929267	4,68547041	3,9026385	7,02990215	2,14765899
701,924683	565	10,0824277	3,81573386	4,40951568	4,30940613	8,52712151	3,4298656
347,741211	570	0	0	0	0	0	0
349,808533	570	0	0	0	0	0	0
351,874329	570	0	0	0	0	0	0
353,938568	570	0	0	0	0	0	0
356,001343	570	0	0	0	0	0	0
358,062561	570	0	0	0	0	0	0
360,122253	570	0	0	0	0	0	0
362,180481	570	0	0	0	0	0	0
364,237152	570	0	0	0	0	0	0
366,292328	570	0	0	0	0	0	0
368,345978	570	0	0	0	0	0	0
370,398132	570	0	0	0	0	0	0
372,44873	570	0	0	0	0	0	0
374,497803	570	0	0	0	0	0	0
376,545349	570	0	0	0	0	0	0
378,5914	570	0	0	0	0	0	0
380,635925	570	0	0	0	0	0	0
382,678955	570	0	0	0	0	0	0
384,720398	570	0	0	0	0	0	0
386,760376	570	0	0	0	0	0	0

This journal is © The Royal Society of Chemistry 20xx

Please do not adjust margins

388,798767	570	0	0	0	0	0	0
390,835663	570	0	0	0	0	0	0
392,871033	570	0	0	0	0	0	0
394,904877	570	0	0	0	0	0	0
396,937195	570	0	0	0	0	0	0
398,967957	570	0	0	0	0	0	0
400,997253	570	0	0	0	0	0	0
403,024963	570	0	0	0	0	0	0
405,051147	570	0	0	0	0	0	0
407,075806	570	0	0	0	0	0	0
409,098938	570	0	0	0	0	0	0
411,120544	570	0	0	0	0	0	0
413,140564	570	0	0	0	0	0	0
415,159119	570	0	0	0	0	0	0
417,176086	570	0	0	0	0	0	0
419,191528	570	0	0	0	0	0	0
421,205444	570	0	0	0	0	0	0
423,217834	570	0	0	0	0	0	0
425,228668	570	0	0	0	0	0	0
427,237976	570	0	0	0	0	0	0
429,245728	570	0	0	0	0	0	0
431,251953	570	0	0	0	0	0	0
433,256592	570	0	0	0	0	0	0
435,259766	570	0	0	0	0	0	0
437,261353	570	0	0	0	0	0	0
439,261383	570	0	0	0	0	0	0
441,259888	570	0	0	0	0	0	0
443,256836	570	0	0	0	0	0	0
445,252258	570	0	0	0	0	0	0
447,246094	570	0	0	0	0	0	0
449,238434	570	0	0	0	0	0	0
451,229187	570	0	0	0	0	0	0
453,218445	570	0	0	0	0	0	0
455,206116	570	0	0	0	0	0	0
457,192261	570	0	0	0	0	0	0
459,176819	570	0	0	0	0	0	0
461,15979	570	0	0	0	0	0	0
463,141296	570	0	0	0	0	0	0
465,121216	570	0	0	0	0	0	0
467,099579	570	0	0	0	0	0	0
469,076385	570	0	0	0	0	0	0
471,051636	570	0	0	0	0	0	0
473,02536	570	0	0	0	0	0	0
474,997498	570	0	0	0	0	0	0
476,968079	570	0	0	0	0	0	0
478,937134	570	0	0	0	0	0	0
480,904572	570	0	0	0	0	0	0

 This journal is © The Royal Society of Chemistry 20xx

Please do not adjust margins

482,870453	570	0	0	0	0	0	0
484,834808	570	0	0	0	0	0	0
486,797607	570	0	0	0	0	0	0
488,75885	570	0	0	0	0	0	0
490,718506	570	0	0	0	0	0	0
492,676605	570	0	0	0	0	0	0
494,633118	570	0	0	0	0	0	0
496,588104	570	0	0	0	0	0	0
498,541504	570	0	0	0	0	0	0
500,493347	570	0	0	0	0	0	0
502,443665	570	0	0	0	0	0	0
504,392365	570	0	0	0	0	0	0
506,339539	570	0	0	0	0	0	0
508,285095	570	0	0	0	0	0	0
510,229126	570	0	0	0	0	0	0
512,17157	570	0	0	0	0	0	0
514,112427	570	0	0	0	0	0	0
516,051697	570	0	0	0	0	0	0
517,989441	570	0	0	0	0	0	0
519,925598	570	0	0	0	0	0	0
521,860168	570	0	0	0	0	0	0
523,793152	570	0	0	0	0	0	0
525,724609	570	0	0	0	0	0	0
527,654419	570	0	0	0	0	0	0
529,582764	570	0	0	0	0	0	0
531,50946	570	0	0	0	0	0	0
533,43457	570	0	0	0	0	0	0
535,358093	570	0	0	0	0	0	0
537,280029	570	0	0	0	0	0	0
539,200439	570	0	0	0	0	0	0
541,119263	570	0	0	0	0	0	0
543,036438	570	0	0	0	0	0	0
544,952087	570	0	0	0	0	0	0
546,866211	570	0	0	0	0	0	0
548,778687	570	0	0	0	0	0	0
550,689514	570	0	0	0	0	0	0
552,598816	570	0	0	0	0	0	0
554,506531	570	0	0	0	0	0	0
556,41272	570	0	0	0	0	0	0
558,317261	570	0	0	0	0	0	0
560,220215	570	0	0	0	0	0	0
562,121582	570	0	0	0	0	0	0
564,021362	570	0	0	0	0	0	0
565,919556	570	0	0	0	0	0	0
567,816162	570	0	0	0	0	0	0
569,711182	570	0	0	0	0	0	0
571,604614	570	0	0	0	0	0	0

This journal is © The Royal Society of Chemistry 20xx

Please do not adjust margins

573,49646	570	0	0	0	0	0	0
575,386719	570	0	0	0	0	0	0
577,27533	570	0	0	0	0	0	0
579,162354	570	0	0	0	0	0	0
581,047852	570	12,540195	7,12256342	12,7869129	39,5272447	19,93516	6,09511837
582,931641	570	15,1169878	7,89491063	10,2982124	28,5284353	16,7558904	6,61021643
584,813965	570	14,3808928	6,88281602	11,357201	22,4852209	16,6267356	7,05757643
586,69458	570	13,3010926	6,90340547	9,14745889	16,7631489	15,4667809	3,88464621
588,57373	570	13,8909323	8,32701683	10,6695113	14,504914	15,0258075	4,51218728
590,451111	570	14,4541815	6,12652765	10,0655012	14,5480547	15,1612835	3,06443184
592,326965	570	15,9412135	5,04348261	9,0256201	15,4164862	14,441649	3,80618323
594,201233	570	16,0781559	6,78241125	7,85370177	16,7999835	15,9954722	5,89223455
596,073914	570	16,824901	7,46097872	11,5965452	19,532504	18,5420794	5,21568394
597,944946	570	15,7309968	7,63385521	11,6758781	23,0221265	20,0541086	4,13283805
599,814453	570	16,142054	8,60130448	12,1857763	23,2366401	24,2231356	4,6608164
601,682251	570	17,0881758	8,22350501	10,9056152	22,1973583	25,6876597	4,1133203
603,548523	570	16,5158816	8,20587046	9,99024314	24,3825967	26,7936322	5,08601045
605,413147	570	16,6661175	8,45908199	11,9323404	25,0545396	21,7551941	2,6723076
607,276245	570	17,2636881	6,66806636	12,8033126	24,6603307	20,8318584	5,24754565
609,137695	570	18,3915574	8,34416363	12,9607168	23,2882432	23,0212144	5,77210042
610,997437	570	16,7365214	6,31498749	11,3853221	21,1968624	25,026146	5,69455358
612,855713	570	17,7222392	8,45270905	12,5462123	22,7191968	22,6297387	5,87464752
614,712341	570	17,0556781	8,20786582	13,7399245	23,3165025	20,3681121	5,97974669
616,567322	570	18,7724388	7,50218011	11,8790319	20,8374514	17,8859373	4,19924894
618,420715	570	16,2933844	6,61878742	11,6281673	18,6290422	18,7183045	4,29490453
620,272522	570	17,5840307	7,26029352	11,2047038	19,0276651	17,6810842	5,73871553
622,122681	570	17,6219025	5,83870962	13,5644339	17,629503	19,7879001	5,93079645
623,971313	570	16,6784441	8,37671139	13,7817142	18,4894905	17,0461347	5,85692313
625,818237	570	14,7530838	7,32465244	12,0274857	15,846032	13,8537371	4,79450125
627,663574	570	18,0430941	6,43166011	11,1427158	16,5088417	14,6037409	5,98100596
629,507324	570	17,5852711	6,64540443	11,9258971	17,5928558	16,8633476	5,37299123
631,349365	570	17,2335016	7,10580239	12,2080002	16,967269	15,3250293	5,10354694
633,18988	570	14,8060668	6,11795518	11,871239	15,4524971	16,8237505	3,65390325
635,028809	570	14,7058618	6,29680862	10,9515062	15,2604856	16,7222996	4,47335173
636,865967	570	15,278234	7,31229107	12,5229099	11,6237881	14,0947609	4,48048602
638,70166	570	13,822047	4,02397145	9,23729459	11,0807221	14,4688099	3,47657317
640,535645	570	13,9110282	6,76634536	10,5157799	10,9871275	15,5648475	3,5674066
642,368042	570	14,1110555	6,04212232	10,0706956	11,6420585	15,5836077	3,38854194
644,198853	570	14,9679503	5,59643367	11,0099153	13,0451882	13,9636393	1,83559592
646,028015	570	16,1804309	6,52142585	8,81813312	12,0485833	14,9915081	2,66469663
647,855591	570	14,5192256	4,95777625	8,53880936	10,2965484	13,5140028	3,21459874
649,681519	570	14,5969883	5,72274849	9,23069021	11,3683794	11,7378944	4,80155466
651,505798	570	12,6161011	3,79994147	8,24905622	9,93018424	12,528536	2,68878831
653,328491	570	11,7244758	6,7866046	9,0182574	7,16804775	10,3329939	3,5341001
655,149536	570	12,7009452	6,34472558	7,27812883	6,25978203	8,31509939	2,70687058
656,968994	570	12,1907876	4,8719021	8,33885499	4,69078677	11,7267787	2,90551061
658,786743	570	13,379967	6,77806908	6,96668895	4,99608527	12,2543521	2,63692082

This journal is © The Royal Society of Chemistry 20xx

Please do not adjust margins

660,602905	570	12,1838603	4,62378975	8,58745741	4,25201698	10,204677	2,26557687
662,41748	570	11,5744829	4,83413463	7,77290972	5,12534144	8,54209864	3,79292382
664,230469	570	11,4072018	3,41906359	7,78824734	5,04035383	10,6511428	2,85030605
666,041748	570	11,6184852	4,09133398	8,37337142	3,23933507	9,24148408	3,33142535
667,85144	570	11,1729475	5,53371373	8,2055624	3,82145865	8,1204693	1,71801426
669,659424	570	8,80344954	3,44171061	6,31011089	4,88227799	8,13699939	1,24331383
671,46582	570	9,48296333	3,82803294	6,12515177	6,13304465	6,13294622	3,25506867
673,27063	570	10,7702377	3,45872923	6,43739372	4,32919407	8,46584364	3,94061026
675,073792	570	9,63480171	2,59904227	5,39086941	6,16893267	10,988235	0,38519011
676,875305	570	10,8852619	3,07976242	5,67858924	7,42059006	8,28779874	5,68047709
678,675171	570	10,1162313	3,17650973	5,9682794	8,28924889	7,90357558	1,15553488
680,47345	570	9,62715205	4,13592909	7,50274934	5,20090435	8,09016581	1,92442145
682,27002	570	7,72841046	2,31642494	6,56352448	6,66862904	8,11820071	1,54487212
684,065063	570	7,26946714	4,55141011	5,42322153	7,36957057	8,82394946	1,35625612
685,858398	570	9,21202802	3,10019079	6,78199849	4,85052699	7,95473659	2,42294756
687,650024	570	6,50335789	3,39420207	7,17666336	7,18591122	9,90474568	2,03729776
689,440186	570	8,46417821	3,40204762	6,02677863	7,00785841	11,1929275	1,2640995
691,228577	570	7,49496937	3,11197367	7,1967905	6,13489258	9,54301306	1,75115264
693,015381	570	6,83082836	3,21733342	5,45998391	4,49076614	8,29802165	1,95064253
694,800476	570	8,09738772	4,77605941	5,5560955	5,46565412	7,807952	0,58504659
696,583984	570	7,24849585	3,91456009	3,91475123	4,50776516	6,17357928	0,68530922
698,365845	570	8,05468642	1,37394586	5,79048313	4,716972	6,87880709	2,45441024
700,146118	570	8,18547694	2,16768102	3,84289491	3,35453316	6,90628096	0,88711673
701,924683	570	8,90994087	2,86838279	5,63813112	3,16934536	7,72515533	4,45263594
347,741211	575	0	0	0	0	0	0
349,808533	575	0	0	0	0	0	0
351,874329	575	0	0	0	0	0	0
353,938568	575	0	0	0	0	0	0
356,001343	575	0	0	0	0	0	0
358,062561	575	0	0	0	0	0	0
360,122253	575	0	0	0	0	0	0
362,180481	575	0	0	0	0	0	0
364,237152	575	0	0	0	0	0	0
366,292328	575	0	0	0	0	0	0
368,345978	575	0	0	0	0	0	0
370,398132	575	0	0	0	0	0	0
372,44873	575	0	0	0	0	0	0
374,497803	575	0	0	0	0	0	0
376,545349	575	0	0	0	0	0	0
378,5914	575	0	0	0	0	0	0
380,635925	575	0	0	0	0	0	0
382,678955	575	0	0	0	0	0	0
384,720398	575	0	0	0	0	0	0
386,760376	575	0	0	0	0	0	0
388,798767	575	0	0	0	0	0	0
390,835663	575	0	0	0	0	0	0
392,871033	575	0	0	0	0	0	0

Please do not adjust margins

394,904877	575	0	0	0	0	0	0
396,937195	575	0	0	0	0	0	0
398,967957	575	0	0	0	0	0	0
400,997253	575	0	0	0	0	0	0
403,024963	575	0	0	0	0	0	0
405,051147	575	0	0	0	0	0	0
407,075806	575	0	0	0	0	0	0
409,098938	575	0	0	0	0	0	0
411,120544	575	0	0	0	0	0	0
413,140564	575	0	0	0	0	0	0
415,159119	575	0	0	0	0	0	0
417,176086	575	0	0	0	0	0	0
419,191528	575	0	0	0	0	0	0
421,205444	575	0	0	0	0	0	0
423,217834	575	0	0	0	0	0	0
425,228668	575	0	0	0	0	0	0
427,237976	575	0	0	0	0	0	0
429,245728	575	0	0	0	0	0	0
431,251953	575	0	0	0	0	0	0
433,256592	575	0	0	0	0	0	0
435,259766	575	0	0	0	0	0	0
437,261353	575	0	0	0	0	0	0
439,261383	575	0	0	0	0	0	0
441,259888	575	0	0	0	0	0	0
443,256836	575	0	0	0	0	0	0
445,252258	575	0	0	0	0	0	0
447,246094	575	0	0	0	0	0	0
449,238434	575	0	0	0	0	0	0
451,229187	575	0	0	0	0	0	0
453,218445	575	0	0	0	0	0	0
455,206116	575	0	0	0	0	0	0
457,192261	575	0	0	0	0	0	0
459,176819	575	0	0	0	0	0	0
461,15979	575	0	0	0	0	0	0
463,141296	575	0	0	0	0	0	0
465,121216	575	0	0	0	0	0	0
467,099579	575	0	0	0	0	0	0
469,076385	575	0	0	0	0	0	0
471,051636	575	0	0	0	0	0	0
473,02536	575	0	0	0	0	0	0
474,997498	575	0	0	0	0	0	0
476,968079	575	0	0	0	0	0	0
478,937134	575	0	0	0	0	0	0
480,904572	575	0	0	0	0	0	0
482,870453	575	0	0	0	0	0	0
484,834808	575	0	0	0	0	0	0
486,797607	575	0	0	0	0	0	0

 This journal is © The Royal Society of Chemistry 20xx

488,75885	575	0	0	0	0	0	0
490,718506	575	0	0	0	0	0	0
492,676605	575	0	0	0	0	0	0
494,633118	575	0	0	0	0	0	0
496,588104	575	0	0	0	0	0	0
498,541504	575	0	0	0	0	0	0
500,493347	575	0	0	0	0	0	0
502,443665	575	0	0	0	0	0	0
504,392365	575	0	0	0	0	0	0
506,339539	575	0	0	0	0	0	0
508,285095	575	0	0	0	0	0	0
510,229126	575	0	0	0	0	0	0
512,17157	575	0	0	0	0	0	0
514,112427	575	0	0	0	0	0	0
516,051697	575	0	0	0	0	0	0
517,989441	575	0	0	0	0	0	0
519,925598	575	0	0	0	0	0	0
521,860168	575	0	0	0	0	0	0
523,793152	575	0	0	0	0	0	0
525,724609	575	0	0	0	0	0	0
527,654419	575	0	0	0	0	0	0
529,582764	575	0	0	0	0	0	0
531,50946	575	0	0	0	0	0	0
533,43457	575	0	0	0	0	0	0
535,358093	575	0	0	0	0	0	0
537,280029	575	0	0	0	0	0	0
539,200439	575	0	0	0	0	0	0
541,119263	575	0	0	0	0	0	0
543,036438	575	0	0	0	0	0	0
544,952087	575	0	0	0	0	0	0
546,866211	575	0	0	0	0	0	0
548,778687	575	0	0	0	0	0	0
550,689514	575	0	0	0	0	0	0
552,598816	575	0	0	0	0	0	0
554,506531	575	0	0	0	0	0	0
556,41272	575	0	0	0	0	0	0
558,317261	575	0	0	0	0	0	0
560,220215	575	0	0	0	0	0	0
562,121582	575	0	0	0	0	0	0
564,021362	575	0	0	0	0	0	0
565,919556	575	0	0	0	0	0	0
567,816162	575	0	0	0	0	0	0
569,711182	575	0	0	0	0	0	0
571,604614	575	0	0	0	0	0	0
573,49646	575	0	0	0	0	0	0
575,386719	575	0	0	0	0	0	0
577,27533	575	0	0	0	0	0	0

This journal is © The Royal Society of Chemistry 20xx

Please do not adjust margins

579,162354	575	0	0	0	0	0	0
581,047852	575	0	0	0	0	0	0
582,931641	575	0	0	0	0	0	0
584,813965	575	0	0	0	0	0	0
586,69458	575	12,3184018	6,64181095	9,52720298	35,1669385	17,0661436	5,51573444
588,57373	575	12,9091427	6,67623053	8,96156597	24,4454547	13,9875845	5,36830829
590,451111	575	13,2027526	6,11596472	8,86506416	18,0114016	13,6698727	3,55194108
592,326965	575	13,7955256	5,10775491	7,52836181	15,1592422	12,8327313	3,59091452
594,201233	575	13,552632	4,06707858	7,23146085	11,580188	13,5765423	4,52720266
596,073914	575	13,2871679	6,37257964	10,1976771	13,8514874	13,9487898	4,104116
597,944946	575	14,0934899	4,8205921	9,36974437	14,0210593	13,298579	4,55600719
599,814453	575	13,9693329	6,17023243	10,7087954	16,1676986	15,0844183	5,09061812
601,682251	575	14,298421	6,60832687	10,6835766	16,4920877	13,4170871	3,62759278
603,548523	575	12,5520161	5,32952328	10,1185988	20,5256917	20,2633963	5,42972062
605,413147	575	12,9814732	7,12403826	10,6425858	19,9492704	22,396425	3,34264886
607,276245	575	12,8717189	6,03271731	9,72585813	23,8846916	24,3461226	5,59267707
609,137695	575	15,277973	7,91110508	11,3289857	19,8875986	26,5585502	4,4130662
610,997437	575	15,3082281	6,0351556	9,63969846	20,7384883	22,0105737	4,78276915
612,855713	575	14,5931547	7,3890004	12,0765432	25,1658517	20,4845092	4,24286855
614,712341	575	15,3551195	7,40893519	11,8380247	21,7064487	22,8923476	5,34052357
616,567322	575	16,3662294	6,6028371	10,6747013	22,9973096	23,0074934	4,53072402
618,420715	575	13,7642376	7,51838096	10,8716005	19,4041937	22,224919	4,99112914
620,272522	575	13,9749975	5,62808142	9,98682339	19,263719	18,6359017	4,27421557
622,122681	575	14,6416916	6,91379717	11,0091659	18,2124608	14,3031127	3,18978174
623,971313	575	14,4081941	6,38540086	11,4042346	16,8013253	13,246038	3,56405679
625,818237	575	13,3664697	6,86851402	11,0828868	15,9509431	14,3071783	3,85336987
627,663574	575	14,5822225	7,155811	11,1023683	17,9074795	16,1698054	5,14685777
629,507324	575	13,6402437	5,16280957	7,83759842	16,7036832	15,2338576	5,35692979
631,349365	575	13,3736017	6,55052932	11,7189248	16,2542264	14,0439573	3,69715722
633,18988	575	12,7577014	5,64106165	11,283838	13,8853829	15,4659054	4,26165927
635,028809	575	13,4891976	6,37705285	9,61325068	16,6524839	14,9013175	2,77767904
636,865967	575	16,2868842	7,40547624	9,07308718	15,2891218	14,6469757	3,61674171
638,70166	575	11,1107997	3,24167731	10,374944	13,3505704	13,0782224	3,43315446
640,535645	575	11,9419159	5,09312873	9,72472356	13,1627278	11,6847756	2,59758565
642,368042	575	12,697786	4,91383953	9,82917274	14,0138556	12,5344919	2,87936469
644,198853	575	12,818065	5,66775258	10,5938032	11,6259019	11,4449533	3,53715854
646,028015	575	12,3679497	5,20920875	9,3035723	11,5462514	13,0418631	3,63444602
647,855591	575	12,734315	3,06836221	8,18354283	13,4957268	15,364007	3,35339774
649,681519	575	12,895967	4,76741182	6,91847243	13,3808629	14,1356996	3,09041152
651,505798	575	13,4180976	5,63175591	6,38362679	13,9995615	12,6897834	2,91503752
653,328491	575	12,4241154	6,1198752	8,09829631	9,70737989	11,4088651	0,66026326
655,149536	575	10,7688825	4,0632347	8,4112291	8,89133345	11,5449259	3,88129981
656,968994	575	9,58028527	5,21864099	8,63579106	6,5535922	8,64697077	3,23194179
658,786743	575	11,0558807	5,62503241	7,43761377	6,58502911	10,3115663	2,19680109
660,602905	575	11,4642356	4,10932013	7,1685082	5,73968902	9,18756915	3,92532173
662,41748	575	11,1318774	4,41576466	5,76056844	4,32410462	9,51724281	2,78891942
664,230469	575	11,5384582	3,07789768	7,11872017	5,29544523	10,499257	2,21626617

This journal is © The Royal Society of Chemistry 20xx

Please do not adjust margins

Please do not adjust margins

666,041748	575	11,3668556	3,56531351	7,80633207	3,7618043	8,97442879	3,47526865
667,85144	575	9,65940896	3,09199082	9,3740217	4,44919705	5,51557686	2,32321467
669,659424	575	7,74325738	3,58239192	6,10067549	3,48906825	8,92045624	2,03695084
671,46582	575	9,59207857	3,87681599	5,33143218	6,20913014	6,89130407	2,13613039
673,27063	575	8,9487783	2,14060367	7,88251139	4,09070788	7,3080703	1,36468212
675,073792	575	10,1354906	3,80201397	5,46013186	4,00099998	8,59131496	1,5626401
676,875305	575	10,62076	3,60635507	3,99683867	4,4880758	6,83270495	2,92939473
678,675171	575	8,86828214	3,89938184	5,94746111	5,07428327	7,90767222	1,17194411
680,47345	575	8,56937522	3,70158518	7,30687062	5,55794953	6,92612567	2,83003646
682,27002	575	6,64473647	2,73693538	6,84337841	7,33843358	7,83112881	2,5460665
684,065063	575	8,52961095	3,82483092	7,06229953	7,06831218	7,75894356	0,58950673
685,858398	575	7,74867405	2,84535175	4,9065245	6,38391232	7,46757211	1,37611866
687,650024	575	5,10545969	2,55353915	4,12557479	6,58687719	5,90130811	2,45979584
689,440186	575	8,06952655	2,55944154	6,30112134	6,70064131	6,80219103	-0,0986193
691,228577	575	5,4151499	3,05314304	6,40272452	5,1265405	6,01648945	0,98667776
693,015381	575	5,82365984	3,06085916	4,24635316	6,42437082	8,60258104	1,58267418
694,800476	575	7,99319701	4,54078558	2,66564879	6,42276616	8,99585449	1,18670915
696,583984	575	6,44018269	2,97333436	4,95631045	6,15105735	8,63514461	0,09929157
698,365845	575	5,66341383	3,37924767	5,26845139	2,38774497	4,47900437	3,08668762
700,146118	575	7,48162293	1,99573196	2,49504411	4,29512952	8,29427016	1,49952378
701,924683	575	8,71204723	1,4023831	4,60853051	3,50947593	5,41701776	2,20775662
347,741211	580	0	0	0	0	0	0
349,808533	580	0	0	0	0	0	0
351,874329	580	0	0	0	0	0	0
353,938568	580	0	0	0	0	0	0
356,001343	580	0	0	0	0	0	0
358,062561	580	0	0	0	0	0	0
360,122253	580	0	0	0	0	0	0
362,180481	580	0	0	0	0	0	0
364,237152	580	0	0	0	0	0	0
366,292328	580	0	0	0	0	0	0
368,345978	580	0	0	0	0	0	0
370,398132	580	0	0	0	0	0	0
372,44873	580	0	0	0	0	0	0
374,497803	580	0	0	0	0	0	0
376,545349	580	0	0	0	0	0	0
378,5914	580	0	0	0	0	0	0
380,635925	580	0	0	0	0	0	0
382,678955	580	0	0	0	0	0	0
384,720398	580	0	0	0	0	0	0
386,760376	580	0	0	0	0	0	0
388,798767	580	0	0	0	0	0	0
390,835663	580	0	0	0	0	0	0
392,871033	580	0	0	0	0	0	0
394,904877	580	0	0	0	0	0	0
396,937195	580	0	0	0	0	0	0
398,967957	580	0	0	0	0	0	0

This journal is © The Royal Society of Chemistry 20xx

Please do not adjust margins

Please do not adjust margins

400,997253	580	0	0	0	0	0	0
403,024963	580	0	0	0	0	0	0
405,051147	580	0	0	0	0	0	0
407,075806	580	0	0	0	0	0	0
409,098938	580	0	0	0	0	0	0
411,120544	580	0	0	0	0	0	0
413,140564	580	0	0	0	0	0	0
415,159119	580	0	0	0	0	0	0
417,176086	580	0	0	0	0	0	0
419,191528	580	0	0	0	0	0	0
421,205444	580	0	0	0	0	0	0
423,217834	580	0	0	0	0	0	0
425,228668	580	0	0	0	0	0	0
427,237976	580	0	0	0	0	0	0
429,245728	580	0	0	0	0	0	0
431,251953	580	0	0	0	0	0	0
433,256592	580	0	0	0	0	0	0
435,259766	580	0	0	0	0	0	0
437,261353	580	0	0	0	0	0	0
439,261383	580	0	0	0	0	0	0
441,259888	580	0	0	0	0	0	0
443,256836	580	0	0	0	0	0	0
445,252258	580	0	0	0	0	0	0
447,246094	580	0	0	0	0	0	0
449,238434	580	0	0	0	0	0	0
451,229187	580	0	0	0	0	0	0
453,218445	580	0	0	0	0	0	0
455,206116	580	0	0	0	0	0	0
457,192261	580	0	0	0	0	0	0
459,176819	580	0	0	0	0	0	0
461,15979	580	0	0	0	0	0	0
463,141296	580	0	0	0	0	0	0
465,121216	580	0	0	0	0	0	0
467,099579	580	0	0	0	0	0	0
469,076385	580	0	0	0	0	0	0
471,051636	580	0	0	0	0	0	0
473,02536	580	0	0	0	0	0	0
474,997498	580	0	0	0	0	0	0
476,968079	580	0	0	0	0	0	0
478,937134	580	0	0	0	0	0	0
480,904572	580	0	0	0	0	0	0
482,870453	580	0	0	0	0	0	0
484,834808	580	0	0	0	0	0	0
486,797607	580	0	0	0	0	0	0
488,75885	580	0	0	0	0	0	0
490,718506	580	0	0	0	0	0	0
492,676605	580	0	0	0	0	0	0

This journal is © The Royal Society of Chemistry 20xx

Please do not adjust margins

Please do not adjust margins

494,633118	580	0	0	0	0	0	0
496,588104	580	0	0	0	0	0	0
498,541504	580	0	0	0	0	0	0
500,493347	580	0	0	0	0	0	0
502,443665	580	0	0	0	0	0	0
504,392365	580	0	0	0	0	0	0
506,339539	580	0	0	0	0	0	0
508,285095	580	0	0	0	0	0	0
510,229126	580	0	0	0	0	0	0
512,17157	580	0	0	0	0	0	0
514,112427	580	0	0	0	0	0	0
516,051697	580	0	0	0	0	0	0
517,989441	580	0	0	0	0	0	0
519,925598	580	0	0	0	0	0	0
521,860168	580	0	0	0	0	0	0
523,793152	580	0	0	0	0	0	0
525,724609	580	0	0	0	0	0	0
527,654419	580	0	0	0	0	0	0
529,582764	580	0	0	0	0	0	0
531,50946	580	0	0	0	0	0	0
533,43457	580	0	0	0	0	0	0
535,358093	580	0	0	0	0	0	0
537,280029	580	0	0	0	0	0	0
539,200439	580	0	0	0	0	0	0
541,119263	580	0	0	0	0	0	0
543,036438	580	0	0	0	0	0	0
544,952087	580	0	0	0	0	0	0
546,866211	580	0	0	0	0	0	0
548,778687	580	0	0	0	0	0	0
550,689514	580	0	0	0	0	0	0
552,598816	580	0	0	0	0	0	0
554,506531	580	0	0	0	0	0	0
556,41272	580	0	0	0	0	0	0
558,317261	580	0	0	0	0	0	0
560,220215	580	0	0	0	0	0	0
562,121582	580	0	0	0	0	0	0
564,021362	580	0	0	0	0	0	0
565,919556	580	0	0	0	0	0	0
567,816162	580	0	0	0	0	0	0
569,711182	580	0	0	0	0	0	0
571,604614	580	0	0	0	0	0	0
573,49646	580	0	0	0	0	0	0
575,386719	580	0	0	0	0	0	0
577,27533	580	0	0	0	0	0	0
579,162354	580	0	0	0	0	0	0
581,047852	580	0	0	0	0	0	0
582,931641	580	0	0	0	0	0	0

This journal is © The Royal Society of Chemistry 20xx

Please do not adjust margins

Please do not adjust margins

584,813965	580	0	0	0	0	0	0
586,69458	580	0	0	0	0	0	0
588,57373	580	0	0	0	0	0	0
590,451111	580	11,8569605	7,90132146	9,43565044	44,9314692	20,1378529	6,29774926
592,326965	580	12,1686823	3,90323616	8,99408635	30,6160974	14,6328977	5,36634576
594,201233	580	9,70866411	5,03539934	5,95593666	18,3258776	12,8335661	5,32070877
596,073914	580	10,4250761	4,24205448	8,58354808	15,3211038	13,4809144	3,97335283
597,944946	580	9,30956631	3,77754416	8,5757898	13,3707957	11,6236982	5,16992196
599,814453	580	11,2185313	6,15840991	7,54350404	10,5792575	11,5040799	3,31563379
601,682251	580	12,4764527	5,50200633	8,25995783	11,9309972	10,2833814	2,38898515
603,548523	580	11,8089048	3,66013852	7,14327909	12,2717327	11,4523768	4,21760233
605,413147	580	11,9716499	5,38954797	9,41679336	14,6279636	12,6219805	2,74594511
607,276245	580	10,8584715	5,01651414	8,58089702	16,9791464	14,0639774	4,38683051
609,137695	580	12,0257445	6,64780879	9,75225216	17,4992907	18,9656216	5,6574095
610,997437	580	13,3275428	5,0185417	7,03187363	18,9037841	22,5662755	5,30289597
612,855713	580	11,9626306	7,02851767	7,85664179	21,2859233	25,8647055	6,58530325
614,712341	580	12,5442896	5,85764565	10,8091269	22,1677719	24,2849427	5,04401161
616,567322	580	11,6411264	6,32205096	9,07840559	24,3006747	20,5496773	3,76411622
618,420715	580	12,2090378	6,4231114	9,36727071	21,3058544	21,3149091	4,78102511
620,272522	580	12,6949598	5,42527644	9,38718805	20,6149096	20,9919505	5,43615885
622,122681	580	12,6301111	5,52911307	9,86855314	19,6447896	24,1742828	3,23178553
623,971313	580	12,2024663	5,82145916	10,1730101	17,3865219	18,5966813	4,90724161
625,818237	580	12,2506468	4,91675506	7,3349183	16,8052445	17,5554754	3,4393367
627,663574	580	11,1565281	6,87697044	11,3472534	18,2299327	13,8643589	4,09721147
629,507324	580	14,294635	5,97694462	8,87949667	17,4785105	13,0910771	4,11739185
631,349365	580	12,7157071	4,11217796	9,91495106	17,86554	13,1943021	4,86959491
633,18988	580	11,52709	5,99531717	9,75059267	18,1885248	14,4444496	4,69323673
635,028809	580	12,1758093	6,27259008	8,05816287	16,8658474	14,0608459	3,47091593
636,865967	580	12,5704656	5,15735319	10,2295415	17,7373831	11,9238359	5,35561447
638,70166	580	11,0756011	4,87872411	9,0144583	15,1179306	13,5273739	3,85440227
640,535645	580	11,3550982	4,78402365	8,73115254	15,8662175	12,5856609	2,34981362
642,368042	580	11,744612	4,88370724	9,02366568	15,2273682	10,6260733	3,76423333
644,198853	580	12,5232269	5,08248361	9,79672802	14,4124301	11,8741043	4,4325164
646,028015	580	10,9351636	4,14607916	7,54467237	12,5429621	12,4539446	4,15439566
647,855591	580	12,6264436	3,95588041	7,44708722	9,04958479	11,1281753	3,02004983
649,681519	580	11,936214	3,88237541	7,67651628	9,95099543	11,1877761	3,03622475
651,505798	580	10,1779406	5,32453614	7,4225621	10,8483118	10,8529222	3,81086891
653,328491	580	8,01478831	4,10108569	6,77726117	9,83175775	10,7908814	1,81574248
655,149536	580	9,57606342	4,7860214	5,93966302	10,538065	9,20076526	2,97328535
656,968994	580	7,78867013	4,42133825	7,79195461	11,3511921	9,71997997	1,63725037
658,786743	580	9,66176739	5,69804936	6,5727724	9,27916688	11,0236936	2,12895824
660,602905	580	8,0382808	4,25946815	6,3945814	9,78560383	7,75426738	2,03700577
662,41748	580	9,04720845	4,08412059	7,29921263	8,17508183	8,17855613	3,60513271
664,230469	580	10,7221146	2,53325394	6,82604117	5,85086645	7,90202653	2,53833532
666,041748	580	9,66751279	2,4402661	4,59156273	3,81000322	7,91644655	2,64077384
667,85144	580	10,1836888	3,91515885	6,95525777	4,70212522	7,05618533	1,27497895
669,659424	580	8,53638876	4,21736322	5,39884334	2,74848986	6,48133654	1,96549894

This journal is © The Royal Society of Chemistry 20xx

Please do not adjust margins

Please do not adjust margins

671,46582	580	9,62553402	2,6508194	5,20784117	5,0112966	5,99645111	2,85288746
673,27063	580	8,18418125	2,8583326	7,00387842	4,34041242	7,40157449	2,46902245
675,073792	580	8,69391388	2,56757789	5,33715143	2,86623526	5,24051822	0,69265757
676,875305	580	7,21062949	1,28354557	4,54560041	3,65622757	6,52469117	3,56156361
678,675171	580	7,80452408	3,85124865	5,04048832	4,15097195	7,61334943	0,49474023
680,47345	580	7,10755466	2,86156311	7,40682491	3,45650294	6,02675104	1,48308778
682,27002	580	6,5378358	2,27738013	5,35139411	3,96397804	6,54334341	2,57959369
684,065063	580	7,15589058	3,07971485	3,57945411	5,0708708	7,95768761	1,09499406
685,858398	580	8,05390578	3,67739997	4,37680609	4,27731423	7,46360237	1,1950626
687,650024	580	6,27037164	2,58668592	5,27729905	7,16912914	6,7737217	1,69468716
689,440186	580	7,38222277	3,78927951	4,79048812	5,68867938	6,58969125	0,99917903
691,228577	580	5,78891285	3,69137665	4,79284472	5,29207587	5,5940037	1,79940701
693,015381	580	7,50458147	3,00057223	4,30444113	5,80596457	7,11032196	1,4030758
694,800476	580	6,30227388	3,19981094	4,00313114	4,1031912	5,50662012	0,40077867
696,583984	580	5,12243235	2,50994198	3,01446616	6,22986907	5,42832098	0,30179719
698,365845	580	7,05054528	3,6244723	4,43364019	4,1313282	5,14115321	4,43879642
700,146118	580	8,08994864	2,83029319	2,63034206	5,46299386	6,17378238	0,40513863
701,924683	580	6,19230056	1,92793955	4,16379322	4,06221924	7,31510213	3,25356926
347,741211	585	0	0	0	0	0	0
349,808533	585	0	0	0	0	0	0
351,874329	585	0	0	0	0	0	0
353,938568	585	0	0	0	0	0	0
356,001343	585	0	0	0	0	0	0
358,062561	585	0	0	0	0	0	0
360,122253	585	0	0	0	0	0	0
362,180481	585	0	0	0	0	0	0
364,237152	585	0	0	0	0	0	0
366,292328	585	0	0	0	0	0	0
368,345978	585	0	0	0	0	0	0
370,398132	585	0	0	0	0	0	0
372,44873	585	0	0	0	0	0	0
374,497803	585	0	0	0	0	0	0
376,545349	585	0	0	0	0	0	0
378,5914	585	0	0	0	0	0	0
380,635925	585	0	0	0	0	0	0
382,678955	585	0	0	0	0	0	0
384,720398	585	0	0	0	0	0	0
386,760376	585	0	0	0	0	0	0
388,798767	585	0	0	0	0	0	0
390,835663	585	0	0	0	0	0	0
392,871033	585	0	0	0	0	0	0
394,904877	585	0	0	0	0	0	0
396,937195	585	0	0	0	0	0	0
398,967957	585	0	0	0	0	0	0
400,997253	585	0	0	0	0	0	0
403,024963	585	0	0	0	0	0	0
405,051147	585	0	0	0	0	0	0

Please do not adjust margins

Please do not adjust margins

407,075806	585	0	0	0	0	0	0
409,098938	585	0	0	0	0	0	0
411,120544	585	0	0	0	0	0	0
413,140564	585	0	0	0	0	0	0
415,159119	585	0	0	0	0	0	0
417,176086	585	0	0	0	0	0	0
419,191528	585	0	0	0	0	0	0
421,205444	585	0	0	0	0	0	0
423,217834	585	0	0	0	0	0	0
425,228668	585	0	0	0	0	0	0
427,237976	585	0	0	0	0	0	0
429,245728	585	0	0	0	0	0	0
431,251953	585	0	0	0	0	0	0
433,256592	585	0	0	0	0	0	0
435,259766	585	0	0	0	0	0	0
437,261353	585	0	0	0	0	0	0
439,261383	585	0	0	0	0	0	0
441,259888	585	0	0	0	0	0	0
443,256836	585	0	0	0	0	0	0
445,252258	585	0	0	0	0	0	0
447,246094	585	0	0	0	0	0	0
449,238434	585	0	0	0	0	0	0
451,229187	585	0	0	0	0	0	0
453,218445	585	0	0	0	0	0	0
455,206116	585	0	0	0	0	0	0
457,192261	585	0	0	0	0	0	0
459,176819	585	0	0	0	0	0	0
461,15979	585	0	0	0	0	0	0
463,141296	585	0	0	0	0	0	0
465,121216	585	0	0	0	0	0	0
467,099579	585	0	0	0	0	0	0
469,076385	585	0	0	0	0	0	0
471,051636	585	0	0	0	0	0	0
473,02536	585	0	0	0	0	0	0
474,997498	585	0	0	0	0	0	0
476,968079	585	0	0	0	0	0	0
478,937134	585	0	0	0	0	0	0
480,904572	585	0	0	0	0	0	0
482,870453	585	0	0	0	0	0	0
484,834808	585	0	0	0	0	0	0
486,797607	585	0	0	0	0	0	0
488,75885	585	0	0	0	0	0	0
490,718506	585	0	0	0	0	0	0
492,676605	585	0	0	0	0	0	0
494,633118	585	0	0	0	0	0	0
496,588104	585	0	0	0	0	0	0
498,541504	585	0	0	0	0	0	0

This journal is © The Royal Society of Chemistry 20xx

Please do not adjust margins

Please do not adjust margins

500,493347	585	0	0	0	0	0	0
502,443665	585	0	0	0	0	0	0
504,392365	585	0	0	0	0	0	0
506,339539	585	0	0	0	0	0	0
508,285095	585	0	0	0	0	0	0
510,229126	585	0	0	0	0	0	0
512,17157	585	0	0	0	0	0	0
514,112427	585	0	0	0	0	0	0
516,051697	585	0	0	0	0	0	0
517,989441	585	0	0	0	0	0	0
519,925598	585	0	0	0	0	0	0
521,860168	585	0	0	0	0	0	0
523,793152	585	0	0	0	0	0	0
525,724609	585	0	0	0	0	0	0
527,654419	585	0	0	0	0	0	0
529,582764	585	0	0	0	0	0	0
531,50946	585	0	0	0	0	0	0
533,43457	585	0	0	0	0	0	0
535,358093	585	0	0	0	0	0	0
537,280029	585	0	0	0	0	0	0
539,200439	585	0	0	0	0	0	0
541,119263	585	0	0	0	0	0	0
543,036438	585	0	0	0	0	0	0
544,952087	585	0	0	0	0	0	0
546,866211	585	0	0	0	0	0	0
548,778687	585	0	0	0	0	0	0
550,689514	585	0	0	0	0	0	0
552,598816	585	0	0	0	0	0	0
554,506531	585	0	0	0	0	0	0
556,41272	585	0	0	0	0	0	0
558,317261	585	0	0	0	0	0	0
560,220215	585	0	0	0	0	0	0
562,121582	585	0	0	0	0	0	0
564,021362	585	0	0	0	0	0	0
565,919556	585	0	0	0	0	0	0
567,816162	585	0	0	0	0	0	0
569,711182	585	0	0	0	0	0	0
571,604614	585	0	0	0	0	0	0
573,49646	585	0	0	0	0	0	0
575,386719	585	0	0	0	0	0	0
577,27533	585	0	0	0	0	0	0
579,162354	585	0	0	0	0	0	0
581,047852	585	0	0	0	0	0	0
582,931641	585	0	0	0	0	0	0
584,813965	585	0	0	0	0	0	0
586,69458	585	0	0	0	0	0	0
588,57373	585	0	0	0	0	0	0

Please do not adjust margins

590,451111	585	0	0	0	0	0	0
592,326965	585	0	0	0	0	0	0
594,201233	585	0	0	0	0	0	0
596,073914	585	9,52284412	5,33070616	9,07047836	36,1289048	17,5037546	6,08214891
597,944946	585	8,95457587	4,67183156	8,6885777	26,4643488	14,3083094	6,35711237
599,814453	585	8,09582545	5,87253775	7,26990004	16,4193341	11,0089778	3,73060324
601,682251	585	10,1191592	3,71981469	8,46157629	13,588508	9,49382464	4,37313591
603,548523	585	9,26372435	4,54700141	6,86608692	11,4233037	11,052377	2,22830202
605,413147	585	10,3577744	3,52026539	6,2986763	11,2184998	9,64283795	2,31721121
607,276245	585	11,450096	3,42241408	6,38158398	10,8311431	7,96175852	3,79444699
609,137695	585	10,4179483	4,24820014	7,20261679	11,368675	12,5708825	4,80489924
610,997437	585	10,5309559	5,45956872	6,29163916	12,2246902	11,1139207	4,90699933
612,855713	585	12,0133592	4,53586974	7,40462468	14,3599787	13,4342126	4,07521921
614,712341	585	11,2118321	6,21884028	8,44548435	18,9505741	16,7219464	6,22218911
616,567322	585	9,46143841	3,15921583	8,08291852	20,7378079	22,87785	3,90466225
618,420715	585	12,0764381	5,58328945	7,62959199	19,9301363	24,4016899	3,72419736
620,272522	585	11,8228363	2,89083319	8,57823096	21,9324327	21,6535469	4,5718421
622,122681	585	11,6617124	4,67267638	8,03605165	20,9508343	18,8941252	4,20767334
623,971313	585	11,1323374	5,3414312	9,18242581	18,9448756	20,7278749	3,65663148
625,818237	585	8,45265818	5,17436275	7,99579547	19,4904619	20,0564242	4,61236923
627,663574	585	12,0426898	4,2410113	9,23488646	21,1282019	20,6576422	4,62047686
629,507324	585	10,0219727	6,44020436	7,5758138	20,3791844	19,1479298	3,41135598
631,349365	585	11,8270745	4,92849189	7,77093227	17,1690744	13,9446545	5,12080533
633,18988	585	9,29407898	5,89000085	7,31413483	17,6845583	13,1214695	3,23173982
635,028809	585	11,6580338	5,50670202	8,44893868	18,7192055	12,8285352	2,94482221
636,865967	585	11,2019669	5,89586258	8,27224682	17,3214664	11,5165064	4,28155947
638,70166	585	7,31377734	2,47381798	7,23030395	17,9023419	13,0465199	2,95114054
640,535645	585	9,59163424	3,8051748	7,98992081	14,8524063	12,3776356	2,66505671
642,368042	585	9,60319496	4,38122531	7,8090855	18,0159499	10,2000254	2,57297357
644,198853	585	11,1484934	5,15427356	7,92137122	16,048727	8,59797019	2,76952638
646,028015	585	9,63505904	4,30020248	7,3572529	14,728371	11,0946626	4,20690661
647,855591	585	10,2029495	2,57898672	5,4438828	13,4791361	12,3326036	3,53607013
649,681519	585	9,87413338	2,40074016	6,72127644	12,0135889	10,476383	3,07460218
651,505798	585	8,37454263	4,62835292	6,45964426	12,8349587	10,5194116	4,82379717
653,328491	585	7,43479857	3,67540721	5,51245796	10,7449017	11,5198957	1,35482657
655,149536	585	9,78755237	3,78582659	5,14423202	9,32663426	10,8816274	1,84537025
656,968994	585	6,42226082	3,89894743	6,14011502	9,56029003	7,21936216	1,9505235
658,786743	585	9,28850661	3,81970903	5,28820159	10,5863691	10,5869083	1,95987996
660,602905	585	9,50654103	4,81050042	6,47868227	10,6114697	10,317232	2,94679033
662,41748	585	9,54924627	4,83211014	4,43712667	9,37609402	9,37657151	2,7626927
664,230469	585	10,2585696	2,17379976	5,82904538	8,20790937	9,88955105	3,36131781
666,041748	585	9,48672924	3,06867115	6,13661554	5,54797235	7,82700244	3,16936633
667,85144	585	9,31462432	4,56602943	6,64973358	6,35796968	6,25894514	1,2910945
669,659424	585	6,15619452	3,38176127	5,46984837	4,67863851	6,66988817	0,89565415
671,46582	585	9,04494356	4,77913272	4,18124593	4,48412769	4,98261784	1,69352097
673,27063	585	7,78318692	2,89870647	6,49633134	5,90222631	6,30269821	2,90026742
675,073792	585	7,49824517	2,30340123	6,00816121	1,30299489	5,11200953	-0,6012109

This journal is © The Royal Society of Chemistry 20xx

676,875305	585	5,99745893	2,20283566	4,50526664	4,10866637	6,0129887	3,90712969
678,675171	585	6,49827972	3,10448899	4,40584967	3,70841012	6,91603594	2,00397468
680,47345	585	7,09270296	3,40232444	6,60373006	4,50678589	4,00623592	1,9023228
682,27002	585	5,71387376	2,30954807	4,51814591	2,61294412	5,82917219	1,30609969
684,065063	585	6,53747834	2,11572679	5,64127001	3,32745227	6,55440644	0,80642518
685,858398	585	8,45213692	3,83013621	4,938275	3,42978695	5,0440612	1,61355736
687,650024	585	5,43891415	1,9169705	3,0264371	4,54393328	5,3520162	0,20189503
689,440186	585	4,64385841	2,62928626	5,35906422	4,95927518	3,94737922	0,70826596
691,228577	585	5,75717705	1,41646599	4,85587972	6,4806071	4,253115	0,809845
693,015381	585	5,06291842	2,13006867	2,43407592	4,46667743	5,98971339	1,11635108
694,800476	585	6,98508226	2,63656916	2,63625694	4,16109163	6,59718855	0,50730557
696,583984	585	5,08208321	1,42542111	3,35952332	2,75129095	4,89143301	0,91683559
698,365845	585	5,91178146	2,85885279	5,41075912	3,67869572	4,49641263	2,45176481
700,146118	585	7,36807411	1,33262583	2,97242866	3,7959825	7,18195426	0,61538928
701,924683	585	5,54730014	2,98420935	3,70409707	3,29563322	5,35567671	1,85326532
347,741211	590	0	0	0	0	0	0
349,808533	590	0	0	0	0	0	0
351,874329	590	0	0	0	0	0	0
353,938568	590	0	0	0	0	0	0
356,001343	590	0	0	0	0	0	0
358,062561	590	0	0	0	0	0	0
360,122253	590	0	0	0	0	0	0
362,180481	590	0	0	0	0	0	0
364,237152	590	0	0	0	0	0	0
366,292328	590	0	0	0	0	0	0
368,345978	590	0	0	0	0	0	0
370,398132	590	0	0	0	0	0	0
372,44873	590	0	0	0	0	0	0
374,497803	590	0	0	0	0	0	0
376,545349	590	0	0	0	0	0	0
378,5914	590	0	0	0	0	0	0
380,635925	590	0	0	0	0	0	0
382,678955	590	0	0	0	0	0	0
384,720398	590	0	0	0	0	0	0
386,760376	590	0	0	0	0	0	0
388,798767	590	0	0	0	0	0	0
390,835663	590	0	0	0	0	0	0
392,871033	590	0	0	0	0	0	0
394,904877	590	0	0	0	0	0	0
396,937195	590	0	0	0	0	0	0
398,967957	590	0	0	0	0	0	0
400,997253	590	0	0	0	0	0	0
403,024963	590	0	0	0	0	0	0
405,051147	590	0	0	0	0	0	0
407,075806	590	0	0	0	0	0	0
409,098938	590	0	0	0	0	0	0
411,120544	590	0	0	0	0	0	0

This journal is © The Royal Society of Chemistry 20xx

Please do not adjust margins

413,140564	590	0	0	0	0	0	0
415,159119	590	0	0	0	0	0	0
417,176086	590	0	0	0	0	0	0
419,191528	590	0	0	0	0	0	0
421,205444	590	0	0	0	0	0	0
423,217834	590	0	0	0	0	0	0
425,228668	590	0	0	0	0	0	0
427,237976	590	0	0	0	0	0	0
429,245728	590	0	0	0	0	0	0
431,251953	590	0	0	0	0	0	0
433,256592	590	0	0	0	0	0	0
435,259766	590	0	0	0	0	0	0
437,261353	590	0	0	0	0	0	0
439,261383	590	0	0	0	0	0	0
441,259888	590	0	0	0	0	0	0
443,256836	590	0	0	0	0	0	0
445,252258	590	0	0	0	0	0	0
447,246094	590	0	0	0	0	0	0
449,238434	590	0	0	0	0	0	0
451,229187	590	0	0	0	0	0	0
453,218445	590	0	0	0	0	0	0
455,206116	590	0	0	0	0	0	0
457,192261	590	0	0	0	0	0	0
459,176819	590	0	0	0	0	0	0
461,15979	590	0	0	0	0	0	0
463,141296	590	0	0	0	0	0	0
465,121216	590	0	0	0	0	0	0
467,099579	590	0	0	0	0	0	0
469,076385	590	0	0	0	0	0	0
471,051636	590	0	0	0	0	0	0
473,02536	590	0	0	0	0	0	0
474,997498	590	0	0	0	0	0	0
476,968079	590	0	0	0	0	0	0
478,937134	590	0	0	0	0	0	0
480,904572	590	0	0	0	0	0	0
482,870453	590	0	0	0	0	0	0
484,834808	590	0	0	0	0	0	0
486,797607	590	0	0	0	0	0	0
488,75885	590	0	0	0	0	0	0
490,718506	590	0	0	0	0	0	0
492,676605	590	0	0	0	0	0	0
494,633118	590	0	0	0	0	0	0
496,588104	590	0	0	0	0	0	0
498,541504	590	0	0	0	0	0	0
500,493347	590	0	0	0	0	0	0
502,443665	590	0	0	0	0	0	0
504,392365	590	0	0	0	0	0	0

This journal is © The Royal Society of Chemistry 20xx

Please do not adjust margins

Please do not adjust margins

506,339539	590	0	0	0	0	0	0
508,285095	590	0	0	0	0	0	0
510,229126	590	0	0	0	0	0	0
512,17157	590	0	0	0	0	0	0
514,112427	590	0	0	0	0	0	0
516,051697	590	0	0	0	0	0	0
517,989441	590	0	0	0	0	0	0
519,925598	590	0	0	0	0	0	0
521,860168	590	0	0	0	0	0	0
523,793152	590	0	0	0	0	0	0
525,724609	590	0	0	0	0	0	0
527,654419	590	0	0	0	0	0	0
529,582764	590	0	0	0	0	0	0
531,50946	590	0	0	0	0	0	0
533,43457	590	0	0	0	0	0	0
535,358093	590	0	0	0	0	0	0
537,280029	590	0	0	0	0	0	0
539,200439	590	0	0	0	0	0	0
541,119263	590	0	0	0	0	0	0
543,036438	590	0	0	0	0	0	0
544,952087	590	0	0	0	0	0	0
546,866211	590	0	0	0	0	0	0
548,778687	590	0	0	0	0	0	0
550,689514	590	0	0	0	0	0	0
552,598816	590	0	0	0	0	0	0
554,506531	590	0	0	0	0	0	0
556,41272	590	0	0	0	0	0	0
558,317261	590	0	0	0	0	0	0
560,220215	590	0	0	0	0	0	0
562,121582	590	0	0	0	0	0	0
564,021362	590	0	0	0	0	0	0
565,919556	590	0	0	0	0	0	0
567,816162	590	0	0	0	0	0	0
569,711182	590	0	0	0	0	0	0
571,604614	590	0	0	0	0	0	0
573,49646	590	0	0	0	0	0	0
575,386719	590	0	0	0	0	0	0
577,27533	590	0	0	0	0	0	0
579,162354	590	0	0	0	0	0	0
581,047852	590	0	0	0	0	0	0
582,931641	590	0	0	0	0	0	0
584,813965	590	0	0	0	0	0	0
586,69458	590	0	0	0	0	0	0
588,57373	590	0	0	0	0	0	0
590,451111	590	0	0	0	0	0	0
592,326965	590	0	0	0	0	0	0
594,201233	590	0	0	0	0	0	0

This journal is © The Royal Society of Chemistry 20xx

Please do not adjust margins

Please do not adjust margins

596,073914	590	0	0	0	0	0	0
597,944946	590	0	0	0	0	0	0
599,814453	590	0	0	0	0	0	0
601,682251	590	8,42210667	6,34191427	8,70606139	31,4536835	10,8045353	7,10888518
603,548523	590	8,87618367	3,87255073	3,6826988	20,70357	10,9705127	4,35076637
605,413147	590	8,38979276	4,80890542	8,29558591	17,7427843	10,4798415	3,02148686
607,276245	590	8,94177737	3,38935907	5,74160417	14,9830445	9,23840148	5,46811633
609,137695	590	8,92771177	3,66602984	5,92052571	9,31439063	10,4474438	3,20040527
610,997437	590	8,66290538	3,29654205	4,80229778	8,20158317	11,411208	3,58400696
612,855713	590	7,34731255	3,86307021	6,59379011	11,5052601	9,245502	3,86836347
614,712341	590	9,72839605	6,42432885	6,89493292	11,3471805	9,45963822	3,87879994
616,567322	590	10,2115943	4,35053295	7,37509412	16,66039	10,416768	3,3147238
618,420715	590	8,90094595	6,06182113	8,04878641	15,9264979	12,9926993	3,41444653
620,272522	590	7,87605698	3,98652856	9,01482974	18,2404137	15,6814136	4,84741763
622,122681	590	10,8410156	3,61462748	7,51268838	17,0420112	24,096621	2,38130284
623,971313	590	8,86819328	3,71990322	8,77290033	18,9025367	23,6850547	4,48910294
625,818237	590	9,09467545	3,92610843	7,18005929	19,8397704	18,8886246	4,31504788
627,663574	590	8,15164502	5,75562403	7,76809484	20,5467343	16,3284802	4,89898393
629,507324	590	9,05916162	4,14518046	7,90273052	21,2268491	16,5054274	5,69536642
631,349365	590	7,42627189	4,9200003	8,77656714	19,8906987	19,4154154	2,80148063
633,18988	590	7,34694313	4,06122648	7,83035175	17,3240255	19,3639339	4,64776144
635,028809	590	8,59850617	3,86551911	6,27985367	18,861243	20,1264345	2,51603022
636,865967	590	9,87018408	4,45243661	7,7413773	17,6319021	12,8898312	4,16776325
638,70166	590	7,45511826	2,42113303	8,42337686	18,9017111	12,0241949	3,87912083
640,535645	590	9,38978768	4,26041149	8,42182974	21,0303485	11,9250349	3,29664711
642,368042	590	9,01343068	2,7144387	8,52889982	19,6972642	10,4833827	3,10646638
644,198853	590	9,51851499	3,49752201	7,77027298	20,3232007	9,82505535	3,11316835
646,028015	590	7,97369577	4,66876355	7,77927211	17,0367423	10,3233864	3,9933665
647,855591	590	8,06738628	3,59725576	6,41507683	17,7104194	12,1684506	3,79689749
649,681519	590	8,50146595	4,00749747	6,64487241	16,4356372	12,1357539	3,22996645
651,505798	590	7,75142839	3,92581176	5,10224323	16,3066757	10,3184498	3,43979212
653,328491	590	8,46427442	4,7254955	5,51166074	13,0067236	8,67450028	3,35181242
655,149536	590	7,90233642	3,6557971	5,13655613	12,2628075	8,31027361	3,36398422
656,968994	590	6,84393533	4,36540273	5,9512915	11,1218626	6,95385059	2,88113965
658,786743	590	8,47137715	3,48913657	4,68420741	10,7760983	9,48263875	0,79860971
660,602905	590	7,29266821	3,29755743	4,59541348	9,0013739	6,90372041	3,80239033
662,41748	590	9,63343997	4,01499479	5,21815136	9,94599083	8,54278737	2,31178533
664,230469	590	9,55190247	3,62062551	6,0328245	7,24772102	9,0631526	2,1149255
666,041748	590	7,95765609	2,21664216	4,93579736	7,5634865	9,78588974	3,32951917
667,85144	590	6,06040354	3,33410341	5,55541042	9,91012347	5,15930233	2,83281249
669,659424	590	6,07274014	2,32849932	4,45337524	5,47179608	6,18349183	1,62204513
671,46582	590	5,77500184	3,95236197	6,07899364	7,30318775	5,58098005	3,24740724
673,27063	590	6,71304558	1,93305448	6,10281321	4,68420819	7,23276772	1,63006585
675,073792	590	6,11452079	1,52903444	3,9744675	3,67296148	6,3281006	0,81660243
676,875305	590	7,23414456	1,63066087	2,44536247	2,55018414	6,22485413	2,3472869
678,675171	590	5,70671094	2,85411003	3,87243923	3,57082519	4,38870927	-0,8165774
680,47345	590	5,8041828	3,15749559	5,6005681	1,32529413	7,44491246	1,32592538

This journal is © The Royal Society of Chemistry 20xx

Please do not adjust margins

Please do not adjust margins

682,27002	590	4,08722531	0,91986889	3,88289236	3,3758662	6,9590188	1,33052012
684,065063	590	5,84365496	2,25604222	4,81848747	3,38708696	6,0580413	-0,1026879
685,858398	590	8,20521617	3,07776975	3,38467635	0,92415591	4,31439436	1,13006189
687,650024	590	5,03068734	1,95119053	4,72272051	4,0086538	4,21585449	1,13118448
689,440186	590	6,07135955	1,44104257	3,91039568	4,53303753	4,94704303	2,26759832
691,228577	590	6,48616633	1,33876922	4,73596551	3,09222783	3,91833567	0,51561678
693,015381	590	3,81896301	1,03242511	1,44502357	3,82338603	5,89235755	0,31015193
694,800476	590	5,57222952	3,09650171	4,74674865	3,61581314	5,16744366	1,5503723
696,583984	590	3,7298131	2,38356631	4,86951373	4,97884383	4,04687403	0,51887659
698,365845	590	5,09095502	1,03924517	4,15591195	2,70445167	3,53795746	1,56100374
700,146118	590	5,42422424	0,41735836	2,19056807	4,69947673	7,2086492	1,04482558
701,924683	590	6,5969206	0,6284443	2,19898957	3,04019474	4,82424159	2,20256892
347,741211	595	0	0	0	0	0	0
349,808533	595	0	0	0	0	0	0
351,874329	595	0	0	0	0	0	0
353,938568	595	0	0	0	0	0	0
356,001343	595	0	0	0	0	0	0
358,062561	595	0	0	0	0	0	0
360,122253	595	0	0	0	0	0	0
362,180481	595	0	0	0	0	0	0
364,237152	595	0	0	0	0	0	0
366,292328	595	0	0	0	0	0	0
368,345978	595	0	0	0	0	0	0
370,398132	595	0	0	0	0	0	0
372,44873	595	0	0	0	0	0	0
374,497803	595	0	0	0	0	0	0
376,545349	595	0	0	0	0	0	0
378,5914	595	0	0	0	0	0	0
380,635925	595	0	0	0	0	0	0
382,678955	595	0	0	0	0	0	0
384,720398	595	0	0	0	0	0	0
386,760376	595	0	0	0	0	0	0
388,798767	595	0	0	0	0	0	0
390,835663	595	0	0	0	0	0	0
392,871033	595	0	0	0	0	0	0
394,904877	595	0	0	0	0	0	0
396,937195	595	0	0	0	0	0	0
398,967957	595	0	0	0	0	0	0
400,997253	595	0	0	0	0	0	0
403,024963	595	0	0	0	0	0	0
405,051147	595	0	0	0	0	0	0
407,075806	595	0	0	0	0	0	0
409,098938	595	0	0	0	0	0	0
411,120544	595	0	0	0	0	0	0
413,140564	595	0	0	0	0	0	0
415,159119	595	0	0	0	0	0	0
417,176086	595	0	0	0	0	0	0

This journal is © The Royal Society of Chemistry 20xx

Please do not adjust margins

Please do not adjust margins

419,191528	595	0	0	0	0	0	0
421,205444	595	0	0	0	0	0	0
423,217834	595	0	0	0	0	0	0
425,228668	595	0	0	0	0	0	0
427,237976	595	0	0	0	0	0	0
429,245728	595	0	0	0	0	0	0
431,251953	595	0	0	0	0	0	0
433,256592	595	0	0	0	0	0	0
435,259766	595	0	0	0	0	0	0
437,261353	595	0	0	0	0	0	0
439,261383	595	0	0	0	0	0	0
441,259888	595	0	0	0	0	0	0
443,256836	595	0	0	0	0	0	0
445,252258	595	0	0	0	0	0	0
447,246094	595	0	0	0	0	0	0
449,238434	595	0	0	0	0	0	0
451,229187	595	0	0	0	0	0	0
453,218445	595	0	0	0	0	0	0
455,206116	595	0	0	0	0	0	0
457,192261	595	0	0	0	0	0	0
459,176819	595	0	0	0	0	0	0
461,15979	595	0	0	0	0	0	0
463,141296	595	0	0	0	0	0	0
465,121216	595	0	0	0	0	0	0
467,099579	595	0	0	0	0	0	0
469,076385	595	0	0	0	0	0	0
471,051636	595	0	0	0	0	0	0
473,02536	595	0	0	0	0	0	0
474,997498	595	0	0	0	0	0	0
476,968079	595	0	0	0	0	0	0
478,937134	595	0	0	0	0	0	0
480,904572	595	0	0	0	0	0	0
482,870453	595	0	0	0	0	0	0
484,834808	595	0	0	0	0	0	0
486,797607	595	0	0	0	0	0	0
488,75885	595	0	0	0	0	0	0
490,718506	595	0	0	0	0	0	0
492,676605	595	0	0	0	0	0	0
494,633118	595	0	0	0	0	0	0
496,588104	595	0	0	0	0	0	0
498,541504	595	0	0	0	0	0	0
500,493347	595	0	0	0	0	0	0
502,443665	595	0	0	0	0	0	0
504,392365	595	0	0	0	0	0	0
506,339539	595	0	0	0	0	0	0
508,285095	595	0	0	0	0	0	0
510,229126	595	0	0	0	0	0	0

This journal is © The Royal Society of Chemistry 20xx

Please do not adjust margins

Please do not adjust margins

512,17157	595	0	0	0	0	0	0
514,112427	595	0	0	0	0	0	0
516,051697	595	0	0	0	0	0	0
517,989441	595	0	0	0	0	0	0
519,925598	595	0	0	0	0	0	0
521,860168	595	0	0	0	0	0	0
523,793152	595	0	0	0	0	0	0
525,724609	595	0	0	0	0	0	0
527,654419	595	0	0	0	0	0	0
529,582764	595	0	0	0	0	0	0
531,50946	595	0	0	0	0	0	0
533,43457	595	0	0	0	0	0	0
535,358093	595	0	0	0	0	0	0
537,280029	595	0	0	0	0	0	0
539,200439	595	0	0	0	0	0	0
541,119263	595	0	0	0	0	0	0
543,036438	595	0	0	0	0	0	0
544,952087	595	0	0	0	0	0	0
546,866211	595	0	0	0	0	0	0
548,778687	595	0	0	0	0	0	0
550,689514	595	0	0	0	0	0	0
552,598816	595	0	0	0	0	0	0
554,506531	595	0	0	0	0	0	0
556,41272	595	0	0	0	0	0	0
558,317261	595	0	0	0	0	0	0
560,220215	595	0	0	0	0	0	0
562,121582	595	0	0	0	0	0	0
564,021362	595	0	0	0	0	0	0
565,919556	595	0	0	0	0	0	0
567,816162	595	0	0	0	0	0	0
569,711182	595	0	0	0	0	0	0
571,604614	595	0	0	0	0	0	0
573,49646	595	0	0	0	0	0	0
575,386719	595	0	0	0	0	0	0
577,27533	595	0	0	0	0	0	0
579,162354	595	0	0	0	0	0	0
581,047852	595	0	0	0	0	0	0
582,931641	595	0	0	0	0	0	0
584,813965	595	0	0	0	0	0	0
586,69458	595	0	0	0	0	0	0
588,57373	595	0	0	0	0	0	0
590,451111	595	0	0	0	0	0	0
592,326965	595	0	0	0	0	0	0
594,201233	595	0	0	0	0	0	0
596,073914	595	0	0	0	0	0	0
597,944946	595	0	0	0	0	0	0
599,814453	595	0	0	0	0	0	0

This journal is © The Royal Society of Chemistry 20xx

Please do not adjust margins

Please do not adjust margins

601,682251	595	0	0	0	0	0	0
603,548523	595	0	0	0	0	0	0
605,413147	595	8,30844651	4,7319148	8,60280783	38,7824997	16,7292211	7,55613851
607,276245	595	9,16397726	3,76049023	7,62458705	30,4187245	11,4898828	7,83482584
609,137695	595	6,35653791	4,42847296	7,90167925	21,018574	11,1826037	4,53898235
610,997437	595	6,85163536	1,83277416	4,82763844	11,4961627	9,8524515	4,93503231
612,855713	595	8,8814094	4,63185291	6,18162812	12,3701248	10,2425518	3,67842232
614,712341	595	8,03419301	2,41893187	6,29515407	10,8530609	7,26664045	3,49422285
616,567322	595	9,39943355	3,9713052	4,16895533	11,4467449	9,69925078	3,69230421
618,420715	595	8,9280433	4,94718438	3,98089897	9,61775486	10,6848735	4,08697218
620,272522	595	8,1690244	3,6939751	5,15698199	12,0720949	9,05278271	3,02298875
622,122681	595	9,06375621	3,79935027	6,53324445	14,3420968	9,56003723	2,54087059
623,971313	595	8,11129962	3,7120636	8,31113077	14,5770173	13,4989404	3,03779159
625,818237	595	5,88674198	3,53057719	6,38066502	15,4203223	16,203765	4,3287286
627,663574	595	10,0250525	5,01044293	6,58855423	20,3669913	22,3316381	5,2233158
629,507324	595	9,38304738	3,45547542	6,91746838	18,7864168	19,5746378	3,86250311
631,349365	595	8,89575432	5,23643359	8,60376147	18,305519	15,6316779	4,65824213
633,18988	595	7,6285845	3,76319127	7,33523098	19,6376029	17,3539973	4,5697769
635,028809	595	8,91118911	4,25579859	8,32149344	19,824087	18,6319899	4,76560803
636,865967	595	9,42125668	4,46084573	6,1518503	19,8557033	18,661705	3,57990632
638,70166	595	7,14424696	3,27308559	8,53790369	20,0652872	17,2815006	3,48237821
640,535645	595	7,53976449	3,6691492	8,43707583	17,77736	14,7958083	1,98956492
642,368042	595	7,64817911	4,17000019	6,95655468	22,0744732	10,339723	3,28673883
644,198853	595	8,36146852	4,37799649	8,06709424	22,3213953	10,4616651	3,49345576
646,028015	595	7,27493001	5,17998811	6,48109152	21,4493664	10,1745303	5,4960741
647,855591	595	8,56669611	3,28585436	7,87355453	19,545241	11,0674133	4,89434885
649,681519	595	8,31213924	2,60272045	7,51492792	19,9506864	9,22212034	3,81594621
651,505798	595	6,8379152	3,71908395	4,82930766	19,2272961	9,96456245	3,93245114
653,328491	595	5,44684722	6,0495371	5,65155944	17,7719118	9,28854099	3,94457277
655,149536	595	7,18760209	3,03575272	3,44376441	16,6203274	8,00499398	3,85738693
656,968994	595	6,30243893	3,04829975	4,98358505	16,9943076	8,64856637	2,54824333
658,786743	595	8,06904864	4,4922858	6,23381508	14,6218164	9,30346814	2,86772303
660,602905	595	5,01671957	2,45614752	3,99500374	12,0941625	8,19826539	2,4638764
662,41748	595	8,43304009	1,95318497	3,91005393	7,51559244	6,48513621	3,29992616
664,230469	595	7,62532123	2,98705956	4,63946365	6,91148349	8,25134321	4,6496779
666,041748	595	7,63923632	3,40527057	4,95779198	8,47426666	8,3697307	1,96677988
667,85144	595	5,3828687	2,89726309	5,38570578	8,60119083	4,35179044	0,93419359
669,659424	595	5,18637125	3,73263529	3,42480915	8,93021873	7,26774829	0,93609524
671,46582	595	7,47601077	3,73645178	4,36330479	9,77091698	7,89876612	2,29057245
673,27063	595	6,15017994	3,23010741	4,06751587	7,51343191	5,63427202	1,14977385
675,073792	595	6,99755191	0,73078366	2,19441852	4,49582947	5,01788615	0,41890471
676,875305	595	5,63874876	3,02695833	1,67162095	5,33125197	5,7485712	3,03648341
678,675171	595	5,63964488	3,2362283	4,28420943	4,39114053	4,80865999	0,52361482
680,47345	595	6,57456352	3,44238766	5,11624455	2,92519253	3,23814514	0,10464303
682,27002	595	3,03687374	1,98884894	3,98144915	3,87882789	2,83009323	1,8901017
684,065063	595	4,93818911	3,04570136	4,20492921	3,15544898	5,25833322	0,21070934
685,858398	595	6,51706241	1,26084266	3,15507933	0,94704965	5,5762765	1,68641363

This journal is © The Royal Society of Chemistry 20xx

Please do not adjust margins

Please do not adjust margins

687,650024	595	3,78785981	3,15523791	4,21095138	4,00262627	3,15951842	1,26606666
689,440186	595	4,11299989	1,26501244	4,43171905	4,32860539	6,43920374	-0,8459954
691,228577	595	5,48669762	4,11331292	4,32833013	2,95757497	2,95715408	1,58702173
693,015381	595	3,49074253	0,95162501	2,11671658	3,07094419	5,08221877	0,63641302
694,800476	595	3,59562428	2,74845224	2,01037848	3,49364985	4,12827134	-0,2120847
696,583984	595	4,14103906	1,06136358	2,97462066	1,59443209	4,25121385	-0,319411
698,365845	595	4,25915379	3,0866031	6,39209792	1,17253567	3,62368541	1,17446464
700,146118	595	4,38306891	-0,5342984	2,13920929	3,4246364	5,02921892	-0,3215878
701,924683	595	6,00964592	2,57449207	3,22115001	3,97495878	4,51147327	2,36737725
347,741211	600	0	0	0	0	0	0
349,808533	600	0	0	0	0	0	0
351,874329	600	0	0	0	0	0	0
353,938568	600	0	0	0	0	0	0
356,001343	600	0	0	0	0	0	0
358,062561	600	0	0	0	0	0	0
360,122253	600	0	0	0	0	0	0
362,180481	600	0	0	0	0	0	0
364,237152	600	0	0	0	0	0	0
366,292328	600	0	0	0	0	0	0
368,345978	600	0	0	0	0	0	0
370,398132	600	0	0	0	0	0	0
372,44873	600	0	0	0	0	0	0
374,497803	600	0	0	0	0	0	0
376,545349	600	0	0	0	0	0	0
378,5914	600	0	0	0	0	0	0
380,635925	600	0	0	0	0	0	0
382,678955	600	0	0	0	0	0	0
384,720398	600	0	0	0	0	0	0
386,760376	600	0	0	0	0	0	0
388,798767	600	0	0	0	0	0	0
390,835663	600	0	0	0	0	0	0
392,871033	600	0	0	0	0	0	0
394,904877	600	0	0	0	0	0	0
396,937195	600	0	0	0	0	0	0
398,967957	600	0	0	0	0	0	0
400,997253	600	0	0	0	0	0	0
403,024963	600	0	0	0	0	0	0
405,051147	600	0	0	0	0	0	0
407,075806	600	0	0	0	0	0	0
409,098938	600	0	0	0	0	0	0
411,120544	600	0	0	0	0	0	0
413,140564	600	0	0	0	0	0	0
415,159119	600	0	0	0	0	0	0
417,176086	600	0	0	0	0	0	0
419,191528	600	0	0	0	0	0	0
421,205444	600	0	0	0	0	0	0
423,217834	600	0	0	0	0	0	0

This journal is © The Royal Society of Chemistry 20xx

Please do not adjust margins

Please do not adjust margins

425,228668	600	0	0	0	0	0	0
427,237976	600	0	0	0	0	0	0
429,245728	600	0	0	0	0	0	0
431,251953	600	0	0	0	0	0	0
433,256592	600	0	0	0	0	0	0
435,259766	600	0	0	0	0	0	0
437,261353	600	0	0	0	0	0	0
439,261383	600	0	0	0	0	0	0
441,259888	600	0	0	0	0	0	0
443,256836	600	0	0	0	0	0	0
445,252258	600	0	0	0	0	0	0
447,246094	600	0	0	0	0	0	0
449,238434	600	0	0	0	0	0	0
451,229187	600	0	0	0	0	0	0
453,218445	600	0	0	0	0	0	0
455,206116	600	0	0	0	0	0	0
457,192261	600	0	0	0	0	0	0
459,176819	600	0	0	0	0	0	0
461,15979	600	0	0	0	0	0	0
463,141296	600	0	0	0	0	0	0
465,121216	600	0	0	0	0	0	0
467,099579	600	0	0	0	0	0	0
469,076385	600	0	0	0	0	0	0
471,051636	600	0	0	0	0	0	0
473,02536	600	0	0	0	0	0	0
474,997498	600	0	0	0	0	0	0
476,968079	600	0	0	0	0	0	0
478,937134	600	0	0	0	0	0	0
480,904572	600	0	0	0	0	0	0
482,870453	600	0	0	0	0	0	0
484,834808	600	0	0	0	0	0	0
486,797607	600	0	0	0	0	0	0
488,75885	600	0	0	0	0	0	0
490,718506	600	0	0	0	0	0	0
492,676605	600	0	0	0	0	0	0
494,633118	600	0	0	0	0	0	0
496,588104	600	0	0	0	0	0	0
498,541504	600	0	0	0	0	0	0
500,493347	600	0	0	0	0	0	0
502,443665	600	0	0	0	0	0	0
504,392365	600	0	0	0	0	0	0
506,339539	600	0	0	0	0	0	0
508,285095	600	0	0	0	0	0	0
510,229126	600	0	0	0	0	0	0
512,17157	600	0	0	0	0	0	0
514,112427	600	0	0	0	0	0	0
516,051697	600	0	0	0	0	0	0

This journal is © The Royal Society of Chemistry 20xx

Please do not adjust margins

Please do not adjust margins

517,989441	600	0	0	0	0	0	0
519,925598	600	0	0	0	0	0	0
521,860168	600	0	0	0	0	0	0
523,793152	600	0	0	0	0	0	0
525,724609	600	0	0	0	0	0	0
527,654419	600	0	0	0	0	0	0
529,582764	600	0	0	0	0	0	0
531,50946	600	0	0	0	0	0	0
533,43457	600	0	0	0	0	0	0
535,358093	600	0	0	0	0	0	0
537,280029	600	0	0	0	0	0	0
539,200439	600	0	0	0	0	0	0
541,119263	600	0	0	0	0	0	0
543,036438	600	0	0	0	0	0	0
544,952087	600	0	0	0	0	0	0
546,866211	600	0	0	0	0	0	0
548,778687	600	0	0	0	0	0	0
550,689514	600	0	0	0	0	0	0
552,598816	600	0	0	0	0	0	0
554,506531	600	0	0	0	0	0	0
556,41272	600	0	0	0	0	0	0
558,317261	600	0	0	0	0	0	0
560,220215	600	0	0	0	0	0	0
562,121582	600	0	0	0	0	0	0
564,021362	600	0	0	0	0	0	0
565,919556	600	0	0	0	0	0	0
567,816162	600	0	0	0	0	0	0
569,711182	600	0	0	0	0	0	0
571,604614	600	0	0	0	0	0	0
573,49646	600	0	0	0	0	0	0
575,386719	600	0	0	0	0	0	0
577,27533	600	0	0	0	0	0	0
579,162354	600	0	0	0	0	0	0
581,047852	600	0	0	0	0	0	0
582,931641	600	0	0	0	0	0	0
584,813965	600	0	0	0	0	0	0
586,69458	600	0	0	0	0	0	0
588,57373	600	0	0	0	0	0	0
590,451111	600	0	0	0	0	0	0
592,326965	600	0	0	0	0	0	0
594,201233	600	0	0	0	0	0	0
596,073914	600	0	0	0	0	0	0
597,944946	600	0	0	0	0	0	0
599,814453	600	0	0	0	0	0	0
601,682251	600	0	0	0	0	0	0
603,548523	600	0	0	0	0	0	0
605,413147	600	0	0	0	0	0	0

This journal is © The Royal Society of Chemistry 20xx

Please do not adjust margins

Please do not adjust margins

607,276245	600	0	0	0	0	0	0
609,137695	600	0	0	0	0	0	0
610,997437	600	7,10022143	4,14196996	5,61999252	37,2420788	15,6095573	8,60451404
612,855713	600	7,59605832	5,32732979	5,12887831	26,4841188	11,8596784	7,22250954
614,712341	600	5,73714287	4,15465742	7,2195814	20,0157657	10,6034099	3,47218946
616,567322	600	7,62472485	3,76300942	4,55420702	17,8549699	8,7299192	4,36972218
618,420715	600	5,65258133	3,76854597	5,45325918	9,73534187	7,84861735	3,08319758
620,272522	600	6,06211604	4,0747083	4,86868835	10,452902	6,96925815	3,68777005
622,122681	600	6,47354434	1,79274918	5,27747428	10,7747126	7,28359404	3,69571262
623,971313	600	7,39005337	3,49544245	5,59146187	11,2043598	7,80377176	3,40535561
625,818237	600	6,5168933	3,70977214	5,31281396	11,549901	9,24079173	3,41880137
627,663574	600	6,72922097	4,31893532	5,62340677	16,0976743	10,0619948	4,63356774
629,507324	600	7,5698116	3,43179219	6,05475456	17,3902338	11,8305183	3,74535708
631,349365	600	9,29247928	3,73734987	7,87698216	18,1113145	17,6070713	3,3429156
633,18988	600	6,3781876	4,65728514	6,68070293	17,1394393	21,5023884	4,16301805
635,028809	600	7,18381273	4,75568159	6,67670326	19,4603681	20,4758587	4,76938312
636,865967	600	7,39795345	3,5471131	6,18073402	19,1868511	16,1428406	4,26879916
638,70166	600	4,36009469	2,02803627	4,86620262	17,8769449	14,6279699	4,98300409
640,535645	600	6,79238813	3,24426206	7,60204507	18,6861006	17,8753461	3,35528434
642,368042	600	8,2215906	4,56774235	6,39341452	21,2505552	16,6766486	3,66472193
644,198853	600	7,83244766	3,96725066	7,52592665	20,6850217	16,8145335	3,46859338
646,028015	600	8,04519461	2,03684375	6,61826732	18,7706993	13,4672057	3,06406811
647,855591	600	6,10758702	2,44313752	4,78341165	22,1274473	11,6256533	4,59408076
649,681519	600	6,03796001	4,19604691	4,39975164	22,6560222	11,4828673	4,20813608
651,505798	600	6,88482814	5,0353845	5,03426261	22,23439	9,05930903	5,56518697
653,328491	600	6,69989959	6,39094212	7,11091543	22,7159438	9,91334617	3,10130079
655,149536	600	5,89663216	3,31052915	3,93037748	19,8968157	8,39476032	4,66884432
656,968994	600	3,42794937	3,42809349	6,4392255	19,8749934	10,5108039	3,43797012
658,786743	600	5,63627929	3,4445377	4,9047637	18,7156512	9,09729125	2,93105842
660,602905	600	6,27738115	5,64988055	3,45193551	15,5110833	7,12738572	3,14786574
662,41748	600	6,09539437	5,78035834	5,35877444	14,7385588	9,68625157	4,21600877
664,230469	600	4,84381736	2,21140089	5,05350443	13,9237759	8,12296822	3,48507048
666,041748	600	6,43504463	2,1099394	5,37914672	10,7789155	6,44682178	3,27982839
667,85144	600	6,45273427	4,33726602	5,28817034	8,90115573	7,52430508	2,97056921
669,659424	600	4,45191015	1,69603707	3,17936099	7,00800178	7,11485419	2,65769297
671,46582	600	4,88088704	1,90999261	2,75826354	7,54661928	5,42132199	2,1283283
673,27063	600	5,96523203	2,23705605	3,94060155	8,32313778	6,40301715	2,88450155
675,073792	600	6,93726342	1,70770586	4,16160562	5,45253137	7,37764954	0,10703912
676,875305	600	6,40241378	2,13422765	2,45381496	6,94798724	5,13130497	3,31758363
678,675171	600	5,44291657	2,13456682	4,26818247	5,77309133	4,27676703	0,21407167
680,47345	600	4,58562287	3,30605315	5,33115573	3,63213485	5,87606607	2,13908269
682,27002	600	3,31737017	2,03331235	5,34962982	3,32312825	4,93155833	0,32197429
684,065063	600	5,69048433	2,36218714	3,00575016	2,47374179	6,56141185	0,43072596
685,858398	600	5,37073467	3,43741469	2,14790552	1,50641592	4,08922862	0,75410085
687,650024	600	3,65572751	2,36557019	2,04253725	2,26186855	4,20100896	0,64701425
689,440186	600	6,25065585	1,40106797	4,84877011	1,83526881	2,69917911	0,86467973
691,228577	600	4,52856365	1,07827477	4,31213811	2,37622212	3,99675025	0,64882882

This journal is © The Royal Society of Chemistry 20xx

Please do not adjust margins

Please do not adjust margins

693,015381	600	3,24286325	0,97289988	3,35035294	1,94909521	6,82247623	1,51776004
694,800476	600	5,72762743	2,05338672	2,59317376	2,59814449	3,68105163	0,32515306
696,583984	600	3,5806524	2,17018359	3,14606511	2,93470975	3,58720556	-0,3264654
698,365845	600	4,6788087	0,32644224	3,26369506	1,74397391	0,7630605	1,63691373
700,146118	600	3,60505492	1,42023285	2,73060851	3,06414381	2,62665654	1,75301498
701,924683	600	4,93488316	-0,7676808	2,41217342	1,53796185	3,95513182	0,98986181
347,741211	605	0	0	0	0	0	0
349,808533	605	0	0	0	0	0	0
351,874329	605	0	0	0	0	0	0
353,938568	605	0	0	0	0	0	0
356,001343	605	0	0	0	0	0	0
358,062561	605	0	0	0	0	0	0
360,122253	605	0	0	0	0	0	0
362,180481	605	0	0	0	0	0	0
364,237152	605	0	0	0	0	0	0
366,292328	605	0	0	0	0	0	0
368,345978	605	0	0	0	0	0	0
370,398132	605	0	0	0	0	0	0
372,44873	605	0	0	0	0	0	0
374,497803	605	0	0	0	0	0	0
376,545349	605	0	0	0	0	0	0
378,5914	605	0	0	0	0	0	0
380,635925	605	0	0	0	0	0	0
382,678955	605	0	0	0	0	0	0
384,720398	605	0	0	0	0	0	0
386,760376	605	0	0	0	0	0	0
388,798767	605	0	0	0	0	0	0
390,835663	605	0	0	0	0	0	0
392,871033	605	0	0	0	0	0	0
394,904877	605	0	0	0	0	0	0
396,937195	605	0	0	0	0	0	0
398,967957	605	0	0	0	0	0	0
400,997253	605	0	0	0	0	0	0
403,024963	605	0	0	0	0	0	0
405,051147	605	0	0	0	0	0	0
407,075806	605	0	0	0	0	0	0
409,098938	605	0	0	0	0	0	0
411,120544	605	0	0	0	0	0	0
413,140564	605	0	0	0	0	0	0
415,159119	605	0	0	0	0	0	0
417,176086	605	0	0	0	0	0	0
419,191528	605	0	0	0	0	0	0
421,205444	605	0	0	0	0	0	0
423,217834	605	0	0	0	0	0	0
425,228668	605	0	0	0	0	0	0
427,237976	605	0	0	0	0	0	0
429,245728	605	0	0	0	0	0	0

Please do not adjust margins

Please do not adjust margins

431,251953	605	0	0	0	0	0	0
433,256592	605	0	0	0	0	0	0
435,259766	605	0	0	0	0	0	0
437,261353	605	0	0	0	0	0	0
439,261383	605	0	0	0	0	0	0
441,259888	605	0	0	0	0	0	0
443,256836	605	0	0	0	0	0	0
445,252258	605	0	0	0	0	0	0
447,246094	605	0	0	0	0	0	0
449,238434	605	0	0	0	0	0	0
451,229187	605	0	0	0	0	0	0
453,218445	605	0	0	0	0	0	0
455,206116	605	0	0	0	0	0	0
457,192261	605	0	0	0	0	0	0
459,176819	605	0	0	0	0	0	0
461,15979	605	0	0	0	0	0	0
463,141296	605	0	0	0	0	0	0
465,121216	605	0	0	0	0	0	0
467,099579	605	0	0	0	0	0	0
469,076385	605	0	0	0	0	0	0
471,051636	605	0	0	0	0	0	0
473,02536	605	0	0	0	0	0	0
474,997498	605	0	0	0	0	0	0
476,968079	605	0	0	0	0	0	0
478,937134	605	0	0	0	0	0	0
480,904572	605	0	0	0	0	0	0
482,870453	605	0	0	0	0	0	0
484,834808	605	0	0	0	0	0	0
486,797607	605	0	0	0	0	0	0
488,75885	605	0	0	0	0	0	0
490,718506	605	0	0	0	0	0	0
492,676605	605	0	0	0	0	0	0
494,633118	605	0	0	0	0	0	0
496,588104	605	0	0	0	0	0	0
498,541504	605	0	0	0	0	0	0
500,493347	605	0	0	0	0	0	0
502,443665	605	0	0	0	0	0	0
504,392365	605	0	0	0	0	0	0
506,339539	605	0	0	0	0	0	0
508,285095	605	0	0	0	0	0	0
510,229126	605	0	0	0	0	0	0
512,17157	605	0	0	0	0	0	0
514,112427	605	0	0	0	0	0	0
516,051697	605	0	0	0	0	0	0
517,989441	605	0	0	0	0	0	0
519,925598	605	0	0	0	0	0	0
521,860168	605	0	0	0	0	0	0

This journal is © The Royal Society of Chemistry 20xx

Please do not adjust margins

Please do not adjust margins

523,793152	605	0	0	0	0	0	0
525,724609	605	0	0	0	0	0	0
527,654419	605	0	0	0	0	0	0
529,582764	605	0	0	0	0	0	0
531,50946	605	0	0	0	0	0	0
533,43457	605	0	0	0	0	0	0
535,358093	605	0	0	0	0	0	0
537,280029	605	0	0	0	0	0	0
539,200439	605	0	0	0	0	0	0
541,119263	605	0	0	0	0	0	0
543,036438	605	0	0	0	0	0	0
544,952087	605	0	0	0	0	0	0
546,866211	605	0	0	0	0	0	0
548,778687	605	0	0	0	0	0	0
550,689514	605	0	0	0	0	0	0
552,598816	605	0	0	0	0	0	0
554,506531	605	0	0	0	0	0	0
556,41272	605	0	0	0	0	0	0
558,317261	605	0	0	0	0	0	0
560,220215	605	0	0	0	0	0	0
562,121582	605	0	0	0	0	0	0
564,021362	605	0	0	0	0	0	0
565,919556	605	0	0	0	0	0	0
567,816162	605	0	0	0	0	0	0
569,711182	605	0	0	0	0	0	0
571,604614	605	0	0	0	0	0	0
573,49646	605	0	0	0	0	0	0
575,386719	605	0	0	0	0	0	0
577,27533	605	0	0	0	0	0	0
579,162354	605	0	0	0	0	0	0
581,047852	605	0	0	0	0	0	0
582,931641	605	0	0	0	0	0	0
584,813965	605	0	0	0	0	0	0
586,69458	605	0	0	0	0	0	0
588,57373	605	0	0	0	0	0	0
590,451111	605	0	0	0	0	0	0
592,326965	605	0	0	0	0	0	0
594,201233	605	0	0	0	0	0	0
596,073914	605	0	0	0	0	0	0
597,944946	605	0	0	0	0	0	0
599,814453	605	0	0	0	0	0	0
601,682251	605	0	0	0	0	0	0
603,548523	605	0	0	0	0	0	0
605,413147	605	0	0	0	0	0	0
607,276245	605	0	0	0	0	0	0
609,137695	605	0	0	0	0	0	0
610,997437	605	0	0	0	0	0	0

Please do not adjust margins

612,855713	605	0	0	0	0	0	0
614,712341	605	0	0	0	0	0	0
616,567322	605	5,90402477	4,40172724	6,90826915	33,9708476	14,613904	6,21233123
618,420715	605	6,01292688	3,20596622	6,21656331	20,5730679	14,0339506	3,51212101
620,272522	605	5,12185518	4,71877498	3,41633173	16,5940192	8,13690737	6,23470003
622,122681	605	5,13288643	2,81723971	6,04180534	12,7998749	9,36249636	2,2170777
623,971313	605	5,34877628	4,43920838	3,43306068	9,80304109	7,5710665	2,52630235
625,818237	605	3,64747617	2,73481541	5,77815007	9,53736362	8,20903704	3,14498378
627,663574	605	6,29280661	4,66750529	4,16351901	12,2983993	5,78688412	4,67495335
629,507324	605	5,50782681	2,34524729	6,02091542	12,1546932	8,26397071	3,88093945
631,349365	605	6,4305339	3,5714849	7,86359625	11,8570048	9,29105667	3,16836298
633,18988	605	6,54786055	3,47754418	6,14178624	12,7043268	12,1782152	3,38064947
635,028809	605	7,97542738	3,47546221	5,21739284	18,1235452	13,3982443	2,25241701
636,865967	605	8,19297122	4,09530004	5,22571376	16,8192186	17,6196438	2,87128451
638,70166	605	4,61108032	2,15121472	6,66388555	16,8284667	20,4992232	3,89888589
640,535645	605	5,12248157	1,6387197	6,25265165	19,9031885	17,7285713	0,92325074
642,368042	605	6,97497169	2,87121596	5,74705777	20,5434821	14,3641128	2,25955528
644,198853	605	6,27045881	4,41888024	5,14237197	20,176047	12,955592	3,19078787
646,028015	605	4,42527869	5,0413	4,83942795	19,374948	17,6029524	2,67924404
647,855591	605	8,02371925	3,29082958	5,04314205	20,19053	20,2705057	4,22310358
649,681519	605	6,51540593	3,51523317	5,07015234	18,7451979	17,0687653	3,52084252
651,505798	605	6,85369771	4,25636789	4,77928402	21,1099679	12,3607609	4,26315989
653,328491	605	6,04151196	5,62322806	5,62775788	21,6965912	9,68986728	2,71180057
655,149536	605	6,69070472	4,18048022	3,87005924	19,7862831	7,73821592	3,97779356
656,968994	605	3,77907637	4,82742229	5,77656756	23,8626983	9,97525503	3,25845416
658,786743	605	6,43415159	3,69065789	4,53788942	21,5477608	10,5506372	5,06955041
660,602905	605	5,0749433	3,69940853	2,75034582	18,8460563	9,41233114	5,08157043
662,41748	605	7,75281435	2,9728216	5,95043274	19,6751829	7,64867583	4,89171459
664,230469	605	6,91681233	1,3829621	4,36516327	15,771205	9,89903407	4,68826408
666,041748	605	6,1831877	2,66439578	5,54640757	16,0134984	8,63747275	4,80356537
667,85144	605	6,09328526	3,84727692	5,13383482	13,9165163	7,48500214	1,71262939
669,659424	605	4,92739797	3,53384942	4,39407702	9,11778317	6,32162973	1,50160117
671,46582	605	4,39630172	3,75185434	3,21846572	9,55661665	4,39748862	3,75784128
673,27063	605	4,736469	1,93708563	3,01567165	6,68350438	7,2142977	2,04796428
675,073792	605	6,57911341	0,43129242	3,3452088	4,96827807	5,71782214	0,64797096
676,875305	605	4,8525242	0,10780266	1,94201107	7,88294655	5,60887517	3,56316465
678,675171	605	4,63759336	2,1563959	5,61114579	7,34418567	5,28612616	0,75594292
680,47345	605	6,03504642	0,96963747	5,39121425	6,90689871	6,03667575	0,97118474
682,27002	605	3,13612199	0,5405542	3,89512545	4,44006504	5,19222401	2,27395046
684,065063	605	3,58055221	2,82022469	3,58239945	5,43271097	7,59716127	0,21728654
685,858398	605	3,90776917	0,7596241	1,30326174	4,02196837	5,64607938	2,17381786
687,650024	605	1,95582554	0,7603787	2,82653883	3,04667525	3,58664822	1,19678751
689,440186	605	5,009774	1,85090239	3,81375111	2,61747215	2,83237587	1,30860418
691,228577	605	3,81365971	-0,8714414	5,55991394	1,96406983	2,72477808	0,21820799
693,015381	605	2,73092704	1,31046561	2,95092283	2,40659658	5,35406209	1,31255675
694,800476	605	4,04076248	2,72945475	3,82431487	2,18726861	5,46196406	0,65611445
696,583984	605	4,38602318	0,76733192	1,42619294	1,86668217	3,94848658	-0,7685564

 This journal is © The Royal Society of Chemistry 20xx

Please do not adjust margins

698,365845	605	3,40871927	1,86875667	4,51063201	0,33034012	2,85969769	2,20204552
700,146118	605	4,30551653	1,87622046	2,09864149	1,76885063	4,52753426	0,33162607
701,924683	605	4,76535791	-0,2215804	1,77407122	0,66586907	3,3255659	1,10966995
347,741211	610	0	0	0	0	0	0
349,808533	610	0	0	0	0	0	0
351,874329	610	0	0	0	0	0	0
353,938568	610	0	0	0	0	0	0
356,001343	610	0	0	0	0	0	0
358,062561	610	0	0	0	0	0	0
360,122253	610	0	0	0	0	0	0
362,180481	610	0	0	0	0	0	0
364,237152	610	0	0	0	0	0	0
366,292328	610	0	0	0	0	0	0
368,345978	610	0	0	0	0	0	0
370,398132	610	0	0	0	0	0	0
372,44873	610	0	0	0	0	0	0
374,497803	610	0	0	0	0	0	0
376,545349	610	0	0	0	0	0	0
378,5914	610	0	0	0	0	0	0
380,635925	610	0	0	0	0	0	0
382,678955	610	0	0	0	0	0	0
384,720398	610	0	0	0	0	0	0
386,760376	610	0	0	0	0	0	0
388,798767	610	0	0	0	0	0	0
390,835663	610	0	0	0	0	0	0
392,871033	610	0	0	0	0	0	0
394,904877	610	0	0	0	0	0	0
396,937195	610	0	0	0	0	0	0
398,967957	610	0	0	0	0	0	0
400,997253	610	0	0	0	0	0	0
403,024963	610	0	0	0	0	0	0
405,051147	610	0	0	0	0	0	0
407,075806	610	0	0	0	0	0	0
409,098938	610	0	0	0	0	0	0
411,120544	610	0	0	0	0	0	0
413,140564	610	0	0	0	0	0	0
415,159119	610	0	0	0	0	0	0
417,176086	610	0	0	0	0	0	0
419,191528	610	0	0	0	0	0	0
421,205444	610	0	0	0	0	0	0
423,217834	610	0	0	0	0	0	0
425,228668	610	0	0	0	0	0	0
427,237976	610	0	0	0	0	0	0
429,245728	610	0	0	0	0	0	0
431,251953	610	0	0	0	0	0	0
433,256592	610	0	0	0	0	0	0
435,259766	610	0	0	0	0	0	0

Please do not adjust margins

Please do not adjust margins

437,261353	610	0	0	0	0	0	0
439,261383	610	0	0	0	0	0	0
441,259888	610	0	0	0	0	0	0
443,256836	610	0	0	0	0	0	0
445,252258	610	0	0	0	0	0	0
447,246094	610	0	0	0	0	0	0
449,238434	610	0	0	0	0	0	0
451,229187	610	0	0	0	0	0	0
453,218445	610	0	0	0	0	0	0
455,206116	610	0	0	0	0	0	0
457,192261	610	0	0	0	0	0	0
459,176819	610	0	0	0	0	0	0
461,15979	610	0	0	0	0	0	0
463,141296	610	0	0	0	0	0	0
465,121216	610	0	0	0	0	0	0
467,099579	610	0	0	0	0	0	0
469,076385	610	0	0	0	0	0	0
471,051636	610	0	0	0	0	0	0
473,02536	610	0	0	0	0	0	0
474,997498	610	0	0	0	0	0	0
476,968079	610	0	0	0	0	0	0
478,937134	610	0	0	0	0	0	0
480,904572	610	0	0	0	0	0	0
482,870453	610	0	0	0	0	0	0
484,834808	610	0	0	0	0	0	0
486,797607	610	0	0	0	0	0	0
488,75885	610	0	0	0	0	0	0
490,718506	610	0	0	0	0	0	0
492,676605	610	0	0	0	0	0	0
494,633118	610	0	0	0	0	0	0
496,588104	610	0	0	0	0	0	0
498,541504	610	0	0	0	0	0	0
500,493347	610	0	0	0	0	0	0
502,443665	610	0	0	0	0	0	0
504,392365	610	0	0	0	0	0	0
506,339539	610	0	0	0	0	0	0
508,285095	610	0	0	0	0	0	0
510,229126	610	0	0	0	0	0	0
512,17157	610	0	0	0	0	0	0
514,112427	610	0	0	0	0	0	0
516,051697	610	0	0	0	0	0	0
517,989441	610	0	0	0	0	0	0
519,925598	610	0	0	0	0	0	0
521,860168	610	0	0	0	0	0	0
523,793152	610	0	0	0	0	0	0
525,724609	610	0	0	0	0	0	0
527,654419	610	0	0	0	0	0	0

This journal is © The Royal Society of Chemistry 20xx

Please do not adjust margins

Please do not adjust margins

529,582764	610	0	0	0	0	0	0
531,50946	610	0	0	0	0	0	0
533,43457	610	0	0	0	0	0	0
535,358093	610	0	0	0	0	0	0
537,280029	610	0	0	0	0	0	0
539,200439	610	0	0	0	0	0	0
541,119263	610	0	0	0	0	0	0
543,036438	610	0	0	0	0	0	0
544,952087	610	0	0	0	0	0	0
546,866211	610	0	0	0	0	0	0
548,778687	610	0	0	0	0	0	0
550,689514	610	0	0	0	0	0	0
552,598816	610	0	0	0	0	0	0
554,506531	610	0	0	0	0	0	0
556,41272	610	0	0	0	0	0	0
558,317261	610	0	0	0	0	0	0
560,220215	610	0	0	0	0	0	0
562,121582	610	0	0	0	0	0	0
564,021362	610	0	0	0	0	0	0
565,919556	610	0	0	0	0	0	0
567,816162	610	0	0	0	0	0	0
569,711182	610	0	0	0	0	0	0
571,604614	610	0	0	0	0	0	0
573,49646	610	0	0	0	0	0	0
575,386719	610	0	0	0	0	0	0
577,27533	610	0	0	0	0	0	0
579,162354	610	0	0	0	0	0	0
581,047852	610	0	0	0	0	0	0
582,931641	610	0	0	0	0	0	0
584,813965	610	0	0	0	0	0	0
586,69458	610	0	0	0	0	0	0
588,57373	610	0	0	0	0	0	0
590,451111	610	0	0	0	0	0	0
592,326965	610	0	0	0	0	0	0
594,201233	610	0	0	0	0	0	0
596,073914	610	0	0	0	0	0	0
597,944946	610	0	0	0	0	0	0
599,814453	610	0	0	0	0	0	0
601,682251	610	0	0	0	0	0	0
603,548523	610	0	0	0	0	0	0
605,413147	610	0	0	0	0	0	0
607,276245	610	0	0	0	0	0	0
609,137695	610	0	0	0	0	0	0
610,997437	610	0	0	0	0	0	0
612,855713	610	0	0	0	0	0	0
614,712341	610	0	0	0	0	0	0
616,567322	610	0	0	0	0	0	0

Please do not adjust margins

Please do not adjust margins

618,420715	610	0	0	0	0	0	0
620,272522	610	5,44959379	4,14228245	6,05886139	40,5161272	16,7665445	9,09922552
622,122681	610	4,85451636	4,65744831	5,05992561	25,2125783	11,8428356	6,68713691
623,971313	610	4,56356594	2,63967613	5,78410366	18,8850414	8,62731885	5,07987901
625,818237	610	3,46164184	4,58670923	3,7694183	13,5571386	8,76328171	3,05996189
627,663574	610	5,40557401	2,45054494	3,1637127	11,9471686	5,51219808	4,18929895
629,507324	610	4,81723271	2,25739693	3,58952687	11,0824736	6,05225039	2,97775741
631,349365	610	4,61564341	2,56711441	5,64484239	8,83143685	4,61952536	3,28822299
633,18988	610	3,80394672	1,74971414	4,21779971	8,85205968	8,02587535	3,08990767
635,028809	610	5,65113009	4,11450967	5,24339031	12,5500549	8,74090999	1,44109363
636,865967	610	8,33584661	2,16356263	3,81009509	12,5700703	9,16684333	1,6495908
638,70166	610	2,9860799	1,7524185	5,04857604	13,0924321	10,5115971	3,610464
640,535645	610	4,32387314	3,60725777	6,79887386	16,9036574	15,3523558	1,34078318
642,368042	610	4,84445189	2,27015207	4,43490826	17,2336172	18,9814646	3,51089025
644,198853	610	5,37138252	3,30916328	5,89151501	17,3742154	18,2987368	3,10452771
646,028015	610	6,72200422	3,62358913	4,4496236	16,8766487	16,2498809	3,31533142
647,855591	610	4,65164849	1,75925493	4,13736429	15,9377965	14,0701391	2,89963914
649,681519	610	6,44326319	2,49695608	4,15952337	17,2717145	14,4575296	2,60282961
651,505798	610	5,1131713	2,08934298	3,34128589	16,9247576	14,7257859	5,0179583
653,328491	610	3,03549063	3,45804244	3,77053342	20,8543739	15,6092585	2,30698689
655,149536	610	4,8324011	3,36543031	2,20746503	19,6679878	13,5631311	4,20975364
656,968994	610	2,95361886	3,4849443	6,22755867	19,1156107	9,18503426	2,95900705
658,786743	610	4,76965779	3,92610501	5,93925715	20,9051897	10,9263986	4,3536159
660,602905	610	4,14350452	2,5527009	3,50821778	20,9547564	7,97497959	3,83175081
662,41748	610	7,15030521	4,9146556	5,76650844	21,5831251	10,0402086	6,62877097
664,230469	610	5,56044094	1,92692084	3,95892258	21,1974813	8,98980522	3,96368525
666,041748	610	6,64185484	2,89565578	5,78843079	20,4853901	9,86394459	5,58075017
667,85144	610	3,97458356	3,97902907	6,23429425	18,7133843	4,94553006	4,19706855
669,659424	610	3,22919534	2,37072524	2,4772526	15,1951627	8,72616032	3,12725004
671,46582	610	5,49524504	3,02037174	4,31267045	15,8579631	6,9017936	3,77812636
673,27063	610	5,9494657	3,46537896	5,95315733	12,3462	7,57841567	2,70923596
675,073792	610	4,44359682	1,51902285	3,57877278	10,7423467	6,29135065	1,95440625
676,875305	610	3,79259522	0,65088637	2,81910462	7,48567075	4,44649095	4,34230164
678,675171	610	3,25131253	1,62747454	3,57866296	6,83582905	5,31494347	1,30289752
680,47345	610	4,6566575	3,1440491	4,11773916	6,07165448	4,87734483	1,51888686
682,27002	610	3,36875864	0,97912061	4,45821958	5,87509838	3,697875	2,06848965
684,065063	610	3,37995577	0,5457639	2,83656061	5,23966767	6,11086174	0,9830676
685,858398	610	6,981032	1,63800942	2,7286577	3,82226576	4,69432569	1,52988659
687,650024	610	3,27560946	2,40480033	4,69795348	5,79375214	4,48043133	1,75017869
689,440186	610	4,37757451	2,19123538	3,94226171	4,60188782	4,27172484	1,20602911
691,228577	610	1,86138439	3,83654832	3,94420104	3,72717037	1,64377932	0,87754356
693,015381	610	1,97585854	1,64839042	2,08692258	3,07719172	5,82269871	1,86949286
694,800476	610	4,71892753	2,19730492	3,51393906	2,08757282	2,74586997	1,099427
696,583984	610	3,305563	0,88246939	2,20507607	3,64041844	4,52140227	-0,6623186
698,365845	610	3,64633642	1,54866082	1,87958128	1,32750823	2,65411138	2,21393231
700,146118	610	5,32494522	0,5553022	0,77703636	2,66562056	4,77427542	-0,1111387
701,924683	610	4,89996523	1,11487404	2,67436672	2,11839579	3,56660823	2,67758405

This journal is © The Royal Society of Chemistry 20xx

Please do not adjust margins

Please do not adjust margins

347,741211	615	0	0	0	0	0	0
349,808533	615	0	0	0	0	0	0
351,874329	615	0	0	0	0	0	0
353,938568	615	0	0	0	0	0	0
356,001343	615	0	0	0	0	0	0
358,062561	615	0	0	0	0	0	0
360,122253	615	0	0	0	0	0	0
362,180481	615	0	0	0	0	0	0
364,237152	615	0	0	0	0	0	0
366,292328	615	0	0	0	0	0	0
368,345978	615	0	0	0	0	0	0
370,398132	615	0	0	0	0	0	0
372,44873	615	0	0	0	0	0	0
374,497803	615	0	0	0	0	0	0
376,545349	615	0	0	0	0	0	0
378,5914	615	0	0	0	0	0	0
380,635925	615	0	0	0	0	0	0
382,678955	615	0	0	0	0	0	0
384,720398	615	0	0	0	0	0	0
386,760376	615	0	0	0	0	0	0
388,798767	615	0	0	0	0	0	0
390,835663	615	0	0	0	0	0	0
392,871033	615	0	0	0	0	0	0
394,904877	615	0	0	0	0	0	0
396,937195	615	0	0	0	0	0	0
398,967957	615	0	0	0	0	0	0
400,997253	615	0	0	0	0	0	0
403,024963	615	0	0	0	0	0	0
405,051147	615	0	0	0	0	0	0
407,075806	615	0	0	0	0	0	0
409,098938	615	0	0	0	0	0	0
411,120544	615	0	0	0	0	0	0
413,140564	615	0	0	0	0	0	0
415,159119	615	0	0	0	0	0	0
417,176086	615	0	0	0	0	0	0
419,191528	615	0	0	0	0	0	0
421,205444	615	0	0	0	0	0	0
423,217834	615	0	0	0	0	0	0
425,228668	615	0	0	0	0	0	0
427,237976	615	0	0	0	0	0	0
429,245728	615	0	0	0	0	0	0
431,251953	615	0	0	0	0	0	0
433,256592	615	0	0	0	0	0	0
435,259766	615	0	0	0	0	0	0
437,261353	615	0	0	0	0	0	0
439,261383	615	0	0	0	0	0	0
441,259888	615	0	0	0	0	0	0

Please do not adjust margins

443,256836	615	0	0	0	0	0	0
445,252258	615	0	0	0	0	0	0
447,246094	615	0	0	0	0	0	0
449,238434	615	0	0	0	0	0	0
451,229187	615	0	0	0	0	0	0
453,218445	615	0	0	0	0	0	0
455,206116	615	0	0	0	0	0	0
457,192261	615	0	0	0	0	0	0
459,176819	615	0	0	0	0	0	0
461,15979	615	0	0	0	0	0	0
463,141296	615	0	0	0	0	0	0
465,121216	615	0	0	0	0	0	0
467,099579	615	0	0	0	0	0	0
469,076385	615	0	0	0	0	0	0
471,051636	615	0	0	0	0	0	0
473,02536	615	0	0	0	0	0	0
474,997498	615	0	0	0	0	0	0
476,968079	615	0	0	0	0	0	0
478,937134	615	0	0	0	0	0	0
480,904572	615	0	0	0	0	0	0
482,870453	615	0	0	0	0	0	0
484,834808	615	0	0	0	0	0	0
486,797607	615	0	0	0	0	0	0
488,75885	615	0	0	0	0	0	0
490,718506	615	0	0	0	0	0	0
492,676605	615	0	0	0	0	0	0
494,633118	615	0	0	0	0	0	0
496,588104	615	0	0	0	0	0	0
498,541504	615	0	0	0	0	0	0
500,493347	615	0	0	0	0	0	0
502,443665	615	0	0	0	0	0	0
504,392365	615	0	0	0	0	0	0
506,339539	615	0	0	0	0	0	0
508,285095	615	0	0	0	0	0	0
510,229126	615	0	0	0	0	0	0
512,17157	615	0	0	0	0	0	0
514,112427	615	0	0	0	0	0	0
516,051697	615	0	0	0	0	0	0
517,989441	615	0	0	0	0	0	0
519,925598	615	0	0	0	0	0	0
521,860168	615	0	0	0	0	0	0
523,793152	615	0	0	0	0	0	0
525,724609	615	0	0	0	0	0	0
527,654419	615	0	0	0	0	0	0
529,582764	615	0	0	0	0	0	0
531,50946	615	0	0	0	0	0	0
533,43457	615	0	0	0	0	0	0

This journal is © The Royal Society of Chemistry 20xx

Please do not adjust margins

535,358093	615	0	0	0	0	0	0
537,280029	615	0	0	0	0	0	0
539,200439	615	0	0	0	0	0	0
541,119263	615	0	0	0	0	0	0
543,036438	615	0	0	0	0	0	0
544,952087	615	0	0	0	0	0	0
546,866211	615	0	0	0	0	0	0
548,778687	615	0	0	0	0	0	0
550,689514	615	0	0	0	0	0	0
552,598816	615	0	0	0	0	0	0
554,506531	615	0	0	0	0	0	0
556,41272	615	0	0	0	0	0	0
558,317261	615	0	0	0	0	0	0
560,220215	615	0	0	0	0	0	0
562,121582	615	0	0	0	0	0	0
564,021362	615	0	0	0	0	0	0
565,919556	615	0	0	0	0	0	0
567,816162	615	0	0	0	0	0	0
569,711182	615	0	0	0	0	0	0
571,604614	615	0	0	0	0	0	0
573,49646	615	0	0	0	0	0	0
575,386719	615	0	0	0	0	0	0
577,27533	615	0	0	0	0	0	0
579,162354	615	0	0	0	0	0	0
581,047852	615	0	0	0	0	0	0
582,931641	615	0	0	0	0	0	0
584,813965	615	0	0	0	0	0	0
586,69458	615	0	0	0	0	0	0
588,57373	615	0	0	0	0	0	0
590,451111	615	0	0	0	0	0	0
592,326965	615	0	0	0	0	0	0
594,201233	615	0	0	0	0	0	0
596,073914	615	0	0	0	0	0	0
597,944946	615	0	0	0	0	0	0
599,814453	615	0	0	0	0	0	0
601,682251	615	0	0	0	0	0	0
603,548523	615	0	0	0	0	0	0
605,413147	615	0	0	0	0	0	0
607,276245	615	0	0	0	0	0	0
609,137695	615	0	0	0	0	0	0
610,997437	615	0	0	0	0	0	0
612,855713	615	0	0	0	0	0	0
614,712341	615	0	0	0	0	0	0
616,567322	615	0	0	0	0	0	0
618,420715	615	0	0	0	0	0	0
620,272522	615	0	0	0	0	0	0
622,122681	615	0	0	0	0	0	0

This journal is © The Royal Society of Chemistry 20xx

Please do not adjust margins

Please do not adjust margins

623,971313	615	0	0	0	0	0	0
625,818237	615	1,64306318	3,49046628	6,05505614	30,9096404	12,0270743	7,3087645
627,663574	615	4,21775035	4,73069659	5,65446585	25,4089749	10,2976201	6,90912669
629,507324	615	3,51487392	3,10043291	3,71933432	17,9876016	6,72642094	3,62702224
631,349365	615	5,06928701	1,96505473	4,44581246	13,4489175	4,45306575	2,48893401
633,18988	615	2,48871409	3,42095968	3,62713477	8,81405728	5,50147935	2,80658933
635,028809	615	4,35264222	2,38287777	3,832104	8,70514768	5,08322826	1,86993937
636,865967	615	4,35958401	3,11305838	5,08304229	8,0962431	4,88352561	2,39317764
638,70166	615	3,63498427	0,31147701	3,73653346	9,55466569	6,7575256	1,87395146
640,535645	615	4,15350473	2,80277821	5,08490309	11,9411385	6,13262743	2,18587515
642,368042	615	4,78228755	3,01401981	3,94814718	13,410987	6,97256399	1,87586551
644,198853	615	4,79260497	2,60389856	6,35148856	14,2734036	7,92624051	1,67103338
646,028015	615	3,54646278	2,8154674	5,42065435	15,4373013	15,6620137	1,35928706
647,855591	615	3,75342671	1,45922992	3,95950521	14,7006903	19,4123555	3,34446579
649,681519	615	5,34583344	1,57183428	2,933156	16,142067	18,4670603	3,78267551
651,505798	615	2,84177109	2,52526418	2,41927394	16,1031165	13,0643217	2,7431529
653,328491	615	2,5338051	3,58848496	2,84877147	18,0530777	12,5761809	3,38659515
655,149536	615	4,13238536	3,60151621	1,58839807	17,1650234	11,4551245	2,54916994
656,968994	615	4,25586141	3,19094259	4,14690394	19,3638652	15,3366261	4,26617648
658,786743	615	5,13153161	3,84749906	3,20522786	17,1048367	17,3364686	3,64364476
660,602905	615	3,85777395	2,14256754	2,24897927	15,6451708	15,9830771	3,97454427
662,41748	615	2,26047723	3,55111741	2,25908213	18,2988139	9,58982541	3,23708003
664,230469	615	7,54979212	1,9407952	3,98814152	20,276266	7,77336932	3,67592982
666,041748	615	4,97034559	3,99669244	3,02356054	20,3132672	8,32835697	5,19901813
667,85144	615	3,46713658	4,76588873	5,19749515	22,4276874	6,07363726	5,21330999
669,659424	615	2,27994002	2,60486739	2,9295423	18,7820679	7,93353676	3,37378312
671,46582	615	4,12982404	4,56317886	5,75646282	18,3665605	4,35158824	5,44714951
673,27063	615	3,49137356	2,61774797	3,05306643	15,8200379	6,77139083	4,92164397
675,073792	615	4,59125216	2,62278896	2,29420929	12,7897158	7,11270938	3,1778339
676,875305	615	4,59038175	0,87409724	1,9660923	13,5523427	5,79849431	2,41031351
678,675171	615	3,38867736	2,29486991	2,5126283	11,1496665	5,36172406	1,86281098
680,47345	615	3,05840459	1,96552993	3,82064629	11,5780641	5,24829206	3,50379125
682,27002	615	2,95939565	1,75319208	5,0388216	7,12435909	6,25394381	3,40606006
684,065063	615	4,17891931	3,40810002	2,85749592	6,48822015	5,61423279	0,99214292
685,858398	615	4,6208302	0,2199738	3,73836343	5,50090191	4,73564378	2,6468742
687,650024	615	3,30387175	-0,3302885	2,86158834	6,16713039	3,96866352	-0,4415839
689,440186	615	3,42189213	2,20701287	3,64041122	5,07756661	2,32040486	1,5491162
691,228577	615	2,20875837	1,32485914	5,4081182	4,6383197	2,542646	0
693,015381	615	2,10362347	2,65641485	1,10648693	4,42861142	6,76058214	2,10872209
694,800476	615	5,09171103	1,65984458	0,33186317	4,31681762	5,87248124	0,44383061
696,583984	615	2,33385862	0,77772049	1,66601302	2,88949385	3,11496472	-0,222811
698,365845	615	3,23200376	1,11415114	3,67552755	2,89761302	1,89654273	2,79296317
700,146118	615	4,36384763	1,90162178	2,3483139	1,79026834	3,92024191	0,22432946
701,924683	615	4,94224264	1,12290143	1,34705248	2,4710825	3,48556297	1,35115127
347,741211	620	0	0	0	0	0	0
349,808533	620	0	0	0	0	0	0
351,874329	620	0	0	0	0	0	0

 This journal is © The Royal Society of Chemistry 20xx

Please do not adjust margins

Please do not adjust margins

353,938568	620	0	0	0	0	0	0
356,001343	620	0	0	0	0	0	0
358,062561	620	0	0	0	0	0	0
360,122253	620	0	0	0	0	0	0
362,180481	620	0	0	0	0	0	0
364,237152	620	0	0	0	0	0	0
366,292328	620	0	0	0	0	0	0
368,345978	620	0	0	0	0	0	0
370,398132	620	0	0	0	0	0	0
372,44873	620	0	0	0	0	0	0
374,497803	620	0	0	0	0	0	0
376,545349	620	0	0	0	0	0	0
378,5914	620	0	0	0	0	0	0
380,635925	620	0	0	0	0	0	0
382,678955	620	0	0	0	0	0	0
384,720398	620	0	0	0	0	0	0
386,760376	620	0	0	0	0	0	0
388,798767	620	0	0	0	0	0	0
390,835663	620	0	0	0	0	0	0
392,871033	620	0	0	0	0	0	0
394,904877	620	0	0	0	0	0	0
396,937195	620	0	0	0	0	0	0
398,967957	620	0	0	0	0	0	0
400,997253	620	0	0	0	0	0	0
403,024963	620	0	0	0	0	0	0
405,051147	620	0	0	0	0	0	0
407,075806	620	0	0	0	0	0	0
409,098938	620	0	0	0	0	0	0
411,120544	620	0	0	0	0	0	0
413,140564	620	0	0	0	0	0	0
415,159119	620	0	0	0	0	0	0
417,176086	620	0	0	0	0	0	0
419,191528	620	0	0	0	0	0	0
421,205444	620	0	0	0	0	0	0
423,217834	620	0	0	0	0	0	0
425,228668	620	0	0	0	0	0	0
427,237976	620	0	0	0	0	0	0
429,245728	620	0	0	0	0	0	0
431,251953	620	0	0	0	0	0	0
433,256592	620	0	0	0	0	0	0
435,259766	620	0	0	0	0	0	0
437,261353	620	0	0	0	0	0	0
439,261383	620	0	0	0	0	0	0
441,259888	620	0	0	0	0	0	0
443,256836	620	0	0	0	0	0	0
445,252258	620	0	0	0	0	0	0
447,246094	620	0	0	0	0	0	0

This journal is © The Royal Society of Chemistry 20xx

Please do not adjust margins

Please do not adjust margins

449,238434	620	0	0	0	0	0	0
451,229187	620	0	0	0	0	0	0
453,218445	620	0	0	0	0	0	0
455,206116	620	0	0	0	0	0	0
457,192261	620	0	0	0	0	0	0
459,176819	620	0	0	0	0	0	0
461,15979	620	0	0	0	0	0	0
463,141296	620	0	0	0	0	0	0
465,121216	620	0	0	0	0	0	0
467,099579	620	0	0	0	0	0	0
469,076385	620	0	0	0	0	0	0
471,051636	620	0	0	0	0	0	0
473,02536	620	0	0	0	0	0	0
474,997498	620	0	0	0	0	0	0
476,968079	620	0	0	0	0	0	0
478,937134	620	0	0	0	0	0	0
480,904572	620	0	0	0	0	0	0
482,870453	620	0	0	0	0	0	0
484,834808	620	0	0	0	0	0	0
486,797607	620	0	0	0	0	0	0
488,75885	620	0	0	0	0	0	0
490,718506	620	0	0	0	0	0	0
492,676605	620	0	0	0	0	0	0
494,633118	620	0	0	0	0	0	0
496,588104	620	0	0	0	0	0	0
498,541504	620	0	0	0	0	0	0
500,493347	620	0	0	0	0	0	0
502,443665	620	0	0	0	0	0	0
504,392365	620	0	0	0	0	0	0
506,339539	620	0	0	0	0	0	0
508,285095	620	0	0	0	0	0	0
510,229126	620	0	0	0	0	0	0
512,17157	620	0	0	0	0	0	0
514,112427	620	0	0	0	0	0	0
516,051697	620	0	0	0	0	0	0
517,989441	620	0	0	0	0	0	0
519,925598	620	0	0	0	0	0	0
521,860168	620	0	0	0	0	0	0
523,793152	620	0	0	0	0	0	0
525,724609	620	0	0	0	0	0	0
527,654419	620	0	0	0	0	0	0
529,582764	620	0	0	0	0	0	0
531,50946	620	0	0	0	0	0	0
533,43457	620	0	0	0	0	0	0
535,358093	620	0	0	0	0	0	0
537,280029	620	0	0	0	0	0	0
539,200439	620	0	0	0	0	0	0

This journal is © The Royal Society of Chemistry 20xx

Please do not adjust margins

Please do not adjust margins

541,119263	620	0	0	0	0	0	0
543,036438	620	0	0	0	0	0	0
544,952087	620	0	0	0	0	0	0
546,866211	620	0	0	0	0	0	0
548,778687	620	0	0	0	0	0	0
550,689514	620	0	0	0	0	0	0
552,598816	620	0	0	0	0	0	0
554,506531	620	0	0	0	0	0	0
556,41272	620	0	0	0	0	0	0
558,317261	620	0	0	0	0	0	0
560,220215	620	0	0	0	0	0	0
562,121582	620	0	0	0	0	0	0
564,021362	620	0	0	0	0	0	0
565,919556	620	0	0	0	0	0	0
567,816162	620	0	0	0	0	0	0
569,711182	620	0	0	0	0	0	0
571,604614	620	0	0	0	0	0	0
573,49646	620	0	0	0	0	0	0
575,386719	620	0	0	0	0	0	0
577,27533	620	0	0	0	0	0	0
579,162354	620	0	0	0	0	0	0
581,047852	620	0	0	0	0	0	0
582,931641	620	0	0	0	0	0	0
584,813965	620	0	0	0	0	0	0
586,69458	620	0	0	0	0	0	0
588,57373	620	0	0	0	0	0	0
590,451111	620	0	0	0	0	0	0
592,326965	620	0	0	0	0	0	0
594,201233	620	0	0	0	0	0	0
596,073914	620	0	0	0	0	0	0
597,944946	620	0	0	0	0	0	0
599,814453	620	0	0	0	0	0	0
601,682251	620	0	0	0	0	0	0
603,548523	620	0	0	0	0	0	0
605,413147	620	0	0	0	0	0	0
607,276245	620	0	0	0	0	0	0
609,137695	620	0	0	0	0	0	0
610,997437	620	0	0	0	0	0	0
612,855713	620	0	0	0	0	0	0
614,712341	620	0	0	0	0	0	0
616,567322	620	0	0	0	0	0	0
618,420715	620	0	0	0	0	0	0
620,272522	620	0	0	0	0	0	0
622,122681	620	0	0	0	0	0	0
623,971313	620	0	0	0	0	0	0
625,818237	620	0	0	0	0	0	0
627,663574	620	0	0	0	0	0	0

This journal is © The Royal Society of Chemistry 20xx

Please do not adjust margins

629,507324	620	0	0	0	0	0	0
631,349365	620	3,9815292	4,18648644	5,3386424	29,3584129	9,01629127	7,3495118
633,18988	620	3,36069618	1,99322472	4,40679564	16,9205075	7,56614994	5,26191007
635,028809	620	4,51323185	2,41140643	4,92846178	13,9694421	6,72144015	3,0500807
636,865967	620	5,4665662	2,62527418	4,72626565	11,0460957	4,94392985	2,10685869
638,70166	620	2,41923377	1,26082449	3,78309151	8,94699427	4,52565693	3,05662486
640,535645	620	3,89109604	2,31108701	3,88746325	9,05059041	4,84051122	3,05606345
642,368042	620	4,10636897	2,10352049	2,94540989	7,90247007	3,47672608	2,21567879
644,198853	620	3,79867214	1,05402934	5,5872683	11,1929203	3,48422685	1,16309755
646,028015	620	4,5425577	1,6884001	3,58843646	11,523031	5,81377018	2,22303057
647,855591	620	4,01258776	1,26574314	3,4813624	12,2576487	6,97345587	2,64530102
649,681519	620	3,71559867	2,12087044	2,96970379	15,1916528	9,98508536	2,55309009
651,505798	620	3,83745536	2,6619766	2,98190095	17,0674662	13,4392352	3,31128585
653,328491	620	4,70468064	3,31102573	4,27298934	15,1940675	17,8672254	2,3571884
655,149536	620	5,36564233	3,32304941	1,28655189	17,9339692	14,388638	1,29040817
656,968994	620	2,6939095	2,0451256	4,41388686	15,5279356	13,4777123	0,8638277
658,786743	620	4,22265779	3,78540816	4,11054326	17,5527242	14,4090747	1,8444392
660,602905	620	2,71324987	3,36073962	1,19271537	15,8566293	13,4658769	2,2838271
662,41748	620	4,57873639	3,92032652	2,72289381	16,6915249	16,1443751	3,82347749
664,230469	620	3,60467738	3,1642723	1,964352	17,5989425	13,2251622	2,40807099
666,041748	620	4,15841529	2,84211077	4,15453293	14,236258	12,154313	3,2897255
667,85144	620	3,07251854	4,16527295	5,48151783	19,1070642	7,68595246	2,30913816
669,659424	620	3,51859769	3,51473837	2,52663098	16,8352395	6,0512556	4,29712894
671,46582	620	4,18260696	2,52880118	3,73883865	22,0293503	6,27771345	4,08093171
673,27063	620	4,08849595	1,65568036	3,42229851	18,7983185	7,40798039	5,42565371
675,073792	620	4,87135792	2,87537241	2,32279565	14,5136583	6,64678739	4,54857504
676,875305	620	4,53835933	2,54311645	2,543532	16,9478529	6,09173335	5,21323166
678,675171	620	4,64978986	3,64939914	2,54393622	13,8484856	6,20347785	2,6624988
680,47345	620	4,53561592	3,97812389	5,96816086	11,8452553	5,09182441	3,32558316
682,27002	620	2,22016254	1,1088637	4,21437055	11,1086941	3,99871498	3,22587041
684,065063	620	3,22993583	2,89262832	3,78328589	11,9258106	6,01800896	1,67409963
685,858398	620	4,45703679	0,1113037	3,89626618	8,36287731	4,90571233	3,34966693
687,650024	620	2,78841521	0,7798999	3,78870419	8,81764804	2,90170966	1,67649722
689,440186	620	2,68306609	1,67507701	3,57408152	6,48867999	2,34910591	2,24049649
691,228577	620	4,47397663	1,34072083	3,35234974	5,7083702	2,57409594	0,44831973
693,015381	620	0,89705672	2,57620928	2,91271243	5,27394999	5,27340281	0,67417914
694,800476	620	4,82047551	3,5833959	1,00799478	5,72136737	1,79475256	3,14538347
696,583984	620	2,02601783	0,89946472	1,91167485	4,39282193	3,37874321	0,78951965
698,365845	620	2,37032918	1,35298818	4,05962778	3,6144946	2,93647217	2,71453067
700,146118	620	2,71976713	1,018794	3,17009924	4,42275946	3,40176969	0,90845749
701,924683	620	5,00540902	-0,227269	2,50036794	1,59376089	1,82125204	2,16588124
347,741211	625	0	0	0	0	0	0
349,808533	625	0	0	0	0	0	0
351,874329	625	0	0	0	0	0	0
353,938568	625	0	0	0	0	0	0
356,001343	625	0	0	0	0	0	0
358,062561	625	0	0	0	0	0	0

This journal is © The Royal Society of Chemistry 20xx

Please do not adjust margins

360,122253	625	0	0	0	0	0	0
362,180481	625	0	0	0	0	0	0
364,237152	625	0	0	0	0	0	0
366,292328	625	0	0	0	0	0	0
368,345978	625	0	0	0	0	0	0
370,398132	625	0	0	0	0	0	0
372,44873	625	0	0	0	0	0	0
374,497803	625	0	0	0	0	0	0
376,545349	625	0	0	0	0	0	0
378,5914	625	0	0	0	0	0	0
380,635925	625	0	0	0	0	0	0
382,678955	625	0	0	0	0	0	0
384,720398	625	0	0	0	0	0	0
386,760376	625	0	0	0	0	0	0
388,798767	625	0	0	0	0	0	0
390,835663	625	0	0	0	0	0	0
392,871033	625	0	0	0	0	0	0
394,904877	625	0	0	0	0	0	0
396,937195	625	0	0	0	0	0	0
398,967957	625	0	0	0	0	0	0
400,997253	625	0	0	0	0	0	0
403,024963	625	0	0	0	0	0	0
405,051147	625	0	0	0	0	0	0
407,075806	625	0	0	0	0	0	0
409,098938	625	0	0	0	0	0	0
411,120544	625	0	0	0	0	0	0
413,140564	625	0	0	0	0	0	0
415,159119	625	0	0	0	0	0	0
417,176086	625	0	0	0	0	0	0
419,191528	625	0	0	0	0	0	0
421,205444	625	0	0	0	0	0	0
423,217834	625	0	0	0	0	0	0
425,228668	625	0	0	0	0	0	0
427,237976	625	0	0	0	0	0	0
429,245728	625	0	0	0	0	0	0
431,251953	625	0	0	0	0	0	0
433,256592	625	0	0	0	0	0	0
435,259766	625	0	0	0	0	0	0
437,261353	625	0	0	0	0	0	0
439,261383	625	0	0	0	0	0	0
441,259888	625	0	0	0	0	0	0
443,256836	625	0	0	0	0	0	0
445,252258	625	0	0	0	0	0	0
447,246094	625	0	0	0	0	0	0
449,238434	625	0	0	0	0	0	0
451,229187	625	0	0	0	0	0	0
453,218445	625	0	0	0	0	0	0

This journal is © The Royal Society of Chemistry 20xx

Please do not adjust margins

Please do not adjust margins

455,206116	625	0	0	0	0	0	0
457,192261	625	0	0	0	0	0	0
459,176819	625	0	0	0	0	0	0
461,15979	625	0	0	0	0	0	0
463,141296	625	0	0	0	0	0	0
465,121216	625	0	0	0	0	0	0
467,099579	625	0	0	0	0	0	0
469,076385	625	0	0	0	0	0	0
471,051636	625	0	0	0	0	0	0
473,02536	625	0	0	0	0	0	0
474,997498	625	0	0	0	0	0	0
476,968079	625	0	0	0	0	0	0
478,937134	625	0	0	0	0	0	0
480,904572	625	0	0	0	0	0	0
482,870453	625	0	0	0	0	0	0
484,834808	625	0	0	0	0	0	0
486,797607	625	0	0	0	0	0	0
488,75885	625	0	0	0	0	0	0
490,718506	625	0	0	0	0	0	0
492,676605	625	0	0	0	0	0	0
494,633118	625	0	0	0	0	0	0
496,588104	625	0	0	0	0	0	0
498,541504	625	0	0	0	0	0	0
500,493347	625	0	0	0	0	0	0
502,443665	625	0	0	0	0	0	0
504,392365	625	0	0	0	0	0	0
506,339539	625	0	0	0	0	0	0
508,285095	625	0	0	0	0	0	0
510,229126	625	0	0	0	0	0	0
512,17157	625	0	0	0	0	0	0
514,112427	625	0	0	0	0	0	0
516,051697	625	0	0	0	0	0	0
517,989441	625	0	0	0	0	0	0
519,925598	625	0	0	0	0	0	0
521,860168	625	0	0	0	0	0	0
523,793152	625	0	0	0	0	0	0
525,724609	625	0	0	0	0	0	0
527,654419	625	0	0	0	0	0	0
529,582764	625	0	0	0	0	0	0
531,50946	625	0	0	0	0	0	0
533,43457	625	0	0	0	0	0	0
535,358093	625	0	0	0	0	0	0
537,280029	625	0	0	0	0	0	0
539,200439	625	0	0	0	0	0	0
541,119263	625	0	0	0	0	0	0
543,036438	625	0	0	0	0	0	0
544,952087	625	0	0	0	0	0	0

This journal is © The Royal Society of Chemistry 20xx

Please do not adjust margins

Please do not adjust margins

546,866211	625	0	0	0	0	0	0
548,778687	625	0	0	0	0	0	0
550,689514	625	0	0	0	0	0	0
552,598816	625	0	0	0	0	0	0
554,506531	625	0	0	0	0	0	0
556,41272	625	0	0	0	0	0	0
558,317261	625	0	0	0	0	0	0
560,220215	625	0	0	0	0	0	0
562,121582	625	0	0	0	0	0	0
564,021362	625	0	0	0	0	0	0
565,919556	625	0	0	0	0	0	0
567,816162	625	0	0	0	0	0	0
569,711182	625	0	0	0	0	0	0
571,604614	625	0	0	0	0	0	0
573,49646	625	0	0	0	0	0	0
575,386719	625	0	0	0	0	0	0
577,27533	625	0	0	0	0	0	0
579,162354	625	0	0	0	0	0	0
581,047852	625	0	0	0	0	0	0
582,931641	625	0	0	0	0	0	0
584,813965	625	0	0	0	0	0	0
586,69458	625	0	0	0	0	0	0
588,57373	625	0	0	0	0	0	0
590,451111	625	0	0	0	0	0	0
592,326965	625	0	0	0	0	0	0
594,201233	625	0	0	0	0	0	0
596,073914	625	0	0	0	0	0	0
597,944946	625	0	0	0	0	0	0
599,814453	625	0	0	0	0	0	0
601,682251	625	0	0	0	0	0	0
603,548523	625	0	0	0	0	0	0
605,413147	625	0	0	0	0	0	0
607,276245	625	0	0	0	0	0	0
609,137695	625	0	0	0	0	0	0
610,997437	625	0	0	0	0	0	0
612,855713	625	0	0	0	0	0	0
614,712341	625	0	0	0	0	0	0
616,567322	625	0	0	0	0	0	0
618,420715	625	0	0	0	0	0	0
620,272522	625	0	0	0	0	0	0
622,122681	625	0	0	0	0	0	0
623,971313	625	0	0	0	0	0	0
625,818237	625	0	0	0	0	0	0
627,663574	625	0	0	0	0	0	0
629,507324	625	0	0	0	0	0	0
631,349365	625	0	0	0	0	0	0
633,18988	625	0	0	0	0	0	0

This journal is © The Royal Society of Chemistry 20xx

Please do not adjust margins

Please do not adjust margins

635,028809	625	3,95022047	3,73463463	7,05002723	37,8283581	13,7741892	8,76528001
636,865967	625	6,84371105	2,67185056	4,600525	22,9044614	6,30987018	5,13907859
638,70166	625	0,64195069	1,60399181	3,10438566	16,7058909	7,49040299	4,07067425
640,535645	625	2,46035902	1,28295776	3,63895608	11,4564231	3,95848582	3,21309994
642,368042	625	3,64143617	2,46196619	3,96481344	9,75505666	4,07037201	1,07232422
644,198853	625	4,18595293	3,00364242	4,08075553	7,84236791	5,25996113	2,25673913
646,028015	625	3,22369299	2,79232672	3,65543095	9,5723164	5,80340528	2,36694099
647,855591	625	4,94082193	0,21469989	2,1493078	8,17051782	5,04889048	1,82819545
649,681519	625	3,45550206	1,18717386	1,83669628	9,9435995	6,37191407	1,40551945
651,505798	625	3,03598268	1,30041799	1,95272466	10,5270724	4,9883373	2,8225844
653,328491	625	3,15410315	2,50015074	4,35276413	12,6278816	6,09147748	0,76226901
655,149536	625	4,3662855	2,83652065	0,87371416	14,5310969	8,18785462	2,73227543
656,968994	625	1,53451611	2,51960068	3,07063854	16,2367742	14,1413167	1,97536908
658,786743	625	3,41415627	2,53168695	3,30575151	15,543021	14,4294481	1,32322982
660,602905	625	2,9806705	2,53768963	2,54041863	17,3478028	15,0157087	1,10530602
662,41748	625	4,76831819	3,76821916	3,32847482	15,6498617	12,1996165	1,77643404
664,230469	625	4,88883704	1,44363267	3,4462107	15,9031641	13,1126845	3,00364761
666,041748	625	3,56200614	2,44752891	2,1160481	14,9294601	12,8026316	3,00912882
667,85144	625	3,34856056	4,016057	3,90869871	16,6463028	13,1727252	1,89984493
669,659424	625	2,34876384	3,91244789	1,45475768	16,3443454	13,6469817	1,90371227
671,46582	625	4,47841023	3,46885415	2,12835825	17,4816773	9,51786475	1,56936603
673,27063	625	3,48436758	2,47141351	3,3737335	15,6376573	8,43102264	2,47580983
675,073792	625	3,26584662	0,1125533	2,81685858	16,5695128	7,43358725	2,36782396
676,875305	625	2,81485128	2,0255754	1,9151007	18,7076033	6,19348166	6,20026805
678,675171	625	4,50447779	3,15139581	4,61950629	18,4851477	5,96921263	5,07375279
680,47345	625	2,58809773	3,14899037	7,43060235	18,020525	4,61416813	5,40787201
682,27002	625	1,24207518	0,45141465	4,0671009	13,5622287	7,56635515	5,8788296
684,065063	625	2,71898969	1,24551645	3,51386653	15,7617973	6,68505604	3,06261506
685,858398	625	4,08027234	0,56639201	3,17520617	13,8401597	6,23456363	3,29091738
687,650024	625	1,58834885	1,4740821	3,0648475	12,6047854	4,65220062	2,72622333
689,440186	625	3,07032477	3,29593785	3,64080801	9,2193476	2,84326462	3,6433665
691,228577	625	2,38920512	0,11370894	4,21175514	7,28800624	1,36543839	1,82257939
693,015381	625	1,25465125	3,53388568	1,94002134	7,306425	5,0192605	2,28398193
694,800476	625	4,33316717	1,70951758	2,16771757	7,19046565	3,30732266	2,28341145
696,583984	625	3,0912558	0,68656695	1,1455088	3,89623117	3,32067138	1,49020791
698,365845	625	2,41106592	1,37699226	3,56105542	4,0220962	0,68896596	3,10374395
700,146118	625	3,4581367	0	1,26864711	5,30729653	5,0725963	1,50036383
701,924683	625	4,05000316	0,69390343	1,50507422	4,28532398	3,35615521	4,40253934
347,741211	630	0	0	0	0	0	0
349,808533	630	0	0	0	0	0	0
351,874329	630	0	0	0	0	0	0
353,938568	630	0	0	0	0	0	0
356,001343	630	0	0	0	0	0	0
358,062561	630	0	0	0	0	0	0
360,122253	630	0	0	0	0	0	0
362,180481	630	0	0	0	0	0	0
364,237152	630	0	0	0	0	0	0

This journal is © The Royal Society of Chemistry 20xx

Please do not adjust margins

Please do not adjust margins

366,292328	630	0	0	0	0	0	0
368,345978	630	0	0	0	0	0	0
370,398132	630	0	0	0	0	0	0
372,44873	630	0	0	0	0	0	0
374,497803	630	0	0	0	0	0	0
376,545349	630	0	0	0	0	0	0
378,5914	630	0	0	0	0	0	0
380,635925	630	0	0	0	0	0	0
382,678955	630	0	0	0	0	0	0
384,720398	630	0	0	0	0	0	0
386,760376	630	0	0	0	0	0	0
388,798767	630	0	0	0	0	0	0
390,835663	630	0	0	0	0	0	0
392,871033	630	0	0	0	0	0	0
394,904877	630	0	0	0	0	0	0
396,937195	630	0	0	0	0	0	0
398,967957	630	0	0	0	0	0	0
400,997253	630	0	0	0	0	0	0
403,024963	630	0	0	0	0	0	0
405,051147	630	0	0	0	0	0	0
407,075806	630	0	0	0	0	0	0
409,098938	630	0	0	0	0	0	0
411,120544	630	0	0	0	0	0	0
413,140564	630	0	0	0	0	0	0
415,159119	630	0	0	0	0	0	0
417,176086	630	0	0	0	0	0	0
419,191528	630	0	0	0	0	0	0
421,205444	630	0	0	0	0	0	0
423,217834	630	0	0	0	0	0	0
425,228668	630	0	0	0	0	0	0
427,237976	630	0	0	0	0	0	0
429,245728	630	0	0	0	0	0	0
431,251953	630	0	0	0	0	0	0
433,256592	630	0	0	0	0	0	0
435,259766	630	0	0	0	0	0	0
437,261353	630	0	0	0	0	0	0
439,261383	630	0	0	0	0	0	0
441,259888	630	0	0	0	0	0	0
443,256836	630	0	0	0	0	0	0
445,252258	630	0	0	0	0	0	0
447,246094	630	0	0	0	0	0	0
449,238434	630	0	0	0	0	0	0
451,229187	630	0	0	0	0	0	0
453,218445	630	0	0	0	0	0	0
455,206116	630	0	0	0	0	0	0
457,192261	630	0	0	0	0	0	0
459,176819	630	0	0	0	0	0	0

This journal is © The Royal Society of Chemistry 20xx

Please do not adjust margins

Please do not adjust margins

461,15979	630	0	0	0	0	0	0
463,141296	630	0	0	0	0	0	0
465,121216	630	0	0	0	0	0	0
467,099579	630	0	0	0	0	0	0
469,076385	630	0	0	0	0	0	0
471,051636	630	0	0	0	0	0	0
473,02536	630	0	0	0	0	0	0
474,997498	630	0	0	0	0	0	0
476,968079	630	0	0	0	0	0	0
478,937134	630	0	0	0	0	0	0
480,904572	630	0	0	0	0	0	0
482,870453	630	0	0	0	0	0	0
484,834808	630	0	0	0	0	0	0
486,797607	630	0	0	0	0	0	0
488,75885	630	0	0	0	0	0	0
490,718506	630	0	0	0	0	0	0
492,676605	630	0	0	0	0	0	0
494,633118	630	0	0	0	0	0	0
496,588104	630	0	0	0	0	0	0
498,541504	630	0	0	0	0	0	0
500,493347	630	0	0	0	0	0	0
502,443665	630	0	0	0	0	0	0
504,392365	630	0	0	0	0	0	0
506,339539	630	0	0	0	0	0	0
508,285095	630	0	0	0	0	0	0
510,229126	630	0	0	0	0	0	0
512,17157	630	0	0	0	0	0	0
514,112427	630	0	0	0	0	0	0
516,051697	630	0	0	0	0	0	0
517,989441	630	0	0	0	0	0	0
519,925598	630	0	0	0	0	0	0
521,860168	630	0	0	0	0	0	0
523,793152	630	0	0	0	0	0	0
525,724609	630	0	0	0	0	0	0
527,654419	630	0	0	0	0	0	0
529,582764	630	0	0	0	0	0	0
531,50946	630	0	0	0	0	0	0
533,43457	630	0	0	0	0	0	0
535,358093	630	0	0	0	0	0	0
537,280029	630	0	0	0	0	0	0
539,200439	630	0	0	0	0	0	0
541,119263	630	0	0	0	0	0	0
543,036438	630	0	0	0	0	0	0
544,952087	630	0	0	0	0	0	0
546,866211	630	0	0	0	0	0	0
548,778687	630	0	0	0	0	0	0
550,689514	630	0	0	0	0	0	0

This journal is © The Royal Society of Chemistry 20xx

Please do not adjust margins

Please do not adjust margins

552,598816	630	0	0	0	0	0	0
554,506531	630	0	0	0	0	0	0
556,41272	630	0	0	0	0	0	0
558,317261	630	0	0	0	0	0	0
560,220215	630	0	0	0	0	0	0
562,121582	630	0	0	0	0	0	0
564,021362	630	0	0	0	0	0	0
565,919556	630	0	0	0	0	0	0
567,816162	630	0	0	0	0	0	0
569,711182	630	0	0	0	0	0	0
571,604614	630	0	0	0	0	0	0
573,49646	630	0	0	0	0	0	0
575,386719	630	0	0	0	0	0	0
577,27533	630	0	0	0	0	0	0
579,162354	630	0	0	0	0	0	0
581,047852	630	0	0	0	0	0	0
582,931641	630	0	0	0	0	0	0
584,813965	630	0	0	0	0	0	0
586,69458	630	0	0	0	0	0	0
588,57373	630	0	0	0	0	0	0
590,451111	630	0	0	0	0	0	0
592,326965	630	0	0	0	0	0	0
594,201233	630	0	0	0	0	0	0
596,073914	630	0	0	0	0	0	0
597,944946	630	0	0	0	0	0	0
599,814453	630	0	0	0	0	0	0
601,682251	630	0	0	0	0	0	0
603,548523	630	0	0	0	0	0	0
605,413147	630	0	0	0	0	0	0
607,276245	630	0	0	0	0	0	0
609,137695	630	0	0	0	0	0	0
610,997437	630	0	0	0	0	0	0
612,855713	630	0	0	0	0	0	0
614,712341	630	0	0	0	0	0	0
616,567322	630	0	0	0	0	0	0
618,420715	630	0	0	0	0	0	0
620,272522	630	0	0	0	0	0	0
622,122681	630	0	0	0	0	0	0
623,971313	630	0	0	0	0	0	0
625,818237	630	0	0	0	0	0	0
627,663574	630	0	0	0	0	0	0
629,507324	630	0	0	0	0	0	0
631,349365	630	0	0	0	0	0	0
633,18988	630	0	0	0	0	0	0
635,028809	630	0	0	0	0	0	0
636,865967	630	0	0	0	0	0	0
638,70166	630	0	0	0	0	0	0

This journal is © The Royal Society of Chemistry 20xx

Please do not adjust margins

Please do not adjust margins

640,535645	630	3,05959355	4,69808781	5,35496106	31,3978286	11,6039361	8,65351846
642,368042	630	2,62566964	1,96901179	5,58024864	24,8637546	5,91856418	5,04483076
644,198853	630	2,41205647	1,64438315	3,94749713	16,1359351	3,51486402	3,29720517
646,028015	630	2,30508408	2,63406014	4,06184861	13,0775518	4,61860187	3,52109208
647,855591	630	3,4012466	-0,5485212	2,85301666	7,90898835	5,05624394	1,42981453
649,681519	630	1,43396842	1,54408512	0,99287203	9,71831382	5,41484556	3,2066692
651,505798	630	0,99682478	1,66117177	1,3292666	8,53845026	4,32747614	2,44263696
653,328491	630	2,33309405	2,99932608	1,66670502	8,56476968	2,67127102	1,44782553
655,149536	630	2,67607596	2,11830146	2,11882617	9,48894945	3,12780008	3,24149324
656,968994	630	1,00767616	2,79875866	3,47132039	11,0975135	4,82326017	0,56118804
658,786743	630	2,47502414	2,5872093	3,71300241	15,0929304	8,34031087	1,804416
660,602905	630	3,9468744	1,35304885	1,01503801	13,7739057	9,15090488	2,37391129
662,41748	630	2,60531155	2,49173286	2,26577279	14,6296371	14,4121678	2,9523314
664,230469	630	2,83744825	1,24832479	1,70268274	14,4312408	14,8954284	2,84438171
666,041748	630	2,95633122	2,84227909	2,04694786	13,8883798	16,0617409	1,48177759
667,85144	630	2,0523171	3,42011086	3,42095803	15,6388401	9,0238696	2,74310941
669,659424	630	2,28499424	2,17047948	1,3711687	14,5268298	10,0723671	0,57264444
671,46582	630	4,23156155	2,85881411	3,54580759	17,9767262	11,342999	1,60504385
673,27063	630	1,83703449	1,60720891	2,41141054	15,1734127	13,917988	0,80566648
675,073792	630	3,10596535	2,76052099	3,10635538	13,4750603	13,2533127	0,11531685
676,875305	630	4,02548809	0,57499951	1,26531227	16,2360966	7,25913396	5,07297949
678,675171	630	3,56599893	2,53039992	3,45140006	17,7358599	6,33834631	0,34593993
680,47345	630	1,37933304	2,18367732	5,28810728	18,5278822	5,06680663	3,91765996
682,27002	630	2,88356844	0,92262923	4,49893164	18,9385234	6,0087946	5,31873086
684,065063	630	0,92580892	2,77708765	4,28240397	18,3062958	7,76783405	3,48026696
685,858398	630	3,70485901	1,73644061	1,27370521	14,7210043	6,37937536	4,9905693
687,650024	630	2,54962081	1,96992097	4,98397553	15,0837136	4,99246616	4,0661265
689,440186	630	3,13631281	1,74218326	3,36905529	14,3045017	6,28410059	3,14397657
691,228577	630	1,39460252	-0,1162027	3,48694411	11,8681051	4,30789078	1,74751288
693,015381	630	1,98068	1,63094907	1,39830262	11,898099	6,30308138	4,20463037
694,800476	630	3,72740757	1,86347622	2,5629145	9,91260596	3,50083723	2,68562065
696,583984	630	1,2864678	-0,701624	1,87146087	7,49373314	3,1634703	0,58618698
698,365845	630	1,87648384	1,28992512	1,99401445	5,28383653	0,93995832	5,40807377
700,146118	630	3,88570565	-0,82414	0,47105377	4,24395209	2,71317343	4,01323697
701,924683	630	5,31905999	-0,2363738	1,18216176	6,62708306	5,32879052	3,6731951
347,741211	635	0	0	0	0	0	0
349,808533	635	0	0	0	0	0	0
351,874329	635	0	0	0	0	0	0
353,938568	635	0	0	0	0	0	0
356,001343	635	0	0	0	0	0	0
358,062561	635	0	0	0	0	0	0
360,122253	635	0	0	0	0	0	0
362,180481	635	0	0	0	0	0	0
364,237152	635	0	0	0	0	0	0
366,292328	635	0	0	0	0	0	0
368,345978	635	0	0	0	0	0	0
370,398132	635	0	0	0	0	0	0

This journal is © The Royal Society of Chemistry 20xx

Please do not adjust margins

Please do not adjust margins

372,44873	635	0	0	0	0	0	0
374,497803	635	0	0	0	0	0	0
376,545349	635	0	0	0	0	0	0
378,5914	635	0	0	0	0	0	0
380,635925	635	0	0	0	0	0	0
382,678955	635	0	0	0	0	0	0
384,720398	635	0	0	0	0	0	0
386,760376	635	0	0	0	0	0	0
388,798767	635	0	0	0	0	0	0
390,835663	635	0	0	0	0	0	0
392,871033	635	0	0	0	0	0	0
394,904877	635	0	0	0	0	0	0
396,937195	635	0	0	0	0	0	0
398,967957	635	0	0	0	0	0	0
400,997253	635	0	0	0	0	0	0
403,024963	635	0	0	0	0	0	0
405,051147	635	0	0	0	0	0	0
407,075806	635	0	0	0	0	0	0
409,098938	635	0	0	0	0	0	0
411,120544	635	0	0	0	0	0	0
413,140564	635	0	0	0	0	0	0
415,159119	635	0	0	0	0	0	0
417,176086	635	0	0	0	0	0	0
419,191528	635	0	0	0	0	0	0
421,205444	635	0	0	0	0	0	0
423,217834	635	0	0	0	0	0	0
425,228668	635	0	0	0	0	0	0
427,237976	635	0	0	0	0	0	0
429,245728	635	0	0	0	0	0	0
431,251953	635	0	0	0	0	0	0
433,256592	635	0	0	0	0	0	0
435,259766	635	0	0	0	0	0	0
437,261353	635	0	0	0	0	0	0
439,261383	635	0	0	0	0	0	0
441,259888	635	0	0	0	0	0	0
443,256836	635	0	0	0	0	0	0
445,252258	635	0	0	0	0	0	0
447,246094	635	0	0	0	0	0	0
449,238434	635	0	0	0	0	0	0
451,229187	635	0	0	0	0	0	0
453,218445	635	0	0	0	0	0	0
455,206116	635	0	0	0	0	0	0
457,192261	635	0	0	0	0	0	0
459,176819	635	0	0	0	0	0	0
461,15979	635	0	0	0	0	0	0
463,141296	635	0	0	0	0	0	0
465,121216	635	0	0	0	0	0	0

This journal is © The Royal Society of Chemistry 20xx

Please do not adjust margins

467,099579	635	0	0	0	0	0	0
469,076385	635	0	0	0	0	0	0
471,051636	635	0	0	0	0	0	0
473,02536	635	0	0	0	0	0	0
474,997498	635	0	0	0	0	0	0
476,968079	635	0	0	0	0	0	0
478,937134	635	0	0	0	0	0	0
480,904572	635	0	0	0	0	0	0
482,870453	635	0	0	0	0	0	0
484,834808	635	0	0	0	0	0	0
486,797607	635	0	0	0	0	0	0
488,75885	635	0	0	0	0	0	0
490,718506	635	0	0	0	0	0	0
492,676605	635	0	0	0	0	0	0
494,633118	635	0	0	0	0	0	0
496,588104	635	0	0	0	0	0	0
498,541504	635	0	0	0	0	0	0
500,493347	635	0	0	0	0	0	0
502,443665	635	0	0	0	0	0	0
504,392365	635	0	0	0	0	0	0
506,339539	635	0	0	0	0	0	0
508,285095	635	0	0	0	0	0	0
510,229126	635	0	0	0	0	0	0
512,17157	635	0	0	0	0	0	0
514,112427	635	0	0	0	0	0	0
516,051697	635	0	0	0	0	0	0
517,989441	635	0	0	0	0	0	0
519,925598	635	0	0	0	0	0	0
521,860168	635	0	0	0	0	0	0
523,793152	635	0	0	0	0	0	0
525,724609	635	0	0	0	0	0	0
527,654419	635	0	0	0	0	0	0
529,582764	635	0	0	0	0	0	0
531,50946	635	0	0	0	0	0	0
533,43457	635	0	0	0	0	0	0
535,358093	635	0	0	0	0	0	0
537,280029	635	0	0	0	0	0	0
539,200439	635	0	0	0	0	0	0
541,119263	635	0	0	0	0	0	0
543,036438	635	0	0	0	0	0	0
544,952087	635	0	0	0	0	0	0
546,866211	635	0	0	0	0	0	0
548,778687	635	0	0	0	0	0	0
550,689514	635	0	0	0	0	0	0
552,598816	635	0	0	0	0	0	0
554,506531	635	0	0	0	0	0	0
556,41272	635	0	0	0	0	0	0

This journal is © The Royal Society of Chemistry 20xx

558,317261	635	0	0	0	0	0	0
560,220215	635	0	0	0	0	0	0
562,121582	635	0	0	0	0	0	0
564,021362	635	0	0	0	0	0	0
565,919556	635	0	0	0	0	0	0
567,816162	635	0	0	0	0	0	0
569,711182	635	0	0	0	0	0	0
571,604614	635	0	0	0	0	0	0
573,49646	635	0	0	0	0	0	0
575,386719	635	0	0	0	0	0	0
577,27533	635	0	0	0	0	0	0
579,162354	635	0	0	0	0	0	0
581,047852	635	0	0	0	0	0	0
582,931641	635	0	0	0	0	0	0
584,813965	635	0	0	0	0	0	0
586,69458	635	0	0	0	0	0	0
588,57373	635	0	0	0	0	0	0
590,451111	635	0	0	0	0	0	0
592,326965	635	0	0	0	0	0	0
594,201233	635	0	0	0	0	0	0
596,073914	635	0	0	0	0	0	0
597,944946	635	0	0	0	0	0	0
599,814453	635	0	0	0	0	0	0
601,682251	635	0	0	0	0	0	0
603,548523	635	0	0	0	0	0	0
605,413147	635	0	0	0	0	0	0
607,276245	635	0	0	0	0	0	0
609,137695	635	0	0	0	0	0	0
610,997437	635	0	0	0	0	0	0
612,855713	635	0	0	0	0	0	0
614,712341	635	0	0	0	0	0	0
616,567322	635	0	0	0	0	0	0
618,420715	635	0	0	0	0	0	0
620,272522	635	0	0	0	0	0	0
622,122681	635	0	0	0	0	0	0
623,971313	635	0	0	0	0	0	0
625,818237	635	0	0	0	0	0	0
627,663574	635	0	0	0	0	0	0
629,507324	635	0	0	0	0	0	0
631,349365	635	0	0	0	0	0	0
633,18988	635	0	0	0	0	0	0
635,028809	635	0	0	0	0	0	0
636,865967	635	0	0	0	0	0	0
638,70166	635	0	0	0	0	0	0
640,535645	635	0	0	0	0	0	0
642,368042	635	0	0	0	0	0	0
644,198853	635	0	0	0	0	0	0

This journal is © The Royal Society of Chemistry 20xx

646,028015	635	3,59683908	2,58574609	4,15884771	29,0133165	9,22647581	6,19631805
647,855591	635	3,81996072	1,79798877	4,38172231	20,9073777	9,22241791	6,30620363
649,681519	635	1,92020992	1,12976157	2,03316468	12,9958374	9,27181175	5,20783963
651,505798	635	2,83543618	1,36128207	1,58784523	10,3258997	4,31434062	2,50093573
653,328491	635	2,73040925	1,47926802	2,38910954	7,51219893	2,73324594	1,25432238
655,149536	635	3,08286504	1,82724904	-0,7992618	8,68182405	2,0573786	2,63219807
656,968994	635	-0,4586084	1,14675076	3,09560867	10,3236002	2,41019559	1,95357879
658,786743	635	2,76484987	2,53495354	1,03681933	8,75952476	4,38222704	2,07841754
660,602905	635	1,50117792	0,23099672	1,61665413	9,70453524	3,12107026	2,31482836
662,41748	635	3,24783094	3,24848171	2,08789258	10,2123266	4,64457884	1,62765891
664,230469	635	3,60290813	1,74369196	3,37046449	10,1161995	6,8642718	2,56279671
666,041748	635	3,84235277	1,8633322	2,79444008	11,8820153	11,8887018	3,73450681
667,85144	635	3,5026502	1,63489761	3,6194074	13,0827841	11,8045069	2,69155545
669,659424	635	1,63789744	1,17016116	3,04181137	13,577609	12,6483358	1,87619784
671,46582	635	2,92780738	2,92839403	2,45935969	18,1610448	10,316589	2,81717429
673,27063	635	1,29328299	2,70467899	2,82171006	15,0562733	10,7100929	1,88547529
675,073792	635	5,41868903	1,41385429	2,70934616	13,3174624	10,0231975	1,88910615
676,875305	635	2,82660613	0,70679313	1,64885458	14,7289134	11,6718639	3,30530902
678,675171	635	2,5914674	0,4712703	2,70926301	14,9669542	12,734966	1,65291715
680,47345	635	1,76556091	2,5900082	2,94260331	14,6022497	10,4865448	1,17975392
682,27002	635	0,59055971	2,12644094	4,13392046	14,1801784	10,4046493	2,60445268
684,065063	635	1,18504524	1,54086749	3,43663328	16,0057245	7,47354139	2,1379986
685,858398	635	6,63916233	1,30438248	4,14947898	16,9616438	5,69662279	4,15904182
687,650024	635	2,37348484	2,01786636	3,08553217	20,5404148	2,61354579	4,40106896
689,440186	635	2,97371382	1,90355818	2,3789725	17,2557486	5,59639017	3,57668257
691,228577	635	1,07106361	1,78546369	4,04624274	15,3592179	3,09739837	2,62419084
693,015381	635	0,71584698	2,14797125	2,14754225	14,5624826	5,73272555	2,8699886
694,800476	635	3,33978484	0,47720772	2,26628395	12,888158	6,3283034	3,3474837
696,583984	635	0,83831615	0,71870067	1,79639283	12,4609103	4,67547095	3,12092348
698,365845	635	2,64211114	1,80180037	4,44355324	10,333168	1,20220731	7,22236834
700,146118	635	1,0851806	0,84419848	0,36172709	8,32367746	4,46593298	1,08768216
701,924683	635	4,59948836	-0,7263805	1,81558861	6,17593539	3,27145279	5,58063659
347,741211	640	0	0	0	0	0	0
349,808533	640	0	0	0	0	0	0
351,874329	640	0	0	0	0	0	0
353,938568	640	0	0	0	0	0	0
356,001343	640	0	0	0	0	0	0
358,062561	640	0	0	0	0	0	0
360,122253	640	0	0	0	0	0	0
362,180481	640	0	0	0	0	0	0
364,237152	640	0	0	0	0	0	0
366,292328	640	0	0	0	0	0	0
368,345978	640	0	0	0	0	0	0
370,398132	640	0	0	0	0	0	0
372,44873	640	0	0	0	0	0	0
374,497803	640	0	0	0	0	0	0
376,545349	640	0	0	0	0	0	0

This journal is © The Royal Society of Chemistry 20xx

Please do not adjust margins

378,5914	640	0	0	0	0	0	0
380,635925	640	0	0	0	0	0	0
382,678955	640	0	0	0	0	0	0
384,720398	640	0	0	0	0	0	0
386,760376	640	0	0	0	0	0	0
388,798767	640	0	0	0	0	0	0
390,835663	640	0	0	0	0	0	0
392,871033	640	0	0	0	0	0	0
394,904877	640	0	0	0	0	0	0
396,937195	640	0	0	0	0	0	0
398,967957	640	0	0	0	0	0	0
400,997253	640	0	0	0	0	0	0
403,024963	640	0	0	0	0	0	0
405,051147	640	0	0	0	0	0	0
407,075806	640	0	0	0	0	0	0
409,098938	640	0	0	0	0	0	0
411,120544	640	0	0	0	0	0	0
413,140564	640	0	0	0	0	0	0
415,159119	640	0	0	0	0	0	0
417,176086	640	0	0	0	0	0	0
419,191528	640	0	0	0	0	0	0
421,205444	640	0	0	0	0	0	0
423,217834	640	0	0	0	0	0	0
425,228668	640	0	0	0	0	0	0
427,237976	640	0	0	0	0	0	0
429,245728	640	0	0	0	0	0	0
431,251953	640	0	0	0	0	0	0
433,256592	640	0	0	0	0	0	0
435,259766	640	0	0	0	0	0	0
437,261353	640	0	0	0	0	0	0
439,261383	640	0	0	0	0	0	0
441,259888	640	0	0	0	0	0	0
443,256836	640	0	0	0	0	0	0
445,252258	640	0	0	0	0	0	0
447,246094	640	0	0	0	0	0	0
449,238434	640	0	0	0	0	0	0
451,229187	640	0	0	0	0	0	0
453,218445	640	0	0	0	0	0	0
455,206116	640	0	0	0	0	0	0
457,192261	640	0	0	0	0	0	0
459,176819	640	0	0	0	0	0	0
461,15979	640	0	0	0	0	0	0
463,141296	640	0	0	0	0	0	0
465,121216	640	0	0	0	0	0	0
467,099579	640	0	0	0	0	0	0
469,076385	640	0	0	0	0	0	0
471,051636	640	0	0	0	0	0	0

This journal is © The Royal Society of Chemistry 20xx

Please do not adjust margins

473,02536	640	0	0	0	0	0	0
474,997498	640	0	0	0	0	0	0
476,968079	640	0	0	0	0	0	0
478,937134	640	0	0	0	0	0	0
480,904572	640	0	0	0	0	0	0
482,870453	640	0	0	0	0	0	0
484,834808	640	0	0	0	0	0	0
486,797607	640	0	0	0	0	0	0
488,75885	640	0	0	0	0	0	0
490,718506	640	0	0	0	0	0	0
492,676605	640	0	0	0	0	0	0
494,633118	640	0	0	0	0	0	0
496,588104	640	0	0	0	0	0	0
498,541504	640	0	0	0	0	0	0
500,493347	640	0	0	0	0	0	0
502,443665	640	0	0	0	0	0	0
504,392365	640	0	0	0	0	0	0
506,339539	640	0	0	0	0	0	0
508,285095	640	0	0	0	0	0	0
510,229126	640	0	0	0	0	0	0
512,17157	640	0	0	0	0	0	0
514,112427	640	0	0	0	0	0	0
516,051697	640	0	0	0	0	0	0
517,989441	640	0	0	0	0	0	0
519,925598	640	0	0	0	0	0	0
521,860168	640	0	0	0	0	0	0
523,793152	640	0	0	0	0	0	0
525,724609	640	0	0	0	0	0	0
527,654419	640	0	0	0	0	0	0
529,582764	640	0	0	0	0	0	0
531,50946	640	0	0	0	0	0	0
533,43457	640	0	0	0	0	0	0
535,358093	640	0	0	0	0	0	0
537,280029	640	0	0	0	0	0	0
539,200439	640	0	0	0	0	0	0
541,119263	640	0	0	0	0	0	0
543,036438	640	0	0	0	0	0	0
544,952087	640	0	0	0	0	0	0
546,866211	640	0	0	0	0	0	0
548,778687	640	0	0	0	0	0	0
550,689514	640	0	0	0	0	0	0
552,598816	640	0	0	0	0	0	0
554,506531	640	0	0	0	0	0	0
556,41272	640	0	0	0	0	0	0
558,317261	640	0	0	0	0	0	0
560,220215	640	0	0	0	0	0	0
562,121582	640	0	0	0	0	0	0

This journal is © The Royal Society of Chemistry 20xx

Please do not adjust margins

564,021362	640	0	0	0	0	0	0
565,919556	640	0	0	0	0	0	0
567,816162	640	0	0	0	0	0	0
569,711182	640	0	0	0	0	0	0
571,604614	640	0	0	0	0	0	0
573,49646	640	0	0	0	0	0	0
575,386719	640	0	0	0	0	0	0
577,27533	640	0	0	0	0	0	0
579,162354	640	0	0	0	0	0	0
581,047852	640	0	0	0	0	0	0
582,931641	640	0	0	0	0	0	0
584,813965	640	0	0	0	0	0	0
586,69458	640	0	0	0	0	0	0
588,57373	640	0	0	0	0	0	0
590,451111	640	0	0	0	0	0	0
592,326965	640	0	0	0	0	0	0
594,201233	640	0	0	0	0	0	0
596,073914	640	0	0	0	0	0	0
597,944946	640	0	0	0	0	0	0
599,814453	640	0	0	0	0	0	0
601,682251	640	0	0	0	0	0	0
603,548523	640	0	0	0	0	0	0
605,413147	640	0	0	0	0	0	0
607,276245	640	0	0	0	0	0	0
609,137695	640	0	0	0	0	0	0
610,997437	640	0	0	0	0	0	0
612,855713	640	0	0	0	0	0	0
614,712341	640	0	0	0	0	0	0
616,567322	640	0	0	0	0	0	0
618,420715	640	0	0	0	0	0	0
620,272522	640	0	0	0	0	0	0
622,122681	640	0	0	0	0	0	0
623,971313	640	0	0	0	0	0	0
625,818237	640	0	0	0	0	0	0
627,663574	640	0	0	0	0	0	0
629,507324	640	0	0	0	0	0	0
631,349365	640	0	0	0	0	0	0
633,18988	640	0	0	0	0	0	0
635,028809	640	0	0	0	0	0	0
636,865967	640	0	0	0	0	0	0
638,70166	640	0	0	0	0	0	0
640,535645	640	0	0	0	0	0	0
642,368042	640	0	0	0	0	0	0
644,198853	640	0	0	0	0	0	0
646,028015	640	0	0	0	0	0	0
647,855591	640	0	0	0	0	0	0
649,681519	640	0	0	0	0	0	0

This journal is © The Royal Society of Chemistry 20xx

Please do not adjust margins

Please do not adjust margins

651,505798	640	1,1642159	1,86391182	1,74766697	31,1375118	9,33673851	5,48405618
653,328491	640	2,21882864	3,97302165	2,92175681	17,5469056	7,02413897	6,08616913
655,149536	640	2,34409064	1,99372466	1,29024144	12,7970546	3,64231735	3,87640242
656,968994	640	1,52995633	1,41315169	2,47336877	10,9637153	2,35959443	1,65133137
658,786743	640	2,95633726	0,23665507	2,36688883	9,35793835	6,04582857	2,96295114
660,602905	640	1,89654196	0,83025666	1,77937558	8,43024003	3,08949505	1,3067896
662,41748	640	3,09572509	0,95312724	0,4766317	8,22957201	2,86465262	2,74465263
664,230469	640	3,57903879	0,71625597	1,55210965	6,81175668	3,94666964	1,19568192
666,041748	640	1,91230401	1,67431374	1,43533109	8,2608581	2,2764716	3,5935916
667,85144	640	2,63664616	2,15860674	4,31783026	11,4049108	2,52301683	3,96381723
669,659424	640	0,96073212	2,28316753	0,6009194	11,909311	4,69514074	3,3700851
671,46582	640	3,84685775	1,6840541	1,80460145	12,4031642	7,1101677	1,80724869
673,27063	640	2,53439225	0,96608731	3,50256683	13,4188913	13,1872038	1,93528545
675,073792	640	3,26477922	0,84695424	1,93617198	14,292598	13,6974644	-0,1211883
676,875305	640	2,17610685	1,08873472	1,69382931	14,5320899	12,604126	2,18097521
678,675171	640	2,17645268	2,6617745	2,78316181	16,9567993	9,45459677	-0,1211845
680,47345	640	2,29561315	1,81346098	3,14378145	15,1284431	8,47841806	1,81638068
682,27002	640	0,24248086	1,69842883	4,12534503	16,881125	10,9385979	2,30872166
684,065063	640	2,67615502	3,52986778	3,53037208	13,5254176	12,5602272	1,09723994
685,858398	640	4,5027791	2,55723162	3,53191961	13,7751546	11,7117511	1,09772091
687,650024	640	0,97454099	2,43787803	4,51071869	15,0090898	5,61745548	0,73254091
689,440186	640	3,1745792	0,97740523	3,66579326	16,5114687	6,48724607	0,85660651
691,228577	640	1,58807044	0,36670727	3,7898498	17,0090606	3,91875463	5,7543302
693,015381	640	2,57182788	3,67634037	3,55430341	15,9479577	6,7523816	6,87355075
694,800476	640	2,6936229	3,06285175	2,08303674	17,6610177	5,03233628	4,66303014
696,583984	640	4,17967356	2,33716246	2,21447023	16,2546079	3,45058843	3,0801649
698,365845	640	2,835371	2,09702125	3,45441081	9,38501063	3,95461054	6,05408688
700,146118	640	3,21800354	1,36231553	0,99091648	14,3817017	5,83153264	4,46566542
701,924683	640	4,34858165	0,49729195	2,11379274	9,08534794	5,35574294	5,47901858
347,741211	645	0	0	0	0	0	0
349,808533	645	0	0	0	0	0	0
351,874329	645	0	0	0	0	0	0
353,938568	645	0	0	0	0	0	0
356,001343	645	0	0	0	0	0	0
358,062561	645	0	0	0	0	0	0
360,122253	645	0	0	0	0	0	0
362,180481	645	0	0	0	0	0	0
364,237152	645	0	0	0	0	0	0
366,292328	645	0	0	0	0	0	0
368,345978	645	0	0	0	0	0	0
370,398132	645	0	0	0	0	0	0
372,44873	645	0	0	0	0	0	0
374,497803	645	0	0	0	0	0	0
376,545349	645	0	0	0	0	0	0
378,5914	645	0	0	0	0	0	0
380,635925	645	0	0	0	0	0	0
382,678955	645	0	0	0	0	0	0

 This journal is © The Royal Society of Chemistry 20xx

Please do not adjust margins

Please do not adjust margins

384,720398	645	0	0	0	0	0	0
386,760376	645	0	0	0	0	0	0
388,798767	645	0	0	0	0	0	0
390,835663	645	0	0	0	0	0	0
392,871033	645	0	0	0	0	0	0
394,904877	645	0	0	0	0	0	0
396,937195	645	0	0	0	0	0	0
398,967957	645	0	0	0	0	0	0
400,997253	645	0	0	0	0	0	0
403,024963	645	0	0	0	0	0	0
405,051147	645	0	0	0	0	0	0
407,075806	645	0	0	0	0	0	0
409,098938	645	0	0	0	0	0	0
411,120544	645	0	0	0	0	0	0
413,140564	645	0	0	0	0	0	0
415,159119	645	0	0	0	0	0	0
417,176086	645	0	0	0	0	0	0
419,191528	645	0	0	0	0	0	0
421,205444	645	0	0	0	0	0	0
423,217834	645	0	0	0	0	0	0
425,228668	645	0	0	0	0	0	0
427,237976	645	0	0	0	0	0	0
429,245728	645	0	0	0	0	0	0
431,251953	645	0	0	0	0	0	0
433,256592	645	0	0	0	0	0	0
435,259766	645	0	0	0	0	0	0
437,261353	645	0	0	0	0	0	0
439,261383	645	0	0	0	0	0	0
441,259888	645	0	0	0	0	0	0
443,256836	645	0	0	0	0	0	0
445,252258	645	0	0	0	0	0	0
447,246094	645	0	0	0	0	0	0
449,238434	645	0	0	0	0	0	0
451,229187	645	0	0	0	0	0	0
453,218445	645	0	0	0	0	0	0
455,206116	645	0	0	0	0	0	0
457,192261	645	0	0	0	0	0	0
459,176819	645	0	0	0	0	0	0
461,15979	645	0	0	0	0	0	0
463,141296	645	0	0	0	0	0	0
465,121216	645	0	0	0	0	0	0
467,099579	645	0	0	0	0	0	0
469,076385	645	0	0	0	0	0	0
471,051636	645	0	0	0	0	0	0
473,02536	645	0	0	0	0	0	0
474,997498	645	0	0	0	0	0	0
476,968079	645	0	0	0	0	0	0

This journal is © The Royal Society of Chemistry 20xx

Please do not adjust margins

Please do not adjust margins

478,937134	645	0	0	0	0	0	0
480,904572	645	0	0	0	0	0	0
482,870453	645	0	0	0	0	0	0
484,834808	645	0	0	0	0	0	0
486,797607	645	0	0	0	0	0	0
488,75885	645	0	0	0	0	0	0
490,718506	645	0	0	0	0	0	0
492,676605	645	0	0	0	0	0	0
494,633118	645	0	0	0	0	0	0
496,588104	645	0	0	0	0	0	0
498,541504	645	0	0	0	0	0	0
500,493347	645	0	0	0	0	0	0
502,443665	645	0	0	0	0	0	0
504,392365	645	0	0	0	0	0	0
506,339539	645	0	0	0	0	0	0
508,285095	645	0	0	0	0	0	0
510,229126	645	0	0	0	0	0	0
512,17157	645	0	0	0	0	0	0
514,112427	645	0	0	0	0	0	0
516,051697	645	0	0	0	0	0	0
517,989441	645	0	0	0	0	0	0
519,925598	645	0	0	0	0	0	0
521,860168	645	0	0	0	0	0	0
523,793152	645	0	0	0	0	0	0
525,724609	645	0	0	0	0	0	0
527,654419	645	0	0	0	0	0	0
529,582764	645	0	0	0	0	0	0
531,50946	645	0	0	0	0	0	0
533,43457	645	0	0	0	0	0	0
535,358093	645	0	0	0	0	0	0
537,280029	645	0	0	0	0	0	0
539,200439	645	0	0	0	0	0	0
541,119263	645	0	0	0	0	0	0
543,036438	645	0	0	0	0	0	0
544,952087	645	0	0	0	0	0	0
546,866211	645	0	0	0	0	0	0
548,778687	645	0	0	0	0	0	0
550,689514	645	0	0	0	0	0	0
552,598816	645	0	0	0	0	0	0
554,506531	645	0	0	0	0	0	0
556,41272	645	0	0	0	0	0	0
558,317261	645	0	0	0	0	0	0
560,220215	645	0	0	0	0	0	0
562,121582	645	0	0	0	0	0	0
564,021362	645	0	0	0	0	0	0
565,919556	645	0	0	0	0	0	0
567,816162	645	0	0	0	0	0	0

This journal is © The Royal Society of Chemistry 20xx

Please do not adjust margins

Please do not adjust margins

569,711182	645	0	0	0	0	0	0
571,604614	645	0	0	0	0	0	0
573,49646	645	0	0	0	0	0	0
575,386719	645	0	0	0	0	0	0
577,27533	645	0	0	0	0	0	0
579,162354	645	0	0	0	0	0	0
581,047852	645	0	0	0	0	0	0
582,931641	645	0	0	0	0	0	0
584,813965	645	0	0	0	0	0	0
586,69458	645	0	0	0	0	0	0
588,57373	645	0	0	0	0	0	0
590,451111	645	0	0	0	0	0	0
592,326965	645	0	0	0	0	0	0
594,201233	645	0	0	0	0	0	0
596,073914	645	0	0	0	0	0	0
597,944946	645	0	0	0	0	0	0
599,814453	645	0	0	0	0	0	0
601,682251	645	0	0	0	0	0	0
603,548523	645	0	0	0	0	0	0
605,413147	645	0	0	0	0	0	0
607,276245	645	0	0	0	0	0	0
609,137695	645	0	0	0	0	0	0
610,997437	645	0	0	0	0	0	0
612,855713	645	0	0	0	0	0	0
614,712341	645	0	0	0	0	0	0
616,567322	645	0	0	0	0	0	0
618,420715	645	0	0	0	0	0	0
620,272522	645	0	0	0	0	0	0
622,122681	645	0	0	0	0	0	0
623,971313	645	0	0	0	0	0	0
625,818237	645	0	0	0	0	0	0
627,663574	645	0	0	0	0	0	0
629,507324	645	0	0	0	0	0	0
631,349365	645	0	0	0	0	0	0
633,18988	645	0	0	0	0	0	0
635,028809	645	0	0	0	0	0	0
636,865967	645	0	0	0	0	0	0
638,70166	645	0	0	0	0	0	0
640,535645	645	0	0	0	0	0	0
642,368042	645	0	0	0	0	0	0
644,198853	645	0	0	0	0	0	0
646,028015	645	0	0	0	0	0	0
647,855591	645	0	0	0	0	0	0
649,681519	645	0	0	0	0	0	0
651,505798	645	0	0	0	0	0	0
653,328491	645	0	0	0	0	0	0
655,149536	645	2,02395902	4,04680759	1,66541098	35,1354491	10,4804196	8,22163898

This journal is © The Royal Society of Chemistry 20xx

Please do not adjust margins

656,968994	645	0,35864545	2,74886083	6,09192914	24,8758602	5,85980762	4,90551318
658,786743	645	2,28231697	2,16160187	1,92036125	16,7035145	6,72904745	2,8852943
660,602905	645	2,04691488	2,04635335	1,56399301	10,9613224	3,85428696	3,25365232
662,41748	645	0,2418953	2,90194726	1,32932358	7,86468776	4,960489	2,90512744
664,230469	645	3,99914803	-0,2423061	0,84760239	8,12267436	5,4551822	1,57671577
666,041748	645	2,54955647	0,36412244	3,15398253	9,47350402	2,6718448	3,03767892
667,85144	645	0,24348239	3,52952613	2,91937171	8,2816557	1,58315748	1,58393527
669,659424	645	0,85392308	1,34151102	0,6094405	7,56629208	4,02696504	0,48835678
671,46582	645	2,56438857	3,90656776	2,07421635	9,28429283	0,61077007	3,05535068
673,27063	645	0,4903689	2,2060547	4,16468768	13,9810078	4,04687763	1,84039355
675,073792	645	3,93050561	1,84191908	2,2090805	9,830127	6,26630925	0,86050421
676,875305	645	1,84207522	0,73662795	1,34973771	14,0052754	8,96772262	2,33521149
678,675171	645	1,10542078	2,3330259	2,57718149	12,5330273	10,8121234	-0,1229254
680,47345	645	2,08642325	2,45394222	4,5372824	13,3829141	13,7503807	2,33379987
682,27002	645	0,86209255	0,61561147	2,58413824	14,1685169	11,8268828	1,84885832
684,065063	645	1,72991598	0,86472071	4,19774919	14,0919965	9,3940696	-0,3710007
685,858398	645	3,83220736	2,34812792	2,71738129	14,0981737	8,65622531	1,97953784
687,650024	645	0,24748479	1,48450137	3,09100079	16,7117904	8,29347465	1,36228419
689,440186	645	3,47279573	1,61192713	2,35459058	12,9040265	10,6699618	0
691,228577	645	3,72268298	0,49622157	3,09966958	13,2827891	7,57195177	0,99353073
693,015381	645	0,74641824	2,36300935	0,12430013	14,8097816	8,46219662	1,74307289
694,800476	645	5,09925066	2,98410837	2,11257443	15,1793451	7,3403661	2,24053394
696,583984	645	1,24873946	0,62419845	1,99633037	16,8646101	3,74745388	3,24938897
698,365845	645	2,25404692	1,62747619	4,50436465	15,2834351	4,63484672	7,64498784
700,146118	645	2,76594945	1,88535728	2,26117751	17,6084161	4,40182536	5,91392665
701,924683	645	2,52416633	0,25234739	0,75662349	12,8783091	5,80749723	5,17879057
347,741211	650	0	0	0	0	0	0
349,808533	650	0	0	0	0	0	0
351,874329	650	0	0	0	0	0	0
353,938568	650	0	0	0	0	0	0
356,001343	650	0	0	0	0	0	0
358,062561	650	0	0	0	0	0	0
360,122253	650	0	0	0	0	0	0
362,180481	650	0	0	0	0	0	0
364,237152	650	0	0	0	0	0	0
366,292328	650	0	0	0	0	0	0
368,345978	650	0	0	0	0	0	0
370,398132	650	0	0	0	0	0	0
372,44873	650	0	0	0	0	0	0
374,497803	650	0	0	0	0	0	0
376,545349	650	0	0	0	0	0	0
378,5914	650	0	0	0	0	0	0
380,635925	650	0	0	0	0	0	0
382,678955	650	0	0	0	0	0	0
384,720398	650	0	0	0	0	0	0
386,760376	650	0	0	0	0	0	0
388,798767	650	0	0	0	0	0	0

 This journal is © The Royal Society of Chemistry 20xx

Please do not adjust margins

390,835663	650	0	0	0	0	0	0
392,871033	650	0	0	0	0	0	0
394,904877	650	0	0	0	0	0	0
396,937195	650	0	0	0	0	0	0
398,967957	650	0	0	0	0	0	0
400,997253	650	0	0	0	0	0	0
403,024963	650	0	0	0	0	0	0
405,051147	650	0	0	0	0	0	0
407,075806	650	0	0	0	0	0	0
409,098938	650	0	0	0	0	0	0
411,120544	650	0	0	0	0	0	0
413,140564	650	0	0	0	0	0	0
415,159119	650	0	0	0	0	0	0
417,176086	650	0	0	0	0	0	0
419,191528	650	0	0	0	0	0	0
421,205444	650	0	0	0	0	0	0
423,217834	650	0	0	0	0	0	0
425,228668	650	0	0	0	0	0	0
427,237976	650	0	0	0	0	0	0
429,245728	650	0	0	0	0	0	0
431,251953	650	0	0	0	0	0	0
433,256592	650	0	0	0	0	0	0
435,259766	650	0	0	0	0	0	0
437,261353	650	0	0	0	0	0	0
439,261383	650	0	0	0	0	0	0
441,259888	650	0	0	0	0	0	0
443,256836	650	0	0	0	0	0	0
445,252258	650	0	0	0	0	0	0
447,246094	650	0	0	0	0	0	0
449,238434	650	0	0	0	0	0	0
451,229187	650	0	0	0	0	0	0
453,218445	650	0	0	0	0	0	0
455,206116	650	0	0	0	0	0	0
457,192261	650	0	0	0	0	0	0
459,176819	650	0	0	0	0	0	0
461,15979	650	0	0	0	0	0	0
463,141296	650	0	0	0	0	0	0
465,121216	650	0	0	0	0	0	0
467,099579	650	0	0	0	0	0	0
469,076385	650	0	0	0	0	0	0
471,051636	650	0	0	0	0	0	0
473,02536	650	0	0	0	0	0	0
474,997498	650	0	0	0	0	0	0
476,968079	650	0	0	0	0	0	0
478,937134	650	0	0	0	0	0	0
480,904572	650	0	0	0	0	0	0
482,870453	650	0	0	0	0	0	0

This journal is © The Royal Society of Chemistry 20xx

Please do not adjust margins

Please do not adjust margins

484,834808	650	0	0	0	0	0	0
486,797607	650	0	0	0	0	0	0
488,75885	650	0	0	0	0	0	0
490,718506	650	0	0	0	0	0	0
492,676605	650	0	0	0	0	0	0
494,633118	650	0	0	0	0	0	0
496,588104	650	0	0	0	0	0	0
498,541504	650	0	0	0	0	0	0
500,493347	650	0	0	0	0	0	0
502,443665	650	0	0	0	0	0	0
504,392365	650	0	0	0	0	0	0
506,339539	650	0	0	0	0	0	0
508,285095	650	0	0	0	0	0	0
510,229126	650	0	0	0	0	0	0
512,17157	650	0	0	0	0	0	0
514,112427	650	0	0	0	0	0	0
516,051697	650	0	0	0	0	0	0
517,989441	650	0	0	0	0	0	0
519,925598	650	0	0	0	0	0	0
521,860168	650	0	0	0	0	0	0
523,793152	650	0	0	0	0	0	0
525,724609	650	0	0	0	0	0	0
527,654419	650	0	0	0	0	0	0
529,582764	650	0	0	0	0	0	0
531,50946	650	0	0	0	0	0	0
533,43457	650	0	0	0	0	0	0
535,358093	650	0	0	0	0	0	0
537,280029	650	0	0	0	0	0	0
539,200439	650	0	0	0	0	0	0
541,119263	650	0	0	0	0	0	0
543,036438	650	0	0	0	0	0	0
544,952087	650	0	0	0	0	0	0
546,866211	650	0	0	0	0	0	0
548,778687	650	0	0	0	0	0	0
550,689514	650	0	0	0	0	0	0
552,598816	650	0	0	0	0	0	0
554,506531	650	0	0	0	0	0	0
556,41272	650	0	0	0	0	0	0
558,317261	650	0	0	0	0	0	0
560,220215	650	0	0	0	0	0	0
562,121582	650	0	0	0	0	0	0
564,021362	650	0	0	0	0	0	0
565,919556	650	0	0	0	0	0	0
567,816162	650	0	0	0	0	0	0
569,711182	650	0	0	0	0	0	0
571,604614	650	0	0	0	0	0	0
573,49646	650	0	0	0	0	0	0

This journal is © The Royal Society of Chemistry 20xx

Please do not adjust margins

Please do not adjust margins

575,386719	650	0	0	0	0	0	0
577,27533	650	0	0	0	0	0	0
579,162354	650	0	0	0	0	0	0
581,047852	650	0	0	0	0	0	0
582,931641	650	0	0	0	0	0	0
584,813965	650	0	0	0	0	0	0
586,69458	650	0	0	0	0	0	0
588,57373	650	0	0	0	0	0	0
590,451111	650	0	0	0	0	0	0
592,326965	650	0	0	0	0	0	0
594,201233	650	0	0	0	0	0	0
596,073914	650	0	0	0	0	0	0
597,944946	650	0	0	0	0	0	0
599,814453	650	0	0	0	0	0	0
601,682251	650	0	0	0	0	0	0
603,548523	650	0	0	0	0	0	0
605,413147	650	0	0	0	0	0	0
607,276245	650	0	0	0	0	0	0
609,137695	650	0	0	0	0	0	0
610,997437	650	0	0	0	0	0	0
612,855713	650	0	0	0	0	0	0
614,712341	650	0	0	0	0	0	0
616,567322	650	0	0	0	0	0	0
618,420715	650	0	0	0	0	0	0
620,272522	650	0	0	0	0	0	0
622,122681	650	0	0	0	0	0	0
623,971313	650	0	0	0	0	0	0
625,818237	650	0	0	0	0	0	0
627,663574	650	0	0	0	0	0	0
629,507324	650	0	0	0	0	0	0
631,349365	650	0	0	0	0	0	0
633,18988	650	0	0	0	0	0	0
635,028809	650	0	0	0	0	0	0
636,865967	650	0	0	0	0	0	0
638,70166	650	0	0	0	0	0	0
640,535645	650	0	0	0	0	0	0
642,368042	650	0	0	0	0	0	0
644,198853	650	0	0	0	0	0	0
646,028015	650	0	0	0	0	0	0
647,855591	650	0	0	0	0	0	0
649,681519	650	0	0	0	0	0	0
651,505798	650	0	0	0	0	0	0
653,328491	650	0	0	0	0	0	0
655,149536	650	0	0	0	0	0	0
656,968994	650	0	0	0	0	0	0
658,786743	650	0	0	0	0	0	0
660,602905	650	1,80768303	3,49631221	3,37520892	30,5450009	10,1336392	7,60861952

This journal is © The Royal Society of Chemistry 20xx

Please do not adjust margins

Please do not adjust margins

662,41748	650	1,57369636	3,75422649	1,93735487	23,0419797	5,08958077	5,70177063
664,230469	650	0,72775459	0,84940131	2,3051485	12,6373391	5,22104321	4,01127038
666,041748	650	1,09362394	1,45877373	3,16017008	9,98223875	3,77087665	3,40971303
667,85144	650	1,5840215	1,58468249	2,31570337	10,1317487	1,95160909	2,32009419
669,659424	650	-0,7325751	1,58790828	1,58765389	6,11588734	1,83335794	0,24471758
671,46582	650	2,68885497	1,10044513	3,54531071	7,7138972	2,07993015	1,83725845
673,27063	650	0,61349791	0,61375392	2,94554686	8,48165839	2,82504945	2,95113193
675,073792	650	2,4587173	0,98389732	2,70528416	6,15796485	3,56887824	-0,1232006
676,875305	650	2,45825118	-0,8607469	-2,2129947	7,75756476	3,44516022	2,95625436
678,675171	650	3,07330231	1,84475086	1,59852795	11,9460852	3,44570773	1,47836209
680,47345	650	1,10554433	2,70356939	2,8260061	9,35267502	4,05791293	0,86171964
682,27002	650	0	0,49326147	3,0823903	10,9904284	7,65037697	3,70588182
684,065063	650	0,74204183	2,10332917	2,10299221	12,2659426	9,90426505	0
685,858398	650	4,33047475	0,74267688	1,2375965	12,7671303	11,0233248	3,0998578
687,650024	650	-0,1238508	-0,3717073	2,22988664	16,6261644	7,68679846	0,99293989
689,440186	650	2,35860366	0,37256651	1,73836516	12,1875397	8,32590214	-0,1244044
691,228577	650	0,86938671	0,62124964	3,85113071	13,9354687	6,21641636	0,74679346
693,015381	650	-1,1206079	1,86845912	2,49087972	13,8459491	10,3453307	1,49736162
694,800476	650	2,73857944	1,99252525	2,98830907	14,8401477	10,0935239	1,37223864
696,583984	650	0,49993321	-0,5001418	2,87535478	14,5244128	6,88128931	0,62626234
698,365845	650	2,38135535	1,7554151	4,13710128	15,1930363	5,14410228	4,52175892
700,146118	650	2,1391963	0,50355034	1,88801128	15,5058447	5,41658179	2,52212153
701,924683	650	4,92643481	1,26371553	2,14797223	15,4389074	5,43740548	6,58272593

 This journal is © The Royal Society of Chemistry 20xx

Please do not adjust margins
